# Supplementary material for: CEA-CD3 bispecific antibody cibisatamab with or without atezolizumab in patients with CEA-positive solid tumours: results of two multi-institutional Phase 1 trials
Source: Nat Commun. 2024 May 15;15:4091. doi: 10.1038/s41467-024-48479-8 (PMC11096172; doi:10.1038/s41467-024-48479-8)
Supplement: Supplementary file 1 — Supplementary Information [file 41467_2024_48479_MOESM1_ESM.pdf]

Supplementary Information

CEA-CD3 bispecific antibody cibisatamab with or without atezolizumab in patients with CEA-positive solid tumours: results of two multi-institutional Phase 1 trials

Contents

Supplementary Methods
Supplementary Results
Supplementary Table 1. Baseline and disease characteristics of patients enrolled in S1 and S2
Supplementary Table 2. Most frequent adverse events (≥20% in either study) in S1 and S2 by causality and severity
Supplementary Table 3. Summary of treatment-related AEs (≥20% in either study) in S1 and S2, by preferred terms.
Supplementary Table 4. Summary of IRR symptoms (≥10% in either study) in S1 and S2, by preferred terms.
Supplementary Table 5. Efficacy in S1 in patients with MSS-CRC
Supplementary Table 6. Efficacy in S1 in patients with diagnoses other than CRC
Supplementary Table 7. Efficacy in S2 in patients with diagnoses other than CRC
Supplementary Table 8. Efficacy for all patients and patients with MSS-CRC, broken down by sex
Supplementary Figure 1. CONSORT diagram for study S1 (BP29541)
Supplementary Figure 2. CONSORT diagram for study S2 (WP29945)
Supplementary Figure 3. Impact of obinutuzumab pretreatment and ADA on observed minimum and maximal serum concentrations of cibisatamab following fourth IV infusion (S1 [BP29541]).
Supplementary Figure 4. Blood flow data showing changes from baseline in **a** peripheral CD8+ cells changes and **b** peripheral CD4+ cells in patients receiving cibisatamab
Supplementary Note. Final study protocol for studies S1 and S2

Supplementary Methods

Main inclusion and exclusion criteria for studies S1 and S2

<u>S1 inclusion criteria:</u>

- For dose escalation, locally advanced and/or metastatic gastrointestinal solid tumor in participants who have progressed on a standard therapy, are intolerant to standard of care (SOC), and/or are non-amenable to SOC and other solid tumours expressing carcinoembryonic antigen (CEA). Only patients with locally advanced and/or metastatic colorectal cancer (CRC) should be included in the scheduled comparison expansion
- Radiologically measurable disease according to Response Evaluation Criteria in Solid Tumors version 1.1 (RECIST 1.1)
- Life expectancy, in the opinion of the investigator, of at least 12 weeks
- Lactate dehydrogenase (LDH) of 2.5 × or less the ULN (upper limit of normal)
- Eastern Cooperative Oncology Group performance status (ECOG PS) of 0-1
- All acute toxic effects of any prior radiotherapy, chemotherapy or surgical procedure must have resolved to grade 1 or below or returned to baseline, except alopecia (any grade) and grade 2 peripheral neuropathy
- Adequate haematological, liver and renal function
- Must agree to remain abstinent or be willing to use effective methods of contraception, as defined in the protocol
- Non-gastrointestinal solid tumours (like non-small cell lung cancer or breast cancer) should have confirmed CEA expression in tumor tissue (20% or more of tumor cells staining with at least moderate to high intensity: immunohistochemistry [IHC] 2+ and IHC 3+)
    - For patients with CRC, pancreatic cancer or gastric cancer, the CEA assessment will be performed retrospectively, and the result is not needed to enrol the participant

<u>S1 exclusion criteria:</u>

- A history or clinical evidence of central nervous system primary tumours or metastases, including leptomeningeal metastases, unless they have been previously treated, are asymptomatic, and have had no requirement for steroids or enzyme-inducing anticonvulsants in the last 14 days before screening
- Spinal cord compression not definitively treated with surgery and/or radiation or previously diagnosed and treated spinal cord compression without evidence that disease has been clinically stable for at least 2 weeks prior to enrollment
- Leptomeningeal disease
- Paraspinal, paratracheal and mediastinal pathologic lesions larger than 2 centimetres unless they are previously irradiated. Irradiation of lesions must be completed at least 14 days prior to initiation of study treatment
- Another invasive malignancy in the last 2 years (with the exception of basal cell carcinoma and tumours deemed by the investigator to be of low likelihood for recurrence)
- Evidence of significant, uncontrolled concomitant diseases that could affect compliance with the protocol or interpretation of results or contraindicate the use of

an investigational drug, including diabetes mellitus, history of relevant cardio-pulmonary disorders and known autoimmune diseases
- Bilateral lung lesions and dyspnoea and/or with bilateral lung lesions and an oxygen saturation (SaO_2) level less than 92%, or lobectomy or pneumonectomy with lung metastases in the remaining lung and either dyspnoea or SaO_2 less than 92% at baseline
- Uncontrolled hypertension (systolic blood pressure greater than 150 mm Hg and/or diastolic blood pressure greater than 100 mm Hg), unstable angina, congestive heart failure of any New York Heart Association classification, serious cardiac arrhythmia that requires treatment (with the exceptions of atrial fibrillation and paroxysmal supraventricular tachycardia) and history of myocardial infarction within 6 months of enrollment
- Active or uncontrolled infections
- Known HIV or known active hepatitis B or hepatitis C infection for participants not receiving obinutuzumab pretreatment
- Known HIV (HIV testing will be performed at screening if required by local regulations) in participants to be pretreated with obinutuzumab
- Pregnancy or breastfeeding
- Known hypersensitivity to any of the components of cibisatamab and/or obinutuzumab
- Concurrent therapy with any other investigational drug
- Last dose of any chemotherapy less than 28 days prior to the first cibisatamab infusion
- Expected need for regular immunosuppressive therapy
- Regular dose of corticosteroids the 28 days prior to Day 1 of this study or anticipated need for corticosteroids that exceeds prednisone 10 mg/day or equivalent within 28 days prior to the first cibisatamab infusion. Inhaled and topical steroids are permitted
- Radiotherapy within the last 28 days prior to the first cibisatamab infusion, with the exception of limited-field palliative radiotherapy

Additional S1 exclusion criteria for participants to be pretreated with obinutuzumab:
- Positive test results for human T-lymphotropic virus 1 or active HIV infection
- Positive test results for chronic hepatitis B infection or hepatitis C
- Known active tuberculosis requiring treatment within 3 years prior to baseline or latent tuberculosis that has not been appropriately treated
- Active bacterial, viral, fungal or other infection, or any major episode of infection requiring treatment with intravenous antibiotics within 4 weeks of Cycle 1, Day 1
- Known hypersensitivity to any of the components of obinutuzumab; hypersensitivity to Chinese hamster ovary cell products or other recombinant human antibodies
- History of progressive multifocal leukoencephalopathy

<u>Study S2 inclusion criteria:</u>
- Confirmed locally advanced and/or metastatic solid tumor, with at least one tumor lesion of accessible non-critical location to biopsy, in participants who have progressed on a standard therapy, are intolerant to standard therapy and/or are non-amenable to standard therapy
- Radiologically measurable and clinically evaluable disease (per RECIST 1.1)

- Life expectancy (in the opinion of the investigator) of at least 12 weeks and LDH levels of 2.5 × or less ULN
- ECOG PS of 0-1
- All acute toxic effects of any prior radiotherapy, chemotherapy or surgical procedure must have resolved to grade 1 or below or returned to baseline, except alopecia (any grade) and grade 2 peripheral neuropathy
- Adequate haematological, liver and renal function
- Negative serum pregnancy test within 7 days prior to study treatment in premenopausal women and women 2 years or less after the start of menopause (menopause is defined as amenorrhea for more than 2 years)
- Must agree to remain abstinent or be willing to use effective methods of contraception as defined in the protocol
- Participants with cancer other than CRC should have confirmed CEA expression in tumor tissue. For those with CRC, the CEA assessment should be performed, but the result is not required for participant selection

S2 exclusion criteria:

- Active or untreated central nervous system metastases as determined by computed tomography or magnetic resonance imaging evaluation during screening and prior radiographic assessments
- Spinal cord compression not definitively treated with surgery and/or radiation or previously diagnosed and treated spinal cord compression without evidence that disease has been clinically stable for at least 2 weeks prior to enrollment
- Leptomeningeal disease
- Paraspinal, paratracheal and mediastinal pathological lesions larger than 2 cm unless they are previously irradiated
- Malignancies within 5 years prior to enrollment, with the exception of those with a negligible risk of metastasis or death and treated with expected curative outcome
- Significant, uncontrolled concomitant diseases that could affect compliance with the protocol or interpretation of results
- Uncontrolled hypertension, unstable angina, congestive heart failure or serious cardiac arrhythmia requiring treatment history of myocardial infarction within 6 months of enrollment
- Administration of a live, attenuated vaccine within 28 days before Cycle 1 Day 1 or anticipation that such a live attenuated vaccine will be required during the study
- HIV, active hepatitis B or hepatitis C
- Severe infections within 28 days prior to Cycle 1 Day 1, including but not limited to hospitalisation for complications of infection, bacteraemia, or severe pneumonia or active tuberculosis
- Oral or intravenous antibiotics within 14 days prior to Day 1
- Any other diseases, metabolic dysfunction, physical examination finding or clinical laboratory finding giving reasonable suspicion of a disease or condition that would contraindicate the use of an investigational drug
- Major surgery or significant traumatic injury less than 28 days prior to Cycle 1 Day 1 (excluding biopsies) or anticipation of the need for major surgery during study treatment
- Known history of autoimmune disease, as defined in the protocol

- History of idiopathic pulmonary fibrosis, pneumonitis (including drug induced), organising pneumonia (i.e., bronchiolitis obliterans, cryptogenic organising pneumonia, etc.) or evidence of active pneumonitis (including drug induced) on screening chest computed tomography scan. History of radiation pneumonitis in the radiation field (fibrosis) is permitted
- Bilateral lung lesions and dyspnoea and/or SaO_2 less than 92% (at rest, room air and exertion) or lobectomy or pneumonectomy with lung metastases in the remaining lung and either dyspnoea or SaO_2 less than 92% (at rest, room air and exertion) at baseline
- Pregnant or breast-feeding
- Known hypersensitivity to any of the components of cibisatamab and atezolizumab; hypersensitivity to Chinese hamster ovary cell products or other recombinant human antibodies
- Investigational therapy (defined as treatment for which there is no regulatory authority approved indication) or last dose of prior immunotherapies within 28 days prior to Cycle 1 Day 1. Participants previously treated with anti–programmed death-ligand 1 or anti–programmed death-1 are excluded
- Last dose of any approved anti-cancer therapy within 28 days prior to the first cibisatamab infusion
- Prior systemic corticosteroids greater than 10 mg prednisone (or equivalent) within 14 days of Cycle 1 Day 1. Inhaled and/or topical steroids are permitted
- Expected need for regular immunosuppressive therapy
- Radiotherapy within the last 28 days before Cycle 1 Day 1, with the exception of limited-field palliative radiotherapy

Supplementary Results

Additional Dosing Details

A total of 22 patients received step-up dosing (40 mg up to 600 mg) in combination with atezolizumab. One patient received flat doses of 10 mg, 2 patients of 20 mg, 2 patients of 40 mg, 1 patient of 100 mg, 3 patients of 160 mg and 2 patients of 300 mg.

Additional Safety Population Details

In S1, 149 patients were included in the safety-evaluable population: 116 patients (77.9%) received cibisatamab only, 27 patients (18.1%) received obinutuzumab pretreatment followed by cibisatamab and 6 patients (4.0%) received obinutuzumab only due to treatment discontinuation prior to receiving the first dose with cibisatamab. In S2, a total of 228 patients were included in the safety-evaluable population.

Description of DLTs and MTD

In S1, 7 dose-limiting toxicities (DLTs) related to cibisatamab were reported in 7 of 102 DLT-evaluable patients (6.9%). Of these, 6 DLTs were reported in the multiple ascending flat-dose cohorts (one event each): grade 3 dyspnea, grade 3 hypoxia, grade 3 diarrhoea, grade 3 colitis, grade 4 colitis and grade 5 respiratory failure. One DLT was reported in the step-up dose cohorts (late-cycle maximum tolerated dose [MTD]): grade 5 respiratory failure. The events of grade 3 dyspnea, grade 3 colitis and grade 4 colitis led to treatment and study discontinuation. The MTD was defined as 400 mg for flat continuous dosing at QW and Q3W.

In S2, 17 DLTs were reported in 15 of 226 DLT-evaluable patients (6.6%). In Part IA (dose escalation; n=73 DLT-evaluable), cibisatamab-related DLTs were (one event each) grade 3 alanine aminotransferase (ALT) increased, grade 3 dyspnea and grade 3 rash maculo-papular. DLTs related to both cibisatamab and atezolizumab were 2 events of grade 3 colitis and a grade 4 event of dyspnea. In Part IB (dose-schedule finding; n=153 DLT-evaluable), for late-cycle MTD determination, cibisatamab-related DLTs were (one event each) grade 3 rash, grade 3 ALT increased, grade 3 aspartate aminotransferase (AST) increased, grade 3 syncope, grade 3 diarrhoea, grade 3 arthralgia, grade 4 platelet count decrease, grade 4 cardiac arrest and grade 5 hypovolemic shock. DLTs related to both cibisatamab and atezolizumab were a grade 3 peripheral ischemia and a grade 3 stomatitis (grade 3 mucositis oral).

During the dose-escalation phase, all three patients at the 300-mg dose level had serious adverse events (SAEs) after their first infusion. While these SAEs were not classified as DLTs, it was decided not to escalate the dose further. Thus, the MTD for cibisatamab in combination with atezolizumab was not reached.

Frequency and Severity of AEs

The most frequently reported adverse events (AEs) in both S1 and S2 were infusion-related reaction (IRR), pyrexia and diarrhoea. The most common AEs (≥20%) in S1 and S2 are presented in Table A5 by causality and severity. A total of 102 of 149 patients (68.5%) in S1 reported at least one grade ≥3 AE: 89 patients (59.7%) reporting a grade 3 event and 7 patients (4.7%) reporting a grade 4 event. In S1, 6 of 149 patients (4.0%) experienced the following grade 5 events: dyspnoea, IRR and respiratory failure, all considered related to

cibisatamab; sepsis considered related to obinutuzumab; cardio-respiratory arrest; and tumor thrombosis not considered treatment related by the investigator.

A total of 154 of 228 patients (67.5%) in S2 reported at least one grade ≥3 AE: 131 patients (57.5%) reporting a grade 3 event and 17 patients (7.5%) reporting a grade 4 event. In S2, 6 of 228 (2.6%) experienced grade 5 events; one cibisatamab-related event of hypovolemic shock and 5 events (1 each) of respiratory tract infection, urinary tract infection, disseminated intravascular coagulation, bile duct obstruction and cerebrovascular accident, not considered treatment-related by the investigator.

IRR, anaemia and diarrhoea were the most significant grade ≥3 AEs in S1 and S2. Laboratory findings of transient grade ≥3 increases in liver enzymes were seen in both studies; however, their incidence was higher in S2 than in S1 (S1: ALT and AST increased, 3.5% each; S2: ALT increased 6.1% and AST increased 9.2%), likely due to the additive effect of atezolizumab, for which increases in liver transaminases is an identified risk.

Obinutuzumab-related AEs were reported in 13 of 33 patients (39.4%) treated with obinutuzumab alone or followed by cibisatamab, of which 10 patients (30.3%) reported related AEs prior to the first cibisatamab infusion (C1D1) and 7 patients (21.2%) reported related AEs following C1D1. IRRs (4 patients [12.1%]) and chills (3 patients [9.1%]) were the only AEs considered related to obinutuzumab reported in 3 patients.

AEs related to both obinutuzumab and cibisatamab were reported in 3 of 27 patients (11.1%) treated with obinutuzumab and cibisatamab. Pyrexia was the only AE reported in 2 patients (7.4%) (all other AEs were reported in 1 patient each).

Supplementary Tables

Supplementary Table 1. Baseline and disease characteristics of patients enrolled in S1 and S2

	S1 (BP29541)				S2 (WP29945)			
	**90-200 mg flat dose w/o obi QW CRC (n=14)**	**All CRC patients (n=125)**	**All step-up patients (n=46)**	**All patients (n=149)**	**100 mg Q3W CRC (n=20)**	**All CRC 100-160 mg QW + Q3W (n=83)**	**All step-up B1 + C2 (n=52)**	**All patients (n=228)**
Age, years								
Median (range)	62.5 (55-72)	60 (29-80)	59 (22-79)	60 (22-80)	57.5 (24-76)	58.0 (24-81)	52.5 (27-79)	57.0 (24-81)
Sex, n (%)								
Male	8 (57.1)	79 (63.2)	25 (54.3)	88 (59.1)	13 (65.0)	51 (61.4)	24 (46.2)	132 (57.9)
Female	6 (42.9)	46 (36.8)	21 (45.7)	61 (40.9)	7 (35.0)	32 (38.6)	28 (53.8)	96 (42.1)
ECOG performance status								
0	7 (50.0)	74 (59.2)	27 (58.7)	82 (55.0)	11 (55.0)	50 (60.2)	28 (53.8)	132 (57.9)
1	7 (50.0)	51 (40.8)	19 (41.3)	67 (45.0)	9 (45.0)	33 (39.8)	24 (46.2)	96 (42.1)
Predominant localization metastasis								
Lung	9 (64.3)	90 (72.0)	27 (58.7)	103 (69.1)	13 (65.0)	59 (71.1)	35 (67.3)	160 (70.2)
Liver	13 (92.9)	96 (76.8)	30 (65.2)	111 (74.5)	19 (95.0)	73 (88.0)	37 (71.2)	183 (80.3)
Peritoneum	5 (35.7)	24 (19.2)	9 (19.6)	27 (18.1)	10 (50.0)	38 (45.8)	20 (38.5)	94 (41.2)
Metastatic site involved								
1-2 organs	1 (7.1)	26 (20.8)	16 (34.8)	34 (22.8)	1 (5.0)	9 (10.8)	9 (17.3)	32 (14.0)
≥3 organs	13 (92.9)	99 (79.2)	30 (65.2)	115 (77.2)	19 (95.0)	74 (89.2)	43 (82.7)	196 (86.0)
Prior adjuvant therapy								
No	8 (57.1)	52 (41.6)	17 (37.0)	61 (40.9)	7 (35.0)	29 (34.9)	22 (42.3)	91 (39.9)
Yes	6 (42.9)	73 (58.4)	29 (63.0)	88 (59.1)	13 (65.0)	54 (65.1)	30 (57.7)	137 (60.1)

No. of prior metastatic therapy								
0	0	2 (1.6)	1 (2.2)	2 (1.3)	0	0	0	1 (0.4)
1	1 (7.1)	8 (6.4)	4 (8.7)	9 (6.0)	3 (15.0)	9 (10.8)	4 (7.7)	28 (12.3)
2	4 (28.6)	36 (28.8)	19 (41.3)	48 (32.2)	7 (35.0)	25 (30.1)	20 (38.5)	59 (25.9)
≥3	9 (64.3)	79 (63.2)	22 (47.8)	90 (60.4)	10 (50.0)	49 (59.0)	28 (53.8)	140 (61.4)
BRAF status								
wt	10 (71.4)	75 (60.0)	25 (54.3)	81 (54.4)	11 (55.0)	40 (48.2)	38 (73.1)	126 (55.3)
mut	2 (14.3)	12 (9.6)	4 (8.7)	13 (8.7)	1 (5.0)	4 (4.8)	1 (1.9)	5 (2.2)
unknown	2 (14.3)	38 (30.4)	17 (37.0)	55 (36.9)	8 (40.0)	39 (47.0)	13 (25.0)	97 (42.5)
KRAS status								
wt	7 (50.0)	45 (36.0)	15 (32.6)	49 (32.9)	10 (50.0)	36 (43.4)	25 (48.1)	91 (39.9)
mut	7 (50.0)	68 (54.4)	23 (50.0)	73 (49.0)	1 (5.0)	20 (24.9)	17 (32.7)	55 (24.1)
unknown	0	12 (9.6)	8 (17.4)	27 (18.1)	9 (45.0)	27 (31.7)	10 (19.2)	82 (36.0)
MSI status								
MSS	11 (78.6)	100 (80.0)	43 (93.5)	115 (77.2)	19 (95.0)	80 (96.4)	49 (94.2)	207 (90.8)
MSI-H	0	0	0	0	1 (5.0)	3 (3.6)	2 (3.8)	8 (3.5)
MSI-L[a]	0	2 (1.6)	1 (2.1)	2 (1.3)	0	0	1 (1.9)	2 (0.9)
unknown	3 (21.4)	23 (18.4)	2 (4.4)	32 (21.5)	0	0	0	0

Data presented are n (%) except where otherwise indicated.

[a] MSI-L patients have been included in the MSS patient population.

CRC, colorectal cancer; ECOG, Eastern Cooperative Oncology Group; MSI, microsatellite instability; MSI-H, microsatellite instability high; MSI-L, microsatellite instability low; MSS, microsatellite stable; mut, mutant; wt, wild-type.

Supplementary Table 2. Most frequent adverse events (≥20% in either study) in S1 and S2 by causality and severity

Preferred term, n (%)	**S1: cibisatamab monotherapy (N=143)***				**S2: cibisatamab + atezolizumab (N=228)**			
	**All causality**	**Related**	**All causality grade ≥3**	**Related grade ≥3**	**All causality**	**Related**	**All causality grade ≥3**	**Related grade ≥3**
IRR	108 (75.5)	108 (75.5)	31 (21.7)	31 (21.7)	167 (73.2)	167 (73.2)	38 (16.7)	38 (16.7)
Pyrexia	79 (55.2)	67 (46.9)	0	0	125 (54.8)	108 (47.4)	5 (2.2)	4 (1.8)
Diarrhea	70 (49.0)	59 (41.3)	9 (6.3)	8 (5.6)	142 (62.3)	117 (51.3)	20 (8.8)	17 (7.5)
Nausea	54 (37.8)	39 (27.3)	4 (2.8)	2 (1.4)	73 (32.0)	58 (25.4)	1 (0.4)	0
Vomiting	47 (32.9)	32 (22.4)	3 (2.1)	2 (1.4)	72 (31.6)	51 (22.4)	2 (0.9)	1 (0.4)
Anemia	47 (32.9)	9 (6.3)	11 (7.7)	3 (2.1)	57 (25.0)	13 (5.7)	19 (8.3)	3 (1.3)
Asthenia	45 (31.5)	28 (19.6)	5 (3.5)	2 (1.4)	74 (32.5)	49 (21.5)	5 (2.2)	1 (0.4)
Decreased appetite	44 (30.8)	28 (19.6)	0	0	72 (31.6)	48 (21.1)	0	0
Chills	37 (25.9)	36 (25.2)	1 (0.7)	1 (0.7)	73 (32.0)	64 (28.1)	0	0
Cough	34 (23.8)	10 (7.0)	0	0	52 (22.8)	27 (11.8)	1 (0.4)	0
Dysgeusia	32 (22.4)	25 (17.5)	0	0	91 (39.9)	78 (34.2)	0	0
Fatigue	30 (21.0)	26 (18.2)	2 (1.4)	2 (1.4)	88 (38.6)	60 (26.3)	3 (1.3)	1 (0.4)
Abdominal Pain	23 (16.1)	7 (4.9)	3 (2.1)	2 (1.4)	60 (26.3)	24 (10.5)	5 (2.2)	0
Arthralgia	17 (11.9)	13 (9.1)	0	0	51 (22.4)	46 (20.2)	3 (1.3)	3 (1.3)
Pruritus	15 (10.5)	13 (9.1)	0	0	56 (24.6)	46 (20.2)	1 (0.4)	1 (0.4)

[a]* Cibisatamab with or without obinutuzumab. Excludes 6 patients who only received obinutuzumab.

Supplementary Table 3. Summary of treatment-related AEs (≥20% in either study) in S1 and S2, by preferred terms.

Related Adverse Events with >= 20% Incidence

Study<br>Preferred Term	n,	N (%)	Time to Onset [Weeks] Med	Time to Onset [Weeks] Min; Max	Duration [Weeks] Med	Duration [Weeks] Min; Max	Events Resolved [%]
CIBI MONOTHERAPY (S1) (N=149)							
IRR	253,	108 (72.5%)	2.0	0.0; 31.2	0.1	0.0; 7.1	99.2
Pyrexia	137,	67 (45.0%)	2.8	0.0; 30.4	0.1	0.0; 4.9	99.3
Diarrhoea	120,	59 (39.6%)	2.2	0.0; 48.2	0.1	0.0; 20.7	100
Nausea	57,	39 (26.2%)	1.2	0.0; 35.1	0.3	0.0; 24.3	93.0
Chills	59,	36 (24.2%)	3.0	0.0; 94.2	0.0	0.0; 9.9	100
Vomiting	37,	32 (21.5%)	1.7	0.0; 32.2	0.1	0.0; 4.7	97.3
Asthenia	49,	28 (18.8%)	2.2	0.0; 35.1	0.9	0.0; 9.1	83.7
Decreased Appetite	31,	28 (18.8%)	1.1	0.2; 40.1	1.9	0.1; 10.1	61.3
Fatigue	33,	26 (17.4%)	2.1	0.1; 78.1	1.9	0.1; 66.6	57.6
Dysgeusia	32,	25 (16.8%)	1.1	0.1; 7.4	2.1	0.0; 56.6	71.9
Arthralgia	19,	13 (8.7%)	2.0	0.1; 26.2	1.3	0.0; 11.9	94.7
Pruritus	15,	13 (8.7%)	3.0	0.4; 18.1	2.2	0.0; 33.3	93.3
CIBI + ATEZOLIZUMAB (S2) (N=228)							
IRR	409,	167 (73.2%)	2.1	0.0; 70.9	0.1	0.0; 2.9	99.8
Pyrexia	174,	108 (47.4%)	1.1	0.0; 40.4	0.1	0.0; 17.4	98.3
Diarrhoea	211,	117 (51.3%)	2.0	0.0; 82.9	0.3	0.0; 62.0	97.2
Nausea	79,	58 (25.4%)	1.5	0.0; 52.6	0.1	0.0; 17.0	92.4
Chills	110,	64 (28.1%)	3.0	0.0; 29.1	0.0	0.0; 5.9	99.1
Vomiting	62,	51 (22.4%)	1.0	0.0; 27.1	0.1	0.0; 20.9	96.8
Asthenia	74,	49 (21.5%)	2.1	0.1; 50.8	2.0	0.0; 58.7	79.7
Decreased Appetite	64,	48 (21.1%)	1.5	0.1; 36.6	1.9	0.1; 8.7	84.4
Fatigue	136,	60 (26.3%)	9.8	0.1; 101	0.3	0.0; 52.3	84.6
Dysgeusia	93,	78 (34.2%)	1.2	0.1; 26.8	4.7	0.0; 34.0	79.6
Arthralgia	83,	46 (20.2%)	7.9	0.5; 69.2	0.7	0.0; 26.9	86.7
Pruritus	57,	46 (20.2%)	4.2	0.2; 76.9	1.7	0.0; 24.0	86.0

AE, adverse events; CIBI, cibisatamab; IRR, infusion-related reaction; Max, maximum; Med, median; Min, minimum.

Supplementary Table 4. Summary of IRR symptoms (≥10% in either study) in S1 and S2, by preferred terms.

IRR Symptoms with >= 10% Incidence

Study<br>Preferred Term	n, N (%)	Time to Onset [Weeks] Med	Time to Onset [Weeks] Min; Max	Duration [Weeks] Med	Duration [Weeks] Min; Max	Events Resolved [%]	Time to Onset since End of Last Infusion [Hours] Med	Time to Onset since End of Last Infusion [Hours] Min; Max
CIBI MONOTHERAPY (S1) (N=149)								
Pyrexia	141, 89 (59.7%)	0.2	0.0; 18.1	0.1	0.0; 1.0	99.3	5.1	0.0; 171.3
Chills	146, 74 (49.7%)	2.0	0.0; 21.1	0.0	0.0; 3.2	100	1.6	0.0; 36.3
Vomiting	95, 63 (42.3%)	0.2	0.0; 31.2	0.0	0.0; 1.9	100	4.4	0.0; 37.3
Nausea	109, 62 (41.6%)	1.0	0.0; 31.2	0.1	0.0; 10.3	99.1	4.0	0.5; 38.0
Diarrhoea	69, 42 (28.2%)	1.1	0.0; 31.0	0.1	0.0; 1.9	100	9.4	0.5; 39.0
Hypotension	69, 36 (24.2%)	3.1	0.0; 31.0	0.0	0.0; 0.7	98.6	3.4	0.5; 33.0
Dyspnoea	22, 15 (10.1%)	1.6	0.0; 16.0	0.0	0.0; 1.7	100	3.1	0.2; 83.2
Abdominal Pain	10, 10 (6.7%)	0.1	0.0; 25.1	0.1	0.0; 0.6	90.0	6.9	1.5; 11.5
Fatigue	9, 9 (6.0%)	0.1	0.0; 2.4	0.1	0.0; 3.0	100	14	5.9; 36.5
Hypertension	11, 9 (6.0%)	2.0	0.0; 7.0	0.0	0.0; 0.1	100	1.8	1.0; 33.3
Tumour Pain	7, 6 (4.0%)	0.2	0.0; 15.9	0.2	0.1; 1.7	85.7	7.3	2.0; 38.5
CIBI + ATEZOLIZUMAB (S2) (N=228)								
Pyrexia	185, 112 (49.1%)	1.0	0.0; 70.9	0.1	0.0; 4.0	100	7.8	0.3; 191.5
Chills	255, 118 (51.8%)	2.2	0.0; 70.9	0.0	0.0; 0.4	100	1.6	0.0; 504.8
Vomiting	102, 70 (30.7%)	0.9	0.0; 51.0	0.0	0.0; 1.0	100	5.9	0.0; 336.0
Nausea	113, 79 (34.6%)	1.2	0.0; 51.0	0.0	0.0; 1.4	100	7.3	0.3; 504.0
Diarrhoea	114, 79 (34.6%)	0.3	0.0; 51.0	0.1	0.0; 1.1	99.1	12	0.0; 57.5
Hypotension	55, 42 (18.4%)	3.0	0.0; 51.1	0.0	0.0; 0.9	100	5.5	0.8; 167.6
Dyspnoea	24, 21 (9.2%)	2.7	0.0; 51.0	0.1	0.0; 2.1	100	11	0.4; 181.0
Abdominal Pain	29, 26 (11.4%)	0.2	0.0; 9.4	0.0	0.0; 1.0	100	5.3	0.7; 180.7
Fatigue	55, 32 (14.0%)	2.1	0.0; 34.2	0.1	0.0; 2.0	98.2	12	1.3; 202.7
Hypertension	40, 31 (13.6%)	2.0	0.0; 32.8	0.0	0.0; 0.4	100	1.5	0.4; 166.9
Tumour Pain	30, 25 (11.0%)	0.2	0.0; 10.3	0.1	0.0; 1.4	100	8.7	0.6; 527.0

CIBI, cibisatamab; IRR, infusion-related reaction; Max, maximum; Med, median; Min, minimum.

Supplementary Table 5. Efficacy in S1 in patients with MSS-CRC

	**90 mg-200 mg (n=11)**	**300-600 mg flat (n=9)**	**All CRC (n=100)**	**Step-up A/B: 40-1200 mg (n=14)**	**Step-up C: 40-600 mg (n=22)**	**All step-up (n=36)**
ORR [90% CI]	1 (9.1) [0.5, 36.4]	0 [0.0, 28.3]	5 (5.0) [2.0, 10.2]	2 (14.3) [2.6, 38.5]	1 (4.5) [0.2, 19.8]	3 (8.3) [2.3, 20.2]
CR [90% CI]	0 [0.0, 23.8]	0 [0.0, 28.3]	0 [0.0, 3.0]	0 [0.0, 19.3]	0 [0.0, 12.7]	0 [0, 8]
PR [90% CI]	1 (9.1) [0.5, 36.4]	0 [0.0, 28.3]	5 (5.0) [2.0, 10.2]	2 (14.3) [2.6, 38.5]	1 (4.5) [0.2, 19.8]	3 (8.3) [2.3, 20.2]
SD [90% CI]	2 (18.2) [3.3, 47.0]	3 (33.3) [9.8, 65.5]	26 (26.0) [18.9, 34.2]	5 (35.7) [15.3, 61.0]	5 (22.7) [9.4, 42.0]	10 (27.8) [15.9, 42.5]
PD [90% CI]	7 (63.6) [35.0, 86.5]	2 (22.2) [4.1, 55.0]	50 (50.0) [41.4, 58.6]	3 (21.4) [6.1, 46.6]	13 (59.1) [39.5, 76.7]	16 (44.4) [30.2, 59.4]
NE	1 (9.1)	4 (44.4)	19 (19.0)	4 (28.6)	3 (13.6)	7 (19.4)
DCR [90% CI]	3 (27.3) [7.9, 56.4]	3 (33.3) [9.8, 65.5]	31 (31.0) [23.4, 39.5]	7 (50.0) [26.4, 73.6]	6 (27.3) [12.6, 46.8]	13 (36.1) [22.9, 51.2]
DOR range, mo	3.1	NA	3.1-11.1	5.6-7.3	11.1	5.6-11.1

Data presented are n (%) except where otherwise indicated.
CR, complete response; DCR, disease control rate; DOR, duration of response; MSS-CRC, microsatellite-stable colorectal cancer; NE, unevaluable or missing; PD, progressive disease; PR, partial response; SD, stable disease; ORR, overall response.

Supplementary Table 6. Efficacy in S1 in patients with diagnoses other than CRC

	**Bile duct (n=2)**	**Breast (n=2)**	**Lung (n=4)**	**Pancreatic (n=8)**	**All non-CRC (n=24)**
ORR [90% CI]	0 [0.0, 77.6]	0 [0.0, 77.6]	0 [0.0, 52.7]	1 (12.5) [0.6, 47.1]	1 (4.2) [0.2, 18.3]
CR [90% CI]	0 [0.0, 77.6]	0 [0.0, 77.6]	0 [0.0, 52.7]	0 [0.0, 31.2]	0 [0.0, 11.7]
PR [90% CI]	0 [0.0, 77.6]	0 [0.0, 77.6]	0 [0.0, 52.7]	1 (12.5) [0.6, 47.1]	1 (4.2) [0.2, 18.3]
SD [90% CI]	1 (50.0) [2.5, 97.5]	1 (50.0) [2.5, 97.5]	2 (50.0) [9.8, 90.2]	2 (25.0) [4.6, 60.0]	7 (29.2) [14.6, 47.9]
PD [90% CI]	1 (50.0) [2.5, 97.5]	1 (50.0) [2.5, 97.5]	1 (25.0) [1.3, 75.1]	3 (37.5) [11.1, 71.1]	10 (41.7) [24.6, 60.3]
NE	0	0	1 (25.0)	2 (25.0)	6 (25.0)
DCR [90% CI]	1 (50.0) [2.5, 97.5]	1 (50.0) [2.5, 97.5]	2 (50.0) [9.8, 90.2]	3 (37.5) [11.1, 71.1]	8 (33.3) [17.8, 52.1]
DOR (range), mo	NE	NE	NE	3.9 (3.9-3.9)	3.9 (3.9-3.9)

Data presented are n (%) except where otherwise indicated.

CR, complete response; CRC, colorectal cancer; DCR, disease control rate; DOR, duration of response; NE, unevaluable or missing; PD, progressive disease; PR, partial response; SD, stable disease; ORR, overall response.

Supplementary Table 7. Efficacy in S2 in patients with diagnoses other than CRC

	**Bile duct (n=2)**	**Breast (n=2)**	**Lung (n=3)**	**Pancreatic (n=17)**	**Gastric (n=12)**
ORR, n (%) [90% CI]	0 [0.0, 77.6]	0 [0.0, 77.6]	0 [0.0, 63.2]	0 [0.0, 16.2]	1 (8.3) [0.4, 33.9]
CR, n (%) [90% CI]	0 [0.0, 77.6]	0 [0.0, 77.6]	0 [0.0, 63.2]	0 [0.0, 16.2]	0 [0.0, 22.1]
PR, n (%) [90% CI]	0 [0.0, 77.6]	0 [0.0, 77.6]	0 [0.0, 63.2]	0 [0.0, 16.2]	1 (8.3) [0.4, 33.9]
SD, n (%) [90% CI]	1 (50.0) [2.5, 97.5]	1 (50.0) [2.5, 97.5]	1 (33.3) [1.7, 86.5]	4 (23.5) [8.5, 46.1]	4 (33.3) [12.3, 60.9]
PD, n (%) [90% CI]	1 (50.0) [2.5, 97.5]	1 (50.0) [2.5, 97.5]	2 (66.7) [13.5, 98.3]	10 (58.8) [36.4, 78.8]	3 (25.0) [7.2, 52.7]
NE, n (%)	0	0	0	3 (17.6)	4 (33.3)
DCR, n (%) [90% CI]	1 (50.0) [2.5, 97.5]	1 (50.0) [2.5, 97.5]	1 (33.3) [1.7, 86.5]	4 (23.5) [8.5, 46.1]	5 (41.7) [18.1, 68.5]
DOR (range), mo	NE	NE	NE	NE	20.3 (20.3-20.3)

CR, complete response; CRC, colorectal cancer; DCR, disease control rate; DOR, duration of response; NE, unevaluable or missing; PD, progressive disease; PR, partial response; SD, stable disease; ORR, overall response.

Supplementary Table 8. Efficacy for all patients and patients with MSS-CRC, broken down by sex

	**All (n=228)**	**All MSS-CRC (n=187)**	**All female (n=96)**	**All female MSS-CRC (n=76)**	**All male (n=132)**	**All male MSS-CRC (n=111)**
ORR, n (%)<br>90% CI	15 (6.6)<br>[4.1, 9.9]	13 (7.0)<br>[4.2, 10.8)	5 (5.2)<br>[2.1, 10.6]	4 (5.3)<br>[1.8, 11.6)	10 (7.6)<br>[4.2, 12.5]	9 (8.1)<br>[4.3, 13.7)
CR, n (%)<br>90% CI	1 (0.4)<br>[0.0, 2.1]	0<br>[0.0, 1.6]	1 (1.0)<br>[0.1, 4.8]	0<br>[0.0, 3.9]	0<br>[0.0, 2.2]	0<br>[0.0, 2.7]
PR, n (%)<br>90% CI	14 (6.1)<br>[3.8, 9.4]	13 (7.0)<br>[4.2, 10.8]	4 (4.2)<br>[1.4, 9.3]	4 (5.3)<br>[1.8, 11.6]	10 (7.6)<br>[4.2, 12.5]	9 (8.1)<br>[4.3, 13.7]
SD, n (%)<br>90% CI	79 (34.6)<br>[29.4, 40.2]	68 (36.4)<br>[30.5, 42.6]	39 (40.6)<br>[32.2, 49.5]	33 (43.4)<br>[33.7, 53.5]	40 (30.3)<br>[23.7, 37.6]	35 (31.5)<br>[24.3, 39.6]
PD, n (%)<br>90% CI	109 (47.8)<br>[42.2, 53.5]	88 (47.1)<br>[40.9, 53.3]	43 (44.8)<br>[36.1, 53.7]	32 (42.1)<br>[32.5, 52.2]	66 (50.0)<br>[42.5, 57.5]	56 (50.5)<br>[42.3, 58.6]
NE, n (%)	25 (11.0)	18 (9.6)	9 (9.4)	7 (9.2)	16 (12.1)	11 (9.9)
DCR, n (%)<br>90% CI	94 (41.2)<br>[35.8, 46.9]	81 (43.3)<br>[37.2, 49.6]	44 (45.8)<br>[37.1, 54.7]	37 (48.7)<br>[38.8, 58.7]	50 (37.9)<br>[30.8, 45.4]	44 (39.6)<br>[31.8, 47.9]
DOR range, mo	1.9-35.6	1.9-28.6	1.9-35.6	1.9-6.9	3.6-28.6	3.6-28.6

CR, complete response; DCR, disease control rate; DOR, duration of response; MSS-CRC, microsatellite-stable colorectal cancer; NA, not applicable; NE, unevaluable or missing; PD, progressive disease; PR, partial response; SD, stable disease; ORR, overall response.

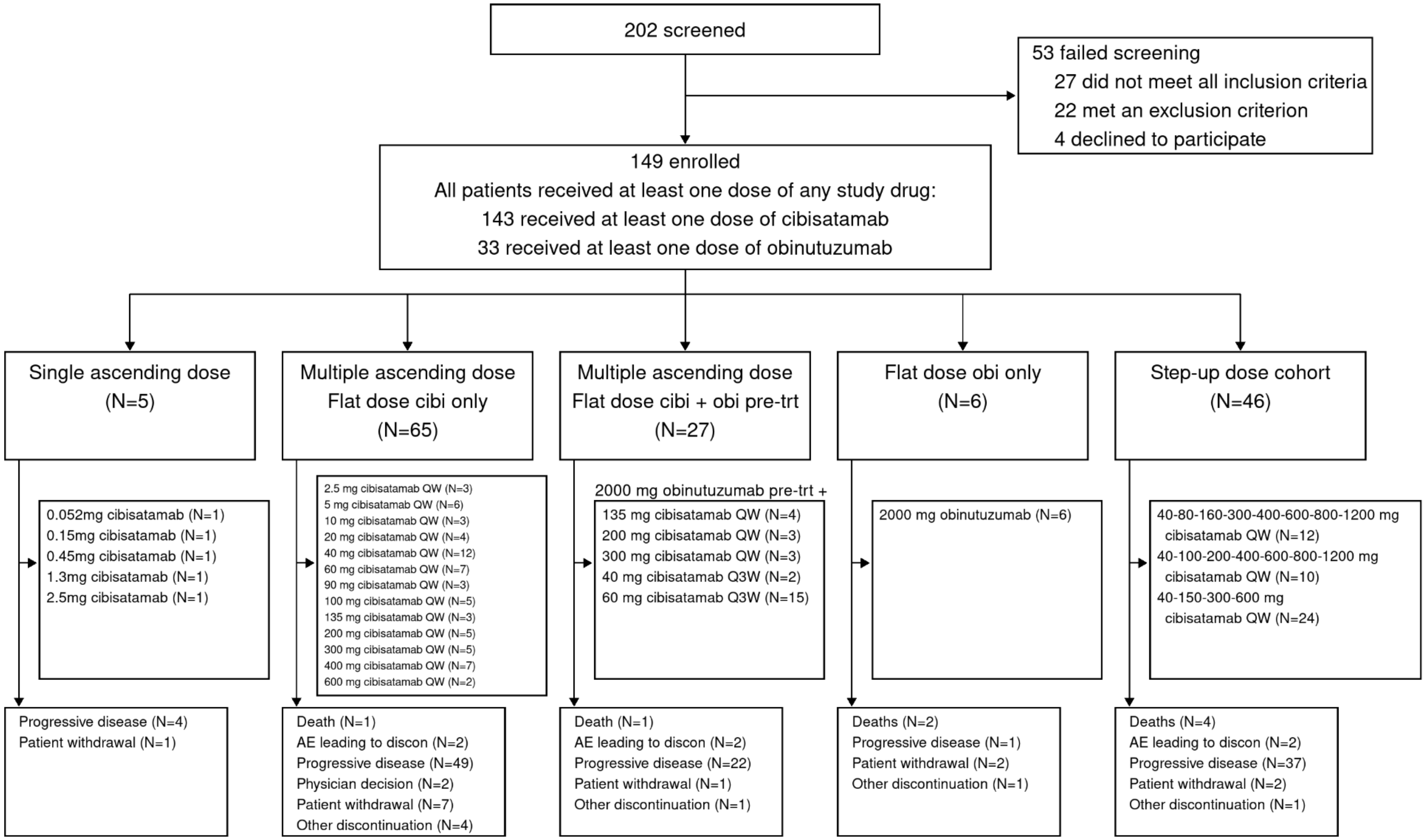

Supplementary Figure 1. CONSORT diagrams for study S1 (BP29541). AE, adverse event; atezo, atezolizumab; cibi, cibisatamab; CRC, colorectal cancer; obi, obinutuzumab; Sympt, symptomatic; Q3W, once every 3 weeks; QW, once weekly.

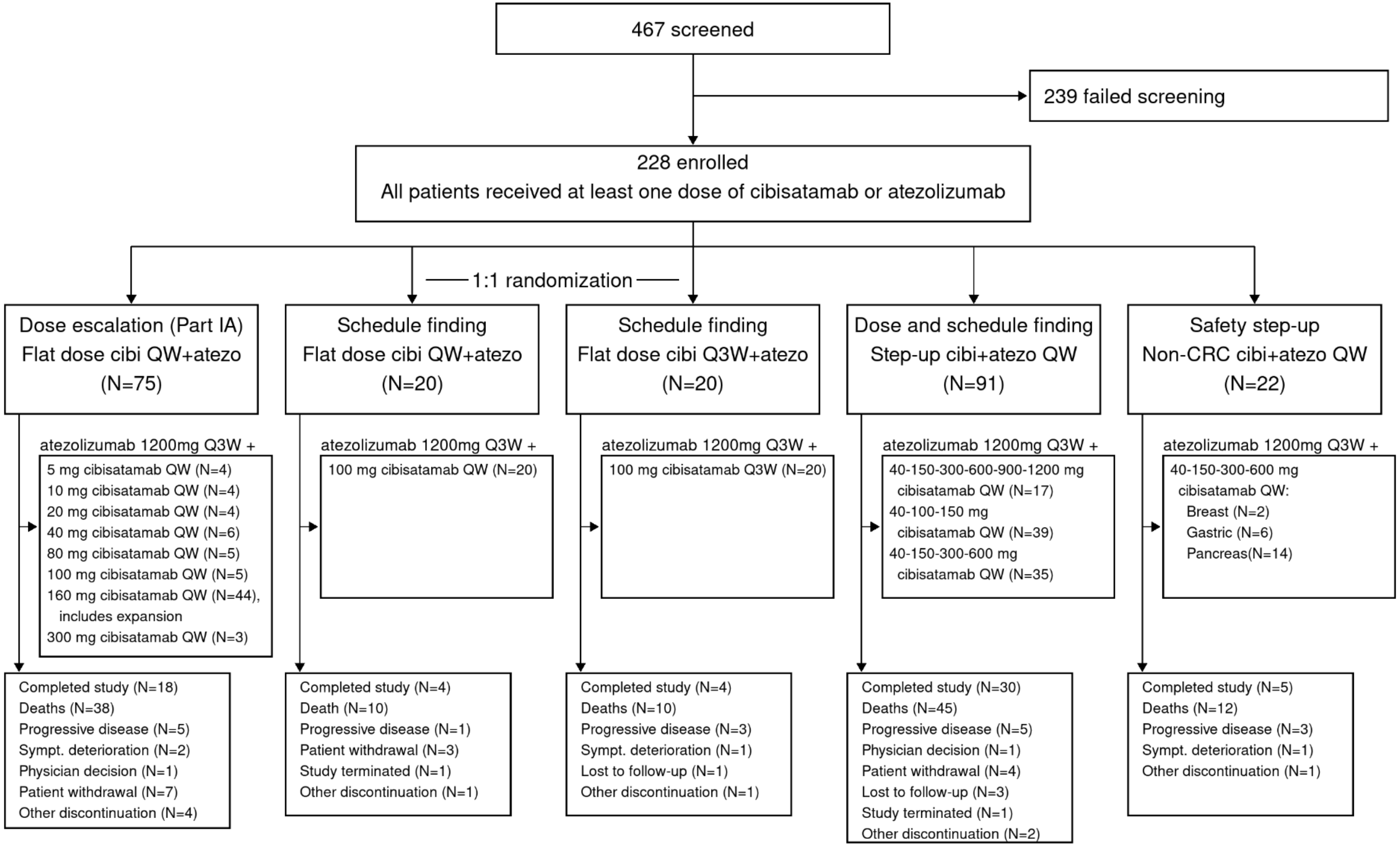

Supplementary Figure 2. CONSORT diagrams for study S2 (WP29945). AE, adverse event; atezo, atezolizumab; cibi, cibisatamab; CRC, colorectal cancer; obi, obinutuzumab; Sympt, symptomatic; Q3W, once every 3 weeks; QW, once weekly.

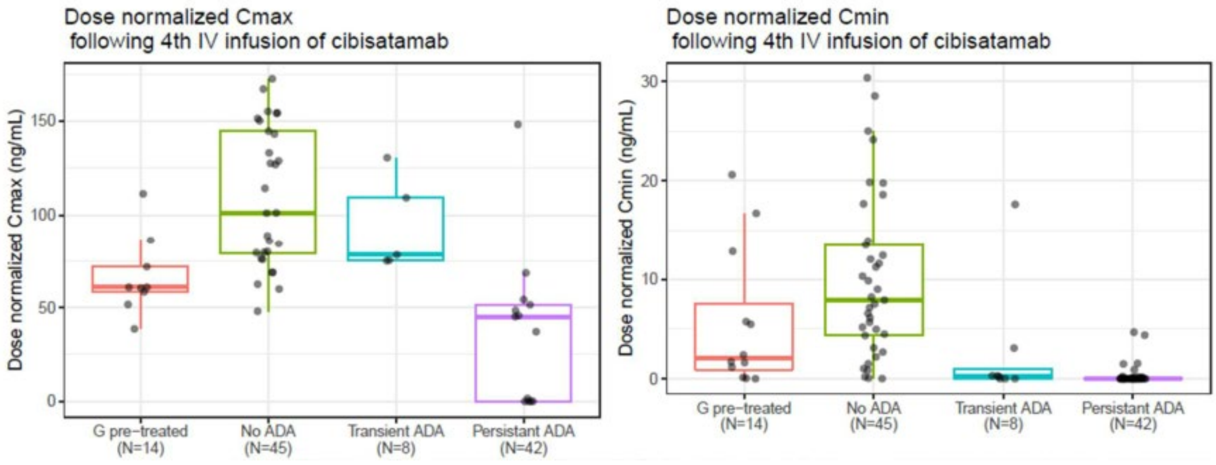

Supplementary Figure 3. Impact of obinutuzumab pretreatment and ADA on observed minimum and maximal serum concentrations of cibisatamab following fourth IV infusion (S1 [BP29541]). G, obinutuzumab; IV, intravenous; QW, once a week; Q3W, every three weeks. For each box and whisker plot, the median value is indicated, and error bars refer to the first and third quartiles. In both panels, red colour refers to G pre-treated patients, green to patients with no ADAs, blue to patients with transient ADAs, and purple to patients with persistent ADA. Patients within the No ADA, Transient ADA, and Persistent ADA subgroups were not pre-treated with G. Dose normalised Cmin and Cmax are shown after the 4th infusion of cibisatamab (i.e., at week 9 for Q3W dosing and week 4 for QW dosing).

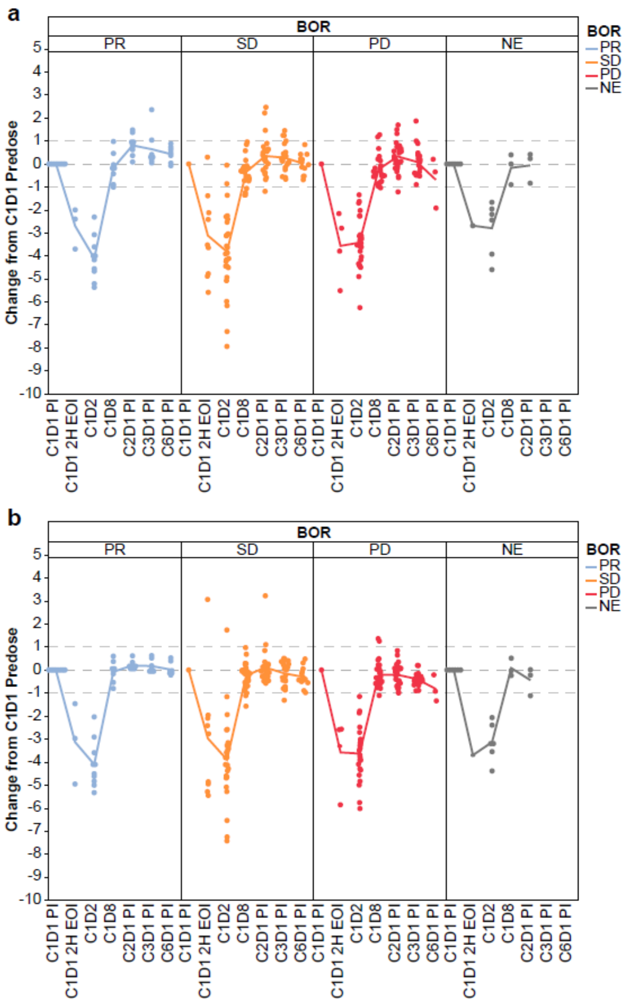

Supplementary Figure 4. Blood flow data showing changes from baseline in patients receiving cibisatamab. a peripheral CD8+ cells changes and **b** peripheral CD4+ cells are shown. Both subfigures show 659 samples for 185 CRC MSS patients, with 2 samples at C1D1 pre-infusion, 34 at C1D1 2 hours after infusion, 159 at C1D2, 162 at C1D8 pre-infusion or 168h follow-up, 147 at C2D1 pre-infusion, 104 at C3D1 pre-infusion, and 51 at C6D1 pre-infusion. Quantitative flow cytometry was performed with the ARUP Laboratories Lymphocyte Subset Panel 3—T-Cell Subsets (CD4 and CD8), Absolute Counts Only (0095853) assay. BOR, best overall response; NE, unevaluable or missing; PD, progressive disease; PR, partial response; SD, stable disease.

PROTOCOL

**TITLE:**	**AN OPEN-LABEL, MULTICENTER, DOSE-ESCALATION PHASE I STUDY TO EVALUATE THE SAFETY, PHARMACOKINETICS, AND THERAPEUTIC ACTIVITY OF RO6958688, A NOVEL T-CELL BISPECIFIC ANTIBODY THAT TARGETS THE HUMAN CARCINOEMBRYONIC ANTIGEN (CEA) ON TUMOR CELLS AND CD3 ON T CELLS, ADMINISTERED INTRAVENOUSLY IN PATIENTS WITH LOCALLY ADVANCED AND/OR METASTATIC CEA($^{+}$) SOLID TUMORS**
**PROTOCOL NUMBER:**	BP29541
**VERSION:**	12
**EUDRACT NUMBER:**	2014-003075-30
**IND NUMBER:**	122931
**TEST PRODUCT:**	RO6958688
**MEDICAL MONITOR:**	[redacted], M.D., Ph.D.
**SPONSOR:**	F. Hoffmann-La Roche Ltd
**DATE FINAL:**	See electronic date stamp below.

FINAL PROTOCOL APPROVAL

CONFIDENTIAL STATEMENT

The information contained in this document, especially any unpublished data, is the property of F. Hoffmann-La Roche Ltd (or under its control) and therefore is provided to you in confidence as an investigator, potential investigator, or consultant, for review by you, your staff, and an applicable Ethics Committee or Institutional Review Board. It is understood that this information will not be disclosed to others without written authorization from Roche except to the extent necessary to obtain informed consent from persons to whom the drug may be administered.

“Use of this document is governed by the terms of use on the first page of this document.”

PROTOCOL AMENDMENT, VERSION 12:
RATIONALE

Protocol BP29541 has been primarily amended to add a mandatory 8-hour observation period after the end of infusion at the subsequent treatment administration visit for situations in which a patient experiences a treatment-related Grade ≥ 3 adverse event within 24 hours of the previous infusion. For Grade 3 infusion-related reaction (IRR)/cytokine release syndrome (CRS), this observation period will be at least 24 hours after the end of infusion. The amendment also aims to clarify that in the event of an urgent safety situation, the use of commercial tocilizumab is permitted. The amendment also aims to cross reference the Tocilizumab Investigator's Brochure in order to bring awareness to the risks related to tocilizumab use.

Changes to the protocol, along with a rationale for each change, are summarized below:

- The secondary objective of PFS on-treatment was changed to standard PFS according to Response Evaluation Criteria in Solid Tumors, Version 1.1 (RECIST v1.1) (Sections 2.2, 3.3.3, and 6).
- The use of commercial tocilizumab will be permitted in the event of an urgent situation where study-specific labeled supply is not accessible. This is to ensure prompt availability of tocilizumab to protect patient safety in the event of an emergency (Section 4.3.1.3).
- Language has been added to clarify that, after withdrawal of consent for participation in the Roche Clinical Repository (RCR), remaining RCR samples will be destroyed or will no longer linked to the patient (Section 4.5.1.7).
- Instructions about patient withdrawal from the RCR after site closure have been modified to indicate that the investigator must inform the Sponsor of patient withdrawal by emailing the study number and patient number to global_rcr-withdrawal@roche.com (Section 4.5.1.7).
- A mandatory 8-hour observation period has been added after the RO6958688 administration at any cycle after which the patient had experienced a Grade 3 or higher adverse event (with the exception of Grade 3 IRR/CRS) within 24 hours of the infusion to ensure more thorough patient monitoring during the study; and a 24-hour observation period following any cycle after which the patient experienced a Grade 3 IRR/CRS (Sections 4.5.2.2, 5.2.1, and 5.2.5.1.5 and Appendix 1).
- A reference has been added to Section 6 of the Tocilizumab Investigator's Brochure (Guidance for the Investigator including Reference Safety Information) for identified risks (Section 5.2.3).
- The adverse event reporting period definition has been modified to include the following: "After initiation of any study drug, all adverse events, regardless of relationship to any of the study drugs, will be reported until 28 days after the last dose of RO6958688 or obinutuzumab if for any reason the patient does not receive any RO6958688 infusion *or until initiation of new systemic anti-cancer therapy, whichever occurs first*" (Section 5.3.1).

"Use of this document is governed by the terms of use on the first page of this document."

- Text has been modified to account for the fact that special situations (i.e., overdoses, and medication errors) are not required to be reported within 24 hours (Sections 5.3.5.11 and 5.4). Note that serious adverse events associated with special situations are still required to be reported within 24 hours. The special situations section has been relocated to Section 5.3.5.11.
- Language has been updated to indicate that therapeutic or elective abortions are not considered adverse events unless performed because of an underlying maternal or embryofetal toxicity. In such cases, the underlying toxicity should be reported as a serious adverse event. Language has also been added to clarify that all abortions are to be reported on the paper Clinical Trial Pregnancy Reporting Form (Section 5.4.4.3).
- In Sections 6.1 and 6.2 and throughout Section 6, the primary and secondary study variables have been aligned with the primary and secondary study objectives as outlined in Sections 2.1 and 2.2 and brought in line with RECIST 1.1 standard definitions.
- It has been clarified that Roche standard reference ranges for lab values are only used where appropriate (Section 6.7.3).
- It has been clarified that the focus of listings is clinically abnormal vital signs, ECG, and other safety data. Concomitant medications will be presented in summary tables and listings as appropriate (Section 6.7).
- As no Statistical Analysis Plan is expected to be produced for this study, additional clarifications and minor changes have been implemented to the statistical analysis strategy throughout Section 6.
- Language has been added for consistency with Roche's current data retention policy and to accommodate more stringent local requirements (if applicable) (Section 7.5).
- Language has been added to indicate that the study will comply with applicable local, regional, and national laws (Section 8.1).

Additional minor changes have been made to improve clarity and consistency. Substantive new information appears in italics. This amendment represents cumulative changes to the original protocol.

“Use of this document is governed by the terms of use on the first page of this document.”

TABLE OF CONTENTS

PROTOCOL ACCEPTANCE FORM 13

SYNOPSIS OF PROTOCOL NUMBER BP29541 14

1. BACKGROUND AND RATIONALE 43
 1.1 Background and Scientific Rationale 43
 1.2 Background on RO6958688 45
 1.2.1 Nonclinical Pharmacology Studies 46
 1.2.2 Previous and ongoing Clinical Studies 47
 1.2.2.1 Safety of RO6958688 47
 1.2.2.2 Efficacy of RO6958688 51
 1.2.2.3 Clinical Pharmacokinetics of RO6958688 53
 1.2.2.4 Immunogenicity of RO6958688 53
 1.3 Background on obinutuzumab 54
 1.3.1 Previous Nonclinical Studies 54
 1.3.2 Previous and ongoing Clinical Studies 55
 1.3.2.1 Studies in B-cell malignancies with obinutuzumab 55
 1.3.2.2 Clinical Studies in solid tumors with obinutuzumab 56
 1.4 Background on Tocilizumab (RO4877533, Actemra, RoActemra) 58
 1.5 Study Rationale and Benefit-Risk Assessment 59
 1.5.1 Identified Risks with RO6958688 65
 1.5.2 Rationale for [18F] FDG-PET Imaging 66
 1.5.3 Rationale for the new RO6958688 dose schedules and for the step up dosing 66

2. OBJECTIVES 68
 2.1 Primary Objectives 68
 2.2 Secondary Objectives 68
 2.3 Exploratory Objectives (Part II only) 69

3. STUDY DESIGN 69
 3.1 Description of Study 69
 3.1.1 Overview of Study Design 71

"Use of this document is governed by the terms of use on the first page of this document."

3.1.2 Dose Escalation Decision Criteria 77
3.1.2.1 Escalation Criteria (Part I)... 77
3.1.2.2 Escalation Criteria (Part II)—Dose Limiting Toxicities Applicable for Part II 77
3.1.3 Communication Strategy 79
3.1.4 End of Study 80
3.2 Rationale for Study Design 80
3.2.1 Rationale for RO6958688 Dosage Selection 80
3.2.2 Rationale for obinutuzumab dose 81
3.2.3 Rationale for the Treatment of Severe Cytokine Release Syndrome (CRS) Using Tocilizumab 81
3.2.4 Rationale for Study Population 83
3.2.5 Rationale for Biomarker Assessments................................... 83
3.2.6 Rationale for Statistical Design 85
3.2.7 Rationale for characterization of Anti-Drug Antibodies directed against RO6958688 on a B cell/antibody level in individual patients 86
3.3 Outcome Measures 87
3.3.1 Safety Outcome Measures 87
3.3.2 Pharmacokinetic and Pharmacodynamic Outcome Measures 87
3.3.2.1 Pharmacokinetic Outcome Measures 87
3.3.2.2 Pharmacodynamic Outcome Measures 88
3.3.3 Efficacy Outcome Measures 89
3.3.4 Exploratory Outcome Measures 90

4. MATERIALS AND METHODS 91
4.1 Center 91
4.2 Study Population 91
4.2.1 Recruitment and Enrollment Procedures 91
4.2.2 Inclusion Criteria 92
4.2.3 Exclusion Criteria 94
4.3 Study Treatments 96
4.3.1 Formulation, Packaging, and Handling 96
4.3.1.1 RO6958688 97

"Use of this document is governed by the terms of use on the first page of this document."

4.3.1.2 Preparation of obinutuzumab 97
4.3.1.3 Tocilizumab 98
4.3.2 Administration 98
4.3.2.1 Administration of RO6958688, obinutuzumab and tocilizumab 98
4.3.2.2 Pre-medication and Post-medication for RO6958688 104
4.3.2.3 Infusion of RO6958688 105
4.3.3 Investigational Medicinal Product Accountability 106
4.3.4 Post-Trial Access to RO6958688 107
4.4 Concomitant Therapy 107
4.4.1 Permitted Therapy 107
4.4.2 Prohibited Therapy 108
4.5 Study Assessments 108
4.5.1 Description of Study Assessments 108
4.5.1.1 Medical History and Demographic Data 109
4.5.1.2 Physical Examinations, Vital Signs, and ECOG Performance Status 109
4.5.1.3 Electrocardiograms 110
4.5.1.4 Pulmonary function tests (FEV1/VC/TLC and DL_{CO}) 110
4.5.1.5 Laboratory Assessments 111
4.5.1.6 Additional Samples 112
4.5.1.7 Samples for Roche Clinical Repository 120
4.5.2 Timing of Study Assessments 123
4.5.2.1 Screening and Pretreatment Assessments 123
4.5.2.2 Assessments during Treatment 124
4.5.2.3 Follow-Up Assessments and Assessments at Study Completion/Early Termination Visit 125
4.6 Patient, Study, and Site Discontinuation 126
4.6.1 Patient Discontinuation 126
4.6.1.1 Discontinuation from Study Drugs 126
4.6.1.2 Withdrawal from Study 127
4.6.2 Study and Site Discontinuation 128

“Use of this document is governed by the terms of use on the first page of this document.”

5. ASSESSMENT OF SAFETY 128
5.1 Safety Parameters and Definitions 128
5.1.1 Adverse Events 128
5.1.2 Serious Adverse Events (Immediately Reportable to the Sponsor) 129
5.1.3 Non-Serious Adverse Events of Special Interest (Immediately Reportable to the Sponsor) 130
5.2 Safety Plan 130
5.2.1 Risks associated with RO6958688 130
5.2.2 Risks associated with Obinutuzumab 131
5.2.3 *Risks associated with Tocilizumab* 131
5.2.4 Dose Modifications and Delays 132
5.2.5 Management of Specific Adverse Events 133
5.2.5.1 Management of Specific Adverse Events related to RO6958688 133
5.2.5.2 Management of Specific Adverse Events related to obinutuzumab 145
5.2.6 Management of Specific Adverse Events related to RO6958688 and obinutuzumab 146
5.2.6.1 Infusion-Related Reactions/Cytokine Release Syndrome 146
5.3 Methods and Timing for Capturing and Assessing Safety Parameters 148
5.3.1 Adverse Event Reporting Period 148
5.3.2 Eliciting Adverse Event Information 148
5.3.3 Assessment of Severity of Adverse Events 149
5.3.4 Assessment of Causality of Adverse Events 149
5.3.5 Procedures for Recording Adverse Events 150
5.3.5.1 Infusion-Related Reactions/Hypersensitivity reactions and Cytokine Release Syndrome Attributed to RO6958688 150
5.3.5.2 Adverse Events Occurring Secondary to Other Events 151
5.3.5.3 Persistent or Recurrent Adverse Events 151
5.3.5.4 Abnormal Laboratory Values 152
5.3.5.5 Abnormal Vital Sign Values 152

"Use of this document is governed by the terms of use on the first page of this document."

5.3.5.6 Abnormal Liver Function Tests 153
5.3.5.7 Deaths 153
5.3.5.8 Preexisting Medical Conditions 154
5.3.5.9 Lack of Efficacy or Worsening of Cancer 154
5.3.5.10 Hospitalization or Prolonged Hospitalization 154
5.3.5.11 *Cases of Accidental Overdose or Medication Error* 155
5.4 Immediate Reporting Requirements from Investigator to Sponsor 156
5.4.1 Emergency Medical Contacts 156
5.4.2 Reporting Requirements for Serious Adverse Events and Non-Serious Adverse Events of Special Interest 156
5.4.3 Reporting Requirements for Pregnancies 156
5.4.3.1 Pregnancies 156
5.4.3.2 Pregnancies in Female Partners of Male Patient 157
5.4.3.3 Abortions 157
5.4.3.4 Congenital Anomalies/Birth Defects 157
5.5 Follow-Up of Patients after Adverse Events 158
5.5.1 Investigator Follow-Up 158
5.5.2 Sponsor Follow-Up 158
5.6 Post-Study Adverse Events 158
5.7 Expedited Reporting to Health Authorities, Investigators, Institutional Review Boards, and Ethics Committees 159

6. STATISTICAL CONSIDERATIONS AND ANALYSIS PLAN 159
6.1 Primary study variables 160
6.2 Secondary study variables 160
6.3 Determination of Sample Size 160
6.3.1 Sample size for the dose escalation part 160
6.3.2 Sample size for cohorts C-H 161
6.4 Summaries of Conduct of Study 162
6.5 Analysis Populations 162
6.5.1 Safety Analysis Population 162

“Use of this document is governed by the terms of use on the first page of this document.”

6.5.2 Pharmacokinetic Analysis Population 162
6.5.3 Pharmacodynamic Analysis Population 162
6.5.4 Efficacy Analysis Population 162
6.6 Summaries of Treatment Group Comparability 162
6.7 Safety Analyses 163
6.7.1 Dose-Escalation Approach 163
6.7.1.1 Modified-Continual Reassessment Method with Overdose Control 163
6.7.1.2 Modified 3+3 dose escalation design 170
6.7.2 Adverse Events 171
6.7.3 Clinical Laboratory Test Results 172
6.7.3.1 Standard Reference Ranges and Transformation of Data 172
6.7.3.2 Definition of Laboratory Abnormalities 172
6.7.4 Vital Signs 173
6.7.5 ECG Data Analysis 173
6.7.6 Concomitant Medications 173
6.8 Efficacy Analyses 173
6.8.1 Primary Efficacy Endpoint 173
6.8.2 Secondary Efficacy Endpoints 174
6.9 Pharmacodynamic Analyses 174
6.10 Pharmacokinetic Analyses 174
6.11 Immunogenicity Analyses 175
6.12 Interim Analyses 175

7. DATA COLLECTION AND MANAGEMENT 175
7.1 Data Quality Assurance 175
7.2 Electronic Case Report Forms 176
7.3 Source Data Documentation 176
7.4 Use of Computerized Systems 177
7.5 Retention of Records 177

8. ETHICAL CONSIDERATIONS 178
8.1 Compliance with Laws and Regulations 178
8.2 Informed Consent 178

"Use of this document is governed by the terms of use on the first page of this document."

8.3 Institutional Review Board or Ethics Committee 179
8.4 Confidentiality 179
8.5 Financial Disclosure 180

9. STUDY DOCUMENTATION, MONITORING, AND ADMINISTRATION 180
9.1 Study Documentation 180
9.2 Site Inspections 180
9.3 Administrative Structure.. 181
9.4 Publication of Data and Protection of Trade Secrets 181
9.5 Protocol Amendments 182

10. REFERENCES 183

"Use of this document is governed by the terms of use on the first page of this document."

LIST OF TABLES

Table 1 Obinutuzumab and RO6958688: Overview of Identified and Potential Risks with Treatment 64
Table 2 Biopsy time points per cohort 84
Table 3 Obinutuzumab Infusion Instructions (2000 mg infusion in one day) 101
Table 4 Obinutuzumab Infusion Instructions (1000 mg infusion in two consecutive days) 103
Table 5 Pre-medications to be administered before RO6958688 infusion 104
Table 6 Guidelines for Management of Pulmonary Events 135
Table 7 Recommendations for management of gastrointestinal events considered related to RO6958688 136
Table 8 Monitoring of patients with increased AST/ALT while on RO6958688 138
Table 9 Cytokine Release Syndrome grading according to NCI CTCAE v5 139
Table 10 Recommendations for Management of Infusion-Related Reactions and Cytokine Release Syndrome related to RO6958688[a] 141
Table 11 Adverse Event Severity Grading Scale 149
Table 12 Operating Characteristics of the mCRM with EWOC Design with Respect to the Chosen Scenarios 161
Table 13 Hypothetical recommendations of the mCRM with EWOC design 167

LIST OF FIGURES

Figure 1 Carcinoembryonic Antigen Expression across Various Tumor Types 44
Figure 2 Design, Structure, and Required Characteristics of RO6958688 46
Figure 3 Prior treatment with obinutuzumab but not rituximab or vehicle results in the attenuation of tetanus toxoid specific de novo IgG antibody responses in cynomolgus monkeys 62
Figure 4 Memory recall responses by measles specific IgG antibody production in response to immune re-challenge with a measles/rubella booster vaccination in animals with baseline titers to measles is not affected by either obinutuzumab or rituximab in cynomolgus monkeys 63
Figure 5 QW step up dosing scheme and late cycle MTD 72
Figure 6 Study Schema 73
Figure 7 Prior Model for the mCRM with EWOC Design 166

"Use of this document is governed by the terms of use on the first page of this document."

LIST OF APPENDICES

Appendix 1 Schedule of Assessments 188
Appendix 2 CKD-EPI equation for Calculation of glomerular filtration rate (GFR) 221
Appendix 3 Eastern Cooperative Oncology Group Performance Status 222
Appendix 4 Response Evaluation Criteria in Solid Tumors Version 1.1 Criteria 223
Appendix 5 Unidimensional Immune–Related Response Criteria (Unidimensional irRC) 228
Appendix 6 Statistical Design of modified Continual Reassessment Method with Escalation with Overdose Control 236
Appendix 7 Modified 3+3 dose escalation design simulation results 249
Appendix 8 GILBERT'S SYNDROME DEFINITION 252
Appendix 9 [^{18}F] FDG-PET 253
Appendix 10 Schedule of Assessments for Tocilizumab Treatment of Severe or Life-Threatening Cytokine Release Syndrome 254
Appendix 11 High Dose Vasopressors 256

"Use of this document is governed by the terms of use on the first page of this document."

PROTOCOL ACCEPTANCE FORM

**TITLE:**	**AN OPEN-LABEL, MULTICENTER, DOSE-ESCALATION PHASE I STUDY TO EVALUATE THE SAFETY, PHARMACOKINETICS, AND THERAPEUTIC ACTIVITY OF RO6958688, A NOVEL T-CELL BISPECIFIC ANTIBODY THAT TARGETS THE HUMAN CARCINOEMBRYONIC ANTIGEN (CEA) ON TUMOR CELLS AND CD3 ON T-CELLS, ADMINISTERED INTRAVENOUSLY IN PATIENTS WITH LOCALLY ADVANCED AND/OR METASTATIC CEA($^{+}$) SOLID TUMORS**
**PROTOCOL NUMBER:**	BP29541
**VERSION NUMBER:**	12
**EUDRACT NUMBER:**	2014-003075-30
**IND NUMBER:**	122931
**TEST PRODUCT:**	RO6958688
**MEDICAL MONITOR:**	[redacted], M.D., Ph.D.
**SPONSOR:**	F. Hoffmann-La Roche Ltd

I agree to conduct the study in accordance with the current protocol.

____

Principal Investigator’s Name (print)

____ ____________________

Principal Investigator’s Signature Date

Please keep the signed original form in your study files, and return a copy to your local study monitor.

“Use of this document is governed by the terms of use on the first page of this document.”

SYNOPSIS OF PROTOCOL NUMBER BP29541

**TITLE**	**AN OPEN-LABEL, MULTICENTER, DOSE-ESCALATION PHASE I STUDY TO EVALUATE THE SAFETY, PHARMACOKINETICS, AND THERAPEUTIC ACTIVITY OF RO6958688, A NOVEL T-CELL BISPECIFIC ANTIBODY THAT TARGETS THE HUMAN CARCINOEMBRYONIC ANTIGEN (CEA) ON TUMOR CELLS AND CD3 ON T CELLS, ADMINISTERED INTRAVENOUSLY IN PATIENTS WITH LOCALLY ADVANCED AND/OR METASTATIC CEA($^{+}$) SOLID TUMORS**
**PROTOCOL NUMBER:**	BP29541
**VERSION:**	12
**EUDRACT NUMBER:**	2014-003075-30
**IND NUMBER:**	122931
**TEST PRODUCT:**	RO6958688
**PHASE:**	Ia/b
**INDICATION:**	Patients with locally advanced and/or metastatic CEA-positive solid tumors who have progressed on standard treatment, are intolerant to standard of care (SOC), and/or are non-amenable to SOC
**Medical Monitor:**	[redacted], M.D., Ph.D.
**SPONSOR:**	F. Hoffmann-La Roche Ltd

“Use of this document is governed by the terms of use on the first page of this document.”

OBJECTIVES

Primary Objectives

The primary objectives of this study are:

- To assess the safety profile of RO6958688 with/without obinutuzumab pretreatment
- To determine the maximum-tolerated dose (MTD) and/or the recommended dose and schedule (optionally with obinutuzumab pretreatment) for further development
- To determine the late cycle maximum tolerated dose (late cycle MTD)
- To establish the pharmacokinetics of RO6958688 as monotherapy with/without obinutuzumab pretreatment
- To assess the effect of obinutuzumab pretreatment in decreasing the rate of patients with positive Anti-Drug Antibodies (ADA) titer against RO6958688 at week 8 and/or delaying the time of onset of ADA against RO6958688

Secondary Objectives

The secondary objectives for this study are:

- To obtain preliminary anti-tumor activity data of RO6958688 with/without obinutuzumab pretreatment on objective overall response rate (ORR), disease control rate (DCR; defined as response rate [RR] + stable disease [SD]) and progression-free survival (PFS) according to Response Evaluation Criteria in Solid Tumors (RECIST), Version 1.1 criteria and immune–related response criteria (irRC), by investigator assessment. If Sponsor decides, independent central read for computed tomography (CT) or magnetic resonance imaging (MRI) might be performed in this study, both prospectively and retrospectively.
- To characterize pharmacodynamic (PD) effects and duration of PD response for the weekly (QW)**,** for the Q3W regimens and for the step up dosing scheme (QW and QWx3/Q3W) on the basis of an increase in activated intratumoral T cells.

Exploratory Objectives (Part II only)

The exploratory objectives for this study are:

- To explore the relationship between exposure, pharmacodynamics, and clinical effects of RO6958688 with/without obinutuzumab pretreatment
- To characterize in tumors and peripheral blood cells the changes in PD biomarkers (proliferation, activation, infiltration) associated with multiple doses and schedules of RO6958688 treatment
- To investigate potential predictive PD biomarkers from paired tumor biopsies and paired blood samples (including but not limited to $CD3^+$, $CD4^+$, $CD8^+$, and T cells)
- To explore the relationship between exposure, metabolic activity by the tumor, pharmacodynamics, and clinical effects of RO6958688.
- To investigate tumor mutations, gene expression and other biomarkers (such as CEA expression in various tumor types) related to RO6958688 therapy
- To explore preliminary safety and efficacy in low/moderate and very low CEA expressing tumors.
- To assess ADA specificity in patients without obinutuzumab pretreatment.
- To make a preliminary assessment of the effectiveness of tocilizumab (Actemra®/RoActemra®) in ameliorating the symptoms of severe CRS following RO6958688 treatment.

"Use of this document is governed by the terms of use on the first page of this document."

STUDY DESIGN

Note: Following an internal review of the clinical development plan of RO6958688, the Sponsor has decided to permanently discontinue further enrollment of patients in this study and to not open enrollment of Cohorts D through H and the Biomarker Cohort.

Description of Study

Study BP29541 is a first in-human, open-label, multicenter, dose-escalation Phase I clinical study of single-agent RO6958688. The study will be conducted in two parts. Part I of the study is single ascending dose in single patient cohorts to evaluate the safety of RO6958688 at the doses that are expected to be below relevant biological effects (starting from a receptor occupancy of 0.11% for the CD3 epsilon chain receptors), and Part II is multiple ascending dose with a dose-finding part where RO6958688 is given QW to define the MTD and/or the recommended dose for further development. For week 1 (and week 2 in the QW schedule) MTD has been defined as 400 mg. Sponsor will open, in Part II, parallel multiple ascending dose escalation cohorts of patients receiving obinutuzumab pre-treatment. QW dosing of RO6958688 (± 1 day) will be implemented initially to generate data that can be analyzed to assess whether different dosing schedules are more effective. Cohorts of patients pretreated with obinutuzumab either with QW flat dose, Q3W flat dose or step up dosing scheme will also be enrolled, in order to assess if obinutuzumab pretreatment would decrease the incidence and/or delay the onset of ADA directed against RO6958688. Interim data from another study (BP28920/obinutuzumab) with another molecule developed by the Sponsor (RO6895882), from BP29435 where RO6895882 was administered with atezolizumab 7 days after receiving obinutuzumab pretreatment and from this ongoing BP29541 study is suggestive that a single dose of 2000 mg of obinutuzumab may be sufficient to prevent or delay ADA formation. There will be a parallel multiple ascending dose escalation in cohorts of patients receiving obinutuzumab pretreatment. QW dosing of RO6958688 (± 1 day) will be implemented initially to generate data that can be analyzed to assess whether different dosing schedules are more effective. For week 1 (and week 2 in the QW schedule) MTD has been defined as 400 mg. This will define the late cycle MTD and the maximum intra-patient dose escalation increments by a modified 3+3 dose escalation in two cohorts of patients, the cohorts will be run in a staggered fashion with Cohort A (late cycle MTD) starting before Cohort B (maximum increments) and cohort B will not be escalated to a dose not already cleared as safe in cohort A:

Cohort A) approximately 6 evaluable patients at 40 mg in cycle 1 without prophylactic steroids and escalating QW by up to 100% of the previous dose until the DLT criteria for that dose level are met.

Cohort B) approximately 6 evaluable patients at 40 mg in cycle 1 without prophylactic steroids and escalating QW by up to 150% of the previous dose up to 200 mg and then QW by up to 100% until the DLT criteria for that dose level are met.

A patient will be evaluable for the purpose of the intra-patient dose escalation in cohorts A and B if continuing escalation of the dose until 1) the DLT criteria for that dose level are met or 2) the highest dose level allowed by the modified 3+3 design is reached or 3) 1200 mg dose is reached (whichever applies first). Otherwise, the patient will not be evaluable for the purpose of the intra-patient dose escalation, and hence may be replaced.

Additional patients will be enrolled to follow a step up dosing schedule where RO6958688 will be administered QW for the first 3 administrations then Q3W from subsequent administration onwards (Cycle 1 with RO6958688 at 40 mg [QW], C2 with RO6958688 at 150 mg [QW], Cycle 3 with RO6958688 at 300 mg [QW], then from Cycle 4 onwards with RO6958688 at 600 mg [Q3W]). Once the late cycle MTD has been defined (see Protocol Section 6.7.1.2) or 1200 mg is declared safe in Cohorts A and B (i.e. at least 6 patients reached the 1200 mg dose level and less than 1/3 of Cohorts A and B patients experienced a DLT at 1200 mg or below), any participating patient in the study can be intra-patient dosed escalated up to a dose limited by the defined RO6958688 late cycle MTD or 1200 mg of RO6958688 if the MTD is not reached, after discussion with the medical monitor. Any participating patient in the study who shows loss of RO6958688 exposure (C_{max} reduction of > 50% and a PK profile consistent with

“Use of this document is governed by the terms of use on the first page of this document.”

ADA mediated decrease in exposure) can be dose escalated up to a dose limited by the defined RO6958688 late cycle MTD or 1200 mg of RO6958688 if the MTD is not reached. In this case the patient should receive an intermediate RO6958688 dose of 900 mg. Up to five different cohorts with/without obinutuzumab pretreatment will enroll up to 30 CRC patients (Cohort C – the first 3 patients will be enrolled in a staggered fashion, 1 week delay between each of them), up to 30 gastric cancer patients (Cohort D), up to 30 pancreatic cancer patients (Cohort E), up to 30 breast cancer patients (Cohort F) and up to 30 non-small cell lung cancer patients (Cohort G). Regarding non-small cell lung cancer patients, an alternative RO6958688 dose/schedule can be explored if supported by data (i.e. 40 mg at C1D1 [QW] followed by 100 mg at C2D1 [QW], 150 mg at C3D1 [QW], 300 mg at C4D1 [Q3W] and 600 mg [Q3W], from C4D1 onwards the cycle length is 3 weeks). For these cohorts patient will also be monitored for DLT and the safety rules from the intra-patient dose escalation design described in Section 6.7.1.2 apply separately for each cohort (i.e. by indication). Note: Cohort C has enrolled 24 CRC patients; no further patients will be enrolled. Cohorts D through G did not and will not enroll any patients.

The investigator, after discussion with the Sponsor, will have the option to reduce the dose of RO6958688 to a lower dose level or to allow for a more convenient dose/schedule to prevent the recurrence of adverse events and limit toxicity. This can be done to allow patients, who could potentially benefit from RO6958688, to remain on the study drug.
Body weight-independent has been selected for all parts of the study because no significant effect on body surface area or body weight in overall exposure is expected. RO6958688 will be administered via intravenous infusion. At the discretion of the investigator and when there is no safety concern, all patients can receive further doses at the same dose level and schedule. The treatment period for this protocol is 24 months for RO6958688 and may be modified if supported by emerging data. Because of the potential for progression prior to response with immune therapies, patients who exhibit clinical benefit will continue treatment beyond radiographic progression after discussion and agreement with the Sponsor.

The patients in the obinutuzumab cohorts will receive according to patient's and/or investigators convenience, either 2000 mg of obinutuzumab IV on Day-13 or 1000 mg of obinutuzumab IV on two consecutive days, Day-13 and Day-12 (± 2 days) before C1D1 RO6958688 administration. In Cohort H, the patients will receive according to patient's and/or investigators convenience, either 2000 mg of obinutuzumab IV on Day-7 or 1000 mg of obinutuzumab IV on two consecutive days, Day-8 and Day-7 (+ 1 day) before C1D1 RO6958688 administration.
Note: Cohort H did not and will not enroll any patients.

The study will be conducted in two parts. Part I and Part II of the trial will enroll patients with locally advanced and/or metastatic carcinoembryonic antigen (CEA)–positive solid tumors who have progressed on standard treatment, are intolerant to standard of care (SOC), and/or are non-amenable to SOC. In Part II, MTD has been defined as 400 mg for flat continuous dosing at QW and Q3W.

Part I Single Ascending Dose

The objective of Part I of the study is to investigate the safety and pharmacokinetics of a single dose (QW; one cycle) of RO6958688 in single patient cohorts with dosing starting from a minimal anticipated biological effect level (MABEL) dose of 0.05 mg and up to a maximum dose of 2.5 mg. Up to five single doses of 0.05, 0.15, 0.45, 1.3, and 2.5 mg of RO6958688 are planned to be tested initially in 1 patient each. The doses from 0.15 to 2.5 mg may be revised at the discretion of the Sponsor and investigator should the pharmacokinetic (PK) exposure be lower than predicted.

The cohorts will include single patients in order to minimize the number of patients treated below the therapeutically relevant dose. The results of Part I of the study will determine the starting dose for Part II. All patients will be monitored for a 14-day safety observation period prior to enrollment of the next patient at the next dose level.

A dose is considered safe for the purposes of Part I if no RO6958688–related adverse event of Grade 3 or higher is reported in a patient during the 14-day safety observation period. If the dose is considered safe by the Sponsor and the participating investigators, a new patient will be enrolled to receive a higher dose of RO6958688.

"Use of this document is governed by the terms of use on the first page of this document."

If an RO6958688-related event of Grade 3 or higher is reported in a patient during the 14-day safety observation period, Part I of the study will be ended and the dose used in Part II of the study will be at least at one dose level below the dose at which the adverse event occurred. If no RO6958688-related event of Grade 3 or higher is reported in any patient during the 14-day safety observation period in Part I of the study up to the maximum dose of 2.5 mg, the starting dose used in Part II will be 2.5 mg.

All patients in Part I of the study will receive, as a minimum, one dose of RO6958688. At the discretion of the investigator and in case there is no safety concern, patients can receive further doses QW at the same dose level. Dose escalation to the next available tolerated dose level can only proceed after a patient has tolerated his or her current dose for at least 2 months.

Part II Multiple Ascending Dose Escalation (Dose Finding with Continual Reassessment and Overdose Control)

Part II of the study will establish the appropriate dose(s) based on safety, pharmacokinetics, and the MTD of RO6958688 if defined for the QW regimen, for the Q3W regimen and for the step up dosing regimen, with/without obinutuzumab pretreatment. The starting dose in Part II will be 2.5 mg or lower if Part I is closed at a dose below 2.5 mg. In that case, the starting dose will be the highest and safe dose achieved in Part I at which no RO6958688–related adverse events of Grade 3 or higher were reported during the 14-day observation period.

In order to assess whether obinutuzumab pretreatment can decrease the incidence and/or delay the onset of ADA directed against RO6958688, parallel cohorts of patients receiving obinutuzumab pretreatment will be enrolled. The RO6958688 starting dose for patients with obinutuzumab pretreatment will be the ongoing dose under assessment when the amendment is approved. The patients in the obinutuzumab cohorts will receive according to patient's convenience and/or investigators, either 2000 mg of obinutuzumab IV on Day-13 or 1000 mg of obinutuzumab IV on two consecutive days, Day-13 and Day-12 (± 2 days) before C1D1 RO6958688 administration. In Cohort H, the patients will receive according to patient's and/or investigators convenience, either 2000 mg of obinutuzumab IV on Day-7 or 1000 mg of obinutuzumab IV on two consecutive days, Day-8 and Day-7 (+ 1 day) before C1D1 RO6958688 administration. Premedication will be given prior to each obinutuzumab dosing. For these patients, the baseline tumor biopsy will be taken before receiving the first dose of obinutuzumab and the on-treatment tumor biopsy will be undertaken either at C2D1 or C4D1 if the patient will receive RO6958688 flat dose or at C2D1 or C7D1 if the patient will receive RO6958688 step up dosing. Note: Cohort H did not and will not enroll any patients.

The Sponsor has completed the enrollment of cohorts with obinutuzumab pretreatment, 11 patients in the MAD cohorts and 17 patients who received RO6958688 at 60 mg Q3W (Note: 2 patients were dosed with 40 mg). In the Cohort H, approximately 40 MSS CRC patients will receive obinutuzumab pre-treatment followed by a step up dose regimen with RO6958688 QWx3 / Q3W (40 mg QW at Cycle 1, 100 mg QW at Cycle 2, 150 mg QW at Cycle 3 and from Cycle 4 onwards 150 mg Q3W). Note: Cohort H did not and will not enroll any patients.

Dose-escalation for the QW regimen will be carried out according to a modified-continual reassessment method with escalation with overdose control (mCRM with EWOC) design in order to define MTD and/or the recommended dose, separately for RO6958688 with/without obinutuzumab pretreatment. At least 10 patients will be enrolled in the dose escalation cohorts with obinutuzumab pretreatment. The MTD is defined as the dose that maximizes the probability of a dose-limiting toxicity (DLT) being in the targeted toxicity interval in the range of 20%–35%, subject to the probability of the DLT being in the excessive toxicity interval in the range of 35%–100% being < 25%. Patients within a cohort (3 patients each) will be enrolled in a sequential manner, which, if required, can be expanded with additional patients. Each patient will be observed for 21 days for DLT assessment, and there is no escalation planned during the DLT period. The first patient in each cohort will be observed for safety for 1 week before additional patients are enrolled in the cohort. Once a minimum of 3 patients have completed the 21-day DLT observation period, the Sponsor and investigators will evaluate the next recommended dose using the EWOC design and agree on the dose for the subsequent cohort.

"Use of this document is governed by the terms of use on the first page of this document."

If a DLT is already reported in the first patient during the first week of safety observation, the Sponsor will organize a teleconference with the investigators to discuss the safety and tolerability of RO6958688 and to decide whether the subsequent patient in the same cohort will be enrolled at the same dose. At the end of the DLT period, the same dose might also be escalated in the second cycle instead of the first cycle for the subsequent cohort if in the second and later cycles, the safety profile is better on average and/or the exposure is lower than in Cycle 1 (note: one cycle equals one week in the QW regimen and one cycle equals three weeks in the Q3W regimen). For patients who enroll in Part II and who will be treated at dose levels equal and greater than 5mg (≥ 5 mg), a tumor biopsy for PD analyses is mandatory at baseline and on-treatment either at C2D1, C3D1, C4D1 or C7D1 (- 48/0 hours) as detailed below. Patients being enrolled in cohorts A and B will undertake Baseline and C7D1 pre-dose biopsies. Baseline and on-treatment tumor biopsies will be performed after the baseline and on-treatment FDG-PET. At the discretion of the investigator and in case there is no safety concern, each patient can receive further doses at the same dose level. For each patient separately, dose escalation to the next available tolerated dose level can only proceed after the patient has tolerated the dose he or she was enrolled at, for at least 2 months.

Cohorts may be expanded to collect additional pharmacodynamic PD information to support and/or confirm the mechanism of action and explore doses below the MTD in relation to the PD effects in order to eventually define a recommended dose.

For week 1 (and week 2 in the QW schedule) MTD has been defined as 400 mg.

The cohort A and cohort B will define the late cycle MTD and the maximum intra-patient dose escalation increments by a modified 3+3 dose escalation in two cohorts of patients, the cohorts will be run in a staggered fashion with Cohort A (late cycle MTD) starting before Cohort B (maximum increments):

Cohort A) approximately 6 evaluable patients at 40 mg in cycle 1 without prophylactic steroids and escalating QW by up to 100% of the previous dose until the DLT criteria for that dose level are met

Cohort B) approximately 6 evaluable patients at 40 mg in cycle 1 without prophylactic steroids and escalating QW by up to 150% of the previous dose up to 200 mg and then QW by up to 100% until the DLT criteria for that dose level are met. Cohort B will not be escalated to a dose not already cleared as safe in cohort A.

This will be undertaken in order to determine the recommended schedule and dose to be used for further assessment that optimally balances efficacy, safety, and pharmacodynamics of the three mentioned dose schemes in the tumor and in peripheral blood. For these patients in Part II, baseline and on-treatment tumor biopsies are mandatory.

Additional patients will be enrolled following a step up dosing schedule where RO6958688 will be administered QW for the first 3 administration then followed by Q3W administration (Cycle 1 with RO6958688 at 40 mg [QW], C2 with RO6958688 at 150 mg [QW], Cycle 3 with RO6958688 at 300 mg [QW], then from Cycle 4 onwards with RO6958688 at 600 mg [Q3W]). Once the late cycle MTD has been defined (see Protocol Section 6.7.1.2) or 1200 mg is declared safe in Cohorts A and B (i.e. at least 6 patients reached the 1200 mg dose level and less than 1/3 of Cohorts A and B patients experienced a DLT at 1200 mg or below), any participating patient in the study can be intra-patient dosed escalated up to a dose limited by the defined RO6958688 late cycle MTD or 1200 mg of RO6958688 if the MTD is not reached, after discussion with the medical monitor. Up to five different cohorts with or without obinutuzumab pretreatment will enroll up to 30 CRC patients (Cohort C), up to 30 gastric cancer patients (Cohort D), up to 30 pancreatic cancer patients (Cohort E), up to 30 breast cancer patients (Cohort F) and up to 30 non-small cell lung cancer patients (Cohort G). Cohorts D, E, F and G will open once the dose regimen has been defined. For these cohorts patient will also be monitored for DLT and the safety rules from the intra-patient dose escalation design described in Section 6.7.1.2 apply separately for each cohort (i.e. by indication). Note: Cohort C has enrolled 24 CRC patients; no further patients will be enrolled. Cohorts D through G did not and will not enroll any patients.

"Use of this document is governed by the terms of use on the first page of this document."

In parallel to the enrollment of Cohorts C to H approximately 20 additional patients (all solid tumors following Cohort C dose/schedule) will be enrolled into a biomarker cohort based on their CEA expression level (moderate/low CEA expression, very low and negative CEA expression) in order to initially explore the correlation between CEA expression and anti-tumor activity of RO6958688. Moderate/low CEA expression is defined as those samples having < 20% of tumor cells with IHC2+/3+ and/or ≥ 20% of tumor cells with IHC1+, whereas very low and negative CEA expression is defined as those samples having < 20% of tumor cells with IHC1+ and IHC0+. In this biomarker cohort no high CEA expression patients will be included (defined as ≥ 20% of tumor cells with IHC2+/3+) as such patients are already enrolled in the remainder of Part II. Note: The biomarker cohort did not and will not enroll any patients.

If supported by data (preclinical or clinical data), this protocol may consider to explore this treatment in solid tumours with lower CEA expression intensity level.

Escalation Criteria (Part II)—Dose Limiting Toxicities Applicable for Part II

During dose escalation in Part II, patients who withdraw before the end of the DLT period (i.e., within 21 days of first dose of RO6958688) for reasons other than DLTs and patients who do not receive at least three completed infusions of RO6958688 will be replaced to ensure that at least 3 patients have been assessed for the full DLT period of 21 days prior to moving to the next dose level.

For the purpose of this study, a DLT will be defined as any of the following events attributed to RO6958688 (i.e., related to RO6958688) and occurring during the DLT period:

- Hematological toxicities defined as:
  - Grade 4 neutropenia (i.e., ANC < 0.5×10^9 cells/L for a minimal duration of 7 days)
  - Grade 3 and 4 febrile neutropenia
  - Grade 4 thrombocytopenia lasting > 48 hours
  - Grade 3 thrombocytopenia associated with bleeding episodes
- Grade ≥ 3 non-hematological toxicity with the following exceptions:
  - Alopecia (any grade)
  - Grade 3 nausea or vomiting that resolves to Grade ≤ 2 with or without supportive therapy within 1 week
  - Grade 3 hypophosphatemia and transient Grade 3 hyperbilirubinemia resolved in < 1 week
  - Grade ≥ 3 fatigue that resolves to Grade ≤ 2 within 1 week
  - Infusion-related reactions (IRRs) are not considered to be DLTs because, on the basis of experience with monoclonal antibodies, IRRs are not dose-related events. Precautions, including premedication, will be taken if IRRs of Grade ≥ 2 occur. If described precautions are not sufficient, other options will be discussed between the Sponsor and the investigator.
  - Fever > 40°C (i.e., Grade 3) that occurs within 48 hours of RO6958688 infusion and resolves within 48 hours to >39°C–40°C (Grade ≤ 2) and fully resolves within 1 week
  - Grade 3 arthralgia that can be adequately managed with supportive care or that resolves to Grade ≤ 2 within 1 week
  - Grade 3 diarrhea, colitis, enteritis that resolves to Grade ≤ 2 within 7 days with no fever or dehydration
  - Laboratory values of Grade ≥ 3 that are judged not clinically significant by the investigator
  - Grade 3 tumor pain that starts within 24 hours of infusion and resolves to Grade ≤ 2 within 1 week
  - Grade 3 hypoxia that starts within 24 hours of infusion and resolves to Grade ≤ 2 within 7 days

"Use of this document is governed by the terms of use on the first page of this document."

- In patients with lung lesions, Grade 3 transient dyspnea secondary to localized lung edema that starts within 24 hours of infusion and recovers to Grade ≤ 2 or baseline within 1 week, and transient bronchospasm that resolves within 24 hours
- In patients with liver lesions, Grade 3 transient increase of bilirubin, transaminases and/ or Gamma GT that starts within 24 hours of infusion and recovers to Grade 1 or baseline within 1 week and Grade 4 AST/ALT or Grade 4 bilirubin increase that start after infusion and recovers to Grade ≤2 or baseline within 3 days.

Failure to recover from any RO6958688-related toxicity that results in a dose delay of > 14 days (any regimen) is defined as a DLT.

NUMBER OF STUDY PATIENTS

In Part I, 5 patients have been enrolled.

In Part II, 144 patients have been enrolled. 24 were enrolled in the combined step up QW x 3 / Q3W schedule Cohort C, while Cohorts D through H and the biomarker cohort have not and will not enroll any patients.

TARGET POPULATION

Part I, Part II and the biomarker cohort of the trial will enroll patients with locally advanced and/or metastatic CEA–positive solid tumors who have progressed on standard treatment, are intolerant to SOC, and/or are non-amenable to SOC.

INCLUSION/EXCLUSION CRITERIA

Inclusion Criteria

Patients must meet the following criteria for study entry:

1. Signed informed consent
2. Age ≥ 18 years
3. For dose escalation, locally advanced and/or metastatic gastrointestinal (GI) solid tumor in patients who have progressed on a standard therapy, are intolerant to SOC, and/or are non-amenable to SOC and other solid tumors expressing CEA as per inclusion criterion 13
4. Radiologically measurable disease according to RECIST v1.1
5. Life expectancy (in the opinion of the investigator) of ≥ 12 weeks and LDH ≤ 2,5 x ULN
6. Eastern Cooperative Oncology Group (ECOG) Performance Status (PS) 0–1
7. All acute toxic effects of any prior radiotherapy, chemotherapy, or surgical procedure must have resolved to Grade ≤ 1 or returned to baseline except alopecia (any grade) and Grade 2 peripheral neuropathy
8. Adequate hematological function (without transfusion within 2 weeks prior to Cycle 1, Day 1): neutrophil count of ≥ 1.5×10^9 cells/L, platelet count of ≥ 100,000/μL, and hemoglobin ≥ 8 g/dL (4.9 mmol/L) including lymphocytes within normal limits (≥ 0.8×10^9 cells/L)

"Use of this document is governed by the terms of use on the first page of this document."

9. Adequate liver function: total bilirubin ≤ 1.5 × the upper limit of normal (ULN; excluding Gilbert's Syndrome as defined in Appendix 7), AST and/or ALT ≤ 2.5 × ULN (in case of liver metastases, ≤ 5 × ULN)

10. Adequate renal function: Creatinine clearance ≥ 60 ml/min calculated by CKD-EPI equation (see Appendix 2)

11. Negative serum pregnancy test within 7 days prior to study treatment in premenopausal women and women ≤ 2 years after start of menopause (menopause is defined as amenorrhea for > 2 years)

12. Patients must agree to either remain completely abstinent or to use two effective contraceptive methods from screening until 2 months if the patient is a male or 4 months if the patient is a female after the last dose of RO6958688 if the patient received only RO6958688, or from screening until 3 months if the patient is a male or 18 months if the patient is a female after the last dose of obinutuzumab and 3 months after the last dose of tocilizumab (if applicable) as follows:

a) All males, including male partners of female patients, must agree to either remain completely abstinent or to use a condom during the entire study period with RO6958688 and for 3 months after the last administration of RO6958688 or obinutuzumab and for 2 months after the last dose of tocilizumab (if applicable). Men must refrain from donating sperm during this same period.

b) All females of childbearing potential and female partners of male patients, must agree to remain abstinent or use combined contraceptive methods that result in a failure rate of < 1% per year during the treatment period and at least through 4 months after last dose of RO6958688, or 18 months after the last dose of obinutuzumab if the patient has received obinutuzumab pretreatment and 3 months after the last dose of tocilizumab (if applicable)

    a. Abstinence is only acceptable if it is in line with the preferred and usual lifestyle of the patient. Periodic abstinence (e.g., calendar, ovulation, symptothermal, or postovulation methods) and withdrawal are not acceptable methods of contraception;

    b. Examples of contraceptive methods with an expected failure rate of < 1% per year include male sterilization, hormonal implants, proper use of combined oral or injected hormonal contraceptives, and certain intrauterine devices. Alternatively, two methods (e.g., two barrier methods such as a condom and a cervical cap) may be combined to achieve a failure rate of < 1% per year; barrier methods must always be supplemented with the use of a spermicide

13. Non-gastrointestinal solid tumors (like NSCLC or breast cancer patients) should have confirmed (centrally for North America) CEA expression in tumor tissue ≥ 20% of tumor cells staining with at least moderate to high intensity (IHC2+ and IHC 3+). For CRC cancer patients only, the CEA assessment should be performed but the result is not needed to enroll the patient.

14. For the biomarker cohort, only patients with moderate/low CEA expression (< 20% of tumor cells with IHC2+/3+ and/or ≥ 20% of tumor cells with IHC1+) and very low/negative CEA expression < 20% of tumor cells with IHC1+ or IHC0+) will be enrolled. CEA expression should be determined prior to enrollment (centrally for North America), if no archival tumor tissue is available, a fresh biopsy will be collected.

"Use of this document is governed by the terms of use on the first page of this document."

Exclusion Criteria

Patients who meet any of the following criteria will be excluded from study entry:

1. Active or untreated central nervous system (CNS) metastases as determined by CT or MRI evaluation during screening and prior radiographic assessments

   Patients with a history of treated asymptomatic CNS metastases are eligible, provided they meet all of the following criteria:

   - No metastases to brain stem, midbrain, pons, medulla, cerebellum, or within 10 mm of the optic apparatus (optic nerves and chiasm)
   - Radiographic demonstration of improvement upon the completion of CNS-directed therapy and no evidence of interim progression between the completion of CNS-directed therapy and the screening radiographic study
   - No history of intracranial hemorrhage or spinal cord hemorrhage
   - No ongoing requirement for dexamethasone as therapy for CNS disease; anticonvulsants at a stable dose allowed
   - No stereotactic radiation or whole-brain radiation within 28 days prior to Cycle 1 Day 1
   - Screening CNS radiographic study ≥ 4 weeks since completion of radiotherapy and ≥ 2 weeks since discontinuation of corticosteroids.
2. Spinal cord compression not definitively treated with surgery and/or radiation or previously diagnosed and treated spinal cord compression without evidence that disease has been clinically stable for ≥ 2 weeks prior to enrollment.
3. Leptomeningeal disease.
4. Patients with paraspinal, paratracheal and mediastinal pathological lesions larger than 2 cm unless they are previously irradiated. Irradiation of lesions must be completed at least 14 days prior to initiation of study treatment.
5. Patients with another invasive malignancy in the last 2 years (with the exception of basal cell carcinoma and tumors deemed by the investigator to be of low likelihood for recurrence)
6. Evidence of significant, uncontrolled concomitant diseases that could affect compliance with the protocol or interpretation of results or contraindicate the use of an investigational drug, including diabetes mellitus, history of relevant pulmonary disorders, and known autoimmune diseases
7. Patients with bilateral lung lesions and dyspnea and/or with bilateral lung lesions and SaO2 < 92% (at rest and with exercise, room air) or patients with lobectomy or pneumonectomy with lung metastases in the remaining lung and either dyspnea or SaO2 less than 92% (at rest and with exercise, room air) at baseline
8. Uncontrolled hypertension (systolic blood pressure [BP] > 150 mmHg and/or diastolic BP > 100 mmHg), unstable angina, congestive heart failure of any New York Heart Association classification, serious cardiac arrhythmia that requires treatment with the

"Use of this document is governed by the terms of use on the first page of this document."

exceptions of atrial fibrillation and paroxysmal supraventricular tachycardia, and history of myocardial infarction within 6 months of enrollment

9. Active or uncontrolled infections
10. Known HIV or known active hepatitis B or hepatitis C infection for patients not receiving obinutuzumab pretreatment
11. Major surgery or significant traumatic injury < 28 days prior to the first RO6958688 infusion (excluding biopsies) or anticipation of the need for major surgery during study treatment
12. Dementia or altered mental status that would prohibit informed consent
13. Pregnant or breastfeeding women
14. Known hypersensitivity to any of the components of RO6958688 and/or obinutuzumab
15. Concurrent therapy with any other investigational drug (defined as treatment for which there is currently no regulatory authority–approved indication) < 28 days prior to the first RO6958688 infusion
16. Last dose of any chemotherapy < 28 days prior to the first RO6958688 infusion
17. Last dose with an antibody or immunotherapy (i.e., interferon-α, interferon-β, IL-2, etanercept, infliximab, tacrolimus, cyclosporine, mycophenolic acid, alefacept, or efalizumab) < 28 days prior to the first RO6958688 infusion
18. Last dose of anti-CTLA4, anti–PD-L1, or anti-PD1 < 28 days prior to the first RO6958688 infusion
19. Expected need for regular immunosuppressive therapy (i.e., for organ transplantation, chronic rheumatologic disease) within 28 days prior to the first RO6958688 infusion
20. Regular dose of corticosteroids the 28 days prior to Day 1 of this study or anticipated need for corticosteroids that exceeds prednisone 10 mg/day or equivalent within 28 days prior to the first RO6958688 infusion, with the exception of the obinutuzumab steroid premedication. Inhaled and topical steroids are permitted.
21. Baseline corrected QT interval of > 470 ms. Patients with baseline resting bradycardia < 45 beats per minute or baseline resting tachycardia > 100 beats per minute
22. Radiotherapy within the last 28 days prior to the first RO6958688 infusion with the exception of limited–field palliative radiotherapy
23. Administration of a live, attenuated vaccine within 28 days before Cycle 1, Day 1 or anticipation that such a live attenuated vaccine will be required during the study.
24. History of progressive multifocal leukoencephalopathy (PML).

Additional exclusion criteria for patients to be pretreated with obinutuzumab:

- Positive test results for human T-lymphotropic virus 1 (HTLV-1) or active HIV infection. HTLV-1 testing is required in participants from endemic countries (Japan, countries in the Caribbean basin, South America, Central America, sub-Saharan Africa, and Melanesia)
- Positive test results for chronic hepatitis B infection. All patients must be tested for both HBsAg and HBcAb at screening, if either of the tests is positive, the patient is not

“Use of this document is governed by the terms of use on the first page of this document.”

eligible for inclusion in the trial. Patients who have protective titers of HBsAb after vaccination are eligible provided they are negative for both HBsAg and HBcAb, patients with negative result by PCR regarding Hepatitis B and C are eligible

- Positive test results for hepatitis C (hepatitis C virus [HCV] antibody serology testing)
- Known active TB requiring treatment within 3 years prior to baseline or latent TB that has not been appropriately treated
- Active bacterial, viral, fungal, or other infection, or any major episode of infection requiring treatment with IV antibiotics within 4 weeks of Day 1 of Cycle 1
- International normalized ratio (INR) or prothrombin time (PT) > 1.5 × ULN in the absence of therapeutic anticoagulation
- PTT or aPTT > 1.5 x ULN in the absence of a lupus anticoagulant
- Known hypersensitivity to any of the components of obinutuzumab; hypersensitivity to Chinese hamster ovary cell products or other recombinant human antibodies.
- History of progressive multifocal leukoencephalopathy (PML)

LENGTH OF STUDY

Patients will be treated for a maximum of 24 months but this may be modified if supported by emerging data. Patients in Part I who receive more than one dose of RO6958688, and patients who participate in Part II will be treated until disease progression, loss of clinical benefit, unacceptable toxicity, loss of RO6958688 exposure, or withdrawal from treatment for other reasons or death. All patients will attend a 28-day (±3 days) safety follow-up visit after receiving the last infusion of RO6958688 or obinutuzumab if for any reason the patient does not receive any RO6958688 infusion.

The total study duration will be approximately 60 months.

END OF STUDY

The study will formally end once the survival update has taken place or the last patient has completed the safety follow-up visit or withdrawn from the study prior to that time (whichever occurs last), but may be prematurely terminated by the Sponsor.

OUTCOME MEASURES

SAFETY OUTCOME MEASURES

The safety outcome measures for this study are:

- Incidence of DLTs
- Incidence and severity of adverse events and IRRs and CRS symptoms
- Incidence of laboratory abnormalities (as examples and not limited to: hematology testing, coagulation, serum chemistries, and urinalysis)
- Incidence of anti-drug antibodies (ADAs) formation and detection of cytokine release
- Physical examination findings
- Triplicate 12-lead ECGs
- Vital signs

All patients who participate in the entry in human study will be clinically evaluated at screening and on a regular basis during the entire course of the study. The routine safety monitoring plan will include clinical examination, vital signs assessment (including heart rate, diastolic and systolic BP, ECG), laboratory analysis (hematology, biochemistry, urinalysis), and regular collection and review of the reported adverse events.

“Use of this document is governed by the terms of use on the first page of this document.”

The National Cancer Institute Common Terminology Criteria for Adverse Events v4.03 will be used to evaluate the clinical safety of the treatment in this study (http://evs.nci.nih.gov/ftp1/CTCAE/CTCAE_4.03_2010-06-14_QuickReference_8.5x11.pdf). CRS severity will be graded according to the NCI CTCAE v5 (Table 9). Patients will be assessed for adverse events at each clinical visit and as necessary throughout the study.

Safety will be determined, but not limited to, by the spontaneous reporting of adverse events; by the assessments of routine laboratory values (hematology testing, serum chemistries); findings on physical examinations; ECGs; chest X-ray; vital signs; by carefully observing patients for IRRs; by the determination of ADAs in cohorts.

Please refer to schedule of assessments for details on collection time of the assessments outlined below.

Laboratory Tests

Hematology and biochemistry will be done at least prior to RO6958688 administration as part of the regular safety assessments.

- Hematology: erythrocytes, hemoglobin, hematocrit, platelets, leukocytes, and differential count (neutrophils, eosinophils, basophils, monocytes, lymphocytes)
- Blood biochemistry: sodium, potassium, chloride, calcium, phosphate, magnesium, urea, creatinine, creatinine clearance (by calculated by CKD-EPI equation), total protein, albumin, glucose, total and direct bilirubin, alkaline phosphatase, ALT, AST, LDH, γ-glutamyl transferase, ferritin and high sensitivity C-reactive protein. Soluble CEA (sCEA) measured in serum or plasma, will also be measured as a disease monitoring marker (as part of the serum chemistry panel – for part II patients only).
- Lipids: total cholesterol, LDL cholesterol, HDL cholesterol, and triglycerides
- Coagulation: PT, INR, PTT, and fibrinogen
- Blood serology for HBV, HIV/HTLV-1 (from endemic countries (Japan, countries in the Caribbean basin, South America, Central America, sub-Saharan Africa, and Melanesia) (HTLV-1 for patients receiving obinutuzumab pretreatment only) and HCV testing (HB surface antigen [HBsAg], total core HB antibody [anti-HB-cAb], Hepatitis C [HC virus [HCV]) for patients to be pretreated with obinutuzumab, if there is a doubt regarding the result, a PCR can be performed
- Pregnancy test: All women of childbearing potential, including those who have had a tubal ligation, will have a serum pregnancy test at screening within 7 days of first dose and on a regular basis during the treatment period (serum or urine).
- Urinalysis: Dipstick for pH, glucose, blood, protein, ketones, and bilirubin. If there is a clinically significant positive result (i.e., confirmed by a positive repeated sample), urine will be sent to the laboratory for microscopy and culture. If there is an explanation for the positive dipstick result (e.g., menses), it should be recorded and there is no need to perform laboratory for microscopy and culture.

Unscheduled hematology, biochemistry, and coagulation will be performed for patients who develop IRRs and as clinically indicated.

Additional Safety Laboratory Assessments

- ADA
- Cytokine release assessment during IRR/CRS
- IgE and tryptase (for patients who experience a Grade≥2 IRR/CRS)

PHARMACOKINETIC OUTCOME MEASURES

Serum samples will be collected for PK analyses. The PK outcome measures for this study are:

- Pharmacokinetics of RO6958688 and obinutuzumab following single and multiple doses
- The PK parameters derived from the serum concentration–time profile following administration of RO6958688 and obinutuzumab include the parameters listed below (if appropriate):

"Use of this document is governed by the terms of use on the first page of this document."

- C_{max}
- Area under the curve (AUC)
- Half-life ($t_{1/2}$)
- Minimum drug concentration (trough concentration, C_{min})
- Clearance (CL)
- Volume of Distribution at Steady State (V_{ss})

In addition, data will be analyzed using population PK modeling; results will be reported separately.

PK concentration data of RO6958688 will be summarized using descriptive statistical methods.

Obinutuzumab serum concentration data (C_{min} and C_{max}) will be tabulated and summarized using descriptive statistics (e.g. mean, median, range, and standard deviation, as appropriate).

PHARMACODYNAMIC OUTCOME MEASURES

The PD outcome measures for this study are:

- Whole blood samples: Peripheral blood immune cells will be assessed with respect to the changes in the characteristics of lineage (CD4+ T cells, CD8+ T cells, natural killer [NK] cells, monocytes, T-regulatory cells, and B cells), activation (including but not limited to CD25, CD69, etc.), and differentiation (including but not limited to Ki67, PD1, TIM3, ICOS, etc.).
- Soluble CEA: will be measure as a disease-monitoring marker. A mandatory whole blood sample will be taken for soluble CEA central assessment.
- TCR Vβ: at Cycle 1 Day 1 predose (baseline) in the QW schedule, at Cycle 1 Day 1 predose (Baseline) and Cycle 3 Day 1 predose in the Q3W schedule, and at Cycle 1 Day 1 predose (baseline) in the QWx3/Q3W schedule, mandatory 2 whole blood samples will be taken for TCR Vβ sequencing
- Serum or plasma samples: PD biomarkers such as cytokines and inflammation markers (including but not limited to tumor necrosis factor-α (TNFα), interferon-γ (INFγ), interleukin (IL)-6, MIP, etc.) will be analyzed. Because these measurements are also safety measure assessments during any IRRs, they will also be examined in patients enrolled in both Part I and Part II of the study. Disease-monitoring markers that include but are not limited to sCEA will also be assessed.
- Tumor biopsy: Tumor biopsy samples obtained in Part II will be assessed centrally for changes in immune cell numbers and activation characteristics as well as changes in tumor markers such as PD-L1. These analyses will be performed by flow cytometric molecular and/or immunohistochemistry methods with respect to changes in the characteristics of lineage (CD4+ T cells, CD8+ T cells, NK cells, monocytes, T-regulatory cells, and B cells), activation (including but not limited to CD25, CD69, etc.), differentiation (including but not limited to Ki67, PD1, TIM3, ICOS, etc.), and TCR Vb repertoire.

Positron Emission Tomography (PET): Baseline and on-treatment 2-[^{18}F]Fluoro-2-deoxyglucose positron emission tomography (FDG-PET) will be collected to determine changes in glucose metabolism of the tumor lesions. Assessment of FDG-PET will be performed locally at the sites during the whole study.

Original or archival tumor: Potential prognostic biomarkers such as CEA expression will be confirmed on archival tumor, if available, or from the freshly obtained biopsy samples. In Part II, these measurements will assess the CEA change over the course of the disease and the stability of the measurements. Further other exploratory biomarker assessments, including but not limited to analysis of microsatellite instability, mutational signatures and genomic mutations, may be carried out.

Blood and Serum or Plasma Samples

Blood samples will be collected for the analyses of immune cell number and activation only from patients enrolled in Part II. Blood samples will be collected and analyzed with respect to

"Use of this document is governed by the terms of use on the first page of this document."

alterations in the number and activation and differentiation of immune cells as a consequence to treatment with RO6958688. The samples will be analyzed by flow cytometry, and the number of cells that belong to lymphocyte subsets (CD4+ T cells, CD8+ T cells, NK cells, B cells, and monocytes) and their activation and differentiation status will be determined (including but not limited to CD25, Ki67, PD1, and TIM3). Additional immune PD biomarkers that are related to the mode of action (MoA) of RO6958688 may also be analyzed from serum or plasma samples taken from all patients. Such PD measures will be considered as disease-monitoring markers and include but are not limited to sCEA. If available, an assessment of tumor growth kinetics will be made by comparing post-treatment scans with the last available pre-study scan.

Tumor Biopsy Samples

Tumor biopsy samples (each consisting of two tissue specimens at least the size of an 18G core needle biopsy, fine-needle aspiration is not acceptable) will be collected in Part II of the study on two occasions (once at baseline [before obinutuzumab pretreatment but after the baseline FDG PET] to confirm eligibility and once during the study treatment period), and a minimum of two core samples will be collected at each occasion. Mandatory biopsies will be collected from all patients treated at dose levels equal and greater than 5 mg ($\geq$ 5 mg). The biopsies will be taken from accessible, "non-critical" tumor locations (metastatic), including, but not limited to, skin, lymph node, rectum, liver, etc (bone marrow biopsy is not accepted). All on-treatment tumor biopsies will be collected either at C2D1, C3D1, C4D1 or C7D1 (-48/0 hours). Baseline tumor biopsy will be performed after the baseline FDG-PET but before obinutuzumab pretreatment or RO6958688 whichever occurs first.

The baseline and on-treatment biopsies should preferentially be taken from the same tumor lesion to ensure comparability when accessible in a non-critical location. Data from FDG-PET should guide which lesion would be more reflective of capturing the PD effect and thus the baseline and on-treatment biopsies should be taken from that tumor lesion. All biopsies must be taken after week 6 FDG-PET. For patients discontinuing from the study due to disease progression, additional optional biopsies, if clinically feasible, may be taken to aid the understanding of immune resistance mechanisms.

Tumor biopsies will be centrally analyzed for immune cell number and activation by flow cytometric and/or immunohistochemical methods for the density of different immune cell lineages (including but not limited to CD4+, CD8+, B lymphocytes, NK cells, macrophages) and their activation and differentiation status (including but not limited to CD25, Ki67, PD1, TIM3) and for the expression of tumor markers such as PD-L1. In addition, analysis of gene expression, genetic alteration and the infiltrating T cell repertoire may be carried out. The goals of these analyses will be (i) to establish a dose–response relationship, (ii) to understand the MoA of RO6958688 at the tumor site and (iii) to improve the understanding on intratumoral B cell depletion upon obinutuzumab pretreatment.

Positron Emission Tomography (PET)

FDG-PET can identify sign of biological effect early, before tumor size is reduced. Moreover, a reduction in the FDG-PET signal within days or weeks of initiating therapy (e.g., in lymphoma, non–small cell lung, and esophageal cancer) has been shown to correlate with prolonged survival and other clinical end points now used. These findings suggest that FDG-PET could facilitate drug development as an early marker of drug effect.

Whole-body imaging PET should begin 60 ± 10 min after FDG injection. Both FDG-PET must be performed before the biopsies.

FDG-PET should be performed according to the schedule of assessment in all eligible patients in Part II of the study.

EFFICACY OUTCOME MEASURES

The exploratory efficacy/activity outcome measures for this study are:

- ORR, DOR, BOR
- The rate of patients with stable disease
- DCR, defined as PR + CR + SD

"Use of this document is governed by the terms of use on the first page of this document."

- PFS *according to RECIST v1.1*

Tumor response will be evaluated according to RECIST v1.1 and irRC using unidimensional measurement such as computed tomography (CT) scan or magnetic resonance imaging. Assessment of CT/MRI scans as tumor assessments will be performed at the sites during the whole study.

Tumor assessment will be performed once during the screening. The first assessment after the start of treatment will be performed at 8 weeks and continue every 8 weeks thereafter for the first year and every 12 weeks thereafter until disease progression or treatment discontinuation. All tumor assessments after baseline may be done within ± 7 days of the scheduled visit. If Sponsor decides, independent central read for computed tomography (CT) or magnetic resonance imaging (MRI) might be performed in this study, both prospectively and retrospectively.

Additionally, FDG-PET based tumor assessment will be performed at baseline and at week 6 (C6D5 -72/0 hours) for the QW schedule, at week 6 (C2D19 -72/0 hours) for the Q3W schedule and at week 6 (C4D19 -72/0 hours) for the QWx3/Q3W schedule. Baseline FDG-PET must be performed before the baseline tumor biopsy and on-treatment week 6 FDG-PET must be performed before the on-treatment biopsy if feasible.
Confirmation of partial and complete responses will be done at the next scheduled visit after at least 28 days from the initial response. A patient is assigned a best overall confirmed response SD if they have a response assessment of SD, PR, or CR at one or more visits at least 42 days (6 weeks) after start of RO6958688, but are not a confirmed CR or PR.

PFS per RECIST v1.1 or irRC criteria is defined as time between *enrollment date* and date of first documented disease progression per RECIST v1.1 or irRC criteria, respectively, or death from any cause, whichever occurs first and only if it occurs no later than 28 days after the last confirmed intake of RO6958688. Patients who neither progressed nor died in this interval, or who are lost to follow-up are censored at the date of last tumor assessment within this time window or last follow-up for progression of disease. Patients for whom no post-baseline tumor assessments are available are censored at Cycle 1 Day 1.

The following criteria are needed for continuing treatment beyond RECIST v1.1–defined progression that has been initially assessed by the investigator:

- Investigator–assessed clinical benefit and
- The patient is tolerating study drug.

Optional submission of the latest pre-study or historical CT scans is highly encouraged for assessment of tumor growth kinetics within 6 weeks of patient entering the study if available. This scan will be compared to those collected during the study to determine tumor growth kinetics.

EXPLORATORY OUTCOME MEASURES

The exploratory objectives for this study include but are not limited to the following:

- Rate of patients with positive ADA titer against RO6958688 at week 8
- Overall survival
- The density, localization and activation status of immune cells and their subsets will be assessed in freshly obtained biopsies taken before treatment with RO6958688 and/or obinutuzumab and during treatment with RO6958688 to identify any potential predictive or prognostic biomarkers of efficacy
- A possible association of expression of activation related immune genes (e.g., IFNγ, CXCLC9, etc.) with PD response will be investigated. In addition, gene expression and mutation analysis may be carried out.
- The baseline values and kinetics of soluble markers of tumor markers (such as CEA) will be explored.
- The specificity of ADA in patients with no obinutuzumab pretreatment.

“Use of this document is governed by the terms of use on the first page of this document.”

“Use of this document is governed by the terms of use on the first page of this document.”

The specimens will be used for research purposes to identify biomarkers useful to predict and monitor response to RO6958688 treatment with or without obinutuzumab pretreatment, identify biomarkers useful for to predict and monitor RO6958688 safety, assess PD effects of RO6958688 treatment, and investigate mechanism of therapy resistance or immune escape. Additional markers may be measured in case a strong scientific rationale for these analyses develops.

Tumor

Tumor biopsy samples (each consisting of a minimum of two tissue specimens at least the size of a 18G core needle biopsy, fine-needle aspiration is not acceptable) will be collected from all patients who participate in Part II of the study on two occasions (once at baseline [before obinutuzumab pretreatment but after the baseline FDG PET] to confirm eligibility and once during the study treatment period), and a minimum of two core samples will be collected at each occasion. Baseline tumor biopsy will be performed after the baseline FDG-PET. The biopsies will be taken from accessible, “non-critical” metastatic tumor locations, including, but not limited to, skin, lymph node, rectum, liver, etc (bone marrow biopsy is not accepted). In particular, archival tumor tissue is to be obtained from all patients, if available, in order to perform CEA assessment for patient eligibility (refer to the Laboratory Manual).

Whole Blood

In Part II of the study, whole blood samples will be collected for the flow cytometry for determination of immune cell markers (e.g., immune cell subsets, activation and proliferation markers).

Serum or plasma

Blood for serum or plasma isolation will be collected for investigation of PD markers such as sCD25, cytokines (such as IL-6, IFNγ, TNFα), and tumor markers. In the event of an IRR/CRS, an additional sample will be collected.

These samples will be destroyed within 2 years after the date of final closure of the clinical database. Archival tumor blocks will be returned. Other residual tissue material (slides, extracts, etc.) will be destroyed within 2 years after the date of final closure of the clinical database unless the patient gives specific consent for the remainder of their sample(s) to be stored for optional exploratory research. If the patient provided consent for optional exploratory research their samples will be destroyed no later than 15 years after the date of final closure of the clinical database.

Whole blood samples

From Part II patients, at Cycle 1 Day 1 predose (baseline) in the QW schedule and at Cycle 1 Day 1 predose (Baseline) and Cycle 3 Day 1 predose in the Q3W schedule, and at Cycle 1 Day 1 predose (baseline) in the QWx3/Q3W schedule, mandatory 2 whole blood samples will be taken for TCR Vβ sequencing (the CDR3-TCR beta chain repertoire). The DNA will be used to determine in peripheral T cells the repertoires of T cell receptor (TCR) Vβ CDR3 and analyze TCR diversity.

Soluble CEA

Blood sample will be taken for soluble CEA central assessment, refer to respective Schedule of assessments Appendix 1.

Clinical Genotyping Samples

From every subject in Part II, a baseline mandatory whole blood sample will be taken for DNA extraction. The DNA will be used to determine if alleles at genes associated with immune responses such as Chemotaxis, HLA, immunosuppression etc, affect the PK/PD/efficacy/safety of RO6958688. Data arising from this study will be subject to the same confidentiality as the rest of the study. This specimen will be destroyed immediately after analysis and the results have been checked.

ROCHE CLINICAL REPOSITORY

The Roche Clinical Repository (RCR) is a centrally administered facility for the long-term storage of human biological specimens including body fluids, solid tissues and derivatives thereof (e.g., DNA, RNA proteins/peptides). Specimens for dynamic (non-inherited) biomarker discovery and validation will be collected from all patients who participate in the trial.

These specimens will be used for research purposes to identify biomarkers that are predictive of response to treatment with RO6958688, and will help to better understand the pathogenesis,

“Use of this document is governed by the terms of use on the first page of this document.”

course, and outcome of the studied cancer types. The collected samples might allow the generation of statistically meaningful biomarker data.

The results of specimen analysis from the RCR will facilitate the rational design of new pharmaceutical agents and the development of diagnostic tests, which may allow for individualized drug therapy for patients in the future.

The specimens in the RCR will also be made available for future biomarker research towards further understanding of RO6958688, treatment of related diseases and adverse events and for the development of potential associated diagnostic assays.

Whole blood samples for DNA and RNA will be collected if patients consent to participate in the RCR. No additional samples will be collected for the RCR; however, patients will also have the option to consent that any tissue material remaining after protocol defined analyses can be stored for up to 15 years in the RCR.

INVESTIGATIONAL MEDICINAL PRODUCTS

Test Products

For the purpose of the study, RO6958688, obinutuzumab and tocilizumab are considered investigational medicinal products (IMPs).

RO6958688

RO6958688 is a novel T-cell bispecific antibody targeting the human CEA on tumor cells and CD3 on T-cells. RO6958688 is administered intravenously, in patients with locally advanced and/or metastatic CEA (+) solid tumors by QW and Q3W schedules.

The starting dose in Part I is based on a MABEL determination and will be 0.05 mg. The starting dose in Part II will be 2.5 mg or lower if Part I is closed earlier due to RO6958688–related adverse events of Grade 3 or above.

Obinutuzumab

Obinutuzumab is a humanized type II anti-CD20 monoclonal antibody that recognizes the CD20 antigen present on normal and malignant B-cells. The glycoengineering of the molecule lead to the following characteristics: high-affinity binding to the CD20 antigen, high antibody-dependent cellular cytotoxicity (ADCC), and antibody-dependent cellular phagocytosis (ADCP). Compared to rituximab obinutuzumab has low complement-dependent cytotoxicity (CDC) activity; and high direct cell death induction leading to a more potent B cell depletion. It is used and is being developed for the treatment of hematological malignancies, namely non-Hodgkin lymphoma (NHL) and chronic lymphocytic leukemia (CLL). The dose of obinutuzumab administered in this study is 2000 mg, either 2000 mg of obinutuzumab IV on Day-13 or 1000 mg of obinutuzumab IV on two consecutive days, Day-13 and Day-12 (± 2 days) prior to treatment start with RO6958688 at C1D1. For Cohort H, the dose of obinutuzumab administered in this study is 2000 mg, either 2000 mg of obinutuzumab IV on Day-7 or 1000 mg of obinutuzumab IV on two consecutive days, Day-8 and Day-7 (+1 day) prior to treatment start with RO6958688 at C1D1.

Tocilizumab

Tocilizumab (Actemra®/RoActemra®) is a recombinant, humanized, anti-human monoclonal antibody directed against soluble and membrane-bound IL-6R, which inhibits IL-6 mediated signaling. Blocking the inflammatory action of IL-6 using tocilizumab could therefore represent a novel approach for the treatment of CRS.

Tocilizumab will be administered if required, for the management of severe CRS (if a study participant experiences severe CRS during or after any infusion of RO6958688). Tocilizumab has been recently approved by the FDA (August 2017) and received a positive CHMP opinion (European Medicines Agency 2018) for a restricted use, limited to Cytokine Release Syndrome (CRS) induced by chimeric antigen receptor (CAR) T cell in adults and pediatric patients 2 years of age and older (Tocilizumab USPI). Since tocilizumab will be used in the event of a severe CRS during or after any infusion of RO6958688, it is therefore classified as an investigational medicinal product (IMP).

NON-INVESTIGATIONAL MEDICINAL PRODUCTS

None

“Use of this document is governed by the terms of use on the first page of this document.”

PROCEDURES

SCREENING: Written informed consent for participation in the study must be obtained before performing any study-specific screening tests or evaluations. Screening and pretreatment assessments will be performed within 28 days prior to Cycle 1 Day 1 predose unless otherwise specified.

TREATMENT: Assessments performed during treatment are specified in the Schedule of Assessment and Hourly Assessment tables.

FOLLOW-UP: Patients who complete the study or discontinue from the study early will be asked to return to the clinic 28 days after the last dose of study drug for a post-study follow-up visit. The visit at which response assessment shows progressive disease may be used as the study completion/early termination visit.

LIST OF SPECIAL SAFETY OR PD TESTS:

Although RO6958688 is a humanized antibody, there is a risk that ADA against RO6958688 may develop, potentially reducing its efficacy and/or potentially resulting in symptomatic hypersensitivity reactions, in particular immune-complex reactions. In this study, samples will be taken to assess the possible presence of ADA. The date and time of each sample will be recorded in the electronic Case Report Form.

For the assessment of cytokine release, serum or plasma samples (blood for pharmacodynamics and additional safety) will be collected at the time of an IRR/CRS (including repetitive occurrence of IRR/CRS). Cytokine analysis will include but is not limited to TNFα, IL-6, and IFNγ.

For patients who experience a Grade $\geq$2 IRR/CRS, tryptase and total IgE will be analyzed.

WITHDRAWAL CRITERIA: Reasons for discontinuation of study drug or withdrawal from the study may include but are not limited to the following:

- Patient withdrawal of consent at any time
- Any medical condition that the investigator or Sponsor determines may jeopardize the patient's safety if he or she continues in the study
- Investigator or Sponsor determines it is in the best interest of the patient.

All patients will attend a 28-day safety follow-up visit after receiving the last infusion of RO6958688 or obinutuzumab if for any reason the patient does not receive any RO6958688 infusion.

BLINDING OR UNBLINDING:

Not applicable

STATISTICAL METHODS

DOSE-ESCALATION APPROACH

The primary endpoint of the study will be safety and, in particular, the occurrence of a DLT. The primary variables of the study are PK, including ADA titers against RO6958688, and adverse event profile, including DLTs and the MTD with/without obinutuzumab pretreatment for the QW regimen and the late cycle MTD for the step up dosing regimen, if achieved.

Multiple Ascending Dose (MAD) cohort

The Multiple Ascending Dose cohort of Part II of the study will employ a mCRM with EWOC design for the QW regimen, in order to define MTD and/or the recommended dose with/without obinutuzumab pretreatment. The design is based on the primary safety variable (the occurrence of a DLT). The MTD is defined separately for treatment with and without obinutuzumab pretreatment as the dose which maximizes the probability of a DLT being in the targeted toxicity interval of 20% to 35%, subject to the probability of DLT being in the excessive toxicity interval of 35% to 100% being < 25%. Patients within a cohort will be enrolled in a sequential manner in cohorts of 3 patients each, which, if required, can be expanded with additional patients.

Dose escalation according to mCRM with EWOC designs will be carried out independently for the cohorts with and without obinutuzumab pretreatment, in order to be able to accommodate potentially different MTDs and/or recommended doses. The starting dose for the dose escalation with obinutuzumab pretreatment will be the highest dose which has been cleared

"Use of this document is governed by the terms of use on the first page of this document."

without obinutuzumab pretreatment (i.e. the cohort without obinutuzumab pretreatment has completed the DLT period, and the dose is admissible in the EWOC model of the dose escalation without obinutuzumab).

The rates of patients with positive ADA titer against RO6958688 at week 8 will be compared between patients with/without obinutuzumab pretreatment, by performing a Bayesian statistical analysis. Furthermore, the onset of ADA formation will be analyzed by survival methods on time-to-first-ADA detection. Separate analyses by doses are possible.

Each patient will be observed for 21 days for DLT assessment. The first patient in each cohort will be observed for safety for 1 week before additional patients are enrolled in the cohort. If a DLT is already reported in the first patient during the first week of safety observation, the Sponsor will organize a teleconference with the investigators to discuss the safety and tolerability of RO6958688 and to decide whether the subsequent patient in the same cohort will be enrolled at the same dose. Based on emerging safety, PK, and PD data, the 1–week safety observation period between initial administration of RO6958688 to the first patient and in subsequent patients within each dose level may be adjusted as deemed appropriate by the investigators and the Sponsor.

At the end of the DLT period, the same dose might also be escalated in the second cycle instead of the first cycle for the subsequent cohort if in the second and later cycles, the safety profile is better on average and/or the exposure is lower than in Cycle 1. In that case, for purposes of the mCRM model fit, DLTs that occur between the first and second cycle will count as DLTs for the first cycle dose, and DLTs that occur at any later timepoint will be attributed to the second cycle dose.

Once a minimum of 3 patients have completed the 21–day DLT observation period, the Sponsor and investigators will evaluate the next dose recommended by the EWOC design and agree on the dose for the subsequent cohort. At each dose-escalation step, the dose can be escalated or de-escalated or an additional cohort at that same dose level could be enrolled. At the discretion of the investigator and in case there is no safety concern, all the patients can receive further doses either at the same dose level or can be escalated to higher dose levels that have been cleared for dose escalation.

The starting dose for RO6958688 in Part II will be 2.5 mg, or a lower dose if Part I is closed earlier due to RO6958688–related adverse events of Grade 3 or above. The maximum allowable increment for RO6958688 between dose levels will be 100%.

Note that this maximum allowable increment applies to doses equal to or above 2.5 mg (a dose grid up to 80 mg with 1 mg spacing, and from 85 to 1000 mg with 5 mg spacing, will be used). Should lower doses be evaluated in Part II, then the dose values will be taken from 0.03 mg, 0.05 mg, 0.10 mg, 0.15 mg, …, 2.45 mg, 2.5 mg.

The selection of the next dose will be subject to clinical judgment and mandated safety constraints that limit the size of dose increments. In addition, the clinical judgment of the Sponsor and investigators in the dose-selection process will also be utilized. This may lead to dose selections which differ from the mCRM recommendations, if the scientific and clinical opinion is that this would be more appropriate for patients.

The model–based dose escalation at each step will identify the next recommended dose level subject to the following criteria:

- The posterior probability of being within the target toxicity interval (of 20% to 35% DLT probability) is maximized and
- The posterior probability of being within the excessive toxicity interval (above 35% DLT probability) is below 25%.

The dose-escalation without obinutuzumab pretreatment will stop under the following circumstances:

- The maximum sample size of 60 patients (only counting the minimum required number of patients per cohort without obinutuzumab) has been reached or
- At least a minimum of 15 patients has been accrued overall (in Parts I and II), at least 6 patients have been accrued near the MTD dose (where near means differing from the MTD by at most 20%), and in addition the probability that the MTD dose lies within the target toxicity interval is above 40%.

“Use of this document is governed by the terms of use on the first page of this document.”

- The dose escalation with obinutuzumab pretreatment will stop under the following circumstances:
  - The maximum sample size of 30 patients (only counting the minimum required number of patients per cohort with obinutuzumab) has been reached or
  - At least a minimum of 10 patients has been accrued overall, at least 6 patients have been accrued near the MTD dose (where near means differing from the MTD by at most 20%), and in addition the probability that the MTD dose lies within the target toxicity interval is above 40%.

The dose-determining population consists of all patients from the safety population who received at least three administrations of RO6958688 within the first 21 days or more if any of the second or third dose is delayed for any reason and have undergone the scheduled safety evaluations or discontinued earlier due to DLT.

Dose-escalation in the step-up dosing regimen

Cohorts A and B of Part II of the study will employ a modified 3+3 dose escalation design for the step up dosing regimen, in order to define the late-cycle MTD and the maximum intra-patient dose escalation increments. Two cohorts of patients will be treated, the cohorts will be run in a staggered fashion with Cohort A (late cycle MTD) starting before Cohort B (maximum increments) and cohort B will not be escalated to a dose not already cleared as safe in cohort A:

Cohort A) Approximately 6 evaluable patients at 40 mg in cycle 1 without prophylactic steroids and escalating QW by up to 100% of the previous dose until the DLT criteria for that dose level are met.

Cohort B) Approximately 6 evaluable patients at 40 mg in cycle 1 without prophylactic steroids and escalating QW by up to 150% of the previous dose up to 200 mg and then QW by up to 100% until the DLT criteria for that dose level are met.

Cohort B could only escalate to doses that have been cleared by the cohort A (≥2 pts treated at that dose level or above, with ≤ 1 DLT being observed). Therefore, enrollment into cohort B starts at least three weeks after enrollment start into cohort A.

The DLT window is defined as 1 week, to allow dose escalation after 1 week, and a patient is defined as DLT-evaluable if the patient received the full planned dose of RO6958688. The following rules apply separately for cohort A and B:

1. The time interval between dosing of the first and second as well as the second and third patient within each cohort is at least 1 week, to allow for sufficient observation time. The following patients may be dosed any time after the third patient has been dosed.
2. The dose is increased every week within each patient until any of the below applies:
   a. A DLT is observed in this patient. The dose is then reduced to the next lower, and previously tolerated dose level, afterwards.
   b. Less than 6 patients have been treated and ≥ 2 DLTs have been observed at the next higher dose level or below. The dose is then kept constant.
   c. At least 6 patients have been treated and >33% of patients have had DLT at the next higher dose level or below. The dose is then kept constant.
3. The late cycle MTD is defined as follows:
   a. A minimum of 6 patients must have been treated at this dose level or above.
   b. If 6 patients are evaluable for DLT, the late cycle MTD is defined as the highest dose with ≤ 1 DLT having been observed
   c. If more than 6 patients are evaluable for DLT, the late cycle MTD is defined as the highest dose where < 33% of patients have had DLT.

The acceptable characteristics of this the dose escalation design are illustrated with simulations for cohort A in the protocol Appendix 7.

SAFETY ANALYSES

All patients enrolled in the study who received at least one dose of RO6958688 or

“Use of this document is governed by the terms of use on the first page of this document.”

obinutuzumab will be included in the safety population.

Safety analysis will be performed for all the patients in the safety analysis population. All *clinically abnormal* safety parameters will be listed by patient within regimen and summarized in tables*, as appropriate*. Safety will be determined, but not limited to, by adverse events, laboratory tests, vital signs, ECG, physical examinations and performance status. Exposure to study medication will be summarized by total duration of study medication, number of cycles started, cumulative dose and dose intensity using descriptive statistics. Dose modifications, interruptions and their reasons will be presented.

PHARMACOKINETIC ANALYSES

All patients in the safety population will be included in the PK analysis population. Patients will be excluded from the PK analysis population if they significantly violate the inclusion or exclusion criteria, deviate significantly from the protocol, or if data are unavailable or incomplete which may influence the PK analysis. Excluded cases will be documented together with the reason for exclusion. All decisions on exclusions from the analysis will be made prior to database closure.

All PK parameters will be presented by listings and descriptive summary statistics separately by group or cohorts.

Individual and mean serum concentration versus time data for RO6958688 and obinutuzumab will be tabulated and plotted by dose level. The serum for RO6958688 and obinutuzumab concentration data will be analyzed using non-compartmental techniques to estimate the following parameters (when appropriate): AUC, C_{max}, and $t_{1/2}$. Estimates for these parameters will be tabulated and summarized (mean, standard deviation, coefficient of variation, median, minimum, and maximum, *as appropriate*). Inter-patient variability and drug accumulation will be evaluated.

Additional PK analyses will be conducted as appropriate, including population PK modelling.

PHARMACODYNAMIC ANALYSES

PD parameters will be listed by patient and tabulated by dose-level/regimen and timepoint, *as appropriate*. Descriptive statistics will be used in summarizing tumor PD markers. Absolute and percentage change from baseline will be calculated for the PD markers. Graphical techniques *may* be employed to better understand the relationship of the PD markers with dose and time. Correlations between PD markers, PK markers of RO6958688, and clinical response *may* be assessed through data tabulations and graphical techniques in all patients treated or not with obinutuzumab. The potential prognostic value of the PD markers may also be investigated.

EFFICACY ANALYSES

Two efficacy analyses will be performed. The primary efficacy analysis population will consist of all patients who receive at least one dose of any study drug. *If different, an* additional ITT population will consist of all patients who are not screen failures and were included in the trial (ICF signature), and will be used for sensitivity analyses.

Tumor response data will be reported using descriptive statistics. ORR and DCR will be summarized using relative frequencies and *95*% confidence limits. Duration of response and PFS will be summarized using time to event analyses and Kaplan-Meier curves. Overall survival data may be tabulated and summarized if mature. Summaries will be carried out by cohort, dose, and overall*, and duration of response will only be analyzed in those cohorts with a sufficient number of responders*. This will be carried out for both RECIST v1.1 as well as the exploratory irRC efficacy endpoints.

The analysis of tumor response is based on the best overall response (BOR). BOR is defined as the best response recorded from the *start of treatment* until disease progression/recurrence *or* death, whichever occurs first. Since tumor assessments take place every 8 weeks, 60 days is chosen so as to cover these assessments sufficiently.

For the ORR analyses in the primary and ITT efficacy analysis populations, patients who withdraw study treatment because of any reason, die or clinically progress before the first tumor assessment on-treatment will be *assigned a best overall response of Non Evaluable*. These patients will be included in the denominator of the corresponding ORR estimate.

"Use of this document is governed by the terms of use on the first page of this document."

SAMPLE SIZE JUSTIFICATION

The sample size for the dose escalation part is based on the operating characteristics across different assumed toxicity scenarios, as detailed in the body of the protocol. This design has previously been used successfully in Bailey et al. (2009) as well as in Roche with only slight modifications from the current set-up.

The maximum sample size of approximately 30 patients in cohorts C, D, E, F and G each allows for a sufficiently precise estimation of ORR per cancer type under the step-up QWx3/Q3W schedule. Note: Cohort C has completed enrollment with 24 patients. This allows a sufficiently precise estimation of ORR to compare to historical control data. Cohorts D, E, F, and G have not and will not enroll any patients.

The sample size of approximately 15 patients in the Q3W cohort with obinutuzumab pretreatment allows an initial exploration, while the sample size of approximately 40 patients in Cohort H allows for a sufficiently precise estimation of ORR in MSS CRC under the step-up QWx3/Q3W schedule. Cohort H has not and will not enroll any patients.

The sample size for the biomarker cohort is deemed sufficient for initial exploration of the correlation between the lower CEA expression levels (moderate/low, very low and negative CEA expression) and pharmacodynamics and efficacy outcomes. The biomarker cohort has not and will not enroll any patients.

Interim Analyses

Dose escalations throughout the study will occur based on a safety review of the data. These data reviews do not classify as formal interim analysis. In addition, the study will have ongoing safety assessments and safety/preliminary efficacy data reviews which do not qualify as formal safety analyses. There will be ongoing PK analysis from Part I Cohort 1 onwards, and data from Part I will be assessed prior to commencement of Part II.

OTHER CONSIDERATIONS

NA.

LIST OF PROHIBITED MEDICATIONS

The use of the following therapies is prohibited during the study and for at least 28 days prior to initiation of study treatment (unless otherwise specified):

- Investigational or unlicensed/unapproved agents
- Immunotherapy/radio-immunotherapy
- Chemotherapy
- Radiotherapy (with the exception of limited–field palliative radiotherapy)
- Biologic agents (e.g., bevacizumab, erlotinib)
- Chronic use of steroids that exceeds prednisone 10 mg/day or equivalent (inhaled and topical steroids are permitted)
- Administration of a live, attenuated vaccine within 28 days before Cycle 1, Day 1 or anticipation that such a live attenuated vaccine will be required during the study.

"Use of this document is governed by the terms of use on the first page of this document."

LIST OF ABBREVIATIONS AND DEFINITIONS OF TERMS

"Use of this document is governed by the terms of use on the first page of this document."

Abbreviation	Definition
ADA	anti-drug antibody
ALL	acute lymphocytic leukemia
ALP	alkaline phosphatase
AUC	area under the curve
BiTE	bispecific T-cell engager
BLQ	below limit of quantification
BP	blood pressure
CCOD	clinical cutoff date
CD3e	CD3 epsilon chain
CD25	late T-cell activation marker
CD45	leukocyte common antigen
CD69	early T-cell activation marker
CEA	carcinoembryonic antigen
CEACAM	carcinoembryonic antigen-related cell adhesion molecule
CEA TCB (RO6958688)	CEA T-cell bispecific antibody
C_{max}	maximum serum concentration
CRC	colorectal cancer
CRO	contract research organization
CRS	Cytokine release syndrome
CSR	clinical study report
CT	Computed Tomography
CTCAE	Common Terminology Criteria for Adverse Events
cyCD3e	cynomolgus monkey CD3e
cyCEA	cynomolgus monkey CEA
cyCEA TCB (RO6958690)	cynomolgus monkey homologue of RO6958688
DCR	disease control rate
DL_{CO}	Diffusing capacity of the lung for Carbon monoxide
DNA	Deoxyribonucleic acid
FEV1	Forced Expiratory Volume in 1 second
DLT	dose-limiting toxicities
EC	Ethics Committee
ECOG	Eastern Cooperative Oncology Group
eCRF	Electronic Case Report Form
EDC	electronic data capture
EpCAM	epithelial cell adhesion molecule
eRO	estimated receptor occupancy
EU	European Union
EWOC	escalation with overdose control
Fc	Fc portion of IgG
FcγR	Fc gamma receptor binding to the Fc portion of IgG

"Use of this document is governed by the terms of use on the first page of this document."

Abbreviation	Definition
FcRn	neonatal Fc receptor
FDA	U.S. Food and Drug Administration
FDG	2-[^{18}F]Fluoro-2-deoxyglucose
GA101	Obinutuzumab – Gazyva® / Gazyvaro®
GCP	Good Clinical Practice
GGT	γ-glutamyl transferase
GI	gastrointestinal
GLP	Good Laboratory Practice
hCD3e	human CD3e
hCEA	human CEA
HIPAA	Health Insurance Portability and Accountability Act
HTLV-1	Human T-cell lymphotropic virus 1
IB	Investigator's Brochure
ICF	Informed Consent Form
ICH	International Conference on Harmonisation
IEC	Independent Ethics Committee
IFNγ	interferon gamma
IgE	immunoglobulin E
IgG	immunoglobulin G
IHC	immunohistochemistry
IL-2	interleukin-2
IL-6	interleukin-6
IL-8	interleukin-8
IL-10	interleukin-10
IMP	investigational medicinal product
IND	Investigational New Drug (application)
IRB	Institutional Review Board
IRR	infusion-related reaction
irRC	immune–related response criteria
IV	intravenous
LLOQ	lower limit of quantification
MAb	monoclonal antibody
MABEL	minimal anticipated biological effect level
MAD	multiple ascending dose
mCRM	modified continual reassessment method
MoA	mode of action
MRI	magnetic resonance imaging
MTD	maximum tolerated dose
NCI	National Cancer Institute
NK	natural killer
NOG	NOD/Shi-scid/IL-2Rynull

"Use of this document is governed by the terms of use on the first page of this document."

Abbreviation	Definition
NSCLC	non–small cell lung cancer
OBD	optimal biological dose
ORR	overall response rate
PBMC	Peripheral Blood Mononuclear Cell
PD	pharmacodynamic
PET	Positron Emission Tomography
PFS	progression-free survival
PK	pharmacokinetic
PML	Progressive multifocal leukoencephalopathy
PR	partial response
PS	Performance Status
QRS	QRS Complex
QTc	corrected QT
QW	once per week
QWx3/Q3W	once per week three times followed by every 3 weeks
Q2W	every 2 weeks
Q3W	every 3 weeks
RCR	Roche Clinical Repository
RECIST	Response Evaluation Criteria in Solid Tumors
RNA	Ribonucleic acid
RO	receptor occupancy
RO6958688 (CEA TCB)	CEA T-cell bispecific antibody
RO6958690 (cyCEA TCB)	cynomolgus monkey homologue of RO6958688
RR	response rate
SAD	single ascending dose
SAP	Statistical Analysis Plan
sCEA	soluble carcinoembryonic antigen
SD	stable disease
SI	SI units; Système International d'Unités
SOC	standard of care
$t_{1/2}$	half-life
TB	Tuberculosis
TCB	T-cell bispecific antibody
TCR	T-cell receptor
TCZ	Tocilizumab
TIL	tumor infiltrating lymphocytes
TLC	Total lung capacity
TLS	Tumor Lysis Syndrome
TNFα	tumor necrosis factor alpha
ULN	upper limit of normal

"Use of this document is governed by the terms of use on the first page of this document."

Abbreviation	Definition
VC	Vital capacity

“Use of this document is governed by the terms of use on the first page of this document.”

1. BACKGROUND AND RATIONALE

1.1 BACKGROUND AND SCIENTIFIC RATIONALE

Cancer is one of the leading causes of death worldwide. Despite the advances in treatment options, prognosis of patients with advanced cancer remains poor in general. Consequently, there is a persistent and urgent medical need to develop new therapies that can increase survival chances without causing unacceptable toxicity in patients with advanced cancer.

RO6958688 is a novel T-cell bispecific antibody (TCB) that targets carcinoembryonic antigen (CEA) expressed on tumor cells and CD3 epsilon chain (CD3e) present on T cells. RO6958688 is currently under development by Roche (the Sponsor) for the treatment of CEA-positive solid tumors (RO6958688 Investigator's Brochure).

CEA, also called carcinoembryonic antigen–related cell adhesion molecule 5 (CEACAM5) or CD66e, is a protein with molecular weight of 180–200 kDa that belongs to the CEACAM superfamily. CEA is closely related to other CEACAM family members, including CEACAM1, CEACAM3, CEACAM4, CEACAM6, CEACAM7, and CEACAM8, and is anchored to the cell surface via glycosylphosphatidylinositol. CEA also plays a role in cell adhesion, invasion, and metastasis of cancer cells. Endogenous expression of CEA affects the expression of various groups of cancer-related genes, especially genes associated with cell cycles and apoptosis, as it protects colonic tumor cells from various apoptotic stimuli such as treatment with 5-fluorouracil (Soeth et al. 2001).
In addition, CEA inhibits anoikis, a process where cells that are detached from the extracellular matrix subsequently undergo apoptosis (Ordoñez et al. 2000). Therefore, CEA expression may be a way that cancer cells gain a survival benefit and overcome apoptosis-inducing therapies.

CEA expression in various tumor types is generally very high (Thompson et al. 1991). Consistent with the published data, analyses performed by the Sponsor confirmed the high prevalence of CEA in colorectal cancer (CRC), pancreatic cancer, gastric cancer, non–small cell lung cancer (NSCLC), head and neck cancer, and breast cancer among others (see Figure 1), whereas low expression was found in small–cell lung cancer and glioblastoma (RO6958688 Investigator's Brochure). A low level of CEA is expressed on the apical surface of glandular epithelia in the gastrointestinal (GI) tract. However, because of the polarized expression pattern and the luminal localization, the accessibility of CEA to therapeutic antibodies in normal tissues is limited (Thompson et al. 1991; Rudert et al. 1992; RO6958688 Investigator's Brochure).

"Use of this document is governed by the terms of use on the first page of this document."

Figure 1 Carcinoembryonic Antigen Expression across Various Tumor Types

Indications			HIGH	MODERATE/LOW	VERY LOW	NEGATIVE
			≥20% IHC 2+, 3+	<20% IHC 2+3+ and/or ≥20% IHC 1+	<20% IHC 1+ and not IHC 0+	IHC 0+
Disease*	Histology incidence	Samples (n)	%	%	%	%
Colon	Adenocarcinoma	34	91	3	3	3
Pancreas	Adenocarcinoma	43	74	2	5	19
Gastric	Adenocarcinoma	81	64	15	1	20
NSCLC	Adenocarcinoma	28	64	18	4	14
	Ad/Squamous cell carcinoma	69	26	28	7	41
Breast	Invasive ductal carcinoma	76	29	20	3	49
Bladder	Transitional cell carcinoma	152	8	17	5	70
Endom	Adenocarcinoma	191	12	34	10	45
Cervix	Squamous carcinoma	58	45	28	2	26
Esophageal	Squamous carcinoma	84	12	27	6	55
	Adenocarcinoma	30	53	33	3	10
HN	SCC	125	12	31	14	42

CEA = carcinoembryonic antigen; NSCLC = non-small cell lung cancer.

Note: Prevalence analyses for CEA expression across various tumor types as determined by analyses performed by the Sponsor

*Several other subtypes were tested but only those with n>10 are represented

In nonclinical models, the binding of RO6958688 to CEA and CD3 resulted in T-cell–mediated killing of cancerous cells that expressed CEA. RO6958688 effectively mediated the killing of cancer cells only by concurrently binding to CEA present on a tumor cell and to CD3 present on a T cell.

Due to the fact that there is no suitable pharmacologically relevant animal model to assess the toxicity of RO6958688, in vivo toxicity studies were not performed. Instead, the minimal anticipated biological effect level (MABEL) approach was used to define the starting-dose for the entry into human study. Results from in vitro studies on human cells using the most sensitive test systems and assay conditions yielded a MABEL dose for RO6958688 of 52 μg (see the RO6958688 Investigator's Brochure), which is the starting dose for this study.

Additional in vitro studies demonstrated that the ability of RO6958688 to induce cytokine release and proliferation of T cells required concurrent engagement of both CD3 on T cells and CEA on target cells whereas in the absence of the CEA target, this was not observed. A pharmacokinetic (PK) study in male cynomolgus monkeys demonstrated only treatment-related, reversible erythema and bruising at the administration site when

"Use of this document is governed by the terms of use on the first page of this document."

RO6958688 was administered via intravenous (IV) injection. There were no changes observed in clinical observations (e.g., respiratory difficulty, diarrhea, appetite loss, abnormal behaviour [e.g., lack of movement]), body weight, serum chemistry, hematology, coagulation, or urinalysis parameters. Results from a Good Laboratory Practice (GLP)–compliant tissue cross-reactivity study on a full panel of human tissues showed expected staining of epithelial cells consistent with literature reports that describe the expression of CEA (see the RO6958688 Investigator's Brochure).

1.2 BACKGROUND ON RO6958688

RO6958688 is a TCB that targets CEA expressed on tumor cells and CD3e chain present on T cells (see Figure 2).

RO6958688 binds with high affinity and in a bivalent-binding mode to human CEA (hCEA; 0.2 nM). The anti-CEA antibody used in RO6958688 targets a membrane-proximal domain of hCEA (RO6958688 Investigator's Brochure). The anti-CEA antibody binds specifically to hCEA and does not cross-react with cynomolgus monkey CEA (cyCEA) (RO6958688 Investigator's Brochure) because the membrane-proximal domain in hCEA is not conserved across species, including cynomolgus monkey. Because CEA is not expressed in rodents, RO6958688 also lacks cross-reactivity with mice and rats. Thus, a cynomolgus monkey surrogate molecule bearing a binder that specifically binds to cyCEA (RO6958690) was developed for nonclinical toxicology studies.

The anti-CEA antibody was developed in house by humanization followed by affinity maturation and stabilization of the parental murine PR1A3 antibody (Durbin et al. 1994; Ashraf et al. 2009; RO6958688 Investigator's Brochure). It does not have any direct functional activity and is not internalized upon binding (RO6958688 Investigator's Brochure). Because of the targeting of a membrane-proximal domain of hCEA, the anti-CEA antibody displays preferential binding to membrane-anchored CEA rather than shed, soluble CEA (sCEA) (RO6958688 Investigator's Brochure), and the killing potency of RO6958688 remains unaffected up to 0.2 μg/mL of sCEA (RO6958688 Investigator's Brochure).

RO6958688 also binds to T cells through its second binding unit-targeting CD3e of the T-cell receptor (TCR) complex (de la Hera et al. 1991; RO6958688 Investigator's Brochure). The anti-CD3 antibody used in RO6958688 (named CH2527 [VL_7-46(13) VH_23-3(12)]) cross reacts with human CD3e (hCD3e) and cynomolgus monkey CD3e (cyCD3e) chains but not with mouse CD3e chain. This antibody has been generated in house by humanization of the parental SP34 antibody (Pessano et al. 1985; Salmerón et al. 1991; Conrad et al. 2007; RO6958688 Investigator's Brochure). The binding to CD3e is monovalent, which prevents activation of T cells in the absence of simultaneous binding to tumor cells that express CEA, and has low affinity for both hCD3e and cyCD3e (80 nM). The differential binding affinity between CEA and CD3 may favor preferential targeting of RO6958688 to tumors and reduce the peripheral sink due to binding to T cells.

"Use of this document is governed by the terms of use on the first page of this document."

RO6958688 is a human immunoglobulin G1 (IgG1) with the Fc region bearing a novel, proprietary modification (P329G LALA mutation; Figure 2) that abrogates its binding in vitro to Fc γ receptors (FcγR) (RO6958688 Investigator's Brochure) and prevents FcγR-mediated co-activation of innate immune effector cells, including natural killer (NK) cells, monocytes/macrophages, and neutrophils, without changes in functional binding to neonatal Fc receptor, also called FcRn (RO6958688 Investigator's Brochure).

Simultaneous binding of RO6958688 to CEA and CD3 leads to T-cell activation and tumor cell lysis. The RO6958688-mediated tumor cell lysis is CEA-specific and does not occur in the absence of CEA expression or in the absence of simultaneous binding (cross-linking) of T cells to CEA-expressing tumor cells (RO6958688 Investigator's Brochure). In addition to killing, T cells undergo activation followed by tumor lysis as detected by increase of late and early T-cell activation markers (CD25 and CD69, respectively), cytokine release (interferon γ [IFNγ], tumor necrosis factor α [TNFα], granzyme B, interleukin [IL]-2, IL-6, IL-10), and proliferation of T cells (RO6958688 Investigator's Brochure).

Figure 2 Design, Structure, and Required Characteristics of RO6958688

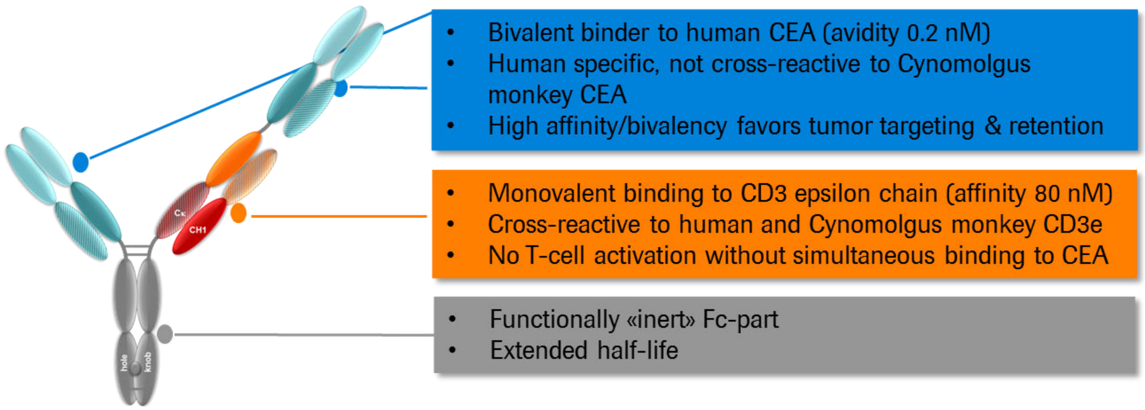

CEA = carcinoembryonic antigen; Fc = Fc portion of IgG; IgG1 = human immunoglobulin G1.
Note: Blue parts correspond to the high-affinity binding antibody (CH1A1A98/99 × 2F1) to hCEA. The red part corresponds to the humanized antibody (CH2527 [VL_7-46(13) VH_23-3(12)]) that binds to the CD3e chain. The grey parts correspond to the heterodimeric Fc region of the human IgG1 bearing the P329G LALA mutation.

1.2.1 Nonclinical Pharmacology Studies

The nonclinical in vitro pharmacology studies conducted with RO6958688 demonstrated that RO6958688 is a potent molecule with proven in vitro activity that displays:

- Target cell–dependent T-cell cytotoxicity (effective concentration that induced 50% of the maximal effect values of tumor cell killing are in the range of 50–750 pM)
- T-cell activation and proliferation and cytokine release in killing assays only in the presence of CEA–expressing target cells

"Use of this document is governed by the terms of use on the first page of this document."

- Activity that correlates with CEA expression levels. Higher potency was observed in high CEA–expressing tumor cells with a threshold of approximately 10,000 CEA binding sites/cell for efficient tumor cell killing.

The nonclinical in vivo pharmacology studies showed that RO6958688 was able to induce:

- Dose- and time-dependent regression of CEA–expressing xenograft tumors grafted with human effector cells from different donors and at different effector to target ratios
- Tumor regression at high (2.5 mg/kg) and low (0.5 mg/kg) antibody dose when administered twice per week and only at 2.5 mg/kg when administered once per week (QW)
- Increased number of tumor-infiltrating leukocytes (TILs), which reflects T-cell recruitment and/or intratumor T-cell expansion (proliferation) during tumor cell killing
- Increased number of intratumor–activated T cells during tumor cell killing, as shown by the high frequency of T cells that express CD69, CD25, 4-1BB, and PD-1 (a hallmark of TCR engagement)
- Higher frequency of apoptotic tumor cells along with strong T-cell infiltration 24 hours after therapy (single injection).

1.2.2 Previous and ongoing Clinical Studies

RO6958688 is currently being tested as a single agent in Study BP29541 and in combination with the PD-L1 checkpoint inhibitor atezolizumab in Study WP29945.

1.2.2.1 Safety of RO6958688

1.2.2.1.1 RO6958688 as monotherapy: Study BP29541

At the clinical cutoff date (CCOD, 27th April 2018), a total of 148 patients have been enrolled and have received at least one dose of either RO6958688 and/or obinutuzumab in Study BP29541. In Study BP29541 on average patients received 10.3 doses/cycles of RO6958688, with a median of 6.0 doses/cycles equating to an average of 76.03 days (1824.8 hours) of treatment with RO6958688 per patient. Overall, adverse events (AEs) were reported in 145 of 148 patients (98.0%). The most common AE reported in ≥25 % of patients was IRR (105 patients [70.9%]), followed by pyrexia (78 patients [52.7%]), diarrhea (67 patients [45.3%]), nausea (53 patients [35.8%]), vomiting and anemia (47 patients [31.8%] each), asthenia (44 patients [29.7%]) and decreased appetite (43 patients [29.1%]). AEs considered related to study treatment by the investigator were reported in 138 of 148 patients (93.2%), with RO6958688–related AEs reported in 137 of 142 patients [96.5%], obinutuzumab-related AEs reported in 8 of 33 patients (24.3%), and AEs related to both RO6958688 and obinutuzumab reported in 3 of 27 patients (11.1%).

A total of 99 of 148 patients (66.9%) reported at least one Grade ≥3 AE with 86 patients (58.1%) reporting an AE with maximum Grade 3 intensity, and 7 patients (4.7%) reporting AEs with maximum Grade 4 intensity. Six Grade 5 AEs were reported: one

“Use of this document is governed by the terms of use on the first page of this document.”

RO6958688-related event of respiratory failure (600 mg RO6958688), one RO6958688-related event of dyspnea (40 mg RO6958688), one RO6958688-related event of IRR (Cohort C: 356 mg RO6958688), one obinutuzumab-related event of sepsis (60 mg RO6958688 Q3W + obinutuzumab), one unrelated cardio-respiratory arrest (Cohort A: 40 mg RO6958688) and one event of unrelated tumor thrombosis (2.5 mg RO6958688).

Serious adverse events (SAEs) were reported in 96 of 148 patients (64.9%) of which IRR (40 patients [27.0%]) was the most frequently reported, followed by pyrexia (8 patients [5.4%]), nausea, pneumonia, dyspnea, tumor pain, and acute kidney injury (5 of 148 patients [3.4%] each). SAEs assessed by the investigator as related to study treatment were reported in 66 of 148 patients (44.6%).

Ninety-four deaths (63.5%) were reported at the CCOD, of which 83 deaths (56.1%) were due to disease progression. There were 6 deaths due to AEs, 4 deaths (2.7%) due to unknown causes (which were confirmed as disease progression following the CCOD), and 1 death was due to sepsis.

Seven dose-limiting toxicities (DLTs) related to RO6958688 as defined in the protocol were reported in 7 of 148 patients (4.7%): Grade 3 dyspnea (40 mg RO6958688; 1 patient), Grade 3 hypoxia (60 mg RO6958688; 1 patient), Grade 3 diarrhea (300 mg RO6958688; 1 patient), Grade 3 colitis (300 mg RO6958688 with obinutuzumab pretreatment; 1 patient) and Grade 4 colitis (600 mg RO6958688; 1 patient), Grade 5 respiratory failure (600 mg RO6958688; 1 patient), and Grade 5 dyspnea (40 mg in Cohort B; 1 patient). The Grade 5 event of dyspnea (40 mg in Cohort B; 1 patient) occurred after the dose escalation part of the trial and was not used to derive the maximum tolerated dose (MTD).

1.2.2.1.2 RO6958688 in combination with atezolizumab: Study WP29945

As of the CCOD (27th April 2018), a total of 226 patients have been enrolled and have received at least one dose of either RO6958688 or atezolizumab in Study WP29945 with all patients reporting at least one AE. In Study WP29945 on average patients received 9.5 doses/cycles of RO6958688, with a median of 5.0 doses/cycles equating to an average of 81.11 days (1946.7 hours) of treatment with RO6958688 per patient. The most common AEs reported in ≥25% patients were IRR (156 of 226 patients [69.0%]), diarrhea (136 patients [60.2%]), pyrexia (120 patients [53.1%]), dysgeusia (81 patients [35.8%]), fatigue (75 patients [33.2%] each), chills (72 patients [31.9%]), vomiting (71 patients [31.4%]), nausea (70 patients [31.0%]), asthenia (69 patients [30.5%]), and decreased appetite (60 patients [26.5%]). AEs considered related to study treatment by the investigator were reported in 222 of 226 patients (98.2%) with RO6958688–related AEs reported in 222 patients (98.2%), atezolizumab-related AEs reported in 183 patients (81.0%) and AEs related to both RO6958688 and atezolizumab reported in 179 patients (79.2%).

"Use of this document is governed by the terms of use on the first page of this document."

A total of 141 of 226 patients (62.4%) reported at least one ≥Grade 3 AE with 122 patients (54.0%) reporting an AE with maximum Grade 3 intensity, and 14 patients (6.2%) reporting an AE with maximum Grade 4 intensity. Five patients (2.2%) experienced a Grade 5 event: one event of hypovolemic shock related to RO6958688 (B1 cohort 600 mg RO6958688), one event of unrelated respiratory tract infection (300 mg RO6958688), one event of unrelated urinary tract infection (Safety cohort pancreatic cohort 600 mg RO6958688), one event of unrelated disseminated intravascular coagulation (DIC; 160 mg RO6958688), and one event of unrelated cerebrovascular accident (80 mg RO6958688).

SAEs were reported in 139 of 226 patients (61.5%) of which IRR (65 patients [28.8%]) was the most frequently reported SAE followed by pyrexia (17 patients [7.5%]) and diarrhea (10 patients [4.4%]). SAEs considered related to study treatment by the investigator were reported in 113 of 226 patients (50.0%).

Fifty-four patients had died (23.9%) at the CCOD, of which 48 deaths (21.2%) were due to progression of disease. There were 5 deaths due to an AE. The remaining death was due to an unknown cause at the time of survival follow-up.

As of the CCOD, 14 DLTs as defined in the protocol have been reported in 12 of 226 patients (5.3%). The following events were considered related to both RO6958688 and atezolizumab: Grade 4 cardiac arrest (100 mg RO6958688; 1 patient), Grade 4 dyspnea (160 mg RO6958688; 1 patient), 2 events of Grade 3 colitis (100 mg and 160 mg RO6958688; 2 patients), 2 events of Grade 3 ALT increased (150 mg and 160 mg RO6958688; 2 patients), Grade 3 AST increased (150 mg RO6958688; 1 patient), Grade 3 stomatitis (40mg RO6958688; 1 patient). The following events were considered related to RO6958688 only: Grade 5 hypovolemic shock (600 mg RO6958688; 1 patient), Grade 4 platelet count decreased (300 mg RO6958688; 1 patient), Grade 3 dyspnea (100 mg RO6958688; 1 patient), Grade 3 rash maculo-papular (120 mg RO6958688; 1 patient; incomplete 160 mg administration), Grade 1 diarrhea (150 mg RO6958688; 1 patient), and Grade 1 dysgeusia (100 mg RO6958688; 1 patient).

A Grade 5 event of hypovolemic shock related to RO6958688 was reported in a [redacted] year old [redacted] patient with microsatellite unstable mCRC and hepatic, pulmonary and peritoneal lesions enrolled into the step-up cohort B1 (atezolizumab 1200 mg Q3W; RO6958688 40 to 1200 mg QW, followed by 1200 mg Q3W) in the WP29945 study. The patient had received two lines of prior standard therapy. Co-morbidities included ongoing hypertension and diabetes mellitus with history of [redacted] and [redacted]. The patient started the first cycle of RO6958688 + atezolizumab on [redacted]. The first 3 administrations (40-150-300 mg) of RO6958688 were associated with adverse events as well as lab abnormalities including constitutional, gastrointestinal, pulmonary symptoms and increased creatinine, liver function tests, hypoalbuminemia and thrombocytopenia. Due to these adverse events, the second cycle (fourth dose of RO6958688) was started with 4 weeks delay on [redacted], consisting of 1200 mg

“Use of this document is governed by the terms of use on the first page of this document.”

atezolizumab and 600 mg RO6958688. Approximately 8 hours after the RO6958688 infusion, the patient developed several symptoms led by severe continuous diarrhea, partially responsive to corticosteroid treatment, with subsequent hypovolemic shock, hypotension, hypoxia, metabolic acidosis and renal failure leading to hyperkalemia. These symptoms initially improved with steroids and supportive care in the ICU. However, on [redacted], the patient experienced ventricular fibrillation, secondary to hyperkalemia per investigator, with successful electric cardioversion, but could not be resuscitated after the 2nd episode of ventricular fibrillation despite intensive care measures. This event was reported by the Investigator as a G5 hypovolemic shock related to RO6958688.

The risk mitigation measures were further optimized following the receipt of two Grade 5 events reported as related to RO6958688 (Grade 5 IRR in BP29541 in section 1.5.1 and Grade 5 Hypovolemic Shock in WP29945 in this section).

1.2.2.1.3 Additional Safety Findings: Infusion-Related Reactions and Cytokine Release Syndrome

As of the clinical cut off (27th April 2018) IRRs were reported as a stand-alone preferred term in 105 of 148 patients (70.9%) in Study BP29541 and in 156 of 226 patients (69.0%) in Study WP29945. The majority was of Grade 1 or Grade 2 severity and readily manageable with protocol-recommended measures. However, 29 patients (19.6%) in Study BP29541 and 39 patients (17.3%) in Study WP29945 reported an IRR with a maximum intensity of Grade 3, and 1 patient in Study BP29541 experienced a Grade 5 IRR (Section 1.5.1). Forty patients (27.0%) in Study BP29541 and 65 patients (28.8%) in Study WP29954 reported at least one serious IRR.

The incidence of IRR was highest at the first infusion. At this timepoint, the majority of patients were anti-drug antibody (ADA) negative and a comparable dose-dependent cytokine release was observed in ADA-negative patients and those who went on to become ADA positive (see 1.2.2.4). The cytokine release that is observed after the first infusion is expected based on the mechanism of action of RO6958688. At the second and subsequent infusions, the incidence of IRR decreased.

IRRs were also more frequent and of higher severity in ADA-positive patients compared with ADA-negative patients (median time to ADA onset is 2.3 weeks). IRRs in ADA-positive patients were more likely to be associated with clinical symptoms of cytokine release syndrome (CRS) (e.g., hypoxia and/or hypotension as observed in the patient from Study BP29541 who experienced a grade 5 IRR). After the fourth infusion of RO6958688, cytokine release remained dose dependent and was higher in ADA-positive patients.

While cytokine peaks observed during initial administrations are thought to represent on-target, tumor-related effects, later cycle peaks likely represent T-cell activation by ADA-mediated crosslinking of CEA-TCB bound CD3.

“Use of this document is governed by the terms of use on the first page of this document.”

Given that IRRs may be indistinguishable from CRS based on symptomatology, single-treatment management guidelines are being recommended for both IRRs and CRS, during or up to 24 hours after infusion of RO6958688. These include the use of tocilizumab as a rescue medication for CRS. See Section 5.2.5.1.5 for more information on IRR/CRS and Table 10 for management guidelines. Please refer to the RO6958688 Investigator's Brochure for additional details on IRR/CRSs adverse events observed in clinical studies.

1.2.2.2 Efficacy of RO6958688

1.2.2.2.1 RO6958688 as monotherapy: Study BP29541

As of 16 June 2017, tumor response assessment data using RECIST criteria v1.1 were available from 86 of 102 patients (84.3%) in Study BP29541. Of the 86 efficacy-evaluable patients, 5 patients treated with 0.052-2.5 mg RO6958688 QW were enrolled in Part I and 81 patients were enrolled in Part II of which 63 patients received flat doses of 2.5 mg-600 mg RO6958688 QW alone, 8 patients received step-up doses of 40-1200 mg RO6958688 QW (Cohorts A and B), and 10 patients received 135-300 mg RO6958688 QW following obinutuzumab pretreatment. Tumor responses were evaluated 8-12 weeks after treatment or at any unscheduled tumor response assessment by using RECIST criteria v1.1. Efficacy evaluable patients had at least one administration of RO6958688 and at least one tumor assessment after treatment start.

Partial response was observed in a total of 2 of 86 patients (2.3%) with CRC. One of 35 patients (2.9%) in the 60-600 mg RO6958688 dose group received a starting dose of 200 mg RO6958688 QW and was dose escalated to 300 mg with the longest PR observed at Day 94 (unscheduled visit following Cycle 8) accompanied by a 68% reduction of target lesions and 1 of 10 patients (10.0%) received obinutuzumab pretreatment followed by RO6958688 QW at a starting dose of 135 mg and was dose-escalated to 200 mg with the longest PR observed at Day 225 (Cycle 32) accompanied by a 61% reduction of target lesions. Progressive disease has been confirmed in both patients, after response durations of 94 and 224 days, respectively. Stable disease was observed as best overall response in a total of 30 of 86 (34.9%) patients which included 1 of 5 patients (20.0%) in Part I who received RO6958688 QW at a starting dose of 0.15 mg and escalated to 20 mg, 23 of 63 patients (36.5%) in Part II who received flat doses of 2.5-600 mg RO6958688 QW with 10 of 28 patients (35.7%) treated with 2.5-40 mg RO6958688 and 13 of 35 patients (37.1%) treated with 60-600 mg RO6958688, 3 of 10 patients (30.0%) in Part II who received 135-300 mg RO6958688 QW following obinutuzumab pretreatment, and 3 of 5 patients (60.0%) in Cohort A in Part II who received step-up doses of RO6958688 QW starting at 40 mg up to 1200 mg. The longest SD was at Day 441 (Cycle 64) observed in a patient dosed with RO6958688 QW at 60 mg and escalated up to 400 mg. This patient continued to have SD at the time of clinical cutoff date.

Forty-four patients (50.6%) had progressive disease as best overall response and

“Use of this document is governed by the terms of use on the first page of this document.”

10 (11.6%) patients had missing or non-evaluable responses.

As of 14 July 2017, there were 2 confirmed partial responses (PR) by RECIST 1.1 out of 31 CRC MSS patients (7%) enrolled in the ≥ 60 mg QW RO6958688 and step up cohorts A and B. Two additional PR responses were confirmed after this latter cutoff date.

Refer to the RO6958688 Investigator's Brochure for additional information on the clinical efficacy of RO6958688.

1.2.2.2.2 RO6958688 in combination with atezolizumab: Study WP29945

As of 16 June 2017, tumor response assessment data using RECIST criteria v1.1 were available for a total of 61 of 82 patients (74.4%) in Study WP29945. Of the 61 efficacy-evaluable patients, 2 patients received previous RO6958688 treatment in Study BP29541 prior to Cycle 1 Day 1 combination treatment of RO6958688 and atezolizumab; data from these 2 patients were analyzed and are presented separately. Efficacy evaluable patients received at least one dose of RO6958688 in combination with at least one dose of atezolizumab and had at least one tumor assessment after treatment start. Tumor responses were evaluated 8 weeks after treatment with 5-300 mg RO6958688 QW in combination with a fixed dose 1200 mg of atezolizumab Q3W or at any unscheduled tumor response assessment.
Of the 59 efficacy-evaluable patients not treated previously with RO6958688, PR was achieved as best overall response in 5 of 44 patients (11.4%) in the 80-300 mg RO6958688 dose group. All 5 patients received 160 mg RO6958688 QW in combination with 1200 mg atezolizumab Q3W. The longest PR was observed at Day 225 (unscheduled visit following Cycle 9) accompanied by 69% reduction of target lesions compared to baseline, and is ongoing at the time of clinical cutoff with a duration of response of 168 days. Additionally, 2 of these 5 patients have ongoing responses of 28 and 87 days duration at the time of clinical cutoff, respectively. The remaining 2 patients achieved a PR followed by progression of disease after 118 and 58 days, respectively.

A total of 25 of 59 patients (42.4%) had SD as best overall response: 4 of 15 patients (33.3%) treated with 5-40 mg RO6958688 and 21 of 44 patients (47.7%) treated with 80-300 mg RO6958688 in combination with atezolizumab. The longest SD was observed at Day 337 (Cycle 17) in a patient who received RO6958688 QW at starting dose of 20 mg and escalated up to 160 mg in combination with atezolizumab. Twenty-six patients (44.1%) had progressive disease as best overall response and 3 patients (5.1%) had missing or non-evaluable responses.

Two patients with CRC who received previous RO6958688 treatment until disease progression in Study BP29541 prior to enrollment in Study WP29945, had SD as the best overall response following combination treatment of atezolizumab with escalating RO6958688 doses from 40 mg to 160 mg (1 patient) and a RO6958688 dose of 160 mg

"Use of this document is governed by the terms of use on the first page of this document."

(1 patient) in Study WP29945.

As of 14 July 2017, there were 4 confirmed partial responses (PR) by RECIST 1.1 out of 31 CRC MSS patients (13%) enrolled in the 160 mg QW RO6958688 cohort. An additional PR response was confirmed after this latter cutoff date.

Refer to the RO6958688 Investigator's Brochure for additional information on the clinical efficacy of RO6958688.

1.2.2.3 Clinical Pharmacokinetics of RO6958688

As of 14th May 2018, PK data from a total of 65 ADA-negative patients from Study BP29541 and 80 ADA-negative patients from Study WP29945 were included in a population PK analysis. RO6958688 concentration time profiles exhibited a biphasic disposition with an initial rapid distribution phase followed by a slower elimination phase. In ADA–negative patients, PK was time independent, i.e., serum exposure was maintained after multiple doses. The median CL and V_{ss} values were 0.071 L/h and 0.059 L/h, 9.8 L and 9.4 L, respectively, in Studies BP29541 and WP29945.

RO6958688 CL does not depend on dose. Similarly, after the first infusion, maximum concentration (C_{max}) and area under the concentration time curve (AUC) values of the first dosing interval are dose proportional, i.e. RO6958688 exhibited linear pharmacokinetics.

1.2.2.4 Immunogenicity of RO6958688

As of 7th June 2018, ADA data from 132 patients from Study BP29541 and 193 patients from WP29945 were available, while PK data of 142 and 226 patients were available. In ADA-positive patients, time dependent PK can occur after multiple IV infusions of RO6958688 with reduced or no detectable exposure.

In patients treated with RO6958688 as a single agent and without obinutuzumab pretreatment (Study BP29541) and RO6958688 in combination with atezolizumab (Study WP29945), the development of ADAs directed against RO6958688 was observed in 48% (51 of 106 patients) and 64% (123 of 193 patients) of patients, respectively; RO6958688 exposure was reduced below the limit of quantification at C_{max} in 26% (30 of 115 patients) and 16% (35 of 226 patients) of patients, respectively. Median time to onset of no detectable exposure of RO6958688 was 5.0 and 7.1 weeks in Studies BP29541 and WP29945, respectively.

After obinutuzumab pretreatment in Study BP29541, 42% (11 of 26 patients) of patients were ADA-positive; of these, 9 of 11 patients (81.8%) had transient ADAs and in the remaining 2 patients the last available ADA sample was early, i.e. at 3 and 9 weeks after the first dose of study drug (RO6958688). The maximal observed ADA-titer of 270 in obinutuzumab pretreated patients was low as compared to 196,830 in non obinutuzumab pretreated patients and RO6958688 exposure was sustained with similar PK profiles to those in ADA-negative patients.

"Use of this document is governed by the terms of use on the first page of this document."

1.3 BACKGROUND ON OBINUTUZUMAB

Obinutuzumab is a humanized and glycoengineered type II anti-CD20 monoclonal antibody that recognizes the CD20 antigen present on normal and malignant B-cells. It is being used and developed for the treatment of hematological malignancies, namely non-Hodgkin lymphoma (NHL) and chronic lymphocytic leukemia (CLL). Showing a significant benefit over rituximab, obinutuzumab in combination with chlorambucil has been approved in more than 86 countries for the treatment of previously untreated CLL indication under the name of Gazyva®/Gazyvaro®. Approval for the first lymphoma indication, relapsed-refractory FL, in combination with bendamustine, was granted on 26 February 2016 by the FDA and on 13 June 2016 by EMA. Obinutuzumab was derived by humanization of the parental B-Ly1 mouse antibody and subsequent glycoengineering leading to the following characteristics: high-affinity binding to the CD20 antigen, high antibody-dependent cellular cytotoxicity (ADCC), and antibody-dependent cellular phagocytosis (ADCP); low complement-dependent cytotoxicity (CDC) activity; and high direct cell death induction.

1.3.1 Previous Nonclinical Studies

Non-clinical in vitro studies show that obinutuzumab mediates superior induction of direct cell death and effector cell-mediated ADCC and ADCP on a panel of NHL cell lines as compared to the Type I CD20 antibodies rituximab and ofatumumab. Its potency to mediate CDC is significantly reduced as compared to these two antibodies.

Ex vivo autologous B-cell depletion assays with whole blood from healthy donors were performed. It can be hypothesized that the autologous whole blood assay measures the combined effect of ADCC-, CDC-, and direct cell death-inducing mechanisms of action and likely reflects the in vivo situation in peripheral blood most closely. Taken together, the comparison of obinutuzumab and rituximab using the whole blood from a panel of 10 healthy donors showed that in comparison to rituximab, obinutuzumab was 10- to 25-fold more potent in terms of half maximal effective concentration (EC50) values and 1.5- to 2.5-fold more efficacious in terms of absolute B-cell depletion.

Obinutuzumab displayed the highest capacity of B-cell depletion, regardless of the FcγRIIIa genotype and antibody concentrations used, both in terms of EC_{50} values and absolute B-cell depletion (Weiner 2010). Mechanistic studies showed that compared to obinutuzumab, ofatumumab and rituximab more strongly rely on CDC for efficient B-cell depletion in the whole blood assay.

These properties of obinutuzumab translated into superior anti-tumor efficacy in direct comparison to rituximab against a number of aggressive subcutaneous (SC) and disseminated NHL xenograft models. The efficacious and optimal dose range of obinutuzumab in xenograft models was in the range of 10-30 mg/kg, corresponding to trough levels of 300-600 μg/mL.

"Use of this document is governed by the terms of use on the first page of this document."

Treatment with obinutuzumab also resulted in potent and superior depletion of B-cells in the peripheral blood and in lymphoid tissues of hCD20 transgenic mice and cynomolgus monkeys. Vaccination studies in cynomolgus monkeys and human CD20 transgenic mice showed that the enhanced efficacy in terms of B-cell depletion of obinutuzumab translated into suppression of de novo antibody responses, but left the protective humoral memory responses intact.

Obinutuzumab (10 and 30 mg/kg) induced a superior depletion of B-cells in the lymph nodes of cynomolgus monkeys compared to rituximab (10 mg/kg) following 2 IV doses administered on Days 0 and 7 (Mössner 2010). In addition, there was a trend for better efficacy of obinutuzumab in depleting peripheral and spleen B-cells at the early time points (Mössner 2010). In a second study, the efficacy of obinutuzumab at depleting B-cells was compared with that of non-glycoengineered obinutuzumab and rituximab at a dose of 30 mg/kg following two IV doses administered on Days 0 and 7. The results demonstrated that obinutuzumab and non-glycoengineered obinutuzumab possess a greater capacity and durability for depleting B-cells from both blood and lymph nodes of cynomolgus monkeys compared to rituximab. These data indicate that the superior activity of obinutuzumab is not only due to the glycoengineering, but also due to the direct type II CD20 antibody-related mechanisms.

1.3.2 <u>Previous and ongoing Clinical Studies</u>

1.3.2.1 Studies in B-cell malignancies with obinutuzumab

Obinutuzumab is being investigated as monotherapy or combination therapy in more than 18 clinical trials in patients with hematological malignancies, including CLL and NHL.

As of 30 April 2017, an estimated 1497 patients with CLL and 2191 patients with NHL from the 18 company sponsored studies have been exposed to obinutuzumab, either as monotherapy or in combination therapy.

Of particular interest, infusion-related reactions (IRRs) and hypersensitivity reactions were observed consistently in all obinutuzumab trials; the highest incidence of IRR was at the first infusion with the incidence decreasing rapidly with subsequent infusions. The incidence of IRR observed with combination therapy (FC and CHOP) appears similar to that observed with monotherapy. Furthermore, the incidence of IRR appears to be higher in CLL compared to NHL patients and higher in obinutuzumab- compared to rituximab-exposed patients based on evidence from studies BO21003 and BO21999.

AEs of particular interest include IRRs and hypersensitivity reactions, Tumor Lysis Syndrome (TLS), thrombocytopenia (including acute thrombocytopenia), neutropenia (including late onset and prolonged neutropenia), prolonged B-cell depletion, infections including progressive multifocal leukoencephalopathy (PML) and hepatitis B virus (HBV) reactivation, worsening of pre-existing cardiac conditions, gastrointestinal (GI) perforation and second malignancies.

"Use of this document is governed by the terms of use on the first page of this document."

Severe and life-threatening neutropenia including febrile neutropenia has been reported during treatment with obinutuzumab. Patients who experience neutropenia should be closely monitored with regular laboratory tests until resolution. Cases of late onset neutropenia (occurring 28 days after the end of treatment) or prolonged neutropenia (lasting more than 28 days after treatment has been completed/stopped) have also been reported.

Severe and life-threatening thrombocytopenia including acute thrombocytopenia (occurring within 24 hours after the infusion) has been observed during treatment with obinutuzumab.

Progressive multifocal leukoencephalopathy has been reported in patients treated with obinutuzumab.

Based on the mechanism of action (rapid tumor lysis) and on the fact that cases of GI perforation were observed in obinutuzumab treated patients (mainly in NHL), it is considered an important identified risk of obinutuzumab. GI perforation may result in peritonitis with a potentially fatal outcome. It generally requires surgical intervention. Please refer to obinutuzumab Investigator's Brochure for more information.

1.3.2.2 Clinical Studies in solid tumors with obinutuzumab

A clinical trial in solid tumors patients where obinutuzumab pretreatment is being investigated to assess whether it can lower the Anti-Drug Antibodies (ADA) formation upon RO6895882 treatment (Roche sponsored BP28920/obinutuzumab sub-study) is ongoing. As of 29 January 2018, 33 patients received a pre-treatment of obinutuzumab 2000 mg, the dose was split in two infusions of 1000 mg, and administered on two consecutive days, Day-13 and Day-12 prior to treatment start with RO6895882 (CEA-IL2v) on Cycle 1 Day 1. Five out of these 21 patients discontinued prior to the first dose of CEA-IL2v.

Based on a preliminary safety analysis, the safety toxicity profile of obinutuzumab pre-treatment was acceptable in patients with locally advanced and/or metastatic solid tumors. Fourteen patients (42.4%) experienced AEs at the C1D1 of obinutuzumab infusion. Six patients (18.2%) experienced adverse events which were of Grade 1/2. Two patients (6.1%) experienced Grade 3 adverse events leading to early discontinuation, one unrelated ileus and one infectious enterocolitis related to obinutuzumab and underlying disease (colorectal cancer patient). In addition, seventeen (51.5%) patients withdrew from the study due to disease progression.

BP29435 is an open label multi center, dose escalation study of the safety, pharmacokinetics and therapeutic activity of cergutuzumab amunaleukin, an immunocytokine, which consists of a variant of interleukin 2 (IL-2v), that targets carcinoembryonic antigen (CEA) and atezolizumab, an antibody that targets programmed death-ligand 1 (PD-L1), administered intravenously, in patients with locally advanced and/or metastatic solid tumors.

"Use of this document is governed by the terms of use on the first page of this document."

In this study a fatal hepatoxicity event has been reported. The adverse event occurred in a [redacted] year-old [redacted] patient with advanced pancreatic cancer. Liver function was assessed as appropriate (baseline levels of AST, ALT, Bilirubin were ≤ Grade 1, GGT was Grade 3). [redacted] received pre-treatment with obinutuzumab 2000 mg and seven days later was started on atezolizumab 1200 mg every 3 weeks (Q3W) and cergutuzumab amunaleukin 10 mg every week (QW). Transient liver function test (LFT) elevations were observed after each administration of study treatment but resolved to baseline levels prior to the next exposure. After cycle 2 (second atezolizumab administration, fourth CEA-IL2v administration), [redacted] developed Grade 4 AST and ALT elevations and Grade 3 hyperbilirubinemia. The drug induced liver damage resulted in liver failure, which did not resolve. The patient succumbed to CNS edema secondary to the liver failure. Transient LFT elevations are a known risk for CEA-IL2v therapy and have been described in obinutuzumab and atezolizumab treated patients. Based on the analyses performed by the Sponsor, it was concluded that LFT elevations are manageable in patients treated with obinutuzumab, CEA-IL2v and atezolizumab given in monotherapy or double combinations. However triple combination (obinutuzumab pre-treatment followed by CEA-IL2v and atezolizumab) was stopped in all trials and obinutuzumab was removed.

In a preliminary analysis of ADAs in patients receiving obinutuzumab pretreatment, none of the 4 analyzed patients presented with positive ADA titers up to Cycle 4 included (i.e. 8 weeks) after the first CEA-IL2v administration, whereas 15/23 patients not pre-treated with obinutuzumab presented positive ADA titers up to 4 weeks included. Patients pre-treated with obinutuzumab did all receive CEA-IL2v in a QW schedule. If those numbers are compared to patients who were not pre-treated with obinutuzumab and who received RO6895882 QW, where 5/7 patients presented with positive ADA titers up to 4 weeks included, it suggests that obinutuzumab pre-treatment reduces ADA formation.

In order to evaluate the impact of pre-medication with obinutuzumab on the occurrence of the infusion-related AEs starting within 24 hours of infusion in BP29541, the frequency and maximum severity of infusion-related AEs between patients pretreated with obinutuzumab has been compared with remaining patients treated with RO6958688 alone in the 60-400 mg range. In total, 33 patients have been treated with obinutuzumab pre-medication, 6 of which did not receive RO6958688 afterwards. Patients pre−treated with obinutuzumab received RO6958688 at doses of 135-300mg QW and 40mg or 60mg Q3W. For comparison, all patients (n=35) at similar dose levels, which ranged from 60-400 mg QW, and who did not receive obinutuzumab pre-medication have been included. It is worth noting that some patients pre-medicated with obinutuzumab received less infusions (Q3W) than patients not pre-medicated (QW) thus making it complex to interpret the results so far. Patients from the step-up cohorts have been excluded from this comparison as the obinutuzumab premedication cohorts did not include a step-up of RO6958688.

Regarding IRR, the overall frequency of IRRs is 20 of 27 patients (74.1%) premedicated with obinutuzumab and 20 of 35 patients (57.1%) in the group of patients that were not pre-medicated. From this overall analysis as well as the analyses of PTs, there was no evident difference in the overall frequencies of infusion-related AEs between the two groups. Obinutuzumab pre-treated patients do seem to have lower frequency of Grade 3 events (33.3%) compared to the patient who were not pre-treated (45.7%).

“Use of this document is governed by the terms of use on the first page of this document.”

Obinutuzumab pre-treated patients (n=27) who also received RO6958688 had all of these events lower than the control group (n=35): pyrexia, chills, vomiting, diarrhea, nausea, hypotension, dyspnea, rash, rash pruritic, rash maculo-papular, urticarial, tachycardia and headache, most based on larger number of patients and notable differences. Apart from IRR, only other AEs which were reported at higher rate in obinutuzumab pre-treated patients were: fatigue [n=5 (18.5%) vs. 4 (11.4%) in control group], malaise [n=2 (7.4%) vs. n=2 (5.7%) in control group] and bronchospasm [n=1 (3.7%) vs. 0 (0%) in control group.

From the above data it is evident that obinutuzumab pre-treatment reduces ADA formation. Also based on data from BP29541, obinutuzumab pre-treatment seems to decrease the incidence and severity of infusion-related AEs. However, the safety of obinutuzumab in patients with solid tumors has not been established especially when given as pre-treatment with an Immune doublet combination and is currently being studied further. The safety of obinutuzumab in patients with solid tumors has not been established and is currently being studied.

For more detailed information, please refer to the Effects in the Human (Section 5) and Guidance For The Investigator (Reference Safety Information, Section 6) of the current obinutuzumab Investigator Brochure.

1.4 BACKGROUND ON TOCILIZUMAB (RO4877533, ACTEMRA®, ROACTEMRA®)

Tocilizumab, blocks IL-6 from binding to its receptor, both in membrane-bound and soluble states (Singh et al 2011). With a primary indication for juvenile idiopathic arthritis (JIA), tocilizumab is approved by the Food and Drug Administration (FDA) for children as young as 2 years. It is also approved for adults with rheumatoid arthritis, adults with Giant Cell Arteritis (GCA) and for Castleman disease in Japan. Tocilizumab has been extensively studied in adults, with 8 randomized controlled trials treating more than 2000 patients (Singh et al 2011) and in children in phase 1 to phase 3 trials for JIA (Woo et al 2005, Yokota et al 2005, De Benedetti et al 2012).

In patients with severe CRS associated with T cell–engaging therapies, IL-6 levels peak during maximal T cell proliferation. A growing body of evidence suggests that IL-6 blockade by tocilizumab result in rapid, dramatic reversal of life-threatening CRS in patients treated with T cell engaging therapies (Grupp et al 2013, Teachey et al 2013). Whereas tocilizumab *IV* is typically dosed every 4 weeks *in rheumatoid arthritis*, extended treatment is not necessary in the management of CRS, which is self-limited and in most cases reported to require a single administration in order to control the clinical signs of CRS after treatment with T cell engaging agents *(Le et al 2018)*. Tocilizumab has been recently approved by the FDA (August 2017) and *the European Medicines Agency has recently approved the extension of indication to include "treatment of chimeric antigen receptor (CAR) T cell-induced severe or life-threatening cytokine release syndrome (CRS) in adults and pediatric patients 2 years of age and older"* (Tocilizumab USPI).

"Use of this document is governed by the terms of use on the first page of this document."

As part of the data review of BP29541 and WP29945, we observed a correlation between peaks of serum IL-6 and the occurrence of IRRs and associated symptoms of hypotension or hypoxia or dyspnea within 24 hours of RO6958688 infusion. However, the majority of these peaks are less than 1000 pg/mL. Given that hypotension and hypoxia are key symptoms of CRS, based on the current information, CRS may have occurred in some patients experiencing IRR with RO6958688 and underlying symptoms of hypotension and hypoxia. Tocilizumab should be administered for management of IRR and CRS considered related to RO6958688 per guidelines in Table 10.

1.5 STUDY RATIONALE AND BENEFIT-RISK ASSESSMENT

Despite great progresses in recent years, management of patients with many types of advanced solid tumors remains highly challenging. The importance in understanding the cellular and molecular events that take place during tumorigenesis and cancer development has promoted further research for more targeted therapies that aim to improve survival chance of patients who suffer from advanced cancer.

There has been renewed interest over the last few years in engagement of the immune system in the battle against cancer, with multiple approaches currently under nonclinical and clinical investigations. One such approach currently in development is to activate T cells against the tumor by using TCBs.

The Sponsor has developed a novel TCB (RO6958688) that targets the human CEA on tumor cells and CD3 on T cells, which results in T-cell–mediated killing of cancerous cells that express CEA.

In addition, we aim to explore whether obinutuzumab given as a pretreatment to the RO6958688 administration delays and/or attenuates the development of ADA directed against RO6958688.

As of 17 June 2016, PK data are available from 60 patients (5 and 49 patients respectively from study Part I and II) treated with RO6958688 monotherapy with and without obinutuzumab pretreatment.

Following multiple IV infusions of RO6958688, time-dependent PK was observed in some patients, with reduction or total loss of exposure. ADAs against RO6958688 were detected with first appearance occurring between Cycle 2 (QW) and Cycle 9 (QW) (pre-infusion). In contrast and as of 21 February 2018, ADAs against RO6958688 were not detected in 17/27 patients pretreated with obinutuzumab 2 weeks prior to RO6958688 administration and 10/17 had low, transient ADA titers (except in one patient, where after positive sample no further ADA sample is available). All patients had maintained RO6958688 exposure. Thus far, there is no clinical evidence of ADA-mediated adverse events in BP29541 study, however the sponsor is conducting ongoing analyses to assess any potential ADA-driven safety impact. More detailed information can be found in the RO6958688 Investigator's Brochure.

"Use of this document is governed by the terms of use on the first page of this document."

Identification and development of strategies to overcome or inhibit ADAs is of great interest. The immunological processes leading to formation of ADAs remain poorly understood. The production of ADAs occurs within days/weeks after treatment exposure and is mainly mediated by the activation of T cells and subsequent stimulation of B cells, leading to generation of plasma B cells. Nevertheless, animal and clinical studies revealed that immunogenicity against recombinant human proteins shares both T-cell dependent (such as requirement of CD4+ T cells, isotype switching) and T-cell independent (involvement of marginal zone B lymphocytes, apparent lack of memory) characteristics (Gomez-Mantila).

Betaferon® is the first commercially available rhIFNβ product used for treatment of patients with multiple sclerosis. The treatment has been shown to evoke ADA formation in up to 60% of patients, resulting in a reduction or complete blockage of its therapeutic effect. In an immune tolerant mouse model it had been demonstrated that the immunogenicity of Betaferon® is a T cell independent (Tind) response, which is characterized by a lack of memory formation and involvement of CD4+ T cells and more importantly marginal zone (MZ) B cells. The inactivation of MZ B cells at the start of Betaferon® treatment lowered ADA levels significantly (Sauerborn).

Morbus Pompe is a rare disease caused by deficiency of lysosomal acid α-glucosidase (GAA) enzyme requiring enzyme replacement therapy (ERT) with recombinant human GAA (rhGAA). In patients in whom GAA is not produced, a status called cross-reactive immunologic material (CRIM)-negative, ERT with rhGAA has led to high titer antibodies against rhGAA, with an ultimately fatal outcome. In an initial report from 2009, successful tolerance induction in a CRIM-negative patient was achieved with the anti-CD20 monoclonal antibody rituximab (4 weekly doses at 375 mg/m^2 followed by a maintenance dose every 4 to 12 weeks) in combination with methotrexate (at a dose of 0.5 mg/kg added to the regimen after 7 weeks) and intravenous immune globulin (at a dose of 500 mg/kg every 4 weeks) resulting in declining antibody titers, finally becoming negative 7.5 months after the start of ERT (Mendelsohn). In a follow-up report, this patient and an additional patient treated with the same regime was reported being off all immune-suppressive therapy, having B-cell recovery, and being tolerant to rhGAA (Messinger). The above described immune tolerance inducing regimen was administered together with the first dose of ERT and was described to be safe and well tolerated.

The pre-treatment prevented or diminished the development of antibody titers, resulting in a better clinical outcome compared to patients treated with ERT monotherapy (Banugaria).

The role of B cell functions in antitumor immunity is not well understood. The possible therapeutic benefit of B-cell depletion in combating tumoral immune escape has been debated and there is recent evidence suggesting that B cells can both promote and inhibit the development and progression of the tumors (Affara, Gunderson).

"Use of this document is governed by the terms of use on the first page of this document."

Nevertheless, there is a lack of clinical evidence of an effect of B cell depletion in patients with solid tumors. Fifteen patients with RCC and six patients with melanoma were treated with rituximab as an adjunct to IL-2. The safety profile of the sequential administration of rituximab followed by IL-2 was favourable and the serum immunoglobulin levels were not affected (Kim, Bodogai, Aklilu).

Obinutuzumab is an anti-human CD20 antibody with improved in vitro effector function and the ability to induce direct B cell death in vitro. In pre-clinical evaluation, obinutuzumab mediated superior B cell depletion with respect to the duration, the dose range for depletion and the depletion of the splenic marginal zone B cell subset as compared to rituximab. Under all doses, peripheral blood B cells, lymph node B cells and splenic B cells were reduced by both obinutuzumab and rituximab in all tissues by day 7 compared to the vehicle treated control animals (0 mg/kg). Obinutuzumab reduced splenic, lymph node and peripheral blood B cell numbers significantly more strongly than rituximab over the entire dose range, reaching lowest dose for maximal B cell depletion in peripheral blood and lymph nodes at the low dose of 0.5 mg/kg compared to 1 mg/kg rituximab and reaching lowest dose for maximal splenic B cell depletion between 1-2 mg/kg dose range compared to the 10-20 mg/kg dose of rituximab. These results demonstrate that obinutuzumab appeared to be more efficacious at depleting tissue B cells of human CD20 transgenic mice compared to rituximab in a mouse model.

Furthermore, the ability to deplete a minor B cell subset, residing in the marginal sinus of the splenic follicular regions called marginal zone B cells (MZ B cells) has been observed with obinutuzumab while not with rituximab. These marginal zone B cells are involved in splenic antigen filtration, trafficking and early humoral responses. The inactivation of this subset by an intraperitoneal injection of 100 µg anti-LFA (αLβ2) and 100 µg anti-CD49 (anti-α4) at 2 days before the start of the treatment with rhIFNβ has resulted in a significant lowering of the overall anti-rhIFNβ IgG titers in Betaferon© treated mice (Sauerborn).

To evaluate the functional impact that the increased extent of B-cell depletion with obinutuzumab versus rituximab has on the humoral immune response to foreign antigens, cynomolgus monkeys were immune challenged after treatment with either a novel antigen that the animals had never experienced before (de novo response to tetanus toxoid) or with a booster immune rechallenge with an immunogen that the animals had already encountered prior to the CD20 antibody administration (memory-recall response to measles/rubella).

Animals were administered on Day-14 and Day-7 rituximab or obinutuzumab at a dose of 30 mg/kg or vehicle by i.v. infusion. Immunization with tetanus toxoid was performed on Day 0. Naïve animals from all groups had a baseline anti-tetanus toxoid IgG measurement of around 0.1 IU/ml at day 0. At Day 7, vehicle treated animals and rituximab treated animals mounted robust humoral anti-tetanus toxoid IgG responses, with an increase to around 1.0 IU/ml, while obinutuzumab treated animals showed

“Use of this document is governed by the terms of use on the first page of this document.”

attenuated responses, resulting in an equal to background signal of 0.1 IU/ml. By Day 21, serological titers in vehicle treated and rituximab treated animals continued to rise to peak levels of 2.5 IU/ml (Figure 3). The obinutuzumab treated animals began to display a slight increase in titers to 0.5 IU/ml, which was significantly below of that of vehicle and rituximab treated groups. The serum IgG response waned by day 51 and 68 in all groups reaching around 1.5 IU/ml vehicle, 1.3 IU/ml rituximab and returning to 0.2 IU/ml in obinutuzumab treated animals.

Figure 3 Prior treatment with obinutuzumab but not rituximab or vehicle results in the attenuation of tetanus toxoid specific de novo IgG antibody responses in cynomolgus monkeys

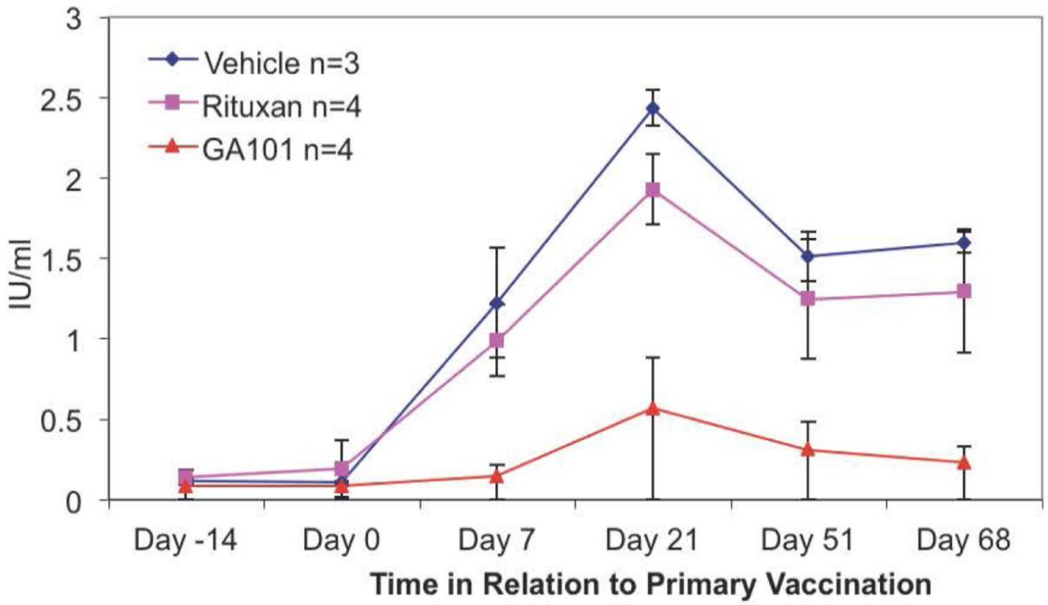

These results indicate that prior treatment with obinutuzumab results in the attenuation of tetanus toxoid specific de novo IgG antibody responses in cynomolgus monkeys.

To investigate the memory recall responses by measles, specific IgG antibody production in response to immune re-challenge with measles/rubella booster vaccination animals that had measurable baseline positive anti-measles titers were selected. Animals were administered rituximab or obinutuzumab at a dose of 30 mg/kg or vehicle by I.V. infusion on Day-14 and Day-7. Immunization with tetanus toxoid was performed on Day 0. Measuring the fold change at optical density OD450 nM reading over baseline Day-14 reading, resulted in measurable increase in anti-measles titers in all three groups at Day 21, Day 51 and Day 68 with no significant difference found in the IgG responses to measles amongst the three different treatment groups. The conclusion is that memory recall responses were left intact regardless of anti-CD20 depletion therapy (Figure 4).

"Use of this document is governed by the terms of use on the first page of this document."

Figure 4 Memory recall responses by measles specific IgG antibody production in response to immune re-challenge with a measles/rubella booster vaccination in animals with baseline titers to measles is not affected by either obinutuzumab or rituximab in cynomolgus monkeys

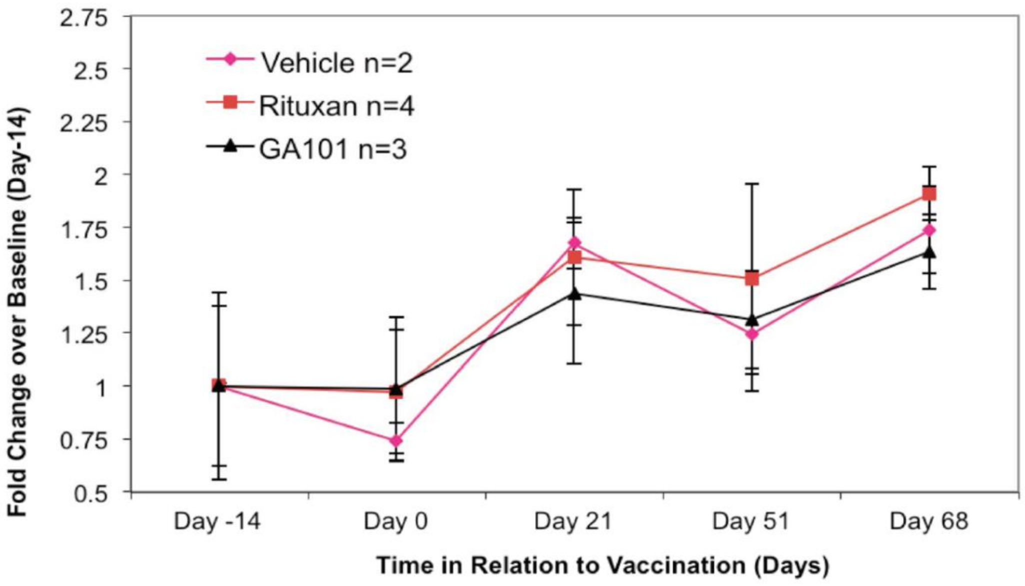

The administration of either obinutuzumab or rituximab had no measurable impact on the memory recall responses to a booster immunization against measles/rubella vaccination with established basal titers as a result of prior vaccination.

Overall, these results showed that the obinutuzumab enhanced efficacy in B-cell depletion translated into stronger suppression of de novo antibody responses, but left the protective humoral memory responses intact. The ability to block de novo humoral antibody responses may possibly be attributed to either the increased extent of endogenous B-cell depletion seen with obinutuzumab and/or the enhanced ability of obinutuzumab to deplete activated, CD20 expressing B cells.

Potential Overlapping Toxicities of obinutuzumab and RO6958688

Based on the mode of action and the non-clinical and/or clinical studies with each molecule as a single agent, infusion related reaction/cytokine release syndrome has been identified as a potential overlapping toxicity in patients treated with obinutuzumab and RO6958688 (Table 1).

Potential and identified risks associated with obinutuzumab and RO6958688 are described in the respective single-agent IBs and summarized in Table 1. It is important to note that some overlaps identified are purely hypothetical, e.g. IRR from obinutuzumab treatment would be resolved by the time of RO6958688 administration. Also, protocol would require for some of these toxicities to resolve before administering RO6958688 (please refer to Section 5.2.4).

"Use of this document is governed by the terms of use on the first page of this document."

Table 1 Obinutuzumab and RO6958688: Overview of Identified and Potential Risks with Treatment

	Identified risk of treatment with study drug	Potential risk of treatment with study drug
RO6958688	Infusion-related reaction (IRR), cytokine release syndrome (CRS) pyrexia, diarrhea, Adverse events associated with Tumor Inflammation/Flare [tumor flare, tumor pain, dyspnea, hypoxia, increased liver enzymes (AST/ALT)], bilirubin, colitis/enteritis, chills, vomiting, rash, skin exfoliation, nausea, dysgeusia, fatigue, arthralgia, hypoxia, hypotension, arthritis	Asthenia, decreased appetite, risk of IgE-mediated Hypersensitivity Reactions Including Anaphylaxis Hematotoxicities, Central Nervous System Disorders
Obinutuzumab	IRR and hypersensitivity reactions, TLS, Thrombocytopenia, Neutropenia, Late onset neutropenia, Prolonged neutropenia, Prolonged B-cell depletion, Infections, PML, Hepatitis B reactivation, Worsening of pre-existing cardiac conditions, GI perforation	Immunogenicity, Impaired Immunization Response, Second Malignancies

Collectively, the data discussed above provides a strong scientific rationale to explore the potential of obinutuzumab given as a pretreatment prior to treatment with RO6958688 in order to attenuate development of ADAs.

Based on a preliminary safety analysis on BP28920/obinutuzumab substudy and on the current BP29541 study (see Section 1.3.2.2), obinutuzumab pre-treatment was well tolerated in the population of locally advanced and/or metastatic solid tumor patients; hence a similar safety profile with RO6958688 is anticipated.

The available nonclinical, clinical and class safety data for both agents, provide guidance for monitoring safety of patients in this trial. Preliminary data from BP28920/obinutuzumab substudy suggest that by administering obinutuzumab prior to first RO6958688 infusion, would prevent or reduce ADAs and their impact on systemic

"Use of this document is governed by the terms of use on the first page of this document."

exposure of RO6958688 and therefore increasing the benefit of treatment to patients and maintain favorable risk/benefit ratio for the obinutuzumab pretreatment.

1.5.1 Identified Risks with RO6958688

The following adverse events are classified as identified risks associated with RO6958688: pyrexia, IRR, CRS, diarrhea, tumor inflammation/tumor flare events at the tumor site (tumor pain, dyspnea, hypoxia, increased liver enzymes [AST/ALT], increased blood bilirubin, colitis), chills, vomiting, rash, skin exfoliation, nausea, dysgeusia, fatigue, hypoxia, hypotension, arthritis, arthralgia, increased liver enzymes (AST/ALT), and bilirubin elevation.

Serious adverse drug reactions that are considered expected and associated with RO6958688 include: pyrexia, IRR, CRS, tumor pain, diarrhea, colitis, dyspnea, hypotension, hypoxia, increased AST, increased ALT, and elevated bilirubin.

The following adverse events are classified as potential risks associated with RO6958688: asthenia, decreased appetite, IgE-mediated hypersensitivity reactions, including anaphylaxis, hematologic toxicities, and CNS disorders.

Refer to Section 6 of the RO6958688 Investigator's Brochure for a detailed description of all anticipated risks for RO6958688.

RO6958688 treatment leading to a grade 5 related AE in BP29541:

On [redacted], a generalized inflammation of large paratracheal pathological lymph nodes induced by RO6958688 causing obstruction of the trachea leading to acute respiratory failure Grade 5 occurred in a patient treated with a dose of 600 mg RO6958688 in the dose-escalation part of the BP29541 study. The patient had received the full planned dose (600 mg) and the event started within 24 hours after administration of the first RO6958688 dose (C1D1).

On [redacted], a mCRC patient with extensive bilateral lung metastasis and dyspnea at baseline treated with an initial dose of 40mg RO6958688 in the step up Cohort A, died due to worsening of dyspnea Grade 5 assessed as related to RO6958688. The patient started treatment on [redacted] and at the end of infusion developed Grade 3 acute respiratory insufficiency with hypoxia, tachypnea and tachycardia, which improved with treatment with corticosteroids and oxygen and was kept hospitalized. A chest CT performed on [redacted] revealed an inflammatory reaction in both lungs. On [redacted], the patient was found unconscious [redacted] without oxygen support and with severe hypoxia. The [redacted] refused advance medical support and the patient passed away likely due to worsening of respiratory insufficiency, reported as worsening dyspnea, Grade 5.

“Use of this document is governed by the terms of use on the first page of this document.”

Another patient was reported to have suffered a Grade 5 IRR following the fifth administration of RO6958688 at a dose of 355.99 mg (planned dose 600 mg). The patient is suspected to have had a fatal CRS event following multiple re-treatment attempts with RO6958688 based on a very high serum IL-6 level of 75198.41 pg/mL, elevated levels for IFN-γ (1694.96 pg/mL), IL-8 (4328.17 pg/mL) and TNF-α (767.06 pg/mL) after the fourth administration, with a very similar clinical course at the fifth administration of RO6958688 (Cycle 5 cytokine levels are not available). This patient had a recurring symptom of Grade 3 hypotension related to RO6958688 infusion at both administrations. Following this G5 event a Dear Investigator Letter was issued on 16 February 2018 (refer to Section 1.2.2.1.3).

For additional information please refer to the RO6958688 Investigator Brochure. For clinical guidance management, please refer to section 5.2.5.

1.5.2 Rationale for [18F] FDG-PET Imaging

2-[18F] Fluoro-2-deoxyglucose (FDG), a glucose analog is taken up by high metabolic activity cells, phosphorylated, and trapped in the cell. 2-[18F] Fluoro-2-deoxyglucose positron emission tomography (FDG-PET) can identify sign of biological effect early before tumor size is reduced. Moreover, a reduction in the FDG-PET signal within days or weeks of initiating therapy (e.g., in lymphoma, non–small cell lung, and esophageal cancer) has been shown to correlate with prolonged survival and other clinical end points now used (Weber).

In preclinical findings, RO6958688 induces T cell-mediated killing of tumor cells at the tumor site, while an untargeted TCB (having the same format but not binding to tumor cells) has no effect on tumor cell killing demonstrating the relevance of tumor targeting for RO6958688 activity. In this clinical study, FDG-PET will be used to assess the effects of RO6958688 at tumor sites by measuring changes in glucose metabolism as a surrogate for cell viability.

[^{18}F] FDG-PET will be performed at baseline and on-treatment at week 6 (C6D5 -72/0 hours) for the QW schedule, at week 6 (C2D19 -72/0 hours) for the Q3W schedule and at week 6 (C4D19 -72/0 hours) for the QWx3/Q3W schedule, to determine changes in glucose metabolism of the tumor lesions. Patients with no evidence of FDG uptake at screening will not be required to undergo follow-up FDG-PET.

1.5.3 Rationale for the new RO6958688 dose schedules and for the step up dosing

In a preclinical model imaging study with radiolabeled CEA CD3 TCB, tumor targeting was shown (Roche Report No.1072962). CEA CD3 TCB was retained in the tumor lesions for at least 120 hours (last time point of the trial). Consistent with the above data, a clinical imaging study with a different labeled molecule (^{89}Zr CEA-IL2v) was undertaken. This drug targeted the same CEA epitope and utilizes the same CEA binding CDR. Tumor accumulation was observed at least until day 8, while over

“Use of this document is governed by the terms of use on the first page of this document.”

90% of the antibody was cleared from serum within 4 days (ESMO 2015 oral presentation, Tabernero et al). The above data suggest that the retention of the antibody in the tumor lesions could be more relevant than the blood exposure to define the best schedule for this T cell bi-specific antibody.

In order to confirm this hypothesis, we propose to enroll patients into a step up dosing regimen combining the QW and the Q3W RO6958688 administration schedules (QWx3 followed by Q3W).

The exploration of these schedules for RO6958688 are intended to help identify a well-tolerated starting dose, a well-tolerated late cycle maximal dose, an appropriate step up dosing regimen, and associations between these parameters on both early efficacy and prolonged/durable efficacy.

The proposed schemes aim to explore the impact of the prophylactic 4 doses of prednisone (40 mg) or methylprednisolone (40 mg) on the prevention of lesion tumour inflammation–mediated safety events reaching ≥ Grade 3, and on the anti-tumor efficacy by comparing the flat dose approach requiring prophylactic steroid (QW and Q3W schedules) with the step up dosing approach (QW schedule) that does not require prophylactic steroid.

So far from the 12 patients treated at 40 mg without prophylactic steroids, only one patient with NSCLC and chronic obstructive pulmonary disease at screening experienced a transient Grade 3 dyspnea adverse event at C1D2, considered related, which resolved in 5 days. Other Grade 3 related events at this dose level were not considered related to tumor inflammation and were not unique to this dose (1 presyncope and 1 syncope in the same patient and 2 events of Grade 3 IRR). Dose cohort of 40 mg is similar to other cohorts over 40 mg (excluding 600 mg which exceeds MTD) in the rate of all and related ≥ Grade 3 events. This initial 40 mg dose is therefore considered appropriate and will be escalated QW until the late cycle MTD is defined.

Overview of Preliminary Safety Data for the Step Cohorts A, B, and C Treated with up to the1200 mg of RO6958668: Study BP29541

As of 27th April 2018, 45 patients with locally advanced and/or metastatic solid tumors had received at least one dose of RO6958688 in one of three step-up dose cohorts with a starting dose of 40 mg and weekly dose escalation up to 1200 mg RO6958688. (Cohort A: 40-80-160-300-400-600-800-1200 mg; Cohort B: 40-100-200-400-800-1200 mg and Cohort C 40-150-300-600 mg).

All 45 patients (100%) experienced at least one adverse event (total number of adverse events at the time of the cutoff was 579 across all step up cohorts). Nineteen patients (42.2%) experienced a Grade ≥3 adverse event related to RO6958688 and 26 patients (57.8%) had a serious related adverse event. Twenty-three patients (51.1%)

“Use of this document is governed by the terms of use on the first page of this document.”

experienced adverse events which led to dose modification or interruption, and one patient (2.2%) had an adverse event which led to withdrawal from treatment. Three adverse events (6.7%) were fatal, 2 of which were related to RO6958688 treatment (Grade 5 dyspnea and Grade 5 IRR) The grade 5 IRR event occurred after the 5th administration of RO6958688, when the patient was positive for anti-RO6958688 ADA and was characterized by high levels of cytokines (see Section 5.2.5.1.5). Serious IRRs have been reported in 18 of 45 patients (40.0%) in the step up cohorts compared to 11 out of 49 patients (22.4%) in the 40-600 mg flat dose cohorts. In general, the incidence of late cycle cytokine peaks is higher in ADA positive patients, and tends to be more pronounced in step up cohorts. Late cycle cytokine peaks likely represent T-cell activation by ADA-mediated crosslinking of CEA-TCB bound CD3 (see section 5.5.1.6.1 of the latest IB). Although AEs associated with infusions have been empirically mostly reported as IRRs, it is plausible that, due to correlation with cytokine peaks, most if not all of these AEs could have been also reported as CRS. Step up dosing will no longer be evaluated in the RO6958688 program as more serious IRRs as well as higher and more frequent late cycle cytokine peaks were observed in the step-up cohorts compared to the flat cohorts.

For more details, please refer to the RO6958688 Investigator Brochure.

2. OBJECTIVES

2.1 PRIMARY OBJECTIVES

The primary objectives of this study are:

- To assess the safety profile of RO6958688 with/without obinutuzumab pretreatment
- To determine the maximum-tolerated dose (MTD) and/or the recommended dose and schedule (optionally with obinutuzumab pretreatment) for further development
- To determine the late cycle maximum tolerated dose (late cycle MTD)
- To establish the pharmacokinetics of RO6958688 as monotherapy with/without obinutuzumab pretreatment
- To assess the effect of obinutuzumab pretreatment in decreasing the rate of patients with positive Anti-Drug Antibodies (ADA) titer against RO6958688 at week 8 and/or delaying the time of onset of ADA against RO6958688.

2.2 SECONDARY OBJECTIVES

The secondary objectives for this study are:

- To obtain preliminary anti-tumor activity data of RO6958688 with/without obinutuzumab pretreatment on objective overall response rate (ORR), duration of response (DOR), disease control rate (DCR; defined as response rate [RR] + stable disease [SD]) and progression-free survival (PFS) according to Response Evaluation Criteria in Solid Tumors (RECIST), Version 1.1 criteria and immune–related response criteria (irRC), by investigator assessment for the whole study. If Sponsor decides, independent central read for computed tomography (CT)

"Use of this document is governed by the terms of use on the first page of this document."

or magnetic resonance imaging (MRI) might be performed in this study, both prospectively and retrospectively.

- To characterize pharmacodynamic (PD) effects and duration of PD response for the QW**,** for the Q3W regimens, for the step up dosing scheme (QW and QWx3/Q3W) on the basis of an increase in activated intratumoral T cells.

2.3 EXPLORATORY OBJECTIVES (PART II ONLY)

The exploratory objectives for this study are:

- To explore the relationship between exposure, pharmacodynamics, and clinical effects of RO6958688 with/without obinutuzumab pretreatment
- To characterize in tumors and peripheral blood cells the changes in PD biomarkers (proliferation, activation, infiltration) associated with multiple doses and schedules of RO6958688 treatment
- To investigate potential predictive PD biomarkers from paired tumor biopsies and paired blood samples (including but not limited to $CD3^+$, $CD4^+$, $CD8^+$, and T cells)
- To explore the relationship between exposure, metabolic activity by the tumor, pharmacodynamics, and clinical effects of RO6958688
- To investigate tumor mutations, gene expression and other biomarkers (such as CEA expression in various tumor types) related to RO6958688 therapy
- To explore preliminary safety and efficacy in low/moderate and very low CEA expressing tumors.
- To assess ADA specificity in patients without obinutuzumab pretreatment
- To make a preliminary assessment of the effectiveness of tocilizumab (Actemra®/RoActemra®) in ameliorating the symptoms of severe CRS following RO6958688 treatment.

3. STUDY DESIGN

3.1 DESCRIPTION OF STUDY

Note: Following an internal review of the clinical development plan of RO6958688, the Sponsor has decided to permanently discontinue further enrollment of patients in this study and to not open enrollment Cohorts D through H and the Biomarker Cohort.

Study BP29541 is a first in-human, open-label, multicenter, dose-escalation Phase I clinical study of single-agent RO6958688. The study will be conducted in two parts. Part I of the study is single ascending dose [SAD] (see Section 3.1.1) in single patient cohorts to evaluate the safety of RO6958688 at the doses that are expected to be below relevant biological effects (starting from a receptor occupancy [RO] of 0.11% for the CD3e receptors), and Part II is multiple ascending dose [MAD] (see Section 3.1.1) with a dose-finding part where RO6958688 is given QW (± 1 day) or Q3W (± 2 days) to define the MTD and/or the recommended dose for further development. Sponsor may also open in Part II (cohorts either with QW flat dose, Q3W flat dose or step up dosing scheme, patients pretreated with obinutuzumab), in order to

"Use of this document is governed by the terms of use on the first page of this document."

assess if obinutuzumab pretreatment would decrease the incidence and/or delay the onset of ADA directed against RO6958688. There will be a parallel multiple ascending dose escalation in cohorts of patients receiving obinutuzumab pretreatment. Interim data from another study (BP28920/obinutuzumab) with another molecule developed by the Sponsor (RO6895882) suggest that a single dose of 2000 mg of obinutuzumab may be sufficient to prevent or delay ADA formation (Section 1.3.2). QW dosing of RO6958688 will be implemented initially to generate data that can be analyzed to assess whether different dosing schedules are more effective. For week 1 (and week 2 in the QW schedule) MTD has been defined as 400 mg.

Cohort A and Cohort B will define the late-cycle MTD and the maximum intra-patient dose escalation increments by a modified 3+3 dose escalation in two cohorts of patients, the cohorts will be run in a staggered approach with Cohort A (late cycle MTD) starting before Cohort B (maximum increments) and cohort B will not be escalated to a dose not already declared as safe in cohort A:
Cohort A) approximately 6 evaluable patients at 40 mg in Cycle 1 without prophylactic steroids and escalating QW by up to 100% of the previous dose until the DLT criteria for that dose level are met and
Cohort B) approximately 6 evaluable patients at 40 mg in Cycle 1 without prophylactic steroids and escalating QW by up to 150% of the previous dose up to 200 mg and then QW by up to 100% until the DLT criteria for that dose level are met.

A patient will be evaluable for the purpose of the intra-patient dose escalation in cohorts A and B if continuing escalation of the dose until 1) the DLT criteria for that dose level are met or 2) the highest dose level allowed by the modified 3+3 design is reached or 3) 1200 mg dose is reached (whichever applies first). Otherwise, the patient will not be evaluable for the purpose of the intra-patient dose escalation, and hence may be replaced.

Additional patients will be enrolled following a step up dosing schedule where RO6958688 will be administered QW for the first 3 administrations then followed by Q3W administration (Cycle 1 with RO6958688 at 40 mg [QW], C2 with RO6958688 at 150 mg [QW], Cycle 3 with RO6958688 at 300 mg [QW], then from Cycle 4 onwards with RO6958688 at 600 mg [Q3W]). Up to five different cohorts (with/without obinutuzumab pretreatment) will enroll up to 30 CRC patients (Cohort C), up to 30 gastric cancer patients (Cohort D), up to 30 pancreatic cancer patients (Cohort E), up to 30 breast cancer patients (Cohort F) and up to 30 non-small cell lung cancer patients (Cohort G). Cohorts D, E, F and G will open once the dose regimen has been defined. An additional cohort (Cohort H) will enroll approximately 40 MSS CRC patients who will receive obinutuzumab pretreatment followed by a step up dose regimen with RO6958688 QWx3 / Q3W (40 mg QW at Cycle 1, 100 mg QW at Cycle 2, 150 mg QW at Cycle 3 and from Cycle 4 onwards 150 mg Q3W). For these cohorts patients will also be monitored for DLT and the safety rules from the intra-patient dose escalation design described in Section 6.7.1.2 apply separately for each cohort (i.e. by indication). Note:

“Use of this document is governed by the terms of use on the first page of this document.”

Cohort C has enrolled 24 CRC patients; no further patients will be enrolled. Cohorts D through H did not and will not enroll any patients.

For RO6958688 flat dosing (i.e. not body weight-adjusted) has been selected for all parts of the study because no significant effect on body surface area or body weight on overall exposure is expected. RO6958688 will be administered via IV infusion. At the discretion of the investigator and in case there is no safety concern, all patients can receive further doses QW at the same dose level or the next available tolerated dose level that has been cleared for dose escalation after they have completed 2 months of treatment at the current dose level. The treatment period for this protocol is 24 months for RO6958688 and may be modified if supported by emerging data. Because of the potential for progression prior to response with immune therapies, patients who exhibit clinical benefit will continue treatment beyond radiographic progression after discussion and agreement with the Sponsor.

The patients in the obinutuzumab cohorts will receive according to patient's and/or investigators convenience, either 2000 mg of obinutuzumab IV on Day-13 or 1000 mg of obinutuzumab IV on two consecutive days, Day-13 and Day-12 (± 2 days) before C1D1 RO6958688 administration. In Cohort H, the patients will receive according to patient's and/or investigators convenience, either 2000 mg of obinutuzumab IV on Day-7 or 1000 mg of obinutuzumab IV on two consecutive days, Day-8 and Day-7 (+ 1 day) before C1D1 RO6958688 administration. Note: Cohort H did not and will not enroll any patients.

3.1.1 Overview of Study Design

The study will be conducted in two parts (see Figure 6). Part I and Part II of the trial will enroll patients with locally advanced and/or metastatic CEA–positive solid tumors who have progressed on standard treatment, are intolerant to SOC, and/or are non-amenable to SOC. For week 1 (and week 2 in the QW schedule) MTD has been defined as 400 mg.

Cohorts A and B will define the late cycle MTD and the maximum intra-patient dose escalation increments by a modified 3+3 dose escalation design**,** where the cohorts will be run in a staggered approach. Cohort A (late cycle MTD) will start before Cohort B (maximum increments) and cohort B will not be escalated to a dose not already declared as safe in cohort A as follows (see Figure 5 and Section 6.7.1.2 for details):
Cohort A) approximately 6 evaluable patients at 40 mg in Cycle 1 without prophylactic steroids and escalating QW by up to 100 % of the previous dose until the DLT criteria for that dose level are met and Cohort B) approximately 6 evaluable patients at 40 mg in Cycle 1 without prophylactic steroids and escalating QW by up to 150% of the previous dose up to 200 mg and then QW by up to 100% until the DLT criteria for that dose level are met.

"Use of this document is governed by the terms of use on the first page of this document."

Figure 5 QW step up dosing scheme and late cycle MTD

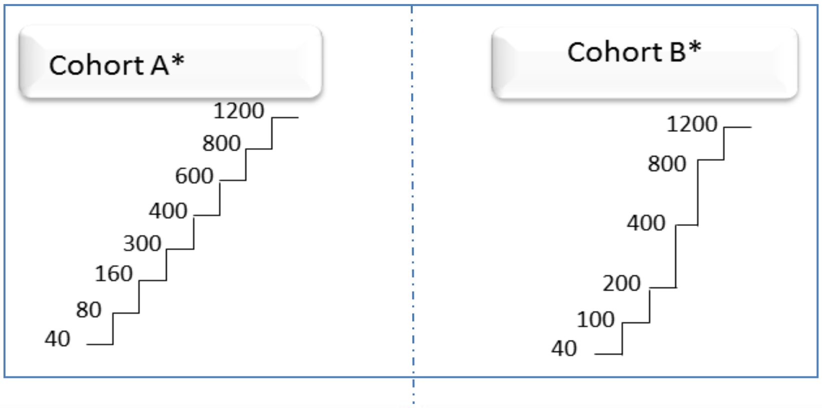

Cohort A: 6 patients

- Start with 40 mg
- Escalate with max. 100% increments up to 300 mg, then max. 50% increments to maximum 1200 mg
- Modified 3+3 dose escalation design

Cohort B: 6 patients**

- Start with 40 mg
- Escalate with max. 150% increments up to 200 mg, then max. 100% increments to 1200 mg
- Modified 3+3 dose escalation design

* Shown doses are maximum doses per cycle, (QW) additional intermediate doses will be considered if safety management requires
** Cohort B could only escalate to doses that have been cleared by the cohort A (i.e. ≥2 pts, ≤1 DLT)

Additional patients will be enrolled following a step up dosing schedule (with/without obinutuzumab pretreatment) where RO6958688 will be administered QW for the first 3 administrations followed by Q3W administrations (Cycle 1 with RO6958688 at 40 mg [QW], C2 with RO6958688 at 150 mg [QW], Cycle 3 with RO6958688 at 300 mg [QW], then from Cycle 4 onwards with RO6958688 at 600 mg [Q3W]). Up to five different cohorts will enroll up to 30 CRC patients (Cohort C), up to 30 gastric cancer patients (Cohort D), up to 30 pancreatic cancer patients (Cohort E), up to 30 breast cancer patients (Cohort F) and up to 30 non-small cell lung cancer patients (Cohort G). For these cohorts patients will also be monitored for DLT and the safety rules from the intra-patient dose escalation design described in Section 6.7.1.2 apply separately for each cohort (i.e. by indication). Note: Cohort C has enrolled 24 CRC patients; no further patients will be enrolled. Cohorts D through G did not and will not enroll any patients.

In parallel to the enrollment of Cohorts A-H, approximately 20 additional patients (all solid tumors following Cohort C dose/schedule) may be enrolled into a biomarker cohort based on their CEA expression level (moderate/low, very low/negative CEA expression) in order to initially explore the correlation between CEA expression, efficacy and safety of RO6958688. Note: The biomarker cohort did not and will not enroll any patients.

"Use of this document is governed by the terms of use on the first page of this document."

Figure 6 Study Schema

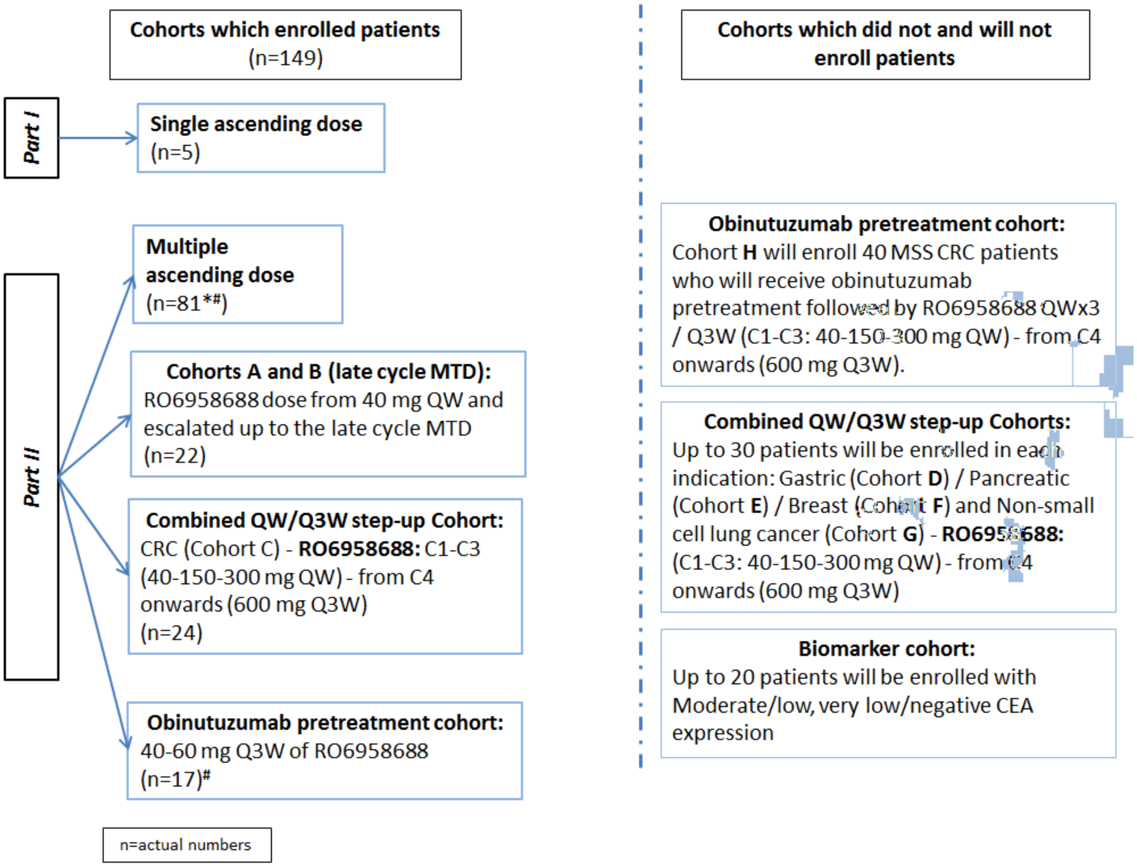

Note: The enrollment of n=149 reflects the 148 patients enrolled as of the clinical cut-off date, plus one last patient enrolled and treated in Part II afterward.

MTD=maximum tolerated dose; CEA =carcinoembryonic antigen.

Note: Part I will include single patient cohorts, whereas Part II will include at least 3 patients within a cohort (with and without obinutuzumab pre-treatment) during the dose escalation part.

* These cohorts have enrolled 10 patients with obinutuzumab pretreatment.

A total of 6 patients received only obinutuzumab pretreatment.

Part I: Single Ascending Dose (SAD)

The objective of Part I of the study is to investigate the safety and pharmacokinetics of a single dose (QW; one cycle) of RO6958688 in single patient cohorts with dosing starting from a MABEL dose of 0.05 mg and up to a maximum dose of 2.5 mg. Up to five single doses of 0.05 mg, 0.15 mg, 0.45 mg, 1.3 mg, and 2.5 mg of RO6958688 are planned to be tested initially in 1 patient each. The doses from 0.15 mg to 2.5 mg may be revised at the discretion of the Sponsor and investigator should the PK exposure be lower than predicted.

The cohorts will include single patients in order to minimize the number of patients treated below the therapeutically relevant dose. The results of Part I of the study will determine the starting dose for Part II. All patients will be monitored for a 14-day safety observation period prior to enrollment of the next patient at the next dose level.

"Use of this document is governed by the terms of use on the first page of this document."

A dose is considered safe for the purposes of Part I if no RO6958688–related adverse event of Grade 3 or higher is reported in a patient during the 14-day safety observation period. If the dose is considered safe by the Sponsor and the participating investigators, a new patient will be enrolled to receive a higher dose of RO6958688.

If an RO6958688-related event of Grade 3 or higher is reported in a patient during the 14-day safety observation period, Part I of the study will be ended and the dose used in Part II of the study will be at least at one dose level below the dose at which the adverse event occurred. If no RO6958688-related event of Grade 3 or higher is reported in any patient during the 14-day safety observation period in Part I of the study up to the maximum dose of 2.5 mg, the starting dose used in Part II will be 2.5 mg.

All patients in Part I of the study will receive, as a minimum, one dose of RO6958688. At the discretion of the investigator and in case there is no safety concern, all patients can receive further doses QW at the same dose level. Dose escalation to the next available tolerated dose level can only proceed after the patient has tolerated the dose he or she was enrolled at, for at least 2 months (this was only applicable to patients in Europe).

Part II: Multiple Ascending Dose [MAD] Escalation (Dose Finding with Continual Reassessment and Overdose Control)

Part II of the study will establish the appropriate dose(s) based on safety, pharmacokinetics, and the MTD and/or the recommended dose of RO6958688 for the QW and/or the Q3W regimens (one cycle equals one week in the QW regimen and one cycle equals three weeks in the Q3W regimen). The starting dose in Part II will be 2.5 mg or lower if Part I is closed at a dose below 2.5 mg. In that case, the starting dose will be the highest and safe dose achieved in Part I at which no RO6958688–related adverse events of Grade 3 or higher were reported during the 14-day observation period.

In order to assess whether obinutuzumab pretreatment can decrease the incidence and/or delay the onset of ADA directed against RO6958688, parallel cohorts of patients receiving obinutuzumab pretreatment will be enrolled. The RO6958688 starting dose for patients with obinutuzumab pretreatment will be the ongoing dose under assessment when the amendment is approved. The patients in the obinutuzumab cohorts will receive according to patient’s and/or investigators convenience, either 2000 mg of obinutuzumab IV on Day-13 or 1000 mg of obinutuzumab IV on two consecutive days, Day-13 and Day-12 (± 2 days) before C1D1 RO6958688 administration. In Cohort H, the patients will receive according to patient’s and/or investigators convenience, either 2000 mg of obinutuzumab IV on Day-7 or 1000 mg of obinutuzumab IV on two consecutive days, Day-8 and Day-7 (+ 1 day) before C1D1 RO6958688 administration. Premedication will be given prior to each obinutuzumab dosing. For these patients, the baseline tumor biopsy will be taken before receiving the first dose of obinutuzumab and the on-treatment tumor biopsy will be undertaken either at C2D1 or C4D1 if the patient will

“Use of this document is governed by the terms of use on the first page of this document.”

receive RO6958688 flat dose or at C2D1 or C7D1 if the patient will receive RO6958688 step up dosing. Note: Cohort H did not and will not enroll any patients.

The Sponsor has completed the enrollment of cohorts with obinutuzumab pretreatment, 11 patients in the MAD cohorts and 17 patients who received RO6958688 at 60 mg Q3W (Note: 2 patients were dosed with 40 mg). In the Cohort H, approximately 40 MSS CRC patients will receive obinutuzumab pre-treatment followed by a step up dose regimen with RO6958688 QWx3 / Q3W (40 mg QW at Cycle 1, 100 mg QW at Cycle 2, 150 mg QW at Cycle 3 and from Cycle 4 onwards 150 mg Q3W) (see Figure 6).
Note: Cohort H did not and will not enroll any patients.

Dose-escalation for the QW regimen will be carried out according to a modified-Continual Reassessment Method with Overdose Control (mCRM with EWOC) design in order to define MTD and/or the recommended dose, separately for RO6958688 with/without obinutuzumab pretreatment. At least 10 patients will be enrolled in the dose escalation cohorts with obinutuzumab pretreatment. The MTD is defined as the dose that maximizes the probability of a DLT being in the targeted toxicity interval in the range of 20%–35%, subject to the probability of the DLT being in the excessive toxicity interval in the range of 35%–100% being < 25%. Patients within a cohort (3 patients each) will be enrolled in a sequential manner, which, if required, can be expanded with additional patients. Each patient will be observed for 21 days for DLT assessment, and there is no escalation planned during the DLT period. The first patient in each cohort will be observed for safety for 1 week before additional patients are enrolled in the cohort. Once a minimum of 3 patients have completed the 21-day DLT observation period, the Sponsor and investigators will evaluate the next recommended dose using the EWOC design and agree on the dose for the subsequent cohort. If a DLT is already reported in the first patient during the first week of safety observation, the Sponsor will organize a teleconference with the investigators to discuss the safety and tolerability of RO6958688 and to decide whether the subsequent patient in the same cohort will be enrolled at the same dose. At the end of the DLT period, the same dose might also be escalated in the second cycle instead of the first cycle for the subsequent cohort if in the second and later cycles, the safety profile is better on average and/or the exposure is lower than in Cycle 1. For patients who enroll in Part II and who will be treated at dose levels equal and greater than 5 mg (≥ 5 mg), a tumor biopsy for PD analyses is mandatory at baseline and on-treatment (see Section 3.3.2.2). Baseline and on-treatment tumor biopsies will be performed after the FDG-PET. At the discretion of the investigator and in case there is no safety concern, all patients can receive further doses QW at the same dose level. For each patient separately, dose escalation to the next available tolerated dose level can only proceed after the patient has tolerated the dose he or she was enrolled at, for at least 2 months.

Cohorts may be expanded to collect additional PD information to support and/or confirm the mechanism of action and explore doses below the MTD in relation to the PD effects in order to eventually define a recommended dose.

"Use of this document is governed by the terms of use on the first page of this document."

For week 1 (and week 2 in the QW schedule) MTD has been defined as 400 mg. This amendment will define the late cycle MTD and the maximum intra-patient dose escalation increments by a modified 3+3 dose escalation design where the cohorts will be run in a staggered fashion with Cohort A (late cycle MTD) starting before Cohort B (maximum increments) and cohort B will not be escalated to a dose not already cleared as safe in cohort A as follows:
Cohort A) approximately 6 evaluable patients at 40 mg in Cycle 1 without prophylactic steroids and escalating QW by up to 100 % of the previous dose until the DLT criteria for that dose level are met and
Cohort B) approximately 6 evaluable patients at 40 mg in Cycle 1 without prophylactic steroids and escalating QW by up to 150% of the previous dose up to 200 mg and then QW by up to 100% until the DLT criteria for that dose level are met.

Additional patients will be enrolled following a step up dosing schedule where RO6958688 will be administered QW for the first 3 administrations then followed by Q3W administration (Cycle 1 with RO6958688 at 40 mg [QW], Cycle 2 with RO6958688 at 150 mg [QW], Cycle 3 with RO6958688 at 300 mg [QW], then from Cycle 4 onwards with RO6958688 at 600 mg [Q3W]). Once the late cycle MTD has been defined (see Protocol Section 6.7.1.2) or 1200 mg is declared safe in Cohorts A and B (i.e. at least 6 patients reached the 1200 mg dose level and less than 1/3 of Cohorts A and B patients experienced a DLT at 1200 mg or below), any participating patient in the study can be intra-patient dosed escalated up to a dose limited by the defined RO6958688 late cycle MTD or 1200 mg of RO6958688 if the MTD is not reached, after discussion with the medical monitor. Any participating patient in the study who shows loss of RO6958688 exposure (C_{max} reduction of > 50% and a PK profile consistent with ADA mediated decrease in exposure) can be dose escalated up to a dose limited by the defined RO6958688 late cycle MTD or 1200 mg of RO6958688 if the MTD is not reached. In this case the patient should receive an intermediate RO6958688 dose of 900 mg. Up to five different cohorts (with/without obinutuzumab) will enroll approximately 30 CRC patients (Cohort C), approximately 30 gastric cancer patients (Cohort D), approximately 30 pancreatic cancer patients (Cohort E), approximately 30 breast cancer patients (Cohort F) and approximately 30 non-small cell lung cancer patients (Cohort G). Regarding non-small cell lung cancer patients, an alternative RO6958688 dose/schedule can be explored if supported by data (i.e. 40 mg at C1D1 [QW] followed by 100 mg at C2D1 [QW], 150 mg at C3D1 [QW], 300 mg at C4D1 [Q3W] and 600 mg [Q3W], from C4D1 onwards the cycle length is 3 weeks). Cohorts D, E, F and G will open once the dose regimen has been defined. Note: Cohort C has enrolled 24 CRC patients; no further patients will be enrolled. Cohort D through G did not and will not enroll any patients.

Baseline and on-treatment tumor biopsies are mandatory for all patients (see Section 3.3.2.2). Assessment of CT scan (or MRI) and FDG-PET will be performed locally. This will be undertaken in order to define the recommended schedule and dose

"Use of this document is governed by the terms of use on the first page of this document."

to be used for further development that optimally balances efficacy, safety, and pharmacodynamics of the two mentioned schedules.

In parallel to the enrollment of Cohorts C to H, up to 20 additional patients (all solid tumors following the Cohort's C dose/schedule) will be enrolled in a biomarker cohort based on their CEA expression level (Moderate/Low CEA expression, Very Low/negative CEA expression) in order to explore the correlation between CEA expression, efficacy and safety. Note: The biomarker cohort did not and will not enroll any patients.

Based on the preliminary safety and efficacy data, the Sponsor could enroll patients with other CEA expressing tumor indications.

3.1.2 Dose Escalation Decision Criteria

During the dose escalation part, the decision to escalate to the higher dose levels that have been cleared for dose escalation will be made by the Sponsor and the participating investigators after the review of all collected relevant safety information (adverse events and ECG results, vital signs, and clinical laboratory test results) is completed. For Part II of the study, available PK data will be considered.

3.1.2.1 Escalation Criteria (Part I)

In Part I of the study, the RO6958688 dose will start at 0.05 mg and will not be escalated higher than 2.5 mg (see Section 3.1.1). The dose (any doses lower than 2.5 mg) will not be escalated further if a patient experiences a study drug–related adverse event of Grade 3 or higher during the 14-day safety observation period. If a Grade 3 or higher RO6958688-related event is reported, Part I of the study will be ended, and the dose level below the dose at which the adverse event has occurred will be the starting dose of Part II.

3.1.2.2 Escalation Criteria (Part II)—Dose Limiting Toxicities Applicable for Part II

During dose escalation in Part II, patients who withdraw before the end of the DLT period (i.e., within 21 days of first dose of RO6958688 for the QW regimen or 7 days for the step-up dosing regimen) for reasons other than DLTs and patients who do not receive at least three completed infusions of RO6958688 QW will be replaced to ensure that for the QW regimen at least 3 patients have been assessed for the full DLT period of 21 days prior to moving to the next dose level. Patients who discontinue obinutuzumab treatment before evaluation of response or obtainment of the paired tumor biopsy will be replaced.

"Use of this document is governed by the terms of use on the first page of this document."

For the purpose of this study, a DLT will be defined as any of the following events attributed to RO6958688 (i.e., related to RO6958688) and occurring during the DLT period:

- Hematological toxicities defined as:
    - Grade 4 neutropenia (i.e., ANC $<0.5 \times 10^9$ cells/L for a minimal duration of 7 days)
    - Grade 3 and 4 febrile neutropenia
    - Grade 4 thrombocytopenia lasting $>$48 hours
    - Grade 3 thrombocytopenia associated with bleeding episodes
- Grade $\geq$3 non-hematological toxicity with the following exceptions:
    - Alopecia (any grade)
    - Grade 3 nausea or vomiting that resolves to Grade $\leq$2 with or without supportive therapy within 1 week
    - Grade 3 hypophosphatemia and transient Grade 3 hyperbilirubinemia resolved in $<$1 week
    - Grade $\geq$3 fatigue that resolves to Grade $\leq$2 within 1 week
    - IRRs are not considered to be DLTs because, on the basis of experience with MAbs, IRRs are not dose-related events. Precautions, including premedication, will be taken if IRRs of Grade $\geq$2 occur. If described precautions are not sufficient, other options will be discussed between the Sponsor and the investigator.
    - Fever $>$40°C (i.e., Grade 3) that occurs within 48 hours of RO6958688 infusion and resolves within 48 hours to $>$39°C–40°C (Grade $\leq$2) and fully resolves within 1 week
    - Grade 3 arthralgia that can be adequately managed with supportive care or that resolves to Grade $\leq$2 within 1 week
    - Grade 3 diarrhea, colitis, enteritis, that resolves to Grade $\leq$2 within 7 days with no fever or dehydration
    - Laboratory values of Grade $\geq$3 that are judged not clinically significant by the investigator
    - Grade 3 tumor pain that starts within 24 hours of infusion and resolves to Grade $\leq$ 2 within 1 week
    - Grade 3 hypoxia that starts within 24 hours of infusion and resolves to Grade $\leq$ 2 within 1 week
    - In patients with lung lesions, Grade 3 transient dyspnea secondary to localized lung edema that starts within 24 hours of infusion and recovers to Grade $\leq$ 2 or baseline within 1 week, and transient bronchospasm that resolves within 24 hours

"Use of this document is governed by the terms of use on the first page of this document."

- In patients with liver lesions, Grade 3 transient increase of bilirubin, transaminases and/ or Gamma GT that starts within 24 hours of infusion and recovers to Grade 1 or baseline within 1 week and Grade 4 AST/ALT or Grade 4 bilirubin increase that start after infusion and recovers to Grade ≤2 or baseline within 3 days.

Failure to recover from any RO6958688-related toxicity that results in a dose delay of > 14 days (any regimen) is defined as a DLT.

3.1.3 Communication Strategy

Upon completion of all screening evaluations and confirmation that a patient has met all of the inclusion and none of the exclusion criteria, investigator sites will contact the IxRS to confirm the patient number and contact the sponsor to confirm cohort assignment (i.e., RO6958688 dose with or without obinutuzumab). Confirmation will be received from Roche via completion of a Confirmation of Enrollment form (see Section 4.2.1). This will guarantee that the Sponsor is notified prior to the administration of RO6958688 or obinutuzumab to any patient.

In Part I, enrollment of a single patient at particular dose level will have to be immediately communicated to all sites in order to stop recruitment at that dose level.

During the dose-escalation of Part I and Part II of this study, approximately twenty-six study sites will be involved for the enrollment of patients. This number of sites will allow the Sponsor and the sites to have frequent and detailed discussions regarding patient eligibility and patient care. After each patient receives RO6958688/obinutuzumab, the investigator(s) must confirm to the Sponsor within 24 hours after dosing that the patient has received the dose and provide a brief summary of the status of the patient in terms of the safety and tolerability of RO6958688/obinutuzumab, which will be communicated by email and/or telephone.

In the event of a DLT during the 14-day safety observation period of Part I, the investigator will contact the Sponsor immediately to discuss patient status and action(s) taken/to be taken.

The investigator will contact the Sponsor immediately to discuss patient status and action(s) taken/to be taken in the event of a DLT during Part II of the study. In addition, as outlined above, after each patient cohort (minimum of 3 patients) has been completed (i.e., last patient in the cohort has reached Day 21), the Sponsor will organize a teleconference with the investigators to discuss the safety and tolerability of RO6958688 with/without obinutuzumab pretreatment and to discuss the dose for the next cohort. Dose decisions will be separate for the dose escalation with and without obinutuzumab pretreatment, and the corresponding teleconferences might be jointly held if timing allows. The next dose levels will be recommended using the EWOC designs during the dose-escalation phase and discussed by the Sponsor and investigators. In addition, the

"Use of this document is governed by the terms of use on the first page of this document."

clinical judgment of the Sponsor and investigators will also be utilized in the dose-selection process. This may lead to dose selections that differ from the mCRM recommendations if the scientific and clinical opinion is that a different dose would be more appropriate for patients. During these teleconferences, toxicities according to National Cancer Institute Common Terminology Criteria for Adverse Events (NCI CTCAE) v4.03 will be discussed along with the results of the available PK data in addition to safety laboratory results and any other available data that may assist the dose-escalation decision process. CRS severity will be graded according to NCI CTCAE v5 (Table 9). Dose escalation will only proceed to the higher dose level if the investigators and the Sponsor are satisfied with the safety profile of the previous patient cohort (i.e., no DLTs have been observed) and agree on the dose escalation. The discussion will be documented in writing by the Sponsor.

In addition to these communications, the Sponsor and investigators will be in regular contact throughout the study by email/telephone/fax as normal interactions during the conduct of a clinical study.

The Sponsor will be available 24 hours a day to discuss any medical or study-related issues that may arise during the conduct of this study.

3.1.4 End of Study

The study will formally end once the last patient has completed the safety follow-up visit or withdrawn from the study prior to that time (whichever occurs last), but may be prematurely terminated by the Sponsor.

3.2 RATIONALE FOR STUDY DESIGN

3.2.1 Rationale for RO6958688 Dosage Selection

The starting dose (RO6958688) in Part I of the study (0.736 μg/kg [0.05 mg flat dose]) corresponds to the MABEL dose obtained by in vitro assessment (tumor cell lysis). This starting dose has an estimated RO (eRO) of 0.11% on the CD3 receptor (CD3 eRO) and 30% on the CEA receptor (CEA eRO). In the NOD/Shi-scid/IL-2/Rynull (NOG) mouse model, the in vivo efficacy was achieved at 403 μg/kg (28 mg flat dose), which has approximately 50% CD3 eRO and 100% CEA eRO. A cytokine release assay showed a high risk of cytokine-mediated events upon first dosing at 100 nM (equivalent flat dose of 57.8 mg) with potential risk at 10 nM (equivalent flat dose of 5.7 mg).

Taking the available data together and in order to minimize the number of patients who receive doses below the relevant biological activity, which appears to be in correlation with the CD3 eRO, the Sponsor has defined the following intermediate and top flat doses in Part I:

- Second cohort: 0.15 mg with approximately 0.3% CD3 eRO (57% CEA eRO)
- Third cohort: 0.45 mg with approximately 1% CD3 eRO (79% CEA eRO)
- Fourth cohort: 1.3 mg with approximately 2.7% CD3 eRO (92% CEA eRO)

"Use of this document is governed by the terms of use on the first page of this document."

- Fifth and last cohort: 2.5 mg with approximately 5% CD3 eRO (96% CEA eRO)

If there is no safety concern reported in Part I during the 14-day safety observation period (stopping rule: any RO6958688-related event of Grade 3 or higher toxicity reported during the observation period), Part II of the study will start with a dose of 2.5 mg with approximately 5% CD3 eRO (96% CEA eRO). Dose-escalation for Part II will follow the EWOC design (see Section 3.2.6).

3.2.2 Rationale for obinutuzumab dose

The dose of obinutuzumab administered in the study will be 2000 mg given either in a single infusion or in two administrations of 1000 mg over two consecutive days. The safety and efficacy of monotherapy with obinutuzumab comparing 1000 mg and 2000 mg dose was established in GAO4768-GAGE study. The data from the study shows that no clinically meaningful differences have been found between 1000 mg and 2000 mg doses from safety perspective. A higher proportion of B-CLL patients (86.8%) had B cell depletion in 2000 mg arm as compared to those in 1000 mg arm (72.5%), respectively.

Obinutuzumab has been shown to be effective in minimizing ADA development when given 12 /13 or 7/8 days before for CEA-IL2v (RO6895882) infusion and when administered 12/13 days before RO6958688 (see 1.3.2.2 for more details).

For safety on the BP28920/obinutuzumab study, see Section 1.3.2.2.

Based on these preliminary results, we consider 2000 mg of obinutuzumab pretreatment administered 7 days prior to receiving RO6958688 should be a correct dose to reduce or delay the ADA formation directed against RO6958688.

For further details please consult the current version of obinutuzumab Investigator Brochure.

3.2.3 Rationale for the Treatment of Severe Cytokine Release Syndrome (CRS) Using Tocilizumab

CRS is a potentially life-threatening symptom complex, caused by the excessive release of cytokines by immune effector or target cells during an exaggerated and sustained immune response. CRS can be triggered by a variety of factors, including infection with virulent pathogens, or by medications that activate or enhance the immune response, resulting in a pronounced and uncontrolled immune response.

Regardless of the inciting agent, severe or life-threatening CRS is a medical emergency. If unsuccessfully managed, it can result in significant disability or fatal outcome. Current clinical management focuses on treating the individual signs and symptoms, providing supportive care, and attempting to dampen down the inflammatory response using high dose corticosteroids. However, this approach is not always successful, especially in the case of late intervention.

“Use of this document is governed by the terms of use on the first page of this document.”

CRS is associated with elevations in a wide array of cytokines, including marked elevations in IFN-γ, IL-6, and TNF-α levels. Emerging evidence implicates IL-6 as a central mediator in CRS. IL-6 is a pro-inflammatory multi-functional cytokine produced by a variety of cell types, which has been shown to be involved in a diverse array of physiological processes, including T cell activation. Regardless of the inciting agent, CRS is associated with high IL-6 levels (Panelli et al 2004, Lee et al 2014, Doessegger and Banholzer 2015), and IL-6 correlates with the severity of CRS with patients who experience severe or life-threatening CRS (CTCAE Grades 4 or 5) having much higher IL-6 levels compared to their counterparts who do not experience CRS or experience milder CRS reactions (CTCAE Grades 0−3) (Chen et al 2016). Based upon this evidence, IL-6 levels will be measured at select timepoints in the study, per the SoA, via central laboratory testing (see Appendix 1 and Appendix 10).

As mentioned in section 1.4, as part of the data review of BP29541 and WP29945, we observed a correlation between peaks of serum IL-6 and the occurrence of IRRs and associated symptoms of hypotension or hypoxia or dyspnea within 24 hours of RO6958688 infusion. However, the majority of these peaks are less than 1000 pg/mL. Given that hypotension and hypoxia are key symptoms of CRS, based on the current information, CRS may have occurred in some patients experiencing IRR with RO6958688 and underlying symptoms of hypotension and hypoxia. A patient with Grade 5 IRR, described in Section 5.2.5.1.5, had documented very high cytokine levels including IL6 at one of the previous RO6958688. Also, 2 other patients described in section 5.2.5.1.5, have been successfully treated with Tocilizumab, further supporting the use of Tocilizumab.

Tocilizumab (Actemra®/RoActemra®) is a recombinant, humanized, anti-human monoclonal antibody directed against soluble and membrane-bound IL-6R, which inhibits IL-6 mediated signaling. Blocking the inflammatory action of IL-6 using tocilizumab could therefore represent a novel approach for the treatment of CRS. Refer to the Tocilizumab Investigator's Brochure for additional preclinical and clinical information regarding tocilizumab.

CRS has classically been associated with therapeutic mAb infusions, most notably anti-CD3 (OKT3), anti-CD52 (alemtuzumab), anti-CD20 (rituximab) and the CD28 super-agonist TGN1412 (Lee et. al 2014). CRS is also observed with T-cell recruiting therapies, including chimeric antigen receptor (CAR)-T cell therapy and bispecific molecules, such as blinatumomab (Nagorsen et al 2012). Tocilizumab has been recently approved by the FDA (August 2017) and received a positive CHMP opinion (European Medicines Agency 2018) for a restricted use, limited to Cytokine Release Syndrome (CRS) induced by chimeric antigen receptor (CAR) T cell in adults and pediatric patients 2 years of age and older (Tocilizumab USPI).

Taken together, these findings indicate that patients treated with RO6958688 who develop severe CRS may benefit from tocilizumab therapy. Section 5.2.5.1.5 describes

"Use of this document is governed by the terms of use on the first page of this document."

recommendations regarding the management of CRS considered related to RO6958688.

The dose *of 8 mg/kg (suitable for patients at or above 30 kg weight)* recommended in this protocol to treat severe CRS is the one approved by the FDA for the management of (CAR)-T cells related CRS (refer to the Tocilizumab IB for details).

3.2.4 Rationale for Study Population

This study will enroll patients with locally advanced and/or metastatic CEA–positive solid tumors who have progressed on standard treatment, are intolerant to SOC, and/or are non-amenable to SOC.

3.2.5 Rationale for Biomarker Assessments

RO6958688 was designed to target tumors that express CEA and to concomitantly engage immune cells to lead to their activation and proliferation, which culminates in tumor lysis. Nonclinical studies have confirmed the expected MoA of RO6958688 by mediating tumor killing, induction of T-cell activation markers (CD25 and CD69), cytokine release (IFNγ, TNFα, granzyme B, IL-2, IL-6, and IL-10), and proliferation of T cells. Furthermore, the MoA of RO6958688 occurs only if simultaneous binding (cross-linking) of T cells to CEA-expressing tumor cells takes place. Therefore, exploratory biomarker samples (both Part I and Part II) and samples for pharmacodynamics (Part II) will be collected in the study in order to assess immune cell alterations both in the peripheral blood and tumor tissue and soluble CEA in the blood. In particular, archival tumor tissue is to be obtained from all patients, if available, in order to perform CEA assessment for patient eligibility. If no archival tissue is available, CEA assessment will be performed with the use of the fresh baseline tumor biopsy. Archival tumor tissue, if available, will be obtained from all patients enrolled in Part II to further assess exploratory biomarkers related to the PD effects of treatment, including but not limited to the expression of tumour PD-L1, etc.

Tumor biopsy samples will only be collected from patients enrolled in Part II of the study. Tumor biopsies will be performed on two occasions, (once during pre-treatment and once during treatment with RO6958688), and on each occasion, two biopsy samples will be collected for patients who will be treated at dose levels equal and greater than 5 mg ($\geq$ 5 mg). These tumor biopsies for PD analyses are mandatory at baseline and on-treatment at either at C2D1, C3D1, C4D1 or C7D1 (-48/0 hours) (see Section 3.3.2.2 and Table 2. If preliminary data suggest that modification of the on-treatment tumor biopsy timepoint would be more appropriate, alternative on-treatment tumor biopsy timepoints could be considered in the future cohorts. All biopsies must be taken after FDG-PET. The biopsies will be taken from accessible, “non-critical” tumor locations, including, but not limited to, skin, lymph node, rectum, liver, etc (bone marrow biopsy is not accepted).

“Use of this document is governed by the terms of use on the first page of this document.”

In the QWx3/Q3W schedule and for each cohort, the biopsy time points will be undertaken as follows (on-treatment biopsies will be undertaken (-48/0 hours)):

- half of the patients will undertake Baseline and C4D1 pre-dose biopsies
- half of the patients will undertake Baseline and C5D1 pre-dose biopsies

Patients being enrolled in cohorts A and B will undertake Baseline and C7D1 pre-dose biopsies.

For patients enrolled in obinutuzumab pre-treatment cohorts, biopsies will be taken at Baseline and C2D1 for Q3W schedule, at Baseline and C4D1 for QW schedule and at Baseline and C4D1 predose for QWx3/Q3W.

Table 2 Biopsy time points per cohort

**Cohorts**	**Biopsy time points**
Cohorts A and B QW	Baseline and Cycle 7 Day 1 predose (- 48/0 hours)
Obinutuzumab pre-treatment QW	Baseline and Cycle 4 Day 1 predose (- 48/0 hours)
Obinutuzumab pre-treatment Q3W	Baseline and Cycle 2 Day 1 predose (- 48/0 hours)
Obinutuzumab pre-treatment QWx3/Q3W (Cohort H)	Baseline and Cycle 4 Day 1 predose (- 48/0 hours)
Cohorts C, D, E, F, G QWx3/Q3W)	half of the patients at Baseline and Cycle 4 Day 1 predose (- 48/0 hours) and the other half of the patients at Baseline and Cycle 5 Day 1 predose (- 48/0 hours)

For patients continuing in the study, additional biopsies may be taken at the discretion of the investigator to evaluate viable tumor given the mechanism of action of RO6958688 to induce tumor inflammation, and a portion of this biopsy should be sent to Roche (or their designated laboratory) for analysis. An optional biopsy at the time of progression can be undertaken in order to provide information regarding potential follow up treatment and escaping mechanism of current treatment.

It is expected that upon treatment, the number and activation/differentiation status of intratumoral immune cells as well as their location will change. Such changes will be determined by flow cytometric and/or immunohistochemical (IHC) methods and will be analyzed centrally. Changes such as the density of different immune cell lineages (including but not limited to CD4+ T cells, CD8+ T cells, B cells, NK cells, and macrophages) and their activation and differentiation status (including but not limited to CD25, Ki67, PD1, and TIM3) will be examined. The expression of tumor markers (including but not limited to PD-L1) will also be examined. Tumor biopsies will also be

"Use of this document is governed by the terms of use on the first page of this document."

used for the assessment of other biomarkers, including but not limited to mutational signatures and tumor mutational load.

If available, an assessment of tumor growth kinetics will be made by comparing post-treatment scans with the last available prestudy scan.

Blood samples will be collected and analyzed with respect to alterations in the number and activation and differentiation of immune cells as a consequence to treatment with RO6958688. The samples will be analyzed by flow cytometry, and the number of cells that belongs to lymphocyte subsets (CD4+ T cells, CD8+ T cells, NK cells, B cells, and monocytes) and their activation and differentiation status will be determined (including but not limited to Ki67, PD1, and TIM3).

Administration of therapeutic antibodies is frequently associated with IRRs and cytokine release. RO6958688 is expected to bind to T cells, which may lead to the release of cytokines as a response to immune cell activation. Therefore, cytokines, including but not limited to IL-6, IFNγ, TNFα, and inflammation markers, will be assessed in serum or plasma samples. Because these measurements also represent safety measures, they will be examined in patients enrolled in both Part I and Part II.

Additional immune PD biomarkers that are related to the MoA of RO6958688 may also be analyzed from serum or plasma samples taken from all patients. Such PD measures will be considered as disease-monitoring markers and include but are not limited to sCEA.

The specimens will be used for research purposes to identify biomarkers that are useful to predict and monitor response to RO6958688 treatment and safety, assess PD effects of RO6958688 treatment, and investigate the mechanism of resistance to therapy.

3.2.6 Rationale for Statistical Design

The study design for Part I follows a traditional single-patient cohorts dose escalation design and targets a range of doses which have been deemed nonclinically to be safe and to be well below the dose at which relevant biological effects occur.

For Part II, the EWOC design uses a statistical model that actively seeks a dose level close to the MTD with the use of toxicity data from all enrolled evaluable patients to compute a precise dose-toxicity curve. It locates the MTD efficiently and minimizes the number of patients treated at possibly pharmacologically inactive dose levels. Such model-based designs have been successfully applied in many Phase I dose-escalation studies (Bailey et al. 2009; Le Tourneau et al. 2009). The operating characteristics (see Table 12) from extensive simulations intend to show that the EWOC design provides more accurate estimates of the true probability of toxicity and an estimate of the MTD which mostly lies within the true targeted interval without exposing patients to undue risk of toxicity. The EWOC design is known to have better chances than the

"Use of this document is governed by the terms of use on the first page of this document."

“3 + 3” design to select an MTD within the target toxicity interval while keeping both the chances of under- and overdosing at a desired minimum. The EWOC design’s MTD estimates only lie below the target toxicity interval for scenarios which have very low toxicity. The more classical “3 + 3” design is known to have a general tendency to underdose (Kang and Ahn 2001; Kang and Ahn 2002) and does not typically achieve the target of the dose interval of approximately 33% toxicity rate. Furthermore, the EWOC design provides more information on the whole observed toxicity curve. It can be run incrementally at any timepoint for decision making and, ultimately, it accounts for all the data accrued until that point (not only the last 3 patients as expected in the “3 + 3” design). In the current design, an extension of the EWOC allows for continuous doses over a prespecified range. Stopping criteria are made explicit, and different dose increments that depend on the dose interval can be prespecified to allow for an optimal tailoring of the tested experimental therapy’s study design. All doses and restrictions are based on nonclinical efficacy and safety and prediction of the pharmacokinetics of RO6958688 in humans.

Cohorts C, D, E, F, G and H are designed to estimate the ORR per cancer type under the step-up QWx3/Q3W schedule. The sample size of up to 30 patients for each of the cohorts C, D, E, F and G has been selected in order to obtain sufficiently precise estimates of ORR. Specifically, if no response is observed in 30 patients (i.e. 0% estimated ORR), the 95% confidence interval (CI) for ORR would be 0 to 12%; on the other hand if for example 3 responses are observed (i.e. 10% estimated ORR), the 95% CI would be 2 to 27%. For cohort H, the sample size of approximately 40 patients allows a cross-study comparison with a corresponding cohort with obinutuzumab pretreatment in study WP29945 of the same sample size.

The sample size of approximately 15 patients in the Q3W cohort with obinutuzumab pretreatment allows an initial exploration.

3.2.7 Rationale for characterization of Anti-Drug Antibodies directed against RO6958688 on a B cell/antibody level in individual patients

ADA responses are diverse and specific. A deeper understanding of the ADA formation to RO6958688 and its characteristics at the clonal diversity level, such as specificity and affinity, will help improve our understanding of any clinically relevant biological activity and examining monoclonal B-cell specificities will provide deeper insights on ADA specificity. Here, CellSpot™ technology, an automated microscopy technology, will be utilized to examine the secreted IgG footprints from single B-cells. The whole blood samples will be shipped within 24 hours to central laboratory where Peripheral Blood Mononuclear Cell (PBMCs) will be isolated according to specific protocol. Samples will be collected at Cycle 1 Day 1 predose, at Cycle 7 Day 1 predose and at Cycle 12 Day 1 predose from patients receiving RO6958688 QW and at Cycle 1 Day 1 predose, at Cycle 5 Day 1 predose and at Cycle 6 Day 1 predose from patients receiving RO6958688 QWx3/Q3W but not from patients Q3W schedule or with obinutuzumab

“Use of this document is governed by the terms of use on the first page of this document.”

pretreatment. Antibody secreting B-cells will be isolated, and the corresponding monoclonal antibodies will be characterized (see Section 4.5.1.6.2).

3.3 OUTCOME MEASURES

3.3.1 Safety Outcome Measures

The safety outcome measures for this study are:

- Incidence of DLTs
- Incidence and severity of adverse events and IRRs and CRS symptoms
- Incidence of laboratory abnormalities (as examples and not limited to: hematology testing, coagulation, serum chemistries, and urinalysis)
- Incidence of anti-drug antibodies (ADAs) formation and detection of cytokine release
- Physical examination findings
- Triplicate 12-lead ECGs
- Vital signs

The NCI CTCAE v4.03 (NCI CTCAE v.4 data files) will be used to evaluate the clinical safety of the treatment in this study. Patients will be assessed for adverse events at each clinical visit and as necessary throughout the study. CRS severity will be graded according to NCI CTCAE v5 (Table 9).

Further details can be found in the Schedules of Assessment tables (see Appendix 1).

3.3.2 Pharmacokinetic and Pharmacodynamic Outcome Measures

3.3.2.1 Pharmacokinetic Outcome Measures

RO6958688 and obinutuzumab serum samples will be collected for PK analyses following single and multiple doses. The PK outcome measures for this study for RO6958688/obinutuzumab include (when appropriate):

- C_{max}
- Area under the curve (AUC)
- Half-life ($t_{1/2}$)
- Minimum drug concentration (trough concentration, C_{min})
- Clearance (CL)
- Volume of Distribution at Steady State (V_{ss}).

In addition, data will be analyzed using population PK modeling; results will be reported in a document outside the Clinical Study Report.

Obinutuzumab serum concentration data (C_{min} and C_{max}) will be tabulated and summarized. Descriptive statistics will include mean, median, range, and standard

"Use of this document is governed by the terms of use on the first page of this document."

deviation, as appropriate. Provided the data permit, population pharmacokinetic method will be applied for analyzing the PK outcome measures of obinutuzumab.

3.3.2.2 Pharmacodynamic Outcome Measures

The PD outcome measures for this study are:

- Whole blood samples: Peripheral blood immune cells will be assessed with respect to the changes in the characteristics of lineage (CD4+ T cells, CD8+ T cells, NK cells, monocytes, T-regulatory cells, and B cells), activation and differentiation (including but not limited to Ki67, PD1, TIM3, ICOS, etc.).
- Serum or plasma samples: PD biomarkers such as cytokines and inflammation markers (including but not limited to TNFα, INFγ, IL-6, MIP, etc.) will be analyzed. Because these measurements are also safety measure assessments during any IRRs, they will also be examined in patients enrolled in both Part I and Part II of the study. Disease-monitoring markers that include but are not limited to sCEA will also be assessed.
- Soluble CEA: will be measure as a disease-monitoring marker. A mandatory blood sample will be taken for soluble CEA central assessment.
- Whole blood samples: From Part II patients, at Cycle 1 Day 1 predose (baseline) in the QW schedule, at Cycle 1 Day 1 predose (Baseline) and Cycle 3 Day 1 predose in the Q3W schedule, and at Cycle 1 Day 1 predose (baseline) in the QWx3/Q3W schedule, mandatory 2 whole blood samples will be taken for TCR Vβ sequencing (the CDR3-TCR beta chain repertoire). The DNA will be used to determine in peripheral T cells the repertoires of T cell receptor (TCR) Vβ CDR3 and analyze TCR diversity.
- Tumor biopsy: Tumor biopsy samples obtained in Part II (for detailed timepoints, see Section 4.5.1.6.2) will be assessed centrally for changes in immune cell numbers and activation characteristics as well as changes in tumor markers such as PD-L1. These analyses will be performed by flow cytometric molecular and/or IHC methods with respect to changes in the characteristics of lineage (CD4+ T cells, CD8+ T cells, NK cells, monocytes, T-regulatory cells, and B cells), activation (including but not limited to CD25, CD69, etc.), differentiation (including but not limited to Ki67, PD1, TIM3, ICOS, etc.), and TCR Vb repertoire.
- Original or archival tumor: Potential prognostic biomarkers such as CEA expression will be confirmed on archival tumor, if available, or from the freshly obtained biopsy samples. In Part II, these measurements will assess the CEA change over the course of the disease and the stability of the measurements. Further other exploratory biomarker assessments, including but not limited to analysis of microsatellite instability, mutational signatures and genomic mutations, may be carried out.
- Positron Emission Tomography: Drug-induced changes on tumor glycolysis and metabolic responses will be determined in Part II of the study. Assessment of FDG PET will be performed locally at the sites during the whole study.

“Use of this document is governed by the terms of use on the first page of this document.”

3.3.3 Efficacy Outcome Measures

The exploratory efficacy/activity outcome measures for this study are:

- ORR, defined as partial response (PR) or complete response (CR)
- Best overall response (BOR) defined as the best response recorded from the start of the study treatment until the end of treatment taking into account any requirement for confirmation.
- DOR, defined as the time interval from the first occurrence of a documented objective response to the time of disease progression
- The rate of patients with stable disease
- DCR, defined as PR+CR+SD
- PFS *according to Response Evaluation Criteria in Solid Tumors, Version 1.1 (RECIST v1.1)*

Tumor response will be evaluated according to RECIST v1.1 (see Appendix 4) and irRC using unidimensional measurement (see Appendix 5) such as computed tomography (CT) scan or magnetic resonance imaging (MRI). Assessment of CT/MRI scans as tumor assessments will be performed at the sites during the whole study. If Sponsor decides, independent central read for computed tomography (CT) or magnetic resonance imaging (MRI) might be performed in this study, both prospectively and retrospectively.

Tumor assessment will be performed once during the screening. The first assessment after the start of treatment will be performed at 8 weeks and continue every 8 weeks thereafter for the first year and every 12 weeks thereafter until disease progression (by investigator assessment) or treatment discontinuation. All tumor assessments after baseline may be done within ±7 days of the scheduled visit.

Additionally, FDG-PET based tumor assessment will be performed at baseline (Day-14 to Day-1) and at week 6 (C6D5 -72/0 hours) for the QW schedule, at week 6 (C2D19 -72/0 hours) for the Q3W schedule and at week 6 (C4D19 -72/0 hours) for the QWx3/Q3W schedule). Baseline FDG-PET and on-treatment FDG-PET must be performed before the baseline and the on-treatment biopsy if feasible.

Confirmation of partial and complete responses will be done at the next scheduled visit after at least 28 days from the initial response. A patient is assigned a best overall confirmed response SD if they have a response assessment of SD, PR, or CR at one or more visits at least 42 days (6 weeks) after start of study treatment, but are not a confirmed CR or PR.

PFS per RECIST v1.1 or irRC criteria is defined as time between *enrollment date* and date of first documented disease progression per RECIST v1.1 or irRC criteria, respectively, or death from any cause, whichever occurs first. Patients who neither

“Use of this document is governed by the terms of use on the first page of this document.”

progressed nor died in this interval, or who are lost to follow-up are censored at the date of last tumor assessment within this time window or last follow-up for progression of disease. Patients for whom no post-baseline tumor assessments are available are censored at Cycle 1 Day 1.

The following criteria are needed for continuing treatment beyond RECIST v1.1–defined progression that has been initially assessed by the investigator:

- Investigator–assessed clinical benefit and
- The patient is tolerating study drug and signs the appropriate Inform consent form.

Optional submission of the latest pre-study or historical CT scans is highly encouraged for assessment of tumor growth kinetics within 6 weeks of patient entering the study if available. This scan will be compared to those collected during the study to determine tumor growth kinetics.

3.3.4 Exploratory Outcome Measures

The exploratory outcome measures for this study include but are not limited to the following:

- Rate of patients with positive ADA titer against RO6958688 at week 8
- Overall survival
- Changes in various T-cell subpopulations in blood (e.g., effector/memory T cells, regulatory T cells, and other T-cell types)
- Identification and profiling of exploratory biomarkers in PBMCs (e.g., changes in expression of CD25 or human leukocyte antigen DR [HLA-DR], interferon [IFN]-γ production, and other markers)
- Changes in tumor-infiltrating, $CD8^+$ T cells (and other exploratory markers) in freshly obtained tumor tissue before and during treatment with RO6958688 and/or obinutuzumab
- Identification and profiling of exploratory biomarkers in plasma (i.e., interleukin [IL]-2, IFN-γ, and other markers)
- Changes in tumor-infiltrating T-cell activity (measured by expression of granzyme B and other markers) in freshly obtained tumor tissue prior to and during treatment with RO6958688 and/or obinutuzumab.
- In addition, gene expression and mutation analysis will also be carried out.
- The baseline values and kinetics of soluble markers of tumor markers (such as CEA) will be explored.
- The specificity of ADA in patients with no obinutuzumab pretreatment.

“Use of this document is governed by the terms of use on the first page of this document.”

4. MATERIALS AND METHODS

4.1 CENTER

This multicenter (approximately twenty-six centers) study is planned to be conducted in North America and in Europe. Additional sites (and countries) may be included for back-up purposes and may be activated if needed.

Administrative and contact information and list of investigators are provided separately.

4.2 STUDY POPULATION

Patients with locally advanced and/or metastatic CEA-positive solid tumors who have progressed on standard treatment, are intolerant to SOC, and /or are non-amenable to SOC will be enrolled into this study.

This study has enrolled 149 patients. For the purpose of this protocol, evaluable patients will be those with sufficient tumor tissue material to be collected during baseline and on-treatment biopsies to allow for the required PD studies. Patients who do not meet this requirement will be replaced. However, any patient who receives one or more doses of RO6958688 will be evaluable for safety assessments.

In Part I, 5 patients were enrolled.

In Part II, 144 patients were enrolled: At least 3 patients per QW cohort were enrolled, plus 22 patients for the step up dosing scheme dose escalation (Cohorts A and B). 24 patients were enrolled into Cohort C while no patients were enrolled into the combined QWx3/Q3W schedule cohorts (Cohorts D, E, F, G and H) and 17 patients were enrolled and received obinutuzumab pre-treatment (cohort with RO6958688 Q3W). The biomarker cohort did not and will not enroll any patients.

If supported by data (preclinical or clinical data), this protocol may consider to explore this treatment in solid tumours with lower CEA expression intensity level.

4.2.1 Recruitment and Enrollment Procedures

Patients will be identified for potential recruitment using prescreening enrollment logs, Independent Ethics Committee (IEC)- or Institutional Review Board (IRB)-approved newspaper/radio advertisements, and mailing lists (if applicable) prior to consenting to take part in this study.

Once a patient has been confirmed as meeting all of the inclusion and none of the exclusion criteria, the Sponsor will approve enrollment of this patient using a Confirmation of Enrollment form, which will be sent to the investigator.

For Part I of the study, the assigned dose will be documented in the Confirmation of Enrollment form and a patient number will be allocated. A Patient Enrollment and Identification Code List must be maintained by the investigator.

“Use of this document is governed by the terms of use on the first page of this document.”

For Part II of the study, the assigned dose will be documented in the Confirmation of Enrollment form and a confirmation on whether the patient should be pretreated with obinutuzumab. The screening numbers will be allocated sequentially by site in the order in which the patients are screened and once eligibility is confirmed the IxRS will allocate a patient number. A Patient Enrollment and Identification Code List must be maintained by the investigator.

For the combined QW/Q3W step-up Cohorts, the first three patients of the CRC cohort will be observed for safety for 1 week each (Patient 1 will be the first patient dosed and there will then be a 1 week gap before Patient 2 is dosed. Following dosing of Patient 2 there will be a further week before Patient 3 is dosed. A further week will be observed before all remaining patients in the cohort can be treated). Cohorts D, E, F and G may open (with/without obinutuzumab) based on Cohort C preliminary results. The patients to be enrolled into the gastric and the pancreatic patients' cohorts will start their enrollment at least after the first three CRC patients have started their treatment. The assigned dose, schedule and biopsy time point will be documented in the Confirmation of Enrollment form. A Patient Enrollment and Identification Code List must be maintained by the investigator. Note: Cohorts D through G did not and will not enroll any patients.

4.2.2 Inclusion Criteria

Patients must meet the following criteria for study entry:

1. Signed informed consent
2. Age ≥ 18 years
3. For dose escalation locally advanced and/or metastatic gastrointestinal (GI) solid tumor in patients who have progressed on a standard therapy, are intolerant to SOC, and/or are non-amenable to SOC and other solid tumors expressing CEA as per inclusion criterion 13
4. Radiologically measurable disease according to RECIST v1.1
5. Life expectancy (in the opinion of the investigator) of ≥ 12 weeks and LDH ≤ 2,5 x ULN
6. Eastern Cooperative Oncology Group (ECOG) Performance Status (PS) 0–1
7. All acute toxic effects of any prior radiotherapy, chemotherapy, or surgical procedure must have resolved to Grade ≤ 1 or returned to baseline except alopecia (any grade) and Grade 2 peripheral neuropathy
8. Adequate hematological function (without transfusion within 2 weeks prior to Cycle 1, Day 1): neutrophil count of $\geq 1.5 \times 10^9$ cells/L, platelet count of $\geq 100{,}000/\mu L$, and hemoglobin ≥ 8 g/dL (4.9 mmol/L) including lymphocytes within normal limits ($\geq 0.8 \times 10^9$ cells/L)
9. Adequate liver function: total bilirubin ≤ 1.5 × the upper limit of normal (ULN; excluding Gilbert's Syndrome as defined in Appendix 8), AST and/or ALT ≤ 2.5 × ULN (in case of liver metastases, ≤ 5 × ULN)

"Use of this document is governed by the terms of use on the first page of this document."

10. Adequate renal function: Creatinine clearance ≥ 60 ml/min calculated by CKD-EPI equation (see Appendix 2)
11. Negative serum pregnancy test within 7 days prior to study treatment in premenopausal women and women ≤ 2 years after start of menopause (menopause is defined as amenorrhea for > 2 years)
12. Patients must agree to either remain completely abstinent or to use two effective contraceptive methods from screening until 2 months if the patient is a male or 4 months if the patient is a female after the last dose of RO6958688 if the patient received only RO6958688, or from screening until 3 months if the patient is a male or 18 months if the patient is a female after the last dose of obinutuzumab and 3 months after the last dose of tocilizumab (if applicable) as follows:
    a) All males, including male partners of female patients, must agree to either remain completely abstinent or to use a condom during the entire study period with RO6958688 and for 3 months after the last administration of RO6958688 or obinutuzumab and for 2 months after the last dose of tocilizumab (if applicable). Men must refrain from donating sperm during this same period.
    b) All females of childbearing potential and female partners of male patients, must agree to remain abstinent or use combined contraceptive methods that result in a failure rate of < 1% per year during the treatment period and at least through 4 months after last dose of RO6958688 or 18 months after the last dose of obinutuzumab if the patient has received obinutuzumab pretreatment and 3 months after the last dose of tocilizumab (if applicable)
        a. Abstinence is only acceptable if it is in line with the preferred and usual lifestyle of the patient. Periodic abstinence (e.g., calendar, ovulation, symptothermal, or postovulation methods) and withdrawal are not acceptable methods of contraception;
        b. Examples of contraceptive methods with an expected failure rate of < 1% per year include male sterilization, hormonal implants, proper use of combined oral or injected hormonal contraceptives, and certain intrauterine devices. Alternatively, two methods (e.g., two barrier methods such as a condom and a cervical cap) may be combined to achieve a failure rate of < 1% per year; barrier methods must always be supplemented with the use of a spermicide
13. Non-gastrointestinal solid tumors (like NSCLC or breast cancer patients) should have confirmed (centrally for North America) CEA expression in tumor tissue ≥ 20% of tumor cells staining with at least moderate to high intensity of CEA expression (IHC2+ and IHC 3+). For CRC patients only, the CEA assessment should be performed but the result is not required to enroll the patient.
14. For the biomarker cohort, only patients with moderate/low CEA expression (< 20% of tumor cells with IHC2+/3+ and/or ≥ 20% of tumor cells with IHC1+) and very low/negative CEA expression (< 20% of tumor cells with IHC1+) or IHC0+ will be enrolled. CEA expression should be determined prior to enrollment (centrally for North America), if no archival tumor tissue is available, a fresh biopsy will be collected.

"Use of this document is governed by the terms of use on the first page of this document."

4.2.3 Exclusion Criteria

Patients who meet any of the following criteria will be excluded from study entry:

1. Active or untreated central nervous system (CNS) metastases as determined by CT or MRI evaluation during screening and prior radiographic assessments

   Patients with a history of treated asymptomatic CNS metastases are eligible, provided they meet all of the following criteria:

   - No metastases to brain stem, midbrain, pons, medulla, cerebellum, or within 10 mm of the optic apparatus (optic nerves and chiasm)
   - Radiographic demonstration of improvement upon the completion of CNS-directed therapy and no evidence of interim progression between the completion of CNS-directed therapy and the screening radiographic study
   - No history of intracranial hemorrhage or spinal cord hemorrhage
   - No ongoing requirement for dexamethasone as therapy for CNS disease; anticonvulsants at a stable dose allowed
   - No stereotactic radiation or whole-brain radiation within 28 days prior to Cycle 1 Day 1
   - Screening CNS radiographic study ≥ 4 weeks since completion of radiotherapy and ≥ 2 weeks since discontinuation of corticosteroids

2. Spinal cord compression not definitively treated with surgery and/or radiation or previously diagnosed and treated spinal cord compression without evidence that disease has been clinically stable for ≥ 2 weeks prior to enrollment
3. Leptomeningeal disease.
4. Patients with paraspinal, paratracheal and mediastinal pathological lesions larger than 2 cm unless they are previously irradiated. Irradiation of lesions must be completed at least 14 days prior to initiation of study treatment.
5. Patients with another invasive malignancy in the last 2 years (with the exception of basal cell carcinoma and tumors deemed by the investigator to be of low likelihood for recurrence)
6. Evidence of significant, uncontrolled concomitant diseases that could affect compliance with the protocol or interpretation of results or contraindicate the use of an investigational drug, including diabetes mellitus, history of relevant cardio-pulmonary disorders, and known autoimmune diseases
7. Patients with bilateral lung lesions and dyspnea and/or with bilateral lung lesions and SaO2 < 92% (at rest and with exercise, room air) or patients with lobectomy or pneumonectomy with lung metastases in the remaining lung and either dyspnea or SaO2 less than 92% (at rest and with exercise, room air) at baseline
8. Uncontrolled hypertension (systolic blood pressure (BP) > 150 mmHg and/or diastolic BP > 100 mmHg), unstable angina, congestive heart failure of any New York Heart Association classification, serious cardiac arrhythmia that requires treatment with the exceptions of atrial fibrillation and paroxysmal supraventricular tachycardia, and history of myocardial infarction within 6 months of enrollment

“Use of this document is governed by the terms of use on the first page of this document.”

9. Active or uncontrolled infections
10. Known HIV or known active hepatitis B or hepatitis C infection for patients not receiving obinutuzumab pretreatment
11. Major surgery or significant traumatic injury < 28 days prior to the first RO6958688 infusion (excluding biopsies) or anticipation of the need for major surgery during study treatment
12. Dementia or altered mental status that would prohibit informed consent
13. Pregnant or breastfeeding women
14. Known hypersensitivity to any of the components of RO6958688 and/or obinutuzumab
15. Concurrent therapy with any other investigational drug (defined as treatment for which there is currently no regulatory authority–approved indication) < 28 days prior to the first RO6958688 infusion
16. Last dose of any chemotherapy < 28 days prior to the first RO6958688 infusion
17. Last dose with an antibody or immunotherapy (i.e., interferon-α, interferon-β, IL-2, etanercept, infliximab, tacrolimus, cyclosporine, mycophenolic acid, alefacept, or efalizumab) < 28 days prior to the first RO6958688 infusion
18. Last dose of anti-CTLA4, anti–PD-L1, or anti-PD1 < 28 days prior to the first RO6958688 infusion
19. Expected need for regular immunosuppressive therapy (i.e., for organ transplantation, chronic rheumatologic disease) within 28 days prior to the first RO6958688 infusion
20. Regular dose of corticosteroids the 28 days prior to Day 1 of this study or anticipated need for corticosteroids that exceeds prednisone 10 mg/day or equivalent within 28 days prior to the first RO6958688 infusion, with the exception of the obinutuzumab steroid premedication. Inhaled and topical steroids are permitted
21. Baseline corrected QT (QTc) interval of > 470 ms. Patients with baseline resting bradycardia < 45 beats per minute or baseline resting tachycardia > 100 beats per minute
22. Radiotherapy within the last 28 days prior to the first RO6958688 infusion with the exception of limited–field palliative radiotherapy
23. Administration of a live, attenuated vaccine within 28 days before Cycle 1, Day 1 or anticipation that such a live attenuated vaccine will be required during the study.
24. History of progressive multifocal leukoencephalopathy (PML).

Additional exclusion criteria for patients to be pretreated with obinutuzumab:

- Positive test results for human T-lymphotropic virus 1 (HTLV-1) or active HIV infection. HTLV-1 testing is required participants from endemic countries (Japan, countries in the Caribbean basin, South America, Central America, sub--Saharan Africa, and Melanesia)

“Use of this document is governed by the terms of use on the first page of this document.”

- Positive test results for chronic hepatitis B infection. All patients must be tested for both HBsAg and HBcAb at screening, if either of the tests is positive, the patient is not eligible for inclusion in the trial. Patients who have protective titers of HBsAb after vaccination are eligible provided they are negative for both HBsAg and HBcAb, patients with negative result by PCR regarding Hepatitis B and C are eligible
- Positive test results for hepatitis C (hepatitis C virus [HCV] antibody serology testing)
- Known active TB requiring treatment within 3 years prior to baseline or latent TB that has not been appropriately treated
- Active bacterial, viral, fungal, or other infection, or any major episode of infection requiring treatment with IV antibiotics within 4 weeks of Cycle 1 Day 1
- International normalized ratio (INR) or prothrombin time (PT) > 1.5 × ULN in the absence of therapeutic anticoagulation
- PTT or aPTT > 1.5 x ULN in the absence of a lupus anticoagulant
- Known hypersensitivity to any of the components of obinutuzumab; hypersensitivity to Chinese hamster ovary cell products or other recombinant human antibodies.
- History of progressive multifocal leukoencephalopathy (PML)

4.3 STUDY TREATMENTS

For the purpose of the study, RO6958688, obinutuzumab and tocilizumab are considered the investigational medicinal products (IMPs).

4.3.1 Formulation, Packaging, and Handling

Study drugs packaging will be overseen by the Sponsor's clinical trial supplies department and bear a label with the identification required by local law, the protocol number, drug identification, and dosage. The packaging and labeling of the study medications will be in accordance with the Sponsor's standard and local regulations. Upon arrival of investigational products at the site, site personnel should check them for damage and verify proper identity, quantity, integrity of seals, and temperature conditions and report any deviations or product complaints to the monitor upon discovery.

The qualified individual responsible for dispensing the study drugs will prepare the correct dose according to the schedule. This individual will write the date of dispensing and patient number and initials on the study drug vial label and on the Drug Accountability Record. This individual will also record the study drug batch or lot number received by each patient during the study.

"Use of this document is governed by the terms of use on the first page of this document."

4.3.1.1 RO6958688

The manufacture of RO6958688 drug substance consists of fermentation cell culture and purification.

Ro 695-8688/F01 drug product is provided as a sterile, preservative-free, liquid concentrate for infusion in 6-mL glass vials with L-histidine/L-histidine hydrochloride aqueous buffer solution (pH 5.5) containing sucrose, methionine, and polysorbate 20. Each single-use, 6-mL vial contains 25 mg (nominal strength) of RO6958688, and the drug product is formulated at a concentration of 5 mg/mL.

A variant of the current formulation with higher protein concentration (the composition stays the same except for the protein concentration) has been developed to reduce the drug product volume and increase handling/patient convenience. Ro 695-8688/F03 drug product is provided as a sterile, preservative-free, liquid concentrate for infusion in 6-mL glass vials with L-histidine/L-histidine hydrochloride aqueous buffer solution (pH 5.5) containing sucrose, methionine, and polysorbate 20. Each single-use, 6-mL vial contains 100 mg (nominal strength) of RO6958688, and the drug product is formulated at a concentration of 20 mg/mL.

	Phase I formulation	New formulation
Roformis No.	Ro 695-8688/F01	Ro 695-8688/F03
Protein concentration	5 mg/ml	20 mg/ml
Vial strength	25 mg/5 ml	100 mg/5 ml

The recommended storage condition for Ro 695-8688/F01 and Ro 695-8688/F03 drug products is between 2°C–8°C, and it should be protected from light.

Upon arrival of investigational products at the site, site personnel should check them for damage and verify proper identity, quantity, integrity of seals and temperature conditions, and report any deviations or product complaints to the monitor upon discovery.

For further details on the storage and preparation of RO6958688, please refer to the BP29541 Pharmacy manual and RO6958688 Investigator’s Brochure.

4.3.1.2 Preparation of obinutuzumab

Obinutuzumab is provided as a single dose 1000 mg liquid concentrate for infusion containing of 25 mg/mL obinutuzumab. The 1000 mg dose is supplied in 50 mL glass vials containing 40 mL of the 25 mg/mL liquid concentrate. In addition to the drug substance, the liquid is also composed of histidine/histidine-HCl, trehalose and poloxamer 188. Obinutuzumab should be prepared by a healthcare professional using aseptic technique. The required amount of liquid concentrate should be drawn from the vial and diluted in PVC or non-PVC polyolefin infusion bags containing sterile, non-pyrogenic 0.9% aqueous sodium chloride (NaCl) solution. Other diluents such as dextrose (5%) solution should not be used.

“Use of this document is governed by the terms of use on the first page of this document.”

The recommended storage conditions for the drug product obinutuzumab are between 2°C and 8°C (36 – 46°F), protected from light. Obinutuzumab vials should not be frozen or shaken. Mix gently. All transfer procedures require strict adherence to aseptic techniques. Obinutuzumab drug product intended for IV infusion is prepared by dilution of the drug product into an infusion bag containing 0.9% NaCl.

For further details on the storage and preparation of obinutuzumab, please refer to the BP29541 obinutuzumab Pharmacy manual and obinutuzumab Investigator’s Brochure.

4.3.1.3 Tocilizumab

The tocilizumab drug product will be supplied by the Sponsor. Tocilizumab will be supplied in vials containing sterile solution of tocilizumab (20 mg/mL). Tocilizumab will be supplied as 200-mg vials.

Tocilizumab should be administered when necessary, as described in Table 10 For information on the formulation and handling of tocilizumab, see the pharmacy manual and the Tocilizumab Investigator's Brochure.

In an emergency situation where the study specific labeled supply of tocilizumab is not accessible, it may be supplied locally by the study sites and will be formulated, prepared, and handled according to the local prescribing information. For further instructions regarding recommended storage conditions and packaging configuration, refer to the local prescribing information.

4.3.2 Administration

4.3.2.1 Administration of RO6958688, obinutuzumab and tocilizumab

RO6958688, obinutuzumab and tocilizumab must be administered in a hospital or clinic equipped for IV chemotherapy. Full emergency resuscitation facilities should be immediately available on site, and patients should be under close supervision of the investigator at all times.

4.3.2.1.1 RO6958688

RO6958688 is given as an IV infusion as a single administration in Part I (exception may apply for patients to receive further administrations at the discretion of the investigator in case there is no safety concern) and as either QW or Q3W administration (one cycle equals one week in the QW regimen and one cycle equals three weeks in the Q3W regimen), in the step up dosing regimen. RO6958688 is administered as QW cycle for the first 3 cycles followed by Q3W cycles in Part II of the study.

If premedication is needed prior to RO6958688 infusion, it should be administered at least 30 minutes before the start of RO6958688 infusion. Please refer to section 4.3.2.2 for details of pre-medications prior to RO6958688 infusion.

Hypotension may occur as a result of an IRR/CRS, therefore, it is recommended that antihypertensive drugs must not be given on the morning of, and throughout the infusion

“Use of this document is governed by the terms of use on the first page of this document.”

of RO6958688 if clinically indicated. Patients with a history of cardiac disease should be monitored closely.

At the discretion of the investigator and in case there is no safety concern, all patients can receive further doses QW at the same dose level. Dose escalation to the next available tolerated dose level can only proceed after a patient has tolerated his or her current dose for at least 2 months (for the dose escalation phase of Part I and Part II), this was only applicable to patients enrolled in Europe.

During infusion, vital signs including, if possible, supine diastolic and systolic BP, heart rate, and temperature have to be monitored pre-infusion, every 15 minutes until the end of infusion, and thereafter, every 30 minutes until the infusion line is removed. After the first infusion, vital signs will be obtained on Day 1 pre-infusion, every 30 minutes during infusion, and at 30 minutes after infusion. Vital signs during infusion are not required to be captured in the electronic Case Report Form (eCRF) unless abnormalities are observed.

After the end of the first infusion, the IV line should remain in place for 2 hours. If no infusion-related symptoms occur during this time, the infusion line may be removed. For subsequent infusions and if no IRR has been reported, the IV line should remain in place for 30 minutes after the end of infusion and if no adverse events occur during the 30 minutes, the infusion line may be removed. If feasible, the line for drawing blood for PK samples (opposite extremity to the one with the infusion line) will remain in place until the 24-hour sample is taken (if required).

Guidelines for dosage modification and treatment interruption or discontinuation are provided in Section 5.2.4.

4.3.2.1.2 Obinutuzumab

The dose level of obinutuzumab in this study is 2000 mg administered by IV infusion of either 2000 mg on Day-13 or 1000 mg on Day-13 and Day-12. In Cohort H, the patients will receive according to patient’s and/or investigators convenience, either 2000 mg of obinutuzumab IV on Day-7 or 1000 mg of obinutuzumab IV on two consecutive days, Day-8 and Day-7 (+ 1 day) before C1D1 RO6958688 administration.

Obinutuzumab drug product intended for IV infusion is prepared by dilution of the drug product into an infusion bag containing 0.9% NaCl.

DO NOT USE OTHER DILUENTS SUCH AS GLUCOSE (5%) SOLUTION.

Obinutuzumab drug product intended for IV infusion is prepared by dilution of the drug product into an infusion bag containing 0.9% NaCl to the final drug concentration of 4 mg/mL. Using a 250-mL infusion bag containing 0.9% NaCl, withdraw and discard 40 mL of the sodium chloride. Withdraw 40 mL of obinutuzumab from a single glass vial

“Use of this document is governed by the terms of use on the first page of this document.”

and inject into the infusion bag (discard any unused portion of obinutuzumab left in the vial). Gently invert the infusion bag to mix the solution; do not shake. Administration sets with polyvinyl chloride (PVC), polyurethane (PUR), or polyethylene as product contact surface and IV bags with polyolefine, polypropylene (PP), PVC, or polyethylene, as product contact surface are compatible and may be used.

Chemical and physical in-use stability for obinutuzumab diluted in 0.9% NaCl in the concentration range 0.4 to 20 mg/mL have been demonstrated for 24 hours at 2°C to 8°C and an additional 24 hours at ambient temperature (≤30°C).

From a microbiological point of view, the product should be used immediately. If not used immediately, in-use storage times and conditions prior to use are the responsibility of the user and would normally not be longer than 24 hours at 2°C to 8°C, unless reconstitution/dilution has taken place in controlled and validated aseptic conditions.

Obinutuzumab vials do not contain antimicrobial preservatives. Therefore, care must be taken to ensure that the solution for infusion is not microbiologically compromised during preparation.

Obinutuzumab will be provided by Roche to investigation centers.

For further details, see the obinutuzumab Investigator's Brochure.

Premedication Requirements for Infusion-related Reactions:

Premedication must be administered at least 60 minutes (unless contraindicated) prior to each obinutuzumab administration with:

- corticosteroids (100 mg IV prednisone/prednisolone or 20 mg dexamethasone or 80 mg methylprednisolone)
- paracetamol / oral acetaminophen (1000 mg orally)
- diphenhydramine (50-100 mg orally or IV; or an alternative anti-histamine at an adequate dose)

Hypotension may occur as a result of an IRR, therefore, we recommend withholding of antihypertensive treatments to be considered for 12 hours prior to and throughout each obinutuzumab infusion and for the first hour after administration. Patients with a history of cardiac disease should be monitored closely.

Administration

Obinutuzumab will be administered as pretreatment either on Day-13 at a dose of 2000 mg or on two consecutive days, Day-13 and Day-12 at a dose of 1000 mg prior to treatment start with RO6958688 on Cycle 1 Day 1. In Cohort H, the patients will receive according to patient’s and/or investigators convenience, either 2000 mg of obinutuzumab

“Use of this document is governed by the terms of use on the first page of this document.”

IV on Day-7 or 1000 mg of obinutuzumab IV on two consecutive days, Day-8 and Day-7 (+ 1 day) before C1D1 RO6958688 administration.

If obinutuzumab is administered in 1 dose of 2000 mg, the following should apply:

Day-7 (2000 mg) Infusion of obinutuzumab:

Please ensure that the premedication (acetaminophen, antihistamine and corticosteroids) for IRRs are administered. During infusion, vital signs (including, if possible, supine diastolic and systolic blood pressure, pulse rate and temperature) have to be monitored pre-infusion, every 15 minutes for the first 90 minutes of the infusion, and then every 30 minutes until 1 hour after completion of the infusion.

The obinutuzumab infusion will be administered at an initial rate of 50 mg/hr. In the absence of IRRs/hypersensitivity reaction, the rate of the infusion will be escalated in increments of 50 mg/hr every 30 minutes to a maximum rate of 400 mg/hr. The total administration time is over 9 hours, i.e. 50 mg for 1h, 100, followed by 150, 200, 250, 300 and 350 mg/h for each 30 min followed by finally 400 mg/h until end (see Table 3 for more details).

If a hypersensitivity or IRR develops, the infusion should be temporarily interrupted or slowed down and concomitant medication may be administered if deemed appropriate by the investigator.

Upon the resolution of symptoms, the infusion will resume at one-half the previous rate (the rate being used at the time that the hypersensitivity or IRR occurred) and infusion-rate escalation may resume at the increments and intervals described above.

Table 3 Obinutuzumab Infusion Instructions (2000 mg infusion in one day)

**Administration (2000 mg) on Day-7**
Begin infusion at an initial rate of 50 mg/h for 1 hour.<br>If no infusion-related reaction occurs, increase the infusion rate in 50 mg/h increments every 30 min, to a maximum of 400 mg/h.<br><br>If an IRR develops, stop or slow the infusion. Administer symptomatic treatment and supportive care which may include acetaminophen (≥ 500 mg), H1- and H2-histamine-receptor antagonists such as diphenhydramine hydrochloride and ranitidine, supplemental oxygen, β2-agonists, epinephrine, and/or corticosteroids.<br>In case of one Grade 4 IRR obinutuzumab administration has to be permanently discontinued.<br><br>Resume the infusion at a 50% reduction in rate (the rate being used at the time that the hypersensitivity or infusion-related reaction occurred) if the IRR has resolved.

“Use of this document is governed by the terms of use on the first page of this document.”

If obinutuzumab is administered in 2 doses of 1000 mg, the following should apply:

Day-8 (1000 mg) Infusion of obinutuzumab:

Please ensure that the premedication (acetaminophen, antihistamine and corticosteroids) for IRRs are administered. During infusion, vital signs (including, if possible, supine diastolic and systolic blood pressure, pulse rate and temperature) have to be monitored pre-infusion, every hour until the end of infusion, and thereafter, until the infusion line is removed.

The obinutuzumab infusion will be administered at an initial rate of 50 mg/hr. In the absence of IRRs/hypersensitivity reaction, the rate of the infusion will be escalated in increments of 50 mg/hr every 30 minutes to a maximum rate of 400 mg/hr. The total administration time is over. 5 hours, i.e. 50 mg for 1h, 100, followed by 150, 200, 250, 300 and 350 mg/h for each 30 min followed by finally 400 mg/h until end.

If a hypersensitivity or IRR develops, the infusion should be temporarily interrupted or slowed down and concomitant medication may be administered if deemed appropriate by the investigator.

Upon the resolution of symptoms, the infusion will resume at one-half the previous rate (the rate being used at the time that the hypersensitivity or IRR occurred) and infusion-rate escalation may resume at the increments and intervals described above.

Day-7 (1000 mg) Infusion of obinutuzumab:

Please ensure that the premedication (acetaminophen, antihistamine and corticosteroids) for IRRs is administered.

If the patient's infusion of obinutuzumab on Day-8 is well tolerated (defined by the absence of Grade 2 IRRs during a final infusion rate of ≥ 100 mg/hr), the infusion on Day-7 will be administered at an initial rate of 100 mg/hr and increased by 100 mg/hr increments at 30-minute intervals, as tolerated, to a maximum rate of 400 mg/hr. If the previous infusion rate was not well tolerated, as defined above, instructions for the second split dose infusion rate will be identical as on Day-8. If a hypersensitivity or IRR develops, the infusion should be temporarily interrupted. Upon the resolution of symptoms, the infusion will resume at one-half the previous rate (the rate being used at the time that the hypersensitivity or infusion-related reaction occurred) and infusion-rate escalation may resume at the increments and intervals described below in the Table 4.

For the management of IRRs, hypersensitivity and anaphylaxis, see Section 5.2.6.1.

"Use of this document is governed by the terms of use on the first page of this document."

Table 4 Obinutuzumab Infusion Instructions (1000 mg infusion in two consecutive days)

First administration (1000 mg) on Day-8	Second administration (1000 mg) on Day-7
Begin infusion at an initial rate of 50 mg/h for 1 hour.<br>If no infusion-related reaction occurs, increase the infusion rate in 50 mg/h increments every 30 min, to a maximum of 400 mg/h.<br><br>If an IRR develops, stop or slow the infusion (depending on severity). Administer symptomatic treatment and supportive care which may include acetaminophen (≥ 500 mg), H1- and H2-histamine-receptor antagonists such as diphenhydramine hydrochloride and ranitidine, supplemental oxygen, β2-agonists, epinephrine, and/or corticosteroids.<br>In case of a Grade 4 IRR stop infusion immediately, give aggressive supportive care and obinutuzumab therapy permanently discontinued.<br><br>Resume the infusion at a 50% reduction in rate (the rate being used at the time that the hypersensitivity or infusion-related reaction occurred) if the IRR has resolved.	If the patient experienced an infusion-related reaction during the first split dose infusion (Day-8), start at the same rate as the first split dose infusion (50 mg/hr) and follow directions as noted.<br><br>If the patient tolerated the first split dose infusion well (Day-8), begin infusion at a rate of 100 mg/hr.<br><br>If no IRR occurs, increase the infusion rate in 100 mg/hr increments every 30 minutes, to a maximum of 400 mg/hr.<br><br>If an IRR develops, stop or slow the infusion.<br><br>Administer symptomatic treatment and supportive care which may include acetaminophen (≥ 500 mg), H1- and H2-histamine-receptor antagonists such as diphenhydramine hydrochloride and ranitidine, supplemental oxygen, β2-agonists, epinephrine, and/or corticosteroids. In case of a Grade 3 or 4 IRR, stop infusion immediately, give aggressive supportive care and permanently discontinue obinutuzumab therapy.<br><br>In case of Grade 1 or 2 IRR, resume the infusion at a 50% reduction in rate (the rate being used at the time that the IRR occurred) if the IRR has resolved.

4.3.2.1.3 Administration of tocilizumab

Tocilizumab infusion will follow the methods described in the Investigator Brochure or other similar local prescribing documents and will be administered for the treatment of CRS considered related to RO6958688 (refer to Section 5.2.5.1.5 for management for CRS).

“Use of this document is governed by the terms of use on the first page of this document.”

Tocilizumab (8 mg/kg) will be administered at room temperature by controlled IV infusion into over a 1-hour period. The infusion rate must be 10 mL/hr for 15 minutes and then increased to 130 mL/hr to complete the dosing over the 1-hour time period. Normal saline (20 mL) will be administered following infusion of study drug to flush the remaining study drug through the IV set.

4.3.2.2 Pre-medication and Post-medication for RO6958688

The use of analgesics, NSAID and/or antihistamines is allowed to minimize expected flu-like symptoms associated with the administration of RO6958688 as follows:

- Paracetamol/acetaminophen (500–1000 mg orally or IV)
- Diphenhydramine (50-100 mg [orally or IV] or an alternative antihistamine at an adequate dose)

Pre-medicate all patients in step-up cohorts with 10 mg dexamethasone IV until the RO6958688 dose escalation has been completed (Table 5). If an IRR/CRS occurs, follow IRR/CRS management guidelines in Table 10

.Table 5 Pre-medications to be administered before RO6958688 infusion

Infusion of RO6958688	Patients requiring pre-medications	Pre-medication**	Administration
During step-up phase	All patients	IV dexamethasone 10mg	Completed at least 1 hour prior to RO6958688 infusion
		Analgesic and/or NSAID	At least 30 min before RO6958688 infusion
		Anti-histamine	
During step-up phase	IRR/CRS G2*** with previous RO6958688 infusion despite pre-medications	For corticosteroid use see CRS/IRR management Table 10	
		Analgesic, anti-emetics and/or NSAID	At least 30 min before RO6958688 infusion
		Anti-histamine	
During flat dose phase†	Patient with no IRR/CRS at previous infusion	Analgesic, anti-emetics and/or NSAID	At least 30 min before RO6958688 infusion
		Anti-histamine	
	Patient with G1 or G2 IRR/CRS at previous infusion	Analgesic, anti-emetics and/or NSAID	At least 30 min before RO6958688 infusion
		Anti-histamine	
		For corticosteroid use see CRS/IRR management Table 10	

**Analgesic- Paracetamol/acetaminophen 500-1000 mg orally or IV. Anti-histamine diphenhydramine 50-100 mg orally or IV or alternate anti-histamine at adequate dose
***Per Table 10, in patients with IRR/CRS recurring at ≥Grade 3, not recovering within 8 hours of corticosteroids and tocilizumab and IRR/CRS G4 RO6958688 should be permanently discontinued
† Patients in flat dose cohorts to follow this recommendation

“Use of this document is governed by the terms of use on the first page of this document.”

4.3.2.3 Infusion of RO6958688

Part I

RO6958688 will be administered by IV infusion over a minimum of 30 minutes.

Part II

RO6958688 should be administered at Cycle 1 Day 1 by IV infusion over a minimum of 2 hours (120 minutes), subsequent infusions should be administered in at least 4 hours during the dose/escalation phase, in patients with no Grade ≥ 2 IRR/CRS for more than two RO6958688 administrations, the infusion time can be progressively reduced to a minimum of 1 hour. For more details, please refer to the RO6958688 pharmacy manual.

Premedication should be administered at least 30 minutes before the start of RO6958688 infusion.

Based on chemical and physical in-use stability data of RO6958688, the infusion time should not exceed 8 hours at room temperature including interruptions (if any), the Pharmacy Manual includes detailed instructions regarding refrigerated storage of the dose solution prior to administration.

Information on cohorts, doses, and administration parameters for both Part I and Part II are described in the Pharmacy Manual.

In case of IRR occurring during the administration of RO6958688, the infusion will be interrupted until complete resolution of symptoms and may be resumed at 50% of the previous rate. The infusion can be re-escalated to initial rate if the infusion is considered well tolerated after 1 hour of infusion.

In case of a ≥Grade 2 IRR occurring for the first time in a patient at the second or subsequent infusions, the subsequent infusion rate of the patient will be decreased to 50% of the previous infusion rate.

A detailed administration manual (e.g., doses and infusion rate) will be provided to the investigators. For pre-medication please refer to section 4.3.2.2.

In the case when the infusion is stopped and restarted on the same day, the need for additional premedication will be decided by the investigator. If the infusion is restarted >1 day after the IRR episode (within 48 hours), the patient will receive full premedication 30 minutes before restarting the infusion, including corticosteroids, as judged necessary by the investigator and the Sponsor.

If all above specified precautions failed, further risk-minimization options such as capping of first dose and escalation of second dose or splitting of first dose will be discussed between the Sponsor and investigator and put in place.

“Use of this document is governed by the terms of use on the first page of this document.”

Dose, date, and time of the infusion commencement, date and time of end of infusion, total dose administrated, and interruption or adjustment of infusion rate and reason will be recorded for each patient. If infusion is interrupted, the date and time of stopping and resuming infusion will also be recorded.

4.3.3 Investigational Medicinal Product Accountability

All IMPs required for completion of this study (RO6958688 and obinutuzumab are the main IMPs) will be provided by the Sponsor. The investigational site will acknowledge receipt of IMPs to confirm the shipment condition and content. Any damaged shipments will be replaced.

Since tocilizumab will be used in the event of a severe CRS, it is therefore classified as an investigational medicinal product (IMP). Tocilizumab will be provided by the Sponsor.

The investigator is responsible for the control of IMP. Adequate records of the receipt (i.e., Drug Receipt Record) and disposition (i.e., Drug Dispensing Log) of the study drug must be maintained. The Drug Dispensing Log must be kept current and should contain the following information:

- The identification of the patient to whom the study drug was dispensed (for example, patient's initials and date of birth)
- All records and drug supplies, which must be available for inspection by the Sponsor's Medical Monitor at every monitoring visit.

IMPs will either be disposed of at the study site according to the study site's institutional Standard Operating Procedure or returned to the Sponsor with the appropriate documentation. The site's method of IMP destruction must be agreed upon by the Sponsor. Local or institutional regulations may require immediate destruction of used IMP for safety reasons. In these cases, it may be acceptable for staff at the investigational study site to destroy dispensed IMP before a monitoring inspection provided that source document verification is performed on the remaining inventory and reconciled against the documentation of quantity shipped, dispensed, returned, and destroyed and provided that the adequate storage and integrity of drug have been confirmed.

The site must obtain written authorization from the Sponsor before any IMP is destroyed, and IMP destruction must be documented on the appropriate form.

Written documentation of destruction must contain the following:

- Identity batch numbers of IMPs destroyed
- Quantity of IMPs destroyed
- Date of destruction
- Method of destruction
- Name and signature of responsible person (or company) who destroyed the IMPs.

"Use of this document is governed by the terms of use on the first page of this document."

Accurate records of all IMPs received at, dispensed from, returned to, and disposed of by the study site should be recorded on the Drug Inventory Log.

4.3.4 Post-Trial Access to RO6958688

The Sponsor will offer post-trial access to the study drug (RO6958688) free of charge to eligible patients in accordance with the Roche Global Policy on Continued Access to Investigational Medicinal Product, as outlined below.

A patient will be eligible to receive study drug after the end of the study if all of the following conditions are met:

- The patient has a life-threatening or severe medical condition and requires continued study drug treatment for his or her well-being
- There are no appropriate alternative treatments available to the patient
- The patient and his or her doctor comply with and satisfy any legal or regulatory requirements that apply to them.

A patient will not be eligible to receive study drug after the end of the study if any of the following conditions are met:

- The study drug is commercially marketed in the patient's country and is reasonably accessible to the patient (e.g., is covered by the patient's insurance or wouldn't otherwise create a financial hardship for the patient)
- The Sponsor has discontinued development of the study drug or data suggest that the study drug is not effective for cancer
- The Sponsor has reasonable safety concerns regarding the study drug as treatment for cancer
- Provision of study drug is not permitted under the laws and regulations of the patient's country.

F. Hoffmann La-Roche does not intend to provide obinutuzumab or other study intervention to patients after conclusion of the study or in case of earlier patient withdrawal.

4.4 CONCOMITANT THERAPY

4.4.1 Permitted Therapy

Concomitant therapy includes any medication (i.e., prescription drugs, over-the-counter drugs and approved dietary, and nutritional supplements) used by a patient from the screening period until the follow-up visit. If any treatment as specified in the exclusion criteria (see Section 4.2.3) is given within 4 weeks prior to screening, it should be recorded in the eCRF. All concomitant medications should be reported to the investigator and recorded in the eCRF. Concomitant use of herbal therapies is not recommended because their pharmacokinetics, safety profiles, and potential drug-drug interactions are generally unknown.

“Use of this document is governed by the terms of use on the first page of this document.”

All therapies and/or medications administered to manage adverse events should be recorded in the Adverse Event eCRF.

Use of Steroids

The concomitant administration of corticosteroids (i.e., as a premedication for RO6958688 or a treatment of nausea/vomiting) must be carefully considered, and duplicate use of steroids should be avoided because this may have an impact on the efficacy of RO6958688. Therefore, the chronic use of systemic steroids will not be allowed (see exclusion criteria, Section 4.2.3). Use of steroids as a premedication if deemed necessary after discussion with the Sponsor Medical monitor, post first RO6958688 infusion for the QW and Q3W flat dose schedules and for step up cohorts or as a treatment for adverse events is allowed as described in Section 4.3.2.1.3. Premedication including steroid must be administered prior to obinutuzumab administration.

Radiotherapy

The use of palliative radiotherapy is allowed at any time during the study except for:

- Days where study drugs are administered
- One day prior or post study drug administration is observed.

No delay of study drug administration is foreseen although patients should not receive study treatment during radiation treatment.

4.4.2 Prohibited Therapy

The use of the following therapies is prohibited during the study and for at least 28 days prior to initiation of study treatment (unless otherwise specified):

- Investigational or unlicensed/unapproved agents
- Immunotherapy/radio-immunotherapy
- Chemotherapy
- Radiotherapy (with the exception of limited–field palliative radiotherapy)
- Biologic agents (e.g., bevacizumab, erlotinib)
- Chronic use of steroids (inhaled and topical steroids are permitted)
- Administration of a live, attenuated vaccine within 28 days before Cycle 1, Day 1 or anticipation that such a live attenuated vaccine will be required during the study

4.5 STUDY ASSESSMENTS

4.5.1 Description of Study Assessments

All examinations listed below will be performed according to the Schedule of Assessment and Hourly Assessment tables outlined in Appendix 1.

"Use of this document is governed by the terms of use on the first page of this document."

4.5.1.1 Medical History and Demographic Data

Medical history includes clinically significant diseases, demographic data (including age, sex, and self-reported race/ethnicity), all surgeries, cancer history (including any biomarker information (e.g. tumor mutations like KRAS, BRAF and NRAS, CEA expression level, all prior cancer therapies [including adjuvant and neoadjuvant therapies] and procedures), reproductive status, smoking history, and use of alcohol, drugs of abuse, and concomitant medications.

4.5.1.2 Physical Examinations, Vital Signs, and ECOG Performance Status

A complete physical examination should include an evaluation of the head, eyes, ears, nose, throat, neck and lymph nodes, and the cardiovascular, dermatological, musculoskeletal, respiratory, gastrointestinal, genitourinary, and neurological systems.

Any abnormality identified at baseline should be recorded on the General Medical History and Baseline Conditions eCRF.

A physical examination will include careful examinations of the areas of known and possible malignancy. The physical examination includes weight (height measurement is only done at screening and at follow-up visit). For the purpose of the study, the same calibrated balance should be used at each site. Vital signs (include resting BP in supine position and heart rate, oxygen saturation at rest, in patients with bilateral lung lesions or patients with lobectomy or pneumonectomy with lung metastases in the remaining lung oxygen saturation with exercise is also required) and ECOG PS will be obtained in all patients at screening and as described in the Schedule of Assessment and Hourly Assessment tables (see Appendix 1).

PS will be measured using the ECOG PS scale (see Appendix 3). PS will be assessed with each physical examination, prior to each study drug administration, and at the safety follow-up visit. It is recommended, where possible, that a patient's PS be assessed by the same person throughout the study.

Results will be recorded on the eCRF. Changes from baseline abnormalities should be recorded in patient's notes. New or worsened clinically significant abnormalities should be recorded as adverse events on the Adverse Event eCRF.

Routine vital signs monitoring includes BP, heart rate, oxygen saturation and body temperature will be recorded at the timepoints specified in Schedule of Assessment and Hourly Assessment tables (see Appendix 1).

During the day, BP and heart rate should be obtained in a quiet room at a comfortable temperature, with the patient's arm unconstrained by clothing or other material.
All measurements will be obtained from the same arm and with the same cuff size using an automatic instrument with a digital readout throughout the study. The "ideal" cuff

"Use of this document is governed by the terms of use on the first page of this document."

should have a bladder length that is 80% and a width that is at least 40% of arm circumference (a length-to-width ratio of 2:1). The automatic cuff should be placed on the designated arm at least 10 minutes prior to dosing. The patient should have his or her back and arm supported such that the middle of the cuff on the upper arm is at the level of the right atrium (the midpoint of the sternum). After the patient has been resting in supine decubitus position for at least 5 minutes, BP and heart rate will be obtained.

4.5.1.3 Electrocardiograms

Triplicate 12-lead ECG recordings (i.e., three qualitatively acceptable ECGs without artifacts) must be obtained at the timepoints as specified in the Schedule of Assessments (see Appendix 1) and within approximately 2–5 minutes at each specified timepoint. The average of the three readings will be used to determine the ECG intervals (i.e., PR, QRS, QT). Additional unscheduled ECG assessments should be performed in case of abnormalities and if clinical symptoms occur.

To minimize variability, it is important that the patient is in a supine position for ≥5 minutes prior to each ECG evaluation. The conditions should be as close as possible to predose timepoints; this includes but is not limited to food intake, activity level, stressors, and room temperature. Body position should be consistently maintained for each ECG evaluation to prevent changes in heart rate. Environmental distractions (e.g., television, radio, conversation) should be avoided during the pre-ECG resting period and during ECG recording. Whenever possible, ECGs should be performed prior to meals, any scheduled vital sign measurements, and blood draws. In some cases, it may be appropriate to repeat abnormal ECGs to rule out improper lead placement that may contribute to the abnormality in ECG results.

For safety monitoring purposes, the investigator or designee must review, sign, and date all ECG tracings. Paper or electronic copies will be kept as part of the patient's permanent study file at the site. If considered appropriate by the Sponsor, ECGs may be analyzed retrospectively at a central laboratory.

ECG characteristics including heart rate, QRS duration, and PR and QT intervals will be recorded on the eCRF. QTcB (Bazett's correction), QTcF (Fridericia's correction), and RR will be recorded on the eCRF. Changes in T-wave and U-wave morphology and overall ECG interpretation will be documented on the eCRF. T-wave information will be captured as normal or abnormal, whereas U-wave information will be captured in two categories, absent/normal or abnormal.

4.5.1.4 Pulmonary function tests (FEV1/VC/TLC and DL_{CO})

FEV1/VC/TLC (including all derived parameters: inspiratory reserve volume, expiratory reserve volume, tidal volume and total lung capacity) and DL_{CO} (corrected for both alveolar volume and hemoglobin) will be evaluated at screening in patients with bilateral lung metastases or patients with lobectomy or pneumonectomy with lung metastases in the remaining lung. Additional tests may be performed if clinically indicated.

"Use of this document is governed by the terms of use on the first page of this document."

4.5.1.5 Laboratory Assessments

Normal ranges for the study laboratory parameters must be supplied to the Sponsor before the start of the study. Laboratory safety tests shall be collected at timepoints specified in the Schedule of Assessments (see Appendix 1).

Additional blood or urine samples may be taken at the discretion of the investigator if the results of any test fall outside the reference ranges or the clinical symptoms necessitate additional testing to monitor patient safety. When the clinical significance of abnormal laboratory results is considered uncertain, screening lab tests may be repeated before enrollment to confirm eligibility. If there is an alternative explanation for a positive urine or blood test for drugs of abuse (e.g., previous occasional intake of a medication or food containing codeine, benzodiazepines, or opiates), the test can be repeated to confirm washout.

In the event of unexplained abnormal, clinically significant laboratory test values, the tests should be repeated immediately and followed up until they have returned to the normal range and/or an adequate explanation of the abnormality is found. Results of clinical laboratory testing will be recorded on the eCRF.

Urinalysis, hematology, and blood biochemistry samples will be sent to the study site's local laboratory for analysis. Freshly obtained tumor biopsies, PK, ADA, serum samples, and samples for flow cytometry and clinical genotyping will be analyzed centrally.
The following assessments should be done:

- Hematology: erythrocytes, hemoglobin, hematocrit, platelets, leukocytes, and differential count (neutrophils, eosinophils, basophils, monocytes, lymphocytes)
- Blood biochemistry: sodium, potassium, chloride, calcium, phosphate, magnesium, urea, creatinine, creatinine clearance (by calculated by CKD-EPI equation), total protein, albumin, glucose, total and direct bilirubin, alkaline phosphatase (ALP), ALT, AST, LDH, GGT, ferritin and high sensitivity C-reactive protein. sCEA will also be measured as a disease monitoring marker (as part of the serum chemistry panel – for part II patients only).
- Lipids: total cholesterol, LDL cholesterol, HDL cholesterol, and triglycerides
- Coagulation: PT, INR, PTT, and fibrinogen
- Blood serology for HBV, HIV, HTLV-1 (from endemic countries (Japan, countries in the Caribbean basin, South America, Central America, sub-Saharan Africa, and Melanesia)) and HCV testing (HB surface antigen [HBsAg], total core HB antibody [anti-HB-cAb], Hepatitis C [HC virus [HCV]) for patients to be pretreated with obinutuzumab, if there is a doubt regarding the result, a PCR can be performed
- Pregnancy test: All women of childbearing potential, including those who have had a tubal ligation, will have a serum pregnancy test at screening within 7 days of first dose and on a regular basis during the study treatment period (serum or urine).
- Urinalysis: Dipstick for pH, glucose, blood, protein, ketones, and bilirubin. If there is a clinically significant positive result (i.e., confirmed by a positive repeated sample),

"Use of this document is governed by the terms of use on the first page of this document."

urine will be sent to the laboratory for microscopy and culture. If there is an explanation for the positive dipstick result (e.g., menses), it should be recorded and there is no need to perform laboratory for microscopy and culture.

Unscheduled hematology, biochemistry, and coagulation will be performed for patients who develop IRRs/CRS and as clinically indicated.

Based on continuous analysis of the data in this study and other studies, any sample type not considered to be critical for safety may be stopped at any time if the data from the samples collected do not produce useful information.

4.5.1.6 Additional Samples

Samples for the following laboratory tests will be sent to one or several central laboratories or to the Sponsor for analysis. During the multiple ascending dose component of Part II, some samples might also be analyzed at sites. Instruction manuals and supply kits will be provided for all central laboratory assessments.

Based on continuous analysis of the data in this study and other studies, any sample type not considered to be critical for safety may be stopped at any time if the data from the samples collected do not produce useful information.

4.5.1.6.1 Pharmacokinetic Assessments

PK samples are mandatory for both Part I and Part II of the study. In order to evaluate the serum levels of RO6958688, blood samples will be collected from an IV line from the arm opposite to that used for study drug administration. The date and time of each sample collection will be recorded in the eCRF. The PK assessments will be performed as outlined in the Schedule of Assessment and Hourly Assessment tables (see Appendix 1). During the course of the study, PK sampling timepoints may be modified on the basis of emerging data to ensure that the pharmacokinetics of RO6958688 can be adequately characterized. Additional PK samples will be taken at the time of treatment discontinuation if the patient experiences an IRR/CRS or an adverse event that leads to dose reduction or delay of RO6958688 administration (see Section 5.2.4). Time windows for PK samples can be found in Appendix 10.

Remaining volumes of PK samples may also be used for assay validation during development of the study, for compound-related exploratory analyses, or to help develop further blood tests, after they are used for the mentioned intended uses.

4.5.1.6.2 Pharmacodynamic, Exploratory, and Clinical Genotyping Assessments

The following samples will be used for research purposes to identify biomarkers useful for predicting and monitoring response to RO6958688 treatment, identifying biomarkers useful for predicting and monitoring RO6958688 safety, assessing PD effects of RO6958688 treatment, and investigating mechanism of resistance to therapy. Additional markers may be measured in the case of a strong scientific rationale.

“Use of this document is governed by the terms of use on the first page of this document.”

Whole Blood Samples for Flow Cytometry

In Part II of the study, whole blood samples will be collected for the flow cytometry. Whole blood samples will be collected as outlined in the Schedule of Assessment and Hourly Assessment tables (see Appendix 1).

Serum or Plasma Samples

Blood for serum or plasma isolation (blood for pharmacodynamics and additional safety) will be collected according to the Schedule of Assessment and Hourly Assessment tables (see Appendix 1). Cytokines, inflammation markers, soluble CEA will be measured in these samples. Additionally, in case of IRR(s) or CRS, an assessment of cytokines released during the reaction will be done on serum or plasma samples from the time of the IRR or CRS. The sample used for these PD analyses will be identical with the one collected for cytokine release at IRR/CRS and IgE and tryptase assessment.

Whole blood samples

From Part II patients, at Cycle 1 Day 1 predose (baseline) in the QW schedule and at Cycle 1 Day 1 predose (Baseline) and Cycle 3 Day 1 predose in the Q3W schedule, and at Cycle 1 Day 1 predose (baseline) in the QWx3/Q3W schedule, mandatory 2 whole blood samples will be taken for TCR Vβ sequencing (the CDR3-TCR beta chain repertoire). The DNA will be used to determine in peripheral T cells the repertoires of T cell receptor (TCR) Vβ CDR3 and analyze TCR diversity.

Soluble CEA

Blood sample will be taken for soluble CEA central assessment, refer to respective Schedule of assessments Appendix 1.

Tumor Biopsy Samples

Tumor biopsy samples (each consisting of two tissue specimens at least the size of a 18G core needle biopsy, fine-needle aspiration is not acceptable) will be collected from all patients who participate in Part II of the study on two occasions (once at baseline [before obinutuzumab pretreatment but after the baseline FDG PET] to confirm eligibility and once during the study treatment period), and two core samples will be collected at each occasion, two biopsy samples will be collected for patients who will be treated at dose levels equal and greater than 5 mg ($\geq$ 5 mg). These tumor biopsies for PD analyses are mandatory at baseline (before obinutuzumab pretreatment but after the baseline FDG PET) and on-treatment. The biopsies will be taken from accessible, "non-critical" tumor locations, including, but not limited to, skin, lymph node, rectum, liver, etc. All on-treatment tumor biopsies will be collected at either at C2D1, C3D1, C4D1 or C7D1 (- 48/0 hours), see Table 2. All biopsies must be taken after FDG-PET, unless, in the absence of available archival tumor tissue, the baseline sample is being used to assess CEA expression for eligibility in which case the biopsy may be taken before the FDG-PET scan.

"Use of this document is governed by the terms of use on the first page of this document."

If preliminary data suggest that modification of the on-treatment tumor biopsy timepoint would be more appropriate, alternative on-treatment tumor biopsy timepoints could be considered in the future cohorts.

Baseline and on-treatment tumor biopsies are mandatory for all patients in the QWx3/Q3W schedule and for each cohort the biopsy will be undertaken as follows (on-treatment biopsies will be undertaken (-48/0 hours)):

- half of the patients will undertake Baseline and C4D1 biopsies
- half of the patients will undertake Baseline and C5D1 biopsies.

Patients being enrolled in cohorts A and B will undertake Baseline and C7D1 pre-dose biopsies.

For patients enrolled in obinutuzumab pre-treatment cohorts, biopsies will be taken at Baseline and C2D1 for Q3W schedule, at Baseline and C4D1 for QW schedule and at Baseline and C4D1 predose for QWx3/Q3W.

For patients continuing in the study, additional biopsies may be taken at the discretion of the investigator to make decisions regarding patient status, and a portion of this biopsy should be sent to Roche (or their designated laboratory) for analysis. An additional optional biopsy at the time of progression can be undertaken in order to provide information regarding potential follow up treatment and escaping mechanism of current treatment.

In addition, if intratumoral PD data suggest the need for PD characterization at an earlier or a later cycle, an additional cohort will be proposed.

The baseline and on-treatment biopsies should ideally be taken from the same tumor lesion to ensure comparability, when accessible in a non-critical location. Data from FDG-PET should guide which lesion would be more reflective of capturing the PD effect and thus the baseline and on-treatment biopsies should be taken from that tumor lesion. All biopsies must be taken after week 6 FDG-PET if feasible, unless, in the absence of available archival tumor tissue, the baseline sample is being used to assess CEA expression for eligibility in which case the biopsy may be taken before the FDG-PET scan.

Positron Emission Tomography (PET)

FDG-PET can identify sign of biological effect early, before tumor size is reduced. Moreover, a reduction in the FDG-PET signal within days or weeks of initiating therapy (e.g., in lymphoma, non–small cell lung, and esophageal cancer) has been shown to correlate with prolonged survival and other clinical end points now used (Weber). These findings suggest that FDG-PET could facilitate drug development as an early marker of drug effect.

“Use of this document is governed by the terms of use on the first page of this document.”

Whole-body imaging PET should begin 60 ± 10 min after FDG injection. Both FDG-PET must be performed before the biopsies.

FDG-PET should be performed according to the schedule of assessment in all eligible patients in Part II of the study (see FDG PET Appendix 9 for more details). Functional imaging will be assessed via [^{18}F]-FDG PET locally. Uptake and retention of [^{18}F]-FDG will be measured by PET/CT imaging in all patients enrolled in Part II.

Archival Tumor Tissue

Formalin–fixed archival tumor tissue embedded in paraffin is to be assessed locally for CEA expression from all patients for confirmation of CEA status at screening (see Appendix 1). These tissues are preferably from the most recent metastatic site (or site of local recurrence) but may be from the primary tumor if metastatic tumor tissue is unavailable. If archival tissue is unavailable, then a fresh tumor biopsy must be obtained for assessment of CEA expression at screening and, if the patient is enrolled (for Part II only), sent to a central laboratory for further exploratory biomarker assessments.

If available, formalin–fixed archival tumor tissue embedded in paraffin blocks is to be submitted for exploratory biomarker assessments, including but not limited to analysis of mutational signatures, microsatellite instability and genomic mutations, from all patients enrolled in Part II only. The tissue block submitted is preferably from the sample used for CEA assessment at screening, if possible. Unstained slides will be accepted if, despite all efforts, tumor block cannot be obtained.

Clinical Genotyping Samples

A mandatory whole blood sample from patients in the Part II of the study will be collected at baseline for DNA extraction. The DNA will be used to determine if genes associated with immune responses including but not limited to chemotaxis, HLA, and immunosuppression affect the pharmacokinetics, pharmacodynamics, efficacy, and safety of RO6958688. These samples will be destroyed after analysis completion and after the results have been checked.

The blood, serum and plasma samples will be destroyed within 2 years after the date of final closure of the clinical database. Archival tumor blocks will be returned. Other residual tissue material (e.g., slides, extracts, on-study blocks, etc.) will be destroyed within 2 years after the final closure of the clinical database unless the patient provides specific consent for the remainder of the tissue sample(s) to be stored for optional exploratory research. If the patient provides consent for optional exploratory research, the tissue samples will be destroyed no later than 15 years after the date of final closure of the clinical database.

For sampling procedures, storage conditions, and shipment instructions, see the separate laboratory manual.

"Use of this document is governed by the terms of use on the first page of this document."

PBMC B-cell isolation (ADA Specificity Assessment)

Examination at the monoclonal B-cell level can provide deeper insights on characterizing the ADA specificity. Whole blood samples (20 mL each time) will be collected from patients receiving RO6958688 with QW schedule (flat or step up dosing schemes) at the clinical site and shipped within 24 hours to a central laboratory according to specific shipment requirements (see specific protocol). Patients receiving obinutuzumab pretreatment or scheduled at Q3W will not be collected and excluded from the PBMC B-cell isolation. In the central laboratory, Peripheral Blood Mononuclear Cell (PBMCs) will be prepared for memory B cell isolation at 3 different timepoints according to the Schedule of Assessment and Hourly Assessment tables (see Appendix 1) at Cycle 1 Day 1 predose, Cycle 7 Day 1 predose and Cycle 12 Day 1 predose from patients receiving RO6958688 QW and at Cycle 1 Day 1 predose, at Cycle 5 Day 1 predose and at Cycle 6 Day 1 predose from patients receiving RO6958688 QWx3/Q3W.

Remaining plasma volumes of PD samples may also be used for assay validation during development of the study, for compound-related exploratory analyses, or to help develop further blood tests, after they are used for the mentioned intended uses.

4.5.1.6.3 Special Safety Assessments

Anti-Drug Antibodies

Although RO6958688 is a humanized antibody, there is a risk that ADA against RO6958688 may develop, potentially reducing its efficacy and/or potentially resulting in symptomatic hypersensitivity reactions, in particular immune-complex reactions. In this study, samples will be taken to assess the possible presence of ADA. The date and time of each sample will be recorded in the eCRF.

Serum samples for ADA determination will be obtained as specified in the Schedule of Assessment and Hourly Assessment tables (see Appendix 1). For ADA, approximately 1.0 mL of blood will be collected per timepoint. Additional samples will be drawn at the time of treatment discontinuation or during the safety follow-up visit and in patients who experience an IRR and in patients with clinical signs of hypersensitivity reaction, in particular immune-complex reactions. In any case, for each collected ADA sample, a corresponding PK sample will be collected at the same timepoint for the determination of the RO6958688 concentration.

Remaining volume of ADA samples may also be used for assay validation during development of the study, for compound-related exploratory analyses, or to help develop further blood tests, after being used for the mentioned intended uses.

Cytokine Release Assessment during IRR and CRS

For the assessment of cytokine release, serum or plasma samples (blood for pharmacodynamics and additional safety) will be collected at the time of an IRR/CRS (including repetitive occurrence of IRR/CRS). Cytokine analysis will include but is not

“Use of this document is governed by the terms of use on the first page of this document.”

limited to TNFα, IL-6, and IFNγ. In the event of an IRR/CRS, an additional blood sample will be collected (11 ml).

IgE and Tryptase

For patients who experience a Grade ≥2 IRR/CRS, tryptase and total IgE will be analyzed.

4.5.1.6.4 Disease-Specific Assessments

Tumor and Response Evaluations

Tumor response will be evaluated according to both RECIST v1.1 criteria and irRC with the use of unidimensional measurement (see Appendix 4 and Appendix 5, respectively). Assessment of CT/MRI scans as tumor assessments will be performed at the sites during the whole study. If Sponsor decides, independent central read for computed tomography (CT) or magnetic resonance imaging (MRI) could be considered in this study, both prospectively and retrospectively.

Response will be assessed by the investigator on the basis of physical examinations (CT scans or MRI) of chest, abdomen, and pelvis. Ultrasound and X-rays are not acceptable for monitoring target lesions. All measurable disease must be documented at screening and reassessed at each subsequent tumor evaluation. Consistency of consecutive CT scans or MRIs should be ensured during all assessments for each patient; the same method of assessment and the same technique must be used to evaluate lesions throughout the entire study. Use of spiral CT or MRI is required for baseline lesions <20 mm and must be documented in medical records and used consistently throughout the study. The same radiographic procedure that is used to define measurable disease sites at screening must be used throughout the study (e.g., the same contrast protocol for CT scans). Tumor measurements should be made by the same investigator/ radiologist for each patient during the study to the extent that this is feasible.

In case of clinically measurable superficial lesions such as skin lesions, repeated photographs should be used to document tumor response. These photos must include a ruler for documentation purposes.

If more than one method of assessment is used, the most accurate method is selected according to RECIST v1.1 and irRC, respectively, when recording data. At the investigator’s discretion, CT scans may be repeated at any time if progressive disease is suspected.

If available, an assessment of tumor growth kinetics will be made by comparing post-treatment scans with the last available prestudy scan.

Immune-related Response Criteria

irRC were developed from standard modified WHO response criteria because increasing clinical experience indicates that traditional response criteria (i.e., RECIST) may

“Use of this document is governed by the terms of use on the first page of this document.”

insufficiently evaluate response of immunotherapeutic agents. As described for ipilimumab, four distinct patterns of tumor response could be seen; all reported to be associated with favorable survival (Wolchok et al. 2009; Hoos et al. 2010; Nishino et al 2013; Nishino et al. 2014).

The four patterns of tumor response are:

- Type A: reduction in size of baseline lesions with no new lesions
- Type B: stable disease with no significant change in the size of the baseline lesions that may or may not be followed by a slow, steady decline in tumor size
- Type C: initial increase in tumor burden followed by response
- Type D: reduction in total tumor burden in spite of the appearance of new lesions.

For the irRC, anti-tumor response is based on total measurable tumor burden. At screening, index lesions that represent all involved organs will be identified and selected on the basis of size (i.e., lesions with longest diameter). All other sites of measurable (other than index lesions) and non-measurable disease will be identified as non-index lesions. During the study treatment period, all new measurable lesions will be taken into account. The index and the non-index lesions will also be evaluated, but changes in non-index lesions will only contribute to a complete response assessment.

Because of a delayed onset of tumor response that may be caused by immunotherapy, in addition to borderline progression, apparent radiologic progression with improving clinical status, or mixed responses, confirmation of progressive disease may be necessary. In the absence of clinical deterioration, any initial assessment of radiological progressive disease or mixed response should be confirmed by a repeat evaluation at the next timepoint for tumor assessment.

As described in Section 4.6, the criteria below are needed for continuation of treatment beyond the initial apparent progressive disease per RECIST v1.1 (e.g., radiological progression secondary to tumor inflammation):

- Absence of clinical deterioration and investigator–assessed potential clinical benefit for the patient and
- The patient is tolerating study drug.

Scheduling of Tumor Assessments

Tumor assessments will be performed once during screening for all patients. The first assessment after the start of treatment will be done at 8 weeks, and the subsequent assessments will continue every 8 weeks thereafter for the first year and every 12 weeks thereafter until disease progression or treatment discontinuation. All tumor assessments after baseline may be done ±7 days of the scheduled visit.

Baseline total tumor burden must be assessed within a maximum of 4 weeks before the first dose of study drug treatment. If performed within a reasonable time window prior to

“Use of this document is governed by the terms of use on the first page of this document.”

signature of the Informed Consent Form (ICF), the assessment can be accepted if agreed with the Sponsor and if done as SOC and according to RECIST v1.1 and irRC in a qualified facility in which all further scans for this patient will be performed. Post-baseline assessments must not be delayed (even if administration/cycle days are delayed) and are to be performed as indicated above until progression, unacceptable toxicity, or withdrawal of consent. The same radiographic procedure used to define measurable and/or evaluable disease at study entry must be used throughout the study. If there is a suspicion of disease progression based on clinical or laboratory findings before the next scheduled assessment, an unscheduled assessment should be performed.

Tumor response will be confirmed a minimum of 4 weeks after the initial response was noted or at the next scheduled tumor assessment if it is to occur more than 4 weeks after the initial response.

If a patient inadvertently misses a prescribed tumor evaluation or a technical error prevents the evaluation, the patient may continue treatment until the next assessment and an unscheduled tumor assessment should be planned as soon as possible.

In patients who are not in progression at time of study discontinuation, the same metastatic workup to cover all target and non-target lesions should be performed at the safety follow-up visit.

FDG-PET

[^{18}F]-Fluorodeoxyglucose (FDG) is a radioactive analogue of glucose which is taken up avidly in most malignancies and is used to visualize cancer and its metastases, usually as whole body images.

Patients who have had a hypersensitivity reaction to FDG will be excluded from FDG-PET. However, the patient will be eligible for the remainder of the study. Diabetic patients with high glucose levels (target blood glucose level $\leq$ 180 mg/dL) that can't be adequately controlled may be excluded from FDG-PET at the discretion of the investigator. PET involves exposure to ionizing radiation. The level of this exposure should be estimated and local guidelines for approval need to be followed.

[^{18}F]-FDG-PET will be performed according to the schedule of assessment as a non-invasive method for determining drug effect on the metabolic activity of tumor. The patient should fast for 4-6 hours prior to the [^{18}F]-FDG-PET investigation. The patient should rest after the tracer administration and scanning will start 60 minutes ± 10 minutes after tracer administration. The patient is examined with sufficient number of bed positions to ensure coverage over the area from the lower part of the brain (inclusive) to mid-thigh. It is also essential that the patient is examined under the same conditions in baseline and follow up scan, specifically with respect to uptake time

"Use of this document is governed by the terms of use on the first page of this document."

and scanning time. Additionally, it is important that the same model of scanner is used or at least that the same spatial resolution is ensured.

Blood glucose concentration needs to be measured immediately prior to the tracer injection and appropriate action needs to be taken for diabetic patients. The scanning procedure should be performed closely to NCI as described in Appendix 9.

[^{18}F]-FDG-PET will be performed at baseline prior to biopsy. It is important to be able to follow the biopsied lesion (selected target lesion as per RECIST) in the PET procedure for screening as well as on study visits. On-study FDG-PET assessment at Cycle 6 Day 5 (- 72/0 hours) for the QW schedule and Cycle 2 Day 19 (– 72/0 hours) for the Q3W schedule must be performed prior to the on-treatment biopsy and will be performed concurrent with RECIST assessment whenever possible as shown in the Schedule of assessments Appendix 1.

Patients with no evidence of FDG uptake at the screening PET assessment will not be required to undergo the on study assessment. For patients with suggestion of PD on follow-up FDG-PET assessments, a confirmatory CT/MRI assessment is strongly encouraged.

4.5.1.7 Samples for Roche Clinical Repository

Overview of the Roche Clinical Repository

The RCR is a centrally administered group of facilities for the long-term storage of human biologic specimens, including body fluids, solid tissues, and derivatives thereof (e.g., DNA, RNA, proteins, peptides). The collection and analysis of RCR specimens will facilitate the rational design of new pharmaceutical agents and the development of diagnostic tests, which may allow for individualized drug therapy for patients in the future.

RCR specimens from patients who give specific consent to participate in this optional research will be stored. These specimens will be used to achieve the following investigative purposes:

- To study the association of biomarkers with efficacy, adverse events, or disease progression
- To increase knowledge and understanding of disease biology
- To study drug response including drug effects and the processes of drug absorption and disposition
- To develop biomarker or diagnostic assays and establish the performance characteristics of these assays.

Approval by the Institutional Review Board or Ethics Committee

Sampling for the RCR is contingent upon the review and approval of the exploratory research and the RCR portion of the ICF by each site's IRB/Ethics Committee (EC) and,

“Use of this document is governed by the terms of use on the first page of this document.”

if applicable, an appropriate regulatory body. If a site has not been granted approval for RCR sampling, this section of the protocol will not be applicable at that site.

Sample Collection

The following samples will be collected for identification of genetic (inherited) biomarkers:

- Whole blood for extraction of DNA or RNA to examine possible predictive biomarkers, including but not limited to the following: variants in immune genes or other genes of interest, etc.

Patients will also have the option to consent that any tissue material remaining after protocol-defined analyses can be stored for up to 15 years in the RCR.

For all samples, dates of consent and specimen collection should be recorded on the associated RCR page of the eCRF. For sampling procedures, storage conditions, and shipment instructions, see the separate laboratory manual.

RCR specimens will be destroyed no later than 15 years after the date of final closure of the associated clinical database. The RCR storage period will be in accordance with the IRB/EC-approved ICF and applicable laws (e.g., health authority requirements).

The biomarker specimens will be subject to the confidentiality standards described in Section 8.4. The genetic biomarker specimens will undergo additional processes to ensure confidentiality as described below.

Confidentiality

Given the sensitive nature of genetic data, the Sponsor has implemented additional processes to ensure the confidentiality of patient for RCR specimens and associated data. Upon receipt by the RCR, each specimen is "double-coded" by replacing the study subject identification number with a new independent number. Data generated from the use of these specimens and all clinical data transferred from the clinical database and considered relevant are also labeled with this same independent number. A “linking key” between the study subject identification number and this new independent number is stored in a secure database system. Access to the linking key is restricted to authorized individuals and is monitored by audit trail. Legitimate operational reasons for accessing the linking key are documented in a Standard Operating Procedure. Access to the linking key for any other reason requires written approval from the Pharma Repository Governance Committee and Roche's Legal Department as applicable.

Data generated from RCR specimens must be available for inspection upon request by representatives of national and local health authorities and Roche monitors, representatives, and collaborators, as appropriate.

“Use of this document is governed by the terms of use on the first page of this document.”

Each patient's medical information associated with RCR specimens is confidential and may only be disclosed to third parties as permitted by the ICF (or separate authorization for use and disclosure of personal health information) signed by the patient unless permitted or required by law.

Data derived from RCR specimen analysis on individual patients will generally not be provided to study investigators unless a request for research use is granted. The aggregate results of any research conducted using RCR specimens will be available in accordance with the effective Roche policy on study data publication.

Any inventions and resulting patents, improvements, and/or know-how originating from the use of the RCR data will become and remain the exclusive and unburdened property of Roche except where agreed otherwise.

Consent to Participate in the Roche Clinical Repository

The ICF will contain a separate section that addresses participation in the RCR. The investigator or authorized designee will explain to each patient the objectives, methods, and potential hazards of participation in the RCR. Patients will be told that they are free to refuse to participate and may withdraw their specimens at any time and for any reason during the storage period. A separate, specific signature will be required to document a patient's agreement to provide optional RCR specimens. Patients who decline to participate will not provide a separate signature.

The investigator should document whether or not the patient has given consent to participate by completing the RCR Research Sample Informed Consent eCRF.

In the event of an RCR participant's death or loss of competence, the participant's specimens and data will continue to be used as part of the RCR research.

Withdrawal from the Roche Clinical Repository

Patients who give consent to provide RCR specimens have the right to withdraw their specimens from the RCR at any time for any reason. *After withdrawal of consent, any remaining samples will be destroyed or will no longer be linked to the patient. However, if RCR samples have been tested prior to withdrawal of consent, results from those tests will remain as part of the overall research data.* If a patient wishes to withdraw consent to the testing of his or her specimens, the investigator must inform the Medical Monitor in writing of the patient's wishes using the RCR Withdrawal Form and if the trial is ongoing, the date of withdrawal must be entered on the RCR Research Sample Withdrawal of Informed Consent eCRF.

If a patient wishes to withdraw consent to the testing of his or her RCR samples after closure of the site, the investigator must inform the Sponsor by emailing the study number and patient number to the following email address:

global_rcr-withdrawal@roche.com

"Use of this document is governed by the terms of use on the first page of this document."

A patient's withdrawal from Study BP29541 does not, by itself, constitute withdrawal of specimens from the RCR. Likewise, a patient's withdrawal from the RCR does not constitute withdrawal from Study BP29541.

Monitoring and Oversight

RCR specimens will be tracked in a manner consistent with Good Clinical Practice (GCP) by a quality-controlled, auditable, and appropriately validated laboratory information management system to ensure compliance with data confidentiality as well as adherence to authorized use of specimens as specified in this protocol and in the ICF. Roche's monitors and auditors will have direct access to appropriate parts of records relating to patient's participation in the RCR for the purposes of verifying the data provided to Roche. The site will permit monitoring, audits, IRB/EC review, and health authority inspections by providing direct access to source data and documents related to the RCR samples.

4.5.2 Timing of Study Assessments

4.5.2.1 Screening and Pretreatment Assessments

Written informed consent for participation in the study must be obtained before performing any study-specific screening tests or evaluations. ICFs for enrolled patients and for patients who are not subsequently enrolled will be maintained at the study site.

All screening and pretreatment assessments must be completed and reviewed to confirm that the patient meets all eligibility criteria. The investigator will maintain a screening log to record details of all screened patients and to confirm eligibility or record reasons for screening failure.

An Eligibility Screening Form, which documents the investigator's assessment of each screened patient with regard to the protocol's inclusion and exclusion criteria, is to be completed by the investigator and kept at the investigational site.

Screening and pretreatment assessments will be performed within 28 days prior to Cycle 1 Day 1 predose or to obinutuzumab if the patient is to be pretreated with obinutuzumab unless otherwise specified. Serum pregnancy test will be done up to 7 days prior to first dose of RO6958688 or obinutuzumab. Where the clinical significance of an abnormal screening test result (laboratory or any other tests) is considered uncertain, the test may be repeated. Baseline tumor assessments performed in a reasonable time window before ICF signature can be accepted if agreed with Sponsor and if done as SOC and according to RECIST v1.1 and irRC in a qualified facility in which all further scans for the patient will be performed.

Assessments will be performed during screening or before enrollment as specified in the Schedule of Assessment and Hourly Assessment tables (see Appendix 1).

"Use of this document is governed by the terms of use on the first page of this document."

The following will be assessed during baseline (after enrollment; before first dose) (see Schedule of Assessment and Hourly Assessment tables [see Appendix 1]):

- Informed consent
- Medical history, including demographics
- Complete physical examination (including height and weight) and ECOG performance status
- Vital signs (including oxygen saturation measured by pulse oximeter – finger clip at rest and with exercise (if required))
- Triplicate 12-lead ECGs
- Hematology
- Biochemistry
- Coagulation
- Urinalysis (dipstick)
- HBV and HCV, HIV and HTLV-1 serology for patients receiving obinutuzumab pre-treatment
- Serum pregnancy test (in women of child bearing potential)
- Primary archival tumor block (if available)
- Assessment of tumor CEA expression (refer to inclusion criterion #13)
- Tumor assessment
- FDG-PET
- Concomitant medications
- AEs
- Pulmonary function tests (FEV1/VC/TLC and DL_{CO} and derived parameters) for patients with bilateral lung metastasis or patients with lobectomy or pneumonectomy with lung metastases in the remaining lung
- For patients receiving Obinutuzumab pretreatment tumor biopsy can be obtained up to 14 days (+3/0 days) before first administration of RO6958688 but after the FDG-PET.
- Clinical genotyping, PK determination, ADA and PD blood serum or plasma samples, and safety assessments. Tumor biopsy (Part II) can be obtained up to 14 days (+3/0 days) before first administration of RO6958688 but after the FDG-PET.

4.5.2.2 Assessments during Treatment

Under no circumstances will patients who enroll in this study and have completed treatment as specified be permitted to be allocated a new enrollment number and re-enroll in the study.

All assessments must be performed per Schedule of Assessment and Hourly Assessment tables (see Appendix 1). Assessments scheduled on the day of study

"Use of this document is governed by the terms of use on the first page of this document."

treatment administration should be performed prior to administration of study treatment, unless otherwise noted in the Schedule of Assessment and Hourly Assessment tables. A 24-hour window (a 72-hour window if during weekend) prior to drug administration for hematology, biochemistry, coagulation, urinalysis, and physical examinations is allowed.

Assessments performed during treatment are specified in the Schedule of Assessment and Hourly Assessment tables (see Appendix 1).

For the following assessments, performed as indicated in the Schedule of Assessment and Hourly Assessment tables (see Appendix 1), results must be available before dosing:

- Physical examination (includes weight), vital signs (includes BP and heart rate), and weight assessment
- Triplicate 12-lead ECG (recording must be done prior to PK sampling)
- ECOG PS
- Hematology
- Biochemistry
- Coagulation
- Urinalysis

Note that patients enrolled in both parts of the study will be required to stay in the hospital overnight following administration of study drug on Cycle 1 Day 1.

If a Grade 3 or higher adverse event related to RO6958688 was observed within 24 hours of the previous RO6958688 infusion, an observation period of at least 8 hours (at least 24 hours for Grade 3 IRR/CRS) after completion of the subsequent RO6958688 infusion is required (for further details, see Appendix 1).

For further details for hematology, blood biochemistry, coagulation, and urinalysis, see Section 4.5.1.5.

4.5.2.3 Follow-Up Assessments and Assessments at Study Completion/Early Termination Visit

Patients who complete the study or discontinue from the study early will be asked to return to the clinic 28 days after the last dose of RO6958688 or obinutuzumab if for any reason the patient does not receive any RO6958688 infusion for a post-study follow-up visit. The visit at which response assessment shows progressive disease may be used as the study completion/early termination visit. Assessments should be completed as detailed in the Schedule of Assessment and Hourly Assessment tables (Appendix 1).

Post-Study Survival Follow-Up

Every 3 months, the sites will provide an update on survival status of each of the

“Use of this document is governed by the terms of use on the first page of this document.”

patients who are enrolled in the study to the Sponsor. The sites will use a designated section of the eCRF for this purpose.

4.6 PATIENT, STUDY, AND SITE DISCONTINUATION

4.6.1 Patient Discontinuation

Patients will be treated for 24 months (the treatment period may be modified if supported by emerging data) or until disease progression, loss of clinical benefit, unacceptable toxicities, loss of exposure (RO6958688 concentration BLQ or close to LLOQ of 0.925 ng/mL at end of infusion), or withdrawal from treatment for other reasons or death.

As with other immunotherapies, treatment beyond RECIST progression could be considered. The criteria below will be needed to continue treatment beyond initial apparent progressive disease per RECIST v1.1 (e.g., radiological progression secondary to tumor inflammation):

- Absence of clinical deterioration and investigator–assessed potential clinical benefit for the patient and
- The patient is tolerating study drug.

At the time of progression, patients can receive therapies such as: radiotherapy, radio-ablation or surgery.

The investigator has the right to discontinue a patient from RO6958688 or withdraw a patient from the study at any time. In addition, patients have the right to voluntarily discontinue study drug or withdraw from the study at any time for any reason. Reasons for discontinuation of study drug or withdrawal from the study may include but are not limited to the following*:*

- Patient withdrawal of consent at any time
- Any medical condition that the investigator or Sponsor determines may jeopardize the patient's safety if he or she continues in the study
- Investigator or Sponsor determines it is in the best interest of the patient.

All patients will attend a 28-day safety follow-up visit after receiving the last infusion of RO6958688 or obinutuzumab if for any reason the patient does not receive any RO6958688 infusion.

4.6.1.1 Discontinuation from Study Drugs

Patients must discontinue study drug if they experience any of the following:

- Pregnancy
- Symptomatic deterioration attributed to disease progression as determined by the investigator after integrated assessment of radiographic data, biopsy results, and clinical status

"Use of this document is governed by the terms of use on the first page of this document."

- Any medical condition that may jeopardize the patient's safety if he or she continues on study treatment
- IRR/CRS related to RO6958688 meeting any of the following criteria:
    - Grade 3 IRR/CRS that does not recover within 8 hours of corticosteroids and tocilizumab treatment *or following repeat treatment with tocilizumab for the same event*
    - Grade 4 IRR/CRS
    - Grade 3 IRR/CRS recurrence, or G3 IRR/CRS occurrence after previous G2 IRR/CRS
- AST or ALT increase >10 x ULN
- Grade 4 diarrhea, enteritis, or colitis related to RO6958688.
- Grade 2 diarrhea, enteritis, or colitis related to RO6958688 that fails to resolve to Grade 1 or better after following the recommended management guidelines in Table 7 for this grade
- Grade 3 diarrhea, enteritis, or colitis related to RO6958688 that fails to resolve to Grade 1 or better after following the recommended management guidelines in Table 7 for this grade.
- Grade 4 pulmonary event related to RO6958688
- Recurrent Grade 3 pulmonary event related to RO6958688
- Use of another non-protocol anti-cancer therapy (see Section 4.4.2)
- IgE–mediated hypersensitivity reactions, including anaphylaxis.

Patients who discontinue any study drug prematurely will be asked to return to the clinic for a study completion/early termination visit (see Section 4.5.2.3) and may undergo follow-up assessments (see Section 4.5.2.3). The primary reason for premature study drug discontinuation should be documented on the appropriate eCRF.

4.6.1.2 Withdrawal from Study

Every effort should be made to obtain information on patients who withdraw from the study. The primary reason for withdrawal from the study should be documented on the appropriate eCRF.

If a patient withdraws consent prior to the first treatment administration, the patient will be replaced. Patients will not be followed for any reason after consent has been withdrawn. Patients enrolled in Part I of the study will have a safety observation period for 14 days after infusion of study drug but will not be replaced.

During Part II of the study, patients who withdraw before the end of the DLT period (i.e., within 21 days of the first dose) for reasons other than DLTs will be replaced to ensure that at least 3 patients have been assessed for a full DLT period prior to moving

"Use of this document is governed by the terms of use on the first page of this document."

to the next dose level. Additionally, patients in the extension phase who discontinue treatment before evaluation of response will be replaced.

4.6.2 Study and Site Discontinuation

The Sponsor has the right to terminate this study at any time. Reasons for terminating the study may include but are not limited to the following:

- The incidence or severity of adverse events in this or other studies indicates a potential health hazard to patients
- Patient enrollment is unsatisfactory.

The Sponsor will notify the investigator and Health Authorities if the study is placed on hold or if the Sponsor decides to discontinue the study or development program.

The Sponsor has the right to replace a site at any time. Reasons for replacing a site may include but are not limited to the following:

- Excessively slow recruitment
- Poor protocol adherence
- Inaccurate or incomplete data recording
- Non-compliance with the International Conference on Harmonisation (ICH) GCP guideline.

5. ASSESSMENT OF SAFETY

5.1 SAFETY PARAMETERS AND DEFINITIONS

Safety assessments will consist of monitoring and recording adverse events, including serious adverse events and non-serious adverse events of special interest, measurement of protocol–specified safety laboratory assessments, measurement of protocol–specified vital signs, ECGs, and other protocol-specified tests that are deemed critical to the safety evaluation of the study.

Certain types of events require immediate reporting to the Sponsor, as outlined in Section 5.1.3.

5.1.1 Adverse Events

According to the ICH guideline for GCP, an adverse event is any untoward medical occurrence in a clinical investigation subject administered a pharmaceutical product, regardless of causal attribution. An adverse event can therefore be any of the following:

- Any unfavorable and unintended sign (including an abnormal laboratory finding), symptom, or disease temporally associated with the use of a medicinal product, whether or not considered related to the medicinal product
- Any new disease or exacerbation of an existing disease (a worsening in the character, frequency, or severity of a known condition), except as described in Section 5.3.5.9

“Use of this document is governed by the terms of use on the first page of this document.”

- Recurrence of an intermittent medical condition (e.g., headache) not present at baseline
- Any deterioration in a laboratory value or other clinical test (e.g., ECG, X-ray) that is associated with symptoms or leads to a change in study treatment or concomitant treatment or discontinuation from study drug
- Adverse events that are related to a protocol-mandated intervention, including those that occur prior to assignment of study treatment (e.g., screening invasive procedures, such as biopsies).

5.1.2 Serious Adverse Events (Immediately Reportable to the Sponsor)

A serious adverse event is any adverse event that meets any of the following criteria:

- Fatal (i.e., the adverse event actually causes or leads to death)
- Life threatening (i.e., the adverse event, in the view of the investigator, places the patient at immediate risk of death)

This does not include any adverse event that, had it occurred in a more severe form or was allowed to continue, might have caused death.

- Requires or prolongs inpatient hospitalization (see Section 5.3.5.10)
- Results in persistent or significant disability/incapacity (i.e., the adverse event results in substantial disruption of the patient's ability to conduct normal life functions)
- Congenital anomaly/birth defect in a neonate/infant born to a mother exposed to study drug
- Significant medical event in the investigator's judgment (e.g., may jeopardize the patient or may require medical/surgical intervention to prevent one of the outcomes listed above).

The terms "severe" and "serious" are not synonymous. Severity refers to the intensity of an adverse event (rated as mild, moderate, or severe), or according to a pre-defined grading criterion (e.g., NCI CTCAE criteria; see Section 5.3.3); the event itself may be of relatively minor medical significance (such as severe headache without any further findings).

Severity and seriousness need to be independently assessed for each adverse event recorded on the eCRF.

Serious adverse events are required to be reported by the investigator to the Sponsor immediately (i.e., no more than 24 hours after learning of the event; see Section 5.4.2 for reporting instructions).

"Use of this document is governed by the terms of use on the first page of this document."

5.1.3 Non-Serious Adverse Events of Special Interest (Immediately Reportable to the Sponsor)

Non-serious adverse events of special interest are required to be reported by the investigator to the Sponsor immediately (i.e., no more than 24 hours after learning of the event; see Section 5.4.2 for reporting instructions). Adverse events of special interest for this study include the following:

1. Related to RO6958688

- Cases of an elevated ALT or AST in combination with either an elevated bilirubin or clinical jaundice, as defined in Section 5.3.5.6
- Grade ≥ 2 CRS and IRR
- Grade ≥ 2 hypersensitivity
- Suspected transmission of an infectious agent by the study drugs, as defined below:
    - Any organism, virus, or infectious particle (e.g., prion protein transmitting transmissible spongiform encephalopathy), pathogenic or non-pathogenic, is considered an infectious agent. A transmission of an infectious agent may be suspected from clinical symptoms or laboratory findings that indicate an infection in a patient exposed to a medicinal product. This term applies only when contamination of the study drugs is suspected.

2. Related to obinutuzumab

- Cases of an elevated ALT or AST in combination with either an elevated bilirubin or clinical jaundice, as defined in Section 5.3.5.6
- Suspected transmission of an infectious agent by the study drugs, as defined below:
- Any organism, virus, or infectious particle (e.g., prion protein transmitting transmissible spongiform encephalopathy), pathogenic or non-pathogenic, is considered an infectious agent. A transmission of an infectious agent may be suspected from clinical symptoms or laboratory findings that indicate an infection in a patient exposed to a medicinal product. This term applies only when a contamination of the study drugs is suspected.
- Tumour lysis syndrome of any grade, irrespective of causality.
- Second malignancies for patients who have received obinutuzumab pre-treatment.

5.2 SAFETY PLAN

5.2.1 Risks associated with RO6958688

The following adverse events are classified as identified risks associated with RO6958688: pyrexia, IRR, CRS, hypoxia, hypotension, diarrhea, colitis, tumor inflammation/tumor flare events at the tumor site (tumor pain, dyspnea, hypoxia, increased liver enzymes (AST/ALT), blood bilirubin increased, colitis, enteritis), chills, vomiting, rash, skin exfoliation, nausea, dysgeusia, fatigue, arthritis and arthralgia.

"Use of this document is governed by the terms of use on the first page of this document."

Serious adverse drug reactions which are considered expected and associated with RO6958688 are: pyrexia, IRR, CRS, tumor pain, diarrhea, colitis, dyspnea, hypoxia, hypotension, AST increased, ALT increased and blood bilirubin increased.

The following adverse events are classified as potential risks associated with RO6958688: asthenia, decreased appetite, IgE-mediated hypersensitivity reactions including anaphylaxis, hematotoxicities, and CNS disorders.

An observation period of at least 8 hours is required following completion of the RO6958688 infusion in any treatment administration visit where a Grade 3 or higher adverse event (with the exception of Grade 3 IRR/CRS) was observed within 24 hours of the previous RO6958688 administration. In a situation where the patient experienced a Grade 3 IRR/CRS in the previous administration visit, a 24-hour hospitalization will be required following the completion of RO6958688 infusion at the next administration visit.

For detailed information regarding identified and potential risks associated with RO6958688, refer to Section 6 of the most recent version of the RO6958688 Investigator's Brochure.

For management of specific adverse events, refer to Section 5.2.5.1.

5.2.2 Risks associated with Obinutuzumab

The following adverse events are considered as important risks associated or potentially associated with obinutuzumab: IRRs, TLS, thrombocytopenia including acute thrombocytopenia, neutropenia including prolonged and late onset neutropenia, prolonged B-cell depletion, infections including PML and hepatitis B reactivation, worsening of preexisting cardiac conditions, impaired immunization response, immunogenicity, GI perforation and second malignancies. The most frequently observed adverse drug reactions in patients receiving obinutuzumab were IRR.

For detailed information regarding identified and potential risks associated with Obinutuzumab, refer to Section 6 of the most recent version of the Obinutuzumab Investigator's Brochure.

5.2.3 *Risks associated with Tocilizumab*

Refer to Section 6 of the Tocilizumab Investigator's Brochure for a detailed description of anticipated safety risks for tocilizumab. Because the use of tocilizumab remains experimental in this protocol, it should be noted that the risks contained in the Tocilizumab Investigator's Brochure have been identified in patients treated in alternate settings and for different indications.

"Use of this document is governed by the terms of use on the first page of this document."

5.2.4 Dose Modifications and Delays

The RO6958688 dose should not be escalated beyond 150 mg in any cohorts of the study. Patients who are already at doses higher than 150 mg should not be escalated any further, but can stay at their current RO6958688 dose levels following discussion with the Medical Monitor if the therapy is adequately tolerated.

Should a patient experience a DLT and/or grade ≥ 2 related adverse events in the first or in subsequent cycles or experience the same toxicity of same or higher grade following re-exposure to RO6958688, the investigator, after discussion with the Sponsor, will have the option to reduce the dose of RO6958688 to a lower dose level or to allow for a more convenient dose/schedule to prevent the recurrence of adverse events and limit toxicity. This can be done to allow patients, who could potentially benefit from RO6958688, to remain on the study drug.

Prior to dosing (Day 1 at each cycle), toxicities from previous administration should be resolved in individual patient:

- For treatment-related non-hematological blood parameters abnormalities the values of AST, ALT, bilirubin, total albumin, creatinine, GGT, must be resolved to screening value, Grade 1, or normal value (AST and/or ALT must be resolved to $\leq 3 \times$ ULN).
- Regarding fever, patients can continue treatment with RO6958688 if fever is Grade 1 or normal value.
- Other non-hematological toxicities should be resolved to baseline values or Grade ≤ 1 (or Grade ≤ 2 if considered non-clinically significant by the investigator).
- For hematological blood parameters the blood values of thrombocyte, erythrocyte, and combination of erythrocyte, lymphocyte and thrombocyte must be resolved to screening baseline values or as defined by NCI-CTCAE Grade ≤ 2.

A delay of RO6958688 administration for up to 14 days in the QW phase and for up to 21 days in the Q3W phase will be acceptable to allow for resolution of toxicity NCI CTCAE as described above (with the exception of toxicity considered as non-RO6958688 related). No other delays of RO6958688 administration are foreseen.

Dose interruptions for reason(s) other than toxicity, such as surgical procedures, may be allowed with Medical Monitor approval. The acceptable length of interruption will depend on agreement between the investigator and the Medical Monitor.

It should be noted that in case of a dose delay, the infusions/cycles are not considered as missed but as delayed (visits should be followed as per SoA). In case the delay is ≥ 2 weeks the investigator should contact the medical monitor.

For patients participating in the QWx3 – Q3W step up cohorts C-G who experience Grade 3 related adverse events associated with tumor inflammation:

- For patients who are in the step-up phase of the QWx3 – Q3W dosing scheme, patient management should follow the guidelines provided in Table 6, Table 7, and

"Use of this document is governed by the terms of use on the first page of this document."

Table 8; the event should be resolved to Grade 1 prior to resuming RO6958688 treatment. The next RO6958688 dose for such patients will be up to or at the same level of RO6958688 dose that caused the event in the dose escalation phase.

- For patients who have reached the flat dose phase (Q3W), the event should be managed as per the guidelines provided in Table 6, Table 7, and Table 8 and should be resolved to Grade 1 prior to resuming RO6958688 treatment, and the patient should continue with the per-protocol planned dose. However, the Investigator, after discussion with the Sponsor, will have the option to reduce the dose of RO6958688 to a lower dose level or to allow for a more convenient dose/schedule to prevent the recurrence of adverse events and limit toxicity.

For patients participating in the step up cohorts who experience Grade 2 IRR/CRS considered related to RO6958688:

For patients who are in the step-up phase of the QW – Q3W dosing scheme, patient management should follow the guidelines provided in Table 10 *(IRR/CRS management table)*.

- The next RO6958688 dose for such patients will be up to or at the same level of the RO6958688 dose that caused the event in the dose escalation phase.

- For patients who have reached the flat dose phase (Q3W), patient management should follow the guidelines provided in Table 10 *(IRR/CRS management table)*. The Investigator, after discussion with the Sponsor, will have the option to reduce the dose of RO6958688 to a lower dose level or to allow for a more convenient dose/schedule to prevent the recurrence of adverse events and limit toxicity.

Further dose reductions may be implemented once safety and toxicity data from the Part II dose escalation have been evaluated.

5.2.5 Management of Specific Adverse Events

5.2.5.1 Management of Specific Adverse Events related to RO6958688

Refer to the RO6958688 Investigator's Brochure for additional information on the clinical safety of RO6958688.

5.2.5.1.1 Management of Pyrexia

Investigators should provide guidance to patients for the management of isolated (no other signs and symptoms) episodes of fever, in particular patients should regularly check their body temperature during the days following each RO6958688 administration and take early intervention with standard anti-pyrexia treatments (e.g., paracetamol, NSAIDs) as current practice. Occasionally, fever is accompanied with symptoms, such as chills, hypotension, shortness of breath, skin rash, headache, nausea, and/or vomiting, in which case this is considered an IRR/CRS and should be managed with urgency (see Table 10 below).

"Use of this document is governed by the terms of use on the first page of this document."

5.2.5.1.2 Adverse Events Associated with Tumor Inflammation/Tumor Flare

Clinical evidence suggests that certain types of adverse events related to RO6958688 may be mediated by its mechanism of action at the tumor site (i.e., intratrumor immune activation and consequent tumor inflammation and flare). These adverse events tend to have a short onset following RO6958688 administration and affect organ systems in and around the location of the tumor lesions.

Tumor inflammation and tumor flare events are either associated with pain at the tumor site (e.g., tumor, abdominal, and GI pain) or affect function of the organ system with the tumor lesion (e.g., dyspnea, hypoxia, colitis, enteritis, and increased levels of liver function enzymes and bilirubin). These adverse events are more common after the first or second dose of RO6958688.

Guidelines for management of patients who experience adverse events associated with tumor inflammation and tumor flare are provided in Table 6 (pulmonary events), Table 7 (gastrointestinal events), and Table 8 (hepatotoxic events).

"Use of this document is governed by the terms of use on the first page of this document."

Table 6 Guidelines for Management of Pulmonary Events

Event	Action to Be Taken
**Pulmonary event, including dyspnea and hypoxia [a, b, c]**	
Grade 1	• Continue RO6958688.<br>• Monitor patient closely.
Grade 2	• Withhold RO6958688 for up to 14 days in the QW dosed cohorts and for up to 21 days in the Q3W dosed cohorts after last administered dose.<br>• Monitor patient closely.<br>• If event resolves to Grade 1 or better, resume RO6958688.<br>• For recurrent events, treat as a Grade 3 event.
Grade 3 [d]	• Withhold RO6958688 for up to 14 days in the QW dosed cohorts and for up to 21 days in the Q3W dosed cohorts after last administered dose, until resolution to Grade 1.<br>• Treat with IV or oral methylprednisolone 2 mg/kg/day or equivalent. [e]<br>• If event resolves to Grade 1 or better, resume RO6958688.<br>• For recurrent events, treat as a Grade 4 event.
Grade 4 [d]	• Permanently discontinue RO6958688.<br>• Treat with IV or oral methylprednisolone 2 mg/kg/day (or equivalent) until resolution to Grade 1 or better. [e]<br>• If event does not improve within 48 hours after initiating corticosteroids, consider adding an immunosuppressive agent.

[a] Pulmonary events include respiratory toxicities (e.g., dyspnea and hypoxia).
[b] For hypoxia, exclude CRS etiologies before following these guidelines.
[c] Provide supportive measures (e.g., oxygen support and intubation), as clinically indicated.
[d] Ensure patient has access to an ICU.
[e] If corticosteroids are administered for longer than 3 weeks, taper before stopping treatment.

5.2.5.1.3 Management of Gastrointestinal events: Diarrhea and Colitis

Diarrhea has been commonly observed in patients receiving RO6958688. Diarrhea events are usually transient and reversible. Diarrhea can be one of the manifestations of cytokine-release syndrome, and dehydration secondary to diarrhea can worsen the hypotension associated with cytokine-release syndrome. Patients should be closely monitored (including monitoring of renal function) and should be hydrated if clinically indicated to prevent renal insufficiency due to fluid depletion. Diarrhea can also be a symptom of undiagnosed colitis. All events of diarrhea or colitis should be thoroughly evaluated for other etiologies.

Guidelines for management of patients who experience diarrhea and colitis are provided in Table 7. Please consider use of tocilizumab per cytokine-release syndrome management guidelines (Table 10) if per investigator clinical judgement diarrhea is deemed secondary to cytokine-release syndrome.

“Use of this document is governed by the terms of use on the first page of this document.”

Table 7 Recommendations for management of gastrointestinal events considered related to RO6958688

Event	Action to Be Taken
**Gastrointestinal events** [a]	
Diarrhea, enteritis or colitis, Grade 1	• Continue RO6958688 and monitor patient closely.<br>• Initiate standard-of care-symptomatic treatment with anti-diarrheal agents (e.g., loperamide) [b].<br>• If diarrhea does not improve within 48 hours of anti-diarrheal agent treatment, initiate corticosteroids at a dose of 1 mg/kg/day IV or oral methylprednisolone (or equivalent). [c]<br>• If diarrhea does not improve within 48 hours of corticosteroid treatment, increase dose to 2 mg/kg/day IV or oral methylprednisolone (or equivalent).
Diarrhea, enteritis or colitis, Grade 2	• Withhold RO6958688 for up to 14 days in the QW dosed cohorts and for up to 21 days in the Q3W dosed cohorts after last administered dose and monitor patient closely.<br>• Initiate standard-of care-symptomatic treatment with anti-diarrheal agents (e.g., loperamide) [b].<br>• If diarrhea does not improve within 48 hours of anti-diarrheal agent treatment, initiate corticosteroids at a dose of 1 mg/kg/day IV or oral methylprednisolone (or equivalent). [c]<br>• If diarrhea does not improve within 48 hours of corticosteroid treatment, increase dose to 2 mg/kg/day IV or oral methylprednisolone (or equivalent).<br>• Consider rectoscopy/colonoscopy with biopsy if symptoms of diarrhea persist for > 48 hours.<br>• Patient referral to GI specialist is recommended.<br>• If event resolves to Grade 1 or better, resume RO6958688.<br>• If event does not resolve to Grade 1 or better while withholding RO6958688, permanently discontinue and contact Medical Monitor.
Diarrhea, enteritis or colitis, Grade 3	• Withhold RO6958688 for up to 14 days in the QW dosed cohorts and for up to 21 days in the Q3W dosed cohorts after last administered dose and monitor patient closely.<br>• Initiate corticosteroids at a dose of 2 mg/kg/day IV or oral methylprednisolone (or equivalent).<br>• Refer patient to GI specialist for evaluation and confirmatory biopsy.<br>• If event resolves to Grade 1 or better, resume RO6958688.<br>• If event does not resolve to Grade 1 or better within 24 hours, treat as Grade 4.

"Use of this document is governed by the terms of use on the first page of this document."

Event	Action to Be Taken
**Gastrointestinal events** [a]	
Diarrhea, enteritis or colitis, Grade 4	• Permanently discontinue RO6958688 and contact medical monitor.<br>• Follow all Grade 3 management guidelines.<br>• Consider adding an immunosuppressive agent if diarrhea does not improve within 48 hours of initiating corticosteroids. [d]

Note: If diarrhea occurs in context of CRS please follow IRR/CRS management guidelines (Table 10).

[a] Exclude infectious and other etiologies of diarrhea/colitis before executing these management guidelines.

[b] Loperamide treatment should begin at an initial dose of 4 mg. After each loose stool, the dose may be increased by 4 mg to a maximum of 16 mg/day.

[c] If corticosteroids are administered for longer than 3 weeks, taper before stopping treatment.

[d] Patient must have a rectoscopy/colonoscopy prior to treatment with immunosuppressive agent.

5.2.5.1.4 Elevated Liver Enzymes and Hepatotoxicity

Transient AST and ALT elevations have been observed in patients receiving RO6958688 and could occur in the setting of tumor inflammation/tumor flare. These events tend to occur predominantly after the first two infusions of RO6958688. AST and ALT elevations that occur after the third infusion (or later) are regarded as a consequence of cytokine-release syndrome.

Please use guidelines in Table 8 to manage AST/ALT elevation secondary to tumor inflammation and Table 10 to manage AST/ALT elevation occurring as a consequence of CRS. Table 8 below provides guidance for monitoring of patients who develop increase AST/ALT while on RO6958688.

“Use of this document is governed by the terms of use on the first page of this document.”

Table 8 Monitoring of patients with increased AST/ALT while on RO6958688

Event	Action to Be Taken
**AST/ALT abnormality**	
AST/ALT ULN-3 × ULN	• Monitor AST/ALT weekly.<br>• Continue RO6958688 with the above monitoring.
AST/ALT > 3 × ULN to < 5 × ULN	• Withhold RO6958688 for up to 14 days in the QW dosed cohorts and for up to 21 days in the Q3W dosed cohorts after last dose until resolution to < 3xULN.<br>• Monitor LFTs every 24–48 hours until resolution to AST/ALT ≤ 3 × ULN, then monitor weekly.<br>• If no improvement to AST/ALT ≤ 3 × ULN within 48 hours, administer 1 mg/kg/day of IV or oral methylprednisolone or equivalent [b]<br>• If no improvement to AST/ALT ≤ 3 × ULN within 48 hours of treatment with 1 mg/kg/day corticosteroid, increase dose to 2 mg/kg/day IV or oral methylprednisolone or equivalent. [b]<br>• Consider patient referral to hepatologist.
AST/ALT > 5 x ULN to < 10 × ULN	• Withhold RO6958688 for up to 14 days in the QW dosed cohorts and for up to 21 days in the Q3W dosed cohorts after last dose until resolution to < 3xULN.<br>• Monitor AST/ALT every 24–48 hours until resolution to AST/ALT ≤ 3 × ULN, then monitor weekly. [a]<br>• Consider referral for hepatology specialist for consultation.<br>• Administer 2 mg/kg/day of IV or oral methylprednisolone [b] or equivalent until improvement to AST/ALT ≤ 3 × ULN<br>• If event improves to AST/ALT ≤ 3 × ULN or better, resume RO6958688
AST/ALT > 10 × ULN	• Monitor AST/ALT every 24–48 hours until resolution to AST/ALT ≤ 3 × ULN, then monitor weekly. [a]<br>• Administer 2 mg/kg/day of IV or oral methylprednisolone [b] or equivalent until improvement to AST/ALT ≤ 3 × ULN<br>• Refer for hepatology specialist consultation and consider liver biopsy to assess hepatic injury.<br>• Permanently discontinue RO6958688, if the LFT elevation is considered related to RO6958688.

ULN = upper limit of normal.
[a] Monitoring should also include bilirubin.
[b] If corticosteroids are administered for longer than 3 weeks, taper before stopping treatment.

5.2.5.1.5 Management of IRR/CRS

Administration of RO6958688 may cause a spectrum of infusion-related adverse events involving IRRs and CRS. The incidence and severity typically decrease with subsequent infusions, although instances of CRS have been reported in late cycles, as in the patient described below.

"Use of this document is governed by the terms of use on the first page of this document."

Given the overlap in signs and symptoms, IRRs may be indistinguishable from CRS, which is defined as a disorder characterized by fever, tachypnea, headache, tachycardia, hypotension, rash, and/or hypoxia caused by the release of cytokines (NCI CTCAE v5). Severe CRS may be associated with other clinical sequelae such as disseminated intravascular coagulation and capillary leak syndrome.

In Study BP29541, as of 23 March 2018, there was one case of Grade 5 IRR that met the criteria of CRS based on symptomatology and cytokine levels, as described below:

- A [redacted] year old [redacted] patient enrolled in study BP29541 was reported to have suffered a Grade 5 IRR following the fifth administration of RO6958688 at a dose of 355.99 mg (planned dose 600 mg). The patient is suspected to have had a fatal CRS event following multiple re-treatment attempts with RO6958688 based on a very high serum IL-6 level of 75198.41 pg/mL, elevated levels for IFN-γ (1694.96 pg/mL), IL-8 (4328.17 pg/mL) and TNF-α (767.06 pg/mL) after the fourth administration, with a very similar clinical course at the fifth administration of RO6958688 (Cycle 5 cytokine levels will not be available). This patient had a recurring symptom of Grade 3 hypotension related to RO6958688 infusion at both administrations. Following this Grade 5 event, safety management guidelines were updated via a Dear Investigator Letter (issued on 16 Feb 2018) and an Urgent Safety Measure (issued on 6th April 2018). In view of the above data, and given that IRRs may be indistinguishable from CRS based on symptomatology, single treatment guidelines are being recommended for management of IRRs and CRS, during or up to 24 hours after infusion of RO6958688 (Table 10).

Table 9 Cytokine Release Syndrome grading according to NCI CTCAE v5

Grade 1	Grade 2	Grade 3	Grade 4	Grade 5
Fever with or without constitutional symptoms*	Hypotension responding to fluids; hypoxia responding to <40% O2	Hypotension managed with one pressor; hypoxia requiring ≥ 40% O2	Life-threatening consequences; urgent intervention indicated	Death
Definition: A disorder characterized by fever, tachypnea, headache, tachycardia, hypotension, rash, and/or hypoxia caused by the release of cytokines.				
Navigational Note: Also consider reporting other organ dysfunctions including neurological toxicities such as: Psychiatric disorders: Hallucinations or Confusion; Nervous system disorders: Seizure, Dysphasia, Tremor, or Headache				

* e.g. rigors, malaise, fatigue, anorexia, myalgia, arthralgia, nausea, vomiting and headache

The risk of IRRs/CRS is managed in the clinic with appropriate risk minimization measures. RO6958688 should only be administered under the close supervision of an experienced clinician trained to monitor medical situations and to respond to medical

“Use of this document is governed by the terms of use on the first page of this document.”

emergencies in a clinical environment with full resuscitation equipment available for immediate use. Patients should receive full supportive care to treat IRRs/CRS according to institutional practice. In the clinical studies with RO6958688, patients are required to stay overnight in the hospital after the first dose for clinical monitoring. If infusion-associated signs or symptoms or CRS manifestations occur, patients should be monitored until complete resolution. If an IRR/CRS develops during the administration of RO6958688, the infusion should be temporarily slowed down or interrupted. Treatment or concomitant medication may include acetaminophen/paracetamol, antihistamine, IV saline, oxygen, bronchodilators, corticosteroids, and vasopressors and tocilizumab depending on the symptoms and the severity. Resuscitation equipment should be available for immediate use.

In addition, to reduce the risk of the development of IRRs/CRS in a patient receiving RO6958688, premedication will be administered as per section 4.3.2.2.

In a situation where the patient experienced a Grade 3 IRR/CRS in the previous administration visit, a 24-hour hospitalization will be required at the completion of the subsequent treatment administration.

Please refer to section 5.3.5.1 for adverse event reporting procedures related to IRRs and CRS. Refer to Table 9 for CRS grading scale.

Please note that infusion-related reactions (i.e., infusion-related events associated with the first and second RO6958688 infusion without hypotension or hypoxia) and cytokine-release syndrome events (i.e., infusion-related events occurring within 24 hours of the RO6958688 infusion with hypotension or hypoxia and all infusion-related events associated with the third infusion of RO6958688 or later, irrespective of associated symptoms) should both be managed using the guidance in Table 10.

Infusion-related reactions (regardless of grade) without hypotension or hypoxia should be managed according to the Grade 1 cytokine-release syndrome guidelines in Table 10

In patients with lung metastasis, hypoxia could also be secondary to tumor inflammation after the first and second dose of RO6958688. In such situations, please consider further evaluation with radiological imaging per clinical judgment, and if there is no associated hypotension, please follow guidelines in Table 6.

“Use of this document is governed by the terms of use on the first page of this document.”

Table 10 Recommendations for Management of Infusion-Related Reactions and Cytokine Release Syndrome related to RO6958688[a]

Symptoms[b]	Guidance [g]
Grade 1 CRS defined as fever, constitutional symptoms* (includes first and second dose IRRs with no hypotension/hypoxia	Immediate actions:<br>- If RO6958688 infusion is still ongoing, slow the infusion rate by ≤50% or interrupt the infusion.<br>- Treat with antihistamines, antipyretics, and/or analgesics as clinically indicated.<br>- Monitor fluid balance and administer IV fluids as clinically indicated.<br>Restarting infusion:<br>- If RO6958688 infusion was interrupted, wait until 30 minutes after the event has resolved before restarting the infusion at 50% of the original infusion rate.<br>Next cycle:<br>- Pretreat with antihistamines, antipyretics/NSAIDS, and antiemetic medication as per the guidelines described for the first dose of RO6958688

"Use of this document is governed by the terms of use on the first page of this document."

Symptoms[b]	Guidance [g]
Grade 2 CRS defined as <u>hypotension</u> (responds to fluids) OR <u>hypoxia</u> (requires <40% FiO_2 to maintain adequate hemoglobin oxygen saturation)	Immediate actions:<br>• If RO6958688 infusion is ongoing, stop the infusion immediately.<br>• Treat with antihistamines, antipyretics, and/or analgesics as clinically indicated.<br>• Consider treatment with IV corticosteroids (methylprednisolone 2 mg/kg/day or, if neurologic symptoms are present, dexamethasone 10 mg).<br>• Consider administering a single dose of tocilizumab IV 8 mg/kg (if administered, see Appendix 10).<br>• Monitor cardiac and other organ functions closely.<br>• Provide hemodynamic support as clinically indicated.<br>• Provide oxygen for hypoxia and fluids for hypotension.<br>• Collect all samples (including a sample for the cytokine panel) as described in Appendix 10.<br>• Admit to ICU if clinically indicated and consider 24-hour hospitalization.<br>• If there is no improvement of hypotension or hypoxia within 24 hours, manage as a Grade 3 event.<br>• Notify the Medical Monitor.<br>Restarting infusion:<br>• Wait until 30 minutes after the event has resolved before restarting the infusion at ≤25% of the original infusion rate.<br>• If symptoms recur, the infusion must be stopped immediately and RO6958688 should not be administered (re-started) again this cycle.<br>• If hypotension or hypoxia recurs, manage as a Grade 3 event.<br>Next cycle:<br>• Patient may receive RO6958688 if symptoms resolve to Grade ≤1 and with approval of the Medical Monitor, as follows:<br>– Pretreat with antihistamines, antipyretics and/or analgesics as clinically indicated<br>– Pretreat with IV corticosteroids (methylprednisolone 80 mg or dexamethasone 20 mg) at least 60 minutes prior to the administration of RO6958688). [b]<br>– Administer RO6958688 at 50% of the infusion rate of the previous cycle.<br>Subsequent cycles:<br>• If there is an occurrence of IRR or CRS Grade ≥3 in any of the subsequent cycles, permanently discontinue RO6958688 regardless of recovery (see Grade 3 management guidelines)<br>- If there is an occurrence of a Grade ≤2 CRS in subsequent cycles, manage as indicated by severity (see Grade 1 or Grade 2 management guidelines).

"Use of this document is governed by the terms of use on the first page of this document."

Symptoms[b]	Guidance [g]
Grade 3 CRS defined as hypotension (managed with one vasopressor [see Appendix 11 for definitions and calculations]) OR hypoxia (requires ≥40% FiO_2 to maintain adequate hemoglobin oxygen saturation)	Immediate actions:<br>- If RO6958688 infusion is ongoing, stop the infusion immediately.<br>- Treat with antihistamines, antipyretics, and/or analgesics as clinically indicated.<br>- Treat with IV corticosteroids (methylprednisolone 2 mg/kg/day or, if neurologic symptoms are present, dexamethasone 10 mg).<br>- Administer tocilizumab IV 8 mg/kg (see Appendix 10).<br>○ If there is no improvement after 24 hours, repeat tocilizumab administration (see Appendix 10).<br>- Closely monitor and maintain fluid balance and administer IV fluids as clinically indicated.<br>- Provide oxygen for hypoxia and fluids for hypotension.<br>- Provide vasopressor support for hypotension with high and repeated doses if required (see Appendix 11).<br>- Hospitalize patient for 24 hours.<br>- Perform a work up for organ functions (e.g., liver, cardiac) based on clinical assessment of the investigator.<br>- Strongly consider cardiopulmonary and organ function monitoring in ICU.<br>- Collect all samples (including a sample for the cytokine panel) as described in Appendix 10.<br>- Notify the Medical Monitor.<br>Restarting Infusion:<br>- RO6958688 should not be administered again during this cycle.<br>Next Cycle:<br>- If the patient had a Grade 2 IRR or CRS in any previous cycle, permanently discontinue RO6958688.<br>- If patient does not recover (is febrile and/or still on vasopressors) within 8 hours after corticosteroid and tocilizumab treatment, permanently discontinue RO6958688.<br>- If patient recovers (is afebrile and off vasopressors) within 8 hours following corticosteroid and tocilizumab treatment, they can be dosed in next cycle, as follows:<br>- Pretreat with antihistamines, antipyretics and/or analgesics as clinically indicated<br>- Pretreat with IV corticosteroids (methylprednisolone 80 mg or dexamethasone 16 mg) at least 60 minutes prior to the administration of RO6958688). [b]<br>- Hospitalize patient for 24 hours.<br>- Collect all samples (including a sample for the cytokine panel) as described in Appendix 10.<br>- Administer RO6958688 at 50% of the infusion rate of the previous cycle.<br>Subsequent cycles:<br>- If a Grade ≥3 CRS recurs, permanently discontinue RO6958688.<br>- If there is an occurrence of a Grade ≤2 CRS in subsequent cycles, manage as indicated by severity (i.e. Grade 1 or Grade 2 management guidelines).

“Use of this document is governed by the terms of use on the first page of this document.”

Symptoms[b]	Guidance [g]
Grade 4 CRS defined as life threatening consequences; urgent intervention indicated (multiple vasopressors)	• Permanently discontinue RO6958688.<br>• Manage as a Grade 3 event

* e.g. rigors, malaise, fatigue, anorexia, myalgia, arthralgia, nausea, vomiting and headache

a- Guidance for IRR and CRS management based on CRS grading in CTCAE V5

b- Refer to CTCAE v5 for complete description of grading of symptoms

c- In patients with lung metastasis, hypoxia could be secondary to tumor inflammation following 1st & 2nd dose of RO6958688, please manage per tumor inflammation guideline

d- Refer to Appendix 10 for tocilizumab schedule of assessments

e- Please perform all laboratory assessment, including cytokines as per cycle 1 Day 1 visit. For cardiac work-up, also consider echocardiogram if clinically indicated.

f- Refer to Appendix 11 for a description and calculation of high dose vasopressors

g- Corticosteroid premedication guidelines refer only to the first cycle following the event; if an IRR/CRS event has occurred in the previous cycle please refer to the table above for corticosteroid dosing guidelines. Pre-medication is not required for subsequent cycles if no event has occurred in the previous cycle. For all other pretreatments please follow pre-medication guidance in section 4.3.2.2.

5.2.5.1.6 Management of IgE-mediated Hypersensitivity Reactions Including Anaphylaxis

Patients may also develop IgE-mediated hypersensitivity reactions to RO6958688. The signs and symptoms of IRRs/CRS may be indistinguishable from an anaphylactic reaction. Medications including epinephrine, corticosteroids, diphenhydramine hydrochloride for IV injection, and resuscitation equipment should be available for immediate use. Guidelines for management of IRRs/CRS related to RO6958688 are summarized in Table 10.

Patients with IRRs/CRS of Grade 2 or higher should undergo special laboratory assessments including IgE and tryptase analysis *(Appendix 1, Table A9)*. If the results support an allergic origin (i.e., increased tryptase levels and/or increased IgE levels), the reaction should be reported as an allergic reaction or anaphylactic reaction, as appropriate, and patients should stop treatment with RO6958688.

“Use of this document is governed by the terms of use on the first page of this document.”

5.2.5.2 Management of Specific Adverse Events related to obinutuzumab

5.2.5.2.1 Infusion Related Reactions

No dose reductions of obinutuzumab are recommended. For management of symptomatic AEs, please refer to the table below

Grade 1-2	Reduce infusion rate and treat symptoms.<br>Give supportive treatment. [a]<br>Upon resolution of symptoms, continue infusion.<br>If patient does not experience any IRR symptoms, infusion rate escalation may resume at the increments and intervals as appropriate for the treatment dose
Grade 3	Temporarily interrupt infusion and treat symptoms.<br>Give supportive treatment. [a]<br>Upon resolution of symptoms, restart infusion at no more than half the previous rate (the rate being used at the time that the IRR occurred).<br>If patient does not experience any further IRR symptoms, infusion rate escalation may resume at the increments of 50 mg/hr every 30 minutes without exceeding 400 mg/hr.<br>If the patient experiences a second occurrence of a Grade 3 IRR, stop infusion and permanently discontinue therapy.
Grade 4	Stop infusion and treat symptoms aggressively. [a]<br>Permanently discontinue obinutuzumab.

[a] Supportive treatment should include acetaminophen/paracetamol and an antihistamine (e.g., diphenhydramine), if not administered within the previous 4 hours. Saline (IV) may be indicated. For bronchospasm, urticaria, or dyspnea, patients may require antihistamines, oxygen, corticosteroids (e.g., 100 mg oral prednisone [or equivalent]), and/or bronchodilators.

5.2.5.2.2 Neutropenia and Infections

Patients who experience neutropenia should be closely monitored with regular laboratory tests until resolution. If treatment is necessary, it should be administered in accordance with local guidelines and administration of granulocyte colony-stimulating factors should be considered. Any signs of concomitant infection should be treated as appropriate.

5.2.5.2.3 Thrombocytopenia

Patients should be closely monitored for thrombocytopenia, regular laboratory tests should be performed until the event resolves. Transfusion of blood products (i.e. platelet transfusion) according to institutional practice is at the discretion of the treating physician. Use of all concomitant therapies, which could possibly worsen thrombocytopenia related events such as platelet inhibitors and anticoagulants, should also be taken into consideration, especially during the first cycle.

“Use of this document is governed by the terms of use on the first page of this document.”

5.2.5.2.4 Infections

Obinutuzumab should not be administered in the presence of an active infection. Serious, bacterial, fungal, and new or reactivated viral infections can occur during and following the completion of obinutuzumab therapy.

Reactivation of hepatitis B in patients with chronic hepatitis (HBsAg positive) with evidence of prior hepatitis B exposure, or in patients who are carriers (HBsAg negative and HBcAb positive) has been reported with other anti-CD20 antibodies. The risk is increased particularly when anti-CD20 antibodies are administered with immunosuppressive therapies, such as steroids or chemotherapy. Patients positive for HBsAg and HBcAb are not eligible for this study.

5.2.5.2.5 Progressive multifocal leukoencephalopathy

The diagnosis of PML should be considered in any patient presenting with new-onset or changes to pre-existing neurologic manifestations. The symptoms of PML are unspecific and can vary depending on the affected region of the brain. Motor symptoms with corticospinal tract findings (e.g. muscular weakness, paralysis, and sensory disturbances), sensory abnormalities, cerebellar symptoms, and visual field defects are common. Some signs/symptoms regarded as “cortical” (e.g. aphasia or visual-spatial disorientation) may occur. Evaluation of PML includes, but is not limited to, consultation with a neurologist, brain magnetic resonance imaging (MRI), and lumbar puncture (CSF testing for JC viral DNA). The patient should be referred to a neurologist for the evaluation and treatment of PML.

5.2.5.2.6 Risk of Cardiac Events

No direct cardiac toxicity is expected as the expression of CD20 antigen has not been identified in cardiac tissue. In nonclinical studies, no cardiac toxicity was observed in monkeys. IRRs may contribute to the incidence of cardiac events. Fluid overload and hypotension may trigger cardiac complications such as decompensation of chronic heart failure and ischemic events, respectively. Please see the obinutuzumab Investigator’s Brochure for further clinical information and for risk minimization measures.

In addition to the toxicities to obinutuzumab listed above, TLS, GI perforation and impaired immunization response have been observed after obinutuzumab treatment but in hematological malignancies (see obinutuzumab Investigator’s Brochure). Impaired immunization response has limited applicability as recent and during treatment vaccinations are not allowed.

5.2.6 <u>Management of Specific Adverse Events related to RO6958688 and obinutuzumab</u>

5.2.6.1 Infusion-Related Reactions/Cytokine Release Syndrome

Administration of therapeutic antibodies may cause IRRs/CRS characterized by symptoms such as fever, chills, dizziness, hypertension, hypotension, dyspnea, restlessness, sweating, flushing, skin rash, tachycardia, tachypnoea, headache, tumor

“Use of this document is governed by the terms of use on the first page of this document.”

pain, nausea, and/or vomiting. Such reactions typically occur during or shortly after an infusion, or within 24 hours after study drug infusion predominantly at the first infusion. The incidence and severity typically decrease with subsequent infusions.

Patients may also develop IgE–mediated hypersensitivity reactions to RO6958688 or obinutuzumab. IRRs/CRS may be indistinguishable from an anaphylactic reaction.

If a hypersensitivity reaction is suspected during or after an infusion (e.g. symptoms typically occurring after previous exposure and very rarely with the first infusion), the infusion should be stopped and treatment permanently discontinued. Patients with known hypersensitivity to obinutuzumab must not be treated.

However, if a patient experienced an isolated episode of fever within 24 hours after study drug infusion, not accompanied by other IRR-like symptoms as described above, then the adverse event reporting term will be fever and not IRR.

If a hypersensitivity or IRR/CRS develops during the infusion of obinutuzumab, the infusion should be temporarily interrupted or slowed down and concomitant medication may be administered if deemed appropriate by the investigator. Upon the resolution of symptoms, the infusion will resume at one-half the previous rate (the rate being used at the time that the hypersensitivity or IRR occurred), and infusion-rate escalation may resume at the increments and intervals described in Table 3 and Table 4.

Premedication and Administration of Obinutuzumab

Prior to administration of obinutuzumab, premedication with acetaminophen, an anti-histamine and corticosteroids (e.g., 100 mg IV prednisolone or equivalent) is mandatory and must be administered at least 60 minutes prior to the administration of each dose of obinutuzumab. An equivalent dose of dexamethasone (20 mg) or methylprednisolone (80 mg) is permitted, but hydrocortisone should not be used.

If an IRR develops, the infusion of RO6958688 or obinutuzumab (see Table 4) should be slowed down or interrupted. The patient should be monitored until complete resolution of the symptoms and treated as clinically indicated. Treatment or concomitant medication may include acetaminophen/paracetamol, antihistamine, IV saline, oxygen, bronchodilators, corticosteroids, and vasopressors, depending on the symptoms.

In case of one Grade 4 IRR related to RO6958688 or obinutuzumab, the administration of the study drug in question has to be permanently discontinued.

“Use of this document is governed by the terms of use on the first page of this document.”

5.3 METHODS AND TIMING FOR CAPTURING AND ASSESSING SAFETY PARAMETERS

The investigator is responsible for ensuring that all adverse events (see Section 5.2.5 for definition) are recorded on the Adverse Event eCRF and reported to the Sponsor in accordance with instructions provided in this section and in Sections 5.5–5.7.

For each adverse event recorded on the Adverse Event eCRF, the investigator will make an assessment of seriousness (see Section 5.1.2 for seriousness criteria), severity (see Section 5.3.3), and causality (see Section 5.3.4).

5.3.1 Adverse Event Reporting Period

Investigators will seek information on adverse events at each patient contact. All adverse events, whether reported by the patient or noted by study personnel, will be recorded in the patient's medical record. Adverse events will then be reported on the Adverse Event eCRF as follows:

After informed consent has been obtained but prior to initiation of any study drug, only serious adverse events caused by a protocol-mandated intervention should be reported (e.g., serious adverse events related to invasive procedures such as biopsies).
Any other adverse event should not be reported.

After initiation of any study drug, all adverse events, regardless of relationship to any of the study drugs, will be reported until 28 days after the last dose of RO6958688 or obinutuzumab if for any reason the patient does not receive any RO6958688 infusion *or until initiation of new systemic anti-cancer therapy, whichever occurs first.* Second malignancies will be recorded indefinitely, regardless of relationship to study treatment, for patients who received obinutuzumab pre-treatment (even if the study has been closed) and Grade 3 or higher infections (related and unrelated), should be reported until up to 2 years after the last dose.

After a period of 28 days from the last dose of any of the study drugs, investigators should report any deaths, serious adverse events, or other adverse events of concern that are believed to be related to prior treatment with study drugs (see Section 5.6).

5.3.2 Eliciting Adverse Event Information

A consistent methodology of non-directive questioning should be adopted for eliciting adverse event information at all patient evaluation time points. Examples of non-directive questions include the following:

- "How have you felt since your last clinic visit?"
- "Have you had any new or changed health problems since you were last here?"

"Use of this document is governed by the terms of use on the first page of this document."

5.3.3 Assessment of Severity of Adverse Events

The adverse event severity grading scale NCI CTCAE v4.03 will be used to assess adverse event severity. NCI CTCAE v4.03 can be found at:

http://evs.nci.nih.gov/ftp1/CTCAE/CTCAE_4.03_2010-06-14_QuickReference_8.5x11.pdf.

Table 11 will be used for assessing severity for adverse events that are not specifically listed in the NCI CTCAE.

Table 11 Adverse Event Severity Grading Scale

Grade	Severity
1	Mild; asymptomatic or mild symptoms; clinical or diagnostic observations only; or intervention not indicated
2	Moderate; minimal, local, or non-invasive intervention indicated; or limiting age-appropriate instrumental activities of daily living [a]
3	Severe or medically significant, but not immediately life-threatening; hospitalization or prolongation of hospitalization indicated; disabling; or limiting self-care activities of daily living [b,c]
4	Life-threatening consequences or urgent intervention indicated [d]
5	Death related to adverse event [d]

[a] Instrumental activities of daily living refer to preparing meals, shopping for groceries or clothes, using the telephone, managing money, etc.

[b] Examples of self-care activities of daily living include bathing, dressing and undressing, feeding one's self, using the toilet, and taking medications, as performed by patients who are not bedridden.

[c] If an event is assessed as a "significant medical event," it must be reported as a serious adverse event (see Section 5.4.2 for reporting instructions), per the definition of serious adverse event in Section 5.1.2.

[d] Grade 4 and 5 events must be reported as serious adverse events (see Section 5.4.2 for reporting instructions), per the definition of serious adverse event in Section 5.1.2.

CRS severity will be graded according to NCI CTCAE v5 for events that are considered related to RO6958688 only (Table 9).

5.3.4 Assessment of Causality of Adverse Events

Investigators should use their knowledge of the patient, the circumstances surrounding the event, and an evaluation of any potential alternative causes to determine whether or not an adverse event is considered to be related to the study drug, indicating "yes" or "no" accordingly.

The following guidance should be taken into consideration:

- Temporal relationship of event onset to the initiation of study drug
- Course of the event, considering especially the effects of dose reduction, discontinuation of study drug, or reintroduction of study drug (where applicable)
- Known association of the event with the study drug or with similar treatments

"Use of this document is governed by the terms of use on the first page of this document."

- Known association of the event with the disease under study
- Presence of risk factors in the patient or use of concomitant medications known to increase the occurrence of the event
- Presence of non-treatment-related factors that are known to be associated with the occurrence of the event.

5.3.5 Procedures for Recording Adverse Events

Investigators should use correct medical terminology/concepts when recording adverse events on the Adverse Event eCRF. Avoid colloquialisms and abbreviations.

Only one adverse event term should be recorded in the event field on the Adverse Event eCRF.

5.3.5.1 Infusion-Related Reactions/Hypersensitivity reactions and Cytokine Release Syndrome Attributed to RO6958688

As IRRs and CRS may be indistinguishable from one another, the following procedure should be followed to record IRR/CRS events considered related to RO6958688:

- All infusion-related events occurring within 24 hours of the first two infusions of RO6958688 should be recorded as IRR unless hypotension or hypoxia is one of the predominant symptoms

- All infusion-related events occurring within 24 hours of RO6958688 infusion with hypotension or hypoxia as the predominant symptom, and infusion related events associated with 3rd infusion of RO6958688 or later should be recorded as CRS. Adverse events of CRS considered related to RO695688 will be graded according to CTCAE v5 (Table 9).

The one exception to this reporting guidance is if a clinical presentation suggests an immediate, acute hypersensitivity (e.g., generalized hives, mucosal edema, with or without wheezing and hypotension). *Elevated IgE and/or tryptase levels support an allergic origin. In this case* a diagnosis of “allergic reaction” or “hypersensitivity reaction” or “anaphylaxis” should be used.

For adverse events with a diagnosis of IRR/CRS associated signs, symptoms, and laboratory abnormalities should be recorded on the dedicated Infusion-Related Reaction/Cytokine Release Syndrome eCRF. Each IRR/CRS should be recorded separately on the Adverse Event eCRF, with signs, symptoms, and laboratory abnormalities also recorded separately on the dedicated Infusion-Related Reaction/Cytokine Release Syndrome eCRF. Ambiguous terms such as "systemic reaction" should be avoided. In addition to documentation in the Adverse Event eCRF, non-serious Grade ≥ 2 CRS and IRR events should be reported as a non-serious adverse event of special interest (see Section 5.1.3).

“Use of this document is governed by the terms of use on the first page of this document.”

Adverse events of IRR considered related to another study drug, and not RO6958688, will be graded using CTCAE v4.03.

Other Adverse Events

A diagnosis (if known) should be recorded on the Adverse Event eCRF rather than individual signs and symptoms (e.g., record only liver failure or hepatitis rather than jaundice, asterixis, and elevated transaminases). However, if a constellation of signs and/or symptoms cannot be medically characterized as a single diagnosis or syndrome at the time of reporting, each individual event should be recorded on the Adverse Event eCRF. If a diagnosis is subsequently established, all previously reported adverse events based on signs and symptoms should be nullified and replaced by one adverse event report based on the single diagnosis, with a starting date that corresponds to the starting date of the first symptom of the eventual diagnosis.

5.3.5.2 Adverse Events Occurring Secondary to Other Events

In general, adverse events that occur secondary to other events (e.g., cascade events or clinical sequelae) should be identified by their primary cause, with the exception of severe or serious secondary events. However, medically significant adverse events that occur secondary to an initiating event that are separated in time should be recorded as independent events on the Adverse Event eCRF. For example:

- If vomiting results in mild dehydration with no additional treatment in a healthy adult, only vomiting should be reported on the eCRF
- If vomiting results in severe dehydration, both events should be reported separately on the eCRF
- If a severe GI hemorrhage leads to renal failure, both events should be reported separately on the eCRF
- If dizziness leads to a fall and subsequent fracture, all three events should be reported separately on the eCRF.

All adverse events should be recorded separately on the Adverse Event eCRF if it is unclear as to whether the events are associated.

5.3.5.3 Persistent or Recurrent Adverse Events

A persistent adverse event is one that extends continuously, without resolution, between patient evaluation timepoints. Such events should only be recorded once on the Adverse Event eCRF. The initial severity of the event should be recorded, and the severity should be updated to reflect the most extreme severity any time the event worsens. If the event becomes serious, the Adverse Event eCRF should be updated to reflect this.

“Use of this document is governed by the terms of use on the first page of this document.”

A recurrent adverse event is one that resolves between patient evaluation timepoints and subsequently recurs. Each recurrence of an adverse event should be recorded separately on the Adverse Event eCRF.

5.3.5.4 Abnormal Laboratory Values

Not every laboratory abnormality qualifies as an adverse event. A laboratory test result should be reported as an adverse event if it meets any of the following criteria:

- Accompanied by clinical symptoms
- Results in a change in study treatment (e.g., dosage modification, treatment interruption, or treatment discontinuation)
- Results in a medical intervention (e.g., potassium supplementation for hypokalemia) or a change in concomitant therapy
- Clinically significant in the investigator's judgment.

It is the investigator's responsibility to review all laboratory findings. Medical and scientific judgment should be exercised in deciding whether an isolated laboratory abnormality should be classified as an adverse event.

If a clinically significant laboratory abnormality is a sign of a disease or syndrome (e.g., ALP and bilirubin 5 × ULN associated with cholecystitis), only the diagnosis (i.e., cholecystitis) should be recorded on the Adverse Event eCRF.

If a clinically significant laboratory abnormality is not a sign of a disease or syndrome, the abnormality itself should be recorded on the Adverse Event eCRF, along with a descriptor indicating if the test result is above or below the normal range (e.g., "elevated potassium," as opposed to "abnormal potassium"). If the laboratory abnormality can be characterized by a precise clinical term per standard definitions, the clinical term should be recorded as the adverse event. For example, an elevated serum potassium level of 7.0 mEq/L should be recorded as "hyperkalemia".

Observations of the same clinically significant laboratory abnormality from visit to visit should not be repeatedly recorded on the Adverse Event eCRF, unless the etiology changes. The initial severity of the event should be recorded, and the severity or seriousness should be updated any time the event worsens.

5.3.5.5 Abnormal Vital Sign Values

Not every vital sign abnormality qualifies as an adverse event. A vital sign result should be reported as an adverse event if it meets any of the following criteria:

- Accompanied by clinical symptoms
- Results in a change in study treatment (e.g., dosage modification, treatment interruption, or treatment discontinuation)
- Results in a medical intervention or a change in concomitant therapy

"Use of this document is governed by the terms of use on the first page of this document."

- Clinically significant in the investigator's judgment.

It is the investigator's responsibility to review all vital sign findings. Medical and scientific judgment should be exercised in deciding whether an isolated vital sign abnormality should be classified as an adverse event.

If a clinically significant vital sign abnormality is a sign of a disease or syndrome (e.g., high BP), only the diagnosis (i.e., hypertension) should be recorded on the Adverse Event eCRF.

Observations of the same clinically significant vital sign abnormality from visit to visit should not be repeatedly recorded on the Adverse Event eCRF, unless the etiology changes. The initial severity of the event should be recorded, and the severity or seriousness should be updated any time the event worsens.

5.3.5.6 Abnormal Liver Function Tests

The finding of an elevated ALT or AST ($>3\times$ ULN) in combination with either an elevated total bilirubin ($>2\times$ ULN) or clinical jaundice in the absence of cholestasis or other causes of hyperbilirubinemia is considered to be an indicator of severe liver injury. Therefore, investigators must report as an adverse event the occurrence of either of the following:

- Treatment-emergent ALT or AST $>3\times$ ULN in combination with total bilirubin $>2\times$ ULN
- Treatment-emergent ALT or AST $>3\times$ ULN in combination with clinical jaundice.

The most appropriate diagnosis or (if a diagnosis cannot be established) the abnormal laboratory values should be recorded on the Adverse Event eCRF (see Section 5.3.5.1 and reported to the Sponsor immediately (i.e., no more than 24 hours after learning of the event), either as a serious adverse event or a non-serious adverse event of special interest (see Section 5.4.2).

5.3.5.7 Deaths

For this protocol, mortality is an efficacy endpoint. Deaths that occur during the protocol–specified adverse event reporting period (see Section 5.3.5.7) that are attributed by the investigator solely to progression of cancer should be recorded only on the Study Completion/Early Discontinuation eCRF. All other on-study deaths, regardless of relationship to study drug, must be recorded on the Adverse Event eCRF and immediately reported to the Sponsor (see Section 5.3.5.7).

Death should be considered an outcome and not a distinct event. The event or condition that caused or contributed to the fatal outcome should be recorded as the single medical concept on the Adverse Event eCRF. Generally, only one such event should be reported. The term "sudden death" should only be used for the occurrence of an abrupt and unexpected death due to presumed cardiac causes in a patient with or without

"Use of this document is governed by the terms of use on the first page of this document."

preexisting heart disease, within 1 hour of the onset of acute symptoms or, in the case of an unwitnessed death, within 24 hours after the patient was last seen alive and stable. If the cause of death is unknown and cannot be ascertained at the time of reporting, "unexplained death" should be recorded on the Adverse Event eCRF. If the cause of death later becomes available (e.g., after autopsy), "unexplained death" should be replaced by the established cause of death.

5.3.5.8 Preexisting Medical Conditions

A preexisting medical condition is one that is present at the screening visit for this study. Such conditions should be recorded on the General Medical History and Baseline Conditions eCRF.

A preexisting medical condition should be recorded as an adverse event only if the frequency, severity, or character of the condition worsens during the study. When recording such events on the Adverse Event eCRF, it is important to convey the concept that the preexisting condition has changed by including applicable descriptors (e.g., "more frequent headaches").

5.3.5.9 Lack of Efficacy or Worsening of Cancer

Events that are clearly consistent with the expected pattern of progression of the underlying disease should not be recorded as adverse events. These data will be captured as efficacy assessment data only. In most cases, the expected pattern of progression will be based on RECIST v1.1 criteria and irRC. In rare cases, the determination of clinical progression will be based on symptomatic deterioration. However, every effort should be made to document progression using objective criteria. If there is any uncertainty as to whether an event is due to disease progression, it should be reported as an adverse event.

5.3.5.10 Hospitalization or Prolonged Hospitalization

Any adverse event that results in hospitalization or prolonged hospitalization should be documented and reported as a serious adverse event (per the definition of serious adverse event in Section 5.3.5.10), except as outlined below.

The following hospitalization scenarios are not considered to be serious adverse events:

- Hospitalization for respite care
- Planned hospitalization required by the protocol (e.g., for study drug administration or insertion of access device for study drug administration)
- Hospitalization for a preexisting condition, provided that all of the following criteria are met:
    - The hospitalization was planned prior to the study or was scheduled during the study when elective surgery became necessary because of the expected normal progression of the disease
    - The patient has not suffered an adverse event

"Use of this document is governed by the terms of use on the first page of this document."

- Hospitalization due solely to progression of the underlying cancer.

5.3.5.11 *Cases of Accidental Overdose or Medication Error*

Accidental overdose and medication error (hereafter collectively referred to as "special situations"), are defined as follows:

- *Accidental overdose: accidental administration of a drug in a quantity that is higher than the assigned dose*
- *Medication error: accidental deviation in the administration of a drug*

  In some cases, a medication error may be intercepted prior to administration of the drug.

Special situations are not in themselves adverse events, but may result in adverse events. Each adverse event associated with a special situation should be recorded separately on the Adverse Event eCRF. If the associated adverse event fulfills seriousness criteria, the event should be reported to the Sponsor immediately (i.e., no more than 24 hours after learning of the event; see Section 5.4.2). For RO6958688, obinutuzumab, or tocilizumab, adverse events associated with special situations should be recorded as described below for each situation:

- *Accidental overdose: Enter the adverse event term. Check the "Accidental overdose" and "Medication error" boxes.*
- *Medication error that does not qualify as an overdose: Enter the adverse event term. Check the "Medication error" box.*
- *Medication error that qualifies as an overdose: Enter the adverse event term. Check the "Accidental overdose" and "Medication error" boxes.*

In addition, all special situations associated with RO6958688, obinutuzumab, or tocilizumab, regardless of whether they result in an adverse event, should be recorded on the Adverse Event eCRF as described below:

- *Accidental overdose: Enter the drug name and "accidental overdose" as the event term. Check the "Accidental overdose" and "Medication error" boxes.*
- *Medication error that does not qualify as an overdose: Enter the name of the drug administered and a description of the error (e.g., wrong dose administered, wrong dosing schedule, incorrect route of administration, wrong drug, expired drug administered) as the event term. Check the "Medication error" box.*
- *Medication error that qualifies as an overdose: Enter the drug name and "accidental overdose" as the event term. Check the "Accidental overdose" and "Medication error" boxes. Enter a description of the error in the additional case details.*
- *Intercepted medication error: Enter the drug name and "intercepted medication error" as the event term. Check the "Medication error" box. Enter a description of the error in the additional case details.*

"Use of this document is governed by the terms of use on the first page of this document."

As an example, an accidental overdose that resulted in a headache would require two entries on the Adverse Event eCRF, one entry to report the accidental overdose and one entry to report the headache. The "Accidental overdose" and "Medication error" boxes would need to be checked for both entries.

5.4 IMMEDIATE REPORTING REQUIREMENTS FROM INVESTIGATOR TO SPONSOR

Certain events require immediate reporting to allow the Sponsor to take appropriate measures to address potential new risks in a clinical trial. The investigator must report such events to the Sponsor immediately; under no circumstances should reporting take place more than 24 hours after the investigator learns of the event. The following is a list of events that the investigator must report to the Sponsor within 24 hours after learning of the event, regardless of relationship to study drug:

- Serious adverse events (defined in Section 5.1.2; see Section 5.4.2 for details on reporting requirements)
- Adverse events of special interest (defined in Section 5.1.3; see Section 5.4.2 for details on reporting requirements)
- Pregnancies (see Section 5.4.3 for details on reporting requirements)

5.4.1 Emergency Medical Contacts

To ensure the safety of study patients, access to the Medical Monitors is available 24 hours a day 7 days a week. Medical monitors contact details are listed in the "Protocol Administrative and Contact Information & List of Investigators".

5.4.2 Reporting Requirements for Serious Adverse Events and Non-Serious Adverse Events of Special Interest

For reports of serious adverse events and non-serious adverse events of special interest (see Sections 5.1.2 and 5.1.3), investigators should record all case details that can be gathered on the Serious Adverse Reporting Form and forward this form to the Serious Adverse Event Responsible within 24 hours.

5.4.3 Reporting Requirements for Pregnancies

5.4.3.1 Pregnancies

Female patients of childbearing potential will be instructed to immediately inform the investigator if they become pregnant during the study or within 4 months after the last dose of RO6958688 or within 18 months after the last dose of obinutuzumab or 3 months after the last dose of tocilizumab. A Clinical Trial Pregnancy Reporting Form should be completed by the investigator and submitted to the Sponsor within 24 hours after learning of the pregnancy. Pregnancy should not be recorded on the Adverse Event eCRF. The investigator should discontinue study drug, and counsel the patient

"Use of this document is governed by the terms of use on the first page of this document."

discussing the risks of the pregnancy and the possible effects on the fetus. Monitoring of the patient should continue until conclusion of the pregnancy. Any serious adverse events associated with the pregnancy (e.g., an event in the fetus, an event in the mother during or after the pregnancy, or a congenital anomaly/birth defect in the child) should be reported on the Adverse Event eCRF.

5.4.3.2 Pregnancies in Female Partners of Male Patient

Pregnancies in Female Partners of Male patients will be instructed through the Informed Consent Form to immediately inform the investigator if their partner becomes pregnant during the study or within 4 months after the last dose of RO6958688 or within 18 months after the last dose of obinutuzumab or 2 months after the last dose of tocilizumab. A Clinical Trial Pregnancy Reporting Form should be completed by the investigator and submitted to the Sponsor within 24 hours after learning of the pregnancy. Attempts should be made to collect and report details of the course and outcome of any pregnancy in the partner of a male patient exposed to study drug. The pregnant partner will need to sign an Authorization for Use and Disclosure of Pregnancy Health Information to allow for follow-up on her pregnancy. Once the authorization has been signed, the investigator will update the Clinical Trial Pregnancy Reporting Form with additional information on the course and outcome of the pregnancy. An investigator who is contacted by the male Patient or his pregnant partner may provide information on the risks of the pregnancy and the possible effects on the fetus, to support an informed decision in cooperation with the treating physician and/or obstetrician.

5.4.3.3 Abortions

A spontaneous abortion should be classified as a serious adverse event (as the Sponsor considers spontaneous abortions to be medically significant events), recorded on the Adverse Event eCRF, and reported to the Sponsor immediately (i.e., no more than 24 hours after learning of the event; see Section 5.4.2).

If a therapeutic or elective abortion was performed because of an underlying maternal or embryofetal toxicity, the toxicity should be classified as a serious adverse event, recorded on the Adverse Event eCRF, and reported to the Sponsor immediately (i.e., no more than 24 hours after learning of the event; see Section 5.4.2). A therapeutic or elective abortion performed for reasons other than an underlying maternal or embryofetal toxicity is not considered an adverse event.

All abortions should be reported as pregnancy outcomes on the paper Clinical Trial Pregnancy Reporting Form.

5.4.3.4 Congenital Anomalies/Birth Defects

Any congenital anomaly/birth defect in a child born to a female patient or female partner of a male patient exposed to study drug should be classified as a serious adverse event, recorded on the Adverse Event eCRF, and reported to the Sponsor immediately (i.e., no more than 24 hours after learning of the event; see Section 5.4.2).

"Use of this document is governed by the terms of use on the first page of this document."

5.5 FOLLOW-UP OF PATIENTS AFTER ADVERSE EVENTS

5.5.1 Investigator Follow-Up

The investigator should follow each adverse event until the event has resolved to baseline grade or better, the event is assessed as stable by the investigator, the patient is lost to follow-up, or the patient withdraws consent. Every effort should be made to follow all serious adverse events considered to be related to study drug or trial-related procedures until a final outcome can be reported.

During the study period, resolution of adverse events (with dates) should be documented on the Adverse Event eCRF and in the patient's medical record to facilitate source data verification. If, after follow-up, return to baseline status or stabilization cannot be established, an explanation should be recorded on the Adverse Event eCRF.

All pregnancies reported during the study should be followed until pregnancy outcome and reported according to the instructions provided in Section 5.4.3.

The investigator must report new significant follow-up information for these events to the Sponsor immediately (i.e., no more than 24 hours after becoming aware of the information). New significant information includes the following:

- New signs or symptoms or a change in the diagnosis
- Significant new diagnostic test results
- Change in causality based on new information
- Change in the event's outcome, including recovery
- Additional narrative information on the clinical course of the event

Investigators must also comply with local requirements for reporting serious adverse events to the local health authority and IRB/EC.

5.5.2 Sponsor Follow-Up

For serious adverse events, non-serious adverse events of special interest, and pregnancies, the Sponsor or a designee may follow up by telephone, fax, electronic mail, and/or a monitoring visit to obtain additional case details and outcome information (e.g., from hospital discharge summaries, consultant reports, autopsy reports) in order to perform an independent medical assessment of the reported case.

5.6 POST-STUDY ADVERSE EVENTS

The investigator is not required to actively monitor patients for adverse events after the end of the adverse event reporting period, (defined as 28 days after the last dose of study drug). However, the Sponsor should be notified if the investigator becomes aware of any death, other serious adverse event, or non-serious adverse event of special interest occurring after the end of the adverse event reporting period, if the event is believed to be related to prior study drug treatment.

"Use of this document is governed by the terms of use on the first page of this document."

The sponsor should also be notified of events of second malignancies indefinitely, regardless of relationship to study treatment, (even if the study has been closed) for patients who received obinutuzumab pre-treatment.

The investigator should report these events to Roche Safety Risk Management on the Adverse Event eCRF. If the Adverse Event eCRF is no longer available, the investigator should report the event directly to Roche Safety Risk Management via telephone (see "Protocol Administrative and Contact Information & List of Investigators").

5.7 EXPEDITED REPORTING TO HEALTH AUTHORITIES, INVESTIGATORS, INSTITUTIONAL REVIEW BOARDS, AND ETHICS COMMITTEES

The Sponsor will promptly evaluate all serious adverse events and non-serious adverse events of special interest against cumulative product experience to identify and expeditiously communicate possible new safety findings to Investigators, IRBs, ECs, and applicable health authorities based on applicable legislation.

To determine reporting requirements for single adverse event cases, the Sponsor will assess the expectedness of these events using the following reference document:

- RO6958688 Investigator's Brochure
- Obinutuzumab Investigator's Brochure
- Tocilizumab Investigator's Brochure

The Sponsor will compare the severity of each event and the cumulative event frequency reported for the study with the severity and frequency reported in the applicable reference document.

Reporting requirements will also be based on the Investigator's assessment of causality and seriousness, with allowance for upgrading by the Sponsor as needed.

6. STATISTICAL CONSIDERATIONS AND ANALYSIS PLAN

The data will be analyzed by the Sponsor and/or designated contract research organization (CRO). Any data analysis carried out independently by the investigator should be submitted to the Sponsor before publication or presentation. The data will be summarized with respect to demographic and baseline characteristics, efficacy observations and measurements, safety observations and measurements, and PK and biomarker measurements. Patients will be followed up until disease progression (according to RECIST v1.1 or to irRC, whichever occurs later) or until discontinuation of treatment due to withdrawal from the study. All data up until this point will be listed by patient and summarized by regimen and dose, *as appropriate*.

“Use of this document is governed by the terms of use on the first page of this document.”

6.1 PRIMARY STUDY VARIABLES

The primary endpoint of the study will be safety and, in particular, the occurrence of a DLT. The primary variables of the study are PK, including ADA titers against RO6958688, and AE profile, including DLTs, the MTD with/without obinutuzumab pretreatment for the QW regimen and the late cycle MTD for the step up dosing regimen, if achieved.

6.2 SECONDARY STUDY VARIABLES

The secondary variables will be as follows:

- *The efficacy endpoints of objective ORR, DOR, DCR, and PFS according to RECIST v1.1 criteria and to irRC (survival will also be evaluated if data is mature).*
- *The PD effect for the different dosing regimens on the basis of an increase in activated intratumoral T cells*

6.3 DETERMINATION OF SAMPLE SIZE

6.3.1 Sample size for the dose escalation part

Table 12 provides the operating characteristics of the mCRM with EWOC design for the dose escalation without obinutuzumab pretreatment across different assumed toxicity scenarios (see Appendix 6 for more details):

- Dose: This is the final dose [mg] determined as the MTD by the mCRM design.
- Tox. at dose: This is the resulting true probability [%] of a DLT at the determined MTD.
- Prop. of DLTs: This is the proportion of patients [%] with a DLT.
- n : This is the total number of patients in the dose escalation.
- n_{overdose}: This is the number of overdosed patients, which have been exposed to doses with DLT probability above the target of 35%.

These operating characteristics comprise both part I and part II of the dose escalation phase. Operating characteristics for the mCRM with EWOC design for the dose escalation with obinutuzumab pretreatment are not provided, because 1) it will have similar performance to the escalation without obinutuzumab pretreatment and 2) the results are also depending on the escalation data without obinutuzumab pretreatment (see below for the model definition). The operating characteristics below indicate that, across a wide range of scenarios, the design performs reasonably well, both with steep as well as gradually increasing dose-toxicity curves. In particular, the resulting MTD estimates do not exceed the target toxicity range of 20% to 35%. The required sample size is in most scenarios below 50 patients. The reasonable behavior of the dose-escalation design is also reflected in the number of overdosed patients, which is on average and for most scenarios not exceeding 10 patients. Exceptions are scenarios 3 and 8, which have very high toxicity. These scenarios also have the highest proportion of DLTs in the trials (44% and 27% on average), while for the other scenarios the DLT

"Use of this document is governed by the terms of use on the first page of this document."

proportion is below 21% on average. For all scenarios, the final MTD estimate is on average in the target dose range.

This design has previously been used successfully in (Bailey et al. 2009) as well as in Roche with only slight modifications from the current set-up.

Table 12 Operating Characteristics of the mCRM with EWOC Design with Respect to the Chosen Scenarios

No	Dose	Tox. at dose	Prop. of DLTs	n	n_{overdose}
1	252.2 (155, 355)	32 % (16 %, 50 %)	14 % (11 %, 17 %)	48 (41, 56)	10 (4, 16)
2	7.9 (1.8, 17.1)	25 % (17 %, 34 %)	21 % (16 %, 27 %)	32 (20, 47)	5 (0, 15)
3	0.1 (0, 0.2)	25 % (0 %, 44 %)	44 % (29 %, 67 %)	18 (5, 29)	16 (2, 27)
4	94.2 (50.9, 150)	31 % (16 %, 50 %)	16 % (12 %, 19 %)	42 (35, 48)	9 (1, 16)
5	69.3 (48, 90)	33 % (7 %, 62 %)	16 % (14 %, 19 %)	41 (36, 46)	10 (6, 13)
6	898.1 (598.5, 1000)	30 % (16 %, 35 %)	7 % (2 %, 11 %)	49 (45, 54)	6 (2, 9)
7	1000 (1000, 1000)	1 % (1 %, 1 %)	1 % (0 %, 2 %)	50 (45, 57)	0 (0, 0)
8	1.3 (0.6, 2.2)	31 % (15 %, 52 %)	27 % (22 %, 32 %)	25 (18, 33)	11 (5, 18)
9	491.8 (284.5, 710.5)	32 % (14 %, 51 %)	12 % (9 %, 16 %)	51 (46, 58)	9 (4, 15)

DLT = dose-limiting toxicity; Prop = proportion; Tox. = toxicity.

Note: Means (10% and 90% quantiles) from 200 simulations per scenario are shown. Both part I and part II of the dose escalation phase are included in these simulations.

6.3.2 Sample size for cohorts C-H

Cohorts C, D, E, F, G and H are designed to estimate the ORR per cancer type under the step-up QWx3/Q3W schedule. The sample size of up to 30 patients for each of the cohorts C, D, E, F and G has been selected in order to obtain sufficiently precise estimates of ORR. Specifically, if no response is observed in 30 patients (i.e. 0% estimated ORR), the 95% confidence interval (CI) for ORR would be 0 to 12%; on the other hand if for example 3 responses are observed (i.e. 10% estimated ORR), the 95% CI would be 2 to 27%. For cohort H, the sample size of approximately 40 patients allows a cross-study comparison with a corresponding cohort with obinutuzumab pretreatment in study WP29945 of the same sample size.

Only Cohort C has enrolled patients; Cohorts D, E, F, G and H have not and will not enroll patients. With the 24 patients enrolled in Cohort C, if no response is observed (i.e., 0% estimated ORR), the 95% confidence interval (CI) for ORR would be 0 to 14%. If, however, 3 responses are observed (i.e. 13% estimated ORR), the 95% CI would be 3% to 32%.

“Use of this document is governed by the terms of use on the first page of this document.”

6.4 SUMMARIES OF CONDUCT OF STUDY

All protocol deviations will be listed. The study is open-label; therefore, no blinded treatment will be administered. Patients will be assigned to a dose/regimen/extension rather than randomized.

6.5 ANALYSIS POPULATIONS

6.5.1 Safety Analysis Population

All patients enrolled in the study who received at least one dose of RO6958688 or obinutuzumab will be included in the safety population.

For the purpose of establishing the maximum tolerated dose, the dose-determining population for the QW flat dose escalation design in Part II consists of all patients *in the multiple ascending dose (MAD) cohort* from the safety population who received at least three administrations of RO6958688 within the first 21 days or more if any of the second or third dose is delayed for any reason and have undergone the scheduled safety evaluations or discontinued earlier due to DLT.

6.5.2 Pharmacokinetic Analysis Population

All patients in the safety population will be included in the PK analysis population. Patients will be excluded from the PK analysis population if they significantly violate the inclusion or exclusion criteria, deviate significantly from the protocol, or if data are unavailable or incomplete which may influence the PK analysis. Excluded cases will be documented together with the reason for exclusion. All decisions on exclusions from the analysis will be made prior to database closure.

6.5.3 Pharmacodynamic Analysis Population

The PD analysis population will be a subset of the safety population, based on the availability of evaluable blood or tumor samples.

6.5.4 Efficacy Analysis Population

Two efficacy analyses will be performed. The primary efficacy analysis population will consist of all patients who receive at least one dose of any study treatment. *If different, an* additional ITT population will consist of all patients who are not screen failures and were included in the trial (ICF signature), and will be used for sensitivity analyses.

6.6 SUMMARIES OF TREATMENT GROUP COMPARABILITY

Comparability across dose levels will be evaluated descriptively via tabulation by demographic characteristics (including age, sex, patient disposition, and previous therapies). Treatment administration will be reported based on number of cycles and dose intensity and obinutuzumab pretreatment status. Baseline status *of key pharmacodynamics endpoints* will also be tabulated to determine comparability. The analysis population will be the safety population.

"Use of this document is governed by the terms of use on the first page of this document."

6.7 SAFETY ANALYSES

Safety analysis will be performed for all the patients in the safety analysis population. All *clinically abnormal* safety parameters will be listed by patient within regimen and summarized in tables*, as appropriate*. Safety will be determined, but not limited to, by adverse events, laboratory tests, vital signs, ECG, physical examinations and performance status. Exposure to study medication will be summarized by total duration of study medication, number of cycles started, cumulative dose and dose intensity using descriptive statistics. Dose modifications, interruptions and their reasons will be presented.

6.7.1 Dose-Escalation Approach

6.7.1.1 Modified-Continual Reassessment Method with Overdose Control

The Multiple Ascending Dose (MAD) cohort in Part II of the study will employ an mCRM with EWOC design, in order to define MTD and/or the recommended dose with/without obinutuzumab pretreatment for the QW flat dose regimen. The design is based on the primary safety variable (the occurrence of a DLT). The MTD is defined separately for treatment with and without obinutuzumab pretreatment as the dose which maximizes the probability of a DLT being in the targeted toxicity interval of 20% to 35%, subject to the probability of DLT being in the excessive toxicity interval of 35% to 100% being $<$ 25%. Patients within a cohort will be enrolled in a sequential manner in cohorts of 3 patients each, which, if required, can be expanded with additional patients.

Each patient will be observed for 21 days after the first RO6958688 dose for DLT assessment or more if any of the second or third dose is delayed for any reason. The first patient in each cohort will be observed for safety for 1 week before additional patients are enrolled in the cohort. If a DLT is already reported in the first patient during the first week of safety observation, the Sponsor will organize a teleconference with the investigators to discuss the safety and tolerability of RO6958688 and to decide whether the subsequent patient in the same cohort will be enrolled at the same dose. Based on emerging safety, PK, and PD data, the 1–week safety observation period between initial administration of RO6958688 to the first patient and in subsequent patients within each dose level may be adjusted as deemed appropriate by the investigators and the Sponsor.

At the end of the DLT period, the same dose might also be escalated in the second cycle instead of the first cycle for the subsequent cohort if in the second and later cycles, the safety profile is better on average and/or the exposure is lower than in Cycle 1. In that case, for purposes of the mCRM model fit, DLTs that occur between the first and second cycle will count as DLTs for the first cycle dose, and DLTs that occur at any later timepoint will be attributed to the second cycle dose.

Once a minimum of 3 patients have completed the 21–day DLT observation period, Sponsor and investigators will evaluate the next dose recommended by the EWOC

“Use of this document is governed by the terms of use on the first page of this document.”

design and agree on the dose for the subsequent cohort. Dose decisions will be separate for the dose escalation with and without obinutuzumab pretreatment, and the corresponding teleconferences might be jointly held if timing allows. Patients in the upcoming obinutuzumab pretreatment cohort may already be pretreated before the decision on the RO6958688 dose is taken. At each dose-escalation step, the dose can be escalated or de-escalated or an additional cohort at that same dose level could be enrolled. At the discretion of the investigator and in case there is no safety concern, all the patients can receive further doses at the same dose level or can be escalated to the next available tolerated dose level that has been cleared for dose escalation after they have completed 2 months of treatment at the current dose level.

The starting dose for RO6958688 in Part II will be 2.5 mg, or a lower dose if Part I is closed earlier due to RO6958688–related adverse events of Grade 3 or above.
The maximum allowable increment for RO6958688 between dose levels will be 100%. Note that this maximum allowable increment applies to doses equal to or above 2.5 mg (a dose grid up to 80 mg with 1 mg spacing, and from 85 to 1000 mg with 5 mg spacing, will be used). Should lower doses be evaluated in Part II, then the dose values will be taken from 0.03 mg, 0.05 mg, 0.10 mg, 0.15 mg, …, 2.45 mg, 2.5 mg. The starting dose for the dose escalation with obinutuzumab pretreatment will be the highest dose which has been cleared without obinutuzumab pretreatment (i.e. the cohort without obinutuzumab pretreatment has completed the DLT period, and the dose is admissible in the EWOC model of the dose escalation without obinutuzumab).

The selection of the next dose will be subject to clinical judgment and mandated safety constraints that limit the size of dose increments. In addition, the clinical judgment of the Sponsor and investigators in the dose-selection process will also be utilized. This may lead to dose selections which differ from the mCRM recommendations, if the scientific and clinical opinion is that this would be more appropriate for patients.

The model–based dose escalation at each step will identify the next recommended dose level subject to the following criteria:

- The posterior probability of being within the target toxicity interval (of 20% to 35% DLT probability) is maximized and
- The posterior probability of being within the excessive toxicity interval (above 35% DLT probability) is below 25%.

The dose-escalation without obinutuzumab pretreatment will stop under the following circumstances:

- The maximum sample size of 60 patients (only counting the minimum required number of patients per cohort) has been reached or
- At least a minimum of 15 patients has been accrued overall (in Parts I and II), at least 6 patients have been accrued near the MTD dose (where near means differing

"Use of this document is governed by the terms of use on the first page of this document."

from the MTD by at most 20%), and in addition the probability that the MTD dose lies within the target toxicity interval is above 40%.

The dose escalation with obinutuzumab pretreatment will stop under the following circumstances:

- The maximum sample size of 30 patients (only counting the minimum required number of patients per cohort) has been reached or
- At least a minimum of 10 patients has been accrued overall, at least 6 patients have been accrued near the MTD dose (where near means differing from the MTD by at most 20%), and in addition the probability that the MTD dose lies within the target toxicity interval is above 40%.

For the mCRM with EWOC design for the dose escalation without obinutuzumab pretreatment, the dose-toxicity relationship is described by a two-parameter logistic regression model:

$$p(x_j) = \frac{\text{expâÅ¡ (Î} \pm +\text{Î\_}x_j)}{1 + \text{expâÅ¡ (Î} \pm +\text{Î\_}x_j)}$$

where $p(x_j)$ denotes the probability to experience a DLT at dose d_j [mg] where $x_j = \log(d_j/d^*)$ is the transformed dose using the reference dose d^*=500. A minimally informative prior distribution (Neuenschwander et al 2008) is used as prior distribution for α and logâÅ¡ (Î_). Specifically the prior distribution is the bivariate normal prior

$$\begin{pmatrix} \alpha \\ \log(\beta) \end{pmatrix} \sim N_2\left(\begin{pmatrix} 1.021 \\ -0.682 \end{pmatrix}, \begin{pmatrix} 1.887 & 0.023 \\ 0.023 & 0.001 \end{pmatrix}\right)$$

This prior model is shown in Figure 7 and has been constructed as follows: At the minimum dose of 0.05 mg, any DLT probability above 20% is unlikely (probability 5%). At the maximum dose of 1000 mg, any DLT probability below 30% is unlikely (probability 5%). At the intermediate doses of 1, 50, 100 and 500 mg, corresponding assumptions (linear on the log-dose scale) were made. The resulting minimally informative beta distributions at the design doses 0.05, 1, 50, 100, 500 and 1000 mg were then approximated by the above bivariate log-normal distribution on the two logistic regression parameters.

"Use of this document is governed by the terms of use on the first page of this document."

Figure 7 Prior Model for the mCRM with EWOC Design

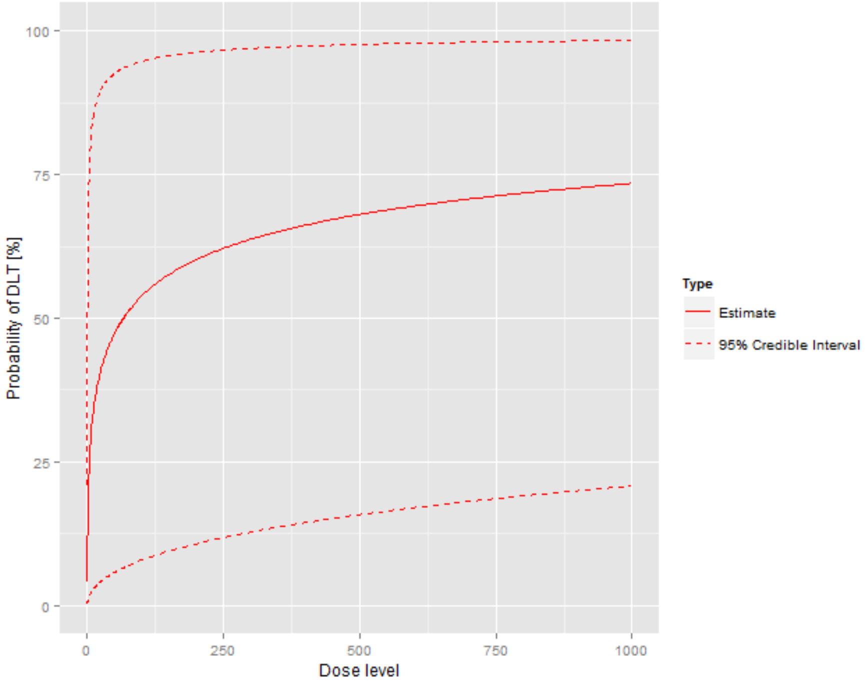

The prior model can be characterized as follows. Based on the target toxicity range from 20% to 35% DLT rate, the MTD estimate is 11 mg. Based on the overdose control rule, the highest dose with acceptable overdosing risk, before seeing any data from Part I, is estimated as 3 mg. This a priori MTD estimate and the very low value of the first acceptable dose are a proof for the conservative nature of the utilized prior distribution.

Furthermore, it is illustrative to consider various hypothetical trial realizations. Table 13 shows hypothetical recommendations of the mCRM with EWOC design, assuming 4 patients per cohort and no DLTs having been observed until the respective dose. For example, it is shown that the design would recommend 5 mg, if no DLTs had been observed in the 5 patients from part I at 0.052, 0.15, 0.45, 1.3 and 2.5 mg also no DLTs had been observed in the first 4 patients in part II at 2.5 mg. If one DLT had been observed at 2.5 mg among the 4 part II patients, then still 5 mg would be recommended. However, if two, three or four DLTs had been observed in those patients, the design would stay at the 2.5 mg dose or reduce to 1.1 mg or 0.45 mg, corresponding to 0%, -56% and -82% increments, respectively. The table shows a reasonable behavior of the design, with higher dose reductions in the beginning of the escalation when only few patient data is yet available. Note that the final next dose will always be subject to clinical judgment and is especially allowed to be lower than the doses listed in this Table 13.

"Use of this document is governed by the terms of use on the first page of this document."

Table 13 Hypothetical recommendations of the mCRM with EWOC design

"Use of this document is governed by the terms of use on the first page of this document."

dose [mg]	DLTs	next dose [mg]	increment [%]
0.05	0	0.15	200
0.05	1	0.03	-40
0.15	0	0.45	200
0.15	1	0.05	-67
0.45	0	1.30	189
0.45	1	0.15	-67
1.30	0	2.50	92
1.30	1	0.45	-65
2.50	0	2.50	0
2.50	1	1.30	-48
2.50	0	5.00	100
2.50	1	5.00	100
2.50	2	2.50	0
2.50	3	1.10	-56
2.50	4	0.45	-82
5.00	0	10.00	100
5.00	1	10.00	100
5.00	2	8.00	60
5.00	3	3.00	-40
5.00	4	1.80	-64
10.00	0	20.00	100
10.00	1	20.00	100
10.00	2	20.00	100
10.00	3	9.00	-10
10.00	4	5.00	-50
20.00	0	40.00	100
20.00	1	40.00	100
20.00	2	40.00	100
20.00	3	21.00	5
20.00	4	12.00	-40
40.00	0	80.00	100
40.00	1	80.00	100
40.00	2	79.00	98
40.00	3	46.00	15
40.00	4	25.00	-38
80.00	0	160.00	100
80.00	1	160.00	100
80.00	2	155.00	94
80.00	3	85.00	6
80.00	4	49.00	-39
160.00	0	320.00	100
160.00	1	320.00	100
160.00	2	295.00	84
160.00	3	160.00	0
160.00	4	95.00	-41
320.00	0	640.00	100

“Use of this document is governed by the terms of use on the first page of this document.”

dose [mg]	DLTs	next dose [mg]	increment [%]
320.00	1	640.00	100
320.00	2	590.00	84
320.00	3	320.00	0
320.00	4	190.00	-41
640.00	0	1000.00	56
640.00	1	1000.00	56
640.00	2	955.00	49
640.00	3	605.00	-5
640.00	4	385.00	-40

For the mCRM with EWOC design for the dose escalation with obinutuzumab pretreatment, the model is a mixture of two logistic regression models: The probability of a DLT at dose d_j is

$$p(d_j) = \pi\, p_1(d_j) + (1 - \pi) p_2(d_j)$$

where π is the probability for the pretreatment model $p(d_j)$ being the same as the model $p_1(d_j)$, without obinutuzumab pretreatment and $1 - \pi$ is the probability of a separate model $p_2(d_j)$, where for $k = 1,2$:

$$p_k(d_j) = \frac{\exp\left(\hat{I}\pm_k + \hat{I}\_{k}\, log\left(\frac{d_j}{d^*}\right)\right)}{1 + \exp\left(\hat{I}\pm_k + \hat{I}\_{k}\, log\left(\frac{d_j}{d^*}\right)\right)}$$

and $d^* = 500$ is the reference dose. The component $p_1(d_j)$ will always be informed by the latest available DLT data from the patients without obinutuzumab pretreatment, by the corresponding likelihood contribution to the parameters α_1 and $\hat{I}\_{1}$. The prior distribution for α_k and $\log(\hat{I}\_{k})$ will be the same bivariate normal distribution as shown above, hence the same minimally informative prior distribution is used for both components here and the mCRM with EWOC design for dose escalation without obinutuzumab pretreatment.

This mixture model allows for dynamic borrowing from the data without obinutuzumab pretreatment (Schmidli et al 2014). Starting from a prior probability of $\pi = 10\%$, π is updated throughout the trial by the DLT data from all patients in this study, in order to obtain the posterior probability π. If the pretreatment DLT data is consistent with the DLT data from patients without obinutuzumab pretreatment, then the posterior probability will increase above 10%. On the other hand, if the pretreatment DLT data is not consistent with the DLT data from patients without obinutuzumab pretreatment (e.g. if there are more DLTs at lower doses), then the posterior probability will decrease appropriately. Note that the borrowing is implemented in a one-way-only fashion here: The DLT data from patients with obinutuzumab pretreatment will not inform the dose escalation without obinutuzumab pretreatment.

"Use of this document is governed by the terms of use on the first page of this document."

After each cohort of patients completes dosing, the posterior distribution of the model parameters of the logistic regression model will be updated with the observed DLT occurrence data and hence a new estimate of the MTD will be derived. Subject to clinical judgment, a new cohort of patients will be dosed at the next recommended estimate of the MTD or the highest allowable dose based on the pre-specified safety constraints, whichever is lower. The design will continue as described, assigning patients to the MTD as estimated from all of the DLT data cumulatively, until one of the pre-defined stopping criteria is satisfied or the pre-determined sample size of 60 DLT-evaluable patients (only counting the minimum required number of patients per cohort, and including Part I patients) for dose escalation without obinutuzumab pretreatment or 30 DLT-evaluable patients for dose escalation with obinutuzumab pretreatment is reached, whichever comes first. This sample size is deemed sufficient to identify the MTD under the different toxicity scenarios explored. With the end of the mCRM phase, a tentative MTD estimate will be defined, separately for treatment with and without obinutuzumab. Moreover, the recommended dose will be defined by taking into consideration all available data gathered so far, separately for treatment with and without obinutuzumab. The DLT occurrence data from the expansions will be used to update the statistical model used in the dose-escalation phase. This will lead to a final MTD estimate, separately for treatment with and without obinutuzumab.

There will be no dose-escalation in the Q3W schedule, however, DLTs observed in the Q3W patients would still be considered in the EWOC model without obinutuzumab. DLT-free Q3W patients would not be considered in the EWOC model.

In addition to modeling the probability of toxicity, models relating PK and PD data will be explored in parallel to the mCRM model and may inform the dose escalation as part of the available exploratory data (provided that appropriate PK and PD data for all patients enrolled up to that timepoint are available).

6.7.1.2 Modified 3+3 dose escalation design

Cohorts A and B in Part II of the study will employ a modified 3+3 dose escalation design for the step up dosing regimen, in order to define the late cycle MTD and the maximum intra-patient dose escalation increments. Two cohorts of patients will be treated, the cohorts will be run in a staggered fashion with Cohort A (late cycle MTD) starting before Cohort B (maximum increments) and cohort B will not be escalated to a dose not already cleared as safe in cohort A:

Cohort A) Approximately 6 evaluable patients at 40 mg in cycle 1 without prophylactic steroids and escalating QW by up to 100% of the previous dose until the DLT criteria for that dose level are met.

Cohort B) Approximately 6 evaluable patients at 40 mg in cycle 1 without prophylactic steroids and escalating QW by up to 150% of the previous dose up to 200 mg and then QW by up to 100% until the DLT criteria for that dose level are met.

"Use of this document is governed by the terms of use on the first page of this document."

Cohort B could only escalate to doses that have been cleared by the cohort A (≥ 2 patients treated at that dose level or above, with ≤ 1 DLT being observed). Therefore, enrollment into cohort B starts at least three weeks after enrollment start into cohort A.

The DLT window is defined as 1 week, to allow dose escalation after 1 week, and a patient is defined as DLT-evaluable if the patient received the full planned dose of RO6958688. The following rules apply separately for cohort A and B:

1. The time interval between dosing of the first and second as well as the second and third patient within each cohort is at least 1 week, to allow for sufficient observation time. The following patients may be dosed any time after the third patient has been dosed.
2. The dose is increased every week within each patient until any of the below applies:
    a. A DLT is observed in this patient. The dose is then reduced to the next lower, and previously tolerated dose level, afterwards.
    b. Less than 6 patients have been treated and ≥ 2 DLTs have been observed at the next higher dose level or below. The dose is then kept constant.
    c. At least 6 patients have been treated and >33% of patients have had DLT at the next higher dose level or below. The dose is then kept constant.
3. The late cycle MTD is defined as follows:
    a. A minimum of 6 patients must have been treated at this dose level or above.
    b. If 6 patients are evaluable for DLT, the late cycle MTD is defined as the highest dose with ≤ 1 DLT having been observed
    c. If more than 6 patients are evaluable for DLT, the late cycle MTD is defined as the highest dose where < 33% of patients have had DLT.

The acceptable characteristics of this dose escalation design are illustrated with simulations for cohort A in the protocol Appendix 7.

6.7.2 Adverse Events

The original terms recorded on the eCRF by the investigator for adverse events will be standardized by the Sponsor.

Adverse event data will be reported in listings and presented in frequency tables by MedDRA terms. Adverse events will be summarized by mapped term and appropriate thesaurus level. The severity of adverse events will be graded according to the NCI CTCAE v4.03 (NCI CTCAE [v5] will be used for CRS). Summaries of adverse event

“Use of this document is governed by the terms of use on the first page of this document.”

by grade, seriousness, and relationship to study treatment will be presented, as well as summaries of adverse events leading to death, and premature withdrawal from study treatment.

6.7.3 Clinical Laboratory Test Results

All clinical laboratory data will be stored on the database in the units in which they were reported. Patient listings and summary statistics at each assessment time will be presented using the International System of Units (SI units; Système International d'Unités). Laboratory data not reported in SI units will be converted to SI units before processing.

*Clinically abnormal l*aboratory test values will be presented by individual listings with flagging of values outside the normal ranges. Summary tables of change from baseline over time based on SI units will be displayed *as needed*. Shifts in NCI CTCAE v4.03 from baseline to the worst grade observed during treatment will be presented for selected laboratory parameters.

6.7.3.1 Standard Reference Ranges and Transformation of Data

Where appropriate, Roche standard reference ranges, rather than the reference ranges of the Investigator, *may* be used. For most parameters, the measured laboratory test result *can* be assessed directly using the Roche standard reference range. Certain laboratory parameters *need to* be transformed to Roche's standard *measurement units first*.

A transformation *may* be performed on certain laboratory tests that lack sufficiently common procedures and have a wide range of investigator ranges, e.g., enzyme tests that include AST, ALT, and ALP, and total bilirubin. *If* the standard reference ranges for parameters have a lower limit of zero, only the upper limits of the ranges *need to* be used in transforming the data.

6.7.3.2 Definition of Laboratory Abnormalities

Laboratory values falling outside *the* standard reference range will be labeled "H" for high or "L" for low in patient listings of laboratory data.

In addition to the standard reference range, a marked reference range has been predefined by Roche for *some* laboratory parameters. The marked reference range is broader than the standard reference range. Values that fall outside the marked reference range that also represent a defined change from baseline *are* considered marked laboratory abnormalities (i.e., potentially clinically relevant). If a baseline value is not available for a patient, the midpoint of the standard reference range will be used as the patient's baseline value for the purposes of determining marked laboratory abnormalities. Marked laboratory abnormalities will be labeled in the patient listings as "HH" for very high or "LL" for very low.

"Use of this document is governed by the terms of use on the first page of this document."

6.7.4 Vital Signs

Clinically abnormal vital signs data *may* be presented by individual listings with flagging of values outside the normal ranges/marked abnormalities *as appropriate*. In addition, tabular summaries will be used, as appropriate.

6.7.5 ECG Data Analysis

Clinically abnormal ECG data *may* be presented by individual listings with flagging of values outside the normal ranges/marked abnormalities *as appropriate*. In addition, the 12-lead triplicates will be averaged out and tabulated by dose level/regimen.

6.7.6 Concomitant Medications

The original terms recorded on the patients' eCRF by the investigator for concomitant medications will be standardized by the Sponsor by assigning preferred terms.

Concomitant medications will be presented in summary tables and listings*, as appropriate*.

6.8 EFFICACY ANALYSES

Efficacy analyses will include all patients in the respective efficacy analysis population with patients grouped according to dose level/extension/obinutuzumab pretreatment status.

Tumor response data will be reported using descriptive statistics. ORR and DCR will be summarized using relative frequencies and *95*% confidence limits. Duration of response and PFS will be summarized using time to event analyses and Kaplan-Meier curves. Overall survival data may be tabulated and summarized if mature. Summaries will be carried out by cohort, dose, and overall*, and duration of response will only be analyzed in those cohorts with a sufficient number of responders*. This will be carried out for both RECIST v1.1 as well as the exploratory irRC efficacy endpoints.

6.8.1 Primary Efficacy Endpoint

The primary efficacy endpoints, for the evaluation of the preliminary anti-tumor activity of single-agent RO6958688, will be ORR, DOR, DCR, PFS, and survival (if data is mature at the time of analysis) according to RECIST v1.1.

The analysis of tumor response is based on the best overall response (BOR). BOR is defined as the best response recorded from the *start of treatment* until disease progression/recurrence *or* death, whichever occurs first. Since tumor assessments take place every 8 weeks, 60 days is chosen so as to cover these assessments sufficiently.

Confirmation of partial and complete responses will be done at the next scheduled visit after at least 28 days from the initial response. A patient is assigned a best overall response SD if they have a response assessment of SD, PR, or CR at one or more visits at least 42 days (6 weeks) after start of RO6958688, but are not a confirmed CR or PR.

"Use of this document is governed by the terms of use on the first page of this document."

For the ORR analyses in the primary and ITT efficacy analysis populations, patients who withdraw study treatment because of any reason, die or clinically progress before the first tumor assessment on-treatment will be *assigned a best overall response of Non Evaluable*. These patients will be included in the denominator of the corresponding ORR estimate.

PFS per RECIST v1.1 is defined as time between enrollment date and date of first documented disease progression per RECIST v1.1 or death from any cause, whichever occurs first. Patients who neither progressed nor died in this interval, or who are lost to follow-up are censored at the date of last tumor assessment within this time window or last follow-up for progression of disease. Patients for whom no post-baseline tumor assessments are available are censored at first study treatment administration.

6.8.2 Secondary Efficacy Endpoints

ORR, DOR, DCR, and PFS will also be reported according to irRC as exploratory endpoints.

6.9 PHARMACODYNAMIC ANALYSES

PD parameters will be listed by patient and tabulated by dose-level/regimen and timepoint*, as appropriate*. Descriptive statistics will be used in summarizing tumor PD markers. Absolute and percentage change from baseline will be calculated for the PD markers. Graphical techniques *may* be employed to better understand the relationship of the PD markers with dose and time. Correlations between PD markers, PK markers of RO6958688, and clinical response *may* be assessed through data tabulations and graphical techniques in all patients treated or not with obinutuzumab. The potential prognostic value of the PD markers may also be investigated.

6.10 PHARMACOKINETIC ANALYSES

PK parameters for RO6958688 will be presented by listings and descriptive summary statistics separately by group or cohorts.

Individual and mean serum RO6958688 concentration versus time data will be tabulated and plotted by dose level. The serum RO6958688 concentration data will be analyzed to estimate the following parameters (if applicable): AUC, C_{max}, and $t_{1/2}$. Estimates for these parameters will be tabulated and summarized (mean, standard deviation, coefficient of variation, median, minimum, and maximum*, as appropriate*). Inter-patient variability and drug accumulation will be evaluated.

Additional PK analyses will be conducted as appropriate, including population PK modelling.

Obinutuzumab serum concentration data (C_{min} and C_{max}) will be tabulated and summarized (e.g. mean, median, range, and standard deviation, as appropriate).

"Use of this document is governed by the terms of use on the first page of this document."

Provided the data permit, population pharmacokinetic method will be applied for analyzing the PK outcome measures of obinutuzumab.

The rates of patients with positive ADA titer against RO6958688 at week 8 will be compared between patients with/without obinutuzumab pretreatment. Furthermore, the onset of ADA formation will be analyzed by survival methods on time-to-first-ADA detection. Separate analyses by doses are possible.

6.11 IMMUNOGENICITY ANALYSES

Immunogenicity will be assessed for RO6958688. The immunogenicity analyses will include all patients with at least one ADA assessment. Patients will be grouped according to treatment received or, if no treatment is received prior to study discontinuation, according to treatment assigned.

The number and proportion of ADA-positive patients and ADA-negative patients at baseline (baseline prevalence) and after baseline (post-baseline incidence) will be summarized by treatment group. When determining the post-baseline incidence, patients are considered to be ADA positive if they are ADA negative or are missing data at baseline but develop an ADA response following study drug exposure (treatment-induced ADA response), or if they are ADA positive at baseline and the titer of one or more post baseline samples is at least 4-fold (e.g., ≥0.60-titer unit) greater than the titer of the baseline sample (treatment-enhanced ADA response) considering drug concentration in individual post-dose sample. Patients are considered to be ADA negative if they are ADA negative or are missing data at baseline and all post-baseline samples are negative, or if they are ADA positive at baseline but do not have any post-baseline samples with a titer that is at least 4-fold (e.g., ≥0.60-titer unit considering drug concentration in individual post-dose sample) greater than the titer of the baseline sample (treatment unaffected).

The relationship between ADA status and safety, efficacy, PK, and biomarker endpoints may be analyzed and reported via descriptive statistics and exploratory PK and PK−PD analyses.

6.12 INTERIM ANALYSES

Dose escalations throughout the study will occur based on a safety review of the data. These data reviews do not classify as formal interim analysis. In addition, the study will have ongoing safety assessments and safety/preliminary efficacy data reviews which do not qualify as formal safety analyses. There will be ongoing PK analysis from Part I Cohort 1 onwards, and data from Part I will be assessed prior to commencement of Part II.

7. <u>DATA COLLECTION AND MANAGEMENT</u>

7.1 DATA QUALITY ASSURANCE

“Use of this document is governed by the terms of use on the first page of this document.”

The Sponsor will be responsible for data management of this study, including quality checking of the data. Sites will be responsible for data entry into the Electronic Data Capture (EDC) system.

A comprehensive validation check program will verify the data. Discrepancies will be generated automatically in the system at the point of entry or added manually for resolution by the Investigator.

The Sponsor will produce a Data Handling Manual that describes the quality checking to be performed on the data. Central laboratory data will be sent directly to the Sponsor, using the Sponsor's standard procedures to handle and process the electronic transfer of these data.

System backups for data stored by the Sponsor and records retention for the study data will be consistent with the Sponsor's standard procedures.

7.2 ELECTRONIC CASE REPORT FORMS

Data for this study will be captured via an online EDC system. The data collected in the source documents is entered onto the study eCRF. An audit trail will maintain a record of initial entries and changes made; reasons for change; time and date of entry; and user name of person authorizing entry or change. For each patient enrolled, an eCRF must be completed and electronically signed by the principal investigator or authorized delegate from the study staff. If a patient withdraws from the study, the reason must be noted on the eCRF. If a patient is withdrawn from the study because of a treatment–limiting adverse event, thorough efforts should be made to clearly document the outcome.

The investigator should ensure the accuracy, completeness, and timeliness of the data reported to the Sponsor in the eCRFs and in all required reports.

eCRFs will be submitted electronically to the Sponsor and should be handled in accordance with instructions from the Sponsor.

At the end of the study, the investigator will receive patient data for his or her site in a readable format on a compact disc that must be kept with the study records. Acknowledgement of receipt of the compact disc is required.

7.3 SOURCE DATA DOCUMENTATION

Study monitors will perform ongoing source data verification to confirm that critical protocol data (i.e., source data) entered into the eCRFs by authorized site personnel are accurate, complete, and verifiable from source documents.

Source documents (paper or electronic) are those in which patient data are recorded and documented for the first time. They include, but are not limited to, hospital records,

"Use of this document is governed by the terms of use on the first page of this document."

clinical and office charts, laboratory notes, memoranda, patient-reported outcomes, evaluation checklists, pharmacy dispensing records, recorded data from automated instruments, copies of transcriptions that are certified after verification as being accurate and complete, microfiche, photographic negatives, microfilm or magnetic media, X-rays, patient files, and records kept at pharmacies, laboratories, and medico-technical departments involved in a clinical trial.

Before study initiation, data to be entered directly into the eCRFs (i.e., no prior written or electronic record of the data) and considered source data must be defined in the Trial Monitoring Plan.

Source documents that are required to verify the validity and completeness of data entered into the eCRFs must not be obliterated or destroyed and must be retained per the policy for retention of records described in Section 7.5.

To facilitate source data verification, the investigators and institutions must provide the Sponsor direct access to applicable source documents and reports for trial-related monitoring, Sponsor audits, and IRB/EC review. The investigational site must also allow inspection by applicable health authorities.

7.4 USE OF COMPUTERIZED SYSTEMS

When clinical observations are entered directly into an investigational site's computerized medical record system (i.e., in lieu of original hardcopy records), the electronic record can serve as the source document if the system has been validated in accordance with health authority requirements pertaining to computerized systems used in clinical research. An acceptable computerized data collection system allows preservation of the original entry of data. If original data are modified, the system should maintain a viewable audit trail that shows the original data as well as the reason for the change, name of the person making the change, and date of the change.

7.5 RETENTION OF RECORDS

Records and documents pertaining to the conduct of this study and the distribution of IMP, including eCRFs, ICFs, laboratory test results, and medication inventory records, must be retained by the Principal investigator for 15 years after completion or discontinuation of the study, or for the length of time required by relevant national or local health authorities, whichever is longer. After that period of time, the documents may be destroyed, subject to local regulations. No records may be disposed of without the written approval of the Sponsor. Written notification should be provided to the Sponsor prior to transferring any records to another party or moving them to another location.

Roche will retain study data for 25 years after the final Clinical Study Report has been completed or for the length of time required by relevant national or local health authorities, whichever is longer.

"Use of this document is governed by the terms of use on the first page of this document."

8. ETHICAL CONSIDERATIONS

8.1 COMPLIANCE WITH LAWS AND REGULATIONS

This study will be conducted in full conformance with the ICH E6 guideline for GCP and the principles of the Declaration of Helsinki, or the *applicable* laws and regulations of the country in which the research is conducted, whichever affords the greater protection to the individual. The study will comply with the requirements of the ICH E2A guideline (Clinical Safety Data Management: Definitions and Standards for Expedited Reporting). Studies conducted in the United States or under a U.S. Investigational New Drug (IND) application will comply with U.S. Food and Drug Administration (FDA) regulations and applicable local, state, and federal laws. Studies conducted in the European Union (EU)/European Economic Area will comply with the EU Clinical Trial Directive (2001/20/EC) *and applicable local, regional, and national laws*.

8.2 INFORMED CONSENT

The Sponsor’s sample ICF will be provided to each site. If applicable, it will be provided in a certified translation of the local language. The Sponsor or its designee must review and approve any proposed deviations from the Sponsor's sample ICFs or any alternate consent forms proposed by the site (collectively, the “Consent Forms”) before IRB/EC submission. The final IRB/EC–approved Consent Forms must be provided to the Sponsor for health authority submission purposes according to local requirements.

The Consent Forms must be signed and dated by the patient or the patient’s legally authorized representative before his or her participation in the study. The case history or clinical records for each patient shall document the informed consent process and that written informed consent was obtained prior to participation in the study.

The Consent Forms should be revised whenever there are changes to study procedures or when new information becomes available that may affect the willingness of the patient to participate. The final revised IRB/EC-approved Consent Forms must be provided to the Sponsor for health authority submission purposes.

Patients must be re-consented to the most current version of the Consent Forms (or to a significant new information/findings addendum in accordance with applicable laws and IRB/EC policy) during their participation in the study. For any updated or revised Consent Forms, the case history or clinical records for each patient shall document the informed consent process and that written informed consent was obtained using the updated/revised Consent Forms for continued participation in the study.

A copy of each signed Consent Form must be provided to the patient or the patient’s legally authorized representative. All signed and dated Consent Forms must remain in each patient’s study file or in the site file and must be available for verification by study monitors at any time.

“Use of this document is governed by the terms of use on the first page of this document.”

For sites in the United States, each Consent Form may also include patient authorization to allow use and disclosure of personal health information in compliance with the U.S. Health Insurance Portability and Accountability Act of 1996 (HIPAA). If the site utilizes a separate Authorization Form for patient authorization for use and disclosure of personal health information under the HIPAA regulations, the review, approval, and other processes outlined above apply except that IRB review and approval may not be required per study site policies.

8.3 INSTITUTIONAL REVIEW BOARD OR ETHICS COMMITTEE

This protocol, the Informed Consent Forms, any information to be given to the patient and relevant supporting information must be submitted to the IRB/EC by the principal investigator and reviewed and approved by the IRB/EC before the study is initiated. In addition, any patient recruitment materials must be approved by the IRB/EC.

The principal investigator is responsible for providing written summaries of the status of the study to the IRB/EC annually or more frequently in accordance with the requirements, policies, and procedures established by the IRB/EC. Investigators are also responsible for promptly informing the IRB/EC of any protocol amendments (see Section 9.5).

In addition to the requirements for reporting all adverse events to the Sponsor, investigators must comply with requirements for reporting serious adverse events to the local health authority and IRB/EC. Investigators may receive written IND safety reports or other safety-related communications from the Sponsor. Investigators are responsible for ensuring that such reports are reviewed and processed in accordance with health authority requirements and the policies and procedures established by their IRB/EC, and archived in the site's study file.

8.4 CONFIDENTIALITY

The Sponsor maintains confidentiality standards by coding each patient enrolled in the study through assignment of a unique patient identification number. This means that patient names are not included in data sets that are transmitted to any Sponsor location.

Patient medical information obtained by this study is confidential and may only be disclosed to third parties as permitted by the ICF (or separate authorization for use and disclosure of personal health information) signed by the patient, unless permitted or required by law.

Medical information may be given to a patient's personal physician or other appropriate medical personnel responsible for the patient's welfare, for treatment purposes.

Data generated by this study must be available for inspection upon request by representatives of the U.S. FDA and other national and local health authorities, Sponsor

"Use of this document is governed by the terms of use on the first page of this document."

monitors, representatives, and collaborators, and the IRB/EC for each study site, as appropriate.

8.5 FINANCIAL DISCLOSURE

Investigators will provide the Sponsor with sufficient, accurate financial information in accordance with local regulations to allow the Sponsor to submit complete and accurate financial certification or disclosure statements to the appropriate health authorities. Investigators are responsible for providing information on financial interests during the course of the study and for one year after completion of the study (i.e., last patient last visit).

9. STUDY DOCUMENTATION, MONITORING, AND ADMINISTRATION

9.1 STUDY DOCUMENTATION

The investigator must maintain adequate and accurate records to enable the conduct of the study to be fully documented, including but not limited to the protocol, protocol amendments, ICFs, and documentation of IRB/EC and governmental approval.
In addition, at the end of the study, the investigator will receive the patient data, which includes an audit trail containing a complete record of all changes to data.

The Sponsor shall also submit an Annual Safety Report once a year to the IEC and Competent Authorities according to local regulatory requirements and timelines of each country participating in the study.

Sampling for the RCR is contingent on review and approval for the exploratory biomarker assessments and written informed consent by an appropriate regulatory body (depending on the country where the study is performed) and a site's IRB/EC. If a regulatory or site's IRB/EC does not approve the sampling for the exploratory assessments the section on biomarker sampling will not be applicable.

It is the understanding of the Sponsor that this protocol (and any modifications) as well as appropriate consent procedures and advertisements, will be reviewed and approved by an IRB. This board must operate in accordance with the current Federal Regulations. The Sponsor will be sent a letter or certificate of approval prior to initiation of the study, and also whenever subsequent amendments /modifications are made to the protocol. The Sponsor shall also submit an IND Annual Report to FDA according to local regulatory requirements and timelines.

9.2 SITE INSPECTIONS

Site visits will be conducted by the Sponsor or an authorized representative for inspection of study data, patients' medical records, and eCRFs. The investigator will permit national and local health authorities, Sponsor monitors, representatives, collaborators, and the IRBs/ECs to inspect facilities and records relevant to this study.

"Use of this document is governed by the terms of use on the first page of this document."

9.3 ADMINISTRATIVE STRUCTURE

The Sponsor of the trial is F. Hoffmann-La Roche Ltd. The Sponsor is responsible for the study management (monitoring will be outsourced to a CRO), data management, statistical analysis, and medical writing for the clinical study report (CSR). The CSR will be written and submitted to Health Authorities in the timeframe as required by applicable regulatory requirements.

The protocol will be submitted to country's IRB/EC.

9.4 PUBLICATION OF DATA AND PROTECTION OF TRADE SECRETS

The results of this study may be published or presented at scientific meetings. If this is foreseen, the investigator agrees to submit all manuscripts or abstracts to the Sponsor prior to submission. This allows the Sponsor to protect proprietary information and to provide comments based on information from other studies that may not yet be available to the investigator.

The Sponsor will comply with the requirements for publication of study results.
In accordance with standard editorial and ethical practice, the Sponsor will generally support publication of multicenter trials only in their entirety and not as individual center data. In this case, a coordinating investigator will be designated by mutual agreement.

Any formal publication of the study in which contribution of Sponsor personnel exceeded that of conventional monitoring will be considered as a joint publication by the investigator and the appropriate Sponsor personnel.

General Guidelines

- Authorship will follow the guidelines for the target journal. This especially considering the maximum number of authors permitted for the target journal.
- The maximum number of authors will usually be included.
- The investigator who recruits the highest number of evaluable patients may choose to be the first or the last author and will be presenter/corresponding author on the main publication.
- Roche authors should not outnumber non-Roche authors and usually should not exceed three.
- Roche authors should not be first author.

Publication

The following are based on general rules for Roche–sponsored oncology studies:

- Subsets per country can be published by the country's primary investigator, after the main publication and in accordance with the requirements of the study contract held with the site

"Use of this document is governed by the terms of use on the first page of this document."

- Acknowledgement of supporting bodies and other investigators must be made
- Any manuscript must be passed through the Sponsor for review prior to submission. The Sponsor shall review such manuscripts within 30 working days
- Authorship will be in line with International Committee of Medical Journal Editors authorship requirements.

Any inventions and resulting patents, improvements, and/or know-how originating from the use of data from this study will become and remain the exclusive and unburdened property of the Sponsor, except where agreed otherwise.

9.5 PROTOCOL AMENDMENTS

Any substantial protocol amendments will be prepared by the Sponsor. Substantial protocol amendments will be submitted to the IRB/EC and to regulatory authorities in accordance with local regulatory requirements.

Approval must be obtained from the IRB/EC and regulatory authorities (as locally required) before implementation of any changes, except for changes necessary to eliminate an immediate hazard to patients or any non-substantial changes, as defined by regulatory requirements.

"Use of this document is governed by the terms of use on the first page of this document."

10. REFERENCES

Affara NI, Ruffell B, Medler TR, et al. B cells regulate macrophage phenotype and response to chemotherapy in squamous carcinomas. Cancer Cell 2014; 25(6):809-21.

Aklilu M, Stadler WM, Markiewicz M, et al Depletion of normal B cells with rituximab as an adjunct to IL-2 therapy for renal cell carcinoma and melanoma Ann Oncol. 2004 Jul;15(7):1109-14.

Ashraf SQ, Umana P, Mössner E, et al. Humanised IgG1 antibody variants targeting membrane-bound carcinoembryonic antigen by antibody-dependent cellular cytotoxicity and phagocytosis. Br J Cancer 2009;101:1758–68.

Bailey S, Neuenschwander B, Laird G, Branson M. A Bayesian Case Study in Oncology: Phase I Combination Dose-Finding Using Logistic Regression with Covariates. J Biopharm Stat 2009;19:469–84.

Banugaria SG, Prater SN, Patel TT, DeArmey SM, Milleson C, et al. (2013) Algorithm for the Early Diagnosis and Treatment of Patients with Cross Reactive Immunologic Material-Negative Classic Infantile Pompe Disease: A Step towards Improving the Efficacy of ERT. PLoS ONE 8(6): e67052. doi:10.1371/ journal.pone.0067052.

Bengtsson, T., Sanabria-Bohorquez, S. M., McCarthy, T. J., Binns, D. S., Hicks, R. J., & De Crespigny, A. J. (2015). STatistically Assigned Response Criteria in Solid Tumors (STARCIST). Cancer Imaging, 15(1), 1–11. http://doi.org/10.1186/s40644-015-0042-4.

Bodogai M, Lee Chang C, Wejksza K, et al Anti-CD20 Antibody Promotes Cancer Escape via Enrichment of Tumor-Evoked Regulatory B Cells Expressing Low Levels of CD20 and CD137L 2013 Cancer Res 2013; 73(7); 2127–38.

Teachey DT, Rheingold SR, Maude SL, et al. Cytokine release syndrome after blinatumomab treatment related to abnormal macrophage activation and ameliorated with cytokine-directed therapy. Blood. 2013;121:5154–7.

Conrad ML, Davis WC, Koop BF. TCR and CD3 antibody cross-reactivity in 44 species. Cytometry Part A.71A: 925933, 2007.

De Benedetti F, Brunner HI, Ruperto N, Kenwright A, Wright S, et al. Randomized trial of tocilizumab in systemic juvenile idiopathic arthritis. N Engl J Med. 2012;367(25):2385-95.

de la Hera A, Mueller U, Olsson C, et al. Structure of the T cell Antigen Receptor (TCR): Two CD3e Subunits in a Functional TCR/CD3 Complex. J Exp Med 1991;173:7–17.

Doessegger L, Banholzer ML. Clinical development methodology for infusion-related reactions with monoclonal antibodies. Clin Transl Immunology. 2015;4:e39.

“Use of this document is governed by the terms of use on the first page of this document.”

Durbin H, Young S, Stewart LM, et al. An epitope on carcinoembryonic antigen defined by the clinically relevant antibody PR1A3. Proc Natl Acad Sci U S A. 1994;91:4313–7.

European Medicines Agency: Assessment Report for Removab (catumaxomab); Document Reference: EMEA/CHMP/100434/2009. 2009 [resource on the Internet] London, UK. Available at: http://www.ema.europa.eu/docs/en_GB/document_library/EPAR_-_Public_assessment_report/human/000972/WC500051808.pdf

European Medicines Agency (EMA). Summary of opinion (post authorisation): RoActemra (tocilizumab). Committee for Medicinal Products for Human Use (CHMP); EMA/CHMP/419360/2018 (28 June 2018).

Fiedler WM, Ritter B, Seggewiss R, et al. Phase I safety and pharmacology study of the EpCAM/CD3-bispecific BiTE antibody MT110 in patients with metastatic colorectal, gastric, or lung cancer. J Clin Oncol 2010;28:7s(suppl; abstr 2573).

Gomez-Mantila JD, Troconiz IF Review on modeling anti-antibody responses to monoclonal antibodies. J Pharmacokinet Pharmacodyn 2014;41:523-536.

Grupp SA, Kalos M, Barrett D, Aplenc R, Porter DL, et al. Chimeric antigen receptor-modified T cells for acute lymphoid leukemia. N Engl J Med. 2013; 368(16):1509-18.

Gunderson AJ, Coussens LM. B cells and their mediators as targets for therapy in solid tumors. Exp Cell Res 2013; 319(11):1644-9.

Hoos A, Eggermont AM, Janetzki S, et al. Improved endpoints for cancer immunotherapy trials. J Natl Cancer Inst 2010:102:1388–97.

Kang SH, Ahn C. An investigation of the traditional algorithm-based designs for phase I cancer clinical trials. Drug Information J 2002;36:865–73.

Kang SH, Ahn C. The expected toxicity rate at the maximum tolerated dose in the standard phase I cancer clinical trial design. Drug Information J 2001;35:1189–1200.

Kim S, Fridlender ZG, Dunn R, et al B-cell Depletion Using an Anti-CD20 Antibody Augments Antitumor Immune Responses and Immunotherapy in Nonhematopoetic Murine Tumor Model J Immunother 2008;31:446–457.

Le RQ, Li L, Yuan W, et al. FDA Approval Summary: Tocilizumab for Treatment of Chimeric Antigen Receptor T Cell ‐Induced Severe or Life ‐Threatening Cytokine Release Syndrome. Oncologist 2018; 23:943–7.

Le Tourneau C, Lee JJ, Siu LL. Dose escalation methods in phase I cancer clinical trials. J Natl Cancer Inst 2009;101:708–20.

“Use of this document is governed by the terms of use on the first page of this document.”

Lee DW, Gardner R, Porter DL, et al. Current concepts in the diagnosis and management of cytokine release syndrome. Blood. 2014;124:188–95.

Liersch T, Meller J, Kulle B, et al. Phase II trial of carcinoembryonic antigen radioimmunotherapy with 131I-labetuzumab after salvage resection of colorectal metastases in the liver: five-year safety and efficacy results. J Clin Oncol 2005;23:6763–70.

Mendelsohn NJ, Messinger YH, Rosenberg AS, et al Elimination of Antibodies to Recombinant Enzyme in Pompe's Disease NEJM 2009, 360:2, 194-195.

Messinger YH, Mendelsohn NJ, Rhead W, et al Successful immune tolerance induction to enzyme replacement therapy in CRMI-negative infantile Pompe disease Genet Med 2012:14(1)135-142.

Mössner E, Brünker P, Moser S, et al. Increasing the efficacy of CD20 antibody therapy through the engineering of a new type II anti-CD20 antibody with enhanced direct and immune effector cell–mediated B-cell cytotoxicity. Blood. 2010;115:4393-4402.

Nagorsen D, Bargou R, Ruttlinger D, et al. Immunotherapy of lymphoma and leukemia with T-cell engaging BiTE antibody blinatumomab. Leuk Lymphoma 2009;50:886–91.

Nagorsen D, Kufer P, Patrick A, et al. Blinatumomab: A historical perspective. Pharmacology & Therapeutics 2012;136:334–342.

National Institutes of Health (NIH). Recombinant DNA Advisory Committee (RAC). Cytokine Release Syndrome after T Cell Immunotherapy. Bethesda, Maryland. NIH Videocast 9 June 2015. Available at: https://videocast.nih.gov/summary.asp?Live=16420&bhcp=1. Accessed 30 January 2017.

Neuenschwander, B., Branson, M., & Gsponer, T. (2008). Critical aspects of the Bayesian approach to phase I cancer trials. Statistics in Medicine, 27(13), 2420–39.

Nishino M, Gargano M, Suda M, et al. Optimizing immune-related tumor response assessment: does reducing the number of lesions impact response assessment in melanoma patients treated with ipilimumab? J Immunother Cancer 2014;2:17.

Nishino M, Giobbie-Hurder A, Gargano M, et al. Developing a common language for tumor response to immunotherapy: immune-related response criteria using unidimensional measurements. Clin Cancer Res 2013;19:3936–4.

Ordoñez C, Screaton RA, Ilantzis C, Stanners CP. Human carcinoembryonic antigen functions as a general inhibitor of anoikis. Cancer Res 2000;60:3419–24.

"Use of this document is governed by the terms of use on the first page of this document."

Orthoclone OKT®3 [package insert]. Raritan, NJ: Ortho Biotech Products, L.P.; 2001.

Panelli MC, White R, Foster M, et al. Forecasting the cytokine storm following systemic interleukin (IL)-2 administration. J Transl Med. 2004;2:17.

Pessano S, Oettgen H, Bhan AK, et al. The T3/T cell receptor complex: antigenic distinction between the two 20-kd T3 (T3-delta and T3-epsilon) subunits. EMBO J. 1985;4:337–44.

Plummer M (2016). rjags: Bayesian Graphical Models using MCMC. R package version 4-6. URL https://CRAN.R-project.org/package=rjags

Roche Report No.1072962, 2016. Quantitative in vivo imaging and biodistribution of CEA-targeted T-cell bispecific antibodies in tumor bearing CD34+ human hematopoetic stem cell engrafted NSG mice.

Rudert F, Saunders AM, Rebstock S, et al. Characterization of murine carcinoembryonic antigen gene family members. Mamm Genome 1992;3:262–73.

Russell et al. Vasopressin versus norepinephrine infusion in patients with septic shock. N Engl J Med 2008;358:877–88.

Salmerón A, Sanchez-Madrid F, Ursa MA, et al. A conformational epitope expressed upon association of CD3e with either CD3d or CD3g is the main target for recognition by anti-CD3 monoclonal antibodies. J Immunol 1991;147:3047–52.

Sauerborn M, Beers van M.C.M.M, Jiskoot W,. et al Antibody Response Against Betaferon® in Immune Tolerant Mice: Involvement of Marginal Zone B-cells and CD4+ T-cells and Apparent Lack of Immunological Memory J Clin Immunol 2013; 33:255–263.

Sebastian M, Passlick B, Friccius-Quecke H, et al. Treatment of non-small cell lung cancer patients with the trifunctional monoclonal antibody catumaxomab (anti-EpCAM × anti-CD3): a phase I study. Cancer Immunol Immunother 2007;56:1637–44.

Schmidli, H., Gsteiger, S., Roychoudhury, S., O'Hagan, A., Spiegelhalter, D., & Neuenschwander, B. Robust meta-analytic-predictive priors in clinical trials with historical control information. Biometrics 2014;70(4): 1023-32.

Shankar L et al. JNM 2006 47: 1059-1066.

Singh JA, Beg S, Lopez-Olivo MA. Tocilizumab for rheumatoid arthritis: a Cochrane systematic review. J Rheumatol. 2011;38(1):10-20.

"Use of this document is governed by the terms of use on the first page of this document."

Soeth E, Wirth T, List HJ, et al. Controlled ribozyme targeting demonstrates an antiapoptotic effect of carcinoembryonic antigen in HT29 colon cancer cells. Clin Cancer Res 2001;7:2022–30.

Tabernero et al. Clinical evidence of intra-tumoral immune activation and tumor targeting with RG7813, a CEA-targeted engineered IL-2 immunocytokine. ESMO 2015 oral presentation. https://www.ecco-org.eu/Vienna2015/Scientific-Programme/Searchable-Programme?trackid=00179#anchorScpr

Teachey DT, Rheingold SR, Maude SL, et al. Cytokine release syndrome after blinatumomab treatment related to abnormal macrophage activation and ameliorated with cytokine-directed therapy. Blood 2013 Jun 27;121(26):5154-7.

Teachey DT, Lacey SF, Shaw PA, et al. Identification of Predictive Biomarkers for Cytokine Release Syndrome after Chimeric Antigen Receptor T cell Therapy for Acute Lymphoblastic Leukemia. Cancer Discov. 2016;6:664-679.

Thompson JA, Grunert F, Zimmermann W. Carcinoembryonic antigen gene family: molecular biology and clinical perspectives. J Clin Lab Anal 1991;5:344–66.

Tocilizumab (Actemra®) United States Package Insert. Available at: https://www.gene.com/download/pdf/actemra_prescribing.pdf, accessed on 17 September 2017.

Weber WA. Assessing tumor response to therapy. J Nucl Med. 2009 May;50 Suppl 1:1S-10S.

Weiner GJ: Making a better antibody: all is not lost, Blood 2010, 115:5127-5128.

Wolchok JD, Hoos A, O'Day S, et al. Guidelines for the evaluation of immune therapy activity in solid tumors: immune-related response criteria. Clin. Cancer Res 2009;15:7412–20.

Woo P, Wilkinson N, Prieur AM, Southwood T, Leone V, Livermore P, Wythe H, Thomson D, Kishimoto T. Open label phase II trial of single, ascending doses of MRA in Caucasian children with severe systemic juvenile idiopathic arthritis: proof of principle of the efficacy of IL-6 receptor blockade in this type of arthritis and demonstration of prolonged clinical improvement. Arthritis Res Ther. 2005;7(6):R1281-8.

Yokota S, Miyamae T, Imagawa T, Iwata N, Katakura S, et al. Therapeutic efficacy of humanized recombinant anti-interleukin-6 receptor antibody in children with systemic-onset juvenile idiopathic arthritis. Arthritis Rheum. 2005 Mar;52(3):818-25.

Young H et al. Eur J Cancer 1999;35 (13):1773-1782.

"Use of this document is governed by the terms of use on the first page of this document."

Appendix 1
Schedule of Assessments

TABLE A1: SCHEDULE OF ASSESSMENTS FOR PART I

Cycle	Screening	Cycle 1				Cycle 2–3			Cycle X	End of Treatment	28 Days Safety Follow Up[m]
Day	D-28 to D-1	Day 1[p]	Day 2	Day 3	Day 6	Day 1	Day 2	Day 6	Day 1		
Assessments[a]											
Informed Consent[b]	X										
Eligibility	X										
Demography	X										
Medical History	X										
Physical Examination[c]	X	X				X			X	X	X
Anthropometric Measurements[d]	X	X	X	X	X	X	X	X	X	X	X
Vital Signs	X	X	X	X	X	X	X	X	X	X	X
Administration of Study Medication		X				X			X		
ECG-12 lead[e]	X	X				X			X		X
ECOG Performance Status	X	X				X			X		X
Pregnancy Test[f]	X								X[n]	X[o]	X
PK Sample		X	X	X	X	X			X	X	X
PD Blood Cytokines[g]		X	X	X	X	X	X		X		
Anti-Drug Antibody (ADA)[h]		X			X	X			X	X	X
Hematology[i]	X	X	X	X	X	X	X	X	X	X	X
Blood Chemistry[i]	X	X	X	X	X	X	X	X	X	X	X
Coagulation[ij]	X	X	X	X	X	X	X	X	X	X	X
Urinalysis[i]	X	X	X	X	X	X	X	X	X	X	X
CEA confirmation on Archival Tissue[k]	X										
Tumor Assessment[l]	X								X	X	
Adverse Events	X										
Previous and Concomitant Treatments	X										

"Use of this document is governed by the terms of use on the first page of this document."

Appendix 1
Schedule of Assessments (cont.)

Notes:

ADA = anti-drug antibody; CEA = carcinoembryonic antigen; D = day; ECOG = Eastern Cooperative Oncology Group; PD = pharmacodynamics; PK = pharmacokinetic.

a. All visits and their assigned safety assessments (hematology, blood chemistry, coagulation, urinalysis, physical examination) (unless otherwise indicated) should occur within a 3–day time window
b. Informed consent must be obtained before any study-specific procedures
c. Physical examinations (including weight) and ECOG Performance Status will be done at screening, at the time of treatment administration, and at the 28–day safety follow-up. Results must be obtained prior to infusion.
d. Weight only will be collected as part of anthropometric measurements on non-infusion days.
e. Triplicate 12-lead ECG at screening (within 7 days before first dose of RO6958688) pre- and end of infusion on Cycle 1 Day 1 and Cycle 3 Day 1 and at the 28–day safety follow-up visit. Pre-infusion at all other study drug administrations. Additional unscheduled ECG assessments should be performed in case of abnormalities and if clinical symptoms occur. Recording must be done prior to PK sampling.
f. Serum pregnancy test at screening, within 7 days prior to first dose.
g. Serum samples for the assessment of cytokine (PD blood cytokines) release will be collected. At the time of an IRR, see IRR SoA Table A9.
h. For ADA at the time of an IRR, see IRR SoA Table A9.
i. Hematology, blood chemistry, coagulation, and urinalysis can be performed up to a maximum 72 hours prior to schedule dosing. Results must be obtained prior to infusion. Serum CEA will be measured by the site as part of the blood chemistry measurements on Cycle 1 Day 1 and every 6 weeks thereafter for patients continuing treatment.
j. For coagulation (including PT/INR and PTT), at the time of an IRR see IRR SoA Table A9.
k. As part of the eligibility procedure, CEA expression will be confirmed locally on archival tumor material, if available. If no archival tumor sample, fresh frozen sample will be collected which will be assessed centrally.
l. Measurable lesions will be assessed at screening, then at 12 weeks after Cycle 1 Day 1, and every 8 weeks thereafter for the first year, and every 12 weeks thereafter until disease progression or treatment discontinuation. Optional latest pre-study CT scan should be provided for assessment of tumor growth kinetics within 6 weeks of patient entering the study. For the scheduled tumor assessments beyond Screening a ± 7-day window is permitted.
m. Patients who complete the study or discontinue from the study early will be asked to return to the clinic 28 days after the last dose of study drug for a post-study follow-up.
n. Serum or urine pregnancy test to be done on Day 1 of Cycle 4 and on Day 1 of every fourth cycle thereafter or monthly.
o. Serum or urine pregnancy test to be done if 14 days or more after latest test.
p. At least 24 hours (overnight) hospital stay required following administration of study drug at Cycle 1 Day 1.

"Use of this document is governed by the terms of use on the first page of this document."

TABLE A2: SCHEDULE OF HOURLY ASSESSMENTS FOR PART I

Cycle	Day	Scheduled Time (h)	Vital Signs[a]	ECG-12 lead[b]	PK Sample	PD Blood Cytokines[c]	Anti-Drug Antibody (ADA)[d]
Cycle 1	Day 1	Pre-infusion	X	X	X	X	X
		End of Infusion	X	X	X	X	
		2 Hours Post Infusion	X		X	X	
	Day 2	24 Hours			X	X	
		Anytime	X				
	Day 3	48 Hours			X	X	
		Anytime	X				
	Day 6	120 Hours			X	X	X
		Anytime	X				
Cycle 2–3	Day 1	Pre-infusion	X	X	X	X	X
		End of Infusion	X	X[b]	X	X	
		2 Hours Post Infusion	X		X		
	Day 2	Anytime	X			X	
	Day 6	Anytime	X				
Cycle X	Day 1	Pre-infusion	X	X	X	X	X
		End of Infusion			X	X	
		2 Hours Post Infusion			X		
End of Treatment		Anytime	X		X		X
28–Day Safety Follow Up		Anytime	X	X	X		X

"Use of this document is governed by the terms of use on the first page of this document."

Appendix 1
Schedule of Assessments (cont.)

Notes:

ADA = anti-drug antibody; h = hour; PD = pharmacodynamics; PK = pharmacokinetic.

a. Vital signs (including supine blood pressure and heart rate) will be monitored on Day 1 pre-infusion, every 15 minutes until the end of infusion and, thereafter, every 30 minutes until the infusion line is removed. Starting Cycle 3: pre-infusion, every 30 minutes during infusion and every 30 minutes after the end of infusion until infusion line is removed. From Cycle 5 onwards vital signs will only be obtained on Day 1 pre-infusion. For the purpose of the eCRF, vital signs will only be captured pre-infusion and in case of abnormalities.

b. Triplicate 12-lead ECG at screening (within 7 days before first dose of RO6958688) pre- and end of infusion on Cycle 1 Day 1 and Cycle 3 Day 1 and at the 28–day safety follow-up visit. Pre-infusion at all other study drug administrations. Additional unscheduled ECG assessments should be performed in case of abnormalities and if clinical symptoms occur. Recording must be done prior to PK sampling.

c. Serum samples for the assessment of cytokine (PD blood cytokines) release will be collected. At the time of an IRR see IRR SoA Table A9.

d. For ADA at the time of an IRR see IRR SoA Table A9.

"Use of this document is governed by the terms of use on the first page of this document."

Appendix 1
Schedule of Assessments (cont.)

TABLE A3: SCHEDULE OF ASSESSMENTS FOR PART II / QW

Cycle = one week (QW)	Screening	Cycle 1				Cycle 2			Cycle 3			Cycle 4			Cycle X	End of Treatment	28–Day Safety Follow Up[n]
Day	D-28 to D-1	Day 1[q]	Day 2	Day 3	Day 6	Day 1	Day 2	Day 6	Day 1	Day 2	Day 6	Day 1	Day 2	Day 6	Day 1		
Assessments[a]																	
Informed Consent[b]	X																
Eligibility	X																
Demography	X																
Medical History	X																
Physical Examination[c]	X	X				X			X			X			X		X
Anthropometric Measurements[d]	X	X	X	X	X	X	X	X	X	X	X	X	X	X	X		X
Vital Signs	X[cc]	X	X	X	X	X	X	X	X	X	X	X	X	X	X	X	X
Administration of RO6958688 QW		X				X			X			X			X		
Administration of obinutuzumab	X[x]																
ECG-12 lead[e]	X	X				X			X			X			X		X
ECOG Performance Status	X	X				X			X			X			X		X
Pregnancy Test[f]	X														X[o]	X[p]	X
PK Sample RO6958688		X[g]	X	X	X	X[g]	X	X	X[g]			X[g]			X[g]	X	X
PD Blood Flow Cytometry	X[s]	X	X			X											
PD Blood Cytokines[h]		X	X	X	X	X	X		X	X							

"Use of this document is governed by the terms of use on the first page of this document."

Appendix 1
Schedule of Assessments (cont.)

TABLE A3: SCHEDULE OF ASSESSMENTS FOR PART II / QW (CONT.)

Cycle = one week (QW)	Screening	Cycle 1				Cycle 2			Cycle 3			Cycle 4			Cycle X	End of Treatment	28 Days Safety Follow Up[n]
Day	D-28 to D-1	Day 1[q]	Day 2	Day 3	Day 6	Day 1	Day 2	Day 6	Day 1	Day 2	Day 6	Day 1	Day 2	Day 6	Day 1		
Anti-Drug Antibody (ADA) RO6958688		X				X			X			X			X[ee]	X	X
PBMC B-cell isolation (ADA specificity assessment)[w]		X													X		
PK Sample obinutuzumab	X[t]	X				X						X			X[u]		
TCR Vβ[y]		X															
Soluble CEA		X															
Hematology[i]	X[v]	X	X	X	X	X	X	X	X	X	X	X	X	X	X	X	X
Blood Chemistry[i]	X[v]	X	X	X	X	X	X	X	X	X	X	X	X	X	X	X	X
Coagulation[ij]	X	X	X	X	X	X	X	X	X	X	X	X	X	X	X	X	X
Urinalysis[i]	X	X	X	X	X	X	X	X	X	X	X	X	X	X	X	X	X
Hepatitis B and C serology	X[dd]																
Tumor Biopsy	X[k]														X[k]		
Clinical Genotyping		X															
RCR Sample (DNA and RNA)		X															
Archival Tissue[l]	X																

"Use of this document is governed by the terms of use on the first page of this document."

Appendix 1
Schedule of Assessments (cont.)

TABLE A3: SCHEDULE OF ASSESSMENTS FOR PART II / QW (CONT.)

Cycle = one week (QW)	Screening	Cycle 1				Cycle 2			Cycle 3			Cycle 4			Cycle X	End of Treatment	28 Days Safety Follow Up[n]
Day	D-28 to D-1	Day 1[q]	Day 2	Day 3	Day 6	Day 1	Day 2	Day 6	Day 1	Day 2	Day 6	Day 1	Day 2	Day 6	Day 1		
FDG-PET[r]	X														X		
Tumor Assessment[m]	X														X	X	
DL_{CO} / FEV1/VC/TLC [bb]	X																
Adverse Events	X																
Previous and Concomitant Treatments	X																

"Use of this document is governed by the terms of use on the first page of this document."

Appendix 1
Schedule of Assessments (cont.)

Notes: ADA = anti-drug antibody; CEA = carcinoembryonic antigen; D = day; ECOG = Eastern Cooperative Oncology Group; PD = pharmacodynamics; PK = pharmacokinetic; RCR = Roche Clinical Repository.

a. All visit and their assigned safety assessments (hematology, blood chemistry, coagulation, urinalysis, physical examination) (unless otherwise indicated) should occur within a 3–day time window
b. Informed consent must be obtained before any study-specific procedures
c. Physical examinations (including weight) and ECOG Performance Status will be done at screening, at the time of treatment administration, and at the 28–day safety follow-up. Results must be obtained prior to infusion.
d. Weight only will be collected as part of anthropometric measurements on non-infusion days.
e. Triplicate 12-lead ECG at screening (within 7 days before first dose of RO6958688) pre- and end of infusion on Cycle 1 Day 1 and Cycle 3 Day 1 and at the 28-day safety
f. Serum pregnancy test at screening, within 7 days prior to first dose.
g. PK pre-dose, end infusion, and 2 hours post End of infusion at Cycles 1, 2, 3, and 4. From Cycle 5 to Cycle 29, RO6958688 PK samples will be obtained pre-infusion and at end of infusion on Day 1 of each cycle. At Cycle 30 and every 6th Cycle, RO6958688 PK samples will be obtained pre-infusion and end of infusion on Day 1 at the mentioned timepoints.
h. Serum or plasma samples for the assessment of cytokine (PD blood cytokines) release will be collected. At the time of an IRR, please see IRR SoA Table A9.
i. Hematology, blood chemistry, coagulation, and urinalysis can be performed within 24 hours (up to 72 hours if during weekend) prior to schedule dosing. Results must be obtained prior to infusion. Soluble CEA will be measured by the site as part of the blood chemistry measurements on Cycle 1 Day 1 and every 6 weeks thereafter for patients who continue treatment.
j. For coagulation (including PT/INR and PTT), at the time of an IRR please see IRR SoA Table A9.
k. If archival tumor tissue is NOT available for CEA assessment Tumor Biopsy will be required for CEA. Tumor biopsy samples will be collected on two occasions after the FDG PET (once at baseline to confirm eligibility and once during the study treatment period) for patients who will be treated at dose levels equal and greater than 5 mg ($\geq$ 5 mg). All on-treatment tumor biopsies will be collected as per Section 3.2.5 but after the FDG-PET scan if feasible.
l. Archival tissue, if available, should be submitted for additional biomarker assessments for all patients enrolled in Part II, and who have consented in such tumor biopsies in case they are receiving a dose level equal or greater than 5 mg, the 28-day screening period does not apply to the CEA testing.
m. Measurable lesions will be assessed at screening, then at 8 weeks after Cycle 1 Day 1, and every 8 weeks thereafter for the first year, and every 12 weeks thereafter until disease progression or treatment discontinuation. Optional latest pre-study CT scan should be provided for assessment of tumor growth kinetics within 6 weeks of patient entering the study. For the scheduled tumor assessments beyond Screening a (± 7day window is permitted)

“Use of this document is governed by the terms of use on the first page of this document.”

Appendix 1
Schedule of Assessments (cont.)

n. Patients who complete the study or discontinue from the study early will be asked to return to the clinic 28 days after the last dose of study drug for a post-study follow-up visit.

o. Serum or urine pregnancy test to be done on Day 1 of Cycle 5 and on Day 1 of every fourth cycle thereafter or monthly.

p. Serum or urine pregnancy test to be done if 14 days or more after latest test.

q. At least 24 hours (overnight) hospital stay required following administration of study drug at Cycle 1 Day 1

r. FDG-PET must be done at baseline (Day-14 to D-1) before the baseline biopsy. The on-treatment FDG-PET must be done at (C6D5 -72/0 hours) and before the on-treatment tumor biopsy. Refer to Appendix 9 for further information.

s. PD blood samples at baseline to be obtained only from patients pretreated with obinutuzumab. Baseline sample should be taken during the screening period prior to administration of obinutuzumab.

t. PK samples for obinutuzumab to be drawn pre-dose and end of infusion at Day-13 if obinutuzumab is administered as a single infusion or pre-dose and end of infusion at Day-13 and Day-12 if obinutuzumab is administered as split infusions during 2 consecutive days.

u. PK samples for obinutuzumab to be drawn pre-dose and end of infusion at Cycle 8 Day 1 and Cycle 12 Day 1.

v. Hematology and blood chemistry to be assessed prior to obinutuzumab infusion and prior to first infusion of RO6958688.

w. Whole blood sample to be drawn at Cycle 1 Day 1 predose, Cycle 7 Day 1 predose and Cycle 12 Day 1 predose in patients participating at this ADA assessment.

x. Obinutuzumab will be administered either on Day-13 (2000 mg) or on Day-13 and Day-12 (1000 mg each day).

y. TCR Vβ samples to be drawn at Cycle 1 Day 1 predose (baseline).

z. [Footnote no longer used]

aa. Soluble CEA samples to be drawn pre-dose at Day 1 of Cycle 1.

bb. FEV1/VC/TLC and DL_{CO} (corrected for both alveolar volume and hemoglobin) will be evaluated at screening in patients with bilateral lung metastases or patients with lobectomy or pneumonectomy with lung metastases in the remaining lung. Additional tests may be performed if clinically indicated. The same pulmonary function testing laboratory should be used throughout the study.

cc. Vital signs including oxygen saturation

dd. Hepatitis B and C serology is mandatory only for patients receiving obinutuzumab pretreatment.

ee. From Cycle 5 to Cycle 29, RO6958688 ADA samples will be collected pre-infusion on Day 1 of each cycle. At Cycle 30 and every 6th Cycle, ADA samples will be collected pre-infusion on Day 1 at the mentioned timepoints.

"Use of this document is governed by the terms of use on the first page of this document."

Appendix 1
Schedule of Assessments (cont.)
Table A4: Schedule of Hourly Assessments for Part II / QW

Cycle	Day	Scheduled Time (h)	Vital Signs[a]	ECG-12 lead[b]	RO6958688 PK Sample[q]	PD Blood Flow Cytometry	PD Blood Cytokines[c]	RO6958688 Anti-Drug Antibody (ADA)[d]	TCR Vβ[m]	Tumor Biopsy	Clinical Genotyping	RCR Sample (DNA and RNA)	FDG-PET	Obinutuzumab PK Sample	ADA specificity assessment[l]	Soluble CEA[p]
Screening		D-28 to D-1[g]	x	x		x[i]				x[e]			x[f]	x[j]		
Cycle 1	Day 1	Pre-infusion	x	x	x	x	x	x	X		x	x		x	x	x
		EOI	x	x	x		x									
		2 Hours Post EOI			x		x									
		4-6 Hours Post EOI					x									
	Day 2	24 Hours	x		x	x	x									
	Day 3	48 Hours	x		x		x									
	Day 6	120 Hours	x		x		x									
Cycle 2	Day 1	Pre-infusion	x	x	x	x	x	x						x		
		End of Infusion	x		x		x									
		2 Hours Post End of Infusion			x		x									
	Day 2	24 Hours	x		x		x									
	Day 6	120 Hours	x		x											

"Use of this document is governed by the terms of use on the first page of this document."

Appendix 1
Schedule of Assessments (cont.)

Table A4: Schedule of Hourly Assessments for Part II / QW (cont.)

Cycle	Day	Scheduled Time (h)	Vital Signs[a]	ECG-12 lead[b]	RO6958688 PK Sample	PD Blood Flow Cytometry	PD Blood Cytokines[c]	RO6958688 Anti-Drug Antibody (ADA)[d]	TCR Vβ[m]	Tumor Biopsy	Clinical Genotyping	RCR Sample (DNA and RNA)	FDG-PET	Obinutuzumab PK Sample	ADA specificity assessment[l]	Soluble CEA[p]
Cycle 3	Day 1	Pre-infusion	x	x	x		x	x								
		End of Infusion	x	x	x		x									
		2 Hours Post End of Infusion			x											
	Day 2	24 Hours	x				x									
	Day 6	120 Hours	x													
Cycle 4	Day 1	Pre-infusion	x	x	x			x						x		
		End of Infusion	x		x											
		2 Hours Post End of Infusion			x											
	Day 2	24 Hours	x													
	Day 6	120 Hours	x													

“Use of this document is governed by the terms of use on the first page of this document.”

Table A4: Schedule of Hourly Assessments for Part II / QW (cont.)

Cycle	Day	Scheduled Time (h)	Vital Signs[a]	ECG-12 lead[b]	RO6958688 PK Sample	PD Blood Flow Cytometry	PD Blood Cytokines[c]	RO6958688 Anti-Drug Antibody (ADA)[d]	TCR Vβ[m]	Tumor Biopsy	Clinical Genotyping	RCR Sample (DNA and RNA)	FDG-PET	Obinutuzumab PK Sample	ADA specificity assessment[l]	Soluble CEA[p]
Cycle 5-29	Day 1	Pre-infusion	x	x	x			x					x[f]	x[k]	x[l]	
		End of Infusion			x											
Cycle 30 and every 6th cycle	Day 1	Pre-infusion	x	x	x			x								
		End of Infusion			x											
End of Treatment		Anytime	x		x			x								
28 Days Safety Follow Up		Anytime	x	x	x			x								

"Use of this document is governed by the terms of use on the first page of this document."

TABLE A4: SCHEDULE OF HOURLY ASSESSMENTS FOR PART II (CONT.)

Notes:

ADA = anti-drug antibody; h = hour; PD = pharmacodynamics; PK = pharmacokinetic; RCR = Roche Clinical Repository.

a. Vital signs (including supine blood pressure, heart rate and temperature) will be monitored on Day 1 pre-infusion, every 15 minutes until the end of infusion and, thereafter, every 30 minutes until the infusion line is removed. Starting Cycle 3: pre-infusion, every 30 minutes during infusion and every 30 minutes after the end of infusion until infusion line is removed. From Cycle 5 onwards vital signs will only be obtained on Day 1 pre-infusion. For the purposes of the eCRF vital signs will only be captured pre-infusion and in case of abnormalities.
b. Triplicate 12-lead ECG at screening (within 7 days before first dose of RO6958688) pre- and end of infusion on Cycle 1 Day 1 and Cycle 3 Day 1 and at the 28–day safety follow-up visit. Pre-infusion at all other study drug administrations. Additional unscheduled ECG assessments should be performed in case of abnormalities and if clinical symptoms occur. Recording must be done prior to PK sampling.
c. Plasma samples for the assessment of cytokine (PD blood cytokines) release will be collected. At the time of an IRR please see IRR SoA Table A9.
d. For ADA at the time of an IRR see IRR SoA Table A9.
e. Tumor biopsy samples will be collected on two occasions (once at baseline to confirm eligibility and once during the study treatment period). For patients who will be treated at dose levels equal and greater than 5 mg ($\geq$ 5 mg), a tumor biopsy for PD analyses is mandatory at baseline and on-treatment at Cycle 7 Day 1 (- 48/0 hours), this will be communicated at the time of the enrollment of each patient. All biopsies must be taken after FDG-PET, unless the sample is used to assess CEA expression. Patients being enrolled in cohorts A and B will undertake Baseline and C7D1 pre-dose biopsies.
f. FDG-PET should be done at baseline (Day-14 to D-1) but before the baseline biopsy. The on-treatment FDG-PET scan must be done at (C6D5 -72/0 hours) and before the on-treatment tumor biopsy.
g. Screening assessment should be performed between D28 and D-1, unless specified.
h. [Footnote no longer used]
i. PD blood samples at baseline to be obtained only from patients pretreated with obinutuzumab. Baseline sample should be taken during the screening period prior to administration of obinutuzumab, for patients receiving obinutuzumab pretreatment.
j. PK samples for obinutuzumab to be drawn during screening at predose and end of infusion (EOI) of obinutuzumab infusion on Day-13 or Day-13 and Day-12 (depending on if the patient receive the obinutuzumab infusion on one or two days), for patients receiving obinutuzumab pretreatment.
k. PK samples for obinutuzumab to be drawn predose and end of infusion on Cycle 8 Day 1, Cycle 12 Day 1, only, for patients receiving obinutuzumab pretreatment.

“Use of this document is governed by the terms of use on the first page of this document.”

Appendix 1
Schedule of Assessments (cont.)

l. Whole blood sample to be drawn at Cycle 1 Day 1 predose, Cycle 7 Day 1 predose and Cycle 12 Day 1 predose in patients participating at this ADA assessment.

m. For TCR Vβ, at each time point (Cycle 1 Day 1), 2 whole blood samples should be drawn.

n. [Footnote no longer used]

o. [Footnote no longer used]

p. Soluble CEA samples to be drawn pre-dose at Day 1 of Cycle 1

q. PK time windows: Pre-infusion: up to – 4 h - EOI: up to + 30 min - 2 h post EOI: +/- 30 min - 24 h: +/- 2 h - 48 h: +/- 4 h - Later: +/- 12 h.

“Use of this document is governed by the terms of use on the first page of this document.”

Appendix 1
Schedule of Assessments (cont.)

TABLE A5: SCHEDULE OF ASSESSMENTS FOR PART II / Q3W

Cycle	Screening	Cycle 1					Cycle 2					Cycle 3			Cycle 4			Cycle X	End of Treat ment	28 Days Safety Follow Up[n]
Day	D-28 to D-1	Day 1[p]	Day 2	Day 3	Day 6	Day 15	Day 1	Day 2	Day 3	Day 6	Day 19	Day 1	Day 2	Day 6	Day 1	Day 2	Day 6	Day 1		
Assessments[a]																				
Informed Consent[b]	X																			
Eligibility	X																			
Demography	X																			
Medical History	X																			
Physical Examination[c]	X	X					X					X			X			X		X
Anthropometric Measurements[d]	X	X	X	X	X	X	X	X	X	X		X	X	X	X	X	X	X		X
Vital Signs	X[aa]	X	X	X	X	X	X	X	X	X		X	X	X	X	X	X	X	X	X
Administration of RO6958688 Q3W		X					X					X			X			X		
Administration of obinutuzumab[y]	X[y]																			
ECG-12 lead[e]	X	X					X					X			X			X		X
ECOG Performance Status	X	X					X					X			X			X		X
Pregnancy Test[f]	X																	X[o]	X	X
RO6958688 PK Sample		X[g]	X	X	X	X	X[g]	X	X	X		X[g]	X	X	X[g]	X	X	X	X	X

“Use of this document is governed by the terms of use on the first page of this document.”

Appendix 1
Schedule of Assessments (cont.)

TABLE A5: SCHEDULE OF ASSESSMENTS FOR PART II / Q3W (CONT.)

Cycle	Screening	Cycle 1					Cycle 2					Cycle 3			Cycle 4			Cycle X	End of Treatment	28 Days Safety Follow Up[n]
Day	D-28 to D-1	Day 1[p]	Day 2	Day 3	Day 6	Day 15	Day 1	Day 2	Day 3	Day 6	Day 19	Day 1	Day 2	Day 6	Day 1	Day 2	Day 6	Day 1		
Obinutuzumab PK[y]	X[u]	X			X		X							X			X			
PD Blood Flow Cytometry	x[w]	X	X			X	X			X		X						X[s]		x[w]
PD Blood Cytokines[h]		X	X	X	X		X	X	X			X	X		X	X		X[t]		
Anti-Drug Antibody (ADA) RO6958688		X			X	X	X			X		X		X	X		X	X	X	X
TCR Vβ[r]		X										X						X		
Soluble CEA		X					X					X			X			X[y]	X	
Hematology[i]	X	X	X	X	X	X	X	X	X	X		X	X	X	X	X	X	X	X	X
Blood Chemistry[i]	X	X	X	X	X	X	X	X	X	X		X	X	X	X	X	X	X	X	X
Coagulation[i]	X	X	X	X	X	X	X	X	X	X		X	X	X	X	X	X	X	X	X
Urinalysis[ij]	X	X	X	X	X	X	X	X	X	X		X	X	X	X	X	X	X	X	X
Fresh Tumor Biopsy	X[k]						X[k]													
Clinical Genotyping		X																		
RCR Sample (DNA and RNA)		X																		
Archival Tissue[l]	X																			
FDG-PET[q]	X										X[q]									

“Use of this document is governed by the terms of use on the first page of this document.”

TABLE A5: SCHEDULE OF ASSESSMENTS FOR PART II / Q3W (CONT.)

Cycle	Screening	Cycle 1					Cycle 2					Cycle 3			Cycle 4			Cycle X	End of Treatment	28 Days Safety Follow Up[n]
Day	D-28 to D-1	Day 1[p]	Day 2	Day 3	Day 6	Day 15	Day 1	Day 2	Day 3	Day 6	Day 19	Day 1	Day 2	Day 6	Day 1	Day 2	Day 6	Day 1		
Serology	X[bb]																			
Tumor Assessment[m]	X																	X	X	
DL_{CO} / FEV1/VC/TLC[z]	X																			
Adverse Events	X																			
Previous and Concomitant Treatments	X																			

"Use of this document is governed by the terms of use on the first page of this document."

Appendix 1
Schedule of Assessments (cont.)

Notes: ADA = anti-drug antibody; CEA = carcinoembryonic antigen; D = day; ECOG = Eastern Cooperative Oncology Group; PD = pharmacodynamics; PK = pharmacokinetic; RCR = Roche Clinical Repository.

a) The safety assessments of all visits (hematology, blood chemistry, coagulation, urinalysis, physical examination) (unless otherwise indicated) should occur within a 3-day time window.

b) Informed consent must be obtained before any study-specific procedures.

c) Physical examinations (including weight) and ECOG Performance Status will be done at screening, at the time of treatment administration, and at the 28-day safety follow-up. Results must be obtained prior to infusion.

d) Weight only will be collected as part of anthropometric measurements on non-infusion days.

e) Triplicate 12-lead ECG at screening (within 7 days before first dose of RO6958688) pre-and end of infusion on Cycle 1 Day 1 and Cycle 2 Day 1 and at the 28-day safety follow-up visit.

f) Serum pregnancy test at screening, within 7 days prior to first dose.

g) PK pre-dose, end infusion, and 2 hours post End of infusion.

h) Serum or plasma samples for the assessment of cytokine (PD blood cytokines) release will be collected. At the time of an IRR, please see IRR SoA Table A9.

i) Hematology, blood chemistry, coagulation, and urinalysis can be performed within 24 hours (up to 72 hours if during weekend) prior to schedule dosing. Results must be obtained prior to infusion. Soluble CEA will be measured by the site as part of the blood chemistry measurements on Cycle 1 Day 1 and every 6 week thereafter for patients who continue treatment.

j) For coagulation (including PT/INR and PTT), at the time of an IRR please see IRR SoA Table A9.

k) If archival tumor tissue is NOT available for CEA assessment Tumor Biopsy will be required for CEA and will be assessed centrally as per Inclusion criterion #13. Tumor biopsy samples will be collected on two occasions after the FDG PET (once at baseline to confirm eligibility and once during the study treatment period). All on-treatment tumor biopsies will be collected as per section 3.2.5 of the protocol.

l) Archival tissue, if available, should be submitted for additional biomarker assessments for all patients enrolled in Part II, and who have consented in such tumor biopsies, the 28-day screening period does not apply to the CEA testing.

m) Measurable lesions will be assessed at screening, then at 8 weeks after Cycle 1 Day 1, and every 8 weeks thereafter for the first year, and every 12 weeks thereafter until disease progression or treatment discontinuation. Optional latest pre-study CT scan should be provided for assessment of tumor growth kinetics within 6 weeks of patient entering the study. For the scheduled tumor assessments beyond Screening a (± 7 day window is permitted)

n) Patients who complete the study or discontinue from the study early will be asked to return to the clinic 28 days after the last dose of study drug for a post-study follow-up visit.

o) For women of childbearing potential, a monthly pregnancy test (serum or urine) is mandatory during study treatment, starting from C1D1 until 28 Days Safety Follow Up. The patient must have a negative pregnancy test dated within the last month before dosing.

p) At least 24 hours (overnight) hospital stay required following administration of study drug at Cycle 1 Day 1.

"Use of this document is governed by the terms of use on the first page of this document."

Appendix 1
Schedule of Assessments (cont.)

q) FDG-PET must be done at baseline (Day-14 to D-1) before the baseline biopsy. The on-treatment FDG-PET must be done at (C2D19 – 72/0 hours). Refer to Appendix 9 for further information.

r) TCR Vβ samples to be drawn at Cycle 1 Day 1 predose (baseline), Cycle 3 Day 1 predose and Cycle 6 Day 1 predose.

s) PD Blood Flow Cytometry to be drawn at Cycle 6 Day 1.

t) PD blood cytokines samples to be drawn on Cycle 5 Day 1 and Cycle 6 Day 1, each pre and post RO6958688 infusion.

u) PK samples for obinutuzumab to be drawn pre-dose and end of infusion at Day-13 if obinutuzumab is administered as a single infusion or pre-dose and end of infusion at Day-13 and Day-12 if obinutuzumab is administered as split infusions during 2 consecutive days.

v) Obinutuzumab will be administered either on Day-13 (2000 mg) or on Day-13 and Day-12 (1000 mg each day).

w) PD blood samples at baseline and 28-day safety follow-up to be obtained only from patients pretreated with obinutuzumab. Baseline sample should be taken during the screening period prior to administration of obinutuzumab.

x) Hematology and blood chemistry to be assessed prior to obinutuzumab infusion and prior to first infusion of RO6958688.

y) Soluble CEA samples to be drawn pre-dose at Day 1 of each cycle up to Cycle 7 and during the end of treatment visit.

z) FEV1/VC/TLC and DL_{CO} (corrected for both alveolar volume and hemoglobin) will be evaluated at screening in patients with bilateral lung metastases or patients with lobectomy or pneumonectomy with lung metastases in the remaining lung. Additional tests may be performed if clinically indicated. The same pulmonary function testing laboratory should be used throughout the study.

aa) Vital signs including oxygen saturation.

bb) Hepatitis B and C and HIV or HTLV-1 serology is mandatory only for patients receiving obinutuzumab pretreatment.

“Use of this document is governed by the terms of use on the first page of this document.”

Appendix 1
Schedule of Assessments (cont.)

TABLE A6: SCHEDULE OF HOURLY ASSESSMENTS FOR PART II / Q3W

Cycle = 21 days (Q3W)	Day	Scheduled Time (h)	Vital Signs[a]	ECG-12 lead[b]	RO6958688 PK Sample	Obinutuzumab PK	PD Blood Flow Cytometry	PD Blood Cytokines[c]	Anti-Drug Antibody (ADA)[d]	Tumor Biopsy	Clinical Genotyping	RCR Sample (DNA and RNA)	FDG PET	TCR Vβ	Soluble CEA
Screening		D-28 to D-1	X	X		X[j]	x[l]			X			X[f]		
Cycle 1	Day 1	Pre-infusion	X	X	X	X	X	X	X		X	X		X	X
		End of Infusion	X	X	X			X							
		2 Hours Post Infusion			X			X							
		4-6 Hours post inf.						X							
	Day 2	24 Hours			X		X	X							
		Anytime	X												
	Day 3	48 Hours			X			X							
		Anytime	X												
	Day 6	120 Hours			X	X		X	X						
		Anytime	X												
	Day 15	336 Hours			X		X		X						
		Anytime	X												
Cycle 2	Day 1	Pre-infusion	X	X	X	X	X	X	X	X[h]					X
		End of Infusion	X	X	X			X							
		2 Hours Post Infusion			X			X							

"Use of this document is governed by the terms of use on the first page of this document."

Appendix 1
Schedule of Assessments (cont.)

TABLE A6: SCHEDULE OF HOURLY ASSESSMENTS FOR PART II / Q3W (CONT.)

Cycle = 21 days (Q3W)	Day	Scheduled Time (h)	Vital Signs[a]	ECG-12 lead[b]	RO6958688 PK Sample	Obinutuzumab PK	PD Blood Flow Cytometry	PD Blood Cytokines[c]	Anti-Drug Antibody (ADA)[d]	Tumor Biopsy	Clinical Genotyping	RCR Sample (DNA and RNA)	FDG PET	TCR Vβ	Soluble CEA
Cycle 2	Day 2	24 Hours			X			X							
		Anytime	X												
	Day 3	48 Hours			X			X							
		Anytime	X												
	Day 6	120 Hours			X		X		X						
		Anytime	X												
	D19	Anytime											X[f]		
Cycle 3	Day 1	Pre-infusion	X	X	X		X	X	X					X	X
		End of Infusion	X		X			X							
		2 Hours Post Infusion			X										
	Day 2	24 Hours			X			X							
		Anytime	X												
	Day 6	120 Hours			X	X			X						
		Anytime	X												

"Use of this document is governed by the terms of use on the first page of this document."

Appendix 1
Schedule of Assessments (cont.)

TABLE A6: SCHEDULE OF HOURLY ASSESSMENTS FOR PART II / Q3W (CONT.)

Cycle = 21 days (Q3W)	Day	Scheduled Time (h)	Vital Signs[a]	ECG-12 lead[b]	RO6958688 PK Sample	Obinutuzumab PK	PD Blood Flow Cytometry	PD Blood Cytokines[c]	Anti-Drug Antibody (ADA)[d]	Tumor Biopsy	Clinical Genotyping	RCR Sample (DNA and RNA)	FDG PET	TCR Vβ	Sol ubl e CE A
Cycle 4	Day 1	Pre-infusion	X	X	X			X	X						X
		End of Infusion	X		X			X							
		2 Hours Post Infusion			X			X							
	Day 2	24 Hours			X			X							
		Anytime	X												
	Day 6	120 Hours			X	X			X						
		Anytime	X												
Cycle X	Day 1	Pre-infusion	X	X	X		X[g]	X[i]	X					X[e]	X[k]
		End of Infusion			X			X[i]							
End of Treatment		Anytime	X		X				X						X
28 Days Safety Follow Up		Anytime	X	X	X		x[l]		X						

"Use of this document is governed by the terms of use on the first page of this document."

TABLE A6: SCHEDULE OF HOURLY ASSESSMENTS FOR PART II / Q3W (CONT.)

a Vital signs (including supine blood pressure and heart rate) will be monitored on Day 1 pre-infusion, every 15 minutes until the end of infusion and, thereafter, every 30 minutes until the infusion line is removed. Starting cycle 3: pre-infusion, every 30 minutes during infusion and every 30 minutes after the end of infusion until infusion line is removed. From Cycle 5 onwards vital signs will only be obtained on Day 1 pre-infusion. For the purposes of the eCRF vital signs will only be captured pre-infusion and in case of emergencies.

b Triplicate 12-lead ECG at screening (within 7 days before first dose of RO6958688) pre- and end of infusion on Cycle 1 Day 1 and Cycle 2 Day 1 and at the 28-day safety follow-up visit. Pre-infusion at all other study drug administrations. Additional unscheduled ECG assessments should be performed in case of abnormalities and if clinical symptoms occur. Recording must be done prior to PK sampling.

c Serum or plasma samples for the assessment of cytokine (PD Blood Cytokines) release will be collected. At the time of an IRR please see IRR SoA Table A9.

d For ADA at the time of an IRR please see IRR SoA Table A9.

e TCR Vβ samples to be drawn at Cycle 1 Day 1 predose (baseline), Cycle 3 Day 1 predose and Cycle 6 Day 1 predose.

f FDG-PET must be done at baseline (Day-14 to D-1) before the baseline biopsy. The on-treatment FDG-PET must be done at (C2D19 -72/0 hours). Refer to Appendix 9 for further information.

g PD Blood Flow Cytometry to be drawn at Cycle 6 Day 1.

h Fresh tumour biopsy to be undertaken either at Cycle 2 Day 1, unscheduled biopsy can be undertaken at the time of disease progression or if clinically indicated. Patients being enrolled in obinutuzumab pre-treatment cohorts will undertake Baseline and C2D1 pre-dose biopsies.

i PD blood cytokines samples to be drawn on Cycle 5 Day 1 and Cycle 6 Day 1, each pre- and post-RO6958688 infusion.

j PK samples for obinutuzumab to be drawn during screening at predose and end of infusion (EOI) of obinutuzumab infusion on Day-13 or Day-13 and Day-12 (depending on if the patient receive the obinutuzumab infusion on one or two days), for patients receiving obinutuzumab pretreatment.

k Soluble CEA samples to be drawn pre-dose at Day 1 of each cycle up to Cycle 7 and during the end of treatment visit.

l PD blood samples at baseline and 28-day safety follow-up to be obtained only from patients pretreated with obinutuzumab. Baseline sample should be taken during the screening period prior to administration of obinutuzumab, for patients receiving obinutuzumab pretreatment.

m PK time windows: Pre-infusion: up to – 4 h - EOI: up to + 30 min - 2 h post EOI: +/- 30 min - 24 h: +/- 2 h - 48 h: +/- 4 h - Later: +/- 12 h.

"Use of this document is governed by the terms of use on the first page of this document."

Appendix 1
Schedule of Assessments (cont.)

TABLE A7: SCHEDULE OF ASSESSMENTS FOR PART II / QWx3 - Q3W

Cycle	Screening	Cycle 1 (QW)				Cycle 2 (QW)			Cycle 3 (QW)			Cycle 4 (Q3W)				Cycle 5 (Q3W)			Cycle ≥ 6 (Q3W)	End of Treatment	28-Day Safety Follow Up[n]
Day	D-28 to D-1	Day 1[o]	Day 2	Day 3	Day 6	Day 1	Day 2	Day 6	Day 1	Day 2	Day 6	Day 1	Day 2	Day 6	Day 19	Day 1	Day 2	Day 15	Day 1		
Assessments[a]																					
Informed Consent[b]	x																				
Eligibility	x																				
Demography	x																				
Medical History	x																				
Physical Examination[c,d]	x	x				x			x			x				x			x		x
Anthropometric Measurements[e]	x	x	x	x	x	x	x	x	x	x	x	x	x	x		x	x	x	x		x
Vital Signs	x[v]	x	x	x	x	x	x	x	x	x	x	x	x	x		x			x	x	x
Administration of RO6958688		x				x			x			x				x			x		
Administration of obinutuzumab	x																				
ECG-12 lead[f]	x	x				x			x			x				x			x		x
ECOG Performance Status	x	x				x			x			x				x			x		x
Pregnancy Test[g]	x																		x	x	x
PK Sample RO6958688		x	x	x	x	x	x	x	x	x		x	x	x		x	x		x[aa]	x	x
Obinutuzumab PK	x[w]	x				x						x				x			x[x]		
PD Blood Flow Cytometry	x[y]	x	x			x			x												

"Use of this document is governed by the terms of use on the first page of this document."

Appendix 1
Schedule of Assessments (cont.)

TABLE A7: SCHEDULE OF ASSESSMENTS FOR PART II / QWx3 - Q3W (CONT.)

Cycle	Screening	Cycle 1 (QW)				Cycle 2 (QW)			Cycle 3 (QW)			Cycle 4 (Q3W)				Cycle 5 (Q3W)			Cycle ≥ 6 (Q3W)	End of Treatment	28-Day Safety Follow Up[n]
Day	D-28 to D-1	Day 1[o]	Day 2	Day 3	Day 6	Day 1	Day 2	Day 6	Day 1	Day 2	Day 6	Day 1	Day 2	Day 6	Day 19	Day 1	Day 2	Day 15	Day 1		
PD Blood Cytokines[h]		x	X	x	x	x	x		x	x											
Anti-Drug Antibody (ADA) RO6958688		x				x			x			x				x			x[bb]	x	x
TCR Vβ[q]		x																			
Soluble CEA[r]		x																			
Hematology[i]	x	x	X	x	x	x	x	x	x	x	x	x	x	x		x	x	x	x	x	x
Blood Chemistry[i]	x	x	X	x	x	x	x	x	x	x	x	x	x	x		x	x	x	x	x	x
Coagulation[i]	x	x	X	x	x	x	x	x	x	x	x	x	x	x		x	x	x	x	x	x
Urinalysis[i,j]	x	x	X	x	x	x	x	x	x	x	x	x	x	x		x	x	x	x	x	x
Serology	x[z]																				
Tumor Biopsy[i]	x[k]											x[s]				x[s]					
Clinical Genotyping		x																			
RCR Sample (DNA and RNA)		x																			
Archival Tissue[l]	x																				
FDG-PET[l]	x														x						
Tumor Assessment[m,p]	x																	x	x[m]	x	

"Use of this document is governed by the terms of use on the first page of this document."

TABLE A7: SCHEDULE OF ASSESSMENTS FOR PART II / QWx3 - Q3W (CONT.)

Cycle	Screening	Cycle 1 (QW)				Cycle 2 (QW)			Cycle 3 (QW)			Cycle 4 (Q3W)				Cycle 5 (Q3W)			Cycle ≥ 6 (Q3W)	End of Treatment	28-Day Safety Follow Up[n]
Day	D-28 to D-1	Day 1[o]	Day 2	Day 3	Day 6	Day 1	Day 2	Day 6	Day 1	Day 2	Day 6	Day 1	Day 2	Day 6	Day 19	Day 1	Day 2	Day 15	Day 1		
DL_{CO} / FEV1/VC/TLC[t]	x																				
PBMC B-cell isolation (ADA specificity assessment)[u]		x														x			x[u]		
Adverse Events	X																				
Previous and Concomitant Treatments	X																				

"Use of this document is governed by the terms of use on the first page of this document."

Appendix 1
Schedule of Assessments (cont.)

Notes: ADA = anti-drug antibody; CEA = carcinoembryonic antigen; D = day; ECOG = Eastern Cooperative Oncology Group; PD = pharmacodynamics; PK = pharmacokinetic; RCR = Roche Clinical Repository.

a The safety assessments of all visits (hematology, blood chemistry, coagulation, urinalysis, physical examination) (unless otherwise indicated) should occur within 24 hour or a 3-day time window if the dosing date follows a weekend.

b Informed consent must be obtained before any study-specific procedures.

c Physical examinations (including weight, height and oxygen saturation) and ECOG Performance Status will be done at screening, at the time of treatment administration, and at the 28-day safety follow-up. Results must be obtained prior to infusion. Height will be done at screening and at follow up visit only.

d Physical examinations (including vital signs and oxygen saturation) will be done according to Exclusion Criterion #7.

e Weight only will be collected as part of anthropometric measurements on non-infusion days.

f Triplicate 12-lead ECG at screening (within 7 days before first dose of RO6958688) pre-and end of infusion on Cycle 1 Day 1 and Cycle 3 Day 1 and at the 28-day safety follow-up visit.

g For women of childbearing potential, a monthly pregnancy test (serum or urine) is mandatory during study treatment, starting from C1D1 until 28 Days Safety Follow Up. The patient must have a negative pregnancy test dated within the last month before dosing.

h Serum or plasma samples for the assessment of cytokine (PD blood cytokines) release will be collected. At the time of an IRR, please see IRR SoA Table A9.

i Hematology, blood chemistry, coagulation, and urinalysis can be performed within 24 hours (up to 72 hours if during weekend) prior to schedule dosing. Results must be obtained prior to infusion. Soluble CEA will be measured by the site as part of the blood chemistry measurements on Cycle 1 Day 1 and every 6 week thereafter for patients who continue treatment.

j For coagulation (including PT/INR, fibrinogen and PTT), at the time of an IRR please see IRR SoA Table A8.

k If archival tumor tissue is NOT available for CEA assessment Tumor Biopsy will be required for CEA and will be assessed centrally as per Inclusion criterion #13. Tumor biopsy samples will be collected on two occasions after the FDG PET (once at baseline to confirm eligibility and once during the study treatment period). All on-treatment tumor biopsies will be collected as per section 3.2.5 of the protocol.

l Archival tissue, if available, should be submitted for additional biomarker assessments for all patients enrolled in Part II, and who have consented in such tumor biopsies, the 28-day screening period does not apply to the CEA testing.

m Measurable lesions will be assessed at screening, then at 8 weeks after Cycle 1 Day 1, and every 8 weeks thereafter for the first year, and every 12 weeks thereafter until disease progression or treatment discontinuation. Optional latest pre-study CT scan should be provided for assessment of tumor growth kinetics within 6 weeks of patient entering the study. For the scheduled tumor assessments beyond Screening a (± 7 day window is permitted)

n Patients who complete the study or discontinue from the study early will be asked to return to the clinic 28 days after the last dose of study drug for a post-study follow-up visit.

o At least 24 hours (overnight) hospital stay required following administration of study drug at Cycle 1 Day 1.

p FDG-PET must be done at baseline (Day-14 to D-1) before the baseline biopsy. The on-treatment FDG-PET must be done at (C4D19 – 72/0 hours). Refer to Appendix 9 for further information.

“Use of this document is governed by the terms of use on the first page of this document.”

q TCR Vβ samples to be drawn at Cycle 1 Day 1 predose (baseline).

r Soluble CEA samples to be drawn at Cycle 1 Day 1 predose (Baseline).

s Patients will undergo either Cycle 4 Day 1 predose or Cycle 5 Day 1 predose fresh tumor biopsy (-48/0 hours).

t FEV1/VC/TLC and DL_{CO} (corrected for both alveolar volume and hemoglobin) will be evaluated at screening in patients with bilateral lung metastases or patients with lobectomy or pneumonectomy with lung metastases in the remaining lung. Additional tests may be performed if clinically indicated. The same pulmonary function testing laboratory should be used throughout the study.

u Whole blood sample to be drawn at Cycle 1 Day 1 predose, Cycle 5 Day 1 predose and Cycle 6 Day 1 predose in patients participating to this ADA assessment.

v Vital signs including oxygen saturation.

w PK samples for obinutuzumab to be drawn during screening at predose and end of infusion (EOI) of obinutuzumab infusion on Day-7 or Day-8 and Day-7 (depending on if the patient receive the obinutuzumab infusion on one or two days), for patients receiving obinutuzumab pretreatment.

x PK samples for obinutuzumab to be drawn predose and end of infusion at Cycle 6 Day 1, Cycle 7 Day 1, Cycle 8 Day 1, and Cycle 9 Day 1 only.

y PD blood samples at baseline to be obtained only from patients pretreated with obinutuzumab. Baseline sample should be taken during the screening period prior to administration of obinutuzumab.

z Hepatitis B and C and HIV or HTLV-1 serology is mandatory only for patients receiving obinutuzumab pretreatment.

aa From Cycle 10 to Cycle 12, RO6958688 PK samples will be obtained pre-infusion and at end of infusion on Day 1 of each cycle. At Cycle 13 and every 2nd Cycle, RO6958688 PK samples will be obtained pre-infusion and at end of infusion on Day 1 at the mentioned timepoints.

bb From Cycle 1 to Cycle 12, RO6958688 ADA samples will be obtained pre-infusion on Day 1 of each cycle. At Cycle 13 and every 2nd Cycle, ADA samples will be collected pre-infusion on Day 1 at the mentioned timepoints.

"Use of this document is governed by the terms of use on the first page of this document."

Appendix 1
Schedule of Assessments (cont.)

TABLE A8: SCHEDULE OF HOURLY ASSESSMENTS FOR PART II - QWX3/Q3W

Cycle	Day	Scheduled Time (h)	Vital Signs[a]	ECG-12 lead[b]	RO6958688 PK Sample[m]	Obinutuzumab PK Sample	PD Blood Flow Cytometry	PD Blood Cytokines[c]	Anti-Drug Antibody (ADA)[d]	Soluble CEA[i]	ADA specificity assessment	Tumor Biopsy	Clinical Genotyping	RCR Sample (DNA and RNA)	FDG PET[f]	TCR Vβ[e]
Screening		D-28 to D-1	x	x		x[l]	x[k]					x			x	
Cycle 1	Day 1	Pre-infusion	x	x	x	x	x	x	x	x	x		x	x		x
		EOI	x	x	x			x								
		2 H post EOI			x			x								
		4 - 6 H post EOI					x	x								
	Day 2	24 H	x		x		x	x								
	Day 3	48 H	x		x			x								
	Day 6	120 H	x		x			x								
Cycle 2	Day 1	Pre-infusion	x	x	x	x	x	x	x							
		EOI	x		x			x								
		2 H post EOI			x			x								
	Day 2	24 H	x		x			x								
	Day 6	120 H	x		x											

"Use of this document is governed by the terms of use on the first page of this document."

Appendix 1
Schedule of Assessments (cont.)

TABLE A8: SCHEDULE OF HOURLY ASSESSMENTS FOR PART II - QWX3/Q3W (CONT.)

Cycle	Day	Scheduled Time (h)	Vital Signs [a, n, o]	ECG-12 lead [b]	RO6958688 PK Sample	Obinutuzumab PK Sample	PD Blood Flow Cytometry	PD Blood Cytokines[c]	Anti-Drug Antibody (ADA)[d]	Soluble CEA[i]	ADA specificity assessment	Tumor Biopsy	Clinical Genotyping	RCR Sample (DNA and RNA)	FDG PET[f]	TCR Vβ[e]
Cycle 3	Day 1	Pre-infusion	x	x	x		x	x	x							
		EOI	x	x	x			x								
		2 H post EOI			x											
	Day 2	24 H	x		x			x								
	Day 6	120 H	x													
Cycle 4	Day 1	Pre-infusion	x	x	x	x			x			x[h]				
		EOI	x		x											
		2 H post EOI			x											
	Day 2	24 H	x		x											
	Day 6	120 H	x		x											
	Day 19	432 H													x	
Cycle 5	Day 1	Pre-infusion	x	x	x	x			x		x	x[h]				
		EOI			x											
	Day 2	24 H			x											

"Use of this document is governed by the terms of use on the first page of this document."

Appendix 1
Schedule of Assessments (cont.)

TABLE A8: SCHEDULE OF HOURLY ASSESSMENTS FOR PART II - QWX3/Q3W (CONT.)

Cycle	Day	Scheduled Time (h)	Vital Signs [a, n, o]	ECG-12 lead[b]	RO6958688 PK Sample	Obinutuzumab PK Sample	PD Blood Flow Cytometry	PD Blood Cytokines[c]	Anti-Drug Antibody (ADA)[d]	Soluble CEA[i]	ADA specificity assessment	Tumor Biopsy	Clinical Genotyping	RCR Sample (DNA and RNA)	FDG PET[f]	TCR Vβ[e]
Cycle 6 and 7	Day 1	Pre-infusion	x	x	x	x			X		x[j]					
		EOI			x											
Cycle 8 and 9	Day 1	Pre-infusion	x	x	x	x			X							
		EOI			x											
Cycle 10 to 12	Day 1	Pre-infusion	x	x	x				X							
		EOI			x											
Cycle 13 and every 2nd cycle	Day 1	Pre-infusion	x	x	x				x							
		EOI			x											
End of Treatment		Anytime	x		x				X							
28 day Safety Follow Up		Anytime	x	x	x				X							

"Use of this document is governed by the terms of use on the first page of this document."

TABLE A8: SCHEDULE OF HOURLY ASSESSMENTS FOR PART II - QWX3/Q3W (CONT.)

a Vital signs (including supine blood pressure, heart rate and temperature) will be monitored on Day 1 pre-infusion, every 15 minutes until the end of infusion and, thereafter, every 30 minutes until the infusion line is removed. Starting cycle 3: pre-infusion, every 30 minutes during infusion and every 30 minutes after the end of infusion until infusion line is removed. From Cycle 5 onwards vital signs will only be obtained on Day 1 pre-infusion. For the purposes of the eCRF vital signs will only be captured pre-infusion and in case of emergencies. During obinutuzumab infusion, vital signs (including, if possible, supine diastolic and systolic blood pressure, pulse rate and temperature) have to be monitored pre-infusion, every 15 minutes for the first 90 minutes of the infusion, and then every 30 minutes until 1 hour after *completion of the infusion.*

b Triplicate 12-lead ECG at screening (within 7 days before first dose of RO6958688) pre- and end of infusion on Cycle 1 Day 1 and Cycle 3 Day 1 and at the 28-day safety follow-up visit. Pre-infusion at all other study drug administrations. Additional unscheduled ECG assessments should be performed in case of abnormalities and if clinical symptoms occur. Recording must be done prior to PK sampling.

c Serum or plasma samples for the assessment of cytokine (PD Blood Cytokines) release will be collected. At the time of an IRR please see IRR SoA Table A9.

d For ADA at the time of an IRR please see IRR SoA Table A9.

e TCR Vβ samples to be drawn at Cycle 1 Day 1 predose (baseline).

f FDG-PET must be done at baseline (Day-14 to D-1) before the baseline biopsy. The on-treatment FDG-PET must be done at (C4D19 -72/0 hours). Refer to Appendix 9 for further information.

g **[Footnote no longer used]**

h Fresh tumor biopsy to be undertaken at either Cycle 4 Day 1 or Cycle 5 Day 1 (see protocol Section 3.2.5), unscheduled biopsy can be undertaken at the time of disease progression or if clinically indicated.

i Soluble CEA samples to be drawn at Cycle 1 Day 1 predose (Baseline).

j Samples for ADA specificity test assessment to be collected at Cycle 1 Day 1 predose, Cycle 5 Day 1 predose and Cycle 6 Day 1 predose.

k PD blood samples at baseline. Baseline sample should be taken during the screening period prior to administration of obinutuzumab, for patients receiving obinutuzumab pretreatment.

l PK samples for obinutuzumab to be drawn during screening at predose and end of infusion (EOI) of obinutuzumab infusion on Day-7 or Day-8 and Day-7 (depending on if the patient receive the obinutuzumab infusion on one or two days), for patients receiving obinutuzumab pretreatment.

m PK time windows: Pre-infusion: up to – 4 h - EOI: up to + 30 min - 2 h post EOI: +/- 30 min - 24 h: +/- 2 h - 48 h: +/- 4 h - Later: +/- 12 h.

n *If a patient has experienced a Grade 3 IRR/CRS event during the previous treatment administration visit, the patient should be hospitalized for at least 24 hours after the end of infusion at next treatment administration visit during which vital signs will be monitored as follows: every 30 (± 10) minutes for the first hour post infusion, every 60 (± 15) minutes during the following 3 hours, every 120 (± 20) minutes for the next 8 hours, and every 240 (± 30) minutes for the remaining 12 hours.*

o *If a patient experiences a Grade 3 or higher treatment-related adverse event (with the exception of IRR/CRS) within the 24-hour period following the previous infusion, the patient should be observed for at least 8 hours after the end of RO6958688 infusion at the next administration visit during which vital signs will be monitored as follows: every 30 (± 10) minutes for the first hour post infusion, every 60 (± 15) minutes during the following 3 hours, and every 120 (± 20) minutes for the next 4 hours.*

“Use of this document is governed by the terms of use on the first page of this document.”

Appendix 1
Schedule of Assessments (cont.)

TABLE A9: SCHEDULE OF ASSESSMENTS FOR INFUSION-RELATED REACTION

Cycle	Day	Scheduled Time (h)	PK Sample	PD Blood Cytokines[a]	IgE and Tryptase	Hematology	Blood Chemistry	Coagulation
IRR	Unscheduled	Anytime	x	x	x	x	x	x

Notes:

ADA = anti-drug antibody; h = hour; IRR = infusion-related reaction; PD = pharmacodynamics; PK = pharmacokinetic.

a. At the time of an IRR (including repetitive occurrence of IRR) or hypersensitivity reaction, unscheduled samples will be collected. Cytokine analysis will include but not be limited to TNF-α, IL-6, and IFNγ. For patients who experience an IRR ≥ Grade 2 for the first time, with the second or subsequent study drug infusion, tryptase and total IgE will be analyzed.

"Use of this document is governed by the terms of use on the first page of this document."

Appendix 2 CKD-EPI equation for Calculation of glomerular filtration rate (GFR)

CKD EPI Equation for Estimating GFR Expressed for Specified Race, Sex and Serum Creatinine in mg/dL (From Levey et al. Ann Intern Med 2009;150:604-612).

Race	Sex	Serum Creatinine, S_{cr} (mg/dL)	Equation (age in years for ≥ 18)
Black	Female	$\leqslant$ 0.7	GFR = 166 × $(S_{cr}/0.7)^{-0.329}$ × $(0.993)^{Age}$
Black	Female	> 0.7	GFR = 166 × $(S_{cr}/0.7)^{-1.209}$ × $(0.993)^{Age}$
Black	Male	$\leqslant$ 0.9	GFR = 163 × $(S_{cr}/0.9)^{-0.411}$ × $(0.993)^{Age}$
Black	Male	> 0.9	GFR = 163 × $(S_{cr}/0.9)^{-1.209}$ × $(0.993)^{Age}$
White or other	Female	$\leqslant$ 0.7	GFR = 144 × $(S_{cr}/0.7)^{-0.329}$ × $(0.993)^{Age}$
White or other	Female	> 0.7	GFR = 144 × $(S_{cr}/0.7)^{-1.209}$ × $(0.993)^{Age}$
White or other	Male	$\leqslant$ 0.9	GFR = 141 × $(S_{cr}/0.9)^{-0.411}$ × $(0.993)^{Age}$
White or other	Male	> 0.9	GFR = 141 × $(S_{cr}/0.9)^{-1.209}$ × $(0.993)^{Age}$

Normal glomerular filtration rate by age:

Age	Average estimated GFR
20-29	116
30-39	107
40-49	99
50-59	93
60-69	85
70+	75

“Use of this document is governed by the terms of use on the first page of this document.”

Appendix 3
Eastern Cooperative Oncology Group Performance Status

ECOG PERFORMANCE STATUS	
**Grade**	**ECOG**
0	Fully active, able to carry on all pre-disease performance without restriction
1	Restricted in physically strenuous activity but ambulatory and able to carry out work of a light or sedentary nature, e.g., light house work, office work
2	Ambulatory and capable of all selfcare but unable to carry out any work activities. Up and about more than 50% of waking hours
3	Capable of only limited selfcare, confined to bed or chair more than 50% of waking hours
4	Completely disabled. Cannot carry on any selfcare. Totally confined to bed or chair
5	Dead

REFERENCE

Oken MM, Creech RH, Tormey DC, et al. Toxicity And Response Criteria of the Eastern Cooperative Oncology Group. Am J Clin Oncol 1982;5:649–55.

"Use of this document is governed by the terms of use on the first page of this document."

Appendix 4
Response Evaluation Criteria in Solid Tumors Version 1.1 Criteria

The investigator will evaluate response to treatment using Response Evaluation Criteria in Solid Tumors (RECIST) Version 1.1 and immune–related response criteria (irRC).

DEFINITIONS OF MEASURABLE/NON-MEASURABLE LESIONS

At baseline, tumor lesions/lymph nodes will be categorized measurable or non-measurable as follows:

Measurable Tumor Lesions

Tumor lesions must be accurately measured in at least one dimension (longest diameter in the plane of measurement is to be recorded) with a minimum size of 10 mm by computed tomography (CT) or magnetic resonance imaging (MRI) scan (CT/MRI scan slice thickness/interval no greater than 5 mm).

Malignant lymph nodes: To be considered pathologically enlarged and measurable, a lymph node must be ≥15 mm in short axis when assessed by CT scan1. Only the short axis will be measured and followed.

Non-Measurable Tumor Lesions

Non-measurable tumor lesions include:

- Small lesions (longest diameter <10 mm)
- Pathological lymph nodes with ≥10 to <15 mm short axis
- Truly non-measurable lesions. (e.g., ascites, pleural or pericardial effusion, lymphangitic involvement of skin or lung, peritoneal spread, abdominal masses/abdominal organomegaly identified by physical exam that is not measurable by reproducible imaging techniques).

Special considerations regarding lesion measurability:

Bone lesions, cystic lesions, and lesions previously treated with local therapy require particular comment:

Bone lesions:

- Lytic bone lesions or mixed lytic-blastic lesions with identifiable soft tissue components that can be evaluated by cross–sectional imaging techniques such as CT or MRI can be considered as measurable lesions if the soft tissue component meets the definition of measurability described above.
- Blastic bone lesions are non-measurable.

“Use of this document is governed by the terms of use on the first page of this document.”

Lesions with prior local treatment:

- Tumor lesions situated in a previously irradiated area, or in an area subjected to other loco-regional therapy, are usually not considered measurable unless there has been demonstrated progression in the lesion. Study protocols should detail the conditions under which such lesions would be considered measurable.

TARGET LESIONS: SPECIFICATIONS BY METHODS OF MEASUREMENTS

The same method of assessment and the same technique should be used to characterize each identified and reported lesion at baseline and during study.

TUMOR RESPONSE EVALUATION

Baseline documentation of 'target' and 'non-target' lesions

- When more than one measurable lesion is present at baseline, all lesions up to a maximum of five lesions total (and a maximum of two lesions per organ) representative of all involved organs should be identified as target lesions and will be recorded and measured at baseline.
- Where patients have only one or two organ sites involved a maximum of two (one site) and four lesions (two sites), respectively, can be recorded. Other lesions (including measurable lesion) in that organ will be recorded as non-measurable lesions.
- Target lesions should be selected on the basis of their size (lesions with the longest diameter), be representative of all involved organs, but in addition should be reproducible in repeated measurements.
- Lymph nodes: target lesions must have a short axis of $\geq$15 mm by CT scan. Only the short axis of these nodes will contribute to the baseline sum. Pathological lymph nodes with a short axis $\geq$10 mm but $<$15 mm should be considered as non-target lesions. Nodes that have a short axis $<$10 mm are considered non-pathological and should not be recorded or followed.
- A sum of the longest diameters (except for nodal lesions) for all target lesions will be calculated and reported as the baseline sum diameters. If lymph nodes are to be included in the sum then only the short axis is added to the sum. The baseline sum diameters will be used as reference to further characterize any objective tumor regression in the measurable dimension of the disease.
- Measurements are not required for non-target lesions. These should be followed as 'present', 'absent', or in rare cases 'unequivocal progression'.

RESPONSE CRITERIA

This section provides the definitions of the criteria used to determine objective tumor response for target lesions.

"Use of this document is governed by the terms of use on the first page of this document."

EVALUATION OF TARGET LESIONS

- Complete Response (CR): Disappearance of all target lesions. Any pathological lymph nodes (whether target or non-target) must have reduction in short axis to < 10 mm.
- Partial Response (PR): At least a 30% decrease in the sum of diameters of target lesions, taking as reference the baseline sum diameters.
- Progressive Disease (PD): At least a 20% increase in the sum of diameters of target lesions, taking as reference the smallest sum on study including baseline (nadir). In addition to the relative increase of 20%, the sum must also demonstrate an absolute increase of at least 5 mm. The appearance of one or more new lesions is also considered progression.
- Stable Disease (SD): Neither sufficient shrinkage to qualify for PR nor sufficient increase to qualify for PD taking as a reference the smallest sum diameters while on study.

EVALUATION OF NON-TARGET LESIONS

- Complete Response (CR): Disappearance of all non-target lesions (and, if applicable, normalization of tumor marker level). All lymph nodes must be non-pathological in size (< 10 mm short axis).
- Non-CR/Non-PD: Persistence of one or more non-target lesion(s) and/or maintenance of tumor marker level above the normal limits.
- Progressive Disease (PD): Unequivocal progression of existing non-target lesions. The appearance of one or more new lesions is also considered progression.

NEW LESIONS

- The appearance of new malignant lesions denotes disease progression.
The finding of a new lesion should be unequivocal: i.e., not attributable to differences in scanning technique, change in imaging modality or findings thought to represent something other than tumor.
- A lesion identified during the study in an anatomical location that was not scanned at baseline is considered a new lesion and will indicate disease progression.
- If a new lesion is equivocal, for example because of its small size, continued therapy and follow-up evaluation will clarify if it represents truly new disease. If repeat scans confirm there is definitely a new lesion, then progression should be declared using the date of the initial scan.

EVALUATION OF RESPONSE

TIMEPOINT RESPONSE (OVERALL RESPONSE)

The table below provides a summary of the overall response status calculation at each timepoint for patients who have measurable disease at baseline.

“Use of this document is governed by the terms of use on the first page of this document.”

Target lesions	Non-target lesions	New lesions	Overall response
CR	CR	No	CR
CR	Non-CR/non-PD	No	PR
CR	Not evaluated	No	PR
PR	Non-PD or not all evaluated	No	PR
SD	Non-PD or not all evaluated	No	SD
Not all evaluated	Non-PD	No	NE
PD	Any	Yes or No	PD
Any	PD	Yes or No	PD
Any	Any	Yes	PD

CR = complete response, PR = partial response, SD = stable disease, PD = progressive disease, and NE = inevaluable.

Missing assessments and not-evaluable designation

When no imaging/measurement is done at all at a particular timepoint, the subject is not evaluable at that timepoint. If only a subset of measurements are made at an assessment, subject is also considered not evaluable at that timepoint, unless the missing lesion(s) would not change the assigned timepoint response, e.g., in the case of PD. If one or more target lesions were not assessed the Response for Target Lesions should be “Unable to Assess” (except where there is clear progression).

Overall response First time point	Overall response Subsequent time point	BEST overall response
CR	CR	CR
CR	PR	SD, PD or PR[a]
CR	SD	SD provided minimum criteria for SD duration met, otherwise, PD
CR	PD	SD provided minimum criteria for SD duration met, otherwise, PD
CR	NE	SD provided minimum criteria for SD duration met, otherwise NE
PR	CR	PR
PR	PR	PR
PR	SD	SD
PR	PD	SD provided minimum criteria for SD duration met, otherwise, PD
PR	NE	SD provided minimum criteria for SD duration met, otherwise NE
NE	NE	NE

CR = complete response, PR = partial response, SD = stable disease, PD = progressive disease, and NE = inevaluable.
a If a CR is truly met at first time point, then any disease seen at a subsequent time point, even disease meeting PR criteria relative to baseline, makes the disease PD at that point (since disease must have reappeared after CR). Best response would depend on whether minimum duration for SD was met. However, sometimes ‘CR’ may be claimed when subsequent scans suggest small lesions were likely still present and in fact the patient had PR, not CR at the first time point. Under these circumstances, the original CR should be changed to PR and the best response is PR.

“Use of this document is governed by the terms of use on the first page of this document.”

Appendix 4
Response Evaluation Criteria in Solid Tumors Version 1.1 Criteria (cont.)

Special notes on response assessment

When nodal disease is included in the sum of target lesions and the nodes decrease to 'normal' size (< 10 mm), they may still have a measurement reported on scans.
This measurement should be recorded even though the nodes are normal in order not to overstate progression should it be based on increase in size of the nodes. As noted earlier, this means that patients with CR may not have a total sum of 'zero' on the eCRF.
Patients with a global deterioration of health status requiring discontinuation of treatment without objective evidence of disease progression at that time should be reported as 'symptomatic deterioration'. Every effort should be made to document objective progression even after discontinuation of treatment. Symptomatic deterioration is not a descriptor of an objective response: it is a reason for stopping study therapy.
The objective response status of such patients is to be determined by evaluation of target and non-target disease.

In patients with advanced disease and the primary disease is still present or partially present, the primary tumor should be also captured under target or non-target lesions.

REFERENCES

Bogaerts J, Ford R, Sargent D, et al. Individual patient data analysis to assess modifications to the RECIST criteria. Eur J Cancer 2009;45:248–60.

Eisenhauer EA, Therasse P, Bogaerts J, et al. New response evaluation criteria in solid tumors: Revised RECIST guideline (version 1.1). Eur J Cancer 2009;45:228–47.

"Use of this document is governed by the terms of use on the first page of this document."

Appendix 5
Unidimensional Immune–Related Response Criteria (Unidimensional irRC)

Increasing clinical experience indicates that traditional response criteria (e.g., Response Evaluation Criteria in Solid Tumors [RECIST] [1] and WHO) may not be sufficient to characterize fully activity in the new era of target therapies and/or biologics. In studies with cytokines, cancer vaccines, and monoclonal antibodies, complete response, partial response, or stable disease has been shown to occur after an increase in tumor burden as characterized by progressive disease by traditional response criteria. Therefore, conventional response criteria may not adequately assess the activity of immunotherapeutic agents because progressive disease (by initial radiographic evaluation) does not necessarily reflect therapeutic failure. Long-term effect on the target disease must also be captured.

The immune–related response criteria[2,3,4] (irRC) attempt to do that by enhancing characterization of new response patterns that have been observed with immunotherapeutic agents (i.e., ipilimumab).

The investigator will evaluate response to treatment using RECIST version 1.1 and irRC based on unidimensional measurement[3,4].

---

1 Eisenhauer EA, Therasse P, Bogaerts J, et al. New response evaluation criteria in solid tumours: revised RECIST guideline (version 1.1). Eur J Cancer 2009;45:228–47.

2 Wolchok JD, Hoos A, O'Day S, et al. Guidelines for the evaluation of immune therapy activity in solid tumors: immune-related response criteria. Clin Can Res 2009;15:7412–20.
Topalian SL, Hodi FS, Brahmer JR, et al. Safety, activity, and immune correlates of anti–PD-1 antibody in cancer. N Engl J Med 2012;366:2443–54.

3 Nishino M, Giobbie-Hurder A, Gargano M, et al. Developing a common language for tumor response to immunotherapy: immune-related response criteria using unidimensional measurements. Clin. Cancer Res 2013 3936-43.

4 Nishino M, Gargano M, Suda M, Ramaiya NH, Hodi FS. Optimizing immune-related tumor response assessment: does reducing the number of lesions impact response assessment in melanoma patients treated with ipilimumab? J Immunother Cancer 2014, 2:17

"Use of this document is governed by the terms of use on the first page of this document."

Undimensional irRC and RECIST, v1.1: Summary of Changes

	RECIST v1.1	unidimensional irRC
New lesions after baseline	Define progression.	New measurable lesions are added into the total tumor burden and followed.
Non-target lesions	May contribute to the designation of overall progression	Contribute only in the assessment of a complete response
Radiographic progression	First instance of ≥20% increase in the sum of diameters or unequivocal progression in non-target disease	Determined only on the basis of measurable disease

irRC = immune–related response criteria; RECIST = Response Evaluation Criteria in Solid Tumors.

DEFINITIONS OF MEASURABLE/NON-MEASURABLE LESIONS

All measurable and non-measurable lesions should be assessed at screening and at the protocol-specified tumor assessment timepoints. Additional assessments may be performed, as clinically indicated for suspicion of progression. The investigator will evaluate response to treatment using unidimensional irRC.

MEASURABLE LESIONS

Tumor Lesions

Tumor lesions must be accurately measured in at least one dimension (longest diameter in the plane of measurement is to be recorded) with a minimum size as follows:

- 10 mm by computed tomography (CT) or magnetic resonance imaging (MRI) scan (CT/MRI scan slice thickness/interval no greater than 5 mm)
- 10-mm caliper measurement by clinical examination (lesions that cannot be accurately measured with calipers should be recorded as non-measurable)

Malignant Lymph Nodes. To be considered pathologically enlarged and measurable, a lymph node must be ≥ 15 mm in the short axis when assessed by CT scan (CT scan slice thickness recommended to be no greater than 5 mm). At baseline and follow-up, only the short axis will be measured and followed.

"Use of this document is governed by the terms of use on the first page of this document."

NON-MEASURABLE LESIONS

Non-measurable tumor lesions encompass small lesions (longest diameter < 10 mm or pathological lymph nodes with short axis ≥ 10 but < 15 mm), as well as truly non-measurable lesions. Lesions considered truly non-measurable include leptomeningeal disease, ascites, pleural or pericardial effusion, inflammatory breast disease, lymphangitic involvement of skin or lung, peritoneal spread, and abdominal mass/abdominal organomegaly identified by physical examination that is not measurable by reproducible imaging techniques.

SPECIAL CONSIDERATIONS REGARDING LESION MEASURABILITY

Bone lesions, cystic lesions, and lesions previously treated with local therapy require particular comment, as outlined below.

<u>Bone lesions</u>

Bone scan, positron emission tomography (PET) scan, or plain films are not considered adequate imaging techniques for measuring bone lesions. However, these techniques can be used to confirm the presence or disappearance of bone lesions.

Lytic bone lesions or mixed lytic–blastic lesions, with identifiable soft tissue components, that can be evaluated by cross-sectional imaging techniques such as CT or MRI can be considered as measurable lesions if the soft tissue component meets the definition of measurability described above.

Blastic bone lesions are non-measurable

<u>Cystic lesions</u>

Lesions that meet the criteria for radiographically defined simple cysts should not be considered as malignant lesions (neither measurable nor non-measurable) since they are, by definition, simple cysts.

Cystic lesions thought to represent cystic metastases can be considered as measurable lesions, if they meet the definition of measurability described above. However, if non-cystic lesions are present in the same patient, these are preferred for selection as target lesions.

"Use of this document is governed by the terms of use on the first page of this document."

Lesions with prior local treatment

Tumor lesions situated in a previously irradiated area or in an area subjected to other loco-regional therapy are usually not considered measurable unless there has been demonstrated progression in the lesion. Study protocols should detail the conditions under which such lesions would be considered measurable.

TUMOR RESPONSE EVALUATION

DEFINITIONS OF TARGET/NON-TARGET LESIONS

Target Lesions

When more than one measurable lesion is present at baseline, all lesions up to a maximum of five lesions total (and a maximum of two lesions per organ) representative of all involved organs should be identified as target lesions and will be recorded and measured at baseline.

This means that, for instances in which patients have only one or two organ sites involved, a maximum of two lesions (one site) and four lesions (two sites), respectively, will be recorded.

Other lesions (albeit measurable) in those organs will be recorded as non-measurable lesions (even if the size is ≥ 10 mm by CT scan).

Target lesions should be selected on the basis of their size (lesions with the longest diameter) and be representative of all involved organs, but in addition, should lend themselves to reproducible repeated measurements. It may be the case that, on occasion, the largest lesion does not lend itself to reproducible measurement, in which circumstance, the next largest lesion that can be measured reproducibly should be selected.

Lymph nodes merit special mention since they are normal anatomical structures that may be visible by imaging even if not involved by tumor. As noted above, pathological nodes that are defined as measurable and may be identified as target lesions must meet the criterion of a short axis of ≥ 15 mm by CT scan. Only the short axis of these nodes will contribute to the baseline sum. The short axis of the node is the diameter normally used by radiologists to judge if a node is involved by solid tumor. Nodal size is normally reported as two dimensions in the plane in which the image is obtained (for CT, this is almost always the axial plane; for MRI, the plane of acquisition may be axial, sagittal, or coronal). The smaller of these measures is the short axis. For example, an abdominal

“Use of this document is governed by the terms of use on the first page of this document.”

node that is reported as being 20 mm x 30 mm has a short axis of 20 mm and qualifies as a malignant, measurable node. In this example, 20 mm should be recorded as the node measurement.

All other pathological nodes (those with short axis $\geq$10 mm but$<$15 mm) should be considered non-target lesions. Nodes that have a short axis of $<$10 mm are considered non-pathological and should not be recorded or followed.

Lesions irradiated within 3 weeks prior to Cycle 1, Day 1 may not be counted as target lesions.

Non-Target Lesions

All other lesions (or sites of disease), including pathological lymph nodes, should be identified as non-target lesions and should also be recorded at baseline. Measurements are not required.

It is possible to record multiple non-target lesions involving the same organ as a single item on the Case Report Form (CRF) (e.g., "multiple enlarged pelvic lymph nodes" or "multiple liver metastases").

After baseline, changes in non-target lesions will contribute only in the assessment of complete response (i.e., a complete response is attained only with the complete disappearance of all tumor lesions, including non-target lesions) and will not be used to assess progressive disease.

CALCULATION OF SUM OF THE DIAMETERS

A sum of the diameters (longest for non-nodal lesions, short axis for nodal lesions) for all target lesions will be calculated as a measure of tumor burden.

The sum of the diameters is calculated at baseline and at each tumor assessment for the purpose of classification of tumor responses.

Sum of the Diameters at Baseline: The sum of the diameters for all target lesions identified at baseline prior to treatment on Day 1.

Sum of the Diameters at Tumor Assessment: For every on-study tumor assessment collected per protocol or as clinically indicated, the sum of the diameters at tumor assessment will be calculated using tumor imaging scans. All target lesions and *up to five* new measurable lesions that have emerged after baseline will contribute to the sum of the

"Use of this document is governed by the terms of use on the first page of this document."

diameters at tumor assessment. Hence, each net percentage change in tumor burden per assessment using unidimensional irRC accounts for the size and growth kinetics of both old and new lesions as they appear.

RESPONSE CRITERIA

For clarity all tumor assessment using unidimensional irRC uses 'ir' (immune related) as an abbreviation to the respective response category.

Evaluation of Target Lesions

Complete Response (irCR): Disappearance of all target *and non-target* lesions. Lymph nodes that shrink to $<$10 mm short axis are considered normal.

Partial Response (irPR): At least a 30% decrease in the sum of the diameters of all target and all new measurable lesions, taking as reference the baseline sum of diameters, in the absence of CR.

Stable Disease (irSD): Neither sufficient shrinkage to qualify for PR nor sufficient increase to qualify for progressive disease (PD), taking as reference the smallest sum of the diameters while on study.

Progressive Disease (irPD): At least a 20% increase in the sum of diameters of all target and all new measurable lesions, taking as reference the smallest sum on study (this includes the baseline sum if that is the smallest on study). In addition to the relative increase of 20%, the sum must also demonstrate an absolute increase of at least 5 mm.

Impact of New Lesions on unidimensional irRC: New lesions alone do not qualify as progressive disease. However, their contribution to total tumor burden is included in the sum of the diameters (in case of measurable new lesions), which is used to determine the overall unidimensional irRC tumor response.

EVALUATION OF BEST OVERALL RESPONSE USING UNIDIMENSIONAL irRC

TIMEPOINT RESPONSE

It is assumed that at each protocol-specified timepoint, a response assessment occurs.

Table 1 provides a summary of the overall response status calculation at each timepoint for patients who have measurable disease at baseline.

MISSING ASSESSMENTS AND INEVALUABLE DESIGNATION

When no imaging/measurement is done at all at a particular timepoint, the patient is not evaluable (NE) at that timepoint. If only a subset of lesion measurements are made at an assessment, usually the case is also considered NE at that timepoint, unless a convincing argument can be made that the contribution of the individual missing lesion(s) would not change the assigned time point response. This would be most likely to happen in the case of PD. For example, if a patient had a baseline sum of 50 mm with three measured lesions and at follow-up only two lesions were assessed but those gave a sum of 80 mm, the patient will have achieved PD status, regardless of the contribution of the missing lesion.

"Use of this document is governed by the terms of use on the first page of this document."

Table 1 Unidimensional irRC Timepoint Response

% Change in sum of the diameters (including measurable new lesions when present)	Target lesion assessment	Non-target lesion assessment	New measurable lesions	New unmeasurable lesions	Overall unidimensional irRC timepoint response
-100%[1]	irCR	irCR	No	No	irCR
-100%[1]	irCR	Non-irCR or NE	No	No	irPR
≤-30%	irPR	Any	Yes or no	Yes or no	irPR
>-30% to <+20%	irSD	Any	Yes or no	Yes or no	irSD
Not all evaluated	NE	Any	Yes or no	Yes or no	NE
≥+20%	irPD	Any	Yes or no	Yes or no	irPD

irCR = complete response; NE = not evaluable; irPD = progressive disease; irPR = partial response; RECIST = Response Evaluation Criteria in Solid Tumors; irSD = stable disease.

[1] When lymph nodes are included as target lesions, the percent change in the sum of the diameters may not be 100% even if complete response criteria are met since a normal lymph node is defined as having a short axis of < 10 mm. Any pathological lymph nodes (whether target or non-target) must have reduction in short axis to < 10 mm in order to meet the definition of irCR.

BEST OVERALL RESPONSE: ALL TIMEPOINTS

The best overall response is determined once all the data for the patient are known.

The best overall response according to unidimensional irRC is interpreted as below:

- **irCR**: Complete disappearance of all tumor lesions (target and non-target) and no new measurable or unmeasurable lesions, confirmed by a consecutive assessment ≥4 weeks from the date first documented. All lymph nodes short axes must be <10 mm.
- **irPR**: Decrease in the sum of the diameters of all target and all new measurable lesions ≥30% relative to baseline, in the absence of irCR, confirmed by a consecutive assessment ≥4 weeks from the date first documented.
- **irSD**: Criteria for irCR and irPR are not met *and no irPD observed prior to the first irSD*.
- **irPD**: *Criteria for irCR, irPR, and irSD not met and increase* in the sum of the diameters of all target and all new measurable lesions ≥20% relative to the nadir.

Patients may achieve a best overall response of irPR or irCR based on tumor regression achieved at any time prior to study treatment discontinuation.

"Use of this document is governed by the terms of use on the first page of this document."

Table 2 Summary of Measurement and Response Assessment Approaches for Unidimensional Assessment Based on irRC[4]

Measurable lesions	≥10 mm in the longest diameter (cm)<br>Malignant lymph nodes must be ≥15 mm in short axis
Measurement	Longest diameter for non-nodal lesions<br>Short axis for malignant lymph nodes
Target lesions	Measurable lesions, defined at baseline<br>Up to a maximum of 5 lesions in total, maximum of 2 per organ
Non-target lesions	Defined at baseline<br>All non-measurable lesions and excess measurable lesions
New lesions	Lesions not present at baseline<br>The presence of new lesion(s) alone does not define progression. The measurements of the new lesion(s) are included in the sum of the diameters
The sum of the diameters	The sum of the diameters of all target lesions and *up to 5* new measurable lesions
Response assessment	irCR: Complete disappearance of all lesions (target, non-target and new lesions)<br>irPR: ≥30% decrease from baseline<br>irSD: Not meeting criteria for irPD,irPR/irCR<br>irPD: ≥20% increase from the nadir<br>New measurable lesions do not define irPD, incorporated into the sum of the diameters<br>New non-measurable lesions do not define irPD but preclude irCR
Confirmation	Confirmation by 2 consecutive observations not less than 4 weeks apart is required for irCR and irPR

"Use of this document is governed by the terms of use on the first page of this document."

Appendix 6
Statistical Design of modified Continual Reassessment Method with Escalation with Overdose Control

Various dose-toxicity scenarios have been investigated in order to cover a wide range of dose-toxicity possibilities and to be able to quantify the risk and benefit, should these scenarios actually occur.

In Table 1 the scenario settings are described, in terms of the assumed parameters α and Î_ and the reference dose d^* for the logistic model (see Section 6.7.1). The dose range [mg] that results from the requirement of target toxicity between 20% and 35% is given in the last two columns. Scenario 2 is closest to the prior model, and scenarios 3 and 8 are extreme cases with very high toxicity already at very low doses.

Table 1 Scenario Settings

No.	α	Î_	d^*	Lower target	Upper target
1	-3.00	2.00	80	179.27	234.716
2	0.70	0.40	500	2.72	10.448
3	1.30	0.30	45	< 0.05	0.035
4	-2.00	1.50	40	60.22	86.258
5	-5.00	5.00	30	61.80	68.837
6	-2.00	2.00	500	679.57	889.767
7	-5.00	0.20	35	> 1000	> 1000
8	1.30	1.50	5	0.83	1.195
9	-2.50	2.00	200	349.03	456.993

In this appendix, the resulting dose-toxicity curves from the scenarios (different colours and line types as per legend) are compared with the prior mean curve (black continuous line). Scenario 2 is closest to the prior, scenarios 6, 7, 9 have lower toxicity with varying steepness of the dose-toxicity curve and scenarios 1, 3, 4, 5 and 8 have higher toxicity.

“Use of this document is governed by the terms of use on the first page of this document.”

Figure 1 Comparison of Scenario Dose-Toxicity Curves

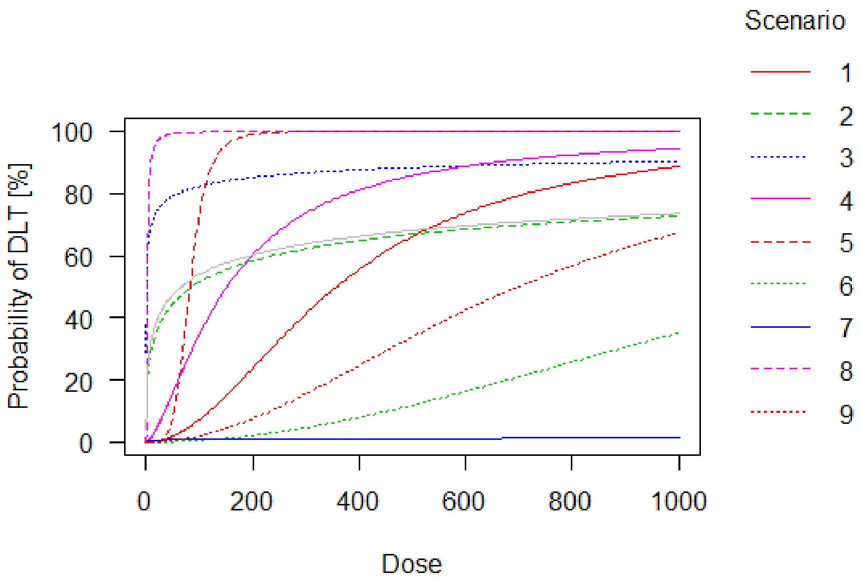

DLT = dose-limiting toxicity.

"Use of this document is governed by the terms of use on the first page of this document."

Operating characteristics were tabulated for each of these scenarios, as per Table 1. The numbers were computed based on 200 simulations for each of the scenarios. A dose grid of 0.03, 0.05, 0.10, 0.15, …, 2.45, 2.50, 3, 4, …, 79, 80, 85, 90, …, 995, 1000 mg, was used throughout. In each scenario, cohorts of 1 patient were used for Part I, while 4 patients were used for Part II, reflecting the typically used cohort size. In line with the protocol, the first patient was simulated separately. If a dose-limiting toxicity (DLT) occurred in this first patient, the cohort was closed and the next dose was recommended by the modified continual reassessment method (mCRM) with escalation with overdose control (EWOC) design. Otherwise 3 additional patients were recruited into the cohort, and only then the next dose was recommended by the mCRM with EWOC design. If at one point during the trial, no dose was acceptable because of the overdosing rule (the risk of having more than 35% DLT probability must be $<$ 25%), then the trial is stopped and a dose 0 is returned as the dose recommendation.

In Figures 2–10, additional insight into the operating characteristics can be gained by graphical summaries of the distributions of important read-outs and resulting model fits, separately for each scenario. The frequentist distributions of the sample size n ("number of patients in total"), the final maximum-tolerated dose (MTD) estimate, the proportion of DLTs (%) and the number of overdosed patients n_{overdose} ("number of patients above target") are shown as histograms. In addition, the bottom panel in each figure compares the true toxicity curve from the scenario with the average model fit from the 200 simulation runs.

"Use of this document is governed by the terms of use on the first page of this document."

Figure 2 True versus Average Estimated Toxicity Curve in Scenario 1

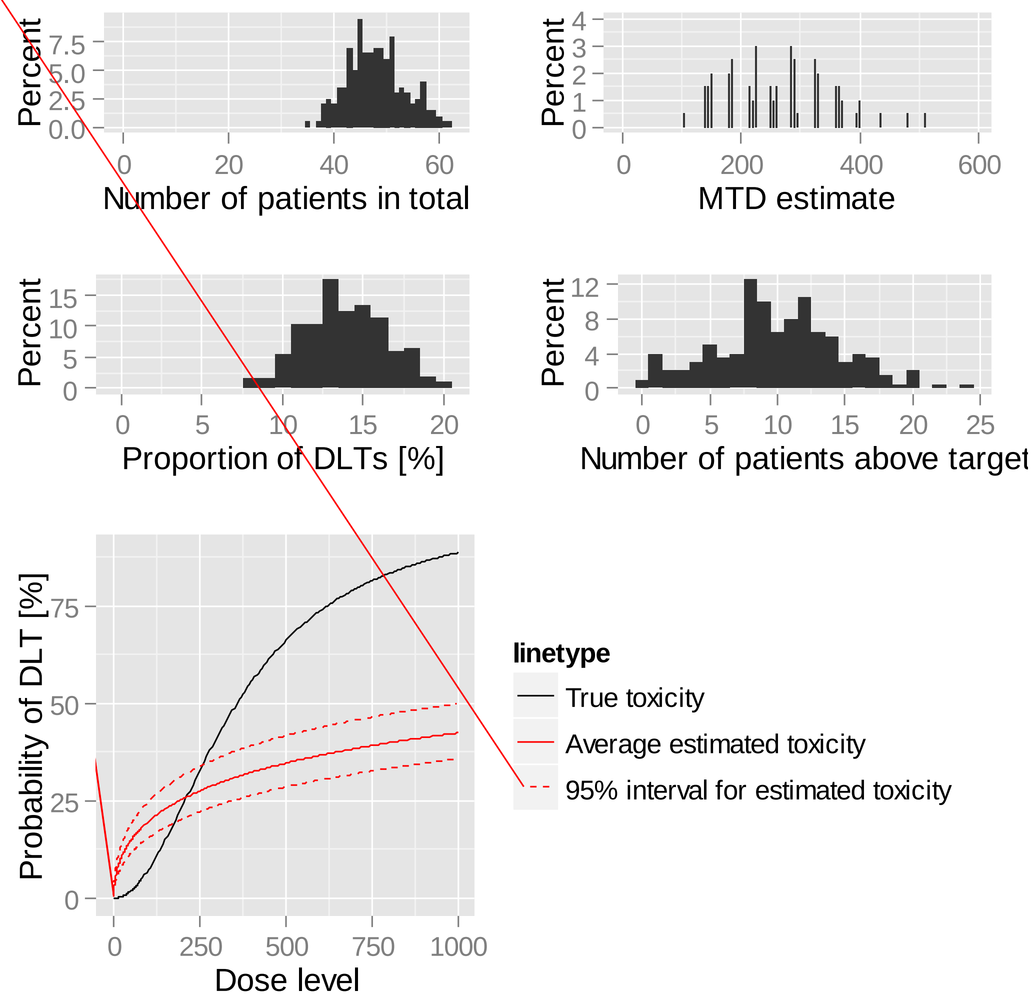

DLT = dose-limiting toxicity; MTD = maximum-tolerated dose.

"Use of this document is governed by the terms of use on the first page of this document."

Figure 3 True versus Average Estimated Toxicity Curve in Scenario 2

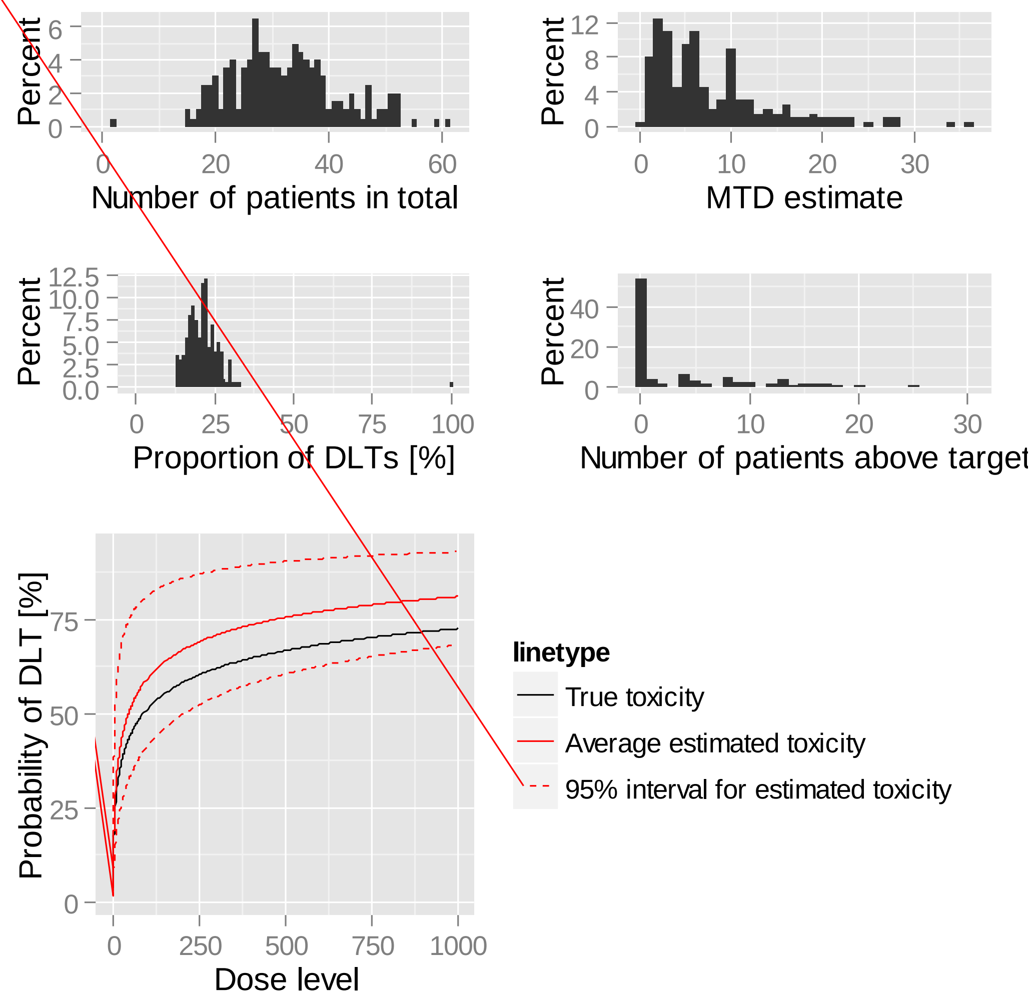

DLT = dose-limiting toxicity; MTD = maximum-tolerated dose.

"Use of this document is governed by the terms of use on the first page of this document."

Figure 4 True versus Average Estimated Toxicity Curve in Scenario 3

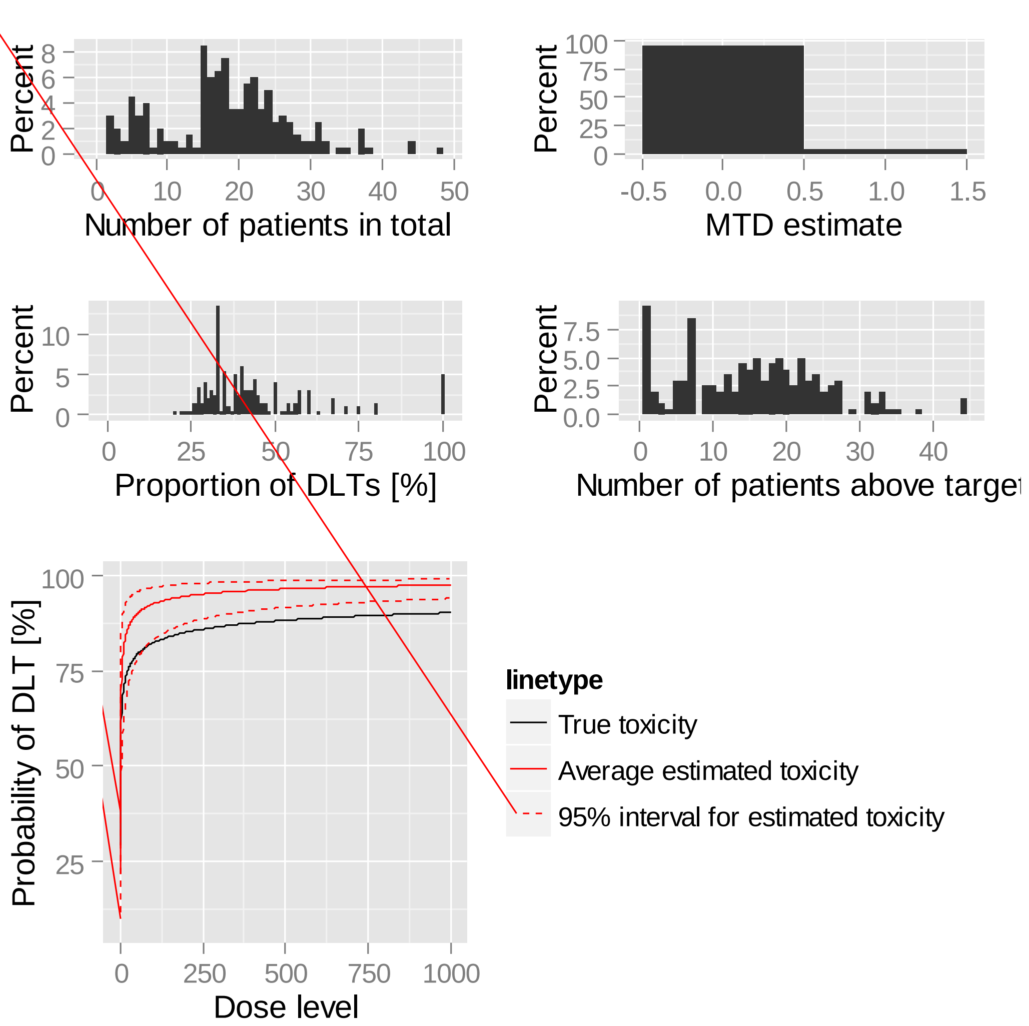

DLT = dose-limiting toxicity; MTD = maximum-tolerated dose.

“Use of this document is governed by the terms of use on the first page of this document.”

Figure 5 True versus Average Estimated Toxicity Curve in Scenario 4

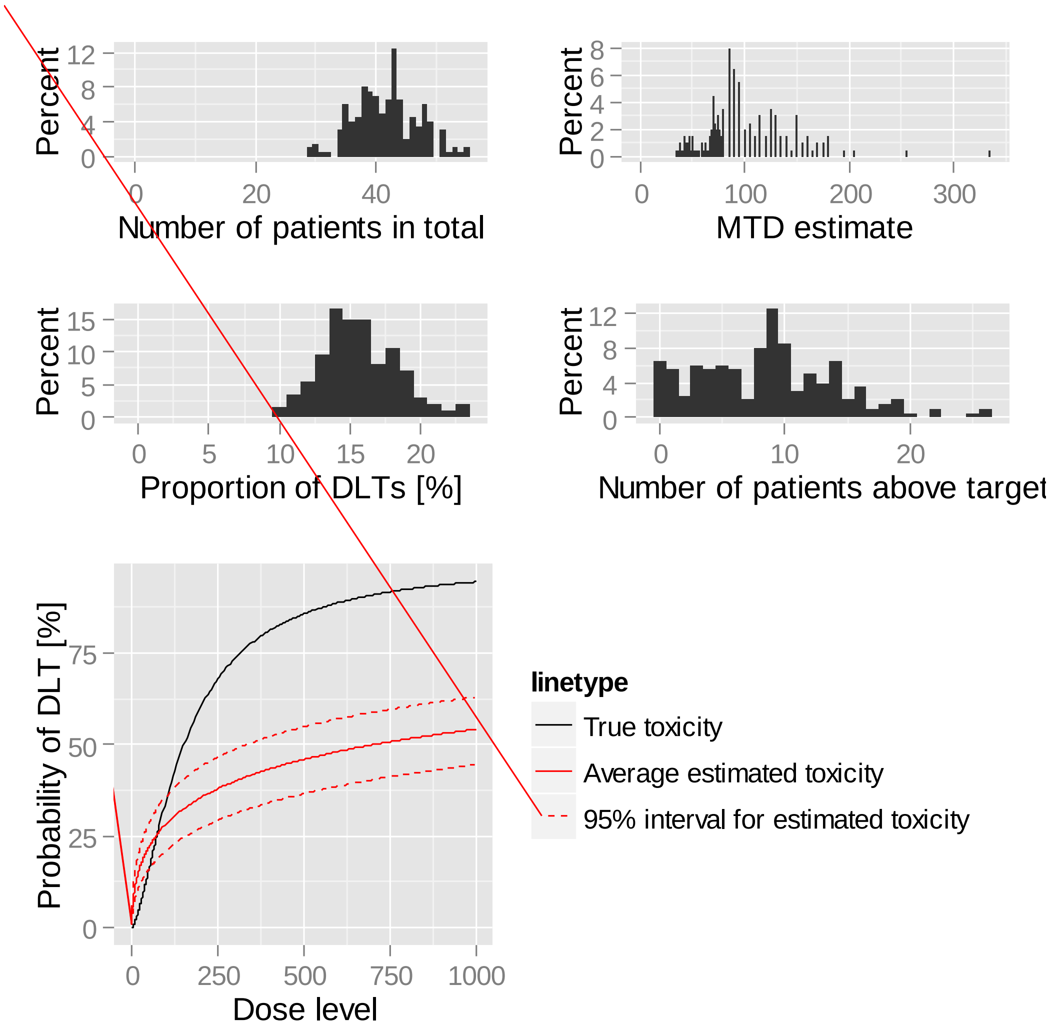

DLT = dose-limiting toxicity; MTD = maximum-tolerated dose.

“Use of this document is governed by the terms of use on the first page of this document.”

Figure 6 True versus Average Estimated Toxicity Curve in Scenario 5

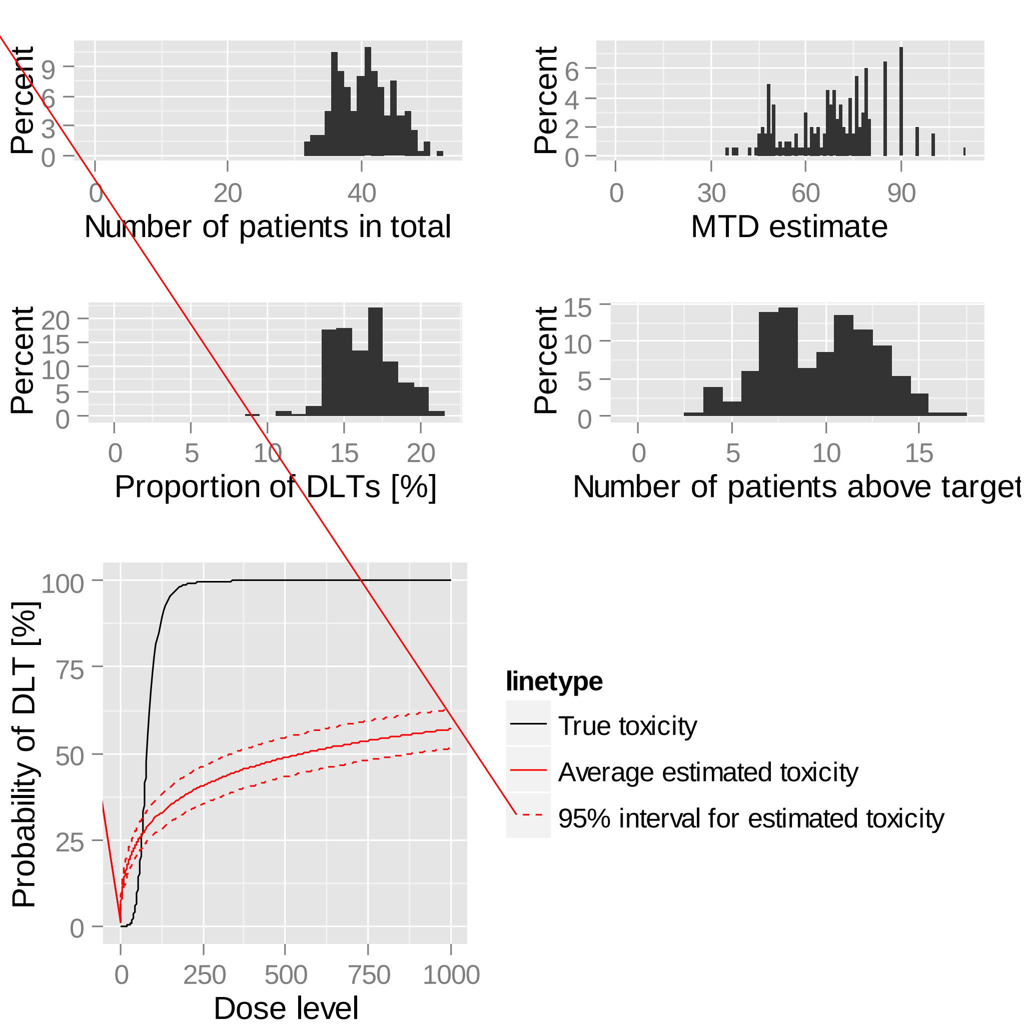

DLT = dose-limiting toxicity; MTD = maximum-tolerated dose.

“Use of this document is governed by the terms of use on the first page of this document.”

Figure 7 True versus Average Estimated Toxicity Curve in Scenario 6

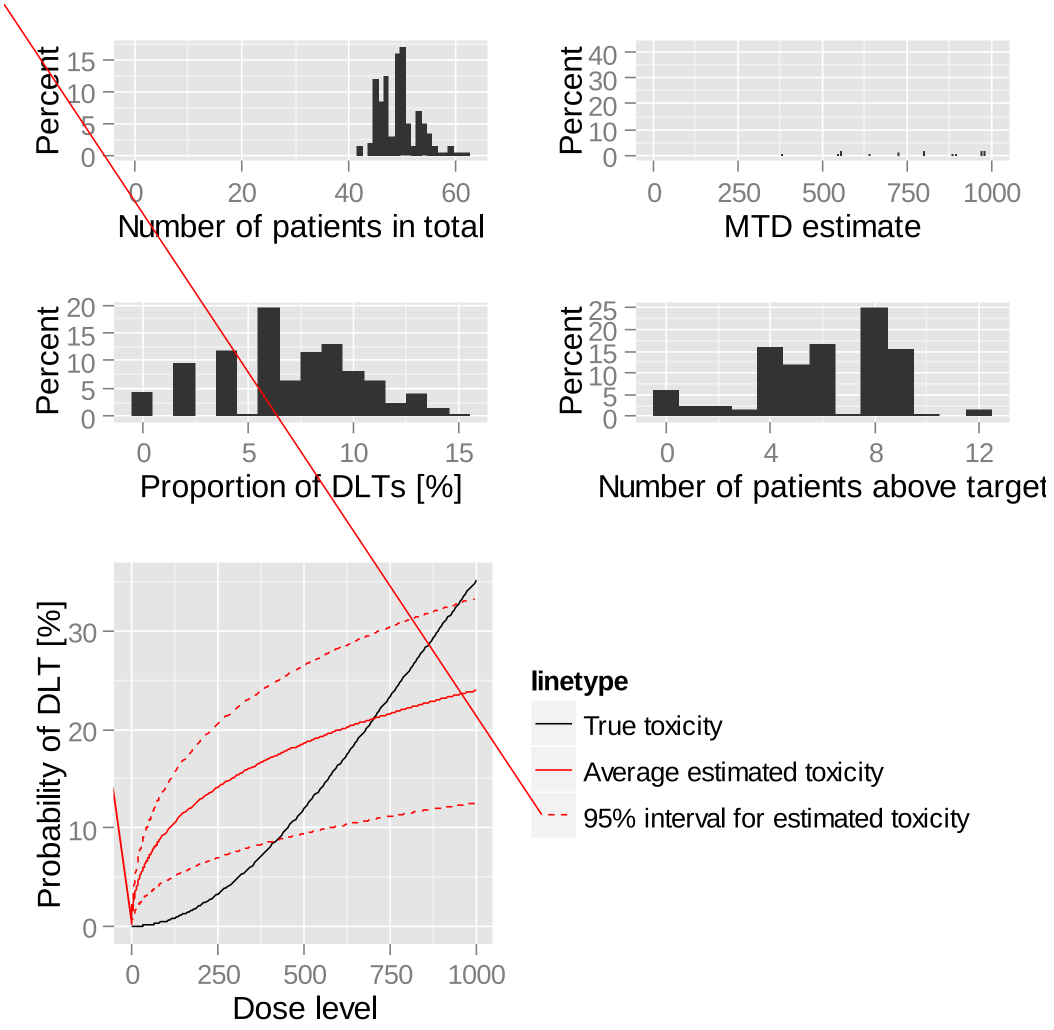

DLT = dose-limiting toxicity; MTD = maximum-tolerated dose.

"Use of this document is governed by the terms of use on the first page of this document."

Figure 8 True versus Average Estimated Toxicity Curve in Scenario 7

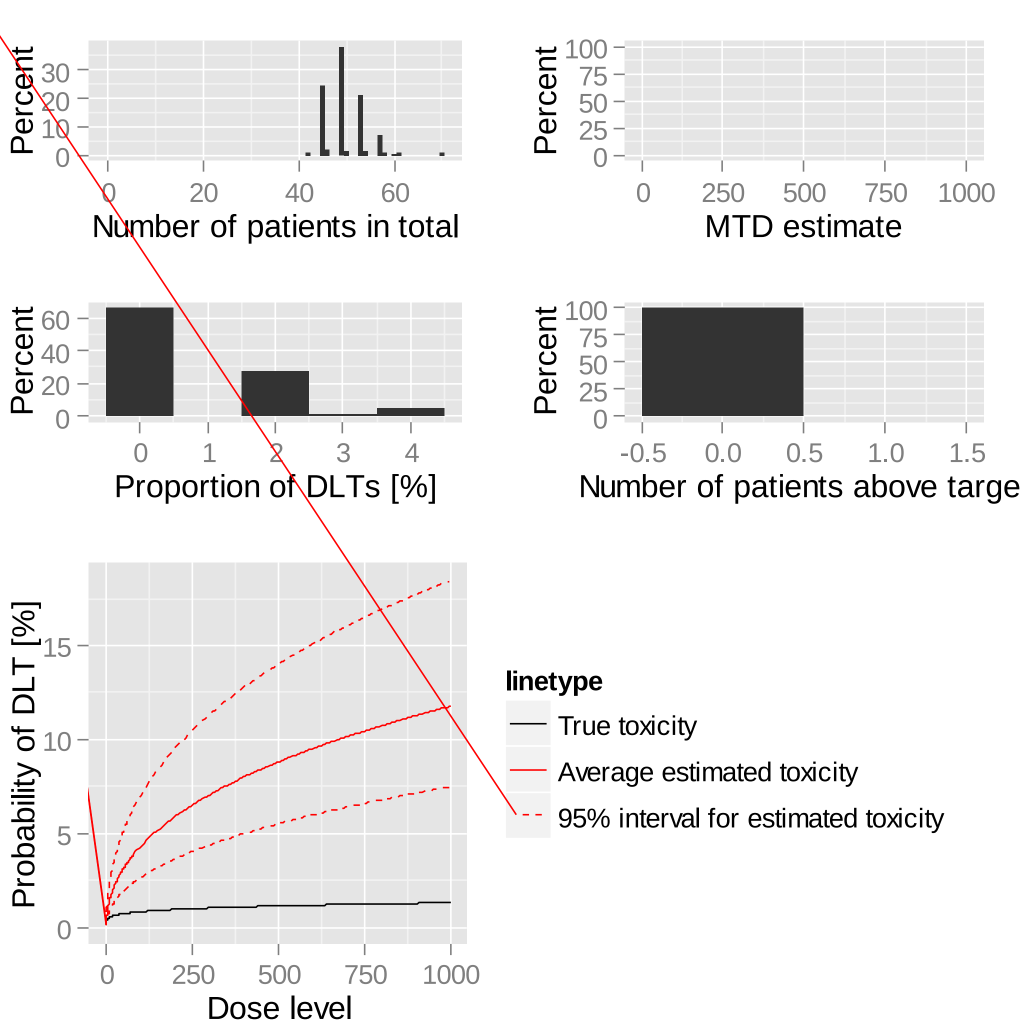

DLT = dose-limiting toxicity; MTD = maximum-tolerated dose.

"Use of this document is governed by the terms of use on the first page of this document."

Figure 9 True versus Average Estimated Toxicity Curve in Scenario 8

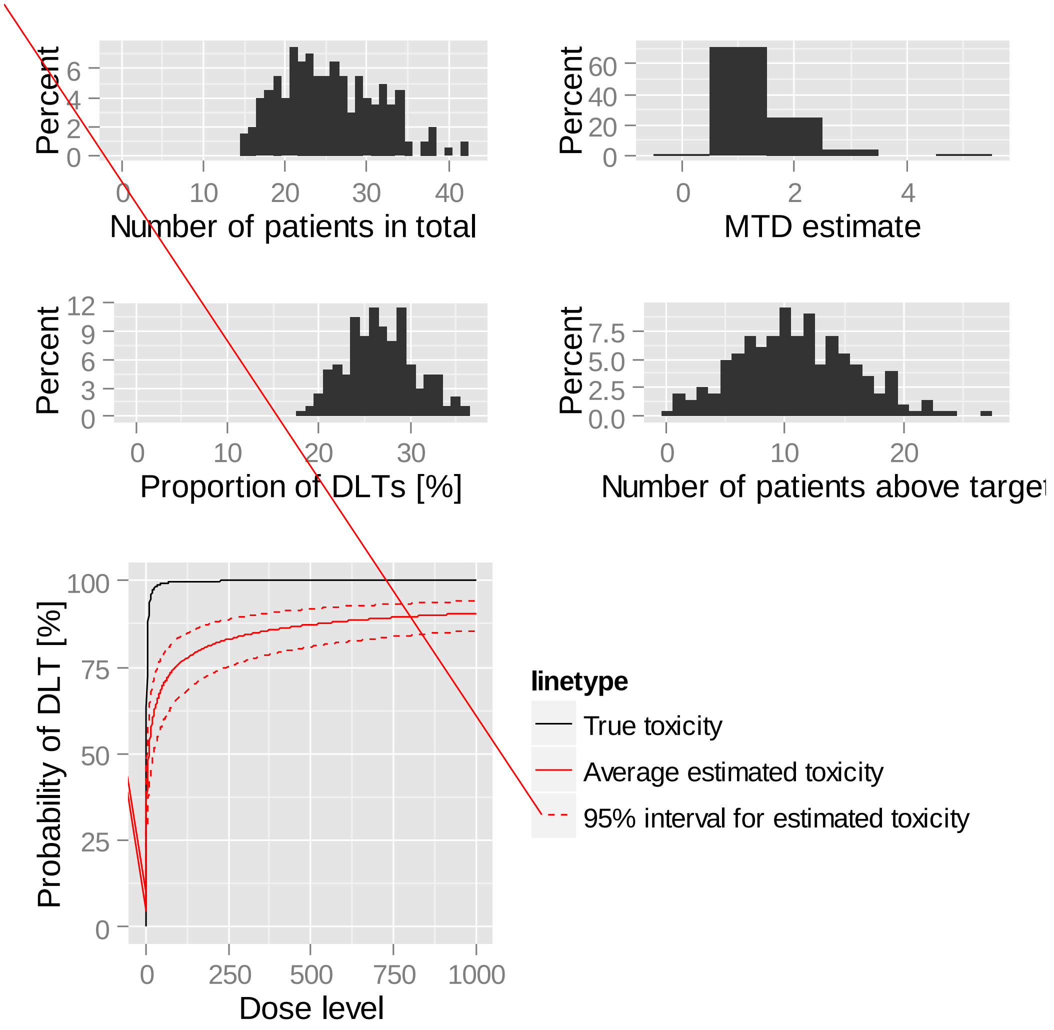

DLT = dose-limiting toxicity; MTD = maximum-tolerated dose.

"Use of this document is governed by the terms of use on the first page of this document."

Figure 10 True versus Average Estimated Toxicity Curve in Scenario 9

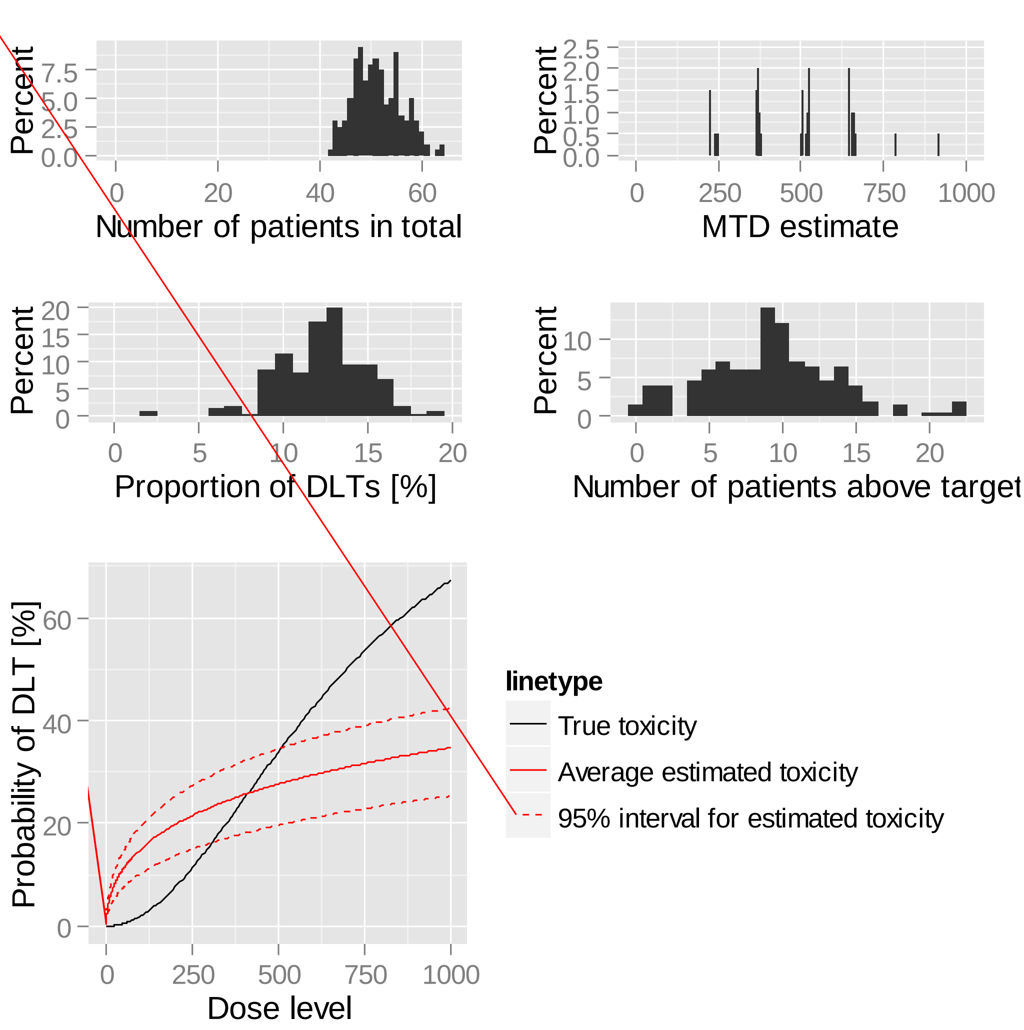

DLT = dose-limiting toxicity; MTD = maximum-tolerated dose.

“Use of this document is governed by the terms of use on the first page of this document.”

As the key read-outs were already summarized in Section 6.7.1, the focus is now on summarizing the comparison of true dose-toxicity curves and the average estimated dose-toxicity curves at the end of the dose escalation stage. Overall it can be said that the estimated curves approximate the true curves reasonably well in all scenarios in the relevant range up to 35% probability of DLT. It is obviously more difficult for the model to approximate higher dose ranges, because only very few patients should and will be allocated there. However, there are differences between the scenarios. Scenarios 2 and 3 look particularly good in terms of model fit. Even for the other high toxicity scenario 8 there is reasonable model fit in the relevant dose range. In scenarios 6, 7 and 9, which have much flatter dose-toxicity curves than expected a priori, the model fit is not as good as in the other scenarios. However, this results in a tendency to be more conservative than what is actually needed in the scenario, with the red line in scenario 7 being always above the true line.

"Use of this document is governed by the terms of use on the first page of this document."

Appendix 7 Modified 3+3 dose escalation design simulation results

The modified 3+3 dose escalation design for cohort A was tested in three scenarios, which featured low (40 mg), medium (400 mg) and high (1200 mg) late-cycle MTDs, respectively. The corresponding conditional probabilities of experiencing a DLT, given that no DLT appeared in lower dose levels, were defined as in Table 1. Please note that the mathematical definition of the late-cycle MTD is the highest dose level where the cumulative probability of experiencing a DLT at this or lower dose levels does not exceed 33%.

Table 1 Conditional probabilities of DLTs defining the scenarios

Dose:	40	80	160	300	400	600	800	1200
“Low”	0.1	0.2	0.3	0.4	0.5	0.5	0.6	0.7
“Medium”	0.01	0.02	0.04	0.05	0.2	0.4	0.6	0.8
“High”	0.01	0.01	0.01	0.01	0.04	0.05	0.05	0.05

The trials were executed as described in Section 6.7.1.2 with 6 patients in total, and 10,000 trials were simulated for each scenario. The performance of the modified 3+3 dose escalation design is summarized in the below figures. They comprise the scenario definition (cumulative DLT probabilities), the distribution of the final late-cycle MTD estimates, the number of weeks until it took to define the late cycle MTD, and the number of DLTs which occurred during the trial.

Overall, the design performs acceptably well. The true MTD is indeed the final MTD estimate in most of the simulations. The patients’ safety is protected, as even in the most severe toxicity scenario (low late cycle MTD), not more than 3 patients experience a DLT in more than 80% of all simulations.

“Use of this document is governed by the terms of use on the first page of this document.”

Figure 1 Simulation results for the low late-cycle MTD scenario

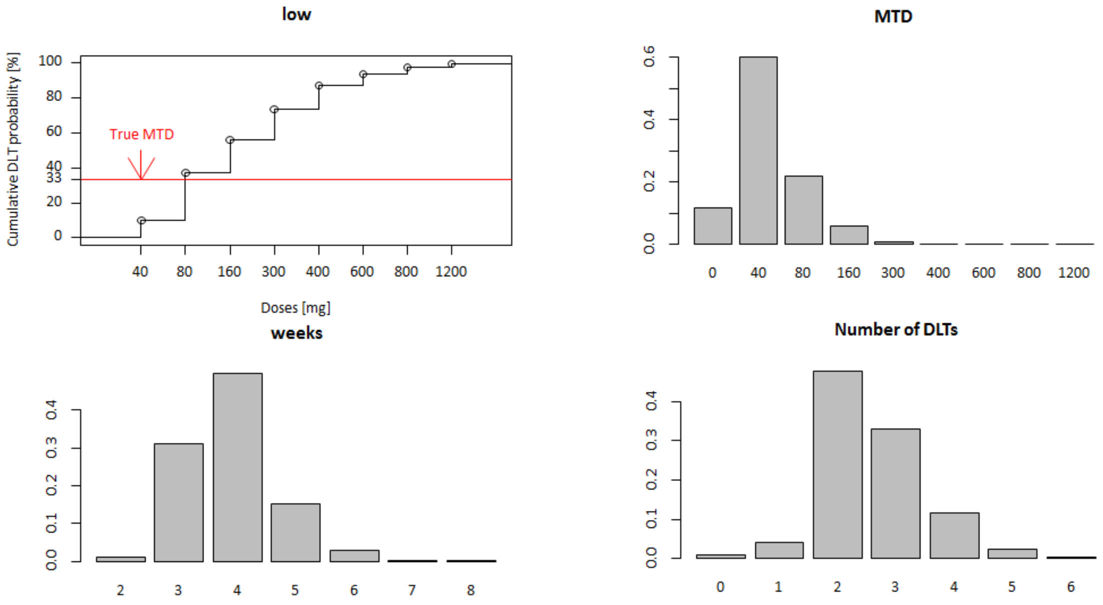

Figure 2 Simulation results for the medium late-cycle MTD scenario

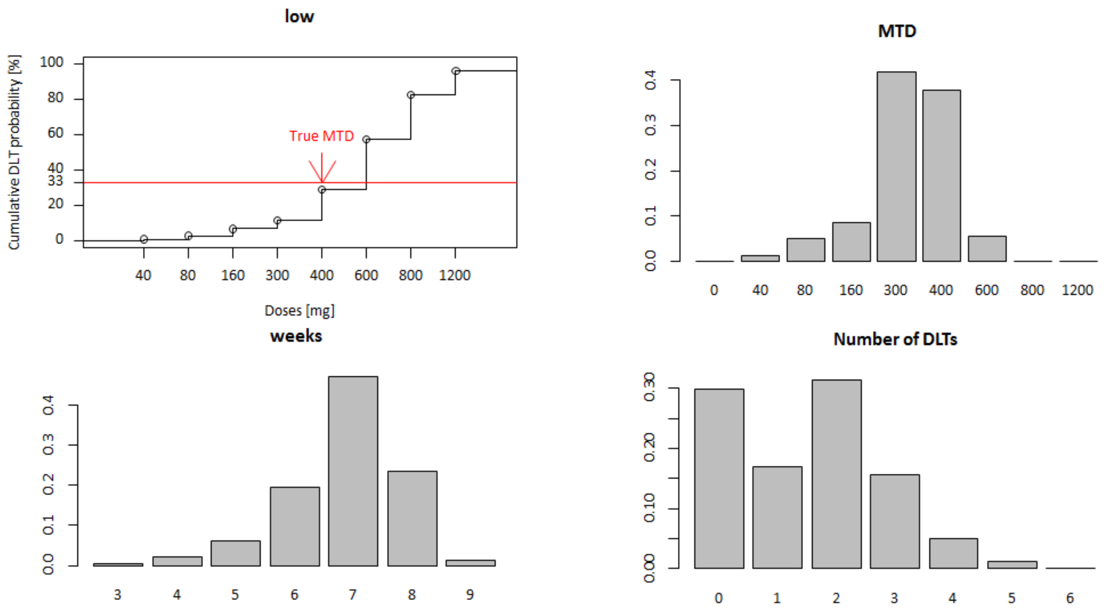

“Use of this document is governed by the terms of use on the first page of this document.”

Appendix 7 Modified 3+3 dose escalation design simulation results (cont.)

Figure 3 Simulation results for the high late-cycle MTD scenario

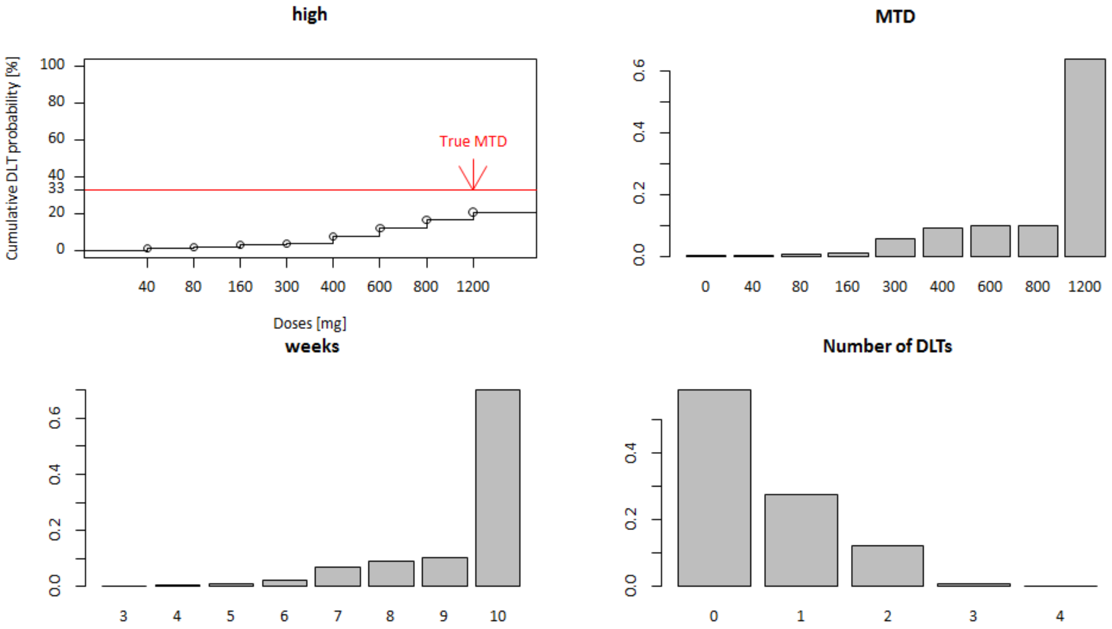

"Use of this document is governed by the terms of use on the first page of this document."

Appendix 8 GILBERT’S SYNDROME DEFINITION

Patients with Gilbert’s syndrome will be eligible for the study. The diagnosis of Gilbert's syndrome is suspected in people who have persistent, slightly elevated levels of unconjugated bilirubin without any other apparent cause. A diagnosis of Gilbert’s syndrome will be based on the exclusion of other diseases based on the following criteria:

- Unconjugated hyperbilirubinemia noted on several occasions
- No evidence of hemolysis (normal hemoglobin, reticulocyte count and LDH)
- Normal liver function tests
- Absence of other diseases associated with unconjugated hyperbilirubinemia

“Use of this document is governed by the terms of use on the first page of this document.”

Appendix 9 [^{18}F] FDG-PET

Introduction

FDG (^{18}F Fluoro-Deoxy-Glucose) PET imaging will be performed in this study.
The patient needs to fast for typically 4 to 6 hours prior to the FDG-PET scan. The patient can drink water freely during the fast and should be encouraged to do so. He/she may also take regular medications as scheduled during the fast. Blood glucose level will be checked on the day of the FDG-PET scan and results assessed prior to the administration of FDG. The patient should have a blood glucose level ≤ 180 mg/dL (≤ 10 mmol/L) in order to have the FDG-PET scan. If the level is higher, the scan should be rescheduled if possible.
Regular diet can be resumed after the scan.
The interval between FDG administration and scanning must be 60 minutes +/- 10 minutes and it is particularly important that the time interval between injection and start of the scan is the same at follow-up compared to baseline.
Of note PET must be performed before biopsy to avoid potential false positive findings whenever possible. However, during the screening period, baseline FDG PET assessment should be performed at the closest date as possible from start of study treatment. All patients should be encouraged to increase fluid intake for a few hours after the scan to promote excretion of the FDG. A diuretic (furosemide, typically 20-40 mg IV) may be administered at the discretion of the investigator before or during the FDG-PET scan in order to accelerate elimination of the [^{18}F]-FDG from the renal collecting system.
Diazepam may be used to promote muscle relaxation and reduce muscular uptake if tumor deposits are in the neck or shoulder girdle area. Diazepam administration and diuretic administration must be recorded in the Concomitant Medications eCRF page.

Scan Acquisition

Attenuation corrected FDG PET scans (from skull base to mid-thigh) will be performed. The patient will be administered 370–740MBq (10-20mCi) 2-[F-18]-fluoro-2-deoxy-D glucose (FDG) intravenously (dose is dependent on local practice and scanner type). The administered activity and time of FDG administration and body weight on the day of scanning must be recorded for subsequent calculation of maximum tumor standardized uptake value (SUVmax). Approximately one hour following the administration of FDG, a whole body PET scan (base of skull to thighs) is performed and the time of the commencement of the scanning is recorded. The sequence of the scan must be the same for the initial scan and all follow-up scans, and the interval between FDG administration and scanning should be as similar as practical.

Preferably for a given patient the same scanner should be used at all time-points. The FDG-PET images will be analyzed by the site.

Local and central Scan Interpretation

The FDG-PET images will be analyzed for SUVmax uptake by an experienced reader (the same reader should interpret all scans for each subject) and by central reading if needed. In addition to SUVmax evaluation, a visual assessment of extent, intensity and changes in FDG pattern of uptake, including appearance of new FDG lesions will be made.

“Use of this document is governed by the terms of use on the first page of this document.”

Appendix 10 Schedule of Assessments for Tocilizumab Treatment of Severe or Life-Threatening Cytokine Release Syndrome

Assessment/Procedure	Pre-TCZ Treatment (within 24 hours)	TCZ Administration	Post-TCZ Treatment [c]		
			End of infusion	2 hours	24 hours
TCZ Administration (8 mg/kg)		x			
Vital signs [a]	x [g]		Measure at least every 6 hours until resolution to baseline [g]		
Pressor documentation [b]	x [g]		Record at least every 6 hours until pressors are discontinued [g]		
FiO_2	x [g]		Record at least every 6 hours until patient on room air [g]		
Pulse oximetry, resting	x [g]		Measure at least every 6 hours until resolution to baseline [g]		
Local Laboratory Assessments					
Hematology	x		x	x	x
Liver function tests (AST, ALT, total bilirubin)	x		x	x	x
Serum chemistry and creatinine [d]	x		x	x	x
CRP, LDH, and serum ferritin	x		x	x	x
Coagulation (aPTT, PT/INR, fibrinogen)	x		x	x	x
Infection workup [e]	x				
Central Laboratory Assessments					
Plasma cytokines	x		x	x	x
Plasma IL-6 pharmacodynamic markers [f]	x	x	x	x	x

"Use of this document is governed by the terms of use on the first page of this document."

Appendix 10 Schedule of Assessments for Tocilizumab Treatment of Severe or Life-Threatening Cytokine Release Syndrome (cont.)

aPTT = activated partial thromboplastin time; CRP = C-reactive protein; CRS = cytokine release syndrome; eCRF = electronic Case Report Form; INR = international normalized ratio; IL-6 = interleukin 6; LDH = lactate dehydrogenase; PT = prothrombin time; TCZ = tocilizumab.

Record abnormalities or worsened clinically significant abnormalities on the Adverse Event eCRF.

[a] Includes respiratory rate, heart rate, and systolic and diastolic blood pressure while the patient is in a seated or supine position, and temperature.

[b] Document vasopressor type and dose in the concomitant medication eCRF.

[c] If TCZ dose is repeated, follow Schedule of Assessments following the second TCZ dose.

[d] Includes sodium, potassium, chloride, bicarbonate, glucose and blood urea nitrogen

[e] Includes assessment for bacterial, fungal, and viral infections.

[f] Includes IL-6, and other cytokines such as soluble IL-6R, sgp130, IL-8 etc.

[g] The maximum and minimum values for any 24-hour period should be recorded in the clinical database.

“Use of this document is governed by the terms of use on the first page of this document.”

Appendix 11
High Dose Vasopressors

High-Dose Vasopressor (duration ≥3 hours)	
**Pressor**	**Dose**
Norepinephrine monotherapy	≥20 mcg/min
Dopamine monotherapy	≥10 mcg/kg/min
Phenylephrine monotherapy	≥200 mcg/min
Epinephrine monotherapy	≥10 mcg/min
If on vasopressin	Vasopressin + norepinephrine equivalent of ≥10 mcg/min [a]
If on combination vasopressors (not vasopressin)	Norepinephrine equivalent of ≥20 mcg/min [a]

mcg = microgram; min = minute; VASST = Vasopressin and Septic Shock Trial.

Source: Russell et al. N Engl J Med 2008;358:877–88.

[a] VASST vasopressor equivalent equation: norepinephrine equivalent dose = [norepinephrine (mcg/min)] + [dopamine (mcg/kg/min)] + [phenylephrine (mcg/min) ÷ 10].

“Use of this document is governed by the terms of use on the first page of this document.”

PROTOCOL

**TITLE:**	**AN OPEN-LABEL, MULTICENTER, DOSE ESCALATION AND EXPANSION PHASE Ib STUDY TO EVALUATE THE SAFETY, PHARMACOKINETICS, AND THERAPEUTIC ACTIVITY OF RO6958688 IN COMBINATION WITH ATEZOLIZUMAB IN PATIENTS WITH LOCALLY ADVANCED AND/OR METASTATIC CEA-POSITIVE SOLID TUMORS**
**PROTOCOL NUMBER:**	WP29945
**VERSION:**	11
**EUDRACT NUMBER:**	2015-003771-30
**IND NUMBER:**	122931
**NCT NUMBER:**	NCT02650713
**TEST PRODUCTS:**	RO6958688 and Atezolizumab
**MEDICAL MONITOR:**	[redacted]
**SPONSOR:**	F. Hoffmann-La Roche Ltd
**DATE FINAL:**	Version 1: 18 September 2015
**DATES AMENDED:**	Version 2: 19 February 2016<br>Version 3: 29 April 2016<br>Version 4: 13 December 2016<br>Version 5: 3 August 2017<br>Version 6: 12 October 2017<br>Version 7: 19 January 2018<br>Version 8: 18 April 2018<br>Version 9: 24 September 2018<br>Version 10: 18 May 2019<br>Version 11: See electronic date stamp below.

PROTOCOL AMENDMENT APPROVAL

Date and Time (UTC)	Title	Approver's Name
05-Nov-2019 18:32:00	[redacted]	[redacted]

CONFIDENTIAL

This clinical study is being sponsored globally by F. Hoffmann-La Roche Ltd of Basel, Switzerland. However, it may be implemented in individual countries by Roche's local affiliates, including Genentech, Inc. in the United States. The information contained in this document, especially any unpublished data, is the property of F. Hoffmann-La Roche Ltd (or under its control) and therefore is provided to you in confidence as an investigator, potential investigator, or consultant, for review by you, your staff, and an applicable Ethics Committee or Institutional Review Board. It is understood that this information will not be disclosed to others without written authorization from Roche except to the extent necessary to obtain informed consent from persons to whom the drug may be administered.

"Use of this document is governed by the terms of use on the first page of this document."

PROTOCOL AMENDMENT, VERSION 11: RATIONALE

Protocol WP29945 has been amended to remove the 120-day sample pharmacokinetic (PK) and anti-drug antibody (ADA) collections and to update atezolizumab safety risks to align with latest Atezolizumab Investigator's Brochure. Changes to the protocol, along with a rationale for each change, are summarized below:

- Background information on atezolizumab has been updated to account for additional approved indications (Section 1.4).
- To align with the Atezolizumab Investigator's Brochure, Version 15, "immune-related" has been changed to "immune-mediated" when describing events associated with atezolizumab (Sections 1.4.2.1, 3.1.2.1, 4.4.2, and 5.2.2 and Appendices 10 and 11).
- To address a request by the French National Agency for the Safety of Medicines and Health Products (ANSM), systemic immune activation has been replaced by hemophagocytic lymphohistiocytosis (HLH) and macrophage activation syndrome (MAS) in the list of potential risks for atezolizumab (Sections 5.2.2, 5.2.4, and 5.2.6.3.5) and the management guidelines for systemic immune activation have been replaced with management guidelines for HLH and MAS (Appendices 10 and 11). In addition, systemic immune activation has been removed from the list of adverse events of special interest (Sections 1.4.2.1, 1.6.2, and 5.1.3).
- It has been clarified that eligibility will be confirmed, not approved, by the Medical Monitor, as approval resides with Principal Investigator (Section 4.2.3).
- The 120-day sample PK and ADA collections have been removed because the results do not change the ADA incidence rate; most of atezolizumab-ADA positivity is transient and occurs around 21 days after the first atezolizumab dose, which makes this sample result uninformative and unnecessary (Sections 4.5.2.3, 4.5.2.4, 4.6.1, and 5.6 and Appendix 1 Tables A1, A2, A4–A9). Nevertheless, as per Section 5.3.1, after initiation of study drugs, all adverse events, regardless of relationship to study drugs, will be reported until 28 days (for RO6958688) or 120 days (for atezolizumab) after the final dose of study drugs or until initiation of new systemic anti-cancer therapy, whichever occurs first.
- To address a request by the French ANSM, the atezolizumab adverse event management guidelines have been revised to add laboratory (e.g., B-type natriuretic peptide) and cardiac imaging abnormalities as signs or symptoms that are suggestive of myocarditis (Appendix 11).

Additional minor changes have been made to improve clarity and consistency. Substantive new information appears in italics. This amendment represents cumulative changes to the original protocol.

“Use of this document is governed by the terms of use on the first page of this document.”

TABLE OF CONTENTS

PROTOCOL AMENDMENT ACCEPTANCE FORM 11

PROTOCOL SYNOPSIS 12

1. BACKGROUND AND RATIONALE 43

1.1 Background on Disease 43

1.2 Background on Immunotherapy 43

1.3 Background on RO6958688 44

1.3.1 Previous Non-Clinical Studies 46

1.3.2 Previous Clinical Studies 52

1.3.2.1 Safety of RO6958688 52

1.3.2.2 Efficacy of RO6958688 56

1.3.2.3 Clinical Pharmacokinetics of RO6958688 58

1.3.2.4 Immunogenicity of RO6958688 59

1.4 Background on Atezolizumab 59

1.4.1 Previous Non-Clinical Studies 60

1.4.2 Ongoing Clinical Studies 60

1.4.2.1 Safety of Atezolizumab 61

1.4.2.2 Efficacy of Atezolizumab 62

1.4.2.3 Clinical Pharmacokinetics and Immunogenicity of Atezolizumab 63

1.5 Background on Tocilizumab (RO4877533, Actemra, RoActemra) 64

1.6 Study Rationale and Benefit–Risk Assessment 65

1.6.1 Combination of RO6958688 and Atezolizumab 65

1.6.2 Potential for Overlapping Toxicities with RO6958688 and Atezolizumab 66

1.6.3 Rationale for [18F] FDG-PET Imaging 66

1.6.4 Rationale for the New Dose Schedule 66

2. OBJECTIVES 67

2.1 Primary Objectives 67

2.2 Secondary Objectives 68

2.3 Exploratory Objectives 68

“Use of this document is governed by the terms of use on the first page of this document.”

3. STUDY DESIGN 69
3.1 Description of Study 69
3.1.1 Overview of Study Design 70
3.1.2 Dose Escalation Decision Criteria 76
3.1.2.1 Escalation Criteria (Part I) – Dose Limiting Toxicities 77
3.1.2.2 Expansion Part (Part II) 78
3.1.3 Communication Strategy 78
3.1.4 End of Study 80
3.2 Rationale for Study Design 80
3.2.1 Rationale for Dosage Selection 80
3.2.1.1 Starting Dose for RO6958688 80
3.2.1.2 Dose of Atezolizumab 82
3.2.2 Rationale for the Treatment of Severe Cytokine Release Syndrome (CRS) Using Tocilizumab 83
3.2.3 Rationale for Study Population 84
3.2.4 Rationale for Biomarker Assessments 85
3.2.5 Rationale for Statistical Design 87
3.3 Outcome Measures 88
3.3.1 Safety Outcome Measures 88
3.3.2 Pharmacokinetic (PK) and Pharmacodynamic (PD) Outcome Measures 90
3.3.2.1 Pharmacokinetic Outcome Measures 90
3.3.2.2 Pharmacodynamic Outcome Measures 90
3.3.3 Efficacy Outcome Measures 92
3.3.4 Exploratory Outcome Measures 94
4. MATERIALS AND METHODS 95
4.1 Center 95
4.2 Study Population 95
4.2.1 Recruitment Procedures 96
4.2.2 Inclusion Criteria 96
4.2.3 Exclusion Criteria 98
4.3 Study Treatments 101
4.3.1 Formulation, Packaging, and Handling 101

"Use of this document is governed by the terms of use on the first page of this document."

4.3.1.1 RO6958688 101
4.3.1.2 Atezolizumab 103
4.3.1.3 Tocilizumab 103
4.3.2 Dosage, Administration, and Compliance 104
4.3.2.1 RO6958688 and Atezolizumab 104
4.3.2.2 Administration of Atezolizumab 104
4.3.2.3 Administration of RO6958688 104
4.3.2.4 Administration of Tocilizumab 105
4.3.2.5 Premedication and Prophylactic Treatment for RO6958688 and Atezolizumab 106
4.3.3 Investigational Medicinal Products Accountability 107
4.3.4 Post-Trial Access to RO6958688 and Atezolizumab 108
4.4 Concomitant Therapy 108
4.4.1 Permitted Therapy 108
4.4.2 Prohibited Therapy 110
4.5 Study Assessments 111
4.5.1 Description of Study Assessments 111
4.5.1.1 Medical History and Demographic Data 111
4.5.1.2 Physical Examinations, Vital Signs, and ECOG Performance Status 111
4.5.1.3 Electrocardiograms 112
4.5.1.4 Pulmonary Function Tests (FEV1/VC/TLC and DLCO) 113
4.5.1.5 Laboratory Assessments 113
4.5.1.6 Additional Samples 114
4.5.1.7 Disease-Specific Assessments 119
4.5.1.8 Samples for Roche Clinical Repository 122
4.5.2 Timing of Study Assessments 125
4.5.2.1 Screening and Pretreatment Assessments 125
4.5.2.2 Assessments during Treatment 126
4.5.2.3 Assessments at Study Completion/Early Termination Visit 127
4.5.2.4 Follow-Up Assessments 128

"Use of this document is governed by the terms of use on the first page of this document."

4.5.2.5 Assessments at Unscheduled Visits 129
4.6 Patient, Study, and Site Discontinuation 129
4.6.1 Patient Discontinuation 129
4.6.1.1 Discontinuation from Study Drugs 129
4.6.1.2 Withdrawal from Study 133
4.6.2 Study and Site Discontinuation 133

5. ASSESSMENT OF SAFETY 133
5.1 Safety Parameters and Definitions 133
5.1.1 Adverse Events 134
5.1.2 Serious Adverse Events (Immediately Reportable to the Sponsor) 134
5.1.3 Non-Serious Adverse Events of Special Interest (Immediately Reportable to the Sponsor) 135
5.2 Safety Plan 136
5.2.1 Dose Modifications and Delays 136
5.2.2 Risks Associated with Atezolizumab 139
5.2.3 Risks Associated with RO6958688 139
5.2.4 Risks Associated with Combination Use of RO6958688 and Atezolizumab 140
5.2.5 Risks Associated with Tocilizumab 140
5.2.6 Management of Specific Adverse Events 140
5.2.6.1 Management of Atezolizumab-Specific Adverse Events 140
5.2.6.2 Management of Specific Adverse Events to RO6958688 141
5.2.6.3 Management of Adverse Events Specific to Atezolizumab and to RO6958688 145
5.3 Methods and Timing for Capturing and Assessing Safety Parameters 146
5.3.1 Adverse Event Reporting Period 146
5.3.2 Eliciting Adverse Event Information 147
5.3.3 Assessment of Severity of Adverse Events 147
5.3.4 Assessment of Causality of Adverse Events 148
5.3.5 Procedures for Recording Adverse Events 148

“Use of this document is governed by the terms of use on the first page of this document.”

5.3.5.1 Infusion-Related Reactions/Hypersensitivity Reactions and Cytokine Release Syndrome Attributed to RO6958688 148
5.3.5.2 Other Adverse Events 149
5.3.5.3 Adverse Events Occurring Secondary to Other Events 149
5.3.5.4 Persistent or Recurrent Adverse Events 150
5.3.5.5 Abnormal Laboratory Values 150
5.3.5.6 Abnormal Vital Sign Values 151
5.3.5.7 Abnormal Liver Function Tests 151
5.3.5.8 Deaths 151
5.3.5.9 Preexisting Medical Conditions 152
5.3.5.10 Lack of Efficacy or Worsening of Cancer 152
5.3.5.11 Hospitalization or Prolonged Hospitalization 152
5.3.5.12 Cases of Accidental Overdose or Medication Error 153
5.4 Immediate Reporting Requirements from Investigator to Sponsor 154
5.4.1 Emergency Medical Contacts 154
5.4.2 Reporting Requirements for Serious Adverse Events and Non-Serious Adverse Events of Special Interest 155
5.4.3 Reporting Requirements for Pregnancies 155
5.4.3.1 Pregnancies in Female Patients 155
5.4.3.2 Pregnancies in Female Partners of Male Patient 155
5.4.3.3 Abortions 155
5.4.3.4 Congenital Anomalies/Birth Defects 156
5.5 Follow-Up of Patients after Adverse Events 156
5.5.1 Investigator Follow-Up 156
5.5.2 Sponsor Follow-Up 157
5.6 Post-Study Adverse Events 157
5.7 Expedited Reporting to Health Authorities, Investigators, Institutional Review Boards, and Ethics Committees 157

6. STATISTICAL CONSIDERATIONS AND ANALYSIS PLAN 158

“Use of this document is governed by the terms of use on the first page of this document.”

6.1 Primary Study Variables 158
6.2 Secondary Study Variables 158
6.3 Determination of Sample Size 159
6.3.1 Dose-Escalation 159
6.3.2 Dose/Schedule-Finding 160
6.4 Summaries of Conduct of Study 161
6.5 Analysis Populations 161
6.5.1 Safety Analysis Population 161
6.5.2 Pharmacokinetic Analysis Population 161
6.5.3 Pharmacodynamic Analysis Population 161
6.5.4 Efficacy Analysis Population 162
6.6 Summaries of Treatment Group Comparability 162
6.7 Safety Analyses 162
6.7.1 Dose-Escalation Approach 162
6.7.1.1 Modified Continual Reassessment Method with Overdose Control in Part IA 162
6.7.1.2 Intra-Patient Dose Escalation Design 166
6.7.2 Adverse Events 168
6.7.3 Clinical Laboratory Test Results 168
6.7.3.1 Standard Reference Ranges and Transformation of Data 168
6.7.3.2 Definition of Laboratory Abnormalities 168
6.7.4 Vital Signs 169
6.7.5 ECG Data Analysis 169
6.7.6 Concomitant Medications 169
6.8 Efficacy Analyses 169
6.8.1 Primary Efficacy Endpoint 170
6.8.2 Secondary Efficacy Endpoints 170
6.9 Pharmacodynamic Analyses 170
6.10 Pharmacokinetic Analyses 171
6.11 Immunogenicity Analyses 171
6.12 Interim Analyses 172

7. DATA COLLECTION AND MANAGEMENT 172

“Use of this document is governed by the terms of use on the first page of this document.”

7.1 Data Quality Assurance 172
7.2 Electronic Case Report Forms 172
7.3 Source Data Documentation 173
7.4 Use of Computerized Systems 173
7.5 Retention of Records 174

8. ETHICAL CONSIDERATIONS 174
8.1 Compliance with Laws and Regulations 174
8.2 Informed Consent 174
8.3 Institutional Review Board or Ethics Committee 175
8.4 Confidentiality 176
8.5 Financial Disclosure 176

9. STUDY DOCUMENTATION, MONITORING, AND ADMINISTRATION 176
9.1 Study Documentation 176
9.2 Site Inspections 177
9.3 Administrative Structure 177
9.4 Publication of Data and Protection of Trade Secrets 177
9.5 Protocol Amendments 178

10. REFERENCES 179

LIST OF TABLES

Table 1 Overall Safety Profile of RO6958688 (Ongoing Study BP29541) 81
Table 2 Pre-Medications to be Administered before RO6958688 Infusion 106
Table 3 Cytokine Release Syndrome Grading According to CTCAE v5 143
Table 4 Adverse Event Severity Grading Scale 147
Table 5 Operating Characteristics of the mCRM with EWOC Design with Respect to the Chosen Scenarios 160
Table 6 Hypothetical Recommendations of the mCRM with EWOC Design 166

“Use of this document is governed by the terms of use on the first page of this document.”

LIST OF FIGURES

Figure 1	Design, Structure, and Characteristics of RO6958688	46
Figure 2	RO6958688-Mediated Lysis of MKN45 Cells	47
Figure 3	RO6958688 Upregulation of PD-1 on T cells, Respective of PD-L1 on Surviving Tumor Cells after Tumor Cell Lysis	48
Figure 4	In Vivo Anti-Tumor Activity upon Combination of RO6958688 with the Anti-Human PD-L1 Blocking Antibody in MKN45 Tumor Model in Fully Humanized Mice	50
Figure 5	Study Schema	71
Figure 6	Conditions for Continuing RO6958688 and/or Atezolizumab in the Presence of Increased Radiographic Tumor Size	132

LIST OF APPENDICES

Appendix 1	Schedule of Assessments	185
Appendix 2	CKD-EPI equation for Calculation of Glomerular Filtration Rate (GFR)	227
Appendix 3	Eastern Cooperative Oncology Group Performance Status	228
Appendix 4	Response Evaluation Criteria in Solid Tumors Version 1.1 Criteria	229
Appendix 5	Modified Response Evaluation Criteria in Solid Tumors	234
Appendix 6	Statistical Design of Modified Continual Reassessment Method with Escalation with Overdose Control	241
Appendix 7	Intrapatient Dose Escalation Design Simulation Results	253
Appendix 8	Gilbert's Syndrome Definition	256
Appendix 9	[18F]-FDG-PET	257
Appendix 10	Overall Guidelines for Management of Patients Who Experience Adverse Events	258
Appendix 11	Risks Associated with Atezolizumab and Guidelines for Management of Adverse Events Associated with Atezolizumab	273
Appendix 12	Schedule of Assessments for Tocilizumab Treatment of Severe or Life-Threatening Cytokine Release Syndrome	298
Appendix 13	Vasopressor Use for CRS	300
Appendix 14	Anaphylaxis Precautions	301

"Use of this document is governed by the terms of use on the first page of this document."

PROTOCOL AMENDMENT ACCEPTANCE FORM

**TITLE:**	**AN OPEN-LABEL, MULTICENTER, DOSE ESCALATION AND EXPANSION PHASE Ib STUDY TO EVALUATE THE SAFETY, PHARMACOKINETICS, AND THERAPEUTIC ACTIVITY OF RO6958688 IN COMBINATION WITH ATEZOLIZUMAB IN PATIENTS WITH LOCALLY ADVANCED AND/OR METASTATIC CEA-POSITIVE SOLID TUMORS**
**PROTOCOL NUMBER:**	WP29945
**VERSION NUMBER:**	11
**EUDRACT NUMBER:**	2015-003771-30
**IND NUMBER:**	122931
**NCT NUMBER:**	NCT02650713
**TEST PRODUCTS:**	RO6958688 and Atezolizumab
**MEDICAL MONITOR:**	[redacted]
**SPONSOR:**	F. Hoffmann-La Roche Ltd

I agree to conduct the study in accordance with the current protocol.

Principal Investigator's Name (print)

Principal Investigator's Signature

Date

Please keep the signed original form in your study files, and return a copy to your local study monitor.

"Use of this document is governed by the terms of use on the first page of this document."

PROTOCOL SYNOPSIS

**TITLE:**	**AN OPEN-LABEL, MULTICENTER, DOSE ESCALATION AND EXPANSION PHASE Ib STUDY TO EVALUATE THE SAFETY, PHARMACOKINETICS, AND THERAPEUTIC ACTIVITY OF RO6958688 IN COMBINATION WITH ATEZOLIZUMAB IN PATIENTS WITH LOCALLY ADVANCED AND/OR METASTATIC CEA-POSITIVE SOLID TUMORS**
**PROTOCOL NUMBER:**	WP29945
**VERSION NUMBER:**	11
**EUDRACT NUMBER:**	2015-003771-30
**IND NUMBER:**	122931
**NCT NUMBER:**	NCT02650713
**TEST PRODUCTS:**	RO6958688 and Atezolizumab
**PHASE:**	Ib
**INDICATION:**	Patients with locally advanced and/or metastatic CEA-positive solid tumors, whose disease has progressed on or who are intolerant to the standard therapy
**SPONSOR:**	F. Hoffmann-La Roche Ltd

“Use of this document is governed by the terms of use on the first page of this document.”

OBJECTIVES

Primary Objectives

The primary objectives of this study are:

- To establish the preliminary safety and tolerability profile of RO6958688 in combination with atezolizumab
- To determine the maximum tolerated dose (MTD) in cycle 1 and in later cycles, if achieved, of RO6958688 in combination with atezolizumab
- To identify a recommended phase II dose and schedule (RP2D) of RO6958688 in combination with atezolizumab

Secondary Objectives

The secondary objectives for this study are:

- To describe the preliminary pharmacodynamic (PD) effects and duration of PD response for RO6958688 in combination with atezolizumab in mandatory paired tumor biopsies and paired blood samples on the basis of alterations in the quantity and quality of intratumoral T cells and peripheral blood cells (including but not limited to $CD3^+$, $CD4^+$, $CD8^+$ T cells, and other immune cells that might act as potential predictors of anti-tumor activity of RO6958688 in combination with atezolizumab)
- To describe the pharmacokinetics (PK) of RO6958688 and atezolizumab when administered in combination
- To obtain preliminary anti-tumor activity data of RO6958688 in combination with atezolizumab based on objective overall response rate (ORR), duration of response (DOR) and derived measures, disease control rate (DCR; defined as response rate [RR] + stable disease rate [SDR]), preliminary progression-free survival (PFS) and preliminary overall survival (OS) according to Response Evaluation Criteria in Solid Tumors (RECIST), Version 1.1 criteria and modified RECIST criteria, by investigator assessment for the whole study and by central assessment for prospective and retrospective analysis
- To estimate the PFS rate at relevant timepoints for RO6958688 in combination with atezolizumab

Exploratory Objectives

The exploratory objectives for this study are:

- To explore the relationship between exposure, pharmacodynamics, metabolic activity of the tumor and clinical effects of RO6958688 when administered in combination with atezolizumab
- To explore the immunogenicity of RO6958688 when administered in combination with atezolizumab
- To explore the relationship of host and tumor genetic factors with PD or clinical response to therapy
- To investigate and define CEA expression in different solid tumors
- To investigate tumor mutations, gene expression and other biomarkers (such as CEA expression in various tumor indications) related to RO6958688 + atezolizumab combination therapy
- To characterize the natural growth of the tumor using tumor growth kinetics modeling
- To explore preliminary safety and efficacy in low/moderate and very low CEA expressing tumors
- To make a preliminary assessment of the effectiveness, PK and PD effects of tocilizumab (Actemra®/RoActemra®) in ameliorating the symptoms of severe CRS following RO6958688 treatment

"Use of this document is governed by the terms of use on the first page of this document."

STUDY DESIGN

Note: Following an internal review of the clinical development plan of RO6958688, the Sponsor has decided to permanently discontinue further enrollment of patients in this study and to not open the planned cohorts B2, C3, G1, G2, and G3 and the Biomarker cohort as well as Part II of the study.

Description of Study

This is an open-label, multi-center, dose escalation and expansion Phase Ib clinical study of RO6958688 in combination with atezolizumab. Each treatment cycle will be 21 days in duration and consists of IV infusions of RO6958688 given weekly (QW) (± 1 day) or every 3 weeks (Q3W) (± 2 days) in combination with atezolizumab given every 3 weeks (Q3W) (± 2 days).

The initial dose of atezolizumab will be delivered over 60 (± 15) minutes. If the first infusion is tolerated without infusion-related reaction (IRR), the second infusion may be delivered over 30 (± 10) minutes. If the 30-minute infusion is well tolerated, all subsequent infusions may be delivered over 30 (± 10) minutes. RO6958688 should be administered at C1D1 by IV infusion over a minimum of 2 hours, subsequent infusions should be administered in at least 4 hours during the dose/escalation phase. In patients with no grade ≥ 2 IRR/CRS for more than two RO6958688 administrations, the infusion time can be progressively reduced to a minimum of 1 hour. For more details, please refer to the RO6958688 pharmacy manual. At Day 1 of each cycle when atezolizumab and RO6958688 are administered, atezolizumab will be first administered, then RO6958688 will be administered:

- at least half an hour after the end of atezolizumab infusion if no premedication is given prior to RO6958688 administration.
- at least one hour after the end of atezolizumab infusion if premedication is given prior to RO6958688 administration.

During the trial if the clinical pharmacology and/or safety data support an alternate dose sequencing, the above could be modified accordingly.

Patients will be treated until loss of clinical benefit, unacceptable toxicities, loss of RO6958688 exposure (in which case they can continue to receive atezolizumab alone), or withdrawal of consent. The treatment period for this protocol is 24 months for both RO6958688 and atezolizumab and may be modified if emerging data supports an alternative duration of therapy. In case one of the treatments is permanently discontinued, treatment with the other drug alone may be continued as long as the patient experiences clinical benefit in the opinion of the investigator or until unacceptable toxicity or symptomatic deterioration develops, which is attributed to disease progression as determined by the investigator and the Sponsor after an integrated assessment of radiographic data, biopsy results (if available) and clinical status, or withdrawal of consent.

The study will be conducted in two parts. Part I is subdivided into parts IA and IB. Part IA, is a dose escalation part with a starting dose of 5 mg of RO6958688 given QW (once a week) and a fixed, flat dose of 1200 mg given Q3W (every 3 weeks) of atezolizumab, to evaluate the safety and determine the MTD of RO6958688 in combination with atezolizumab. Part IB is a dose/schedule finding part that will explore different administration schedules of RO6958688 in combination with atezolizumab (1200 mg Q3W). Part II is an expansion part to confirm the safety and tolerability of the recommended dose and selected schedule as determined in Part I, in order to define an RP2D and schedule of RO6958688 in combination with atezolizumab, and to explore preliminary antitumor activity, pharmacokinetic and pharmacodynamic effects. Note: Part II has not and will not enroll any patients.

“Use of this document is governed by the terms of use on the first page of this document.”

Part IA: Dose Escalation Part

Patients will receive atezolizumab (1200 mg fixed dose) IV on Day 1 of each cycle, followed by RO6958688 given IV on Day 1, Day 8 and Day 15 of each cycle.

Dose escalation of RO6958688 will be pursued according to a modified-Continual Reassessment Method with Overdose Control (mCRM with EWOC) design, aimed at reaching the MTD, which is defined as a dose with 20-30% probability of dose-limiting toxicity (DLT). For RO6958688, the starting dose is 5 mg administered on a QW schedule, to be administered after the administration of 1200 mg of atezolizumab when they are both administered on the same day (Day 1 of each cycle). The RO6958688 dose will be escalated according to the mCRM, but will not exceed the RO6958688 MTD if defined in the BP29541 study. The atezolizumab dose is fixed at 1200 mg and is administered on a Q3W schedule.

Patients within a cohort (at least 3 patients each) will be enrolled in a sequential manner, which, if required, can be expanded with additional patients to acquire additional safety, PK and PD data. Each patient will be observed for 21 days for DLT assessment. Enrollment will be staggered so that the first patient in each cohort will be observed for safety for 1 week (2 weeks in Spain) before additional patients are enrolled in the cohort. Once a minimum of 3 patients have completed the 21-day DLT observation period, the Sponsor and investigators will evaluate and agree on the dose, sequence, and timing for administration of both drugs for the subsequent cohort.

In Part IA (dose escalation), in order to overcome ADA and their impact on PK, optional intra-patient dose escalation of RO6958688 to the next available tolerated dose level may be permitted depending on emerging clinical and safety data at the discretion of the treating physician, and after discussion with the patient. Intra-patient dose escalation may only proceed after patients have tolerated at least the first 3 consecutive doses of RO6958688 after discussion and alignment with the Medical Monitor.

Part IB: Dose/schedule finding part

The first cohort (cohort A) in Part IB will compare the QW vs. Q3W schedules at a flat dose of 100 mg RO6958688 in combination with atezolizumab 1200 mg Q3W. This QW vs. Q3W schedule comparison will enroll approximately 20 to 40 randomized patients per arm.

Part IB will also explore RO6958688 step up dosing schedules in combination with atezolizumab 1200 mg Q3W.

For the step up dosing schedule, the late cycle MTD will be estimated by an intra-patient dose escalation design (see Protocol Section 6.7.1.2) in cohort B1. In this cohort, the RO6958688 dose will be escalated up to 300% of the previous dose until the DLT criteria for that dose level are met. The dose of RO6958688 will not be escalated in the intra patient dose escalation above the monotherapy late cycle MTD of RO6958688 if defined in the BP29541 protocol.

Cohort B1 has enrolled 15 MSS CRC and 2 MSI-H CRC patients. One of the objectives of cohort B1 is to generate initial safety and efficacy data to compare the effect of treatment combination between MSS and MSI-H CRC patients. The second objective of cohort B1 is to define the late cycle MTD for RO6958688 in combination with atezolizumab. Note: No further patients will be enrolled in cohort B1.

The RO6958688 dose at C1D1 will be 40 mg followed by 150 mg at C1D8, 300 mg at C1D15, 600 mg at C2D1, 900 mg at C2D8 and 1200 mg at C2D15 and 1200mg at C3D1 then 1200 mg every 3 weeks (Q3W) thereafter (i.e., C4D1, C5D1…). The Q3W RO6958688 recommended dose will not exceed the late cycle MTD if defined or 1200 mg.

"Use of this document is governed by the terms of use on the first page of this document."

Cohort B2 will enroll approximately 20 MSS CRC evaluable patients. This new cohort is intended to explore a faster RO6958688 escalation in combination with atezolizumab (Q3W) in order to reach the target dose within the first atezolizumab cycle and prevent a potential early impact of anti-drug antibodies (ADA) on RO6958688 exposure within this first cycle and asses if a more rapid escalation may also increase anti-tumor activity of this combination. Enrollment in Cohort B2 can start at any time after the safety observation period of the 3rd patient in cohort B1 has been completed. Note: No patients have been enrolled into Cohort B2, and this cohort will not be opened for enrollment.

Cohort B1 will be used to define the late cycle MTD or if not defined, to assess 1200 mg as a safe dose.

The RO6958688 dose at C1D1 will be 40 mg followed by 150 mg at C1D8, 600 mg at C1D15, 1200 mg at C2D1 then 1200 mg every 3 weeks (Q3W) thereafter (i.e., C3D1, C4D1…). The Q3W RO6958688 recommended dose will not exceed the late cycle MTD if defined or 1200 mg.

Part IB of the study will also explore two additional RO6958688 step-up dose regimens (cohort C1 and C2) in combination with atezolizumab 1200 mg Q3W, in a randomized schedule comparison expansion. These randomized cohorts will start in parallel to the step up cohort B1.

Enrollment in cohorts B1, C1, and C2 will be staggered, the first patient in each cohort will be observed for safety for 1 week (2 weeks in Spain) before the second patient in each cohort is enrolled. A safety observation period of 1 week will be observed between the second and the third patient in each cohort, and from the third to subsequent patients. In the current study, we have not observed so far significant differences regarding the safety profile in patients with MSI-H tumors when compared to patients with MSS tumors; however since only 4 MSI-H patients have been treated; in cohort B1 the same safety observation period will be applied to the first 3 MSI-H patients.

In the randomized cohorts, RO6958688 will be administered as follows:

Cohort C1: The RO6958688 starting dose will be 40 mg, followed by 100 mg in C1D8, 150 mg in C1D15, 150 mg at C2D1 and 150 mg RO6958688 Q3W thereafter (i.e., C3D1, C4D1…).

Cohort C2: The RO6958688 starting dose will be 40 mg, followed by 150 mg in C1D8, 300 mg in C1D15, 600 mg at C2D1 and 600 mg (or late cycle MTD if defined) RO6958688 Q3W thereafter.

Based on the clinical data from the randomized cohorts (C1 and C2) the sponsor may consider opening a third cohort (cohort C3) with a starting dose of RO6958688 at 100 mg with mandatory prophylactic corticosteroids post dose at C1D1 (as detailed in Table 3), followed by 150 mg in C1D8, 300 mg in C1D15, and 600 mg at C2D1 and 600 mg (or late cycle MTD if defined) RO6958688 Q3W thereafter (i.e., C3D1, C4D1…). Note: No patients have been enrolled into Cohort C3, and this cohort will not be opened for enrollment.

The Q3W RO6958688 recommended dose for cohorts C1, C2 and the optional C3 cohort defined above, will not exceed the late cycle MTD if defined.

Each of the cohorts C1-2 and the optional cohort C3 will consist of approximately 40 locally advanced or metastatic (as defined by eligibility criteria #3) microsatellite stable (MSS) colorectal cancer patients. Approximately 80 patients will be randomized 1:1 to cohorts C1-2. Note: 39 CRC

“Use of this document is governed by the terms of use on the first page of this document.”

patients were enrolled in cohort C1, and 35 CRC patients were enrolled in cohort C2. No further patients will be enrolled into these cohorts, and no patients have or will be enrolled in cohort C3.

Additional safety cohorts in other solid tumor, including NSCLC, gastric, pancreatic and breast cancer will be explored (without formal estimation of maximum tolerated doses). These cohorts can be opened after the safety observation period (1 week) of the third patient in cohorts B1 or C has been completed. Note: 14 patients were enrolled in the pancreatic cohort, 6 patients were enrolled in the gastric cohort and 2 patients were enrolled in the breast cohort. No patients were enrolled in the NSCLC cohort. No further patients will be enrolled in the safety cohorts.

These safety cohorts will explore RO6958688 administered weekly (QW) at an initial dose of 40 mg followed by 150 mg in C1D8, 300 mg in C1D15, 600 mg at C2D1 and 600 mg (or MTD if defined) Q3W thereafter. Atezolizumab will be administered Q3W (1200 mg). Regarding lung cancer patients an alternative RO6958688 dose/schedule can be explored if supported by data (i.e., 40 mg at C1D1 followed by 100 mg at C1D8, 150 mg at C1D15, 300 mg at C2D1 and 600 mg every 3 weeks).

Finally, once the safety observation period of the third patient in cohort B1 has been completed, the sponsor may explore the safety and preliminary efficacy of RO6958688 in combination with atezolizumab in a separate biomarker cohort of approximately 20 patients with very low/negative CEA expressing solid tumors. Very low/negative CEA expression is defined as those samples having $<$ 20% of tumor cells with IHC1+or IHC0+. Patients enrolled in the biomarker cohort (very low/negative CEA expression) will follow the same dose/schedule as patients in cohort C1. Note: No patients have been enrolled into the biomarker cohort, and this cohort will not be opened for enrollment.

Once the late cycle MTD has been defined (see Protocol Section 6.7.1.2) or 1200 mg is declared safe in cohort B1, ongoing patients in the trial experiencing clinical benefit and showing >50% reduction of RO6958688 C_{max} and a PK profile consistent with ADA mediated decrease in exposure, can be progressively dose escalated up to the late cycle MTD or 1200 mg of RO6958688 after discussion with the Medical Monitor. However, the above optional dose escalation does not apply to patients enrolled in cohort C1 since this cohort has been designed to assess a potential biological dose.

The sponsor may consider opening additional obinutuzumab cohorts (G1 to G3). Patients participating in these cohorts will receive according to patient's and/or investigators convenience, either 2000 mg of obinutuzumab IV on Day-7 (+ 1 day) or 1000 mg of obinutuzumab IV on two consecutive days, Day-8 and Day-7 (+ 1 day) before C1D1 RO6958688 and atezolizumab administrations. Premedication will be given prior to each obinutuzumab dosing. For these patients, the baseline tumor biopsy will be taken before receiving the first dose of obinutuzumab and the on-treatment tumor biopsy remains unchanged. Note: No patients have been enrolled into the obinutuzumab cohorts, and these cohorts will not be opened for enrollment.

Cohort G1 (MSS CRC): approximately 40 patients with locally advanced or metastatic (as defined by eligibility criteria #3) microsatellite stable (MSS) colorectal cancer patients will be enrolled. The RO6958688 starting dose will be 40 mg, followed by 100 mg on C1D8, 150 mg on C1D15 and 150 mg RO6958688 Q3W thereafter (i.e., C3D1, C4D1…) in combination with 1200 mg atezolizumab Q3W.

"Use of this document is governed by the terms of use on the first page of this document."

Based on preliminary efficacy and safety data from Cohort G1 the sponsor may consider opening additional Cohorts for patients with gastric, pancreatic and other indications:

Cohort G2: approximately 40 patients with locally advanced or metastatic Gastric Cancer (as defined by eligibility criteria #3) will be enrolled.

The RO6958688 starting dose will be 40 mg, followed by 100 mg on C1D8, 150 mg on C1D15 and 150 mg RO6958688 Q3W thereafter (i.e., C3D1, C4D1…) in combination with 1200 mg atezolizumab Q3W.

Cohort G3: approximately 40 patents with locally advanced or metastatic Pancreatic Cancer (as defined by eligibility criteria #3) will be enrolled. The RO6958688 starting dose will be 40 mg, followed by 100 mg on C1D8, 150 mg on C1D15 and 150 mg RO6958688 Q3W thereafter (i.e., C3D1, C4D1…) in combination with 1200 mg atezolizumab Q3W.

Enrollment in Cohorts G1-G3 will be independently staggered, the first patient will be observed for safety for 1 week (2 weeks in Spain) before the second patient in each cohort is enrolled. A safety observation period of 1 week will be observed between the second and the third patient in each cohort, and from the third to subsequent patients.

For all patients who enroll in the study, tumor biopsies for PD analysis are mandatory at baseline and on-treatment, except for NSCLC patients for whom there is no accessible lesion. For patients continuing in the study additional biopsies may be taken at the discretion of the investigator. These biopsies may serve to evaluate viable tumor given the mechanism of action of RO6958688 to induce tumor inflammation. For patients discontinuing from the study due to disease progression, additional optional biopsies if clinically feasible, may be taken to aid the understanding of immune resistance mechanisms.

Part II: Expansion Part

Once the recommended dose and schedule have been determined, additional patients (approximately 15 patients) with solid tumors with high CEA expression may be enrolled in this study. The purpose is to confirm the safety and tolerability of the recommended dose and schedule as determined in Part I and to explore preliminary antitumor activity, pharmacokinetic and pharmacodynamic effects. Note: No patients have been enrolled in Part II of the study, and Part II will not be opened for enrollment.

Baseline and on-treatment tumor biopsies will be mandatory for all patients, except for NSCLC patients for whom there is no accessible lesion. DLT data will still be collected and might lead to refinement of the MTD definition for RO6958688 in combination with atezolizumab at the end of the trial.

Patients who discontinue both study drugs prior to the first on-treatment tumor assessment due to toxicity will not be considered evaluable for response and will be replaced. Patients who discontinue treatment due to clinical progression will remain evaluable.

Dose Limiting Toxicities (DLTs)

At least 3 patients will be enrolled in each cohort; additional patients may be enrolled to study safety, PK, or PD in more detail. Patients in a cohort will be enrolled in a sequential manner. The first patient in each cohort will be observed for safety for 1 week (2 weeks in Spain) prior to enrollment of additional patients in that cohort. During the dose escalation part (Part IA), patients who discontinue treatment before the end of the DLT period, for reasons other than DLTs, and patients who did not receive the assigned dose of RO6958688 (3 doses QW for the QW regimen)

“Use of this document is governed by the terms of use on the first page of this document.”

or atezolizumab (1 dose Q3W) during the DLT period, will be replaced to ensure that at least 3 patients in each cohort have been assessed for the full DLT period of 21 days prior to moving to the next dose level. During the step up dose escalation (Part IB), patients who discontinue treatment for reasons other than DLTs or receiving the 1200 mg dose or the highest possible safe dose according to the intra-patient dose escalation rules (the lower one applies), may be replaced in order to ensure that at least 6 evaluable patients will be available for estimating the late cycle MTD.

A DLT is defined as one of the following toxicities that occur during the DLT assessment period and is considered by the investigator to be related to RO6958688, atezolizumab, or the combination of both products. For potential overlapping toxicities, investigators are encouraged to perform additional tests to determine the underlying etiology and most appropriate attribution. Dose reductions or delays will not, in themselves, constitute DLTs.

The following adverse events are considered DLTs:

Hematological toxicities defined as:

- Grade ≥ 4 neutropenia (ANC < 500/μL) lasting ≥ 7 days
- Grade ≥ 3 febrile neutropenia
- Grade ≥ 4 thrombocytopenia lasting > 48 hours (recovery to ≤ Grade 2)
- Grade 3 or 4 thrombocytopenia associated with bleeding episodes

Any non-hematological toxicity ≥ Grade 3 including:

- Grade 3 hyperbilirubinemia lasting for > 48 hours or Grade 4
- Grade ≥ 3 AST/ALT elevations with hyperbilirubinemia of ≥ Grade 2
- Grade 4 AST/ALT elevations
- For patients with Grade 2 AST, ALT, and/or alkaline phosphatase abnormality at baseline, an increase to ≥ 10 × the upper limit of normal (ULN) that does not resolve to Grade ≤ 2 within 48 hours (if symptomatic) or that does not resolve to Grade ≤ 1 within 3 weeks of onset (if asymptomatic)

Failure to recover from any drug-related toxicity that results in a dose delay of ≥ 21 days (1 Cycle) is defined as a DLT.

The following are not considered DLTs:

- Grade 3 nausea, vomiting, diarrhea, colitis or enteritis that resolves to Grade ≤ 2 with or without treatment prior to the next planned infusion of RO6958688 (1 week)
- Grade 3 *immune-mediated* adverse event that resolves to Grade ≤ 1 with immunosuppressant therapy within 3 weeks of its onset
- Grade ≥ 3 fatigue that resolves to Grade ≤ 2 within 1 week
- Grade 3 arthralgia that can be adequately managed with supportive care or that resolves to Grade ≤ 2 within 1 week
- Fever > 40 degrees Celsius that occurs within 72 hours of RO6958688 infusion and resolves to ≤ Grade 2 within 4 days and is resolved to Grade ≤ 1 within 10 days

"Use of this document is governed by the terms of use on the first page of this document."

- Fever ≥ Grade 3 that resolves to Grade ≤ 2 within 72 hours
- Grade 3 hypophosphatemia reversible to < Grade 2 within 1 week
- Grade ≥ 3 laboratory abnormality that is asymptomatic and deemed by the investigator not to be clinically significant
- Grade 3 autoimmune thyroiditis or other endocrine abnormality that can be managed by endocrine therapy or hormonal replacement
- Grade 3 tumor flare defined as local pain, irritation, or rash localized at sites of known or suspected tumor
- Alopecia (any grade)
- Grade 3 tumor pain that starts within 24 hours of infusion and resolves to Grade ≤ 2 within 1 week
- Grade 3 hypoxia that starts within 24 hours of infusion and resolves to Grade ≤ 2 within 1 week
- In patients with lung lesions, Grade 3 transient dyspnea secondary to localized lung edema that starts within 24 hours of infusion and recovers to Grade ≤ 2 or baseline within 1 week, and transient bronchospasm that resolves within 24 hours
- In patients with liver lesions, Grade 3 transient increase of bilirubin, transaminases and/ or Gamma GT that starts after infusion and recovers to Grade ≤ 2 or baseline within 1 week and grade 4 AST/ALT or grade 4 bilirubin increase that start after infusion and recovers to grade ≤2 or baseline within 3 days.

Infusion related reactions (IRRs): IRRs are not considered to be DLTs because based on experience with monoclonal antibodies, IRRs are not dose-related events. Precautions will be taken if IRRs Grade ≥ 2 occur. If described precautions are not sufficient, other options will be discussed between the Sponsor and investigators.

NUMBER OF PATIENTS

Overall, this study has enrolled 228 patients in the dose escalation and dose/schedule finding part. No further enrollment will take place.

TARGET POPULATION

Part I and Part II of the trial will enroll patients with locally advanced and/or metastatic CEA–positive solid tumors who have progressed on standard treatment, are intolerant to standard treatment, and/or are non-amenable to standard therapy.

Based on the preliminary safety and efficacy data from Part I, the Sponsor could focus on other specific CEA expressing tumor indications for which to enroll a certain number of patients in Part II.

“Use of this document is governed by the terms of use on the first page of this document.”

INCLUSION/EXCLUSION CRITERIA

Inclusion criteria:

Patients must meet the following criteria for study entry:

1. Signed informed consent
2. Age ≥ 18 years
3. Confirmed locally advanced and/or metastatic solid tumor, with at least one tumor lesion of accessible non-critical location to biopsy, in patients who have progressed on a standard therapy, are intolerant to standard therapy, and/or are non-amenable to standard therapy
4. Radiologically measurable and clinically evaluable disease (as per RECIST v1.1 - previously irradiated lesions should not be counted as target lesions)
5. Life expectancy (in the opinion of the investigator) of ≥ 12 weeks and LDH levels ≤ 2.5 ULN
6. Eastern Cooperative Oncology Group (ECOG) Performance Status (PS) 0–1
7. All acute toxic effects of any prior radiotherapy, chemotherapy, or surgical procedure must have resolved to Grade ≤ 1 or returned to baseline except alopecia (any grade) and Grade 2 peripheral neuropathy
8. Adequate hematological function (without transfusion within 2 weeks prior to Cycle 1, Day 1): neutrophil count of ≥ 1.5 × 10^9 cells/L and hemoglobin ≥ 9 g/dL (5.5 mmol/L), and lymphocytes within normal limits (≥ 0.8 × 10^9 cells/L), platelet count of ≥ 100,000/μL; INR and aPTT ≤ 1.5 × ULN. This applies only to patients who are not receiving therapeutic anticoagulation; patients receiving therapeutic anticoagulation should be on a stable dose. Genetic deficiencies affecting aPTT are not excluded unless assessed as clinically significant by the site hematologist.
9. Adequate liver function: total bilirubin ≤ 1.5 × the upper limit of normal (ULN; excluding Gilbert's Syndrome, when < 3 × ULN is allowed), AST and/or ALT ≤ 2.5 × ULN (in case of liver metastases, ≤ 5 × ULN); Patients with documented liver metastases: alkaline phosphatase ≤ 5 × ULN. For patients with bone disease, this criterion should only take into consideration the specific liver isoform of alkaline phosphatase (not the total since it is influenced by the bone isoform).
10. Adequate renal function: Creatinine clearance ≥ 60 ml/min calculated by CKD-EPI equation (see Appendix 2)
11. Negative serum pregnancy test within 7 days prior to study treatment in premenopausal women and women ≤ 2 years after start of menopause (menopause is defined as amenorrhea for > 2 years)
12. For women of childbearing potential and female partners of male patients: agreement to remain abstinent (refrain from heterosexual intercourse) or use contraceptive methods that result in a failure rate of < 1% per year during the treatment period and for at least 5 months after the last dose of RO6958688 and atezolizumab and 2 months after the last dose of tocilizumab (if applicable).

    For male participants, patient must agree to either remain completely abstinent or to use a condom and not donate sperm during the entire study period and for 3 months after the

"Use of this document is governed by the terms of use on the first page of this document."

last administration of RO6958688 and for 2 months after the last dose of tocilizumab (if applicable). Male patients who have received only atezolizumab are not required to use contraception during atezolizumab treatment; pregnancies in female partners of male patients receiving atezolizumab monotherapy are not required to be reported.

A woman is considered to be of childbearing potential if she is postmenarcheal, has not reached a postmenopausal state (> 2 years of amenorrhea with no identified cause other than menopause), and has not undergone surgical sterilization (removal of ovaries and/or uterus).

Examples of contraceptive methods with an expected failure rate of < 1% per year include bilateral tubal ligation, male sterilization, established, proper use of hormonal contraceptives that inhibit ovulation, hormone-releasing intrauterine devices, and copper intrauterine devices.

The reliability of sexual abstinence should be evaluated in relation to the duration of the clinical trial and the preferred and usual lifestyle of the patient. Periodic abstinence (e.g., calendar, ovulation, symptothermal, or postovulation methods) and withdrawal are not acceptable methods of contraception.

13. Patients with non-colorectal cancer should have confirmed CEA expression in tumor tissue (≥ 20% of tumor cells staining with at least moderate to high intensity of both cytoplasmic and/or membranous (IHC 2+ and IHC 3+). CEA expression should be centrally confirmed for U.S. and Canada patients.

    For CRC cancer patients, the CEA assessment should be performed but the result is not required for patient selection. If no archival tumor tissue is available, fresh biopsy will be collected.

14. For the biomarker cohort (in Part IB), patients should have very low/negative CEA expression. Very low/negative CEA expression is defined as tumor samples having < 20% of tumor cells with IHC1+ or IHC 0+. CEA should be determined prior to enrollment, if no archival tumor is available, a fresh biopsy will be collected.

Exclusion criteria:

Patients who meet any of the following criteria will be excluded from study entry:

1. Active or untreated central nervous system (CNS) metastases as determined by CT or MRI evaluation during screening and prior radiographic assessments

    Patients with a history of treated asymptomatic CNS metastases are eligible, provided they meet all of the following criteria:

    - No metastases to brain stem, midbrain, pons, medulla, cerebellum, or within 10 mm of the optic apparatus (optic nerves and chiasm)
    - Radiographic demonstration of improvement upon the completion of CNS-directed therapy and no evidence of interim progression between the completion of CNS-directed therapy and the screening radiographic study
    - No history of intracranial hemorrhage or spinal cord hemorrhage
    - No ongoing requirement for dexamethasone as therapy for CNS disease; anticonvulsants at a stable dose allowed

“Use of this document is governed by the terms of use on the first page of this document.”

- No stereotactic radiation or whole-brain radiation within 28 days prior to Cycle 1 Day 1
- Screening CNS radiographic study ≥ 4 weeks since completion of radiotherapy and ≥ 2 weeks since discontinuation of corticosteroids.

2. Spinal cord compression not definitively treated with surgery and/or radiation or previously diagnosed and treated spinal cord compression without evidence that disease has been clinically stable for ≥ 2 weeks prior to enrollment.
3. Leptomeningeal disease.
4. Patients with paraspinal, paratracheal and mediastinal pathological lesions larger than 2 cm unless they are previously irradiated. Irradiation of lesions must be completed at least 14 days prior to initiation of study treatment.
5. Malignancies within 5 years prior to enrollment, with the exception of those with a negligible risk of metastasis or death and treated with expected curative outcome (such as adequately treated carcinoma in situ of the cervix, basal or squamous cell skin cancer, localized prostate cancer treated surgically with curative intent, ductal carcinoma in situ treated surgically with curative intent).
6. Significant, uncontrolled concomitant diseases which could affect compliance with the protocol or interpretation of results, including diabetes mellitus, pulmonary disorders, and known autoimmune diseases.
7. Uncontrolled hypertension (systolic blood pressure (BP) > 150 mmHg and/or diastolic BP > 100 mmHg), unstable angina, congestive heart failure (CHF) of any New York Heart Association (NYHA) classification (Class II or greater), serious cardiac arrhythmia requiring treatment (exceptions: atrial fibrillation, paroxysmal supraventricular tachycardia), history of myocardial infarction within 6 months of enrollment.
8. Administration of a live, attenuated vaccine within 28 days before Cycle 1 Day 1 or anticipation that such a live attenuated vaccine will be required during the study. Influenza vaccination should be given during influenza season only (approximately October to March). Patients must not receive live, attenuated influenza vaccine (e.g., FluMist®) within 4 weeks prior to Cycle 1 Day 1 or at any time during the study.
9. Known HIV.
10. Active Hepatitis B (HBV) or Hepatitis C (HCV) infection (required at screening):
    - Patients with active hepatitis B (defined as having a positive hepatitis B surface antigen [HBsAg] test at screening)
        - Patients with past HBV infection or resolved HBV infection (defined as having a negative HBsAg test and a positive antibody to hepatitis B core antigen [anti-HBc] antibody test) are eligible.
    - Patients with active hepatitis C
        - Patients positive for HCV antibody are eligible only if PCR is negative for HCV RNA.

“Use of this document is governed by the terms of use on the first page of this document.”

11. Severe infections within 28 days prior to Cycle 1 Day 1, including but not limited to hospitalization for complications of infection, bacteremia, or severe pneumonia.
12. Received oral or intravenous (IV) antibiotics within 14 days prior to Cycle 1 Day 1. Patients receiving prophylactic antibiotics (e.g., for prevention of a urinary tract infection) are eligible.
13. Any other diseases, metabolic dysfunction, physical examination finding, or clinical laboratory finding giving reasonable suspicion of a disease or condition that would contraindicate the use of an investigational drug.
14. Major surgery or significant traumatic injury < 28 days prior to Cycle 1 Day 1 (excluding biopsies) or anticipation of the need for major surgery during study treatment.
15. Dementia or altered mental status that would prohibit informed consent.
16. Known history of autoimmune disease, including but not limited to myasthenia gravis, myositis, autoimmune hepatitis, systemic lupus erythematosus, rheumatoid arthritis, inflammatory bowel disease, vascular thrombosis associated with antiphospholipid syndrome, Wegener's granulomatosis, Sjögren's syndrome, Bell's palsy, Guillain-Barré syndrome, multiple sclerosis, vasculitis, or glomerulonephritis.

    The protocol allows:

    - Patients with a history of autoimmune hypothyroidism on a stable dose of thyroid replacement hormone
    - Patients with Type 1 diabetes mellitus on appropriate medical management may be considered for this study *after consultation with* the Medical Monitor
    - Patients with positive serology of auto-antibody panel (anti-nuclear antibody, anti-double stranded DNA, cytoplasmic anti-neutrophil cytoplasmic antibody [c-ANCA], and perinuclear anti-neutrophil cytoplasmic antibody [p-ANCA]) at screening should be referred to a specialist (i.e., Rheumatologist) for further assessments if the Investigator, after discussion with the Medical Monitor, considers the results as clinically significant.
17. History of idiopathic pulmonary fibrosis, pneumonitis (including drug induced), organizing pneumonia (i.e., bronchiolitis obliterans, cryptogenic organizing pneumonia, etc.), or evidence of active pneumonitis (including drug induced) on screening chest CT scan. History of radiation pneumonitis in the radiation field (fibrosis) is permitted.
18. Patients with bilateral lung lesions and dyspnea and/or SaO2 <92% (at rest, room air and exertion) or patients with lobectomy or pneumonectomy with lung metastases in the remaining lung and either dyspnea or SaO2 <92% (at rest, room air and exertion) at baseline.
19. Baseline QTc interval of > 470 ms, baseline resting bradycardia < 45 beats per minute, or baseline resting tachycardia > 100 beats per minute.
20. Pregnant or breast-feeding women.
21. Known hypersensitivity to any of the components of RO6958688 and atezolizumab; hypersensitivity to Chinese hamster ovary cell products or other recombinant human antibodies.

"Use of this document is governed by the terms of use on the first page of this document."

22. Investigational therapy (defined as treatment for which there is no regulatory authority approved indication) or last dose of prior immunotherapies including but not limited to: interferon alpha (IFN-α), interferon-beta (IFN-β), IL-2, conjugated IL-2, CEA-IL2v, cytokines, anti-CTLA4, within 28 days prior to Cycle 1 Day 1. Patients previously treated with anti-programmed death-ligand 1 (PD-L1), or anti-PD-1 are excluded.

23. Any approved anti-cancer therapy, including chemotherapy or hormonal therapy, within 28 days prior to Cycle 1 Day 1, with the following exceptions:

    - Hormone-replacement therapy or oral contraceptives
    - Tyrosine kinase inhibitors (TKIs) (both small molecules and antiangiogenic monoclonal antibodies) that have been discontinued > 21 days prior to Cycle 1 Day 1; baseline scans must be obtained after discontinuation of prior TKIs.

24. Prior systemic corticosteroids > 10 mg prednisone (or equivalent) within 14 days of Cycle 1 Day 1. The use of inhaled and/or topical corticosteroids and mineralocorticoids (e.g., fludrocortisone) is allowed.

25. Last dose with any of the following agents including but not limited to: etanercept, infliximab, tacrolimus, cyclosporine, mycophenolic acid, alefacept, or efalizumab < 28 days prior to first dose of study drugs.

26. Regular immunosuppressive therapy (i.e., for organ transplantation, chronic rheumatologic disease).

27. Patients with prior allogeneic bone marrow transplantation or prior solid organ transplantation.

28. Treatment with systemic immunosuppressive medications including, but not limited to: corticosteroids, cyclophosphamide, azathioprine, methotrexate, thalidomide, and anti-TNF agents within 14 days prior to Cycle 1, Day 1. Patients who have received acute and/or low-dose systemic immunosuppressant medications (e.g., a one-time dose of dexamethasone for nausea or chronic use of ≤ 10 mg/day of prednisone or dose-equivalent corticosteroid) may be enrolled in the study after discussion with the Medical Monitor. The use of inhaled corticosteroids and mineralocorticoids (e.g., fludrocortisone) for patients is allowed.

29. Radiotherapy within the last 28 days before Cycle 1 Day 1 with the exception of limited field palliative radiotherapy e.g., for bone pain relief.

LENGTH OF STUDY

The maximum treatment period for this study is 24 months for both RO6958688 and atezolizumab and may be modified if emerging data suggest a different treatment period. Patients will be treated until lack of clinical benefit, unacceptable toxicities, or withdrawal from treatment for other reasons or death. All patients will attend a safety follow-up visit 28 days (± 2 days) after receiving the last infusion of RO6958688 or atezolizumab.

The total study duration is estimated to be 60 months.

"Use of this document is governed by the terms of use on the first page of this document."

END OF STUDY

The study will formally end once all patients have completed the safety follow-up visit, withdrawn from the study or when all patients have been enrolled in an extension study, whichever occurs last (the option to enroll in an extension study is for patients receiving atezolizumab only).
The Sponsor may also decide to terminate the study at any time.

OUTCOME MEASURES

SAFETY OUTCOME MEASURES

The safety outcome measures for this study are:

- Incidence and nature of DLTs
- Incidence and severity of adverse events and IRRs and CRS symptoms
- Incidence of laboratory abnormalities (as examples and not limited to: hematology testing, coagulation, serum chemistries, and urinalysis)
- Incidence of ADAs (anti-atezolizumab antibodies and anti-RO6958688 antibodies) formation, detection of cytokine release and potential correlation with PK, PD, safety, and efficacy parameters
- Incidence of autoantibodies (anti-nuclear antibody, anti-double-stranded DNA, cytoplasmic anti-neutrophil cytoplasmic antibody, and perinuclear anti-neutrophil cytoplasmic antibody) in comparison to baseline
- Changes in vital signs, physical findings and ECG findings

All patients who participate in the study will be clinically evaluated at screening and on a regular basis during the entire course of the study. The routine safety monitoring plan will include clinical examination, vital signs assessment (body temperature, respiratory rate, heart rate, diastolic and systolic BP, oxygen saturation measured by pulse oximeter – finger clip), ECG, laboratory analysis (hematology, coagulation, biochemistry, urinalysis, presence of autoantibodies), and regular collection and review of the reported adverse events.

The National Cancer Institute Common Terminology Criteria for Adverse Events v4.03 will be used to evaluate the clinical safety of the treatment in this study (https://evs.nci.nih.gov/ftp1/CTCAE/CTCAE_4.03/CTCAE_4.03_2010-06-14_QuickReference_8.5x11.pdf). However, CRS adverse events considered related to RO6958688 will be evaluated using NCI CTCAE v5 (Table 3). Patients will be assessed for adverse events at each clinical visit and as necessary throughout the study.

Safety will be determined, but not limited to, by the spontaneous reporting of adverse events; by the assessments of routine laboratory values (hematology testing, serum chemistries); findings on physical examinations; ECGs; chest X-ray; vital signs; by carefully observing patients for IRRs; by the determination of ADAs.

Please refer to schedule of assessments for details on collection time of the assessments outlined below.

Laboratory Tests

Hematology and biochemistry will be analyzed at least prior to RO6958688 and atezolizumab administration as part of the regular safety assessments.

1. Hematology: erythrocytes, hemoglobin, hematocrit, platelets, leucocytes and differential count (i.e., neutrophils, eosinophils, basophils, monocytes, lymphocytes).
2. Coagulation: prothrombin time (PT)/international normalized ratio (INR) and partial thromboplastin time (PTT) and fibrinogen. Additional coagulation parameters (i.e., first chromogenic antithrombin III then antigenic antithrombin III in case of chromogenic antithrombin III decrease in order to determine type 1 and type 2 antithrombin

“Use of this document is governed by the terms of use on the first page of this document.”

deficiencies. If a deficiency is detected, both antithrombin tests will need to be repeated at a later date/visit to confirm test findings, fibrinogen, prothrombin time, fibrin degradation products, D-dimer) could be assessed according to clinical judgment, or if any of the above parameter cannot be assessed locally.

3. Blood biochemistry: sodium, potassium, chloride, calcium, phosphate, magnesium, urea, creatinine, normal glomerular filtration rate (by CKD-EPI equation), total protein, albumin, glucose, total and direct bilirubin, alkaline phosphatase (ALP), alanine aminotransferase (ALT), aspartate aminotransferase (AST), lactate dehydrogenase (LDH), γ-glutamyl transferase (GGT), C reactive protein (CRP)., NS cholesterol (total, LDL cholesterol, HDL cholesterol), triglycerides, Thyroid-stimulating hormone (TSH), ferritin, soluble CD25.

   Soluble CEA (sCEA) measured in serum or plasma, will also be measured as a disease monitoring marker.
4. Urinalysis: dipstick for pH, glucose, blood, protein, ketones, and bilirubin. If there is a clinically significant positive result (i.e., confirmed by a positive repeated sample), urine will be sent to the laboratory for microscopy and culture. If there is an explanation for the positive dipstick result, e.g., menses, it should be recorded, and there is no need to perform laboratory for microscopy and culture.
5. HBV and HCV screening tests at screening.
6. All women of childbearing potential (including those who have had a tubal ligation) will have a serum pregnancy test at screening, within 7 days of first dose, on a regular basis during the treatment period (urine) and at the follow up visit.
7. Autoantibodies panel: anti-nuclear antibody, anti-double stranded DNA, cytoplasmic anti-neutrophil cytoplasmic antibody [c-ANCA], and perinuclear anti-neutrophil cytoplasmic antibody [p-ANCA]

Unscheduled hematology, biochemistry, and coagulation assay will be obtained in patients who develop infusion–related reactions (IRRs) and as clinically indicated.

Additional safety laboratory assessments:

- ADA
- Cytokine release assessment
- IgE and tryptase for patients who experience a Grade ≥ 2 IRR, see Schedule of assessments – Table A3.

PHARMACOKINETIC OUTCOME MEASURES

Pharmacokinetic (PK) concentration data of RO6958688 and atezolizumab will be summarized with the use of descriptive statistical methods. PK parameters (area under the concentration curve [AUC], volume of distribution at steady state [V_{ss}], minimum and maximum serum concentration [C_{min} and C_{max}], clearance [CL], and half-life (t1/2) (terminal and effective)) of RO6958688 and atezolizumab will be estimated using non-compartmental analysis (NCA) methods when applicable.

PHARMACODYNAMIC OUTCOME MEASURES

The PD outcome measures for this study are:

- **Whole blood samples:** Peripheral blood immune cells will be assessed with respect to the changes in the characteristics of lineage (CD4+ T cells, CD8+ T cells, natural killer [NK] cells, monocytes, T-regulatory cells, and B cells), activation (including but not limited to CD25, CD69, etc.), and differentiation (including but not limited to CD45RO Ki67, PD1, TIM3, ICOS, etc.). In addition, whole blood samples will be taken for TCR Vβ sequencing

“Use of this document is governed by the terms of use on the first page of this document.”

(the CDR3-TCR beta chain repertoire). The DNA will be used to determine the immune repertoires of peripheral T cell receptor (TCR) Vβ CDR3 and analyze TCR diversity.

- **Serum or plasma samples:** PD biomarkers such as cytokines and inflammation markers (including but not limited to tumor necrosis factor-α (TNFα), interferon-γ (INFγ), interleukin (IL)-6, MIP, etc.) will be analyzed. Because these measurements are also safety measure assessments during any IRRs, they will also be examined in patients enrolled in both Part I and Part II of the study. Disease-monitoring markers that include but are not limited to sCEA will also be assessed.
- **Tumor biopsy:** Tumor biopsy samples will be obtained from all patients enrolled. These paired tumor biopsies for PD analyses are mandatory at baseline and on-treatment, except for NSCLC patients for whom there is no accessible lesion. If feasible, biopsies may be repeated if the initial biopsy did not contain sufficient tumor material for analysis. For patients discontinuing from the study due to disease progression, additional optional biopsies, if clinically feasible, may be taken to aid the understanding of immune resistance mechanisms. Biopsies will be assessed centrally for changes in immune cell numbers and activation characteristics as well as changes in tumor markers such as PD-L1. These analyses will be performed by flow cytometric molecular and/or immunohistochemistry methods with respect to changes in the characteristics of lineage (CD4+ T cells, CD8+ T cells, NK cells, monocytes, T-regulatory cells, and B cells), activation (including but not limited to CD25, CD69, etc.), differentiation (including but not limited to CD45RO Ki67, PD1, TIM3, ICOS, etc.), and TCR Vβ repertoire and tumor mutational load.
- **Positron Emission Tomography (PET):** Baseline and on-treatment 2-[18F]Fluoro-2-deoxyglucose positron emission tomography (FDG-PET) will be collected to determine changes in glucose metabolism of the tumor lesions.
- **Original or archival tumor:** Potential prognostic biomarkers such as MMR status and CEA expression will be confirmed on archival tumor, if available, or from the freshly obtained biopsy samples. These measurements will assess the CEA change over the course of the disease and the stability of the measurements.

Blood, Serum or Plasma Samples

Blood samples will be collected for the analyses of immune cell number and activation. Blood samples will be collected and analyzed with respect to alterations in the number and activation and differentiation of immune cells as a consequence to treatment with RO6958688 in combination with atezolizumab. The samples will be analyzed by flow cytometry, and the number of cells that belongs to lymphocyte subsets (CD4+ T cells, CD8+ T cells, NK cells, B cells, and monocytes) and their activation and differentiation status will be determined (including but not limited to CD25, Ki67, PD1, and TIM3). Additional immune PD biomarkers that are related to the mode of action (MoA) of RO6958688 in combination with atezolizumab may also be analyzed from serum or plasma samples taken from all patients. Such PD measures will be considered as disease-monitoring markers and include but are not limited to sCEA. If available, an assessment of tumor growth kinetics will be made by comparing post-treatment scans with the last available pre-study scan.

Tumor Biopsy Samples

Tumor biopsy samples (each consisting of two tissue specimens at least the size of an 18G core needle biopsy, fine-needle aspiration is not acceptable) will be collected in all enrolled patients (once at baseline [after the baseline FDG PET] to confirm eligibility and once during the study treatment period), and two core samples will be collected at each occasion. Mandatory biopsies will be collected from all patients enrolled, except for NSCLC patients for whom there is no accessible lesion. The biopsies will be taken from accessible, “non-critical” tumor locations, including, but not limited to, skin, lymph node, rectum, liver, etc. On-treatment tumor biopsies will

“Use of this document is governed by the terms of use on the first page of this document.”

be randomized within dose schemes as follows: half of the patients at week 3, half of the patients at week 6.

Tumor biopsies should be taken predose (and up to 48h before the visit).

If feasible, biopsies may be repeated if the initial biopsy did not contain sufficient tumor material for analysis.

If preliminary data suggest that modification of the on-treatment tumor biopsy timepoint would be more appropriate, alternative on-treatment tumor biopsy timepoints could be considered in the future cohorts.

The baseline and on-treatment biopsies should preferentially be taken from the same tumor lesion to ensure comparability when accessible in a non-critical location. Data from FDG-PET should guide which lesion would be more reflective of capturing the PD effect and thus the baseline and on-treatment biopsies should be taken from that tumor lesion. For patients continuing in the study, additional biopsies may be taken at the discretion of the investigator. These biopsies may serve to evaluate viable tumor given the mechanism of action of RO6958688 to induce tumor inflammation. For patients discontinuing from the study due to disease progression, additional optional biopsies, if clinically feasible, may be taken to aid the understanding of immune resistance mechanisms.

Tumor biopsies will be centrally analyzed for immune cell number and activation by flow cytometric and/or immunohistochemical methods for the density of different immune cell lineages (including but not limited to CD4+, CD8+, B cells, NK cells, macrophages) and their activation and differentiation status (including but not limited to CD25, Ki67, PD1, TIM3) and for the expression of tumor markers such as PD-L1, IDO etc... In addition, gene expression analysis may be carried out. The goals of these analyses will be (i) to establish a dose–response and/or an exposure-response relationship and (ii) to understand the MoA of RO6958688 in combination with atezolizumab at the tumor site.

Positron Emission Tomography (PET)

FDG-PET can identify sign of biological effect early, before tumor size is reduced. Moreover, a reduction in the FDG-PET signal within days or weeks of initiating therapy (e.g., in lymphoma, non–small cell lung, and esophageal cancer) has been shown to correlate with prolonged survival and other clinical end points now used. These findings suggest that FDG-PET could facilitate drug development as an early marker of drug effect.

Whole-body PET should begin 60 ± 10 min after FDG injection (please refer to the WP29945 imaging manual for more details).

EFFICACY OUTCOME MEASURES

Any evaluable or measurable disease must be documented at screening and re-assessed at each subsequent tumor evaluation. The efficacy/activity outcome measures for this study are:

- ORR, defined as the proportion of patients achieving objective partial (PR) or complete (CR) responses
- Best overall response (BOR) defined as the best response recorded from the start of the study treatment until the end of treatment taking into account any requirement for confirmation
- Duration of response (DOR) defined as the time from initial objective response (PR or CR) to the first objective progression or death from any cause
- The rate of patients with stable disease (SDR)
- DCR, defined as ORR + SDR
- PFS according to Response Evaluation Criteria in Solid Tumors, Version 1.1 (RECIST v1.1)
- OS, defined as the time from first study treatment (primary efficacy analysis) or randomization date (ITT, only in randomized schedule comparison expansions) to death from any cause

“Use of this document is governed by the terms of use on the first page of this document.”

Tumor response will be evaluated according to RECIST v1.1 and modified RECIST criteria using unidimensional measurement such as computed tomography (CT) scan or magnetic resonance imaging. Assessment of CT/MRI scans as tumor assessments will be performed at the sites during the whole study, and in addition centrally by an independent reviewer for prospective and retrospective analysis. Advanced volumetric analyses of CT/MRI will be performed.

Tumor assessment will be performed once during the screening. The first assessment after the start of treatment will be performed at 8 weeks (C3D15) and continue every 8 weeks thereafter for the first year and every 12 weeks thereafter until disease progression or treatment discontinuation. All tumor assessments after baseline may be done within ±7 days of the scheduled visit. Additionally, FDG-PET based tumor assessment will be performed at baseline and at week 4 (C2D8 + 1 week) and week 16 (C6D8) after the first dose of RO6958688, in case of dose delay the FDG PET should be done at week 4 (+1 week) and week 16. The week 16 FDG PET scan should be performed predose (and up to a maximum of 3 days before the visit).

Based on data generated during this trial, the timepoints at which the two on treatment FDG PET scans are performed may be modified.

Confirmation of partial and complete responses will be done at the next scheduled visit after at least 28 days from the initial response. A patient is assigned a best overall confirmed response SD if they have a response assessment of SD, PR, or CR at one or more visits at least 42 days (6 weeks) after start of study treatment, but are not a confirmed CR or PR.

PFS per RECIST v1.1 or modified RECIST criteria is defined as time between enrollment or randomization date (whichever is applicable) and date of first documented disease progression per RECIST v1.1 or modified RECIST criteria, respectively, or death from any cause, whichever occurs first. Patients who neither progressed nor died in this interval, or who are lost to follow-up are censored at the date of last tumor assessment within this time window or last follow-up for progression of disease. Patients for whom no post-baseline tumor assessments are available are censored at first study treatment.

Only for the randomized schedule comparison expansions, PFS and OS in the ITT efficacy analysis population may be analyzed for comparison of the schedules, where the start is defined as the date of obtaining informed consent by the patient.

Conventional response criteria may not adequately assess the activity of immunotherapeutic agents because progressive disease (by initial radiographic evaluation) does not necessarily reflect therapeutic failure. Because of the potential for pseudoprogression/tumor immune infiltration, this study will allow patients to receive atezolizumab and/or RO6958688 to remain on study treatment after apparent radiographic progression, provided the benefit-risk ratio is judged to be favorable.

Patients should be discontinued for unacceptable toxicity or loss of RO6958688 exposure (in which case they can continue to receive atezolizumab alone) or symptomatic deterioration attributed to disease progression as determined by the investigator after an integrated assessment of radiographic data and clinical status (see Section 4.6.1.1).

Patients will be permitted to continue study treatment after meeting RECIST criteria for progressive disease (by investigator assessment) if they meet all of the following criteria:

- Evidence of clinical benefit as assessed by the investigators
- Absence of significant symptoms and signs (including worsening of laboratory values; e.g., new or worsening hypercalcemia) that indicate unequivocal progression of disease
- No decline in ECOG performance status that can be attributed to disease progression
- Absence of tumor growth at critical anatomical sites that cannot be managed by protocol–allowed medical interventions

- Patients for whom approved therapies exist must provide written consent to acknowledge that they defer these treatment options in favor of continued study treatment at the time of initial apparent progression.

“Use of this document is governed by the terms of use on the first page of this document.”

Patients in whom radiographic disease progression is confirmed at a subsequent tumor assessment may be considered for continued study treatment at the discretion of the investigators if they continue to meet the criteria above and have evidence of clinical benefit.

Optional submission of the latest pre-study or historical CT scans is highly encouraged for assessment of tumor growth kinetics within 6 weeks of patient entering the study if available. This scan will be compared to those collected during the study to determine tumor growth kinetics.

EXPLORATORY OUTCOME MEASURES

The exploratory objectives for this study include but are not limited to the following:

- A possible association of expression of activation related immune genes (e.g., IFNγ, CXCLC9, etc.) with PD response will be investigated
- The baseline values and kinetics of soluble markers of immune cell activation (such as sCD25) and tumor markers (such as CEA) will be explored.

Additional markers may be measured in case a scientific rationale for these analyses develops.

BIOMARKER/GENOTYPING SAMPLE COLLECTION

The specimens will be used for research purposes to identify biomarkers useful to predict and monitor response to RO6958688 and atezolizumab treatments, identify biomarkers useful to predict and monitor safety of the combination of the two compounds, assess PD effects of RO6958688 and atezolizumab treatments, and investigate mechanisms of immune escape. Additional markers may be measured in case a scientific rationale for these analyses develops.

Tumor

Tumor biopsy samples (each consisting of at least two tissue specimens at least the size of a 18G core needle biopsy, fine-needle aspiration is not acceptable) will be collected from all patients who participate in the study on two occasions (once at baseline [after the baseline FDG PET] to confirm eligibility and once during the study treatment period), and at least two core samples will be collected at each occasion. For patients discontinuing from the study due to disease progression, additional optional biopsies, if clinically feasible, may be taken to aid the understanding of immune resistance mechanisms. The biopsies will be taken from accessible, “non-critical” tumor locations, including, but not limited to, skin, lymph node, rectum, liver, etc. If feasible, biopsies may be repeated if the initial biopsy did not contain sufficient tumor material for analysis. In particular, archival tumor tissue is to be obtained from all patients, if available, in order to perform CEA assessment for patient eligibility (refer to the Laboratory Manual).

If a patient undergoes any medical procedure during the course of the study that may yield tumor tissue, any remaining samples or any portion of the tumor sample not used for medical diagnosis may be obtained for exploratory analysis. Patients must provide specific consent in order for discarded samples from routine care to be used for exploratory analysis.

Whole Blood

Whole blood samples will be collected for the flow cytometry for determination of immune cell markers (e.g., immune cell subsets, activation and proliferation markers, etc.). In addition, whole blood samples will be taken for TCR Vβ sequencing (the CDR3-TCR beta chain repertoire). The DNA will be used to determine the immune repertoires of peripheral T cell receptor (TCR) Vβ CDR3 and analyze TCR diversity.

Soluble CEA

A mandatory blood sample will be taken for soluble CEA central assessment.

“Use of this document is governed by the terms of use on the first page of this document.”

Serum or plasma

Blood for serum or plasma isolation will be collected for investigation of PD markers such as sCD25, cytokines (such as IL-6, IFNγ, TNFα), and tumor markers. In the event of an IRR/CRS, an additional sample will be collected.

These samples will be destroyed within 2 years after the date of final closure of the clinical database. Archival tumor blocks will be returned. Other residual tissue material (slides, extracts, etc.) will be destroyed within 2 years after the date of final closure of the clinical database unless the patient gives specific consent for the remainder of the tissue sample(s) to be stored for optional exploratory research. If the patient provided consent for optional exploratory research the tissue samples will be destroyed no later than 15 years after the date of final closure of the clinical database.

Clinical Genotyping Samples

A mandatory baseline whole blood sample will be taken from every patient for DNA extraction. The DNA may be used to determine if alleles at genes associated with immune responses such as Chemotaxis, HLA, immunosuppression etc, affect the PK/PD/efficacy/safety of RO6958688 and atezolizumab. In addition, the DNA may be used as a reference to identify tumor mutations. Data arising from this study will be subject to the same confidentiality as the rest of the study. This specimen will be destroyed immediately after analysis and the results have been checked.

ROCHE CLINICAL REPOSITORY

The Roche Clinical Repository (RCR) is a centrally administered facility for the long-term storage of human biological specimens including body fluids, solid tissues and derivatives thereof (e.g., DNA, RNA proteins/peptides). Specimens for dynamic (non-inherited) biomarker discovery and validation will be collected from patients who consent to participate in the RCR.

These specimens will be used for research purposes to identify biomarkers that are predictive of response to treatment with RO6958688 in combination with atezolizumab, and will help to better understand the pathogenesis, course, and outcome of the studied cancer types. The collected samples might allow the generation of statistically meaningful biomarker data.

The results of specimen analysis from the RCR will facilitate the rational design of new pharmaceutical agents and the development of diagnostic tests, which may allow for individualized drug therapy for patients in the future.

The specimens in the RCR will also be made available for future biomarker research towards further understanding of RO6958688 in combination with atezolizumab, treatment of related diseases and adverse events and for the development of potential associated diagnostic assays.

Whole blood samples for extraction of DNA and RNA will be collected if patients consent to participate in the RCR. Patients will also have the option to consent that any tissue material remaining after protocol defined analyses can be stored for up to 15 years in the RCR.

INVESTIGATIONAL MEDICINAL PRODUCTS

Test Products

For the purpose of the study, RO6958688, atezolizumab, and tocilizumab are considered investigational medicinal products (IMPs).

RO6958688

RO6958688 is a novel T-cell bispecific antibody targeting the human CEA on tumor cells and CD3 on T-cells. RO6958688 is administered intravenously, in patients with locally advanced and/or metastatic CEA (+) solid tumors. The starting dose of RO6958688 is 5 mg. QW dosing will be implemented initially to generate data that can be analyzed to assess whether different dosing schedules are more effective. If the accumulated data (safety, PK, PD) support that a different

“Use of this document is governed by the terms of use on the first page of this document.”

schedule might be more appropriate, an alternative dosing schedule, such as every-other-week dosing, could be considered.

In general, if patients experience adverse events that require a RO6958688 dose to be held, the dose may be delayed up to 14 days from the last one for the QW part and up to 21 days for the Q3W part. However, the acceptable length of interruption will depend on an agreement between the investigator and the Medical Monitor.

Atezolizumab

Atezolizumab is a human monoclonal antibody engineered to eliminate Fc-effector function. Atezolizumab targets human PD-L1 and inhibits its interaction with its receptor, programmed death−1 (PD-1). Atezolizumab also blocks the binding of PD-L1 to B7.1, an interaction that is reported to provide additional inhibitory signals to T cells. Atezolizumab is administered intravenously at 1200 mg IV Q3W (every 3 weeks).

In general, if patients experience adverse events that require an atezolizumab dose to be held, the dose may be delayed up to 105 days from the last one. However, the acceptable length of interruption will depend on an agreement between the investigator and the Medical Monitor.

Tocilizumab

Tocilizumab (Actemra®/RoActemra®) is a recombinant, humanized, anti-human monoclonal antibody directed against soluble and membrane-bound IL-6R, which inhibits IL-6 mediated signaling. Blocking the inflammatory action of IL-6 using tocilizumab could therefore represent a novel approach for the treatment of CRS.

Tocilizumab will be administered if required, for the management of severe CRS (if a study participant experiences severe CRS during or after any infusion of RO6958688. Tocilizumab has been recently approved by the FDA (August 2017) and received a positive CHMP opinion (European Medicines Agency 2018) for a restricted use, limited to Cytokine Release Syndrome (CRS) induced by chimeric antigen receptor (CAR) T cell in adults and pediatric patients 2 years of age and older (Tocilizumab USPI). Since tocilizumab will be used in the event of a severe CRS during or after any infusion of RO6958688, it is therefore classified as an investigational medicinal product (IMP).

NON-INVESTIGATIONAL MEDICINAL PRODUCTS

None.

PROCEDURES

SCREENING: Written informed consent for participation in the study must be obtained before performing any study-specific screening tests or evaluations. Screening and pretreatment assessments will be performed within 28 days prior to Cycle 1 Day 1 predose unless otherwise specified.

TREATMENT: Assessments performed during treatment are specified in the Schedule of Assessment and Hourly Assessment tables.

FOLLOW-UP: Patients who complete the study or discontinue from the study early will be asked to return to the clinic 28 days after the last dose of study drugs for a safety follow-up visit. The visit at which response assessment shows progressive disease may be used as the study completion/early termination visit.

LIST OF SPECIAL SAFETY OR PD TESTS: Although RO6958688 and atezolizumab are humanized antibodies, there is a risk that ADA against RO6958688 and atezolizumab may develop, potentially reducing its efficacy and/or potentially resulting in symptomatic hypersensitivity reactions, in particular immune-complex reactions. In Part IA in order to overcome ADA and impact on PK, optional intra-patient dose escalation of RO6958688 to the

“Use of this document is governed by the terms of use on the first page of this document.”

next available tolerated dose level may be permitted depending on emerging clinical and safety data at the discretion of the treating physician, and after discussion with the patient. Intra-patient dose escalation may only proceed after patients have tolerated at least the first 3 consecutive doses of RO6958688 after discussion and alignment with the Medical Monitor. In this study, samples will be taken to assess the possible presence of ADA. The date and time of each sample will be recorded in the electronic Case Report Form.

For the assessment of cytokine release, serum or plasma samples (blood for PK, pharmacodynamics and additional safety) will be collected at the time of an IRR/CRS (including repetitive occurrence of IRR/CRS). Cytokine analysis will include but is not limited to TNFα, IL-6, and IFNγ.

For patients who experience a Grade ≥2 IRR/CRS within 24 hours after the study drugs infusion, tryptase and total IgE will be analyzed.

WITHDRAWAL CRITERIA: Reasons for discontinuation of study drugs or withdrawal from the study may include but are not limited to the following:

- Patient withdrawal of consent at any time
- Any medical condition that the investigator or Sponsor determines may jeopardize the patient's safety if he or she continues in the study
- Investigator or Sponsor determines it is in the best interest of the patient.

All patients will attend a 28-day safety follow-up visit after receiving the last infusion of RO6958688 or atezolizumab.

BLINDING OR UNBLINDING:

Not applicable.

STATISTICAL METHODS

DOSE-FINDING APPROACH

Part IA: Dose escalation

A modified continual reassessment method (mCRM) with overdose control (EWOC) for dose escalation, based on occurrence of DLT, will be used for the dose escalation of RO6958688 in combination with 1200 mg Q3W atezolizumab. The model will be estimated using Bayesian inference with the priors properly pre-defined (based on safety data from monotherapy studies with RO6958688 and atezolizumab). Details on the statistical model, the priors, and the dose-escalation algorithm are defined in the protocol.

The maximum allowable increment for RO6958688 between dose-levels will be 100% from the current dose level throughout the study. The dose of RO6958688 will not be escalated above the monotherapy MTD if defined. Clinical judgment may always override mCRM recommendations in the dose-selection process.

With the end of Part I, a tentative MTD estimate will be defined. The DLT occurrence data from the additional patients with QW schedule in Part I and Part II of the study will be used to update the statistical model used in Part I and lead to a final MTD definition at the end of this trial.

Part IB: Dose/schedule finding

An intra-patient dose escalation design is used to estimate the late cycle MTD of RO6958688 in combination with 1200 mg Q3W atezolizumab in Cohort B1 in Part IB (see Protocol Section 6.7.1.2). The dose of RO6958688 will not be escalated in the intra patient dose escalation above the monotherapy late cycle MTD of RO6958688 if defined in the BP29541 protocol. In addition, a fast escalation design of RO6958688 in combination with 1200 mg Q3W atezolizumab is investigated in cohort B2. Note: Cohort B2 has not and will not enroll any patients. Cohort B1 will not enroll any additional patients.

"Use of this document is governed by the terms of use on the first page of this document."

Furthermore, Part IB of the study will explore two additional step-up dose regimens (cohorts C1 and C2) in combination with atezolizumab 1200 mg Q3W, in a randomized schedule comparison expansion, in order to contribute to determination of a recommended dose and schedule for further development (RP2D). Based on the clinical data from the randomized cohorts (C1 and C2), an additional step-up dose regimen (C3) might be explored as well. In addition, MSS CRC Cohort G1 will explore one schedule with obinutuzumab pretreatment, and the optional Cohorts G2 and G3 could generate data for gastric and pancreatic cancer patients with obinutuzumab pretreatment. Descriptive and model-based comparisons of efficacy endpoints as well as safety summaries will be produced in order to support this decision. Note: Cohort C1 enrolled 39 patients, and Cohort C2 enrolled 35 patients; no further patients will be enrolled into these cohorts. Optional cohort C3 and cohorts G1, G2, and G3 have not and will not enroll any patients.

Finally, maximum five additional safety cohorts in other tumor types will be explored, without formal estimation of maximum tolerated doses. Note: These cohorts will not enroll any additional patients.

Part II: Expansion

The expansion part may generate more efficacy data at the recommended dose and schedule. Statistical models will be used to summarize the efficacy data obtained across different dose levels and schedules, in order to contribute to the final selection of a recommended dose and schedule. Note: Part II has not and will not enroll any patients.

SAFETY ANALYSES

One of the primary objectives of the study is to evaluate the safety profile of RO6958688 in combination with atezolizumab. Therefore, the primary endpoints of the study contain safety and, in particular, the occurrence of DLT. All patients enrolled in the study who receive at least one dose of any of the study medications will be included in the safety evaluation. Safety will be determined, but not limited to, by adverse events, laboratory tests, ADAs, autoantibodies, vital signs, ECGs, physical examinations, and performance status, as well as by DLTs. As appropriate, listings, summary tables, and graphs will be provided for safety and tolerability assessments.

PHARMACOKINETIC AND IMMUNOGENICITY ANALYSES

PK data from patients who will receive at least one dose of RO6958688 or one dose of atezolizumab will be included in the PK analysis of RO6958688, atezolizumab respectively. Patients will be excluded from the PK analysis if they violate the inclusion or exclusion criteria, deviate significantly from the protocol, or if data are unavailable or incomplete, which may influence the PK analysis.

Extensive PK sampling will be done in the study to appropriately characterize the PK of RO6958688 and atezolizumab when given in combination.

PK parameters will be derived from the plasma concentrations of RO6958688, atezolizumab using standard non-compartmental methods, as appropriate. Individual data will be listed and summarized using descriptive statistics, including but not limited to mean, standard deviation, geometric mean, coefficient of variation, median, and range. The parameters will include, e.g., AUC, CL, Vss, accumulation ratio, and terminal elimination half-life. Mean concentration versus time will be plotted on either semi-logarithmic or normal scales.

In addition, non-linear mixed effect modeling will be used to analyze the sparse sampling dose-concentration-time data of RO6958688. Population PK parameters such as CL and V will be estimated and the influence of various covariates on these parameters will be investigated. Secondary parameters such as AUC and Cmax will be derived from the individual post-hoc predictions. The linearity of PK (AUC, C_{max}) will also be investigated.

“Use of this document is governed by the terms of use on the first page of this document.”

PK sampling times, samples for ADA will be collected for this study according to the Schedule of Assessments and Hourly tables (SoA).

PHARMACODYNAMIC ANALYSES

The PD analysis population will be a subset of the efficacy analysis population, based on the availability of the outcome measures. Descriptive statistics and graphical outputs will be used in summarizing peripheral blood, skin, and tumor PD markers. Absolute and percentage change from baseline will be calculated for the PD markers.

Correlations between the CEA expression level and pharmacodynamics and efficacy outcomes will be explored using graphical and modelling techniques.

PHARMACOKINETIC-PHARMACODYNAMIC ANALYSES

The time course of tumor size, imaging parameters, PD markers and/or safety measurements may be regarded as response variables to drug exposure. Exploratory graphical analyses of exposure-efficacy relationships will be done for selected PD and/or safety measurements if possible. If the data permit, a PK/PD modeling approach may be considered in order to further explore the exposure-response relationship of selected response variables.

EFFICACY ANALYSES

Two efficacy analyses will be performed. The primary efficacy analysis population will consist of all patients who receive at least one dose of any study drug. An additional ITT population will consist of all patients who are not screen failures and were included in the trial (ICF signature), and will be used for sensitivity analyses. Patients who have been treated before Cycle 1 Day 1 with RO6958688 will be analyzed separately.

Tumor response data will be reported using descriptive statistics. ORR, SDR, and DCR at relevant timepoints will be summarized using relative frequencies and 95% confidence limits. Duration of response (DOR) and progression free survival (PFS) on treatment will be summarized using time to event analyses and Kaplan Meier curves. Duration of response will only be analyzed in those cohorts with a sufficient number of responders. Preliminary overall survival (OS) data may as well be tabulated and summarized using time to event analyses and Kaplan Meier curves. Summaries will be carried out by cohort, dose, and overall. This will be carried out for both RECIST and modified RECIST efficacy endpoints, for investigator assessed data for the whole study and for centrally assessed data for prospective and retrospective analysis.

The analysis of tumor response is based on the best overall response (BOR). BOR is defined as the best response recorded from the start of treatment until disease progression/recurrence or death whichever occurs first. Since tumor assessments take place every 8 weeks, 60 days is chosen so as to cover these assessments sufficiently.

For the ORR analyses in the primary and ITT efficacy analysis populations, patients who withdraw study treatment because of any reason, die or clinically progress before the first tumor assessment on treatment will be assigned a best overall response of Non Evaluable. These patients will be included in the denominator of the corresponding ORR estimate.

SAMPLE SIZE JUSTIFICATION

The sample size estimation for the dose escalation in Part IA is based on study simulations of the mCRM with EWOC design, see Section 6.2 for details.

The sample size of 20 patients per dose scheme in the schedule comparison expansion (cohort A) in Part IB allows for a reasonably precise differentiation between the QW and Q3W dose schemes. Specifically, an observed difference of ca. 10% points in ORR would lead to approximately 80% posterior probability of a true response rate difference between two dose schemes. For example, if the number of objective responses is 4 in one and 2 in the other dose

"Use of this document is governed by the terms of use on the first page of this document."

scheme out of 20 patients each, then the posterior probability of a positive response rate difference is 79.5%.

For cohort C, the randomization of approximately 40 patients to each of the arms C1, C2 and possibly 40 patients enrolled in a later optional arm C3 allows for a reasonably precise differentiation between the arms. Specifically, if one of the arms has 20% true ORR, and the other two arms have only 10% true ORR, then n=40 patients per arm gives 83% power to decide for the correct arm as the best one. In another scenario, where the first arm has 10% true ORR, the second arm 20% true ORR, and the third arm 30% true ORR, then n=40 patients per arm gives 85% power to pick the correct third arm as the best one. Hence, the sample size of 40 patients per arm in cohort C is justified. Note: Cohort C1 enrolled 39 patients, and Cohort C2 enrolled 35 patients; no further patients will be enrolled into these cohorts. Cohort C3 has not and will not enroll any patients.

For Cohort G1, the inclusion of 40 patients will allow a reasonably precise estimation of the incidence of ADA. Specifically, if none of the 40 patients develops ADA by week 8, then the 95% confidence interval for the ADA incidence will be 0% to 9%, i.e., exclude values of 10% or higher. In addition to the assessment of immunogenicity reduction by obinutuzumab, Cohort G1, as well as optionally Cohorts G2 and G3 will allow for a descriptive assessment of the primary safety and efficacy endpoints in the MSS CRC and optionally in the Gastric and Pancreatic indications, respectively. Specifically, observing an ORR of 20% in a cohort would result in a 95% confidence interval from 9% to 36%, i.e., exclude values of 9% or lower. Note: Cohorts G1, G2, and G3 have not and will not enroll any patients.

The sample size of approximately 10-20 patients in each of the cohorts B1 (MSS and MSI-H CRC separately), B2 and the safety cohorts in other indications allows for an initial assessment of safety and tolerability. This is shown in detail with simulations of the intra-patient dose escalation design in cohort B1 in protocol Appendix 7. Note: Cohort B2 has not and will not enroll any patients. The other cohorts have stopped enrollment.

Furthermore, mandatory tumor biopsies of all patients (except for NSCLC patients for whom there is no accessible lesion) are required for the secondary objective of characterizing the pharmacodynamic (PD) effects and duration of PD response on the basis of an increase in activated intratumoral T cells. In Part I, the collected PD data will allow to better define the dose range for the final RP2D and schedule selection. Therefore, it is necessary to have mandatory biopsies for all patients in Part I, because potentially many different doses will be tested in Part I, hence the sample size per dose and schedule combination will be small and has to be maximized.

Interim Analyses

Throughout the whole study, there will be ongoing safety assessments and PK data analyses, which do not qualify as formal interim analyses. In particular, dose escalations in Part I will be based on the ongoing safety review of the data and especially DLT data as per the mCRM with EWOC design.

In addition, there will be an internal interim analysis after Part I for selecting the recommended dose and schedule for Part II. Note: Part II will not open.

CENTERS

Approximately 35 centers in North America, Europe and Asia will be involved for the enrollment of patients. If necessary, additional sites may be added. Note: No patients were enrolled in Asia.

“Use of this document is governed by the terms of use on the first page of this document.”

LIST OF PROHIBITED MEDICATIONS

The use of the following therapies is prohibited during the study and for at least 28 days prior to initiation of study treatments (unless otherwise specified):

- Investigational or unlicensed/unapproved agents
- Immunotherapy/radio-immunotherapy
- Chemotherapy
- Hormonal anticancer therapy
- Immunostimulatory agent (all patients, including those who discontinue the study early, should not receive other immunostimulatory agents for 10 weeks after the last dose of atezolizumab)
- Radiotherapy (with the exception of limited–field palliative radiotherapy, which can be given any day except on days of study drug administration and one day prior and post study drug administration)
- Biologic agents (e.g., bevacizumab, erlotinib)
- Immunosuppressive medications, including but not limited to cyclophosphamide, azathioprine, methotrexate, and thalidomide; these agents could potentially alter the activity and the safety of atezolizumab. Systemic corticosteroids administered at a dose equal or higher than prednisone 10 mg/day or equivalent (inhaled and topical steroids are permitted).

  Systemic corticosteroids, TNF-α inhibitors, mycophenolate, and other immunosuppressive medications may be administered for the treatment or prevention of *immune-mediated* toxicities at the discretion of the treating physician after consultation with the Medical Monitor.
- Other systemic anti-neoplastic agents and targeted therapies
- Patients must not receive live, attenuated vaccines (such as FluMist®) at any time during the study and up to 5 months after the last dose of atezolizumab. Vaccination with live vaccines is not recommended during treatment and is forbidden 28 days prior to dosing with atezolizumab.
- Initiation or increased dose of granulocyte colony-stimulating factors (e.g., granulocyte colony-stimulating factor, granulocyte/macrophage colony-stimulating factor, and/or pegfilgrastim) is prohibited.

“Use of this document is governed by the terms of use on the first page of this document.”

LIST OF ABBREVIATIONS AND DEFINITIONS OF TERMS

Abbreviation	Definition
ADA	anti-drug antibody
AE	Adverse Events
ALT	Alanine aminotransferase
AML	Acute Myeloid Leukaemia
aPTT	Activated partial thromboplastin time
AST	Aspartate aminotransferase
AUC	Area under the curve
BiTE	bispecific T-cell engager
BLQ	below limit of quantification
BP	Blood Pressure
CA	Competent Authority
CCOD	clinical cut off date
CD3e	CD3 epsilon chain
CD25	late T-cell activation marker
CD45	leukocyte common antigen
CD69	early T-cell activation marker
CDR	Complementarity determining region
CEA	carcinoembryonic antigen
CEACAM	carcinoembryonic antigen-related cell adhesion molecule
CEA TCB (RO6958688)	CEA T-cell bispecific antibody
CKD-EPI equation	Chronic Kidney Disease Epidemiology Collaboration
CL	Clearance
C_{max}	maximum serum concentration
CNS	Central Nervous System
CRC	colorectal cancer
CRO	Contract research organization
CRS	Cytokine release syndrome
CSAP	Clinical statistical analysis plan
CSR	Clinical study report
CT	Computed Tomography
CTCAE	Common Terminology Criteria for Adverse Events
cyCD3e	cynomolgus monkey CD3e
cyCEA	cynomolgus monkey CEA
cyCEA TCB (RO6958690)	cynomolgus monkey homologue of RO6958688
DCR	Disease control rate

“Use of this document is governed by the terms of use on the first page of this document.”

Abbreviation	Definition
DL_{CO}	Diffusing capacity of the lung for carbon monoxide
DLT	Dose-limiting Toxicities
DNA	Deoxyribonucleic acid
EC	Ethics Committee
ECG	Electrocardiogram
ECOG	Eastern Cooperative Oncology Group
eCRF	Electronic Case Report Form
EDC	Electronic data capture
EpCAM	Epithelial cell adhesion molecule
ESF	Eligibility Screening Form
EU	European Commission
EWOC	Escalation with overdose control
Fc	Fc portion of IgG
FcγR	Fc gamma receptor binding to the Fc portion of IgG
FcRn	neonatal Fc receptor
FEV1	Forced expiratory volume in 1 second
FDA	U.S. Food and Drug Administration
[18F]-FDG PET	Fluoro Deoxy Glucose Positron Emission Tomography
FFPE	Formaldehyde fixed-paraffin-embedded
FSH	Follicle stimulating hormone
GCP	Good Clinical Practice
GGT	γ-glutamyl transferase
HBsAG	Hepatitis B surface antigen
HBcAb	Total Hepatitis B core antibody
hCD3e	human CD3e
hCEA	human CEA
HCV	Hepatitis C
HDL	High density lipoproteins
HIPAA	Health Insurance Portability and Accountability Act
HIV	Human immunodeficiency virus
IB	Investigator's Brochure
ICH	International Conference on Harmonisation
ICF	Informed Consent Form
IFNγ	interferon gamma
IgA	Immunoglobulin A
IgE	Immunoglobulin E

"Use of this document is governed by the terms of use on the first page of this document."

Abbreviation	Definition
IgG	Immunoglobulin G
IHC	Immunohistochemistry
IL-2	interleukin-2
IL-6	interleukin-6
IL-8	interleukin-8
IL-10	interleukin-10
IMP	Investigational medicinal product
IND	Investigational New Drug (application)
INR	International normalized ratio
IRB	Institutional Review Board
IRR	infusion-related reaction
irRC	immune–related response criteria
IUD	Intrauterine Device
IV	Intravenous
IxRS	Interactive (voice/web) response system
LDH	Lactate dehydrogenase
LDL	Low density lipoproteins
LH	Luteinizing Hormone
LLOQ	lower limit of quantification
LPLV	Last patient, last visit
MAb	monoclonal antibody
MAD	Multiple Ascending Doses
mCRM	modified continual reassessment method
MoA	mode of action
MRI	Magnetic resonance imaging
MSI-H	Microsatellite instability high
MSS	Microsatellite stable
MTD	Maximal tolerated dose
mUC	Metastatic Urothelial carcinoma
NCI	National Cancer Institute
NK	natural killer
NOAEL	No observed adverse effect level
NOG	NOD/Shi-scid/IL-2Rynull
NSAID	Non-steroideal anti-inflammatory drug
NSCLC	Non-Small Cell Lung Cancer
ORR	overall response rate
PD	Pharmacodynamic

"Use of this document is governed by the terms of use on the first page of this document."

Abbreviation	Definition
PDR	Product Development Regulatory
PFS	Progression-free survival
PK	Pharmacokinetic
PR	Partial response
PS	Performance Status
PT	Prothrombin time
QRS	QRS Complex
QT	QT Interval
QW	once per week
Q3W	every three weeks
RBC	Red Blood Cell
RCR	Roche Clinical Repository
RECIST	Response Evaluation Criteria in Solid Tumors
RNA	Ribonucleic acid
RO6958688 (CEA TCB)	CEA T-cell bispecific antibody
RO6958690 (cyCEA TCB)	cynomolgus monkey homologue of RO6958688
RP2D	Recommended Phase 2 Dose
RR	response rate
SAD	Single Ascending Dose
SAP	Statistical Analysis Plan
SAE	Serious Adverse Event
sCEA	soluble carcinoembryonic antigen
SD	stable disease
SI	SI units; Système International d'Unités
SoA	Schedule of Assessments
$t_{1/2}$	half-life
TB	Tuberculosis
TCB	T-cell bispecific antibody
TCR	T-cell receptor
TCZ	Tocilizumab
TIL	tumor infiltrating lymphocytes
TLC	Total lung capacity
TNFα	tumor necrosis factor alpha
TSH	Thyroid-stimulating hormone
ULN	Upper limit of normal
US	United States
VC	Vital Capacity
WBC	White Blood Cell

"Use of this document is governed by the terms of use on the first page of this document."

1. BACKGROUND AND RATIONALE

1.1 BACKGROUND ON DISEASE

Cancer is the leading cause of death worldwide. Based on the GLOBOCAN estimates, there were 14.1 million new cancer cases, 8.2 million cancer deaths, and 32.6 million people living with cancer (within 5 years of diagnosis) in 2012 worldwide (http://globocan.iarc.fr/Pages/fact_sheets_cancer.aspx#). A large percentage of patients with cancer are diagnosed with advanced disease and are considered to be incurable by surgery and/or radiation. Despite the advances in chemotherapy and targeted therapies, the prognosis of patients with advanced cancer remains poor in general. Consequently, there is a persisting and urgent medical need to develop new therapies that can be added to existing treatments to increase survival without causing unacceptable toxicity.

1.2 BACKGROUND ON IMMUNOTHERAPY

Recent clinical results of immune-based therapies in randomized human studies have shown that immune therapies are valid approaches in cancer therapy. The success of immune therapies, particularly immune checkpoint blocking monoclonal antibodies, has shown that these agents can extend overall survival in patients with cancer and, in some, provide durable responses that few other/existing cancer therapies can approach (Hodi et al. 2010; Honeychurch 2015; Mahoney 2015; Carosella 2015).

Immune-based therapies appear to be a relevant strategy in multiple tumor types, and tumor-specific mutations across tumor types may be an important stimulus of responsiveness to immune-based therapies. Human cancer cells are characterized by a multitude of genetic aberrations (Alexandrov et al. 2013; Lawrence et al. 2013), many of which may be associated with immunogenicity by presenting novel epitopes for immune-cell recognition (Segal et al. 2008). The immune response, largely mediated by tumor-reactive T cells, can be rapid, durable, and adaptable. It has been demonstrated that novel epitopes generated from missense mutations may be the target of tumor-reactive T cells and may mediate response to T cell checkpoint inhibitors (van Rooij et al. 2013). Moreover, the durable responses observed in a subset of patients long after completion of therapy suggest the generation of T cell memory, which has been associated with improved overall survival in patients with cancer (Pages et al. 2005; Kilinc et al. 2009).

Concurrent engagement of the target cell antigen and CD3 leads to activation of polyclonal cytotoxic T cells, resulting in target lysis. Blinatumomab has emerged as a recombinant bispecific T cell engager targeting CD19 and CD3 and has demonstrated remarkable antitumor activity in patients with B-cell malignancies (Steiglmaier 2015). Solitomab, another bispecific antibody construct targeting EpCam and CD3, induced an increased T-cell activation, proliferation, cytokine production and direct tumor cell killing of EpCam positive uterine serous carcinoma cell lines (Bellone 2015). MEDI-565/AMG211, a CEA/CD3 bispecific antibody induced in vitro and in vivo T-cell activation with subsequent killing of human tumor cell lines. The cytotoxicity observed

"Use of this document is governed by the terms of use on the first page of this document."

was CEA dependent and independent of mutations commonly found in colorectal adenocarcinomas (Oberst 2014).

RO6958688 is a novel T- cell bispecific antibody (TCB) that targets carcinoembryonic antigen (CEA) expressed on tumor cells and CD3 epsilon chain (CD3e) present on T cells. In non-clinical models, the binding of RO6958688 to CEA and CD3 resulted in T-cell-mediated killing of the cancerous cells that expressed CEA. RO6958688 effectively mediated the killing of cancer cells only by concurrently binding to CEA present on a tumor cell and to CD3 present on a T cell. RO6958688 is currently being investigated as a single agent in a Phase I study in patients with advanced and/or metastatic CEA expressing tumors.

Current immune-based therapies only lead to durable responses in a proportion of patients with cancer and combination strategies are needed to improve therapeutic efficacy. Programmed death-ligand 1 (PD-L1) is found on the surface of cells in various tumor types and its expression is induced by interferon gamma (IFNγ). It prevents the immune system from destroying cancer cells by interacting with the inhibitory programmed death-1 (PD-1) and B7.1 receptors on activated T cells, which results in a T-cell inhibitory signal (Chen et al. 2012). Blockade of PD-L1/PD-1 pathway increased the proliferation of cytotoxic T cells in a co-culture of T cells along with ovarian cancer and non-small cell lung carcinoma (NSCLC) tumor cells in vitro (Curiel 2003; Zhang 2010). Atezolizumab blocks the interaction of PD-L1 with PD-1 and B7.1. Interruption of the PD-L1/PD-1 and PD-L1/B7.1 interactions represent an attractive strategy to reinvigorate tumor-specific T cell immunity and is currently being investigated in clinical trials as a possible treatment for various cancers (Swaika 2015). Recently it has been shown that blockade of the PD1/PD-L1 pathway augmented the enhancement CD33/CD3 bispecific T-cell engager mediated cytotoxicity in AML (Krupka 2015).

1.3 BACKGROUND ON RO6958688

RO6958688 is a TCB that targets CEA expressed on tumor cells and CD3e chain present on T cells (see Figure 1).

RO6958688 binds with high affinity and in a bivalent-binding mode to human CEA (hCEA; 0.2 nM). The anti-CEA binding domain targets a membrane-proximal domain of hCEA (RO6958688 Investigator’s Brochure), binding specifically to hCEA and does not cross-react with cynomolgus monkey CEA (cyCEA) (RO6958688 Investigator’s Brochure) because the membrane-proximal domain in hCEA is not conserved across species, including cynomolgus monkey. Because CEA is not expressed in rodents, RO6958688 also lacks cross-reactivity with mice and rats. Therefore, alternative nonclinical safety evaluation approaches were considered. This included development of a Cynomolgus monkey cross-reactive homologous (surrogate) antibody (cyCEA TCB; RO6958690) for evaluation in Cynomolgus monkey, and development of double transgenic mice, expressing human CEA and human CD3e (hCEA/hCD3e Tg) as a potential alternative toxicology species. However, both the cyCEA TCB (a surrogate

“Use of this document is governed by the terms of use on the first page of this document.”

molecule) and a hCEA/hCD3e transgenic mouse model were shown to be unsuitable for nonclinical safety testing. An in vitro MABEL approach was therefore used to determine a starting dose in the EIH study (Study BP29541).CH1A1A98/99 × 2F1 does not have any direct functional activity and is not internalized upon binding (RO6958688 Investigator's Brochure). Because of the targeting of a membrane-proximal domain of hCEA, CH1A1A98/99 × 2F1 displays preferential binding to membrane-anchored CEA rather than shed, soluble CEA (sCEA) (RO6958688 Investigator's Brochure), and the killing potency of RO6958688 remains unaffected up to 0.2 μg/mL of sCEA (RO6958688 Investigator's Brochure).

RO6958688 also binds to T cells through its second binding unit-targeting CD3e of the T-cell receptor (TCR) complex (de la Hera et al. 1991; RO6958688 Investigator's Brochure). The anti-CD3 antibody used in RO6958688 cross reacts with human CD3e (hCD3e) and cynomolgus monkey CD3e (cyCD3e) chains but not with mouse CD3e chain. This antibody has been generated in house by humanization of the parental SP34 antibody (Pessano et al. 1985; Salmerón et al. 1991; Conrad et al. 1991; RO6958688 Investigator's Brochure). The binding to CD3e is monovalent, which prevents activation of T cells in the absence of simultaneous binding to tumor cells that express CEA, and has low affinity for both hCD3e and cyCD3e (80 nM). The differential binding affinity between CEA and CD3 may favor preferential targeting of RO6958688 to tumors and reduce the peripheral sink due to binding to T cells.

RO6958688 is a human immunoglobulin G1 (IgG1) with the Fc region bearing a novel, proprietary modification (P329G LALA mutation; Figure 1) that abrogates its binding in vitro to Fc γ receptors (FcγR) (RO6958688 Investigator's Brochure) and prevents FcγR-mediated co-activation of innate immune effector cells, including natural killer (NK) cells, monocytes/macrophages, and neutrophils, without changes in functional binding to neonatal Fc receptor, also called FcRn (RO6958688 Investigator's Brochure).

Simultaneous binding of RO6958688 to CEA and CD3 leads to T-cell activation and tumor cell lysis. The RO6958688-mediated tumor cell lysis is CEA-specific and does not occur in the absence of CEA expression or in the absence of simultaneous binding (cross-linking) of T cells to CEA-expressing tumor cells (RO6958688 Investigator's Brochure). In addition to killing, T cells undergo activation followed by tumor lysis as detected by increase of late and early T-cell activation markers (CD25 and CD69, respectively), cytokine release (interferon γ [IFNγ], tumor necrosis factor α [TNFα], granzyme B, interleukin [IL]-2, IL-6, IL-10), and proliferation of T cells (RO6958688 Investigator's Brochure).

"Use of this document is governed by the terms of use on the first page of this document."

Figure 1 Design, Structure, and Characteristics of RO6958688

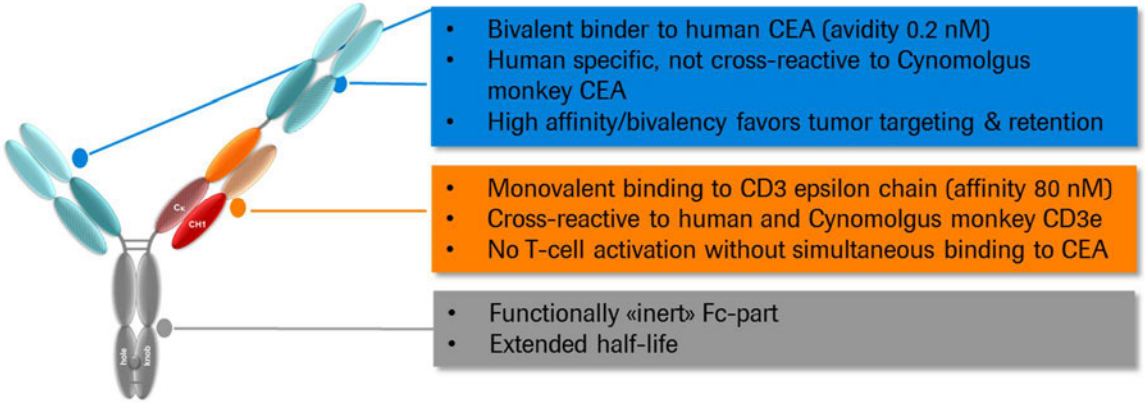

CEA = carcinoembryonic antigen; Fc = Fc portion of IgG; IgG1 = human immunoglobulin G1.
Note: Blue parts correspond to the high-affinity binding antibody (CH1A1A98/99 × 2F1) to hCEA. The red part corresponds to the humanized antibody (CH2527 [VL_7-46(13) VH_23-3(12)]) that binds to the CD3e chain. The grey parts correspond to the heterodimeric Fc region of the human IgG1 bearing the P329G LALA mutation.

1.3.1 Previous Non-Clinical Studies

The anti-tumor activity upon combination of RO6958688 with the anti-PD-L1 blocking antibody was assessed in vitro and in vivo.

RO6958688-mediated tumor lysis leads to upregulation of PD-1 on human T cells and of PD-L1 on surviving tumor cells - in vitro

The lysis of CEA-expressing MKN-45 target cells mediated by RO6958688 was assessed after 24 h and 48 h of incubation with human PBMCs (E:T 10:1, LDH release), Figure 2 (A 24 h, B 48 h).

"Use of this document is governed by the terms of use on the first page of this document."

Figure 2 RO6958688-Mediated Lysis of MKN45 Cells

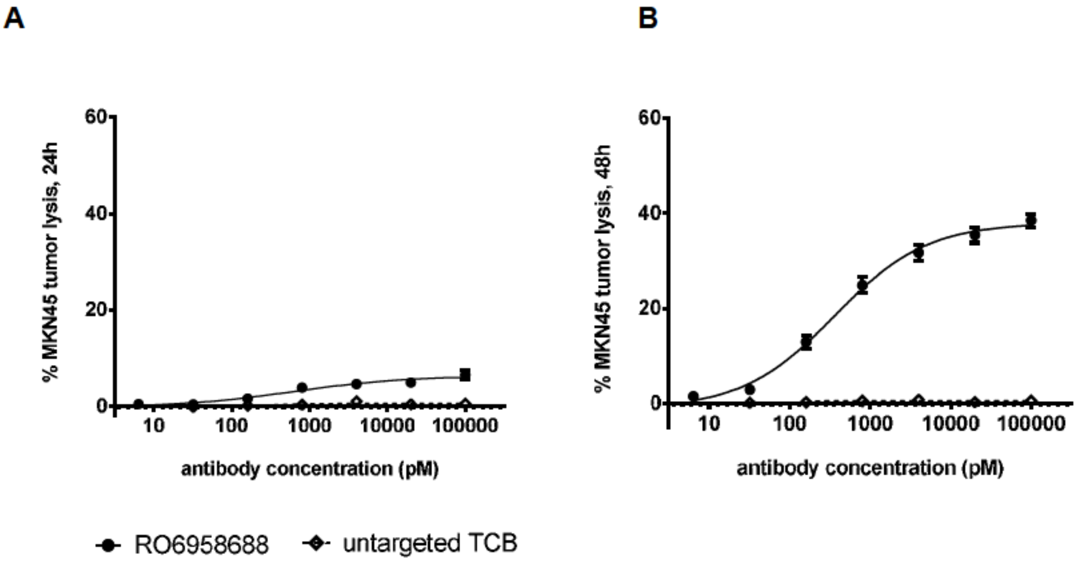

Representative graphs of RO6958688-mediated lysis of MKN-45 tumor cells assessed (A) 24 hours and (B) 48 hours after incubation of tumor cells with human PBMCs (E:T 10:1). The EC50 values of tumor cell killing are: 615 pM (24 h), 362 pM (48 h). Target cell killing was assessed by quantification of LDH released into cell supernatants.

Following killing, the surface expression of PD-1 receptor (on CD4+ or CD8+ T cells) and of PD-L1 (on tumor cells that survived killing) was assessed by flow cytometry. Figure 3 displays a dose-dependent upregulation of PD-1 receptor on CD8+ (A) and on CD4+ (B) T cells as well as of PD-L1 on tumor cells that resisted to killing (C) and were harvested after 48 h of incubation.

"Use of this document is governed by the terms of use on the first page of this document."

Figure 3 RO6958688 Upregulation of PD-1 on T cells, Respective of PD-L1 on Surviving Tumor Cells after Tumor Cell Lysis

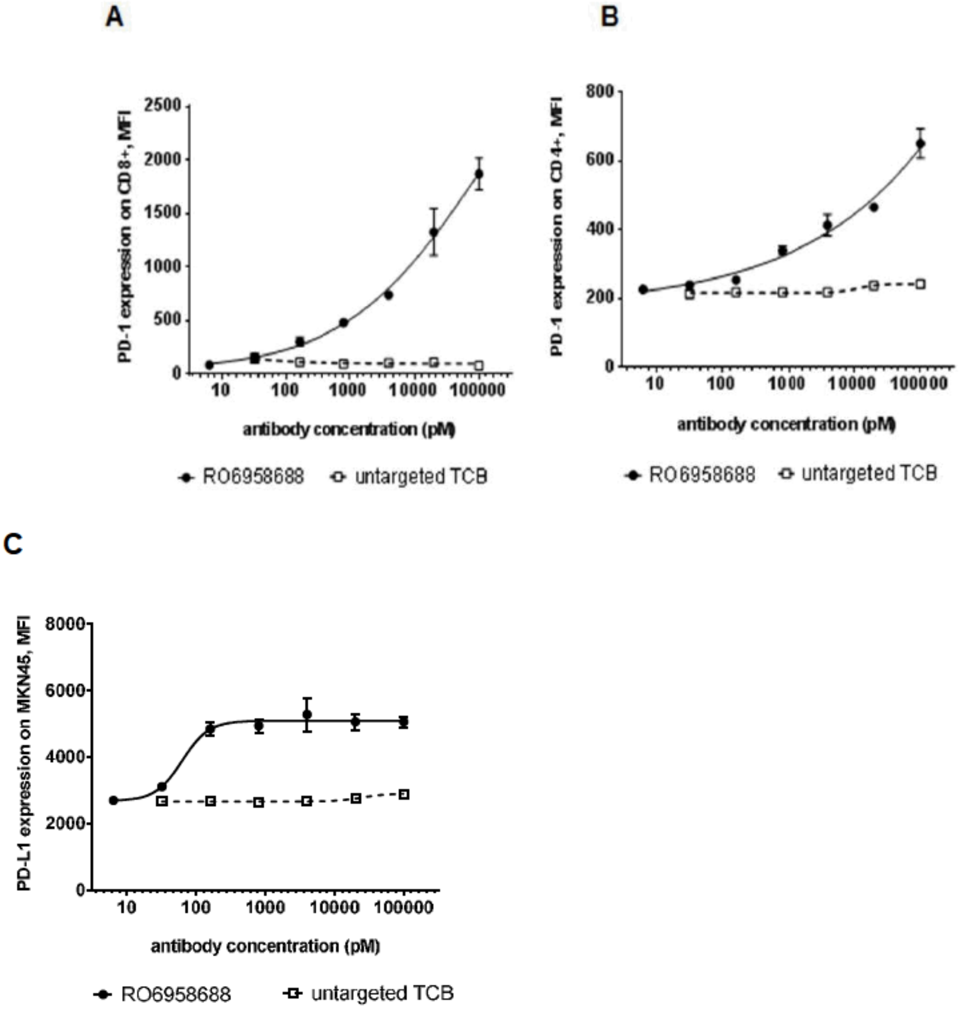

Representative graphs of RO6958688-mediated upregulation of PD-1 receptor expression on CD8+ T cells (C), CD4+ T cells (D), and of PD-L1 ligand expression on tumor cells that resisted killing (C) analyzed 48 h post incubation of MKN-45 tumor cells with human PBMCs (E:T 10:1, as in panel B). PD-1 and PD-L1 expression was analyzed by flow cytometry.

“Use of this document is governed by the terms of use on the first page of this document.”

RO6958688-mediated tumor lysis leads to upregulation of PD-1 on human T cells and of PD-L1 on surviving tumor cells - in vivo

Please refer to RO6958688 IB Section 4.1.3.

- In vivo studies – Xenograft Experiment with Co-Grafting of Effector Cells – PD-L1 upregulation in RO6958688-treated tumors as compared to vehicle, assessed by IHC
- In vivo studies – Xenograft Experiment with IP Transfer of Effector Cells –Tumor panel showing PD-1 upregulation on intra-tumor T cells upon RO6958688 treatment as compared to vehicle, assessed by flow cytometry and PD-L1 upregulation in RO6958688-treated tumors as compared to vehicle, assessed by IHC
- In vivo studies – Xenograft Experiment in Fully Humanized Mice – Increase of the percentage of PD-1-expressing intra-tumor CD8+ T cells and CD4+ T cells upon treatment with RO6958688 as compared to vehicle, assessed by flow cytometry; Anti-PD-L1 staining of vehicle and RO6958688-treated tumors collected at study termination denoting strong induction of intra-tumor PD-L1 expression upon RO6958688-treatment.

Assessment of in vivo anti-tumor activity upon combination of RO6958688 with the anti-PD-L1 blocking antibody

The anti-tumor efficacy of RO6958688 in combination with the anti-human PD-L1 blocking antibody (clone YW243.55.S70 re-synthesized in house with murine Fc) assessed in fully humanized mice bearing the gastric carcinoma tumor cell line (MKN45).

In summary, 1 x10^6 MKN45 tumor cells were injected subcutaneously in fully humanized NOG mice. 7 days after tumor cell injection, mice were randomized in four groups: the first group received phosphate-buffer saline (PBS, vehicle) as control, the second group received RO6958688 (at the dose of 2.5 mg/kg, administered twice a week for 7 weeks, 15 administrations in total), the third group received a-PD-L1 (at the dose of 10 mg/kg, administered once a week for 7 weeks, 8 administrations in total), and the fourth group received a combination of RO6958688 and a-PD-L1, administered concomitantly with the respective dose and schedules used in the single therapeutic groups for 7 weeks (8 administrations of a-PD-L1 10 mg/kg given once a week and 15 administrations of RO6958688 2.5 mg/kg given twice a week).

The results show a significant increase in anti-tumor activity upon combination of RO6958688 with anti-PD-L1 blocking antibody as compared to RO6958688 single agent. Treatment with a-PD-L1 as single agent doesn't show any anti-tumor activity. No overt test item-related toxicities were observed following treatment with the combination (Roche Report No. 1066291).

"Use of this document is governed by the terms of use on the first page of this document."

Figure 4 In Vivo Anti-Tumor Activity upon Combination of RO6958688 with the Anti-Human PD-L1 Blocking Antibody in MKN45 Tumor Model in Fully Humanized Mice

Average tumor burden and standard error of mean (SEM). Tumor burden was measured by digital caliper 3 times a week. Blue arrow indicates the day of start of therapy. At day 60: n=7 (vehicle), n=8 (RO6958688); n=4 (a-PD-L1); n=5 (combination RO6958688 with a-PD-L1). a-PD-L1 antibody = clone YW243.55.S70 re-synthesized in house with murine Fc.

A

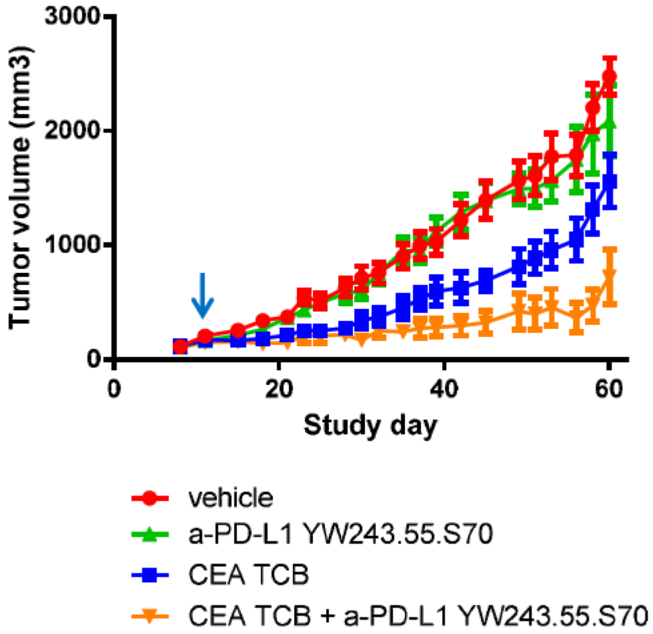

"Use of this document is governed by the terms of use on the first page of this document."

Figure 4 In vivo anti-tumor activity upon combination of RO6958688 with the anti-human PD-L1 blocking antibody in MKN45 tumor model in fully humanized mice. (cont.)

B

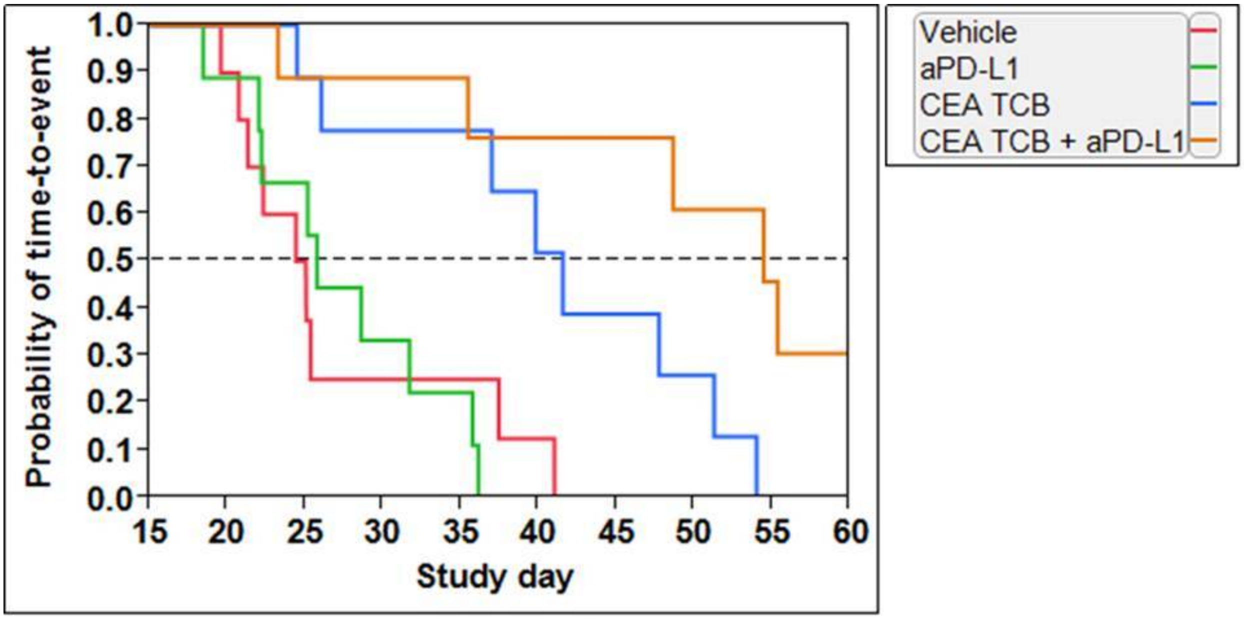

Kaplan-Meier curves give an estimation of the survival functions for one or more groups of right-censored data.

Time-to-event statistical analysis. The event was defined as reaching 500 mm^3 tumor volume. The pairwise log-rank test was used to compare the following treatment groups, as it can take the different drop-outs into account: vehicle vs RO6958688: p= 0.005; RO6958688 vs. a-PD-L1: p= 0.001; RO6958688 vs combination of RO6958688 with a-PD-L1: p= 0.03.

The pharmacology, pharmacokinetics, and in vitro toxicology of RO6958688 and cyno-cross reactive surrogate antibody (cyCEA TCB) have been investigated in several nonclinical studies. RO6958688 contains the high-affinity anti-CEA antibody (CH1A1A 98-99 x 2F1), which binds to hCEA and does not cross-react with Cynomolgus monkey CEA. Furthermore, CEA is not expressed in rodents, and the CD3e antibody in RO6958688 does not cross-react with mouse CD3e. Therefore, alternative nonclinical safety evaluation approaches, as described in the ICH S6 Guideline, were considered. This included development of a Cynomolgus monkey cross-reactive homologous (surrogate) antibody (cyCEA TCB; RO6958690) for evaluation in Cynomolgus monkey – and development of double transgenic mice, expressing human CEA and human CD3e (hCEA/hCD3e Tg) as a potential alternative toxicology species (RO6958688 IB).

However, both the cyCEA TCB (a surrogate molecule) and a hCEA/hCD3e transgenic mouse model were shown to be unsuitable for nonclinical safety testing. An in vitro

“Use of this document is governed by the terms of use on the first page of this document.”

MABEL approach was therefore used to determine a starting dose in the EIH study, instead of an in vivo approach (i.e., NOAEL approach) (RO6958688 IB).

A human whole blood cytokine release assay was performed to assess the risk of cytokine-mediated infusion-related reactions (IRRs). The data indicated a risk for cytokine-mediated events particularly at 100 nM (a concentration estimated to be reached at C_{max} with a dose of 58 mg), but also at lower concentrations in some donors. The potency of RO6958688 to induce cytokines is less than that of the CD3 antibody muromonab-CD3, where responses were observed starting at 10 nM.

In addition, a tissue cross-reactivity study with RO6958688 in normal human tissues, and an assessment of blood compatibility and hemolysis were performed (details of which can be found in the RO6958688 IB).

1.3.2 Previous Clinical Studies

RO6958688 is being investigated as a single agent in patients with locally advanced and/or metastatic solid tumors expressing CEA in Study BP29541 (first patient dosed Dec 30, 2014). The main purpose of this EIH study is to describe the safety profile of the single agent RO6958688 when given QW and to establish its MTD and/or recommended dose.

BP29541 is being conducted in 2 parts: Part I was a single ascending dose part in single-patient cohorts to evaluate the safety of RO6958688 at doses up to 2.5 mg. Part II is a multiple ascending dose part with RO6958688 as single agent given QW.

Refer to the RO6958688 Investigator's Brochure for additional information on the clinical development and clinical safety of RO6958688.

1.3.2.1 Safety of RO6958688

1.3.2.1.1 BP29541

At the clinical cutoff date (CCOD) of 27 April 2018 a total of 148 patients have been enrolled and have received at least one dose of either RO6958688 and/or obinutuzumab in Study BP29541. In Study BP29541 on average patients received 10.3 doses/cycles of RO6958688, with a median of 6.0 doses/cycles equating to an average of 76.03 days of treatment with RO6958688 per patient. Overall, adverse events (AEs) were reported in 145 of 148 patients (98.0%). The most common AE reported in ≥25 % of patients was IRR (105 patients [70.9%]), followed by pyrexia (78 patients [52.7%]), diarrhea (67 patients [45.3%]), nausea (53 patients [35.8%]), vomiting and anemia (47 patients [31.8%] each), asthenia (44 patients [29.7%]) and decreased appetite (43 patients [29.1%]). AEs considered related to study treatment by the investigator were reported in 138 of 148 patients (93.2%), with RO6958688–related AEs reported in 137 of 142 patients [96.5%], obinutuzumab-related AEs reported in 8 of 33 patients (24.3%), and AEs related to both RO6958688 and obinutuzumab reported in 3 of 27 patients (11.1%).

"Use of this document is governed by the terms of use on the first page of this document."

A total of 99 of 148 patients (66.9%) reported at least one Grade ≥3 AE with 86 patients (58.1%) reporting an AE with maximum Grade 3 intensity, and 7 patients (4.7%) reporting AEs with maximum Grade 4 intensity. Six Grade 5 AEs were reported: one RO6958688-related event of respiratory failure (600 mg RO6958688), one RO6958688-related event of dyspnea (40 mg RO6958688), one RO6958688-related event of IRR (Cohort C: 356 mg RO6958688), one obinutuzumab-related event of sepsis (60 mg RO6958688 Q3W + obinutuzumab), one unrelated cardio-respiratory arrest (Cohort A: 40 mg RO6958688) and one event of unrelated tumor thrombosis (2.5 mg RO6958688).

Serious adverse events (SAEs) were reported in 96 of 148 patients (64.9%) of which IRR (40 patients [27.0%]) was the most frequently reported, followed by pyrexia (8 patients [5.4%]), nausea, pneumonia, dyspnea, tumor pain, and acute kidney injury (5 of 148 patients [3.4%] each). SAEs assessed by the investigator as related to study treatment were reported in 66 of 148 patients (44.6%).

Ninety-four deaths (63.5%) were reported at the CCOD, of which 83 deaths (56.1%) were due to disease progression. There were 6 deaths due to AEs, 4 deaths (2.7%) due to unknown causes (which were confirmed as disease progression following the CCOD), and 1 death was due to sepsis.

Seven DLTs related to RO6958688 have been reported in 7 of 148 patients (4.7%) in Part II only at the time of the clinical cutoff date: Grade 3 dyspnea (40 mg RO6958688; 1 patient), Grade 3 hypoxia (60 mg RO6958688; 1 patient), Grade 3 diarrhea (300 mg RO6958688; 1 patient), Grade 3 colitis (300 mg RO6958688 with obinutuzumab pretreatment; 1 patient) and Grade 4 colitis (600 mg RO6958688; 1 patient), Grade 5 respiratory failure (600 mg RO68958688; 1 patient), and Grade 5 dyspnea (40 mg in Cohort B; 1 patient). The Grade 5 event of dyspnea (40 mg in Cohort B; 1 patient) occurred after the dose escalation part of the trial and was not used to derive the maximum tolerated dose (MTD).

The risk mitigation measures were further optimized following the receipt of two Grade 5 events reported as related to RO6958688 (Grade 5 IRR in BP29541 in this section and Grade 5 Hypovolemic Shock in WP29945 in Section 1.3.2.1.2).

1.3.2.1.2 WP29945

As of the CCOD of 27 April 2018, a total of 226 patients have been enrolled and have received at least one dose of either RO6958688 or atezolizumab in Study WP29945 with all patients reporting at least one AE. In Study WP29945 on average patients received 9.5 doses/cycles of RO6958688, with a median of 5 doses/cycles equating to an average of 81.11 days of treatment with RO6958688 per patient. The most common AEs reported in ≥25% patients were IRR (156 of 226 patients [69.0%]), diarrhea (136 patients [60.2%]), pyrexia (120 patients [53.1%]), dysgeusia (81 patients [35.8%]), fatigue (75 patients [33.2%] each), chills (72 patients [31.9%]), vomiting (71 patients [31.4%]), nausea (70 patients [31.0%]), asthenia (69 patients [30.5%]), and decreased

"Use of this document is governed by the terms of use on the first page of this document."

appetite (60 patients [26.5%]). AEs considered related to study treatment by the investigator were reported in 222 of 226 patients (98.2%) with RO6958688–related AEs reported in 222 patients (98.2%), atezolizumab-related AEs reported in 183 patients (81.0%) and AEs related to both RO6958688 and atezolizumab reported in 179 patients (79.2%).

A total of 141 of 226 patients (62.4%) reported at least one ≥Grade 3 AE with 122 patients (54.0%) reporting an AE with maximum Grade 3 intensity, and 14 patients (6.2%) reporting an AE with maximum Grade 4 intensity. Five patients (2.2%) experienced a Grade 5 event: one event of hypovolemic shock related to RO6958688 (B1 cohort 600 mg RO6958688), one event of unrelated respiratory tract infection (300 mg RO6958688), one event of unrelated urinary tract infection (Safety cohort pancreatic cohort 600 mg RO6958688), one event of unrelated disseminated intravascular coagulation (DIC; 160 mg RO6958688), and one event of unrelated cerebrovascular accident (80 mg RO6958688).

SAEs were reported in 139 of 226 patients (61.5%) of which IRR (65 patients [28.8%]) was the most frequently reported SAE followed by pyrexia (17 patients [7.5%]) and diarrhea (10 patients [4.4%]). SAEs considered related to study treatment by the investigator were reported in 113 of 226 patients (50.0%).

Fifty-four patients had died (23.9%) at the CCOD, of which 48 deaths (21.2%) were due to progression of disease. There were 5 deaths due to an AE. The remaining death was due to an unknown cause at the time of survival follow-up.

As of the CCOD, 14 DLTs as defined in the protocol have been reported in 12 of 226 patients (5.3%). The following events were considered related to both RO6958688 and atezolizumab: Grade 4 cardiac arrest (100 mg RO6958688; 1 patient), Grade 4 dyspnea (160 mg RO6958688; 1 patient), 2 events of Grade 3 colitis (100 mg and 160 mg RO6958688; 2 patients), 2 events of Grade 3 ALT increased (150 mg and 160 mg RO6958688; 2 patients), Grade 3 AST increased (150 mg RO6958688; 1 patient), Grade 3 stomatitis (40mg RO6958688; 1 patient). The following events were considered related to RO6958688 only: Grade 5 hypovolemic shock (600 mg RO6958688; 1 patient), Grade 4 platelet count decreased (300 mg RO6958688; 1 patient), Grade 3 dyspnea (100 mg RO6958688; 1 patient), Grade 3 rash maculo-papular (120 mg RO6958688; 1 patient; incomplete 160 mg administration), Grade 1 diarrhea (150 mg RO6958688; 1 patient), and Grade 1 dysgeusia (100 mg RO6958688; 1 patient).

A Grade 5 event of hypovolemic shock related to RO6958688 was reported in a [redacted] year-old [redacted] patient with microsatellite unstable mCRC and hepatic, pulmonary and peritoneal lesions enrolled into the step-up cohort B1 (atezolizumab 1200 mg Q3W; RO6958688 40 to 1200 mg QW, followed by 1200 mg Q3W) in the WP29945 study. The patient had received two lines of prior standard therapy. Co-morbidities included

“Use of this document is governed by the terms of use on the first page of this document.”

ongoing hypertension and diabetes mellitus with history of [redacted] and [redacted]. The patient started the first cycle of RO6958688 + atezolizumab on [redacted]. The first 3 administrations (40-150-300 mg) of RO6958688 were associated with adverse events as well as lab abnormalities including constitutional, gastrointestinal, pulmonary symptoms and increased creatinine, liver function tests, hypoalbuminemia and thrombocytopenia. Due to these adverse events, the second cycle (fourth dose of RO6958688) was started with 4 weeks' delay on [redacted], consisting of 1200 mg atezolizumab and 600 mg RO6958688. Approximately 8 hours after the RO6958688 infusion, the patient developed several symptoms led by severe continuous diarrhea, partially responsive to corticosteroid treatment, with subsequent hypovolemic shock, hypotension, hypoxia, metabolic acidosis and renal failure leading to hyperkalemia. These symptoms initially improved with steroids and supportive care in the ICU. However, on [redacted], the patient experienced ventricular fibrillation, secondary to hyperkalemia per investigator, with successful electric cardioversion, but could not be resuscitated after the 2nd episode of ventricular fibrillation despite intensive care measures. This event was reported by the Investigator as a G5 hypovolemic shock related to RO6958688.

The risk mitigation measures were further optimized (in Section 5.2.6) following the receipt of two Grade 5 events reported as related to RO6958688 (Grade 5 IRR in BP29541 in Section 1.3.2.1.1 and Grade 5 Hypovolemic Shock in WP29945 in this section).

1.3.2.1.3 Additional Safety Findings: Infusion-Related Reactions and Cytokine Release Syndrome

As of the CCOD of 27 April 2018, IRRs were reported as a stand-alone preferred term in 105 of 148 patients (70.9%) in Study BP29541 and in 156 of 226 patients (69.0%) in Study WP29945. The majority was of Grade 1 or Grade 2 severity and readily manageable with protocol-recommended measures. However, 29 patients (19.6%) in Study BP29541 and 39 patients (17.3%) in Study WP29945 reported an IRR with a maximum intensity of Grade 3, and 1 patient in Study BP29541 experienced a Grade 5 IRR). Forty patients (27.0%) in Study BP29541 and 65 patients (28.8%) in Study WP29954 reported at least one serious IRR.

The incidence of IRR was highest at the first infusion. At this timepoint, the majority of patients were anti-drug antibody (ADA) negative and a comparable dose-dependent cytokine release was observed in ADA-negative patients and those who went on to become ADA positive (see Section 1.3.2.4). The cytokine release that is observed after the first infusion is expected based on the mechanism of action of RO6958688. At the second and subsequent infusions, the incidence of IRR decreased.

"Use of this document is governed by the terms of use on the first page of this document."

IRRs were also more frequent and of higher severity in ADA-positive patients compared with ADA-negative patients (median time to ADA onset is 2.3 weeks). IRRs in ADA-positive patients were more likely to be associated with clinical symptoms of cytokine release syndrome (CRS) (e.g., hypoxia and/or hypotension, as observed in the patient from Study BP29541 who experienced a grade 5 IRR). After the fourth infusion of RO6958688, cytokine release remained dose dependent and was higher in ADA-positive patients.

While cytokine peaks observed during initial administrations are thought to represent on-target, tumor-related effects, later cycle peaks likely represent T-cell activation by ADA-mediated crosslinking of CEA-TCB bound CD3.

Given that IRRs may be indistinguishable from CRS based on symptomatology, single-treatment management guidelines are being recommended for both IRRs and CRS, during or up to 24 hours after infusion of RO6958688. These include the use of tocilizumab as a rescue medication for CRS. See Section 5.2.6.2.4 for more information on IRR/CRS and Appendix 10 for management guidelines.

Please refer to the RO6958688 Investigator's Brochure for additional details on IRR/CRSs adverse events observed in clinical studies.

1.3.2.2 Efficacy of RO6958688

1.3.2.2.1 BP29541

As of 16 June 2017, tumor response assessment data using RECIST criteria v1.1 were available from 86 of 102 patients (84.3%) in Study BP29541. Of the 86 efficacy-evaluable patients, 5 patients treated with 0.052-2.5 mg RO6958688 QW were enrolled in Part I and 81 patients were enrolled in Part II of which 63 patients received flat doses of 2.5 mg-600 mg RO6958688 QW alone, 8 patients received step-up doses of 40-1200 mg RO6958688 QW (Cohorts A and B), and 10 patients received 135-300 mg RO6958688 QW following obinutuzumab pretreatment. Tumor responses were evaluated 8-12 weeks after treatment or at any unscheduled tumor response assessment by using RECIST criteria v1.1. Efficacy evaluable patients had at least one administration of RO6958688 and at least one tumor assessment after treatment start.

Partial response was observed in a total of 2 of 86 patients (2.3%) with CRC. One of 35 patients (2.9%) in the 60-600 mg RO6958688 dose group received a starting dose of 200 mg RO6958688 QW and was dose escalated to 300 mg with the longest PR observed at Day 94 (unscheduled visit following Cycle 8) accompanied by a 68% reduction of target lesions and 1 of 10 patients (10.0%) received obinutuzumab pretreatment followed by RO6958688 QW at a starting dose of 135 mg and was dose-escalated to 200 mg with the longest PR observed at Day 225 (Cycle 32) accompanied by a 61% reduction of target lesions. Progressive disease has been confirmed in both patients, after response durations of 94 and 224 days, respectively.

"Use of this document is governed by the terms of use on the first page of this document."

Stable disease was observed as best overall response in a total of 30 of 86 (34.9%) patients which included 1 of 5 patients (20.0%) in Part I who received RO6958688 QW at a starting dose of 0.15 mg and escalated to 20 mg, 23 of 63 patients (36.5%) in Part II who received flat doses of 2.5-600 mg RO6958688 QW with 10 of 28 patients (35.7%) treated with 2.5-40 mg RO6958688 and 13 of 35 patients (37.1%) treated with 60-600 mg RO6958688, 3 of 10 patients (30.0%) in Part II who received 135-300 mg RO6958688 QW following obinutuzumab pretreatment, and 3 of 5 patients (60.0%) in Cohort A in Part II who received step-up doses of RO6958688 QW starting at 40 mg up to 1200 mg. The longest SD was at Day 441 (Cycle 64) observed in a patient dosed with RO6958688 QW at 60 mg and escalated up to 400 mg. This patient continued to have SD at the time of clinical cutoff date.

Forty-four patients (50.6%) had progressive disease as best overall response and 10 (11.6%) patients had missing or non-evaluable responses.

As of 14 July 2017, there were 2 confirmed partial responses (PR) by RECIST 1.1 out of 31 CRC MSS patients (7%) enrolled in the ≥ 60 mg QW RO6958688 and step up cohorts A and B. Two additional PR responses were confirmed after this latter cutoff date.

Please refer to the RO6958688 Investigator's Brochure for additional details on the efficacy of RO6958688.

1.3.2.2.2 WP29945

As of 16 June 2017, tumor response assessment data using RECIST criteria v1.1 were available for a total of 61 of 82 patients (74.4%) in Study WP29945. Of the 61 efficacy-evaluable patients, 2 patients received previous RO6958688 treatment in Study BP29541 prior to Cycle 1 Day 1 combination treatment of RO6958688 and atezolizumab; data from these 2 patients were analyzed and are presented separately. Efficacy evaluable patients received at least one dose of RO6958688 in combination with at least one dose of atezolizumab and had at least one tumor assessment after treatment start. Tumor responses were evaluated 8 weeks after treatment with 5–300 mg RO6958688 QW in combination with a fixed dose 1200 mg of atezolizumab Q3W or at any unscheduled tumor response assessment.

Of the 59 efficacy-evaluable patients not treated previously with RO6958688, PR was achieved as best overall response in 5 of 44 patients (11.4%) in the 80-300 mg RO6958688 dose group. All 5 patients received 160 mg RO6958688 QW in combination with 1200 mg atezolizumab Q3W. The longest PR was observed at Day 225 (unscheduled visit following Cycle 9) accompanied by 69% reduction of target lesions compared to baseline, and is ongoing at the time of clinical cutoff with a duration of response of 168 days. Additionally, 2 of these 5 patients have ongoing responses of 28 and 87 days duration at the time of clinical cutoff, respectively. The remaining 2 patients achieved a PR followed by progression of disease after 118 and 58 days, respectively.

“Use of this document is governed by the terms of use on the first page of this document.”

A total of 25 of 59 patients (42.4%) had SD as best overall response: 4 of 15 patients (33.3%) treated with 5-40 mg RO6958688 and 21 of 44 patients (47.7%) treated with 80-300 mg RO6958688 in combination with atezolizumab. The longest SD was observed at Day 337 (Cycle 17) in a patient who received RO6958688 QW at starting dose of 20 mg and escalated up to 160 mg in combination with atezolizumab. Twenty-six patients (44.1%) had progressive disease as best overall response and 3 patients (5.1%) had missing or non-evaluable responses.

Two patients with CRC who received previous RO6958688 treatment until disease progression in Study BP29541 prior to enrollment in Study WP29945, had SD as the best overall response following combination treatment of atezolizumab with escalating RO6958688 doses from 40 mg to 160 mg (1 patient) and a RO6958688 dose of 160 mg (1 patient) in Study WP29945.

As of 14 July 2017, there were 4 confirmed partial responses (PR) by RECIST 1.1 out of 31 CRC MSS patients (13%) enrolled in the 160 mg QW RO6958688 cohort. An additional PR response was confirmed after this latter cutoff date.

As of 11 January 2018, there were 5 confirmed partial responses (PR) by RECIST 1.1 out of 37 CRC MSS patients (13%) enrolled in the 160 mg QW RO6958688 cohort. In the 100 mg RO6958688 QW cohort, there were 2 confirmed partial responses (PR) by RECIST 1.1 out of 20 CRC MSS patients (10%) enrolled and 3 confirmed partial responses out of 19 CRC MSS patients (15.8%) enrolled in the 100 mg RO6958688 Q3W RO6958688 cohort. Disease control rate in the 160 mg QW RO6958688 cohort was 51.4%, in the 100 mg RO6958688 QW cohort the disease control rate was 55% and in the 100 mg Q3W RO6958688 cohort was 52.6%.

Please refer to the RO6958688 Investigator's Brochure for additional details on the efficacy of RO6958688.

1.3.2.3 Clinical Pharmacokinetics of RO6958688

As of 14 May 2018, PK data from a total of 65 ADA-negative patients from Study BP29541 and 80 ADA-negative patients from Study WP29945 were included in a population PK analysis. RO6958688 concentration time profiles exhibited a biphasic disposition with an initial rapid distribution phase followed by a slower elimination phase. In ADA–negative patients, PK was time independent, i.e., serum exposure was maintained after multiple doses. The median CL and V_{ss} values were 0.071 L/h and 0.059 L/h, 9.8 L and 9.4 L, respectively, in Studies BP29541 and WP29945.

RO6958688 CL does not depend on dose. Similarly, after the first infusion, maximum concentration (C_{max}) and area under the concentration time curve (AUC) values of the first dosing interval are dose proportional, i.e., RO6958688 exhibited linear pharmacokinetics.

“Use of this document is governed by the terms of use on the first page of this document.”

1.3.2.4 Immunogenicity of RO6958688

As of 7 June 2018, ADA data from 132 patients from Study BP29541 and 193 patients from WP29945 were available, while PK data of 142 and 226 patients were available. In ADA-positive patients, time dependent PK can occur after multiple IV infusions of RO6958688 with reduced or no detectable exposure.

In patients treated with RO6958688 as a single agent and without obinutuzumab pretreatment (Study BP29541) and RO6958688 in combination with atezolizumab (Study WP29945), the development of ADAs directed against RO6958688 was observed in 48% (51 of 106 patients) and 64% (123 of 193 patients), respectively; RO6958688 exposure was reduced below the limit of quantification at C_{max} in 26% (30 of 115 patients) and 16% (35 of 226 patients) of patients, respectively. Median time to onset of no detectable exposure was 5.0 and 7.1 weeks in Studies BP29541 and WP29945, respectively.

After obinutuzumab pretreatment in Study BP29541, 42% (11 of 26 patients) of patients were ADA-positive; of these, 9 of 11 patients (81.8%) had transient ADAs and in the remaining 2 patients the last available ADA sample was early, i.e., at 3 and 9 weeks after the first dose of study drug (RO6958688). The maximal observed ADA-titer of 270 in obinutuzumab pretreated patients was low as compared to 196,830 in non obinutuzumab pretreated patients and RO6958688 exposure was sustained with similar PK profiles to those in ADA-negative patients.

More detailed information can be found in the RO6958688 Investigator's Brochure.

1.4 BACKGROUND ON ATEZOLIZUMAB

Atezolizumab is a humanized immunoglobulin (Ig) G1 monoclonal antibody that targets PD-L1 and inhibits the interaction between PD-L1 and its receptors, PD-1 and B7-1 (also known as CD80), both of which function as inhibitory receptors expressed on T cells. Therapeutic blockade of PD L1 binding by atezolizumab has been shown to enhance the magnitude and quality of tumor specific T cell responses, resulting in improved anti-tumor activity (Fehrenbacher et al. 2016; Rosenberg et al. 2016). Atezolizumab has minimal binding to Fc receptors, thus eliminating detectable Fc effector function and associated antibody-mediated clearance of activated effector T cells.

Atezolizumab shows anti-tumor activity in both nonclinical models and cancer patients and is being investigated as a potential therapy in a wide variety of malignancies. Atezolizumab is being studied as a single agent in the advanced cancer and adjuvant therapy settings, as well as in combination with chemotherapy, targeted therapy, and cancer immunotherapy

"Use of this document is governed by the terms of use on the first page of this document."

Targeting the PD-L1 pathway with atezolizumab has demonstrated activity in patients with advanced malignancies who have failed standard-of-care therapies. Objective responses have been observed across a broad range of malignancies, including NSCLC, urothelial carcinoma, RCC, melanoma, colorectal cancer, head and neck cancer, gastric cancer, breast cancer, and sarcoma.

Atezolizumab is approved for the treatment of locally advanced or metastatic urothelial carcinoma, metastatic non-small cell lung cancer, *small-cell lung cancer, and triple-negative breast cancer*.

Refer to the Atezolizumab Investigator's Brochure for details on nonclinical and clinical studies.

1.4.1 Previous Non-Clinical Studies

The pharmacology, pharmacokinetics, and toxicology of atezolizumab have been investigated in several nonclinical studies. Comprehensive pharmacology, PK, and toxicology evaluations were performed with atezolizumab. The safety, pharmacokinetics, and toxicokinetics of atezolizumab were investigated in mice and cynomolgus monkeys to support IV administration and to aid in projecting the appropriate starting dose in humans. Given the similar binding of atezolizumab for cynomolgus monkey and human PD-L1, the cynomolgus monkey was selected as the primary and relevant nonclinical model for understanding the safety, pharmacokinetics, and toxicokinetics of atezolizumab. Overall, the nonclinical pharmacokinetics and toxicokinetics observed for atezolizumab supported entry into clinical studies, including providing adequate safety factors for the proposed Phase I starting doses. The results of the toxicology program are consistent with the anticipated pharmacologic activity of down-modulating the PD-L1/PD-1 pathway; heightened immune responses and the potential to increase immune-associated inflammatory lesions were identified as possible safety risks in patients.

For more detailed information, please refer to the Nonclinical Studies section of the atezolizumab IB.

1.4.2 Ongoing Clinical Studies

As of 17 May 2018, clinical data on atezolizumab as a single agent or in combination with chemotherapy or targeted agents are available from more than 20 studies as follows:

- Monotherapy: Studies JO28944, PCD4989g, GO29293 (hereinafter referred to as IMvigor210), GO28753 (hereinafter referred to as POPLAR), GO28754 (hereinafter referred to as BIRCH), GO28915 (hereinafter referred to as OAK), WO29074 (hereinafter referred to as IMmotion150, where Arm B evaluates atezolizumab monotherapy), GO29664, and GO29294 (hereinafter referred to as IMvigor211).

"Use of this document is governed by the terms of use on the first page of this document."

- Combination: Studies GP28328, GP28384, GP28363, GO29383, IMmotion150 (where Arm A evaluates atezolizumab in combination with bevacizumab), WO29637 (hereinafter referred to as IMmotion151), WP29158, GO29695, GO29754, GO29322, GO30140, GO29436, GO30139 (hereinafter referred to as IMpower150), and GO29437 (hereinafter referred to as IMpower131).

Additional safety information is also gleaned from the entire development program for atezolizumab. Details of all ongoing studies can be found in the atezolizumab IB.

1.4.2.1 Safety of Atezolizumab

As of 17 May 2018, an estimated > 16,000 patients with solid tumor and hematologic malignancies have received atezolizumab in clinical trial participation as a single agent or in combination with cytotoxic chemotherapy and/or targeted therapy.

Safety findings of single-agent atezolizumab across multiple tumor types in the clinical development program are consistent with the known mechanism of action of atezolizumab and the underlying disease. Overall, treatment with atezolizumab is well tolerated, with a manageable adverse event profile. Currently, no maximum tolerated dose, no dose-limiting toxicities (DLTs), and no clear dose-related trends in the incidence of adverse events (AEs) have been determined.

Among 3075 patients treated with single-agent atezolizumab for whom pooled safety data are available (see Section 5.6.2 of atezolizumab IB for details), the most commonly reported AEs (≥ 10%) include fatigue, decreased appetite, cough, nausea, dyspnea, constipation, diarrhea, pyrexia, vomiting, arthralgia, back pain, asthenia, anemia, pruritus, rash, headache, and peripheral edema.

The AEs observed with atezolizumab in combination with chemotherapy and/or targeted therapies are consistent with the known risks of the individual study treatment.

Atezolizumab-related AEs were comparable between patients who received atezolizumab monotherapy and those who were treated with atezolizumab in combination with targeted therapy and/or chemotherapy. There are no atezolizumab-related AEs that are exacerbated when used in combination with other agents.

Immune-mediated AEs are consistent with the role of the PD-L1/PD-1 pathway in regulating peripheral tolerance. Given the mechanism of action of atezolizumab, events associated with inflammation and/or *immune-mediated* AEs are closely monitored during the atezolizumab clinical program. *Immune-mediated* AEs associated with atezolizumab include pneumonitis, hepatitis, colitis, pancreatitis, diabetes mellitus, hypothyroidism, hyperthyroidism, adrenal insufficiency, hypophysitis, Guillain-Barré syndrome, myasthenic syndrome/myasthenia gravis, meningoencephalitis, myocarditis, *myositis,* and nephritis. These AEs are described in further detail in Sections 6.4 and 6.6 of the atezolizumab IB. Guidance regarding the management of *immune-mediated* AEs is provided in Appendix 11.

"Use of this document is governed by the terms of use on the first page of this document."

Refer to the latest atezolizumab IB for a detailed discussion of available safety data from select studies in the atezolizumab clinical program.

1.4.2.2 Efficacy of Atezolizumab

As of 17 May 2018, efficacy data were most extensive for patients with non–small cell lung cancer (NSCLC; 3134 patients enrolled in Studies BIRCH, POPLAR, OAK, IMpower150, IMpower151, GO28625 [FIR, data not shown], and the NSCLC cohort of PCD4989g) and patients with metastatic urothelial carcinoma (mUC; 1104 efficacy-evaluable patients in studies PCD4989g, IMvigor210, and IMvigor211 who were administered atezolizumab.

Efficacy parameters (ORR in Cohort 1 [CCOD of 12 July 2017] and ORR, progression free survival [PFS], and OS in Cohort 2 [CCOD 12 July 2017]) observed in the mUC cohort of Phase II IMvigor210 study after an additional 10 months from the original CCOD, and 26 months of follow-up (Cohorts 1 and 2, respectively) were consistent with those obtained at primary analyses of each cohort. Similar response rates and consistency between primary and updated analyses were seen in the mUC cohort of Study PCD4989g. In both studies higher ORR results in the IC2/3 expression group suggest that higher levels of PD-L1 expression on ICs may be associated with increased benefit. The primary analysis of IMvigor211 (CCOD 13 March 2017) showed a clinically meaningful OS improvement for atezolizumab treatment compared to chemotherapy, but the results were not statistically significant. The results of IMvigor211 are consistent with those of IMvigor210.

The results from the 5 studies evaluating atezolizumab as monotherapy in patients with locally advanced or metastatic NSCLC (PCD4989g, FIR, OAK, POPLAR, and BIRCH) single-agent treatment with atezolizumab resulted in clinically meaningful OS improvement in the 2L/3L NSCLC ITT population, in comparison with standard of care, in both non-squamous and squamous histologies, and across all PD-L1 expression subgroups. Higher PD-L1 expression on TCs or ICs was associated with higher ORRs and longer median PFS and OS duration. Responses were highly durable across all PD-L1 expression groups.

Other available efficacy data suggested that treatment with atezolizumab as a single agent or in combination with other therapeutic agents resulted in anti-tumor activity across a range of other tumor types and hematologic malignancies (including pediatric-type tumors), across lines of therapy, and across PD-L1 expression subgroups.

For more detailed information, please refer to Section 5.5 of the atezolizumab IB.

“Use of this document is governed by the terms of use on the first page of this document.”

1.4.2.3 Clinical Pharmacokinetics and Immunogenicity of Atezolizumab

There have been no dedicated clinical pharmacology studies conducted for atezolizumab. Atezolizumab pharmacokinetics and other data have been analyzed from the following atezolizumab monotherapy studies: PCD4989g, JO28944, IMvigor210, IMvigor211, BIRCH, POPLAR, FIR, and OAK. PK data are available from IMmotion150 and IMpower150 where atezolizumab has been dosed in combination with other anti-cancer agents.

The key PK findings from the above-listed atezolizumab monotherapy clinical studies are summarized below:

- The pharmacokinetics of atezolizumab monotherapy have been characterized in patients in Study PCD4989g at doses 0.01 mg/kg to 20 mg/kg q3w, including the fixed dose 1200 mg (equivalent to 15 mg/kg). Exposure to atezolizumab increased dose proportionally over the dose range of 1 mg/kg to 20 mg/kg. While a subset of ADA-positive patients in Study PCD4989g receiving 0.3 to 3 mg/kg atezolizumab q3w experienced a reduction of atezolizumab Cmin to below the PK assay lower limit of quantification (LOQ), patients receiving 10 to 20 mg/kg atezolizumab, including the fixed 1200 mg dose, maintained geometric mean Cmin that was in excess of both the LOQ and the target serum concentration of 6 μg/mL (Deng et al. 2016).
- A Phase I popPK analysis that included 472 patients from Studies PCD4989g and JO28944 described atezolizumab pharmacokinetics for the dose range 1–20 mg/kg with a linear two-compartment disposition model with first-order elimination. The popPK analysis indicated that central compartment volume of distribution (V1) was 3.28 L and the Vss was 6.91 L in the typical patient. Further, the CL of atezolizumab was 0.20 L/day and the t1/2 was 27 days. Steady state was obtained after 6 to 9 weeks (2 to 3 cycles) of repeated dosing. The systemic accumulation in AUC, Cmax, and Cmin was 1.91, 1.46, and 2.75-fold, respectively.
- Based on an analysis of exposure, safety, and efficacy data, the following factors had no clinically relevant effect: age (21– 89 years), body weight, sex, albumin levels, tumor burden, region or race, renal impairment, mild hepatic impairment, level of PD-L1 expression, or ECOG status. Positive ADA status against atezolizumab led to approximately 13% reduction in overall exposure.
- The effect of moderate or severe hepatic impairment on the pharmacokinetics of atezolizumab is unknown.
- In the Phase II, randomized Study IMmotion150 of atezolizumab administered as monotherapy or in combination with bevacizumab versus sunitinib in patients with untreated advanced RCC, a total of 202 patients have evaluable atezolizumab PK data. Atezolizumab concentrations in serum were consistent and stable over 295 days. No apparent PK DDI was observed when atezolizumab and bevacizumab were dosed in combination.

“Use of this document is governed by the terms of use on the first page of this document.”

- In the Phase III, open-label, randomized Study IMpower150 to investigate the safety and efficacy of atezolizumab in combination with carboplatin + paclitaxel with or without bevacizumab compared with treatment with carboplatin + paclitaxel + bevacizumab in chemotherapy-naïve patients with Stage IV non-squamous NSCLC, the pharmacokinetics of atezolizumab in serum was available in 778 of 802 ITT patients (97%) with 4386 samples. The co-administration of bevacizumab or chemotherapy (carboplatin +paclitaxel) did not seem to influence atezolizumab pharmacokinetics.

See the atezolizumab IB for additional details on nonclinical and clinical studies.

1.5 BACKGROUND ON TOCILIZUMAB (RO4877533, ACTEMRA®, ROACTEMRA®)

Tocilizumab blocks IL-6 from binding to its receptor, both in membrane-bound and soluble states (Singh et al. 2011). With a primary indication for juvenile idiopathic arthritis (JIA), tocilizumab is approved by the Food and Drug Administration (FDA) for children as young as 2 years. It is also approved for adults with rheumatoid arthritis, adults with Giant Cell Arteritis (GCA) and for Castleman disease in Japan. Tocilizumab has been extensively studied in adults, with 8 randomized controlled trials treating more than 2000 patients (Singh et al. 2011) and in children in phase 1 to phase 3 trials for JIA (Woo et al. 2005, Yokota et al. 2005, De Benedetti et al. 2012).

In patients with severe CRS associated with T cell–engaging therapies, IL-6 levels peak during maximal T cell proliferation. A growing body of evidence suggests that IL-6 blockade by tocilizumab result in rapid, dramatic reversal of life-threatening CRS in patients treated with T cell engaging therapies (Grupp et al. 2013, Teachey et al. 2013). Whereas tocilizumab IV is typically dosed every 4 weeks in rheumatoid arthritis, extended treatment is not necessary in the management of CRS, which is self-limited and in most cases reported to require a single administration in order to control the clinical signs of CRS after treatment with T cell engaging agents (Le et al. 2018). Tocilizumab has been recently approved by the FDA (August 2017) and the European Medicines Agency has recently approved the extension of indication to include “treatment of chimeric antigen receptor (CAR) T cell-induced severe or life-threatening cytokine release syndrome (CRS) in adults and pediatric patients 2 years of age and older” (Tocilizumab USPI).

As part of the data review of BP29541 and WP29945, we observed a correlation between peaks of serum IL-6 and the occurrence of IRRs and associated symptoms of hypotension or hypoxia or dyspnea within 24 hours of RO6958688 infusion. However, the majority of these peaks are less than 1000 pg/mL. Given that hypotension and hypoxia are key symptoms of CRS, based on the current information, CRS may have occurred in some patients experiencing IRR with RO6958688 and underlying symptoms of hypotension and hypoxia. Tocilizumab should be administered for management of IRR and CRS considered related to RO6958688 per guidelines in Table 3.

“Use of this document is governed by the terms of use on the first page of this document.”

1.6 STUDY RATIONALE AND BENEFIT–RISK ASSESSMENT

1.6.1 Combination of RO6958688 and Atezolizumab

Based on the biology and mode of action of RO6958688 and PD-L1/PD-1, RO6958688 is a promising combination partner for PD-L1/PD-1 pathway antagonists.

The rationale for the combination of RO6958688 with atezolizumab is to counteract one of the immune evasion mechanisms mediated by the suppressive PD-L1 /PD-1 pathway and unleash the full potential of T-cell activity against tumors.

RO6958688 is a novel TCB-immune engager that targets human CEA on tumor cells and CDε on T cells. RO6958688 recruits and engages T cells through simultaneous binding of the CD3ε subunit of the TCR complex and CEA, a tumor cell surface antigen. Crosslinking of T cells with tumor cells leads to T-cell activation and tumor cell killing. Subsequently, expansion of preexisting and recruitment of new T cells takes place, as well as upregulation of PD-L1/ PD-1 expression.

In nonclinical models, the binding of RO6958688 to CEA and CD3ε resulted in T cell–mediated killing of cancerous cells that express CEA. RO6958688 effectively mediated the killing of cancer cells only by concurrently binding to CEA present on tumor cells and to CD3ε present on T cells. Furthermore, RO6958688 demonstrated efficacy in non-inflamed and poorly T cell–infiltrated tumors and the ability to increase T-cell infiltration in tumors, thus converting non-inflamed PD-L1–negative tumors into highly inflamed and PD-L1–positive tumors, resulting in the generation of a more inflamed tumor microenvironment (Bacac et al. 2016) and suggesting that combining RO6958688 with the anti-PD-L1 antibody atezolizumab may help prevent immune evasion mediated by the PD-1/PD-L1 pathway.

In addition to the anti-cancer cytotoxic effect of RO6958688, it has been demonstrated that exposure to RO6958688 can lead to tumor inflammation and immune stimulatory cytokine release (e.g., IFN-γ, TNF-α, granzyme B, IL-2, IL-6, and IL-10). The presence of these stimulatory cytokines, in the presence of PD-L1 inhibition with atezolizumab, may increase the activation, proliferation, and function of endogenous anti-cancer T cells, generating an anti-cancer immune response to cancer antigens beyond CEA. The addition of atezolizumab to RO6958688 is predicted to not only enhance the anti-cancer cytotoxic effects of RO6958688 but also lead to stronger endogenous anti-cancer immunity, resulting in deeper, more durable responses and preventing immune escape that is mediated by CEA downregulation or loss of cancer cells.

In nonclinical models the combination with PD-L1 blocking antibody also showed enhanced anti-tumor efficacy (Bacac et al. 2016; Roche unpublished data). In addition, early promising activity of RO6958688 administered in combination with atezolizumab, has been observed in the current ongoing study WP29945 in heavily pretreated patients with CEA-expressing tumors.

“Use of this document is governed by the terms of use on the first page of this document.”

Data discussed above demonstrate that RO6958688 and atezolizumab act synergistically in their anti-cancer properties and collectively their combination could provide meaningful clinical benefit in patients with cancer. The available safety data for both classes of agents provides guidance for enhancing and monitoring the safety of patients in this trial.

Further details on nonclinical studies conducted with the combination of RO6958688 and atezolizumab are provided in the atezolizumab and RO6958688 single agent IBs, respectively.

1.6.2 Potential for Overlapping Toxicities with RO6958688 and Atezolizumab

The following adverse events are classified as identified risks associated with use of RO6958688 in combination with atezolizumab: conjunctivitis, diarrhea and colitis.

The following adverse events are potential risks associated with combination use of atezolizumab and RO6958688, based on the evidence from Study WP29945: IRR, dysphonia, paraesthesia, musculoskeletal pain, pulmonary events, hepatic events, and GI events (e.g., vomiting, nausea, and gastrointestinal pain).

For a full discussion of potential risks associated with the combination of atezolizumab and RO6958688, see also Section 6.5.3 of the most recent RO6958688 IB.

The available nonclinical, clinical and class safety data for the two agents, provide guidance for monitoring safety of patients in this trial. Details on the monitoring for the potential overlapping toxicities are provided in Appendix 10 and Appendix 11 of the protocol.

1.6.3 Rationale for [18F] FDG-PET Imaging

In this study, [^{18}F]-FDG-PET scans will be acquired at baseline and on-treatment in order to detect a PD effect on the tumor glucose metabolism. Patients with no evidence of FDG uptake at screening will not be required to undergo on-treatment FDG-PET. Assessment of FDG-PET will be performed centrally by an independent reviewer and per local site assessment.

1.6.4 Rationale for the New Dose Schedule

In a preclinical model imaging study with radiolabeled CEA CD3 TCB, tumor targeting was shown (Roche Report No.1072962). CEA CD3 TCB was retained in the tumor lesions for at least 120 hours (last time point of the study). Consistent with the above data, a clinical imaging study with a different labeled molecule (^{89}Zr CEA-IL2v) was undertaken. This drug targeted the same CEA epitope and utilizes the same CEA binding Complementary Determining Region (CDR). Tumor accumulation was observed at least until day 8, while over 90% of the antibody was cleared from serum within 4 days (ESMO 2015 oral presentation, Tabernero et al). The above data

“Use of this document is governed by the terms of use on the first page of this document.”

suggest that the retention of the antibody in the tumor lesions could be more relevant than the blood exposure to define the best schedule for this T cell bi-specific antibody.

As reported for other immunotherapies (Topalian 2012), the pharmacodynamic effect in the tumor of the combination of RO6958688 and atezolizumab may also last for a longer period of time than either the half-life of these two molecules and the retention in the tumor of RO6958688, especially as a core element of the MOA of RO6958688 is thought to be by mediated by T-cell activation, proliferation and cytokine release.

In order to confirm this hypothesis, and further explore optimization of dose and schedule as well as direct anti-tumor cytotoxicity and endogenous anti-cancer immunity, we propose to randomize patients into different dose schedules; a QW schedule, a Q3W schedule, and a step up dosing combining the QW and the Q3W RO6958688 administration schedules (to manage cycle 1 safety, optimize RO6958688-mediated anti-cancer cytotoxicity and target to minimize the impact of ADA on RO6958688 exposure).

The exploration of these different dose and schedules for the combination of RO6958688 and atezolizumab are intended to help identify a well-tolerated starting dose, a well-tolerated late cycle maximal dose, an appropriate step up dosing regimen, and associations between these parameters on both early efficacy and prolonged/durable efficacy.

The criteria for selection of the part II schedule and dose will be based on a composite of safety, efficacy and PK/PD.

2. OBJECTIVES

2.1 PRIMARY OBJECTIVES

The primary objectives of this study are:

- To establish the preliminary safety and tolerability profile of RO6958688 in combination with atezolizumab
- To determine the maximum-tolerated dose (MTD) in cycle 1 and in later cycles, if achieved, of RO6958688 in combination with atezolizumab
- To identify a recommended phase II dose and schedule (RP2D) of RO6958688 in combination with atezolizumab.

"Use of this document is governed by the terms of use on the first page of this document."

2.2 SECONDARY OBJECTIVES

The secondary objectives for this study are:

- To describe the preliminary pharmacodynamic (PD) effects and duration of PD response for RO6958688 in combination with atezolizumab in mandatory paired tumor biopsies and paired blood samples on the basis of alterations in the quantity and quality of intratumoral T cells and peripheral blood cells (including but not limited to $CD3^+$, $CD4^+$, $CD8^+$ T cells, and other immune cells that might act as potential predictors of anti-tumor activity of RO6958688 in combination with atezolizumab)
- To describe the pharmacokinetics (PK) of RO6958688 and atezolizumab when administered in combination
- To obtain preliminary anti-tumor activity data of RO6958688 in combination with atezolizumab based on objective overall response rate (ORR), duration of response (DOR) and derived measures, disease control rate (DCR; defined as response rate [RR] + stable disease rate [SDR]), preliminary progression-free survival (PFS) and preliminary overall survival (OS) according to Response Evaluation Criteria in Solid Tumors (RECIST), Version 1.1 criteria and modified RECIST criteria, by investigator assessment for the whole study and by central assessment for prospective and retrospective analysis
- To estimate the PFS rate at relevant timepoints for RO6958688 in combination with atezolizumab

2.3 EXPLORATORY OBJECTIVES

The exploratory objectives for this study are:

- To explore the relationship between exposure, pharmacodynamics, metabolic activity of the tumor and clinical effects of RO6958688 when administered in combination with atezolizumab
- To explore the immunogenicity of RO6958688 when administered in combination with atezolizumab
- To explore the relationship of host and tumor genetic factors with PD or clinical response to therapy
- To investigate and define CEA expression in different solid tumors
- To investigate tumor mutations, gene expression and other biomarkers (such as CEA expression in various tumor indications) related to RO6958688 + atezolizumab combination therapy
- To characterize the natural growth of the tumor using tumor growth kinetics modeling
- To explore preliminary safety and efficacy in low/moderate and very low CEA expressing tumors

“Use of this document is governed by the terms of use on the first page of this document.”

- To make a preliminary assessment of the effectiveness, PK and PD effects of tocilizumab (Actemra®/RoActemra®) in ameliorating the symptoms of severe CRS following RO6958688 treatment

Functional imaging will be assessed via [^{18}F]-FDG PET/CT centrally by an independent reviewer and per local site assessment. Uptake and retention of [^{18}F]-FDG will be measured by PET/CT imaging in all patients. Patients with no evidence of FDG uptake on screening PET scan will not be required to undergo follow-up studies.

3. STUDY DESIGN

Note: Following an internal review of the clinical development plan of RO6958688, the Sponsor has decided to permanently discontinue further enrollment of patients in this study and to not open the planned cohorts B2, C3, G1, G2 and G3 and the Biomarker cohort as well as Part II of the study.

3.1 DESCRIPTION OF STUDY

This is an open-label, multi-center, dose escalation and dose/schedule finding Phase Ib clinical study of RO6958688 in combination with atezolizumab. Each treatment cycle will be 21 days in duration and consists of IV infusions of RO6958688 given weekly (QW) (± 1 day) and/or every 3 weeks (Q3W) (± 2 days) in combination with atezolizumab given every 3 weeks (Q3W) (± 2 days). During the trial, if the clinical pharmacology and/or safety data support an alternate dose sequencing and or schedule, the above could be modified accordingly. Initially all patients in this trial will receive full dose of atezolizumab (1200 mg Q3W), however if this trial data supports, investigators in agreement with the Sponsor would be allowed to consider to delay the subsequent dose up to 105 days from the last one to prevent or to manage potential adverse events suspected to be related to the atezolizumab treatment.

The initial dose of atezolizumab will be delivered over 60 (± 15) minutes. If the first infusion is tolerated without infusion-related reaction (IRR), the second infusion may be delivered over 30 (± 10) minutes. If the 30-minute infusion is well tolerated, all subsequent infusions may be delivered over 30 (± 10) minutes. The first infusion of RO6958688 will be administered over a minimum of 2 hours, subsequent infusions should be administered in at least 4 hours during the dose/escalation phase. In patients with no Grade ≥ 2 IRR/CRS for more than two RO6958688 administrations, the infusion time can be progressively reduced to a minimum of 1 hour. For more details, please refer to the RO6958688 pharmacy manual. Patients will be treated until loss of clinical benefit, unacceptable toxicities, loss of RO6958688 exposure (in which case they can continue to receive atezolizumab alone), or withdrawal of consent. The treatment period for this protocol is 24 months for both RO6958688 and atezolizumab and may be modified if emerging data supports an alternative duration of therapy. In case one of the treatments is permanently discontinued, treatment with the other drug alone may be continued as long as the patient experiences clinical benefit in the opinion of the

"Use of this document is governed by the terms of use on the first page of this document."

investigator or until unacceptable toxicity or symptomatic deterioration develops, which is attributed to disease progression as determined by the investigator and the Sponsor after an integrated assessment of radiographic data, biopsy results (if available), and clinical status, or withdrawal of consent.

3.1.1 Overview of Study Design

The study will be conducted in two parts (Figure 5). Part I is subdivided in Part IA: a dose escalation part and Part IB: dose/schedule finding. Part I objective is to evaluate the safety and determine the recommended dose and schedule of RO6958688 in combination with atezolizumab. Part II is an expansion part to confirm the safety and tolerability of the MTD dose (or OBD) as determined in Part I in order to define a RP2D and schedule of RO6958688 in combination with atezolizumab, and to explore preliminary antitumor activity, pharmacokinetic and pharmacodynamic effects.
Note: Part II has not and will not enroll any patients.

In Part IA, in order to overcome ADA and impact on PK, optional intra-patient dose escalation of RO6958688 to the next available tolerated dose level may be permitted depending on emerging clinical and safety data at the discretion of the treating physician, and after discussion with the patient. Intra-patient dose escalation may only proceed after patients have tolerated at least the first 3 consecutive doses of RO6958688 after discussion and alignment with the Medical Monitor.

“Use of this document is governed by the terms of use on the first page of this document.”

Figure 5 Study Schema

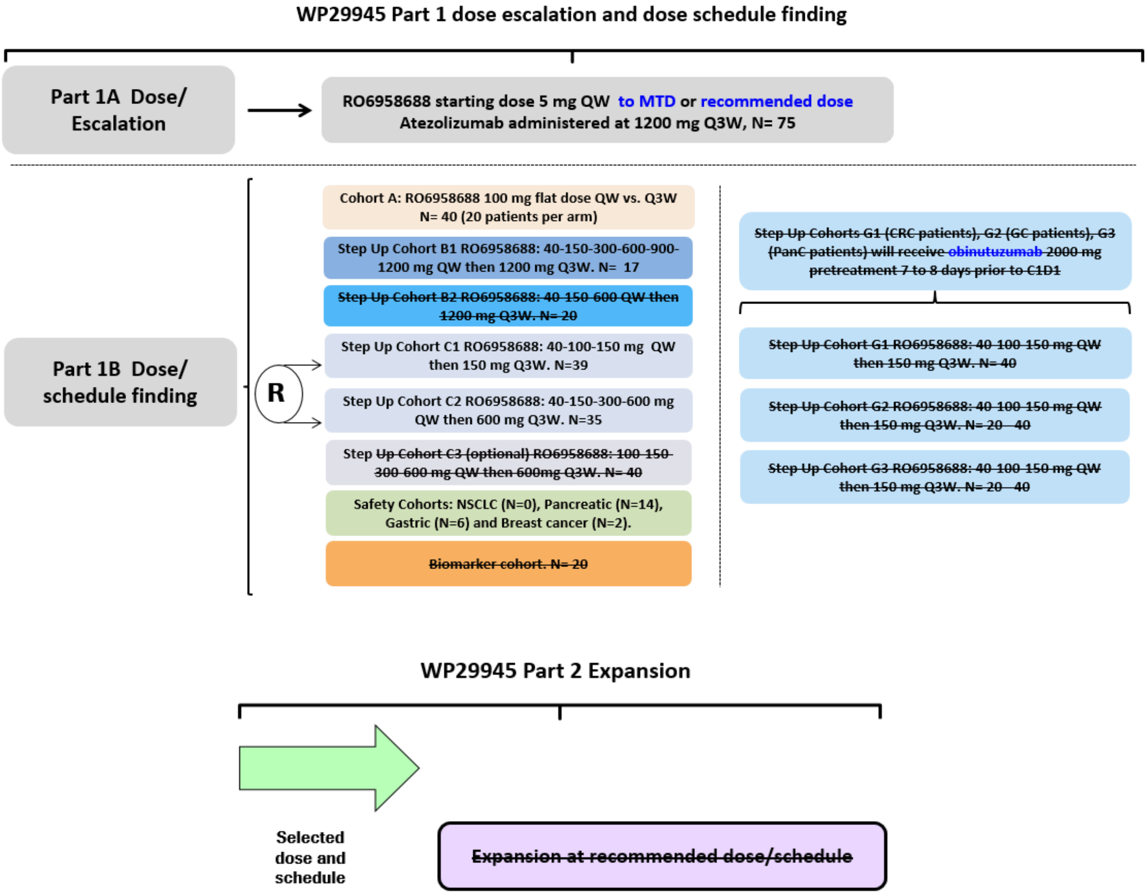

- Patient number (N) represents the actual number of patients that were enrolled in each cohort
- In part 1B: atezolizumab is administered 1200 mg Q3W
- Cohorts A and C will only enroll CRC MSS patients
- Cohort B1 will enroll CRC MSS patients and MSI^{hi} CRC patients
- Safety cohorts: RO6958688 will be administered: 40-150-300-600 mg QW then 600 mg Q3W, for the NSCLC safety cohort an alternative dose can be considered i.e., 40-100-150-300 mg QW then 600 mg Q3W

"Use of this document is governed by the terms of use on the first page of this document."

Part IA: Dose Escalation Part

Dose escalation of RO6958688 will be pursued according to a modified-Continual Reassessment Method with Overdose Control (mCRM with EWOC) design, aimed at reaching the MTD in cycle 1, which is defined as a dose with 20-30% probability of dose-limiting toxicity (DLT). For RO6958688, the starting dose is 5 mg administered in QW schedule, to be administered after the infusion of 1200 mg of atezolizumab when they are both administered on the same day (Day 1 of each cycle). For RO6958688, QW dosing will be implemented initially to generate data that can be analyzed to assess whether different dosing schedules are more effective. The RO6958688 dose will be escalated according to the mCRM, but will not exceed the RO6958688 MTD if defined in the BP29541 study. The atezolizumab dose is fixed at 1200 mg and is administered in Q3W schedule.

Patients within a cohort (at least 3 patients each) will be enrolled in a sequential manner, which, if required, can be expanded with additional patients to acquire additional safety, PK and PD data. Each patient will be observed for 21 days for DLT assessment. Enrollment will be staggered so that the first patient in each cohort will be observed for safety for 1 week before additional patients are enrolled in the cohort*. Once a minimum of 3 patients have completed the 21-day DLT observation period, the Sponsor and investigators will evaluate and agree on the dose, sequence, and timing for administration of both drugs for the subsequent cohort.

*In Spain the safety observation period between the first and subsequent patients enrolled in each cohort is 2 weeks.

Part IB: Dose/schedule finding

The first cohort (cohort A) in Part IB will compare the QW vs. Q3W schedules at a flat dose of 100 mg RO6958688 in combination with atezolizumab 1200 mg Q3W. This QW vs. Q3W schedule comparison will enroll approximately 20 to 40 randomized patients per arm.

Part IB will also explore RO6958688 step up dosing schedules in combination with atezolizumab 1200 mg Q3W.

For the step up dosing schedule, the late cycle MTD will be estimated by an intra-patient dose escalation design (see Protocol Section 6.7.1.2) in cohort B1. In this cohort, the RO6958688 dose will be escalated up to 300 % of the previous dose until the DLT criteria for that dose level are met. The dose of RO6958688 will not be escalated in the intra patient dose escalation above the monotherapy late cycle MTD of RO6958688 if defined in the BP29541 protocol.

Cohort B1 has enrolled 15 MSS CRC and 2 MSI-H CRC evaluable patients. One of the objectives of cohort B1 is to generate initial safety and efficacy data to compare the effect of treatment combination between MSS and MSI-H CRC patients. The second

“Use of this document is governed by the terms of use on the first page of this document.”

objective of cohort B1 is to define the late cycle MTD for RO6958688 in combination with atezolizumab. Note: No further patients will be enrolled in cohort B1.

The RO6958688 dose at C1D1 will be 40 mg followed by 150 mg at C1D8, 300 mg at C1D15, 600 mg at C2D1, 900 mg at C2D8 and 1200 mg at C2D15 and 1200mg at C3D1 then 1200 mg every 3 weeks (Q3W) thereafter (i.e., C4D1, C5D1…). The Q3W RO6958688 recommended dose will not exceed the late cycle MTD if defined or 1200 mg.

Cohort B2 will enroll approximately 20 MSS CRC evaluable patients. This new cohort is intended to explore a faster RO6958688 escalation in combination with atezolizumab (Q3W) in order to reach the target dose within the first atezolizumab cycle and prevent a potential early impact of anti-drug antibodies (ADA) on RO6958688 exposure within this first cycle and asses if a more rapid escalation may also increase anti-tumor activity of this combination. Enrollment in Cohort B2 can start at any time after the safety observation period of the 3rd patient in cohort B1 has been completed. Note: No patients have been enrolled into Cohort B2, and this cohort will not be opened for enrollment.

Cohort B1 will be used to define the late cycle MTD or if not defined, to assess 1200 mg as a safe dose.

The RO6958688 dose at C1D1 will be 40 mg followed by 150 mg at C1D8, 600 mg at C1D15, 1200 mg at C2D1 then 1200 mg every 3 weeks (Q3W) thereafter (i.e., C3D1, C4D1…). The Q3W RO6958688 recommended dose will not exceed the late cycle MTD if defined or 1200 mg.

Part IB of the study will also explore two additional RO6958688 step-up dose regimens (cohort C1 and C2) in combination with atezolizumab 1200 mg Q3W, in a randomized schedule comparison expansion. These randomized cohorts will start in parallel to the step up cohort B1.

Enrollment in cohorts B1, C1, and C2 will be staggered, the first patient in each cohort will be observed for safety for 1 week (2 weeks in Spain) before the second patient in each cohort is enrolled. A safety observation period of 1 week will be observed between the second and the third patient in each cohort, and from the third to subsequent patients. In the current study, we have not observed so far significant differences regarding the safety profile in patients with MSI-H tumors when compared to patients with MSS tumors; however, since only 4 MSI-H patients have been treated; in cohort B1 the same safety observation period will be applied to the first 3 MSI-H patients.

“Use of this document is governed by the terms of use on the first page of this document.”

In the randomized cohorts, RO6958688 will be administered as follows:

Cohort C1: The RO6958688 starting dose will be 40 mg, followed by 100 mg in C1D8, 150 mg in C1D15, 150 mg at C2D1 and 150 mg RO6958688 Q3W thereafter (i.e., C3D1, C4D1…).

Cohort C2: The RO6958688 starting dose will be 40 mg, followed by 150 mg in C1D8, 300 mg in C1D15, 600 mg at C2D1 and 600 mg (or late cycle MTD if defined) RO6958688 Q3W thereafter.

Based on the clinical data from the randomized cohorts (C1 and C2) the sponsor may consider opening a third cohort (cohort C3) with a starting dose of RO6958688 at 100 mg with mandatory prophylactic corticosteroids post dose at C1D1 (as detailed in Table 3), followed by 150 mg in C1D8, 300 mg in C1D15, and 600 mg at C2D1 and 600 mg (or late cycle MTD if defined) RO6958688 Q3W thereafter (i.e., C3D1, C4D1…). Note: No patients have been enrolled into Cohort C3, and this cohort will not be opened for enrollment.

The Q3W RO6958688 recommended dose for cohorts C1, C2 and the optional C3 cohort defined above, will not exceed the late cycle MTD if defined.

Each of the cohorts C1-2 and the optional cohort C3 will consist of approximately 40 locally advanced or metastatic (as defined by eligibility criteria #3) microsatellite stable (MSS) colorectal cancer patients. Approximately 80 patients will be randomized 1:1 to cohorts C1-2. Note: 39 CRC patients were enrolled in cohort C1, and 35 CRC patients were enrolled in cohort C2. No further patients will be enrolled into these cohorts, and no patients have or will be enrolled in cohort C3.

Additional safety cohorts in other solid tumor, including NSCLC, gastric, pancreatic and breast cancer will be explored (without formal estimation of maximum tolerated doses). These cohorts can be opened after the safety observation period (1 week) of the third patient in cohorts B1 or C has been completed. Note: 14 patients were enrolled in the pancreatic cohort, 6 patients were enrolled in the gastric cohort and 2 patients were enrolled in the breast cohort. No patients were enrolled in the NSCLC cohort. No further patients will be enrolled in the safety cohorts.

These safety cohorts will explore RO6958688 administered weekly (QW) at an initial dose of 40 mg followed by 150 mg in C1D8, 300 mg in C1D15, 600 mg at C2D1 and 600 mg (or MTD if defined) Q3W thereafter. Atezolizumab will be administered Q3W (1200 mg). Regarding lung cancer patients an alternative RO6958688 dose/schedule can be explored if supported by data (i.e., 40 mg at C1D1 followed by 100 mg at C1D8, 150 mg at C1D15, 300 mg at C2D1 and 600 mg every 3 weeks).

"Use of this document is governed by the terms of use on the first page of this document."

Finally, once the safety observation period of the third patient in cohort B1 has been completed, the sponsor may explore the safety and preliminary efficacy of RO6958688 in combination with atezolizumab in a separate biomarker cohort of approximately 20 patients with very low/negative CEA expressing solid tumors. Very low/negative CEA expression is defined as those samples having $<$ 20% of tumor cells with IHC1+or IHC0+. Patients enrolled in the biomarker cohort will follow the same dose/schedule as patients in cohort C1. Note: No patients have been enrolled into the biomarker cohort, and this cohort will not be opened for enrollment.

Once the late cycle MTD has been defined (see Protocol Section 6.7.1.2) or 1200 mg is declared safe in cohort B1, ongoing patients in the trial experiencing clinical benefit and showing >50% reduction of RO6958688 C_{max} and a PK profile consistent with ADA mediated decrease in exposure, can be progressively dose escalated up to the late cycle MTD or 1200 mg of RO6958688 after discussion with the Medical Monitor. However, the above optional dose escalation does not apply to patients enrolled in cohort C1 since this cohort has been designed to assess a potential biological dose.

The sponsor may consider opening additional obinutuzumab cohorts (G1 to G3). Patients participating in these cohorts will receive according to patient's and/or investigators convenience, either 2000 mg of obinutuzumab IV on Day-7 (+ 1 day) or 1000 mg of obinutuzumab IV on two consecutive days, Day-8 and Day-7 (+ 1 day) before C1D1 RO6958688 and atezolizumab administrations. Premedication will be given prior to each obinutuzumab dosing. For these patients, the baseline tumor biopsy will be taken before receiving the first dose of obinutuzumab and the on-treatment tumor biopsy remains unchanged. Note: No patients have been enrolled into the obinutuzumab cohorts (G1, G2, and G3), and these cohorts will not be opened for enrollment.

Cohort G1 (MSS CRC): approximately 40 patients with locally advanced or metastatic (as defined by eligibility criteria #3) microsatellite stable (MSS) colorectal cancer patients will be enrolled. The RO6958688 starting dose will be 40 mg, followed by 100 mg on C1D8, 150 mg on C1D15 and 150 mg RO6958688 Q3W thereafter (i.e., C3D1, C4D1…) in combination with 1200 mg atezolizumab Q3W.

Based on preliminary efficacy and safety data form Cohort G1 the sponsor may consider opening additional Cohorts for patients with gastric, pancreatic and other indications:

Cohort G2: approximately 40 patients with locally advanced or metastatic Gastric Cancer (as defined by eligibility criteria #3) will be enrolled.

The RO6958688 starting dose will be 40 mg, followed by 100 mg on C1D8, 150 mg on C1D15 and 150 mg RO6958688 Q3W thereafter (i.e., C3D1, C4D1…) in combination with 1200 mg atezolizumab Q3W.

"Use of this document is governed by the terms of use on the first page of this document."

Cohort G3: approximately 40 patents with locally advanced or metastatic Pancreatic Cancer (as defined by eligibility criteria #3) will be enrolled. The RO6958688 starting dose will be 40 mg, followed by 100 mg on C1D8, 150 mg on C1D15 and 150 mg RO6958688 Q3W thereafter (i.e., C3D1, C4D1…) in combination with 1200 mg atezolizumab Q3W.

Enrollment in Cohorts G1-G3 will be independently staggered, the first patient will be observed for safety for 1 week (2 weeks in Spain) before the second patient in each cohort is enrolled. A safety observation period of 1 week will be observed between the second and the third patient in each cohort, and from the third to subsequent patients.

For all patients who enroll in the study, tumor biopsies for PD analysis are mandatory at baseline and on-treatment, except for NSCLC patients for whom there is no accessible lesion. For patients continuing in the study, additional biopsies may be taken at the discretion of the investigator. These biopsies may serve to evaluate viable tumor given the mechanism of action of RO6958688 to induce tumor inflammation.

For patients discontinuing from the study due to disease progression, additional optional biopsies, if clinically feasible, may be taken to aid the understanding of immune resistance mechanisms.

Part II: Expansion Part

The expansion part of this study will be initiated once the recommended dose scheme has been determined. The purpose is to confirm the safety and tolerability of the recommended dose and schedule as determined in Part I and to explore preliminary antitumor activity, pharmacokinetic and pharmacodynamic effects. Based on the preliminary safety and efficacy data from Part I, the Sponsor could focus on other specific CEA expressing tumor indications for which to enroll a certain number of patients in Part II. Note: No patients have been enrolled in Part II of the study, and Part II will not be opened for enrollment.

Baseline and on-treatment tumor biopsies will be mandatory for all patients, except for NSCLC patients for whom there is no accessible lesion. DLT data will still be collected and might lead to refinement of the MTD definition for RO6958688 in combination with atezolizumab at the end of the trial.

3.1.2 Dose Escalation Decision Criteria

The decision to escalate to the next dose level will be made by the Sponsor and the participating investigators following review of all relevant safety information collected, including Adverse Events (AEs), ECGs, vital signs, clinical laboratory test results, and available pharmacokinetic data at the previous dose levels.

"Use of this document is governed by the terms of use on the first page of this document."

3.1.2.1 Escalation Criteria (Part I) – Dose Limiting Toxicities

In Part IA of the study, the RO6958688 dose will start at 5 mg (QW) and will not be escalated higher than the RO6958688 MTD if defined in the BP29541 study (RO6958688 Phase I single agent study). The atezolizumab dose is fixed at 1200 mg and is administered in Q3W schedule. During the dose escalation (Part IA), patients who discontinue treatment before the end of the DLT period, for reasons other than DLTs, and patients who did not receive the assigned dose of RO6958688 (3 doses QW for the QW regimen) or atezolizumab (1 dose Q3W) during the DLT period, will be replaced to ensure that at least 3 patients in each cohort have been assessed for the full DLT period of 21 days prior to moving to the next dose level. During the step up dose escalation (Part IB), patients who discontinue treatment for reasons other than DLTs or receiving the 1200 mg dose or the highest possible safe dose according to the intra-patient dose escalation rules (the lower one applies), may be replaced in order to ensure that at least evaluable 6 patients will be available for estimating the late cycle MTD.

For the purpose of this study, a DLT will be defined as any of the following events attributed to RO6958688 (i.e., related to RO6958688) or/and to atezolizumab and occurring during the DLT period:

Hematological toxicities defined as:

- Grade ≥ 4 neutropenia (ANC < 500/μL) lasting ≥ 7 days
- Grade ≥ 3 febrile neutropenia
- Grade ≥ 4 thrombocytopenia lasting > 48 hours (recovery to ≤ Grade 2)
- Grade 3 thrombocytopenia associated with bleeding episodes

Any non-hematological toxicity ≥ Grade 3 including:

- Grade 3 hyperbilirubinemia lasting for > 48 hours or Grade 4
- Grade ≥ 3 AST/ALT elevations with hyperbilirubinemia of ≥ Grade 2
- Grade 4 AST/ALT elevations
- For patients with Grade 2 AST, ALT, and/or alkaline phosphatase abnormality at baseline, an increase to ≥ 10 × the upper limit of normal (ULN) that does not resolve to Grade ≤ 2 within 48 hours (if symptomatic) or that does not resolve to Grade ≤ 1 within 3 weeks of onset (if asymptomatic)

Failure to recover from any drug-related toxicity that results in a dose delay of ≥ 21 days (1 Cycle) is defined as a DLT.

The following are not considered DLTs:

- Grade 3 nausea, vomiting, diarrhea, colitis or enteritis that resolves to Grade ≤ 2 with or without treatment prior to the next planned infusion of RO6958688 (1 week)
- Grade 3 *immune-mediated* adverse event that resolves to Grade ≤ 1 with immunosuppressant therapy within 3 weeks of its onset
- Grade ≥ 3 fatigue that resolves to Grade ≤ 2 within 1 week

“Use of this document is governed by the terms of use on the first page of this document.”

- Grade 3 arthralgia that can be adequately managed with supportive care or that resolves to Grade ≤ 2 within 1 week
- Fever > 40 degrees Celsius that occurs within 72 hours of RO6958688 infusion and resolves to Grade < 2 within 4 days and is resolved to Grade ≤ 1 within 10 days
- Fever ≥ Grade 3 that resolves to Grade ≤ 2 within 72 hours
- Grade 3 hypophosphatemia reversible to Grade < 2 within 1 week
- Grade ≥ 3 laboratory abnormality that is asymptomatic and deemed by the investigator not to be clinically significant
- Grade 3 autoimmune thyroiditis or other endocrine abnormality that can be managed by endocrine therapy or hormonal replacement
- Grade 3 tumor flare defined as local pain, irritation, or rash localized at sites of known or suspected tumor
- Alopecia (any grade)
- Grade 3 tumor pain that starts within 24 hours of infusion and resolves to Grade ≤ 2 within 1 week
- Grade 3 hypoxia that starts within 24 hours of infusion and resolves to Grade ≤ 2 within 1 week
- In patients with lung lesions, Grade 3 transient dyspnea secondary to localized lung edema that starts within 24 hours of infusion and recovers to Grade ≤ 2 or baseline within 1 week, and transient bronchospasm that resolves within 24 hours
- In patients with liver lesions, Grade 3 transient increase of bilirubin, transaminases and/ or Gamma GT that starts after infusion and recovers to Grade ≤ 2 or baseline within 1 week and grade 4 AST/ALT or grade 4 bilirubin increase that start after infusion and recovers to Grade ≤ 2 or baseline within 3 days.

Infusion related reactions (IRRs): IRRs are not considered to be DLTs because based on experience with monoclonal antibodies, IRRs are not dose-related events. Guidance on management of IRR/CRS and premedication that should be given is provided in sections 5.2.6 and 4.3.2.5, respectively.

3.1.2.2 Expansion Part (Part II)

The expansion part of this study will be initiated once the dose and schedule have been determined in Part I. The purpose is to confirm the safety and tolerability of the recommended dose and schedule as determined in Part I and to explore preliminary antitumor activity, pharmacokinetic and pharmacodynamic effects. DLT data will still be collected and might lead to refinement of the MTD definition for RO6958688 in combination with atezolizumab at the end of the trial. Note: No patients have been enrolled in Part II of the study, and Part II will not be opened for enrollment.

3.1.3 Communication Strategy

Upon completion of all screening evaluations and confirmation that a patient has met all of the inclusion and none of the exclusion criteria, investigator sites will contact the

"Use of this document is governed by the terms of use on the first page of this document."

Sponsor to confirm the patient number and cohort assignment (i.e., RO6958688 dose and schedule to be administered in combination with atezolizumab) via a Confirmation of Enrollment form (see Section 4.2.1). This will guarantee that the Sponsor is notified prior to the administration of RO6958688 and atezolizumab to any patient.

During Part I and Part II of this study, approximately 35 study sites will be involved for the enrollment of patients. The Sponsor and the sites will have frequent and detailed discussions regarding patient eligibility and patient care. After each patient receives RO6958688 in combination with atezolizumab (within 24 hours after study drugs infusions), the investigator must confirm to the Sponsor that the patient has received the doses and provide a brief summary of the status of the patient in terms of the safety and tolerability of RO6958688 in combination with atezolizumab, which will be communicated by email and/or telephone.

The investigator will contact the Sponsor immediately to discuss patient status and action(s) taken/to be taken in the event of a DLT during Part I and Part II of the study. In addition, as outlined above, in Part IA after each patient cohort (minimum of 3 patients) has been completed (i.e., the third patient in the cohort has reached Day 21), the Sponsor will organize a teleconference with the investigators to discuss the safety and tolerability of RO6958688 in combination with atezolizumab and to discuss the dose for the next cohort. The next dose level will be recommended using the EWOC design during the dose-escalation phase (Part IA) and discussed by the Sponsor and investigators. In addition, the clinical judgment of the Sponsor and investigators will also be utilized in the dose-selection process. This may lead to dose selections that differ from the mCRM recommendations if the scientific and clinical opinion is that a different dose would be more appropriate for patients. However, the dose selections cannot be higher than the 25% overdose probability dose estimated by the mCRM. During these teleconferences, toxicities according to National Cancer Institute Common Terminology Criteria for Adverse Events (NCI CTCAE) v4.03 will be discussed (CRS will be assessed based on NCI CTCAE v5 [Table 3]) along with the results of the available PK data in addition to safety laboratory results and any other available data that may assist the dose-escalation decision process. Dose escalation will only proceed to the higher dose level if the investigators and the Sponsor are satisfied with the safety profile of the previous patient cohort (i.e., no DLTs have been observed) and agree on the dose escalation. The discussion will be documented in writing by the Sponsor.

In addition to these communications, the Sponsor and investigators will be in regular contact throughout the study by email/telephone/fax as normal interactions during the conduct of a clinical study.

The Sponsor will be available 24 hours a day to discuss any medical or study-related issues that may arise during the conduct of this study.

“Use of this document is governed by the terms of use on the first page of this document.”

3.1.4 End of Study

The study will formally end once all patients have completed the safety follow-up visit, withdrawn from the study or when all patients have been enrolled in an extension study, whichever occurs last (the option to enroll in an extension study is for patients receiving atezolizumab only). The Sponsor may also decide to terminate the study at any time.

3.2 RATIONALE FOR STUDY DESIGN

3.2.1 Rationale for Dosage Selection

3.2.1.1 Starting Dose for RO6958688

For this study, the proposed starting dose for RO6958688 is 5 mg. This was selected on the basis of the preliminary Clinical, Safety and PD data from the EiH trial (BP29541) as summarized below. Dose escalation for Part I will follow the mCRM with EWOC design (see Section 3.2.5).

In the ongoing Phase I study BP29541 (safety cut-off: 17 August 2015), a total of 5 patients (Part I) have received at least three doses of RO6958688 (doses from 0.052 to 2.5 mg). These patients received subsequent doses up to either 2.5 mg, 5 mg and then 10 mg QW as allowed by the protocol. One of the patients in Part I continued to receive treatment (10 mg QW) by the time of the data cut-off. The number of treatment cycles received up to 17 August 2015 ranged from 3 to 29 cycles in Part I.

As of 17 August 2015, a total number of 16 patients (Part II) have received a RO6958688 dose up to 20 mg QW. By the time of the DLT data cut-off, 13 out of 16 patients in Part II continued to receive RO6958688 among who some had their initial dose escalated to 10 mg as per protocol. The number of treatment cycles received until the IB Version 2 cut-off date (12 June 2015) ranged from 4 to 18 cycles in Part II.

The safety profile, PK and the immune-related PD effect in the tumor and in peripheral blood has been analyzed and defined with full available data (cut-off date: 07 September 2015) to justify the starting dose.

As of DLT cut-off 17 August 2015, RO6958688 was well tolerated and none of the 21 patients (5 in Part I and 16 in Part II) experienced any DLT. A total of 212 AEs, including 7 SAEs, were reported in 21 patients enrolled in the ongoing Study BP29541. Five SAEs (two Grade 1 and three Grade 2) were assessed to be related to RO6958688.

At 5 mg QW we have observed signs suggesting biological activity in the CT scans and in the FDG PET scans of some patients. This dose was safe and well tolerated with no G3-G4 related safety events reported, no DLTs were reported at 5 mg neither at the two subsequent dose level cohorts, 10 mg and 20 mg. Considering all the above we propose 5 mg as the appropriate starting dose for this Phase Ib trial.

An overview of the safety profile of RO6958688 is provided in Table 1.

“Use of this document is governed by the terms of use on the first page of this document.”

Table 1 Overall Safety Profile of RO6958688 (Ongoing Study BP29541)

	Part I (SAD) 0.052 – 2.5 mg (N = 5)	Part II (MAD) 2.5 – 20 mg (N = 16)	Total 0.052 – 20 mg (N = 21)
**Total number of events** [a]			
AEs	58	154	212
SAEs	2	5	7
Deaths	2	1	3
**Number of patients with at least one event** [b]			
AE	5 (100%)	15 (93.7%)	20 (95.2%)
Related AE	2 (40.0%)	12 (75.0%)	14 (66.6%)
AE of Grade ≥ 3	2 (40.0%)	3 (18.7%)0	5 (23.8%)
SAE	2 (40.0%)	5 (31.2%)	7 (33.3%)
Related SAE	0	5 (31.2%)	5 (23.8%)
AE with fatal outcome	1 (20.0%)	0	1 (4.7%)
AE leading to withdrawal from treatment	1 (20.0%)	0	1 (4.7%)
AE leading to dose modification/interruption	1 (20.0%)	3 (18.7%)0	4 (19.0%)

AE = adverse event; MAD = multiple ascending dose; SAD = single ascending dose; SAE = serious adverse event.

Note: Percentages are based on N in the column headings of each part.

Data cut-off: 17 August 2015 (using data from patients who had received at least two complete cycles of study treatment).

[a] Multiple occurrences of the same AE in an individual are counted only once.

[b] Multiple occurrences of the same AE in an individual are counted separately.

Most patients experienced at least one AE (20/21 [95.2%]). The most frequent AE was pyrexia (11/21 [52.4%]), followed by nausea (7/21 [33.3%]), diarrhea and cough (5/21 [23.8%] each), anemia, hyponatremia, asthenia and aspartate aminotransferase increased (4/21 [19.0%] each), and hypocalcemia and headache (3/21 [14.3%] each). Pyrexia occurred at dose levels of ≥ 1.3 mg. The remaining AEs affected 2 or less patients.

Pharmacokinetics overview/summary

As of 07 September 2015, following single IV infusion of RO6958688, the serum concentrations analyzed by NCA suggest non-linear PK. However, available PK data were not sufficient to conclude on the dose-proportionality of exposure. In Part I, exposure (C_{max} and AUC_{last}) was highly variable as based on single-patient cohorts; while in Part II, exposure (C_{max} and AUC_{last}) increased approximately dose-proportionally with the dose increasing from 2.5 to 10 mg.

"Use of this document is governed by the terms of use on the first page of this document."

The terminal elimination half-life was in the range of 35.5–103.2 h following single IV infusion of RO6958688. The apparent volume of distribution ranged from 2.0 to 6.7 L, which approximates the serum volume and indicates that distribution is largely restricted to plasma and interstitial fluid, which is consistent with monoclonal antibodies.

Following multiple IV infusions of RO6958688, time-dependent PK was observed in some patients, with total loss of exposure in 3 patients enrolled in Part I (initially treated at 0.15, 1.3, 2.5 mg QW, respectively), 7 patients enrolled in Part II (dosed initially with 2.5, 5 and 10 mg) and partial reduction of exposure in 2 patients enrolled in Part II initially treated at 5 mg. In all 12 patients with reduced exposure, ADAs against RO6958688 were detected with first appearance occurring between Cycle 2 and Cycle 6 (pre-infusion). ADAs are likely to interfere with the bi-functional bio-analytical assay suggesting a decrease in active drug, or an accelerated clearance of RO6958688 via drug-ADA complexes.

3.2.1.2 Dose of Atezolizumab

The dose and schedule of atezolizumab in this study will be at a flat dose of 1200 mg (equivalent to an average body weight−based dose of 15 mg/kg) IV administered Q3W.

The target exposure for atezolizumab was projected on the basis of nonclinical tissue distribution data in tumor-bearing mice, target-receptor occupancy in the tumor, observed atezolizumab interim PK in humans, and other factors. The target trough concentration (C_{trough}) was determined on the basis of several assumptions, including: 1) 95% tumor-receptor saturation is needed for efficacy; 2) the tumor-interstitial concentration to plasma ratio is 0.30 based on tissue distribution data in tumor-bearing mice.

The atezolizumab dose is also informed by available clinical activity, safety, PK, and immunogenicity data. Anti-tumor activity has been observed across doses from 1–20 mg/kg administered Q3W. The MTD of atezolizumab was not reached and no DLTs have been observed at any dose in the above 7 Studies mentioned. Available preliminary PK data (0.03–20 mg/kg) from study PCD4989g suggest that for doses $\geq$ 1 mg/kg Q3W, overall atezolizumab exhibits pharmacokinetics that are both linear and consistent with typical IgG_1 antibodies. Detectable ADAs were observed in patients at all dose levels but were associated with changes in PK for some patients in only the lower dose cohorts (0.3, 1, and 3 mg/kg). It is unclear from currently available data in these lower dose cohorts whether administration of higher doses to patients with both detectable ADAs and reduced exposure would necessarily restore exposure to expected levels. No clear relationship between the development of measurable ADAs and safety or efficacy has been observed. Available data suggest that development of detectable ADAs does not appear to have a significant impact on PK for doses from 10–20 mg/kg administered Q3W in most patients. Correspondingly, patients dosed at the 10-, 15-, and 20-mg/kg Q3W dose levels have maintained target trough levels of drug despite the detection of ADAs.

“Use of this document is governed by the terms of use on the first page of this document.”

Currently available PK and ADA data suggest that the 15-mg/kg atezolizumab Q3W regimen for Phase II and Phase III studies would be sufficient to both maintain concentrations $\geq C_{trough}$ and further safeguard against both inter-patient variability and potential effect of ADAs that could lead to sub-therapeutic levels of atezolizumab relative to the 10-mg/kg atezolizumab Q3W regimen. From inspection of available observed C_{trough} data, moving further to the 20 mg/kg atezolizumab Q3W regimen does not appear to be warranted to maintain targeted C_{trough} levels relative to the proposed 15-mg/kg atezolizumab Q3W level.

Simulations (Bai et al. 2012) do not suggest any clinically meaningful differences in exposure following a fixed dose or dose adjusted for weight. On the basis of this analysis, a fixed dose of 1200 mg q3W was selected (equivalent to a body weight–based dose of 15 mg/kg Q3W), this dose is the one generally used in most of the studies with atezolizumab.

3.2.2 Rationale for the Treatment of Severe Cytokine Release Syndrome (CRS) Using Tocilizumab

CRS is a potentially life-threatening symptom complex, caused by the excessive release of cytokines by immune effector or target cells during an exaggerated and sustained immune response. CRS can be triggered by a variety of factors, including infection with virulent pathogens, or by medications that activate or enhance the immune response, resulting in a pronounced and uncontrolled immune response.

Regardless of the inciting agent, severe or life-threatening CRS is a medical emergency. If unsuccessfully managed, it can result in significant disability or fatal outcome. Current clinical management focuses on treating the individual signs and symptoms, providing supportive care, and attempting to dampen down the inflammatory response using high dose corticosteroids. However, this approach is not always successful, especially in the case of late intervention.

CRS is associated with elevations in a wide array of cytokines, including marked elevations in IFN-γ, IL-6, and TNF-α levels. Emerging evidence implicates IL-6 as a central mediator in CRS. IL-6 is a pro-inflammatory multi-functional cytokine produced by a variety of cell types, which has been shown to be involved in a diverse array of physiological processes, including T cell activation. Regardless of the inciting agent, CRS is associated with high IL-6 levels (Panelli et al. 2004, Lee et al. 2014, Doessegger and Banholzer 2015), and IL-6 correlates with the severity of CRS with patients who experience severe or life-threatening CRS (CTCAE Grades 4 or 5) having much higher IL-6 levels compared to their counterparts who do not experience CRS or experience milder CRS reactions (CTCAE Grades 0−3) (Chen et al. 2016). Based upon this evidence, IL-6 levels will be measured at select timepoints in the study, per the SoA, via central laboratory testing (see Appendix 1 and Appendix 12).

"Use of this document is governed by the terms of use on the first page of this document."

As mentioned in Section 1.5, as part of the data review of BP29541 and WP29945, we observed a correlation between peaks of serum IL-6 and the occurrence of IRRs and associated symptoms of hypotension or hypoxia or dyspnea within 24 hours of RO6958688 infusion. However, the majority of these peaks are less than 1000 pg/mL. Given that hypotension and hypoxia are key symptoms of CRS, based on the current information, CRS may have occurred in some patients experiencing IRR with RO6958688 and underlying symptoms of hypotension and hypoxia. A patient with Grade 5 IRR, described in Section 5.2.6.2.4, had documented very high cytokine levels including IL6 at one of the previous RO6958688. Also, 2 other patients described in Section 5.2.6.2.4, have been successfully treated with Tocilizumab, further supporting the use of Tocilizumab.

Tocilizumab (Actemra®/RoActemra®) is a recombinant, humanized, anti-human monoclonal antibody directed against soluble and membrane-bound IL-6R, which inhibits IL-6 mediated signaling. Blocking the inflammatory action of IL-6 using tocilizumab could therefore represent a novel approach for the treatment of CRS. Refer to the Tocilizumab Investigator's Brochure for additional preclinical and clinical information regarding tocilizumab.

CRS has classically been associated with therapeutic mAb infusions, most notably anti-CD3 (OKT3), anti-CD52 (alemtuzumab), anti-CD20 (rituximab) and the CD28 super-agonist TGN1412 (Lee et. al 2014). CRS is also observed with T-cell recruiting therapies, including chimeric antigen receptor (CAR)-T cell therapy and bispecific molecules, such as blinatumomab (Nagorsen et al. 2012). Tocilizumab has been recently approved by the FDA (August 2017) and received a positive CHMP opinion (European Medicines Agency 2018) for a restricted use, limited to Cytokine Release Syndrome (CRS) induced by chimeric antigen receptor (CAR) T cell in adults and pediatric patients 2 years of age and older (Tocilizumab USPI).

Taken together, these findings indicate that patients treated with RO6958688 who develop severe CRS may benefit from tocilizumab therapy. Section 5.2.6.2.4 describes recommendations regarding the management of CRS considered related to RO6958688.

The dose of 8 mg/kg (suitable for patients at or above 30 kg weight) recommended in this protocol to treat severe CRS is the one approved by the FDA for the management of (CAR)-T cells related CRS (refer to the Tocilizumab IB for details).

3.2.3 Rationale for Study Population

This study is designed to obtain safety data for the combination of RO6958688 with atezolizumab in the treatment of patients with locally advanced and/or metastatic CEA-positive solid tumors who have progressed on standard treatment or who are intolerant to standard treatment.

"Use of this document is governed by the terms of use on the first page of this document."

Based on the preliminary safety and efficacy data from Part I, the Sponsor may focus on specific tumor indications for which to enroll a certain number of patients in Part II.

RO6958688 was designed to target tumors that express CEA and concomitantly engage immune cells to lead to their activation and proliferation, which culminates in tumor lysis. Therefore, a higher activity is expected in patients with CEA-expressing tumors.

Combinations of treatments may also have the potential to expand therapy to patients least likely to respond to atezolizumab. Whereas responses to single-agent atezolizumab in PD-L1−negative patients are less frequent than in PD-L1−positive patients, early correlative studies suggest limited association between PD-L1 expression and response to complimentary immunotherapy, e.g., ipilimumab and PD-1 blockade (Callahan et al. 2013).

3.2.4 Rationale for Biomarker Assessments

RO6958688 was designed to target tumors that express CEA and to concomitantly engage immune cells to lead to their activation and proliferation, which culminates in tumor lysis. Nonclinical studies have confirmed the expected MoA of RO6958688 by mediating tumor killing, induction of T-cell activation markers (CD25 and CD69), cytokine release (IFNγ, TNFα, granzyme B, IL-2, IL-6, and IL-10), and proliferation of T cells. Furthermore, the MoA of RO6958688 occurs only if simultaneous binding (cross-linking) of T cells to CEA-expressing tumor cells takes place. Moreover, the PD readouts from the limited number of patients tested thus far, confirm some of the preclinical observations.

Therefore, samples for exploratory and pharmacodynamic analyses will be collected in the study in order to assess immune cell alterations both in the peripheral blood and tumor tissue. In particular, archival tumor tissue is to be obtained from all patients, if available, in order to perform CEA assessment for patient eligibility. If no archival tissue is available, CEA assessment will be performed with the use of the fresh baseline tumor biopsy. Archival tumor tissue, if available, will be obtained from all patients to further assess exploratory biomarkers related to the PD effects of treatment, including but not limited to the expression of tumour PD-L1, etc.

Tumor biopsies will be performed on two occasions, (once during pre-treatment and once during treatment with RO6958688 and atezolizumab), and on each occasion, two biopsy samples will be collected. These tumor biopsies for PD analyses are mandatory at baseline and on-treatment at (see Section 3.3.2.2), except for NSCLC patients for whom there is no accessible lesion. For patients discontinuing from the study due to disease progression, additional optional biopsies, if clinically feasible, may be taken to aid the understanding of immune resistance mechanisms. If preliminary data suggest that modification of the on-treatment tumor biopsy timepoint would be more appropriate, alternative on-treatment tumor biopsy timepoints could be considered in the future

“Use of this document is governed by the terms of use on the first page of this document.”

cohorts. The biopsies will be taken from accessible, “non-critical” tumor locations, including, but not limited to, skin, lymph node, rectum, liver, etc.

Tumor biopsies will be used for the assessment of different biomarkers, including but not limited to mutational signatures and tumor mutational load.

It is expected that upon treatment, the number and activation/differentiation status of intratumoral immune cells as well as their location will change. Such changes will be determined by flow cytometric and/or immunohistochemical (IHC) methods and will be analyzed centrally. Changes such as the density of different immune cell lineages (including but not limited to CD4+ T cells, CD8+ T cells, B cells, NK cells, and macrophages) and their activation and differentiation status (including but not limited to CD25, Ki67, and PD1) will be examined. The expression of tumor markers (including but not limited to PD-L1, IDO etc..) will also be examined. In addition, analysis of gene expression, genetic alteration and the infiltrating T cell repertoire may be carried out.

If available, an assessment of tumor growth kinetics will be made by comparing post-treatment scans with the last available pre-study scan.

Blood samples will be collected and analyzed with respect to alterations in the number and activation and differentiation of immune cells as a consequence to treatment with RO6958688. The samples will be analyzed by flow cytometry, and the number of cells that belongs to lymphocyte subsets (CD4+ T cells, CD8+ T cells, NK cells, B cells, and monocytes) and their activation and differentiation status will be determined (including but not limited to CD25, Ki67, and PD1).

Administration of therapeutic antibodies is frequently associated with IRRs and cytokine release. RO6958688 is expected to bind to T cells, which may lead to the release of cytokines as a response to immune cell activation. This effect might be exacerbated by concurrent administration of atezolizumab. Therefore, cytokines, including but not limited to IL-6, IFNγ, TNFα, and inflammation markers, will be assessed in serum or plasma samples. These measurements also represent safety measures.

Additional immune PD biomarkers that are related to the MoA of RO6958688 in combination with atezolizumab may also be analyzed from serum or plasma samples taken from all patients. Such PD measures will be considered as disease-monitoring markers and include but are not limited to sCEA. The specimens will be used for research purposes to identify biomarkers that are useful to predict and monitor response to RO6958688 and atezolizumab treatment and safety, assess PD effects of treatment, and investigate the mechanism of resistance to therapy.

“Use of this document is governed by the terms of use on the first page of this document.”

3.2.5 Rationale for Statistical Design

The mCRM with EWOC design uses a statistical model that actively seeks a dose-level close to the MTD by using toxicity data from all enrolled evaluable patients to compute a precise dose-toxicity curve. It locates the MTD efficiently and minimizes the number of patients treated at possibly pharmacologically inactive dose levels. Such model-based designs have been successfully applied in many Phase I dose escalation studies (Bailey et al. 2009; Le Tourneau et al. 2009). The operating characteristics (see Table 5) from extensive simulations are used to show that the mCRM provides more accurate estimates of the true probability of toxicity and provides an estimate of the MTD, which lies within the true targeted interval, without exposing patients to undue risk of toxicity. The mCRM design is known to have better chances than the "3 + 3" design of selecting an MTD within the target toxicity interval, while keeping both the chances of under- and over-dosing at a desired minimum. The more classical "3 + 3" design is known to have a tendency to under-dose (Kang and Ahn 2001, 2002) and typically leads to dose estimates with far less than 30% toxicity rate. Furthermore, the mCRM provides information on the whole observed toxicity curve. It can be run incrementally at any timepoint for decision-making and, ultimately, it accounts for all the data accrued until that point (not only the last 3 patients as in the "3 + 3"). In the current design, an extension of the mCRM allows for continuous doses over a pre-specified range. Stopping criteria are made explicit and different dose-increments depending on the dose-interval can be pre-specified for an optimal tailoring of the design to the experimental therapy being tested.

The intra-patient dose escalation design for the determination of the late cycle MTD has shown acceptable operating characteristics in simulations (Appendix 7). The Sponsor is using this simple design, since model-based dose escalation designs for intra-patient dose escalations are complex and not standard use in clinical trials.

The schedule comparison expansion (cohort A) is designed to compare dose schemes in a randomized way in order to balance cohorts on important confounders such as tumor lesion location and overall tumor burden. For cohort A, the sample size of 20 patients randomized to each dose scheme has been selected in order to obtain initial descriptive statistics on the primary safety and efficacy endpoints for the two dose schemes, which allows for a reasonably precise differentiation between the dose schemes. Specifically, an observed difference of ca. 10% points in ORR would lead to approximately 80% posterior probability of a true response rate difference between two dose schemes. For example, if the number of objective responses is 4 in one and 2 in the other dose scheme out of 20 patients each, then the posterior probability of a positive response rate difference is 79.5%. For cohort C, the randomization of 40 patients to each of the arms C1, C2 and possibly 40 patients enrolled in a later opened arm C3, allows for this reasonably precise differentiation between the dose schemes.

"Use of this document is governed by the terms of use on the first page of this document."

3.3 OUTCOME MEASURES

3.3.1 Safety Outcome Measures

The safety outcome measures for this study are:

- Incidence and nature of DLTs
- Incidence and severity of adverse events and IRRs and CRS symptoms
- Incidence of laboratory abnormalities (as examples and not limited to: hematology testing, coagulation, serum chemistries including liver enzymes, and urinalysis)
- Incidence of ADAs (anti-atezolizumab antibodies and anti-RO6958688 antibodies) formation, detection of cytokine release and potential correlation with PK, PD, safety, and efficacy parameters
- Incidence of autoantibodies (anti-nuclear antibody, anti-double-stranded DNA, cytoplasmic anti-neutrophil cytoplasmic antibody, and perinuclear anti-neutrophil cytoplasmic antibody) in comparison to baseline
- Changes in vital signs, physical findings and ECG findings.

All patients who participate in the study will be clinically evaluated at screening and on a regular basis during the entire course of the study. The routine safety monitoring plan will include clinical examination, vital signs assessment (including body temperature, respiratory rate, heart rate, diastolic and systolic BP, oxygen saturation measured by pulse oximeter – finger clip), ECG, laboratory analysis (hematology, coagulation, biochemistry, urinalysis, presence of autoantibodies), and regular collection and review of the reported adverse events.

The National Cancer Institute Common Terminology Criteria for Adverse Events v4.03 will be used to evaluate the clinical safety of the treatment in this study (http://evs.nci.nih.gov/ftp1/CTCAE/CTCAE_4.03_2010-06-14_QuickReference_8.5x11.pdf). Patients will be assessed for adverse events at each clinical visit and as necessary throughout the study. CRS severity will be graded according to the CTCAE v5 (Table 3).

Safety will be determined, but not limited to, by the spontaneous reporting of adverse events; by the assessments of routine laboratory values (hematology testing, serum chemistries); findings on physical examinations; ECGs; chest X-ray; vital signs; by carefully observing patients for IRRs; by the determination of ADAs.

Please refer to schedule of assessments for details on collection time of the assessments outlined below.

Laboratory Tests

Hematology and biochemistry will be analyzed at least prior to RO6958688 and atezolizumab administration as part of the regular safety assessments.

“Use of this document is governed by the terms of use on the first page of this document.”

1. Hematology: erythrocytes, hemoglobin, hematocrit, platelets, leucocytes and differential count (i.e., neutrophils, eosinophils, basophils, monocytes, lymphocytes).
2. Coagulation: prothrombin time (PT)/international normalized ratio (INR) and partial thromboplastin time (PTT) and fibrinogen. Additional coagulation parameters (i.e., first chromogenic antithrombin III then antigenic antithrombin III in case of chromogenic antithrombin III decrease in order to determine type 1 and type 2 antithrombin deficiencies. If a deficiency is detected, both antithrombin tests will need to be repeated at a later date/visit to confirm test findings, fibrinogen, prothrombin time, fibrin degradation products, D-dimer) could be assessed according to clinical judgment, or if any of the above parameter cannot be assessed locally.
3. Blood biochemistry: sodium, potassium, chloride, calcium, phosphate, magnesium, urea, creatinine, normal glomerular filtration rate (by CKD-EPI equation), total protein, albumin, glucose, total and direct bilirubin, alkaline phosphatase (ALP), alanine aminotransferase (ALT), aspartate aminotransferase (AST), lactate dehydrogenase (LDH), γ-glutamyl transferase (GGT), C reactive protein (CRP). NS cholesterol (total, LDL cholesterol, HDL cholesterol), triglycerides, Thyroid-stimulating hormone (TSH), free T3 (or total T3 for sites where free T3 is not performed) and T4, ferritin, soluble CD25.

   Soluble CEA (sCEA) measured in serum or plasma, will also be measured as a disease monitoring marker.

4. Urinalysis: dipstick for pH, glucose, blood, protein, ketones, and bilirubin. If there is a clinically significant positive result (i.e., confirmed by a positive repeated sample), urine will be sent to the laboratory for microscopy and culture. If there is an explanation for the positive dipstick result, e.g., menses, it should be recorded, and there is no need to perform laboratory for microscopy and culture.
5. HBV and HCV screening tests at screening.
6. All women of childbearing potential (including those who have had a tubal ligation) will have a serum pregnancy test at screening, within 7 days of first dose, on a regular basis during the treatment period (urine) and at the follow up visit.
7. Autoantibodies panel: anti-nuclear antibody, anti-double stranded DNA, cytoplasmic anti-neutrophil cytoplasmic antibody [c-ANCA], and perinuclear anti-neutrophil cytoplasmic antibody [p-ANCA].

Unscheduled hematology, biochemistry, and coagulation assay will be obtained in patients who develop infusion–related reactions (IRRs) or Cytokine Release Syndrome (refer to management Section 5.2.6.2.4) and as clinically indicated.

Additional safety laboratory assessments:

- ADA
- Cytokine release assessment/CRS
- IgE and tryptase for patients who experience a Grade ≥ 2 IRR/CRS.

“Use of this document is governed by the terms of use on the first page of this document.”

3.3.2 Pharmacokinetic (PK) and Pharmacodynamic (PD) Outcome Measures

3.3.2.1 Pharmacokinetic Outcome Measures

Pharmacokinetic (PK) concentration data of RO6958688 and atezolizumab will be summarized with the use of descriptive statistical methods. PK parameters (area under the concentration curve [AUC], volume of distribution at steady state [V_{ss}], minimum and maximum serum concentration [C_{min} and C_{max}], time to C_{max} [T_{max}], clearance [CL], and half-life (t1/2) (terminal and effective)) of RO6958688 and atezolizumab will be estimated using non-compartmental analysis (NCA) methods when applicable.

Nonlinear mixed effect modeling will also be used to analyze the sparse concentration-time-data of RO6958688.

3.3.2.2 Pharmacodynamic Outcome Measures

The PD outcome measures for this study are the following and will be examined in patients enrolled in both Parts I and II:

- **Whole blood samples:** Peripheral blood immune cells will be assessed with respect to the changes in the characteristics of lineage (CD4+ T cells, CD8+ T cells, natural killer [NK] cells, monocytes, T-regulatory cells, and B cells), activation (including but not limited to CD25, CD69, etc.), and differentiation (including but not limited to CD45RO Ki67, PD1, TIM3, ICOS, etc.).
- **Whole blood samples:** A baseline, C3D1 and C6D1 mandatory whole blood sample will be taken for TCR Vβ sequencing (the CDR3-TCR beta chain repertoire). The DNA will be used to determine in peripheral T cells the repertoire of T cell receptor (TCR) Vβ CDR3 and analyze TCR Vβ diversity.
- **Soluble CEA:** will be measured as a disease-monitoring marker. A mandatory blood sample will be taken for soluble CEA central assessment.
- **Serum or plasma samples:** PD biomarkers such as cytokines and inflammation markers (including but not limited to tumor necrosis factor-α (TNFα), interferon-γ (INFγ), interleukin (IL)-6 etc.) will be analyzed. These measurements are also safety measure assessments during any IRRs. Disease-monitoring markers that include but are not limited to sCEA will also be assessed.
- **Tumor biopsy:** Tumor biopsy samples obtained from all patients enrolled. These paired tumor biopsies for PD analyses are mandatory at baseline and on-treatment, except for NSCLC patients for whom there is no accessible lesion. If feasible, biopsies may be repeated if the initial biopsy did not contain sufficient tumor material for analysis. Biopsies will be assessed centrally for changes in immune cell numbers and activation characteristics as well as changes in tumor markers such as PD-L1. These analyses will be performed by flow cytometric molecular and/or immunohistochemistry methods with respect to changes in the characteristics of lineage (CD4+ T cells, CD8+ T cells, NK cells, monocytes, T-regulatory cells, and B cells), activation (including but not limited to CD25, CD69, etc.), differentiation (including but not limited to CD45RO Ki67, PD1, TIM3, ICOS, etc.), TCR Vβ repertoire and tumor mutational load.

“Use of this document is governed by the terms of use on the first page of this document.”

- **Positron Emission Tomography (PET):** Baseline and on-treatment 2-[18F]-Fluoro-2-deoxyglucose positron emission tomography (FDG-PET) will be collected to determine changes in glucose metabolism of the tumor lesions.
- **Original or archival tumor:** Potential predictive/prognostic biomarkers such as MMR status and CEA expression will be confirmed on archival tumor, if available, or from the freshly obtained biopsy samples. These measurements will assess the CEA change over the course of the disease and the stability of the measurements. Further other exploratory biomarker assessments, including but not limited to analysis of microsatellite instability, mutational signatures and genomic mutations, may be carried out.

Blood, Serum or Plasma Samples

Blood samples will be collected for the analyses of immune cell number and activation. Blood samples will be collected and analyzed with respect to alterations in the number and activation and differentiation of immune cells as a consequence to treatment with RO6958688 in combination with atezolizumab. The samples will be analyzed by flow cytometry, and the number of cells that belongs to lymphocyte subsets (CD4+ T cells, CD8+ T cells, NK cells, B cells, and monocytes) and their activation and differentiation status will be determined (including but not limited to CD25, Ki67 and PD1). Additional immune PD biomarkers that are related to the mode of action (MoA) of RO6958688 in combination with atezolizumab may also be analyzed from serum or plasma samples taken from all patients. Such PD measures will be considered as disease-monitoring markers and include but are not limited to sCEA. If available, an assessment of tumor growth kinetics will be made by comparing post-treatment scans with the last available pre-study scan.

Tumor Biopsy Samples

Tumor biopsy samples (each consisting of two tissue specimens at least the size of an 18G core needle biopsy, fine-needle aspiration is not acceptable) will be collected in all enrolled patients (once at baseline [after the baseline FDG PET] to confirm eligibility and once during the study treatment period), and two core samples will be collected at each occasion. Mandatory biopsies will be collected from all patients enrolled, except for NSCLC patients for whom there is no accessible lesion. The biopsies will be taken from accessible, “non-critical” tumor locations, including, but not limited to, skin, lymph node, rectum, liver, etc. On-treatment tumor biopsies will be randomized within dose schemes as follows: half of the patients at week 3 half of the patients at week 6.

If feasible, biopsies may be repeated if the initial biopsy did not contain sufficient tumor material for analysis.

If preliminary data suggest that modification of the on-treatment tumor biopsy timepoint would be more appropriate, alternative on-treatment tumor biopsy timepoints could be considered in the future cohorts.

“Use of this document is governed by the terms of use on the first page of this document.”

The baseline and on-treatment biopsies should preferentially be taken from the same tumor lesion to ensure comparability when accessible in a non-critical location.
Data from FDG-PET should guide which lesion would be more reflective of capturing the PD effect and thus the baseline and on-treatment biopsies should be taken from that tumor lesion.

For patients discontinuing from the study due to disease progression, additional optional biopsies, if clinically feasible, may be taken to aid the understanding of immune resistance mechanisms. Tumor biopsies will be centrally analyzed for immune cell number and activation by flow cytometric and/or immunohistochemical methods for the density of different immune cell lineages (including but not limited to CD4+, CD8+, NK cells, B cells, macrophages) and their activation and differentiation status (including but not limited to CD25, Ki67, PD1) and for the expression of tumor markers such as PD-L1, IDO etc. In addition, analysis of gene expression, genetic alteration and the infiltrating T cell repertoire may be carried out. The goals of these analyses will be (i) to establish a dose–response and/or an exposure-response relationship and (ii) to understand the MoA of RO6958688 in combination with atezolizumab at the tumor site.

Positron Emission Tomography (PET)

FDG, a glucose analog is taken up by cells with high metabolic activity, phosphorylated, and trapped in the cell. Since it is measuring cellular metabolism, FDG-PET can identify signs of biological effect early, before tumor size is reduced. Moreover, a reduction in the FDG-PET signal within days or weeks of initiating therapy (e.g., in lymphoma, non–small cell lung, and esophageal cancer) has been shown in some cases, to correlate with prolonged survival and other clinical end points now used. These findings suggest that FDG-PET could facilitate drug development as an early marker of drug effect.

Whole-body imaging PET should begin 60 ± 10 min after FDG injection.

If preliminary data suggest that modification of the on-treatment FDG-PET timepoint would be more appropriate, alternative on-treatment FDG-PET timepoints could be considered in the future cohorts.

3.3.3 Efficacy Outcome Measures

Any evaluable or measurable disease must be documented at screening and re-assessed at each subsequent tumor evaluation. The efficacy/activity outcome measures for this study are:

- ORR, defined as the proportion of patients achieving objective partial (PR) or complete (CR) responses
- Best overall response (BOR) defined as the best response recorded from the start of the study treatment until the end of treatment taking into account any requirement for confirmation

“Use of this document is governed by the terms of use on the first page of this document.”

- Duration of response (DOR), defined as the time from initial objective response (PR or CR) to the first objective progression or death from any cause
- The rate of patients with stable disease (SDR)
- DCR, defined as ORR + SDR
- PFS according to Response Evaluation Criteria in Solid Tumors, Version 1.1 (RECIST v1.1)
- OS, defined as the time from first study treatment (primary efficacy analysis) or randomization date (ITT, only in randomized schedule comparison expansions) to death from any cause

Tumor response will be evaluated according to RECIST v1.1 and modified RECIST criteria using unidimensional measurement such as computed tomography (CT) scan or magnetic resonance imaging. Assessment of CT/MRI scans as tumor assessments will be performed at the sites during the whole study, and in addition centrally by an independent reviewer for prospective and retrospective analysis. Advanced volumetric analyses of CT/MRI will be performed.

Tumor assessment will be performed once during the screening period. The first assessment after the start of treatment will be performed at 8 weeks (C3D15) and continue every 8 weeks thereafter for the first year and every 12 weeks thereafter until disease progression or treatment discontinuation. All tumor assessments after baseline may be done within ± 7 days of the scheduled visit. Additionally, FDG-PET based tumor assessment will be performed at baseline and at week 4 (C2D8 + 1 week) and week 16 (C6D8) after the first dose of RO6958688, in case of dose delay the FDG PET should be done at week 4 (+1 week) and week 16. The week 16 FDG PET scan should be performed predose (and up to a maximum of 3 days before the visit). Baseline FDG-PET must be performed before the baseline tumor biopsy. Snapshots of CT scans and FDG-PET will be made available to the Sponsor. Based on data generated during this trial, the timepoints at which the two on treatment FDG PET scans are performed may be modified.

Confirmation of partial and complete responses will be done at the next scheduled visit after at least 28 days from the initial response. A patient is assigned a best overall confirmed response SD if they have a response assessment of SD, PR, or CR at one or more visits at least 42 days (6 weeks) after start of study treatment, but are not a confirmed CR or PR.

PFS per RECIST v1.1 or modified RECIST criteria is defined as time between enrollment or randomization date (whichever is applicable) and date of first documented disease progression per RECIST v1.1 or modified RECIST criteria, respectively, or death from any cause, whichever occurs first. Patients who neither progressed nor died in this interval, or who are lost to follow-up are censored at the date of last tumor assessment within this time window or last follow-up for progression of disease. Patients

"Use of this document is governed by the terms of use on the first page of this document."

for whom no post-baseline tumor assessments are available are censored at first study treatment. Conventional response criteria may not adequately assess the activity of immunotherapeutic agents because progressive disease (by initial radiographic evaluation) does not necessarily reflect therapeutic failure. Because of the potential for pseudoprogression/tumor immune infiltration, this study will allow patients to receive atezolizumab and/or RO6958688 to remain on study treatment after apparent radiographic progression, provided the benefit-risk ratio is judged to be favorable.

Patients should be discontinued for unacceptable toxicity or loss of RO6958688 exposure (in which case they can continue to receive atezolizumab alone) or symptomatic deterioration attributed to disease progression as determined by the investigator after an integrated assessment of radiographic data and clinical status (see Section 4.6.1.1).

Patients will be permitted to continue study treatment after meeting RECIST criteria for progressive disease (by investigator assessment) if they meet all of the following criteria:

- Evidence of clinical benefit as assessed by the investigators
- Absence of symptoms and signs (including worsening of laboratory values; e.g., new or worsening hypercalcemia) that indicate unequivocal progression of disease
- No decline in ECOG performance status that can be attributed to disease progression
- Absence of tumor growth at critical anatomical sites (e.g., leptomeningeal disease) that cannot be managed by protocol–allowed medical interventions
- Patients for whom approved therapies exist must provide written consent to acknowledge that they defer these treatment options in favor of continued study treatment at the time of initial apparent progression.

Patients in whom radiographic disease progression is confirmed at a subsequent tumor assessment may be considered for continued study treatment at the discretion of the investigators if they continue to meet the criteria above and have evidence of clinical benefit.

Optional submission of the latest pre-study or historical CT scans is highly encouraged for assessment of tumor growth kinetics within 6 weeks of patient entering the study if available. This scan will be compared to those collected during the study to determine tumor growth kinetics.

3.3.4 Exploratory Outcome Measures

The exploratory objectives for this study include but are not limited to the following:

- The density and localization of immune cells will be determined in freshly obtained biopsy in order to describe the immune infiltration before treatment

"Use of this document is governed by the terms of use on the first page of this document."

- The density and activation status of immune cell subsets in the tumor will be assessed in biopsies taken before and during treatment
- A possible association of expression of activation related immune genes (e.g., IFNγ, CXCLC9, etc.) with PD response will be investigated
- The baseline values and kinetics of soluble markers of immune cell activation (such as sCD25) and tumor markers (such as CEA and PD-L1) will be explored.

Additional markers may be measured in case a scientific rationale for these analyses develops.

4. MATERIALS AND METHODS

4.1 CENTER

This is a multi-center study (approximately 35 centers) to be conducted in North America, Europe, and Asia. Additional sites (and countries) may be included for back-up purposes and may be activated if needed. Note: No patients were enrolled in Asia.

Administrative and Contact Information, and List of Investigators are provided separately.

4.2 STUDY POPULATION

This study will enroll patients with locally advanced and/or metastatic CEA-positive solid tumors who have progressed on standard treatment or who are intolerant to standard treatment will be enrolled into this study.

This study has enrolled a total of 228 patients. For the purpose of this protocol, evaluable patients will be those with sufficient tumor tissue material to be collected during baseline and on-treatment biopsies to allow for the required PD studies. Patients who do not meet this requirement will be replaced. However, any patient who receives one or more doses of RO6958688 and/or atezolizumab will be evaluable for safety assessments.

In Part I, 228 patients were enrolled. No further enrollment will take place.

For the biomarker cohort, this study will enroll patients with very low/negative CEA expressing solid tumors. Very low/negative CEA expression is defined as those samples having $<$ 20% of tumor cells with IHC1+or IHC0+. Patients enrolled in the biomarker cohort will follow the same dose/schedule as patients in cohort C1.

Based on the preliminary safety and efficacy data from Part I, the Sponsor could focus on other specific CEA expressing tumor indications for which to enroll a certain number of patients in Part II. Note: Part II will not open for enrollment.

“Use of this document is governed by the terms of use on the first page of this document.”

4.2.1 Recruitment Procedures

Patients will be identified for potential recruitment using pre-screening enrollment logs, IEC/IRB approved newspaper/radio advertisements and mailing lists prior to consenting to take place on this study.

Once a patient has been confirmed as meeting all of the inclusion and none of the exclusion criteria, the Sponsor will approve enrollment of this patient in writing (via Confirmation of enrollment), which will be sent to the investigator.

For Part I of the study, the assigned dose of RO6958688 and the possible dosing date will be documented in the Confirmation of Enrollment, the screening and patient number is confirmed via the IXRS system. A Patient Enrollment and Identification Code List must be maintained by the investigator.

In Part IA, at least 3 patients will be initially enrolled in each cohort; additional patients might be enrolled to study safety, PK, or PD in more detail. Patients in a cohort will be enrolled in a sequential manner. Thus, the first patient in each cohort will be observed for safety for 1 week before enrolling additional patients in a cohort. For the schedule comparison cohorts B1), and C (part IB), the first patient in each cohort will be observed for safety for 1 week (2 weeks in Spain) before the second patient in each cohort is enrolled. A safety observation period of 1 week will be observed between the second and the third patient in each cohort, and from the third to subsequent patients.

During the dose escalation part, patients who withdraw before the end of the DLT period, for reasons other than DLTs, and patients who did not receive the assigned dose of RO6958688 (3 doses QW for the QW regimen) or atezolizumab (1 dose Q3W) during the DLT period, will be replaced to ensure that at least 3 patients in each cohort have been assessed for the full DLT period of 21 days prior to moving to the next dose level.

For Part II of the study, the screening and patient number, assigned dose and schedule of RO6958688 will be confirmed via the IXRS system. A Patient Enrollment and Identification Code List must be maintained by the investigator.

4.2.2 Inclusion Criteria

Patients must meet the following criteria for study entry:

1. Signed informed consent
2. Age ≥ 18 years
3. Confirmed locally advanced and/or metastatic solid tumor, with at least one tumor lesion of accessible non-critical location to biopsy, in patients who have progressed on a standard therapy, are intolerant to standard therapy, and/or are non-amenable to standard therapy
4. Radiologically measurable and clinically evaluable disease (as per RECIST v1.1 - previously irradiated lesions should not be counted as target lesions)

"Use of this document is governed by the terms of use on the first page of this document."

5. Life expectancy (in the opinion of the investigator) of ≥ 12 weeks and LDH levels ≤ 2.5 ULN
6. Eastern Cooperative Oncology Group (ECOG) Performance Status (PS) 0–1
7. All acute toxic effects of any prior radiotherapy, chemotherapy, or surgical procedure must have resolved to Grade ≤ 1 or returned to baseline except alopecia (any grade) and Grade 2 peripheral neuropathy
8. Adequate hematological function (without transfusion within 2 weeks prior to Cycle 1, Day 1): neutrophil count of ≥ 1.5 × 10^9 cells/L and hemoglobin ≥ 9 g/dL (5.5 mmol/L), and lymphocytes within normal limits (≥ 0.8 × 10^9 cells/L), platelet count of ≥ 100,000/μL; INR and aPTT ≤ 1.5 × ULN. This applies only to patients who are not receiving therapeutic anticoagulation; patients receiving therapeutic anticoagulation should be on a stable dose. Genetic deficiencies affecting aPTT are not excluded unless assessed as clinically significant by the site hematologist.
9. Adequate liver function: total bilirubin ≤ 1.5 × the upper limit of normal (ULN; excluding Gilbert's Syndrome as defined in Appendix 8), when < 3 × ULN is allowed), AST and/or ALT ≤ 2.5 × ULN (in case of liver metastases, ≤ 5 × ULN). Patients with documented liver metastases: alkaline phosphatase ≤ 5 × ULN. For patients with bone disease, this criterion should only take into consideration the specific liver isoform of alkaline phosphatase (not the total since it is influenced by the bone isoform).
10. Adequate renal function: Creatinine clearance ≥ 60 ml/min calculated by CKD-EPI equation (see Appendix 2)
11. Negative serum pregnancy test within 7 days prior to study treatment in premenopausal women and women ≤ 2 years after start of menopause (menopause is defined as amenorrhea for > 2 years)
12. All women of childbearing potential and female partners of male patients: agreement to remain abstinent (refrain from heterosexual intercourse) or use contraceptive methods that result in a failure rate of < 1% per year during the treatment period and for at least 5 months after the last dose of RO6958688 and atezolizumab and 2 months after the last dose of tocilizumab (if applicable).

    For male participants, patient must agree to either remain completely abstinent or to use a condom and not donate sperm during the entire study period and for 3 months after the last administration of RO6958688 and for 2 months after the last dose of tocilizumab (if applicable). Male patients who have received only atezolizumab are not required to use contraception during atezolizumab treatment; pregnancies in female partners of male patients receiving atezolizumab monotherapy are not required to be reported.

    A woman is considered to be of childbearing potential if she is postmenarcheal, has not reached a postmenopausal state (> 2 years of amenorrhea with no identified cause other than menopause), and has not undergone surgical sterilization (removal of ovaries and/or uterus).

"Use of this document is governed by the terms of use on the first page of this document."

Examples of contraceptive methods with an expected failure rate of < 1% per year include bilateral tubal ligation, male sterilization, established, proper use of hormonal contraceptives that inhibit ovulation, hormone-releasing intrauterine devices, and copper intrauterine devices.

The reliability of sexual abstinence should be evaluated in relation to the duration of the clinical trial and the preferred and usual lifestyle of the patient. Periodic abstinence (e.g., calendar, ovulation, symptothermal, or postovulation methods) and withdrawal are not acceptable methods of contraception.

13. Patients with non-colorectal cancer should have confirmed CEA expression in tumor tissue (≥ 20% of tumor cells staining with at least moderate to high intensity of both cytoplasmic and/or membranous (IHC 2+ and IHC 3+). CEA expression should be centrally confirmed for U.S. and Canada patients.

    For CRC, the CEA assessment should be performed but the result is not required for patient selection; if no archival tumor tissue is available, fresh biopsy will be collected.

14. For the biomarker cohort (in Part IB), patients should have very low/negative CEA expression. Very low/negative CEA expression is defined as tumor samples having < 20% of tumor cells with IHC1+ or IHC 0+. CEA should be determined prior to enrollment, if no archival tumor is available, a fresh biopsy will be collected.

4.2.3 Exclusion Criteria

Patients who meet any of the following criteria will be excluded from study entry:

1. Active or untreated central nervous system (CNS) metastases as determined by CT or MRI evaluation during screening and prior radiographic assessments

    Patients with a history of treated asymptomatic CNS metastases are eligible, provided they meet all of the following criteria:

    - No metastases to brain stem, midbrain, pons, medulla, cerebellum, or within 10 mm of the optic apparatus (optic nerves and chiasm)
    - Radiographic demonstration of improvement upon the completion of CNS-directed therapy and no evidence of interim progression between the completion of CNS-directed therapy and the screening radiographic study
    - No history of intracranial hemorrhage or spinal cord hemorrhage
    - No ongoing requirement for dexamethasone as therapy for CNS disease; anticonvulsants at a stable dose allowed
    - No stereotactic radiation or whole-brain radiation within 28 days prior to Cycle 1 Day 1
    - Screening CNS radiographic study ≥ 4 weeks since completion of radiotherapy and ≥ 2 weeks since discontinuation of corticosteroids.

2. Spinal cord compression not definitively treated with surgery and/or radiation or previously diagnosed and treated spinal cord compression without evidence that disease has been clinically stable for ≥ 2 weeks prior to enrollment.

“Use of this document is governed by the terms of use on the first page of this document.”

3. Leptomeningeal disease.
4. Patients with paraspinal, paratracheal and mediastinal pathological lesions larger than 2 cm unless they are previously irradiated. Irradiation of lesions must be completed at least 14 days prior to initiation of study treatment.
5. Malignancies within 5 years prior to enrollment, with the exception of those with a negligible risk of metastasis or death and treated with expected curative outcome (such as adequately treated carcinoma in situ of the cervix, basal or squamous cell skin cancer, localized prostate cancer treated surgically with curative intent, ductal carcinoma in situ treated surgically with curative intent).
6. Significant, uncontrolled concomitant diseases which could affect compliance with the protocol or interpretation of results, including diabetes mellitus, pulmonary disorders, and known autoimmune diseases.
7. Uncontrolled hypertension (systolic blood pressure (BP) $>$ 150 mmHg and/or diastolic BP $>$ 100 mmHg), unstable angina, congestive heart failure (CHF) of any New York Heart Association (NYHA) classification (Class II or greater), serious cardiac arrhythmia requiring treatment (exceptions: atrial fibrillation, paroxysmal supraventricular tachycardia), history of myocardial infarction within 6 months of enrollment.
8. Administration of a live, attenuated vaccine within 28 days before Cycle 1 Day 1 or anticipation that such a live attenuated vaccine will be required during the study. Influenza vaccination should be given during influenza season only (approximately October to March). Patients must not receive live, attenuated influenza vaccine (e.g., FluMist®) within 4 weeks prior to Cycle 1 Day 1 or at any time during the study.
9. Known HIV.
10. Active Hepatitis B (HBV) or Hepatitis C (HCV) infection (required at screening):
    - Patients with active hepatitis B (defined as having a positive hepatitis B surface antigen [HBsAg] test at screening)
        - Patients with past HBV infection or resolved HBV infection (defined as having a negative HBsAg test and a positive antibody to hepatitis B core antigen [anti-HBc] antibody test) are eligible.
    - Patients with active hepatitis C
        - Patients positive for HCV antibody are eligible only if PCR is negative for HCV RNA.
11. Severe infections within 28 days prior to Cycle 1 Day 1, including but not limited to hospitalization for complications of infection, bacteremia, or severe pneumonia or active tuberculosis.
12. Received oral or intravenous (IV) antibiotics within 14 days prior to Cycle 1 Day 1. Patients receiving prophylactic antibiotics (e.g., for prevention of a urinary tract infection) are eligible.

"Use of this document is governed by the terms of use on the first page of this document."

13. Any other diseases, metabolic dysfunction, physical examination finding, or clinical laboratory finding giving reasonable suspicion of a disease or condition that would contraindicate the use of an investigational drug.

14. Major surgery or significant traumatic injury < 28 days prior to Cycle 1 Day 1 (excluding biopsies) or anticipation of the need for major surgery during study treatment.

15. Dementia or altered mental status that would prohibit informed consent.

16. Known history of autoimmune disease, including but not limited to myasthenia gravis, myositis, autoimmune hepatitis, systemic lupus erythematosus, rheumatoid arthritis, inflammatory bowel disease, vascular thrombosis associated with antiphospholipid syndrome, Wegener's granulomatosis, Sjögren's syndrome, Bell's palsy, Guillain-Barré syndrome, multiple sclerosis, vasculitis, or glomerulonephritis

    - The protocol allows:

        - Patients with a history of autoimmune hypothyroidism on a stable dose of thyroid replacement hormone are eligible

        - Patients with Type 1 diabetes mellitus on appropriate medical management may be eligible for this study *after consultation with* the Medical Monitor.

        - Patients with positive serology of auto-antibody panel (anti-nuclear antibody, anti-double stranded DNA, cytoplasmic anti-neutrophil cytoplasmic antibody [c-ANCA], and perinuclear anti-neutrophil cytoplasmic antibody [p-ANCA]) at screening should be referred to a specialist (i.e., Rheumatologist) for further assessments if the Investigator, after discussion with the Medical Monitor, considers the results as clinically significant.

17. History of idiopathic pulmonary fibrosis, pneumonitis (including drug induced), organizing pneumonia (i.e., bronchiolitis obliterans, cryptogenic organizing pneumonia, etc.), or evidence of active pneumonitis (including drug induced) on screening chest CT scan. History of radiation pneumonitis in the radiation field (fibrosis) is permitted.

18. Patients with bilateral lung lesions and dyspnea and/or SaO2 <92% (at rest, room air and exertion) or patients with lobectomy or pneumonectomy with lung metastases in the remaining lung and either dyspnea or SaO2 <92% (at rest, room air and exertion) at baseline.

19. Baseline QTc interval of > 470 ms, baseline resting bradycardia < 45 beats per minute, or baseline resting tachycardia > 100 beats per minute.

20. Pregnant or breast-feeding women.

21. Known hypersensitivity to any of the components of RO6958688 and atezolizumab; hypersensitivity to Chinese hamster ovary cell products or other recombinant human antibodies.

"Use of this document is governed by the terms of use on the first page of this document."

22. Investigational therapy (defined as treatment for which there is no regulatory authority approved indication) or last dose of prior immunotherapies including but not limited to: interferon alpha (IFN-α), interferon-beta (IFN-β), IL-2, conjugated IL-2, CEA-IL2v, cytokines, anti-CTLA4, within 28 days prior to Cycle 1 Day 1. Patients previously treated with anti-programmed death-ligand 1 (PD-L1), or anti-PD-1 are excluded.
23. Any approved anti-cancer therapy, including chemotherapy or hormonal therapy, within 28 days prior to Cycle 1 Day 1, with the following exceptions:
    - Hormone-replacement therapy or oral contraceptives
    - Tyrosine kinase inhibitors (TKIs) (both small molecules and antiangiogenic monoclonal antibodies) that have been discontinued > 21 days prior to Cycle 1 Day 1; baseline scans must be obtained after discontinuation of prior TKIs.
24. Prior systemic corticosteroids >10mg prednisone (or equivalent) within 14 days of Cycle 1 Day 1. The use of inhaled and/or topical corticosteroids and mineralocorticoids (e.g., fludrocortisone) is allowed.
25. Last dose with any of the following agents including but not limited to: etanercept, infliximab, tacrolimus, cyclosporine, mycophenolic acid, alefacept, or efalizumab < 28 days prior to first dose of study drugs.
26. Regular immunosuppressive therapy (i.e., for organ transplantation, chronic rheumatologic disease).
27. Patients with prior allogeneic bone marrow transplantation or prior solid organ transplantation.
28. Treatment with systemic immunosuppressive medications including, but not limited to: prednisone, cyclophosphamide, azathioprine, methotrexate, thalidomide, and anti-TNF agents within 14 days prior to Cycle 1, Day 1. Patients who have received acute and/or low-dose systemic immunosuppressant medications (e.g., a one-time dose of dexamethasone for nausea or chronic use of ≤ 10 mg/day of prednisone or dose-equivalent corticosteroid) may be enrolled in the study after discussion with the Medical Monitor. The use of inhaled corticosteroids and mineralocorticoids (e.g., fludrocortisone) for patients is allowed.
29. Radiotherapy within the last 28 days before Cycle 1 Day 1 with the exception of limited field palliative radiotherapy e.g., for bone pain relief.

4.3 STUDY TREATMENTS

For the purpose of the study, RO6958688, atezolizumab, and tocilizumab are considered investigational medicine products (IMPs).

4.3.1 Formulation, Packaging, and Handling

4.3.1.1 RO6958688

Study drug packaging will be overseen by the Sponsor's clinical trial supplies department and bear a label with the identification required by local law, the protocol number, drug identification, and dosage. The packaging and labeling of the study

"Use of this document is governed by the terms of use on the first page of this document."

medication will be in accordance with the Sponsor's standard and local regulations. Upon arrival of investigational products at the site, site personnel should check them for damage and verify proper identity, quantity, integrity of seals, and temperature conditions and report any deviations or product complaints to the monitor upon discovery.

The qualified individual responsible for dispensing the study drug will prepare the correct dose according to the schedule. This individual will write the date of dispensing and patient number and initials on the study drug vial label and on the Drug Accountability Record. This individual will also record the study drug batch or lot number received by each patient during the study.

The manufacture of RO6958688 drug substance consists of fermentation cell culture and purification.

Ro 695-8688/F01-01 drug product is provided as a sterile, preservative-free, liquid concentrate for infusion in 6-mL glass vials with L-histidine/L-histidine hydrochloride aqueous buffer solution (pH 5.5) containing sucrose, methionine, and polysorbate 20. Each single-use, 6-mL vial contains 25 mg (nominal strength) of RO6958688, and the drug product is formulated at a concentration of 5 mg/mL.

Ro 695-8688/F03-01 drug product is provided as a sterile, preservative-free, liquid concentrate for infusion in 6-mL glass vials with L-histidine/L-histidine hydrochloride aqueous buffer solution (pH 5.5) containing sucrose, methionine, and polysorbate 20. Each single-use, 6-mL vial contains 100 mg (nominal strength) of RO6958688, and the drug product is formulated at a concentration of 20 mg/mL.

A variant of the current formulations with higher protein concentration has been developed to reduce the drug product volume. Ro 695-8688/F04-03 drug product is provided as a sterile, preservative-free, liquid concentrate for infusion in 2-mL glass vials with L-histidine/L-histidine hydrochloride aqueous buffer solution (pH 5.5) containing sucrose, methionine, and polysorbate 20. Each single-use, 2-mL vial contains 100 mg (nominal strength) of RO6958688, and the drug product is formulated at a concentration of 50 mg/mL.

Roformis No.	Ro 695-8688/F01-01	Ro 695-8688/F03-01	Ro 695-8688/F04-03
Protein concentration	5 mg/mL	20 mg/mL	50 mg/mL
Vial strength	25 mg / 5 mL	100 mg / 5 mL	100 mg / 2 mL

The recommended storage condition for Ro 695-8688/F01-01, Ro 695-8688/F03-01 and Ro 695-8688/F04-03 drug products is between 2°C–8°C, and it should be protected from light.

"Use of this document is governed by the terms of use on the first page of this document."

Upon arrival of investigational products at the site, site personnel should check them for damage and verify proper identity, quantity, integrity of seals and temperature conditions, and report any deviations or product complaints to the monitor upon discovery.

For further details on the storage and preparation of RO6958688, please refer to the WP29945 Pharmacy manual and RO6958688 Investigator's Brochure.

4.3.1.2 Atezolizumab

The atezolizumab drug product is provided in a single-use, 20-cc USP/Ph. Eur. Type 1 glass vial as a colorless-to-slightly-yellow, sterile, preservative-free clear liquid solution intended for IV administration. The vial is designed to deliver 20 mL (1200 mg) of atezolizumab solution but may contain more than the stated volume to enable delivery of the entire 20 mL volume. The atezolizumab drug product is formulated as 60 mg/mL atezolizumab in 20 mM histidine acetate, 120 mM sucrose, 0.04% polysorbate 20, pH 5.8.

Atezolizumab must be refrigerated at 2–8°C (36–46°F) upon receipt until use. Atezolizumab vials should not be used beyond the expiration date provided by the manufacturer. No preservative is used in atezolizumab drug product; therefore, the vial is intended for single use only. Discard any unused portion of drug left in a vial. Vial contents should not be frozen or shaken and should be protected from direct sunlight.

Upon arrival of investigational products at the site, site personnel should check them for damage and verify proper identity, quantity, integrity of seals and temperature conditions, and report any deviations or product complaints to the monitor upon discovery.

For further details on the storage and preparation of atezolizumab, please refer to the WP29945 Pharmacy Manual and atezolizumab Investigator's Brochure.

4.3.1.3 Tocilizumab

The tocilizumab drug product will be supplied by the Sponsor. Tocilizumab will be supplied in vials containing sterile solution of tocilizumab (20 mg/mL). Tocilizumab will be supplied as 200-mg vials.

Tocilizumab should be administered when necessary, as described in Appendix 10. For information on the formulation and handling of tocilizumab, see the pharmacy manual and the Tocilizumab Investigator's Brochure.

In an emergency situation where the study-specific labeled supply of tocilizumab is not accessible, it may be supplied locally by the study sites and will be formulated, prepared, and handled according to the local prescribing information. For further instructions regarding recommended storage conditions and packaging configuration, refer to the local prescribing information.

"Use of this document is governed by the terms of use on the first page of this document."

4.3.2 Dosage, Administration, and Compliance

4.3.2.1 RO6958688 and Atezolizumab

The qualified individual responsible for dispensing the study drugs will prepare the correct dose according to the cohort allocation schedule. This individual will write the date dispensed and patient number and initials on the study drugs vial labels and on the Drug Accountability Record. This individual will also record the study drugs batches or lot numbers received by each patient during the study.

Atezolizumab and RO6958688 should be administrated in a hospital or clinic equipped for IV chemotherapy, with full emergency resuscitation facilities immediately available. For anaphylaxis precautions, see Appendix 14. During and after the administration of both study drugs patients should be under close supervision of the investigator at all times. In case of infusion-associated adverse events in a patient, the signs and symptoms should be fully resolved before the patient is discharged.

Guidelines for dosage modification and treatment interruption or discontinuation are provided in Section 5.2.1.

4.3.2.2 Administration of Atezolizumab

Each treatment cycle will be 21 days in duration and consists of IV infusions of RO6958688 given weekly (QW ± 1 day) or every 3 weeks (Q3W ± 2 days) in combination with atezolizumab given every 3 weeks (Q3W ± 2 days).

Patients will first receive atezolizumab (1200 mg fixed dose) IV on Day 1 of each cycle, followed by RO6958688, given IV. Initially all patients in this trial will receive full dose of atezolizumab (1200 mg Q3W). In order to allow for resolution of toxicities, investigators in agreement with the sponsor are allowed to consider delaying the subsequent dose up to 105 days from the last one to prevent or to manage potential adverse events suspected to be related to the atezolizumab treatment.

The initial dose of atezolizumab will be delivered over 60 (± 15) minutes. If the first infusion is tolerated without infusion-related reaction (IRR), the second infusion may be delivered over 30 (± 10) minutes. If the 30-minute infusion is well tolerated, all subsequent infusions may be delivered over 30 (± 10) minutes.

If premedication is needed prior to atezolizumab infusion, it should be administered at least 30 minutes before the start of atezolizumab infusion.

4.3.2.3 Administration of RO6958688

RO6958688 will be administered by IV infusion on Day 1, Day 8 and Day 15 of each cycle for the QW regimen and on Day 1 of each cycle for the Q3W regimen.

For cohorts B, C and cohort G in part IB of the study, RO6958688 will be administered as described in Section 3.1.1.

A delay of RO6958688 administration for up to 14 days in the QW part and 21 days in the Q3W part will be acceptable to allow for resolution of toxicity NCI CTCAE Grade ≤ 2 hematological toxicities or Grade ≤ 1 non-hematological toxicities (with the exception of

"Use of this document is governed by the terms of use on the first page of this document."

toxicity considered as non-RO6958688 related). RO6958688 administration may be delayed in case of a Grade ≥2 infusion related reaction during the atezolizumab administration. No other delays of RO6958688 administration are foreseen.

On Day 1 of each cycle, RO6958688 will be administered:

- at least half an hour after the end of atezolizumab infusion if no premedication is given prior to RO6958688 administration.
- at least one hour after the end of atezolizumab infusion if premedication is given prior to RO6958688 administration.

RO6958688 should be administered at C1D1 by IV infusion over a minimum of 2 hours, subsequent infusions should be administered in at least 4 hours during the dose/escalation phase, in patients with no grade ≥ 2 IRR/CRS for more than two RO6958688 administrations, the infusion time can be progressively reduced to a minimum of 1 hour. For more details, please refer to the RO6958688 pharmacy manual.

If premedication is needed prior to RO6958688 infusion, it should be administered at least 30 minutes before the start of RO6958688 infusion. Please refer to Section 4.3.2.5 for details of pre-medications prior to RO6958688 infusion.

Hypotension may occur as a result of an IRR/CRS, therefore, it is recommended that antihypertensive drugs must not be given on the morning of, and throughout the infusion of RO6958688 if clinically indicated. Patients with a history of cardiac disease should be monitored closely.

Based on chemical and physical in-use stability data the infusion time should not exceed 8 hours at room temperature including interruptions (if any), the Pharmacy Manual includes detailed instructions regarding refrigerated storage of the dose solution prior to administration.

Information on cohorts, doses, and administration parameters are described in the Pharmacy Manual.

In Part IA (dose escalation) optional RO6958688 dose escalation to the next available tolerated dose level can only proceed after a patient has tolerated at least the first 3 consecutive doses of RO6958688 after discussion with and approval by the Medical Monitor.

4.3.2.4 Administration of Tocilizumab

Tocilizumab infusion will follow the methods described in the Investigator Brochure or other similar local prescribing documents and will be administered for the treatment of CRS considered related to RO6958688 (see Appendix 10, Table 2).

“Use of this document is governed by the terms of use on the first page of this document.”

Tocilizumab (8 mg/kg) will be administered at room temperature by controlled IV infusion into over a 1-hour period. The infusion rate must be 10 mL/hr for 15 minutes and then increased to 130 mL/hr to complete the dosing over the 1-hour time period. Normal saline (20 mL) will be administered following infusion of study drug to flush the remaining study drug through the IV set.

4.3.2.5 Premedication and Prophylactic Treatment for RO6958688 and Atezolizumab

The use of analgesics, NSAID and/or antihistamines is allowed to minimize expected flu-like symptoms associated with the administration of RO6958688 as follows:

- Paracetamol/acetaminophen (500–1000 mg orally or IV) and/or NSAID
- Diphenhydramine (50–100 mg [orally or IV] or an alternative antihistamine at an adequate dose)

Pre-medicate all patients in step-up cohorts with 10 mg dexamethasone IV until the RO6958688 dose escalation has been completed (Table 2).

Table 2 Pre-Medications to Be Administered before RO6958688 Infusion

Infusion of RO6958688	Patients requiring pre-medications	Pre-medication**	Administration
During step-up phase	All patients	IV dexamethasone 10mg	Completed at least 1 hour prior to RO6958688 infusion
		Analgesic and/or NSAID	At least 30 min before RO6958688 infusion
		Anti-histamine	
During step-up phase	IRR/CRS G2*** with previous RO6958688 infusion despite pre-medications	For corticosteroid use, see management guidelines in Appendix 10**.**	
		Analgesic, anti-emetics and/or NSAID	At least 30 min before RO6958688 infusion
		Anti-histamine	
During flat dose phase†	Patient with no IRR/CRS at previous infusion	Analgesic, anti-emetics and/or NSAID	At least 30 min before RO6958688 infusion
		Anti-histamine	
	Patient with G1 or G2 IRR/CRS at previous infusion	Analgesic, anti-emetics and/or NSAID	At least 30 min before RO6958688 infusion
		Anti-histamine	
		For corticosteroid use, see management guidelines in Appendix 10.	

**Analgesic- Paracetamol/acetaminophen 500-1000 mg orally or IV. Anti-histamine diphenhydramine 50-100 mg orally or IV or alternate anti-histamine at adequate dose
***Per Appendix 10, in patients with IRR/CRS recurring at ≥Grade 3, patients not recovering within 8 hours of corticosteroids and tocilizumab administration, and patients with Grade 4 IRR/CRS, RO6958688 should be permanently discontinued.
† Patients in flat dose cohorts to follow this recommendation

"Use of this document is governed by the terms of use on the first page of this document."

If an IRR/CRS occurs, follow IRR/CRS management guidelines in Appendix 10 for premedication recommendations. Corticosteroid administration should be completed at least 60 minutes before RO6958688 administration.

4.3.3 Investigational Medicinal Products Accountability

All investigational medicinal products (IMPs) required for completion of this study (RO6958688, atezolizumab, and tocilizumab) will be provided by the Sponsor where required by local health authority regulations. The investigational site will acknowledge receipt of IMPs supplied by the Sponsor using IXRS, to confirm the shipment condition and content. Any damaged shipments will be replaced.

Tocilizumab will be provided by the Sponsor.

The investigator is responsible for the control of drugs under investigation. Adequate records of the receipt (e.g., Drug Receipt Record) and disposition (e.g., Drug Dispensing Log) of the study drugs must be maintained. The Drug Dispensing Log must be kept current and should contain the following information:

- The identification of the patient to whom the study drugs were dispensed (for example patient initials and date of birth)
- All records and drug supplies must be available for inspection by the Roche Monitor (at every monitoring visit).

IMPs will either be disposed of at the study site according to the study site's institutional standard operating procedure or returned to the Sponsor with the appropriate documentation. The site's method of IMP destruction must be agreed upon by the Sponsor. Local or institutional regulations may require immediate destruction of used investigational medicinal product for safety reasons. In these cases, it may be acceptable for investigational study site staff to destroy dispensed investigational product before a monitoring inspection provided that source document verification is performed on the remaining inventory and reconciled against the documentation of quantity shipped, dispensed, returned, destroyed and provided that adequate storage and integrity of drugs has been confirmed.

The site must obtain written authorization from the Sponsor before any IMP is destroyed, and IMP destruction must be documented on the appropriate form.

Written documentation of destruction must contain the following:

- Identity batch numbers of investigational product[s] destroyed
- Quantity of investigational product[s] destroyed
- Date of destruction
- Method of destruction
- Name and signature of responsible person [or company] who destroyed investigational products

"Use of this document is governed by the terms of use on the first page of this document."

Accurate records of all IMPs received at, dispensed from, returned to, and disposed of by the study site should be recorded on the Drug Inventory Log.

4.3.4 Post-Trial Access to RO6958688 and Atezolizumab

The Sponsor will offer post-trial access to the study drugs (RO6958688 and atezolizumab) free of charge to eligible patients in accordance with the Roche Global Policy on Continued Access to Investigational Medicinal Product, as outlined below.

A patient will be eligible to receive study drugs after the end of the study if all of the following conditions are met:

- The patient has a life-threatening or severe medical condition and requires continued study drugs treatments for his or her well-being
- There are no appropriate alternative treatments available to the patient
- The patient and his or her doctor comply with and satisfy any legal or regulatory requirements that apply to them

A patient will not be eligible to receive study drugs after the end of the study if any of the following conditions are met:

- The study drugs are commercially marketed in the patient's country and is reasonably accessible to the patient (e.g., is covered by the patient's insurance or wouldn't otherwise create a financial hardship for the patient)
- The Sponsor has discontinued development of the study drugs or data suggest that the study drugs are not effective for cancer
- The Sponsor has reasonable safety concerns regarding the study drugs as treatment for cancer
- Provision of study drugs is not permitted under the laws and regulations of the patient's country

F. Hoffmann La-Roche does not intend to provide study intervention to patients after conclusion of the study or in case of earlier patient withdrawal.

4.4 CONCOMITANT THERAPY

4.4.1 Permitted Therapy

Concomitant therapy includes any medication, e.g., prescription drugs, over the counter drugs, approved dietary and nutritional supplements used by a patient from 4 weeks prior to screening until the follow-up visit. Concomitant use of herbal therapies is not recommended because their pharmacokinetics, safety profiles, and potential drug-drug interactions are generally unknown.

“Use of this document is governed by the terms of use on the first page of this document.”

All concomitant medications should be reported to the investigator and recorded on the Concomitant Medications electronic Case Report Form (eCRF).

All therapy and/or medication administered to manage adverse events should be recorded on the Adverse Event eCRF.

Patients who experience infusion-associated symptoms may be treated symptomatically as described in Section 5.2.1.

Use of Steroids

Systemic corticosteroids and immune suppressants may attenuate potential beneficial immunologic effects of treatment with atezolizumab and RO6958688 but may be administered at the discretion of the treating physician after consultation with the Medical Monitor. If feasible, alternatives to corticosteroids should be considered (such as infliximab).

Megestrol administered as an appetite stimulant is acceptable while the patient is enrolled in the study.

Infusion related reaction/CRS

Premedication including steroids may be administered as described in Section 4.3.2.5.

The use of inhaled corticosteroids and mineralocorticoids (e.g., fludrocortisone) for patients with orthostatic hypotension or adrenocortical insufficiency is allowed.

Radiotherapy

Use of limited field palliative radiotherapy is allowed at any time during the study except for:

- Days where study drugs are administered
- One day prior or post study drug administration is observed

No delay of study drugs administration is foreseen although subjects should not receive study treatments during radiation.

Influenza vaccination

Influenza vaccination should be given during influenza season only (approximately October to March). Patients must not receive live, attenuated influenza vaccine (e.g., FluMist®) within 4 weeks prior to Cycle 1, Day 1 or at any time during the study and up to 5 months after the last dose of atezolizumab but may receive inactivated vaccine.

“Use of this document is governed by the terms of use on the first page of this document.”

4.4.2 Prohibited Therapy

The use of the following therapies is prohibited during the study and for at least 28 days prior to initiation of study treatments (unless otherwise specified):

- Investigational or unlicensed/unapproved agents
- Immunotherapy/radio-immunotherapy
- Chemotherapy
- Hormonal anticancer therapy
- Immunostimulatory agent (all patients, including those who discontinue the study early, should not receive other immunostimulatory agents for 10 weeks after the last dose of atezolizumab)
- Radiotherapy (with the exception of limited–field palliative radiotherapy)
- Biologic agents (e.g., bevacizumab, erlotinib)
- Immunosuppressive medications, including but not limited to cyclophosphamide, azathioprine, methotrexate, and thalidomide; these agents could potentially alter the activity and the safety of atezolizumab. Systemic corticosteroids administered at a dose equal or higher than prednisone 10 mg/day or equivalent (inhaled and topical steroids are permitted).

    Systemic corticosteroids, TNF-α inhibitors, mycophenolate, and other immunosuppressive medications may be administered for the treatment or prevention of *immune-mediated* toxicities at the discretion of the treating physician after consultation with the Medical Monitor.

- Other systemic anti-neoplastic agents and targeted therapies
- Patients must not receive live, attenuated vaccines (such as FluMist®) at any time during the study and up to 5 months after the last dose of atezolizumab. Vaccination with live vaccines is not recommended during treatment and is forbidden 28 days prior to dosing with atezolizumab.
- Initiation or increased dose of granulocyte colony-stimulating factors (e.g., granulocyte colony-stimulating factor, granulocyte/macrophage colony-stimulating factor, and/or pegfilgrastim) is prohibited.

If any anti-neoplastic or investigational therapies listed above are needed, the patient will be considered to have evidence of progressive neoplastic disease and have experienced treatment failure with study treatment and should be withdrawn from study treatment.

Patients who experience a mixed response that requires local therapy (e.g., surgery, stereotactic radiosurgery, radiotherapy, and/or radiofrequency ablation) for control of three or fewer lesions may still be eligible to continue study treatment. Patients who receive local therapy directed at a target lesion will no longer be evaluable for radiographic response but will remain evaluable for progression. Such cases must be discussed with and approved by the Medical Monitor.

"Use of this document is governed by the terms of use on the first page of this document."

All concomitant treatments must be documented in the eCRF.

4.5 STUDY ASSESSMENTS

4.5.1 Description of Study Assessments

All examinations listed below will be performed according to the Schedule of Assessment and Hourly Assessment tables outlined in Appendix 1.

4.5.1.1 Medical History and Demographic Data

Medical history includes clinically significant diseases, demographic data (including age, sex, and self-reported race/ethnicity), all surgeries, cancer history (including any biomarker information (e.g., tumor mutations like KRAS, Braf and NRAS, CEA expression), all prior cancer therapies and procedures), reproductive status, and concomitant medications.

4.5.1.2 Physical Examinations, Vital Signs, and ECOG Performance Status

A complete physical examination should include an evaluation of the head, eyes, ears, nose, throat, neck and lymph nodes, and the cardiovascular, dermatological, musculoskeletal, respiratory rate, gastrointestinal, genitourinary, and neurological systems.

Any abnormality identified at baseline should be recorded on the General Medical History and Baseline Conditions eCRF.

A physical examination will include careful examinations of the areas of known and possible malignancy. The physical examination includes weight (height measurement is only done at screening and at follow-up visit, the body mass index (BMI) will be derived). For the purpose of the study, the same calibrated balance should be used at each site. Vital signs (including body temperature, respiratory rate, heart rate, diastolic and systolic BP, oxygen saturation at rest). In patients with bilateral lung lesions or patients with lobectomy or pneumonectomy with lung metastases in the remaining lung, oxygen saturation under exercise is also required. ECOG PS will be obtained in all patients at screening and as described in the Schedule of Assessment and Hourly Assessment tables (see Appendix 1).

PS will be measured using the ECOG PS scale (Appendix 3). PS will be assessed with each physical examination, prior to each study drug administration, and at the safety follow-up visit. It is recommended, where possible, that a patient's PS be assessed by the same person throughout the study.

Results will be recorded on the eCRF. Changes from baseline abnormalities should be recorded in patient's notes. New or worsened clinically significant abnormalities should be recorded as adverse events on the Adverse Event eCRF.

"Use of this document is governed by the terms of use on the first page of this document."

Routine vital signs monitoring including body temperature, respiratory rate, heart rate, diastolic and systolic BP, oxygen saturation will be recorded at the timepoints specified in Schedule of Assessment and Hourly Assessment tables (see Appendix 1).

During the day, BP and heart rate should be obtained in a quiet room at a comfortable temperature, with the patient's arm unconstrained by clothing or other material.
All measurements will be obtained from the same arm and with the same cuff size using an automatic instrument with a digital readout throughout the study. The "ideal" cuff should have a bladder length that is 80% and a width that is at least 40% of arm circumference (a length-to-width ratio of 2:1). The automatic cuff should be placed on the designated arm at least 10 minutes prior to dosing. The patient should have his or her back and arm supported such that the middle of the cuff on the upper arm is at the level of the right atrium (the midpoint of the sternum). After the patient has been resting in supine decubitus position for at least 5 minutes, BP and heart rate will be obtained.

4.5.1.3 Electrocardiograms

Triplicate 12-lead ECG recordings (i.e., three qualitatively acceptable ECGs without artifacts) must be obtained at the timepoints as specified in the Schedule of Assessments (see Appendix 1) and within approximately 2–5 minutes at each specified timepoint. The average of the three readings will be used to determine the ECG intervals (i.e., PR, QRS, QT). Additional unscheduled ECG assessments should be performed in case of abnormalities and if clinical symptoms occur.

To minimize variability, it is important that the patient is in a supine position for ≥ 5 minutes prior to each ECG evaluation. The conditions should be as close as possible to predose timepoints; this includes but is not limited to food intake, activity level, stressors, and room temperature. Body position should be consistently maintained for each ECG evaluation to prevent changes in heart rate. Environmental distractions (e.g., television, radio, conversation) should be avoided during the pre-ECG resting period and during ECG recording. Whenever possible, ECGs should be performed prior to meals, any scheduled vital sign measurements, and blood draws. In some cases, it may be appropriate to repeat abnormal ECGs to rule out improper lead placement that may contribute to the abnormality in ECG results.

For safety monitoring purposes, the investigator or designee must review, sign, and date all ECG tracings. Paper or electronic copies will be kept as part of the patient's permanent study file at the site. If considered appropriate by the Sponsor, ECGs may be analyzed retrospectively at a central laboratory.

ECG characteristics including heart rate, QRS duration, and PR and QT intervals and QTcB (Bazett's correction) will be recorded on the eCRF, QTcF (Fridericia's correction), and RR will be derived on the eCRF. Changes in T-wave and U-wave morphology and overall ECG interpretation will be documented on the eCRF. T-wave information will be

"Use of this document is governed by the terms of use on the first page of this document."

captured as normal or abnormal, whereas U-wave information will be captured in two categories, absent/normal or abnormal.

4.5.1.4 Pulmonary Function Tests (FEV1/VC/TLC and DLCO)

FEV1/VC/TLC (including all derived parameters: inspiratory reserve volume, expiratory reserve volume, tidal volume and total lung capacity) and DL_{CO} (corrected for both alveolar volume and hemoglobin) will be evaluated at screening in patients with bilateral lung metastases or patients with lobectomy or pneumonectomy with lung metastases in the remaining lung. Additional tests may be performed if clinically indicated.

4.5.1.5 Laboratory Assessments

Normal ranges for the study laboratory parameters must be supplied to Roche before the study starts. Laboratory safety tests shall be collected at time-points specified in the Schedule of Assessments (see Appendix 1).

Additional blood or urine samples may be taken at the discretion of the investigator if the results of any test fall outside the reference ranges or clinical symptoms necessitate additional testing to monitor patient safety. Where the clinical significance of abnormal lab results is considered uncertain, screening lab tests may be repeated before enrollment to confirm eligibility. If there is an alternative explanation for a positive urine or blood test for drugs of abuse, e.g., previous occasional intake of a medication or food containing for example codeine, benzodiazepines, or opiates, the test may be repeated to confirm washout.

In the event of unexplained abnormal clinically significant laboratory test values, the tests should be repeated immediately and followed up until they have returned to the normal range and/or an adequate explanation of the abnormality is found. Results of clinical laboratory testing will be recorded on the eCRF or will be received as electronically produced laboratory reports submitted directly from the local or central lab.

Urinalysis, hematology, blood biochemistry, coagulation, and Hepatitis B and Hepatitis C virus serology samples will be sent to the study site's local laboratory for analysis.

- Hematology: erythrocytes, hemoglobin, hematocrit, platelets, leucocytes, and differential count (neutrophils, eosinophils, basophils, monocytes, lymphocytes).
- Blood biochemistry: sodium, potassium, chloride, calcium, phosphate, magnesium, urea, creatinine, normal glomerular filtration rate (by CKD-EPI equation), total protein, albumin, glucose, total and direct bilirubin, alkaline phosphatase (ALP), alanine aminotransferase (ALT), aspartate aminotransferase (AST), lactate dehydrogenase (LDH), γ-glutamyl transferase (GGT), C reactive protein (CRP); cholesterol (total, LDL cholesterol, HDL cholesterol), triglycerides, ferritin, soluble CD25.

Serum CEA will also be measured as a disease-monitoring marker (as part of the serum chemistry panel).

"Use of this document is governed by the terms of use on the first page of this document."

- Coagulation: prothrombin time (PT)/international normalized ratio (INR) and partial thromboplastin time (PTT) and fibrinogen. Additional coagulation parameters (i.e., first chromogenic antithrombin III then antigenic antithrombin III in case of chromogenic antithrombin III decrease in order to determine type 1 and type 2 antithrombin deficiencies. If a deficiency is detected, both antithrombin tests will need to be repeated at a later date/visit to confirm test findings, fibrinogen, prothrombin time, fibrin degradation products, D-dimer) could be assessed according to clinical judgment, or if any of the above parameter cannot be assessed locally.
- Thyroid function: thyroid stimulating hormone (TSH) free T3 (or total T3 for sites where free T3 is not performed) and T4.

Unscheduled hematology, biochemistry and coagulation may be obtained in patients who develop IRRs/CRS.

- Pregnancy test: All women of childbearing potential (including those who have had a tubal ligation) will have a serum pregnancy test at screening within 7 days of first dose, on a regular basis during the treatment period (urine) and at Safety follow-up visit.
- Urinalysis: Dipstick for pH, glucose, blood, protein, ketones, and bilirubin. If there is a clinically significant positive result (i.e., confirmed by a positive repeated sample), urine will be sent to the laboratory for microscopy and culture. If there is an explanation for the positive dipstick result, e.g., menses, it should be recorded, and there is no need to perform laboratory for microscopy and culture.
- Hepatitis B virus (HBV) serology (HBsAg, antibodies against HBsAg, hepatitis B core antigen)
    - HBV DNA is required if patient has positive anti-HBc test.
- HCV serology (anti-HCV).

Based on continuous analysis of the data in this study and other studies, any sample type not considered to be critical for safety may be stopped at any time if the data from the samples collected does not produce useful information.

4.5.1.6 Additional Samples

Thyroid stimulating hormone (TSH) and auto-antibody samples will be sent to the study site's local laboratory for analysis. Freshly obtained tumor biopsies, PK, ADA, plasma samples, and whole blood samples for flow cytometry and clinical genotyping will be analyzed centrally by one or several central laboratories or by the Sponsor. Instruction manuals and supply kits will be provided for all central laboratory assessments.

Based on continuous analysis of the data in this study and other studies, any sample type not considered to be critical for safety may be stopped at any time if the data from the samples collected do not produce useful information.

“Use of this document is governed by the terms of use on the first page of this document.”

4.5.1.6.1 Pharmacokinetic Assessments

PK samples are mandatory for all parts of the study. Blood samples to evaluate RO6958688 and atezolizumab serum levels will be collected from an IV line from the arm opposite to that which is used for study drugs administration. The date and time of each sample collection will be recorded in the eCRF. The PK assessments will be performed as outlined in the Schedule of Assessments and Hourly Assessment tables (see Appendix 1). During the course of the study, PK sampling time points may be modified based on emerging data to ensure the PK of RO6958688 can be adequately characterized (but without increasing overall blood collection volume for PK). No new PK sampling time points will be introduced. Additional PK samples will be taken at the time of treatment discontinuation, as an unscheduled sample, if the patient experiences an IRR/CRS or an AE leading to dose reduction or delay of RO6958688 and atezolizumab administration (see Section 5.2.1). Remaining PK sample volume may also be used for assay validation during the development of study or compound related assays after the mentioned intended uses.

4.5.1.6.2 Special Safety Assessments

Anti-Drug Antibodies (ADAs)

Although RO6958688 and atezolizumab are both humanized antibodies, there is a risk that ADAs against RO6958688 and atezolizumab could develop, potentially reducing the efficacy of either antibody and/or potentially resulting in symptomatic hypersensitivity reactions, in particular immune complex reactions.

In this study, samples will be taken to assess the possible presence of ADAs. The date and time of each sample will be recorded in the eCRF.

Blood samples for ADA determination will be obtained as specified in the Schedule of Assessments and Hourly Assessment tables (see Appendix 1). Additional samples will be drawn as an unscheduled ADA sample in patients with clinical signs of hypersensitivity reaction, in particular immune complex reactions. In any case, for each collected ADA sample, a corresponding PK sample will be collected at the same time point for the determination of the RO6958688 and/or atezolizumab concentration.
If required, the residual of the PK samples taken during the study (any timepoint) can be used for additional ADA analysis. Remaining ADA sample volume may also be used for assay validation during the development of study or compound related assays after the mentioned intended uses.

Cytokine Release Assessment during IRR/CRS

Plasma samples (PD plasma and additional safety) for the assessment of cytokine release will be collected at the time of an IRR/CRS (including repetitive occurrence of IRR/CRS hypersensitivity reaction) as an unscheduled plasma sample. These samples will be analyzed as described in Section 4.5.1.6.3 – Serum or Plasma samples.

"Use of this document is governed by the terms of use on the first page of this document."

Serum Thyroid-Stimulating hormone (TSH)

Serum TSH and free T3 (or total T3 for sites where free T3 is not performed) and T4 level will be assessed at baseline pre-dose at cycle 1, on study (every 3 months), and at study completion/early termination visit and safety follow-up visit (28 days after last dose of study drugs). In case of abnormalities, the patient should be monitored until full resolution and further analysis (i.e., free T3 and T4 serum level) performed. An endocrinologist should be consulted if an endocrinopathy is suspected.

Autoantibody Panel

The panel should include anti-nuclear antibody, anti−double-stranded DNA, circulating anti-neutrophil cytoplasmic antibody, and perinuclear anti-neutrophil cytoplasmic antibody. The autoantibody panel will be assessed at screening, pre-dose every second cycle, and every 3 months after third month on therapy (samples can be drawn up to 1 week before Day 1 visit), if possible locally. In patients who develop signs and/or symptoms suggestive of auto-immune disease while on treatment, the antibody panel should be repeated. Patients with confirmed positive serology of at least one of the auto-antibody panel (anti-nuclear antibody, anti-double stranded DNA, cytoplasmic anti-neutrophil cytoplasmic antibody [c-ANCA], and peri nuclear anti-neutrophil cytoplasmic antibody [p-ANCA]) at screening and during the course of the study should be referred to a specialist (i.e., Rheumatologist) for further assessment if the investigator, after discussion with the Medical Monitor, considers it as clinically significant.

4.5.1.6.3 Pharmacodynamic, Exploratory and Clinical Genotyping Assessments

The following samples will be used for research purposes to identify biomarkers useful for predicting and monitoring response to RO6958688 and atezolizumab treatments, identifying biomarkers useful for predicting and monitoring RO6958688 in combination with atezolizumab safety, assessing PD effects of RO6958688 and atezolizumab treatments, and investigating mechanism of resistance to therapy. Additional markers may be measured in the case of a strong scientific rationale.

The following samples will be collected as specified in the Schedule of Assessments and Hourly Assessment tables (see Appendix 1):

Whole Blood Samples for Flow Cytometry

In this study, whole blood samples will be collected for the flow cytometry. The samples will be analyzed for changes in the numbers and activation status of lymphocyte subsets including but not limited to $CD8^+$, $CD4^+$, and regulatory T cells, B cells and/or NK cells.

Whole Blood Samples

In addition, a baseline and Cycle 3 Day 1 mandatory whole blood sample will be taken for TCR Vβ sequencing (the CDR3-TCR beta chain repertoire). The DNA will be used to determine in peripheral T cells the repertoires of T cell receptor (TCR) Vβ CDR3 and analyze TCR diversity.

“Use of this document is governed by the terms of use on the first page of this document.”

Soluble CEA

A mandatory blood sample will be taken for soluble CEA central assessment. Samples will be collected as per the schedule of assessments in Appendix 1.

Serum or Plasma Samples

Blood for serum or plasma isolation (blood for pharmacodynamics and additional safety) will be collected. Cytokines, inflammation markers, soluble CEA will be measured in these samples. Additionally, in case of IRR(s) or CRS, an assessment of cytokines released during the reaction will be done on serum or plasma samples from the time of the IRR/CRS. The sample used for these PD analyses will be identical with the one collected for cytokine release at IRR/CRS and IgE and tryptase assessment.

Tumor Biopsy Samples

Tumor biopsy samples (each consisting of two tissue specimens at least the size of an 18G core needle biopsy, fine-needle aspiration is not acceptable) will be collected in all enrolled patients (once at baseline [after the baseline FDG PET] to confirm eligibility and once during the study treatment period), and two core samples will be collected at each occasion. Mandatory biopsies will be collected from all patients enrolled, except for NSCLC patients for whom there is no accessible lesion. The biopsies will be taken from accessible, “non-critical” tumor locations, including, but not limited to, skin, lymph node, rectum, liver, etc. On-treatment tumor biopsies will be randomized within dose schemes as follows: half of the patients at week 3, half of the patients at week 6. All biopsies must be taken after FDG-PET, unless, in the absence of available archival tumor tissue, the baseline sample is being used to assess CEA expression for eligibility in which case the biopsy may be taken before the FDG-PET scan.

Tumor biopsies should be taken predose (and up to 48h before the visit).

If feasible, biopsies may be repeated if the initial biopsy did not contain sufficient tumor material for analysis.

If preliminary data suggest that modification of the on-treatment tumor biopsy timepoint would be more appropriate, alternative on-treatment tumor biopsy timepoints could be considered in the future cohorts.

The baseline and on-treatment biopsies should preferentially be taken from the same tumor lesion to ensure comparability when accessible in a non-critical location. Data from FDG-PET should guide which lesion would be more reflective of capturing the PD effect and thus the baseline and on-treatment biopsies should be taken from that tumor lesion.

For patients continuing in the study, additional biopsies may be taken at the discretion of the investigator. These biopsies may serve to evaluate viable tumor given the mechanism of action of RO6958688 to induce tumor inflammation. For patients discontinuing from the study due to disease progression, additional optional biopsies,

“Use of this document is governed by the terms of use on the first page of this document.”

if clinically feasible, may be taken to aid the understanding of immune resistance mechanisms. Additional biopsies may be taken at the discretion of the investigator to make decisions regarding patient status, and a portion of this biopsy should be sent to Roche (or their designated laboratory) for analysis.

Tumor biopsies will be centrally analyzed for immune cell number and activation by flow cytometric and/or immunohistochemical methods for the density of different immune cell lineages (including but not limited to CD4+, CD8+, B cells, NK cells, macrophages) and their activation and differentiation status (including but not limited to CD25, Ki67, PD1) and for the expression of tumor markers such as PD-L1, IDO etc. In addition, analysis of gene expression, genetic alteration and the infiltrating T cell repertoire may be carried out. The goals of these analyses will be (i) to establish a dose–response and/or an exposure-response relationship and (ii) to understand the MoA of RO6958688 in combination with atezolizumab at the tumor site.

Positron Emission Tomography (PET)

FDG-PET can identify sign of biological effect early, before tumor size is reduced. Moreover, a reduction in the FDG-PET signal within days or weeks of initiating therapy (e.g., in lymphoma, non–small cell lung, and esophageal cancer) has been shown to correlate with prolonged survival and other clinical end points now used (Weber). These findings suggest that FDG-PET could facilitate drug development as an early marker of drug effect.

Whole-body PET should begin 60 ± 10 min after FDG injection. FDG-PET should be performed according to the schedule of assessment in all eligible patients of the study (see FDG PET Appendix 9 for more details).

Archival Tumor Tissue

Formalin–fixed archival tumor tissue embedded in paraffin is to be assessed for CEA expression from all patients for confirmation of CEA status before or at screening (see Appendix 1). These tissues are preferably from the most recent metastatic site (or site of local recurrence) but may be from the primary tumor if metastatic tumor tissue is unavailable. If archival tissue is unavailable, then a fresh tumor biopsy must be obtained for assessment of CEA expression at screening and, if the patient is enrolled. Further other exploratory biomarker assessments, including but not limited to analysis of microsatellite instability and genomic mutations, may be carried out.

If available, formalin–fixed archival tumor tissue embedded in paraffin blocks is to be submitted for exploratory biomarker assessments from all patients enrolled. The tissue block submitted is preferably from the sample used for CEA assessment at screening, if possible. Unstained slides will be accepted if despite all efforts, tumor blocks cannot be obtained.

"Use of this document is governed by the terms of use on the first page of this document."

Clinical Genotyping Samples

A mandatory whole blood sample from all patients of the study will be collected at baseline for DNA extraction. The DNA may be used to determine if genes associated with immune responses including but not limited to chemotaxis, HLA, and immunosuppression affect the pharmacokinetics, pharmacodynamics, efficacy, and safety of RO6958688 in combination with atezolizumab. In addition, the DNA may be used as a reference to identify tumor mutations. These samples will be destroyed after analysis completion and after the results have been checked.

The blood, serum and plasma samples will be destroyed within 2 years after the date of final closure of the clinical database. Archival tumor blocks will be returned. Other residual tissue material (e.g., slides, extracts, on-study blocks, etc.) will be destroyed within 2 years after the final closure of the clinical database unless the patient provides specific consent for the remainder of the tissue sample(s) to be stored for optional exploratory research. If the patient provides consent for optional exploratory research, the tissue samples will be destroyed no later than 15 years after the date of final closure of the clinical database.

For sampling procedures, storage conditions, and shipment instructions, see the separate laboratory manual.

4.5.1.7 Disease-Specific Assessments

Tumor and Response Evaluations

Tumor response will be evaluated according to both RECIST v1.1 and modified RECIST criteria with the use of unidimensional measurement (see Appendix 4 and Appendix 5, respectively). Assessment of CT/MRI scans as tumor assessments will be performed at the sites during the whole study, and in addition centrally by an independent reviewer for prospective and retrospective analysis. Advanced volumetric analyses of CT/MRI will be performed. For central reading analysis, outputs will be produced using immune-related response criteria (Nishino et al. 2014).

Response will be assessed by the investigator on the basis of physical examinations (CT scans or MRI) of chest, abdomen, and pelvis. Ultrasound and X-rays are not acceptable for monitoring target lesions. All measurable disease must be documented at screening and reassessed at each subsequent tumor evaluation. Consistency of consecutive CT scans or MRIs should be ensured during all assessments for each patient; the same method of assessment and the same technique must be used to evaluate lesions throughout the entire study. Use of spiral CT or MRI is required for baseline lesions $<$20 mm and must be documented in medical records and used consistently throughout the study. The same radiographic procedure that is used to define measurable disease sites at screening must be used throughout the study (e.g., the same contrast protocol for CT scans). Tumor measurements should be made by the same investigator/ radiologist for each patient during the study to the extent that this is feasible.

“Use of this document is governed by the terms of use on the first page of this document.”

In case of clinically measurable superficial lesions such as skin lesions, repeated photographs should be used to document tumor response. These photos must include a ruler for documentation purposes.

If more than one method of assessment is used, the most accurate method is selected according to RECIST v1.1 and modified RECIST criteria, respectively, when recording data. At the investigator's discretion, CT scans may be repeated at any time if progressive disease is suspected.

If available, an assessment of tumor growth kinetics will be made by comparing post-treatment scans with the last available pre-study scan.

Modified RECIST Criteria

Modified RECIST (Appendix 5) is derived from RECIST, Version 1.1 conventions and immune-related response criteria (irRC).

Because of a delayed onset of tumor response that may be caused by immunotherapy, in addition to borderline progression, apparent radiologic progression with improving clinical status, or mixed responses, confirmation of progressive disease may be necessary. In the absence of clinical deterioration, any initial assessment of radiological progressive disease or mixed response should be confirmed by a repeat evaluation at the next timepoint for tumor assessment.

As described in Section 4.6, the criteria below are needed for continuation of treatment beyond the initial apparent progressive disease per RECIST v1.1 (e.g., radiological progression secondary to tumor inflammation):

- Absence of clinical deterioration and investigator–assessed potential clinical benefit for the patient and
- The patient is tolerating study drugs.

Scheduling of Tumor Assessments

Tumor assessments will be performed once during screening for all patients. The first assessment after the start of treatment will be done at 8 weeks (C3D15), and the subsequent assessments will continue every 8 weeks thereafter for the first year and every 12 weeks thereafter until disease progression or treatment discontinuation. All tumor assessments after baseline may be done ± 7 days of the scheduled visit.

Baseline total tumor burden must be assessed within a maximum of 4 weeks before the first dose of study drug treatments. If performed within a reasonable time window prior to signature of the Informed Consent Form (ICF), the assessment can be accepted if agreed with the Sponsor and if done as SOC and according to RECIST v1.1 and modified RECIST criteria in a qualified facility in which all further scans for this patient will be performed. Post-baseline assessments must not be delayed (even if administration/cycle days are delayed) and are to be performed as indicated above until

"Use of this document is governed by the terms of use on the first page of this document."

progression, unacceptable toxicity, or withdrawal of consent. The same radiographic procedure used to define measurable and/or evaluable disease at study entry must be used throughout the study. If there is a suspicion of disease progression based on clinical or laboratory findings before the next scheduled assessment, an unscheduled assessment should be performed.

Tumor response will be confirmed a minimum of 28 days after the initial response was noted or at the next scheduled tumor assessment if it is to occur more than 28 days after the initial response.

If a patient inadvertently misses a prescribed tumor evaluation or a technical error prevents the evaluation, the patient may continue treatment until the next assessment and an unscheduled tumor assessment should be planned as soon as possible.

In patients who are not in progression at time of study discontinuation, the same metastatic workup to cover all target and non-target lesions should be performed at the safety follow-up visit.

FDG-PET

[^{18}F]-Fluorodeoxyglucose (FDG) is a radioactive analogue of glucose which is taken up avidly in most malignancies and is used to visualize cancer and its metastases, usually as whole body images.

Patients who have had a hypersensitivity reaction to FDG will be excluded from FDG-PET. However, the patient will be eligible for the remainder of the study. Diabetic patients with high glucose levels (target blood glucose level ≤ 180 mg/dL) that can't be adequately controlled may be excluded from FDG-PET at the discretion of the investigator. PET involves exposure to ionizing radiation. The level of this exposure should be estimated and local guidelines for approval need to be followed.

[^{18}F]-FDG-PET will be performed according to the schedule of assessment as a non-invasive method for determining drugs effect on the metabolic activity of tumor. The patient should fast for 4-6 hours prior to the [^{18}F]-FDG-PET investigation. The patient should rest after the tracer administration and scanning will start 60 minutes ± 10 minutes after tracer administration. The patient is examined with sufficient number of bed positions to ensure coverage over the area from the lower part of the brain (inclusive) to mid-thigh. It is also essential that the patient is examined under the same conditions in baseline and follow up scan, specifically with respect to uptake time and scanning time. Additionally, it is important that the same model of scanner is used or at least that the same spatial resolution is ensured.

Blood glucose concentration needs to be measured immediately prior to the tracer injection and appropriate action needs to be taken for diabetic patients. The scanning procedure should be performed closely to NCI as described in Appendix 9.

"Use of this document is governed by the terms of use on the first page of this document."

[^{18}F]-FDG-PET will be performed at baseline prior to biopsy. It is important to be able to follow the biopsied lesion (selected target lesion as per RECIST) in the PET procedure for screening as well as on study visits. On-study FDG-PET assessment at week 4 (C2D8 + 1 week) and week 16 (C6D8) will be performed concurrent with RECIST assessment whenever possible as shown in the Schedule of assessments Appendix 1.

Patients with no evidence of FDG uptake at the screening PET assessment will not be required to undergo the on-study assessment. For patients with suggestion of PD on follow-up FDG-PET assessments, a confirmatory CT/MRI assessment is strongly encouraged.

Assessment of FDG-PET will be performed centrally by an independent reviewer and per local site assessment.

4.5.1.8 Samples for Roche Clinical Repository

Overview of the Roche Clinical Repository

The Roche Clinical Repository (RCR) is a centrally administered group of facilities for the long-term storage of human biologic specimens, including body fluids, solid tissues, and derivatives thereof (e.g., DNA, RNA, proteins, peptides). The collection and analysis of RCR specimens will facilitate the rational design of new pharmaceutical agents and the development of diagnostic tests, which may allow for individualized drug therapy for patients in the future.

Specimens for the RCR will be collected from patients who give specific consent to participate in this optional research. RCR specimens will be used to achieve the following objectives:

- To study the association of biomarkers with efficacy, adverse events, or disease progression
- To increase knowledge and understanding of disease biology
- To study drugs response, including drugs effects and the processes of drugs absorption and disposition
- To develop biomarker or diagnostic assays and establish the performance characteristics of these assays.

Approval by the Institutional Review Board or Ethics Committee

Collection and submission of biological samples to the RCR is contingent upon the review and approval of the exploratory research and the RCR portion of the Informed Consent Form by each site's Institutional Review Board or Ethics Committee (IRB/EC) and, if applicable, an appropriate regulatory body. If a site has not been granted approval for RCR sampling, this section of the protocol will not be applicable at that site.

“Use of this document is governed by the terms of use on the first page of this document.”

Sample Collection

The following samples will be collected for identification of genetic (inherited) biomarkers:

- Whole blood for extraction of DNA and RNA to examine possible predictive biomarkers, including but not limited to the following: variants in immune genes or other genes of interest.

For all samples, dates of consent and specimen collection should be recorded on the associated RCR page of the eCRF. For sampling procedures, storage conditions, and shipment instructions, see the separate laboratory manual.

Patients will also have the option to consent that any tissue material remaining after protocol defined analysis can be stored for up to 15 years in the RCR

RCR specimens will be destroyed no later than 15 years after the date of final closure of the associated clinical database. The RCR storage period will be in accordance with the IRB/EC‑approved Informed Consent Form and applicable laws (e.g., health authority requirements).

The dynamic biomarker specimens will be subject to the confidentiality standards described in Section 8.4. The genetic biomarker specimens will undergo additional processes to ensure confidentiality, as described below.

Confidentiality

Given the sensitive nature of genetic data, Roche has implemented additional processes to ensure study subject confidentiality for RCR specimens and associated data. Upon receipt by the RCR, each specimen is "double-coded" by replacing the study subject identification number with a new independent number. Data generated from the use of these specimens and all clinical data transferred from the clinical database and considered relevant are also labeled with this same independent number. A "linking key" between the study subject identification number and this new independent number is stored in a secure database system. Access to the linking key is restricted to authorized individuals and is monitored by audit trail. Legitimate operational reasons for accessing the linking key are documented in a standard operating procedure. Access to the linking key for any other reason requires written approval from the Pharma Repository Governance Committee and Roche's Legal Department, as applicable.

Data generated from RCR specimens must be available for inspection upon request by representatives of national and local health authorities, and Roche monitors, representatives, and collaborators, as appropriate.

Patient medical information associated with RCR specimens is confidential and may be disclosed to third parties only as permitted by the Informed Consent Form (or separate authorization for use and disclosure of personal health information) signed by the patient, unless permitted or required by law.

“Use of this document is governed by the terms of use on the first page of this document.”

Data derived from RCR specimen analysis on individual patients will generally not be provided to study investigators unless a request for research use is granted. The aggregate results of any research conducted using RCR specimens will be available in accordance with the effective Roche policy on study data publication.

Any inventions and resulting patents, improvements, and/or know-how originating from the use of the RCR data will become and remain the exclusive and unburdened property of Roche, except where agreed otherwise.

Consent to Participate in the Roche Clinical Repository

The Informed Consent Form will contain a separate section that addresses participation in the RCR. The investigator or authorized designee will explain to each patient the objectives, methods, and potential hazards of participation in the RCR. Patients will be told that they are free to refuse to participate and may withdraw their specimens at any time and for any reason during the storage period. A separate, specific signature will be required to document a patient's agreement to provide optional RCR specimens. Patients who decline to participate will not provide a separate signature.

The investigator should document whether or not the patient has given consent to participate by completing the RCR Research Sample Informed Consent eCRF.

In the event of an RCR participant's death or loss of competence, the participant's specimens and data will continue to be used as part of the RCR research.

Withdrawal from the Roche Clinical Repository

Patients who give consent to provide RCR specimens have the right to withdraw their specimens from the RCR at any time for any reason. After withdrawal of consent, any remaining samples will be destroyed or will no longer be linked to the patient. However, if RCR samples have been tested prior to withdrawal of consent, results from those tests will remain as part of the overall research data. If a patient wishes to withdraw consent to the testing of his or her specimens, the investigator must inform the Medical Monitor in writing of the patient's wishes using the RCR Subject Withdrawal Form and, if the trial is ongoing, must enter the date of withdrawal on the RCR Research Sample Withdrawal of Informed Consent eCRF.

If a patient wishes to withdraw consent to the testing of his or her RCR samples after closure of the site, the investigator must inform the Sponsor by emailing the study number and patient number to the following email address:

global_rcr-withdrawal@roche.com

A patient's withdrawal from Study WP29945 does not, by itself, constitute withdrawal of specimens from the RCR. Likewise, a patient's withdrawal from the RCR does not constitute withdrawal from Study WP29945.

"Use of this document is governed by the terms of use on the first page of this document."

Monitoring and Oversight

RCR specimens will be tracked in a manner consistent with Good Clinical Practice by a quality-controlled, auditable, and appropriately validated laboratory information management system, to ensure compliance with data confidentiality as well as adherence to authorized use of specimens as specified in this protocol and in the Informed Consent Form. Roche monitors and auditors will have direct access to appropriate parts of records relating to patient participation in the RCR for the purposes of verifying the data provided to Roche. The site will permit monitoring, audits, IRB/EC review, and health authority inspections by providing direct access to source data and documents related to the RCR samples.

4.5.2 Timing of Study Assessments

4.5.2.1 Screening and Pretreatment Assessments

Written informed consent for participation in the study must be obtained before performing any study‑specific screening tests or evaluations. Informed Consent Forms for enrolled patient and for patients who are not subsequently enrolled will be maintained at the study site.

All screening and pre-treatment assessments must be completed and reviewed to confirm that patients meet all eligibility criteria. The investigator will maintain a screening log to record details of all patients screened and to confirm eligibility or record reasons for screening failure.

An Eligibility Screening Form (ESF) documenting the investigator’s assessment of each screened patient with regard to the protocol’s inclusion and exclusion criteria is to be completed by the investigator and kept at the investigational site.

Screening and pre-treatment assessments will be performed within 28 days prior to Cycle 1 Day 1 pre-dose unless otherwise specified. Serum pregnancy test will be done 7 days prior to first dose of RO6958688 and/or atezolizumab. Where the clinical significance of an abnormal screening test result (lab or any other tests) is considered uncertain, the test may be repeated. Screening tumor assessments performed in a reasonable time window before ICF signature can be accepted if agreed with Sponsor and if done as standard of care and according to RECIST 1.1 and modified RECIST criteria in a qualified facility in which all further scans for the patient will be performed.

The following will be assessed during Screening (before enrollment); see Schedule of Assessments and Hourly Assessment tables (see Appendix 1):

- Informed consent
- Medical history, including demographics
- Complete physical examination (including height and weight) and ECOG performance status
- Vital signs (including oxygen saturation measured by pulse oximeter – finger clip at rest and exertion)

“Use of this document is governed by the terms of use on the first page of this document.”

- Triplicate 12-lead ECGs
- Hematology
- Biochemistry
- Coagulation
- Urinalysis (dipstick)
- HBV and HCV serology
- Serum pregnancy test (in women with child bearing potential)
- Thyroid-stimulating hormone free T3 (or total T3 for sites where free T3 is not performed) and T4
- Autoantibodies panel
- Primary archival tumor block (if available)
- Assessment of tumor CEA expression (refer to inclusion criteria #13 and #14)
- Tumor assessment
- FDG-PET
- Concomitant medications
- AEs
- Pulmonary function tests (FEV1/VC/TLC and derived parameters and D_{LCO}) for patients with bilateral lung metastasis or patients with lobectomy or pneumonectomy with lung metastases in the remaining lung

For further details for hematology, blood biochemistry, coagulation and urinalysis please see Section 4.5.1.5.

The following will be assessed during Baseline (after enrollment, before first doses); see Schedule of Assessments and Hourly Assessment tables (see Appendix 1):

Clinical genotyping, PK determination, ADA and PD blood/plasma samples and safety assessments. Tumor biopsy can be obtained up to 28 days before first administration of RO6958688 and atezolizumab.

4.5.2.2 Assessments during Treatment

Under no circumstances will patients who enroll in this study and have completed treatment as specified, be permitted to be allocated a new number and re-enroll in the study.

All assessments must be performed as per SoA and Hourly Assessment tables (see Appendix 1). Assessments scheduled on the day of study treatment administration should be performed prior to administration of study treatments, unless otherwise noted in the SoA and Hourly Assessment tables. A 24-h (72-h if during weekend) window prior to drugs administration for hematology, biochemistry, coagulation, urinalysis, and physical examinations/ECOG is allowed.

"Use of this document is governed by the terms of use on the first page of this document."

The following assessments will be performed as indicated in the SoA:

- Tumor Assessment (according to RECIST 1.1 and modified RECIST criteria) evaluated by chest-abdominal CT scan (or MRI). The same radiographic procedure used to define measurable and/or evaluable disease at study entry must be used throughout the study
- FDG-PET assessment
- PK (RO6958688 and atezolizumab)
- PD plasma and serum and additional safety
- PD whole blood (FC)
- ADA (anti-RO6958688 and anti-atezolizumab)

For the following assessments, performed as indicated in the Schedule of Assessments (see Appendix 1), results must be available before dosing (except for sCD25):

- Complete physical exam, vital signs including body temperature, respiratory rate, heart rate, diastolic and systolic BP, oxygen saturation), and weight assessment
- Triplicate 12-lead ECGs (recording must be done prior to PK sampling)
- ECOG performance status
- Hematology
- Biochemistry
- Coagulation
- Urinalysis
- Urine pregnancy test (in women with child bearing potential)
- Thyroid-stimulating hormone free T3 (or total T3 for sites where free T3 is not performed) and T4.

Note that all patients enrolled in the study will be required to stay in the hospital overnight following administration of study drugs on Cycle 1 Day 1.

If a Grade 3 or higher adverse event related to RO6958688 was observed within 24 hours of the previous RO6958688 infusion, an observation period of at least 8 hours (at least 24 hours for Grade 3 IRR/CRS) after completion of the subsequent RO6958688 infusion is required (for further details, see Appendix 1).

For further details for hematology, blood biochemistry, coagulation, and urinalysis, see Section 4.5.1.5.

4.5.2.3 Assessments at Study Completion/Early Termination Visit

Patients who complete the study or discontinue from the study early will be asked to return to the clinic 28 days after the last dose of RO6958688 or atezolizumab for safety follow-up visits. The visit at which response assessment shows progressive disease may be used as the study completion/early termination visit.

“Use of this document is governed by the terms of use on the first page of this document.”

The following assessments should be done at Study Completion/Early Termination Visit (for further details, see Appendix 1):

- Complete physical exam, vital signs (including body temperature, respiratory rate, heart rate, diastolic and systolic BP, oxygen saturation)
- Triplicate 12-lead ECGs (recording must be done prior to PK sampling)
- ECOG performance status
- Hematology
- Biochemistry
- TSH free T3 (or total T3 for sites where free T3 is not performed) and T4
- Coagulation
- Urinalysis
- Serum pregnancy test (in women with child bearing potential)
- PK (RO6958688 and atezolizumab)
- ADA (anti-RO6958688 and anti-atezolizumab)
- Soluble CEA
- PD whole blood
- Tumor Assessment (according to RECIST 1.1 and modified RECIST criteria) evaluated by chest-abdominal CT scan (or MRI). The same radiographic procedure used to define measurable and/or evaluable disease at study entry must be used throughout the study.

4.5.2.4 Follow-Up Assessments

The following assessments should be done at 28-day Safety follow-up visit (for further details, see Appendix 1):

- Complete physical exam, vital signs (including body temperature, respiratory rate, heart rate, diastolic and systolic BP, oxygen saturation)
- Triplicate 12-lead ECGs
- ECOG performance status
- Hematology
- Biochemistry
- TSH free T3 (or total T3 for sites where free T3 is not performed) and T4
- Coagulation
- Urinalysis
- Serum pregnancy test (in women with child bearing potential)
- PK (RO6958688 and atezolizumab)
- ADA (anti-RO6958688 and anti-atezolizumab).
- PD whole blood

"Use of this document is governed by the terms of use on the first page of this document."

Post-study anti-cancer therapy should be collected and reported as appropriate in the eCRF.

Post-study survival follow-up:

The sites will provide to the Sponsor every three months (starting at *Day 28* visit) an update on survival status of each of the patients enrolled in the study. The sites will use designated section of the eCRF for this purpose.

After the study completion/early termination visit, adverse events should be followed as outlined in Sections 5.5 and 5.6.

4.5.2.5 Assessments at Unscheduled Visits

Please see Appendix 1 for assessments that are required to be performed in case of an unscheduled visit.

4.6 PATIENT, STUDY, AND SITE DISCONTINUATION

4.6.1 Patient Discontinuation

Patients have the right to voluntarily withdraw from the study at any time for any reason. In addition, the investigator has the right to withdraw a patient from the study at any time. Reasons for withdrawal from the study may include, but are not limited to, the following:

- Patient withdrawal of consent at any time
- Any medical condition that the investigator or Sponsor determines may jeopardize the patient's safety if he or she continues in the study
- Investigator or Sponsor determines it is in the best interest of the patient
- Patient non-compliance.

Every effort should be made to obtain information on patients who withdraw from the study. The primary reason for withdrawal from the study should be documented on the appropriate eCRF. However, patients will not be followed for any reason after consent has been withdrawn. Patients who withdraw from the study will not be replaced, with the exception of patients during the dose-escalation stage of each arm who withdraw or are withdrawn from the study prior to completing the DLT assessment window for any reason other than a DLT.

All patients will attend a 28-day safety follow-up visit after receiving the last infusion of RO6958688 and atezolizumab.

4.6.1.1 Discontinuation from Study Drugs

Patients will be treated for 24 months or longer if emerging data suggest this, until loss of clinical benefit, unacceptable toxicities, loss of exposure (RO6958688 concentration BLQ or close to LLOQ of 2.0 ng/mL at end of infusion, in which case patients can continue to receive atezolizumab alone), or withdrawal from treatment for other reasons or death.

"Use of this document is governed by the terms of use on the first page of this document."

Patients must discontinue study treatments if they experience any of the following:

- Symptomatic deterioration attributed to disease progression as determined by the investigator after integrated assessment of radiographic data, biopsy results, and clinical status
- Intolerable toxicity related to study treatment, including development of an immune-related adverse event (irAE) determined by the investigator and Medical Monitor to be unacceptable given the individual patient's potential response to therapy and severity of the event (see Section 5.2.6.1)
- Any medical condition that may jeopardize the patient's safety if he or she continues on study treatment
- IRR Grade $\geq$ 3 related to atezolizumab
- IRR/CRS related to RO6958688 meeting any of the following criteria:
    - Grade 3 IRR/CRS that does not recover within 8 hours of corticosteroids and tocilizumab treatment or following repeat treatment with tocilizumab for the same event
    - Grade 4 IRR/CRS
    - Grade 3 IRR/CRS recurrence, or G3 IRR/CRS occurrence after previous G2 IRR/CRS
- AST or ALT increase >10 x ULN
- Grade 4 diarrhea, enteritis, or colitis related to RO6958688
- Grade 2 diarrhea, enteritis, or colitis related to RO6958688 that fails to resolve to Grade 1 or better after following the recommended management guidelines in Appendix 10 for this grade
- Grade 3 diarrhea, enteritis, or colitis related to RO6958688 that fails to resolve to Grade 1 or better after following the recommended management guidelines in Appendix 10 for this grade
- Grade 4 pulmonary event related to RO6958688
- Recurrent Grade 3 pulmonary event related to RO6958688
- IgE-mediated hypersensitivity reactions including anaphylaxis
- Use of another non-protocol anti-cancer therapy (see Section 4.4.2)
- Pregnancy.

"Use of this document is governed by the terms of use on the first page of this document."

Patients will be permitted to continue one or both study treatment(s) after RECIST v1.1 criteria for progressive disease are met if they meet all of the following criteria:

- Evidence of clinical benefit as assessed by the investigator
- Absence of symptoms and signs (including worsening of laboratory values; e.g., new or worsening hypercalcemia) indicating unequivocal progression of disease
- No decline in ECOG performance status that can be attributed to disease progression
- Absence of tumor growth at critical anatomical sites (e.g., leptomeningeal disease) that cannot be managed by protocol-allowed medical interventions
- Patients for whom approved therapies exist must provide written consent to acknowledge deferring these treatment options in favor of continuing study treatment at the time of initial apparent progression.

Patients in whom radiographic disease progression is confirmed at a subsequent tumor assessment may be considered for continued study treatment at the discretion of the investigator if they continue to meet the criteria above and have evidence of clinical benefit (Figure 6). At the time of progression, patients can receive therapies such as: radiotherapy, radio-ablation or surgery.

“Use of this document is governed by the terms of use on the first page of this document.”

Figure 6 Conditions for Continuing RO6958688 and/or Atezolizumab in the Presence of Increased Radiographic Tumor Size

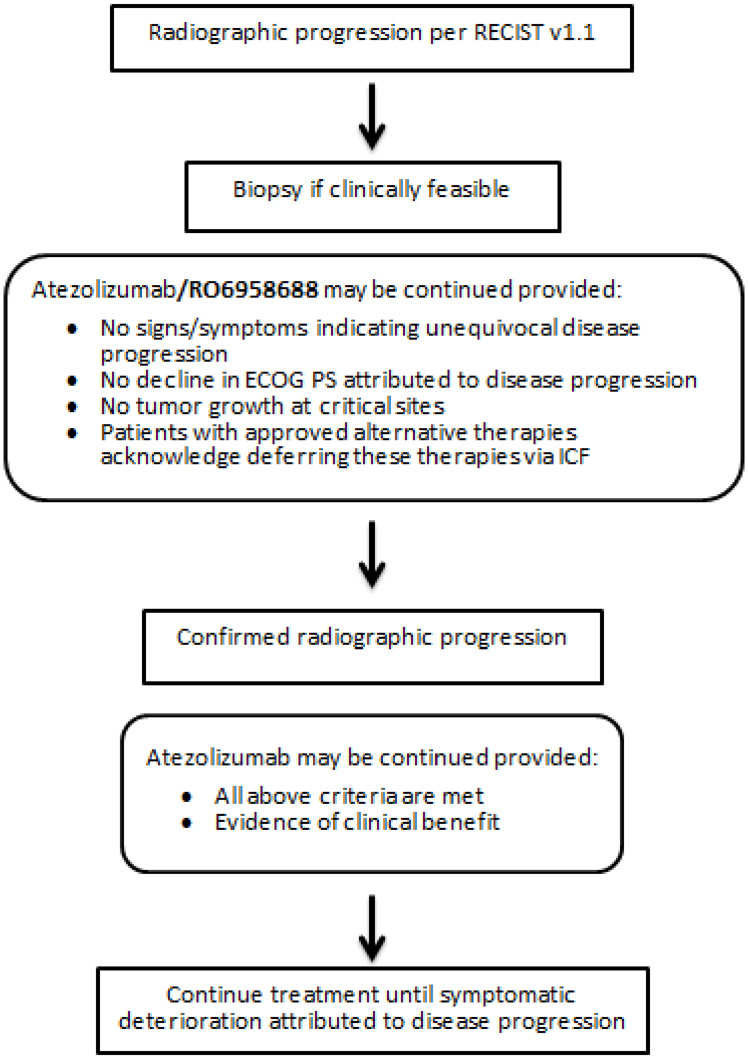

ECOG=Eastern Cooperative Oncology Group; ICF=Informed Consent Form; PS=performance status; RECIST=Response Evaluation Criteria in Solid Tumors.

Patients who discontinue study drugs prematurely will be asked to return to the clinic for a study completion/early termination visit (see Section 4.5.2.3) and may undergo follow-up assessments (see Section 4.5.2.3). The primary reason for premature study drugs discontinuation should be documented on the appropriate eCRF.

"Use of this document is governed by the terms of use on the first page of this document."

4.6.1.2 Withdrawal from Study

Every effort should be made to obtain information on patients who withdraw from the study. The primary reason for withdrawal from the study should be documented on the appropriate eCRF.

If a patient withdraws consent prior to the first treatment administration, the patient will be replaced. Patients will not be followed for any reason after consent has been withdrawn.

During the study, patients who withdraw before the end of the DLT period (i.e., within 21 days of the first dose) for reasons other than DLTs will be replaced to ensure that at least 3 patients have been assessed for a full DLT period prior to moving to the next dose level.

4.6.2 Study and Site Discontinuation

The Sponsor has the right to terminate this study at any time. Reasons for terminating the study may include, but are not limited to, the following:

- The incidence or severity of adverse events in this or other studies indicates a potential health hazard to patients
- Patient enrollment is unsatisfactory.

The Sponsor will notify the investigator and Health Authorities if the study is placed on hold, or if the Sponsor decides to discontinue the study or development program.

The Sponsor has the right to replace a site at any time. Reasons for replacing a site may include, but are not limited to, the following:

- Excessively slow recruitment
- Poor protocol adherence
- Inaccurate or incomplete data recording
- Non-compliance with the International Conference on Harmonisation (ICH) guideline for Good Clinical Practice.

5. ASSESSMENT OF SAFETY

5.1 SAFETY PARAMETERS AND DEFINITIONS

Safety assessments will consist of monitoring and recording adverse events, including serious adverse events and non-serious adverse events of special interest; measurement of protocol-specified safety laboratory assessments; measurement of protocol-specified vital signs, ECGs; and other protocol-specified tests that are deemed critical to the safety evaluation of the study.

Certain types of events require immediate reporting to the Sponsor, as outlined in Sections 5.1.3 and 5.4.

"Use of this document is governed by the terms of use on the first page of this document."

5.1.1 Adverse Events

According to the ICH guideline for Good Clinical Practice, an adverse event is any untoward medical occurrence in a clinical investigation subject administered a pharmaceutical product, regardless of causal attribution. An adverse event can therefore be any of the following:

- Any unfavorable and unintended sign (including an abnormal laboratory finding), symptom, or disease temporally associated with the use of a medicinal product, whether or not considered related to the medicinal product
- Any new disease or exacerbation of an existing disease (a worsening in the character, frequency, or severity of a known condition), except as described in Section 5.3.5.10
- Recurrence of an intermittent medical condition (e.g., headache) not present at baseline
- Any deterioration in a laboratory value or other clinical test (e.g., ECG) that is associated with symptoms or leads to a change in study treatment or concomitant treatment or discontinuation from study drugs
- Adverse events that are related to a protocol-mandated intervention, including those that occur prior to assignment of study treatment (e.g., screening invasive procedures such as biopsies).

5.1.2 Serious Adverse Events (Immediately Reportable to the Sponsor)

A serious adverse event is any adverse event that meets any of the following criteria:

- Fatal (i.e., the adverse event actually causes or leads to death)
- Life threatening (i.e., the adverse event, in the view of the investigator, places the patient at immediate risk of death)

This does not include any adverse event that had it occurred in a more severe form or was allowed to continue might have caused death.

- Requires or prolongs inpatient hospitalization (see Section 5.3.5.11)
- Results in persistent or significant disability/incapacity (i.e., the adverse event results in substantial disruption of the patient's ability to conduct normal life functions)
- Congenital anomaly/birth defect in a neonate/infant born to a mother exposed to study drugs
- Significant medical event in the investigator's judgment (e.g., may jeopardize the patient or may require medical/surgical intervention to prevent one of the outcomes listed above).

"Use of this document is governed by the terms of use on the first page of this document."

The terms “severe” and “serious” are not synonymous. Severity refers to the intensity of an adverse event (rated as mild, moderate, or severe, or according to a pre-defined grading criteria (e.g., NCI CTCAE criteria; see Section 5.3.3); the event itself may be of relatively minor medical significance (such as severe headache without any further findings).

Severity and seriousness need to be independently assessed for each adverse event recorded on the eCRF.

Serious adverse events are required to be reported by the investigator to the Sponsor immediately (i.e., no more than 24 hours after learning of the event; see Section 5.4.2 for reporting instructions).

5.1.3 Non-Serious Adverse Events of Special Interest (Immediately Reportable to the Sponsor)

Non-serious adverse events of special interest are required to be reported by the investigator to the Sponsor immediately (i.e., no more than 24 hours after learning of the event; see Section 5.4.2 for reporting instructions). Adverse events of special interest for this study include the following, depending on causality assessment of the adverse event, if:

1. **Related to RO6958688**

- Cases of an elevated ALT or AST in combination with either an elevated bilirubin or clinical jaundice, as defined in Section 5.3.5.7
- Grade ≥ 2 CRS and IRR
- Grade ≥ 2 hypersensitivity
- Suspected transmission of an infectious agent by the study drugs, as defined below:
    - Any organism, virus, or infectious particle (e.g., prion protein transmitting transmissible spongiform encephalopathy), pathogenic or non-pathogenic, is considered an infectious agent. A transmission of an infectious agent may be suspected from clinical symptoms or laboratory findings that indicate an infection in a patient exposed to a medicinal product. This term applies only when a contamination of the study drugs is suspected.

2. **Related to Atezolizumab**

- Cases of an elevated ALT or AST in combination with either an elevated bilirubin or clinical jaundice, as defined in Section 5.3.5.7

“Use of this document is governed by the terms of use on the first page of this document.”

- Suspected transmission of an infectious agent by the study drugs, as defined below:
    - Any organism, virus, or infectious particle (e.g., prion protein transmitting transmissible spongiform encephalopathy), pathogenic or non-pathogenic, is considered an infectious agent. A transmission of an infectious agent may be suspected from clinical symptoms or laboratory findings that indicate an infection in a patient exposed to a medicinal product. This term applies only when a contamination of the study drugs is suspected.
- Pneumonitis
- Colitis
- Endocrinopathies: diabetes mellitus, pancreatitis, adrenal insufficiency, or hyperthyroidism
- Hepatitis
- Transaminitis Grade ≥ 2 (AST or ALT $>3 \times$ ULN and bilirubin $>2 \times$ ULN) or AST/ALT $>10 \times$ ULN
- Systemic lupus erythematosus
- Neurological: Guillain-Barre syndrome, myasthenia gravis, meningoencephalitis
- Nephritis
- Events suggestive of hypersensitivity, cytokine release syndrome, influenza like illness, systemic inflammatory response syndrome (SIRS), *and* infusion reactions syndrome.

5.2 SAFETY PLAN

5.2.1 Dose Modifications and Delays

The RO6958688 dose should not be escalated beyond 150 mg in any cohorts of the study. Patients who are already at doses higher than 150 mg should not be escalated any further, but can stay at their current RO6958688 dose levels following discussion with the Medical Monitor if the therapy is adequately tolerated.

Should a patient experience a DLT and/or grade ≥ 2 related adverse events in the first or in subsequent cycles or experience the same toxicity of same or higher grade following re-exposure to atezolizumab and RO6958688 combination, the investigator, after discussion with the Sponsor, will have the option to reduce the dose of RO6958688 and/or to delay the RO6958688 dose up to 14 days in the QW part and up to 21 days in the Q3W part to prevent the recurrence of adverse events. This can be done to allow patients who could potentially benefit from the combination of atezolizumab and RO6958688 to remain on the study drugs.

"Use of this document is governed by the terms of use on the first page of this document."

Prior to dosing (Day 1, Day 8 and Day 15 at each cycle), toxicities from previous administration should be resolved in individual patient:

- For treatment-related non-hematological blood parameters abnormalities the values of total albumin, creatinine, GGT, must be resolved to screening baseline values or as defined by NCI CTCAE Grade ≤ 1. Total bilirubin levels must be resolved to screening values or as defined by NCI-CTCAE Grade ≤ 1, AST and/or ALT must be resolved to $\leq 3 \times$ ULN.
- Regarding fever, patients can continue treatment with RO6958688 if fever is Grade ≤ 1.
- Other non-hematological toxicities should be resolved to baseline values or Grade ≤ 1 (or Grade ≤ 2 if considered non-clinically significant by the investigator).
- For hematological blood parameters the blood values of thrombocyte, erythrocyte, and combination of erythrocyte, lymphocyte and thrombocyte must be resolved to screening baseline values or as defined by NCI-CTCAE Grade ≤ 2.

Investigators should contact the Sponsor before the next administration in case a patient experienced a change in blood inflammatory/immune activation parameters like serum CRP, sCD25 and serum ferritin and/or seroconversion of at least one of the autoantibodies (anti-nuclear antibody, anti-double-stranded DNA, cytoplasmic anti-neutrophil cytoplasmic antibody, and perinuclear anti-neutrophil cytoplasmic antibody).

A delay of RO6958688 administration for up to 14 days in the QW part and up to 21 days in the Q3W part will be acceptable to allow for resolution of toxicity NCI CTCAE as described above (with the exception of toxicity considered as non-RO6958688 related). No other delays of RO6958688 administration are foreseen.

There will be no dose reduction for atezolizumab in this study. Patients may temporarily suspend study treatment for up to 105 days beyond the last dose if they experience adverse events that require a dose to be withheld. If atezolizumab is withheld because of adverse events for > 12 weeks beyond the last dose, then the patient will be discontinued from atezolizumab and will be followed up for safety and efficacy as specified in Section 4.6.1.

If, in the judgment of the investigator, the patient is likely to derive clinical benefit from atezolizumab after a hold of >12 weeks, study drug may be restarted with the approval of the Medical Monitor.

If a patient must be tapered off steroids used to treat adverse events, atezolizumab may be withheld for additional time beyond 12 weeks from the last dose until steroids are discontinued or reduced to prednisone dose (or dose equivalent) ≤ 10 mg/day. The acceptable length of interruption will depend on an agreement between the investigator and the Medical Monitor.

"Use of this document is governed by the terms of use on the first page of this document."

Dose interruptions for reason(s) other than toxicity, such as surgical procedures, may be allowed with Medical Monitor approval. The acceptable length of interruption will depend on agreement between the investigator and the Medical Monitor.

It should be noted that in case of a dose delay, the infusions/cycles are not considered as missed but as delayed (visits should be followed as per SoA). In case the delay is ≥ 2 weeks the investigator should contact the Medical Monitor.

Management of atezolizumab-specific adverse events is presented in Appendix 11.

For patients participating in the step up cohorts who experience Grade 3 related adverse events associated with tumor inflammation:

- For patients who are in the step-up phase of the QW – Q3W dosing scheme, patient management should follow the guidelines provided in Appendix 10; the event should be resolved to Grade 1 prior to resuming RO6958688 treatment. The next RO6958688 dose for such patients will be up to or at the same level of RO6958688 dose that caused the event in the dose escalation phase.

- For patients who have reached the flat dose phase (Q3W), the event should be resolved to Grade 1 prior to resuming RO6958688 treatment and the patient should continue with the per-protocol planned dose. However, the Investigator, after discussion with the Sponsor, will have the option to reduce the dose of RO6958688 to a lower dose level or to allow for a more convenient dose/schedule to prevent the recurrence of adverse events and limit toxicity.

For patients participating in the step up cohorts who experience Grade 2 IRR/CRS considered related to RO6958688:

- For patients who are in the step-up phase of the QW – Q3W dosing scheme, patient management should follow the guidelines provided in Appendix 10. The next RO6958688 dose for such patients will be up to or at the same level of the RO6958688 dose that caused the event in the dose escalation phase.

- For patients who have reached the flat dose phase (Q3W), patient management should follow the guidelines provided in Appendix 10. The Investigator, after discussion with the Sponsor, will have the option to reduce the dose of RO6958688 to a lower dose level or to allow for a more convenient dose/schedule to prevent the recurrence of adverse events and limit toxicity.

Further RO6958688 dose reductions may be implemented once safety and toxicity data from the dose escalation Part I have been evaluated.

“Use of this document is governed by the terms of use on the first page of this document.”

5.2.2 Risks Associated with Atezolizumab

Atezolizumab has been associated with risks such as the following: IRRs, *immune-mediated* hepatitis, pneumonitis, colitis, pancreatitis, diabetes mellitus, hypothyroidism, hyperthyroidism, adrenal insufficiency, hypophysitis, myocarditis, Guillain-Barré syndrome, myasthenic syndrome or myasthenia gravis, meningoencephalitis, myositis, and nephritis. *Immune-mediated reactions may involve any organ system and may lead to hemophagocytic lymphohistiocytosis and macrophage activation syndrome (considered to be potential risks for atezolizumab).*

Please refer to Appendix 11 of the current protocol and Section 6 of the atezolizumab Investigator's Brochure for a detailed description of anticipated safety risks for atezolizumab.

5.2.3 Risks Associated with RO6958688

The following adverse events are classified as identified risks associated with RO6958688: pyrexia, IRR, CRS, hypoxia, hypotension, diarrhea, colitis, tumor inflammation/tumor flare events at the tumor site (tumor pain, dyspnea, hypoxia, increased liver enzymes (AST/ALT), blood bilirubin increased, colitis, enteritis), chills, vomiting, rash, skin exfoliation, nausea, dysgeusia, fatigue, arthritis and arthralgia.

Serious adverse drug reactions which are considered expected and associated with RO6958688 are: pyrexia, IRR, CRS, tumor pain, diarrhea, colitis, dyspnea, hypoxia, hypotension, AST increased, ALT increased and blood bilirubin increased.

The following adverse events are classified as potential risks associated with RO6958688: decreased appetite, IgE-mediated hypersensitivity reactions including anaphylaxis, hematotoxicities, and CNS disorders.

An observation period of at least 8 hours is required following completion of the RO6958688 infusion in any treatment administration visit where a Grade 3 or higher adverse event (with the exception of Grade 3 IRR/CRS) was observed within 24 hours of the previous RO6958688 administration. In a situation where the patient experienced a Grade 3 IRR/CRS in the previous administration visit, a 24-hour hospitalization will be required following the completion of RO6958688 infusion at the next administration visit.

For detailed information regarding identified and potential risks associated with RO6958688, refer to Section 6 of the most recent version of the RO6958688 Investigator's Brochure.

For management of specific adverse events, refer to Section 5.2.6.2.

"Use of this document is governed by the terms of use on the first page of this document."

5.2.4 Risks Associated with Combination Use of RO6958688 and Atezolizumab

The following adverse events are classified as identified risks associated with use of RO6958688 in combination with atezolizumab: conjunctivitis, diarrhea and colitis.

The following adverse events are potential risks associated with combination use of atezolizumab and RO6958688, based on the evidence from Study WP29945: IRR, dysphonia, paraesthesia, musculoskeletal pain, pulmonary events, hepatic events, and GI events (e.g., vomiting, nausea, and gastrointestinal pain).

Based on the mode of action and the nonclinical and/or clinical studies with each molecule as a single agent, the evidence indicates a potential for overlapping toxicity in patients treated with a combination of atezolizumab and RO6958688. Heightened anti-tumor T-cell activity locally in the tumor microenvironment represents the primary single-agent adverse events common to both molecules (see Section 6 of the Atezolizumab and RO6958688 Investigator's Brochures, respectively).

No nonclinical toxicology studies have been conducted with the combination of atezolizumab with RO6958688.

5.2.5 Risks Associated with Tocilizumab

Refer to Section 6 of the Tocilizumab Investigator's Brochure for a detailed description of anticipated safety risks for tocilizumab. Because the use of tocilizumab remains experimental in this protocol, it should be noted that the risks contained in the Tocilizumab Investigator's Brochure have been identified in patients treated in alternate settings and for different indications.

5.2.6 Management of Specific Adverse Events

5.2.6.1 Management of Atezolizumab-Specific Adverse Events

Guidelines for the management of atezolizumab-specific adverse events are outlined in Appendix 11.

Toxicities associated or possibly associated with atezolizumab treatment should be managed according to standard medical practice. Additional tests, such as autoimmune serology or biopsies, should be used to determine a possible immunogenic etiology.

Although most immune-mediated adverse events observed with immunomodulatory agents have been mild and self-limiting, such events should be recognized early and treated promptly to avoid potential major complications. Discontinuation of atezolizumab may not have an immediate therapeutic effect. Refer to Appendix 11 for specific management guidelines.

The investigator should consider the benefit-risk balance a given patient may be experiencing prior to further administration of atezolizumab. Atezolizumab should be

"Use of this document is governed by the terms of use on the first page of this document."

permanently discontinued in patients with life-threatening immune- related adverse events.

5.2.6.1.1 Management of Infusion-Related Reactions

Administration of therapeutic antibodies such as atezolizumab may cause IRRs characterized by symptoms such as fever, chills, dizziness, hypertension, hypotension, dyspnea, restlessness, sweating, flushing, skin rash, tachycardia, tachypnoea, headache, tumor pain, nausea, and/or vomiting. Such reactions typically occur during or shortly after an infusion, or within 24 h after study drugs infusion predominantly at the first infusion. The incidence and severity typically decrease with subsequent infusions. Patients may also develop IgE-mediated hypersensitivity reactions to atezolizumab. IRRs may be indistinguishable from an anaphylactic reaction.

However, if a patient experiences an isolated episode of e.g **"**fever**"** within 24 h after study drugs infusion, not accompanied by other IRR-like symptoms as described above, then the AE reporting term will be fever and not IRR.

If an IRR is considered related to atezolizumab, the infusion of atezolizumab should be managed as per Appendix 11. The patient should be monitored until complete resolution of the symptoms and treated as clinically indicated. Treatment or concomitant medication may include acetaminophen/paracetamol, antihistamine, IV saline, oxygen, bronchodilators, corticosteroids, and vasopressors depending on the symptoms. The infusion rate can be re-escalated to initial rate if considered well tolerated after 1 hour of infusion.

IRR related symptoms management should be symptomatic treatment including corticosteroids when required, see Appendix 11 IRR management guidelines.

5.2.6.2 Management of Specific Adverse Events to RO6958688

Refer to the RO6958688 Investigator's Brochure for additional information on the clinical safety of RO6958688.

5.2.6.2.1 Pyrexia

Investigators should provide guidance to patients for the management of isolated (no other signs and symptoms) episode of fever, in particular patient should regularly check their body temperature during the days following each RO6958688 administration and take early intervention with standard anti-pyrexia treatments (e.g., paracetamol, NSAIDs) as current practice. Occasionally, fever is accompanied with symptoms like chills, hypotension, shortness of breath, skin rash, headache, nausea, and/or vomiting, in which case this is considered an IRR/CRS and should be managed with urgency (Appendix 10).

"Use of this document is governed by the terms of use on the first page of this document."

5.2.6.2.2 Adverse Events Associated with Tumor Inflammation/Tumor Flare

Clinical evidence suggests that certain types of adverse events related to RO6958688 may be mediated by its mechanism of action at the tumor site (i.e., intratrumor immune activation and consequent tumor inflammation and flare). These adverse events tend to have a short onset following RO6958688 administration and affect organ systems with tumor lesions.

Tumor inflammation and tumor flare events are either associated with pain at the tumor site (e.g., tumor, abdominal, and GI pain) or affect function of the organ system with the tumor lesion (e.g., dyspnea, hypoxia, colitis, enteritis, and increased levels of liver function enzymes and bilirubin). These adverse events are more common after the first or second dose of RO6958688.

Guidelines for management of patients who experience adverse events associated with tumor inflammation and tumor flare are provided in Appendix 10.

5.2.6.2.3 Management of Gastrointestinal Toxicities: Diarrhea and Colitis

Diarrhea has been commonly observed in patients receiving RO6958688. Diarrhea events are usually transient and reversible. Diarrhea can be one of the manifestations of CRS, and dehydration secondary to diarrhea can worsen the hypotension associated with CRS. Patients should be closely monitored (including monitoring of renal function) and should be hydrated if clinically indicated to prevent renal insufficiency due to fluid depletion. Diarrhea can also be a symptom of undiagnosed colitis. All events of diarrhea or colitis should be thoroughly evaluated for other etiologies.

Guidelines for management of patients who experience diarrhea and colitis are provided in Appendix 10. Even though CTCAE v5 criteria for CRS is driven by the presence of hypotension and hypoxia, please consider use of tocilizumab per CRS management guidelines (Appendix 10) if per investigator clinical judgement diarrhea is deemed secondary to CRS.

5.2.6.2.4 Management of IRR/CRS

Administration of RO6958688 may cause a spectrum of infusion-related adverse events involving IRRs and CRS. The incidence and severity typically decrease with subsequent infusions, although instances of CRS have been reported in later cycles, as described below.

Infusion-related reactions associated with RO6958688 are typically seen with the first two cycles and without symptoms of hypoxia and/or hypotension. In contrast, cytokine-release syndrome is more likely to be associated with symptoms of hypoxia and/or hypotension along with other constitutional symptoms and can occur at any cycle. Please refer to Section 5.3.5 for guidance on recording and Section 5.4.2 for guidance on reporting of infusion-related reaction and cytokine-release syndrome events.

"Use of this document is governed by the terms of use on the first page of this document."

Given the overlap in signs and symptoms, IRRs may be indistinguishable from CRS, which is defined as a disorder characterized by fever, tachypnea, headache, tachycardia, hypotension, rash, and/or hypoxia caused by the release of cytokines (CTCAE v5). Severe CRS may be associated with other clinical sequelae such as disseminated intravascular coagulation and capillary leak syndrome.

In Study BP29541, as of 23 March 2018, there was one case of Grade 5 IRR that met the criteria of CRS based on symptomatology and cytokine levels, as described below:

A [redacted] year old [redacted] patient enrolled in study BP29541 was reported to have suffered a Grade 5 IRR following the fifth administration of RO6958688 at a dose of 355.99 mg (planned dose 600 mg). The patient is suspected to have had a fatal CRS event following multiple re-treatment attempts with RO6958688 based on a very high serum IL-6 level of 75198.41 pg/mL, elevated levels for IFN-γ (1694.96 pg/mL), IL-8 (4328.17 pg/mL) and TNF-α (767.06 pg/mL) after the fourth administration, with a very similar clinical course at the fifth administration of RO6958688 (Cycle 5 cytokine levels will not be available). This patient had a recurring symptom of Grade 3 hypotension related to RO6958688 infusion at both administrations.

In view of the above data, and given that IRRs may be indistinguishable from CRS based on symptomatology, single treatment guidelines are being recommended for management of IRRs and CRS, during or up to 24 hours after infusion of RO6958688 (Appendix 10, Table 2 and Section 1.5). The approach to have single treatment guidelines for IRR and CRS will continue to be reviewed as further safety data is being accumulated.

Table 3 Cytokine Release Syndrome Grading According to CTCAE v5

Grade 1	Grade 2	Grade 3	Grade 4	Grade 5
Fever with or without constitutional symptoms*	Hypotension responding to fluids; hypoxia responding to <40% O2	Hypotension managed with one pressor; hypoxia requiring ≥ 40% O2	Life-threatening consequences; urgent intervention indicated	Death
Definition: A disorder characterized by fever, tachypnea, headache, tachycardia, hypotension, rash, and/or hypoxia caused by the release of cytokines.				
Navigational Note: Also consider reporting other organ dysfunctions including neurological toxicities such as: Psychiatric disorders: Hallucinations or Confusion; Nervous system disorders: Seizure, Dysphasia, Tremor, or Headache				

*e.g., rigors, malaise, fatigue, anorexia, myalgia, arthralgia, nausea, vomiting and headache

"Use of this document is governed by the terms of use on the first page of this document."

The risk of IRRs/CRS is managed in the clinic with appropriate risk minimization measures. RO6958688 should only be administered under the close supervision of an experienced clinician trained to monitor medical situations and to respond to medical emergencies in a clinical environment with full resuscitation equipment available for immediate use. Patients should receive full supportive care to treat IRRs/CRS according to institutional practice. In clinical studies with RO6958688, patients are required to stay overnight in the hospital after the first dose for clinical monitoring. If infusion-associated signs/symptoms or CRS manifestations occur, patients should be monitored until complete resolution. If a IRRs/CRS develops during the administration of RO6958688, the infusion should be temporarily slowed down or interrupted. Treatment or concomitant medication may include acetaminophen/paracetamol, antihistamine, IV saline, oxygen, bronchodilators, corticosteroids, vasopressors and tocilizumab depending on the symptoms and the severity. Resuscitation equipment should be available for immediate use.

In addition, to reduce the risk of the development of IRRs/CRS in a patient receiving RO6958688, premedication will be administered as per Section 4.3.2.5.

In a situation where the patient experienced a Grade 3 IRR/CRS in the previous administration visit, a 24-hour hospitalization will be required at the completion of the subsequent treatment administration.

5.2.6.2.5 Management of IgE-mediated Hypersensitivity Reactions Including Anaphylaxis

Patients may also develop IgE-mediated hypersensitivity reactions to RO6958688. The signs and symptoms of IRRs/CRS may be indistinguishable from an anaphylactic reaction. Medications including epinephrine, corticosteroids, diphenhydramine hydrochloride for IV injection, and resuscitation equipment should be available for immediate use. Guidelines for management of IRRs/CRS related to RO6958688 are summarized in Appendix 10. Guidelines for management of anaphylaxis are given in Appendix 14.

Patients with IRRs/CRS of Grade 2 or higher, should undergo special laboratory assessments including IgE and tryptase analysis (Appendix 1, Table A3). If the results support an allergic origin (i.e., increased tryptase levels and/or increased IgE levels), the reaction should be reported as an allergic reaction or anaphylactic reaction, as appropriate, and patients should stop treatment with RO6958688.

5.2.6.2.6 Elevated Liver Enzymes and Hepatotoxicity

Transient AST and ALT elevations have been observed in patients receiving RO6958688 and could occur in the setting of tumor inflammation/tumor flare.

“Use of this document is governed by the terms of use on the first page of this document.”

These events tend to occur predominantly after the first two infusions of RO6958688 in patients with liver lesions. AST and ALT elevations that occur after the third infusion (or later) are potentially a consequence of cytokine-release syndrome.

Please use guidelines in Appendix 10, Table 1 to manage AST/ALT elevation secondary to tumor inflammation and Appendix 10, Table 2 to manage AST/ALT elevation occurring as a consequence of CRS.

5.2.6.3 Management of Adverse Events Specific to Atezolizumab and to RO6958688

The attribution and management of certain adverse events that have been associated with each agent separately (e.g., pulmonary events of dyspnea and hypoxia, hepatic events, GI events) may be ambiguous when the agents are administered together. It is theoretically possible that allergic or inflammatory adverse events associated with RO6958688 symptoms could be exacerbated by the immunostimulatory activity of atezolizumab.

Toxicities should initially be managed according to the recommendations in Appendix 10 with dose holds applied to the component of the study treatment judged to be the primary cause. If an individual component for causality for the toxicity cannot be adequately determined, then the most conservative management recommendation should be applied.

5.2.6.3.1 Gastrointestinal Toxicities: Diarrhea and Colitis

Diarrhea that occurs during treatment with RO6958688 in combination with atezolizumab could be secondary to RO6958688-associated IRR and CRS, tumor inflammation, or an atezolizumab immune-mediated toxicity.

Colitis that occurs during treatment with RO6958688 in combination with atezolizumab could be secondary to RO6958688-associated tumor inflammation, or an atezolizumab immune-mediated toxicity.

Enteritis can also be a consequence of RO6958688-associated tumor inflammation.

Guidelines for management of patients who experience diarrhea and colitis are provided in Appendix 10.

5.2.6.3.2 Hepatotoxicity

Immune-mediated hepatitis has been associated with the administration of atezolizumab. Hepatic toxicity can also occur with RO6958688 either secondary to tumor inflammation or as a part of CRS. RO6958688-associated hepatic toxicity is usually transient compared with immune-mediated hepatitis that is associated with atezolizumab (which has a median duration of 2.1 months).

“Use of this document is governed by the terms of use on the first page of this document.”

Guidelines for management of patients who experience hepatotoxicity are provided in Appendix 10.

5.2.6.3.3 Pulmonary Toxicity

Immune-mediated pneumonitis has been associated with the administration of atezolizumab. Pulmonary toxicity (dyspnea, hypoxia or respiratory failure) can also occur with RO6958688 secondary to tumor inflammation or as a part of CRS.

Guidelines for management of patients who experience pulmonary toxicities are provided in Appendix 10.

5.2.6.3.4 Conjunctivitis

Conjunctivitis is not considered to be the risk that affects the benefit–risk profile of the combination or that requires active management given that events have been of mild severity and are manageable with current clinical practice.

5.3 METHODS AND TIMING FOR CAPTURING AND ASSESSING SAFETY PARAMETERS

The investigator is responsible for ensuring that all adverse events (see Section 5.1 for definition) are recorded on the Adverse Event eCRF and reported to the Sponsor in accordance with instructions provided in this section and in Sections 5.4-5.6.

For each adverse event recorded on the Adverse Event eCRF, the investigator will make an assessment of seriousness (see Section 5.1.2 for seriousness criteria), severity (see Section 5.3.3), and causality (see Section 5.3.4).

5.3.1 Adverse Event Reporting Period

Investigators will seek information on adverse events at each patient contact. All adverse events, whether reported by the patient or noted by study personnel, will be recorded in the patient's medical record. Adverse events will then be reported on the Adverse Event eCRF as follows:

After informed consent has been obtained but prior to initiation of study drugs, only serious adverse events caused by a protocol-mandated intervention should be reported (e.g., serious adverse events related to invasive procedures such as biopsies).
Any other adverse event should not be reported.

After initiation of study drugs, all adverse events, regardless of relationship to study drugs, will be reported until 28 days (for RO6958688) or 120 days (for atezolizumab) after the last dose of study drugs or until initiation of new systemic anti-cancer therapy, whichever occurs first.

After a period of 28 days or 120 days from the last dose, investigators should report any deaths, serious adverse events, or other adverse events of concern that are believed to be related to prior treatment with study drugs (see Section 5.6).

"Use of this document is governed by the terms of use on the first page of this document."

5.3.2 Eliciting Adverse Event Information

A consistent methodology of non-directive questioning should be adopted for eliciting adverse event information at all patient evaluation time-points. Examples of non-directive questions include the following:

"How have you felt since your last clinic visit?"

"Have you had any new or changed health problems since you were last here?"

5.3.3 Assessment of Severity of Adverse Events

The adverse event severity grading scale for the NCI CTCAE (v4.03) will be used for assessing adverse event severity (NCI CTCAE [v5] will be used for CRS). Table 4 will be used for assessing severity for adverse events that are not specifically listed in the NCI CTCAE.

Table 4 Adverse Event Severity Grading Scale

Grade	Severity
1	Mild; asymptomatic or mild symptoms; clinical or diagnostic observations only; or intervention not indicated.
2	Moderate; minimal, local, or non-invasive intervention indicated; or limiting age-appropriate instrumental activities of daily living [a].
3	Severe or medically significant, but not immediately life-threatening; hospitalization or prolongation of hospitalization indicated; disabling; or limiting self-care activities of daily living [b,c].
4	Life-threatening consequences or urgent intervention indicated [d].
5	Death related to adverse event [d].

NCI CTCAE = National Cancer Institute Common Terminology Criteria for Adverse Events.

Note: Based on the NCI CTCAE (v4.03) (except for CRS, which will be based on NCI CTCAE v5), which can be found at: https://evs.nci.nih.gov/ftp1/CTCAE/CTCAE_4.03/CTCAE_4.03_2010-06-14_QuickReference_8.5x11.pdf or https://ctep.cancer.gov/protocolDevelopment/electronic_applications/docs/CTCAE_v5_Quick_Reference_5x7.pdf

[a] Instrumental activities of daily living refer to preparing meals, shopping for groceries or clothes, using the telephone, managing money, etc.

[b] Examples of self-care activities of daily living include bathing, dressing and undressing, feeding one's self, using the toilet, and taking medications, as performed by patients who are not bedridden.

[c] If an event is assessed as a "significant medical event," it must be reported as a serious adverse event (see Section 5.4.2 for reporting instructions), per the definition of serious adverse event in Section 5.1.2.

[d] Grade 4 and 5 events must be reported as serious adverse events (see Section 5.4.2 for reporting instructions), per the definition of serious adverse event in Section 5.1.2.

CRS severity will be graded according to the CRS NCI CTCAE v5 for events that are considered related to RO6958688 only.

"Use of this document is governed by the terms of use on the first page of this document."

5.3.4 Assessment of Causality of Adverse Events

Investigators should use their knowledge of the patient, the circumstances surrounding the event, and an evaluation of any potential alternative causes to determine whether or not an adverse event is considered to be related to the study drugs, indicating "yes" or "no" accordingly. The following guidance should be taken into consideration:

- Temporal relationship of event onset to the initiation of study drugs
- Course of the event, considering especially the effects of dose reduction, discontinuation of study drugs, or reintroduction of study drugs (where applicable)
- Known association of the event with the study drugs or with similar treatments
- Known association of the event with the disease under study
- Presence of risk factors in the patient or use of concomitant medications known to increase the occurrence of the event
- Presence of non-treatment-related factors that are known to be associated with the occurrence of the event.

For patient receiving combination therapy, causality will be assessed individually for each protocol-mandated therapy.

5.3.5 Procedures for Recording Adverse Events

Investigators should use correct medical terminology/concepts when recording adverse events on the Adverse Event eCRF. Avoid colloquialisms and abbreviations.

Only one adverse event term should be recorded in the event field on the Adverse Event eCRF.

5.3.5.1 Infusion-Related Reactions/Hypersensitivity Reactions and Cytokine Release Syndrome Attributed to RO6958688

As IRRs and CRS may be indistinguishable from one another, the following procedure should be followed to record IRR/CRS events considered related to RO6958688:

- All infusion-related events occurring within 24 hours of the first two infusions of RO6958688 should be recorded as IRR unless hypotension and/or hypoxia is one of the predominant symptoms
- All infusion-related events occurring within 24 hours of RO6958688 infusion with hypotension as the predominant symptom, and infusion related events associated with 3rd infusion of RO6958688 or later should be recorded as CRS. Adverse events of CRS considered related to RO695688 will be graded according to CTCAE v5.)
- The one exception to this reporting guidance is if a clinical presentation suggests an immediate, acute hypersensitivity (e.g., generalized hives, mucosal edema, with or without wheezing and hypotension). Elevated IgE and/or tryptase levels support an allergic origin. In this case a diagnosis of “allergic reaction” or “hypersensitivity reaction” or “anaphylaxis” should be used.

“Use of this document is governed by the terms of use on the first page of this document.”

For adverse events with a diagnosis of IRR/CRS associated signs, symptoms, and laboratory abnormalities should be recorded on the dedicated Infusion-Related Reaction/Cytokine Release Syndrome eCRF. Each IRR/CRS should be recorded separately on the Adverse Event eCRF, with signs, symptoms, and laboratory abnormalities also recorded separately on the dedicated Infusion-Related Reaction/Cytokine Release Syndrome eCRF. Ambiguous terms such as "systemic reaction" should be avoided. In addition to documentation in the Adverse Event eCRF, non-serious Grade ≥ 2 CRS and IRR events should be reported as a non-serious adverse event of special interest (see Section 5.1.3).

Adverse events of IRR considered related to another study drug, and not RO6958688, will be graded using CTCAE v4.03.

5.3.5.2 Other Adverse Events

A diagnosis (if known) should be recorded on the Adverse Event eCRF rather than individual signs and symptoms (e.g., record only liver failure or hepatitis rather than jaundice, asterixis, and elevated transaminases). However, if a constellation of signs and/or symptoms cannot be medically characterized as a single diagnosis or syndrome at the time of reporting, each individual event should be recorded on the Adverse Event eCRF. If a diagnosis is subsequently established, all previously reported adverse events based on signs and symptoms should be nullified and replaced by one adverse event report based on the single diagnosis, with a starting date that corresponds to the starting date of the first symptom of the eventual diagnosis.

5.3.5.3 Adverse Events Occurring Secondary to Other Events

In general, adverse events occurring secondary to other events (e.g., cascade events or clinical sequelae) should be identified by their primary cause, with the exception of severe or serious secondary events. However, medically significant adverse events occurring secondary to an initiating event that are separated in time should be recorded as independent events on the Adverse Event eCRF. For example:

- If vomiting results in mild dehydration with no additional treatment in a healthy adult, only vomiting should be reported on the eCRF.
- If vomiting results in severe dehydration, both events should be reported separately on the eCRF.
- If a severe gastrointestinal hemorrhage leads to renal failure, both events should be reported separately on the eCRF.
- If dizziness leads to a fall and subsequent fracture, all three events should be reported separately on the eCRF.

All adverse events should be recorded separately on the Adverse Event eCRF if it is unclear as to whether the events are associated.

“Use of this document is governed by the terms of use on the first page of this document.”

5.3.5.4 Persistent or Recurrent Adverse Events

A persistent adverse event is one that extends continuously, without resolution, between patient evaluation time‑points. Such events should only be recorded once on the Adverse Event eCRF. The initial severity of the event should be recorded, and the severity should be updated to reflect the most extreme severity any time the event worsens. If the event becomes serious, the Adverse Event eCRF should be updated to reflect this.

A recurrent adverse event is one that resolves between patient evaluation time‑points and subsequently recurs. Each recurrence of an adverse event should be recorded separately on the Adverse Event eCRF.

5.3.5.5 Abnormal Laboratory Values

Not every laboratory abnormality qualifies as an adverse event. A laboratory test result should be reported as an adverse event if it meets any of the following criteria:

- Accompanied by clinical symptoms
- Results in a change in study treatment (e.g., dosage modification, treatment interruption, or treatment discontinuation)
- Results in a medical intervention (e.g., potassium supplementation for hypokalemia) or a change in concomitant therapy
- Clinically significant in the investigator's judgment.

It is the investigator's responsibility to review all laboratory findings. Medical and scientific judgment should be exercised in deciding whether an isolated laboratory abnormality should be classified as an adverse event.

If a clinically significant laboratory abnormality is a sign of a disease or syndrome (e.g., alkaline phosphatase and bilirubin 5 times the upper limit of normal (ULN) associated with cholecystitis), only the diagnosis (i.e., cholecystitis) should be recorded on the Adverse Event eCRF.

If a clinically significant laboratory abnormality is not a sign of a disease or syndrome, the abnormality itself should be recorded on the Adverse Event eCRF, along with a descriptor indicating if the test result is above or below the normal range (e.g., "elevated potassium", as opposed to "abnormal potassium"). If the laboratory abnormality can be characterized by a precise clinical term per standard definitions, the clinical term should be recorded as the adverse event. For example, an elevated serum potassium level of 7.0 mEq/L should be recorded as "hyperkalemia".

Observations of the same clinically significant laboratory abnormality from visit to visit should not be repeatedly recorded on the Adverse Event eCRF, unless the etiology changes. The initial severity of the event should be recorded, and the severity or seriousness should be updated any time the event worsens.

"Use of this document is governed by the terms of use on the first page of this document."

5.3.5.6 Abnormal Vital Sign Values

Not every vital sign abnormality qualifies as an adverse event. A vital sign result should be reported as an adverse event if it meets any of the following criteria:

- Accompanied by clinical symptoms
- Results in a change in study treatment (e.g., dosage modification, treatment interruption, or treatment discontinuation)
- Results in a medical intervention or a change in concomitant therapy
- Clinically significant in the investigator's judgment.

It is the investigator's responsibility to review all vital sign findings. Medical and scientific judgment should be exercised in deciding whether an isolated vital sign abnormality should be classified as an adverse event.

If a clinically significant vital sign abnormality is a sign of a disease or syndrome (e.g., high blood pressure), only the diagnosis (i.e., hypertension) should be recorded on the Adverse Event eCRF.

Observations of the same clinically significant vital sign abnormality from visit to visit should not be repeatedly recorded on the Adverse Event eCRF, unless the etiology changes. The initial severity of the event should be recorded, and the severity or seriousness should be updated any time the event worsens.

5.3.5.7 Abnormal Liver Function Tests

The finding of an elevated ALT or AST ($> 3 \times$ ULN) in combination with either an elevated total bilirubin ($> 2 \times$ ULN) or clinical jaundice in the absence of cholestasis or other causes of hyperbilirubinemia is considered to be an indicator of severe liver injury. Therefore, investigators must report as an adverse event the occurrence of either of the following:

- Treatment-emergent ALT or AST $> 3 \times$ ULN in combination with total bilirubin $> 2 \times$ ULN
- Treatment-emergent ALT or AST $> 3 \times$ ULN in combination with clinical jaundice.

The most appropriate diagnosis or (if a diagnosis cannot be established) the abnormal laboratory values should be recorded on the Adverse Event eCRF (see Section 5.3.5.1) and reported to the Sponsor immediately (i.e., no more than 24 hours after learning of the event), either as a serious adverse event or a non-serious adverse event of special interest (see Section 5.4.2).

5.3.5.8 Deaths

For this protocol, mortality is an efficacy endpoint. Deaths that occur during the protocol-specified adverse event reporting period (see Section 5.3.1) that are attributed by the investigator solely to progression of cancer should be recorded on the Death Attributed to Progressive Disease eCRF. All other on-study deaths, regardless of

"Use of this document is governed by the terms of use on the first page of this document."

relationship to study drugs, must be recorded on the Adverse Event eCRF and immediately reported to the Sponsor (see Section 5.4).

Death should be considered an outcome and not a distinct event. The event or condition that caused or contributed to the fatal outcome should be recorded as the single medical concept on the Adverse Event eCRF. Generally, only one such event should be reported. The term “sudden death” should only be used for the occurrence of an abrupt and unexpected death due to presumed cardiac causes in a patient with or without preexisting heart disease, within 1 hour of the onset of acute symptoms or, in the case of an unwitnessed death, within 24 hours after the patient was last seen alive and stable. If the cause of death is unknown and cannot be ascertained at the time of reporting, “unexplained death” should be recorded on the Adverse Event eCRF. If the cause of death later becomes available (e.g., after autopsy), “unexplained death” should be replaced by the established cause of death.

During post-study survival follow-up, deaths attributed to progression of cancer should be recorded only on the Survival eCRF.

5.3.5.9 Preexisting Medical Conditions

A preexisting medical condition is one that is present at the screening visit for this study. Such conditions should be recorded on the General Medical History and Baseline Conditions eCRF.

A preexisting medical condition should be recorded as an adverse event only if the frequency, severity, or character of the condition worsens during the study. When recording such events on the Adverse Event eCRF, it is important to convey the concept that the preexisting condition has changed by including applicable descriptors (e.g., “more frequent headaches”).

5.3.5.10 Lack of Efficacy or Worsening of Cancer

Events that are clearly consistent with the expected pattern of progression of the underlying disease should not be recorded as adverse events. These data will be captured as efficacy assessment data only. In most cases, the expected pattern of progression will be based on RECIST v1.1 criteria and modified RECIST criteria. In rare cases, the determination of clinical progression will be based on symptomatic deterioration. However, every effort should be made to document progression using objective criteria. If there is any uncertainty as to whether an event is due to disease progression, it should be reported as an adverse event.

5.3.5.11 Hospitalization or Prolonged Hospitalization

Any adverse event that results in hospitalization or prolonged hospitalization should be documented and reported as a serious adverse event (per the definition of serious adverse event in Section 5.1.2), except as outlined below.

“Use of this document is governed by the terms of use on the first page of this document.”

The following hospitalization scenarios are <u>not</u> considered to be serious adverse events:

- Hospitalization for respite care
- Planned hospitalization required by the protocol (e.g., for study drugs administration or insertion of access device for study drugs administration)
- Hospitalization for a preexisting condition, provided that all of the following criteria are met:
    - The hospitalization was planned prior to the study or was scheduled during the study when elective surgery became necessary because of the expected normal progression of the disease
    - The patient has not suffered an adverse event.
- Hospitalization due solely to progression of the underlying cancer.

The following hospitalization scenarios are not considered to be serious adverse events, but should be reported as adverse events instead:

- Hospitalization for an adverse event that would ordinarily have been treated in an outpatient setting had an outpatient clinic been available

5.3.5.12 Cases of Accidental Overdose or Medication Error

Accidental overdose and medication error (hereafter collectively referred to as "special situations"), are defined as follows:

- Accidental overdose: accidental administration of a drug in a quantity that is higher than the assigned dose
- Medication error: accidental deviation in the administration of a drug

  In some cases, a medication error may be intercepted prior to administration of the drug.

Special situations are not in themselves adverse events, but may result in adverse events. Each adverse event associated with a special situation should be recorded separately on the Adverse Event eCRF. If the associated adverse event fulfills seriousness criteria, the event should be reported to the Sponsor immediately (i.e., no more than 24 hours after learning of the event; see Section 5.4.2). For atezolizumab, RO6958688, or tocilizumab, adverse events associated with special situations should be recorded as described below for each situation:

- Accidental overdose: Enter the drug name and "accidental overdose" as the event term. Check the "Accidental overdose" and "Medication error" boxes.
- Medication error that does not qualify as an overdose: Enter the adverse event term. Check the "Medication error" box.
- Medication error that qualifies as an overdose: Enter the adverse event term. Check the "Accidental overdose" and "Medication error" boxes.

"Use of this document is governed by the terms of use on the first page of this document."

In addition, all special situations associated with atezolizumab, RO6958688, or tocilizumab, regardless of whether they result in an adverse event, should be recorded on the Adverse Event eCRF as described below:

- Accidental overdose: Enter the adverse event term. Check the "Accidental overdose" and "Medication error" boxes.
- Medication error that does not qualify as an overdose: Enter the name of the drug administered and a description of the error (e.g., wrong dose administered, wrong dosing schedule, incorrect route of administration, wrong drug, expired drug administered) as the event term. Check the "Medication error" box.
- Medication error that qualifies as an overdose: Enter the drug name and "accidental overdose" as the event term. Check the "Accidental overdose" and "Medication error" boxes. Enter a description of the error in the additional case details.
- Intercepted medication error: Enter the drug name and "intercepted medication error" as the event term. Check the "Medication error" box. Enter a description of the error in the additional case details.

As an example, an accidental overdose that resulted in a headache would require the completion of two Adverse Event eCRF pages, one to report the accidental overdose and one to report the headache. The "Accidental overdose" and "Medication error" boxes need to be checked for both entries.

5.4 IMMEDIATE REPORTING REQUIREMENTS FROM INVESTIGATOR TO SPONSOR

Certain events require immediate reporting to allow the Sponsor to take appropriate measures to address potential new risks in a clinical trial. The investigator must report such events to the Sponsor immediately; under no circumstances should reporting take place more than 24 hours after the investigator learns of the event. The following is a list of events that the investigator must report to the Sponsor within 24 hours after learning of the event, regardless of relationship to study drug:

- Serious adverse events (defined in Section 5.1.2; see Section 5.4.2 for details on reporting requirements)
- Adverse events of special interest (defined in Section 5.1.3; see Section 5.4.2 for details on reporting requirements)
- Pregnancies (see Section 5.4.3 for details on reporting requirements)

5.4.1 Emergency Medical Contacts

To ensure the safety of study patients, access to the Medical Monitor is available 24 hours a day 7 days a week. Medical Monitor contact details are listed in the “Protocol Administrative and Contact Information & List of Investigators”.

“Use of this document is governed by the terms of use on the first page of this document.”

5.4.2 Reporting Requirements for Serious Adverse Events and Non-Serious Adverse Events of Special Interest

For reports of serious adverse events and non-serious adverse events of special interest (see Sections 5.1.2 and 5.1.3), investigators should record all case details that can be gathered on the Serious Adverse Reporting Form and forward this form to the Serious Adverse Event Responsible within 24 hours.

5.4.3 Reporting Requirements for Pregnancies

5.4.3.1 Pregnancies in Female Patients

Female patients of childbearing potential will be instructed to immediately inform the investigator if they become pregnant during the study or within 5 months after the last dose of RO6958688 and atezolizumab or within 2 months after the last dose of tocilizumab A Clinical Trial Pregnancy Reporting Form should be completed by the investigator and submitted to the sponsor within 24 hours after learning of the pregnancy. Pregnancy should not be recorded on the Adverse Event eCRF. The investigator should discontinue study drugs and counsel the patient, discussing the risks of the pregnancy and the possible effects on the fetus. Monitoring of the patient should continue until conclusion of the pregnancy. Any serious adverse events associated with the pregnancy (e.g., an event in the fetus, an event in the mother during or after the pregnancy, or a congenital anomaly/birth defect in the child) should be reported on the Adverse Event eCRF.

5.4.3.2 Pregnancies in Female Partners of Male Patient

Male patients will be instructed through the Informed Consent Form to immediately inform the investigator if their partner becomes pregnant during the study or within 3 months after the last dose of RO6958688 if the patient received RO6958688 only and within 2 months after the last dose of tocilizumab (if applicable). Men should not donate sperm for 3 months after the last dose of RO6958688 and for 2 months after the last dose of tocilizumab (if applicable). A Clinical Trial Pregnancy Reporting Form should be completed by the investigator and submitted to the sponsor within 24 hours after learning of the pregnancy. Attempts should be made to collect and report details of the course and outcome of any pregnancy in the partner of a male patient exposed to study drugs. The pregnant partner will need to sign an Authorization for Use and Disclosure of Pregnancy Health Information to allow for follow-up on her pregnancy. Once the authorization has been signed, the investigator will update the Clinical Trial Pregnancy Reporting Form with additional information on the course and outcome of the pregnancy. An investigator who is contacted by the male patient or his pregnant partner may provide information on the risks of the pregnancy and the possible effects on the fetus, to support an informed decision in cooperation with the treating physician and/or obstetrician.

5.4.3.3 Abortions

A spontaneous abortion should be classified as a serious adverse event (as the Sponsor considers spontaneous abortions to be medically significant events), recorded on the

"Use of this document is governed by the terms of use on the first page of this document."

Adverse Event eCRF, and reported to the Sponsor immediately (i.e., no more than 24 hours after learning of the event; see Section 5.4.2).

If a therapeutic or elective abortion was performed because of an underlying maternal or embryofetal toxicity, the toxicity should be classified as a serious adverse event, recorded on the Adverse Event eCRF, and reported to the Sponsor immediately (i.e., no more than 24 hours after learning of the event; see Section 5.4.2). A therapeutic or elective abortion performed for reasons other than an underlying maternal or embryofetal toxicity is not considered an adverse event. All abortions should be reported as pregnancy outcomes on the paper Clinical Trial Pregnancy Reporting Form.

5.4.3.4 Congenital Anomalies/Birth Defects

Any congenital anomaly/birth defect in a child born to a female patient or female partner of a male patient exposed to study drugs should be classified as a serious adverse event, recorded on the Adverse Event eCRF, and reported to the Sponsor immediately (i.e., no more than 24 hours after learning of the event; see Section 5.4.2).

5.5 FOLLOW-UP OF PATIENTS AFTER ADVERSE EVENTS

5.5.1 Investigator Follow-Up

The investigator should follow each adverse event until the event has resolved to baseline grade or better, the event is assessed as stable by the investigator, the patient is lost to follow-up, or the patient withdraws consent. Every effort should be made to follow all serious adverse events considered to be related to study drugs or trial-related procedures until a final outcome can be reported.

During the study period, resolution of adverse events (with dates) should be documented on the Adverse Event eCRF and in the patient's medical record to facilitate source data verification. If, after follow-up, return to baseline status or stabilization cannot be established, an explanation should be recorded on the Adverse Event eCRF.

All pregnancies reported during the study should be followed until pregnancy outcome and reported according to the instructions provided in Section 5.4.3.

The investigator must report new significant follow-up information for these events to the Sponsor immediately (i.e., no more than 24 hours after becoming aware of the information). New significant information includes the following:

- New signs or symptoms or a change in the diagnosis
- Significant new diagnostic test results
- Change in causality based on new information
- Change in the event's outcome, including recovery
- Additional narrative information on the clinical course of the event

"Use of this document is governed by the terms of use on the first page of this document."

Investigators must also comply with local requirements for reporting serious adverse events to the local health authority and IRB/EC.

5.5.2 Sponsor Follow-Up

For serious adverse events, non-serious adverse events of special interest, and pregnancies, the Sponsor or a designee may follow up by telephone, fax, electronic mail, and/or a monitoring visit to obtain additional case details and outcome information (e.g., from hospital discharge summaries, consultant reports, autopsy reports) in order to perform an independent medical assessment of the reported case.

5.6 POST-STUDY ADVERSE EVENTS

At the 28-day safety and follow-up visit, the investigator should instruct each patient to report to the investigator any subsequent AEs that the patient's personal physician believes could be related to prior study drug treatments or study procedures.

The investigator is not required to actively monitor patients for adverse events after the end of the adverse event reporting period (defined as 28 days or 120 days after the last dose of study drugs as applicable). However, the Sponsor should be notified if the investigator becomes aware of any death or any other serious adverse event occurring after the end of the adverse event reporting period, if the event is believed to be related to prior study drug treatments either by faxing or by scanning and emailing the Serious Adverse Event Reporting Form using the fax number or email address provided to investigators. The Sponsor should also be notified if the investigator becomes aware of the development of cancer or a congenital anomaly/birth defect in a subsequently conceived offspring of a patient that participated in this study.

During post-study survival follow-up, deaths attributed to progression of cancer should be recorded only on the Survival eCRF.

5.7 EXPEDITED REPORTING TO HEALTH AUTHORITIES, INVESTIGATORS, INSTITUTIONAL REVIEW BOARDS, AND ETHICS COMMITTEES

The Sponsor will promptly evaluate all serious adverse events and non-serious adverse events of special interest against cumulative product experience to identify and expeditiously communicate possible new safety findings to investigators, IRBs, ECs, and applicable health authorities based on applicable legislation.

To determine reporting requirements for single adverse event cases, the Sponsor will assess the expectedness of these events using the following reference documents:

- RO6958688 and Atezolizumab Investigator's Brochures
- Tocilizumab Investigator's Brochure

"Use of this document is governed by the terms of use on the first page of this document."

The Sponsor will compare the severity of each event and the cumulative event frequency reported for the study with the severity and frequency reported in the applicable reference document.

Reporting requirements will also be based on the investigator's assessment of causality and seriousness, with allowance for upgrading by the Sponsor as needed.

6. STATISTICAL CONSIDERATIONS AND ANALYSIS PLAN

The data will be analyzed by the Sponsor and/or designated CRO. Any data analysis carried out independently by the investigator should be submitted to the Sponsor before publication or presentation. The data will be summarized with respect to demographic and baseline characteristics, efficacy observations and measurements, safety observations and measurements, PK and biomarker measurements. Patients will be followed up until disease progression (according to RECIST 1.1 or to modified RECIST criteria, whichever occurs later) or until withdrawal from the study. All data up until this point will be listed by patient and summarized by regimen and dose, if appropriate.

6.1 PRIMARY STUDY VARIABLES

The primary study variables are as follows:

- Administration of study drugs data, including start and end of infusions and dose
- Adverse events data, including dates of onset and resolution, grade according to the NCI CTCAE v4.03 and whether the adverse event qualifies as dose-limiting toxicity (DLT). An exception to this is made for CRS adverse events considered related to RO6958688 which will be evaluated using NCI CTCAE v5.

6.2 SECONDARY STUDY VARIABLES

The secondary study variables are as follows:

- The number and quality (e.g., proliferation and activation markers) of immune cells in the tumor and peripheral blood
- The pharmacokinetics (PK) data, i.e., plasma concentrations of RO6958688 and atezolizumab when administered in combination and ADA titers against RO6958688
- Anti-tumor activity data, including CT tumor assessment data and derived per-patient summaries best overall response according to RECIST v1.1 and modified RECIST criteria, by investigator assessment for the whole study and by central assessment.
- Progression-free survival (PFS) data, by investigator assessment for the whole study and by central assessment
- Preliminary overall survival (OS) data

“Use of this document is governed by the terms of use on the first page of this document.”

6.3 DETERMINATION OF SAMPLE SIZE

6.3.1 Dose-Escalation

Approximately 30 patients will be enrolled in the dose-escalation Part IA to define the MTD, based on Table 5, see column n (number of patients needed to complete dose-escalation). The exact number of patients will depend on the number of dose-levels required to determine the MTD and/or the recommended dose and on the adaptive nature of the design. The estimated chance to require more than 45 patients for the dose escalation patients in the scenarios is usually less than 10% (see Table 1 in Appendix 6 for the scenario settings). Table 5 provides the operating characteristics across different assumed toxicity scenarios. These operating characteristics have been calculated with a Roche-internal R-package, using R version 3.1.2. Table 5 shows the following metrics, with their frequency distribution under the assumed scenarios summarized by means and 10 and 90–percent quantiles from 400 simulation runs (see Appendix 6 for more details):

- Dose: This is the final dose (mg) determined as the MTD by the mCRM design.
- Tox. at dose: This is the resulting true probability (%) of a DLT at the determined MTD.
- Prop. of DLTs: This is the proportion of patients (%) with a DLT.
- n: This is the total number of patients in the dose escalation.
- $n_{overdose}$: This is the number of overdosed patients, who have been exposed to doses with DLT probability above the target of 30%.

The operating characteristics indicate that, across a wide range of scenarios, the design performs reasonably well, both with steep as well as gradually increasing dose-toxicity curves. In particular, the resulting MTD estimates are on average in the target toxicity range of 20–30%, even for the very high toxicity scenario 3. An exception is clearly the very low toxicity scenario 7, where even at the highest considered dose of 1000 mg the DLT probability is only 1%. The reasonable behavior of the dose escalation design is also reflected in the number of overdosed patients (i.e., those treated at doses above the target interval), which is on average not exceeding 9 patients, except for the very high toxicity scenario 3 where on average 18 patients are overdosed.

This design has previously been used successfully in the literature (Neuenschwander et al. 2008, Bailey et al. 2009) as well as in Roche, with only slight modifications from the current set-up.

“Use of this document is governed by the terms of use on the first page of this document.”

Table 5 Operating Characteristics of the mCRM with EWOC Design with Respect to the Chosen Scenarios

No.	Dose	Tox. at dose	Prop. of DLTs	n	$n_{overdose}$
1	230 (145, 340)	29 % (14 %, 47 %)	16 % (12 %, 19 %)	38 (32, 45)	8 (2, 15)
2	3.3 (0.4, 5)	24 % (17 %, 29 %)	25 % (19 %, 31 %)	28 (21, 37)	3 (0, 11)
3	0.1 (0, 0.2)	17 % (0 %, 42 %)	52 % (32 %, 80 %)	18 (5, 32)	18 (5, 32)
4	81 (45, 125)	27 % (14 %, 43 %)	18 % (15 %, 21 %)	34 (27, 42)	7 (1, 15)
5	66 (45, 86)	30 % (5 %, 56 %)	18 % (15 %, 21 %)	31 (26, 36)	8 (4, 12)
6	897 (640, 1000)	30 % (18 %, 35 %)	8 % (3 %, 13 %)	41 (36, 46)	6 (2, 12)
7	1000 (1000, 1000)	1 % (1 %, 1 %)	1 % (0 %, 3 %)	38 (36, 44)	0 (0, 0)
8	1 (0.6, 1.5)	25 % (13 %, 38 %)	24 % (20 %, 29 %)	29 (22, 36)	8 (1, 17)
9	465 (305, 671)	30 % (16 %, 48 %)	14 % (11 %, 17 %)	42 (36, 48)	9 (2, 14)

DLT = dose limiting toxicity; Prop. = proportion; Tox. = toxicity
note: Means (10% and 90% quantiles) from 400 simulations per scenario are shown.

6.3.2 Dose/Schedule-Finding

The sample size of 20 evaluable patients per dose scheme in the schedule comparison expansion (cohort A) in Part IB allows for a reasonably precise differentiation between the QW and Q3W dose schemes. Specifically, an observed difference of ca. 10% points in ORR would lead to approximately 80% posterior probability of a true response rate difference between two dose schemes. For example, if the number of objective responses is 4 in one and 2 in the other dose scheme out of 20 patients each, then the posterior probability of a positive response rate difference is 79.5%.

For cohort C, the randomization of approximately 40 patients to each of the arms C1, C2 and the possibly 40 patients enrolled to a later optional arm C3 allows for a reasonably precise differentiation between the arms. Specifically, if one of the arms has 20% true ORR, and the other two arms have only 10% true ORR, then n=40 patients per arm gives 83% power to decide for the correct arm as the best one. In another scenario, where the first arm has 10% true ORR, the second arm 20% true ORR, and the third arm 30% true ORR, then n=40 patients per arm gives 85% power to pick the correct third arm as the best one. Hence, the sample size of 40 patients per arm in cohort C is justified. Note: Cohort C1 enrolled 39 patients, and Cohort C2 enrolled 35 patients; no further patients will be enrolled into these cohorts. Optional cohort C3 has not and will not enroll any patients.

For cohort G1, the inclusion of 40 patients will allow a reasonably precise estimation of the incidence of ADA. Specifically, if none of the 40 patients develops ADA by week 8, then the 95% confidence interval for the ADA incidence will be 0% to 9%, i.e., exclude values of 10% or higher. In addition to the assessment of immunogenicity reduction by obinutuzumab, cohort G1, as well as optionally cohorts G2, G3, will allow for a descriptive assessment of the primary safety and efficacy endpoints in the MSS CRC and optionally in the mGC, Pancreatic indications, respectively. Specifically, observing

“Use of this document is governed by the terms of use on the first page of this document.”

an ORR of 20% in a cohort would result in a 95% confidence interval from 9% to 36%, i.e., exclude values of 9% or lower. Note: Cohorts G1, G2 and G3 have not and will not enroll any patients.

The sample size of approximately 10-20 patients in each of the cohorts B (MSS and MSI-H CRC separately) and the safety cohorts in other indications allows for an initial assessment of safety and tolerability. This is shown in detail with simulations of the intra-patient dose escalation design in cohort B in Appendix 7. Note: Cohort B1 enrolled 17 patients; no further patients will be enrolled. Cohort B2 has not and will not enroll any patients.

6.4 SUMMARIES OF CONDUCT OF STUDY

All protocol deviations will be listed. The study is open-label therefore no blinded treatment will be administered.

6.5 ANALYSIS POPULATIONS

6.5.1 Safety Analysis Population

All patients enrolled in the study who received at least one dose of RO6958688 or atezolizumab will be included in the safety population.

For the purpose of establishing the maximum-tolerated dose, the dose-determining population, which is used to recommend the next dose level based on the mCRM with EWOC design, consists of all patients in Part IA from the safety population who did not receive RO6958688 before Cycle 1 Day 1, received the assigned doses of RO6958688 (three doses) and atezolizumab (one dose) within the DLT period and have undergone the scheduled safety evaluations, or experienced a DLT after administration of a study drug within the DLT period.

Patients who have been treated before Cycle 1 Day 1 with RO6958688 will be analyzed separately.

6.5.2 Pharmacokinetic Analysis Population

All patients who received at least one dose of RO6958688 or atezolizumab will be included in the PK analysis population. Patients will be excluded from the PK analysis population if they significantly violate the inclusion or exclusion criteria, deviate significantly from the protocol, or if data are unavailable or incomplete, which may influence the PK analysis. Excluded cases will be documented together with the reason for exclusion. All decisions on exclusions from the analysis will be made prior to database closure.

6.5.3 Pharmacodynamic Analysis Population

The PD analysis population will be a subset of the safety population, based on the availability of evaluable blood or tumor samples.

"Use of this document is governed by the terms of use on the first page of this document."

6.5.4 Efficacy Analysis Population

Two efficacy analyses will be performed. The primary efficacy analysis population will consist of all patients who receive at least one dose of any study treatment. If different, an additional ITT population will consist of all patients who are not screen failures and were included in the trial (ICF signature), and will be used for sensitivity analyses.

Patients who have been treated before Cycle 1 Day 1 with RO6958688 will be analyzed separately.

6.6 SUMMARIES OF TREATMENT GROUP COMPARABILITY

Comparability across dose-levels will be evaluated descriptively via tabulation by demographic characteristics (including age, sex, patient disposition, and previous therapies). Treatment administration will be reported on the basis of number of cycles and dose intensity. Baseline status of key pharmacodynamics endpoints will also be tabulated to determine comparability. The analysis population will be the safety population.

6.7 SAFETY ANALYSES

Safety analyses will be performed for all the patients in the safety analysis population. All clinically abnormal safety parameters will be listed by patient within regimen and summarized in tables, as appropriate. Safety will be determined, but not limited to, by AEs, laboratory tests, vital signs, electrocardiogram, physical examinations, and performance status. Exposure to study medication will be summarized by total duration of study medication, number of cycles started, cumulative dose, and dose-intensity using descriptive statistics. Dose modifications, interruptions, and their reasons will be presented.

6.7.1 Dose-Escalation Approach

6.7.1.1 Modified Continual Reassessment Method with Overdose Control in Part IA

Overview

The dose escalation in Part IA will employ a modified-Continual Reassessment Method with Overdose Control (mCRM with EWOC) design, in order to estimate the MTD of RO6958688 given QW in combination with 1200 mg atezolizumab given Q3W. The design is based on the binary variable “occurrence of a DLT”. The MTD is defined as the dose that maximizes the probability of a DLT being in the targeted toxicity interval of 20–30%, subject to the probability of DLT being in the overdosing interval of 30–100% being less than 25%. Patients within a cohort will be enrolled in a sequential manner in cohorts of at least 3 patients each, which, if required, can be expanded with additional patients. Each patient will be observed for 21 days for DLT assessment. The first patient in each cohort will be observed for safety for one week (2 weeks in Spain) before enrolling additional patients in the cohort. After the last patient in each cohort has completed the 21 day DLT observation period, the Sponsor and investigators will evaluate the next dose recommended by the mCRM design and agree on the dose and

“Use of this document is governed by the terms of use on the first page of this document.”

schedule for the subsequent cohort. If a DLT is already reported in the first patient during the first two weeks of safety observation, the Sponsor will organize a teleconference with the investigators to discuss the safety and tolerability of RO6958688 and atezolizumab and to decide whether the subsequent patients in the same cohort will be enrolled at the same dose. At each dose-escalation step, the dose can be escalated, de-escalated, or an additional cohort at the same dose-level could be enrolled.

There will be no dose-escalation in the Q3W schedule, however, DLTs observed in the Q3W patients would still be considered in the EWOC model. DLT-free Q3W patients would not be considered in the EWOC model.

Next dose recommendation

The starting dose for RO6958688 will be 5 mg and the fixed dose for atezolizumab will be 1200 mg. The dose-escalation is for the dose of RO6958688. The maximum allowable increment for RO6958688 between dose-levels will be 100% from the current dose level throughout the study. The dose of RO6958688 will not be escalated above the monotherapy MTD if defined in the BP29541 study. The mCRM with EWOC design will identify the next recommended dose-level subject to the following criteria:

- Overdose threshold: The posterior probability of being within the overdosing interval (above 30% DLT probability) must be below 25%.
- MTD target: The posterior probability of being within the target toxicity interval (20–30% DLT probability) is maximized.

Clinical judgment can always override mCRM recommendations in the dose-selection process, but cannot select doses exceeding the 100% maximum increment or the above described overdose threshold (i.e., the probability of overdosing must always be below 25%). In addition to modeling the probability of toxicity with the mCRM model (see below for the details), models relating PK and PD data will be explored in parallel to the mCRM model and may inform the dose escalation as part of the available exploratory data (provided that appropriate PK and PD data for all patients enrolled up to that timepoint are available).

Stopping rules

The dose-escalation will stop under any one of the following circumstances:

- At least 50 patients are DLT-evaluable with the same schedule/sequence/timing of the two study drugs.
- At least 15 patients are DLT-evaluable with the same schedule/sequence/timing of the two study drugs, of which at least 6 patients have been accrued near the estimated MTD dose (where near means differing from the estimated MTD by at most 20%), and in addition the probability that the estimated MTD dose lies within the target toxicity interval is above 40%.

Note that further patients might still be added to cohorts in order to obtain more safety, efficacy or PD data. The tentative MTD estimate of Part I is then defined as the lastly

"Use of this document is governed by the terms of use on the first page of this document."

estimated MTD (mCRM with EWOC next recommended dose level). The DLT occurrence data from the Part II of the study will be used to update the statistical model used in the dose-escalation phase. This will lead to a final MTD estimate.

Model specification

The model is a mixture of two logistic regression models: The probability of a DLT at dose d_j is

$$p(d_j) = \pi\, p_1(d_j) + (1 - \pi) p_2(d_j)$$

where π is the probability for the combination model $p(d_j)$ being the same as the RO6958688 monotherapy model $p_1(d_j)$, and $1 - \pi$ is the probability of a separate model $p_2(d_j)$, where for $k = 1{,}2$:

$$p_k(d_j) = \frac{\exp\left(\alpha_k + \beta_k\, log\left(\frac{d_j}{d^*}\right)\right)}{1 + \exp\left(\alpha_k + \beta_k\, log\left(\frac{d_j}{d^*}\right)\right)}$$

and $d^* = 500$ is the reference dose. The monotherapy component $p_1(d_j)$ will always be informed by the latest available DLT data from the BP29541 study, by the corresponding likelihood contribution to the parameters α_1 and β_1.

This mixture model allows for dynamic borrowing from the BP29541 study (see e.g., Schmidli et al., 2004). Starting from a prior probability of $\pi = 10\%$, π is updated throughout the trial by the combination DLT data from this study and the monotherapy DLT data from the BP29541 study, in order to obtain the posterior probability π. If the combination DLT data is consistent with the monotherapy DLT data, then the posterior probability will increase above 10%. On the other hand, if the combination DLT data is not consistent with the monotherapy DLT data (e.g., if there are more DLTs at lower doses), then the posterior probability will decrease appropriately.

Minimally informative prior distributions (see Neuenschwander et al., 2008) are used for the parameters α_k and β_k $(k = 1{,}2)$, such that the prior 95% quantiles of $p_k(0.05)$ and $p_k(1000)$ are (0.3%, 24.7%) and (18.4%, 98.4%), giving a quite low probability on high toxicity at 0.05 mg and on low toxicity at 1000 mg, respectively. Specifically, the prior distributions are bivariate normal distributions on α_k and $\log(\beta_k)$.

$$\begin{pmatrix} \alpha_k \\ \log(\beta_k) \end{pmatrix} \sim \mathcal{N}\left(\begin{pmatrix} 0.996 \\ -0.729 \end{pmatrix}, \begin{pmatrix} 2.023 & 0.068 \\ 0.068 & 0.003 \end{pmatrix}\right)$$

for $k = 1{,}2$.

The dose grid for the design is 0.052, 0.1, 0.15, 0.2, 0.3, 0.45, 0.6, 0.8, 1, 1.3, 1.5, 2, 2.5, 5, 10, …, 995, 1000 mg. This grid includes also the low doses of the Part I of the

"Use of this document is governed by the terms of use on the first page of this document."

BP29541 study, and also ensures that all doses are within 100% of each other, allowing potential escalation from all doses.

Design characteristics

For all characterizations of the mCRM with EWOC design, including the trial simulations in Section 6.3, we use the available monotherapy DLT data from BP29541 as of 17th August 2015.

The recommended starting dose from the prior model would be 2 mg, while 5 mg is the dose maximizing the probability of reaching a dose with 20 to 30% DLT rate. These quite low starting recommendations from the mCRM show that a conservative prior model has been specified, ensuring the safety of the patients.

If the first patient at the starting dose 5 mg in the trial had a DLT, and this cohort was closed subsequently, then the next recommended dose would be 0.3 mg. The strong reduction of the dose in this situation is another sign of the conservative nature of the prior model.

More generally, Table 6 shows hypothetical recommendations of the mCRM with EWOC design, assuming 4 patients per cohort and no DLTs having been observed until the respective dose. For example, it is shown that the design would recommend 15 mg, if no DLTs had been observed in the 4 patients at 5 mg and one DLT had been observed in the subsequent 4 patients at 10 mg, corresponding to only 50% increment compared to the maximum allowed increment of 100%. The table shows a reasonable behavior of the design, with higher dose reductions in the beginning of the escalation when only few patient data is yet available. Note that the final next dose will always be subject to clinical judgment and is always allowed to be lower than the doses listed in this table (see subsection “Next dose recommendation” above).

“Use of this document is governed by the terms of use on the first page of this document.”

Table 6 Hypothetical Recommendations of the mCRM with EWOC Design

dose [mg]	DLTs	next dose [mg]	increment [%]
5	0	10.000	100
5	1	2.500	-50
5	2	0.800	-84
5	3	0.200	-96
5	4	0.052	-99
10	0	20	100
10	1	15	50
10	2	5	-50
10	3	2	-80
10	4	0.8	-92
20	0	40	100
20	1	40	100
20	2	15	-25
20	3	5	-75
20	4	2.5	-88
40	0	80	100
40	1	80	100
40	2	45	12
40	3	20	-50
40	4	10	-75
80	0	160	100
80	1	160	100
80	2	115	44
80	3	55	-31
80	4	30	-62
160	0	320	100
160	1	320	100
160	2	245	53
160	3	125	-22
160	4	70	-56
320	0	640	100
320	1	640	100
320	2	505	58
320	3	255	-20
320	4	150	-53
640	0	1000	56
640	1	1000	56
640	2	1000	56
640	3	540	-16
640	4	320	-50

6.7.1.2 Intra-Patient Dose Escalation Design

Cohort B1 in Part IB of the study will employ an intra-patient dose escalation design for the step up dosing regimen, in order to define the late cycle MTD of RO6958688 in combination with 1200 mg Q3W atezolizumab. In B1 the RO6958688 dose at C1D1 will

"Use of this document is governed by the terms of use on the first page of this document."

be 40 mg followed by 150 mg at C1D8, 300 mg at C1D15, 600 mg at C2D1, 900 mg at C2D8 and 1200 mg at C2D15 and C3D1. Once the 1200 mg RO6958688 dose level is reached, RO6958688 will be administered every 3 weeks (1200 mg Q3W).

The dose of RO6958688 will not be escalated above the monotherapy late cycle MTD of RO6958688 if defined in the BP29541 protocol.

The late cycle MTD will initially be estimated separately in the MSS and MSI-H patients, since potentially the safety profile could be different. However, the Sponsor may in the end define only one late cycle MTD if appropriate. A patient will be considered evaluable for the purpose of estimating the late cycle MTD, if the patient was dosed up to reaching a DLT or the maximum possible dose at the time of treatment (i.e., 1200 mg or according to the safety rules below).

The DLT window is defined as 1 week, to allow dose escalation after 1 week, and a patient is defined as DLT-evaluable if the patient received the full planned dose of RO6958688. The following rules apply for the intra-patient dose escalation, separately for MSS and MSI-H patients:

1. The time interval between dosing of the first and second as well as the second and third patient within each cohort is at least 1 week, to allow for sufficient observation time. The following patients may be dosed one week after the third patient has been dosed.

2. The dose is increased every week within each patient until any of the below applies:

    a. A DLT is observed in this patient. The dose is then reduced to the next lower, and previously tolerated dose level, afterwards.

    b. Less than 6 patients have been treated and ≥ 2 DLTs have been observed at the next higher dose level or below. The dose is then kept constant.

    c. At least 6 patients have been treated and >33% of patients have had DLT at the next higher dose level or below. The dose is then kept constant.

The late cycle MTD is defined as follows:

1. A minimum of 6 patients must have been treated at this dose level or above.

2. If 6 patients are evaluable for DLT, the late cycle MTD is defined as the highest dose with ≤ 1 DLT having been observed

3. If more than 6 patients are evaluable for DLT, the late cycle MTD is defined as the highest dose where < 33% of patients have had DLT.

"Use of this document is governed by the terms of use on the first page of this document."

The acceptable characteristics of this dose escalation design are illustrated with simulations in Appendix 7.

6.7.2 Adverse Events

The original terms recorded on the eCRF by the investigator for adverse events will be standardized by the sponsor.

Adverse event data will be reported in listings and presented in frequency tables by MedDRA terms. Adverse events will be summarized by mapped term and appropriate thesaurus level. The severity of adverse events will be graded according to the NCI CTCAE v4.03 (or for CRS, NCI CTCAE v5). Summaries of adverse event by grade, seriousness, and relationship to study treatment will be presented, as well as summaries of adverse events leading to death, and premature withdrawal from study treatment.

6.7.3 Clinical Laboratory Test Results

All clinical laboratory data will be stored on the database in the units in which they were reported. Patient listings and summary statistics at each assessment time will be presented using the International System of Units (SI units; Système International d'Unités) where required. Laboratory data not reported in SI units will be converted to SI units before processing.

Clinically abnormal laboratory test values will be presented by individual listings with flagging of values outside the normal ranges where required. Summary tables of change from baseline over time based on SI units will be displayed as needed. Shifts in NCI CTCAE v4.03 from baseline to the worst grade observed during treatment will be presented for selected laboratory parameters.

6.7.3.1 Standard Reference Ranges and Transformation of Data

Where appropriate, Roche standard reference ranges, rather than the reference ranges of the investigator, may be used. For most parameters, the measured laboratory test result can be assessed directly using the Roche standard reference range. Certain laboratory parameters need to be transformed to Roche's standard measurement units first.

A transformation may be performed on certain laboratory tests that lack sufficiently common procedures and have a wide range of investigator ranges, e.g., enzyme tests that include AST, ALT, and alkaline phosphatase and total bilirubin. If the standard reference ranges for parameters have a lower limit of zero, only the upper limits of the ranges need to be used in transforming the data.

6.7.3.2 Definition of Laboratory Abnormalities

Laboratory values falling outside the standard reference range will be labeled "H" for high or "L" for low in patient listings of laboratory data.

"Use of this document is governed by the terms of use on the first page of this document."

In addition to the standard reference range, a marked reference range has been predefined by Roche for some laboratory parameters. The marked reference range is broader than the standard reference range. Values falling outside the marked reference range that also represent a defined change from baseline are considered marked laboratory abnormalities (i.e., potentially clinically relevant). If a baseline value is not available for a patient, the midpoint of the standard reference range will be used as the patient's baseline value for the purposes of determining marked laboratory abnormalities. Marked laboratory abnormalities will be labeled in the patient listings as "HH" for very high or "LL" for very low.

6.7.4 Vital Signs

Clinically abnormal vital signs data may be presented by individual listings with flagging of values outside the normal ranges/marked abnormalities as appropriate. In addition, tabular summaries will be used, as appropriate.

6.7.5 ECG Data Analysis

Clinically abnormal ECG data may be presented by individual listings with flagging of values outside the normal ranges/marked abnormalities as appropriate. In addition, the 12-lead triplicates will be averaged out and tabulated by dose level/regimen.

6.7.6 Concomitant Medications

The original terms recorded on the Patients' eCRF by the investigator for concomitant medications will be standardized by the sponsor by assigning preferred terms.

Concomitant medications will be presented in summary tables and listings, as appropriate.

6.8 EFFICACY ANALYSES

Tumor response data will be reported using descriptive statistics. ORR, SDR, and DCR and best overall response will be summarized using relative frequencies and 95% confidence limits. Duration of response (DOR) and progression free survival (PFS) on treatment will be summarized using time to event analyses and Kaplan Meier curves. Duration of response will only be analyzed in those cohorts with a sufficient number of responders. Preliminary overall survival (OS) data may be tabulated and summarized using time to event analyses and Kaplan Meier curves. Summaries will be carried out by cohort, dose, and overall. Statistical models may be used to summarize the efficacy data obtained across different dose levels and schedules, in order to contribute to the final selection of a recommended dose and schedule. This will be carried out for all patients in the respective efficacy analysis populations, for both RECIST and modified RECIST criteria efficacy endpoints, for investigator assessed data for the whole study and for centrally assessed data.

"Use of this document is governed by the terms of use on the first page of this document."

6.8.1 Primary Efficacy Endpoint

The primary efficacy endpoints, for the evaluation of the preliminary anti-tumor activity of RO6958688 in combination with atezolizumab, will be ORR, SDR and DCR according to RECIST v1.1.

The analysis of tumor response is based on the best overall response (BOR). BOR is defined as the best response recorded from the start of treatment until disease progression/recurrence or death whichever occurs first. Since tumor assessments take place every 8 weeks, 60 days is chosen so as to cover these assessments sufficiently.

Confirmation of partial and complete responses will be done at the next scheduled visit after at least 28 days from the initial response. A patient is assigned a best overall response SD if they have a response assessment of SD, PR, or CR at one or more visits at least 42 days (6 weeks) after start of study treatment, but are not a confirmed CR or PR.

For the ORR analyses in the primary and ITT efficacy analysis populations, patients who withdraw study treatment because of any reason, die or clinically progress before the first tumor assessment on treatment will be assigned a best overall response of Non Evaluable. These patients will be included in the denominator of the corresponding ORR estimate.

6.8.2 Secondary Efficacy Endpoints

The time-to-event endpoints duration of response (DOR), progression-free survival (PFS) and preliminary overall survival (OS) are the secondary efficacy endpoints in this study.

PFS per RECIST v1.1 is defined as time between enrollment or randomization date (whichever is applicable) and date of first documented disease progression per RECIST v1.1 or death from any cause, whichever occurs first. Patients who neither progressed nor died in this interval, or who are lost to follow-up are censored at the date of last tumor assessment within this time window or last follow-up for progression of disease. Patients for whom no post-baseline tumor assessments are available are censored at first study treatment.

6.9 PHARMACODYNAMIC ANALYSES

PD parameters will be listed by patient and tabulated by dose-level/regimen and timepoint, as appropriate. Descriptive statistics will be used in summarizing peripheral blood, and tumor PD markers. Absolute and percentage change from baseline will be calculated for the PD markers. Graphical techniques may be employed to better understand the relationship of the PD markers with dose and time. Correlations between PD markers, PK markers, and clinical response may be assessed through data tabulations and graphical techniques. The potential prognostic value of the PD markers may also be investigated.

"Use of this document is governed by the terms of use on the first page of this document."

6.10 PHARMACOKINETIC ANALYSES

PK data from patients who will receive at least one dose of RO6958688 or one dose of atezolizumab will be included in the PK analysis of RO6958688 and atezolizumab, respectively. Patients will be excluded from the PK analysis if they significantly violate the inclusion or exclusion criteria, deviate significantly from the protocol, or if data are unavailable or incomplete, which may influence the PK analysis.

Extensive and sparse PK sampling will be done in that study to properly characterize the PK of RO6958688 and atezolizumab when given in combination.

When appropriate, PK parameters will be derived from the plasma concentrations of RO6958688 and atezolizumab using standard non-compartmental methods. Individual data will be listed and summarized using descriptive statistics including but not limited to mean, standard deviation, geometric mean, coefficient of variation, median, and range, as appropriate. The parameters will include, e.g., AUC, CL, Vss, accumulation ratio, and terminal elimination half-life. Mean concentration versus time may be plotted on either semi-logarithmic or normal scales.

In addition, non-linear mixed effect modeling will be used to analyze the sparse sampling dose-concentration-time data of RO6958688. Population PK parameters such as CL and V will be estimated and the influence of various covariates on these parameters will be investigated. Secondary parameters such as AUC and C_{max} will be derived from the individual post-hoc predictions. The linearity of PK (AUC, C_{max}) will also be investigated.

Actual PK sampling times are presented in the Schedule of Assessments and Hourly tables (see Appendix 1).

6.11 Immunogenicity Analyses

Immunogenicity will be assessed for atezolizumab and RO6958688. The immunogenicity analyses will include all patients with at least one ADA assessment. Patients will be grouped according to treatment received or, if no treatment is received prior to study discontinuation, according to treatment assigned.

For atezolizumab and RO6958688, the number and proportion of ADA-positive patients and ADA-negative patients at baseline (baseline prevalence) and after baseline (post-baseline incidence) will be summarized by treatment group. When determining the post-baseline incidence, patients are considered to be ADA positive if they are ADA negative or have missing data at baseline but develop an ADA response following study drug exposure (treatment-induced ADA response), or if they are ADA positive at baseline and the titer of one or more post baseline samples is at least 4-fold (e.g., $\geq$0.60-titer unit) greater than the titer of the baseline sample (treatment-enhanced ADA response) considering drug concentration in individual post-dose sample. Patients are considered to be ADA negative if they are ADA negative or have missing data at baseline and all post-baseline samples are negative, or if they are ADA positive at

“Use of this document is governed by the terms of use on the first page of this document.”

baseline but do not have any post-baseline samples with a titer that is at least 4-fold (e.g., ≥ 0.60-titer unit considering drug concentration in individual post-dose sample) greater than the titer of the baseline sample (treatment unaffected).

The relationship between ADA status and safety, efficacy, PK, and biomarker endpoints may be analyzed and reported via descriptive statistics and exploratory PK and PK–PD analyses.

6.12 INTERIM ANALYSES

Throughout the whole study, there will be ongoing safety and efficacy assessments and PK data analyses, which do not qualify as formal interim analyses. In particular, dose escalations in Part I will be based on the ongoing safety review of the data and especially DLT data.

In addition, there will be an internal interim analysis after Part I for selecting the recommended dose and schedule for Part II. Note: Part II will not open.

7. DATA COLLECTION AND MANAGEMENT

7.1 DATA QUALITY ASSURANCE

The Sponsor will be responsible for data management of this study, including quality checking of the data. Sites will be responsible for data entry into the EDC system.

A comprehensive validation check program will verify the data. Discrepancies will be generated automatically in the system at the point of entry or added manually for resolution by the investigator.

The Sponsor will produce a Data Handling Manual that describes the quality checking to be performed on the data. Central laboratory data will be sent directly to the Sponsor, using the Sponsor's standard procedures to handle and process the electronic transfer of these data.

System backups for data stored by the Sponsor and records retention for the study data will be consistent with the Sponsor's standard procedures.

7.2 ELECTRONIC CASE REPORT FORMS

Data for this study will be captured via an on line Electronic Data Capture (EDC) system. The data collected in the source documents is entered onto the study eCRF. An audit trail will maintain a record of initial entries and changes made; reasons for change; time and date of entry; and user name of person authorizing entry or change. For each patient enrolled, an eCRF must be completed and electronically signed by the principal investigator or authorized delegate from the study staff. If a patient withdraws from the study, the reason must be noted on the eCRF. If a patient is withdrawn from the study

"Use of this document is governed by the terms of use on the first page of this document."

because of a treatment‑limiting adverse event, thorough efforts should be made to clearly document the outcome.

The investigator should ensure the accuracy, completeness and timeliness of the data reported to the sponsor/CRO in the eCRFs and in all required reports.

eCRFs will be submitted electronically to the Sponsor/CRO and should be handled in accordance with instructions from the Sponsor/CRO.

At the end of the study, the investigator will receive patient data for his or her site in a readable format on a compact disc that must be kept with the study records. Acknowledgement of receipt of the compact disc is required.

7.3 SOURCE DATA DOCUMENTATION

Study monitors will perform ongoing source data verification to confirm that critical protocol data (i.e., source data) entered into the eCRFs by authorized site personnel are accurate, complete, and verifiable from source documents.

Source documents (paper or electronic) are those in which patient data are recorded and documented for the first time. They include, but are not limited to, hospital records, clinical and office charts, laboratory notes, memoranda, patient-reported outcomes, evaluation checklists, pharmacy dispensing records, recorded data from automated instruments, copies of transcriptions that are certified after verification as being accurate and complete, microfiche, photographic negatives, microfilm or magnetic media, X‑rays, patient files, and records kept at pharmacies, laboratories, and medico‑technical departments involved in a clinical trial.

Before study initiation, data to be entered directly into the eCRFs (i.e., no prior written or electronic record of the data) and considered source data must be defined in the Trial Monitoring Plan.

Source documents that are required to verify the validity and completeness of data entered into the eCRFs must not be obliterated or destroyed and must be retained per the policy for retention of records described in Section 7.5.

To facilitate source data verification, the investigators and institutions must provide the Sponsor direct access to applicable source documents and reports for trial‑related monitoring, Sponsor audits, and IRB/EC review. The investigational site must also allow inspection by applicable health authorities.

7.4 USE OF COMPUTERIZED SYSTEMS

When clinical observations are entered directly into an investigational site's computerized medical record system (i.e., in lieu of original hardcopy records), the electronic record can serve as the source document if the system has been validated in

"Use of this document is governed by the terms of use on the first page of this document."

accordance with health authority requirements pertaining to computerized systems used in clinical research. An acceptable computerized data collection system allows preservation of the original entry of data. If original data are modified, the system should maintain a viewable audit trail that shows the original data as well as the reason for the change, name of the person making the change, and date of the change.

7.5 RETENTION OF RECORDS

Records and documents pertaining to the conduct of this study and the distribution of IMP, including eCRFs, Informed Consent Forms, laboratory test results, and medication inventory records, must be retained by the Principal Investigator for 15 years after completion or discontinuation of the study, or for the length of time required by relevant national or local health authorities, whichever is longer. After that period of time, the documents may be destroyed, subject to local regulations. No records may be disposed of without the written approval of the Sponsor. Written notification should be provided to the Sponsor prior to transferring any records to another party or moving them to another location.

Roche will retain study data for 25 years after the final Clinical Study Report has been completed or for the length of time required by relevant national or local health authorities, whichever is longer.

8. ETHICAL CONSIDERATIONS

8.1 COMPLIANCE WITH LAWS AND REGULATIONS

This study will be conducted in full conformance with the ICH E6 guideline for Good Clinical Practice and the principles of the Declaration of Helsinki, or the applicable laws and regulations of the country in which the research is conducted, whichever affords the greater protection to the individual. The study will comply with the requirements of the ICH E2A guideline (Clinical Safety Data Management: Definitions and Standards for Expedited Reporting). Studies conducted in the United States or under a U.S. Investigational New Drug (IND) application will comply with U.S. FDA regulations and applicable local, state, and federal laws. Studies conducted in the EU/EEA will comply with the EU Clinical Trial Directive (2001/20/EC) and applicable local, regional, and national laws.

8.2 INFORMED CONSENT

The Sponsor's sample Informed Consent Form (and ancillary sample Informed Consent Forms such as a Child's Assent or Caregiver's Informed Consent Form, if applicable) will be provided to each site. If applicable, it will be provided in a certified translation of the local language. The Sponsor or its designee must review and approve any proposed deviations from the Sponsor's sample Informed Consent Forms or any alternate consent forms proposed by the site (collectively, the "Consent Forms") before IRB/EC submission. The final IRB/EC‑approved Consent Forms must be provided to the Sponsor for health authority submission purposes according to local requirements.

"Use of this document is governed by the terms of use on the first page of this document."

The Consent Forms must be signed and dated by the patient or the patient's legally authorized representative before his or her participation in the study. The case history or clinical records for each patient shall document the informed consent process and that written informed consent was obtained prior to participation in the study.

The Consent Forms should be revised whenever there are changes to study procedures or when new information becomes available that may affect the willingness of the patient to participate. The final revised IRB/EC-approved Consent Forms must be provided to the Sponsor for health authority submission purposes.

Patients must be re-consented to the most current version of the Consent Forms (or to a significant new information/findings addendum in accordance with applicable laws and IRB/EC policy) during their participation in the study. For any updated or revised Consent Forms, the case history or clinical records for each patient shall document the informed consent process and that written informed consent was obtained using the updated/revised Consent Forms for continued participation in the study.

A copy of each signed Consent Form must be provided to the patient or the patient's legally authorized representative. All signed and dated Consent Forms must remain in each patient's study file or in the site file and must be available for verification by study monitors at any time.

For sites in the United States, each Consent Form may also include patient authorization to allow use and disclosure of personal health information in compliance with the U.S. Health Insurance Portability and Accountability Act of 1996 (HIPAA). If the site utilizes a separate Authorization Form for patient authorization for use and disclosure of personal health information under the HIPAA regulations, the review, approval, and other processes outlined above apply except that IRB review and approval may not be required per study site policies.

8.3 INSTITUTIONAL REVIEW BOARD OR ETHICS COMMITTEE

This protocol, the Informed Consent Forms, any information to be given to the patient, and relevant supporting information must be submitted to the IRB/EC by the Principal Investigator and reviewed and approved by the IRB/EC before the study is initiated. In addition, any patient recruitment materials must be approved by the IRB/EC.

The Principal Investigator is responsible for providing written summaries of the status of the study to the IRB/EC annually or more frequently in accordance with the requirements, policies, and procedures established by the IRB/EC. Investigators are also responsible for promptly informing the IRB/EC of any protocol amendments (see Section 9.5).

In addition to the requirements for reporting all adverse events to the Sponsor, investigators must comply with requirements for reporting serious adverse events to the local health authority and IRB/EC. Investigators may receive written IND safety reports

"Use of this document is governed by the terms of use on the first page of this document."

or other safety‑related communications from the Sponsor. Investigators are responsible for ensuring that such reports are reviewed and processed in accordance with health authority requirements and the policies and procedures established by their IRB/EC, and archived in the site's study file.

8.4 CONFIDENTIALITY

The Sponsor maintains confidentiality standards by coding each patient enrolled in the study through assignment of a unique patient identification number. This means that patient names are not included in data sets that are transmitted to any Sponsor location.

Patient medical information obtained by this study is confidential and may only be disclosed to third parties as permitted by the Informed Consent Form (or separate authorization for use and disclosure of personal health information) signed by the patient, unless permitted or required by law.

Medical information may be given to a patient's personal physician or other appropriate medical personnel responsible for the patient's welfare, for treatment purposes.

Data generated by this study must be available for inspection upon request by representatives of the U.S. FDA and other national and local health authorities, Sponsor monitors, representatives, and collaborators, and the IRB/EC for each study site, as appropriate.

8.5 FINANCIAL DISCLOSURE

Investigators will provide the Sponsor with sufficient, accurate financial information in accordance with local regulations to allow the Sponsor to submit complete and accurate financial certification or disclosure statements to the appropriate health authorities. Investigators are responsible for providing information on financial interests during the course of the study and for one year after completion of the study (i.e., LPLV).

9. STUDY DOCUMENTATION, MONITORING, AND ADMINISTRATION

9.1 STUDY DOCUMENTATION

The investigator must maintain adequate and accurate records to enable the conduct of the study to be fully documented, including but not limited to the protocol, protocol amendments, Informed Consent Forms, and documentation of IRB/EC and governmental approval. In addition, at the end of the study, the investigator will receive the patient data, which includes an audit trail containing a complete record of all changes to data.

Roche shall also submit an Annual Safety Report once a year to the IEC and CAs according to local regulatory requirements and timelines of each country participating in the study.

"Use of this document is governed by the terms of use on the first page of this document."

Sampling for the RCR is contingent on review and approval for the exploratory biomarker assessments and written informed consent by an appropriate regulatory body (depending on the country where the study is performed) and a site's Institutional Review Board (IRB) / Ethics Committee (EC). If a regulatory or site's IRB/ EC does not approve the sampling for the exploratory assessments the section on biomarker sampling will not be applicable.

It is the understanding of the sponsor that this protocol (and any modifications) as well as appropriate consent procedures and advertisements, will be reviewed and approved by an Institutional Review Board (IRB). This board must operate in accordance with the current Federal Regulations. The sponsor will be sent a letter or certificate of approval prior to initiation of the study, and also whenever subsequent amendments /modifications are made to the protocol. Roche shall also submit an IND Annual Report to FDA according to local regulatory requirements and timelines.

9.2 SITE INSPECTIONS

Site visits will be conducted by the Sponsor or an authorized representative for inspection of study data, patients' medical records, and eCRFs. The investigator will permit national and local health authorities, Sponsor monitors, representatives, and collaborators, and the IRBs/ECs to inspect facilities and records relevant to this study.

9.3 ADMINISTRATIVE STRUCTURE

The Sponsor of the trial is F. Hoffmann-La Roche Ltd. The Sponsor is responsible for the study management (monitoring), data management, statistical analysis, and medical writing for this clinical study report. A clinical study report will be written and distributed to Health Authorities as required by applicable regulatory requirements.

The protocol will be submitted to country/institutional ethics committees.

9.4 PUBLICATION OF DATA AND PROTECTION OF TRADE SECRETS

The results of this study may be published or presented at scientific meetings. If this is foreseen, the investigator agrees to submit all manuscripts or abstracts to the Sponsor prior to submission. This allows the Sponsor to protect proprietary information and to provide comments based on information from other studies that may not yet be available to the investigator.

The Sponsor will comply with the requirements for publication of study results. In accordance with standard editorial and ethical practice, the Sponsor will generally support publication of multicenter trials only in their entirety and not as individual center data. In this case, a coordinating investigator will be designated by mutual agreement.

"Use of this document is governed by the terms of use on the first page of this document."

Any formal publication of the study in which contribution of Sponsor personnel exceeded that of conventional monitoring will be considered as a joint publication by the investigator and the appropriate Sponsor personnel.

General Guidelines

- Authorship will follow the guidelines for the target journal. This especially considering the maximum number of authors permitted for the target journal.
- The maximum number of authors will usually be included.
- The investigator who recruits the highest number of evaluable patients may choose to be the first or the last author and will be presenter/corresponding author on the main publication.
- Roche authors should not outnumber non-Roche authors and usually should not exceed three.
- Roche authors should not be first author.

Publication

The following are based on general rules for Roche–sponsored oncology studies:

- Subsets per country can be published by the country's primary investigator, after the main publication and in accordance with the requirements of the study contract held with the site
- Acknowledgement of supporting bodies and other investigators must be made
- Any manuscript must be passed through the Sponsor for review prior to submission. The Sponsor shall review such manuscripts within 30 working days
- Authorship will be in line with International Committee of Medical Journal Editors authorship requirements.

Any inventions and resulting patents, improvements, and/or know-how originating from the use of data from this study will become and remain the exclusive and unburdened property of the Sponsor, except where agreed otherwise.

9.5 PROTOCOL AMENDMENTS

Any substantial protocol amendments will be prepared by the Sponsor. Substantial protocol amendments will be submitted to the IRB/EC and to regulatory authorities in accordance with local regulatory requirements.

Approval must be obtained from the IRB/EC and regulatory authorities (as locally required) before implementation of any changes, except for changes necessary to eliminate an immediate hazard to patients or any non-substantial changes, as defined by regulatory requirements.

"Use of this document is governed by the terms of use on the first page of this document."

10. REFERENCES

Affara NI, Ruffell B, Medler TR, et al. B cells regulate macrophage phenotype and response to chemotherapy in squamous carcinomas. Cancer Cell 2014; 25(6):809–21.

Alexandrov LB, Nik-Zainal S, Wedge DC, et al. Signatures of mutational processes in human cancer. Nature 2013;500:415-21.

Bai S, Jorga K, Xin Yet al. A guide to rational dosing of monoclonal antibodies. Clin Pharmacokinet 2012;51:119-35.

Bacac M, Fauti T, Colombetti S, et al. Abstract 1494: combination of CEA TCB, a novel T-cell bispecific antibody for the treatment of solid tumors, with PD-L1 checkpoint blockade [abstract]. Cancer Res 2016b;76(14 Suppl)1494.

Bailey S, Neuenschwander B, Laird G, Branson M. A Bayesian Case Study in Oncology: Phase I Combination Dose-Finding Using Logistic Regression with Covariates. J Biopharm Stat 2009;19:469–84.

Bellone S, Black J, English DP, et al. Solitomab, an EpCam/CD3 bispecific antibody construct (BiTE©), is highly active against primary uterine serous papillary carcinoma cell lines in virto. Am J of Obstetrics and Gynecology 2015.

Callahan MK, Horak CE, Curran MA, et al. Peripheral and tumor immune correlates in patients with advanced melanoma treated with combination nivolumab (anti-PD-1, BMS-936558, ONO-4538) and ipilimumab [abstract]. J Clin Oncol 2013;31:3003.

Carosella ED, Ploussarrd G, LeMaoult, et al. A Systematic Review of Immunotherapy in Urologic Cancer: Evolving Roles for Trageting of CTLA-4, PD-1/PD-L1, and HLA-G. European Urology 2015; 68:267-279.

Chen DS, Irving BA, Hodi FS. Molecular pathways: next-generation immunotherapy inhibiting programmed death ligand 1 and programmed death-1. Clin. Cancer Res 2012;18:6580-7.

Chen F, Teachey DT, Pequignot E, et al. Measuring IL-6 and sIL-6R in serum from patients treated with tocilizumab and/or siltuximab following CAR T cell therapy. J Immunol Methods 2016;434,1−8.

Conrad ML, Davis WC, Koop BF. TCR and CD3 antibody cross-reactivity in 44 species. J Immunol 1991;147:3047–52.

Curiel TJ, Wei S, Dong H, et al. Blockade of B7-H1 improves myeloid dendritic cell-mediated antitumor immunity. Nat Med. 2003 9, 562-567.

De Benedetti F, Brunner HI, Ruperto N, Kenwright A, Wright S, et al. Randomized trial of tocilizumab in systemic juvenile idiopathic arthritis. N Engl J Med. 2012;367(25):2385-95.

"Use of this document is governed by the terms of use on the first page of this document."

de la Hera A, Mueller U, Olsson C, et al. Structure of the T cell Antigen Receptor (TCR): Two CD3e Subunits in a Functional TCR/CD3 Complex. J Exp Med 1991;173:7–17.

Deng R, Bumbaca D, Pastuskovas CV, et al. Preclinical pharmacokinetics, pharmacodynamics, tissue distribution, and tumor penetration of anti-PD-L1 monoclonal antibody, an immune checkpoint inhibitor. MAbs 2016;8:593 –603.

Di Giacomo AM, Biagioli M, Maio M. The emerging toxicity profiles of anti–CTLA-4 antibodies across clinical indications. Semin Oncol 2010;37:499–507.

Doessegger L, Banholzer ML. Clinical development methodology for infusion-related reactions with monoclonal antibodies. Clin Transl Immunology. 2015;4:e39.

European Medicines Agency (EMA). Summary of opinion (post authorisation): RoActemra (tocilizumab). Committee for Medicinal Products for Human Use (CHMP); EMA/CHMP/419360/2018 (28 June 2018).

Fehrenbacher L,Spira A, Ballinger M, et al. Atezolizumab versus docetaxel for patients with previously treated non-small-cell lung cancer (POPLAR): a multicentre, open-label, phase 2 randomised controlled trial. Lancet, 2016;387(10030):1837-46.

Gomez-Mantila JD, Troconiz IF Review on modeling anti-antibody responses to monoclonal antibodies. J Pharmacokinet Pharmacodyn 2014;41:523-536.

Gunderson AJ, Coussens LM. B cells and their mediators as targets for therapy in solid tumors. Exp Cell Res 2013; 319(11):1644-9.

Grupp SA, Kalos M, Barrett D, Aplenc R, Porter DL, et al. Chimeric antigen receptor-modified T cells for acute lymphoid leukemia. N Engl J Med. 2013; 368(16):1509–18.

Hejblum G. et al. A web-based delphi study for eliciting helpful criteria in the positive diagnosis of hemophagocytic syndrome in adult patients. PLoS One (Public Library of Science) April 2014; Volume 9, Issue 4.

Hodi FS, O'Day SJ, McDermott DF, et al. Improved survival with ipilimumab in patients with metastatic melanoma. N Engl J Med 2010;363:711-23.

Honeychurch J, Cheadle EJ, Dovedi SJ, et al. Immuno-regulatory antibodies for the treatment of cancer. Expert Opin. Biol. Ther. 2015, 15(6): 787-801.

Hoos A, Eggermont AM, Janetzki S, et al. Improved endpoints for cancer immunotherapy trials. J Natl Cancer Inst 2010:102:1388–97.

"In vivo anti-tumor activity of CEA TCB (RO6958688) in combination with anti-PD-L1 blocking antibody". Roche Report No. 1066291, August, 2015.

Kang SH, Ahn C. An investigation of the traditional algorithm-based designs for phase I cancer clinical trials. Drug Information J 2002;36:865–73.

“Use of this document is governed by the terms of use on the first page of this document.”

Kang SH, Ahn C. The expected toxicity rate at the maximum tolerated dose in the standard phase I cancer clinical trial design. Drug Information J 2001;35:1189–1200.

Kilinc MO, Gu T, Harden JL, et al. Central role of tumor-associated CD8+ T effector/memory cells in restoring systemic antitumor immunity. J Immunol 2009;182:4217-25.

Krupka C, Kufer P, Kischel R, et al. Blockade of the PD-1/PD-L1 axis augments lysis of AML cells by the CD33/CD3-BiTE® antibody construct AMG 330: reversing a T-cell induced immune escape mechanism. Leukemia 2015; accepted article preview online 4 August 2015; Published ahead of advanced online publication.

Lawrence MS, Stojanov P, Polak P, et al. Mutational heterogeneity in cancer and the search for new cancer-associated genes. Nature 2013;499:214-18.

Le RQ, Li L, Yuan W, et al. FDA Approval Summary: Tocilizumab for Treatment of Chimeric Antigen Receptor T Cell ‐ Induced Severe or Life ‐ Threatening Cytokine Release Syndrome. Oncologist 2018; 23:943–7.

Lee DW, Gardner R, Porter DL, et al. Current concepts in the diagnosis and management of cytokine release syndrome. Blood. 2014;124:188–95.

Le Tourneau C, Lee JJ, Siu LL. Dose escalation methods in phase I cancer clinical trials. J Natl Cancer Inst 2009;101:708–20.

Levey AS, Stevens LA, Schmid CH, et al. A new equation to estimate glomerular filtration rate. Ann Intern Med 2009; 150(9):604-12.

Mahoney KM, Freeman GJ, McDermott DF The Next Immune-Checkpoint Inhibitors:PD-1/PD-L1 Blockade in Melanoma Clinical Therapeutics 2015; 37(4):764-782.

Maude SL, Barrett D, Teachey DT, Grupp SA. Managing cytokine release syndrome associated with novel T cell-engaging therapies. Cancer J. 2014;20:119–22.

Mössner E, Brünker P, Moser S, et al. Increasing the efficacy of CD20 antibody therapy through the engineering of a new type II anti-CD20 antibody with enhanced direct and immune effector cell–mediated B-cell cytotoxicity. Blood. 2010;115:4393-4402.

Nagorsen D and Baeuerle PA. Immunomodulatory therapy of cancer with T cell engaging BiTE antibody blinatumomab. Exp Cell Res. 2011;317(9):1255-1260.

Nagorsen D, Kufer P, Patrick A, et al. Blinatumomab: A historical perspective. Pharmacology & Therapeutics 2012;136:334–342.

National Institutes of Health (NIH). Recombinant DNA Advisory Committee (RAC). Cytokine Release Syndrome after T Cell Immunotherapy. Bethesda, Maryland. NIH Videocast 9 June 2015. Available at: https://videocast.nih.gov/summary.asp?Live=16420&bhcp=1. Accessed 30 January 2017.

"Use of this document is governed by the terms of use on the first page of this document."

Neuenschwander, B., Branson, M., & Gsponer, T. (2008). Critical aspects of the Bayesian approach to phase I cancer trials. Statistics in Medicine, 27(13), 2420–39. doi:10.1002/sim.3230.

Nishino M, Giobbie-Hurder A, Gargano M, et al. Developing a common language for tumor response to immunotherapy: immune-related response criteria using unidimensional measurements. Clin Cancer Res 2013;19:3936–4.

Nishino M, Gargano M, Suda M, et al. Optimizing immune-related tumor response assessment: does reducing the number of lesions impact response assessment in melanoma patients treated with ipilimumab? J Immunother Cancer 2014;2:17.

Oberst MD, Fuhrmann S, Mulgrew K, et al. CEA/CD3 bispecific antibody MEDI-565/AMG211 activation of T cells and subsequent killing of human tumors is independent of mutations commonly found in colorectal adenocarcinomas. mAbs 2014, 6:6, 1571-1584.

Pages F, Berger A, Camus M , et al. Effector memory T cells, early metastasis, and survival in colorectal cancer. N Engl J Me d 2005;353:2654-66.

Panelli MC, White R, Foster M, et al. Forecasting the cytokine storm following systemic interleukin (IL)-2 administration. J Transl Med. 2004;2:17.

Pessano S, Oettgen H, Bhan AK, et al. The T3/T cell receptor complex: antigenic distinction between the two 20-kd T3 (T3-delta and T3-epsilon) subunits. EMBO J. 1985;4:337–44.

Roche Report No.1072962, 2016. Quantitative in vivo imaging and biodistribution of CEA-targeted T-cell bispecific antibodies in tumor bearing CD34+ human hematopoetic stem cell engrafted NSG mice.

Rosenberg JE, Hoffman-Censits J, Powles T, et al. Atezolizumab in patients with locally advanced and metastatic urothelial carcinoma who have progressed following treatment with platinum-based chemotherapy: a single-arm, multicentre, phase 2 trial. Lancet, 2016 ;387(10031):1909-20.

Russell et al. Vasopressin versus norepinephrine infusion in patients with septic shock. N Engl J Med 2008;358:877–88.

Salmerón A, Sanchez-Madrid F, Ursa MA, et al. A conformational epitope expressed upon association of CD3e with either CD3d or CD3g is the main target for recognition by anti-CD3 monoclonal antibodies. J Immunol 1991;147:3047–52.

Sauerborn M, Beers van M.C.M.M, Jiskoot W,. et al. Antibody Response Against Betaferon® in Immune Tolerant Mice: Involvement of Marginal Zone B-cells and CD4+ T-cells and Apparent Lack of Immunological Memory J Clin Immunol 2013; 33:255–263.

Schmidli, H., Gsteiger, S., Roychoudhury, S., O'Hagan, A., Spiegelhalter, D., & Neuenschwander, B. (2014). Robust meta-analytic-predictive priors in clinical trials with historical control information. Biometrics, 4(2004).

"Use of this document is governed by the terms of use on the first page of this document."

Segal NH, Parsons DW, Peggs KS, et al. Epitope landscape in breast and colorectal cancer. Cancer Res 2008;68:889-92.

Singh JA, Beg S, Lopez-Olivo MA. Tocilizumab for rheumatoid arthritis: a Cochrane systematic review. J Rheumatol. 2011;38(1):10-20.

Steiglmaier J, Benjamin J, Nagorsen D. Utilizing the BiTE (bispecific T-cell engager) platform for immunotherapy of cancer. Expert Opin. Biol. Ther 2015 15(8), 1093–1099.

Swaika A, Hammond WA, Joseph RW. Current state of anti-PD-L1 and anti-PD-1 agents in cancer therapy, Volume 67, Issue 2, Part A, October 2015, Pages 4–17.

Tabernero et al. Clinical evidence of intra-tumoral immune activation and tumor targeting with RG7813, a CEA-targeted engineered IL-2 immunocytokine. ESMO 2015 oral presentation. https://www.ecco-org.eu/Vienna2015/Scientific-Programme/Searchable-Programme?trackid=00179#anchorScpr

Teachey DT, Rheingold SR, Maude SL, et al. Cytokine release syndrome after blinatumomab treatment related to abnormal macrophage activation and ameliorated with cytokine-directed therapy. Blood. 2013;121:5154–7.

Tocilizumab (Actemra®) United States Package Insert. Available at: https://www.gene.com/download/pdf/actemra_prescribing.pdf, accessed on 17 September 2017.

Topalian et al. Safety, Activity, and Immune Correlates of Anti–PD-1 Antibody in Cancer. N Engl J Med 2012;366(26):2443-2454.

van Rooij N, van Buuren MM, Philips D, et al. Tumor exome analysis reveals neoantigen-specific T-cell reactivity in an ipilimumab-responsive melanoma. Clin Oncol 2013;31:e439-42.

Weber WA. Assessing tumor response to therapy. J Nucl Med. 2009 May;50 Suppl 1:1S-10S.

Weiner GJ: Making a better antibody: all is not lost, Blood 2010, 115:5127-5128.

Wolchok JD, Hoos A, O'Day S, et al. Guidelines for the evaluation of immune therapy activity in solid tumors: immune-related response criteria. Clin. Cancer Res 2009;15:7412–20.

Woo P, Wilkinson N, Prieur AM, Southwood T, Leone V, Livermore P, Wythe H, Thomson D, Kishimoto T. Open label phase II trial of single, ascending doses of MRA in Caucasian children with severe systemic juvenile idiopathic arthritis: proof of principle of the efficacy of IL-6 receptor blockade in this type of arthritis and demonstration of prolonged clinical improvement. Arthritis Res Ther. 2005;7(6):R1281-8.

"Use of this document is governed by the terms of use on the first page of this document."

Yokota S, Miyamae T, Imagawa T, Iwata N, Katakura S, et al. Therapeutic efficacy of humanized recombinant anti-interleukin-6 receptor antibody in children with systemic-onset juvenile idiopathic arthritis. Arthritis Rheum. 2005 Mar;52(3):818–25.

Zhang Y,Huang S, Gong D, et al. Programmed death-1 upregulation is correlated with dysfunction of tumor-infiltrating CD8+ T lymphocytes in human non-small cell lung cancer. Cell.Mol.Immunol 2010, 7, 389-395.

“Use of this document is governed by the terms of use on the first page of this document.”

Appendix 1
Schedule of Assessments

TABLE A1: SCHEDULE OF ASSESSMENTS: QW RO6958688 ADMINISTRATION SCHEDULE

Cycle	Screening	Cycle 1							Cycle 2				Cycle 3			Cycle 4			Cycle 5			Cycle 6			Cycle 7 onwards	End of treatment	28-Day Safety Follow Up[m]
Day	D-28 to D1	Day 1[o]	Day 2	Day 3	Day 8	Day 9	Day 15	Day 16	Day 1	Day 2	Day 8	Day 15	Day 1	Day 8	Day 15	Day 1	Day 8	Day 15	Day 1	Day 8	Day 15	Day 1	Day 8	Day 15	Day 1		
Assessments[a]																											
Informed Consent[b]	x																										
Eligibility	x																										
Demography	x																										
Medical History	x																										
Physical Examination[c]	x	x			x		x		x		x	x	x	x	x	x	x	X	x	x	x	x	x	x	x	x	x
Vital Signs	x	x	x	x	x	x	x	x	x	x	x	x	x	x	x	x	x	X	x	x	x	x	x	x	x[p]	x	x
Administration of atezolizumab Q3W[x]		x[z]							x				x			x			x			x			x		
Administration of RO6958688 QW[y]		x[z]			x		x		x		x	x	x	x	x	x	x	X	x	x	x	x	x	x	x[ae]		
ECG-12 lead[d]	x	x			x		x		x		x	x	x	x	x	x	x	X	x	x	x	x	x	x	x	x	x
ECOG Performance Status	x	x			x		x		x		x	x	x	x	x	x	x	X	x	x	x	x	x	x	x[q]	x	x
Pulmonary Function test[al]	x																										
Hematology[g]	x	x	x	x	x	x	x	x	x	x	x	x	x	x	x	x	x	X	x	x	x	x	x	x	x[r]	x	x
Blood Chemistry[g,aa]	x	x	x	x	x	x	x	x	x	x	x	x	x	x	x	x	x	X	x	x	x	x	x	x	x[r]	x	x
Serum Thyroid-Stimulating Hormone Free T3-T4[l]		x																	x							x	x
Coagulation[g,h]	x	x	x	x	x	x	x	x	x	x	x	x	x	x	x	x	x	X	x	x	x	x	x	x	x[r]	x	x
Urinalysis[g]	x	x	x	x	x	x	x	x	x	x	x	x	x	x	x	x	x	X	x	x	x	x	x	x	x[t]	x	x
HBV and HCV Serology	x																										
Auto-Antibody Panel[v]	x								x							x						x			x[w]		
Pregnancy Test[e]	X	X																								x	x

"Use of this document is governed by the terms of use on the first page of this document."

Appendix 1
Schedule of Assessments (cont.)

TABLE A1: SCHEDULE OF ASSESSMENTS: QW RO6958688 ADMINISTRATION SCHEDULE (CON'T)

Cycle	Screening	Cycle 1							Cycle 2				Cycle 3			Cycle 4			Cycle 5			Cycle 6			Cycle 7 onwards	End of treatment	28-Day Safety Follow Up[m]
Day	D-28 to D1	Day 1[o]	Day 2	Day 3	Day 8	Day 9	Day 15	Day 16	Day 1	Day 2	Day 8	Day 15	Day 1	Day 8	Day 15	Day 1	Day 8	Day 15	Day 1	Day 8	Day 15	Day 1	Day 8	Day 15	Day 1		
Atezolizumab PK Sampling[ab]		x			x		x		x		x	x	x			x						x			x[af]	x	x
RO6958688 PK Sampling[ab]		x	x	x	x	x	x	x	x	x	x	x	x	x	x	x	x	X	x	x	x	x	x	x	x[t]	x	x
Atezolizumab Anti-Drug Antibody (ADA)[ac]		x							x				x			x									x[af]	x	
RO6958688 Anti-Drug Antibody (ADA)[ac]		x			x		x		x		x	x	x	x	x	x	x	X	x	x	x	x	x	x	x[u]	x	x
PD Blood Flow Cytometry		x	x		x				x				x														
PD Blood Cytokines[f]		x	x	x	x	x	x		x	x	x	x	x														
Archival tumor Biopsy	X																										
Fresh Tumor Biopsy[j]	X								x				x														
Clinical Genotyping[ag]		x																									
RCR Sample (DNA and RNA)[ag]		x																									
CEA confirmation on Archival Tissue[k]	X																										
FDG-PET[n]	x[s]										x[s]												x[s]				
TCR Vβ[ah]		x											x														
Sol CEA[ai]		x							x				x														
Tumor Assessment[l]	x														x								X			x	
Tumor Growth Kinetic[ad]	x																										
Adverse Events	X																										
Previous and Concomitant Treatments	X																										

"Use of this document is governed by the terms of use on the first page of this document."

TABLE A1: SCHEDULE OF ASSESSMENTS: QW RO6958688 ADMINISTRATION SCHEDULE (CON'T)

ADA = anti-drug antibody; CEA = carcinoembryonic antigen; D = day; ECOG = Eastern Cooperative Oncology Group; PD = pharmacodynamics; PK = pharmacokinetic.

[a] All visit and their assigned safety assessments (hematology, blood chemistry, coagulation, urinalysis, physical examination), unless otherwise indicated should occur within a 3-day time window.

[b] Informed consent must be obtained before any study-specific procedures. All patients will be tested for HBV and HCV serology. Patients with HIV infection, or active hepatitis B (chronic or acute), or active hepatitis C infection are not eligible.

[c] Physical examinations (including weight) and ECOG Performance Status will be done at screening, at the time of treatment administration, at the EOT and at the 28-day safety follow-up visits. Results must be obtained prior to infusion.

[d] Triplicate 12-lead ECG at screening (within 7 days before first dose of RO6958688) pre- and end of infusion on Cycle 1 Day 1 and Cycle 1 Day 15 and at the 28-day safety follow-up visit. Pre-infusion at all other study drug administrations. Additional unscheduled ECG assessments should be performed in case of abnormalities and if clinical symptoms occur. Recording must be done prior to PK sampling.

[e] Serum pregnancy test at screening, within 7 days prior to first dose. From the date of first serum pregnancy test at screening, a urine pregnancy test must be performed every 4 weeks. Also to be performed at study termination and safety follow-up visit (28 days post last infusion).

[f] Serum or plasma samples for the assessment of cytokine (PD blood cytokines) release will be collected. At the time of an IRR, please see IRR SoA Table A3.

[g] Hematology, blood chemistry, coagulation, and urinalysis can be performed up to 24 hours (72h if during weekend) prior to scheduled dosing. Results must be obtained prior to infusion. Soluble CEA will be measured as part of the blood chemistry measurements on Cycle 1 Day 1 and every 6 weeks thereafter for patients who continue treatment and at the end of treatment visit. Soluble CD25 is to be obtained at screening and during the treatment period at Day 1 of every cycle (samples can be drawn 7 days prior to Day 1 of each cycle). Before IMP administration on Day 1 of each cycle, the investigator should confirm that the result of soluble CD25 measurement from the previous week or if available from the same day is not significantly high. If there is a significantly high level, the investigator should discuss with the Medical Monitor if the patient can or cannot receive further IMP administration. Ferritin should be obtained at screening and every week during the treatment period: Day 1, Day 8 and Day 15 of every cycle (-2 days).

[h] For coagulation (including PT/INR and PTT), an additional sample will be taken at the time of an IRR or hypersensitivity reaction, see IRR SoA Table A3. Additional coagulation parameters (i.e., first chromogenic antithrombin III then antigenic antithrombin III in case of chromogenic antithrombin III decrease, fibrinogen, prothrombin time, fibrin degradation products, D-dimer) could be assessed according to clinical judgment.

[i] Serum TSH, free T3 (or total T3 for sites where free T3 is not performed) and T4 levels will be assessed at baseline pre-dose at Cycle 1, on study (every 3 months) and at study completion/early termination visit and safety follow-up visit (28 days after last dose of study drug).

"Use of this document is governed by the terms of use on the first page of this document."

TABLE A1: SCHEDULE OF ASSESSMENTS: QW RO6958688 ADMINISTRATION SCHEDULE (CON'T)

[j] Mandatory tumor biopsy samples will be collected on two occasions (once at baseline and once during the study treatment period), except for NSCLC patients for whom there is no accessible lesion. On treatment biopsies will be collected as per Section 3.3.2.2. If preliminary data suggest that modification of the on-treatment tumor biopsy timepoint would be more appropriate, alternative on-treatment tumor biopsy timepoints could be considered in the future cohorts. Unscheduled tumor biopsies can be taken at any time point, if considered necessary for tumor assessment and patient management as decided by the investigator.

[k] CEA expression for eligibility will be confirmed centrally for U.S. and Canada patients on archival tumor material (refer to inclusion criterion #13), if available, or confirmed from a freshly obtained tumor biopsy. Results available prior to screening (outside the 28-day window) can be used.

[l] Tumor assessments will be performed at screening, then every 8 weeks after Cycle 1 Day 1 for the first year, and thereafter every 12 weeks for the second year until disease progression or treatment discontinuation. Optional latest pre-study CT scan should be provided for assessment of tumor growth kinetics within 6 weeks of patient entering the study. For the scheduled tumor assessments beyond Screening a (+/- 7-day window is permitted).

[m] Patients who complete the study or discontinue from the study early will be asked to return to the clinic 28 days after the last dose of study drugs for post-study follow-up visits.

[n] FDG-PET should be done at baseline (Day-14 to D-1) but before the baseline biopsy. The on-treatment FDG-PET scan must be done at week 4 (C2D8 +1 week) and at week 16 (C6D8 pre-dose and up to 72h before the visit) in case of dose delay the FDG-PET should be obtained at week 4 (+1week) and week 16. If preliminary data suggest that modification of the on-treatment FDG-PET timepoint would be more appropriate, alternative on-treatment FDG PET timepoints could be considered. Please refer to Appendix 9 for further information.

[o] At least 24 hours (overnight) hospital stay required following administration of study drug at Cycle 1 Day 1.

[p] Vital signs to be assessed at D1, D8 and D15 from cycle 7 onwards.

[q] ECOG to be assessed at D1, D8 and D15 from cycle 7 onwards.

[r] Hematology, blood chemistry, coagulation and urinalysis (to be drawn at D1, D8 and D15 from cycle 7 onwards) can be performed up to 24-h (72-h if during weekend) hours prior to scheduled dosing. Results must be obtained prior to atezolizumab and RO6958688 infusion. Before each IMP administration, the investigator should confirm that the result of soluble CD25 measurement from the previous week or if available from the same day is not significantly high. If there is a significantly high level, the investigator should discuss with the Medical Monitor if the patient can or cannot receive further IMP administration.

[s] For FDG PET assessment, body weight, last calorific meal information, glucose level are needed (see Appendix 9 of the protocol for more information).

[t] RO6958688 PK sampling to be drawn preinfusion and at end of infusion at D1, D8 and D15 of each cycle from Cycle 7 to Cycle 10. At Cycle 11 and every second cycle, RO6958688 PK samples to be drawn preinfusion and at end of infusion on D1.

[u] RO6958688 ADA sampling to be drawn preinfusion at D1, D8 and D15 of each cycle from Cycle 7 to Cycle 10. At Cycle 11 and every second cycle, RO6958688 ADA samples to be drawn preinfusion on Day 1 at the mentioned timepoints.

"Use of this document is governed by the terms of use on the first page of this document."

TABLE A1: SCHEDULE OF ASSESSMENTS: QW RO6958688 ADMINISTRATION SCHEDULE (CON'T)

[v] In patients who develop signs and/or symptoms suggestive of auto-immune disease while on treatment, the antibody panel should be repeated. The autoantibody panel will be assessed at screening, pre-dose every second cycle, and every 3 months after the third month on therapy (samples can be drawn up to 1 week before Day 1 visit).

[w] To be performed every 3 months after Cycle 6 (ie. Cycle 10, Cycle 14…).

[x] Atezolizumab will be delivered over 60 (+/-15) minutes. If the first infusion is tolerated without infusion associated adverse events, the second infusion may be delivered over 30 (+/-10) minutes. If the 30-minute infusion is well tolerated, all subsequent infusions may be delivered over 30 (+/-10) minutes.

[y] The start of the RO6958688 infusion on Day 1 of each cycle should be after the end of the atezolizumab infusion as per Section 4.3.2.3.

[z] Prophylactic corticosteroids to be administered at C1D1 as per Table 2. The use of analgesics and/or antihistamines is allowed to minimize expected flu like symptoms prior to the first RO6958688 administration.

[aa] Serum CEA will also be measured as a disease monitoring marker (as part of the serum chemistry panel).

[ab] Blood for PK should always be drawn at the indicated time point.

[ac] Additional samples will be drawn at the time of an IRR, see IRR SoA Table A3 (if occurring at Cycle 2 or subsequent cycles) or hypersensitivity reaction, treatment discontinuation *and* at the 28-day safety follow-up visit (RO6958688 only). A PK sample will be taken at the time of ADA sampling.

[ad] An exploratory assessment of tumor growth kinetics will be made by comparing post-treatment scans with at least 2 pre-treatment scans not older than 12 weeks prior to C1D1, if available. The two pre-treatment scans will consist of a pre-treatment scan (if available) and the study baseline scan, and will allow estimation of tumor growth rate before start of treatment.

[ae] Administration of RO6958688 to be done at D1, D8 and D15 from Cycle 7 onwards.

[af] Atezolizumab PK and ADA sampling to be drawn at Cycle 8 Day 1 then every 8 cycles from this timepoint onwards.

[ag] Clinical genotyping and RCR (DNA and RNA) samples to be drawn before any IMP administration.

[ah] For TCR Vβ, at each time point Cycle 1 Day 1 and Cycle 3 Day 1, 2 whole blood samples should be drawn. Samples should be taken prior to IMP administration.

[ai] For soluble CEA central assessment: samples will be collected pre-dose at: C1D1 (baseline), C2D1 and C3D1

[aj] Evaluate DLCO (corrected for both alveolar volume and hemoglobin) and FEV1/VC/TLC at screening for patients with bilateral lung metastases or patients with lobectomy or pneumonectomy with lung metastases in the remaining lung. Additional tests may be performed if clinically indicated.

"Use of this document is governed by the terms of use on the first page of this document."

Appendix 1
Schedule of Assessments (cont.)

TABLE A2: SCHEDULE OF HOURLY ASSESSMENTS: QW RO6958688 ADMINISTRATION SCHEDULE

Cycle	Day	Scheduled Time (h)	Vital Signs[b,n,o]	ECG-12 lead[c]	RO6958688 PK Sampling[d]	Atezolizumab PK Sampling[e]	PD Blood Flow Cytometry[m]	PD Blood Cytokines[f]	RO6958688 Anti-Drug Ant body (ADA)[g]	Atezolizumab Anti-Drug Ant body (ADA)[g]	Tumor Biopsy[h]	FDG PET[i]	TCR Vβ[l]
Screening	D-28 to D-1[a]		x	x							x	x	
Cycle 1	Day 1	Pre-infusion[j]	x	x	x	x	x	x	x	x			x
		0.5h (+/- 10 min) atezolizumab post EOI				x							
		End of RO6958688 infusion	x	x	x			x					
		2 hours post end of RO6958688 infusion			x			x					
		4 hours post end of RO6958688 infusion						x					
	Day 2	24 hours	x		x		x	x					
	Day 3	48 hours	x		x			x					
	Day 8	Pre-infusion	x	x	x	x	x	x	x				
		End of RO6958688 infusion	x		x			x					
		2 hours post end of RO6958688 infusion			x			x					

“Use of this document is governed by the terms of use on the first page of this document.”

Appendix 1
Schedule of Assessments (cont.)

TABLE A2: SCHEDULE OF HOURLY ASSESSMENTS: QW RO6958688 ADMINISTRATION SCHEDULE (CON'T)

Cycle	Day	Scheduled Time (h)	Vital Signs[b,n,o]	ECG-12 lead[c]	RO6958688 PK Sampling[d]	Atezolizumab PK Sampling[e]	PD Blood Flow Cytometry[m]	PD Blood Cytokines[f]	RO6958688 Anti-Drug Antibody (ADA)[g]	Atezolizumab Anti-Drug Antibody (ADA)[g]	Tumor Biopsy[h]	FDG PET[i]	TCR Vβ[l]
Cycle 1	Day 9	192 hours	x		x			x					
	Day 15	Pre-infusion	x	X	x	x		x	x				
		End of infusion	x	X	x			x					
		2 hours post end of infusion			x								
		4 hours post end of RO6958688 infusion						x					
	Day 16	360 hours	x		x								
Cycle 2	Day 1	Pre-infusion[j]	x	X	x	x	X	x	x	x	x		
		0.5h (+/- 10 min) atezolizumab post EOI				x							
		End of RO6958688 infusion	x		x			x					
		2 hours post end of RO6958688 infusion			x								
	Day 2	24 hours	x		x			x					
	Day 8	Pre-infusion	x	X	x	x		x	x			x	
		End of RO6958688 infusion			x			x					

"Use of this document is governed by the terms of use on the first page of this document."

Appendix 1
Schedule of Assessments (cont.)

TABLE A2: SCHEDULE OF HOURLY ASSESSMENTS: QW RO6958688 ADMINISTRATION SCHEDULE (CON'T)

Cycle	Day	Scheduled Time (h)	Vital Signs[b,n,o]	ECG-12 lead[c]	RO6958688 PK Sampling[d]	Atezolizumab PK Sampling[e]	PD Blood Flow Cytometry[m]	PD Blood Cytokines[f]	RO6958688 Anti-Drug Antibody (ADA)[g]	Atezolizumab Anti-Drug Antibody (ADA)[g]	Tumor Biopsy[h]	FDG PET[i]	TCR Vβ[l]
Cycle 2	Day 15	Pre-infusion	x	X	x	x		x	x				
		End of RO6958688 infusion			x			x					
Cycle 3	Day 1	Pre-infusion[j]	x	X	x	x	X	x	x	x	x		X
		End of RO6958688 infusion			x			x					
	Day 8	Pre-infusion	x	X	x				x				
		End of RO6958688 infusion			x								
	Day 15	Pre-infusion	x	X	x				x				
		End of RO6958688 infusion			x								
Cycle 4	Day 1	Pre-infusion[j]	x	X	x	x			x	x			
		End of RO6958688 infusion			x								

"Use of this document is governed by the terms of use on the first page of this document."

Appendix 1
Schedule of Assessments (cont.)

TABLE A2: SCHEDULE OF HOURLY ASSESSMENTS: QW RO6958688 ADMINISTRATION SCHEDULE (CON'T)

Cycle	Day	Scheduled Time (h)	Vital Signs[b,n,o]	ECG-12 lead[c]	RO6958688 PK Sampling[d]	Atezolizumab PK Sampling[e]	PD Blood Flow Cytometry[m]	PD Blood Cytokines[f]	RO6958688 Anti-Drug Antibody (ADA)[g]	Atezolizumab Anti-Drug Antibody (ADA)[g]	Tumor Biopsy[h]	FDG PET[i]	TCR Vβ[l]
Cycle 4	Day 8	Pre-infusion	x	X	x				x				
		End of RO6958688 infusion			x								
	Day 15	Pre-infusion	x	X	x				x				
		End of RO6958688 infusion			x								
Cycle 5	Day 1	Pre-infusion[j]	x	X	x				x				
		End of RO6958688 infusion			x								
	Day 8	Pre-infusion	x	X	x				x				
		End of RO6958688 infusion			x								
	Day 15	Pre-infusion	x	X	x				x				
		End of RO6958688 infusion			x								

"Use of this document is governed by the terms of use on the first page of this document."

TABLE A2: SCHEDULE OF HOURLY ASSESSMENTS: QW RO6958688 ADMINISTRATION SCHEDULE (CON'T)

Cycle	Day	Scheduled Time (h)	Vital Signs[b,n,o]	ECG-12 lead[c]	RO6958688 PK Sampling[d]	Atezolizumab PK Sampling[e]	PD Blood Flow Cytometry[m]	PD Blood Cytokines[f]	RO6958688 Anti-Drug Antibody (ADA)[g]	Atezolizumab Anti-Drug Antibody (ADA)[g]	Tumor Biopsy[h]	FDG PET[i]	TCR Vβ[l]
Cycle 6	Day 1	Pre-infusion[j]	x	X	x	x			x				
		End of RO6958688 infusion			x								
	Day 8	Pre-infusion	x	X	x				x			x	
		End of RO6958688 infusion			x								
	Day 15	Pre-infusion	x	X	x				x				
		End of RO6958688 infusion			x								
Cycle 7 to Cycle 10	Day 1	Pre-infusion[j]	x	X	x	x[k]			x	x[k]			
		End of RO6958688 infusion			x								
	Day 8	Pre-infusion	x	X	x				x				
		End of RO6958688 infusion			x								

"Use of this document is governed by the terms of use on the first page of this document."

Appendix 1
Schedule of Assessments (cont.)

TABLE A2: SCHEDULE OF HOURLY ASSESSMENTS: QW RO6958688 ADMINISTRATION SCHEDULE (CON'T)

Cycle	Day	Scheduled Time (h)	Vital Signs[b,n,o]	ECG-12 lead[c]	RO6958688 PK Sampling[d]	Atezolizumab PK Sampling[e]	PD Blood Flow Cytometry[m]	PD Blood Cytokines[f]	RO6958688 Anti-Drug Antibody (ADA)[g]	Atezolizumab Anti-Drug Antibody (ADA)[g]	Tumor Biopsy[h]	FDG PET[i]	TCR Vβ[l]
Cycle 7 to Cycle 10	Day 15	Pre-infusion	x	X	x				x				
		End of RO6958688 infusion			x								
Cycle 11 and every 2nd cycle	Day 1	Pre-infusion	x	x	x				x				
		End of RO6958688 infusion			x								
End of treatment	Anytime		x	X	x	x			x	x			
28-Day Safety Follow-up	Anytime		x	X	x	x			x				

"Use of this document is governed by the terms of use on the first page of this document."

TABLE A2: SCHEDULE OF HOURLY ASSESSMENTS: QW RO6958688 ADMINISTRATION SCHEDULE (CON'T)

ADA = anti-drug antibody; CEA = carcinoembryonic antigen; D = day; ECOG = Eastern Cooperative Oncology Group; PD = pharmacodynamics; PK = pharmacokinetic.

[a] Screening assessments should be performed between D-28 and D-1, unless specified.

[b] The patient's vital signs (heart rate, respiratory rate, blood pressure, oxygen saturation and temperature) should be determined up to 60 (± 10) minutes before each atezolizumab infusion. Vital signs should also be obtained during or after the atezolizumab infusion if clinically indicated. Vital signs (including supine blood pressure and heart rate) will be monitored on Day 1 RO6958688 pre-infusion, every 15 minutes until the end of RO6958688 infusion and, thereafter, every 30 minutes until the infusion line is removed. Starting Cycle 1 Day 15: RO6958688 pre-infusion, every 30 minutes during RO6958688 infusion and every 30 minutes after the end of RO6958688 infusion until infusion line is removed. From Cycle 3 onwards vital signs will only be obtained on Day 1 RO6958688 pre-infusion. For the purposes of the eCRF vital signs will only be captured pre-infusion and in case of abnormalities

[c] Triplicate 12-lead ECG at screening (within 7 days before first dose of RO6958688) pre- and end of infusion on Cycle 1 Day 1 and Cycle 1 Day 15 and at the 28-day safety follow-up visit. Pre-infusion at all other study drug administrations. Additional unscheduled ECG assessments should be performed in case of abnormalities and if clinical symptoms occur. Recording must be done prior to PK sampling.

[d] All RO6958688 PK sampling Timepoints are reference from the start of RO6958688 infusion. PK time windows: Pre-infusion: up to – 4 h - EOI: up to + 30 min - 2 h post EOI: +/- 30 min - 24 h: +/- 2 h - 48 h: +/- 4 h - Later: +/- 12 h.

[e] All atezolizumab PK sampling Timepoints are reference from the start of atezolizumab infusion.

[f] Serum or plasma samples for the assessment of cytokine (PD blood cytokines) release will be collected. At the time of an IRR please see IRR SoA Table A3.

[g] Additional samples will be drawn at the time of an IRR, see IRR SoA Table A3 or hypersensitivity reaction, treatment discontinuation, *and* at the 28-day safety follow-up visit (RO6958688 only). A PK sample will be taken at the time of ADA sampling.

[h] Mandatory tumor biopsy samples will be collected on two occasions (once at baseline and once during the study treatment period), except for NSCLC patients for whom there is no accessible lesion. If preliminary data suggest that modification of the on-treatment tumor biopsy timepoint would be more appropriate, alternative on-treatment tumor biopsy timepoints could be considered in the future cohorts.

[i] FDG-PET should be done at baseline (Day-14 to D-1) but before the baseline biopsy. The on-treatment FDG-PET scan must be done at week 4 (C2D8 +1 week) and at week 16 (C6D8 pre-dose and up to 72h before the visit) in case of dose delay the FDG-PET should be obtained at week 4 (+1week) and week 16. If preliminary data suggest that modification of the on-treatment FDG-PET timepoint would be more appropriate, alternative on-treatment FDG PET timepoints could be considered in the future cohorts.

[j] Pre-infusion timepoints relate to RO6958688 or atezolizumab. On Cycle 1, pre-infusion PK samples (for both RO6958688 and atezolizumab) can be taken prior to both infusions. On Cycle 2 and beyond, pre-dose PK samples can be taken up to 1-hour before infusion start for atezolizumab; after atezolizumab end of infusion and before the start of infusion RO6958688 for the RO6958688 pre-dose sample.

[k] Atezolizumab PK and ADA sampling to be drawn at Cycle 8 Day 1 then every 8 cycles from this timepoint onwards.

"Use of this document is governed by the terms of use on the first page of this document."

TABLE A2: SCHEDULE OF HOURLY ASSESSMENTS: QW RO6958688 ADMINISTRATION SCHEDULE (CON'T)

[l] For TCR Vβ, at each time point Cycle 1 Day 1 and Cycle 3 Day 1, two whole blood samples should be drawn. Samples should be taken prior to IMP administration.

[m] Blood Flow Cytometry sample to be drawn pre-dose at C1D1, C1D8, C2D1, and C3D1.

[n] If a patient has experienced a Grade 3 IRR/CRS event during the previous treatment administration visit, the patient should be hospitalized for at least 24 hours after the end of infusion at next treatment administration visit during which vital signs will be monitored as follows: every 30 (± 10) minutes for the first hour post infusion, every 60 (± 15) minutes during the following 3 hours, every 120 (± 20) minutes for the next 8 hours and every 240 (± 30) minutes for the remaining 12 hours.

[o] If a patient experiences a Grade 3 or higher treatment-related adverse event (with the exception of IRR/CRS) within the 24 hours period following the previous infusion, the patient should be observed for at least 8 hours after the end of RO6958688 infusion at the next administration visit during which vital signs will be monitored as follows: every 30 (± 10) minutes for the first hour post infusion, every 60 (± 15) minutes during the following 3 hours, every 120 (± 20) minutes for the next 4 hours.

"Use of this document is governed by the terms of use on the first page of this document."

TABLE A3: SCHEDULE OF ASSESSMENTS FOR INFUSION-RELATED REACTION

Cycle	Day	Scheduled Time (h)	PK Sample	PD Blood Cytokines[a]	IgE and Tryptase		Hematology	Blood Chemistry	Coagulation
IRR	Unscheduled	Anytime	x	x	x		x	x	x

ADA = anti-drug antibody; h = hour; IRR = infusion-related reaction; PD = pharmacodynamics; PK = pharmacokinetic.

[a] At the time of an IRR (including repetitive occurrence of IRR) or hypersensitivity reaction, unscheduled samples will be collected. Cytokine analysis will include but not be limited to TNF-a, IL-6, and IFNγ. For patients who experience an IRR ≥ Grade 2, tryptase and total IgE will be analyzed.

"Use of this document is governed by the terms of use on the first page of this document."

Appendix 1
Schedule of Assessments (cont.)

TABLE A4: SCHEDULE OF ASSESSMENTS: Q3W RO6958688 ADMINISTRATION SCHEDULE

Cycle	Screening	Cycle 1					Cycle 2					Cycle 3			Cycle 4		Cycle 5		Cycle 6		Cycle 7 onwards	End of treatment	28-Day Safety Follow Up[m]
Day	D-28 to D1	Day 1	Day 2	Day 3	Day 8	Day 15	Day 1	Day 2	Day 3	Day 8	Day 15	Day 1	Day 8	Day 15	Day 1	Day 8	Day 1	Day 8	Day 1	Day 8	Day 1		
Assessments[a]																							
Informed Consent[b]	x																						
Eligibility	x																						
Demography	x																						
Medical History	x																						
Physical Examination[c]	x	x			x	x	X		x	x	x	x	x	x	x	x	x	x	X	x	x	x	x
Vital Signs	x	x	x	x	x	x	X	x	x	x	x	x	x	x	x	x	x	x	X	x	x[p]	x[p]	x
Administration of atezolizumab Q3W[x]		x[z]					X					x			x		x		X		x		
Administration of RO6958688 Q3W[y]		x[z]					X					x			x		x		X		x[ae]		
ECG-12 lead[d]	x	x			x	x	X			x	x	x	x	x	x	x	x	x	X	x	x	x	x
ECOG Performance Status	x	x			x	x	X		x	x	x	x	x	x	x	x	x	x	X	x	x[q]	x[q]	x
Pulmonary Function test[al]	x																						
Hematology[g]	x	x	x	x	x	x	X	x	X	x	x	x	x	x	x	x	x	x	X	x	x[r]	x[r]	x
Blood Chemistry[g,aa]	x	x	x	x	x	x	X	x	X	x	x	x	x	x	x	x	x	x	X	x	x[r]	x[r]	x
Serum Thyroid-Stimulating Hormone freeT3-T4[l]		x															x					x	x
Coagulation[g,h]	x	x	x	x	x	x	X	x	X	x	x	x	x	x	x	x	x	x	X	x	x[r]	x[r]	x
Urinalysis[g]	x	x	x	x	x	x	X	x	X	x	x	x	x	x	x	x	x	x	X	x	x[r]	x[r]	x
HBV and HCV Serology	x																						
Auto-Antibody Panel[v]	x						X								x				X		x[w]		
Pregnancy Test[e]	x	X																				x	x

"Use of this document is governed by the terms of use on the first page of this document."

Appendix 1
Schedule of Assessments (cont.)

TABLE A4: SCHEDULE OF ASSESSMENTS Q3W RO6958688 ADMINISTRATION SCHEDULE (CONT.)

Cycle	Screening	Cycle 1					Cycle 2					Cycle 3			Cycle 4		Cycle 5		Cycle 6		Cycle 7 onwards	End of treatment	28-Day Safety Follow Up[m]
Day	D-28 to D1	Day 1	Day 2	Day 3	Day 8	Day 15	Day 1	Day 2	Day 3	Day 8	Day 15	Day 1	Day 8	Day 15	Day 1	Day 8	Day 1	Day 8	Day 1	Day 8	Day 1		
Atezolizumab PK Sampling[ab]		x			x	x	x			x	x	x			x				X		x[af]	x	x
RO6958688 PK Sampling[ab]		x	x	x	x	x	x	X	x	x	x	x	x	x	x	x	X	x	X	x	x[t]	x	x
Atezolizumab Anti-Drug Antibody (ADA)[ac]		x					x					x			x						x[af]	x	
RO6958688 Anti-Drug Antibody (ADA)[ac]		x			x	x	x			x	x	x	x	x	x		X		X		x[u]	x	x
PD Blood Flow Cytometry		x	x		x		x					x											
PD Blood Cytokines[f]		x	x	x	x		x	X	x	x		x	x										
Archival Tumor Biopsy	x																						
Fresh Tumor Biopsy[j]	x						x					x											
Clinical Genotyping[ag]		x																					
RCR Sample (DNA and RNA) [ag]		x																					
CEA confirmation on Archival Tissue[k]	x																						
FDG-PET[n]	x[s]									x[s]										x[s]			
TCR Vβ[ah]		x										x											
Sol CEA[ai]		x					x					x											

"Use of this document is governed by the terms of use on the first page of this document."

Appendix 1
Schedule of Assessments (cont.)

TABLE A4: SCHEDULE OF ASSESSMENTS Q3W RO6958688 ADMINISTRATION SCHEDULE (CONT.)

Cycle	Screening	Cycle 1					Cycle 2					Cycle 3			Cycle 4		Cycle 5		Cycle 6		Cycle 7 onwards	End of treatment	28-Day Safety Follow Up[m]
Tumor Assessment[l]	x													x						x		x	
Tumor Growth Kinetic[ad]	x																						
Adverse Events	X																						
Previous and Concomitant Treatments	X																						

"Use of this document is governed by the terms of use on the first page of this document."

TABLE A4: SCHEDULE OF ASSESSMENTS Q3W RO6958688 ADMINISTRATION SCHEDULE (CONT.)

ADA = anti-drug antibody; CEA = carcinoembryonic antigen; D = day; ECOG = Eastern Cooperative Oncology Group; PD = pharmacodynamics; PK = pharmacokinetic.

[a] All visit and their assigned safety assessments (hematology, blood chemistry, coagulation, urinalysis, physical examination), unless otherwise indicated should occur within a 3-day time window.

[b] Informed consent must be obtained before any study-specific procedures. All patients will be tested for HBV and HCV serology. Patients with HIV infection, or active hepatitis B (chronic or acute), or active hepatitis C infection are not eligible.

[c] Physical examinations (including weight) and ECOG Performance Status will be done at screening, at the time of treatment administration, at the EOT and at the 28-day safety follow-up visits. Results must be obtained prior to infusion.

[d] Triplicate 12-lead ECG at screening (within 7 days before first dose of RO6958688) pre- and end of infusion on Cycle 1 Day 1. Pre-infusion at all other study drug administrations and at EOT and the 28-day safety follow-up visit Additional unscheduled ECG assessments should be performed in case of abnormalities and if clinical symptoms occur. Recording must be done prior to PK sampling.

[e] Serum pregnancy test at screening, within 7 days prior to first dose. From the date of first serum pregnancy test at screening, a urine pregnancy test must be performed every 4 weeks. Also to be performed at study termination and safety follow-up visit (28 days post last infusion).

[f] Serum or plasma samples for the assessment of cytokine (PD blood cytokines) release will be collected. At the time of an IRR, please see IRR SoA Table A3.

[g] Hematology, blood chemistry, coagulation, and urinalysis can be performed up to 24-h (72-h if during weekend) hours prior to scheduled dosing. Results must be obtained prior to infusion. Soluble CEA will be measured by the site as part of the blood chemistry measurements on Cycle 1 Day 1 and every 6 weeks thereafter for patients who continue treatment and at the end of treatment visit. Soluble CD25 is to be obtained at screening and during the treatment period at Day 1 of every cycle (samples can be drawn 7 days prior to Day 1 of each cycle). Before IMP administration on Day 1 of each cycle, the investigator should confirm that the result of soluble CD25 measurement from the previous week or if available from the same day is not significantly high. If there is a significantly high level, the investigator should discuss with the Medical Monitor if the patient can or cannot receive further IMP administration. Ferritin should be obtained at screening and during the treatment period: Day 1 of each cycle (-2 days).

[h] For coagulation (including PT/INR and PTT), an additional sample will be taken at the time of an IRR or hypersensitivity reaction, see IRR SoA Table A3. Additional coagulation parameters (i.e., first chromogenic antithrombin III then antigenic antithrombin III in case of chromogenic antithrombin III decrease, fibrinogen, prothrombin time, fibrin degradation products, D-dimer) could be assessed according to clinical judgment.

[i] Serum TSH, free T3 (or total T3 for sites where free T3 is not performed) and T4 levels will be assessed at baseline pre-dose at Cycle 1, on study (every 3 months) and at study completion/early termination visit and safety follow-up visit (28 days after last dose of study drug).

“Use of this document is governed by the terms of use on the first page of this document.”

TABLE A4: SCHEDULE OF ASSESSMENTS Q3W RO6958688 ADMINISTRATION SCHEDULE (CONT.)

[j] Mandatory tumor biopsy samples will be collected on two occasions (once at baseline and once during the study treatment period), except for NSCLC patients for whom there is no accessible lesion. On treatment biopsies will be collected as per Section 3.3.2.2. If preliminary data suggest that modification of the on-treatment tumor biopsy timepoint would be more appropriate, alternative on-treatment tumor biopsy timepoints could be considered in the future cohorts. Unscheduled tumor biopsies can be taken at any time point, if considered necessary for tumor assessment and patient management as decided by the investigator.

[k] CEA expression for eligibility will be confirmed centrally for U.S. patients on archival tumor material (refer to inclusion criterion #13), if available, or confirmed from a freshly obtained tumor biopsy. Results available prior to screening (outside the 28-day window) can be used.

[l] Tumor assessments will be performed at screening, then every 8 weeks after Cycle 1 Day 1 for the first year, and thereafter every 12 weeks for the second year until disease progression or treatment discontinuation. Optional latest pre-study CT scan should be provided for assessment of tumor growth kinetics within 6 weeks of patient entering the study. For the scheduled tumor assessments beyond Screening a (+/- 7-day window is permitted).

[m] Patients who complete the study or discontinue from the study early will be asked to return to the clinic 28 days after the last dose of study drugs for post-study follow-up visits.

[n] FDG-PET should be done at baseline (Day-14 to D-1) but before the baseline biopsy. The on-treatment FDG-PET scan must be done at week 4 (C2D8 + 1 week) and at week 16 (C6D8 and up to 72h before the visit) in case of dose delay the FDG-PET should be obtained at week 4 (+1week) and week 16. If preliminary data suggest that modification of the on-treatment FDG-PET timepoint would be more appropriate, alternative on-treatment FDG-PET timepoints could be considered in the future cohorts. Please refer to Appendix 9 for further information.

[o] At least 24 hours (overnight) hospital stay required following administration of study drug at Cycle 1 Day 1.

[p] Vital signs to be assessed at D1 from cycle 7 onwards.

[q] ECOG to be assessed at D1 from cycle 7 onwards.

[r] Hematology, blood chemistry, coagulation and urinalysis (to be drawn at D1 from cycle 7 onwards) can be performed up to 24 hours (72h if during weekend) prior to scheduled dosing. Results must be obtained prior to atezolizumab and RO6958688 infusion. Before each IMP administration on Day 1 or each cycle, the investigator should confirm that the result of soluble CD25 measurement from the previous week or if available from the same day is not significantly high. If there is a significantly high level, the investigator should discuss with the Medical Monitor if the patient can or cannot receive further IMP administration.

[s] For FDG PET assessment, body weight, last calorific meal information, glucose level are needed (see Appendix 9 of the protocol for more information).

[t] RO6958688 PK sampling to be drawn preinfusion and at end of infusion at D1 of each cycle from Cycle 7 to Cycle 10. At Cycle 11 and every second cycle, RO6958688 PK samples to be drawn preinfusion and at end of infusion on D1.

[u] RO6958688 ADA sampling to be drawn preinfusion at D1 of each cycle from Cycle 7 to Cycle 10. At Cycle 11 and every second cycle, RO6958688 ADA samples to be drawn preinfusion on Day 1 at the mentioned timepoints.

“Use of this document is governed by the terms of use on the first page of this document.”

TABLE A4: SCHEDULE OF ASSESSMENTS Q3W RO6958688 ADMINISTRATION SCHEDULE (CONT.)

[v] In patients who develop signs and/or symptoms suggestive of auto-immune disease while on treatment, the antibody panel should be repeated. The autoantibody panel will be assessed at screening, pre-dose every second cycle, and every 3 months after the third month on therapy (samples can be drawn up to 1 week before Day 1 visit).

[w] To be performed every 3 months after Cycle 6 (ie. Cycle 10, Cycle 14…).

[x] Atezolizumab will be delivered over 60 (+/-15) minutes. If the first infusion is tolerated without infusion associated adverse events, the second infusion may be delivered over 30 (+/-10) minutes. If the 30-minute infusion is well tolerated, all subsequent infusions may be delivered over 30 (+/-10) minutes.

[y] The start of the RO6958688 infusion on Day 1 of each cycle should be after the end of the atezolizumab infusion as per Section 4.3.2.3.

[z] Prophylactic corticosteroids to be administered at C1D1 as per Table 2. The use of analgesics and/or antihistamines is allowed to minimize expected flu like symptoms prior to the first RO6958688 administration.

[aa] Serum CEA will also be measured as a disease monitoring marker (as part of the serum chemistry panel).

[ab] Blood for PK should always be drawn at the indicated time point.

[ac] Additional samples will be drawn at the time of an IRR, see IRR SoA Table A3 or hypersensitivity reaction, treatment discontinuation, and at the 28-day safety follow-up visit (RO6958688 only). A PK sample will be taken at the time of ADA sampling.

[ad] An exploratory assessment of tumor growth kinetics will be made by comparing post-treatment scans with at least 2 pre-treatment scans not older than 12 weeks prior to C1D1, if available. The two pre-treatment scans will consist of a pre-treatment scan (if available) and the study baseline scan, and will allow estimation of tumor growth rate before start of treatment.

[ae] Administration of RO6958688 to be done at D1 from Cycle 7 onwards.

[af] Atezolizumab PK and ADA sampling to be drawn at Cycle 8 Day 1 then every 8 cycles from this timepoint onwards.

[ag] Clinical genotyping and RCR (DNA and RNA) samples to be drawn before any IMP administration.

[ah] For TCR Vβ, at each time point Cycle 1 Day 1 and Cycle 3 Day 1, 2 whole blood samples should be drawn. Samples should be taken prior to IMP administration

[ai] For soluble CEA central assessment: samples will be collected pre-dose at: C1D1 (baseline), C2D1 and C3D1.

[aj] Evaluate DLCO (corrected for both alveolar volume and hemoglobin) and FEV1/VC/TLC at screening for patients with bilateral lung metastases or patients with lobectomy or pneumonectomy with lung metastases in the remaining lung. Additional tests may be performed if clinically indicated.

“Use of this document is governed by the terms of use on the first page of this document.”

Appendix 1
Schedule of Assessments (cont.)

TABLE A5: SCHEDULE OF HOURLY ASSESSMENTS Q3W RO6958688 ADMINISTRATION SCHEDULE (CONT.)

Cycle	Day	Scheduled Time (h)	Vital signs[b,n,o]	ECG-12 lead[c]	RO6958688 PK Sampling[d]	Atezolizumab PK Sampling[e]	RO6958688 Anti-Drug Antibody (ADA)[g]	Atezolizumab Anti-Drug Antibody (ADA)[g]	PD Blood Flow Cytometry[m]	PD Blood Cytokines[f]	Tumor Biopsy[h]	FDG-PET[i]	TCR Vβ[l]
Screening	D-28 to D1[a]	Anytime	x	x							x	x	
Cycle 1	Day 1	Pre-infusion[j]	x	x	x	x	x	x	x	x			x
		0.5h (+/- 10 min) post atezolizumab EOI				x							
		RO6958688 EOI	x	x	x					x			
		2 hours post RO6958688 EOI			x					x			
		4 hours post RO6958688 EOI								x			
	Day 2	24 hours	x		x				x	x			
	Day 3	48 hours	x		x					x			
	Day 8	168 hours	x	x	x	x	x		x	x			
	Day 15	336 hours	x	x	x	x	x						
Cycle 2	Day 1	Pre-infusion[j]	x	x	x	x	x	x	x	x	x		
		0.5h (+/- 10 min) post atezolizumab EOI				x							
		RO6958688 EOI	x		x					x			
		2 hours post RO6958688 EOI			x					x			
	Day 2	24 hours	x		x					x			
	Day 3	48 hours	x		x					x			
	Day 8	168 hours	x	x	x	x	x			x		x	
	Day 15	336 hours	x	x	x	x	x						

“Use of this document is governed by the terms of use on the first page of this document.”

Appendix 1
Schedule of Assessments (cont.)

TABLE A5: SCHEDULE OF HOURLY ASSESSMENTS Q3W RO6958688 ADMINISTRATION SCHEDULE (CONT.)

Cycle	Day	Scheduled Time (h)	Vital signs[b,n,o]	ECG-12 lead[c]	RO6958688 PK Sampling[d]	Atezolizumab PK Sampling[e]	RO6958688 Anti-Drug Antibody (ADA)[g]	Atezolizumab Anti-Drug Antibody (ADA)[g]	PD Blood Flow Cytometry[m]	PD Blood Cytokines[f]	Tumor Biopsy[h]	FDG-PET[i]	TCR Vβ[l]
Cycle 3	Day 1	Pre-infusion[j]	x	x	x	x	X	x	X	x	x		x
		RO6958688 EOI[j]			x					x			
	Day 8	168 hours	x	x	x		X			x			
	Day 15	336 hours	x	x	x		X						
Cycle 4	Day 1	Pre-infusion[j]	x	x	x	x	X	x					
		RO6958688 EOI[j]			x								
	Day 8	168 hours	x	x	x								
Cycle 5	Day 1	Pre-infusion[j]	x	x	x		X						
		RO6958688 EOI[j]			x								
	Day 8	168 hours	x	x	x								
Cycle 6	Day 1	Pre-infusion[j]	x	x	x	x	X						
		RO6958688 EOI[j]			x								
	Day 8	168 hours	x	x	x							X	
Cycle 7 to Cycle 10	Day 1	Pre-infusion[j]	x	x	x	x[k]	X	x[k]					
		RO6958688 EOI[j]			x								
Cycle 11 and every 2nd cycle	Day 1	Pre-infusion[j]	x	x	x		x						
		RO6958688 EOI[j]			x								
End of treatment	Anytime		x	x	x	x	X	x					
28-day Safety Follow Up	Anytime		x	x	x	x	X						

"Use of this document is governed by the terms of use on the first page of this document."

TABLE A5: SCHEDULE OF HOURLY ASSESSMENTS Q3W RO6958688 ADMINISTRATION SCHEDULE (CONT.)

ADA = anti-drug antibody; CEA = carcinoembryonic antigen; D = day; ECOG = Eastern Cooperative Oncology Group; PD = pharmacodynamics; PK = pharmacokinetic.

[a] Screening assessments should be performed between D-28 and D-1, unless specified.

[b] The patient's vital signs (heart rate, respiratory rate, blood pressure, oxygen saturation and temperature) should be determined up to 60 (± 10) minutes before each atezolizumab infusion. Vital signs should also be obtained during or after the atezolizumab infusion if clinically indicated. Vital signs (including supine blood pressure and heart rate) will be monitored on Day 1 RO6958688 pre-infusion, every 15 minutes until the end of RO6958688 infusion and, thereafter, every 30 minutes until the infusion line is removed. Starting Cycle 2 Day 1: RO6958688 pre-infusion, every 30 minutes during RO6958688 infusion and every 30 minutes after the end of RO6958688 infusion until infusion line is removed. From Cycle 3 onwards vital signs will only be obtained on Day 1 RO6958688 pre-infusion. For the purposes of the eCRF vital signs will only be captured pre-infusion and in case of abnormalities

[c] Triplicate 12-lead ECG at screening (within 7 days before first dose of RO6958688) pre- and end of infusion on Cycle 1 Day 1. Pre-infusion at all other study drug administrations and at EOT and at 28-day safety follow up visit. Additional unscheduled ECG assessments should be performed in case of abnormalities and if clinical symptoms occur. Recording must be done prior to PK sampling.

[d] All RO6958688 PK sampling Timepoints are reference from the start of RO6958688 infusion. PK time windows: Pre-infusion: up to – 4 h - EOI: up to + 30 min - 2 h post EOI: +/- 30 min - 24 h: +/- 2 h - 48 h: +/- 4 h - Later: +/- 12 h.

[e] All atezolizumab PK sampling Timepoints are reference from the start of atezolizumab infusion.

[f] Serum or plasma samples for the assessment of cytokine (PD blood cytokines) release will be collected. At the time of an IRR please see IRR SoA Table A3.

[g] Additional samples will be drawn at the time of an IRR, see IRR SoA Table A3 or hypersensitivity reaction, treatment discontinuation, *and* at the 28-day safety follow-up (RO6958688 only). A PK sample will be taken at the time of ADA sampling.

[h] Mandatory tumor biopsy samples will be collected on two occasions (once at baseline and once during the study treatment period), except for NSCLC patients for whom there is no accessible lesion. If preliminary data suggest that modification of the on-treatment tumor biopsy timepoint would be more appropriate, alternative on-treatment tumor biopsy timepoints could be considered in the future cohorts.

[i] FDG-PET should be done at baseline (Day-14 to D-1) but before the baseline biopsy. The on-treatment FDG-PET scan must be done at week 4 (C2D81+ 1 week) and at week 16 (C6D8and up to 72h before the visit) in case of dose delay the FDG-PET should be obtained at week 4 (+1week) and week 16. If preliminary data suggest that modification of the on-treatment FDG-PET timepoint would be more appropriate, alternative on-treatment FDG-PET timepoints could be considered in the future cohorts.

[j] Pre-infusion timepoints relate to RO6958688 or atezolizumab. Pre-dose PK samples can be taken up to 1-hour before infusion start for atezolizumab; after atezolizumab end of infusion and/or before the start of RO6958688 infusion for the RO6958688 pre-dose sample.

[k] Atezolizumab PK and ADA sampling to be drawn at Cycle 8 Day 1 then every 8 cycles from this timepoint onwards.

[l] For TCR Vβ, at each time point Cycle 1 Day 1 and Cycle 3 Day 1, 2 whole blood samples should be drawn. Samples should be taken prior to IMP administration.

"Use of this document is governed by the terms of use on the first page of this document."

TABLE A5: SCHEDULE OF HOURLY ASSESSMENTS Q3W RO6958688 ADMINISTRATION SCHEDULE (CONT.)

[m] Blood Flow Cytometry sample to be drawn pre-dose at C1D1, C1D8, C2D1, and C3D1.

[n] If a patient has experienced a Grade 3 IRR/CRS event during the previous treatment administration visit, the patient should be hospitalized for at least 24 hours after the end of infusion at next treatment administration visit during which vital signs will be monitored as follows: every 30 (± 10) minutes for the first hour post infusion, every 60 (± 15) minutes during the following 3 hours, every 120 (± 20) minutes for the next 8 hours and every 240 (± 30) minutes for the remaining 12 hours.

[o] If a patient experiences a Grade 3 or higher treatment-related adverse event (with the exception of IRR/CRS) within the 24 hours period following the previous infusion, the patient should be observed for at least 8 hours after the end of RO6958688 infusion at the next administration visit during which vital signs will be monitored as follows: every 30 (± 10) minutes for the first hour post infusion, every 60 (± 15) minutes during the following 3 hours, every 120 (± 20) minutes for the next 4 hours.

“Use of this document is governed by the terms of use on the first page of this document.”

Appendix 1
Schedule of Assessments (cont.)

TABLE A6: SCHEDULE OF ASSESSMENTS: COHORTS C & SAFETY RO6958688 ADMINISTRATION SCHEDULE

Cycle	Screening	Cycle 1							Cycle 2			Cycle 3			Cycle 4		Cycle 5		Cycle 6		Cycle 7 onwards	End of treatment	28-Day Safety Follow Up[m]
Day	D-28 to D1	Day 1	Day 2	Day 3	Day 8	Day 9	Day 15	Day 16	Day 1	Day 2	Day 8	Day 1	Day 2	Day 15	Day 1	Day 2	Day 1	Day 2	Day 1	Day 8	Day 1		
Assessments[a]																							
Informed Consent[b]	x																						
Eligibility	x																						
Demography	x																						
Medical History	x																						
Physical Examination[c]	x	x			x		x		x		x	x		x	x		x		x	x	x	x	x
Vital Signs	x	x	x	x	x	x	x	x	x	x	x	x	x	x	x	x	x	x	x	x	x[p]	x[p]	x
Administration of atezolizumab Q3W[x]		x[z]							x			x			x		x		x		x		
Administration of RO6958688 QW - Q3W[y]		x[z]			x		x		x			x			x		x		x		x[ae]		
ECG-12 lead[d]	x	x			x		x		x			x		x	x		x		x	x	x	x	x
ECOG Performance Status	x	x			x		x		x		x	x		x	x		x		x	x	x[q]	x[q]	x
Pulmonary Function test[al]	x																						
Hematology[g]	x	x	x	x	x	x	x	x	x	x	x	x	x	x	x	x	x	x	x	x	x[r]	x[r]	x
Blood Chemistry[g,aa]	x	x	x	x	x	x	x	x	x	x	x	x	x	x	x	x	x	x	x	x	x[r]	x[r]	x
Serum Thyroid-Stimulating Hormone freeT3-T4[i]		x															x					x	x
Coagulation[g,h]	x	x	x	x	x	x	x	x	x	x	x	x	x	x	x	x	x	x	x	x	x[r]	x[r]	x
Urinalysis[g]	x	x	x	x	x	x	x	x	x	x	x	x	x	x	x	x	x	x	x	x	x[r]	x[r]	x

"Use of this document is governed by the terms of use on the first page of this document."

Appendix 1
Schedule of Assessments (cont.)

TABLE A6: SCHEDULE OF ASSESSMENTS COHORTS C & SAFETY RO6958688 ADMINISTRATION SCHEDULE (CONT.)

Cycle	Screening	Cycle 1							Cycle 2			Cycle 3			Cycle 4		Cycle 5		Cycle 6		Cycle 7 onwards	End of treatment	28-Day Safety Follow Up[m]
Day	D-28 to D1	Day 1	Day 2	Day 3	Day 8	Day 9	Day 15	Day 16	Day 1	Day 2	Day 8	Day 1	Day 2	Day 15	Day 1	Day 2	Day 1	Day 2	Day 1	Day 8	Day 1		
Serology	x																						
Auto-Antibody Panel[v]	x								x						x				x		x[w]		
Pregnancy Test[e]	x	X																				x	x
Atezolizumab PK Sampling[ab]		x			x	x	x	x	x			X			x				x		x[af]	x	x
RO6958688 PK Sampling[ab]		x	x	x	x	x	x	x	x	x	x	X	x	x	x	x	x	x	x	X	x[t]	x	x
Atezolizumab Anti-Drug Antibody (ADA)[ac]		x							x			X			x						x[af]	x	
RO6958688 Anti-Drug Antibody (ADA)[ac]		x			x		x		x		x	X	x	x	x		x		x		x[u]	x	x
PD Blood Flow Cytometry		x	x		x				x			X											
PD Blood Cytokines[f]		x	x	x	x	x	x	x	x	x		X	x										
Archival Tumor Biopsy	x																						
Fresh Tumor Biopsy[j]	x								x			X											
Clinical Genotyping[ag]		x																					
RCR Sample (DNA and DNA) [ag]		x																					
CEA confirmation on Archival Tissue[k]	x																						
FDG-PET[n]	x[s]										x[s]									x[s]			
TCR Vβ[ah]		x										X											
Sol CEA[ai]		x							x			X											

“Use of this document is governed by the terms of use on the first page of this document.”

Appendix 1
Schedule of Assessments (cont.)

TABLE A6: SCHEDULE OF ASSESSMENTS COHORTS C & SAFETY RO6958688 ADMINISTRATION SCHEDULE (CONT.)

Cycle	Screening	Cycle 1							Cycle 2			Cycle 3			Cycle 4		Cycle 5		Cycle 6		Cycle 7 onwards	End of treatment	28-Day Safety Follow Up[m]
Day	D-28 to D1	Day 1	Day 2	Day 3	Day 8	Day 9	Day 15	Day 16	Day 1	Day 2	Day 8	Day 1	Day 2	Day 15	Day 1	Day 2	Day 1	Day 2	Day 1	Day 8	Day 1		
Tumor Assessment[l]	x													x						x		x	
Tumor Growth Kineticad	x																						
Adverse Events	X																						
Previous and Concomitant Treatments	X																						

“Use of this document is governed by the terms of use on the first page of this document.”

TABLE A6: SCHEDULE OF ASSESSMENTS COHORTS C & SAFETY RO6958688 ADMINISTRATION SCHEDULE (CONT.)

ADA = anti-drug antibody; CEA = carcinoembryonic antigen; D = day; ECOG = Eastern Cooperative Oncology Group; PD = pharmacodynamics; PK = pharmacokinetic.

a All visit and their assigned safety assessments (hematology, blood chemistry, coagulation, urinalysis, physical examination), unless otherwise indicated should occur within a 3-day time window.

b Informed consent must be obtained before any study-specific procedures. All patients will be tested for HBV and HCV serology. Patients with HIV infection, or active hepatitis B (chronic or acute), or active hepatitis C infection are not eligible.

c Physical examinations (including weight) and ECOG Performance Status will be done at screening, at the time of treatment administration, at the EOT and at the 28-day safety follow-up visits. Results must be obtained prior to infusion.

d Triplicate 12-lead ECG at screening (within 7 days before first dose of RO6958688) pre- and end of infusion on Cycle 1 Day 1 and Cycle 1 Day 15 and at the 28-day safety follow-up visit. Pre-infusion at all other study drug administrations. Additional unscheduled ECG assessments should be performed in case of abnormalities and if clinical symptoms occur. Recording must be done prior to PK sampling.

e Serum pregnancy test at screening, within 7 days prior to first dose. From the date of first serum pregnancy test at screening, a urine pregnancy test must be performed every 4 weeks. Also to be performed at study termination and safety follow-up visit (28 days post last infusion).

f Serum or plasma samples for the assessment of cytokine (PD blood cytokines) release will be collected. At the time of an IRR, please see IRR SoA Table A3.

g Hematology, blood chemistry, coagulation, and urinalysis can be performed up to 24 hours (72h if during weekend) prior to scheduled dosing. Results must be obtained prior to infusion. Soluble CEA will be measured as part of the blood chemistry measurements on Cycle 1 Day 1 and every 6 weeks thereafter for patients who continue treatment and at the end of treatment visit. Soluble CD25 is to be obtained at screening and during the treatment period at Day 1 of every cycle (samples can be drawn 7 days prior to Day 1 of each cycle). Before IMP administration on Day 1 of each cycle, the investigator should confirm that the result of soluble CD25 measurement from the previous week or if available from the same day is not significantly high. If there is a significantly high level, the investigator should discuss with the Medical Monitor if the patient can or cannot receive further IMP administration. Ferritin should be obtained at screening and every week during the treatment period: Day 1, Day 8 and Day 15 of every cycle (-2 days) when applicable.

h For coagulation (including PT/INR and PTT), an additional sample will be taken at the time of an IRR or hypersensitivity reaction, see IRR SoA Table A3. Additional coagulation parameters (i.e., first chromogenic antithrombin III then antigenic antithrombin III in case of chromogenic antithrombin III decrease, f brinogen, prothrombin time, f brin degradation products, D-dimer) could be assessed according to clinical judgment.

i Serum TSH, free T3 (or total T3 for sites where free T3 is not performed) and T4 levels will be assessed at baseline pre-dose at Cycle 1, on study (every 3 months) and at study completion/early termination visit and safety follow-up visit (28 days after last dose of study drug).

j Mandatory tumor biopsy samples will be collected on two occasions (once at baseline and once during the study treatment period), except for NSCLC patients for whom there is no accessible lesion. On treatment biopsies will be collected as per Section 3.3.2.2. If preliminary data suggest that modification of the on-treatment tumor biopsy timepoint would be more appropriate, alternative on-treatment tumor biopsy timepoints could be considered in the future cohorts. Unscheduled tumor biopsies can be taken at any time point, if considered necessary for tumor assessment and patient management as decided by the investigator.

k CEA expression for eligibility will be confirmed centrally for U.S. patients on archival tumor material (refer to inclusion criterion #13), if available, or confirmed from a freshly obtained tumor biopsy. Results available prior to screening (outside the 28 day window) can be used.

l Tumor assessments will be performed at screening, then every 8 weeks after Cycle 1 Day 1 for the first year, and thereafter every 12 weeks for the second year until disease progression or treatment discontinuation. Optional latest pre-study CT scan should be provided for assessment of tumor growth kinetics within 6 weeks of patient entering the study. For the scheduled tumor assessments beyond Screening a (+/- 7-day window is permitted).

m Patients who complete the study or discontinue from the study early will be asked to return to the clinic 28 days after the last dose of study drugs for post-study follow-up visits.

n FDG-PET should be done at baseline (Day-14 to D-1) but before the baseline biopsy. The on-treatment FDG-PET scan must be done at week 4 (C2D8 + 1 week) and at week 16 (C6D8and up to 72h before the visit) in case of dose delay the FDG-PET should be obtained at week 4 (+1week) and week 16. If preliminary data suggest that modification of the on-treatment FDG-PET timepoint would be more appropriate, alternative on-treatment FDG PET timepoints could be considered. Please refer to Appendix 9 for further information.

"Use of this document is governed by the terms of use on the first page of this document."

Appendix 1
Schedule of Assessments (cont.)

TABLE A6: SCHEDULE OF ASSESSMENTS COHORTS C & SAFETY RO6958688 ADMINISTRATION SCHEDULE (CONT.)

o At least 24 hours (overnight) hospital stay required following administration of study drug at Cycle 1 Day 1.

p Vital signs to be assessed at D1 from cycle 7 onwards.

q ECOG to be assessed at D1 from cycle 7 onwards.

r Hematology, blood chemistry, coagulation and urinalysis (to be drawn at D1 from cycle 7 onwards) can be performed up to 24 hours (72h if during weekend) prior to scheduled dosing. Results must be obtained prior to atezolizumab and RO6958688 infusion. Before each IMP administration on Day 1 or each cycle, the investigator should confirm that the result of soluble CD25 measurement from the previous week or if available from the same day is not significantly high. If there is a significantly high level, the investigator should discuss with the Medical Monitor if the patient can or cannot receive further IMP administration.

s For FDG PET assessment, body weight, last calorific meal information, glucose level are needed (see Appendix 9 of the protocol for more information).

t RO6958688 PK sampling to be drawn preinfusion and at end of infusion at D1 of each cycle from Cycle 7 to Cycle 10. At Cycle 11 and every second cycle, RO6958688 PK samples to be drawn preinfusion and at end of infusion on D1.

u RO6958688 ADA sampling to be drawn preinfusion at D1 of each cycle from Cycle 7 to Cycle 10. At Cycle 11 and every second cycle, RO6958688 ADA samples to be drawn preinfusion on Day 1 at the mentioned timepoints.

v In patients who develop signs and/or symptoms suggestive of auto-immune disease while on treatment, the antibody panel should be repeated. The autoantibody panel will be assessed at screening, pre-dose every second cycle, and every 3 months after the third month on therapy (samples can be drawn up to 1 week before Day 1 visit).

w To be performed every 3 months after Cycle 6 (ie. Cycle 10, Cycle 14…).

x Atezolizumab will be delivered over 60 (+/-15) minutes. If the first infusion is tolerated without infusion associated adverse events, the second infusion may be delivered over 30 (+/-10) minutes. If the 30 minute infusion is well tolerated, all subsequent infusions may be delivered over 30 (+/-10) minutes.

y The start of the RO6958688 infusion on Day 1 of each cycle should be after the end of the atezolizumab infusion as per Section 4.3.2.3.

z Prophylactic corticosteroids to be administered at C1D1 as per Table 2. The use of analgesics and/or antihistamines is allowed to minimize expected flu like symptoms prior to the first RO6958688 administration.

aa Serum CEA will also be measured as a disease monitoring marker (as part of the serum chemistry panel).

ab Blood for PK should always be drawn at the indicated time point.

ac Additional samples will be drawn at the time of an IRR, see IRR SoA Table A3 or hypersensitivity reaction, treatment discontinuation, *and* at the 28-day safety follow-up visit (RO6958688 only). A PK sample will be taken at the time of ADA sampling.

ad An exploratory assessment of tumor growth kinetics will be made by comparing post-treatment scans with at least 2 pre-treatment scans not older than 12 weeks prior to C1D1, if available. The two pre-treatment scans will consist of a pre-treatment scan (if available) and the study baseline scan, and will allow estimation of tumor growth rate before start of treatment.

ae Administration of RO6958688 to be done at D1 from Cycle 7 onwards.

af Atezolizumab PK and ADA sampling to be drawn at Cycle 8 Day 1 then every 8 cycles from this timepoint onwards.

ag Clinical genotyping and RCR (DNA and RNA) samples to be drawn before any IMP administration.

ah For TCR Vβ, at each time point Cycle 1 Day 1 and Cycle 3 Day 1, 2 whole blood samples should be drawn. Samples should be taken prior to IMP administration

ai For soluble CEA central assessment: samples will be collected pre-dose at: C1D1 (baseline), C2D1 and C3D1.

aj Evaluate DLCO (corrected for both alveolar volume and hemoglobin) and FEV1/VC/TLC at screening for patients with bilateral lung metastases or patients with lobectomy or pneumonectomy with lung metastases in the remaining lung. Additional tests may be performed if clinically indicated.

"Use of this document is governed by the terms of use on the first page of this document."

Appendix 1
Schedule of Assessments (cont.)

TABLE A7: SCHEDULE OF HOURLY ASSESSMENTS COHORTS C & SAFETY RO6958688 ADMINISTRATION SCHEDULE

Cycle	Day	Scheduled Time (h)	Vital signs[b,n,o]	ECG-12 lead[c]	RO6958688 PK Sampling[d]	Atezolizumab PK Sampling[e]	RO6958688 Anti-Drug Antibody (ADA)[g]	Atezolizumab Anti-Drug Antibody (ADA)[g]	PD Blood Flow Cytometry	PD Blood Cytokines[f]	Tumor Biopsy[h]	FDG-PET[i]	TCR Vβ[l]
Screening	D-28 to D1[a]	Anytime	x	x					x[o]		x	x	
Cycle 1	Day 1	Pre-infusion[j]	x	x	x	x	x	X	x	x			x
		0.5h (+/- 10 min) post atezolizumab EOI				x							
		RO6958688 EOI	x	x	x					x			
		2 hours post RO6958688 EOI			x					x			
		4 hours post RO6958688 EOI								x			
	Day 2	24 hours	x		x				x	x			
	Day 3	48 hours	x		x					x			
	Day 8	Pre-infusion[j]	x	x	x	x	x		x	x			
		RO6958688 EOI			x								
		2 hours post RO6958688 EOI			x								
	Day 9	192 hours	x		x	x				x			
	Day 15	Pre-infusion[j]	x	x	x	x	x			x			
		RO6958688 EOI		x	x					x			
		2 hours post RO6958688 EOI			x					x			
	Day 16	360 hours	x		x	x				x			

"Use of this document is governed by the terms of use on the first page of this document."

TABLE A7: SCHEDULE OF HOURLY ASSESSMENTS COHORTS C & SAFETY RO6958688 ADMINISTRATION SCHEDULE (CONT.)

Cycle	Day	Scheduled Time (h)	Vital signs[b,n,o]	ECG-12 lead[c]	RO6958688 PK Sampling[d]	Atezolizumab PK Sampling[e]	RO6958688 Anti-Drug Ant body (ADA)[g]	Atezolizumab Anti-Drug Antibody (ADA)[g]	PD Blood Flow Cytometry	PD Blood Cytokines[f]	Tumor Biopsy[h]	FDG-PET[i]	TCR Vβ[l]
Cycle 2	Day 1	Pre-infusion[j]	x	x	x	x	x	X	x	X	x		
		0.5h (+/- 10 min) post atezolizumab EOI				x							
		RO6958688 EOI	x		x					X			
		2 hours post RO6958688 EOI			x					X			
	Day 2	24 hours	x		x					X			
	Day 8	168 hours	x		x		x					x	
Cycle 3	Day 1	Pre-infusion[j]	x	x	x	x	x	X	x	X	x		x
		RO6958688 EOI[j]			x					X			
	Day 2	24 hours	x		x		x			X			
	Day 15	336 hours	x	x	x		x						
Cycle 4	Day 1	Pre-infusion[j]	x	x	x	x	x	X					
		RO6958688 EOI[j]			x								
	Day 2	24 hours	x		x								

“Use of this document is governed by the terms of use on the first page of this document.”

Appendix 1
Schedule of Assessments (cont.)

TABLE A7: SCHEDULE OF HOURLY ASSESSMENTS COHORTS C & SAFETY RO6958688 ADMINISTRATION SCHEDULE (CONT.)

Cycle	Day	Scheduled Time (h)	Vital signs[b, n,o]	ECG-12 lead[c]	RO6958688 PK Sampling[d]	Atezolizumab PK Sampling[e]	RO6958688 Anti-Drug Antibody (ADA)[g]	Atezolizumab Anti-Drug Antibody (ADA)[g]	PD Blood Flow Cytometry[m]	PD Blood Cytokines[f]	Tumor Biopsy[h]	FDG-PET[i]	TCR Vβ[l]
Cycle 5	Day 1	Pre-infusion[j]	x	x	x		x						
		RO6958688 EOI[j]			x								
	Day 2	24 hours	x		x								
Cycle 6	Day 1	Pre-infusion[j]	x	x	x	x	x						
		RO6958688 EOI[j]			x								
	Day 8	168 hours	x	x	x							X	
Cycle 7 to Cycle 10	Day 1	Pre-infusion[j]	x	x	x	x[k]	x	x[k]					
		RO6958688 EOI[j]			x								
Cycle 11 and every 2nd cycle	Day 1	Pre-infusion[j]	x	x	x		x						
		RO6958688 EOI[j]			x								
End of treatment	Anytime		x	x	x	x	x	x					
28-day Safety Follow Up	Anytime		x	x	x	x	x						

“Use of this document is governed by the terms of use on the first page of this document.”

TABLE A7: SCHEDULE OF HOURLY ASSESSMENTS COHORTS C & SAFETY RO6958688 ADMINISTRATION SCHEDULE (CONT.)

ADA = anti-drug antibody; CEA = carcinoembryonic antigen; D = day; ECOG = Eastern Cooperative Oncology Group; PD = pharmacodynamics; PK = pharmacokinetic.

a Screening assessments should be performed between D-28 and D-1, unless specified.

b The patient's vital signs (heart rate, respiratory rate, blood pressure, oxygen saturation and temperature) should be determined up to 60 (± 10) minutes before each atezolizumab infusion. Vital signs should also be obtained during or after the atezolizumab infusion if clinically indicated. Vital signs (including supine blood pressure and heart rate) will be monitored on Day 1 RO6958688 pre-infusion, every 15 minutes until the end of RO6958688 infusion and, thereafter, every 30 minutes until the infusion line is removed. Starting Cycle 2 Day 1: RO6958688 pre-infusion, every 30 minutes during RO6958688 infusion and every 30 minutes after the end of RO6958688 infusion until infusion line is removed. From Cycle 3 onwards vital signs will only be obtained on Day 1 RO6958688 pre-infusion. For the purposes of the eCRF vital signs will only be captured pre-infusion and in case of abnormalities.

c Triplicate 12-lead ECG at screening (within 7 days before first dose of RO6958688) pre- and end of infusion on Cycle 1 Day 1 and C1D15. Pre-infusion at all other study drug administrations and at EOT and at 28-day safety follow up visit. Additional unscheduled ECG assessments should be performed in case of abnormalities and if clinical symptoms occur. Recording must be done prior to PK sampling.

d All RO6958688 PK sampling Timepoints are reference from the start of RO6958688 infusion. PK time windows: Pre-infusion: up to – 4 h - EOI: up to + 30 min - 2 h post EOI: +/- 30 min - 24 h: +/- 2 h - 48 h: +/- 4 h - Later: +/- 12 h.

e All atezolizumab PK sampling Timepoints are reference from the start of atezolizumab infusion.

f Serum or plasma samples for the assessment of cytokine (PD blood cytokines) release will be collected. At the time of an IRR please see IRR SoA Table A3.

g Additional samples will be drawn at the time of an IRR, see IRR SoA Table A3 or hypersensitivity reaction, treatment discontinuation *and* at the 28-day safety follow-up visit (RO6958688 only). A PK sample will be taken at the time of ADA sampling.

h Mandatory tumor biopsy samples will be collected on two occasions (once at baseline and once during the study treatment period), except for NSCLC patients for whom there is no accessible lesion. If preliminary data suggest that modification of the on-treatment tumor biopsy timepoint would be more appropriate, alternative on-treatment tumor biopsy timepoints could be considered in the future cohorts.

i FDG-PET should be done at baseline (Day-14 to D-1) but before the baseline biopsy. The on-treatment FDG-PET scan must be done at week 4 (C2D8 + 1 week) and at week 16 (C6D8 pre-dose and up to 72h before the visit) in case of dose delay the FDG-PET should be obtained at week 4 (+1week) and week 16. If preliminary data suggest that modification of the on-treatment FDG-PET timepoint would be more appropriate, alternative on-treatment FDG-PET timepoints could be considered in the future cohorts.

j Pre-infusion timepoints relate to RO6958688 or atezolizumab. Pre-dose PK samples can be taken up to 1-hour before infusion start for atezolizumab; after atezolizumab end of infusion and/or before the start of RO6958688 infusion for the RO6958688 pre-dose sample.

k Atezolizumab PK and ADA sampling to be drawn at Cycle 8 Day 1 then every 8 cycles from this timepoint onwards.

l For TCR Vβ, at each time point Cycle 1 Day 1 and Cycle 3 Day 1, 2 whole blood samples should be drawn. Samples should be taken prior to IMP administration.

m Blood Flow Cytometry sample to be drawn pre-dose at C1D1, C1D8, C2D1, and C3D1.

n If a patient has experienced a Grade 3 IRR/CRS event during the previous treatment administration visit, the patient should be hospitalized for at least 24 hours after the end of infusion at next treatment administration visit during which vital signs will be monitored as follows: every 30 (± 10) minutes for the first hour post infusion, every 60 (± 15) minutes during the following 3 hours, every 120 (± 20) minutes for the next 8 hours and every 240 (± 30) minutes for the remaining 12 hours.

o If a patient experiences a Grade 3 or higher treatment-related adverse event (with the exception of IRR/CRS) within the 24 hours period following the previous infusion, the patient should be observed for at least 8 hours after the end of RO6958688 infusion at the next administration visit during which vital signs will be monitored as follows: every 30 (± 10) minutes for the first hour post infusion, every 60 (± 15) minutes during the following 3 hours, every 120 (± 20) minutes for the next 4 hours.

"Use of this document is governed by the terms of use on the first page of this document."

Appendix 1
Schedule of Assessments (cont.)

TABLE A8: SCHEDULE OF ASSESSMENTS: STEP UP 1200 MG (COHORT B1) RO6958688 ADMINISTRATION SCHEDULE

Cycle	Screening	Cycle 1							Cycle 2				Cycle 3			Cycle 4		Cycle 5		Cycle 6		Cycle 7 onwards	End of treatment	28-Day Safety Follow Up[m]
Day	D-28 to D-1	Day 1[o]	Day 2	Day 3	Day 8	Day 9	Day 15	Day 16	Day 1	Day 2	Day 8	Day 15	Day 1	Day 2	Day 15	Day 1	Day 2	Day 1	Day 2	Day 1	Day 8	Day 1		
Assessments[a]																								
Informed Consent[b]	x																							
Eligibility	x																							
Demography	x																							
Medical History	x																							
Physical Examination[c]	x	x			x		x		x		x	x	X		x	x		x		x	x	x	x	x
Vital Signs	x	x	x	X	x	x	x	x	x	x	x	x	X	X	x	x	x	x	x	x	x	x[p]	x	x
Administration of atezolizumab Q3W[x]		x[z]							x				X			x		x		x		x		
Administration of RO6958688 QW[y]		x[z]			x		x		x		x	x	X			x		x		x		x[ae]		
ECG-12 lead[d]	x	x			x		x		x		x	x	X		x	x		x		x	x	x	x	x
ECOG Performance Status	x	x			x		x		x		x	x	X		x	x		x		x	x	x[q]	x	x
Pulmonary Function test[aj]	x																							
Hematology[g]	x	x	x	X	x	x	x	x	x	x	x	x	X	X	x	x	x	x	x	x	x	x[r]	x	x
Blood Chemistry[g,aa]	x	x	x	X	x	x	x	x	x	x	x	x	X	X	x	x	x	x	x	x	x	x[r]	x	x
Serum Thyroid-Stimulating Hormone Free T3-T4[i]		x																x					x	x
Coagulation[g,h]	x	x	x	X	x	x	x	x	x	x	x	x	X	X	x	x	x	x	x	x	x	x[r]	x	x
Urinalysis[g]	x	x	x	X	x	x	x	x	x	x	x	x	X	X	x	x	x	x	x	x	x	x[r]	x	x
HBV and HCV Serology	x																							
Auto-Antibody Panel[v]	x								x							x				x		x[w]		
Pregnancy Test[e]	x	X																					x	x

RO6958688 and Atezolizumab—F. Hoffmann-La Roche Ltd

"Use of this document is governed by the terms of use on the first page of this document."

TABLE A8: SCHEDULE OF ASSESSMENTS STEP UP 1200 MG (COHORT B1) RO6958688 ADMINISTRATION SCHEDULE (CONT.)

Cycle	Screening	Cycle 1							Cycle 2				Cycle 3			Cycle 4		Cycle 5		Cycle 6		Cycle 7 onwards	End of treatment	28-Day Safety Follow Up[m]
Day	D-28 to D-1	Day 1[o]	Day 2	Day 3	Day 8	Day 9	Day 15	Day 16	Day 1	Day 2	Day 8	Day 15	Day 1	Day 2	Day 15	Day 1	Day 2	Day 1	Day 2	Day 1	Day 8	Day 1		
Atezolizumab PK Sampling[ab]		x			x		x		x		x	x	X			X				x		x[af]	x	x
RO6958688 PK Sampling[ab]		x	x	X	x	x	x	x	x	x	x	x	X	x	x	X	x	X	x	x	x	x[t]	x	x
Atezolizumab Anti-Drug Antibody (ADA)[ac]		x							x				X			X						x[af]	x	
RO6958688 Anti-Drug Antibody (ADA)[ac]		x			x		x		x		x	x	X	x	x	X	x	X	x	x	x	x[u]	x	x
PD Blood Flow Cytometry		x	x		x				x				x											
PD Blood Cytokines[f]		x	x	X	x	x	x	x	x	x	x	x	X	x										
Archival tumor Biopsy	x																							
Fresh Tumor Biopsy[j]	x								x				X											
Clinical Genotyping[ag]		x																						
RCR Sample (DNA and RNA)[ag]		x																						
CEA confirmation on Archival Tissue[k]	x																							
FDG-PET[n]	x[s]										x[s]										x[s]			
TCR Vβ[ah]		x											x											
Sol CEA[ai]		x							x				x											
Tumor Assessment[l]	x														x						x		x	
Tumor Growth Kinetic[ad]	x																							
Adverse Events	X																							
Previous and Concomitant Treatments	X																							

“Use of this document is governed by the terms of use on the first page of this document.”

Appendix 1
Schedule of Assessments (cont.)

TABLE A8: SCHEDULE OF ASSESSMENTS STEP UP 1200 MG (COHORT B1) RO6958688 ADMINISTRATION SCHEDULE (CONT.)

Notes:

ADA = anti-drug antibody; CEA = carcinoembryonic antigen; D = day; ECOG = Eastern Cooperative Oncology Group; PD = pharmacodynamics; PK = pharmacokinetic.

a All visit and their assigned safety assessments (hematology, blood chemistry, coagulation, urinalysis, physical examination), unless otherwise indicated should occur within a 3-day time window.

b Informed consent must be obtained before any study-specific procedures. All patients will be tested for HBV and HCV serology. Patients with HIV infection, or active hepatitis B (chronic or acute), or active hepatitis C infection are not eligible.

c Physical examinations (including weight) and ECOG Performance Status will be done at screening, at the time of treatment administration, at the EOT and at the 28-day safety follow-up visits. Results must be obtained prior to infusion.

d Triplicate 12-lead ECG at screening (within 7 days before first dose of RO6958688) pre- and end of infusion on Cycle 1 Day 1 and Cycle 1 Day 15 and at the 28-day safety follow-up visit. Pre-infusion at all other study drug administrations. Additional unscheduled ECG assessments should be performed in case of abnormalities and if clinical symptoms occur. Recording must be done prior to PK sampling.

e Serum pregnancy test at screening, within 7 days prior to first dose. From the date of first serum pregnancy test at screening, a urine pregnancy test must be performed every 4 weeks. Also to be performed at study termination and safety follow-up visit (28 days post last infusion).

f Serum or plasma samples for the assessment of cytokine (PD blood cytokines) release will be collected. At the time of an IRR, please see IRR SoA Table A3.

g Hematology, blood chemistry, coagulation, and urinalysis can be performed up to 24 hours (72h if during weekend) prior to scheduled dosing. Results must be obtained prior to infusion. Soluble CEA will be measured as part of the blood chemistry measurements on Cycle 1 Day 1 and every 6 weeks thereafter for patients who continue treatment and at the end of treatment visit. Soluble CD25 is to be obtained at screening and during the treatment period at Day 1 of every cycle (samples can be drawn 7 days prior to Day 1 of each cycle). Before IMP administration on Day 1 of each cycle, the investigator should confirm that the result of soluble CD25 measurement from the previous week or if available from the same day is not significantly high. If there is a significantly high level, the investigator should discuss with the Medical Monitor if the patient can or cannot receive further IMP administration. Ferritin should be obtained at screening and every week during the treatment period: Day 1, Day 8 and Day 15 of every cycle (-2 days) when applicable.

h For coagulation (including PT/INR and PTT), an additional sample will be taken at the time of an IRR or hypersensitivity reaction, see IRR SoA Table A3. Additional coagulation parameters (i.e., first chromogenic antithrombin III then antigenic antithrombin III in case of chromogenic antithrombin III decrease, f brinogen, prothrombin time, fibrin degradation products, D-dimer) could be assessed according to clinical judgment.

i Serum TSH, free T3 (or total T3 for sites where free T3 is not performed) and T4 levels will be assessed at baseline pre-dose at Cycle 1, on study (every 3 months) and at study completion/early termination visit and safety follow-up visit (28 days after last dose of study drug).

j Mandatory tumor biopsy samples will be collected on two occasions (once at baseline and once during the study treatment period), except for NSCLC patients for whom there is no accessible lesion. On treatment biopsies will be collected as per Section 3.3.2.2. If preliminary data suggest that modification of the on-treatment tumor biopsy timepoint would be more appropriate, alternative on-treatment tumor biopsy timepoints could be considered in the future cohorts. Unscheduled tumor biopsies can be taken at any time point, if considered necessary for tumor assessment and patient management as decided by the investigator.

k CEA expression for elig bility will be confirmed centrally for U.S. patients on archival tumor material (refer to inclusion criterion #13), if available, or confirmed from a freshly obtained tumor biopsy. Results available prior to screening (outside the 28 day window) can be used.

l Tumor assessments will be performed at screening, then every 8 weeks after Cycle 1 Day 1 for the first year, and thereafter every 12 weeks for the second year until disease progression or treatment discontinuation. Optional latest pre-study CT scan should be provided for assessment of tumor growth kinetics within 6 weeks of patient entering the study. For the scheduled tumor assessments beyond Screening a (+/- 7-day window is permitted).

m Patients who complete the study or discontinue from the study early will be asked to return to the clinic 28 days after the last dose of study drugs for post-study follow-up visits.

n FDG-PET should be done at baseline (Day-14 to D-1) but before the baseline biopsy. The on-treatment FDG-PET scan must be done at week 4 (C2D8 + 1 week) and at week 16 (C6D8 and up to 72h before the visit) in case of dose delay the FDG-PET should be obtained at week 4 (+1week) and week 16. If preliminary data suggest that modification of the on-treatment FDG-PET timepoint would be more appropriate, alternative on-treatment FDG PET timepoints could be considered. Please refer to Appendix 9 for further information.

"Use of this document is governed by the terms of use on the first page of this document."

Appendix 1
Schedule of Assessments (cont.)

TABLE A8: SCHEDULE OF ASSESSMENTS STEP UP 1200 MG (COHORT B1) RO6958688 ADMINISTRATION SCHEDULE (CONT.)

o At least 24 hours (overnight) hospital stay required following administration of study drug at Cycle 1 Day 1.

p Vital signs to be assessed at D1 from cycle 7 onwards.

q ECOG to be assessed at D1 from cycle 7 onwards.

r Hematology, blood chemistry, coagulation and urinalysis (to be drawn at D1 from cycle 7 onwards) can be performed up to 24 hours (72h if during weekend) prior to scheduled dosing. Results must be obtained prior to atezolizumab and RO6958688 infusion. Before each IMP administration on Day 1 or each cycle, the investigator should confirm that the result of soluble CD25 measurement from the previous week or if available from the same day is not significantly high. If there is a significantly high level, the investigator should discuss with the Medical Monitor if the patient can or cannot receive further IMP administration.

s For FDG PET assessment, body weight, last calorific meal information, glucose level are needed (see Appendix 9 of the protocol for more information).

t RO6958688 PK sampling to be drawn preinfusion and at end of infusion at D1 of each cycle from Cycle 7 to Cycle 10. At Cycle 11 and every second cycle, RO6958688 PK samples to be drawn preinfusion and at end of infusion on D1.

u RO6958688 ADA sampling to be drawn preinfusion at D1 of each cycle from Cycle 7 to Cycle 10. At cycle 11 and every second cycle, RO6958688 ADA samples to be drawn preinfusion on Day 1 at the mentioned timepoints.

v In patients who develop signs and/or symptoms suggestive of auto-immune disease while on treatment, the antibody panel should be repeated. The autoant body panel will be assessed at screening, pre-dose every second cycle, and every 3 months after the third month on therapy (samples can be drawn up to 1 week before Day 1 visit).

w To be performed every 3 months after Cycle 6 (ie. Cycle 10, Cycle 14…).

x Atezolizumab will be delivered over 60 (+/-15) minutes. If the first infusion is tolerated without infusion associated adverse events, the second infusion may be delivered over 30 (+/-10) minutes. If the 30 minute infusion is well tolerated, all subsequent infusions may be delivered over 30 (+/-10) minutes.

y The start of the RO6958688 infusion on Day 1 of each cycle should be after the end of the atezolizumab infusion as per Section 4.3.2.3.

z Prophylactic corticosteroids to be administered at C1D1 as per Table 2. The use of analgesics and/or antihistamines is allowed to minimize expected flu like symptoms prior to the first RO6958688 administration.

aa Serum CEA will also be measured as a disease monitoring marker (as part of the serum chemistry panel).

ab Blood for PK should always be drawn at the indicated time point.

ac Additional samples will be drawn at the time of an IRR, see IRR SoA Table A3 or hypersensitivity reaction, treatment discontinuation *and* at the 28-day safety follow-up visit (RO6958688 only). A PK sample will be taken at the time of ADA sampling.

ad An exploratory assessment of tumor growth kinetics will be made by comparing post-treatment scans with at least 2 pre-treatment scans not older than 12 weeks prior to C1D1, if available. The two pre-treatment scans will consist of a pre-treatment scan (if available) and the study baseline scan, and will allow estimation of tumor growth rate before start of treatment.

ae Administration of RO6958688 to be done at D1 from Cycle 7 onwards.

af Atezolizumab PK and ADA sampling to be drawn at Cycle 8 Day 1 then every 8 cycles from this timepoint onwards.

ag Clinical genotyping and RCR (DNA and RNA) samples to be drawn before any IMP administration.

ah For TCR Vβ, at each time point Cycle 1 Day 1 and Cycle 3 Day 1, 2 whole blood samples should be drawn. Samples should be taken prior to IMP administration

ai For soluble CEA central assessment: samples will be collected pre-dose at: C1D1 (baseline), C2D1 and C3D1.

aj Evaluate DLCO (corrected for both alveolar volume and hemoglobin) and FEV1/VC/TLC at screening for patients with bilateral lung metastases or patients with lobectomy or pneumonectomy with lung metastases in the remaining lung. Additional tests may be performed if clinically indicated.

"Use of this document is governed by the terms of use on the first page of this document."

Appendix 1
Schedule of Assessments (cont.)

TABLE A9: SCHEDULE OF HOURLY ASSESSMENTS STEP UP 1200 MG (COHORT B1) RO6958688 ADMINISTRATION SCHEDULE

Cycle	Day	Scheduled Time (h)	Vital Signs[b,n,o]	ECG-12 lead[c]	RO6958688 PK Sampling[d]	Atezolizumab PK Sampling[e]	PD Blood Flow Cytometry[m]	PD Blood Cytokines[f]	RO6958688 Anti-Drug Ant body (ADA)[g]	Atezolizumab Anti-Drug Antibody (ADA)[g]	Tumor Biopsy[h]	FDG PET[i]	TCR Vβ[l]
Screening	D-28 to D1[a]		x	x							x	x	
Cycle 1	Day 1	Pre-infusion[j]	x	x	x	x	X	X	x	x			x
		0.5h (+/- 10 min) atezolizumab post EOI				x							
		End of RO6958688 infusion	x	x	x			X					
		2 hours post end of RO6958688 infusion			x			X					
		4 hours post end of RO6958688 infusion					X	X					
	Day 2	24 hours	x		x		X	X					
	Day 3	48 hours	x		x			X					
	Day 8	Pre-infusion	x	x	x	x	X	X	x				
		End of RO6958688 infusion	x		x			X					
		2 hours post end of RO6958688 infusion			x			X					

“Use of this document is governed by the terms of use on the first page of this document.”

Appendix 1
Schedule of Assessments (cont.)

TABLE A9: SCHEDULE OF HOURLY ASSESSMENTS STEP UP 1200 MG (COHORT B1) RO6958688 ADMINISTRATION SCHEDULE (CONT.)

Cycle	Day	Scheduled Time (h)	Vital Signs[b,n,o]	ECG-12 lead[c]	RO6958688 PK Sampling[d]	Atezolizumab PK Sampling[e]	PD Blood Flow Cytometry[m]	PD Blood Cytokines[f]	RO6958688 Anti-Drug Ant body (ADA)[g]	Atezolizumab Anti-Drug Antibody (ADA)[g]	Tumor Biopsy[h]	FDG PET[i]	TCR Vβ[l]
Cycle 1	Day 9	192 hours	x		x			X					
	Day 15	Pre-infusion	x	x	x	x		X	x				
		End of infusion	x	x	x			X					
		2 hours post end of infusion			x								
		4 hours post end of RO6958688 infusion						X					
	Day 16	360 hours	x		x			X					
Cycle 2	Day 1	Pre-infusion[j]	x	x	x	x	X	X	x	x	x		
		0.5h (+/- 10 min) atezolizumab post EOI				x							
		End of RO6958688 infusion	x		x			X					
		2 hours post end of RO6958688 infusion			x								
	Day 2	24 hours	x		x			X					
	Day 8	Pre-infusion	x	x	x	x		X	x			x	
		End of RO6958688 infusion			x			X					

"Use of this document is governed by the terms of use on the first page of this document."

TABLE A9: SCHEDULE OF HOURLY ASSESSMENTS STEP UP 1200 MG (COHORT B1) RO6958688 ADMINISTRATION SCHEDULE (CONT.)

Cycle	Day	Scheduled Time (h)	Vital Signs[b,n,o]	ECG-12 lead[c]	RO6958688 PK Sampling[d]	Atezolizumab PK Sampling[e]	PD Blood Flow Cytometry[m]	PD Blood Cytokines[f]	RO6958688 Anti-Drug Antibody (ADA)[g]	Atezolizumab Anti-Drug Antibody (ADA)[g]	Tumor Biopsy[h]	FDG PET[i]	TCR Vβ[l]
Cycle 2	Day 15	Pre-infusion	x	x	x	x		X	x				
		End of RO6958688 infusion			x			X					
Cycle 3	Day 1	Pre-infusion[j]	x	x	x	x	x	X	x	x	x		x
		End of RO6958688 infusion			x			X					
	Day 2	24 hours	x		x			X	x				
	Day 15	336 hours	x	x	x				x				
Cycle 4	Day 1	Pre-infusion[j]	x	x	x	x			x	x			
		End of RO6958688 infusion			x								
	Day 2	24 hours	x		x				x				
Cycle 5	Day 1	Pre-infusion[j]	x	x	x				x				
		End of RO6958688 infusion			x								
	Day 2	24 hours	x		x				x				

“Use of this document is governed by the terms of use on the first page of this document.”

TABLE A9: SCHEDULE OF HOURLY ASSESSMENTS STEP UP 1200 MG (COHORT B1) RO6958688 ADMINISTRATION SCHEDULE (CONT.)

Cycle	Day	Scheduled Time (h)	Vital Signs[b,n,o]	ECG-12 lead[c]	RO6958688 PK Sampling[d]	Atezolizumab PK Sampling[e]	PD Blood Flow Cytometry[m]	PD Blood Cytokines[f]	RO6958688 Anti-Drug Antibody (ADA)[g]	Atezolizumab Anti-Drug Ant body (ADA)[g]	Tumor Biopsy[h]	FDG PET[i]	TCR Vβ[l]
Cycle 6	Day 1	Pre-infusion[j]	x	x	x	x			x				
		End of RO6958688 infusion			x								
	Day 8	168 hours	x	x	x				x			x	
Cycle 7 to Cycle 10	Day 1	Pre-infusion[j]	x	x	x	x[k]			x	x[k]			
		End of RO6958688 infusion			x								
Cycle 11 and every 2nd cycle	Day 1	Pre-infusion[j]	x	x	x				x				
		RO6958688 EOI[j]			x								
End of treatment	Anytime		x	x	x	x			x	x			
28-Day Safety Follow-up	Anytime		x	x	x	x			x				

“Use of this document is governed by the terms of use on the first page of this document.”

Appendix 1
Schedule of Assessments (cont.)

TABLE A9: SCHEDULE OF HOURLY ASSESSMENTS STEP UP 1200 MG (COHORT B1) RO6958688 ADMINISTRATION SCHEDULE (CONT.)

ADA = anti-drug antibody; CEA = carcinoembryonic antigen; D = day; ECOG = Eastern Cooperative Oncology Group; PD = pharmacodynamics; PK = pharmacokinetic.

a Screening assessments should be performed between D-28 and D-1, unless specified.

b The patient's vital signs (heart rate, respiratory rate, blood pressure, oxygen saturation and temperature) should be determined up to 60 (± 10) minutes before each atezolizumab infusion. Vital signs should also be obtained during or after the atezolizumab infusion if clinically indicated. Vital signs (including supine blood pressure and heart rate) will be monitored on Day 1 RO6958688 pre-infusion, every 15 minutes until the end of RO6958688 infusion and, thereafter, every 30 minutes until the infusion line is removed. Starting Cycle 2 Day 1: RO6958688 pre-infusion, every 30 minutes during RO6958688 infusion and every 30 minutes after the end of RO6958688 infusion until infusion line is removed. From Cycle 3 onwards vital signs will only be obtained on Day 1 RO6958688 pre-infusion. For the purposes of the eCRF vital signs will only be captured pre-infusion and in case of abnormalities

c Triplicate 12-lead ECG at screening (within 7 days before first dose of RO6958688) pre- and end of infusion on Cycle 1 Day 1 and C1D15. Pre-infusion at all other study drug administrations and at EOT and at 28-day safety follow up visit. Additional unscheduled ECG assessments should be performed in case of abnormalities and if clinical symptoms occur. Recording must be done prior to PK sampling.

d All RO6958688 PK sampling Timepoints are reference from the start of RO6958688 infusion. PK time windows: Pre-infusion: up to – 4 h - EOI: up to + 30 min - 2 h post EOI: +/- 30 min - 24 h: +/- 2 h - 48 h: +/- 4 h - Later: +/- 12 h.

e All atezolizumab PK sampling Timepoints are reference from the start of atezolizumab infusion.

f Serum or plasma samples for the assessment of cytokine (PD blood cytokines) release will be collected. At the time of an IRR please see IRR SoA Table A3.

g Additional samples will be drawn at the time of an IRR, see IRR SoA Table A3 or hypersensitivity reaction, treatment discontinuation, *and* at the 28-day safety follow-up visit (RO6958688 only). A PK sample will be taken at the time of ADA sampling.

h Mandatory tumor biopsy samples will be collected on two occasions (once at baseline and once during the study treatment period), except for NSCLC patients for whom there is no accessible lesion. If preliminary data suggest that modification of the on-treatment tumor biopsy timepoint would be more appropriate, alternative on-treatment tumor biopsy timepoints could be considered in the future cohorts.

i FDG-PET should be done at baseline (Day-14 to D-1) but before the baseline biopsy. The on-treatment FDG-PET scan must be done at week 4 (C2D8 + 1 week) and at week 16 (C6D8 pre-dose and up to 72h before the visit) in case of dose delay the FDG-PET should be obtained at week 4 (+1week) and week 16. If preliminary data suggest that modification of the on-treatment FDG-PET timepoint would be more appropriate, alternative on-treatment FDG-PET timepoints could be considered in the future cohorts.

j Pre-infusion timepoints relate to RO6958688 or atezolizumab. Pre-dose PK samples can be taken up to 1-hour before infusion start for atezolizumab; after atezolizumab end of infusion and/or before the start of RO6958688 infusion for the RO6958688 pre-dose sample.

k Atezolizumab PK and ADA sampling to be drawn at Cycle 8 Day 1 then every 8 cycles from this timepoint onwards.

l For TCR Vβ, at each time point Cycle 1 Day 1 and Cycle 3 Day 1, 2 whole blood samples should be drawn. Samples should be taken prior to IMP administration.

m Blood Flow Cytometry sample to be drawn pre-dose at C1D1, C1D8, C2D1, and C3D1.

n If a patient has experienced a Grade 3 IRR/CRS event during the previous treatment administration visit, the patient should be hospitalized for at least 24 hours after the end of infusion at next treatment administration visit during which vital signs will be monitored as follows: every 30 (± 10) minutes for the first hour post infusion, every 60 (± 15) minutes during the following 3 hours, every 120 (± 20) minutes for the next 8 hours and every 240 (± 30) minutes for the remaining 12 hours.

o If a patient experiences a Grade 3 or higher treatment-related adverse event (with the exception of IRR/CRS) within the 24 hours period following the previous infusion, the patient should be observed for at least 8 hours after the end of RO6958688 infusion at the next administration visit during which vital signs will be monitored as follows: every 30 (± 10) minutes for the first hour post infusion, every 60 (± 15) minutes during the following 3 hours, every 120 (± 20) minutes for the next 4 hours.

"Use of this document is governed by the terms of use on the first page of this document."

Appendix 2
CKD-EPI equation for Calculation of Glomerular Filtration Rate (GFR)

CKD EPI Equation for Estimating GFR Expressed for Specified Race, Sex and Serum Creatinine in mg/dL (From Levey et al. Ann Intern Med 2009;150:604-612).

Race	Sex	Serum Creatinine, S_{cr} (mg/dL)	Equation (age in years for ≥ 18)
Black	Female	⩽ 0.7	GFR = 166 × $(S_{cr}/0.7)^{-0.329}$ × $(0.993)^{Age}$
Black	Female	> 0.7	GFR = 166 × $(S_{cr}/0.7)^{-1.209}$ × $(0.993)^{Age}$
Black	Male	⩽ 0.9	GFR = 163 × $(S_{cr}/0.9)^{-0.411}$ × $(0.993)^{Age}$
Black	Male	> 0.9	GFR = 163 × $(S_{cr}/0.9)^{-1.209}$ × $(0.993)^{Age}$
White or other	Female	⩽ 0.7	GFR = 144 × $(S_{cr}/0.7)^{-0.329}$ × $(0.993)^{Age}$
White or other	Female	> 0.7	GFR = 144 × $(S_{cr}/0.7)^{-1.209}$ × $(0.993)^{Age}$
White or other	Male	⩽ 0.9	GFR = 141 × $(S_{cr}/0.9)^{-0.411}$ × $(0.993)^{Age}$
White or other	Male	> 0.9	GFR = 141 × $(S_{cr}/0.9)^{-1.209}$ × $(0.993)^{Age}$

Normal glomerular filtration rate by age:

Age	Average estimated GFR
20-29	116
30-39	107
40-49	99
50-59	93
60-69	85
70+	75

"Use of this document is governed by the terms of use on the first page of this document."

Appendix 3
Eastern Cooperative Oncology Group Performance Status

ECOG PERFORMANCE STATUS	
**Grade**	**ECOG**
0	Fully active, able to carry on all pre-disease performance without restriction
1	Restricted in physically strenuous activity but ambulatory and able to carry out work of a light or sedentary nature, e.g., light house work, office work
2	Ambulatory and capable of all selfcare but unable to carry out any work activities. Up and about more than 50% of waking hours
3	Capable of only limited selfcare, confined to bed or chair more than 50% of waking hours
4	Completely disabled. Cannot carry on any selfcare. Totally confined to bed or chair
5	Dead

“Use of this document is governed by the terms of use on the first page of this document.”

Appendix 4
Response Evaluation Criteria in Solid Tumors Version 1.1 Criteria

The investigator will evaluate response to treatment using Response Evaluation Criteria in Solid Tumors (RECIST) Version 1.1 and modified RECIST criteria.

DEFINITIONS OF MEASURABLE/NON-MEASURABLE LESIONS

At baseline, tumor lesions/lymph nodes will be categorized measurable or non-measurable as follows:

Measurable Tumor Lesions

Tumor lesions must be accurately measured in at least one dimension (longest diameter in the plane of measurement is to be recorded) with a minimum size of 10 mm by computed tomography (CT) or magnetic resonance imaging (MRI) scan (CT/MRI scan slice thickness/interval no greater than 5 mm).

Malignant lymph nodes: To be considered pathologically enlarged and measurable, a lymph node must be ≥ 15 mm in short axis when assessed by CT scan. Only the short axis will be measured and followed.

Non-Measurable Tumor Lesions

Non-measurable tumor lesions include:

- Small lesions (longest diameter < 10 mm)
- Pathological lymph nodes with ≥ 10 to < 15 mm short axis
- Truly non-measurable lesions. (e.g., ascites, pleural or pericardial effusion, lymphangitic involvement of skin or lung, peritoneal spread, abdominal masses/abdominal organomegaly identified by physical exam that is not measurable by reproducible imaging techniques).

Special considerations regarding lesion measurability:

Bone lesions, cystic lesions, and lesions previously treated with local therapy require particular comment:

Bone lesions:

- Lytic bone lesions or mixed lytic-blastic lesions with identifiable soft tissue components that can be evaluated by cross–sectional imaging techniques such as CT or MRI can be considered as measurable lesions if the soft tissue component meets the definition of measurability described above.
- Blastic bone lesions are non-measurable.

“Use of this document is governed by the terms of use on the first page of this document.”

Lesions with prior local treatment:

- Tumor lesions situated in a previously irradiated area, or in an area subjected to other loco-regional therapy, are usually not considered measurable unless there has been demonstrated progression in the lesion. Study protocols should detail the conditions under which such lesions would be considered measurable.

TARGET LESIONS: SPECIFICATIONS BY METHODS OF MEASUREMENTS

The same method of assessment and the same technique should be used to characterize each identified and reported lesion at baseline and during study.

TUMOR RESPONSE EVALUATION

Baseline documentation of 'target' and 'non-target' lesions

- When more than one measurable lesion is present at baseline, all lesions up to a maximum of five lesions total (and a maximum of two lesions per organ) representative of all involved organs should be identified as target lesions and will be recorded and measured at baseline.
- Where patients have only one or two organ sites involved a maximum of two (one site) and four lesions (two sites), respectively, can be recorded. Other lesions (including measurable lesion) in that organ will be recorded as non-measurable lesions.
- Target lesions should be selected on the basis of their size (lesions with the longest diameter), be representative of all involved organs, but in addition should be reproducible in repeated measurements.
- Lymph nodes: target lesions must have a short axis of $\geq$15 mm by CT scan. Only the short axis of these nodes will contribute to the baseline sum. Pathological lymph nodes with a short axis $\geq$10 mm but $<$15 mm should be considered as non-target lesions. Nodes that have a short axis $<$10 mm are considered non-pathological and should not be recorded or followed.
- A sum of the longest diameters (except for nodal lesions) for all target lesions will be calculated and reported as the baseline sum diameters. If lymph nodes are to be included in the sum then only the short axis is added to the sum. The baseline sum diameters will be used as reference to further characterize any objective tumor regression in the measurable dimension of the disease.
- Measurements are not required for non-target lesions. These should be followed as 'present', 'absent', or in rare cases 'unequivocal progression'.

RESPONSE CRITERIA

This section provides the definitions of the criteria used to determine objective tumor response for target lesions.

"Use of this document is governed by the terms of use on the first page of this document."

EVALUATION OF TARGET LESIONS

- Complete Response (CR): Disappearance of all target lesions. Any pathological lymph nodes (whether target or non-target) must have reduction in short axis to < 10 mm.
- Partial Response (PR): At least a 30% decrease in the sum of diameters of target lesions, taking as reference the baseline sum diameters.
- Progressive Disease (PD): At least a 20% increase in the sum of diameters of target lesions, taking as reference the smallest sum on study including baseline (nadir). In addition to the relative increase of 20%, the sum must also demonstrate an absolute increase of at least 5 mm.
- Stable Disease (SD): Neither sufficient shrinkage to qualify for PR nor sufficient increase to qualify for PD taking as a reference the smallest sum diameters while on study.

EVALUATION OF NON-TARGET LESIONS

- Complete Response (CR): Disappearance of all non-target lesions (and, if applicable, normalization of tumor marker level). All lymph nodes must be non-pathological in size (< 10 mm short axis).
- Non-CR/Non-PD: Persistence of one or more non-target lesion(s) and/or maintenance of tumor marker level above the normal limits.
- Progressive Disease (PD): Unequivocal progression of existing non-target lesions. The appearance of one or more new lesions is also considered progression.

NEW LESIONS

- The appearance of new malignant lesions denotes disease progression. The finding of a new lesion should be unequivocal: i.e., not attributable to differences in scanning technique, change in imaging modality or findings thought to represent something other than tumor.
- A lesion identified during the study in an anatomical location that was not scanned at baseline is considered a new lesion and will indicate disease progression.
- If a new lesion is equivocal, for example because of its small size, continued therapy and follow-up evaluation will clarify if it represents truly new disease. If repeat scans confirm there is definitely a new lesion, then progression should be declared using the date of the initial scan.

EVALUATION OF RESPONSE

TIMEPOINT RESPONSE (OVERALL RESPONSE)

The table below provides a summary of the overall response status calculation at each timepoint for patients who have measurable disease at baseline.

“Use of this document is governed by the terms of use on the first page of this document.”

Target lesions	Non-target lesions	New lesions	Overall response
CR	CR	No	CR
CR	Non-CR/non-PD	No	PR
CR	Not evaluated	No	PR
PR	Non-PD or not all evaluated	No	PR
SD	Non-PD or not all evaluated	No	SD
Not all evaluated	Non-PD	No	NE
PD	Any	Yes or No	PD
Any	PD	Yes or No	PD
Any	Any	Yes	PD

CR = complete response, PR = partial response, SD = stable disease, PD = progressive disease, and NE = inevaluable.

Missing assessments and not-evaluable designation

When no imaging/measurement is done at all at a particular timepoint, the subject is not evaluable at that timepoint. If only a subset of measurements are made at an assessment, subject is also considered not evaluable at that timepoint, unless the missing lesion(s) would not change the assigned timepoint response, e.g., in the case of PD. If one or more target lesions were not assessed the Response for Target Lesions should be "Unable to Assess" (except where there is clear progression).

Overall response First time point	Overall response Subsequent time point	BEST overall response
CR	CR	CR
CR	PR	SD, PD or PR[a]
CR	SD	SD provided minimum criteria for SD duration met, otherwise, PD
CR	PD	SD provided minimum criteria for SD duration met, otherwise, PD
CR	NE	SD provided minimum criteria for SD duration met, otherwise NE
PR	CR	PR
PR	PR	PR
PR	SD	SD
PR	PD	SD provided minimum criteria for SD duration met, otherwise, PD
PR	NE	SD provided minimum criteria for SD duration met, otherwise NE
NE	NE	NE

CR = complete response, PR = partial response, SD = stable disease, PD = progressive disease, and NE = inevaluable.
a If a CR is truly met at first time point, then any disease seen at a subsequent time point, even disease meeting PR criteria relative to baseline, makes the disease PD at that point (since disease must have reappeared after CR). Best response would depend on whether minimum duration for SD was met. However, sometimes 'CR' may be claimed when subsequent scans suggest small lesions were likely still present and in fact the patient had PR, not CR at the first time point. Under these circumstances, the original CR should be changed to PR and the best response is PR.

"Use of this document is governed by the terms of use on the first page of this document."

Special notes on response assessment

When nodal disease is included in the sum of target lesions and the nodes decrease to 'normal' size ($<$10 mm), they may still have a measurement reported on scans. This measurement should be recorded even though the nodes are normal in order not to overstate progression should it be based on increase in size of the nodes. As noted earlier, this means that patients with CR may not have a total sum of 'zero' on the eCRF. Patients with a global deterioration of health status requiring discontinuation of treatment without objective evidence of disease progression at that time should be reported as 'symptomatic deterioration'. Every effort should be made to document objective progression even after discontinuation of treatment. Symptomatic deterioration is not a descriptor of an objective response: it is a reason for stopping study therapy. The objective response status of such patients is to be determined by evaluation of target and non-target disease.

In patients with advanced disease and the primary disease is still present or partially present, the primary tumor should be also captured under target or non-target lesions.

REFERENCES

Bogaerts J, Ford R, Sargent D, et al. Individual patient data analysis to assess modifications to the RECIST criteria. Eur J Cancer 2009;45:248–60.

Eisenhauer EA, Therasse P, Bogaerts J, et al. New response evaluation criteria in solid tumors: Revised RECIST guideline (version 1.1). Eur J Cancer 2009;45:228–47.

"Use of this document is governed by the terms of use on the first page of this document."

Appendix 5
Modified Response Evaluation Criteria in Solid Tumors

Conventional response criteria may not be adequate to characterize the anti-tumor activity of immunotherapeutic agents like atezolizumab, which can produce delayed responses that may be preceded by initial apparent radiological progression, including the appearance of new lesions. Therefore, modified response criteria have been developed that account for the possible appearance of new lesions and allow radiological progression to be confirmed at a subsequent assessment.

Modified Response Evaluation Criteria in Solid Tumors (RECIST) is derived from RECIST, Version 1.1 (v1.1) conventions[1] and immune-related response criteria[2] (irRC). When not otherwise specified, RECIST v1.1 conventions will apply.

Modified RECIST and RECIST v1.1: Summary of Changes

	RECIST v1.1	Modified RECIST
New lesions after baseline	Define progression	New measurable lesions are added into the total tumor burden and followed.
Non-target lesions	May contribute to the designation of overall progression	Contribute only in the assessment of a complete response
Radiographic progression	First instance of ≥ 20% increase in the sum of diameters or unequivocal progression in non-target disease	Determined only on the basis of measurable disease

RECIST = Response Evaluation Criteria in Solid Tumors.

A. DEFINITIONS OF MEASURABLE/NON-MEASURABLE LESIONS

All measurable and non-measurable lesions should be assessed at Screening and at the protocol-specified tumor assessment timepoints. Additional assessments may be performed, as clinically indicated for suspicion of progression.

---

1 Eisenhauer et al. Eur J Cancer 2009;45: 228–47; Topalian et al. N Engl J Med 2012;366:2443–54; and Wolchok et al., Clin Can Res 2009;15:7412–20.
2 Wolchok et al. Clin Can Res 2009;15:7412–20; Nishino et al. J Immunother Can 2014;2:17; Nishino et al. Clin Can Res 2013;19:3936–43.

“Use of this document is governed by the terms of use on the first page of this document.”

A.1 MEASURABLE LESIONS

Tumor Lesions. Tumor lesions must be accurately measured in at least one dimension (longest diameter in the plane of measurement is to be recorded) with a minimum size as follows:

- 10 mm by computed tomography (CT) or magnetic resonance imaging (MRI) scan (CT/MRI scan slice thickness/interval no greater than 5 mm)
- 10-mm caliper measurement by clinical examination (lesions that cannot be accurately measured with calipers should be recorded as non-measurable)

Malignant Lymph Nodes. To be considered pathologically enlarged and measurable, a lymph node must be $\geq$15 mm in the short axis when assessed by CT scan (CT scan slice thickness recommended to be no greater than 5 mm). At baseline and follow-up, only the short axis will be measured and followed.

A.2 NON-MEASURABLE LESIONS

Non-measurable tumor lesions encompass small lesions (longest diameter $<$10 mm or pathological lymph nodes with short axis $\geq$10 but $<$15 mm), as well as truly non-measurable lesions. Lesions considered truly non-measurable include leptomeningeal disease, ascites, pleural or pericardial effusion, inflammatory breast disease, lymphangitic involvement of skin or lung, peritoneal spread, and abdominal mass/abdominal organomegaly identified by physical examination that is not measurable by reproducible imaging techniques.

A.3 SPECIAL CONSIDERATIONS REGARDING LESION MEASURABILITY

Bone lesions, cystic lesions, and lesions previously treated with local therapy require particular comment, as outlined below.

Bone Lesions

Bone scan, positron emission tomography (PET) scan, or plain films are not considered adequate imaging techniques for measuring bone lesions. However, these techniques can be used to confirm the presence or disappearance of bone lesions.

Lytic bone lesions or mixed lytic–blastic lesions, with identifiable soft tissue components, that can be evaluated by cross-sectional imaging techniques such as CT or MRI can be considered as measurable lesions if the soft tissue component meets the definition of measurability described above.

Blastic bone lesions are non-measurable.

“Use of this document is governed by the terms of use on the first page of this document.”

Cystic Lesions

Lesions that meet the criteria for radiographically defined simple cysts should not be considered as malignant lesions (neither measurable nor non-measurable) since they are, by definition, simple cysts.

Cystic lesions thought to represent cystic metastases can be considered as measurable lesions, if they meet the definition of measurability described above. However, if non-cystic lesions are present in the same patient, these are preferred for selection as target lesions.

Lesions with Prior Local Treatment

Tumor lesions situated in a previously irradiated area or in an area subjected to other loco-regional therapy are usually not considered measurable unless there has been demonstrated progression in the lesion. Study protocols should detail the conditions under which such lesions would be considered measurable.

B. TUMOR RESPONSE EVALUATION

B.1 DEFINITIONS OF TARGET/NON-TARGET LESIONS

Target Lesions

When more than one measurable lesion is present at baseline, all lesions up to a maximum of five lesions total (and a maximum of two lesions per organ) representative of all involved organs should be identified as target lesions and will be recorded and measured at baseline. This means that, for instances in which patients have only one or two organ sites involved, a maximum of two lesions (one site) and four lesions (two sites), respectively, will be recorded. Other lesions (albeit measurable) in those organs will be recorded as non-measurable lesions (even if the size is $>$10 mm by CT scan).

Target lesions should be selected on the basis of their size (lesions with the longest diameter) and be representative of all involved organs, but in addition, should lend themselves to reproducible repeated measurements. It may be the case that, on occasion, the largest lesion does not lend itself to reproducible measurement, in which circumstance, the next largest lesion that can be measured reproducibly should be selected.

Lymph nodes merit special mention since they are normal anatomical structures that may be visible by imaging even if not involved by tumor. As noted above, pathological nodes that are defined as measurable and may be identified as target lesions must meet the criterion of a short axis of $\geq$15 mm by CT scan. Only the short axis of these nodes will contribute to the baseline sum. The short axis of the node is the

“Use of this document is governed by the terms of use on the first page of this document.”

diameter normally used by radiologists to judge if a node is involved by solid tumor. Nodal size is normally reported as two dimensions in the plane in which the image is obtained (for CT, this is almost always the axial plane; for MRI, the plane of acquisition may be axial, sagittal, or coronal). The smaller of these measures is the short axis. For example, an abdominal node that is reported as being 20 mm $\times$ 30 mm has a short axis of 20 mm and qualifies as a malignant, measurable node. In this example, 20 mm should be recorded as the node measurement. All other pathological nodes (those with short axis $\geq$ 10 mm but $<$ 15 mm) should be considered non-target lesions. Nodes that have a short axis of $<$ 10 mm are considered non-pathological and should not be recorded or followed.

Lesions irradiated within 3 weeks prior to Cycle 1, Day 1 may not be counted as target lesions.

Non-Target Lesions

All other lesions (or sites of disease), including pathological lymph nodes, should be identified as non-target lesions and should also be recorded at baseline. Measurements are not required.

It is possible to record multiple non-target lesions involving the same organ as a single item on the Case Report Form (CRF) (e.g., “multiple enlarged pelvic lymph nodes” or “multiple liver metastases”).

After baseline, changes in non-target lesions will contribute only in the assessment of complete response (i.e., a complete response is attained only with the complete disappearance of all tumor lesions, including non-target lesions) and will not be used to assess progressive disease.

New Lesions

During the study, all new lesions identified and recorded after baseline must be assessed at all tumor assessment timepoints. New lesions will also be evaluated for measurability with use of the same criteria applied to prospective target lesions at baseline per RECIST, (e.g., non–lymph node lesions must be $\geq$ 10mm; see note for new lymph node lesions below). Up to a maximum of five new lesions total (and a maximum of two lesions per organ), all with measurements at all timepoints, can be included in the tumor response evaluation. New lesion types that would not qualify as target lesions per RECIST cannot be included in the tumor response evaluation.

New lesions that are not measurable at first appearance but meet measurability criteria at a subsequent timepoint will be measured from that point on and contribute to the sum

“Use of this document is governed by the terms of use on the first page of this document.”

of longest diameters (SLD), if the maximum number of 5 measurable new lesions being followed has not been reached.

B.2 CALCULATION OF SUM OF THE DIAMETERS

A sum of the diameters (longest for non-nodal lesions, short axis for nodal lesions) for all target lesions will be calculated as a measure of tumor burden.

The sum of the diameters is calculated at baseline and at each tumor assessment for the purpose of classification of tumor responses.

Sum of the Diameters at Baseline: The sum of the diameters for all target lesions identified at baseline prior to treatment on Day 1.

Sum of the Diameters at Tumor Assessment: For every on-study tumor assessment collected per protocol or as clinically indicated the sum of the diameters at tumor assessment will be calculated using tumor imaging scans. All target lesions selected at baseline and up to five new measurable lesions (with a maximum of two new lesions per organ) that have emerged after baseline will contribute to the sum of the diameters at tumor assessment. Hence, each net percentage change in tumor burden per assessment with use of modified RECIST accounts for the size and growth kinetics of both old and new lesions as they appear.

Note: In the case of new lymph nodes, RECIST v1.1 criteria for measurability (equivalent to baseline target lesion selection) will be followed. That is, if at first appearance the short axis of a new lymph node lesion ≥ 15 mm, it will be considered a measureable new lesion and will be tracked and included in the SLD. Thereafter, the lymph node lesion will be measured at subsequent timepoints and measurements will be included in the SLD, even if the short axis diameter decreases to < 15 mm (or even < 10 mm). However, if it subsequently decreases to < 10 mm, and all other lesions are no longer detectable (or have also decreased to a short axis diameter of < 10 mm if lymph nodes), then a response assessment of CR may be assigned.

If at first appearance the short axis of a new lymph node is ≥ 10 mm and < 15 mm, the lymph node will not be considered measurable but will still be considered a new lesion. It will not be included in the SLD unless it subsequently becomes measurable (short axis diameter ≥ 15 mm).

The appearance of new lymph nodes with diameter < 10 mm should not be considered pathological and not considered a new lesion.

“Use of this document is governed by the terms of use on the first page of this document.”

B.3 RESPONSE CRITERIA

Timepoint Response

It is assumed that at each protocol-specified timepoint, a response assessment occurs. Table 1 provides a summary of the overall response status calculation at each timepoint for patients who have measurable disease at baseline.

Complete Response (CR): Disappearance of all target and non-target lesions. Lymph nodes that shrink to <10 mm short axis are considered normal.

Partial Response (PR): At least a 30% decrease in the sum of the diameters of all target and all new measurable lesions, taking as reference the baseline sum of diameters, in the absence of CR.

Note: the appearance of new measurable lesions is factored into the overall tumor burden, but *does not automatically qualify as progressive disease* until the sum of the diameters increases by $\geq$20% when compared with the sum of the diameters at nadir.

Stable Disease (SD): Neither sufficient shrinkage to qualify for PR nor sufficient increase to qualify for PD, taking as reference the smallest sum of the diameters while in the study.

Progressive Disease (PD): At least a 20% increase in the sum of diameters of all target and selected new measurable lesions, taking as reference the smallest sum during the study (nadir SLD; this includes the baseline sum if that is the smallest during the study). In addition to the relative increase of 20%, the sum must also demonstrate an absolute increase of at least 5 mm.

Impact of New Lesions on Modified RECIST

New lesions alone do not qualify as progressive disease. However, their contribution to total tumor burden is included in the sum of the diameters, which is used to determine the overall modified RECIST tumor response.

Missing Assessments and Not Evaluable Designation

When no imaging/measurement is done at all at a particular timepoint, the patient is considered not evaluable (NE) at that timepoint. If only a subset of lesion measurements are made at an assessment, usually the case is also considered NE at that timepoint, unless a convincing argument can be made that the contribution of the individual missing lesion(s) would not change the assigned timepoint response. This would only happen in the case of PD. For example, if a patient had a baseline sum of 50 mm with three measured lesions and at follow-up only two lesions were assessed but

“Use of this document is governed by the terms of use on the first page of this document.”

those gave a sum of 80 mm, the patient will be assigned PD status, regardless of the contribution of the missing lesion.

Table 1 Modified RECIST Timepoint Response Definitions

% Change in Sum of the Diameters [a]	Non-Target Lesion Response Assessment	Overall Modified RECIST Timepoint Response
− 100% from baseline [b]	CR	CR
− 100% from baseline [b]	Non-CR or not all evaluated	PR
≤ − 30% from baseline	Any	PR
> − 30% to < + 20%	Any	SD
Not all evaluated	Any	NE
≥ + 20%from nadir SLD	Any	PD

CR = complete response; NE = not evaluable; PD = progressive disease; PR = partial response; RECIST = Response Evaluation Criteria in Solid Tumors; SD = stable disease; SLD = sum of the longest diameter.

[a] Percent change in sum of the diameters (including measurable new lesions when present).

[b] When lymph nodes are included as target lesions, the % change in the sum of the diameters may not be 100% even if complete response criteria are met, since a normal lymph node is defined as having a short axis of < 10 mm. Any pathological lymph nodes (whether target or non-target) must have reduction in short axis to < 10 mm in order to meet the definition of CR.

“Use of this document is governed by the terms of use on the first page of this document.”

Appendix 6
Statistical Design of Modified Continual Reassessment Method with Escalation with Overdose Control

Various dose-toxicity scenarios have been investigated in order to cover a wide range of dose-toxicity possibilities and to be able to quantify the risk and benefit, should these scenarios actually occur.

In Table 1 the scenario settings are described in terms of the assumed parameters α and β and the reference dose d^* for a standard logistic regression model (see Section 6.7.1 – note that for the scenario specifications no mixture model is used). The dose range [mg] that results from the requirement of target toxicity between 20% and 30% is given in the last two columns. Scenario 2 is closest to the prior model, and scenario 3 is an extreme case with very high toxicity already at very low doses.

Table 1 Scenario Settings

No.	α	β	d^*	Lower target [mg]	Upper target [mg]
1	-3.00	2.00	80	179	235
2	0.50	0.30	500	0.9	5.6
3	1.30	0.30	45	< 0.052	< 0.052
4	-2.00	1.50	40	60	86
5	-5.00	5.00	30	62	69
6	-2.00	2.00	500	680	890
7	-5.00	0.20	35	> 1000	> 1000
8	1.30	1.50	5	0.8	1.2
9	-2.50	2.00	200	349	457

"Use of this document is governed by the terms of use on the first page of this document."

Figure 1, the resulting dose-toxicity curves from the scenarios (different colors and line types as per legend) are compared with the prior mean curve (grey continuous line). Scenario 2 is closest to the prior, while scenarios 6, 7, 9 have lower toxicity with varying steepness of the dose-toxicity curve and scenarios 1, 3, 4, 5 and 8 have higher toxicity than the prior curve from varying dose ranges onwards.

Operating characteristics were tabulated for each of these scenarios, as per Table 5 in Section 6.3. The numbers were computed based on 400 simulations for each of the scenarios. Four patients were used for each cohort, reflecting the typically used cohort size. In line with the protocol, the first patient was simulated separately. If a dose-limiting toxicity (DLT) occurred in this first patient, the cohort was closed and the next dose was recommended by the modified continual reassessment method (mCRM) with escalation with overdose control (EWOC) design. Otherwise 3 additional patients were recruited into the cohort, and only then the next dose was recommended by the mCRM with EWOC design. If at one point during the trial, no dose was acceptable because of the overdosing rule (the risk of having more than 30% DLT probability must be $<$ 25%), then the trial is stopped and a dose 0 is returned as the dose recommendation. Furthermore, if the maximum dose in the dose grid (1000 mg) is reached, at least 3 patients are already enrolled at that dose and the probability of underdosing (< 20% DLT probability) is greater than 50%, then the trial is stopped and 1000 mg is returned as the final dose recommendation.

In Figures 2–10, additional insight into the operating characteristics can be gained by graphical summaries of the distributions of important read-outs and resulting model fits, separately for each scenario. The frequentist distributions of the sample size n (“number of patients in total”), the final maximum-tolerated dose (MTD) estimate, the proportion of DLTs (%) and the number of overdosed patients $n_{\text{overdose}}\mathrm{n}_{\text{overdose}}$ (“number of patients above target”) are shown as histograms. In addition, the bottom panel in each figure compares the true toxicity curve from the scenario with the average model fit from the 400 simulation runs. Overall it can be said that the estimated curves approximate the true curves reasonably well in all scenarios in the relevant range up to ca. 30% probability of DLT.

“Use of this document is governed by the terms of use on the first page of this document.”

Figure 1 Comparison of Scenario Dose-Toxicity Curves

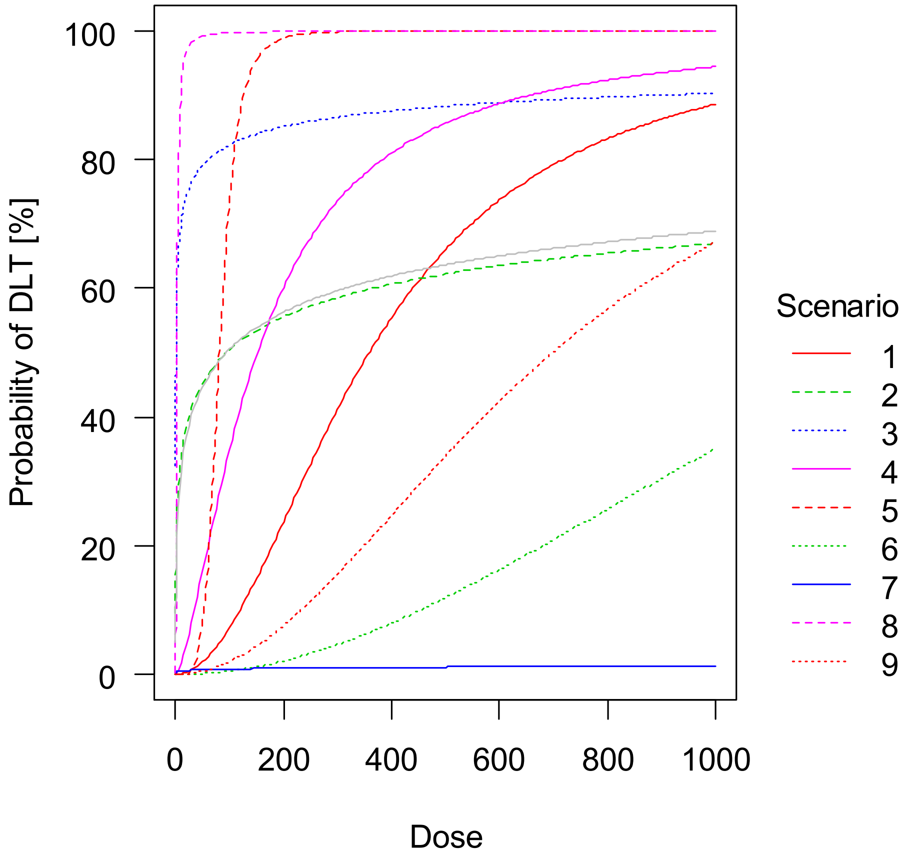

"Use of this document is governed by the terms of use on the first page of this document."

Figure 2 True versus Average Estimated Toxicity Curve in Scenario 1

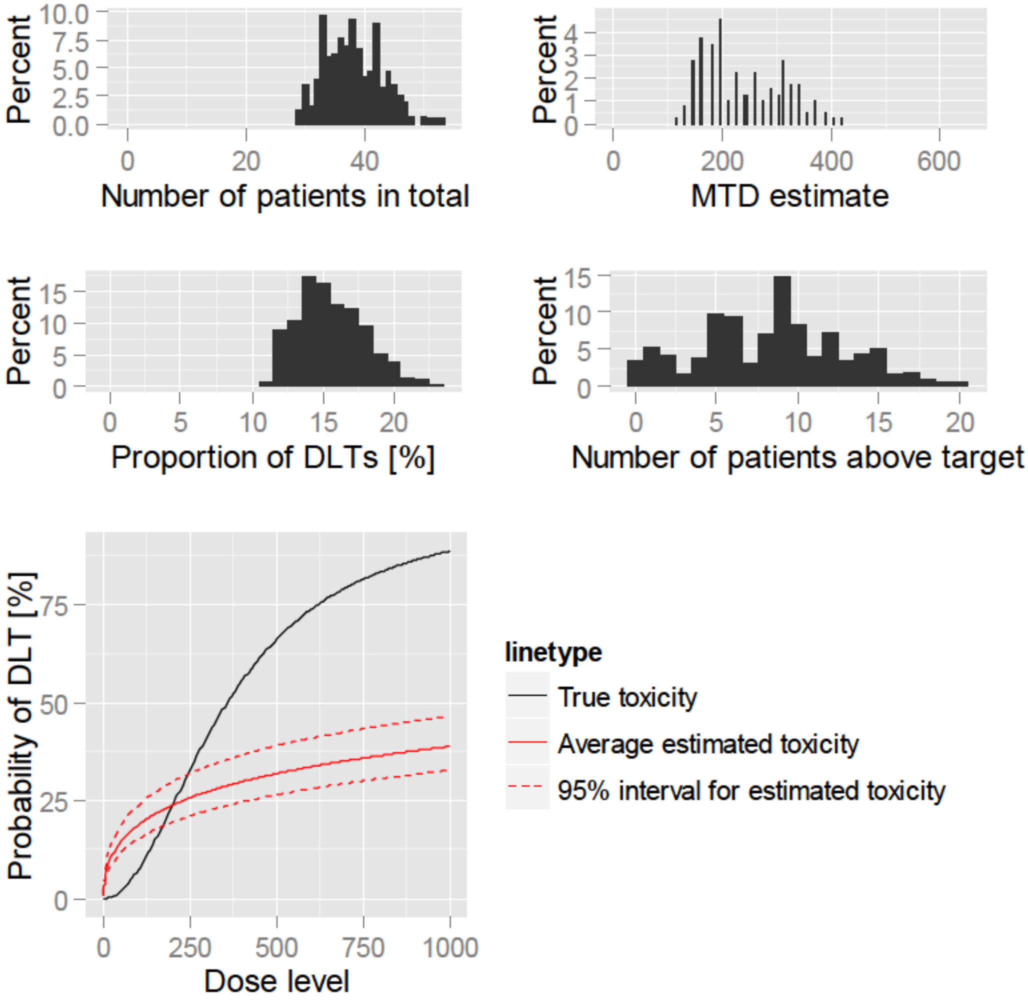

“Use of this document is governed by the terms of use on the first page of this document.”

Figure 3 True versus Average Estimated Toxicity Curve in Scenario 2

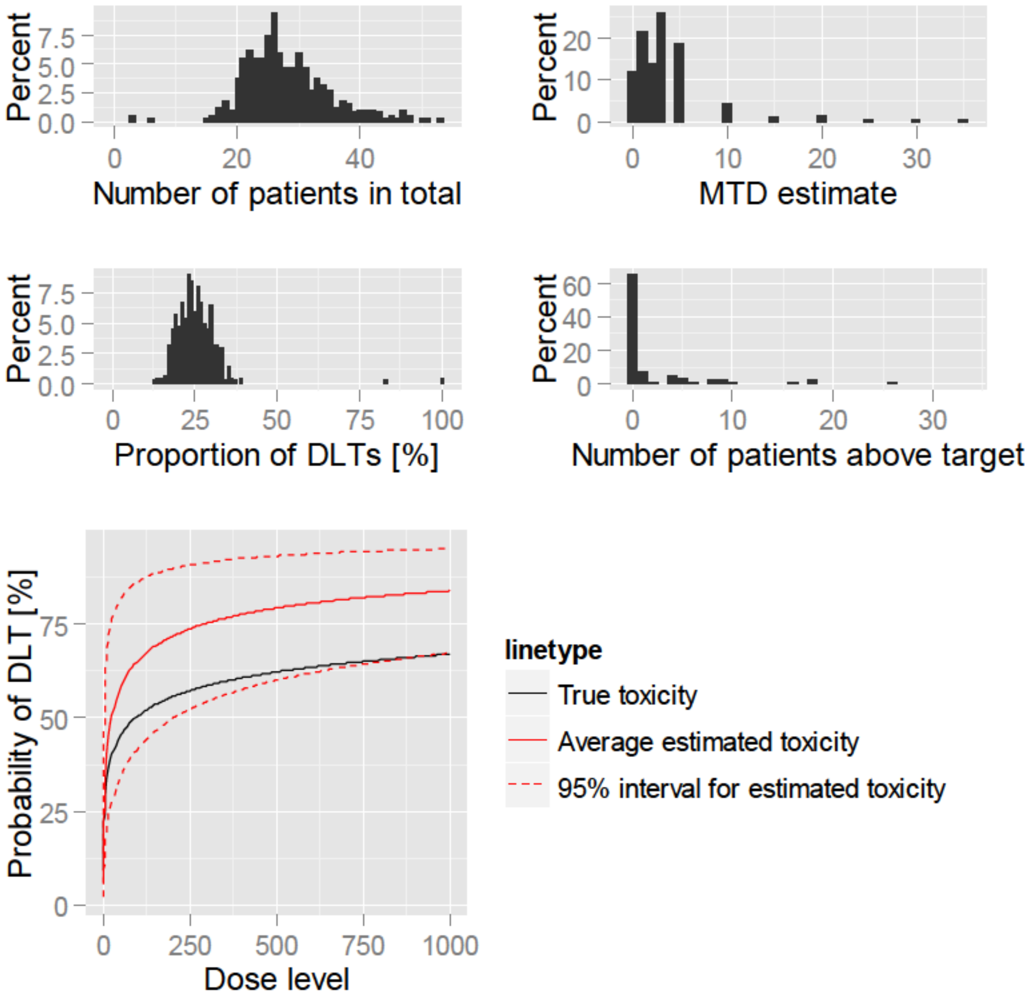

“Use of this document is governed by the terms of use on the first page of this document.”

Figure 4 True versus Average Estimated Toxicity Curve in Scenario 3

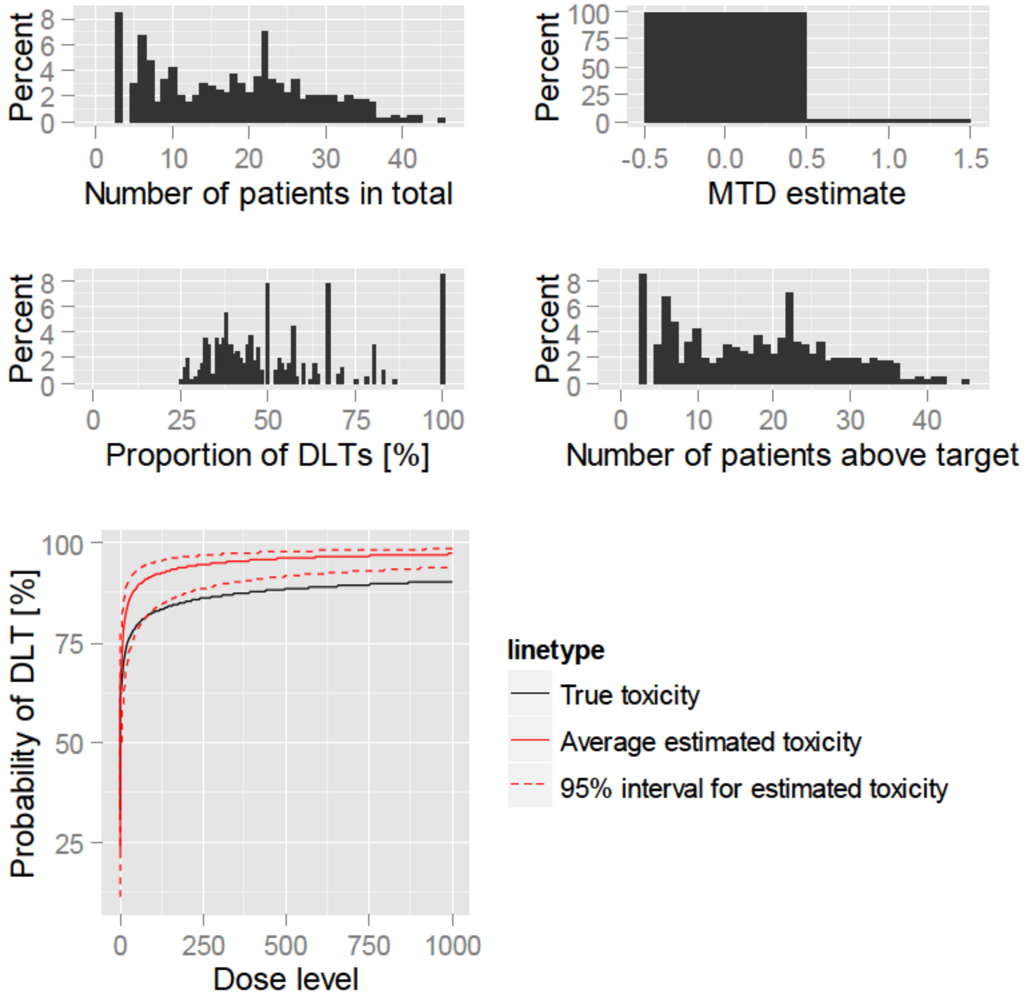

"Use of this document is governed by the terms of use on the first page of this document."

Figure 5 True versus Average Estimated Toxicity Curve in Scenario 4

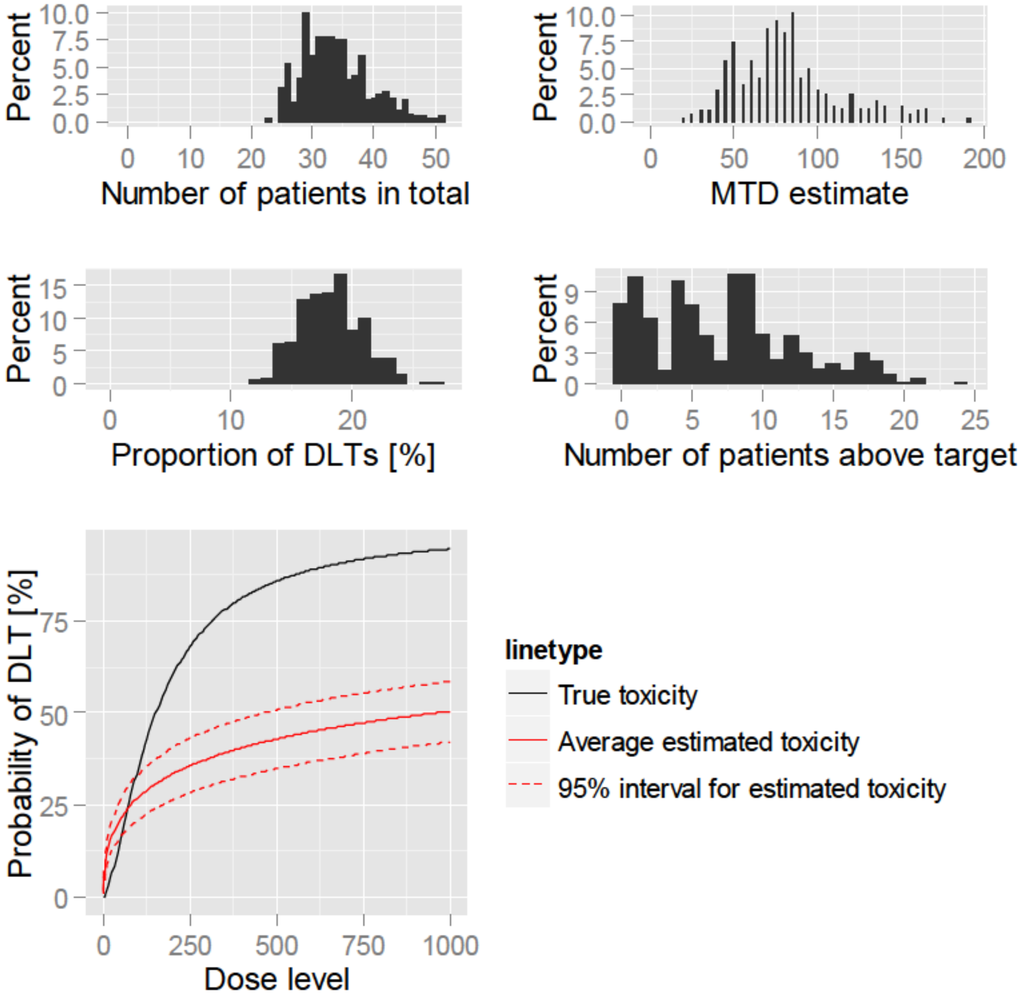

"Use of this document is governed by the terms of use on the first page of this document."

Figure 6 True versus Average Estimated Toxicity Curve in Scenario 5

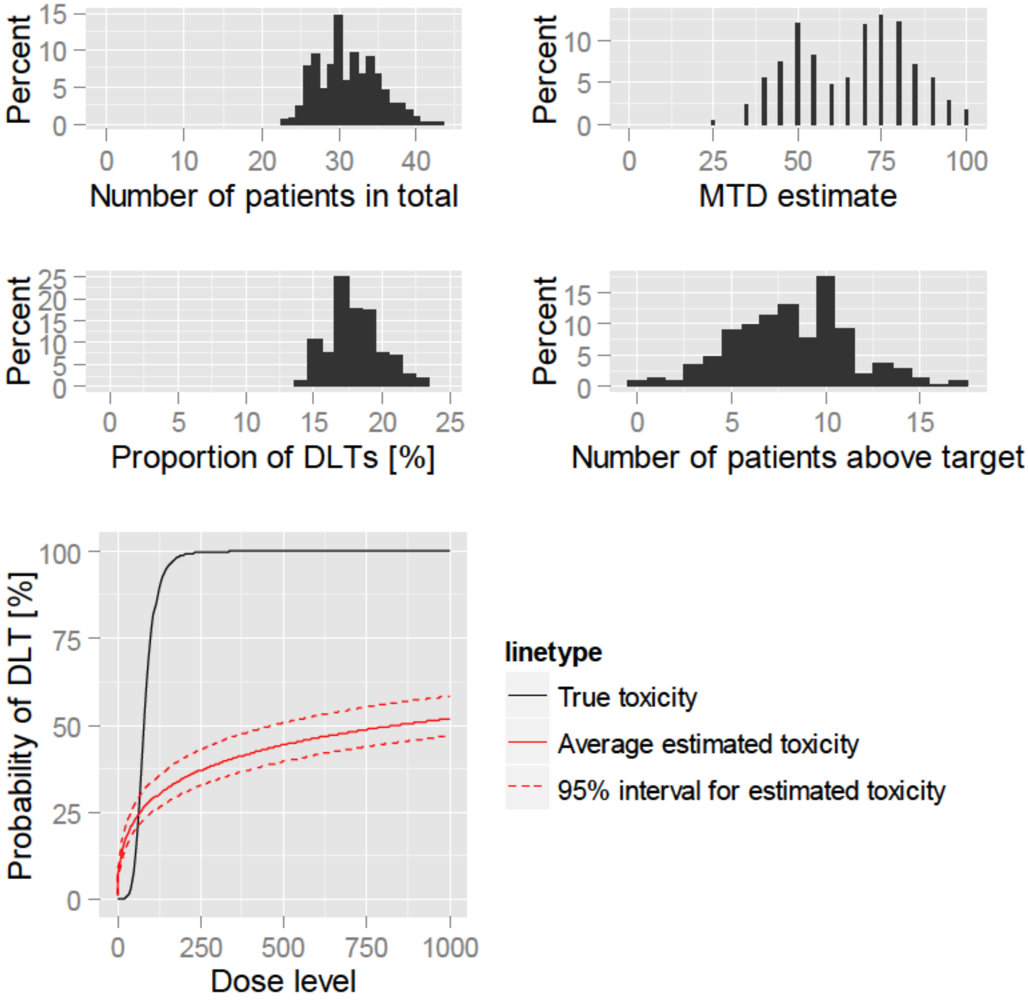

“Use of this document is governed by the terms of use on the first page of this document.”

Figure 7 True versus Average Estimated Toxicity Curve in Scenario 6

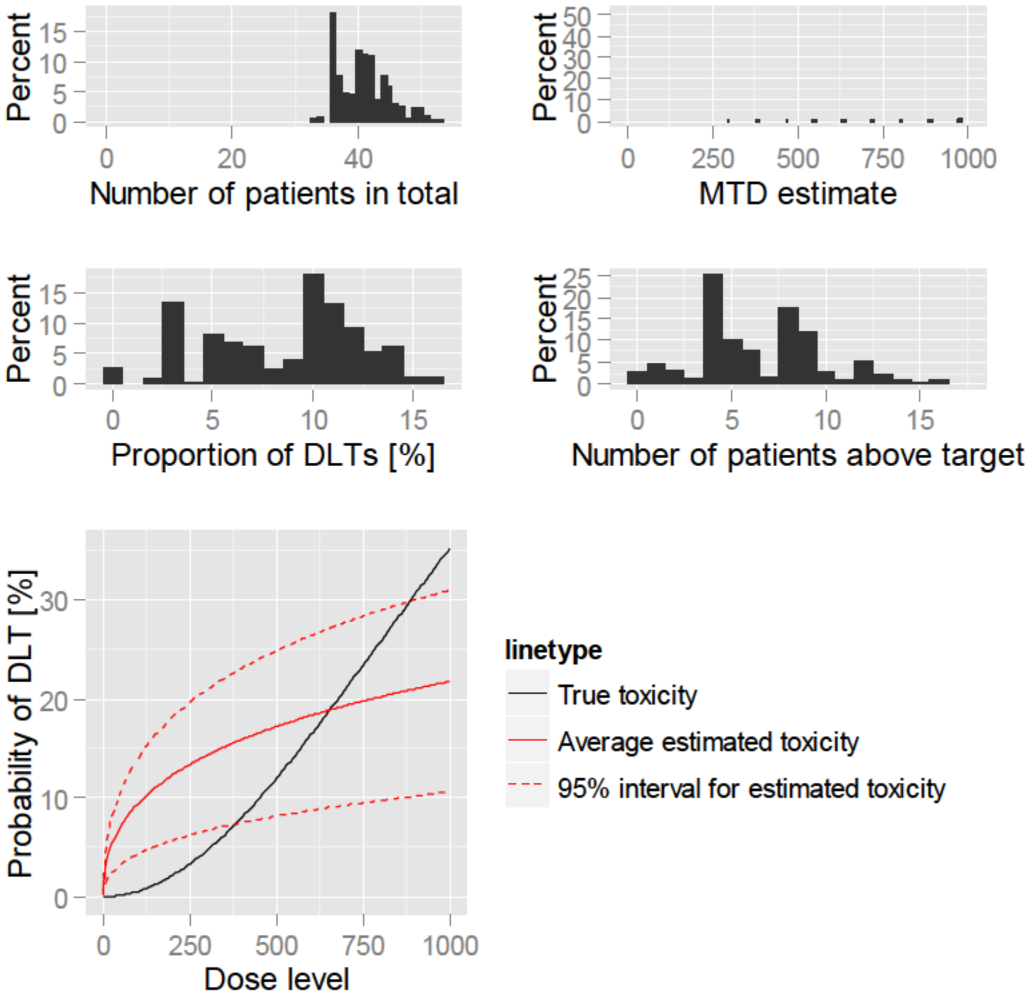

"Use of this document is governed by the terms of use on the first page of this document."

Figure 8 True versus Average Estimated Toxicity Curve in Scenario 7

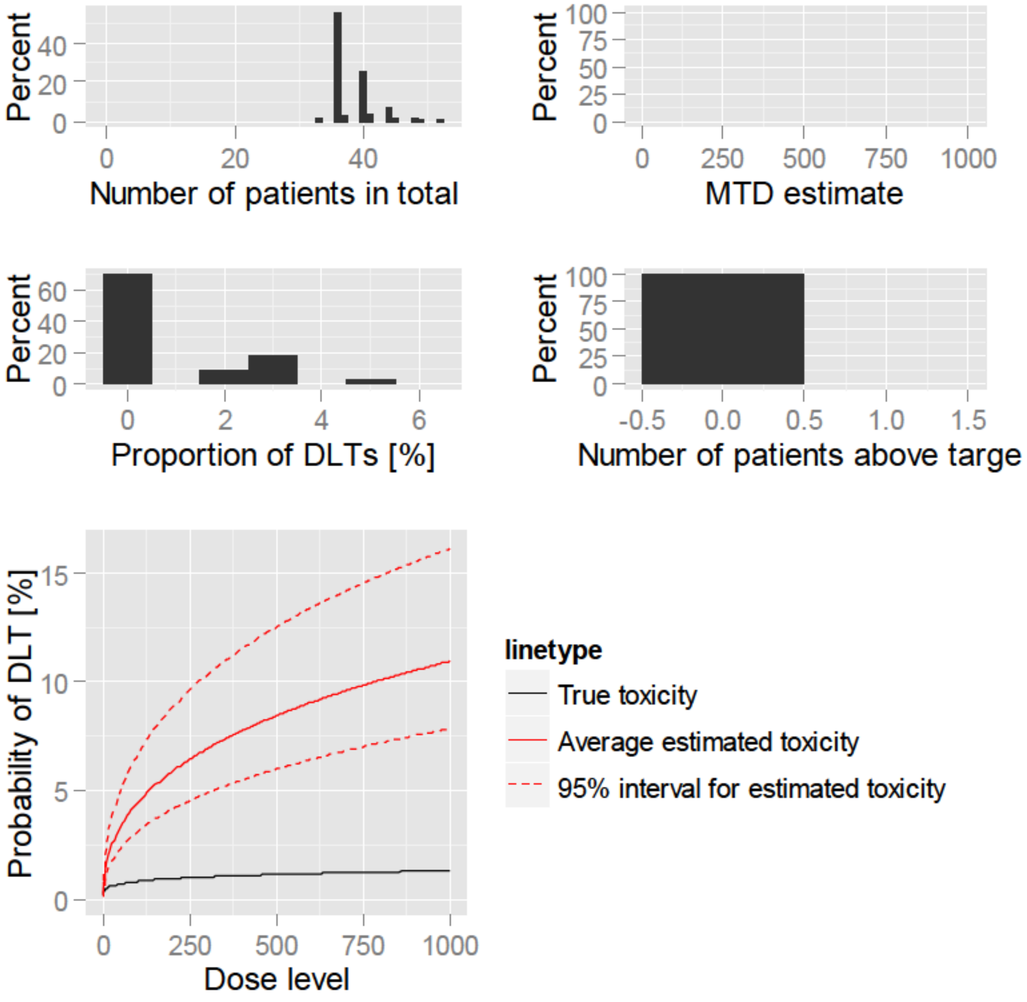

“Use of this document is governed by the terms of use on the first page of this document.”

Figure 9 True versus Average Estimated Toxicity Curve in Scenario 8

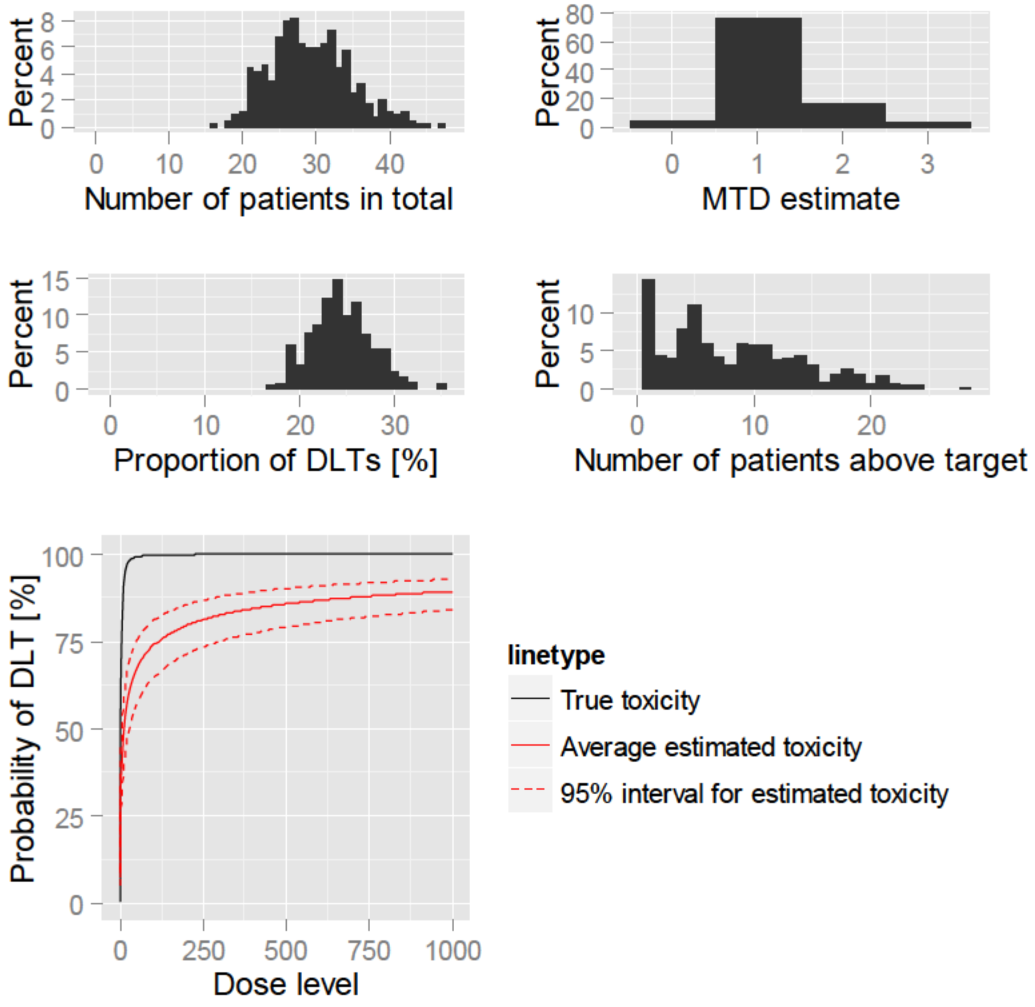

"Use of this document is governed by the terms of use on the first page of this document."

Figure 10 True versus Average Estimated Toxicity Curve in Scenario 9

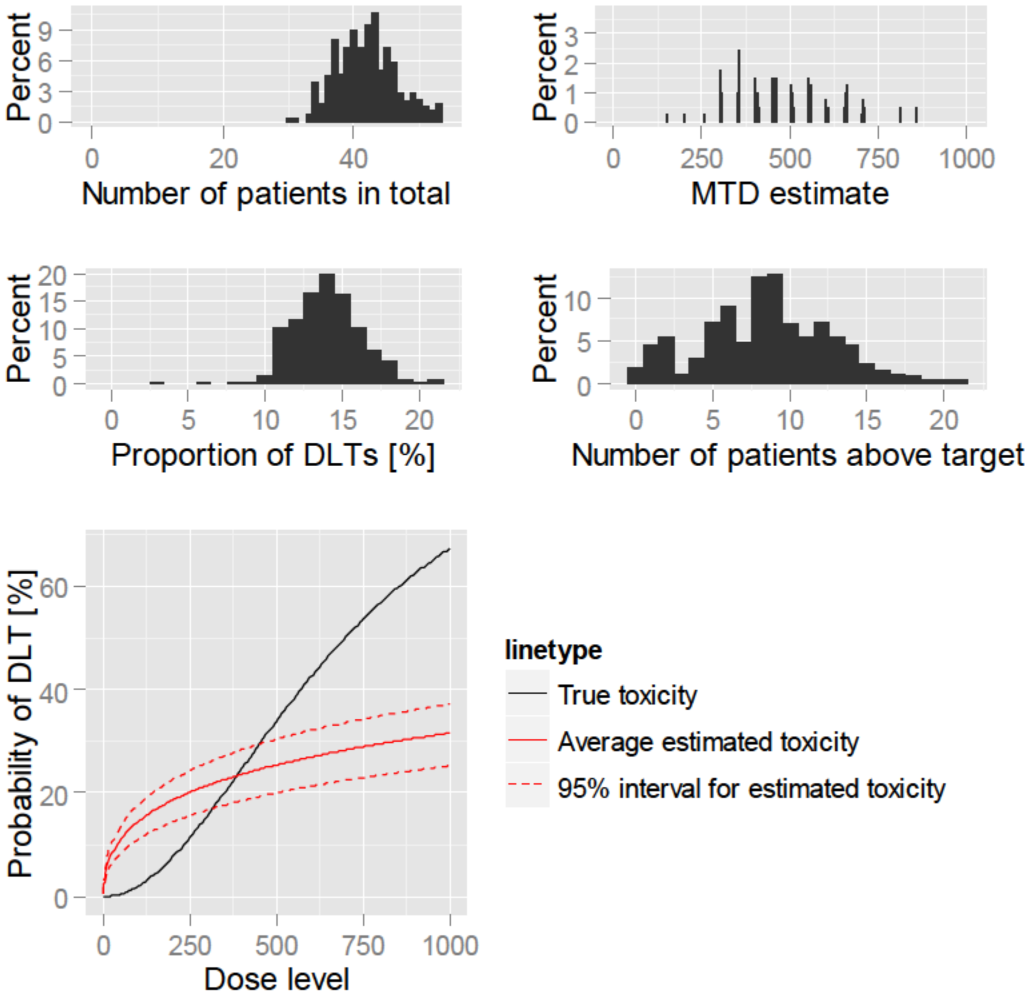

"Use of this document is governed by the terms of use on the first page of this document."

Appendix 7
Intrapatient Dose Escalation Design Simulation Results

The intra-patient dose escalation design for cohort B**1** was tested in three scenarios, which featured low (150 mg), medium (600 mg) and high (1200 mg) late-cycle MTDs, respectively. The corresponding conditional probabilities of experiencing a DLT, given that no DLT appeared in lower dose levels, were defined as in Table 9. Please note that the mathematical definition of the late-cycle MTD is the highest dose level where the cumulative probability of experiencing a DLT at this or lower dose levels does not exceed 33%.

Table 9 Conditional probabilities of DLTs defining the scenarios

Dose:	40	150	300	600	900	1200
“Low”	0.1	0.2	0.3	0.4	0.5	0.5
“Medium”	0.01	0.02	0.04	0.15	0.3	0.4
“High”	0.01	0.01	0.01	0.01	0.04	0.05

The trials were executed as described in Section 6.7.1.2 with 6 patients in total (which is the minimum number of required evaluable patients), and 10,000 trials were simulated for each scenario. The performance of the intra-patient dose escalation design is summarized in the below figures. They comprise the scenario definition (cumulative DLT probabilities), the distribution of the final late-cycle MTD estimates, the number of weeks until it took to define the late cycle MTD, and the number of DLTs which occurred during the trial.

Overall, the design performs acceptably well. The true MTD is indeed the final MTD estimate in most of the simulations. The patients’ safety is protected, as even in the most severe toxicity scenario (low late cycle MTD), not more than 3 patients experience a DLT in more than 80% of all simulations.

“Use of this document is governed by the terms of use on the first page of this document.”

Appendix 7
Intrapatient dose escalation design simulation results (cont.)

Figure 1 Simulation results for the low late-cycle MTD scenario

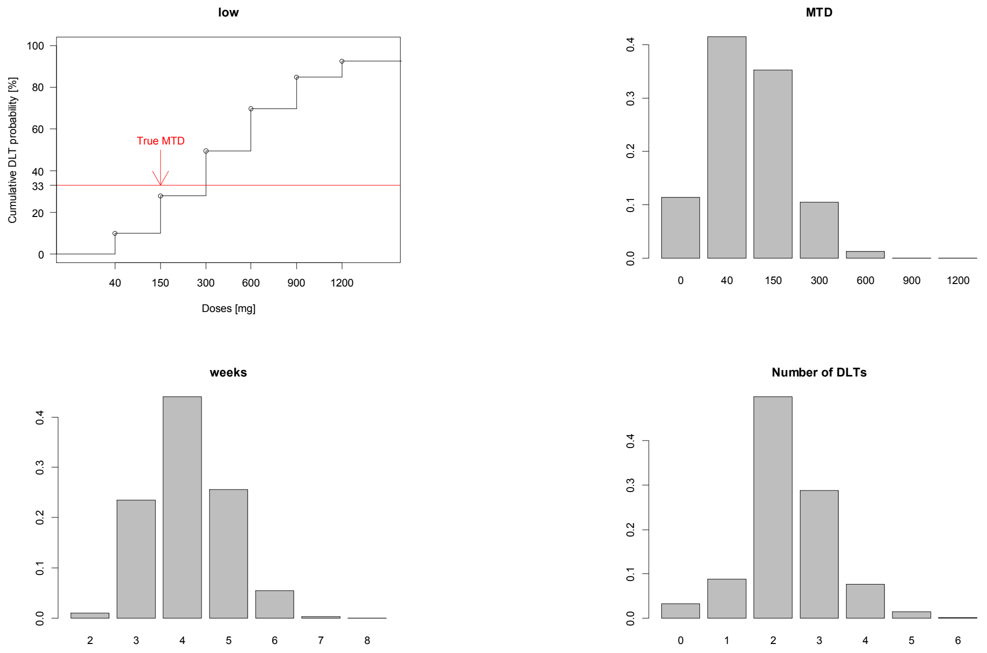

Figure 2 Simulation results for the medium late-cycle MTD scenario

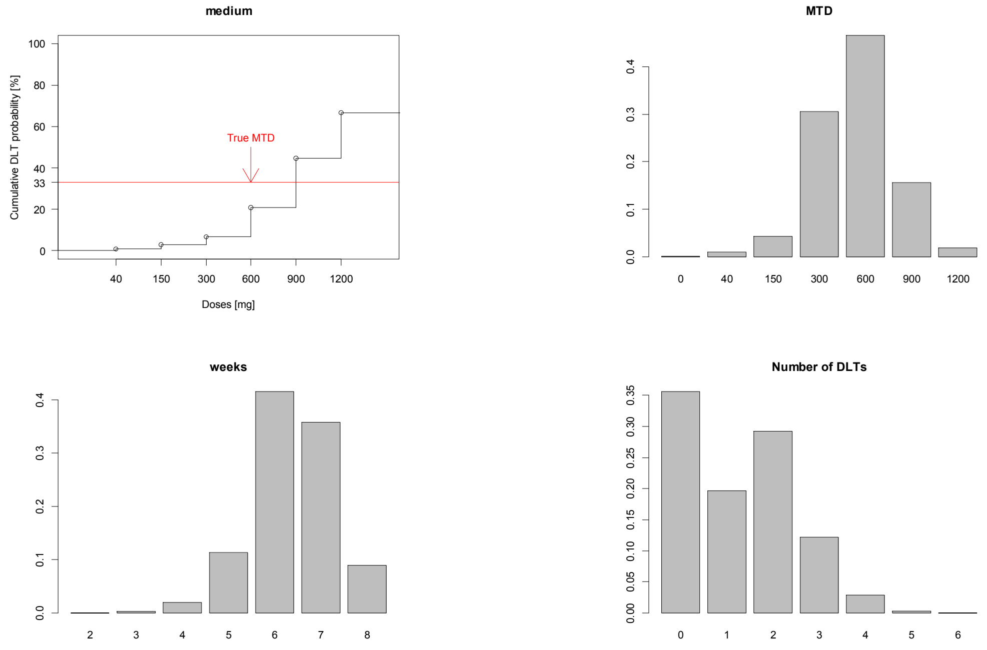

“Use of this document is governed by the terms of use on the first page of this document.”

Appendix 7
Intrapatient dose escalation design simulation results (cont.)

Figure 3 Simulation results for the high late-cycle MTD scenario

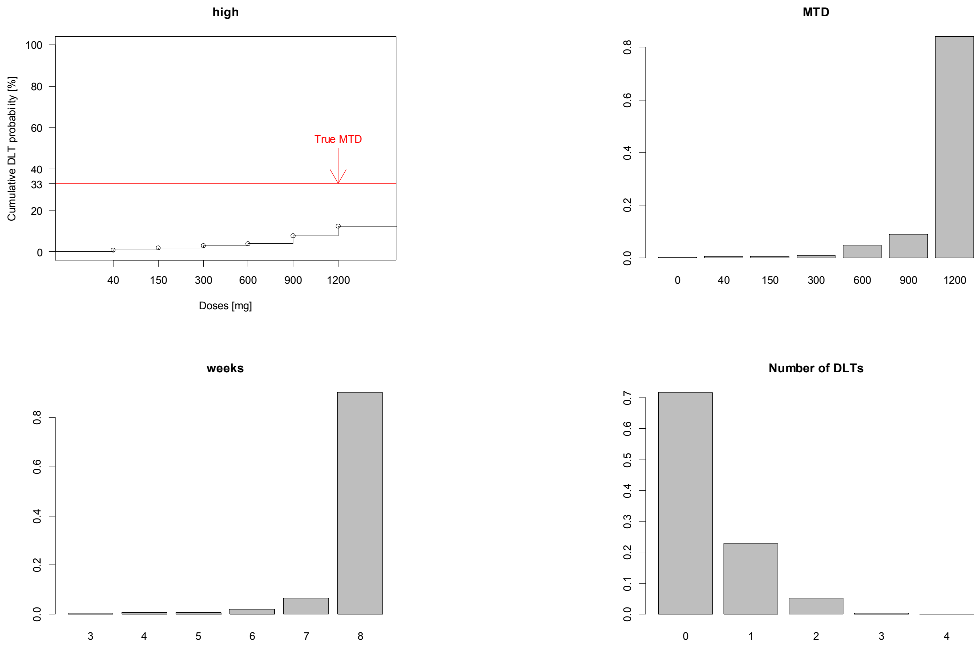

“Use of this document is governed by the terms of use on the first page of this document.”

Appendix 8
Gilbert's Syndrome Definition

Patients with Gilbert's syndrome will be eligible for the study. The diagnosis of Gilbert's syndrome is suspected in people who have persistent, slightly elevated levels of unconjugated bilirubin without any other apparent cause. A diagnosis of Gilbert's syndrome will be based on the exclusion of other diseases based on the following criteria:

- Unconjugated hyperbilirubinemia noted on several occasions
- No evidence of hemolysis (normal hemoglobin, reticulocyte count and LDH)
- Normal liver function tests
- Absence of other diseases associated with unconjugated hyperbilirubinemia.

"Use of this document is governed by the terms of use on the first page of this document."

Appendix 9
[^{18}F]-FDG-PET

Introduction

FDG ([^{18}F]-Fluoro-Deoxy-Glucose) PET imaging will be performed in this study.

The patient needs to fast for typically 4 to 6 hours prior to the FDG-PET scan. The patient can drink water freely during the fast and should be encouraged to do so. He/she may also take regular medications as scheduled during the fast. Blood glucose level will be checked on the day of the FDG PET scan and results assessed prior to the administration of FDG. The patient should have a blood glucose level ≤ 180 mg/dL (≤ 10 mmol/L) in order to have the FDG-PET scan. If the level is higher, the scan should be rescheduled if possible.

Regular diet can be resumed after the scan.

The interval between FDG administration and scanning must be 60 (±10) minutes and it is particularly important that the time interval between injection and start of the scan is the same at follow-up compared to baseline.

Of note PET must be performed before biopsy to avoid potential false positive findings. During the screening period, baseline FDG-PET assessment should be performed at the closest date as possible from start of study treatment. All patients should be encouraged to increase fluid intake for a few hours after the scan to promote excretion of the FDG. A diuretic (furosemide, typically 20-40 mg IV) may be administered at the discretion of the investigator before or during the FDG-PET scan in order to accelerate elimination of the [^{18}F]-FDG from the renal collecting system.

Diazepam may be used to promote muscle relaxation and reduce muscular uptake if tumor deposits are in the neck or shoulder girdle area. Diazepam administration and diuretic administration must be recorded in the Concomitant Medications eCRF page.

Uniform image acquisition and analyses will be implemented across the study centers to minimize methodological variability. The scans will be analyzed by central reading and local assessment and parameters such as Standardized Uptake Value (SUV), Total Lesion Glycolysis (TLG) and Metabolic Tumor Volume (MTV) measured.

"Use of this document is governed by the terms of use on the first page of this document."

Appendix 10
Overall Guidelines for Management of Patients Who Experience Adverse Events

MANAGEMENT GUIDELINES

Guidelines for management of patients who experience specific adverse events during treatment with RO6958688 and/or atezolizumab are provided in Table 1. These guidelines should be followed for both drugs for a given adverse event grade regardless of whether the adverse event is deemed related to atezolizumab, RO6958688, or both.

Guidelines for the management of patients who experience infusion-related reactions and cytokine-release syndrome events related to RO6958688 are provided in Table 2.

"Use of this document is governed by the terms of use on the first page of this document."

Table 1 Guidelines for Management of Patients Who Experience Adverse Events Associated with RO6958688 and/or Atezolizumab

Event	Action to Be Taken
IRRs, CRS, and anaphylaxis	• Follow guidelines for atezolizumab in Appendix 11 for the management of IRRs related to atezolizumab.<br>• Follow guidelines for RO6958688 in Table 2 for the management of IRRs and CRS events related to RO6958688.<br>• For anaphylaxis precautions, see Appendix 14.
Grade 3 or higher treatment-related adverse events within 24 hours of previous infusion	• In the next treatment administration visit, after the end of RO6958688 infusion, patient will remain in the hospital for observation for 8 hours and vital signs will be checked as per Appendix 1.
Grade 3 IRR/CRS at previous infusion	• In the next treatment administration visit after the RO6958688 infusion, patient will remain in the hospital for observation for 24 hours and vital signs will be checked as per Appendix 1.

“Use of this document is governed by the terms of use on the first page of this document.”

Table 1 Guidelines for Management of Patients Who Experience Adverse Events Associated with RO6958688 and/or Atezolizumab (cont.)

Pulmonary events, excluding atezolizumab-related pneumonitis [a, b]	
Grade 1 or 2	• For Grade 1 events, continue atezolizumab and RO6958688.<br>• For Grade 2 events, withhold atezolizumab for up to 12 weeks after event onset and RO6958688 for up to 6 weeks after last administered dose.<br>• Monitor patient closely.<br>• If event resolves to Grade 1 or better, resume atezolizumab and RO6958688.<br>• For recurrent events, treat as a Grade 3 or 4 event.
Grade 3	• Permanently discontinue atezolizumab.<br>• Withhold RO6958688 for up to 6 weeks after last administered dose.<br>• Initiate treatment with corticosteroids equivalent to 2 mg/kg/day of IV or oral methylprednisone until resolution to Grade 1 or better. [c]<br>• If event does not improve within 48 hours after initiating corticosteroids, consider adding an immunosuppressive agent.<br>• If event resolves to Grade 1 or better, resume RO6958688.
Grade 4 [d]	• Permanently discontinue atezolizumab and RO6958688.<br>• Initiate treatment with corticosteroids equivalent to 2 mg/kg/day of IV or oral methylprednisone until resolution to Grade 1 or better. [c]<br>• If event does not improve within 48 hours after initiating corticosteroids, consider adding an immunosuppressive agent.

CRS = cytokine-release syndrome; IRR = infusion-related reaction.

[a] For hypoxia that occurs in the context of a RO6958688–treatment-related CRS event, please follow the IRR/CRS management guidelines in Table 2.

[b] Provide supportive care (e.g., oxygen support and intubation), as clinically indicated.

[c] If corticosteroids have been used for longer than 3 weeks, they must be tapered after event resolves to Grade 1 or better.

[d] Ensure patient has access to an intensive care unit.

"Use of this document is governed by the terms of use on the first page of this document."

Table 1 Guidelines for Management of Patients Who Experience Adverse Events Associated with RO6958688 and/or Atezolizumab (cont.)

Event	Action to Be Taken
Atezolizumab-related pneumonitis	
Grade 1	• Follow guidelines for atezolizumab in Appendix 11.<br>• Continue RO6958688.
Grades ≥ 2	• Follow guidelines for atezolizumab in Appendix 11.<br>• Withhold RO6958688 for up to 6 weeks after last administered dose.<br>• If event resolves to Grade 1 or better, resume RO6958688.
Elevations in ALT and AST [a]	
AST/ALT $>$ ULN to $\leq 3 \times$ ULN	• Continue atezolizumab and RO6958688.<br>• Monitor LFTs weekly.
AST/ALT $> 3 \times$ ULN to $< 5 \times$ ULN	• Withhold atezolizumab for up to 12 weeks after event onset. [b]<br>• Withhold RO6958688 for up to 6 weeks after last administered dose.<br>• Monitor LFTs at least weekly until results return to normal or baseline.<br>• Consider patient referral to hepatologist.<br>• If event does not improve to AST/ALT $\leq 3 \times$ ULN within 48 hours, initiate treatment with corticosteroids equivalent to 1 mg/kg/day of IV or oral methylprednisolone. [c]<br>• If event does not improve to AST/ALT $\leq 3 \times$ ULN within 48 hours of treatment with corticosteroids equivalent to 1 mg/kg/day of corticosteroids, increase dose to 2 mg/kg/day IV or oral methylprednisolone (or equivalent). [c]<br>• If event resolves to $< 3 \times$ ULN, resume atezolizumab and RO6958688.

LFT = liver function test; ULN = upper limit of normal.

[a] For LFT elevations that occur in the context of a RO6958688–treatment-related CRS event, please follow the IRR/CRS management guidelines in Table 2.

[b] Atezolizumab may be withheld for a longer period of time (i.e., > 12 weeks after event onset) to allow for corticosteroids (if initiated) to be reduced to ≤ 10 mg/day oral prednisone or equivalent. The acceptable length of the extended period of time must be agreed upon by the investigator and the Medical Monitor.

[c] If corticosteroids have been used for longer than 3 weeks, they must be tapered before treatment can be resumed.

“Use of this document is governed by the terms of use on the first page of this document.”

Table 1 Guidelines for Management of Patients Who Experience Adverse Events Associated with RO6958688 and/or Atezolizumab (cont.)

Event	Action to Be Taken
Elevations in ALT and AST (cont.) [a]	
AST/ALT $>5\times$ ULN to $<10\times$ ULN	• If event is considered related to atezolizumab, permanently discontinue atezolizumab.<br>• Withhold RO6958688 for up to 6 weeks after last administered dose.<br>• Monitor LFTs at least weekly until results return to normal or baseline.<br>• Consider patient referral to hepatologist.<br>• Initiate treatment with corticosteroids equivalent to 2 mg/kg/day of IV or oral methylprednisolone until resolution to AST/ALT $\leq 3\times$ ULN. [c]<br>• If event is considered related to atezolizumab and does not improve within 48 hours of corticosteroid treatment, consider adding an immunosuppressive agent.<br>• If event resolves to $<3\times$ ULN, resume RO6958688.
AST/ALT $\geq 10\times$ ULN	• Permanently discontinue atezolizumab and RO6958688.<br>• Monitor LFTs every other day until decreased to $<5\times$ ULN and then monitor at least weekly until results return to normal or baseline.<br>• Refer patient to hepatologist and consider liver biopsy to assess hepatic injury.<br>• Initiate treatment with corticosteroids equivalent to 2 mg/kg/day IV or oral methylprednisolone until improvement to AST/ALT $\leq 3\times$ ULN. [c]<br>• If event is considered related to atezolizumab and does not improve within 48 hours of corticosteroid treatment, consider adding an immunosuppressive agent.

LFT = liver function test; ULN = upper limit of normal.

[a] For LFT elevations that occur in the context of a RO6958688–treatment-related CRS event, please follow the IRR/CRS management guidelines.

[b] Atezolizumab may be withheld for a longer period of time (i.e., > 12 weeks after event onset) to allow for corticosteroids (if initiated) to be reduced to ≤ 10 mg/day oral prednisone or equivalent. The acceptable length of the extended period of time must be agreed upon by the investigator and the Medical Monitor.

[c] If corticosteroids have been used for longer than 3 weeks, they must be tapered before treatment can be resumed.

"Use of this document is governed by the terms of use on the first page of this document."

Table 1 Guidelines for Management of Patients Who Experience Adverse Events Associated with RO6958688 and/or Atezolizumab (cont.)

Event	Action to Be Taken
Gastrointestinal events [a, b]	
Diarrhea, enteritis or colitis, Grade 1	• Continue atezolizumab and RO6958688.<br>• Initiate standard-of-care symptomatic treatment with anti-diarrheal agents (e.g., loperamide). [c]<br>• If diarrhea does not improve within 48 hours of anti-diarrheal agent treatment, initiate treatment with corticosteroids equivalent to 1 mg/kg/day IV or oral methylprednisolone. [e]<br>• If diarrhea does not improve within 48 hours of corticosteroid treatment, increase dose to 2 mg/kg/day IV or oral methylprednisolone (or equivalent). [e]<br>• Consider rectoscopy/colonoscopy with biopsy if symptoms of diarrhea persist for > 48 hours.

GI = gastrointestinal.

[a] For diarrhea that that occurs in the context of a RO6958688–treatment-related CRS event, please follow the IRR/CRS management guidelines in Table 2.

[b] Exclude infectious and other etiologies of diarrhea, enteritis, and colitis before following these management guidelines.

[c] Loperamide treatment should begin at an initial dose of 4 mg. After each loose stool, the dose may be increased by 4 mg to a maximum dose of 16 mg/day.

[d] Atezolizumab may be withheld for a longer period of time (i.e., > 12 weeks after event onset) to allow for corticosteroids (if initiated) to be reduced to ≤ 10 mg/day oral prednisone or equivalent. The acceptable length of the extended period of time must be agreed upon by the investigator and the Medical Monitor.

[e] If corticosteroids have been used for longer than 3 weeks, they must be tapered before treatment can be resumed.

[f] Resumption of atezolizumab may be considered in patients who are deriving clinical benefit and have fully recovered from the *immune-mediated* event. Patients can be re-challenged with atezolizumab only after approval has been documented by both the investigator (or an appropriate delegate) and the Medical Monitor.

“Use of this document is governed by the terms of use on the first page of this document.”

Table 1 Guidelines for Management of Patients Who Experience Adverse Events Associated with RO6958688 and/or Atezolizumab (cont.)

Event	Action to Be Taken
Gastrointestinal events (cont.) [a, b]	
Diarrhea, enteritis or colitis, Grade 2	• Withhold atezolizumab for up to 12 weeks after event onset. [d]<br>• Withhold RO6958688 for up to 6 weeks after last administered dose.<br>• Initiate standard-of-care symptomatic treatment with anti-diarrheal agents (e.g., loperamide). [c]<br>• If diarrhea does not improve within 48 hours of anti-diarrheal agent treatment, initiate treatment with corticosteroids equivalent to 1 mg/kg/day IV or oral methylprednisolone. [e]<br>• If diarrhea does not improve within 48 hours of corticosteroid treatment, increase dose to 2 mg/kg/day IV or oral methylprednisolone (or equivalent). [e]<br>• If symptoms of diarrhea persist for > 48 hours, consider rectoscopy/colonoscopy with biopsy.<br>• Patient referral to GI specialist is recommended.<br>• If event resolves to Grade 1 or better, resume atezolizumab and RO6958688. [f]<br>• If event does not resolve to Grade 1 or better while withholding atezolizumab and RO6958688, permanently discontinue atezolizumab and RO6958688 and contact Medical Monitor. [f]

GI = gastrointestinal.

[a] For diarrhea that that occurs in the context of a RO6958688–treatment-related CRS event, please follow the IRR/CRS management guidelines.

[b] Exclude infectious and other etiologies of diarrhea, enteritis, and colitis before following these management guidelines.

[c] Loperamide treatment should begin at an initial dose of 4 mg. After each loose stool, the dose may be increased by 4 mg to a maximum dose of 16 mg/day.

[d] Atezolizumab may be withheld for a longer period of time (i.e., > 12 weeks after event onset) to allow for corticosteroids (if initiated) to be reduced to ≤ 10 mg/day oral prednisone or equivalent. The acceptable length of the extended period of time must be agreed upon by the investigator and the Medical Monitor.

[e] If corticosteroids have been initiated, they must be tapered before treatment can be resumed.

[f] Resumption of atezolizumab may be considered in patients who are deriving clinical benefit and have fully recovered from the *immune-mediated* event. Patients can be re-challenged with atezolizumab only after approval has been documented by both the investigator (or an appropriate delegate) and the Medical Monitor.

“Use of this document is governed by the terms of use on the first page of this document.”

Table 1 Guidelines for Management of Patients Who Experience Adverse Events Associated with RO6958688 and/or Atezolizumab (cont.)

Event	Action to Be Taken
Gastrointestinal events (cont.) [a, b]	
Diarrhea, enteritis or colitis, Grade 3	• Withhold atezolizumab for up to 12 weeks after event onset. [d]<br>• Withhold RO6958688 for up to 6 weeks after last administered dose.<br>• Refer patient to GI specialist for evaluation and confirmatory biopsy.<br>• Initiate treatment with corticosteroids equivalent to 2-mg/kg/day IV or oral methylprednisolone. [e]<br>• If event resolves to Grade 1 or better, resume atezolizumab and RO6958688. [f]<br>• If event does not resolve to Grade 1 or better within 24 hours, manage as a Grade 4 event.
Diarrhea, enteritis or colitis, Grade 4	• Permanently discontinue atezolizumab and RO6958688 and contact Medical Monitor.<br>• Refer patient to GI specialist for evaluation and confirmatory biopsy.<br>• Initiate treatment with corticosteroids equivalent to 2-mg/kg/day IV or oral methylprednisolone. [e]<br>• If event does not improve within 48 hours after initiating corticosteroids, consider adding an immunosuppressive agent (after rectoscopy/colonoscopy).

GI = gastrointestinal.

[a] For diarrhea that that occurs in the context of a RO6958688–treatment-related CRS event, please follow the IRR/CRS management guidelines.

[b] Exclude infectious and other etiologies of diarrhea, enteritis, and colitis before following these management guidelines.

[c] Loperamide treatment should begin at an initial dose of 4 mg. After each loose stool, the dose may be increased by 4 mg to a maximum dose of 16 mg/day.

[d] Atezolizumab may be withheld for a longer period of time (i.e., > 12 weeks after event onset) to allow for corticosteroids (if initiated) to be reduced to ≤ 10 mg/day oral prednisone or equivalent. The acceptable length of the extended period of time must be agreed upon by the investigator and the Medical Monitor.

[e] If corticosteroids have been used for longer than 3 weeks, they must be tapered before treatment can be resumed.

[f] Resumption of atezolizumab may be considered in patients who are deriving clinical benefit and have fully recovered from the *immune-mediated* event. Patients can be re-challenged with atezolizumab only after approval has been documented by both the investigator (or an appropriate delegate) and the Medical Monitor.

“Use of this document is governed by the terms of use on the first page of this document.”

Table 1 Guidelines for Management of Patients Who Experience Adverse Events Associated with RO6958688 and/or Atezolizumab (cont.)

Event	Action to Be Taken
For Atezolizumab-related toxicity not described above	
Grade 1 or 2	• Follow guidelines for atezolizumab in Appendix 11.<br>• Continue RO6958688.
Grade 3 or 4	• Follow guidelines for atezolizumab in Appendix 11.<br>• Withhold RO6958688.<br>• If event resolves to Grade 2 or better within 6 weeks after last administered dose, resume RO6958688. If not, contact Medical Monitor to determine whether RO6958688 can be resumed.

"Use of this document is governed by the terms of use on the first page of this document."

GUIDELINES FOR MANAGEMENT OF INFUSION-RELATED REACTIONS AND CYTOKINE-RELEASE SYNDROME RELATED TO RO6958688

Guidelines for the management of patients who experience infusion-related reactions and cytokine-release syndrome events related to RO6958688 are provided below in Table 2.

Please note that infusion-related reactions (i.e., infusion-related events associated with the first and second RO6958688 infusion without hypotension or hypoxia as predominant symptoms) and cytokine-release syndrome events (i.e., infusion-related events occurring within 24 hours of the RO6958688 infusion with hypotension or hypoxia as the predominant symptom, and all infusion-related events associated with the third infusion of RO6958688 or later, irrespective of associated symptoms) should both be managed using the following guidance.

Infusion-related reactions (regardless of grade) without hypotension or hypoxia should be managed according to the Grade 1 cytokine-release syndrome guidelines below.

In patients with lung metastasis, hypoxia could also be secondary to tumor inflammation after the first and second dose of RO6958688. In such situations, please consider further evaluation with radiological imaging per clinical judgment, and if there is no associated hypotension, please follow guidelines in Table 2.

“Use of this document is governed by the terms of use on the first page of this document.”

Table 2 Guidelines for Management of Infusion-Related Reactions and Cytokine-Release Syndrome Related to RO6958688

Event	Action to Be Taken
CRS	
Grade 1<br>defined as fever, constitutional symptoms (first and second dose IRRs with no hypotension/hypoxia [see Section 5.2.6.2.4])	Immediate actions:<br>• If RO6958688 infusion is still ongoing, slow the infusion rate up to 50% or interrupt the infusion.<br>• Treat with antihistamines, antipyretics, and/or analgesics as clinically indicated.<br>• Monitor fluid balance and administer IV fluids as clinically indicated.<br>• If fluids are administered, manage as a Grade 2 event.<br>Restarting infusion:<br>• If RO6958688 infusion was interrupted, wait until 30 minutes after the event has resolved before restarting the infusion at 50% of the original infusion rate.<br>Next cycle:<br>• Pretreat with antihistamines, antipyretics/NSAIDS, and antiemetic medication. [a]

CRS = cytokine-release syndrome; CTCAE = Common Terminology Criteria for Adverse Events; IRR = infusion-related reaction; NCI = National Cancer Institute; NSAIDS = non-steroidal anti-inflammatory drugs.

Notes: Refer to NCI CTCAE v5 for a complete description of grading of symptoms.

[a] Guidelines for corticosteroid premedication refer only to the first cycle following the event. Premedication is not required for subsequent cycles if no event has occurred in the previous cycle. Please follow premedication guidance in Section 4.3.2.5 in addition to these guidelines.

“Use of this document is governed by the terms of use on the first page of this document.”

Table 2 Guidelines for Management of Infusion-Related Reactions and Cytokine-Release Syndrome Related to RO6958688 (cont.)

Event	Action to Be Taken
CRS (cont.)	
Grade 2<br>defined as hypotension (responding to fluids) OR hypoxia (requiring <40% FiO_2 to maintain adequate hemoglobin oxygen saturation) [a]	Immediate actions:<br>• If RO6958688 infusion is ongoing, stop the infusion immediately.<br>• Treat with antihistamines, antipyretics, and/or analgesics as clinically indicated.<br>• Consider treatment with IV corticosteroids (methylprednisolone 2 mg/kg/day or, if neurologic symptoms are present, dexamethasone 10 mg).<br>• Consider administering a single dose of tocilizumab IV 8 mg/kg (if administered, see Appendix 12).<br>• Monitor cardiac and other organ functions closely.<br>• Provide hemodynamic support as clinically indicated.<br>• Provide oxygen for hypoxia and fluids for hypotension.<br>• Collect all samples (including a sample for the cytokine panel) as described in Appendix 1, Table A3.<br>• Admit to ICU if clinically indicated and consider 24-hour hospitalization.<br>• If there is no improvement of hypotension or hypoxia within 24 hours, manage as a Grade 3 event.<br>• Notify the Medical Monitor.<br>Restarting infusion:<br>• Wait until 30 minutes after the event has resolved before restarting the infusion at up to 25% of the original infusion rate.<br>• If hypotension or hypoxia recurs, stop infusion immediately. RO6958688 should not be re-administered (re-started) again during this cycle.<br>• If hypotension or hypoxia recurs, manage as a Grade 3 event.

CRS = cytokine-release syndrome; CTCAE = Common Terminology Criteria for Adverse Events; FiO_2 = fraction of inspired oxygen; ICU = intensive care unit; IRR = infusion-related reaction; NCI = National Cancer Institute; NSAIDS = non-steroidal anti-inflammatory drugs.

Notes: Refer to NCI CTCAE v5 for a complete description of grading of symptoms.

[a] Guidelines for corticosteroid premedication refer only to the first cycle following the event. Premedication is not required for subsequent cycles if no event has occurred in the previous cycle. Please follow premedication guidance in Section 4.3.2.5 in addition to these guidelines.

"Use of this document is governed by the terms of use on the first page of this document."

Table 2 Guidelines for Management of Infusion-Related Reactions and Cytokine-Release Syndrome Related to RO6958688 (cont.)

Event	Action to Be Taken
CRS (cont.)	
Grade 2 (cont.)<br>defined as hypotension (responding to fluids) OR hypoxia (requiring <40% FiO_2 to maintain adequate hemoglobin oxygen saturation) [a]	Next cycle:<br>• Patient may receive RO6958688 if symptoms resolve to Grade ≤ 1 and with approval of the Medical Monitor, as follows:<br>– Pretreat with antihistamines, antipyretics and/or analgesics as clinically indicated. [a]<br>– Pretreat with IV corticosteroids (methylprednisolone 80 mg or dexamethasone 16 mg) at least 60 minutes prior to the administration of RO6958688. [a]<br>– Administer RO6958688 at 50% of the initial infusion rate of the previous cycle.<br>Subsequent cycles:<br>• If there is an occurrence of IRR or CRS Grade ≥ 3 in any of the subsequent cycles, permanently discontinue RO6958688 regardless of recovery (see Grade 3 management guidelines).<br>• If there is an occurrence of a Grade ≤ 2 CRS in subsequent cycles, manage as indicated by severity (see Grade 1 or Grade 2 management guidelines).

CRS = cytokine-release syndrome; CTCAE = Common Terminology Criteria for Adverse Events; FiO_2 = fraction of inspired oxygen; ICU = intensive care unit; IRR = infusion-related reaction; NCI = National Cancer Institute; NSAIDS = non-steroidal anti-inflammatory drugs.

Notes: Refer to NCI CTCAE v5 for a complete description of grading of symptoms.

[a] Guidelines for corticosteroid premedication refer only to the first cycle following the event. Premedication is not required for subsequent cycles if no event has occurred in the previous cycle. Please follow premedication guidance in Section 4.3.2.5 in addition to these guidelines.

“Use of this document is governed by the terms of use on the first page of this document.”

Table 2 Guidelines for Management of Infusion-Related Reactions and Cytokine-Release Syndrome Related to RO6958688 (cont.)

Event	Action to Be Taken
CRS (cont.)	
Grade 3<br>defined as hypotension (requiring one vasopressor [see Appendix 13 for definitions and calculations])<br>OR hypoxia (requiring ≥40% FiO_2 to maintain adequate hemoglobin oxygen saturation)	Immediate actions:<br>• If RO6958688 infusion is ongoing, stop the infusion immediately.<br>• Treat with antihistamines, antipyretics, and/or analgesics as clinically indicated.<br>• Treat with IV corticosteroids (methylprednisolone 2 mg/kg/day or, if neurologic symptoms are present, dexamethasone 10 mg).<br>• Administer tocilizumab IV 8 mg/kg (see Appendix 12).<br>If there is no improvement after 24 hours, repeat tocilizumab administration (see Appendix 12).<br>• Closely monitor and maintain fluid balance and administer IV fluids as clinically indicated.<br>• Provide oxygen for hypoxia.<br>• Provide vasopressor support for hypotension with high and repeated doses if required (see Appendix 13).<br>• Hospitalize patient for 24 hours.<br>• Perform a work up for organ functions (e.g., liver, cardiac) based on clinical assessment of the investigator.<br>• Cardiopulmonary and organ function monitoring in ICU is recommended.<br>• Collect all samples (including a sample for the cytokine panel) as described in Appendix 1, Table A3.<br>• Notify the Medical Monitor.<br>Restarting Infusion:<br>• RO6958688 should not be administered again during this cycle.

CRS = cytokine-release syndrome; CTCAE = Common Terminology Criteria for Adverse Events; FiO_2 = fraction of inspired oxygen; ICU = intensive care unit; IRR = infusion-related reaction; NCI = National Cancer Institute; NSAIDS = non-steroidal anti-inflammatory drugs.

Notes: Refer to NCI CTCAE v5 for a complete description of grading of symptoms.

[a] Guidelines for corticosteroid premedication refer only to the first cycle following the event. Premedication is not required for subsequent cycles if no event has occurred in the previous cycle. Please follow premedication guidance in Section 4.3.2.5 in addition to these guidelines.

"Use of this document is governed by the terms of use on the first page of this document."

Appendix 10
Overall Guidelines for Management of Patients Who Experience Adverse Events (cont.)

Table 2 Guidelines for Management of Infusion-Related Reactions and Cytokine-Release Syndrome Related to RO6958688 (cont.)

Event	Action to Be Taken
CRS (cont.)	
Grade 3 (cont.)<br>defined as hypotension (managed with one vasopressor [see Appendix 13 for definitions and calculations])<br>OR hypoxia (requiring ≥40% FiO_2 to maintain adequate hemoglobin oxygen saturation)	Next Cycle/administration visit:<br>• If the patient had a Grade ≥2 IRR or CRS in any previous cycle, permanently discontinue RO6958688.<br>• If patient does not recover (is febrile or still on vasopressors) within 8 hours after corticosteroid and tocilizumab treatment, permanently discontinue RO6958688.<br>• If there is no improvement after 24 hours, repeat tocilizumab administration.<br>• If patient recovers (is afebrile and off vasopressors) within 8 hours following corticosteroid and tocilizumab treatment, RO6958688 can be administered in next cycle, as follows:<br>– Pretreat with antihistamines, antipyretics and/or analgesics as clinically indicated. [a]<br>– Pretreat with IV corticosteroids (methylprednisolone 80 mg or dexamethasone 16 mg) at least 60 minutes prior to the administration of RO6958688. [a]<br>– Hospitalize patient for 24 hours.<br>– Collect all samples (including a sample for the cytokine panel) as described in Appendix 1, Table A3.<br>• Administer RO6958688 at 50% of the initial infusion rate of the previous cycle.<br>• Hospitalize the patient for 24-hour observation<br>• Subsequent visit/cycles: If a Grade ≥3 CRS recurs, permanently discontinue RO6958688.<br>• If there is an occurrence of a Grade ≤2 CRS in subsequent cycles, manage as indicated by severity (i.e., Grade 1 or Grade 2 management guidelines).
Grade 4<br>defined as life threatening consequences; urgent intervention indicated (multiple vasopressors)	• Permanently discontinue RO6958688.<br>• Manage as a Grade 3 event.

CRS = cytokine-release syndrome; CTCAE = Common Terminology Criteria for Adverse Events; FiO_2 = fraction of inspired oxygen; ICU = intensive care unit; IRR = infusion-related reaction; NCI = National Cancer Institute; NSAIDS = non-steroidal anti-inflammatory drugs.

Notes: Refer to NCI CTCAE v5 for a complete description of grading of symptoms.

[a] Guidelines for corticosteroid premedication refer only to the first cycle following the event. Premedication is not required for subsequent cycles if no event has occurred in the previous cycle. Please follow premedication guidance in Section 4.3.2.5 in addition to these guidelines.

"Use of this document is governed by the terms of use on the first page of this document."

Appendix 11
Risks Associated with Atezolizumab and Guidelines for Management of Adverse Events Associated with Atezolizumab

Toxicities associated or possibly associated with atezolizumab treatment should be managed according to standard medical practice. Additional tests, such as autoimmune serology or biopsies, should be used to evaluate for a possible immunogenic etiology.

Although most *immune-mediated* adverse events observed with immunomodulatory agents have been mild and self-limiting, such events should be recognized early and treated promptly to avoid potential major complications. Discontinuation of atezolizumab may not have an immediate therapeutic effect, and in severe cases, *immune-mediated* toxicities may require acute management with topical corticosteroids, systemic corticosteroids, or other immunosuppressive agents.

The investigator should consider the benefit–risk balance a given patient may be experiencing prior to further administration of atezolizumab. In patients who have met the criteria for permanent discontinuation, resumption of atezolizumab may be considered if the patient is deriving benefit and has fully recovered from the *immune-mediated* event. Patients can be re-challenged with atezolizumab only after approval has been documented by both the investigator (or an appropriate delegate) and the Medical Monitor.

PULMONARY EVENTS

Dyspnea, cough, fatigue, hypoxia, pneumonitis, and pulmonary infiltrates have been associated with the administration of atezolizumab. Patients will be assessed for pulmonary signs and symptoms throughout the study and will also have computed tomography (CT) scans of the chest performed at every tumor assessment.

All pulmonary events should be thoroughly evaluated for other commonly reported etiologies such as pneumonia or other infection, lymphangitic carcinomatosis, pulmonary embolism, heart failure, chronic obstructive pulmonary disease, or pulmonary hypertension. Management guidelines for pulmonary events are provided in Table 1.

“Use of this document is governed by the terms of use on the first page of this document.”

Appendix 11
Risks Associated with Atezolizumab and Guidelines for Management of Adverse Events Associated with Atezolizumab (cont.)

Table 1 Management Guidelines for Pulmonary Events, Including Pneumonitis

Event	Management
Pulmonary event, Grade 1	• Continue atezolizumab and monitor closely.<br>• Re-evaluate on serial imaging.<br>• Consider patient referral to pulmonary specialist.
Pulmonary event, Grade 2	• Withhold atezolizumab for up to 12 weeks after event onset. [a]<br>• Refer patient to pulmonary and infectious disease specialists and consider bronchoscopy or BAL.<br>• Initiate treatment with 1–2 mg/kg/day oral prednisone or equivalent.<br>• If event resolves to Grade 1 or better, resume atezolizumab. [b]<br>• If event does not resolve to Grade 1 or better while withholding atezolizumab, permanently discontinue atezolizumab and contact Medical Monitor. [c]<br>• For recurrent events, treat as a Grade 3 or 4 event.
Pulmonary event, Grade 3 or 4	• Permanently discontinue atezolizumab and contact Medical Monitor. [c]<br>• Bronchoscopy or BAL is recommended.<br>• Initiate treatment with 1–2 mg/kg/day oral prednisone or equivalent.<br>• If event does not improve within 48 hours after initiating corticosteroids, consider adding an immunosuppressive agent.<br>• If event resolves to Grade 1 or better, taper corticosteroids over ≥ 1 month.

BAL = bronchoscopic alveolar lavage.

[a] Atezolizumab may be withheld for a longer period of time (i.e., > 12 weeks after event onset) to allow for corticosteroids (if initiated) to be reduced to ≤ 10 mg/day oral prednisone or equivalent. The acceptable length of the extended period of time must be agreed upon by the investigator and the Medical Monitor.

[b] If corticosteroids have been initiated, they must be tapered over ≥ 1 month to ≤ 10 mg/day oral prednisone or equivalent before atezolizumab can be resumed.

[c] Resumption of atezolizumab may be considered in patients who are deriving benefit and have fully recovered from the *immune-mediated* event. Patients can be re-challenged with atezolizumab only after approval has been documented by both the investigator (or an appropriate delegate) and the Medical Monitor.

"Use of this document is governed by the terms of use on the first page of this document."

HEPATIC EVENTS

Immune-mediated hepatitis has been associated with the administration of atezolizumab. Eligible patients must have adequate liver function, as manifested by measurements of total bilirubin and hepatic transaminases, and liver function will be monitored throughout study treatment. Management guidelines for hepatic events are provided in Table 2.

Patients with right upper-quadrant abdominal pain and/or unexplained nausea or vomiting should have liver function tests (LFTs) performed immediately and reviewed before administration of the next dose of study drug.

For patients with elevated LFTs, concurrent medication, viral hepatitis, and toxic or neoplastic etiologies should be considered and addressed, as appropriate.

Table 2 Management Guidelines for Hepatic Events

Event	Management
Hepatic event, Grade 1	• Continue atezolizumab.<br>• Monitor LFTs until values resolve to within normal limits.
Hepatic event, Grade 2	**All events:**<br>• Monitor LFTs more frequently until return to baseline values.<br>**Events of > 5 days' duration:**<br>• Withhold atezolizumab for up to 12 weeks after event onset. [a]<br>• Initiate treatment with 1–2 mg/kg/day oral prednisone or equivalent.<br>• If event resolves to Grade 1 or better, resume atezolizumab. [b]<br>• If event does not resolve to Grade 1 or better while withholding atezolizumab, permanently discontinue atezolizumab and contact Medical Monitor. [c]

LFT = liver function test.

[a] Atezolizumab may be withheld for a longer period of time (i.e., > 12 weeks after event onset) to allow for corticosteroids (if initiated) to be reduced to ≤ 10 mg/day oral prednisone or equivalent. The acceptable length of the extended period of time must be agreed upon by the investigator and the Medical Monitor.

[b] If corticosteroids have been initiated, they must be tapered over ≥ 1 month to ≤ 10 mg/day oral prednisone or equivalent before atezolizumab can be resumed.

[c] Resumption of atezolizumab may be considered in patients who are deriving benefit and have fully recovered from the *immune-mediated* event. Patients can be re-challenged with atezolizumab only after approval has been documented by both the investigator (or an appropriate delegate) and the Medical Monitor.

"Use of this document is governed by the terms of use on the first page of this document."

Table 2 Management Guidelines for Hepatic Events (cont.)

Event	Management
Hepatic event, Grade 3 or 4	• Permanently discontinue atezolizumab and contact Medical Monitor. [c]<br>• Consider patient referral to gastrointestinal specialist for evaluation and liver biopsy to establish etiology of hepatic injury.<br>• Initiate treatment with 1–2 mg/kg/day oral prednisone or equivalent.<br>• If event does not improve within 48 hours after initiating corticosteroids, consider adding an immunosuppressive agent.<br>• If event resolves to Grade 1 or better, taper corticosteroids over ≥ 1 month.

LFT = liver function test.

[a] Atezolizumab may be withheld for a longer period of time (i.e., > 12 weeks after event onset) to allow for corticosteroids (if initiated) to be reduced to ≤ 10 mg/day oral prednisone or equivalent. The acceptable length of the extended period of time must be agreed upon by the investigator and the Medical Monitor.

[b] If corticosteroids have been initiated, they must be tapered over ≥ 1 month to ≤ 10 mg/day oral prednisone or equivalent before atezolizumab can be resumed.

[c] Resumption of atezolizumab may be considered in patients who are deriving benefit and have fully recovered from the *immune-mediated* event. Patients can be re-challenged with atezolizumab only after approval has been documented by both the investigator (or an appropriate delegate) and the Medical Monitor.

GASTROINTESTINAL EVENTS

Immune-mediated colitis has been associated with the administration of atezolizumab. Management guidelines for diarrhea or colitis are provided in Table 3.

All events of diarrhea or colitis should be thoroughly evaluated for other more common etiologies. For events of significant duration or magnitude or associated with signs of systemic inflammation or acute-phase reactants (e.g., increased C-reactive protein, platelet count, or bandemia): Perform sigmoidoscopy (or colonoscopy, if appropriate) with colonic biopsy, with three to five specimens for standard paraffin block to check for inflammation and lymphocytic infiltrates to confirm colitis diagnosis.

"Use of this document is governed by the terms of use on the first page of this document."

Table 3 Management Guidelines for Gastrointestinal Events (Diarrhea or Colitis)

Event	Management
Diarrhea or colitis, Grade 1	• Continue atezolizumab.<br>• Initiate symptomatic treatment.<br>• Endoscopy is recommended if symptoms persist for > 7 days.<br>• Monitor closely.
Diarrhea or colitis, Grade 2	• Withhold atezolizumab for up to 12 weeks after event onset. [a]<br>• Initiate symptomatic treatment.<br>• Patient referral to GI specialist is recommended.<br>• For recurrent events or events that persist > 5 days, initiate treatment with 1–2 mg/kg/day oral prednisone or equivalent.<br>• If event resolves to Grade 1 or better, resume atezolizumab. [b]<br>• If event does not resolve to Grade 1 or better while withholding atezolizumab, permanently discontinue atezolizumab and contact Medical Monitor. [c]
Diarrhea or colitis, Grade 3	• Withhold atezolizumab for up to 12 weeks after event onset. [a]<br>• Refer patient to GI specialist for evaluation and confirmatory biopsy.<br>• Initiate treatment with 1–2 mg/kg/day IV methylprednisolone or equivalent and convert to 1–2 mg/kg/day oral prednisone or equivalent upon improvement.<br>• If event resolves to Grade 1 or better, resume atezolizumab. [b]<br>• If event does not resolve to Grade 1 or better while withholding atezolizumab, permanently discontinue atezolizumab and contact Medical Monitor. [c]

GI = gastrointestinal.

[a] Atezolizumab may be withheld for a longer period of time (i.e., > 12 weeks after event onset) to allow for corticosteroids (if initiated) to be reduced to ≤ 10 mg/day oral prednisone or equivalent. The acceptable length of the extended period of time must be agreed upon by the investigator and the Medical Monitor.

[b] If corticosteroids have been initiated, they must be tapered over ≥ 1 month to ≤ 10 mg/day oral prednisone or equivalent before atezolizumab can be resumed.

[c] Resumption of atezolizumab may be considered in patients who are deriving benefit and have fully recovered from the *immune-mediated* event. Patients can be re-challenged with atezolizumab only after approval has been documented by both the investigator (or an appropriate delegate) and the Medical Monitor.

“Use of this document is governed by the terms of use on the first page of this document.”

Table 3 Management Guidelines for Gastrointestinal Events (Diarrhea or Colitis) (cont.)

Event	Management
Diarrhea or colitis, Grade 4	• Permanently discontinue atezolizumab and contact Medical Monitor. [c]<br>• Refer patient to GI specialist for evaluation and confirmation biopsy.<br>• Initiate treatment with 1–2 mg/kg/day IV methylprednisolone or equivalent and convert to 1–2 mg/kg/day oral prednisone or equivalent upon improvement.<br>• If event does not improve within 48 hours after initiating corticosteroids, consider adding an immunosuppressive agent.<br>• If event resolves to Grade 1 or better, taper corticosteroids over ≥ 1 month.

GI = gastrointestinal.

[a] Atezolizumab may be withheld for a longer period of time (i.e., > 12 weeks after event onset) to allow for corticosteroids (if initiated) to be reduced to ≤ 10 mg/day oral prednisone or equivalent. The acceptable length of the extended period of time must be agreed upon by the investigator and the Medical Monitor.

[b] If corticosteroids have been initiated, they must be tapered over ≥ 1 month to ≤ 10 mg/day oral prednisone or equivalent before atezolizumab can be resumed.

[c] Resumption of atezolizumab may be considered in patients who are deriving benefit and have fully recovered from the *immune-mediated* event. Patients can be re-challenged with atezolizumab only after approval has been documented by both the investigator (or an appropriate delegate) and the Medical Monitor.

ENDOCRINE EVENTS

Thyroid disorders, adrenal insufficiency, diabetes mellitus, and pituitary disorders have been associated with the administration of atezolizumab. Management guidelines for endocrine events are provided in Table 4.

Patients with unexplained symptoms such as headache, fatigue, myalgias, impotence, constipation, or mental status changes should be investigated for the presence of thyroid, pituitary, or adrenal endocrinopathies. The patient should be referred to an endocrinologist if an endocrinopathy is suspected. Thyroid-stimulating hormone (TSH) and free triiodothyronine and thyroxine levels should be measured to determine whether thyroid abnormalities are present. Pituitary hormone levels and function tests (e.g., TSH, growth hormone, luteinizing hormone, follicle-stimulating hormone, testosterone, prolactin, adrenocorticotropic hormone [ACTH] levels, and ACTH stimulation test) and magnetic resonance imaging (MRI) of the brain (with detailed pituitary sections) may help to differentiate primary pituitary insufficiency from primary adrenal insufficiency.

“Use of this document is governed by the terms of use on the first page of this document.”

Table 4 Management Guidelines for Endocrine Events

Event	Management
Asymptomatic hypothyroidism	• Continue atezolizumab.<br>• Initiate treatment with thyroid replacement hormone.<br>• Monitor TSH weekly.
Symptomatic hypothyroidism	• Withhold atezolizumab.<br>• Initiate treatment with thyroid replacement hormone.<br>• Monitor TSH weekly.<br>• Consider patient referral to endocrinologist.<br>• Resume atezolizumab when symptoms are controlled and thyroid function is improving.
Asymptomatic hyperthyroidism	**TSH ≥ 0.1 mU/L and < 0.5 mU/L:**<br>• Continue atezolizumab.<br>• Monitor TSH every 4 weeks.<br>**TSH < 0.1 mU/L:**<br>• Follow guidelines for symptomatic hyperthyroidism.
Symptomatic hyperthyroidism	• Withhold atezolizumab.<br>• Initiate treatment with anti-thyroid drug such as methimazole or carbimazole as needed.<br>• Consider patient referral to endocrinologist.<br>• Resume atezolizumab when symptoms are controlled and thyroid function is improving.<br>• Permanently discontinue atezolizumab and contact Medical Monitor for life-threatening *immune-mediated* hyperthyroidism. [c]

MRI = magnetic resonance imaging; TSH = thyroid-stimulating hormone.

[a] Atezolizumab may be withheld for a longer period of time (i.e., > 12 weeks after event onset) to allow for corticosteroids (if initiated) to be reduced to ≤ 10 mg/day oral prednisone or equivalent. The acceptable length of the extended period of time must be agreed upon by the investigator and the Medical Monitor.

[b] If corticosteroids have been initiated, they must be tapered over ≥ 1 month to ≤ 10 mg/day oral prednisone or equivalent before atezolizumab can be resumed.

[c] Resumption of atezolizumab may be considered in patients who are deriving benefit and have fully recovered from the *immune-mediated* event. Patients can be re-challenged with atezolizumab only after approval has been documented by both the investigator (or an appropriate delegate) and the Medical Monitor.

“Use of this document is governed by the terms of use on the first page of this document.”

Table 4 Management Guidelines for Endocrine Events (cont.)

Event	Management
Symptomatic adrenal insufficiency, Grade 2–4	• Withhold atezolizumab for up to 12 weeks after event onset. [a]<br>• Refer patient to endocrinologist.<br>• Perform appropriate imaging.<br>• Initiate treatment with 1–2 mg/kg/day IV methylprednisolone or equivalent and convert to 1–2 mg/kg/day oral prednisone or equivalent upon improvement.<br>• If event resolves to Grade 1 or better and patient is stable on replacement therapy, resume atezolizumab. [b]<br>• If event does not resolve to Grade 1 or better or patient is not stable on replacement therapy while withholding atezolizumab, permanently discontinue atezolizumab and contact Medical Monitor. [c]
Hyperglycemia, Grade 1 or 2	• Continue atezolizumab.<br>• Initiate treatment with insulin if needed.<br>• Monitor for glucose control.
Hyperglycemia, Grade 3 or 4	• Withhold atezolizumab.<br>• Initiate treatment with insulin.<br>• Monitor for glucose control.<br>• Resume atezolizumab when symptoms resolve and glucose levels are stable.

MRI = magnetic resonance imaging; TSH = thyroid-stimulating hormone.

[a] Atezolizumab may be withheld for a longer period of time (i.e., > 12 weeks after event onset) to allow for corticosteroids (if initiated) to be reduced to ≤ 10 mg/day oral prednisone or equivalent. The acceptable length of the extended period of time must be agreed upon by the investigator and the Medical Monitor.

[b] If corticosteroids have been initiated, they must be tapered over ≥ 1 month to ≤ 10 mg/day oral prednisone or equivalent before atezolizumab can be resumed.

[c] Resumption of atezolizumab may be considered in patients who are deriving benefit and have fully recovered from the *immune-mediated* event. Patients can be re-challenged with atezolizumab only after approval has been documented by both the investigator (or an appropriate delegate) and the Medical Monitor.

"Use of this document is governed by the terms of use on the first page of this document."

Table 4 Management Guidelines for Endocrine Events (cont.)

Event	Management
Hypophysitis (pan-hypopituitarism), Grade 2 or 3	• Withhold atezolizumab for up to 12 weeks after event onset. [a]<br>• Refer patient to endocrinologist.<br>• Perform brain MRI (pituitary protocol).<br>• Initiate treatment with 1–2 mg/kg/day IV methylprednisolone or equivalent and convert to 1–2 mg/kg/day oral prednisone or equivalent upon improvement.<br>• Initiate hormone replacement if clinically indicated.<br>• If event resolves to Grade 1 or better, resume atezolizumab. [b]<br>• If event does not resolve to Grade 1 or better while withholding atezolizumab, permanently discontinue atezolizumab and contact Medical Monitor. [c]<br>• For recurrent hypophysitis, treat as a Grade 4 event.
Hypophysitis (pan-hypopituitarism), Grade 4	• Permanently discontinue atezolizumab and contact Medical Monitor. [c]<br>• Refer patient to endocrinologist.<br>• Perform brain MRI (pituitary protocol).<br>• Initiate treatment with 1–2 mg/kg/day IV methylprednisolone or equivalent and convert to 1–2 mg/kg/day oral prednisone or equivalent upon improvement.<br>• Initiate hormone replacement if clinically indicated.

MRI = magnetic resonance imaging; TSH = thyroid-stimulating hormone.

[a] Atezolizumab may be withheld for a longer period of time (i.e., > 12 weeks after event onset) to allow for corticosteroids (if initiated) to be reduced to ≤ 10 mg/day oral prednisone or equivalent. The acceptable length of the extended period of time must be agreed upon by the investigator and the Medical Monitor.

[b] If corticosteroids have been initiated, they must be tapered over ≥ 1 month to ≤ 10 mg/day oral prednisone or equivalent before atezolizumab can be resumed.

[c] Resumption of atezolizumab may be considered in patients who are deriving benefit and have fully recovered from the *immune-mediated* event. Patients can be re-challenged with atezolizumab only after approval has been documented by both the investigator (or an appropriate delegate) and the Medical Monitor.

"Use of this document is governed by the terms of use on the first page of this document."

OCULAR EVENTS

An ophthalmologist should evaluate visual complaints (e.g., uveitis, retinal events). Management guidelines for ocular events are provided in Table 5.

Table 5 Management Guidelines for Ocular Events

Event	Management
Ocular event, Grade 1	• Continue atezolizumab.<br>• Patient referral to ophthalmologist is strongly recommended.<br>• Initiate treatment with topical corticosteroid eye drops and topical immunosuppressive therapy.<br>• If symptoms persist, treat as a Grade 2 event.
Ocular event, Grade 2	• Withhold atezolizumab for up to 12 weeks after event onset. [a]<br>• Patient referral to ophthalmologist is strongly recommended.<br>• Initiate treatment with topical corticosteroid eye drops and topical immunosuppressive therapy.<br>• If event resolves to Grade 1 or better, resume atezolizumab. [b]<br>• If event does not resolve to Grade 1 or better while withholding atezolizumab, permanently discontinue atezolizumab and contact Medical Monitor. [c]
Ocular event, Grade 3 or 4	• Permanently discontinue atezolizumab and contact Medical Monitor. [c]<br>• Refer patient to ophthalmologist.<br>• Initiate treatment with 1–2 mg/kg/day oral prednisone or equivalent.<br>• If event resolves to Grade 1 or better, taper corticosteroids over ≥ 1 month.

[a] Atezolizumab may be withheld for a longer period of time (i.e., > 12 weeks after event onset) to allow for corticosteroids (if initiated) to be reduced to ≤ 10 mg/day oral prednisone or equivalent. The acceptable length of the extended period of time must be agreed upon by the investigator and the Medical Monitor.

[b] If corticosteroids have been initiated, they must be tapered over ≥ 1 month to ≤ 10 mg/day oral prednisone or equivalent before atezolizumab can be resumed.

[c] Resumption of atezolizumab may be considered in patients who are deriving benefit and have fully recovered from the *immune-mediated* event. Patients can be re-challenged with atezolizumab only after approval has been documented by both the investigator (or an appropriate delegate) and the Medical Monitor.

"Use of this document is governed by the terms of use on the first page of this document."

IMMUNE-MEDIATED MYOCARDITIS

Immune-mediated myocarditis has been associated with the administration of atezolizumab. *Immune-mediated* myocarditis should be suspected in any patient presenting with signs or symptoms suggestive of myocarditis, including, but not limited to, *laboratory (e.g., B-type natriuretic peptide) or cardiac imaging abnormalities,* dyspnea, chest pain, palpitations, fatigue, decreased exercise tolerance, or syncope. *Immune-mediated* myocarditis needs to be distinguished from myocarditis resulting from infection (commonly viral, e.g., in a patient who reports a recent history of gastrointestinal illness), ischemic events, underlying arrhythmias, exacerbation of preexisting cardiac conditions, or progression of malignancy.

All patients with possible myocarditis should be urgently evaluated by performing cardiac enzyme assessment, an ECG, a chest X-ray, an echocardiogram, and a cardiac MRI as appropriate per institutional guidelines. A cardiologist should be consulted. An endomyocardial biopsy may be considered to enable a definitive diagnosis and appropriate treatment, if clinically indicated.

Patients with signs and symptoms of myocarditis, in the absence of an identified alternate etiology, should be treated according to the guidelines in Table 6.

“Use of this document is governed by the terms of use on the first page of this document.”

Table 6 Management Guidelines for *Immune-Mediated* Myocarditis

Event	Management
Immune-mediated myocarditis, Grade 1	• Refer patient to cardiologist.<br>• Initiate treatment as per institutional guidelines.
Immune-mediated myocarditis, Grade 2	• Withhold atezolizumab for up to 12 weeks after event onset [a] and contact Medical Monitor.<br>• Refer patient to cardiologist.<br>• Initiate treatment as per institutional guidelines and consider antiarrhythmic drugs, temporary pacemaker, ECMO, or VAD as appropriate.<br>• Consider treatment with 1–2 mg/kg/day IV methylprednisolone or equivalent and convert to 1–2 mg/kg/day oral prednisone or equivalent upon improvement.[a]<br>• If event resolves to Grade 1 or better, resume atezolizumab. [b]<br>• If event does not resolve to Grade 1 or better while withholding atezolizumab, permanently discontinue atezolizumab and contact Medical Monitor. [c]
Immune-mediated myocarditis, Grade 3-4	• Permanently discontinue atezolizumab and contact Medical Monitor. [c]<br>• Refer patient to cardiologist.<br>• Initiate treatment as per institutional guidelines and consider antiarrhythmic drugs, temporary pacemaker, ECMO, or VAD as appropriate.<br>• Initiate treatment with 1–2 mg/kg/day IV methylprednisolone or equivalent and convert to 1–2 mg/kg/day oral prednisone or equivalent upon improvement. [a,b]<br>• If event does not improve within 48 hours after initiating corticosteroids, consider adding an immunosuppressive agent.<br>• If event resolves to Grade 1 or better, taper corticosteroids over ≥ 1 month.

ECMO = extracorporeal membrane oxygenation; VAD = ventricular assist device.

[a] Atezolizumab may be withheld for a longer period of time (i.e., > 12 weeks after event onset) to allow for corticosteroids (if initiated) to be reduced to ≤ 10 mg/day oral prednisone or equivalent. The acceptable length of the extended period of time must be agreed upon by the investigator and the Medical Monitor.

[b] If corticosteroids have been initiated, they must be tapered over ≥ 1 month to ≤ 10 mg/day oral prednisone or equivalent before atezolizumab can be resumed.

[c] Resumption of atezolizumab may be considered in patients who are deriving benefit and have fully recovered from the *immune-mediated* event. Patients can be re-challenged with atezolizumab only after approval has been documented by both the investigator (or an appropriate delegate) and the Medical Monitor.

"Use of this document is governed by the terms of use on the first page of this document."

INFUSION-RELATED REACTIONS

No premedication is indicated for the administration of Cycle 1 of atezolizumab. However, patients who experience an infusion-related reaction (IRR) with Cycle 1 of atezolizumab may receive premedication with antihistamines or antipyretics/analgesics (e.g., acetaminophen) for subsequent infusions. Metamizole (dipyrone) is prohibited in treating atezolizumab-associated IRRs because of its potential for causing agranulocytosis.

Guidelines for medical management of IRRs during Cycle 1 are provided in Table 7. For subsequent cycles, IRRs should be managed according to institutional guidelines.

Table 7 Management Guidelines for Infusion-Related Reactions

Event	Management
IRR, Grade 1	• Reduce infusion rate to half the rate being given at the time of event onset.<br>• After the event has resolved, the investigator should wait for 30 minutes while delivering the infusion at the reduced rate.<br>• If the infusion is tolerated at the reduced rate for 30 minutes after symptoms have resolved, the infusion rate may be increased to the original rate.
IRR, Grade 2	• Interrupt atezolizumab infusion.<br>• Administer aggressive symptomatic treatment (e.g., oral or IV antihistamine, anti-pyretic medication, glucocorticoids, epinephrine, bronchodilators, oxygen, IV fluids).<br>• After symptoms have resolved to baseline, resume infusion at half the rate being given at the time of event onset.<br>• For subsequent infusions, consider administration of oral premedication with antihistamines, anti-pyretics, and/or analgesics and monitor closely for IRRs.
IRR, Grade 3 or 4	• Stop infusion.<br>• Administer aggressive symptomatic treatment (e.g., oral or IV antihistamine, anti-pyretic, glucocorticoids, epinephrine, bronchodilators, oxygen, IV fluids).<br>• Permanently discontinue atezolizumab and contact Medical Monitor. [a]

IRR = infusion-related reaction.

[a] Resumption of atezolizumab may be considered in patients who are deriving benefit and have fully recovered from the event. Patients can be re-challenged with atezolizumab only after approval has been documented by both the investigator (or an appropriate delegate) and the Medical Monitor.

"Use of this document is governed by the terms of use on the first page of this document."

PANCREATIC EVENTS

Symptoms of abdominal pain associated with elevations of amylase and lipase, suggestive of pancreatitis, have been associated with the administration of atezolizumab. The differential diagnosis of acute abdominal pain should include pancreatitis. Appropriate work-up should include an evaluation for ductal obstruction, as well as serum amylase and lipase tests. Management guidelines for pancreatic events, including pancreatitis, are provided in Table 8.

Table 8 Management Guidelines for Pancreatic Events, Including Pancreatitis

Event	Management
Amylase and/or lipase elevation, Grade 2	**Amylase and/or lipase > 1.5–2.0 × ULN:**<br>• Continue atezolizumab.<br>• Monitor amylase and lipase weekly.<br>• For prolonged elevation (e.g., > 3 weeks), consider treatment with 10 mg/day oral prednisone or equivalent.<br>**Asymptomatic with amylase and/or lipase > 2.0–5.0 × ULN:**<br>• Treat as a Grade 3 event.
Amylase and/or lipase elevation, Grade 3 or 4	• Withhold atezolizumab for up to 12 weeks after event onset. [a]<br>• Refer patient to GI specialist.<br>• Monitor amylase and lipase every other day.<br>• If no improvement, consider treatment with 1–2 mg/kg/day oral prednisone or equivalent.<br>• If event resolves to Grade 1 or better, resume atezolizumab. [b]<br>• If event does not resolve to Grade 1 or better while withholding atezolizumab, permanently discontinue atezolizumab and contact Medical Monitor. [c]<br>• For recurrent events, permanently discontinue atezolizumab and contact Medical Monitor. [c]

GI = gastrointestinal.

[a] Atezolizumab may be withheld for a longer period of time (i.e., > 12 weeks after event onset) to allow for corticosteroids (if initiated) to be reduced to ≤ 10 mg/day oral prednisone or equivalent. The acceptable length of the extended period of time must be agreed upon by the investigator and the Medical Monitor.

[b] If corticosteroids have been initiated, they must be tapered over ≥ 1 month to ≤ 10 mg/day oral prednisone or equivalent before atezolizumab can be resumed.

[c] Resumption of atezolizumab may be considered in patients who are deriving benefit and have fully recovered from the *immune-mediated* event. Patients can be re-challenged with atezolizumab only after approval has been documented by both the investigator (or an appropriate delegate) and the Medical Monitor.

"Use of this document is governed by the terms of use on the first page of this document."

Table 8 Management Guidelines for Pancreatic Events, Including Pancreatitis (cont.)

Event	Management
Immune-mediated pancreatitis, Grade 2 or 3	• Withhold atezolizumab for up to 12 weeks after event onset. [a]<br>• Refer patient to GI specialist.<br>• Initiate treatment with 1–2 mg/kg/day IV methylprednisolone or equivalent and convert to 1–2 mg/kg/day oral prednisone or equivalent upon improvement.<br>• If event resolves to Grade 1 or better, resume atezolizumab. [b]<br>• If event does not resolve to Grade 1 or better while withholding atezolizumab, permanently discontinue atezolizumab and contact Medical Monitor. [c]<br>• For recurrent events, permanently discontinue atezolizumab and contact Medical Monitor. [c]
Immune-mediated pancreatitis, Grade 4	• Permanently discontinue atezolizumab and contact Medical Monitor. [c]<br>• Refer patient to GI specialist.<br>• Initiate treatment with 1–2 mg/kg/day IV methylprednisolone or equivalent and convert to 1–2 mg/kg/day oral prednisone or equivalent upon improvement.<br>• If event does not improve within 48 hours after initiating corticosteroids, consider adding an immunosuppressive agent.<br>• If event resolves to Grade 1 or better, taper corticosteroids over ≥ 1 month.

GI = gastrointestinal.

[a] Atezolizumab may be withheld for a longer period of time (i.e., > 12 weeks after event onset) to allow for corticosteroids (if initiated) to be reduced to ≤ 10 mg/day oral prednisone or equivalent. The acceptable length of the extended period of time must be agreed upon by the investigator and the Medical Monitor.

[b] If corticosteroids have been initiated, they must be tapered over ≥ 1 month to ≤ 10 mg/day oral prednisone or equivalent before atezolizumab can be resumed.

[c] Resumption of atezolizumab may be considered in patients who are deriving benefit and have fully recovered from the *immune-mediated* event. Patients can be re-challenged with atezolizumab only after approval has been documented by both the investigator (or an appropriate delegate) and the Medical Monitor.

“Use of this document is governed by the terms of use on the first page of this document.”

DERMATOLOGIC EVENTS

Treatment-emergent rash has been associated with atezolizumab. The majority of cases of rash were mild in severity and self limited, with or without pruritus. A dermatologist should evaluate persistent and/or severe rash or pruritus. A biopsy should be considered unless contraindicated. Management guidelines for dermatologic events are provided in Table 9.

Table 9 Management Guidelines for Dermatologic Events

Event	Management
Dermatologic event, Grade 1	• Continue atezolizumab.<br>• Consider treatment with topical corticosteroids and/or other symptomatic therapy (e.g., antihistamines).
Dermatologic event, Grade 2	• Continue atezolizumab.<br>• Consider patient referral to dermatologist.<br>• Initiate treatment with topical corticosteroids.<br>• Consider treatment with higher-potency topical corticosteroids if event does not improve.
Dermatologic event, Grade 3	• Withhold atezolizumab for up to 12 weeks after event onset. [a]<br>• Refer patient to dermatologist.<br>• Initiate treatment with 10 mg/day oral prednisone or equivalent, increasing dose to 1–2 mg/kg/day if event does not improve within 48–72 hours.<br>• If event resolves to Grade 1 or better, resume atezolizumab. [b]<br>• If event does not resolve to Grade 1 or better while withholding atezolizumab, permanently discontinue atezolizumab and contact Medical Monitor. [c]
Dermatologic event, Grade 4	• Permanently discontinue atezolizumab and contact Medical Monitor. [c]

[a] Atezolizumab may be withheld for a longer period of time (i.e., $>$ 12 weeks after event onset) to allow for corticosteroids (if initiated) to be reduced to $\leq$ 10 mg/day oral prednisone or equivalent. The acceptable length of the extended period of time must be agreed upon by the investigator and the Medical Monitor.

[b] If corticosteroids have been initiated, they must be tapered over $\geq$ 1 month to $\leq$ 10 mg/day oral prednisone or equivalent before atezolizumab can be resumed.

[c] Resumption of atezolizumab may be considered in patients who are deriving benefit and have fully recovered from the *immune-mediated* event. Patients can be re-challenged with atezolizumab only after approval has been documented by both the investigator (or an appropriate delegate) and the Medical Monitor.

"Use of this document is governed by the terms of use on the first page of this document."

NEUROLOGIC DISORDERS

Myasthenia gravis and Guillain-Barré syndrome have been observed with single-agent atezolizumab. Patients may present with signs and symptoms of sensory and/or motor neuropathy. Diagnostic work-up is essential for an accurate characterization to differentiate between alternative etiologies. Management guidelines for neurologic disorders are provided in Table 10.

Table 10 Management Guidelines for Neurologic Disorders

Event	Management
Immune-mediated neuropathy, Grade 1	• Continue atezolizumab.<br>• Investigate etiology.
Immune-mediated neuropathy, Grade 2	• Withhold atezolizumab for up to 12 weeks after event onset. [a]<br>• Investigate etiology.<br>• Initiate treatment as per institutional guidelines.<br>• If event resolves to Grade 1 or better, resume atezolizumab. [b]<br>• If event does not resolve to Grade 1 or better while withholding atezolizumab, permanently discontinue atezolizumab and contact Medical Monitor. [c]
Immune-mediated neuropathy, Grade 3 or 4	• Permanently discontinue atezolizumab and contact Medical Monitor. [c]<br>• Initiate treatment as per institutional guidelines.
Myasthenia gravis and Guillain-Barré syndrome (any grade)	• Permanently discontinue atezolizumab and contact Medical Monitor. [c]<br>• Refer patient to neurologist.<br>• Initiate treatment as per institutional guidelines.<br>• Consider initiation of 1–2 mg/kg/day oral or IV prednisone or equivalent.

[a] Atezolizumab may be withheld for a longer period of time (i.e., > 12 weeks after event onset) to allow for corticosteroids (if initiated) to be reduced to ≤ 10 mg/day oral prednisone or equivalent. The acceptable length of the extended period of time must be agreed upon by the investigator and the Medical Monitor.

[b] If corticosteroids have been initiated, they must be tapered over ≥ 1 month to ≤ 10 mg/day oral prednisone or equivalent before atezolizumab can be resumed.

[c] Resumption of atezolizumab may be considered in patients who are deriving benefit and have fully recovered from the *immune-mediated* event. Patients can be re-challenged with atezolizumab only after approval has been documented by both the investigator (or an appropriate delegate) and the Medical Monitor.

“Use of this document is governed by the terms of use on the first page of this document.”

IMMUNE-MEDIATED MENINGOENCEPHALITIS

Immune-mediated meningoencephalitis is an identified risk associated with the administration of atezolizumab. *Immune-mediated* meningoencephalitis should be suspected in any patient presenting with signs or symptoms suggestive of meningitis or encephalitis, including, but not limited to, headache, neck pain, confusion, seizure, motor or sensory dysfunction, and altered or depressed level of consciousness. Encephalopathy from metabolic or electrolyte imbalances needs to be distinguished from potential meningoencephalitis resulting from infection (bacterial, viral, or fungal) or progression of malignancy, or secondary to a paraneoplastic process.

All patients being considered for meningoencephalitis should be urgently evaluated with a CT scan and/or MRI scan of the brain to evaluate for metastasis, inflammation, or edema. If deemed safe by the treating physician, a lumbar puncture should be performed and a neurologist should be consulted.

Patients with signs and symptoms of meningoencephalitis, in the absence of an identified alternate etiology, should be treated according to the guidelines in Table 11.

Table 11 Management Guidelines for *Immune-Mediated* Meningoencephalitis

Event	Management
Immune-mediated meningoencephalitis, all grades	• Permanently discontinue atezolizumab and contact Medical Monitor. [a]<br>• Refer patient to neurologist.<br>• Initiate treatment with 1–2 mg/kg/day IV methylprednisolone or equivalent and convert to 1–2 mg/kg/day oral prednisone or equivalent upon improvement.<br>• If event does not improve within 48 hours after initiating corticosteroids, consider adding an immunosuppressive agent.<br>• If event resolves to Grade 1 or better, taper corticosteroids over ≥ 1 month.

[a] Resumption of atezolizumab may be considered in patients who are deriving benefit and have fully recovered from the *immune-mediated* event. Patients can be re-challenged with atezolizumab only after approval has been documented by both the investigator (or an appropriate delegate) and the Medical Monitor.

“Use of this document is governed by the terms of use on the first page of this document.”

IMMUNE-MEDIATED NEPHRITIS

Immune-mediated nephritis has been associated with the administration of atezolizumab. Eligible patients must have adequate renal function. *Renal* function, including serum creatinine, should be monitored throughout study treatment. Patients with abnormal renal function should be evaluated and treated for other more common etiologies (including prerenal and postrenal causes, and concomitant medications such as non-steroidal anti-inflammatory drugs). Refer the patient to a renal specialist if clinically indicated. A renal biopsy may be required to enable a definitive diagnosis and appropriate treatment. If no alternative cause of acute kidney injury is identified, patients with signs and symptoms of acute kidney injury, in the absence of an identified alternate etiology, should be treated according to the management guidelines for *immune-mediated* renal events in Table 12.

“Use of this document is governed by the terms of use on the first page of this document.”

Table 12 Management Guidelines for Renal Events

Event	Management
Renal event, Grade 1	• Continue atezolizumab.<br>• Monitor kidney function, including creatinine, closely until values resolve to within normal limits or to baseline values.
Renal event, Grade 2	• Withhold atezolizumab for up to 12 weeks after event onset. [a]<br>• Refer patient to renal specialist.<br>• Initiate treatment with corticosteroids equivalent to 1–2 mg/kg/day oral prednisone.<br>• If event resolves to Grade 1 or better, resume atezolizumab. [b]<br>• If event does not resolve to Grade 1 or better while withholding atezolizumab, permanently discontinue atezolizumab and contact Medical Monitor. [c]
Renal event, Grade 3 or 4	• Permanently discontinue atezolizumab and contact Medical Monitor.<br>• Refer patient to renal specialist and consider renal biopsy.<br>• Initiate treatment with corticosteroids equivalent to 1–2 mg/kg/day oral prednisone.<br>• If event does not improve within 48 hours after initiating corticosteroids, consider adding an immunosuppressive agent.<br>• If event resolves to Grade 1 or better, taper corticosteroids over ≥ 1 month.

Note: Management guidelines are presented by adverse event severity based on NCI CTCAE and are applicable to both CTCAE Version 4.0 and CTCAE Version 5.0.

[a] Atezolizumab may be withheld for a longer period of time (i.e., > 12 weeks after event onset) to allow for corticosteroids (if initiated) to be reduced to the equivalent of ≤ 10 mg/day oral prednisone. The acceptable length of the extended period of time must be agreed upon by the investigator and the Medical Monitor.

[b] If corticosteroids have been initiated, they must be tapered over ≥ 1 month to the equivalent of ≤ 10 mg/day oral prednisone before atezolizumab can be resumed.

[c] Resumption of atezolizumab may be considered in patients who are deriving benefit and have fully recovered from the *immune-mediated* event. Patients can be re-challenged with atezolizumab only after approval has been documented by both the investigator (or an appropriate delegate) and the Medical Monitor.

"Use of this document is governed by the terms of use on the first page of this document."

IMMUNE-MEDIATED MYOSITIS

Immune-mediated myositis has been associated with the administration of atezolizumab. Myositis or inflammatory myopathies are a group of disorders sharing the common feature of inflammatory muscle injury; dermatomyositis and polymyositis are among the most common disorders. Initial diagnosis is based on clinical (muscle weakness, muscle pain, skin rash in dermatomyositis), biochemical (serum creatine kinase increase), and imaging (electromyography/MRI) features, and is confirmed with a muscle biopsy.

Patients with signs and symptoms of myositis, in the absence of an identified alternate etiology, should be treated according to the guidelines in Table 13.

“Use of this document is governed by the terms of use on the first page of this document.”

Table 13 Management Guidelines for *Immune-Mediated* Myositis

Event	Management
Immune-mediated myositis, Grade 1	• Continue atezolizumab.<br>• Refer patient to rheumatologist or neurologist.<br>• Initiate treatment as per institutional guidelines.
Immune-mediated myositis, Grade 2	• Withhold atezolizumab for up to 12 weeks after event onset [a] and contact Medical Monitor.<br>• Refer patient to rheumatologist or neurologist.<br>• Initiate treatment as per institutional guidelines.<br>• Consider treatment with corticosteroids equivalent to 1–2 mg/kg/day IV methylprednisolone and convert to 1–2 mg/kg/day oral prednisone or equivalent upon improvement.<br>• If corticosteroids are initiated and event does not improve within 48 hours after initiating corticosteroids, consider adding an immunosuppressive agent.<br>• If event resolves to Grade 1 or better, resume atezolizumab. [b]<br>• If event does not resolve to Grade 1 or better while withholding atezolizumab, permanently discontinue atezolizumab and contact Medical Monitor. [c]

[a] Atezolizumab may be withheld for a longer period of time (i.e., > 12 weeks after event onset) to allow for corticosteroids (if initiated) to be reduced to the equivalent of ≤ 10 mg/day oral prednisone. The acceptable length of the extended period of time must be agreed upon by the investigator and the Medical Monitor.

[b] If corticosteroids have been initiated, they must be tapered over ≥ 1 month to the equivalent of ≤ 10 mg/day oral prednisone before atezolizumab can be resumed.

[c] Resumption of atezolizumab may be considered in patients who are deriving benefit and have fully recovered from the *immune-mediated* event. Patients can be re-challenged with atezolizumab only after approval has been documented by both the investigator (or an appropriate delegate) and the Medical Monitor.

“Use of this document is governed by the terms of use on the first page of this document.”

Table 13 Management Guidelines for *Immune-Mediated* Myositis (cont.)

Immune-mediated myositis, Grade 3	• Withhold atezolizumab for up to 12 weeks after event onset [a] and contact Medical Monitor.<br>• Refer patient to rheumatologist or neurologist.<br>• Initiate treatment as per institutional guidelines.<br>• Respiratory support may be required in more severe cases.<br>• Initiate treatment with corticosteroids equivalent to 1–2 mg/kg/day IV methylprednisolone, or higher-dose bolus if patient is severely compromised (e.g., cardiac or respiratory symptoms, dysphagia, or weakness that severely limits mobility); convert to 1–2 mg/kg/day oral prednisone or equivalent upon improvement.<br>• If event does not improve within 48 hours after initiating corticosteroids, consider adding an immunosuppressive agent.<br>• If event resolves to Grade 1 or better, resume atezolizumab. [b]<br>• If event does not resolve to Grade 1 or better while withholding atezolizumab, permanently discontinue atezolizumab and contact Medical Monitor. [c]<br>• For recurrent events, treat as a Grade 4 event.
Immune-mediated myositis, Grade 4	• Permanently discontinue atezolizumab and contact Medical Monitor. [c]<br>• Refer patient to rheumatologist or neurologist.<br>• Initiate treatment as per institutional guidelines.<br>• Respiratory support may be required in more severe cases.<br>• Initiate treatment with corticosteroids equivalent to 1–2 mg/kg/day IV methylprednisolone, or higher-dose bolus if patient is severely compromised (e.g., cardiac or respiratory symptoms, dysphagia, or weakness that severely limits mobility); convert to 1–2 mg/kg/day oral prednisone or equivalent upon improvement.<br>• If event does not improve within 48 hours after initiating corticosteroids, consider adding an immunosuppressive agent.<br>• If event resolves to Grade 1 or better, taper corticosteroids over ≥ 1 month.

[a] Atezolizumab may be withheld for a longer period of time (i.e., > 12 weeks after event onset) to allow for corticosteroids (if initiated) to be reduced to the equivalent of ≤ 10 mg/day oral prednisone. The acceptable length of the extended period of time must be agreed upon by the investigator and the Medical Monitor.

[b] If corticosteroids have been initiated, they must be tapered over ≥ 1 month to the equivalent of ≤ 10 mg/day oral prednisone before atezolizumab can be resumed.

[c] Resumption of atezolizumab may be considered in patients who are deriving benefit and have fully recovered from the *immune-mediated* event. Patients can be re-challenged with atezolizumab only after approval has been documented by both the investigator (or an appropriate delegate) and the Medical Monitor.

"Use of this document is governed by the terms of use on the first page of this document."

HEMOPHAGOCYTIC LYMPHOHISTIOCYTOSIS AND MACROPHAGE ACTIVATION SYNDROME

Immune-mediated reactions may involve any organ system and may lead to hemophagocytic lymphohistiocytosis (HLH) and macrophage activation syndrome (MAS).

Patients with suspected HLH should be diagnosed according to published criteria by McClain and Eckstein (2014). A patient should be classified as having HLH if five of the following eight criteria are met:

- *Fever ≥38.5°C*
- *Splenomegaly*
- *Peripheral blood cytopenia consisting of at least two of the following:*
  - *Hemoglobin <90 g/L (9 g/dL) (<100 g/L [10 g/dL] for infants <4 weeks old)*
  - *Platelet count <100 × 10^9/L (100,000/μL)*
  - *ANC <1.0 × 10^9/L (1000/μL)*
- *Fasting triglycerides >2.992 mmol/L (265 mg/dL) and/or fibrinogen <1.5 g/L (150 mg/dL)*
- *Hemophagocytosis in bone marrow, spleen, lymph node, or liver*
- *Low or absent natural killer cell activity*
- *Ferritin >500 mg/L (500 ng/mL)*
- *Soluble interleukin 2 (IL-2) receptor (soluble CD25) elevated ≥2 standard deviations above age-adjusted laboratory-specific norms*

Patients with suspected MAS should be diagnosed according to published criteria for systemic juvenile idiopathic arthritis by Ravelli et al. (2016). A febrile patient should be classified as having MAS if the following criteria are met:

- *Ferritin >684 mg/L (684 ng/mL)*
- *At least two of the following:*
  - *Platelet count ≤181 × 10^9/L (181,000/μL)*
  - *AST ≥48 U/L*
  - *Triglycerides >1.761 mmol/L (156 mg/dL)*
  - *Fibrinogen ≤3.6 g/L (360 mg/dL)*

"Use of this document is governed by the terms of use on the first page of this document."

Patients with suspected HLH or MAS should be treated according to the guidelines in Table 14.

Table 14 Management Guidelines for Suspected Hemophagocytic Lymphohistiocytosis or Macrophage Activation Syndrome

Event	*Management*
Suspected HLH or MAS	• *Permanently discontinue atezolizumab and contact Medical Monitor.*<br>• *Consider patient referral to hematologist.*<br>• *Initiate supportive care, including intensive care monitoring if indicated per institutional guidelines.*<br>• *Consider initiation of IV corticosteroids and/or an immunosuppressive agent.*<br>• *If event does not improve within 48 hours after initiating corticosteroids, consider adding an immunosuppressive agent.*<br>• *If event resolves to Grade 1 or better, taper corticosteroids over ≥1 month.*

HLH =hemophagocytic lymphohistiocytosis; MAS =macrophage activation syndrome.

REFERENCES

McClain KL, Eckstein O. Clinical features and diagnosis of hemophagocytic lymphohistiocytosis. Up to Date [resource on the Internet]. 2014 [updated 29 October 2018; cited: 17 May 2019]. Available from: https://www.uptodate.com/contents/clinical-features-and-diagnosis-of-hemophagocytic-lymphohistiocytosis.

Ravelli A, Minoia F, Davi S, et al. 2016 classification criteria for macrophage activation syndrome complicating systemic juvenile idiopathic arthritis: a European League Against Rheumatism/American College of Rheumatology/Paediatric Rheumatology International Trials Organisation Collaborative Initiative. Ann Rheum Dis 2016;75:481–9.

“Use of this document is governed by the terms of use on the first page of this document.”

Appendix 12 Schedule of Assessments for Tocilizumab Treatment of Severe or Life-Threatening Cytokine Release Syndrome

Assessment/Procedure	Pre-TCZ Treatment (within 24 hours)	TCZ Administration	Post-TCZ Treatment [c]		
			End of infusion	2 hours	24 hours
TCZ Administration (8 mg/kg)		x			
Vital signs [a]	x [g]		Measure at least every 6 hours until resolution to baseline [g]		
Pressor documentation [b]	x [g]		Record at least every 6 hours until pressors are discontinued [g]		
FiO_2	x [g]		Record at least every 6 hours until patient on room air [g]		
Pulse oximetry, resting	x [g]		Measure at least every 6 hours until resolution to baseline [g]		
Local Laboratory Assessments					
Hematology	x		x	x	x
Liver function tests (AST, ALT, total bilirubin)	x		x	x	x
Serum chemistry and creatinine [d]	x		x	x	x
CRP, LDH, and serum ferritin	x		x	x	x
Coagulation (aPTT, PT/INR, fibrinogen)	x		x	x	x
Infection workup [e]	x				
Central Laboratory Assessments					
Plasma cytokines	x		x	x	x
Plasma IL-6 pharmacodynamic markers [f]	x	x	x	x	x

"Use of this document is governed by the terms of use on the first page of this document."

Appendix 12
Schedule of Assessments for Tocilizumab Treatment of Severe or Life-Threatening Cytokine Release Syndrome (cont.)

aPTT = activated partial thromboplastin time; CRP = C-reactive protein; CRS = cytokine release syndrome; eCRF = electronic Case Report Form; INR = international normalized ratio; IL-6 = interleukin 6; LDH = lactate dehydrogenase; PT = prothrombin time; TCZ = tocilizumab.

Record abnormalities or worsened clinically significant abnormalities on the Adverse Event eCRF.

[a] Includes respiratory rate, heart rate, and systolic and diastolic blood pressure while the patient is in a seated or supine position, and temperature.

[b] Document vasopressor type and dose in the concomitant medication eCRF.

[c] If TCZ dose is repeated, follow Schedule of Assessments following the second TCZ dose.

[d] Includes sodium, potassium, chloride, bicarbonate, glucose and blood urea nitrogen

[e] Includes assessment for bacterial, fungal, and viral infections.

[f] Includes IL-6, and other cytokines such as soluble IL-6R, sgp130, IL-8 etc.

[g] The maximum and minimum values for any 24-hour period should be recorded in the clinical database.

“Use of this document is governed by the terms of use on the first page of this document.”

Appendix 13
Vasopressor Use for CRS

High-Dose Vasopressor [a] (duration ≥3 hours)	
**Pressor**	**Dose**
Norepinephrine monotherapy	≥20 mcg/min
Dopamine monotherapy	≥10 mcg/kg/min
Phenylephrine monotherapy	≥200 mcg/min
Epinephrine monotherapy	≥10 mcg/min
If on vasopressin	Vasopressin + norepinephrine equivalent of ≥10 mcg/min [b]
If on combination vasopressors (not vasopressin)	Norepinephrine equivalent of ≥20 mcg/min [b]

mcg = microgram; min = minute; VASST = Vasopressin and Septic Shock Trial.

Source: Russell et al. N Engl J Med 2008;358:877–88.

[a] High-Dose Vasopressor: as defined in table above (low-dose vasopressor defined as single vasopressor at doses below those shown in table above).

[b] VASST vasopressor equivalent equation: norepinephrine equivalent dose = [norepinephrine (mcg/min)] + [dopamine (mcg/kg/min)] + [phenylephrine (mcg/min) ÷ 10].

"Use of this document is governed by the terms of use on the first page of this document."

Appendix 14
Anaphylaxis Precautions

EQUIPMENT NEEDED

- Oxygen
- Epinephrine for subcutaneous, intravenous, and/or endotracheal use in accordance with standard practice
- Antihistamines
- Corticosteroids
- Intravenous infusion solutions, tubing, catheters, and tape

PROCEDURES

In the event of a suspected anaphylactic reaction during study treatment infusion, the following procedures should be performed:

1. Stop the study treatment infusion.
2. Maintain an adequate airway.
3. Administer antihistamines, epinephrine, or other medications as required by patient status and directed by the physician in charge.
4. Continue to observe the patient and document observations.

“Use of this document is governed by the terms of use on the first page of this document.”

PROTOCOL

**TITLE:**	**AN OPEN-LABEL, MULTICENTER, DOSE ESCALATION AND EXPANSION PHASE Ib STUDY TO EVALUATE THE SAFETY, PHARMACOKINETICS, AND THERAPEUTIC ACTIVITY OF RO6958688 IN COMBINATION WITH ATEZOLIZUMAB IN PATIENTS WITH LOCALLY ADVANCED AND/OR METASTATIC CEA-POSITIVE SOLID TUMORS**
**PROTOCOL NUMBER:**	WP29945
**VERSION:**	11
**EUDRACT NUMBER:**	2015-003771-30
**IND NUMBER:**	122931
**NCT NUMBER:**	NCT02650713
**TEST PRODUCTS:**	RO6958688 and Atezolizumab
**MEDICAL MONITOR:**	[redacted]
**SPONSOR:**	F. Hoffmann-La Roche Ltd
**DATE FINAL:**	Version 1: 18 September 2015
**DATES AMENDED:**	Version 2: 19 February 2016<br>Version 3: 29 April 2016<br>Version 4: 13 December 2016<br>Version 5: 3 August 2017<br>Version 6: 12 October 2017<br>Version 7: 19 January 2018<br>Version 8: 18 April 2018<br>Version 9: 24 September 2018<br>Version 10: 18 May 2019<br>Version 11: See electronic date stamp below.

PROTOCOL AMENDMENT APPROVAL

Date and Time (UTC)	Title	Approver's Name
05-Nov-2019 18:32:00	[redacted]	[redacted]

CONFIDENTIAL

This clinical study is being sponsored globally by F. Hoffmann-La Roche Ltd of Basel, Switzerland. However, it may be implemented in individual countries by Roche's local affiliates, including Genentech, Inc. in the United States. The information contained in this document, especially any unpublished data, is the property of F. Hoffmann-La Roche Ltd (or under its control) and therefore is provided to you in confidence as an investigator, potential investigator, or consultant, for review by you, your staff, and an applicable Ethics Committee or Institutional Review Board. It is understood that this information will not be disclosed to others without written authorization from Roche except to the extent necessary to obtain informed consent from persons to whom the drug may be administered.

"Use of this document is governed by the terms of use on the first page of this document."

PROTOCOL AMENDMENT, VERSION 11:
RATIONALE

Protocol WP29945 has been amended to remove the 120-day sample pharmacokinetic (PK) and anti-drug antibody (ADA) collections and to update atezolizumab safety risks to align with latest Atezolizumab Investigator's Brochure. Changes to the protocol, along with a rationale for each change, are summarized below:

- Background information on atezolizumab has been updated to account for additional approved indications (Section 1.4).
- To align with the Atezolizumab Investigator's Brochure, Version 15, "immune-related" has been changed to "immune-mediated" when describing events associated with atezolizumab (Sections 1.4.2.1, 3.1.2.1, 4.4.2, and 5.2.2 and Appendices 10 and 11).
- To address a request by the French National Agency for the Safety of Medicines and Health Products (ANSM), systemic immune activation has been replaced by hemophagocytic lymphohistiocytosis (HLH) and macrophage activation syndrome (MAS) in the list of potential risks for atezolizumab (Sections 5.2.2, 5.2.4, and 5.2.6.3.5) and the management guidelines for systemic immune activation have been replaced with management guidelines for HLH and MAS (Appendices 10 and 11). In addition, systemic immune activation has been removed from the list of adverse events of special interest (Sections 1.4.2.1, 1.6.2, and 5.1.3).
- It has been clarified that eligibility will be confirmed, not approved, by the Medical Monitor, as approval resides with Principal Investigator (Section 4.2.3).
- The 120-day sample PK and ADA collections have been removed because the results do not change the ADA incidence rate; most of atezolizumab-ADA positivity is transient and occurs around 21 days after the first atezolizumab dose, which makes this sample result uninformative and unnecessary (Sections 4.5.2.3, 4.5.2.4, 4.6.1, and 5.6 and Appendix 1 Tables A1, A2, A4–A9). Nevertheless, as per Section 5.3.1, after initiation of study drugs, all adverse events, regardless of relationship to study drugs, will be reported until 28 days (for RO6958688) or 120 days (for atezolizumab) after the final dose of study drugs or until initiation of new systemic anti-cancer therapy, whichever occurs first.
- To address a request by the French ANSM, the atezolizumab adverse event management guidelines have been revised to add laboratory (e.g., B-type natriuretic peptide) and cardiac imaging abnormalities as signs or symptoms that are suggestive of myocarditis (Appendix 11).

Additional minor changes have been made to improve clarity and consistency. Substantive new information appears in italics. This amendment represents cumulative changes to the original protocol.

“Use of this document is governed by the terms of use on the first page of this document.”

TABLE OF CONTENTS

PROTOCOL AMENDMENT ACCEPTANCE FORM 11

PROTOCOL SYNOPSIS 12

1. BACKGROUND AND RATIONALE 43
1.1 Background on Disease.. 43
1.2 Background on Immunotherapy.. 43
1.3 Background on RO6958688 44
1.3.1 Previous Non-Clinical Studies 46
1.3.2 Previous Clinical Studies 52
1.3.2.1 Safety of RO6958688 52
1.3.2.2 Efficacy of RO6958688.. 56
1.3.2.3 Clinical Pharmacokinetics of RO6958688.. 58
1.3.2.4 Immunogenicity of RO6958688 59
1.4 Background on Atezolizumab 59
1.4.1 Previous Non-Clinical Studies 60
1.4.2 Ongoing Clinical Studies.. 60
1.4.2.1 Safety of Atezolizumab 61
1.4.2.2 Efficacy of Atezolizumab.. 62
1.4.2.3 Clinical Pharmacokinetics and Immunogenicity of Atezolizumab 63
1.5 Background on Tocilizumab (RO4877533, Actemra, RoActemra) 64
1.6 Study Rationale and Benefit–Risk Assessment.. 65
1.6.1 Combination of RO6958688 and Atezolizumab.. 65
1.6.2 Potential for Overlapping Toxicities with RO6958688 and Atezolizumab.. 66
1.6.3 Rationale for [18F] FDG-PET Imaging.. 66
1.6.4 Rationale for the New Dose Schedule.. 66

2. OBJECTIVES.. 67
2.1 Primary Objectives 67
2.2 Secondary Objectives.. 68
2.3 Exploratory Objectives.. 68

“Use of this document is governed by the terms of use on the first page of this document.”

3. STUDY DESIGN ... 69
3.1 Description of Study ... 69
3.1.1 Overview of Study Design ... 70
3.1.2 Dose Escalation Decision Criteria ... 76
3.1.2.1 Escalation Criteria (Part I) – Dose Limiting Toxicities ... 77
3.1.2.2 Expansion Part (Part II) ... 78
3.1.3 Communication Strategy ... 78
3.1.4 End of Study ... 80
3.2 Rationale for Study Design ... 80
3.2.1 Rationale for Dosage Selection ... 80
3.2.1.1 Starting Dose for RO6958688 ... 80
3.2.1.2 Dose of Atezolizumab ... 82
3.2.2 Rationale for the Treatment of Severe Cytokine Release Syndrome (CRS) Using Tocilizumab ... 83
3.2.3 Rationale for Study Population ... 84
3.2.4 Rationale for Biomarker Assessments ... 85
3.2.5 Rationale for Statistical Design ... 87
3.3 Outcome Measures ... 88
3.3.1 Safety Outcome Measures ... 88
3.3.2 Pharmacokinetic (PK) and Pharmacodynamic (PD) Outcome Measures ... 90
3.3.2.1 Pharmacokinetic Outcome Measures ... 90
3.3.2.2 Pharmacodynamic Outcome Measures ... 90
3.3.3 Efficacy Outcome Measures ... 92
3.3.4 Exploratory Outcome Measures ... 94
4. MATERIALS AND METHODS ... 95
4.1 Center ... 95
4.2 Study Population ... 95
4.2.1 Recruitment Procedures ... 96
4.2.2 Inclusion Criteria ... 96
4.2.3 Exclusion Criteria ... 98
4.3 Study Treatments ... 101
4.3.1 Formulation, Packaging, and Handling ... 101

“Use of this document is governed by the terms of use on the first page of this document.”

4.3.1.1 RO6958688 101
4.3.1.2 Atezolizumab 103
4.3.1.3 Tocilizumab 103
4.3.2 Dosage, Administration, and Compliance 104
4.3.2.1 RO6958688 and Atezolizumab 104
4.3.2.2 Administration of Atezolizumab 104
4.3.2.3 Administration of RO6958688 104
4.3.2.4 Administration of Tocilizumab 105
4.3.2.5 Premedication and Prophylactic Treatment for RO6958688 and Atezolizumab 106
4.3.3 Investigational Medicinal Products Accountability 107
4.3.4 Post-Trial Access to RO6958688 and Atezolizumab 108
4.4 Concomitant Therapy 108
4.4.1 Permitted Therapy 108
4.4.2 Prohibited Therapy 110
4.5 Study Assessments 111
4.5.1 Description of Study Assessments 111
4.5.1.1 Medical History and Demographic Data 111
4.5.1.2 Physical Examinations, Vital Signs, and ECOG Performance Status 111
4.5.1.3 Electrocardiograms 112
4.5.1.4 Pulmonary Function Tests (FEV1/VC/TLC and DLCO) 113
4.5.1.5 Laboratory Assessments 113
4.5.1.6 Additional Samples 114
4.5.1.7 Disease-Specific Assessments 119
4.5.1.8 Samples for Roche Clinical Repository 122
4.5.2 Timing of Study Assessments 125
4.5.2.1 Screening and Pretreatment Assessments 125
4.5.2.2 Assessments during Treatment 126
4.5.2.3 Assessments at Study Completion/Early Termination Visit 127
4.5.2.4 Follow-Up Assessments 128

"Use of this document is governed by the terms of use on the first page of this document."

4.5.2.5 Assessments at Unscheduled Visits 129
4.6 Patient, Study, and Site Discontinuation 129
4.6.1 Patient Discontinuation 129
4.6.1.1 Discontinuation from Study Drugs 129
4.6.1.2 Withdrawal from Study 133
4.6.2 Study and Site Discontinuation 133

5. ASSESSMENT OF SAFETY 133
5.1 Safety Parameters and Definitions 133
5.1.1 Adverse Events 134
5.1.2 Serious Adverse Events (Immediately Reportable to the Sponsor) 134
5.1.3 Non-Serious Adverse Events of Special Interest (Immediately Reportable to the Sponsor) 135
5.2 Safety Plan 136
5.2.1 Dose Modifications and Delays 136
5.2.2 Risks Associated with Atezolizumab 139
5.2.3 Risks Associated with RO6958688 139
5.2.4 Risks Associated with Combination Use of RO6958688 and Atezolizumab 140
5.2.5 Risks Associated with Tocilizumab 140
5.2.6 Management of Specific Adverse Events 140
5.2.6.1 Management of Atezolizumab-Specific Adverse Events 140
5.2.6.2 Management of Specific Adverse Events to RO6958688 141
5.2.6.3 Management of Adverse Events Specific to Atezolizumab and to RO6958688 145
5.3 Methods and Timing for Capturing and Assessing Safety Parameters 146
5.3.1 Adverse Event Reporting Period 146
5.3.2 Eliciting Adverse Event Information 147
5.3.3 Assessment of Severity of Adverse Events 147
5.3.4 Assessment of Causality of Adverse Events 148
5.3.5 Procedures for Recording Adverse Events 148

“Use of this document is governed by the terms of use on the first page of this document.”

5.3.5.1 Infusion-Related Reactions/Hypersensitivity Reactions and Cytokine Release Syndrome Attributed to RO6958688 ... 148
5.3.5.2 Other Adverse Events ... 149
5.3.5.3 Adverse Events Occurring Secondary to Other Events ... 149
5.3.5.4 Persistent or Recurrent Adverse Events ... 150
5.3.5.5 Abnormal Laboratory Values ... 150
5.3.5.6 Abnormal Vital Sign Values ... 151
5.3.5.7 Abnormal Liver Function Tests ... 151
5.3.5.8 Deaths ... 151
5.3.5.9 Preexisting Medical Conditions ... 152
5.3.5.10 Lack of Efficacy or Worsening of Cancer ... 152
5.3.5.11 Hospitalization or Prolonged Hospitalization ... 152
5.3.5.12 Cases of Accidental Overdose or Medication Error ... 153
5.4 Immediate Reporting Requirements from Investigator to Sponsor ... 154
5.4.1 Emergency Medical Contacts ... 154
5.4.2 Reporting Requirements for Serious Adverse Events and Non-Serious Adverse Events of Special Interest ... 155
5.4.3 Reporting Requirements for Pregnancies ... 155
5.4.3.1 Pregnancies in Female Patients ... 155
5.4.3.2 Pregnancies in Female Partners of Male Patient ... 155
5.4.3.3 Abortions ... 155
5.4.3.4 Congenital Anomalies/Birth Defects ... 156
5.5 Follow-Up of Patients after Adverse Events ... 156
5.5.1 Investigator Follow-Up ... 156
5.5.2 Sponsor Follow-Up ... 157
5.6 Post-Study Adverse Events ... 157
5.7 Expedited Reporting to Health Authorities, Investigators, Institutional Review Boards, and Ethics Committees ... 157

6. STATISTICAL CONSIDERATIONS AND ANALYSIS PLAN ... 158

“Use of this document is governed by the terms of use on the first page of this document.”

6.1 Primary Study Variables 158
6.2 Secondary Study Variables 158
6.3 Determination of Sample Size 159
6.3.1 Dose-Escalation 159
6.3.2 Dose/Schedule-Finding 160
6.4 Summaries of Conduct of Study 161
6.5 Analysis Populations 161
6.5.1 Safety Analysis Population 161
6.5.2 Pharmacokinetic Analysis Population 161
6.5.3 Pharmacodynamic Analysis Population 161
6.5.4 Efficacy Analysis Population 162
6.6 Summaries of Treatment Group Comparability 162
6.7 Safety Analyses 162
6.7.1 Dose-Escalation Approach 162
6.7.1.1 Modified Continual Reassessment Method with Overdose Control in Part IA 162
6.7.1.2 Intra-Patient Dose Escalation Design 166
6.7.2 Adverse Events 168
6.7.3 Clinical Laboratory Test Results 168
6.7.3.1 Standard Reference Ranges and Transformation of Data 168
6.7.3.2 Definition of Laboratory Abnormalities 168
6.7.4 Vital Signs 169
6.7.5 ECG Data Analysis 169
6.7.6 Concomitant Medications 169
6.8 Efficacy Analyses 169
6.8.1 Primary Efficacy Endpoint 170
6.8.2 Secondary Efficacy Endpoints 170
6.9 Pharmacodynamic Analyses 170
6.10 Pharmacokinetic Analyses 171
6.11 Immunogenicity Analyses 171
6.12 Interim Analyses 172

7. DATA COLLECTION AND MANAGEMENT 172

"Use of this document is governed by the terms of use on the first page of this document."

7.1 Data Quality Assurance 172
7.2 Electronic Case Report Forms 172
7.3 Source Data Documentation 173
7.4 Use of Computerized Systems 173
7.5 Retention of Records 174

8. ETHICAL CONSIDERATIONS 174
8.1 Compliance with Laws and Regulations 174
8.2 Informed Consent 174
8.3 Institutional Review Board or Ethics Committee 175
8.4 Confidentiality 176
8.5 Financial Disclosure 176

9. STUDY DOCUMENTATION, MONITORING, AND ADMINISTRATION 176
9.1 Study Documentation 176
9.2 Site Inspections 177
9.3 Administrative Structure 177
9.4 Publication of Data and Protection of Trade Secrets 177
9.5 Protocol Amendments 178

10. REFERENCES 179

LIST OF TABLES

Table 1 Overall Safety Profile of RO6958688 (Ongoing Study BP29541) 81
Table 2 Pre-Medications to be Administered before RO6958688 Infusion 106
Table 3 Cytokine Release Syndrome Grading According to CTCAE v5 143
Table 4 Adverse Event Severity Grading Scale 147
Table 5 Operating Characteristics of the mCRM with EWOC Design with Respect to the Chosen Scenarios 160
Table 6 Hypothetical Recommendations of the mCRM with EWOC Design 166

“Use of this document is governed by the terms of use on the first page of this document.”

LIST OF FIGURES

Figure 1 Design, Structure, and Characteristics of RO6958688 46
Figure 2 RO6958688-Mediated Lysis of MKN45 Cells............................. 47
Figure 3 RO6958688 Upregulation of PD-1 on T cells, Respective of PD-L1 on Surviving Tumor Cells after Tumor Cell Lysis 48
Figure 4 In Vivo Anti-Tumor Activity upon Combination of RO6958688 with the Anti-Human PD-L1 Blocking Antibody in MKN45 Tumor Model in Fully Humanized Mice....................................... 50
Figure 5 Study Schema.. 71
Figure 6 Conditions for Continuing RO6958688 and/or Atezolizumab in the Presence of Increased Radiographic Tumor Size 132

LIST OF APPENDICES

Appendix 1 Schedule of Assessments... 185
Appendix 2 CKD-EPI equation for Calculation of Glomerular Filtration Rate (GFR) 227
Appendix 3 Eastern Cooperative Oncology Group Performance Status...... 228
Appendix 4 Response Evaluation Criteria in Solid Tumors Version 1.1 Criteria 229
Appendix 5 Modified Response Evaluation Criteria in Solid Tumors 234
Appendix 6 Statistical Design of Modified Continual Reassessment Method with Escalation with Overdose Control......................... 241
Appendix 7 Intrapatient Dose Escalation Design Simulation Results 253
Appendix 8 Gilbert's Syndrome Definition.. 256
Appendix 9 [18F]-FDG-PET.. 257
Appendix 10 Overall Guidelines for Management of Patients Who Experience Adverse Events 258
Appendix 11 Risks Associated with Atezolizumab and Guidelines for Management of Adverse Events Associated with Atezolizumab 273
Appendix 12 Schedule of Assessments for Tocilizumab Treatment of Severe or Life-Threatening Cytokine Release Syndrome......... 298
Appendix 13 Vasopressor Use for CRS... 300
Appendix 14 Anaphylaxis Precautions.. 301

"Use of this document is governed by the terms of use on the first page of this document."

PROTOCOL AMENDMENT ACCEPTANCE FORM

**TITLE:**	**AN OPEN-LABEL, MULTICENTER, DOSE ESCALATION AND EXPANSION PHASE Ib STUDY TO EVALUATE THE SAFETY, PHARMACOKINETICS, AND THERAPEUTIC ACTIVITY OF RO6958688 IN COMBINATION WITH ATEZOLIZUMAB IN PATIENTS WITH LOCALLY ADVANCED AND/OR METASTATIC CEA-POSITIVE SOLID TUMORS**
**PROTOCOL NUMBER:**	WP29945
**VERSION NUMBER:**	11
**EUDRACT NUMBER:**	2015-003771-30
**IND NUMBER:**	122931
**NCT NUMBER:**	NCT02650713
**TEST PRODUCTS:**	RO6958688 and Atezolizumab
**MEDICAL MONITOR:**	[redacted]
**SPONSOR:**	F. Hoffmann-La Roche Ltd

I agree to conduct the study in accordance with the current protocol.

____

Principal Investigator's Name (print)

____ ________________

Principal Investigator's Signature Date

Please keep the signed original form in your study files, and return a copy to your local study monitor.

"Use of this document is governed by the terms of use on the first page of this document."

PROTOCOL SYNOPSIS

**TITLE:**	**AN OPEN-LABEL, MULTICENTER, DOSE ESCALATION AND EXPANSION PHASE Ib STUDY TO EVALUATE THE SAFETY, PHARMACOKINETICS, AND THERAPEUTIC ACTIVITY OF RO6958688 IN COMBINATION WITH ATEZOLIZUMAB IN PATIENTS WITH LOCALLY ADVANCED AND/OR METASTATIC CEA-POSITIVE SOLID TUMORS**
**PROTOCOL NUMBER:**	WP29945
**VERSION NUMBER:**	11
**EUDRACT NUMBER:**	2015-003771-30
**IND NUMBER:**	122931
**NCT NUMBER:**	NCT02650713
**TEST PRODUCTS:**	RO6958688 and Atezolizumab
**PHASE:**	Ib
**INDICATION:**	Patients with locally advanced and/or metastatic CEA-positive solid tumors, whose disease has progressed on or who are intolerant to the standard therapy
**SPONSOR:**	F. Hoffmann-La Roche Ltd

"Use of this document is governed by the terms of use on the first page of this document."

OBJECTIVES

Primary Objectives

The primary objectives of this study are:

- To establish the preliminary safety and tolerability profile of RO6958688 in combination with atezolizumab
- To determine the maximum tolerated dose (MTD) in cycle 1 and in later cycles, if achieved, of RO6958688 in combination with atezolizumab
- To identify a recommended phase II dose and schedule (RP2D) of RO6958688 in combination with atezolizumab

Secondary Objectives

The secondary objectives for this study are:

- To describe the preliminary pharmacodynamic (PD) effects and duration of PD response for RO6958688 in combination with atezolizumab in mandatory paired tumor biopsies and paired blood samples on the basis of alterations in the quantity and quality of intratumoral T cells and peripheral blood cells (including but not limited to $CD3^+$, $CD4^+$, $CD8^+$ T cells, and other immune cells that might act as potential predictors of anti-tumor activity of RO6958688 in combination with atezolizumab)
- To describe the pharmacokinetics (PK) of RO6958688 and atezolizumab when administered in combination
- To obtain preliminary anti-tumor activity data of RO6958688 in combination with atezolizumab based on objective overall response rate (ORR), duration of response (DOR) and derived measures, disease control rate (DCR; defined as response rate [RR] + stable disease rate [SDR]), preliminary progression-free survival (PFS) and preliminary overall survival (OS) according to Response Evaluation Criteria in Solid Tumors (RECIST), Version 1.1 criteria and modified RECIST criteria, by investigator assessment for the whole study and by central assessment for prospective and retrospective analysis
- To estimate the PFS rate at relevant timepoints for RO6958688 in combination with atezolizumab

Exploratory Objectives

The exploratory objectives for this study are:

- To explore the relationship between exposure, pharmacodynamics, metabolic activity of the tumor and clinical effects of RO6958688 when administered in combination with atezolizumab
- To explore the immunogenicity of RO6958688 when administered in combination with atezolizumab
- To explore the relationship of host and tumor genetic factors with PD or clinical response to therapy
- To investigate and define CEA expression in different solid tumors
- To investigate tumor mutations, gene expression and other biomarkers (such as CEA expression in various tumor indications) related to RO6958688 + atezolizumab combination therapy
- To characterize the natural growth of the tumor using tumor growth kinetics modeling
- To explore preliminary safety and efficacy in low/moderate and very low CEA expressing tumors
- To make a preliminary assessment of the effectiveness, PK and PD effects of tocilizumab (Actemra®/RoActemra®) in ameliorating the symptoms of severe CRS following RO6958688 treatment

“Use of this document is governed by the terms of use on the first page of this document.”

STUDY DESIGN

Note: Following an internal review of the clinical development plan of RO6958688, the Sponsor has decided to permanently discontinue further enrollment of patients in this study and to not open the planned cohorts B2, C3, G1, G2, and G3 and the Biomarker cohort as well as Part II of the study.

Description of Study

This is an open-label, multi-center, dose escalation and expansion Phase Ib clinical study of RO6958688 in combination with atezolizumab. Each treatment cycle will be 21 days in duration and consists of IV infusions of RO6958688 given weekly (QW) (± 1 day) or every 3 weeks (Q3W) (± 2 days) in combination with atezolizumab given every 3 weeks (Q3W) (± 2 days).

The initial dose of atezolizumab will be delivered over 60 (± 15) minutes. If the first infusion is tolerated without infusion-related reaction (IRR), the second infusion may be delivered over 30 (± 10) minutes. If the 30-minute infusion is well tolerated, all subsequent infusions may be delivered over 30 (± 10) minutes. RO6958688 should be administered at C1D1 by IV infusion over a minimum of 2 hours, subsequent infusions should be administered in at least 4 hours during the dose/escalation phase. In patients with no grade ≥ 2 IRR/CRS for more than two RO6958688 administrations, the infusion time can be progressively reduced to a minimum of 1 hour. For more details, please refer to the RO6958688 pharmacy manual. At Day 1 of each cycle when atezolizumab and RO6958688 are administered, atezolizumab will be first administered, then RO6958688 will be administered:

- at least half an hour after the end of atezolizumab infusion if no premedication is given prior to RO6958688 administration.
- at least one hour after the end of atezolizumab infusion if premedication is given prior to RO6958688 administration.

During the trial if the clinical pharmacology and/or safety data support an alternate dose sequencing, the above could be modified accordingly.

Patients will be treated until loss of clinical benefit, unacceptable toxicities, loss of RO6958688 exposure (in which case they can continue to receive atezolizumab alone), or withdrawal of consent. The treatment period for this protocol is 24 months for both RO6958688 and atezolizumab and may be modified if emerging data supports an alternative duration of therapy. In case one of the treatments is permanently discontinued, treatment with the other drug alone may be continued as long as the patient experiences clinical benefit in the opinion of the investigator or until unacceptable toxicity or symptomatic deterioration develops, which is attributed to disease progression as determined by the investigator and the Sponsor after an integrated assessment of radiographic data, biopsy results (if available) and clinical status, or withdrawal of consent.

The study will be conducted in two parts. Part I is subdivided into parts IA and IB. Part IA, is a dose escalation part with a starting dose of 5 mg of RO6958688 given QW (once a week) and a fixed, flat dose of 1200 mg given Q3W (every 3 weeks) of atezolizumab, to evaluate the safety and determine the MTD of RO6958688 in combination with atezolizumab. Part IB is a dose/schedule finding part that will explore different administration schedules of RO6958688 in combination with atezolizumab (1200 mg Q3W). Part II is an expansion part to confirm the safety and tolerability of the recommended dose and selected schedule as determined in Part I, in order to define an RP2D and schedule of RO6958688 in combination with atezolizumab, and to explore preliminary antitumor activity, pharmacokinetic and pharmacodynamic effects. Note: Part II has not and will not enroll any patients.

"Use of this document is governed by the terms of use on the first page of this document."

Part IA: Dose Escalation Part

Patients will receive atezolizumab (1200 mg fixed dose) IV on Day 1 of each cycle, followed by RO6958688 given IV on Day 1, Day 8 and Day 15 of each cycle.

Dose escalation of RO6958688 will be pursued according to a modified-Continual Reassessment Method with Overdose Control (mCRM with EWOC) design, aimed at reaching the MTD, which is defined as a dose with 20-30% probability of dose-limiting toxicity (DLT). For RO6958688, the starting dose is 5 mg administered on a QW schedule, to be administered after the administration of 1200 mg of atezolizumab when they are both administered on the same day (Day 1 of each cycle). The RO6958688 dose will be escalated according to the mCRM, but will not exceed the RO6958688 MTD if defined in the BP29541 study. The atezolizumab dose is fixed at 1200 mg and is administered on a Q3W schedule.

Patients within a cohort (at least 3 patients each) will be enrolled in a sequential manner, which, if required, can be expanded with additional patients to acquire additional safety, PK and PD data. Each patient will be observed for 21 days for DLT assessment. Enrollment will be staggered so that the first patient in each cohort will be observed for safety for 1 week (2 weeks in Spain) before additional patients are enrolled in the cohort. Once a minimum of 3 patients have completed the 21-day DLT observation period, the Sponsor and investigators will evaluate and agree on the dose, sequence, and timing for administration of both drugs for the subsequent cohort.

In Part IA (dose escalation), in order to overcome ADA and their impact on PK, optional intra-patient dose escalation of RO6958688 to the next available tolerated dose level may be permitted depending on emerging clinical and safety data at the discretion of the treating physician, and after discussion with the patient. Intra-patient dose escalation may only proceed after patients have tolerated at least the first 3 consecutive doses of RO6958688 after discussion and alignment with the Medical Monitor.

Part IB: Dose/schedule finding part

The first cohort (cohort A) in Part IB will compare the QW vs. Q3W schedules at a flat dose of 100 mg RO6958688 in combination with atezolizumab 1200 mg Q3W. This QW vs. Q3W schedule comparison will enroll approximately 20 to 40 randomized patients per arm.

Part IB will also explore RO6958688 step up dosing schedules in combination with atezolizumab 1200 mg Q3W.

For the step up dosing schedule, the late cycle MTD will be estimated by an intra-patient dose escalation design (see Protocol Section 6.7.1.2) in cohort B1. In this cohort, the RO6958688 dose will be escalated up to 300% of the previous dose until the DLT criteria for that dose level are met. The dose of RO6958688 will not be escalated in the intra patient dose escalation above the monotherapy late cycle MTD of RO6958688 if defined in the BP29541 protocol.

Cohort B1 has enrolled 15 MSS CRC and 2 MSI-H CRC patients. One of the objectives of cohort B1 is to generate initial safety and efficacy data to compare the effect of treatment combination between MSS and MSI-H CRC patients. The second objective of cohort B1 is to define the late cycle MTD for RO6958688 in combination with atezolizumab. Note: No further patients will be enrolled in cohort B1.

The RO6958688 dose at C1D1 will be 40 mg followed by 150 mg at C1D8, 300 mg at C1D15, 600 mg at C2D1, 900 mg at C2D8 and 1200 mg at C2D15 and 1200mg at C3D1 then 1200 mg every 3 weeks (Q3W) thereafter (i.e., C4D1, C5D1…). The Q3W RO6958688 recommended dose will not exceed the late cycle MTD if defined or 1200 mg.

“Use of this document is governed by the terms of use on the first page of this document.”

Cohort B2 will enroll approximately 20 MSS CRC evaluable patients. This new cohort is intended to explore a faster RO6958688 escalation in combination with atezolizumab (Q3W) in order to reach the target dose within the first atezolizumab cycle and prevent a potential early impact of anti-drug antibodies (ADA) on RO6958688 exposure within this first cycle and asses if a more rapid escalation may also increase anti-tumor activity of this combination. Enrollment in Cohort B2 can start at any time after the safety observation period of the 3rd patient in cohort B1 has been completed. Note: No patients have been enrolled into Cohort B2, and this cohort will not be opened for enrollment.

Cohort B1 will be used to define the late cycle MTD or if not defined, to assess 1200 mg as a safe dose.

The RO6958688 dose at C1D1 will be 40 mg followed by 150 mg at C1D8, 600 mg at C1D15, 1200 mg at C2D1 then 1200 mg every 3 weeks (Q3W) thereafter (i.e., C3D1, C4D1…). The Q3W RO6958688 recommended dose will not exceed the late cycle MTD if defined or 1200 mg.

Part IB of the study will also explore two additional RO6958688 step-up dose regimens (cohort C1 and C2) in combination with atezolizumab 1200 mg Q3W, in a randomized schedule comparison expansion. These randomized cohorts will start in parallel to the step up cohort B1.

Enrollment in cohorts B1, C1, and C2 will be staggered, the first patient in each cohort will be observed for safety for 1 week (2 weeks in Spain) before the second patient in each cohort is enrolled. A safety observation period of 1 week will be observed between the second and the third patient in each cohort, and from the third to subsequent patients. In the current study, we have not observed so far significant differences regarding the safety profile in patients with MSI-H tumors when compared to patients with MSS tumors; however since only 4 MSI-H patients have been treated; in cohort B1 the same safety observation period will be applied to the first 3 MSI-H patients.

In the randomized cohorts, RO6958688 will be administered as follows:

Cohort C1: The RO6958688 starting dose will be 40 mg, followed by 100 mg in C1D8, 150 mg in C1D15, 150 mg at C2D1 and 150 mg RO6958688 Q3W thereafter (i.e., C3D1, C4D1…).

Cohort C2: The RO6958688 starting dose will be 40 mg, followed by 150 mg in C1D8, 300 mg in C1D15, 600 mg at C2D1 and 600 mg (or late cycle MTD if defined) RO6958688 Q3W thereafter.

Based on the clinical data from the randomized cohorts (C1 and C2) the sponsor may consider opening a third cohort (cohort C3) with a starting dose of RO6958688 at 100 mg with mandatory prophylactic corticosteroids post dose at C1D1 (as detailed in Table 3), followed by 150 mg in C1D8, 300 mg in C1D15, and 600 mg at C2D1 and 600 mg (or late cycle MTD if defined) RO6958688 Q3W thereafter (i.e., C3D1, C4D1…). Note: No patients have been enrolled into Cohort C3, and this cohort will not be opened for enrollment.

The Q3W RO6958688 recommended dose for cohorts C1, C2 and the optional C3 cohort defined above, will not exceed the late cycle MTD if defined.

Each of the cohorts C1-2 and the optional cohort C3 will consist of approximately 40 locally advanced or metastatic (as defined by eligibility criteria #3) microsatellite stable (MSS) colorectal cancer patients. Approximately 80 patients will be randomized 1:1 to cohorts C1-2. Note: 39 CRC

“Use of this document is governed by the terms of use on the first page of this document.”

patients were enrolled in cohort C1, and 35 CRC patients were enrolled in cohort C2. No further patients will be enrolled into these cohorts, and no patients have or will be enrolled in cohort C3.

Additional safety cohorts in other solid tumor, including NSCLC, gastric, pancreatic and breast cancer will be explored (without formal estimation of maximum tolerated doses). These cohorts can be opened after the safety observation period (1 week) of the third patient in cohorts B1 or C has been completed. Note: 14 patients were enrolled in the pancreatic cohort, 6 patients were enrolled in the gastric cohort and 2 patients were enrolled in the breast cohort. No patients were enrolled in the NSCLC cohort. No further patients will be enrolled in the safety cohorts.

These safety cohorts will explore RO6958688 administered weekly (QW) at an initial dose of 40 mg followed by 150 mg in C1D8, 300 mg in C1D15, 600 mg at C2D1 and 600 mg (or MTD if defined) Q3W thereafter. Atezolizumab will be administered Q3W (1200 mg). Regarding lung cancer patients an alternative RO6958688 dose/schedule can be explored if supported by data (i.e., 40 mg at C1D1 followed by 100 mg at C1D8, 150 mg at C1D15, 300 mg at C2D1 and 600 mg every 3 weeks).

Finally, once the safety observation period of the third patient in cohort B1 has been completed, the sponsor may explore the safety and preliminary efficacy of RO6958688 in combination with atezolizumab in a separate biomarker cohort of approximately 20 patients with very low/negative CEA expressing solid tumors. Very low/negative CEA expression is defined as those samples having $<$ 20% of tumor cells with IHC1+or IHC0+. Patients enrolled in the biomarker cohort (very low/negative CEA expression) will follow the same dose/schedule as patients in cohort C1. Note: No patients have been enrolled into the biomarker cohort, and this cohort will not be opened for enrollment.

Once the late cycle MTD has been defined (see Protocol Section 6.7.1.2) or 1200 mg is declared safe in cohort B1, ongoing patients in the trial experiencing clinical benefit and showing >50% reduction of RO6958688 C_{max} and a PK profile consistent with ADA mediated decrease in exposure, can be progressively dose escalated up to the late cycle MTD or 1200 mg of RO6958688 after discussion with the Medical Monitor. However, the above optional dose escalation does not apply to patients enrolled in cohort C1 since this cohort has been designed to assess a potential biological dose.

The sponsor may consider opening additional obinutuzumab cohorts (G1 to G3). Patients participating in these cohorts will receive according to patient's and/or investigators convenience, either 2000 mg of obinutuzumab IV on Day-7 (+ 1 day) or 1000 mg of obinutuzumab IV on two consecutive days, Day-8 and Day-7 (+ 1 day) before C1D1 RO6958688 and atezolizumab administrations. Premedication will be given prior to each obinutuzumab dosing. For these patients, the baseline tumor biopsy will be taken before receiving the first dose of obinutuzumab and the on-treatment tumor biopsy remains unchanged. Note: No patients have been enrolled into the obinutuzumab cohorts, and these cohorts will not be opened for enrollment.

Cohort G1 (MSS CRC): approximately 40 patients with locally advanced or metastatic (as defined by eligibility criteria #3) microsatellite stable (MSS) colorectal cancer patients will be enrolled. The RO6958688 starting dose will be 40 mg, followed by 100 mg on C1D8, 150 mg on C1D15 and 150 mg RO6958688 Q3W thereafter (i.e., C3D1, C4D1…) in combination with 1200 mg atezolizumab Q3W.

“Use of this document is governed by the terms of use on the first page of this document.”

Based on preliminary efficacy and safety data from Cohort G1 the sponsor may consider opening additional Cohorts for patients with gastric, pancreatic and other indications:

Cohort G2: approximately 40 patients with locally advanced or metastatic Gastric Cancer (as defined by eligibility criteria #3) will be enrolled.

The RO6958688 starting dose will be 40 mg, followed by 100 mg on C1D8, 150 mg on C1D15 and 150 mg RO6958688 Q3W thereafter (i.e., C3D1, C4D1…) in combination with 1200 mg atezolizumab Q3W.

Cohort G3: approximately 40 patents with locally advanced or metastatic Pancreatic Cancer (as defined by eligibility criteria #3) will be enrolled. The RO6958688 starting dose will be 40 mg, followed by 100 mg on C1D8, 150 mg on C1D15 and 150 mg RO6958688 Q3W thereafter (i.e., C3D1, C4D1…) in combination with 1200 mg atezolizumab Q3W.

Enrollment in Cohorts G1-G3 will be independently staggered, the first patient will be observed for safety for 1 week (2 weeks in Spain) before the second patient in each cohort is enrolled. A safety observation period of 1 week will be observed between the second and the third patient in each cohort, and from the third to subsequent patients.

For all patients who enroll in the study, tumor biopsies for PD analysis are mandatory at baseline and on-treatment, except for NSCLC patients for whom there is no accessible lesion. For patients continuing in the study additional biopsies may be taken at the discretion of the investigator. These biopsies may serve to evaluate viable tumor given the mechanism of action of RO6958688 to induce tumor inflammation. For patients discontinuing from the study due to disease progression, additional optional biopsies if clinically feasible, may be taken to aid the understanding of immune resistance mechanisms.

Part II: Expansion Part

Once the recommended dose and schedule have been determined, additional patients (approximately 15 patients) with solid tumors with high CEA expression may be enrolled in this study. The purpose is to confirm the safety and tolerability of the recommended dose and schedule as determined in Part I and to explore preliminary antitumor activity, pharmacokinetic and pharmacodynamic effects. Note: No patients have been enrolled in Part II of the study, and Part II will not be opened for enrollment.

Baseline and on-treatment tumor biopsies will be mandatory for all patients, except for NSCLC patients for whom there is no accessible lesion. DLT data will still be collected and might lead to refinement of the MTD definition for RO6958688 in combination with atezolizumab at the end of the trial.

Patients who discontinue both study drugs prior to the first on-treatment tumor assessment due to toxicity will not be considered evaluable for response and will be replaced. Patients who discontinue treatment due to clinical progression will remain evaluable.

Dose Limiting Toxicities (DLTs)

At least 3 patients will be enrolled in each cohort; additional patients may be enrolled to study safety, PK, or PD in more detail. Patients in a cohort will be enrolled in a sequential manner. The first patient in each cohort will be observed for safety for 1 week (2 weeks in Spain) prior to enrollment of additional patients in that cohort. During the dose escalation part (Part IA), patients who discontinue treatment before the end of the DLT period, for reasons other than DLTs, and patients who did not receive the assigned dose of RO6958688 (3 doses QW for the QW regimen)

“Use of this document is governed by the terms of use on the first page of this document.”

or atezolizumab (1 dose Q3W) during the DLT period, will be replaced to ensure that at least 3 patients in each cohort have been assessed for the full DLT period of 21 days prior to moving to the next dose level. During the step up dose escalation (Part IB), patients who discontinue treatment for reasons other than DLTs or receiving the 1200 mg dose or the highest possible safe dose according to the intra-patient dose escalation rules (the lower one applies), may be replaced in order to ensure that at least 6 evaluable patients will be available for estimating the late cycle MTD.

A DLT is defined as one of the following toxicities that occur during the DLT assessment period and is considered by the investigator to be related to RO6958688, atezolizumab, or the combination of both products. For potential overlapping toxicities, investigators are encouraged to perform additional tests to determine the underlying etiology and most appropriate attribution. Dose reductions or delays will not, in themselves, constitute DLTs.

The following adverse events are considered DLTs:

Hematological toxicities defined as:

- Grade ≥ 4 neutropenia (ANC < 500/μL) lasting ≥ 7 days
- Grade ≥ 3 febrile neutropenia
- Grade ≥ 4 thrombocytopenia lasting > 48 hours (recovery to ≤ Grade 2)
- Grade 3 or 4 thrombocytopenia associated with bleeding episodes

Any non-hematological toxicity ≥ Grade 3 including:

- Grade 3 hyperbilirubinemia lasting for > 48 hours or Grade 4
- Grade ≥ 3 AST/ALT elevations with hyperbilirubinemia of ≥ Grade 2
- Grade 4 AST/ALT elevations
- For patients with Grade 2 AST, ALT, and/or alkaline phosphatase abnormality at baseline, an increase to ≥ 10 × the upper limit of normal (ULN) that does not resolve to Grade ≤ 2 within 48 hours (if symptomatic) or that does not resolve to Grade ≤ 1 within 3 weeks of onset (if asymptomatic)

Failure to recover from any drug-related toxicity that results in a dose delay of ≥ 21 days (1 Cycle) is defined as a DLT.

The following are not considered DLTs:

- Grade 3 nausea, vomiting, diarrhea, colitis or enteritis that resolves to Grade ≤ 2 with or without treatment prior to the next planned infusion of RO6958688 (1 week)
- Grade 3 *immune-mediated* adverse event that resolves to Grade ≤ 1 with immunosuppressant therapy within 3 weeks of its onset
- Grade ≥ 3 fatigue that resolves to Grade ≤ 2 within 1 week
- Grade 3 arthralgia that can be adequately managed with supportive care or that resolves to Grade ≤ 2 within 1 week
- Fever > 40 degrees Celsius that occurs within 72 hours of RO6958688 infusion and resolves to ≤ Grade 2 within 4 days and is resolved to Grade ≤ 1 within 10 days

“Use of this document is governed by the terms of use on the first page of this document.”

- Fever ≥ Grade 3 that resolves to Grade ≤ 2 within 72 hours
- Grade 3 hypophosphatemia reversible to < Grade 2 within 1 week
- Grade ≥ 3 laboratory abnormality that is asymptomatic and deemed by the investigator not to be clinically significant
- Grade 3 autoimmune thyroiditis or other endocrine abnormality that can be managed by endocrine therapy or hormonal replacement
- Grade 3 tumor flare defined as local pain, irritation, or rash localized at sites of known or suspected tumor
- Alopecia (any grade)
- Grade 3 tumor pain that starts within 24 hours of infusion and resolves to Grade ≤ 2 within 1 week
- Grade 3 hypoxia that starts within 24 hours of infusion and resolves to Grade ≤ 2 within 1 week
- In patients with lung lesions, Grade 3 transient dyspnea secondary to localized lung edema that starts within 24 hours of infusion and recovers to Grade ≤ 2 or baseline within 1 week, and transient bronchospasm that resolves within 24 hours
- In patients with liver lesions, Grade 3 transient increase of bilirubin, transaminases and/ or Gamma GT that starts after infusion and recovers to Grade ≤ 2 or baseline within 1 week and grade 4 AST/ALT or grade 4 bilirubin increase that start after infusion and recovers to grade ≤2 or baseline within 3 days.

Infusion related reactions (IRRs): IRRs are not considered to be DLTs because based on experience with monoclonal antibodies, IRRs are not dose-related events. Precautions will be taken if IRRs Grade ≥ 2 occur. If described precautions are not sufficient, other options will be discussed between the Sponsor and investigators.

NUMBER OF PATIENTS

Overall, this study has enrolled 228 patients in the dose escalation and dose/schedule finding part. No further enrollment will take place.

TARGET POPULATION

Part I and Part II of the trial will enroll patients with locally advanced and/or metastatic CEA–positive solid tumors who have progressed on standard treatment, are intolerant to standard treatment, and/or are non-amenable to standard therapy.

Based on the preliminary safety and efficacy data from Part I, the Sponsor could focus on other specific CEA expressing tumor indications for which to enroll a certain number of patients in Part II.

"Use of this document is governed by the terms of use on the first page of this document."

INCLUSION/EXCLUSION CRITERIA

Inclusion criteria:

Patients must meet the following criteria for study entry:

1. Signed informed consent
2. Age ≥ 18 years
3. Confirmed locally advanced and/or metastatic solid tumor, with at least one tumor lesion of accessible non-critical location to biopsy, in patients who have progressed on a standard therapy, are intolerant to standard therapy, and/or are non-amenable to standard therapy
4. Radiologically measurable and clinically evaluable disease (as per RECIST v1.1 - previously irradiated lesions should not be counted as target lesions)
5. Life expectancy (in the opinion of the investigator) of ≥ 12 weeks and LDH levels ≤ 2.5 ULN
6. Eastern Cooperative Oncology Group (ECOG) Performance Status (PS) 0–1
7. All acute toxic effects of any prior radiotherapy, chemotherapy, or surgical procedure must have resolved to Grade ≤ 1 or returned to baseline except alopecia (any grade) and Grade 2 peripheral neuropathy
8. Adequate hematological function (without transfusion within 2 weeks prior to Cycle 1, Day 1): neutrophil count of ≥ 1.5 × 10^9 cells/L and hemoglobin ≥ 9 g/dL (5.5 mmol/L), and lymphocytes within normal limits (≥ 0.8 × 10^9 cells/L), platelet count of ≥ 100,000/μL; INR and aPTT ≤ 1.5 × ULN. This applies only to patients who are not receiving therapeutic anticoagulation; patients receiving therapeutic anticoagulation should be on a stable dose. Genetic deficiencies affecting aPTT are not excluded unless assessed as clinically significant by the site hematologist.
9. Adequate liver function: total bilirubin ≤ 1.5 × the upper limit of normal (ULN; excluding Gilbert's Syndrome, when < 3 × ULN is allowed), AST and/or ALT ≤ 2.5 × ULN (in case of liver metastases, ≤ 5 × ULN); Patients with documented liver metastases: alkaline phosphatase ≤ 5 × ULN. For patients with bone disease, this criterion should only take into consideration the specific liver isoform of alkaline phosphatase (not the total since it is influenced by the bone isoform).
10. Adequate renal function: Creatinine clearance ≥ 60 ml/min calculated by CKD-EPI equation (see Appendix 2)
11. Negative serum pregnancy test within 7 days prior to study treatment in premenopausal women and women ≤ 2 years after start of menopause (menopause is defined as amenorrhea for > 2 years)
12. For women of childbearing potential and female partners of male patients: agreement to remain abstinent (refrain from heterosexual intercourse) or use contraceptive methods that result in a failure rate of < 1% per year during the treatment period and for at least 5 months after the last dose of RO6958688 and atezolizumab and 2 months after the last dose of tocilizumab (if applicable).

    For male participants, patient must agree to either remain completely abstinent or to use a condom and not donate sperm during the entire study period and for 3 months after the

"Use of this document is governed by the terms of use on the first page of this document."

last administration of RO6958688 and for 2 months after the last dose of tocilizumab (if applicable). Male patients who have received only atezolizumab are not required to use contraception during atezolizumab treatment; pregnancies in female partners of male patients receiving atezolizumab monotherapy are not required to be reported.

A woman is considered to be of childbearing potential if she is postmenarcheal, has not reached a postmenopausal state (> 2 years of amenorrhea with no identified cause other than menopause), and has not undergone surgical sterilization (removal of ovaries and/or uterus).

Examples of contraceptive methods with an expected failure rate of < 1% per year include bilateral tubal ligation, male sterilization, established, proper use of hormonal contraceptives that inhibit ovulation, hormone-releasing intrauterine devices, and copper intrauterine devices.

The reliability of sexual abstinence should be evaluated in relation to the duration of the clinical trial and the preferred and usual lifestyle of the patient. Periodic abstinence (e.g., calendar, ovulation, symptothermal, or postovulation methods) and withdrawal are not acceptable methods of contraception.

13. Patients with non-colorectal cancer should have confirmed CEA expression in tumor tissue (≥ 20% of tumor cells staining with at least moderate to high intensity of both cytoplasmic and/or membranous (IHC 2+ and IHC 3+). CEA expression should be centrally confirmed for U.S. and Canada patients.

    For CRC cancer patients, the CEA assessment should be performed but the result is not required for patient selection. If no archival tumor tissue is available, fresh biopsy will be collected.

14. For the biomarker cohort (in Part IB), patients should have very low/negative CEA expression. Very low/negative CEA expression is defined as tumor samples having < 20% of tumor cells with IHC1+ or IHC 0+. CEA should be determined prior to enrollment, if no archival tumor is available, a fresh biopsy will be collected.

Exclusion criteria:

Patients who meet any of the following criteria will be excluded from study entry:

1. Active or untreated central nervous system (CNS) metastases as determined by CT or MRI evaluation during screening and prior radiographic assessments

    Patients with a history of treated asymptomatic CNS metastases are eligible, provided they meet all of the following criteria:

    - No metastases to brain stem, midbrain, pons, medulla, cerebellum, or within 10 mm of the optic apparatus (optic nerves and chiasm)
    - Radiographic demonstration of improvement upon the completion of CNS-directed therapy and no evidence of interim progression between the completion of CNS-directed therapy and the screening radiographic study
    - No history of intracranial hemorrhage or spinal cord hemorrhage
    - No ongoing requirement for dexamethasone as therapy for CNS disease; anticonvulsants at a stable dose allowed

“Use of this document is governed by the terms of use on the first page of this document.”

- No stereotactic radiation or whole-brain radiation within 28 days prior to Cycle 1 Day 1
- Screening CNS radiographic study ≥ 4 weeks since completion of radiotherapy and ≥ 2 weeks since discontinuation of corticosteroids.

2. Spinal cord compression not definitively treated with surgery and/or radiation or previously diagnosed and treated spinal cord compression without evidence that disease has been clinically stable for ≥ 2 weeks prior to enrollment.
3. Leptomeningeal disease.
4. Patients with paraspinal, paratracheal and mediastinal pathological lesions larger than 2 cm unless they are previously irradiated. Irradiation of lesions must be completed at least 14 days prior to initiation of study treatment.
5. Malignancies within 5 years prior to enrollment, with the exception of those with a negligible risk of metastasis or death and treated with expected curative outcome (such as adequately treated carcinoma in situ of the cervix, basal or squamous cell skin cancer, localized prostate cancer treated surgically with curative intent, ductal carcinoma in situ treated surgically with curative intent).
6. Significant, uncontrolled concomitant diseases which could affect compliance with the protocol or interpretation of results, including diabetes mellitus, pulmonary disorders, and known autoimmune diseases.
7. Uncontrolled hypertension (systolic blood pressure (BP) > 150 mmHg and/or diastolic BP > 100 mmHg), unstable angina, congestive heart failure (CHF) of any New York Heart Association (NYHA) classification (Class II or greater), serious cardiac arrhythmia requiring treatment (exceptions: atrial fibrillation, paroxysmal supraventricular tachycardia), history of myocardial infarction within 6 months of enrollment.
8. Administration of a live, attenuated vaccine within 28 days before Cycle 1 Day 1 or anticipation that such a live attenuated vaccine will be required during the study. Influenza vaccination should be given during influenza season only (approximately October to March). Patients must not receive live, attenuated influenza vaccine (e.g., FluMist®) within 4 weeks prior to Cycle 1 Day 1 or at any time during the study.
9. Known HIV.
10. Active Hepatitis B (HBV) or Hepatitis C (HCV) infection (required at screening):
    - Patients with active hepatitis B (defined as having a positive hepatitis B surface antigen [HBsAg] test at screening)
        - Patients with past HBV infection or resolved HBV infection (defined as having a negative HBsAg test and a positive antibody to hepatitis B core antigen [anti-HBc] antibody test) are eligible.
    - Patients with active hepatitis C
        - Patients positive for HCV antibody are eligible only if PCR is negative for HCV RNA.

"Use of this document is governed by the terms of use on the first page of this document."

11. Severe infections within 28 days prior to Cycle 1 Day 1, including but not limited to hospitalization for complications of infection, bacteremia, or severe pneumonia.
12. Received oral or intravenous (IV) antibiotics within 14 days prior to Cycle 1 Day 1. Patients receiving prophylactic antibiotics (e.g., for prevention of a urinary tract infection) are eligible.
13. Any other diseases, metabolic dysfunction, physical examination finding, or clinical laboratory finding giving reasonable suspicion of a disease or condition that would contraindicate the use of an investigational drug.
14. Major surgery or significant traumatic injury < 28 days prior to Cycle 1 Day 1 (excluding biopsies) or anticipation of the need for major surgery during study treatment.
15. Dementia or altered mental status that would prohibit informed consent.
16. Known history of autoimmune disease, including but not limited to myasthenia gravis, myositis, autoimmune hepatitis, systemic lupus erythematosus, rheumatoid arthritis, inflammatory bowel disease, vascular thrombosis associated with antiphospholipid syndrome, Wegener's granulomatosis, Sjögren's syndrome, Bell's palsy, Guillain-Barré syndrome, multiple sclerosis, vasculitis, or glomerulonephritis.

    The protocol allows:

    - Patients with a history of autoimmune hypothyroidism on a stable dose of thyroid replacement hormone
    - Patients with Type 1 diabetes mellitus on appropriate medical management may be considered for this study *after consultation with* the Medical Monitor
    - Patients with positive serology of auto-antibody panel (anti-nuclear antibody, anti-double stranded DNA, cytoplasmic anti-neutrophil cytoplasmic antibody [c-ANCA], and perinuclear anti-neutrophil cytoplasmic antibody [p-ANCA]) at screening should be referred to a specialist (i.e., Rheumatologist) for further assessments if the Investigator, after discussion with the Medical Monitor, considers the results as clinically significant.
17. History of idiopathic pulmonary fibrosis, pneumonitis (including drug induced), organizing pneumonia (i.e., bronchiolitis obliterans, cryptogenic organizing pneumonia, etc.), or evidence of active pneumonitis (including drug induced) on screening chest CT scan. History of radiation pneumonitis in the radiation field (fibrosis) is permitted.
18. Patients with bilateral lung lesions and dyspnea and/or SaO2 <92% (at rest, room air and exertion) or patients with lobectomy or pneumonectomy with lung metastases in the remaining lung and either dyspnea or SaO2 <92% (at rest, room air and exertion) at baseline.
19. Baseline QTc interval of > 470 ms, baseline resting bradycardia < 45 beats per minute, or baseline resting tachycardia > 100 beats per minute.
20. Pregnant or breast-feeding women.
21. Known hypersensitivity to any of the components of RO6958688 and atezolizumab; hypersensitivity to Chinese hamster ovary cell products or other recombinant human antibodies.

"Use of this document is governed by the terms of use on the first page of this document."

22. Investigational therapy (defined as treatment for which there is no regulatory authority approved indication) or last dose of prior immunotherapies including but not limited to: interferon alpha (IFN-α), interferon-beta (IFN-β), IL-2, conjugated IL-2, CEA-IL2v, cytokines, anti-CTLA4, within 28 days prior to Cycle 1 Day 1. Patients previously treated with anti-programmed death-ligand 1 (PD-L1), or anti-PD-1 are excluded.

23. Any approved anti-cancer therapy, including chemotherapy or hormonal therapy, within 28 days prior to Cycle 1 Day 1, with the following exceptions:
    - Hormone-replacement therapy or oral contraceptives
    - Tyrosine kinase inhibitors (TKIs) (both small molecules and antiangiogenic monoclonal antibodies) that have been discontinued > 21 days prior to Cycle 1 Day 1; baseline scans must be obtained after discontinuation of prior TKIs.

24. Prior systemic corticosteroids > 10 mg prednisone (or equivalent) within 14 days of Cycle 1 Day 1. The use of inhaled and/or topical corticosteroids and mineralocorticoids (e.g., fludrocortisone) is allowed.

25. Last dose with any of the following agents including but not limited to: etanercept, infliximab, tacrolimus, cyclosporine, mycophenolic acid, alefacept, or efalizumab < 28 days prior to first dose of study drugs.

26. Regular immunosuppressive therapy (i.e., for organ transplantation, chronic rheumatologic disease).

27. Patients with prior allogeneic bone marrow transplantation or prior solid organ transplantation.

28. Treatment with systemic immunosuppressive medications including, but not limited to: corticosteroids, cyclophosphamide, azathioprine, methotrexate, thalidomide, and anti-TNF agents within 14 days prior to Cycle 1, Day 1. Patients who have received acute and/or low-dose systemic immunosuppressant medications (e.g., a one-time dose of dexamethasone for nausea or chronic use of ≤ 10 mg/day of prednisone or dose-equivalent corticosteroid) may be enrolled in the study after discussion with the Medical Monitor. The use of inhaled corticosteroids and mineralocorticoids (e.g., fludrocortisone) for patients is allowed.

29. Radiotherapy within the last 28 days before Cycle 1 Day 1 with the exception of limited field palliative radiotherapy e.g., for bone pain relief.

LENGTH OF STUDY

The maximum treatment period for this study is 24 months for both RO6958688 and atezolizumab and may be modified if emerging data suggest a different treatment period. Patients will be treated until lack of clinical benefit, unacceptable toxicities, or withdrawal from treatment for other reasons or death. All patients will attend a safety follow-up visit 28 days (± 2 days) after receiving the last infusion of RO6958688 or atezolizumab.

The total study duration is estimated to be 60 months.

"Use of this document is governed by the terms of use on the first page of this document."

END OF STUDY

The study will formally end once all patients have completed the safety follow-up visit, withdrawn from the study or when all patients have been enrolled in an extension study, whichever occurs last (the option to enroll in an extension study is for patients receiving atezolizumab only).
The Sponsor may also decide to terminate the study at any time.

OUTCOME MEASURES

SAFETY OUTCOME MEASURES

The safety outcome measures for this study are:

- Incidence and nature of DLTs
- Incidence and severity of adverse events and IRRs and CRS symptoms
- Incidence of laboratory abnormalities (as examples and not limited to: hematology testing, coagulation, serum chemistries, and urinalysis)
- Incidence of ADAs (anti-atezolizumab antibodies and anti-RO6958688 antibodies) formation, detection of cytokine release and potential correlation with PK, PD, safety, and efficacy parameters
- Incidence of autoantibodies (anti-nuclear antibody, anti-double-stranded DNA, cytoplasmic anti-neutrophil cytoplasmic antibody, and perinuclear anti-neutrophil cytoplasmic antibody) in comparison to baseline
- Changes in vital signs, physical findings and ECG findings

All patients who participate in the study will be clinically evaluated at screening and on a regular basis during the entire course of the study. The routine safety monitoring plan will include clinical examination, vital signs assessment (body temperature, respiratory rate, heart rate, diastolic and systolic BP, oxygen saturation measured by pulse oximeter – finger clip), ECG, laboratory analysis (hematology, coagulation, biochemistry, urinalysis, presence of autoantibodies), and regular collection and review of the reported adverse events.

The National Cancer Institute Common Terminology Criteria for Adverse Events v4.03 will be used to evaluate the clinical safety of the treatment in this study (https://evs.nci.nih.gov/ftp1/CTCAE/CTCAE_4.03/CTCAE_4.03_2010-06-14_QuickReference_8.5x11.pdf). However, CRS adverse events considered related to RO6958688 will be evaluated using NCI CTCAE v5 (Table 3). Patients will be assessed for adverse events at each clinical visit and as necessary throughout the study.

Safety will be determined, but not limited to, by the spontaneous reporting of adverse events; by the assessments of routine laboratory values (hematology testing, serum chemistries); findings on physical examinations; ECGs; chest X-ray; vital signs; by carefully observing patients for IRRs; by the determination of ADAs.

Please refer to schedule of assessments for details on collection time of the assessments outlined below.

Laboratory Tests

Hematology and biochemistry will be analyzed at least prior to RO6958688 and atezolizumab administration as part of the regular safety assessments.

1. Hematology: erythrocytes, hemoglobin, hematocrit, platelets, leucocytes and differential count (i.e., neutrophils, eosinophils, basophils, monocytes, lymphocytes).
2. Coagulation: prothrombin time (PT)/international normalized ratio (INR) and partial thromboplastin time (PTT) and fibrinogen. Additional coagulation parameters (i.e., first chromogenic antithrombin III then antigenic antithrombin III in case of chromogenic antithrombin III decrease in order to determine type 1 and type 2 antithrombin

“Use of this document is governed by the terms of use on the first page of this document.”

deficiencies. If a deficiency is detected, both antithrombin tests will need to be repeated at a later date/visit to confirm test findings, fibrinogen, prothrombin time, fibrin degradation products, D-dimer) could be assessed according to clinical judgment, or if any of the above parameter cannot be assessed locally.

3. Blood biochemistry: sodium, potassium, chloride, calcium, phosphate, magnesium, urea, creatinine, normal glomerular filtration rate (by CKD-EPI equation), total protein, albumin, glucose, total and direct bilirubin, alkaline phosphatase (ALP), alanine aminotransferase (ALT), aspartate aminotransferase (AST), lactate dehydrogenase (LDH), γ-glutamyl transferase (GGT), C reactive protein (CRP)., NS cholesterol (total, LDL cholesterol, HDL cholesterol), triglycerides, Thyroid-stimulating hormone (TSH), ferritin, soluble CD25.

   Soluble CEA (sCEA) measured in serum or plasma, will also be measured as a disease monitoring marker.
4. Urinalysis: dipstick for pH, glucose, blood, protein, ketones, and bilirubin. If there is a clinically significant positive result (i.e., confirmed by a positive repeated sample), urine will be sent to the laboratory for microscopy and culture. If there is an explanation for the positive dipstick result, e.g., menses, it should be recorded, and there is no need to perform laboratory for microscopy and culture.
5. HBV and HCV screening tests at screening.
6. All women of childbearing potential (including those who have had a tubal ligation) will have a serum pregnancy test at screening, within 7 days of first dose, on a regular basis during the treatment period (urine) and at the follow up visit.
7. Autoantibodies panel: anti-nuclear antibody, anti-double stranded DNA, cytoplasmic anti-neutrophil cytoplasmic antibody [c-ANCA], and perinuclear anti-neutrophil cytoplasmic antibody [p-ANCA]

Unscheduled hematology, biochemistry, and coagulation assay will be obtained in patients who develop infusion–related reactions (IRRs) and as clinically indicated.

Additional safety laboratory assessments:

- ADA
- Cytokine release assessment
- IgE and tryptase for patients who experience a Grade ≥2 IRR, see Schedule of assessments – Table A3.

PHARMACOKINETIC OUTCOME MEASURES

Pharmacokinetic (PK) concentration data of RO6958688 and atezolizumab will be summarized with the use of descriptive statistical methods. PK parameters (area under the concentration curve [AUC], volume of distribution at steady state [V_{ss}], minimum and maximum serum concentration [C_{min} and C_{max}], clearance [CL], and half-life (t1/2) (terminal and effective)) of RO6958688 and atezolizumab will be estimated using non-compartmental analysis (NCA) methods when applicable.

PHARMACODYNAMIC OUTCOME MEASURES

The PD outcome measures for this study are:

- **Whole blood samples:** Peripheral blood immune cells will be assessed with respect to the changes in the characteristics of lineage (CD4+ T cells, CD8+ T cells, natural killer [NK] cells, monocytes, T-regulatory cells, and B cells), activation (including but not limited to CD25, CD69, etc.), and differentiation (including but not limited to CD45RO Ki67, PD1, TIM3, ICOS, etc.). In addition, whole blood samples will be taken for TCR Vβ sequencing

“Use of this document is governed by the terms of use on the first page of this document.”

(the CDR3-TCR beta chain repertoire). The DNA will be used to determine the immune repertoires of peripheral T cell receptor (TCR) Vβ CDR3 and analyze TCR diversity.

- **Serum or plasma samples:** PD biomarkers such as cytokines and inflammation markers (including but not limited to tumor necrosis factor-α (TNFα), interferon-γ (INFγ), interleukin (IL)-6, MIP, etc.) will be analyzed. Because these measurements are also safety measure assessments during any IRRs, they will also be examined in patients enrolled in both Part I and Part II of the study. Disease-monitoring markers that include but are not limited to sCEA will also be assessed.
- **Tumor biopsy:** Tumor biopsy samples will be obtained from all patients enrolled. These paired tumor biopsies for PD analyses are mandatory at baseline and on-treatment, except for NSCLC patients for whom there is no accessible lesion. If feasible, biopsies may be repeated if the initial biopsy did not contain sufficient tumor material for analysis. For patients discontinuing from the study due to disease progression, additional optional biopsies, if clinically feasible, may be taken to aid the understanding of immune resistance mechanisms. Biopsies will be assessed centrally for changes in immune cell numbers and activation characteristics as well as changes in tumor markers such as PD-L1. These analyses will be performed by flow cytometric molecular and/or immunohistochemistry methods with respect to changes in the characteristics of lineage (CD4+ T cells, CD8+ T cells, NK cells, monocytes, T-regulatory cells, and B cells), activation (including but not limited to CD25, CD69, etc.), differentiation (including but not limited to CD45RO Ki67, PD1, TIM3, ICOS, etc.), and TCR Vβ repertoire and tumor mutational load.
- **Positron Emission Tomography (PET):** Baseline and on-treatment 2-[18F]Fluoro-2-deoxyglucose positron emission tomography (FDG-PET) will be collected to determine changes in glucose metabolism of the tumor lesions.
- **Original or archival tumor:** Potential prognostic biomarkers such as MMR status and CEA expression will be confirmed on archival tumor, if available, or from the freshly obtained biopsy samples. These measurements will assess the CEA change over the course of the disease and the stability of the measurements.

Blood, Serum or Plasma Samples

Blood samples will be collected for the analyses of immune cell number and activation. Blood samples will be collected and analyzed with respect to alterations in the number and activation and differentiation of immune cells as a consequence to treatment with RO6958688 in combination with atezolizumab. The samples will be analyzed by flow cytometry, and the number of cells that belongs to lymphocyte subsets (CD4+ T cells, CD8+ T cells, NK cells, B cells, and monocytes) and their activation and differentiation status will be determined (including but not limited to CD25, Ki67, PD1, and TIM3). Additional immune PD biomarkers that are related to the mode of action (MoA) of RO6958688 in combination with atezolizumab may also be analyzed from serum or plasma samples taken from all patients. Such PD measures will be considered as disease-monitoring markers and include but are not limited to sCEA. If available, an assessment of tumor growth kinetics will be made by comparing post-treatment scans with the last available pre-study scan.

Tumor Biopsy Samples

Tumor biopsy samples (each consisting of two tissue specimens at least the size of an 18G core needle biopsy, fine-needle aspiration is not acceptable) will be collected in all enrolled patients (once at baseline [after the baseline FDG PET] to confirm eligibility and once during the study treatment period), and two core samples will be collected at each occasion. Mandatory biopsies will be collected from all patients enrolled, except for NSCLC patients for whom there is no accessible lesion. The biopsies will be taken from accessible, "non-critical" tumor locations, including, but not limited to, skin, lymph node, rectum, liver, etc. On-treatment tumor biopsies will

"Use of this document is governed by the terms of use on the first page of this document."

be randomized within dose schemes as follows: half of the patients at week 3, half of the patients at week 6.

Tumor biopsies should be taken predose (and up to 48h before the visit).

If feasible, biopsies may be repeated if the initial biopsy did not contain sufficient tumor material for analysis.

If preliminary data suggest that modification of the on-treatment tumor biopsy timepoint would be more appropriate, alternative on-treatment tumor biopsy timepoints could be considered in the future cohorts.

The baseline and on-treatment biopsies should preferentially be taken from the same tumor lesion to ensure comparability when accessible in a non-critical location. Data from FDG-PET should guide which lesion would be more reflective of capturing the PD effect and thus the baseline and on-treatment biopsies should be taken from that tumor lesion. For patients continuing in the study, additional biopsies may be taken at the discretion of the investigator. These biopsies may serve to evaluate viable tumor given the mechanism of action of RO6958688 to induce tumor inflammation. For patients discontinuing from the study due to disease progression, additional optional biopsies, if clinically feasible, may be taken to aid the understanding of immune resistance mechanisms.

Tumor biopsies will be centrally analyzed for immune cell number and activation by flow cytometric and/or immunohistochemical methods for the density of different immune cell lineages (including but not limited to CD4+, CD8+, B cells, NK cells, macrophages) and their activation and differentiation status (including but not limited to CD25, Ki67, PD1, TIM3) and for the expression of tumor markers such as PD-L1, IDO etc... In addition, gene expression analysis may be carried out. The goals of these analyses will be (i) to establish a dose–response and/or an exposure-response relationship and (ii) to understand the MoA of RO6958688 in combination with atezolizumab at the tumor site.

Positron Emission Tomography (PET)

FDG-PET can identify sign of biological effect early, before tumor size is reduced. Moreover, a reduction in the FDG-PET signal within days or weeks of initiating therapy (e.g., in lymphoma, non–small cell lung, and esophageal cancer) has been shown to correlate with prolonged survival and other clinical end points now used. These findings suggest that FDG-PET could facilitate drug development as an early marker of drug effect.

Whole-body PET should begin 60 ± 10 min after FDG injection (please refer to the WP29945 imaging manual for more details).

EFFICACY OUTCOME MEASURES

Any evaluable or measurable disease must be documented at screening and re-assessed at each subsequent tumor evaluation. The efficacy/activity outcome measures for this study are:

- ORR, defined as the proportion of patients achieving objective partial (PR) or complete (CR) responses
- Best overall response (BOR) defined as the best response recorded from the start of the study treatment until the end of treatment taking into account any requirement for confirmation
- Duration of response (DOR) defined as the time from initial objective response (PR or CR) to the first objective progression or death from any cause
- The rate of patients with stable disease (SDR)
- DCR, defined as ORR + SDR
- PFS according to Response Evaluation Criteria in Solid Tumors, Version 1.1 (RECIST v1.1)
- OS, defined as the time from first study treatment (primary efficacy analysis) or randomization date (ITT, only in randomized schedule comparison expansions) to death from any cause

“Use of this document is governed by the terms of use on the first page of this document.”

Tumor response will be evaluated according to RECIST v1.1 and modified RECIST criteria using unidimensional measurement such as computed tomography (CT) scan or magnetic resonance imaging. Assessment of CT/MRI scans as tumor assessments will be performed at the sites during the whole study, and in addition centrally by an independent reviewer for prospective and retrospective analysis. Advanced volumetric analyses of CT/MRI will be performed.

Tumor assessment will be performed once during the screening. The first assessment after the start of treatment will be performed at 8 weeks (C3D15) and continue every 8 weeks thereafter for the first year and every 12 weeks thereafter until disease progression or treatment discontinuation. All tumor assessments after baseline may be done within ±7 days of the scheduled visit. Additionally, FDG-PET based tumor assessment will be performed at baseline and at week 4 (C2D8 + 1 week) and week 16 (C6D8) after the first dose of RO6958688, in case of dose delay the FDG PET should be done at week 4 (+1 week) and week 16. The week 16 FDG PET scan should be performed predose (and up to a maximum of 3 days before the visit).

Based on data generated during this trial, the timepoints at which the two on treatment FDG PET scans are performed may be modified.

Confirmation of partial and complete responses will be done at the next scheduled visit after at least 28 days from the initial response. A patient is assigned a best overall confirmed response SD if they have a response assessment of SD, PR, or CR at one or more visits at least 42 days (6 weeks) after start of study treatment, but are not a confirmed CR or PR.

PFS per RECIST v1.1 or modified RECIST criteria is defined as time between enrollment or randomization date (whichever is applicable) and date of first documented disease progression per RECIST v1.1 or modified RECIST criteria, respectively, or death from any cause, whichever occurs first. Patients who neither progressed nor died in this interval, or who are lost to follow-up are censored at the date of last tumor assessment within this time window or last follow-up for progression of disease. Patients for whom no post-baseline tumor assessments are available are censored at first study treatment.

Only for the randomized schedule comparison expansions, PFS and OS in the ITT efficacy analysis population may be analyzed for comparison of the schedules, where the start is defined as the date of obtaining informed consent by the patient.
Conventional response criteria may not adequately assess the activity of immunotherapeutic agents because progressive disease (by initial radiographic evaluation) does not necessarily reflect therapeutic failure. Because of the potential for pseudoprogression/tumor immune infiltration, this study will allow patients to receive atezolizumab and/or RO6958688 to remain on study treatment after apparent radiographic progression, provided the benefit-risk ratio is judged to be favorable.
Patients should be discontinued for unacceptable toxicity or loss of RO6958688 exposure (in which case they can continue to receive atezolizumab alone) or symptomatic deterioration attributed to disease progression as determined by the investigator after an integrated assessment of radiographic data and clinical status (see Section 4.6.1.1).

Patients will be permitted to continue study treatment after meeting RECIST criteria for progressive disease (by investigator assessment) if they meet all of the following criteria:

- Evidence of clinical benefit as assessed by the investigators
- Absence of significant symptoms and signs (including worsening of laboratory values; e.g., new or worsening hypercalcemia) that indicate unequivocal progression of disease
- No decline in ECOG performance status that can be attributed to disease progression
- Absence of tumor growth at critical anatomical sites that cannot be managed by protocol–allowed medical interventions

- Patients for whom approved therapies exist must provide written consent to acknowledge that they defer these treatment options in favor of continued study treatment at the time of initial apparent progression.

"Use of this document is governed by the terms of use on the first page of this document."

Patients in whom radiographic disease progression is confirmed at a subsequent tumor assessment may be considered for continued study treatment at the discretion of the investigators if they continue to meet the criteria above and have evidence of clinical benefit.

Optional submission of the latest pre-study or historical CT scans is highly encouraged for assessment of tumor growth kinetics within 6 weeks of patient entering the study if available. This scan will be compared to those collected during the study to determine tumor growth kinetics.

EXPLORATORY OUTCOME MEASURES

The exploratory objectives for this study include but are not limited to the following:

- A possible association of expression of activation related immune genes (e.g., IFNγ, CXCLC9, etc.) with PD response will be investigated
- The baseline values and kinetics of soluble markers of immune cell activation (such as sCD25) and tumor markers (such as CEA) will be explored.

Additional markers may be measured in case a scientific rationale for these analyses develops.

BIOMARKER/GENOTYPING SAMPLE COLLECTION

The specimens will be used for research purposes to identify biomarkers useful to predict and monitor response to RO6958688 and atezolizumab treatments, identify biomarkers useful to predict and monitor safety of the combination of the two compounds, assess PD effects of RO6958688 and atezolizumab treatments, and investigate mechanisms of immune escape. Additional markers may be measured in case a scientific rationale for these analyses develops.

Tumor

Tumor biopsy samples (each consisting of at least two tissue specimens at least the size of a 18G core needle biopsy, fine-needle aspiration is not acceptable) will be collected from all patients who participate in the study on two occasions (once at baseline [after the baseline FDG PET] to confirm eligibility and once during the study treatment period), and at least two core samples will be collected at each occasion. For patients discontinuing from the study due to disease progression, additional optional biopsies, if clinically feasible, may be taken to aid the understanding of immune resistance mechanisms. The biopsies will be taken from accessible, “non-critical” tumor locations, including, but not limited to, skin, lymph node, rectum, liver, etc. If feasible, biopsies may be repeated if the initial biopsy did not contain sufficient tumor material for analysis. In particular, archival tumor tissue is to be obtained from all patients, if available, in order to perform CEA assessment for patient eligibility (refer to the Laboratory Manual).

If a patient undergoes any medical procedure during the course of the study that may yield tumor tissue, any remaining samples or any portion of the tumor sample not used for medical diagnosis may be obtained for exploratory analysis. Patients must provide specific consent in order for discarded samples from routine care to be used for exploratory analysis.

Whole Blood

Whole blood samples will be collected for the flow cytometry for determination of immune cell markers (e.g., immune cell subsets, activation and proliferation markers, etc.). In addition, whole blood samples will be taken for TCR Vβ sequencing (the CDR3-TCR beta chain repertoire). The DNA will be used to determine the immune repertoires of peripheral T cell receptor (TCR) Vβ CDR3 and analyze TCR diversity.

Soluble CEA

A mandatory blood sample will be taken for soluble CEA central assessment.

“Use of this document is governed by the terms of use on the first page of this document.”

Serum or plasma

Blood for serum or plasma isolation will be collected for investigation of PD markers such as sCD25, cytokines (such as IL-6, IFNγ, TNFα), and tumor markers. In the event of an IRR/CRS, an additional sample will be collected.

These samples will be destroyed within 2 years after the date of final closure of the clinical database. Archival tumor blocks will be returned. Other residual tissue material (slides, extracts, etc.) will be destroyed within 2 years after the date of final closure of the clinical database unless the patient gives specific consent for the remainder of the tissue sample(s) to be stored for optional exploratory research. If the patient provided consent for optional exploratory research the tissue samples will be destroyed no later than 15 years after the date of final closure of the clinical database.

Clinical Genotyping Samples

A mandatory baseline whole blood sample will be taken from every patient for DNA extraction. The DNA may be used to determine if alleles at genes associated with immune responses such as Chemotaxis, HLA, immunosuppression etc, affect the PK/PD/efficacy/safety of RO6958688 and atezolizumab. In addition, the DNA may be used as a reference to identify tumor mutations. Data arising from this study will be subject to the same confidentiality as the rest of the study. This specimen will be destroyed immediately after analysis and the results have been checked.

ROCHE CLINICAL REPOSITORY

The Roche Clinical Repository (RCR) is a centrally administered facility for the long-term storage of human biological specimens including body fluids, solid tissues and derivatives thereof (e.g., DNA, RNA proteins/peptides). Specimens for dynamic (non-inherited) biomarker discovery and validation will be collected from patients who consent to participate in the RCR.

These specimens will be used for research purposes to identify biomarkers that are predictive of response to treatment with RO6958688 in combination with atezolizumab, and will help to better understand the pathogenesis, course, and outcome of the studied cancer types. The collected samples might allow the generation of statistically meaningful biomarker data.

The results of specimen analysis from the RCR will facilitate the rational design of new pharmaceutical agents and the development of diagnostic tests, which may allow for individualized drug therapy for patients in the future.

The specimens in the RCR will also be made available for future biomarker research towards further understanding of RO6958688 in combination with atezolizumab, treatment of related diseases and adverse events and for the development of potential associated diagnostic assays.

Whole blood samples for extraction of DNA and RNA will be collected if patients consent to participate in the RCR. Patients will also have the option to consent that any tissue material remaining after protocol defined analyses can be stored for up to 15 years in the RCR.

INVESTIGATIONAL MEDICINAL PRODUCTS

Test Products

For the purpose of the study, RO6958688, atezolizumab, and tocilizumab are considered investigational medicinal products (IMPs).

RO6958688

RO6958688 is a novel T-cell bispecific antibody targeting the human CEA on tumor cells and CD3 on T-cells. RO6958688 is administered intravenously, in patients with locally advanced and/or metastatic CEA (+) solid tumors. The starting dose of RO6958688 is 5 mg. QW dosing will be implemented initially to generate data that can be analyzed to assess whether different dosing schedules are more effective. If the accumulated data (safety, PK, PD) support that a different

“Use of this document is governed by the terms of use on the first page of this document.”

schedule might be more appropriate, an alternative dosing schedule, such as every-other-week dosing, could be considered.

In general, if patients experience adverse events that require a RO6958688 dose to be held, the dose may be delayed up to 14 days from the last one for the QW part and up to 21 days for the Q3W part. However, the acceptable length of interruption will depend on an agreement between the investigator and the Medical Monitor.

Atezolizumab

Atezolizumab is a human monoclonal antibody engineered to eliminate Fc-effector function. Atezolizumab targets human PD-L1 and inhibits its interaction with its receptor, programmed death−1 (PD-1). Atezolizumab also blocks the binding of PD-L1 to B7.1, an interaction that is reported to provide additional inhibitory signals to T cells. Atezolizumab is administered intravenously at 1200 mg IV Q3W (every 3 weeks).

In general, if patients experience adverse events that require an atezolizumab dose to be held, the dose may be delayed up to 105 days from the last one. However, the acceptable length of interruption will depend on an agreement between the investigator and the Medical Monitor.

Tocilizumab

Tocilizumab (Actemra®/RoActemra®) is a recombinant, humanized, anti-human monoclonal antibody directed against soluble and membrane-bound IL-6R, which inhibits IL-6 mediated signaling. Blocking the inflammatory action of IL-6 using tocilizumab could therefore represent a novel approach for the treatment of CRS.

Tocilizumab will be administered if required, for the management of severe CRS (if a study participant experiences severe CRS during or after any infusion of RO6958688. Tocilizumab has been recently approved by the FDA (August 2017) and received a positive CHMP opinion (European Medicines Agency 2018) for a restricted use, limited to Cytokine Release Syndrome (CRS) induced by chimeric antigen receptor (CAR) T cell in adults and pediatric patients 2 years of age and older (Tocilizumab USPI). Since tocilizumab will be used in the event of a severe CRS during or after any infusion of RO6958688, it is therefore classified as an investigational medicinal product (IMP).

NON-INVESTIGATIONAL MEDICINAL PRODUCTS

None.

PROCEDURES

SCREENING: Written informed consent for participation in the study must be obtained before performing any study-specific screening tests or evaluations. Screening and pretreatment assessments will be performed within 28 days prior to Cycle 1 Day 1 predose unless otherwise specified.

TREATMENT: Assessments performed during treatment are specified in the Schedule of Assessment and Hourly Assessment tables.

FOLLOW-UP: Patients who complete the study or discontinue from the study early will be asked to return to the clinic 28 days after the last dose of study drugs for a safety follow-up visit. The visit at which response assessment shows progressive disease may be used as the study completion/early termination visit.

LIST OF SPECIAL SAFETY OR PD TESTS: Although RO6958688 and atezolizumab are humanized antibodies, there is a risk that ADA against RO6958688 and atezolizumab may develop, potentially reducing its efficacy and/or potentially resulting in symptomatic hypersensitivity reactions, in particular immune-complex reactions. In Part IA in order to overcome ADA and impact on PK, optional intra-patient dose escalation of RO6958688 to the

“Use of this document is governed by the terms of use on the first page of this document.”

next available tolerated dose level may be permitted depending on emerging clinical and safety data at the discretion of the treating physician, and after discussion with the patient. Intra-patient dose escalation may only proceed after patients have tolerated at least the first 3 consecutive doses of RO6958688 after discussion and alignment with the Medical Monitor. In this study, samples will be taken to assess the possible presence of ADA. The date and time of each sample will be recorded in the electronic Case Report Form.

For the assessment of cytokine release, serum or plasma samples (blood for PK, pharmacodynamics and additional safety) will be collected at the time of an IRR/CRS (including repetitive occurrence of IRR/CRS). Cytokine analysis will include but is not limited to TNFα, IL-6, and IFNγ.

For patients who experience a Grade $\geq$2 IRR/CRS within 24 hours after the study drugs infusion, tryptase and total IgE will be analyzed.

WITHDRAWAL CRITERIA: Reasons for discontinuation of study drugs or withdrawal from the study may include but are not limited to the following:

- Patient withdrawal of consent at any time
- Any medical condition that the investigator or Sponsor determines may jeopardize the patient's safety if he or she continues in the study
- Investigator or Sponsor determines it is in the best interest of the patient.

All patients will attend a 28-day safety follow-up visit after receiving the last infusion of RO6958688 or atezolizumab.

BLINDING OR UNBLINDING:

Not applicable.

STATISTICAL METHODS

DOSE-FINDING APPROACH

Part IA: Dose escalation

A modified continual reassessment method (mCRM) with overdose control (EWOC) for dose escalation, based on occurrence of DLT, will be used for the dose escalation of RO6958688 in combination with 1200 mg Q3W atezolizumab. The model will be estimated using Bayesian inference with the priors properly pre-defined (based on safety data from monotherapy studies with RO6958688 and atezolizumab). Details on the statistical model, the priors, and the dose-escalation algorithm are defined in the protocol.

The maximum allowable increment for RO6958688 between dose-levels will be 100% from the current dose level throughout the study. The dose of RO6958688 will not be escalated above the monotherapy MTD if defined. Clinical judgment may always override mCRM recommendations in the dose-selection process.

With the end of Part I, a tentative MTD estimate will be defined. The DLT occurrence data from the additional patients with QW schedule in Part I and Part II of the study will be used to update the statistical model used in Part I and lead to a final MTD definition at the end of this trial.

Part IB: Dose/schedule finding

An intra-patient dose escalation design is used to estimate the late cycle MTD of RO6958688 in combination with 1200 mg Q3W atezolizumab in Cohort B1 in Part IB (see Protocol Section 6.7.1.2). The dose of RO6958688 will not be escalated in the intra patient dose escalation above the monotherapy late cycle MTD of RO6958688 if defined in the BP29541 protocol. In addition, a fast escalation design of RO6958688 in combination with 1200 mg Q3W atezolizumab is investigated in cohort B2. Note: Cohort B2 has not and will not enroll any patients. Cohort B1 will not enroll any additional patients.

"Use of this document is governed by the terms of use on the first page of this document."

Furthermore, Part IB of the study will explore two additional step-up dose regimens (cohorts C1 and C2) in combination with atezolizumab 1200 mg Q3W, in a randomized schedule comparison expansion, in order to contribute to determination of a recommended dose and schedule for further development (RP2D). Based on the clinical data from the randomized cohorts (C1 and C2), an additional step-up dose regimen (C3) might be explored as well. In addition, MSS CRC Cohort G1 will explore one schedule with obinutuzumab pretreatment, and the optional Cohorts G2 and G3 could generate data for gastric and pancreatic cancer patients with obinutuzumab pretreatment. Descriptive and model-based comparisons of efficacy endpoints as well as safety summaries will be produced in order to support this decision. Note: Cohort C1 enrolled 39 patients, and Cohort C2 enrolled 35 patients; no further patients will be enrolled into these cohorts. Optional cohort C3 and cohorts G1, G2, and G3 have not and will not enroll any patients.

Finally, maximum five additional safety cohorts in other tumor types will be explored, without formal estimation of maximum tolerated doses. Note: These cohorts will not enroll any additional patients.

Part II: Expansion

The expansion part may generate more efficacy data at the recommended dose and schedule. Statistical models will be used to summarize the efficacy data obtained across different dose levels and schedules, in order to contribute to the final selection of a recommended dose and schedule. Note: Part II has not and will not enroll any patients.

SAFETY ANALYSES

One of the primary objectives of the study is to evaluate the safety profile of RO6958688 in combination with atezolizumab. Therefore, the primary endpoints of the study contain safety and, in particular, the occurrence of DLT. All patients enrolled in the study who receive at least one dose of any of the study medications will be included in the safety evaluation. Safety will be determined, but not limited to, by adverse events, laboratory tests, ADAs, autoantibodies, vital signs, ECGs, physical examinations, and performance status, as well as by DLTs. As appropriate, listings, summary tables, and graphs will be provided for safety and tolerability assessments.

PHARMACOKINETIC AND IMMUNOGENICITY ANALYSES

PK data from patients who will receive at least one dose of RO6958688 or one dose of atezolizumab will be included in the PK analysis of RO6958688, atezolizumab respectively. Patients will be excluded from the PK analysis if they violate the inclusion or exclusion criteria, deviate significantly from the protocol, or if data are unavailable or incomplete, which may influence the PK analysis.

Extensive PK sampling will be done in the study to appropriately characterize the PK of RO6958688 and atezolizumab when given in combination.

PK parameters will be derived from the plasma concentrations of RO6958688, atezolizumab using standard non-compartmental methods, as appropriate. Individual data will be listed and summarized using descriptive statistics, including but not limited to mean, standard deviation, geometric mean, coefficient of variation, median, and range. The parameters will include, e.g., AUC, CL, Vss, accumulation ratio, and terminal elimination half-life. Mean concentration versus time will be plotted on either semi-logarithmic or normal scales.

In addition, non-linear mixed effect modeling will be used to analyze the sparse sampling dose-concentration-time data of RO6958688. Population PK parameters such as CL and V will be estimated and the influence of various covariates on these parameters will be investigated. Secondary parameters such as AUC and Cmax will be derived from the individual post-hoc predictions. The linearity of PK (AUC, C_{max}) will also be investigated.

"Use of this document is governed by the terms of use on the first page of this document."

PK sampling times, samples for ADA will be collected for this study according to the Schedule of Assessments and Hourly tables (SoA).

PHARMACODYNAMIC ANALYSES

The PD analysis population will be a subset of the efficacy analysis population, based on the availability of the outcome measures. Descriptive statistics and graphical outputs will be used in summarizing peripheral blood, skin, and tumor PD markers. Absolute and percentage change from baseline will be calculated for the PD markers.

Correlations between the CEA expression level and pharmacodynamics and efficacy outcomes will be explored using graphical and modelling techniques.

PHARMACOKINETIC-PHARMACODYNAMIC ANALYSES

The time course of tumor size, imaging parameters, PD markers and/or safety measurements may be regarded as response variables to drug exposure. Exploratory graphical analyses of exposure-efficacy relationships will be done for selected PD and/or safety measurements if possible. If the data permit, a PK/PD modeling approach may be considered in order to further explore the exposure-response relationship of selected response variables.

EFFICACY ANALYSES

Two efficacy analyses will be performed. The primary efficacy analysis population will consist of all patients who receive at least one dose of any study drug. An additional ITT population will consist of all patients who are not screen failures and were included in the trial (ICF signature), and will be used for sensitivity analyses. Patients who have been treated before Cycle 1 Day 1 with RO6958688 will be analyzed separately.

Tumor response data will be reported using descriptive statistics. ORR, SDR, and DCR at relevant timepoints will be summarized using relative frequencies and 95% confidence limits. Duration of response (DOR) and progression free survival (PFS) on treatment will be summarized using time to event analyses and Kaplan Meier curves. Duration of response will only be analyzed in those cohorts with a sufficient number of responders. Preliminary overall survival (OS) data may as well be tabulated and summarized using time to event analyses and Kaplan Meier curves. Summaries will be carried out by cohort, dose, and overall. This will be carried out for both RECIST and modified RECIST efficacy endpoints, for investigator assessed data for the whole study and for centrally assessed data for prospective and retrospective analysis.

The analysis of tumor response is based on the best overall response (BOR). BOR is defined as the best response recorded from the start of treatment until disease progression/recurrence or death whichever occurs first. Since tumor assessments take place every 8 weeks, 60 days is chosen so as to cover these assessments sufficiently.

For the ORR analyses in the primary and ITT efficacy analysis populations, patients who withdraw study treatment because of any reason, die or clinically progress before the first tumor assessment on treatment will be assigned a best overall response of Non Evaluable. These patients will be included in the denominator of the corresponding ORR estimate.

SAMPLE SIZE JUSTIFICATION

The sample size estimation for the dose escalation in Part IA is based on study simulations of the mCRM with EWOC design, see Section 6.2 for details.

The sample size of 20 patients per dose scheme in the schedule comparison expansion (cohort A) in Part IB allows for a reasonably precise differentiation between the QW and Q3W dose schemes. Specifically, an observed difference of ca. 10% points in ORR would lead to approximately 80% posterior probability of a true response rate difference between two dose schemes. For example, if the number of objective responses is 4 in one and 2 in the other dose

"Use of this document is governed by the terms of use on the first page of this document."

scheme out of 20 patients each, then the posterior probability of a positive response rate difference is 79.5%.

For cohort C, the randomization of approximately 40 patients to each of the arms C1, C2 and possibly 40 patients enrolled in a later optional arm C3 allows for a reasonably precise differentiation between the arms. Specifically, if one of the arms has 20% true ORR, and the other two arms have only 10% true ORR, then n=40 patients per arm gives 83% power to decide for the correct arm as the best one. In another scenario, where the first arm has 10% true ORR, the second arm 20% true ORR, and the third arm 30% true ORR, then n=40 patients per arm gives 85% power to pick the correct third arm as the best one. Hence, the sample size of 40 patients per arm in cohort C is justified. Note: Cohort C1 enrolled 39 patients, and Cohort C2 enrolled 35 patients; no further patients will be enrolled into these cohorts. Cohort C3 has not and will not enroll any patients.

For Cohort G1, the inclusion of 40 patients will allow a reasonably precise estimation of the incidence of ADA. Specifically, if none of the 40 patients develops ADA by week 8, then the 95% confidence interval for the ADA incidence will be 0% to 9%, i.e., exclude values of 10% or higher. In addition to the assessment of immunogenicity reduction by obinutuzumab, Cohort G1, as well as optionally Cohorts G2 and G3 will allow for a descriptive assessment of the primary safety and efficacy endpoints in the MSS CRC and optionally in the Gastric and Pancreatic indications, respectively. Specifically, observing an ORR of 20% in a cohort would result in a 95% confidence interval from 9% to 36%, i.e., exclude values of 9% or lower. Note: Cohorts G1, G2, and G3 have not and will not enroll any patients.

The sample size of approximately 10-20 patients in each of the cohorts B1 (MSS and MSI-H CRC separately), B2 and the safety cohorts in other indications allows for an initial assessment of safety and tolerability. This is shown in detail with simulations of the intra-patient dose escalation design in cohort B1 in protocol Appendix 7. Note: Cohort B2 has not and will not enroll any patients. The other cohorts have stopped enrollment.

Furthermore, mandatory tumor biopsies of all patients (except for NSCLC patients for whom there is no accessible lesion) are required for the secondary objective of characterizing the pharmacodynamic (PD) effects and duration of PD response on the basis of an increase in activated intratumoral T cells. In Part I, the collected PD data will allow to better define the dose range for the final RP2D and schedule selection. Therefore, it is necessary to have mandatory biopsies for all patients in Part I, because potentially many different doses will be tested in Part I, hence the sample size per dose and schedule combination will be small and has to be maximized.

Interim Analyses

Throughout the whole study, there will be ongoing safety assessments and PK data analyses, which do not qualify as formal interim analyses. In particular, dose escalations in Part I will be based on the ongoing safety review of the data and especially DLT data as per the mCRM with EWOC design.

In addition, there will be an internal interim analysis after Part I for selecting the recommended dose and schedule for Part II. Note: Part II will not open.

CENTERS

Approximately 35 centers in North America, Europe and Asia will be involved for the enrollment of patients. If necessary, additional sites may be added. Note: No patients were enrolled in Asia.

“Use of this document is governed by the terms of use on the first page of this document.”

LIST OF PROHIBITED MEDICATIONS

The use of the following therapies is prohibited during the study and for at least 28 days prior to initiation of study treatments (unless otherwise specified):

- Investigational or unlicensed/unapproved agents
- Immunotherapy/radio-immunotherapy
- Chemotherapy
- Hormonal anticancer therapy
- Immunostimulatory agent (all patients, including those who discontinue the study early, should not receive other immunostimulatory agents for 10 weeks after the last dose of atezolizumab)
- Radiotherapy (with the exception of limited–field palliative radiotherapy, which can be given any day except on days of study drug administration and one day prior and post study drug administration)
- Biologic agents (e.g., bevacizumab, erlotinib)
- Immunosuppressive medications, including but not limited to cyclophosphamide, azathioprine, methotrexate, and thalidomide; these agents could potentially alter the activity and the safety of atezolizumab. Systemic corticosteroids administered at a dose equal or higher than prednisone 10 mg/day or equivalent (inhaled and topical steroids are permitted).

    Systemic corticosteroids, TNF-α inhibitors, mycophenolate, and other immunosuppressive medications may be administered for the treatment or prevention of *immune-mediated* toxicities at the discretion of the treating physician after consultation with the Medical Monitor.

- Other systemic anti-neoplastic agents and targeted therapies
- Patients must not receive live, attenuated vaccines (such as FluMist®) at any time during the study and up to 5 months after the last dose of atezolizumab. Vaccination with live vaccines is not recommended during treatment and is forbidden 28 days prior to dosing with atezolizumab.
- Initiation or increased dose of granulocyte colony-stimulating factors (e.g., granulocyte colony-stimulating factor, granulocyte/macrophage colony-stimulating factor, and/or pegfilgrastim) is prohibited.

"Use of this document is governed by the terms of use on the first page of this document."

<u>**LIST OF ABBREVIATIONS AND DEFINITIONS OF TERMS**</u>

Abbreviation	Definition
ADA	anti-drug antibody
AE	Adverse Events
ALT	Alanine aminotransferase
AML	Acute Myeloid Leukaemia
aPTT	Activated partial thromboplastin time
AST	Aspartate aminotransferase
AUC	Area under the curve
BiTE	bispecific T-cell engager
BLQ	below limit of quantification
BP	Blood Pressure
CA	Competent Authority
CCOD	clinical cut off date
CD3e	CD3 epsilon chain
CD25	late T-cell activation marker
CD45	leukocyte common antigen
CD69	early T-cell activation marker
CDR	Complementarity determining region
CEA	carcinoembryonic antigen
CEACAM	carcinoembryonic antigen-related cell adhesion molecule
CEA TCB (RO6958688)	CEA T-cell bispecific antibody
CKD-EPI equation	Chronic Kidney Disease Epidemiology Collaboration
CL	Clearance
C_{max}	maximum serum concentration
CNS	Central Nervous System
CRC	colorectal cancer
CRO	Contract research organization
CRS	Cytokine release syndrome
CSAP	Clinical statistical analysis plan
CSR	Clinical study report
CT	Computed Tomography
CTCAE	Common Terminology Criteria for Adverse Events
cyCD3e	cynomolgus monkey CD3e
cyCEA	cynomolgus monkey CEA
cyCEA TCB (RO6958690)	cynomolgus monkey homologue of RO6958688
DCR	Disease control rate

“Use of this document is governed by the terms of use on the first page of this document.”

Abbreviation	Definition
DL_{CO}	Diffusing capacity of the lung for carbon monoxide
DLT	Dose-limiting Toxicities
DNA	Deoxyribonucleic acid
EC	Ethics Committee
ECG	Electrocardiogram
ECOG	Eastern Cooperative Oncology Group
eCRF	Electronic Case Report Form
EDC	Electronic data capture
EpCAM	Epithelial cell adhesion molecule
ESF	Eligibility Screening Form
EU	European Commission
EWOC	Escalation with overdose control
Fc	Fc portion of IgG
FcγR	Fc gamma receptor binding to the Fc portion of IgG
FcRn	neonatal Fc receptor
FEV1	Forced expiratory volume in 1 second
FDA	U.S. Food and Drug Administration
[18F]-FDG PET	Fluoro Deoxy Glucose Positron Emission Tomography
FFPE	Formaldehyde fixed-paraffin-embedded
FSH	Follicle stimulating hormone
GCP	Good Clinical Practice
GGT	γ-glutamyl transferase
HBsAG	Hepatitis B surface antigen
HBcAb	Total Hepatitis B core antibody
hCD3e	human CD3e
hCEA	human CEA
HCV	Hepatitis C
HDL	High density lipoproteins
HIPAA	Health Insurance Portability and Accountability Act
HIV	Human immunodeficiency virus
IB	Investigator's Brochure
ICH	International Conference on Harmonisation
ICF	Informed Consent Form
IFNγ	interferon gamma
IgA	Immunoglobulin A
IgE	Immunoglobulin E

"Use of this document is governed by the terms of use on the first page of this document."

Abbreviation	Definition
IgG	Immunoglobulin G
IHC	Immunohistochemistry
IL-2	interleukin-2
IL-6	interleukin-6
IL-8	interleukin-8
IL-10	interleukin-10
IMP	Investigational medicinal product
IND	Investigational New Drug (application)
INR	International normalized ratio
IRB	Institutional Review Board
IRR	infusion-related reaction
irRC	immune–related response criteria
IUD	Intrauterine Device
IV	Intravenous
IxRS	Interactive (voice/web) response system
LDH	Lactate dehydrogenase
LDL	Low density lipoproteins
LH	Luteinizing Hormone
LLOQ	lower limit of quantification
LPLV	Last patient, last visit
MAb	monoclonal antibody
MAD	Multiple Ascending Doses
mCRM	modified continual reassessment method
MoA	mode of action
MRI	Magnetic resonance imaging
MSI-H	Microsatellite instability high
MSS	Microsatellite stable
MTD	Maximal tolerated dose
mUC	Metastatic Urothelial carcinoma
NCI	National Cancer Institute
NK	natural killer
NOAEL	No observed adverse effect level
NOG	NOD/Shi-scid/IL-2Rynull
NSAID	Non-steroideal anti-inflammatory drug
NSCLC	Non-Small Cell Lung Cancer
ORR	overall response rate
PD	Pharmacodynamic

"Use of this document is governed by the terms of use on the first page of this document."

Abbreviation	Definition
PDR	Product Development Regulatory
PFS	Progression-free survival
PK	Pharmacokinetic
PR	Partial response
PS	Performance Status
PT	Prothrombin time
QRS	QRS Complex
QT	QT Interval
QW	once per week
Q3W	every three weeks
RBC	Red Blood Cell
RCR	Roche Clinical Repository
RECIST	Response Evaluation Criteria in Solid Tumors
RNA	Ribonucleic acid
RO6958688 (CEA TCB)	CEA T-cell bispecific antibody
RO6958690 (cyCEA TCB)	cynomolgus monkey homologue of RO6958688
RP2D	Recommended Phase 2 Dose
RR	response rate
SAD	Single Ascending Dose
SAP	Statistical Analysis Plan
SAE	Serious Adverse Event
sCEA	soluble carcinoembryonic antigen
SD	stable disease
SI	SI units; Système International d'Unités
SoA	Schedule of Assessments
$t_{1/2}$	half-life
TB	Tuberculosis
TCB	T-cell bispecific antibody
TCR	T-cell receptor
TCZ	Tocilizumab
TIL	tumor infiltrating lymphocytes
TLC	Total lung capacity
TNFα	tumor necrosis factor alpha
TSH	Thyroid-stimulating hormone
ULN	Upper limit of normal
US	United States
VC	Vital Capacity
WBC	White Blood Cell

"Use of this document is governed by the terms of use on the first page of this document."

1. BACKGROUND AND RATIONALE

1.1 BACKGROUND ON DISEASE

Cancer is the leading cause of death worldwide. Based on the GLOBOCAN estimates, there were 14.1 million new cancer cases, 8.2 million cancer deaths, and 32.6 million people living with cancer (within 5 years of diagnosis) in 2012 worldwide (http://globocan.iarc.fr/Pages/fact_sheets_cancer.aspx#). A large percentage of patients with cancer are diagnosed with advanced disease and are considered to be incurable by surgery and/or radiation. Despite the advances in chemotherapy and targeted therapies, the prognosis of patients with advanced cancer remains poor in general. Consequently, there is a persisting and urgent medical need to develop new therapies that can be added to existing treatments to increase survival without causing unacceptable toxicity.

1.2 BACKGROUND ON IMMUNOTHERAPY

Recent clinical results of immune-based therapies in randomized human studies have shown that immune therapies are valid approaches in cancer therapy. The success of immune therapies, particularly immune checkpoint blocking monoclonal antibodies, has shown that these agents can extend overall survival in patients with cancer and, in some, provide durable responses that few other/existing cancer therapies can approach (Hodi et al. 2010; Honeychurch 2015; Mahoney 2015; Carosella 2015).

Immune-based therapies appear to be a relevant strategy in multiple tumor types, and tumor-specific mutations across tumor types may be an important stimulus of responsiveness to immune-based therapies. Human cancer cells are characterized by a multitude of genetic aberrations (Alexandrov et al. 2013; Lawrence et al. 2013), many of which may be associated with immunogenicity by presenting novel epitopes for immune-cell recognition (Segal et al. 2008). The immune response, largely mediated by tumor-reactive T cells, can be rapid, durable, and adaptable. It has been demonstrated that novel epitopes generated from missense mutations may be the target of tumor-reactive T cells and may mediate response to T cell checkpoint inhibitors (van Rooij et al. 2013). Moreover, the durable responses observed in a subset of patients long after completion of therapy suggest the generation of T cell memory, which has been associated with improved overall survival in patients with cancer (Pages et al. 2005; Kilinc et al. 2009).

Concurrent engagement of the target cell antigen and CD3 leads to activation of polyclonal cytotoxic T cells, resulting in target lysis. Blinatumomab has emerged as a recombinant bispecific T cell engager targeting CD19 and CD3 and has demonstrated remarkable antitumor activity in patients with B-cell malignancies (Steiglmaier 2015). Solitomab, another bispecific antibody construct targeting EpCam and CD3, induced an increased T-cell activation, proliferation, cytokine production and direct tumor cell killing of EpCam positive uterine serous carcinoma cell lines (Bellone 2015). MEDI-565/AMG211, a CEA/CD3 bispecific antibody induced in vitro and in vivo T-cell activation with subsequent killing of human tumor cell lines. The cytotoxicity observed

“Use of this document is governed by the terms of use on the first page of this document.”

was CEA dependent and independent of mutations commonly found in colorectal adenocarcinomas (Oberst 2014).

RO6958688 is a novel T- cell bispecific antibody (TCB) that targets carcinoembryonic antigen (CEA) expressed on tumor cells and CD3 epsilon chain (CD3e) present on T cells. In non-clinical models, the binding of RO6958688 to CEA and CD3 resulted in T-cell-mediated killing of the cancerous cells that expressed CEA. RO6958688 effectively mediated the killing of cancer cells only by concurrently binding to CEA present on a tumor cell and to CD3 present on a T cell. RO6958688 is currently being investigated as a single agent in a Phase I study in patients with advanced and/or metastatic CEA expressing tumors.

Current immune-based therapies only lead to durable responses in a proportion of patients with cancer and combination strategies are needed to improve therapeutic efficacy. Programmed death-ligand 1 (PD-L1) is found on the surface of cells in various tumor types and its expression is induced by interferon gamma (IFNγ). It prevents the immune system from destroying cancer cells by interacting with the inhibitory programmed death-1 (PD-1) and B7.1 receptors on activated T cells, which results in a T-cell inhibitory signal (Chen et al. 2012). Blockade of PD-L1/PD-1 pathway increased the proliferation of cytotoxic T cells in a co-culture of T cells along with ovarian cancer and non-small cell lung carcinoma (NSCLC) tumor cells in vitro (Curiel 2003; Zhang 2010). Atezolizumab blocks the interaction of PD-L1 with PD-1 and B7.1. Interruption of the PD-L1/PD-1 and PD-L1/B7.1 interactions represent an attractive strategy to reinvigorate tumor-specific T cell immunity and is currently being investigated in clinical trials as a possible treatment for various cancers (Swaika 2015). Recently it has been shown that blockade of the PD1/PD-L1 pathway augmented the enhancement CD33/CD3 bispecific T-cell engager mediated cytotoxicity in AML (Krupka 2015).

1.3 BACKGROUND ON RO6958688

RO6958688 is a TCB that targets CEA expressed on tumor cells and CD3e chain present on T cells (see Figure 1).

RO6958688 binds with high affinity and in a bivalent-binding mode to human CEA (hCEA; 0.2 nM). The anti-CEA binding domain targets a membrane-proximal domain of hCEA (RO6958688 Investigator's Brochure), binding specifically to hCEA and does not cross-react with cynomolgus monkey CEA (cyCEA) (RO6958688 Investigator's Brochure) because the membrane-proximal domain in hCEA is not conserved across species, including cynomolgus monkey. Because CEA is not expressed in rodents, RO6958688 also lacks cross-reactivity with mice and rats. Therefore, alternative nonclinical safety evaluation approaches were considered. This included development of a Cynomolgus monkey cross-reactive homologous (surrogate) antibody (cyCEA TCB; RO6958690) for evaluation in Cynomolgus monkey, and development of double transgenic mice, expressing human CEA and human CD3e (hCEA/hCD3e Tg) as a potential alternative toxicology species. However, both the cyCEA TCB (a surrogate

"Use of this document is governed by the terms of use on the first page of this document."

molecule) and a hCEA/hCD3e transgenic mouse model were shown to be unsuitable for nonclinical safety testing. An in vitro MABEL approach was therefore used to determine a starting dose in the EIH study (Study BP29541).CH1A1A98/99 × 2F1 does not have any direct functional activity and is not internalized upon binding (RO6958688 Investigator's Brochure). Because of the targeting of a membrane-proximal domain of hCEA, CH1A1A98/99 × 2F1 displays preferential binding to membrane-anchored CEA rather than shed, soluble CEA (sCEA) (RO6958688 Investigator's Brochure), and the killing potency of RO6958688 remains unaffected up to 0.2 μg/mL of sCEA (RO6958688 Investigator's Brochure).

RO6958688 also binds to T cells through its second binding unit-targeting CD3e of the T-cell receptor (TCR) complex (de la Hera et al. 1991; RO6958688 Investigator's Brochure). The anti-CD3 antibody used in RO6958688 cross reacts with human CD3e (hCD3e) and cynomolgus monkey CD3e (cyCD3e) chains but not with mouse CD3e chain. This antibody has been generated in house by humanization of the parental SP34 antibody (Pessano et al. 1985; Salmerón et al. 1991; Conrad et al. 1991; RO6958688 Investigator's Brochure). The binding to CD3e is monovalent, which prevents activation of T cells in the absence of simultaneous binding to tumor cells that express CEA, and has low affinity for both hCD3e and cyCD3e (80 nM). The differential binding affinity between CEA and CD3 may favor preferential targeting of RO6958688 to tumors and reduce the peripheral sink due to binding to T cells.

RO6958688 is a human immunoglobulin G1 (IgG1) with the Fc region bearing a novel, proprietary modification (P329G LALA mutation; Figure 1) that abrogates its binding in vitro to Fc γ receptors (FcγR) (RO6958688 Investigator's Brochure) and prevents FcγR-mediated co-activation of innate immune effector cells, including natural killer (NK) cells, monocytes/macrophages, and neutrophils, without changes in functional binding to neonatal Fc receptor, also called FcRn (RO6958688 Investigator's Brochure).

Simultaneous binding of RO6958688 to CEA and CD3 leads to T-cell activation and tumor cell lysis. The RO6958688-mediated tumor cell lysis is CEA-specific and does not occur in the absence of CEA expression or in the absence of simultaneous binding (cross-linking) of T cells to CEA-expressing tumor cells (RO6958688 Investigator's Brochure). In addition to killing, T cells undergo activation followed by tumor lysis as detected by increase of late and early T-cell activation markers (CD25 and CD69, respectively), cytokine release (interferon γ [IFNγ], tumor necrosis factor α [TNFα], granzyme B, interleukin [IL]-2, IL-6, IL-10), and proliferation of T cells (RO6958688 Investigator's Brochure).

"Use of this document is governed by the terms of use on the first page of this document."

Figure 1 Design, Structure, and Characteristics of RO6958688

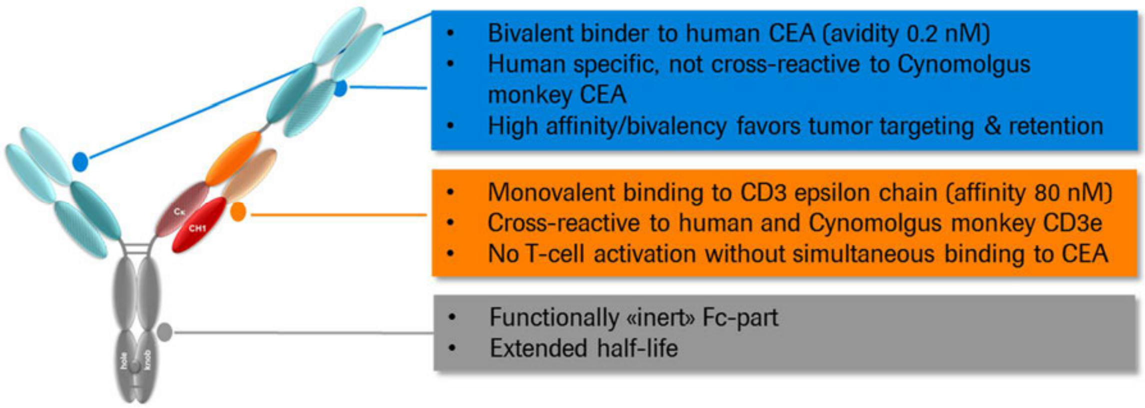

CEA = carcinoembryonic antigen; Fc = Fc portion of IgG; IgG1 = human immunoglobulin G1.

Note: Blue parts correspond to the high-affinity binding antibody (CH1A1A98/99 × 2F1) to hCEA. The red part corresponds to the humanized antibody (CH2527 [VL_7-46(13) VH_23-3(12)]) that binds to the CD3e chain. The grey parts correspond to the heterodimeric Fc region of the human IgG1 bearing the P329G LALA mutation.

1.3.1 Previous Non-Clinical Studies

The anti-tumor activity upon combination of RO6958688 with the anti-PD-L1 blocking antibody was assessed in vitro and in vivo.

RO6958688-mediated tumor lysis leads to upregulation of PD-1 on human T cells and of PD-L1 on surviving tumor cells - in vitro

The lysis of CEA-expressing MKN-45 target cells mediated by RO6958688 was assessed after 24 h and 48 h of incubation with human PBMCs (E:T 10:1, LDH release), Figure 2 (A 24 h, B 48 h).

"Use of this document is governed by the terms of use on the first page of this document."

Figure 2 RO6958688-Mediated Lysis of MKN45 Cells

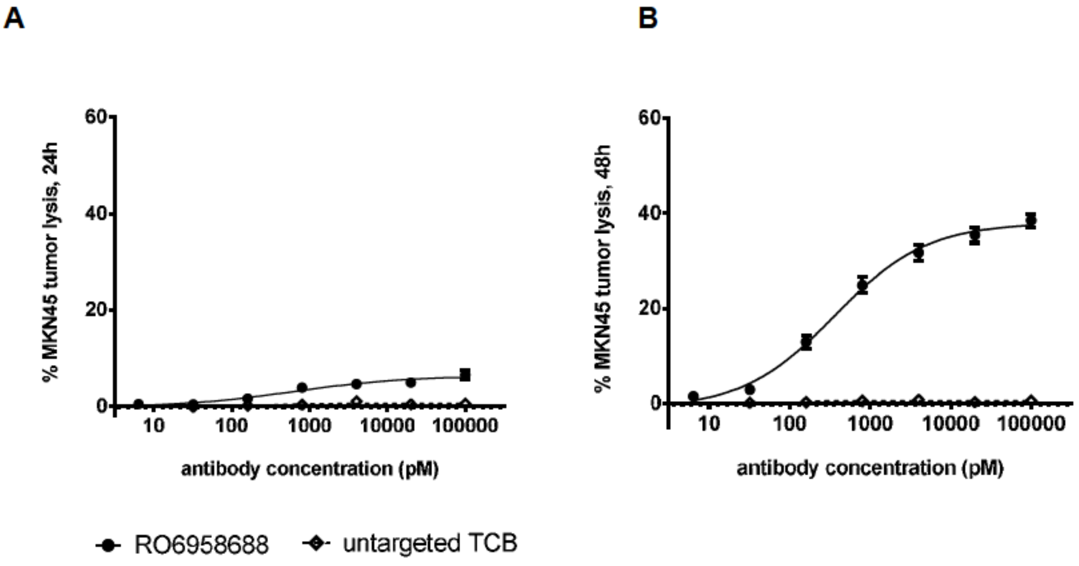

Representative graphs of RO6958688-mediated lysis of MKN-45 tumor cells assessed (A) 24 hours and (B) 48 hours after incubation of tumor cells with human PBMCs (E:T 10:1). The EC50 values of tumor cell killing are: 615 pM (24 h), 362 pM (48 h). Target cell killing was assessed by quantification of LDH released into cell supernatants.

Following killing, the surface expression of PD-1 receptor (on CD4+ or CD8+ T cells) and of PD-L1 (on tumor cells that survived killing) was assessed by flow cytometry. Figure 3 displays a dose-dependent upregulation of PD-1 receptor on CD8+ (A) and on CD4+ (B) T cells as well as of PD-L1 on tumor cells that resisted to killing (C) and were harvested after 48 h of incubation.

“Use of this document is governed by the terms of use on the first page of this document.”

Figure 3 RO6958688 Upregulation of PD-1 on T cells, Respective of PD-L1 on Surviving Tumor Cells after Tumor Cell Lysis

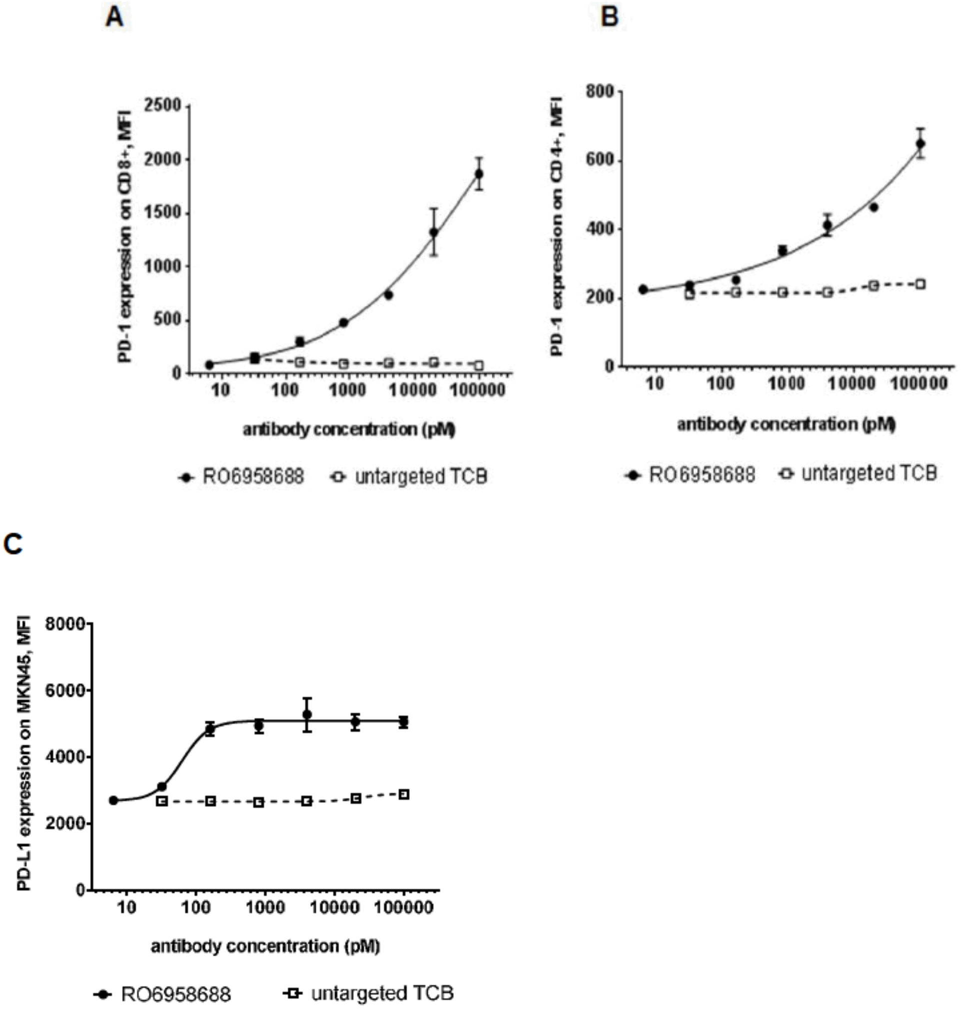

Representative graphs of RO6958688-mediated upregulation of PD-1 receptor expression on CD8+ T cells (C), CD4+ T cells (D), and of PD-L1 ligand expression on tumor cells that resisted killing (C) analyzed 48 h post incubation of MKN-45 tumor cells with human PBMCs (E:T 10:1, as in panel B). PD-1 and PD-L1 expression was analyzed by flow cytometry.

“Use of this document is governed by the terms of use on the first page of this document.”

RO6958688-mediated tumor lysis leads to upregulation of PD-1 on human T cells and of PD-L1 on surviving tumor cells - in vivo

Please refer to RO6958688 IB Section 4.1.3.

- In vivo studies – Xenograft Experiment with Co-Grafting of Effector Cells – PD-L1 upregulation in RO6958688-treated tumors as compared to vehicle, assessed by IHC
- In vivo studies – Xenograft Experiment with IP Transfer of Effector Cells –Tumor panel showing PD-1 upregulation on intra-tumor T cells upon RO6958688 treatment as compared to vehicle, assessed by flow cytometry and PD-L1 upregulation in RO6958688-treated tumors as compared to vehicle, assessed by IHC
- In vivo studies – Xenograft Experiment in Fully Humanized Mice – Increase of the percentage of PD-1-expressing intra-tumor CD8+ T cells and CD4+ T cells upon treatment with RO6958688 as compared to vehicle, assessed by flow cytometry; Anti-PD-L1 staining of vehicle and RO6958688-treated tumors collected at study termination denoting strong induction of intra-tumor PD-L1 expression upon RO6958688-treatment.

Assessment of in vivo anti-tumor activity upon combination of RO6958688 with the anti-PD-L1 blocking antibody

The anti-tumor efficacy of RO6958688 in combination with the anti-human PD-L1 blocking antibody (clone YW243.55.S70 re-synthesized in house with murine Fc) assessed in fully humanized mice bearing the gastric carcinoma tumor cell line (MKN45).

In summary, 1 x10^6 MKN45 tumor cells were injected subcutaneously in fully humanized NOG mice. 7 days after tumor cell injection, mice were randomized in four groups: the first group received phosphate-buffer saline (PBS, vehicle) as control, the second group received RO6958688 (at the dose of 2.5 mg/kg, administered twice a week for 7 weeks, 15 administrations in total), the third group received a-PD-L1 (at the dose of 10 mg/kg, administered once a week for 7 weeks, 8 administrations in total), and the fourth group received a combination of RO6958688 and a-PD-L1, administered concomitantly with the respective dose and schedules used in the single therapeutic groups for 7 weeks (8 administrations of a-PD-L1 10 mg/kg given once a week and 15 administrations of RO6958688 2.5 mg/kg given twice a week).

The results show a significant increase in anti-tumor activity upon combination of RO6958688 with anti-PD-L1 blocking antibody as compared to RO6958688 single agent. Treatment with a-PD-L1 as single agent doesn’t show any anti-tumor activity. No overt test item-related toxicities were observed following treatment with the combination (Roche Report No. 1066291).

“Use of this document is governed by the terms of use on the first page of this document.”

Figure 4 In Vivo Anti-Tumor Activity upon Combination of RO6958688 with the Anti-Human PD-L1 Blocking Antibody in MKN45 Tumor Model in Fully Humanized Mice

Average tumor burden and standard error of mean (SEM). Tumor burden was measured by digital caliper 3 times a week. Blue arrow indicates the day of start of therapy. At day 60: n=7 (vehicle), n=8 (RO6958688); n=4 (a-PD-L1); n=5 (combination RO6958688 with a-PD-L1). a-PD-L1 antibody = clone YW243.55.S70 re-synthesized in house with murine Fc.

A

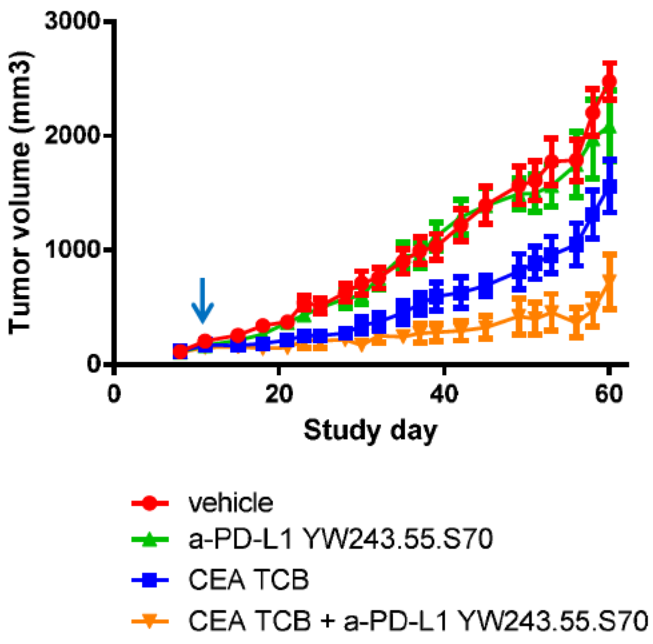

"Use of this document is governed by the terms of use on the first page of this document."

Figure 4 In vivo anti-tumor activity upon combination of RO6958688 with the anti-human PD-L1 blocking antibody in MKN45 tumor model in fully humanized mice. (cont.)

B

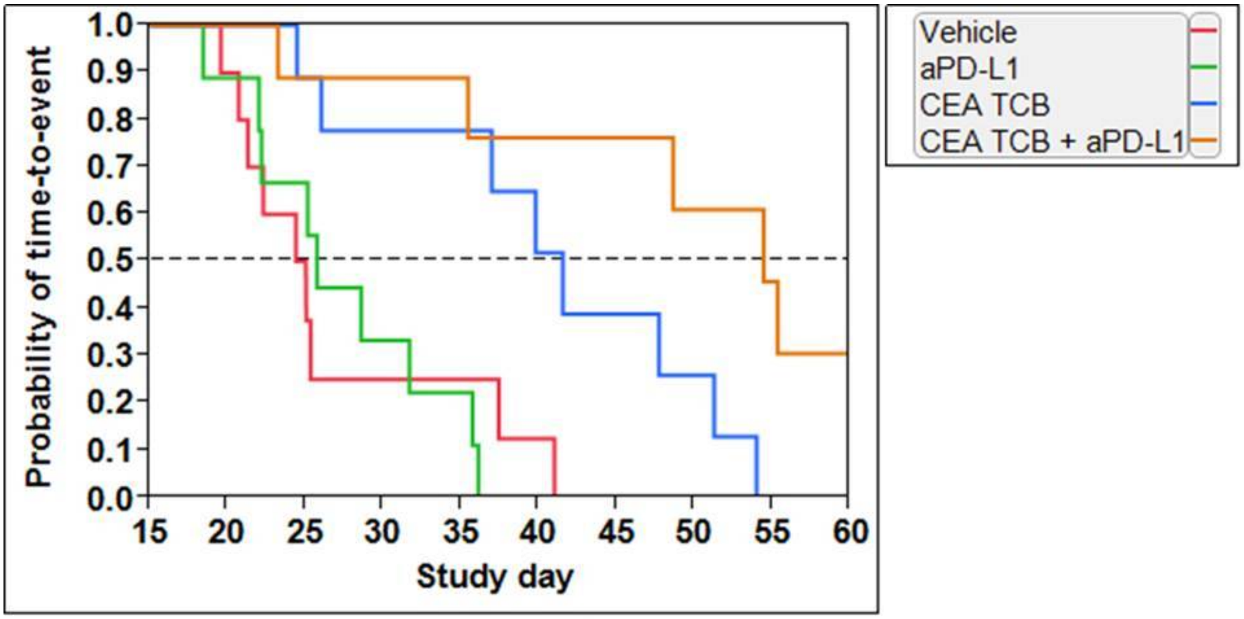

Kaplan-Meier curves give an estimation of the survival functions for one or more groups of right-censored data.

Time-to-event statistical analysis. The event was defined as reaching 500 mm^3 tumor volume. The pairwise log-rank test was used to compare the following treatment groups, as it can take the different drop-outs into account: vehicle vs RO6958688: p= 0.005; RO6958688 vs. a-PD-L1: p= 0.001; RO6958688 vs combination of RO6958688 with a-PD-L1: p= 0.03.

The pharmacology, pharmacokinetics, and in vitro toxicology of RO6958688 and cyno-cross reactive surrogate antibody (cyCEA TCB) have been investigated in several nonclinical studies. RO6958688 contains the high-affinity anti-CEA antibody (CH1A1A 98-99 x 2F1), which binds to hCEA and does not cross-react with Cynomolgus monkey CEA. Furthermore, CEA is not expressed in rodents, and the CD3e antibody in RO6958688 does not cross-react with mouse CD3e. Therefore, alternative nonclinical safety evaluation approaches, as described in the ICH S6 Guideline, were considered. This included development of a Cynomolgus monkey cross-reactive homologous (surrogate) antibody (cyCEA TCB; RO6958690) for evaluation in Cynomolgus monkey – and development of double transgenic mice, expressing human CEA and human CD3e (hCEA/hCD3e Tg) as a potential alternative toxicology species (RO6958688 IB).

However, both the cyCEA TCB (a surrogate molecule) and a hCEA/hCD3e transgenic mouse model were shown to be unsuitable for nonclinical safety testing. An in vitro

“Use of this document is governed by the terms of use on the first page of this document.”

MABEL approach was therefore used to determine a starting dose in the EIH study, instead of an in vivo approach (i.e., NOAEL approach) (RO6958688 IB).

A human whole blood cytokine release assay was performed to assess the risk of cytokine-mediated infusion-related reactions (IRRs). The data indicated a risk for cytokine-mediated events particularly at 100 nM (a concentration estimated to be reached at C_{max} with a dose of 58 mg), but also at lower concentrations in some donors. The potency of RO6958688 to induce cytokines is less than that of the CD3 antibody muromonab-CD3, where responses were observed starting at 10 nM.

In addition, a tissue cross-reactivity study with RO6958688 in normal human tissues, and an assessment of blood compatibility and hemolysis were performed (details of which can be found in the RO6958688 IB).

1.3.2 Previous Clinical Studies

RO6958688 is being investigated as a single agent in patients with locally advanced and/or metastatic solid tumors expressing CEA in Study BP29541 (first patient dosed Dec 30, 2014). The main purpose of this EIH study is to describe the safety profile of the single agent RO6958688 when given QW and to establish its MTD and/or recommended dose.

BP29541 is being conducted in 2 parts: Part I was a single ascending dose part in single-patient cohorts to evaluate the safety of RO6958688 at doses up to 2.5 mg. Part II is a multiple ascending dose part with RO6958688 as single agent given QW.

Refer to the RO6958688 Investigator's Brochure for additional information on the clinical development and clinical safety of RO6958688.

1.3.2.1 Safety of RO6958688

1.3.2.1.1 BP29541

At the clinical cutoff date (CCOD) of 27 April 2018 a total of 148 patients have been enrolled and have received at least one dose of either RO6958688 and/or obinutuzumab in Study BP29541. In Study BP29541 on average patients received 10.3 doses/cycles of RO6958688, with a median of 6.0 doses/cycles equating to an average of 76.03 days of treatment with RO6958688 per patient. Overall, adverse events (AEs) were reported in 145 of 148 patients (98.0%). The most common AE reported in ≥25 % of patients was IRR (105 patients [70.9%]), followed by pyrexia (78 patients [52.7%]), diarrhea (67 patients [45.3%]), nausea (53 patients [35.8%]), vomiting and anemia (47 patients [31.8%] each), asthenia (44 patients [29.7%]) and decreased appetite (43 patients [29.1%]). AEs considered related to study treatment by the investigator were reported in 138 of 148 patients (93.2%), with RO6958688–related AEs reported in 137 of 142 patients [96.5%], obinutuzumab-related AEs reported in 8 of 33 patients (24.3%), and AEs related to both RO6958688 and obinutuzumab reported in 3 of 27 patients (11.1%).

"Use of this document is governed by the terms of use on the first page of this document."

A total of 99 of 148 patients (66.9%) reported at least one Grade ≥3 AE with 86 patients (58.1%) reporting an AE with maximum Grade 3 intensity, and 7 patients (4.7%) reporting AEs with maximum Grade 4 intensity. Six Grade 5 AEs were reported: one RO6958688-related event of respiratory failure (600 mg RO6958688), one RO6958688-related event of dyspnea (40 mg RO6958688), one RO6958688-related event of IRR (Cohort C: 356 mg RO6958688), one obinutuzumab-related event of sepsis (60 mg RO6958688 Q3W + obinutuzumab), one unrelated cardio-respiratory arrest (Cohort A: 40 mg RO6958688) and one event of unrelated tumor thrombosis (2.5 mg RO6958688).

Serious adverse events (SAEs) were reported in 96 of 148 patients (64.9%) of which IRR (40 patients [27.0%]) was the most frequently reported, followed by pyrexia (8 patients [5.4%]), nausea, pneumonia, dyspnea, tumor pain, and acute kidney injury (5 of 148 patients [3.4%] each). SAEs assessed by the investigator as related to study treatment were reported in 66 of 148 patients (44.6%).

Ninety-four deaths (63.5%) were reported at the CCOD, of which 83 deaths (56.1%) were due to disease progression. There were 6 deaths due to AEs, 4 deaths (2.7%) due to unknown causes (which were confirmed as disease progression following the CCOD), and 1 death was due to sepsis.

Seven DLTs related to RO6958688 have been reported in 7 of 148 patients (4.7%) in Part II only at the time of the clinical cutoff date: Grade 3 dyspnea (40 mg RO6958688; 1 patient), Grade 3 hypoxia (60 mg RO6958688; 1 patient), Grade 3 diarrhea (300 mg RO6958688; 1 patient), Grade 3 colitis (300 mg RO6958688 with obinutuzumab pretreatment; 1 patient) and Grade 4 colitis (600 mg RO6958688; 1 patient), Grade 5 respiratory failure (600 mg RO68958688; 1 patient), and Grade 5 dyspnea (40 mg in Cohort B; 1 patient). The Grade 5 event of dyspnea (40 mg in Cohort B; 1 patient) occurred after the dose escalation part of the trial and was not used to derive the maximum tolerated dose (MTD).

The risk mitigation measures were further optimized following the receipt of two Grade 5 events reported as related to RO6958688 (Grade 5 IRR in BP29541 in this section and Grade 5 Hypovolemic Shock in WP29945 in Section 1.3.2.1.2).

1.3.2.1.2 WP29945

As of the CCOD of 27 April 2018, a total of 226 patients have been enrolled and have received at least one dose of either RO6958688 or atezolizumab in Study WP29945 with all patients reporting at least one AE. In Study WP29945 on average patients received 9.5 doses/cycles of RO6958688, with a median of 5 doses/cycles equating to an average of 81.11 days of treatment with RO6958688 per patient. The most common AEs reported in ≥25% patients were IRR (156 of 226 patients [69.0%]), diarrhea (136 patients [60.2%]), pyrexia (120 patients [53.1%]), dysgeusia (81 patients [35.8%]), fatigue (75 patients [33.2%] each), chills (72 patients [31.9%]), vomiting (71 patients [31.4%]), nausea (70 patients [31.0%]), asthenia (69 patients [30.5%]), and decreased

“Use of this document is governed by the terms of use on the first page of this document.”

appetite (60 patients [26.5%]). AEs considered related to study treatment by the investigator were reported in 222 of 226 patients (98.2%) with RO6958688–related AEs reported in 222 patients (98.2%), atezolizumab-related AEs reported in 183 patients (81.0%) and AEs related to both RO6958688 and atezolizumab reported in 179 patients (79.2%).

A total of 141 of 226 patients (62.4%) reported at least one ≥Grade 3 AE with 122 patients (54.0%) reporting an AE with maximum Grade 3 intensity, and 14 patients (6.2%) reporting an AE with maximum Grade 4 intensity. Five patients (2.2%) experienced a Grade 5 event: one event of hypovolemic shock related to RO6958688 (B1 cohort 600 mg RO6958688), one event of unrelated respiratory tract infection (300 mg RO6958688), one event of unrelated urinary tract infection (Safety cohort pancreatic cohort 600 mg RO6958688), one event of unrelated disseminated intravascular coagulation (DIC; 160 mg RO6958688), and one event of unrelated cerebrovascular accident (80 mg RO6958688).

SAEs were reported in 139 of 226 patients (61.5%) of which IRR (65 patients [28.8%]) was the most frequently reported SAE followed by pyrexia (17 patients [7.5%]) and diarrhea (10 patients [4.4%]). SAEs considered related to study treatment by the investigator were reported in 113 of 226 patients (50.0%).

Fifty-four patients had died (23.9%) at the CCOD, of which 48 deaths (21.2%) were due to progression of disease. There were 5 deaths due to an AE. The remaining death was due to an unknown cause at the time of survival follow-up.

As of the CCOD, 14 DLTs as defined in the protocol have been reported in 12 of 226 patients (5.3%). The following events were considered related to both RO6958688 and atezolizumab: Grade 4 cardiac arrest (100 mg RO6958688; 1 patient), Grade 4 dyspnea (160 mg RO6958688; 1 patient), 2 events of Grade 3 colitis (100 mg and 160 mg RO6958688; 2 patients), 2 events of Grade 3 ALT increased (150 mg and 160 mg RO6958688; 2 patients), Grade 3 AST increased (150 mg RO6958688; 1 patient), Grade 3 stomatitis (40mg RO6958688; 1 patient). The following events were considered related to RO6958688 only: Grade 5 hypovolemic shock (600 mg RO6958688; 1 patient), Grade 4 platelet count decreased (300 mg RO6958688; 1 patient), Grade 3 dyspnea (100 mg RO6958688; 1 patient), Grade 3 rash maculo-papular (120 mg RO6958688; 1 patient; incomplete 160 mg administration), Grade 1 diarrhea (150 mg RO6958688; 1 patient), and Grade 1 dysgeusia (100 mg RO6958688; 1 patient).

A Grade 5 event of hypovolemic shock related to RO6958688 was reported in a [redacted] year-old [redacted] patient with microsatellite unstable mCRC and hepatic, pulmonary and peritoneal lesions enrolled into the step-up cohort B1 (atezolizumab 1200 mg Q3W; RO6958688 40 to 1200 mg QW, followed by 1200 mg Q3W) in the WP29945 study. The patient had received two lines of prior standard therapy. Co-morbidities included

“Use of this document is governed by the terms of use on the first page of this document.”

ongoing hypertension and diabetes mellitus with history of [redacted] and [redacted]. The patient started the first cycle of RO6958688 + atezolizumab on [redacted]. The first 3 administrations (40-150-300 mg) of RO6958688 were associated with adverse events as well as lab abnormalities including constitutional, gastrointestinal, pulmonary symptoms and increased creatinine, liver function tests, hypoalbuminemia and thrombocytopenia. Due to these adverse events, the second cycle (fourth dose of RO6958688) was started with 4 weeks' delay on [redacted], consisting of 1200 mg atezolizumab and 600 mg RO6958688. Approximately 8 hours after the RO6958688 infusion, the patient developed several symptoms led by severe continuous diarrhea, partially responsive to corticosteroid treatment, with subsequent hypovolemic shock, hypotension, hypoxia, metabolic acidosis and renal failure leading to hyperkalemia. These symptoms initially improved with steroids and supportive care in the ICU. However, on [redacted], the patient experienced ventricular fibrillation, secondary to hyperkalemia per investigator, with successful electric cardioversion, but could not be resuscitated after the 2nd episode of ventricular fibrillation despite intensive care measures. This event was reported by the Investigator as a G5 hypovolemic shock related to RO6958688.

The risk mitigation measures were further optimized (in Section 5.2.6) following the receipt of two Grade 5 events reported as related to RO6958688 (Grade 5 IRR in BP29541 in Section 1.3.2.1.1 and Grade 5 Hypovolemic Shock in WP29945 in this section).

1.3.2.1.3 Additional Safety Findings: Infusion-Related Reactions and Cytokine Release Syndrome

As of the CCOD of 27 April 2018, IRRs were reported as a stand-alone preferred term in 105 of 148 patients (70.9%) in Study BP29541 and in 156 of 226 patients (69.0%) in Study WP29945. The majority was of Grade 1 or Grade 2 severity and readily manageable with protocol-recommended measures. However, 29 patients (19.6%) in Study BP29541 and 39 patients (17.3%) in Study WP29945 reported an IRR with a maximum intensity of Grade 3, and 1 patient in Study BP29541 experienced a Grade 5 IRR). Forty patients (27.0%) in Study BP29541 and 65 patients (28.8%) in Study WP29954 reported at least one serious IRR.

The incidence of IRR was highest at the first infusion. At this timepoint, the majority of patients were anti-drug antibody (ADA) negative and a comparable dose-dependent cytokine release was observed in ADA-negative patients and those who went on to become ADA positive (see Section 1.3.2.4). The cytokine release that is observed after the first infusion is expected based on the mechanism of action of RO6958688. At the second and subsequent infusions, the incidence of IRR decreased.

"Use of this document is governed by the terms of use on the first page of this document."

IRRs were also more frequent and of higher severity in ADA-positive patients compared with ADA-negative patients (median time to ADA onset is 2.3 weeks). IRRs in ADA-positive patients were more likely to be associated with clinical symptoms of cytokine release syndrome (CRS) (e.g., hypoxia and/or hypotension, as observed in the patient from Study BP29541 who experienced a grade 5 IRR). After the fourth infusion of RO6958688, cytokine release remained dose dependent and was higher in ADA-positive patients.

While cytokine peaks observed during initial administrations are thought to represent on-target, tumor-related effects, later cycle peaks likely represent T-cell activation by ADA-mediated crosslinking of CEA-TCB bound CD3.

Given that IRRs may be indistinguishable from CRS based on symptomatology, single-treatment management guidelines are being recommended for both IRRs and CRS, during or up to 24 hours after infusion of RO6958688. These include the use of tocilizumab as a rescue medication for CRS. See Section 5.2.6.2.4 for more information on IRR/CRS and Appendix 10 for management guidelines.

Please refer to the RO6958688 Investigator's Brochure for additional details on IRR/CRSs adverse events observed in clinical studies.

1.3.2.2 Efficacy of RO6958688

1.3.2.2.1 BP29541

As of 16 June 2017, tumor response assessment data using RECIST criteria v1.1 were available from 86 of 102 patients (84.3%) in Study BP29541. Of the 86 efficacy-evaluable patients, 5 patients treated with 0.052-2.5 mg RO6958688 QW were enrolled in Part I and 81 patients were enrolled in Part II of which 63 patients received flat doses of 2.5 mg-600 mg RO6958688 QW alone, 8 patients received step-up doses of 40-1200 mg RO6958688 QW (Cohorts A and B), and 10 patients received 135-300 mg RO6958688 QW following obinutuzumab pretreatment. Tumor responses were evaluated 8-12 weeks after treatment or at any unscheduled tumor response assessment by using RECIST criteria v1.1. Efficacy evaluable patients had at least one administration of RO6958688 and at least one tumor assessment after treatment start.

Partial response was observed in a total of 2 of 86 patients (2.3%) with CRC. One of 35 patients (2.9%) in the 60-600 mg RO6958688 dose group received a starting dose of 200 mg RO6958688 QW and was dose escalated to 300 mg with the longest PR observed at Day 94 (unscheduled visit following Cycle 8) accompanied by a 68% reduction of target lesions and 1 of 10 patients (10.0%) received obinutuzumab pretreatment followed by RO6958688 QW at a starting dose of 135 mg and was dose-escalated to 200 mg with the longest PR observed at Day 225 (Cycle 32) accompanied by a 61% reduction of target lesions. Progressive disease has been confirmed in both patients, after response durations of 94 and 224 days, respectively.

“Use of this document is governed by the terms of use on the first page of this document.”

Stable disease was observed as best overall response in a total of 30 of 86 (34.9%) patients which included 1 of 5 patients (20.0%) in Part I who received RO6958688 QW at a starting dose of 0.15 mg and escalated to 20 mg, 23 of 63 patients (36.5%) in Part II who received flat doses of 2.5-600 mg RO6958688 QW with 10 of 28 patients (35.7%) treated with 2.5-40 mg RO6958688 and 13 of 35 patients (37.1%) treated with 60-600 mg RO6958688, 3 of 10 patients (30.0%) in Part II who received 135-300 mg RO6958688 QW following obinutuzumab pretreatment, and 3 of 5 patients (60.0%) in Cohort A in Part II who received step-up doses of RO6958688 QW starting at 40 mg up to 1200 mg. The longest SD was at Day 441 (Cycle 64) observed in a patient dosed with RO6958688 QW at 60 mg and escalated up to 400 mg. This patient continued to have SD at the time of clinical cutoff date.

Forty-four patients (50.6%) had progressive disease as best overall response and 10 (11.6%) patients had missing or non-evaluable responses.

As of 14 July 2017, there were 2 confirmed partial responses (PR) by RECIST 1.1 out of 31 CRC MSS patients (7%) enrolled in the ≥ 60 mg QW RO6958688 and step up cohorts A and B. Two additional PR responses were confirmed after this latter cutoff date.

Please refer to the RO6958688 Investigator's Brochure for additional details on the efficacy of RO6958688.

1.3.2.2.2 WP29945

As of 16 June 2017, tumor response assessment data using RECIST criteria v1.1 were available for a total of 61 of 82 patients (74.4%) in Study WP29945. Of the 61 efficacy-evaluable patients, 2 patients received previous RO6958688 treatment in Study BP29541 prior to Cycle 1 Day 1 combination treatment of RO6958688 and atezolizumab; data from these 2 patients were analyzed and are presented separately. Efficacy evaluable patients received at least one dose of RO6958688 in combination with at least one dose of atezolizumab and had at least one tumor assessment after treatment start. Tumor responses were evaluated 8 weeks after treatment with 5–300 mg RO6958688 QW in combination with a fixed dose 1200 mg of atezolizumab Q3W or at any unscheduled tumor response assessment.

Of the 59 efficacy-evaluable patients not treated previously with RO6958688, PR was achieved as best overall response in 5 of 44 patients (11.4%) in the 80-300 mg RO6958688 dose group. All 5 patients received 160 mg RO6958688 QW in combination with 1200 mg atezolizumab Q3W. The longest PR was observed at Day 225 (unscheduled visit following Cycle 9) accompanied by 69% reduction of target lesions compared to baseline, and is ongoing at the time of clinical cutoff with a duration of response of 168 days. Additionally, 2 of these 5 patients have ongoing responses of 28 and 87 days duration at the time of clinical cutoff, respectively. The remaining 2 patients achieved a PR followed by progression of disease after 118 and 58 days, respectively.

“Use of this document is governed by the terms of use on the first page of this document.”

A total of 25 of 59 patients (42.4%) had SD as best overall response: 4 of 15 patients (33.3%) treated with 5-40 mg RO6958688 and 21 of 44 patients (47.7%) treated with 80-300 mg RO6958688 in combination with atezolizumab. The longest SD was observed at Day 337 (Cycle 17) in a patient who received RO6958688 QW at starting dose of 20 mg and escalated up to 160 mg in combination with atezolizumab. Twenty-six patients (44.1%) had progressive disease as best overall response and 3 patients (5.1%) had missing or non-evaluable responses.

Two patients with CRC who received previous RO6958688 treatment until disease progression in Study BP29541 prior to enrollment in Study WP29945, had SD as the best overall response following combination treatment of atezolizumab with escalating RO6958688 doses from 40 mg to 160 mg (1 patient) and a RO6958688 dose of 160 mg (1 patient) in Study WP29945.

As of 14 July 2017, there were 4 confirmed partial responses (PR) by RECIST 1.1 out of 31 CRC MSS patients (13%) enrolled in the 160 mg QW RO6958688 cohort. An additional PR response was confirmed after this latter cutoff date.

As of 11 January 2018, there were 5 confirmed partial responses (PR) by RECIST 1.1 out of 37 CRC MSS patients (13%) enrolled in the 160 mg QW RO6958688 cohort. In the 100 mg RO6958688 QW cohort, there were 2 confirmed partial responses (PR) by RECIST 1.1 out of 20 CRC MSS patients (10%) enrolled and 3 confirmed partial responses out of 19 CRC MSS patients (15.8%) enrolled in the 100 mg RO6958688 Q3W RO6958688 cohort. Disease control rate in the 160 mg QW RO6958688 cohort was 51.4%, in the 100 mg RO6958688 QW cohort the disease control rate was 55% and in the 100 mg Q3W RO6958688 cohort was 52.6%.

Please refer to the RO6958688 Investigator's Brochure for additional details on the efficacy of RO6958688.

1.3.2.3 Clinical Pharmacokinetics of RO6958688

As of 14 May 2018, PK data from a total of 65 ADA-negative patients from Study BP29541 and 80 ADA-negative patients from Study WP29945 were included in a population PK analysis. RO6958688 concentration time profiles exhibited a biphasic disposition with an initial rapid distribution phase followed by a slower elimination phase. In ADA–negative patients, PK was time independent, i.e., serum exposure was maintained after multiple doses. The median CL and V_{ss} values were 0.071 L/h and 0.059 L/h, 9.8 L and 9.4 L, respectively, in Studies BP29541 and WP29945.

RO6958688 CL does not depend on dose. Similarly, after the first infusion, maximum concentration (C_{max}) and area under the concentration time curve (AUC) values of the first dosing interval are dose proportional, i.e., RO6958688 exhibited linear pharmacokinetics.

“Use of this document is governed by the terms of use on the first page of this document.”

1.3.2.4 Immunogenicity of RO6958688

As of 7 June 2018, ADA data from 132 patients from Study BP29541 and 193 patients from WP29945 were available, while PK data of 142 and 226 patients were available. In ADA-positive patients, time dependent PK can occur after multiple IV infusions of RO6958688 with reduced or no detectable exposure.

In patients treated with RO6958688 as a single agent and without obinutuzumab pretreatment (Study BP29541) and RO6958688 in combination with atezolizumab (Study WP29945), the development of ADAs directed against RO6958688 was observed in 48% (51 of 106 patients) and 64% (123 of 193 patients), respectively; RO6958688 exposure was reduced below the limit of quantification at C_{max} in 26% (30 of 115 patients) and 16% (35 of 226 patients) of patients, respectively. Median time to onset of no detectable exposure was 5.0 and 7.1 weeks in Studies BP29541 and WP29945, respectively.

After obinutuzumab pretreatment in Study BP29541, 42% (11 of 26 patients) of patients were ADA-positive; of these, 9 of 11 patients (81.8%) had transient ADAs and in the remaining 2 patients the last available ADA sample was early, i.e., at 3 and 9 weeks after the first dose of study drug (RO6958688). The maximal observed ADA-titer of 270 in obinutuzumab pretreated patients was low as compared to 196,830 in non obinutuzumab pretreated patients and RO6958688 exposure was sustained with similar PK profiles to those in ADA-negative patients.

More detailed information can be found in the RO6958688 Investigator's Brochure.

1.4 BACKGROUND ON ATEZOLIZUMAB

Atezolizumab is a humanized immunoglobulin (Ig) G1 monoclonal antibody that targets PD-L1 and inhibits the interaction between PD-L1 and its receptors, PD-1 and B7-1 (also known as CD80), both of which function as inhibitory receptors expressed on T cells. Therapeutic blockade of PD L1 binding by atezolizumab has been shown to enhance the magnitude and quality of tumor specific T cell responses, resulting in improved anti-tumor activity (Fehrenbacher et al. 2016; Rosenberg et al. 2016). Atezolizumab has minimal binding to Fc receptors, thus eliminating detectable Fc effector function and associated antibody-mediated clearance of activated effector T cells.

Atezolizumab shows anti-tumor activity in both nonclinical models and cancer patients and is being investigated as a potential therapy in a wide variety of malignancies. Atezolizumab is being studied as a single agent in the advanced cancer and adjuvant therapy settings, as well as in combination with chemotherapy, targeted therapy, and cancer immunotherapy

"Use of this document is governed by the terms of use on the first page of this document."

Targeting the PD-L1 pathway with atezolizumab has demonstrated activity in patients with advanced malignancies who have failed standard-of-care therapies. Objective responses have been observed across a broad range of malignancies, including NSCLC, urothelial carcinoma, RCC, melanoma, colorectal cancer, head and neck cancer, gastric cancer, breast cancer, and sarcoma.

Atezolizumab is approved for the treatment of locally advanced or metastatic urothelial carcinoma, metastatic non-small cell lung cancer*, small-cell lung cancer, and triple-negative breast cancer*.

Refer to the Atezolizumab Investigator's Brochure for details on nonclinical and clinical studies.

1.4.1 Previous Non-Clinical Studies

The pharmacology, pharmacokinetics, and toxicology of atezolizumab have been investigated in several nonclinical studies. Comprehensive pharmacology, PK, and toxicology evaluations were performed with atezolizumab. The safety, pharmacokinetics, and toxicokinetics of atezolizumab were investigated in mice and cynomolgus monkeys to support IV administration and to aid in projecting the appropriate starting dose in humans. Given the similar binding of atezolizumab for cynomolgus monkey and human PD-L1, the cynomolgus monkey was selected as the primary and relevant nonclinical model for understanding the safety, pharmacokinetics, and toxicokinetics of atezolizumab. Overall, the nonclinical pharmacokinetics and toxicokinetics observed for atezolizumab supported entry into clinical studies, including providing adequate safety factors for the proposed Phase I starting doses. The results of the toxicology program are consistent with the anticipated pharmacologic activity of down-modulating the PD-L1/PD-1 pathway; heightened immune responses and the potential to increase immune-associated inflammatory lesions were identified as possible safety risks in patients.

For more detailed information, please refer to the Nonclinical Studies section of the atezolizumab IB.

1.4.2 Ongoing Clinical Studies

As of 17 May 2018, clinical data on atezolizumab as a single agent or in combination with chemotherapy or targeted agents are available from more than 20 studies as follows:

- Monotherapy: Studies JO28944, PCD4989g, GO29293 (hereinafter referred to as IMvigor210), GO28753 (hereinafter referred to as POPLAR), GO28754 (hereinafter referred to as BIRCH), GO28915 (hereinafter referred to as OAK), WO29074 (hereinafter referred to as IMmotion150, where Arm B evaluates atezolizumab monotherapy), GO29664, and GO29294 (hereinafter referred to as IMvigor211).

"Use of this document is governed by the terms of use on the first page of this document."

- Combination: Studies GP28328, GP28384, GP28363, GO29383, IMmotion150 (where Arm A evaluates atezolizumab in combination with bevacizumab), WO29637 (hereinafter referred to as IMmotion151), WP29158, GO29695, GO29754, GO29322, GO30140, GO29436, GO30139 (hereinafter referred to as IMpower150), and GO29437 (hereinafter referred to as IMpower131).

Additional safety information is also gleaned from the entire development program for atezolizumab. Details of all ongoing studies can be found in the atezolizumab IB.

1.4.2.1 Safety of Atezolizumab

As of 17 May 2018, an estimated > 16,000 patients with solid tumor and hematologic malignancies have received atezolizumab in clinical trial participation as a single agent or in combination with cytotoxic chemotherapy and/or targeted therapy.

Safety findings of single-agent atezolizumab across multiple tumor types in the clinical development program are consistent with the known mechanism of action of atezolizumab and the underlying disease. Overall, treatment with atezolizumab is well tolerated, with a manageable adverse event profile. Currently, no maximum tolerated dose, no dose-limiting toxicities (DLTs), and no clear dose-related trends in the incidence of adverse events (AEs) have been determined.

Among 3075 patients treated with single-agent atezolizumab for whom pooled safety data are available (see Section 5.6.2 of atezolizumab IB for details), the most commonly reported AEs (≥ 10%) include fatigue, decreased appetite, cough, nausea, dyspnea, constipation, diarrhea, pyrexia, vomiting, arthralgia, back pain, asthenia, anemia, pruritus, rash, headache, and peripheral edema.

The AEs observed with atezolizumab in combination with chemotherapy and/or targeted therapies are consistent with the known risks of the individual study treatment.

Atezolizumab-related AEs were comparable between patients who received atezolizumab monotherapy and those who were treated with atezolizumab in combination with targeted therapy and/or chemotherapy. There are no atezolizumab-related AEs that are exacerbated when used in combination with other agents.

Immune-mediated AEs are consistent with the role of the PD-L1/PD-1 pathway in regulating peripheral tolerance. Given the mechanism of action of atezolizumab, events associated with inflammation and/or *immune-mediated* AEs are closely monitored during the atezolizumab clinical program. *Immune-mediated* AEs associated with atezolizumab include pneumonitis, hepatitis, colitis, pancreatitis, diabetes mellitus, hypothyroidism, hyperthyroidism, adrenal insufficiency, hypophysitis, Guillain-Barré syndrome, myasthenic syndrome/myasthenia gravis, meningoencephalitis, myocarditis, *myositis,* and nephritis. These AEs are described in further detail in Sections 6.4 and 6.6 of the atezolizumab IB. Guidance regarding the management of *immune-mediated* AEs is provided in Appendix 11.

“Use of this document is governed by the terms of use on the first page of this document.”

Refer to the latest atezolizumab IB for a detailed discussion of available safety data from select studies in the atezolizumab clinical program.

1.4.2.2 Efficacy of Atezolizumab

As of 17 May 2018, efficacy data were most extensive for patients with non–small cell lung cancer (NSCLC; 3134 patients enrolled in Studies BIRCH, POPLAR, OAK, IMpower150, IMpower151, GO28625 [FIR, data not shown], and the NSCLC cohort of PCD4989g) and patients with metastatic urothelial carcinoma (mUC; 1104 efficacy-evaluable patients in studies PCD4989g, IMvigor210, and IMvigor211 who were administered atezolizumab.

Efficacy parameters (ORR in Cohort 1 [CCOD of 12 July 2017] and ORR, progression free survival [PFS], and OS in Cohort 2 [CCOD 12 July 2017]) observed in the mUC cohort of Phase II IMvigor210 study after an additional 10 months from the original CCOD, and 26 months of follow-up (Cohorts 1 and 2, respectively) were consistent with those obtained at primary analyses of each cohort. Similar response rates and consistency between primary and updated analyses were seen in the mUC cohort of Study PCD4989g. In both studies higher ORR results in the IC2/3 expression group suggest that higher levels of PD-L1 expression on ICs may be associated with increased benefit. The primary analysis of IMvigor211 (CCOD 13 March 2017) showed a clinically meaningful OS improvement for atezolizumab treatment compared to chemotherapy, but the results were not statistically significant. The results of IMvigor211 are consistent with those of IMvigor210.

The results from the 5 studies evaluating atezolizumab as monotherapy in patients with locally advanced or metastatic NSCLC (PCD4989g, FIR, OAK, POPLAR, and BIRCH) single-agent treatment with atezolizumab resulted in clinically meaningful OS improvement in the 2L/3L NSCLC ITT population, in comparison with standard of care, in both non-squamous and squamous histologies, and across all PD-L1 expression subgroups. Higher PD-L1 expression on TCs or ICs was associated with higher ORRs and longer median PFS and OS duration. Responses were highly durable across all PD-L1 expression groups.

Other available efficacy data suggested that treatment with atezolizumab as a single agent or in combination with other therapeutic agents resulted in anti-tumor activity across a range of other tumor types and hematologic malignancies (including pediatric-type tumors), across lines of therapy, and across PD-L1 expression subgroups.

For more detailed information, please refer to Section 5.5 of the atezolizumab IB.

"Use of this document is governed by the terms of use on the first page of this document."

1.4.2.3 Clinical Pharmacokinetics and Immunogenicity of Atezolizumab

There have been no dedicated clinical pharmacology studies conducted for atezolizumab. Atezolizumab pharmacokinetics and other data have been analyzed from the following atezolizumab monotherapy studies: PCD4989g, JO28944, IMvigor210, IMvigor211, BIRCH, POPLAR, FIR, and OAK. PK data are available from IMmotion150 and IMpower150 where atezolizumab has been dosed in combination with other anti-cancer agents.

The key PK findings from the above-listed atezolizumab monotherapy clinical studies are summarized below:

- The pharmacokinetics of atezolizumab monotherapy have been characterized in patients in Study PCD4989g at doses 0.01 mg/kg to 20 mg/kg q3w, including the fixed dose 1200 mg (equivalent to 15 mg/kg). Exposure to atezolizumab increased dose proportionally over the dose range of 1 mg/kg to 20 mg/kg. While a subset of ADA-positive patients in Study PCD4989g receiving 0.3 to 3 mg/kg atezolizumab q3w experienced a reduction of atezolizumab Cmin to below the PK assay lower limit of quantification (LOQ), patients receiving 10 to 20 mg/kg atezolizumab, including the fixed 1200 mg dose, maintained geometric mean Cmin that was in excess of both the LOQ and the target serum concentration of 6 μg/mL (Deng et al. 2016).
- A Phase I popPK analysis that included 472 patients from Studies PCD4989g and JO28944 described atezolizumab pharmacokinetics for the dose range 1–20 mg/kg with a linear two-compartment disposition model with first-order elimination. The popPK analysis indicated that central compartment volume of distribution (V1) was 3.28 L and the Vss was 6.91 L in the typical patient. Further, the CL of atezolizumab was 0.20 L/day and the t1/2 was 27 days. Steady state was obtained after 6 to 9 weeks (2 to 3 cycles) of repeated dosing. The systemic accumulation in AUC, Cmax, and Cmin was 1.91, 1.46, and 2.75-fold, respectively.
- Based on an analysis of exposure, safety, and efficacy data, the following factors had no clinically relevant effect: age (21– 89 years), body weight, sex, albumin levels, tumor burden, region or race, renal impairment, mild hepatic impairment, level of PD-L1 expression, or ECOG status. Positive ADA status against atezolizumab led to approximately 13% reduction in overall exposure.
- The effect of moderate or severe hepatic impairment on the pharmacokinetics of atezolizumab is unknown.
- In the Phase II, randomized Study IMmotion150 of atezolizumab administered as monotherapy or in combination with bevacizumab versus sunitinib in patients with untreated advanced RCC, a total of 202 patients have evaluable atezolizumab PK data. Atezolizumab concentrations in serum were consistent and stable over 295 days. No apparent PK DDI was observed when atezolizumab and bevacizumab were dosed in combination.

"Use of this document is governed by the terms of use on the first page of this document."

- In the Phase III, open-label, randomized Study IMpower150 to investigate the safety and efficacy of atezolizumab in combination with carboplatin + paclitaxel with or without bevacizumab compared with treatment with carboplatin + paclitaxel + bevacizumab in chemotherapy-naïve patients with Stage IV non-squamous NSCLC, the pharmacokinetics of atezolizumab in serum was available in 778 of 802 ITT patients (97%) with 4386 samples. The co-administration of bevacizumab or chemotherapy (carboplatin +paclitaxel) did not seem to influence atezolizumab pharmacokinetics.

See the atezolizumab IB for additional details on nonclinical and clinical studies.

1.5 BACKGROUND ON TOCILIZUMAB (RO4877533, ACTEMRA®, ROACTEMRA®)

Tocilizumab blocks IL-6 from binding to its receptor, both in membrane-bound and soluble states (Singh et al. 2011). With a primary indication for juvenile idiopathic arthritis (JIA), tocilizumab is approved by the Food and Drug Administration (FDA) for children as young as 2 years. It is also approved for adults with rheumatoid arthritis, adults with Giant Cell Arteritis (GCA) and for Castleman disease in Japan. Tocilizumab has been extensively studied in adults, with 8 randomized controlled trials treating more than 2000 patients (Singh et al. 2011) and in children in phase 1 to phase 3 trials for JIA (Woo et al. 2005, Yokota et al. 2005, De Benedetti et al. 2012).

In patients with severe CRS associated with T cell–engaging therapies, IL-6 levels peak during maximal T cell proliferation. A growing body of evidence suggests that IL-6 blockade by tocilizumab result in rapid, dramatic reversal of life-threatening CRS in patients treated with T cell engaging therapies (Grupp et al. 2013, Teachey et al. 2013). Whereas tocilizumab IV is typically dosed every 4 weeks in rheumatoid arthritis, extended treatment is not necessary in the management of CRS, which is self-limited and in most cases reported to require a single administration in order to control the clinical signs of CRS after treatment with T cell engaging agents (Le et al. 2018). Tocilizumab has been recently approved by the FDA (August 2017) and the European Medicines Agency has recently approved the extension of indication to include "treatment of chimeric antigen receptor (CAR) T cell-induced severe or life-threatening cytokine release syndrome (CRS) in adults and pediatric patients 2 years of age and older" (Tocilizumab USPI).

As part of the data review of BP29541 and WP29945, we observed a correlation between peaks of serum IL-6 and the occurrence of IRRs and associated symptoms of hypotension or hypoxia or dyspnea within 24 hours of RO6958688 infusion. However, the majority of these peaks are less than 1000 pg/mL. Given that hypotension and hypoxia are key symptoms of CRS, based on the current information, CRS may have occurred in some patients experiencing IRR with RO6958688 and underlying symptoms of hypotension and hypoxia. Tocilizumab should be administered for management of IRR and CRS considered related to RO6958688 per guidelines in Table 3.

"Use of this document is governed by the terms of use on the first page of this document."

1.6 STUDY RATIONALE AND BENEFIT–RISK ASSESSMENT

1.6.1 Combination of RO6958688 and Atezolizumab

Based on the biology and mode of action of RO6958688 and PD-L1/PD-1, RO6958688 is a promising combination partner for PD-L1/PD-1 pathway antagonists.

The rationale for the combination of RO6958688 with atezolizumab is to counteract one of the immune evasion mechanisms mediated by the suppressive PD-L1 /PD-1 pathway and unleash the full potential of T-cell activity against tumors.

RO6958688 is a novel TCB-immune engager that targets human CEA on tumor cells and CDε on T cells. RO6958688 recruits and engages T cells through simultaneous binding of the CD3ε subunit of the TCR complex and CEA, a tumor cell surface antigen. Crosslinking of T cells with tumor cells leads to T-cell activation and tumor cell killing. Subsequently, expansion of preexisting and recruitment of new T cells takes place, as well as upregulation of PD-L1/ PD-1 expression.

In nonclinical models, the binding of RO6958688 to CEA and CD3ε resulted in T cell–mediated killing of cancerous cells that express CEA. RO6958688 effectively mediated the killing of cancer cells only by concurrently binding to CEA present on tumor cells and to CD3ε present on T cells. Furthermore, RO6958688 demonstrated efficacy in non-inflamed and poorly T cell–infiltrated tumors and the ability to increase T-cell infiltration in tumors, thus converting non-inflamed PD-L1–negative tumors into highly inflamed and PD-L1–positive tumors, resulting in the generation of a more inflamed tumor microenvironment (Bacac et al. 2016) and suggesting that combining RO6958688 with the anti-PD-L1 antibody atezolizumab may help prevent immune evasion mediated by the PD-1/PD-L1 pathway.

In addition to the anti-cancer cytotoxic effect of RO6958688, it has been demonstrated that exposure to RO6958688 can lead to tumor inflammation and immune stimulatory cytokine release (e.g., IFN-γ, TNF-α, granzyme B, IL-2, IL-6, and IL-10). The presence of these stimulatory cytokines, in the presence of PD-L1 inhibition with atezolizumab, may increase the activation, proliferation, and function of endogenous anti-cancer T cells, generating an anti-cancer immune response to cancer antigens beyond CEA. The addition of atezolizumab to RO6958688 is predicted to not only enhance the anti-cancer cytotoxic effects of RO6958688 but also lead to stronger endogenous anti-cancer immunity, resulting in deeper, more durable responses and preventing immune escape that is mediated by CEA downregulation or loss of cancer cells.

In nonclinical models the combination with PD-L1 blocking antibody also showed enhanced anti-tumor efficacy (Bacac et al. 2016; Roche unpublished data). In addition, early promising activity of RO6958688 administered in combination with atezolizumab, has been observed in the current ongoing study WP29945 in heavily pretreated patients with CEA-expressing tumors.

“Use of this document is governed by the terms of use on the first page of this document.”

Data discussed above demonstrate that RO6958688 and atezolizumab act synergistically in their anti-cancer properties and collectively their combination could provide meaningful clinical benefit in patients with cancer. The available safety data for both classes of agents provides guidance for enhancing and monitoring the safety of patients in this trial.

Further details on nonclinical studies conducted with the combination of RO6958688 and atezolizumab are provided in the atezolizumab and RO6958688 single agent IBs, respectively.

1.6.2 Potential for Overlapping Toxicities with RO6958688 and Atezolizumab

The following adverse events are classified as identified risks associated with use of RO6958688 in combination with atezolizumab: conjunctivitis, diarrhea and colitis.

The following adverse events are potential risks associated with combination use of atezolizumab and RO6958688, based on the evidence from Study WP29945: IRR, dysphonia, paraesthesia, musculoskeletal pain, pulmonary events, hepatic events, and GI events (e.g., vomiting, nausea, and gastrointestinal pain).

For a full discussion of potential risks associated with the combination of atezolizumab and RO6958688, see also Section 6.5.3 of the most recent RO6958688 IB.

The available nonclinical, clinical and class safety data for the two agents, provide guidance for monitoring safety of patients in this trial. Details on the monitoring for the potential overlapping toxicities are provided in Appendix 10 and Appendix 11 of the protocol.

1.6.3 Rationale for [18F] FDG-PET Imaging

In this study, [^{18}F]-FDG-PET scans will be acquired at baseline and on-treatment in order to detect a PD effect on the tumor glucose metabolism. Patients with no evidence of FDG uptake at screening will not be required to undergo on-treatment FDG-PET. Assessment of FDG-PET will be performed centrally by an independent reviewer and per local site assessment.

1.6.4 Rationale for the New Dose Schedule

In a preclinical model imaging study with radiolabeled CEA CD3 TCB, tumor targeting was shown (Roche Report No.1072962). CEA CD3 TCB was retained in the tumor lesions for at least 120 hours (last time point of the study). Consistent with the above data, a clinical imaging study with a different labeled molecule (^{89}Zr CEA-IL2v) was undertaken. This drug targeted the same CEA epitope and utilizes the same CEA binding Complementary Determining Region (CDR). Tumor accumulation was observed at least until day 8, while over 90% of the antibody was cleared from serum within 4 days (ESMO 2015 oral presentation, Tabernero et al). The above data

“Use of this document is governed by the terms of use on the first page of this document.”

suggest that the retention of the antibody in the tumor lesions could be more relevant than the blood exposure to define the best schedule for this T cell bi-specific antibody.

As reported for other immunotherapies (Topalian 2012), the pharmacodynamic effect in the tumor of the combination of RO6958688 and atezolizumab may also last for a longer period of time than either the half-life of these two molecules and the retention in the tumor of RO6958688, especially as a core element of the MOA of RO6958688 is thought to be by mediated by T-cell activation, proliferation and cytokine release.

In order to confirm this hypothesis, and further explore optimization of dose and schedule as well as direct anti-tumor cytotoxicity and endogenous anti-cancer immunity, we propose to randomize patients into different dose schedules; a QW schedule, a Q3W schedule, and a step up dosing combining the QW and the Q3W RO6958688 administration schedules (to manage cycle 1 safety, optimize RO6958688-mediated anti-cancer cytotoxicity and target to minimize the impact of ADA on RO6958688 exposure).

The exploration of these different dose and schedules for the combination of RO6958688 and atezolizumab are intended to help identify a well-tolerated starting dose, a well-tolerated late cycle maximal dose, an appropriate step up dosing regimen, and associations between these parameters on both early efficacy and prolonged/durable efficacy.

The criteria for selection of the part II schedule and dose will be based on a composite of safety, efficacy and PK/PD.

2. OBJECTIVES

2.1 PRIMARY OBJECTIVES

The primary objectives of this study are:

- To establish the preliminary safety and tolerability profile of RO6958688 in combination with atezolizumab
- To determine the maximum-tolerated dose (MTD) in cycle 1 and in later cycles, if achieved, of RO6958688 in combination with atezolizumab
- To identify a recommended phase II dose and schedule (RP2D) of RO6958688 in combination with atezolizumab.

“Use of this document is governed by the terms of use on the first page of this document.”

2.2 SECONDARY OBJECTIVES

The secondary objectives for this study are:

- To describe the preliminary pharmacodynamic (PD) effects and duration of PD response for RO6958688 in combination with atezolizumab in mandatory paired tumor biopsies and paired blood samples on the basis of alterations in the quantity and quality of intratumoral T cells and peripheral blood cells (including but not limited to $CD3^+$, $CD4^+$, $CD8^+$ T cells, and other immune cells that might act as potential predictors of anti-tumor activity of RO6958688 in combination with atezolizumab)
- To describe the pharmacokinetics (PK) of RO6958688 and atezolizumab when administered in combination
- To obtain preliminary anti-tumor activity data of RO6958688 in combination with atezolizumab based on objective overall response rate (ORR), duration of response (DOR) and derived measures, disease control rate (DCR; defined as response rate [RR] + stable disease rate [SDR]), preliminary progression-free survival (PFS) and preliminary overall survival (OS) according to Response Evaluation Criteria in Solid Tumors (RECIST), Version 1.1 criteria and modified RECIST criteria, by investigator assessment for the whole study and by central assessment for prospective and retrospective analysis
- To estimate the PFS rate at relevant timepoints for RO6958688 in combination with atezolizumab

2.3 EXPLORATORY OBJECTIVES

The exploratory objectives for this study are:

- To explore the relationship between exposure, pharmacodynamics, metabolic activity of the tumor and clinical effects of RO6958688 when administered in combination with atezolizumab
- To explore the immunogenicity of RO6958688 when administered in combination with atezolizumab
- To explore the relationship of host and tumor genetic factors with PD or clinical response to therapy
- To investigate and define CEA expression in different solid tumors
- To investigate tumor mutations, gene expression and other biomarkers (such as CEA expression in various tumor indications) related to RO6958688 + atezolizumab combination therapy
- To characterize the natural growth of the tumor using tumor growth kinetics modeling
- To explore preliminary safety and efficacy in low/moderate and very low CEA expressing tumors

"Use of this document is governed by the terms of use on the first page of this document."

- To make a preliminary assessment of the effectiveness, PK and PD effects of tocilizumab (Actemra®/RoActemra®) in ameliorating the symptoms of severe CRS following RO6958688 treatment

Functional imaging will be assessed via [^{18}F]-FDG PET/CT centrally by an independent reviewer and per local site assessment. Uptake and retention of [^{18}F]-FDG will be measured by PET/CT imaging in all patients. Patients with no evidence of FDG uptake on screening PET scan will not be required to undergo follow-up studies.

3. STUDY DESIGN

Note: Following an internal review of the clinical development plan of RO6958688, the Sponsor has decided to permanently discontinue further enrollment of patients in this study and to not open the planned cohorts B2, C3, G1, G2 and G3 and the Biomarker cohort as well as Part II of the study.

3.1 DESCRIPTION OF STUDY

This is an open-label, multi-center, dose escalation and dose/schedule finding Phase Ib clinical study of RO6958688 in combination with atezolizumab. Each treatment cycle will be 21 days in duration and consists of IV infusions of RO6958688 given weekly (QW) (± 1 day) and/or every 3 weeks (Q3W) (± 2 days) in combination with atezolizumab given every 3 weeks (Q3W) (± 2 days). During the trial, if the clinical pharmacology and/or safety data support an alternate dose sequencing and or schedule, the above could be modified accordingly. Initially all patients in this trial will receive full dose of atezolizumab (1200 mg Q3W), however if this trial data supports, investigators in agreement with the Sponsor would be allowed to consider to delay the subsequent dose up to 105 days from the last one to prevent or to manage potential adverse events suspected to be related to the atezolizumab treatment.

The initial dose of atezolizumab will be delivered over 60 (± 15) minutes. If the first infusion is tolerated without infusion-related reaction (IRR), the second infusion may be delivered over 30 (± 10) minutes. If the 30-minute infusion is well tolerated, all subsequent infusions may be delivered over 30 (± 10) minutes. The first infusion of RO6958688 will be administered over a minimum of 2 hours, subsequent infusions should be administered in at least 4 hours during the dose/escalation phase. In patients with no Grade ≥ 2 IRR/CRS for more than two RO6958688 administrations, the infusion time can be progressively reduced to a minimum of 1 hour. For more details, please refer to the RO6958688 pharmacy manual. Patients will be treated until loss of clinical benefit, unacceptable toxicities, loss of RO6958688 exposure (in which case they can continue to receive atezolizumab alone), or withdrawal of consent. The treatment period for this protocol is 24 months for both RO6958688 and atezolizumab and may be modified if emerging data supports an alternative duration of therapy. In case one of the treatments is permanently discontinued, treatment with the other drug alone may be continued as long as the patient experiences clinical benefit in the opinion of the

“Use of this document is governed by the terms of use on the first page of this document.”

investigator or until unacceptable toxicity or symptomatic deterioration develops, which is attributed to disease progression as determined by the investigator and the Sponsor after an integrated assessment of radiographic data, biopsy results (if available), and clinical status, or withdrawal of consent.

3.1.1 Overview of Study Design

The study will be conducted in two parts (Figure 5). Part I is subdivided in Part IA: a dose escalation part and Part IB: dose/schedule finding. Part I objective is to evaluate the safety and determine the recommended dose and schedule of RO6958688 in combination with atezolizumab. Part II is an expansion part to confirm the safety and tolerability of the MTD dose (or OBD) as determined in Part I in order to define a RP2D and schedule of RO6958688 in combination with atezolizumab, and to explore preliminary antitumor activity, pharmacokinetic and pharmacodynamic effects.
Note: Part II has not and will not enroll any patients.

In Part IA, in order to overcome ADA and impact on PK, optional intra-patient dose escalation of RO6958688 to the next available tolerated dose level may be permitted depending on emerging clinical and safety data at the discretion of the treating physician, and after discussion with the patient. Intra-patient dose escalation may only proceed after patients have tolerated at least the first 3 consecutive doses of RO6958688 after discussion and alignment with the Medical Monitor.

“Use of this document is governed by the terms of use on the first page of this document.”

Figure 5 Study Schema

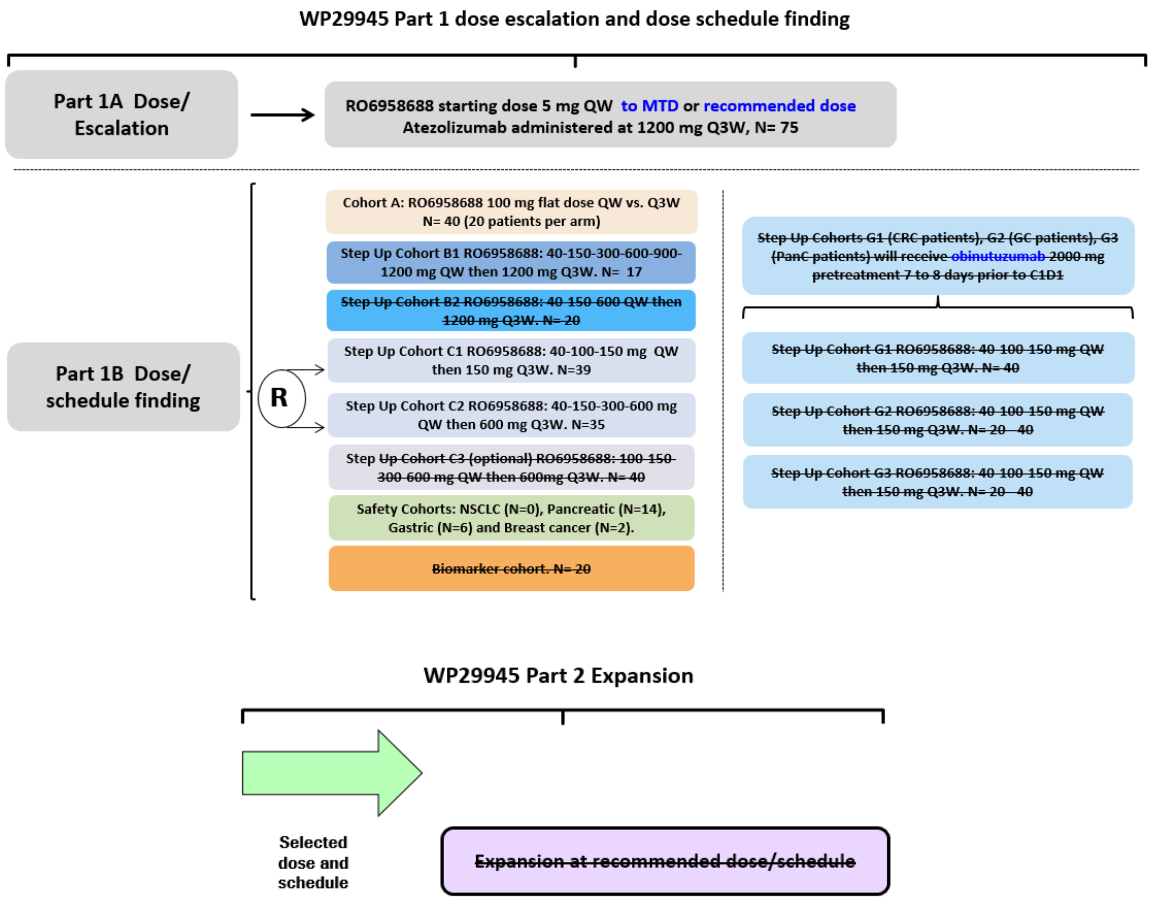

- Patient number (N) represents the actual number of patients that were enrolled in each cohort
- In part 1B: atezolizumab is administered 1200 mg Q3W
- Cohorts A and C will only enroll CRC MSS patients
- Cohort B1 will enroll CRC MSS patients and MSI^{hi} CRC patients
- Safety cohorts: RO6958688 will be administered: 40-150-300-600 mg QW then 600 mg Q3W, for the NSCLC safety cohort an alternative dose can be considered i.e., 40-100-150-300 mg QW then 600 mg Q3W

"Use of this document is governed by the terms of use on the first page of this document."

Part IA: Dose Escalation Part

Dose escalation of RO6958688 will be pursued according to a modified-Continual Reassessment Method with Overdose Control (mCRM with EWOC) design, aimed at reaching the MTD in cycle 1, which is defined as a dose with 20-30% probability of dose-limiting toxicity (DLT). For RO6958688, the starting dose is 5 mg administered in QW schedule, to be administered after the infusion of 1200 mg of atezolizumab when they are both administered on the same day (Day 1 of each cycle). For RO6958688, QW dosing will be implemented initially to generate data that can be analyzed to assess whether different dosing schedules are more effective. The RO6958688 dose will be escalated according to the mCRM, but will not exceed the RO6958688 MTD if defined in the BP29541 study. The atezolizumab dose is fixed at 1200 mg and is administered in Q3W schedule.

Patients within a cohort (at least 3 patients each) will be enrolled in a sequential manner, which, if required, can be expanded with additional patients to acquire additional safety, PK and PD data. Each patient will be observed for 21 days for DLT assessment. Enrollment will be staggered so that the first patient in each cohort will be observed for safety for 1 week before additional patients are enrolled in the cohort*. Once a minimum of 3 patients have completed the 21-day DLT observation period, the Sponsor and investigators will evaluate and agree on the dose, sequence, and timing for administration of both drugs for the subsequent cohort.

*In Spain the safety observation period between the first and subsequent patients enrolled in each cohort is 2 weeks.

Part IB: Dose/schedule finding

The first cohort (cohort A) in Part IB will compare the QW vs. Q3W schedules at a flat dose of 100 mg RO6958688 in combination with atezolizumab 1200 mg Q3W. This QW vs. Q3W schedule comparison will enroll approximately 20 to 40 randomized patients per arm.

Part IB will also explore RO6958688 step up dosing schedules in combination with atezolizumab 1200 mg Q3W.

For the step up dosing schedule, the late cycle MTD will be estimated by an intra-patient dose escalation design (see Protocol Section 6.7.1.2) in cohort B1. In this cohort, the RO6958688 dose will be escalated up to 300 % of the previous dose until the DLT criteria for that dose level are met. The dose of RO6958688 will not be escalated in the intra patient dose escalation above the monotherapy late cycle MTD of RO6958688 if defined in the BP29541 protocol.

Cohort B1 has enrolled 15 MSS CRC and 2 MSI-H CRC evaluable patients. One of the objectives of cohort B1 is to generate initial safety and efficacy data to compare the effect of treatment combination between MSS and MSI-H CRC patients. The second

“Use of this document is governed by the terms of use on the first page of this document.”

objective of cohort B1 is to define the late cycle MTD for RO6958688 in combination with atezolizumab. Note: No further patients will be enrolled in cohort B1.

The RO6958688 dose at C1D1 will be 40 mg followed by 150 mg at C1D8, 300 mg at C1D15, 600 mg at C2D1, 900 mg at C2D8 and 1200 mg at C2D15 and 1200mg at C3D1 then 1200 mg every 3 weeks (Q3W) thereafter (i.e., C4D1, C5D1…). The Q3W RO6958688 recommended dose will not exceed the late cycle MTD if defined or 1200 mg.

Cohort B2 will enroll approximately 20 MSS CRC evaluable patients. This new cohort is intended to explore a faster RO6958688 escalation in combination with atezolizumab (Q3W) in order to reach the target dose within the first atezolizumab cycle and prevent a potential early impact of anti-drug antibodies (ADA) on RO6958688 exposure within this first cycle and asses if a more rapid escalation may also increase anti-tumor activity of this combination. Enrollment in Cohort B2 can start at any time after the safety observation period of the 3rd patient in cohort B1 has been completed. Note: No patients have been enrolled into Cohort B2, and this cohort will not be opened for enrollment.

Cohort B1 will be used to define the late cycle MTD or if not defined, to assess 1200 mg as a safe dose.

The RO6958688 dose at C1D1 will be 40 mg followed by 150 mg at C1D8, 600 mg at C1D15, 1200 mg at C2D1 then 1200 mg every 3 weeks (Q3W) thereafter (i.e., C3D1, C4D1…). The Q3W RO6958688 recommended dose will not exceed the late cycle MTD if defined or 1200 mg.

Part IB of the study will also explore two additional RO6958688 step-up dose regimens (cohort C1 and C2) in combination with atezolizumab 1200 mg Q3W, in a randomized schedule comparison expansion. These randomized cohorts will start in parallel to the step up cohort B1.

Enrollment in cohorts B1, C1, and C2 will be staggered, the first patient in each cohort will be observed for safety for 1 week (2 weeks in Spain) before the second patient in each cohort is enrolled. A safety observation period of 1 week will be observed between the second and the third patient in each cohort, and from the third to subsequent patients. In the current study, we have not observed so far significant differences regarding the safety profile in patients with MSI-H tumors when compared to patients with MSS tumors; however, since only 4 MSI-H patients have been treated; in cohort B1 the same safety observation period will be applied to the first 3 MSI-H patients.

“Use of this document is governed by the terms of use on the first page of this document.”

In the randomized cohorts, RO6958688 will be administered as follows:

Cohort C1: The RO6958688 starting dose will be 40 mg, followed by 100 mg in C1D8, 150 mg in C1D15, 150 mg at C2D1 and 150 mg RO6958688 Q3W thereafter (i.e., C3D1, C4D1…).

Cohort C2: The RO6958688 starting dose will be 40 mg, followed by 150 mg in C1D8, 300 mg in C1D15, 600 mg at C2D1 and 600 mg (or late cycle MTD if defined) RO6958688 Q3W thereafter.

Based on the clinical data from the randomized cohorts (C1 and C2) the sponsor may consider opening a third cohort (cohort C3) with a starting dose of RO6958688 at 100 mg with mandatory prophylactic corticosteroids post dose at C1D1 (as detailed in Table 3), followed by 150 mg in C1D8, 300 mg in C1D15, and 600 mg at C2D1 and 600 mg (or late cycle MTD if defined) RO6958688 Q3W thereafter (i.e., C3D1, C4D1…). Note: No patients have been enrolled into Cohort C3, and this cohort will not be opened for enrollment.

The Q3W RO6958688 recommended dose for cohorts C1, C2 and the optional C3 cohort defined above, will not exceed the late cycle MTD if defined.

Each of the cohorts C1-2 and the optional cohort C3 will consist of approximately 40 locally advanced or metastatic (as defined by eligibility criteria #3) microsatellite stable (MSS) colorectal cancer patients. Approximately 80 patients will be randomized 1:1 to cohorts C1-2. Note: 39 CRC patients were enrolled in cohort C1, and 35 CRC patients were enrolled in cohort C2. No further patients will be enrolled into these cohorts, and no patients have or will be enrolled in cohort C3.

Additional safety cohorts in other solid tumor, including NSCLC, gastric, pancreatic and breast cancer will be explored (without formal estimation of maximum tolerated doses). These cohorts can be opened after the safety observation period (1 week) of the third patient in cohorts B1 or C has been completed. Note: 14 patients were enrolled in the pancreatic cohort, 6 patients were enrolled in the gastric cohort and 2 patients were enrolled in the breast cohort. No patients were enrolled in the NSCLC cohort. No further patients will be enrolled in the safety cohorts.

These safety cohorts will explore RO6958688 administered weekly (QW) at an initial dose of 40 mg followed by 150 mg in C1D8, 300 mg in C1D15, 600 mg at C2D1 and 600 mg (or MTD if defined) Q3W thereafter. Atezolizumab will be administered Q3W (1200 mg). Regarding lung cancer patients an alternative RO6958688 dose/schedule can be explored if supported by data (i.e., 40 mg at C1D1 followed by 100 mg at C1D8, 150 mg at C1D15, 300 mg at C2D1 and 600 mg every 3 weeks).

“Use of this document is governed by the terms of use on the first page of this document.”

Finally, once the safety observation period of the third patient in cohort B1 has been completed, the sponsor may explore the safety and preliminary efficacy of RO6958688 in combination with atezolizumab in a separate biomarker cohort of approximately 20 patients with very low/negative CEA expressing solid tumors. Very low/negative CEA expression is defined as those samples having < 20% of tumor cells with IHC1+or IHC0+. Patients enrolled in the biomarker cohort will follow the same dose/schedule as patients in cohort C1. Note: No patients have been enrolled into the biomarker cohort, and this cohort will not be opened for enrollment.

Once the late cycle MTD has been defined (see Protocol Section 6.7.1.2) or 1200 mg is declared safe in cohort B1, ongoing patients in the trial experiencing clinical benefit and showing >50% reduction of RO6958688 C_{max} and a PK profile consistent with ADA mediated decrease in exposure, can be progressively dose escalated up to the late cycle MTD or 1200 mg of RO6958688 after discussion with the Medical Monitor. However, the above optional dose escalation does not apply to patients enrolled in cohort C1 since this cohort has been designed to assess a potential biological dose.

The sponsor may consider opening additional obinutuzumab cohorts (G1 to G3). Patients participating in these cohorts will receive according to patient's and/or investigators convenience, either 2000 mg of obinutuzumab IV on Day-7 (+ 1 day) or 1000 mg of obinutuzumab IV on two consecutive days, Day-8 and Day-7 (+ 1 day) before C1D1 RO6958688 and atezolizumab administrations. Premedication will be given prior to each obinutuzumab dosing. For these patients, the baseline tumor biopsy will be taken before receiving the first dose of obinutuzumab and the on-treatment tumor biopsy remains unchanged. Note: No patients have been enrolled into the obinutuzumab cohorts (G1, G2, and G3), and these cohorts will not be opened for enrollment.

Cohort G1 (MSS CRC): approximately 40 patients with locally advanced or metastatic (as defined by eligibility criteria #3) microsatellite stable (MSS) colorectal cancer patients will be enrolled. The RO6958688 starting dose will be 40 mg, followed by 100 mg on C1D8, 150 mg on C1D15 and 150 mg RO6958688 Q3W thereafter (i.e., C3D1, C4D1…) in combination with 1200 mg atezolizumab Q3W.

Based on preliminary efficacy and safety data form Cohort G1 the sponsor may consider opening additional Cohorts for patients with gastric, pancreatic and other indications:

Cohort G2: approximately 40 patients with locally advanced or metastatic Gastric Cancer (as defined by eligibility criteria #3) will be enrolled.

The RO6958688 starting dose will be 40 mg, followed by 100 mg on C1D8, 150 mg on C1D15 and 150 mg RO6958688 Q3W thereafter (i.e., C3D1, C4D1…) in combination with 1200 mg atezolizumab Q3W.

"Use of this document is governed by the terms of use on the first page of this document."

Cohort G3: approximately 40 patents with locally advanced or metastatic Pancreatic Cancer (as defined by eligibility criteria #3) will be enrolled. The RO6958688 starting dose will be 40 mg, followed by 100 mg on C1D8, 150 mg on C1D15 and 150 mg RO6958688 Q3W thereafter (i.e., C3D1, C4D1…) in combination with 1200 mg atezolizumab Q3W.

Enrollment in Cohorts G1-G3 will be independently staggered, the first patient will be observed for safety for 1 week (2 weeks in Spain) before the second patient in each cohort is enrolled. A safety observation period of 1 week will be observed between the second and the third patient in each cohort, and from the third to subsequent patients.

For all patients who enroll in the study, tumor biopsies for PD analysis are mandatory at baseline and on-treatment, except for NSCLC patients for whom there is no accessible lesion. For patients continuing in the study, additional biopsies may be taken at the discretion of the investigator. These biopsies may serve to evaluate viable tumor given the mechanism of action of RO6958688 to induce tumor inflammation.

For patients discontinuing from the study due to disease progression, additional optional biopsies, if clinically feasible, may be taken to aid the understanding of immune resistance mechanisms.

Part II: Expansion Part

The expansion part of this study will be initiated once the recommended dose scheme has been determined. The purpose is to confirm the safety and tolerability of the recommended dose and schedule as determined in Part I and to explore preliminary antitumor activity, pharmacokinetic and pharmacodynamic effects. Based on the preliminary safety and efficacy data from Part I, the Sponsor could focus on other specific CEA expressing tumor indications for which to enroll a certain number of patients in Part II. Note: No patients have been enrolled in Part II of the study, and Part II will not be opened for enrollment.

Baseline and on-treatment tumor biopsies will be mandatory for all patients, except for NSCLC patients for whom there is no accessible lesion. DLT data will still be collected and might lead to refinement of the MTD definition for RO6958688 in combination with atezolizumab at the end of the trial.

3.1.2 Dose Escalation Decision Criteria

The decision to escalate to the next dose level will be made by the Sponsor and the participating investigators following review of all relevant safety information collected, including Adverse Events (AEs), ECGs, vital signs, clinical laboratory test results, and available pharmacokinetic data at the previous dose levels.

“Use of this document is governed by the terms of use on the first page of this document.”

3.1.2.1 Escalation Criteria (Part I) – Dose Limiting Toxicities

In Part IA of the study, the RO6958688 dose will start at 5 mg (QW) and will not be escalated higher than the RO6958688 MTD if defined in the BP29541 study (RO6958688 Phase I single agent study). The atezolizumab dose is fixed at 1200 mg and is administered in Q3W schedule. During the dose escalation (Part IA), patients who discontinue treatment before the end of the DLT period, for reasons other than DLTs, and patients who did not receive the assigned dose of RO6958688 (3 doses QW for the QW regimen) or atezolizumab (1 dose Q3W) during the DLT period, will be replaced to ensure that at least 3 patients in each cohort have been assessed for the full DLT period of 21 days prior to moving to the next dose level. During the step up dose escalation (Part IB), patients who discontinue treatment for reasons other than DLTs or receiving the 1200 mg dose or the highest possible safe dose according to the intra-patient dose escalation rules (the lower one applies), may be replaced in order to ensure that at least evaluable 6 patients will be available for estimating the late cycle MTD.

For the purpose of this study, a DLT will be defined as any of the following events attributed to RO6958688 (i.e., related to RO6958688) or/and to atezolizumab and occurring during the DLT period:

Hematological toxicities defined as:

- Grade ≥ 4 neutropenia (ANC < 500/μL) lasting ≥ 7 days
- Grade ≥ 3 febrile neutropenia
- Grade ≥ 4 thrombocytopenia lasting > 48 hours (recovery to ≤ Grade 2)
- Grade 3 thrombocytopenia associated with bleeding episodes

Any non-hematological toxicity ≥ Grade 3 including:

- Grade 3 hyperbilirubinemia lasting for > 48 hours or Grade 4
- Grade ≥ 3 AST/ALT elevations with hyperbilirubinemia of ≥ Grade 2
- Grade 4 AST/ALT elevations
- For patients with Grade 2 AST, ALT, and/or alkaline phosphatase abnormality at baseline, an increase to ≥ 10 × the upper limit of normal (ULN) that does not resolve to Grade ≤ 2 within 48 hours (if symptomatic) or that does not resolve to Grade ≤ 1 within 3 weeks of onset (if asymptomatic)

Failure to recover from any drug-related toxicity that results in a dose delay of ≥ 21 days (1 Cycle) is defined as a DLT.

The following are not considered DLTs:

- Grade 3 nausea, vomiting, diarrhea, colitis or enteritis that resolves to Grade ≤ 2 with or without treatment prior to the next planned infusion of RO6958688 (1 week)
- Grade 3 *immune-mediated* adverse event that resolves to Grade ≤ 1 with immunosuppressant therapy within 3 weeks of its onset
- Grade ≥ 3 fatigue that resolves to Grade ≤ 2 within 1 week

"Use of this document is governed by the terms of use on the first page of this document."

- Grade 3 arthralgia that can be adequately managed with supportive care or that resolves to Grade ≤ 2 within 1 week
- Fever > 40 degrees Celsius that occurs within 72 hours of RO6958688 infusion and resolves to Grade < 2 within 4 days and is resolved to Grade ≤ 1 within 10 days
- Fever ≥ Grade 3 that resolves to Grade ≤ 2 within 72 hours
- Grade 3 hypophosphatemia reversible to Grade < 2 within 1 week
- Grade ≥ 3 laboratory abnormality that is asymptomatic and deemed by the investigator not to be clinically significant
- Grade 3 autoimmune thyroiditis or other endocrine abnormality that can be managed by endocrine therapy or hormonal replacement
- Grade 3 tumor flare defined as local pain, irritation, or rash localized at sites of known or suspected tumor
- Alopecia (any grade)
- Grade 3 tumor pain that starts within 24 hours of infusion and resolves to Grade ≤ 2 within 1 week
- Grade 3 hypoxia that starts within 24 hours of infusion and resolves to Grade ≤ 2 within 1 week
- In patients with lung lesions, Grade 3 transient dyspnea secondary to localized lung edema that starts within 24 hours of infusion and recovers to Grade ≤ 2 or baseline within 1 week, and transient bronchospasm that resolves within 24 hours
- In patients with liver lesions, Grade 3 transient increase of bilirubin, transaminases and/ or Gamma GT that starts after infusion and recovers to Grade ≤ 2 or baseline within 1 week and grade 4 AST/ALT or grade 4 bilirubin increase that start after infusion and recovers to Grade ≤ 2 or baseline within 3 days.

Infusion related reactions (IRRs): IRRs are not considered to be DLTs because based on experience with monoclonal antibodies, IRRs are not dose-related events. Guidance on management of IRR/CRS and premedication that should be given is provided in sections 5.2.6 and 4.3.2.5, respectively.

3.1.2.2 Expansion Part (Part II)

The expansion part of this study will be initiated once the dose and schedule have been determined in Part I. The purpose is to confirm the safety and tolerability of the recommended dose and schedule as determined in Part I and to explore preliminary antitumor activity, pharmacokinetic and pharmacodynamic effects. DLT data will still be collected and might lead to refinement of the MTD definition for RO6958688 in combination with atezolizumab at the end of the trial. Note: No patients have been enrolled in Part II of the study, and Part II will not be opened for enrollment.

3.1.3 Communication Strategy

Upon completion of all screening evaluations and confirmation that a patient has met all of the inclusion and none of the exclusion criteria, investigator sites will contact the

"Use of this document is governed by the terms of use on the first page of this document."

Sponsor to confirm the patient number and cohort assignment (i.e., RO6958688 dose and schedule to be administered in combination with atezolizumab) via a Confirmation of Enrollment form (see Section 4.2.1). This will guarantee that the Sponsor is notified prior to the administration of RO6958688 and atezolizumab to any patient.

During Part I and Part II of this study, approximately 35 study sites will be involved for the enrollment of patients. The Sponsor and the sites will have frequent and detailed discussions regarding patient eligibility and patient care. After each patient receives RO6958688 in combination with atezolizumab (within 24 hours after study drugs infusions), the investigator must confirm to the Sponsor that the patient has received the doses and provide a brief summary of the status of the patient in terms of the safety and tolerability of RO6958688 in combination with atezolizumab, which will be communicated by email and/or telephone.

The investigator will contact the Sponsor immediately to discuss patient status and action(s) taken/to be taken in the event of a DLT during Part I and Part II of the study. In addition, as outlined above, in Part IA after each patient cohort (minimum of 3 patients) has been completed (i.e., the third patient in the cohort has reached Day 21), the Sponsor will organize a teleconference with the investigators to discuss the safety and tolerability of RO6958688 in combination with atezolizumab and to discuss the dose for the next cohort. The next dose level will be recommended using the EWOC design during the dose-escalation phase (Part IA) and discussed by the Sponsor and investigators. In addition, the clinical judgment of the Sponsor and investigators will also be utilized in the dose-selection process. This may lead to dose selections that differ from the mCRM recommendations if the scientific and clinical opinion is that a different dose would be more appropriate for patients. However, the dose selections cannot be higher than the 25% overdose probability dose estimated by the mCRM. During these teleconferences, toxicities according to National Cancer Institute Common Terminology Criteria for Adverse Events (NCI CTCAE) v4.03 will be discussed (CRS will be assessed based on NCI CTCAE v5 [Table 3]) along with the results of the available PK data in addition to safety laboratory results and any other available data that may assist the dose-escalation decision process. Dose escalation will only proceed to the higher dose level if the investigators and the Sponsor are satisfied with the safety profile of the previous patient cohort (i.e., no DLTs have been observed) and agree on the dose escalation. The discussion will be documented in writing by the Sponsor.

In addition to these communications, the Sponsor and investigators will be in regular contact throughout the study by email/telephone/fax as normal interactions during the conduct of a clinical study.

The Sponsor will be available 24 hours a day to discuss any medical or study-related issues that may arise during the conduct of this study.

“Use of this document is governed by the terms of use on the first page of this document.”

3.1.4 End of Study

The study will formally end once all patients have completed the safety follow-up visit, withdrawn from the study or when all patients have been enrolled in an extension study, whichever occurs last (the option to enroll in an extension study is for patients receiving atezolizumab only). The Sponsor may also decide to terminate the study at any time.

3.2 RATIONALE FOR STUDY DESIGN

3.2.1 Rationale for Dosage Selection

3.2.1.1 Starting Dose for RO6958688

For this study, the proposed starting dose for RO6958688 is 5 mg. This was selected on the basis of the preliminary Clinical, Safety and PD data from the EiH trial (BP29541) as summarized below. Dose escalation for Part I will follow the mCRM with EWOC design (see Section 3.2.5).

In the ongoing Phase I study BP29541 (safety cut-off: 17 August 2015), a total of 5 patients (Part I) have received at least three doses of RO6958688 (doses from 0.052 to 2.5 mg). These patients received subsequent doses up to either 2.5 mg, 5 mg and then 10 mg QW as allowed by the protocol. One of the patients in Part I continued to receive treatment (10 mg QW) by the time of the data cut-off. The number of treatment cycles received up to 17 August 2015 ranged from 3 to 29 cycles in Part I.

As of 17 August 2015, a total number of 16 patients (Part II) have received a RO6958688 dose up to 20 mg QW. By the time of the DLT data cut-off, 13 out of 16 patients in Part II continued to receive RO6958688 among who some had their initial dose escalated to 10 mg as per protocol. The number of treatment cycles received until the IB Version 2 cut-off date (12 June 2015) ranged from 4 to 18 cycles in Part II.

The safety profile, PK and the immune-related PD effect in the tumor and in peripheral blood has been analyzed and defined with full available data (cut-off date: 07 September 2015) to justify the starting dose.

As of DLT cut-off 17 August 2015, RO6958688 was well tolerated and none of the 21 patients (5 in Part I and 16 in Part II) experienced any DLT. A total of 212 AEs, including 7 SAEs, were reported in 21 patients enrolled in the ongoing Study BP29541. Five SAEs (two Grade 1 and three Grade 2) were assessed to be related to RO6958688.

At 5 mg QW we have observed signs suggesting biological activity in the CT scans and in the FDG PET scans of some patients. This dose was safe and well tolerated with no G3-G4 related safety events reported, no DLTs were reported at 5 mg neither at the two subsequent dose level cohorts, 10 mg and 20 mg. Considering all the above we propose 5 mg as the appropriate starting dose for this Phase Ib trial.

An overview of the safety profile of RO6958688 is provided in Table 1.

"Use of this document is governed by the terms of use on the first page of this document."

Table 1 Overall Safety Profile of RO6958688 (Ongoing Study BP29541)

	Part I (SAD) 0.052 – 2.5 mg (N = 5)	Part II (MAD) 2.5 – 20 mg (N = 16)	Total 0.052 – 20 mg (N = 21)
**Total number of events** [a]			
AEs	58	154	212
SAEs	2	5	7
Deaths	2	1	3
**Number of patients with at least one event** [b]			
AE	5 (100%)	15 (93.7%)	20 (95.2%)
Related AE	2 (40.0%)	12 (75.0%)	14 (66.6%)
AE of Grade ≥ 3	2 (40.0%)	3 (18.7%)0	5 (23.8%)
SAE	2 (40.0%)	5 (31.2%)	7 (33.3%)
Related SAE	0	5 (31.2%)	5 (23.8%)
AE with fatal outcome	1 (20.0%)	0	1 (4.7%)
AE leading to withdrawal from treatment	1 (20.0%)	0	1 (4.7%)
AE leading to dose modification/interruption	1 (20.0%)	3 (18.7%)0	4 (19.0%)

AE = adverse event; MAD = multiple ascending dose; SAD = single ascending dose; SAE = serious adverse event.

Note: Percentages are based on N in the column headings of each part.

Data cut-off: 17 August 2015 (using data from patients who had received at least two complete cycles of study treatment).

[a] Multiple occurrences of the same AE in an individual are counted only once.

[b] Multiple occurrences of the same AE in an individual are counted separately.

Most patients experienced at least one AE (20/21 [95.2%]). The most frequent AE was pyrexia (11/21 [52.4%]), followed by nausea (7/21 [33.3%]), diarrhea and cough (5/21 [23.8%] each), anemia, hyponatremia, asthenia and aspartate aminotransferase increased (4/21 [19.0%] each), and hypocalcemia and headache (3/21 [14.3%] each). Pyrexia occurred at dose levels of ≥ 1.3 mg. The remaining AEs affected 2 or less patients.

Pharmacokinetics overview/summary

As of 07 September 2015, following single IV infusion of RO6958688, the serum concentrations analyzed by NCA suggest non-linear PK. However, available PK data were not sufficient to conclude on the dose-proportionality of exposure. In Part I, exposure (C_{max} and AUC_{last}) was highly variable as based on single-patient cohorts; while in Part II, exposure (C_{max} and AUC_{last}) increased approximately dose-proportionally with the dose increasing from 2.5 to 10 mg.

“Use of this document is governed by the terms of use on the first page of this document.”

The terminal elimination half-life was in the range of 35.5–103.2 h following single IV infusion of RO6958688. The apparent volume of distribution ranged from 2.0 to 6.7 L, which approximates the serum volume and indicates that distribution is largely restricted to plasma and interstitial fluid, which is consistent with monoclonal antibodies.

Following multiple IV infusions of RO6958688, time-dependent PK was observed in some patients, with total loss of exposure in 3 patients enrolled in Part I (initially treated at 0.15, 1.3, 2.5 mg QW, respectively), 7 patients enrolled in Part II (dosed initially with 2.5, 5 and 10 mg) and partial reduction of exposure in 2 patients enrolled in Part II initially treated at 5 mg. In all 12 patients with reduced exposure, ADAs against RO6958688 were detected with first appearance occurring between Cycle 2 and Cycle 6 (pre-infusion). ADAs are likely to interfere with the bi-functional bio-analytical assay suggesting a decrease in active drug, or an accelerated clearance of RO6958688 via drug-ADA complexes.

3.2.1.2 Dose of Atezolizumab

The dose and schedule of atezolizumab in this study will be at a flat dose of 1200 mg (equivalent to an average body weight−based dose of 15 mg/kg) IV administered Q3W.

The target exposure for atezolizumab was projected on the basis of nonclinical tissue distribution data in tumor-bearing mice, target-receptor occupancy in the tumor, observed atezolizumab interim PK in humans, and other factors. The target trough concentration (C_{trough}) was determined on the basis of several assumptions, including: 1) 95% tumor-receptor saturation is needed for efficacy; 2) the tumor-interstitial concentration to plasma ratio is 0.30 based on tissue distribution data in tumor-bearing mice.

The atezolizumab dose is also informed by available clinical activity, safety, PK, and immunogenicity data. Anti-tumor activity has been observed across doses from 1–20 mg/kg administered Q3W. The MTD of atezolizumab was not reached and no DLTs have been observed at any dose in the above 7 Studies mentioned. Available preliminary PK data (0.03–20 mg/kg) from study PCD4989g suggest that for doses $\geq$ 1 mg/kg Q3W, overall atezolizumab exhibits pharmacokinetics that are both linear and consistent with typical IgG_1 antibodies. Detectable ADAs were observed in patients at all dose levels but were associated with changes in PK for some patients in only the lower dose cohorts (0.3, 1, and 3 mg/kg). It is unclear from currently available data in these lower dose cohorts whether administration of higher doses to patients with both detectable ADAs and reduced exposure would necessarily restore exposure to expected levels. No clear relationship between the development of measurable ADAs and safety or efficacy has been observed. Available data suggest that development of detectable ADAs does not appear to have a significant impact on PK for doses from 10–20 mg/kg administered Q3W in most patients. Correspondingly, patients dosed at the 10-, 15-, and 20-mg/kg Q3W dose levels have maintained target trough levels of drug despite the detection of ADAs.

"Use of this document is governed by the terms of use on the first page of this document."

Currently available PK and ADA data suggest that the 15-mg/kg atezolizumab Q3W regimen for Phase II and Phase III studies would be sufficient to both maintain concentrations $\geq C_{trough}$ and further safeguard against both inter-patient variability and potential effect of ADAs that could lead to sub-therapeutic levels of atezolizumab relative to the 10-mg/kg atezolizumab Q3W regimen. From inspection of available observed C_{trough} data, moving further to the 20 mg/kg atezolizumab Q3W regimen does not appear to be warranted to maintain targeted C_{trough} levels relative to the proposed 15-mg/kg atezolizumab Q3W level.

Simulations (Bai et al. 2012) do not suggest any clinically meaningful differences in exposure following a fixed dose or dose adjusted for weight. On the basis of this analysis, a fixed dose of 1200 mg q3W was selected (equivalent to a body weight–based dose of 15 mg/kg Q3W), this dose is the one generally used in most of the studies with atezolizumab.

3.2.2 Rationale for the Treatment of Severe Cytokine Release Syndrome (CRS) Using Tocilizumab

CRS is a potentially life-threatening symptom complex, caused by the excessive release of cytokines by immune effector or target cells during an exaggerated and sustained immune response. CRS can be triggered by a variety of factors, including infection with virulent pathogens, or by medications that activate or enhance the immune response, resulting in a pronounced and uncontrolled immune response.

Regardless of the inciting agent, severe or life-threatening CRS is a medical emergency. If unsuccessfully managed, it can result in significant disability or fatal outcome. Current clinical management focuses on treating the individual signs and symptoms, providing supportive care, and attempting to dampen down the inflammatory response using high dose corticosteroids. However, this approach is not always successful, especially in the case of late intervention.

CRS is associated with elevations in a wide array of cytokines, including marked elevations in IFN-γ, IL-6, and TNF-α levels. Emerging evidence implicates IL-6 as a central mediator in CRS. IL-6 is a pro-inflammatory multi-functional cytokine produced by a variety of cell types, which has been shown to be involved in a diverse array of physiological processes, including T cell activation. Regardless of the inciting agent, CRS is associated with high IL-6 levels (Panelli et al. 2004, Lee et al. 2014, Doessegger and Banholzer 2015), and IL-6 correlates with the severity of CRS with patients who experience severe or life-threatening CRS (CTCAE Grades 4 or 5) having much higher IL-6 levels compared to their counterparts who do not experience CRS or experience milder CRS reactions (CTCAE Grades 0−3) (Chen et al. 2016). Based upon this evidence, IL-6 levels will be measured at select timepoints in the study, per the SoA, via central laboratory testing (see Appendix 1 and Appendix 12).

"Use of this document is governed by the terms of use on the first page of this document."

As mentioned in Section 1.5, as part of the data review of BP29541 and WP29945, we observed a correlation between peaks of serum IL-6 and the occurrence of IRRs and associated symptoms of hypotension or hypoxia or dyspnea within 24 hours of RO6958688 infusion. However, the majority of these peaks are less than 1000 pg/mL. Given that hypotension and hypoxia are key symptoms of CRS, based on the current information, CRS may have occurred in some patients experiencing IRR with RO6958688 and underlying symptoms of hypotension and hypoxia. A patient with Grade 5 IRR, described in Section 5.2.6.2.4, had documented very high cytokine levels including IL6 at one of the previous RO6958688. Also, 2 other patients described in Section 5.2.6.2.4, have been successfully treated with Tocilizumab, further supporting the use of Tocilizumab.

Tocilizumab (Actemra®/RoActemra®) is a recombinant, humanized, anti-human monoclonal antibody directed against soluble and membrane-bound IL-6R, which inhibits IL-6 mediated signaling. Blocking the inflammatory action of IL-6 using tocilizumab could therefore represent a novel approach for the treatment of CRS. Refer to the Tocilizumab Investigator's Brochure for additional preclinical and clinical information regarding tocilizumab.

CRS has classically been associated with therapeutic mAb infusions, most notably anti-CD3 (OKT3), anti-CD52 (alemtuzumab), anti-CD20 (rituximab) and the CD28 super-agonist TGN1412 (Lee et. al 2014). CRS is also observed with T-cell recruiting therapies, including chimeric antigen receptor (CAR)-T cell therapy and bispecific molecules, such as blinatumomab (Nagorsen et al. 2012). Tocilizumab has been recently approved by the FDA (August 2017) and received a positive CHMP opinion (European Medicines Agency 2018) for a restricted use, limited to Cytokine Release Syndrome (CRS) induced by chimeric antigen receptor (CAR) T cell in adults and pediatric patients 2 years of age and older (Tocilizumab USPI).

Taken together, these findings indicate that patients treated with RO6958688 who develop severe CRS may benefit from tocilizumab therapy. Section 5.2.6.2.4 describes recommendations regarding the management of CRS considered related to RO6958688.

The dose of 8 mg/kg (suitable for patients at or above 30 kg weight) recommended in this protocol to treat severe CRS is the one approved by the FDA for the management of (CAR)-T cells related CRS (refer to the Tocilizumab IB for details).

3.2.3 Rationale for Study Population

This study is designed to obtain safety data for the combination of RO6958688 with atezolizumab in the treatment of patients with locally advanced and/or metastatic CEA-positive solid tumors who have progressed on standard treatment or who are intolerant to standard treatment.

"Use of this document is governed by the terms of use on the first page of this document."

Based on the preliminary safety and efficacy data from Part I, the Sponsor may focus on specific tumor indications for which to enroll a certain number of patients in Part II.

RO6958688 was designed to target tumors that express CEA and concomitantly engage immune cells to lead to their activation and proliferation, which culminates in tumor lysis. Therefore, a higher activity is expected in patients with CEA-expressing tumors.

Combinations of treatments may also have the potential to expand therapy to patients least likely to respond to atezolizumab. Whereas responses to single-agent atezolizumab in PD-L1−negative patients are less frequent than in PD-L1−positive patients, early correlative studies suggest limited association between PD-L1 expression and response to complimentary immunotherapy, e.g., ipilimumab and PD-1 blockade (Callahan et al. 2013).

3.2.4 Rationale for Biomarker Assessments

RO6958688 was designed to target tumors that express CEA and to concomitantly engage immune cells to lead to their activation and proliferation, which culminates in tumor lysis. Nonclinical studies have confirmed the expected MoA of RO6958688 by mediating tumor killing, induction of T-cell activation markers (CD25 and CD69), cytokine release (IFNγ, TNFα, granzyme B, IL-2, IL-6, and IL-10), and proliferation of T cells. Furthermore, the MoA of RO6958688 occurs only if simultaneous binding (cross-linking) of T cells to CEA-expressing tumor cells takes place. Moreover, the PD readouts from the limited number of patients tested thus far, confirm some of the preclinical observations.

Therefore, samples for exploratory and pharmacodynamic analyses will be collected in the study in order to assess immune cell alterations both in the peripheral blood and tumor tissue. In particular, archival tumor tissue is to be obtained from all patients, if available, in order to perform CEA assessment for patient eligibility. If no archival tissue is available, CEA assessment will be performed with the use of the fresh baseline tumor biopsy. Archival tumor tissue, if available, will be obtained from all patients to further assess exploratory biomarkers related to the PD effects of treatment, including but not limited to the expression of tumour PD-L1, etc.

Tumor biopsies will be performed on two occasions, (once during pre-treatment and once during treatment with RO6958688 and atezolizumab), and on each occasion, two biopsy samples will be collected. These tumor biopsies for PD analyses are mandatory at baseline and on-treatment at (see Section 3.3.2.2), except for NSCLC patients for whom there is no accessible lesion. For patients discontinuing from the study due to disease progression, additional optional biopsies, if clinically feasible, may be taken to aid the understanding of immune resistance mechanisms. If preliminary data suggest that modification of the on-treatment tumor biopsy timepoint would be more appropriate, alternative on-treatment tumor biopsy timepoints could be considered in the future

“Use of this document is governed by the terms of use on the first page of this document.”

cohorts. The biopsies will be taken from accessible, “non-critical” tumor locations, including, but not limited to, skin, lymph node, rectum, liver, etc.

Tumor biopsies will be used for the assessment of different biomarkers, including but not limited to mutational signatures and tumor mutational load.

It is expected that upon treatment, the number and activation/differentiation status of intratumoral immune cells as well as their location will change. Such changes will be determined by flow cytometric and/or immunohistochemical (IHC) methods and will be analyzed centrally. Changes such as the density of different immune cell lineages (including but not limited to CD4+ T cells, CD8+ T cells, B cells, NK cells, and macrophages) and their activation and differentiation status (including but not limited to CD25, Ki67, and PD1) will be examined. The expression of tumor markers (including but not limited to PD-L1, IDO etc..) will also be examined. In addition, analysis of gene expression, genetic alteration and the infiltrating T cell repertoire may be carried out.

If available, an assessment of tumor growth kinetics will be made by comparing post-treatment scans with the last available pre-study scan.

Blood samples will be collected and analyzed with respect to alterations in the number and activation and differentiation of immune cells as a consequence to treatment with RO6958688. The samples will be analyzed by flow cytometry, and the number of cells that belongs to lymphocyte subsets (CD4+ T cells, CD8+ T cells, NK cells, B cells, and monocytes) and their activation and differentiation status will be determined (including but not limited to CD25, Ki67, and PD1).

Administration of therapeutic antibodies is frequently associated with IRRs and cytokine release. RO6958688 is expected to bind to T cells, which may lead to the release of cytokines as a response to immune cell activation. This effect might be exacerbated by concurrent administration of atezolizumab. Therefore, cytokines, including but not limited to IL-6, IFNγ, TNFα, and inflammation markers, will be assessed in serum or plasma samples. These measurements also represent safety measures.

Additional immune PD biomarkers that are related to the MoA of RO6958688 in combination with atezolizumab may also be analyzed from serum or plasma samples taken from all patients. Such PD measures will be considered as disease-monitoring markers and include but are not limited to sCEA. The specimens will be used for research purposes to identify biomarkers that are useful to predict and monitor response to RO6958688 and atezolizumab treatment and safety, assess PD effects of treatment, and investigate the mechanism of resistance to therapy.

“Use of this document is governed by the terms of use on the first page of this document.”

3.2.5 Rationale for Statistical Design

The mCRM with EWOC design uses a statistical model that actively seeks a dose-level close to the MTD by using toxicity data from all enrolled evaluable patients to compute a precise dose-toxicity curve. It locates the MTD efficiently and minimizes the number of patients treated at possibly pharmacologically inactive dose levels. Such model-based designs have been successfully applied in many Phase I dose escalation studies (Bailey et al. 2009; Le Tourneau et al. 2009). The operating characteristics (see Table 5) from extensive simulations are used to show that the mCRM provides more accurate estimates of the true probability of toxicity and provides an estimate of the MTD, which lies within the true targeted interval, without exposing patients to undue risk of toxicity. The mCRM design is known to have better chances than the “3 + 3” design of selecting an MTD within the target toxicity interval, while keeping both the chances of under- and over-dosing at a desired minimum. The more classical “3 + 3” design is known to have a tendency to under-dose (Kang and Ahn 2001, 2002) and typically leads to dose estimates with far less than 30% toxicity rate. Furthermore, the mCRM provides information on the whole observed toxicity curve. It can be run incrementally at any timepoint for decision-making and, ultimately, it accounts for all the data accrued until that point (not only the last 3 patients as in the “3 + 3”). In the current design, an extension of the mCRM allows for continuous doses over a pre-specified range. Stopping criteria are made explicit and different dose-increments depending on the dose-interval can be pre-specified for an optimal tailoring of the design to the experimental therapy being tested.

The intra-patient dose escalation design for the determination of the late cycle MTD has shown acceptable operating characteristics in simulations (Appendix 7). The Sponsor is using this simple design, since model-based dose escalation designs for intra-patient dose escalations are complex and not standard use in clinical trials.

The schedule comparison expansion (cohort A) is designed to compare dose schemes in a randomized way in order to balance cohorts on important confounders such as tumor lesion location and overall tumor burden. For cohort A, the sample size of 20 patients randomized to each dose scheme has been selected in order to obtain initial descriptive statistics on the primary safety and efficacy endpoints for the two dose schemes, which allows for a reasonably precise differentiation between the dose schemes. Specifically, an observed difference of ca. 10% points in ORR would lead to approximately 80% posterior probability of a true response rate difference between two dose schemes. For example, if the number of objective responses is 4 in one and 2 in the other dose scheme out of 20 patients each, then the posterior probability of a positive response rate difference is 79.5%. For cohort C, the randomization of 40 patients to each of the arms C1, C2 and possibly 40 patients enrolled in a later opened arm C3, allows for this reasonably precise differentiation between the dose schemes.

“Use of this document is governed by the terms of use on the first page of this document.”

3.3 OUTCOME MEASURES

3.3.1 Safety Outcome Measures

The safety outcome measures for this study are:

- Incidence and nature of DLTs
- Incidence and severity of adverse events and IRRs and CRS symptoms
- Incidence of laboratory abnormalities (as examples and not limited to: hematology testing, coagulation, serum chemistries including liver enzymes, and urinalysis)
- Incidence of ADAs (anti-atezolizumab antibodies and anti-RO6958688 antibodies) formation, detection of cytokine release and potential correlation with PK, PD, safety, and efficacy parameters
- Incidence of autoantibodies (anti-nuclear antibody, anti-double-stranded DNA, cytoplasmic anti-neutrophil cytoplasmic antibody, and perinuclear anti-neutrophil cytoplasmic antibody) in comparison to baseline
- Changes in vital signs, physical findings and ECG findings.

All patients who participate in the study will be clinically evaluated at screening and on a regular basis during the entire course of the study. The routine safety monitoring plan will include clinical examination, vital signs assessment (including body temperature, respiratory rate, heart rate, diastolic and systolic BP, oxygen saturation measured by pulse oximeter – finger clip), ECG, laboratory analysis (hematology, coagulation, biochemistry, urinalysis, presence of autoantibodies), and regular collection and review of the reported adverse events.

The National Cancer Institute Common Terminology Criteria for Adverse Events v4.03 will be used to evaluate the clinical safety of the treatment in this study (http://evs.nci.nih.gov/ftp1/CTCAE/CTCAE_4.03_2010-06-14_QuickReference_8.5x11.pdf). Patients will be assessed for adverse events at each clinical visit and as necessary throughout the study. CRS severity will be graded according to the CTCAE v5 (Table 3).

Safety will be determined, but not limited to, by the spontaneous reporting of adverse events; by the assessments of routine laboratory values (hematology testing, serum chemistries); findings on physical examinations; ECGs; chest X-ray; vital signs; by carefully observing patients for IRRs; by the determination of ADAs.

Please refer to schedule of assessments for details on collection time of the assessments outlined below.

Laboratory Tests

Hematology and biochemistry will be analyzed at least prior to RO6958688 and atezolizumab administration as part of the regular safety assessments.

“Use of this document is governed by the terms of use on the first page of this document.”

1. Hematology: erythrocytes, hemoglobin, hematocrit, platelets, leucocytes and differential count (i.e., neutrophils, eosinophils, basophils, monocytes, lymphocytes).
2. Coagulation: prothrombin time (PT)/international normalized ratio (INR) and partial thromboplastin time (PTT) and fibrinogen. Additional coagulation parameters (i.e., first chromogenic antithrombin III then antigenic antithrombin III in case of chromogenic antithrombin III decrease in order to determine type 1 and type 2 antithrombin deficiencies. If a deficiency is detected, both antithrombin tests will need to be repeated at a later date/visit to confirm test findings, fibrinogen, prothrombin time, fibrin degradation products, D-dimer) could be assessed according to clinical judgment, or if any of the above parameter cannot be assessed locally.
3. Blood biochemistry: sodium, potassium, chloride, calcium, phosphate, magnesium, urea, creatinine, normal glomerular filtration rate (by CKD-EPI equation), total protein, albumin, glucose, total and direct bilirubin, alkaline phosphatase (ALP), alanine aminotransferase (ALT), aspartate aminotransferase (AST), lactate dehydrogenase (LDH), γ-glutamyl transferase (GGT), C reactive protein (CRP). NS cholesterol (total, LDL cholesterol, HDL cholesterol), triglycerides, Thyroid-stimulating hormone (TSH), free T3 (or total T3 for sites where free T3 is not performed) and T4, ferritin, soluble CD25.

   Soluble CEA (sCEA) measured in serum or plasma, will also be measured as a disease monitoring marker.

4. Urinalysis: dipstick for pH, glucose, blood, protein, ketones, and bilirubin. If there is a clinically significant positive result (i.e., confirmed by a positive repeated sample), urine will be sent to the laboratory for microscopy and culture. If there is an explanation for the positive dipstick result, e.g., menses, it should be recorded, and there is no need to perform laboratory for microscopy and culture.
5. HBV and HCV screening tests at screening.
6. All women of childbearing potential (including those who have had a tubal ligation) will have a serum pregnancy test at screening, within 7 days of first dose, on a regular basis during the treatment period (urine) and at the follow up visit.
7. Autoantibodies panel: anti-nuclear antibody, anti-double stranded DNA, cytoplasmic anti-neutrophil cytoplasmic antibody [c-ANCA], and perinuclear anti-neutrophil cytoplasmic antibody [p-ANCA].

Unscheduled hematology, biochemistry, and coagulation assay will be obtained in patients who develop infusion–related reactions (IRRs) or Cytokine Release Syndrome (refer to management Section 5.2.6.2.4) and as clinically indicated.

Additional safety laboratory assessments:

- ADA
- Cytokine release assessment/CRS
- IgE and tryptase for patients who experience a Grade ≥ 2 IRR/CRS.

"Use of this document is governed by the terms of use on the first page of this document."

3.3.2 Pharmacokinetic (PK) and Pharmacodynamic (PD) Outcome Measures

3.3.2.1 Pharmacokinetic Outcome Measures

Pharmacokinetic (PK) concentration data of RO6958688 and atezolizumab will be summarized with the use of descriptive statistical methods. PK parameters (area under the concentration curve [AUC], volume of distribution at steady state [V_{ss}], minimum and maximum serum concentration [C_{min} and C_{max}], time to C_{max} [T_{max}], clearance [CL], and half-life (t1/2) (terminal and effective)) of RO6958688 and atezolizumab will be estimated using non-compartmental analysis (NCA) methods when applicable.

Nonlinear mixed effect modeling will also be used to analyze the sparse concentration-time-data of RO6958688.

3.3.2.2 Pharmacodynamic Outcome Measures

The PD outcome measures for this study are the following and will be examined in patients enrolled in both Parts I and II:

- **Whole blood samples:** Peripheral blood immune cells will be assessed with respect to the changes in the characteristics of lineage (CD4+ T cells, CD8+ T cells, natural killer [NK] cells, monocytes, T-regulatory cells, and B cells), activation (including but not limited to CD25, CD69, etc.), and differentiation (including but not limited to CD45RO Ki67, PD1, TIM3, ICOS, etc.).
- **Whole blood samples:** A baseline, C3D1 and C6D1 mandatory whole blood sample will be taken for TCR Vβ sequencing (the CDR3-TCR beta chain repertoire). The DNA will be used to determine in peripheral T cells the repertoire of T cell receptor (TCR) Vβ CDR3 and analyze TCR Vβ diversity.
- **Soluble CEA:** will be measured as a disease-monitoring marker. A mandatory blood sample will be taken for soluble CEA central assessment.
- **Serum or plasma samples:** PD biomarkers such as cytokines and inflammation markers (including but not limited to tumor necrosis factor-α (TNFα), interferon-γ (INFγ), interleukin (IL)-6 etc.) will be analyzed. These measurements are also safety measure assessments during any IRRs. Disease-monitoring markers that include but are not limited to sCEA will also be assessed.
- **Tumor biopsy:** Tumor biopsy samples obtained from all patients enrolled. These paired tumor biopsies for PD analyses are mandatory at baseline and on-treatment, except for NSCLC patients for whom there is no accessible lesion. If feasible, biopsies may be repeated if the initial biopsy did not contain sufficient tumor material for analysis. Biopsies will be assessed centrally for changes in immune cell numbers and activation characteristics as well as changes in tumor markers such as PD-L1. These analyses will be performed by flow cytometric molecular and/or immunohistochemistry methods with respect to changes in the characteristics of lineage (CD4+ T cells, CD8+ T cells, NK cells, monocytes, T-regulatory cells, and B cells), activation (including but not limited to CD25, CD69, etc.), differentiation (including but not limited to CD45RO Ki67, PD1, TIM3, ICOS, etc.), TCR Vβ repertoire and tumor mutational load.

"Use of this document is governed by the terms of use on the first page of this document."

- **Positron Emission Tomography (PET):** Baseline and on-treatment 2-[18F]-Fluoro-2-deoxyglucose positron emission tomography (FDG-PET) will be collected to determine changes in glucose metabolism of the tumor lesions.
- **Original or archival tumor:** Potential predictive/prognostic biomarkers such as MMR status and CEA expression will be confirmed on archival tumor, if available, or from the freshly obtained biopsy samples. These measurements will assess the CEA change over the course of the disease and the stability of the measurements. Further other exploratory biomarker assessments, including but not limited to analysis of microsatellite instability, mutational signatures and genomic mutations, may be carried out.

Blood, Serum or Plasma Samples

Blood samples will be collected for the analyses of immune cell number and activation. Blood samples will be collected and analyzed with respect to alterations in the number and activation and differentiation of immune cells as a consequence to treatment with RO6958688 in combination with atezolizumab. The samples will be analyzed by flow cytometry, and the number of cells that belongs to lymphocyte subsets (CD4+ T cells, CD8+ T cells, NK cells, B cells, and monocytes) and their activation and differentiation status will be determined (including but not limited to CD25, Ki67 and PD1). Additional immune PD biomarkers that are related to the mode of action (MoA) of RO6958688 in combination with atezolizumab may also be analyzed from serum or plasma samples taken from all patients. Such PD measures will be considered as disease-monitoring markers and include but are not limited to sCEA. If available, an assessment of tumor growth kinetics will be made by comparing post-treatment scans with the last available pre-study scan.

Tumor Biopsy Samples

Tumor biopsy samples (each consisting of two tissue specimens at least the size of an 18G core needle biopsy, fine-needle aspiration is not acceptable) will be collected in all enrolled patients (once at baseline [after the baseline FDG PET] to confirm eligibility and once during the study treatment period), and two core samples will be collected at each occasion. Mandatory biopsies will be collected from all patients enrolled, except for NSCLC patients for whom there is no accessible lesion. The biopsies will be taken from accessible, “non-critical” tumor locations, including, but not limited to, skin, lymph node, rectum, liver, etc. On-treatment tumor biopsies will be randomized within dose schemes as follows: half of the patients at week 3 half of the patients at week 6.

If feasible, biopsies may be repeated if the initial biopsy did not contain sufficient tumor material for analysis.

If preliminary data suggest that modification of the on-treatment tumor biopsy timepoint would be more appropriate, alternative on-treatment tumor biopsy timepoints could be considered in the future cohorts.

“Use of this document is governed by the terms of use on the first page of this document.”

The baseline and on-treatment biopsies should preferentially be taken from the same tumor lesion to ensure comparability when accessible in a non-critical location.
Data from FDG-PET should guide which lesion would be more reflective of capturing the PD effect and thus the baseline and on-treatment biopsies should be taken from that tumor lesion.

For patients discontinuing from the study due to disease progression, additional optional biopsies, if clinically feasible, may be taken to aid the understanding of immune resistance mechanisms. Tumor biopsies will be centrally analyzed for immune cell number and activation by flow cytometric and/or immunohistochemical methods for the density of different immune cell lineages (including but not limited to CD4+, CD8+, NK cells, B cells, macrophages) and their activation and differentiation status (including but not limited to CD25, Ki67, PD1) and for the expression of tumor markers such as PD-L1, IDO etc. In addition, analysis of gene expression, genetic alteration and the infiltrating T cell repertoire may be carried out. The goals of these analyses will be (i) to establish a dose–response and/or an exposure-response relationship and (ii) to understand the MoA of RO6958688 in combination with atezolizumab at the tumor site.

Positron Emission Tomography (PET)

FDG, a glucose analog is taken up by cells with high metabolic activity, phosphorylated, and trapped in the cell. Since it is measuring cellular metabolism, FDG-PET can identify signs of biological effect early, before tumor size is reduced. Moreover, a reduction in the FDG-PET signal within days or weeks of initiating therapy (e.g., in lymphoma, non–small cell lung, and esophageal cancer) has been shown in some cases, to correlate with prolonged survival and other clinical end points now used. These findings suggest that FDG-PET could facilitate drug development as an early marker of drug effect.

Whole-body imaging PET should begin 60 ± 10 min after FDG injection.

If preliminary data suggest that modification of the on-treatment FDG-PET timepoint would be more appropriate, alternative on-treatment FDG-PET timepoints could be considered in the future cohorts.

3.3.3 Efficacy Outcome Measures

Any evaluable or measurable disease must be documented at screening and re-assessed at each subsequent tumor evaluation. The efficacy/activity outcome measures for this study are:

- ORR, defined as the proportion of patients achieving objective partial (PR) or complete (CR) responses
- Best overall response (BOR) defined as the best response recorded from the start of the study treatment until the end of treatment taking into account any requirement for confirmation

"Use of this document is governed by the terms of use on the first page of this document."

- Duration of response (DOR), defined as the time from initial objective response (PR or CR) to the first objective progression or death from any cause
- The rate of patients with stable disease (SDR)
- DCR, defined as ORR + SDR
- PFS according to Response Evaluation Criteria in Solid Tumors, Version 1.1 (RECIST v1.1)
- OS, defined as the time from first study treatment (primary efficacy analysis) or randomization date (ITT, only in randomized schedule comparison expansions) to death from any cause

Tumor response will be evaluated according to RECIST v1.1 and modified RECIST criteria using unidimensional measurement such as computed tomography (CT) scan or magnetic resonance imaging. Assessment of CT/MRI scans as tumor assessments will be performed at the sites during the whole study, and in addition centrally by an independent reviewer for prospective and retrospective analysis. Advanced volumetric analyses of CT/MRI will be performed.

Tumor assessment will be performed once during the screening period. The first assessment after the start of treatment will be performed at 8 weeks (C3D15) and continue every 8 weeks thereafter for the first year and every 12 weeks thereafter until disease progression or treatment discontinuation. All tumor assessments after baseline may be done within ± 7 days of the scheduled visit. Additionally, FDG-PET based tumor assessment will be performed at baseline and at week 4 (C2D8 + 1 week) and week 16 (C6D8) after the first dose of RO6958688, in case of dose delay the FDG PET should be done at week 4 (+1 week) and week 16. The week 16 FDG PET scan should be performed predose (and up to a maximum of 3 days before the visit). Baseline FDG-PET must be performed before the baseline tumor biopsy. Snapshots of CT scans and FDG-PET will be made available to the Sponsor. Based on data generated during this trial, the timepoints at which the two on treatment FDG PET scans are performed may be modified.

Confirmation of partial and complete responses will be done at the next scheduled visit after at least 28 days from the initial response. A patient is assigned a best overall confirmed response SD if they have a response assessment of SD, PR, or CR at one or more visits at least 42 days (6 weeks) after start of study treatment, but are not a confirmed CR or PR.

PFS per RECIST v1.1 or modified RECIST criteria is defined as time between enrollment or randomization date (whichever is applicable) and date of first documented disease progression per RECIST v1.1 or modified RECIST criteria, respectively, or death from any cause, whichever occurs first. Patients who neither progressed nor died in this interval, or who are lost to follow-up are censored at the date of last tumor assessment within this time window or last follow-up for progression of disease. Patients

"Use of this document is governed by the terms of use on the first page of this document."

for whom no post-baseline tumor assessments are available are censored at first study treatment. Conventional response criteria may not adequately assess the activity of immunotherapeutic agents because progressive disease (by initial radiographic evaluation) does not necessarily reflect therapeutic failure. Because of the potential for pseudoprogression/tumor immune infiltration, this study will allow patients to receive atezolizumab and/or RO6958688 to remain on study treatment after apparent radiographic progression, provided the benefit-risk ratio is judged to be favorable.

Patients should be discontinued for unacceptable toxicity or loss of RO6958688 exposure (in which case they can continue to receive atezolizumab alone) or symptomatic deterioration attributed to disease progression as determined by the investigator after an integrated assessment of radiographic data and clinical status (see Section 4.6.1.1).

Patients will be permitted to continue study treatment after meeting RECIST criteria for progressive disease (by investigator assessment) if they meet all of the following criteria:

- Evidence of clinical benefit as assessed by the investigators
- Absence of symptoms and signs (including worsening of laboratory values; e.g., new or worsening hypercalcemia) that indicate unequivocal progression of disease
- No decline in ECOG performance status that can be attributed to disease progression
- Absence of tumor growth at critical anatomical sites (e.g., leptomeningeal disease) that cannot be managed by protocol–allowed medical interventions
- Patients for whom approved therapies exist must provide written consent to acknowledge that they defer these treatment options in favor of continued study treatment at the time of initial apparent progression.

Patients in whom radiographic disease progression is confirmed at a subsequent tumor assessment may be considered for continued study treatment at the discretion of the investigators if they continue to meet the criteria above and have evidence of clinical benefit.

Optional submission of the latest pre-study or historical CT scans is highly encouraged for assessment of tumor growth kinetics within 6 weeks of patient entering the study if available. This scan will be compared to those collected during the study to determine tumor growth kinetics.

3.3.4 Exploratory Outcome Measures

The exploratory objectives for this study include but are not limited to the following:

- The density and localization of immune cells will be determined in freshly obtained biopsy in order to describe the immune infiltration before treatment

"Use of this document is governed by the terms of use on the first page of this document."

- The density and activation status of immune cell subsets in the tumor will be assessed in biopsies taken before and during treatment
- A possible association of expression of activation related immune genes (e.g., IFNγ, CXCLC9, etc.) with PD response will be investigated
- The baseline values and kinetics of soluble markers of immune cell activation (such as sCD25) and tumor markers (such as CEA and PD-L1) will be explored.

Additional markers may be measured in case a scientific rationale for these analyses develops.

4. MATERIALS AND METHODS

4.1 CENTER

This is a multi-center study (approximately 35 centers) to be conducted in North America, Europe, and Asia. Additional sites (and countries) may be included for back-up purposes and may be activated if needed. Note: No patients were enrolled in Asia.

Administrative and Contact Information, and List of Investigators are provided separately.

4.2 STUDY POPULATION

This study will enroll patients with locally advanced and/or metastatic CEA-positive solid tumors who have progressed on standard treatment or who are intolerant to standard treatment will be enrolled into this study.

This study has enrolled a total of 228 patients. For the purpose of this protocol, evaluable patients will be those with sufficient tumor tissue material to be collected during baseline and on-treatment biopsies to allow for the required PD studies. Patients who do not meet this requirement will be replaced. However, any patient who receives one or more doses of RO6958688 and/or atezolizumab will be evaluable for safety assessments.

In Part I, 228 patients were enrolled. No further enrollment will take place.

For the biomarker cohort, this study will enroll patients with very low/negative CEA expressing solid tumors. Very low/negative CEA expression is defined as those samples having $<$ 20% of tumor cells with IHC1+or IHC0+. Patients enrolled in the biomarker cohort will follow the same dose/schedule as patients in cohort C1.

Based on the preliminary safety and efficacy data from Part I, the Sponsor could focus on other specific CEA expressing tumor indications for which to enroll a certain number of patients in Part II. Note: Part II will not open for enrollment.

“Use of this document is governed by the terms of use on the first page of this document.”

4.2.1 Recruitment Procedures

Patients will be identified for potential recruitment using pre-screening enrollment logs, IEC/IRB approved newspaper/radio advertisements and mailing lists prior to consenting to take place on this study.

Once a patient has been confirmed as meeting all of the inclusion and none of the exclusion criteria, the Sponsor will approve enrollment of this patient in writing (via Confirmation of enrollment), which will be sent to the investigator.

For Part I of the study, the assigned dose of RO6958688 and the possible dosing date will be documented in the Confirmation of Enrollment, the screening and patient number is confirmed via the IXRS system. A Patient Enrollment and Identification Code List must be maintained by the investigator.

In Part IA, at least 3 patients will be initially enrolled in each cohort; additional patients might be enrolled to study safety, PK, or PD in more detail. Patients in a cohort will be enrolled in a sequential manner. Thus, the first patient in each cohort will be observed for safety for 1 week before enrolling additional patients in a cohort. For the schedule comparison cohorts B1), and C (part IB), the first patient in each cohort will be observed for safety for 1 week (2 weeks in Spain) before the second patient in each cohort is enrolled. A safety observation period of 1 week will be observed between the second and the third patient in each cohort, and from the third to subsequent patients.

During the dose escalation part, patients who withdraw before the end of the DLT period, for reasons other than DLTs, and patients who did not receive the assigned dose of RO6958688 (3 doses QW for the QW regimen) or atezolizumab (1 dose Q3W) during the DLT period, will be replaced to ensure that at least 3 patients in each cohort have been assessed for the full DLT period of 21 days prior to moving to the next dose level.

For Part II of the study, the screening and patient number, assigned dose and schedule of RO6958688 will be confirmed via the IXRS system. A Patient Enrollment and Identification Code List must be maintained by the investigator.

4.2.2 Inclusion Criteria

Patients must meet the following criteria for study entry:

1. Signed informed consent
2. Age ≥ 18 years
3. Confirmed locally advanced and/or metastatic solid tumor, with at least one tumor lesion of accessible non-critical location to biopsy, in patients who have progressed on a standard therapy, are intolerant to standard therapy, and/or are non-amenable to standard therapy
4. Radiologically measurable and clinically evaluable disease (as per RECIST v1.1 - previously irradiated lesions should not be counted as target lesions)

“Use of this document is governed by the terms of use on the first page of this document.”

5. Life expectancy (in the opinion of the investigator) of ≥ 12 weeks and LDH levels ≤ 2.5 ULN

6. Eastern Cooperative Oncology Group (ECOG) Performance Status (PS) 0–1

7. All acute toxic effects of any prior radiotherapy, chemotherapy, or surgical procedure must have resolved to Grade ≤ 1 or returned to baseline except alopecia (any grade) and Grade 2 peripheral neuropathy

8. Adequate hematological function (without transfusion within 2 weeks prior to Cycle 1, Day 1): neutrophil count of ≥ 1.5 × 10^9 cells/L and hemoglobin ≥ 9 g/dL (5.5 mmol/L), and lymphocytes within normal limits (≥ 0.8 × 10^9 cells/L), platelet count of ≥ 100,000/μL; INR and aPTT ≤ 1.5 × ULN. This applies only to patients who are not receiving therapeutic anticoagulation; patients receiving therapeutic anticoagulation should be on a stable dose. Genetic deficiencies affecting aPTT are not excluded unless assessed as clinically significant by the site hematologist.

9. Adequate liver function: total bilirubin ≤ 1.5 × the upper limit of normal (ULN; excluding Gilbert's Syndrome as defined in Appendix 8), when < 3 × ULN is allowed), AST and/or ALT ≤ 2.5 × ULN (in case of liver metastases, ≤ 5 × ULN). Patients with documented liver metastases: alkaline phosphatase ≤ 5 × ULN. For patients with bone disease, this criterion should only take into consideration the specific liver isoform of alkaline phosphatase (not the total since it is influenced by the bone isoform).

10. Adequate renal function: Creatinine clearance ≥ 60 ml/min calculated by CKD-EPI equation (see Appendix 2)

11. Negative serum pregnancy test within 7 days prior to study treatment in premenopausal women and women ≤ 2 years after start of menopause (menopause is defined as amenorrhea for > 2 years)

12. All women of childbearing potential and female partners of male patients: agreement to remain abstinent (refrain from heterosexual intercourse) or use contraceptive methods that result in a failure rate of < 1% per year during the treatment period and for at least 5 months after the last dose of RO6958688 and atezolizumab and 2 months after the last dose of tocilizumab (if applicable).

    For male participants, patient must agree to either remain completely abstinent or to use a condom and not donate sperm during the entire study period and for 3 months after the last administration of RO6958688 and for 2 months after the last dose of tocilizumab (if applicable). Male patients who have received only atezolizumab are not required to use contraception during atezolizumab treatment; pregnancies in female partners of male patients receiving atezolizumab monotherapy are not required to be reported.

    A woman is considered to be of childbearing potential if she is postmenarcheal, has not reached a postmenopausal state (> 2 years of amenorrhea with no identified cause other than menopause), and has not undergone surgical sterilization (removal of ovaries and/or uterus).

"Use of this document is governed by the terms of use on the first page of this document."

Examples of contraceptive methods with an expected failure rate of < 1% per year include bilateral tubal ligation, male sterilization, established, proper use of hormonal contraceptives that inhibit ovulation, hormone-releasing intrauterine devices, and copper intrauterine devices.

The reliability of sexual abstinence should be evaluated in relation to the duration of the clinical trial and the preferred and usual lifestyle of the patient. Periodic abstinence (e.g., calendar, ovulation, symptothermal, or postovulation methods) and withdrawal are not acceptable methods of contraception.

13. Patients with non-colorectal cancer should have confirmed CEA expression in tumor tissue (≥ 20% of tumor cells staining with at least moderate to high intensity of both cytoplasmic and/or membranous (IHC 2+ and IHC 3+). CEA expression should be centrally confirmed for U.S. and Canada patients.

    For CRC, the CEA assessment should be performed but the result is not required for patient selection; if no archival tumor tissue is available, fresh biopsy will be collected.

14. For the biomarker cohort (in Part IB), patients should have very low/negative CEA expression. Very low/negative CEA expression is defined as tumor samples having < 20% of tumor cells with IHC1+ or IHC 0+. CEA should be determined prior to enrollment, if no archival tumor is available, a fresh biopsy will be collected.

4.2.3 Exclusion Criteria

Patients who meet any of the following criteria will be excluded from study entry:

1. Active or untreated central nervous system (CNS) metastases as determined by CT or MRI evaluation during screening and prior radiographic assessments

    Patients with a history of treated asymptomatic CNS metastases are eligible, provided they meet all of the following criteria:

    - No metastases to brain stem, midbrain, pons, medulla, cerebellum, or within 10 mm of the optic apparatus (optic nerves and chiasm)
    - Radiographic demonstration of improvement upon the completion of CNS-directed therapy and no evidence of interim progression between the completion of CNS-directed therapy and the screening radiographic study
    - No history of intracranial hemorrhage or spinal cord hemorrhage
    - No ongoing requirement for dexamethasone as therapy for CNS disease; anticonvulsants at a stable dose allowed
    - No stereotactic radiation or whole-brain radiation within 28 days prior to Cycle 1 Day 1
    - Screening CNS radiographic study ≥ 4 weeks since completion of radiotherapy and ≥ 2 weeks since discontinuation of corticosteroids.

2. Spinal cord compression not definitively treated with surgery and/or radiation or previously diagnosed and treated spinal cord compression without evidence that disease has been clinically stable for ≥ 2 weeks prior to enrollment.

"Use of this document is governed by the terms of use on the first page of this document."

3. Leptomeningeal disease.

4. Patients with paraspinal, paratracheal and mediastinal pathological lesions larger than 2 cm unless they are previously irradiated. Irradiation of lesions must be completed at least 14 days prior to initiation of study treatment.

5. Malignancies within 5 years prior to enrollment, with the exception of those with a negligible risk of metastasis or death and treated with expected curative outcome (such as adequately treated carcinoma in situ of the cervix, basal or squamous cell skin cancer, localized prostate cancer treated surgically with curative intent, ductal carcinoma in situ treated surgically with curative intent).

6. Significant, uncontrolled concomitant diseases which could affect compliance with the protocol or interpretation of results, including diabetes mellitus, pulmonary disorders, and known autoimmune diseases.

7. Uncontrolled hypertension (systolic blood pressure (BP) > 150 mmHg and/or diastolic BP > 100 mmHg), unstable angina, congestive heart failure (CHF) of any New York Heart Association (NYHA) classification (Class II or greater), serious cardiac arrhythmia requiring treatment (exceptions: atrial fibrillation, paroxysmal supraventricular tachycardia), history of myocardial infarction within 6 months of enrollment.

8. Administration of a live, attenuated vaccine within 28 days before Cycle 1 Day 1 or anticipation that such a live attenuated vaccine will be required during the study. Influenza vaccination should be given during influenza season only (approximately October to March). Patients must not receive live, attenuated influenza vaccine (e.g., FluMist®) within 4 weeks prior to Cycle 1 Day 1 or at any time during the study.

9. Known HIV.

10. Active Hepatitis B (HBV) or Hepatitis C (HCV) infection (required at screening):
    - Patients with active hepatitis B (defined as having a positive hepatitis B surface antigen [HBsAg] test at screening)
        - Patients with past HBV infection or resolved HBV infection (defined as having a negative HBsAg test and a positive antibody to hepatitis B core antigen [anti-HBc] antibody test) are eligible.
    - Patients with active hepatitis C
        - Patients positive for HCV antibody are eligible only if PCR is negative for HCV RNA.

11. Severe infections within 28 days prior to Cycle 1 Day 1, including but not limited to hospitalization for complications of infection, bacteremia, or severe pneumonia or active tuberculosis.

12. Received oral or intravenous (IV) antibiotics within 14 days prior to Cycle 1 Day 1. Patients receiving prophylactic antibiotics (e.g., for prevention of a urinary tract infection) are eligible.

"Use of this document is governed by the terms of use on the first page of this document."

13. Any other diseases, metabolic dysfunction, physical examination finding, or clinical laboratory finding giving reasonable suspicion of a disease or condition that would contraindicate the use of an investigational drug.
14. Major surgery or significant traumatic injury < 28 days prior to Cycle 1 Day 1 (excluding biopsies) or anticipation of the need for major surgery during study treatment.
15. Dementia or altered mental status that would prohibit informed consent.
16. Known history of autoimmune disease, including but not limited to myasthenia gravis, myositis, autoimmune hepatitis, systemic lupus erythematosus, rheumatoid arthritis, inflammatory bowel disease, vascular thrombosis associated with antiphospholipid syndrome, Wegener's granulomatosis, Sjögren's syndrome, Bell's palsy, Guillain-Barré syndrome, multiple sclerosis, vasculitis, or glomerulonephritis
    - The protocol allows:
        - Patients with a history of autoimmune hypothyroidism on a stable dose of thyroid replacement hormone are eligible
        - Patients with Type 1 diabetes mellitus on appropriate medical management may be eligible for this study *after consultation with* the Medical Monitor.
        - Patients with positive serology of auto-antibody panel (anti-nuclear antibody, anti-double stranded DNA, cytoplasmic anti-neutrophil cytoplasmic antibody [c-ANCA], and perinuclear anti-neutrophil cytoplasmic antibody [p-ANCA]) at screening should be referred to a specialist (i.e., Rheumatologist) for further assessments if the Investigator, after discussion with the Medical Monitor, considers the results as clinically significant.
17. History of idiopathic pulmonary fibrosis, pneumonitis (including drug induced), organizing pneumonia (i.e., bronchiolitis obliterans, cryptogenic organizing pneumonia, etc.), or evidence of active pneumonitis (including drug induced) on screening chest CT scan. History of radiation pneumonitis in the radiation field (fibrosis) is permitted.
18. Patients with bilateral lung lesions and dyspnea and/or SaO2 <92% (at rest, room air and exertion) or patients with lobectomy or pneumonectomy with lung metastases in the remaining lung and either dyspnea or SaO2 <92% (at rest, room air and exertion) at baseline.
19. Baseline QTc interval of > 470 ms, baseline resting bradycardia < 45 beats per minute, or baseline resting tachycardia > 100 beats per minute.
20. Pregnant or breast-feeding women.
21. Known hypersensitivity to any of the components of RO6958688 and atezolizumab; hypersensitivity to Chinese hamster ovary cell products or other recombinant human antibodies.

"Use of this document is governed by the terms of use on the first page of this document."

22. Investigational therapy (defined as treatment for which there is no regulatory authority approved indication) or last dose of prior immunotherapies including but not limited to: interferon alpha (IFN-α), interferon-beta (IFN-β), IL-2, conjugated IL-2, CEA-IL2v, cytokines, anti-CTLA4, within 28 days prior to Cycle 1 Day 1. Patients previously treated with anti-programmed death-ligand 1 (PD-L1), or anti-PD-1 are excluded.

23. Any approved anti-cancer therapy, including chemotherapy or hormonal therapy, within 28 days prior to Cycle 1 Day 1, with the following exceptions:

    - Hormone-replacement therapy or oral contraceptives
    - Tyrosine kinase inhibitors (TKIs) (both small molecules and antiangiogenic monoclonal antibodies) that have been discontinued $>$ 21 days prior to Cycle 1 Day 1; baseline scans must be obtained after discontinuation of prior TKIs.

24. Prior systemic corticosteroids >10mg prednisone (or equivalent) within 14 days of Cycle 1 Day 1. The use of inhaled and/or topical corticosteroids and mineralocorticoids (e.g., fludrocortisone) is allowed.

25. Last dose with any of the following agents including but not limited to: etanercept, infliximab, tacrolimus, cyclosporine, mycophenolic acid, alefacept, or efalizumab $<$ 28 days prior to first dose of study drugs.

26. Regular immunosuppressive therapy (i.e., for organ transplantation, chronic rheumatologic disease).

27. Patients with prior allogeneic bone marrow transplantation or prior solid organ transplantation.

28. Treatment with systemic immunosuppressive medications including, but not limited to: prednisone, cyclophosphamide, azathioprine, methotrexate, thalidomide, and anti-TNF agents within 14 days prior to Cycle 1, Day 1. Patients who have received acute and/or low-dose systemic immunosuppressant medications (e.g., a one-time dose of dexamethasone for nausea or chronic use of $\leq$ 10 mg/day of prednisone or dose-equivalent corticosteroid) may be enrolled in the study after discussion with the Medical Monitor. The use of inhaled corticosteroids and mineralocorticoids (e.g., fludrocortisone) for patients is allowed.

29. Radiotherapy within the last 28 days before Cycle 1 Day 1 with the exception of limited field palliative radiotherapy e.g., for bone pain relief.

4.3 STUDY TREATMENTS

For the purpose of the study, RO6958688, atezolizumab, and tocilizumab are considered investigational medicine products (IMPs).

4.3.1 <u>Formulation, Packaging, and Handling</u>

4.3.1.1 RO6958688

Study drug packaging will be overseen by the Sponsor's clinical trial supplies department and bear a label with the identification required by local law, the protocol number, drug identification, and dosage. The packaging and labeling of the study

"Use of this document is governed by the terms of use on the first page of this document."

medication will be in accordance with the Sponsor's standard and local regulations. Upon arrival of investigational products at the site, site personnel should check them for damage and verify proper identity, quantity, integrity of seals, and temperature conditions and report any deviations or product complaints to the monitor upon discovery.

The qualified individual responsible for dispensing the study drug will prepare the correct dose according to the schedule. This individual will write the date of dispensing and patient number and initials on the study drug vial label and on the Drug Accountability Record. This individual will also record the study drug batch or lot number received by each patient during the study.

The manufacture of RO6958688 drug substance consists of fermentation cell culture and purification.

Ro 695-8688/F01-01 drug product is provided as a sterile, preservative-free, liquid concentrate for infusion in 6-mL glass vials with L-histidine/L-histidine hydrochloride aqueous buffer solution (pH 5.5) containing sucrose, methionine, and polysorbate 20. Each single-use, 6-mL vial contains 25 mg (nominal strength) of RO6958688, and the drug product is formulated at a concentration of 5 mg/mL.

Ro 695-8688/F03-01 drug product is provided as a sterile, preservative-free, liquid concentrate for infusion in 6-mL glass vials with L-histidine/L-histidine hydrochloride aqueous buffer solution (pH 5.5) containing sucrose, methionine, and polysorbate 20. Each single-use, 6-mL vial contains 100 mg (nominal strength) of RO6958688, and the drug product is formulated at a concentration of 20 mg/mL.

A variant of the current formulations with higher protein concentration has been developed to reduce the drug product volume. Ro 695-8688/F04-03 drug product is provided as a sterile, preservative-free, liquid concentrate for infusion in 2-mL glass vials with L-histidine/L-histidine hydrochloride aqueous buffer solution (pH 5.5) containing sucrose, methionine, and polysorbate 20. Each single-use, 2-mL vial contains 100 mg (nominal strength) of RO6958688, and the drug product is formulated at a concentration of 50 mg/mL.

Roformis No.	Ro 695-8688/F01-01	Ro 695-8688/F03-01	Ro 695-8688/F04-03
Protein concentration	5 mg/mL	20 mg/mL	50 mg/mL
Vial strength	25 mg / 5 mL	100 mg / 5 mL	100 mg / 2 mL

The recommended storage condition for Ro 695-8688/F01-01, Ro 695-8688/F03-01 and Ro 695-8688/F04-03 drug products is between 2°C–8°C, and it should be protected from light.

"Use of this document is governed by the terms of use on the first page of this document."

Upon arrival of investigational products at the site, site personnel should check them for damage and verify proper identity, quantity, integrity of seals and temperature conditions, and report any deviations or product complaints to the monitor upon discovery.

For further details on the storage and preparation of RO6958688, please refer to the WP29945 Pharmacy manual and RO6958688 Investigator's Brochure.

4.3.1.2 Atezolizumab

The atezolizumab drug product is provided in a single-use, 20-cc USP/Ph. Eur. Type 1 glass vial as a colorless-to-slightly-yellow, sterile, preservative-free clear liquid solution intended for IV administration. The vial is designed to deliver 20 mL (1200 mg) of atezolizumab solution but may contain more than the stated volume to enable delivery of the entire 20 mL volume. The atezolizumab drug product is formulated as 60 mg/mL atezolizumab in 20 mM histidine acetate, 120 mM sucrose, 0.04% polysorbate 20, pH 5.8.

Atezolizumab must be refrigerated at 2–8°C (36–46°F) upon receipt until use. Atezolizumab vials should not be used beyond the expiration date provided by the manufacturer. No preservative is used in atezolizumab drug product; therefore, the vial is intended for single use only. Discard any unused portion of drug left in a vial. Vial contents should not be frozen or shaken and should be protected from direct sunlight.

Upon arrival of investigational products at the site, site personnel should check them for damage and verify proper identity, quantity, integrity of seals and temperature conditions, and report any deviations or product complaints to the monitor upon discovery.

For further details on the storage and preparation of atezolizumab, please refer to the WP29945 Pharmacy Manual and atezolizumab Investigator's Brochure.

4.3.1.3 Tocilizumab

The tocilizumab drug product will be supplied by the Sponsor. Tocilizumab will be supplied in vials containing sterile solution of tocilizumab (20 mg/mL). Tocilizumab will be supplied as 200-mg vials.

Tocilizumab should be administered when necessary, as described in Appendix 10. For information on the formulation and handling of tocilizumab, see the pharmacy manual and the Tocilizumab Investigator's Brochure.

In an emergency situation where the study-specific labeled supply of tocilizumab is not accessible, it may be supplied locally by the study sites and will be formulated, prepared, and handled according to the local prescribing information. For further instructions regarding recommended storage conditions and packaging configuration, refer to the local prescribing information.

"Use of this document is governed by the terms of use on the first page of this document."

4.3.2 Dosage, Administration, and Compliance

4.3.2.1 RO6958688 and Atezolizumab

The qualified individual responsible for dispensing the study drugs will prepare the correct dose according to the cohort allocation schedule. This individual will write the date dispensed and patient number and initials on the study drugs vial labels and on the Drug Accountability Record. This individual will also record the study drugs batches or lot numbers received by each patient during the study.

Atezolizumab and RO6958688 should be administrated in a hospital or clinic equipped for IV chemotherapy, with full emergency resuscitation facilities immediately available. For anaphylaxis precautions, see Appendix 14. During and after the administration of both study drugs patients should be under close supervision of the investigator at all times. In case of infusion-associated adverse events in a patient, the signs and symptoms should be fully resolved before the patient is discharged.

Guidelines for dosage modification and treatment interruption or discontinuation are provided in Section 5.2.1.

4.3.2.2 Administration of Atezolizumab

Each treatment cycle will be 21 days in duration and consists of IV infusions of RO6958688 given weekly (QW ± 1 day) or every 3 weeks (Q3W ± 2 days) in combination with atezolizumab given every 3 weeks (Q3W ± 2 days).

Patients will first receive atezolizumab (1200 mg fixed dose) IV on Day 1 of each cycle, followed by RO6958688, given IV. Initially all patients in this trial will receive full dose of atezolizumab (1200 mg Q3W). In order to allow for resolution of toxicities, investigators in agreement with the sponsor are allowed to consider delaying the subsequent dose up to 105 days from the last one to prevent or to manage potential adverse events suspected to be related to the atezolizumab treatment.

The initial dose of atezolizumab will be delivered over 60 (± 15) minutes. If the first infusion is tolerated without infusion-related reaction (IRR), the second infusion may be delivered over 30 (± 10) minutes. If the 30-minute infusion is well tolerated, all subsequent infusions may be delivered over 30 (± 10) minutes.

If premedication is needed prior to atezolizumab infusion, it should be administered at least 30 minutes before the start of atezolizumab infusion.

4.3.2.3 Administration of RO6958688

RO6958688 will be administered by IV infusion on Day 1, Day 8 and Day 15 of each cycle for the QW regimen and on Day 1 of each cycle for the Q3W regimen.

For cohorts B, C and cohort G in part IB of the study, RO6958688 will be administered as described in Section 3.1.1.

A delay of RO6958688 administration for up to 14 days in the QW part and 21 days in the Q3W part will be acceptable to allow for resolution of toxicity NCI CTCAE Grade ≤ 2 hematological toxicities or Grade ≤ 1 non-hematological toxicities (with the exception of

“Use of this document is governed by the terms of use on the first page of this document.”

toxicity considered as non-RO6958688 related). RO6958688 administration may be delayed in case of a Grade ≥2 infusion related reaction during the atezolizumab administration. No other delays of RO6958688 administration are foreseen.

On Day 1 of each cycle, RO6958688 will be administered:

- at least half an hour after the end of atezolizumab infusion if no premedication is given prior to RO6958688 administration.
- at least one hour after the end of atezolizumab infusion if premedication is given prior to RO6958688 administration.

RO6958688 should be administered at C1D1 by IV infusion over a minimum of 2 hours, subsequent infusions should be administered in at least 4 hours during the dose/escalation phase, in patients with no grade ≥ 2 IRR/CRS for more than two RO6958688 administrations, the infusion time can be progressively reduced to a minimum of 1 hour. For more details, please refer to the RO6958688 pharmacy manual.

If premedication is needed prior to RO6958688 infusion, it should be administered at least 30 minutes before the start of RO6958688 infusion. Please refer to Section 4.3.2.5 for details of pre-medications prior to RO6958688 infusion.

Hypotension may occur as a result of an IRR/CRS, therefore, it is recommended that antihypertensive drugs must not be given on the morning of, and throughout the infusion of RO6958688 if clinically indicated. Patients with a history of cardiac disease should be monitored closely.

Based on chemical and physical in-use stability data the infusion time should not exceed 8 hours at room temperature including interruptions (if any), the Pharmacy Manual includes detailed instructions regarding refrigerated storage of the dose solution prior to administration.

Information on cohorts, doses, and administration parameters are described in the Pharmacy Manual.

In Part IA (dose escalation) optional RO6958688 dose escalation to the next available tolerated dose level can only proceed after a patient has tolerated at least the first 3 consecutive doses of RO6958688 after discussion with and approval by the Medical Monitor.

4.3.2.4 Administration of Tocilizumab

Tocilizumab infusion will follow the methods described in the Investigator Brochure or other similar local prescribing documents and will be administered for the treatment of CRS considered related to RO6958688 (see Appendix 10, Table 2).

“Use of this document is governed by the terms of use on the first page of this document.”

Tocilizumab (8 mg/kg) will be administered at room temperature by controlled IV infusion into over a 1-hour period. The infusion rate must be 10 mL/hr for 15 minutes and then increased to 130 mL/hr to complete the dosing over the 1-hour time period. Normal saline (20 mL) will be administered following infusion of study drug to flush the remaining study drug through the IV set.

4.3.2.5 Premedication and Prophylactic Treatment for RO6958688 and Atezolizumab

The use of analgesics, NSAID and/or antihistamines is allowed to minimize expected flu-like symptoms associated with the administration of RO6958688 as follows:

- Paracetamol/acetaminophen (500–1000 mg orally or IV) and/or NSAID
- Diphenhydramine (50–100 mg [orally or IV] or an alternative antihistamine at an adequate dose)

Pre-medicate all patients in step-up cohorts with 10 mg dexamethasone IV until the RO6958688 dose escalation has been completed (Table 2).

Table 2 Pre-Medications to Be Administered before RO6958688 Infusion

Infusion of RO6958688	Patients requiring pre-medications	Pre-medication**	Administration
During step-up phase	All patients	IV dexamethasone 10mg	Completed at least 1 hour prior to RO6958688 infusion
		Analgesic and/or NSAID	At least 30 min before RO6958688 infusion
		Anti-histamine	
During step-up phase	IRR/CRS G2*** with previous RO6958688 infusion despite pre-medications	For corticosteroid use, see management guidelines in Appendix 10**.**	
		Analgesic, anti-emetics and/or NSAID	At least 30 min before RO6958688 infusion
		Anti-histamine	
During flat dose phase†	Patient with no IRR/CRS at previous infusion	Analgesic, anti-emetics and/or NSAID	At least 30 min before RO6958688 infusion
		Anti-histamine	
	Patient with G1 or G2 IRR/CRS at previous infusion	Analgesic, anti-emetics and/or NSAID	At least 30 min before RO6958688 infusion
		Anti-histamine	
		For corticosteroid use, see management guidelines in Appendix 10.	

**Analgesic- Paracetamol/acetaminophen 500-1000 mg orally or IV. Anti-histamine diphenhydramine 50-100 mg orally or IV or alternate anti-histamine at adequate dose
***Per Appendix 10, in patients with IRR/CRS recurring at ≥Grade 3, patients not recovering within 8 hours of corticosteroids and tocilizumab administration, and patients with Grade 4 IRR/CRS, RO6958688 should be permanently discontinued.
† Patients in flat dose cohorts to follow this recommendation

“Use of this document is governed by the terms of use on the first page of this document.”

If an IRR/CRS occurs, follow IRR/CRS management guidelines in Appendix 10 for premedication recommendations. Corticosteroid administration should be completed at least 60 minutes before RO6958688 administration.

4.3.3 Investigational Medicinal Products Accountability

All investigational medicinal products (IMPs) required for completion of this study (RO6958688, atezolizumab, and tocilizumab) will be provided by the Sponsor where required by local health authority regulations. The investigational site will acknowledge receipt of IMPs supplied by the Sponsor using IXRS, to confirm the shipment condition and content. Any damaged shipments will be replaced.

Tocilizumab will be provided by the Sponsor.

The investigator is responsible for the control of drugs under investigation. Adequate records of the receipt (e.g., Drug Receipt Record) and disposition (e.g., Drug Dispensing Log) of the study drugs must be maintained. The Drug Dispensing Log must be kept current and should contain the following information:

- The identification of the patient to whom the study drugs were dispensed (for example patient initials and date of birth)
- All records and drug supplies must be available for inspection by the Roche Monitor (at every monitoring visit).

IMPs will either be disposed of at the study site according to the study site's institutional standard operating procedure or returned to the Sponsor with the appropriate documentation. The site's method of IMP destruction must be agreed upon by the Sponsor. Local or institutional regulations may require immediate destruction of used investigational medicinal product for safety reasons. In these cases, it may be acceptable for investigational study site staff to destroy dispensed investigational product before a monitoring inspection provided that source document verification is performed on the remaining inventory and reconciled against the documentation of quantity shipped, dispensed, returned, destroyed and provided that adequate storage and integrity of drugs has been confirmed.

The site must obtain written authorization from the Sponsor before any IMP is destroyed, and IMP destruction must be documented on the appropriate form.

Written documentation of destruction must contain the following:

- Identity batch numbers of investigational product[s] destroyed
- Quantity of investigational product[s] destroyed
- Date of destruction
- Method of destruction
- Name and signature of responsible person [or company] who destroyed investigational products

"Use of this document is governed by the terms of use on the first page of this document."

Accurate records of all IMPs received at, dispensed from, returned to, and disposed of by the study site should be recorded on the Drug Inventory Log.

4.3.4 Post-Trial Access to RO6958688 and Atezolizumab

The Sponsor will offer post-trial access to the study drugs (RO6958688 and atezolizumab) free of charge to eligible patients in accordance with the Roche Global Policy on Continued Access to Investigational Medicinal Product, as outlined below.

A patient will be eligible to receive study drugs after the end of the study if all of the following conditions are met:

- The patient has a life-threatening or severe medical condition and requires continued study drugs treatments for his or her well-being
- There are no appropriate alternative treatments available to the patient
- The patient and his or her doctor comply with and satisfy any legal or regulatory requirements that apply to them

A patient will not be eligible to receive study drugs after the end of the study if any of the following conditions are met:

- The study drugs are commercially marketed in the patient's country and is reasonably accessible to the patient (e.g., is covered by the patient's insurance or wouldn't otherwise create a financial hardship for the patient)
- The Sponsor has discontinued development of the study drugs or data suggest that the study drugs are not effective for cancer
- The Sponsor has reasonable safety concerns regarding the study drugs as treatment for cancer
- Provision of study drugs is not permitted under the laws and regulations of the patient's country

F. Hoffmann La-Roche does not intend to provide study intervention to patients after conclusion of the study or in case of earlier patient withdrawal.

4.4 CONCOMITANT THERAPY

4.4.1 Permitted Therapy

Concomitant therapy includes any medication, e.g., prescription drugs, over the counter drugs, approved dietary and nutritional supplements used by a patient from 4 weeks prior to screening until the follow-up visit. Concomitant use of herbal therapies is not recommended because their pharmacokinetics, safety profiles, and potential drug-drug interactions are generally unknown.

"Use of this document is governed by the terms of use on the first page of this document."

All concomitant medications should be reported to the investigator and recorded on the Concomitant Medications electronic Case Report Form (eCRF).

All therapy and/or medication administered to manage adverse events should be recorded on the Adverse Event eCRF.

Patients who experience infusion-associated symptoms may be treated symptomatically as described in Section 5.2.1.

Use of Steroids

Systemic corticosteroids and immune suppressants may attenuate potential beneficial immunologic effects of treatment with atezolizumab and RO6958688 but may be administered at the discretion of the treating physician after consultation with the Medical Monitor. If feasible, alternatives to corticosteroids should be considered (such as infliximab).

Megestrol administered as an appetite stimulant is acceptable while the patient is enrolled in the study.

Infusion related reaction/CRS

Premedication including steroids may be administered as described in Section 4.3.2.5.

The use of inhaled corticosteroids and mineralocorticoids (e.g., fludrocortisone) for patients with orthostatic hypotension or adrenocortical insufficiency is allowed.

Radiotherapy

Use of limited field palliative radiotherapy is allowed at any time during the study except for:

- Days where study drugs are administered
- One day prior or post study drug administration is observed

No delay of study drugs administration is foreseen although subjects should not receive study treatments during radiation.

Influenza vaccination

Influenza vaccination should be given during influenza season only (approximately October to March). Patients must not receive live, attenuated influenza vaccine (e.g., FluMist®) within 4 weeks prior to Cycle 1, Day 1 or at any time during the study and up to 5 months after the last dose of atezolizumab but may receive inactivated vaccine.

"Use of this document is governed by the terms of use on the first page of this document."

4.4.2 Prohibited Therapy

The use of the following therapies is prohibited during the study and for at least 28 days prior to initiation of study treatments (unless otherwise specified):

- Investigational or unlicensed/unapproved agents
- Immunotherapy/radio-immunotherapy
- Chemotherapy
- Hormonal anticancer therapy
- Immunostimulatory agent (all patients, including those who discontinue the study early, should not receive other immunostimulatory agents for 10 weeks after the last dose of atezolizumab)
- Radiotherapy (with the exception of limited–field palliative radiotherapy)
- Biologic agents (e.g., bevacizumab, erlotinib)
- Immunosuppressive medications, including but not limited to cyclophosphamide, azathioprine, methotrexate, and thalidomide; these agents could potentially alter the activity and the safety of atezolizumab. Systemic corticosteroids administered at a dose equal or higher than prednisone 10 mg/day or equivalent (inhaled and topical steroids are permitted).

  Systemic corticosteroids, TNF-α inhibitors, mycophenolate, and other immunosuppressive medications may be administered for the treatment or prevention of *immune-mediated* toxicities at the discretion of the treating physician after consultation with the Medical Monitor.
- Other systemic anti-neoplastic agents and targeted therapies
- Patients must not receive live, attenuated vaccines (such as FluMist®) at any time during the study and up to 5 months after the last dose of atezolizumab. Vaccination with live vaccines is not recommended during treatment and is forbidden 28 days prior to dosing with atezolizumab.
- Initiation or increased dose of granulocyte colony-stimulating factors (e.g., granulocyte colony-stimulating factor, granulocyte/macrophage colony-stimulating factor, and/or pegfilgrastim) is prohibited.

If any anti-neoplastic or investigational therapies listed above are needed, the patient will be considered to have evidence of progressive neoplastic disease and have experienced treatment failure with study treatment and should be withdrawn from study treatment.

Patients who experience a mixed response that requires local therapy (e.g., surgery, stereotactic radiosurgery, radiotherapy, and/or radiofrequency ablation) for control of three or fewer lesions may still be eligible to continue study treatment. Patients who receive local therapy directed at a target lesion will no longer be evaluable for radiographic response but will remain evaluable for progression. Such cases must be discussed with and approved by the Medical Monitor.

“Use of this document is governed by the terms of use on the first page of this document.”

All concomitant treatments must be documented in the eCRF.

4.5 STUDY ASSESSMENTS

4.5.1 Description of Study Assessments

All examinations listed below will be performed according to the Schedule of Assessment and Hourly Assessment tables outlined in Appendix 1.

4.5.1.1 Medical History and Demographic Data

Medical history includes clinically significant diseases, demographic data (including age, sex, and self-reported race/ethnicity), all surgeries, cancer history (including any biomarker information (e.g., tumor mutations like KRAS, Braf and NRAS, CEA expression), all prior cancer therapies and procedures), reproductive status, and concomitant medications.

4.5.1.2 Physical Examinations, Vital Signs, and ECOG Performance Status

A complete physical examination should include an evaluation of the head, eyes, ears, nose, throat, neck and lymph nodes, and the cardiovascular, dermatological, musculoskeletal, respiratory rate, gastrointestinal, genitourinary, and neurological systems.

Any abnormality identified at baseline should be recorded on the General Medical History and Baseline Conditions eCRF.

A physical examination will include careful examinations of the areas of known and possible malignancy. The physical examination includes weight (height measurement is only done at screening and at follow-up visit, the body mass index (BMI) will be derived). For the purpose of the study, the same calibrated balance should be used at each site. Vital signs (including body temperature, respiratory rate, heart rate, diastolic and systolic BP, oxygen saturation at rest). In patients with bilateral lung lesions or patients with lobectomy or pneumonectomy with lung metastases in the remaining lung, oxygen saturation under exercise is also required. ECOG PS will be obtained in all patients at screening and as described in the Schedule of Assessment and Hourly Assessment tables (see Appendix 1).

PS will be measured using the ECOG PS scale (Appendix 3). PS will be assessed with each physical examination, prior to each study drug administration, and at the safety follow-up visit. It is recommended, where possible, that a patient's PS be assessed by the same person throughout the study.

Results will be recorded on the eCRF. Changes from baseline abnormalities should be recorded in patient's notes. New or worsened clinically significant abnormalities should be recorded as adverse events on the Adverse Event eCRF.

"Use of this document is governed by the terms of use on the first page of this document."

Routine vital signs monitoring including body temperature, respiratory rate, heart rate, diastolic and systolic BP, oxygen saturation will be recorded at the timepoints specified in Schedule of Assessment and Hourly Assessment tables (see Appendix 1).

During the day, BP and heart rate should be obtained in a quiet room at a comfortable temperature, with the patient's arm unconstrained by clothing or other material. All measurements will be obtained from the same arm and with the same cuff size using an automatic instrument with a digital readout throughout the study. The "ideal" cuff should have a bladder length that is 80% and a width that is at least 40% of arm circumference (a length-to-width ratio of 2:1). The automatic cuff should be placed on the designated arm at least 10 minutes prior to dosing. The patient should have his or her back and arm supported such that the middle of the cuff on the upper arm is at the level of the right atrium (the midpoint of the sternum). After the patient has been resting in supine decubitus position for at least 5 minutes, BP and heart rate will be obtained.

4.5.1.3 Electrocardiograms

Triplicate 12-lead ECG recordings (i.e., three qualitatively acceptable ECGs without artifacts) must be obtained at the timepoints as specified in the Schedule of Assessments (see Appendix 1) and within approximately 2–5 minutes at each specified timepoint. The average of the three readings will be used to determine the ECG intervals (i.e., PR, QRS, QT). Additional unscheduled ECG assessments should be performed in case of abnormalities and if clinical symptoms occur.

To minimize variability, it is important that the patient is in a supine position for ≥5 minutes prior to each ECG evaluation. The conditions should be as close as possible to predose timepoints; this includes but is not limited to food intake, activity level, stressors, and room temperature. Body position should be consistently maintained for each ECG evaluation to prevent changes in heart rate. Environmental distractions (e.g., television, radio, conversation) should be avoided during the pre-ECG resting period and during ECG recording. Whenever possible, ECGs should be performed prior to meals, any scheduled vital sign measurements, and blood draws. In some cases, it may be appropriate to repeat abnormal ECGs to rule out improper lead placement that may contribute to the abnormality in ECG results.

For safety monitoring purposes, the investigator or designee must review, sign, and date all ECG tracings. Paper or electronic copies will be kept as part of the patient's permanent study file at the site. If considered appropriate by the Sponsor, ECGs may be analyzed retrospectively at a central laboratory.

ECG characteristics including heart rate, QRS duration, and PR and QT intervals and QTcB (Bazett's correction) will be recorded on the eCRF, QTcF (Fridericia's correction), and RR will be derived on the eCRF. Changes in T-wave and U-wave morphology and overall ECG interpretation will be documented on the eCRF. T-wave information will be

"Use of this document is governed by the terms of use on the first page of this document."

captured as normal or abnormal, whereas U-wave information will be captured in two categories, absent/normal or abnormal.

4.5.1.4 Pulmonary Function Tests (FEV1/VC/TLC and DLCO)

FEV1/VC/TLC (including all derived parameters: inspiratory reserve volume, expiratory reserve volume, tidal volume and total lung capacity) and DL_{CO} (corrected for both alveolar volume and hemoglobin) will be evaluated at screening in patients with bilateral lung metastases or patients with lobectomy or pneumonectomy with lung metastases in the remaining lung. Additional tests may be performed if clinically indicated.

4.5.1.5 Laboratory Assessments

Normal ranges for the study laboratory parameters must be supplied to Roche before the study starts. Laboratory safety tests shall be collected at time-points specified in the Schedule of Assessments (see Appendix 1).

Additional blood or urine samples may be taken at the discretion of the investigator if the results of any test fall outside the reference ranges or clinical symptoms necessitate additional testing to monitor patient safety. Where the clinical significance of abnormal lab results is considered uncertain, screening lab tests may be repeated before enrollment to confirm eligibility. If there is an alternative explanation for a positive urine or blood test for drugs of abuse, e.g., previous occasional intake of a medication or food containing for example codeine, benzodiazepines, or opiates, the test may be repeated to confirm washout.

In the event of unexplained abnormal clinically significant laboratory test values, the tests should be repeated immediately and followed up until they have returned to the normal range and/or an adequate explanation of the abnormality is found. Results of clinical laboratory testing will be recorded on the eCRF or will be received as electronically produced laboratory reports submitted directly from the local or central lab.

Urinalysis, hematology, blood biochemistry, coagulation, and Hepatitis B and Hepatitis C virus serology samples will be sent to the study site's local laboratory for analysis.

- Hematology: erythrocytes, hemoglobin, hematocrit, platelets, leucocytes, and differential count (neutrophils, eosinophils, basophils, monocytes, lymphocytes).
- Blood biochemistry: sodium, potassium, chloride, calcium, phosphate, magnesium, urea, creatinine, normal glomerular filtration rate (by CKD-EPI equation), total protein, albumin, glucose, total and direct bilirubin, alkaline phosphatase (ALP), alanine aminotransferase (ALT), aspartate aminotransferase (AST), lactate dehydrogenase (LDH), γ-glutamyl transferase (GGT), C reactive protein (CRP); cholesterol (total, LDL cholesterol, HDL cholesterol), triglycerides, ferritin, soluble CD25.

Serum CEA will also be measured as a disease-monitoring marker (as part of the serum chemistry panel).

“Use of this document is governed by the terms of use on the first page of this document.”

- Coagulation: prothrombin time (PT)/international normalized ratio (INR) and partial thromboplastin time (PTT) and fibrinogen. Additional coagulation parameters (i.e., first chromogenic antithrombin III then antigenic antithrombin III in case of chromogenic antithrombin III decrease in order to determine type 1 and type 2 antithrombin deficiencies. If a deficiency is detected, both antithrombin tests will need to be repeated at a later date/visit to confirm test findings, fibrinogen, prothrombin time, fibrin degradation products, D-dimer) could be assessed according to clinical judgment, or if any of the above parameter cannot be assessed locally.
- Thyroid function: thyroid stimulating hormone (TSH) free T3 (or total T3 for sites where free T3 is not performed) and T4.

Unscheduled hematology, biochemistry and coagulation may be obtained in patients who develop IRRs/CRS.

- Pregnancy test: All women of childbearing potential (including those who have had a tubal ligation) will have a serum pregnancy test at screening within 7 days of first dose, on a regular basis during the treatment period (urine) and at Safety follow-up visit.
- Urinalysis: Dipstick for pH, glucose, blood, protein, ketones, and bilirubin. If there is a clinically significant positive result (i.e., confirmed by a positive repeated sample), urine will be sent to the laboratory for microscopy and culture. If there is an explanation for the positive dipstick result, e.g., menses, it should be recorded, and there is no need to perform laboratory for microscopy and culture.
- Hepatitis B virus (HBV) serology (HBsAg, antibodies against HBsAg, hepatitis B core antigen)
    - HBV DNA is required if patient has positive anti-HBc test.
- HCV serology (anti-HCV).

Based on continuous analysis of the data in this study and other studies, any sample type not considered to be critical for safety may be stopped at any time if the data from the samples collected does not produce useful information.

4.5.1.6 Additional Samples

Thyroid stimulating hormone (TSH) and auto-antibody samples will be sent to the study site's local laboratory for analysis. Freshly obtained tumor biopsies, PK, ADA, plasma samples, and whole blood samples for flow cytometry and clinical genotyping will be analyzed centrally by one or several central laboratories or by the Sponsor. Instruction manuals and supply kits will be provided for all central laboratory assessments.

Based on continuous analysis of the data in this study and other studies, any sample type not considered to be critical for safety may be stopped at any time if the data from the samples collected do not produce useful information.

“Use of this document is governed by the terms of use on the first page of this document.”

4.5.1.6.1 Pharmacokinetic Assessments

PK samples are mandatory for all parts of the study. Blood samples to evaluate RO6958688 and atezolizumab serum levels will be collected from an IV line from the arm opposite to that which is used for study drugs administration. The date and time of each sample collection will be recorded in the eCRF. The PK assessments will be performed as outlined in the Schedule of Assessments and Hourly Assessment tables (see Appendix 1). During the course of the study, PK sampling time points may be modified based on emerging data to ensure the PK of RO6958688 can be adequately characterized (but without increasing overall blood collection volume for PK). No new PK sampling time points will be introduced. Additional PK samples will be taken at the time of treatment discontinuation, as an unscheduled sample, if the patient experiences an IRR/CRS or an AE leading to dose reduction or delay of RO6958688 and atezolizumab administration (see Section 5.2.1). Remaining PK sample volume may also be used for assay validation during the development of study or compound related assays after the mentioned intended uses.

4.5.1.6.2 Special Safety Assessments

Anti-Drug Antibodies (ADAs)

Although RO6958688 and atezolizumab are both humanized antibodies, there is a risk that ADAs against RO6958688 and atezolizumab could develop, potentially reducing the efficacy of either antibody and/or potentially resulting in symptomatic hypersensitivity reactions, in particular immune complex reactions.

In this study, samples will be taken to assess the possible presence of ADAs. The date and time of each sample will be recorded in the eCRF.

Blood samples for ADA determination will be obtained as specified in the Schedule of Assessments and Hourly Assessment tables (see Appendix 1). Additional samples will be drawn as an unscheduled ADA sample in patients with clinical signs of hypersensitivity reaction, in particular immune complex reactions. In any case, for each collected ADA sample, a corresponding PK sample will be collected at the same time point for the determination of the RO6958688 and/or atezolizumab concentration.
If required, the residual of the PK samples taken during the study (any timepoint) can be used for additional ADA analysis. Remaining ADA sample volume may also be used for assay validation during the development of study or compound related assays after the mentioned intended uses.

Cytokine Release Assessment during IRR/CRS

Plasma samples (PD plasma and additional safety) for the assessment of cytokine release will be collected at the time of an IRR/CRS (including repetitive occurrence of IRR/CRS hypersensitivity reaction) as an unscheduled plasma sample. These samples will be analyzed as described in Section 4.5.1.6.3 – Serum or Plasma samples.

"Use of this document is governed by the terms of use on the first page of this document."

Serum Thyroid-Stimulating hormone (TSH)

Serum TSH and free T3 (or total T3 for sites where free T3 is not performed) and T4 level will be assessed at baseline pre-dose at cycle 1, on study (every 3 months), and at study completion/early termination visit and safety follow-up visit (28 days after last dose of study drugs). In case of abnormalities, the patient should be monitored until full resolution and further analysis (i.e., free T3 and T4 serum level) performed. An endocrinologist should be consulted if an endocrinopathy is suspected.

Autoantibody Panel

The panel should include anti-nuclear antibody, anti−double-stranded DNA, circulating anti-neutrophil cytoplasmic antibody, and perinuclear anti-neutrophil cytoplasmic antibody. The autoantibody panel will be assessed at screening, pre-dose every second cycle, and every 3 months after third month on therapy (samples can be drawn up to 1 week before Day 1 visit), if possible locally. In patients who develop signs and/or symptoms suggestive of auto-immune disease while on treatment, the antibody panel should be repeated. Patients with confirmed positive serology of at least one of the auto-antibody panel (anti-nuclear antibody, anti-double stranded DNA, cytoplasmic anti-neutrophil cytoplasmic antibody [c-ANCA], and peri nuclear anti-neutrophil cytoplasmic antibody [p-ANCA]) at screening and during the course of the study should be referred to a specialist (i.e., Rheumatologist) for further assessment if the investigator, after discussion with the Medical Monitor, considers it as clinically significant.

4.5.1.6.3 Pharmacodynamic, Exploratory and Clinical Genotyping Assessments

The following samples will be used for research purposes to identify biomarkers useful for predicting and monitoring response to RO6958688 and atezolizumab treatments, identifying biomarkers useful for predicting and monitoring RO6958688 in combination with atezolizumab safety, assessing PD effects of RO6958688 and atezolizumab treatments, and investigating mechanism of resistance to therapy. Additional markers may be measured in the case of a strong scientific rationale.

The following samples will be collected as specified in the Schedule of Assessments and Hourly Assessment tables (see Appendix 1):

Whole Blood Samples for Flow Cytometry

In this study, whole blood samples will be collected for the flow cytometry. The samples will be analyzed for changes in the numbers and activation status of lymphocyte subsets including but not limited to $CD8^+$, $CD4^+$, and regulatory T cells, B cells and/or NK cells.

Whole Blood Samples

In addition, a baseline and Cycle 3 Day 1 mandatory whole blood sample will be taken for TCR Vβ sequencing (the CDR3-TCR beta chain repertoire). The DNA will be used to determine in peripheral T cells the repertoires of T cell receptor (TCR) Vβ CDR3 and analyze TCR diversity.

“Use of this document is governed by the terms of use on the first page of this document.”

Soluble CEA

A mandatory blood sample will be taken for soluble CEA central assessment. Samples will be collected as per the schedule of assessments in Appendix 1.

Serum or Plasma Samples

Blood for serum or plasma isolation (blood for pharmacodynamics and additional safety) will be collected. Cytokines, inflammation markers, soluble CEA will be measured in these samples. Additionally, in case of IRR(s) or CRS, an assessment of cytokines released during the reaction will be done on serum or plasma samples from the time of the IRR/CRS. The sample used for these PD analyses will be identical with the one collected for cytokine release at IRR/CRS and IgE and tryptase assessment.

Tumor Biopsy Samples

Tumor biopsy samples (each consisting of two tissue specimens at least the size of an 18G core needle biopsy, fine-needle aspiration is not acceptable) will be collected in all enrolled patients (once at baseline [after the baseline FDG PET] to confirm eligibility and once during the study treatment period), and two core samples will be collected at each occasion. Mandatory biopsies will be collected from all patients enrolled, except for NSCLC patients for whom there is no accessible lesion. The biopsies will be taken from accessible, "non-critical" tumor locations, including, but not limited to, skin, lymph node, rectum, liver, etc. On-treatment tumor biopsies will be randomized within dose schemes as follows: half of the patients at week 3, half of the patients at week 6. All biopsies must be taken after FDG-PET, unless, in the absence of available archival tumor tissue, the baseline sample is being used to assess CEA expression for eligibility in which case the biopsy may be taken before the FDG-PET scan.

Tumor biopsies should be taken predose (and up to 48h before the visit).

If feasible, biopsies may be repeated if the initial biopsy did not contain sufficient tumor material for analysis.

If preliminary data suggest that modification of the on-treatment tumor biopsy timepoint would be more appropriate, alternative on-treatment tumor biopsy timepoints could be considered in the future cohorts.

The baseline and on-treatment biopsies should preferentially be taken from the same tumor lesion to ensure comparability when accessible in a non-critical location. Data from FDG-PET should guide which lesion would be more reflective of capturing the PD effect and thus the baseline and on-treatment biopsies should be taken from that tumor lesion.

For patients continuing in the study, additional biopsies may be taken at the discretion of the investigator. These biopsies may serve to evaluate viable tumor given the mechanism of action of RO6958688 to induce tumor inflammation. For patients discontinuing from the study due to disease progression, additional optional biopsies,

"Use of this document is governed by the terms of use on the first page of this document."

if clinically feasible, may be taken to aid the understanding of immune resistance mechanisms. Additional biopsies may be taken at the discretion of the investigator to make decisions regarding patient status, and a portion of this biopsy should be sent to Roche (or their designated laboratory) for analysis.

Tumor biopsies will be centrally analyzed for immune cell number and activation by flow cytometric and/or immunohistochemical methods for the density of different immune cell lineages (including but not limited to CD4+, CD8+, B cells, NK cells, macrophages) and their activation and differentiation status (including but not limited to CD25, Ki67, PD1) and for the expression of tumor markers such as PD-L1, IDO etc. In addition, analysis of gene expression, genetic alteration and the infiltrating T cell repertoire may be carried out. The goals of these analyses will be (i) to establish a dose–response and/or an exposure-response relationship and (ii) to understand the MoA of RO6958688 in combination with atezolizumab at the tumor site.

Positron Emission Tomography (PET)

FDG-PET can identify sign of biological effect early, before tumor size is reduced. Moreover, a reduction in the FDG-PET signal within days or weeks of initiating therapy (e.g., in lymphoma, non–small cell lung, and esophageal cancer) has been shown to correlate with prolonged survival and other clinical end points now used (Weber). These findings suggest that FDG-PET could facilitate drug development as an early marker of drug effect.

Whole-body PET should begin 60 ± 10 min after FDG injection. FDG-PET should be performed according to the schedule of assessment in all eligible patients of the study (see FDG PET Appendix 9 for more details).

Archival Tumor Tissue

Formalin–fixed archival tumor tissue embedded in paraffin is to be assessed for CEA expression from all patients for confirmation of CEA status before or at screening (see Appendix 1). These tissues are preferably from the most recent metastatic site (or site of local recurrence) but may be from the primary tumor if metastatic tumor tissue is unavailable. If archival tissue is unavailable, then a fresh tumor biopsy must be obtained for assessment of CEA expression at screening and, if the patient is enrolled. Further other exploratory biomarker assessments, including but not limited to analysis of microsatellite instability and genomic mutations, may be carried out.

If available, formalin–fixed archival tumor tissue embedded in paraffin blocks is to be submitted for exploratory biomarker assessments from all patients enrolled. The tissue block submitted is preferably from the sample used for CEA assessment at screening, if possible. Unstained slides will be accepted if despite all efforts, tumor blocks cannot be obtained.

"Use of this document is governed by the terms of use on the first page of this document."

Clinical Genotyping Samples

A mandatory whole blood sample from all patients of the study will be collected at baseline for DNA extraction. The DNA may be used to determine if genes associated with immune responses including but not limited to chemotaxis, HLA, and immunosuppression affect the pharmacokinetics, pharmacodynamics, efficacy, and safety of RO6958688 in combination with atezolizumab. In addition, the DNA may be used as a reference to identify tumor mutations. These samples will be destroyed after analysis completion and after the results have been checked.

The blood, serum and plasma samples will be destroyed within 2 years after the date of final closure of the clinical database. Archival tumor blocks will be returned. Other residual tissue material (e.g., slides, extracts, on-study blocks, etc.) will be destroyed within 2 years after the final closure of the clinical database unless the patient provides specific consent for the remainder of the tissue sample(s) to be stored for optional exploratory research. If the patient provides consent for optional exploratory research, the tissue samples will be destroyed no later than 15 years after the date of final closure of the clinical database.

For sampling procedures, storage conditions, and shipment instructions, see the separate laboratory manual.

4.5.1.7 Disease-Specific Assessments

Tumor and Response Evaluations

Tumor response will be evaluated according to both RECIST v1.1 and modified RECIST criteria with the use of unidimensional measurement (see Appendix 4 and Appendix 5, respectively). Assessment of CT/MRI scans as tumor assessments will be performed at the sites during the whole study, and in addition centrally by an independent reviewer for prospective and retrospective analysis. Advanced volumetric analyses of CT/MRI will be performed. For central reading analysis, outputs will be produced using immune-related response criteria (Nishino et al. 2014).

Response will be assessed by the investigator on the basis of physical examinations (CT scans or MRI) of chest, abdomen, and pelvis. Ultrasound and X-rays are not acceptable for monitoring target lesions. All measurable disease must be documented at screening and reassessed at each subsequent tumor evaluation. Consistency of consecutive CT scans or MRIs should be ensured during all assessments for each patient; the same method of assessment and the same technique must be used to evaluate lesions throughout the entire study. Use of spiral CT or MRI is required for baseline lesions <20 mm and must be documented in medical records and used consistently throughout the study. The same radiographic procedure that is used to define measurable disease sites at screening must be used throughout the study (e.g., the same contrast protocol for CT scans). Tumor measurements should be made by the same investigator/ radiologist for each patient during the study to the extent that this is feasible.

“Use of this document is governed by the terms of use on the first page of this document.”

In case of clinically measurable superficial lesions such as skin lesions, repeated photographs should be used to document tumor response. These photos must include a ruler for documentation purposes.

If more than one method of assessment is used, the most accurate method is selected according to RECIST v1.1 and modified RECIST criteria, respectively, when recording data. At the investigator's discretion, CT scans may be repeated at any time if progressive disease is suspected.

If available, an assessment of tumor growth kinetics will be made by comparing post-treatment scans with the last available pre-study scan.

Modified RECIST Criteria

Modified RECIST (Appendix 5) is derived from RECIST, Version 1.1 conventions and immune-related response criteria (irRC).

Because of a delayed onset of tumor response that may be caused by immunotherapy, in addition to borderline progression, apparent radiologic progression with improving clinical status, or mixed responses, confirmation of progressive disease may be necessary. In the absence of clinical deterioration, any initial assessment of radiological progressive disease or mixed response should be confirmed by a repeat evaluation at the next timepoint for tumor assessment.

As described in Section 4.6, the criteria below are needed for continuation of treatment beyond the initial apparent progressive disease per RECIST v1.1 (e.g., radiological progression secondary to tumor inflammation):

- Absence of clinical deterioration and investigator–assessed potential clinical benefit for the patient and
- The patient is tolerating study drugs.

Scheduling of Tumor Assessments

Tumor assessments will be performed once during screening for all patients. The first assessment after the start of treatment will be done at 8 weeks (C3D15), and the subsequent assessments will continue every 8 weeks thereafter for the first year and every 12 weeks thereafter until disease progression or treatment discontinuation. All tumor assessments after baseline may be done ± 7 days of the scheduled visit.

Baseline total tumor burden must be assessed within a maximum of 4 weeks before the first dose of study drug treatments. If performed within a reasonable time window prior to signature of the Informed Consent Form (ICF), the assessment can be accepted if agreed with the Sponsor and if done as SOC and according to RECIST v1.1 and modified RECIST criteria in a qualified facility in which all further scans for this patient will be performed. Post-baseline assessments must not be delayed (even if administration/cycle days are delayed) and are to be performed as indicated above until

"Use of this document is governed by the terms of use on the first page of this document."

progression, unacceptable toxicity, or withdrawal of consent. The same radiographic procedure used to define measurable and/or evaluable disease at study entry must be used throughout the study. If there is a suspicion of disease progression based on clinical or laboratory findings before the next scheduled assessment, an unscheduled assessment should be performed.

Tumor response will be confirmed a minimum of 28 days after the initial response was noted or at the next scheduled tumor assessment if it is to occur more than 28 days after the initial response.

If a patient inadvertently misses a prescribed tumor evaluation or a technical error prevents the evaluation, the patient may continue treatment until the next assessment and an unscheduled tumor assessment should be planned as soon as possible.

In patients who are not in progression at time of study discontinuation, the same metastatic workup to cover all target and non-target lesions should be performed at the safety follow-up visit.

FDG-PET

[^{18}F]-Fluorodeoxyglucose (FDG) is a radioactive analogue of glucose which is taken up avidly in most malignancies and is used to visualize cancer and its metastases, usually as whole body images.

Patients who have had a hypersensitivity reaction to FDG will be excluded from FDG-PET. However, the patient will be eligible for the remainder of the study. Diabetic patients with high glucose levels (target blood glucose level $\leq$ 180 mg/dL) that can't be adequately controlled may be excluded from FDG-PET at the discretion of the investigator. PET involves exposure to ionizing radiation. The level of this exposure should be estimated and local guidelines for approval need to be followed.

[^{18}F]-FDG-PET will be performed according to the schedule of assessment as a non-invasive method for determining drugs effect on the metabolic activity of tumor. The patient should fast for 4-6 hours prior to the [^{18}F]-FDG-PET investigation. The patient should rest after the tracer administration and scanning will start 60 minutes ± 10 minutes after tracer administration. The patient is examined with sufficient number of bed positions to ensure coverage over the area from the lower part of the brain (inclusive) to mid-thigh. It is also essential that the patient is examined under the same conditions in baseline and follow up scan, specifically with respect to uptake time and scanning time. Additionally, it is important that the same model of scanner is used or at least that the same spatial resolution is ensured.

Blood glucose concentration needs to be measured immediately prior to the tracer injection and appropriate action needs to be taken for diabetic patients. The scanning procedure should be performed closely to NCI as described in Appendix 9.

"Use of this document is governed by the terms of use on the first page of this document."

[^{18}F]-FDG-PET will be performed at baseline prior to biopsy. It is important to be able to follow the biopsied lesion (selected target lesion as per RECIST) in the PET procedure for screening as well as on study visits. On-study FDG-PET assessment at week 4 (C2D8 + 1 week) and week 16 (C6D8) will be performed concurrent with RECIST assessment whenever possible as shown in the Schedule of assessments Appendix 1.

Patients with no evidence of FDG uptake at the screening PET assessment will not be required to undergo the on-study assessment. For patients with suggestion of PD on follow-up FDG-PET assessments, a confirmatory CT/MRI assessment is strongly encouraged.

Assessment of FDG-PET will be performed centrally by an independent reviewer and per local site assessment.

4.5.1.8 Samples for Roche Clinical Repository

Overview of the Roche Clinical Repository

The Roche Clinical Repository (RCR) is a centrally administered group of facilities for the long-term storage of human biologic specimens, including body fluids, solid tissues, and derivatives thereof (e.g., DNA, RNA, proteins, peptides). The collection and analysis of RCR specimens will facilitate the rational design of new pharmaceutical agents and the development of diagnostic tests, which may allow for individualized drug therapy for patients in the future.

Specimens for the RCR will be collected from patients who give specific consent to participate in this optional research. RCR specimens will be used to achieve the following objectives:

- To study the association of biomarkers with efficacy, adverse events, or disease progression
- To increase knowledge and understanding of disease biology
- To study drugs response, including drugs effects and the processes of drugs absorption and disposition
- To develop biomarker or diagnostic assays and establish the performance characteristics of these assays.

Approval by the Institutional Review Board or Ethics Committee

Collection and submission of biological samples to the RCR is contingent upon the review and approval of the exploratory research and the RCR portion of the Informed Consent Form by each site's Institutional Review Board or Ethics Committee (IRB/EC) and, if applicable, an appropriate regulatory body. If a site has not been granted approval for RCR sampling, this section of the protocol will not be applicable at that site.

"Use of this document is governed by the terms of use on the first page of this document."

Sample Collection

The following samples will be collected for identification of genetic (inherited) biomarkers:

- Whole blood for extraction of DNA and RNA to examine possible predictive biomarkers, including but not limited to the following: variants in immune genes or other genes of interest.

For all samples, dates of consent and specimen collection should be recorded on the associated RCR page of the eCRF. For sampling procedures, storage conditions, and shipment instructions, see the separate laboratory manual.

Patients will also have the option to consent that any tissue material remaining after protocol defined analysis can be stored for up to 15 years in the RCR

RCR specimens will be destroyed no later than 15 years after the date of final closure of the associated clinical database. The RCR storage period will be in accordance with the IRB/EC-approved Informed Consent Form and applicable laws (e.g., health authority requirements).

The dynamic biomarker specimens will be subject to the confidentiality standards described in Section 8.4. The genetic biomarker specimens will undergo additional processes to ensure confidentiality, as described below.

Confidentiality

Given the sensitive nature of genetic data, Roche has implemented additional processes to ensure study subject confidentiality for RCR specimens and associated data. Upon receipt by the RCR, each specimen is "double-coded" by replacing the study subject identification number with a new independent number. Data generated from the use of these specimens and all clinical data transferred from the clinical database and considered relevant are also labeled with this same independent number. A "linking key" between the study subject identification number and this new independent number is stored in a secure database system. Access to the linking key is restricted to authorized individuals and is monitored by audit trail. Legitimate operational reasons for accessing the linking key are documented in a standard operating procedure. Access to the linking key for any other reason requires written approval from the Pharma Repository Governance Committee and Roche's Legal Department, as applicable.

Data generated from RCR specimens must be available for inspection upon request by representatives of national and local health authorities, and Roche monitors, representatives, and collaborators, as appropriate.

Patient medical information associated with RCR specimens is confidential and may be disclosed to third parties only as permitted by the Informed Consent Form (or separate authorization for use and disclosure of personal health information) signed by the patient, unless permitted or required by law.

"Use of this document is governed by the terms of use on the first page of this document."

Data derived from RCR specimen analysis on individual patients will generally not be provided to study investigators unless a request for research use is granted. The aggregate results of any research conducted using RCR specimens will be available in accordance with the effective Roche policy on study data publication.

Any inventions and resulting patents, improvements, and/or know-how originating from the use of the RCR data will become and remain the exclusive and unburdened property of Roche, except where agreed otherwise.

Consent to Participate in the Roche Clinical Repository

The Informed Consent Form will contain a separate section that addresses participation in the RCR. The investigator or authorized designee will explain to each patient the objectives, methods, and potential hazards of participation in the RCR. Patients will be told that they are free to refuse to participate and may withdraw their specimens at any time and for any reason during the storage period. A separate, specific signature will be required to document a patient's agreement to provide optional RCR specimens. Patients who decline to participate will not provide a separate signature.

The investigator should document whether or not the patient has given consent to participate by completing the RCR Research Sample Informed Consent eCRF.

In the event of an RCR participant's death or loss of competence, the participant's specimens and data will continue to be used as part of the RCR research.

Withdrawal from the Roche Clinical Repository

Patients who give consent to provide RCR specimens have the right to withdraw their specimens from the RCR at any time for any reason. After withdrawal of consent, any remaining samples will be destroyed or will no longer be linked to the patient. However, if RCR samples have been tested prior to withdrawal of consent, results from those tests will remain as part of the overall research data. If a patient wishes to withdraw consent to the testing of his or her specimens, the investigator must inform the Medical Monitor in writing of the patient's wishes using the RCR Subject Withdrawal Form and, if the trial is ongoing, must enter the date of withdrawal on the RCR Research Sample Withdrawal of Informed Consent eCRF.

If a patient wishes to withdraw consent to the testing of his or her RCR samples after closure of the site, the investigator must inform the Sponsor by emailing the study number and patient number to the following email address:

global_rcr-withdrawal@roche.com

A patient's withdrawal from Study WP29945 does not, by itself, constitute withdrawal of specimens from the RCR. Likewise, a patient's withdrawal from the RCR does not constitute withdrawal from Study WP29945.

"Use of this document is governed by the terms of use on the first page of this document."

Monitoring and Oversight

RCR specimens will be tracked in a manner consistent with Good Clinical Practice by a quality-controlled, auditable, and appropriately validated laboratory information management system, to ensure compliance with data confidentiality as well as adherence to authorized use of specimens as specified in this protocol and in the Informed Consent Form. Roche monitors and auditors will have direct access to appropriate parts of records relating to patient participation in the RCR for the purposes of verifying the data provided to Roche. The site will permit monitoring, audits, IRB/EC review, and health authority inspections by providing direct access to source data and documents related to the RCR samples.

4.5.2 Timing of Study Assessments

4.5.2.1 Screening and Pretreatment Assessments

Written informed consent for participation in the study must be obtained before performing any study-specific screening tests or evaluations. Informed Consent Forms for enrolled patient and for patients who are not subsequently enrolled will be maintained at the study site.

All screening and pre-treatment assessments must be completed and reviewed to confirm that patients meet all eligibility criteria. The investigator will maintain a screening log to record details of all patients screened and to confirm eligibility or record reasons for screening failure.

An Eligibility Screening Form (ESF) documenting the investigator's assessment of each screened patient with regard to the protocol's inclusion and exclusion criteria is to be completed by the investigator and kept at the investigational site.

Screening and pre-treatment assessments will be performed within 28 days prior to Cycle 1 Day 1 pre-dose unless otherwise specified. Serum pregnancy test will be done 7 days prior to first dose of RO6958688 and/or atezolizumab. Where the clinical significance of an abnormal screening test result (lab or any other tests) is considered uncertain, the test may be repeated. Screening tumor assessments performed in a reasonable time window before ICF signature can be accepted if agreed with Sponsor and if done as standard of care and according to RECIST 1.1 and modified RECIST criteria in a qualified facility in which all further scans for the patient will be performed.

The following will be assessed during Screening (before enrollment); see Schedule of Assessments and Hourly Assessment tables (see Appendix 1):

- Informed consent
- Medical history, including demographics
- Complete physical examination (including height and weight) and ECOG performance status
- Vital signs (including oxygen saturation measured by pulse oximeter – finger clip at rest and exertion)

"Use of this document is governed by the terms of use on the first page of this document."

- Triplicate 12-lead ECGs
- Hematology
- Biochemistry
- Coagulation
- Urinalysis (dipstick)
- HBV and HCV serology
- Serum pregnancy test (in women with child bearing potential)
- Thyroid-stimulating hormone free T3 (or total T3 for sites where free T3 is not performed) and T4
- Autoantibodies panel
- Primary archival tumor block (if available)
- Assessment of tumor CEA expression (refer to inclusion criteria #13 and #14)
- Tumor assessment
- FDG-PET
- Concomitant medications
- AEs
- Pulmonary function tests (FEV1/VC/TLC and derived parameters and D_{LCO}) for patients with bilateral lung metastasis or patients with lobectomy or pneumonectomy with lung metastases in the remaining lung

For further details for hematology, blood biochemistry, coagulation and urinalysis please see Section 4.5.1.5.

The following will be assessed during Baseline (after enrollment, before first doses); see Schedule of Assessments and Hourly Assessment tables (see Appendix 1):

Clinical genotyping, PK determination, ADA and PD blood/plasma samples and safety assessments. Tumor biopsy can be obtained up to 28 days before first administration of RO6958688 and atezolizumab.

4.5.2.2 Assessments during Treatment

Under no circumstances will patients who enroll in this study and have completed treatment as specified, be permitted to be allocated a new number and re-enroll in the study.

All assessments must be performed as per SoA and Hourly Assessment tables (see Appendix 1). Assessments scheduled on the day of study treatment administration should be performed prior to administration of study treatments, unless otherwise noted in the SoA and Hourly Assessment tables. A 24-h (72-h if during weekend) window prior to drugs administration for hematology, biochemistry, coagulation, urinalysis, and physical examinations/ECOG is allowed.

"Use of this document is governed by the terms of use on the first page of this document."

The following assessments will be performed as indicated in the SoA:

- Tumor Assessment (according to RECIST 1.1 and modified RECIST criteria) evaluated by chest-abdominal CT scan (or MRI). The same radiographic procedure used to define measurable and/or evaluable disease at study entry must be used throughout the study
- FDG-PET assessment
- PK (RO6958688 and atezolizumab)
- PD plasma and serum and additional safety
- PD whole blood (FC)
- ADA (anti-RO6958688 and anti-atezolizumab)

For the following assessments, performed as indicated in the Schedule of Assessments (see Appendix 1), results must be available before dosing (except for sCD25):

- Complete physical exam, vital signs including body temperature, respiratory rate, heart rate, diastolic and systolic BP, oxygen saturation), and weight assessment
- Triplicate 12-lead ECGs (recording must be done prior to PK sampling)
- ECOG performance status
- Hematology
- Biochemistry
- Coagulation
- Urinalysis
- Urine pregnancy test (in women with child bearing potential)
- Thyroid-stimulating hormone free T3 (or total T3 for sites where free T3 is not performed) and T4.

Note that all patients enrolled in the study will be required to stay in the hospital overnight following administration of study drugs on Cycle 1 Day 1.

If a Grade 3 or higher adverse event related to RO6958688 was observed within 24 hours of the previous RO6958688 infusion, an observation period of at least 8 hours (at least 24 hours for Grade 3 IRR/CRS) after completion of the subsequent RO6958688 infusion is required (for further details, see Appendix 1).

For further details for hematology, blood biochemistry, coagulation, and urinalysis, see Section 4.5.1.5.

4.5.2.3 Assessments at Study Completion/Early Termination Visit

Patients who complete the study or discontinue from the study early will be asked to return to the clinic 28 days after the last dose of RO6958688 or atezolizumab for safety follow-up visits. The visit at which response assessment shows progressive disease may be used as the study completion/early termination visit.

"Use of this document is governed by the terms of use on the first page of this document."

The following assessments should be done at Study Completion/Early Termination Visit (for further details, see Appendix 1):

- Complete physical exam, vital signs (including body temperature, respiratory rate, heart rate, diastolic and systolic BP, oxygen saturation)
- Triplicate 12-lead ECGs (recording must be done prior to PK sampling)
- ECOG performance status
- Hematology
- Biochemistry
- TSH free T3 (or total T3 for sites where free T3 is not performed) and T4
- Coagulation
- Urinalysis
- Serum pregnancy test (in women with child bearing potential)
- PK (RO6958688 and atezolizumab)
- ADA (anti-RO6958688 and anti-atezolizumab)
- Soluble CEA
- PD whole blood
- Tumor Assessment (according to RECIST 1.1 and modified RECIST criteria) evaluated by chest-abdominal CT scan (or MRI). The same radiographic procedure used to define measurable and/or evaluable disease at study entry must be used throughout the study.

4.5.2.4 Follow-Up Assessments

The following assessments should be done at 28-day Safety follow-up visit (for further details, see Appendix 1):

- Complete physical exam, vital signs (including body temperature, respiratory rate, heart rate, diastolic and systolic BP, oxygen saturation)
- Triplicate 12-lead ECGs
- ECOG performance status
- Hematology
- Biochemistry
- TSH free T3 (or total T3 for sites where free T3 is not performed) and T4
- Coagulation
- Urinalysis
- Serum pregnancy test (in women with child bearing potential)
- PK (RO6958688 and atezolizumab)
- ADA (anti-RO6958688 and anti-atezolizumab).
- PD whole blood

"Use of this document is governed by the terms of use on the first page of this document."

Post-study anti-cancer therapy should be collected and reported as appropriate in the eCRF.

Post-study survival follow-up:

The sites will provide to the Sponsor every three months (starting at *Day 28* visit) an update on survival status of each of the patients enrolled in the study. The sites will use designated section of the eCRF for this purpose.

After the study completion/early termination visit, adverse events should be followed as outlined in Sections 5.5 and 5.6.

4.5.2.5 Assessments at Unscheduled Visits

Please see Appendix 1 for assessments that are required to be performed in case of an unscheduled visit.

4.6 PATIENT, STUDY, AND SITE DISCONTINUATION

4.6.1 Patient Discontinuation

Patients have the right to voluntarily withdraw from the study at any time for any reason. In addition, the investigator has the right to withdraw a patient from the study at any time. Reasons for withdrawal from the study may include, but are not limited to, the following:

- Patient withdrawal of consent at any time
- Any medical condition that the investigator or Sponsor determines may jeopardize the patient's safety if he or she continues in the study
- Investigator or Sponsor determines it is in the best interest of the patient
- Patient non-compliance.

Every effort should be made to obtain information on patients who withdraw from the study. The primary reason for withdrawal from the study should be documented on the appropriate eCRF. However, patients will not be followed for any reason after consent has been withdrawn. Patients who withdraw from the study will not be replaced, with the exception of patients during the dose-escalation stage of each arm who withdraw or are withdrawn from the study prior to completing the DLT assessment window for any reason other than a DLT.

All patients will attend a 28-day safety follow-up visit after receiving the last infusion of RO6958688 and atezolizumab.

4.6.1.1 Discontinuation from Study Drugs

Patients will be treated for 24 months or longer if emerging data suggest this, until loss of clinical benefit, unacceptable toxicities, loss of exposure (RO6958688 concentration BLQ or close to LLOQ of 2.0 ng/mL at end of infusion, in which case patients can continue to receive atezolizumab alone), or withdrawal from treatment for other reasons or death.

"Use of this document is governed by the terms of use on the first page of this document."

Patients must discontinue study treatments if they experience any of the following:

- Symptomatic deterioration attributed to disease progression as determined by the investigator after integrated assessment of radiographic data, biopsy results, and clinical status
- Intolerable toxicity related to study treatment, including development of an immune-related adverse event (irAE) determined by the investigator and Medical Monitor to be unacceptable given the individual patient's potential response to therapy and severity of the event (see Section 5.2.6.1)
- Any medical condition that may jeopardize the patient's safety if he or she continues on study treatment
- IRR Grade $\geq$ 3 related to atezolizumab
- IRR/CRS related to RO6958688 meeting any of the following criteria:
    - Grade 3 IRR/CRS that does not recover within 8 hours of corticosteroids and tocilizumab treatment or following repeat treatment with tocilizumab for the same event
    - Grade 4 IRR/CRS
    - Grade 3 IRR/CRS recurrence, or G3 IRR/CRS occurrence after previous G2 IRR/CRS
- AST or ALT increase >10 x ULN
- Grade 4 diarrhea, enteritis, or colitis related to RO6958688
- Grade 2 diarrhea, enteritis, or colitis related to RO6958688 that fails to resolve to Grade 1 or better after following the recommended management guidelines in Appendix 10 for this grade
- Grade 3 diarrhea, enteritis, or colitis related to RO6958688 that fails to resolve to Grade 1 or better after following the recommended management guidelines in Appendix 10 for this grade
- Grade 4 pulmonary event related to RO6958688
- Recurrent Grade 3 pulmonary event related to RO6958688
- IgE-mediated hypersensitivity reactions including anaphylaxis
- Use of another non-protocol anti-cancer therapy (see Section 4.4.2)
- Pregnancy.

"Use of this document is governed by the terms of use on the first page of this document."

Patients will be permitted to continue one or both study treatment(s) after RECIST v1.1 criteria for progressive disease are met if they meet all of the following criteria:

- Evidence of clinical benefit as assessed by the investigator
- Absence of symptoms and signs (including worsening of laboratory values; e.g., new or worsening hypercalcemia) indicating unequivocal progression of disease
- No decline in ECOG performance status that can be attributed to disease progression
- Absence of tumor growth at critical anatomical sites (e.g., leptomeningeal disease) that cannot be managed by protocol-allowed medical interventions
- Patients for whom approved therapies exist must provide written consent to acknowledge deferring these treatment options in favor of continuing study treatment at the time of initial apparent progression.

Patients in whom radiographic disease progression is confirmed at a subsequent tumor assessment may be considered for continued study treatment at the discretion of the investigator if they continue to meet the criteria above and have evidence of clinical benefit (Figure 6). At the time of progression, patients can receive therapies such as: radiotherapy, radio-ablation or surgery.

“Use of this document is governed by the terms of use on the first page of this document.”

Figure 6 Conditions for Continuing RO6958688 and/or Atezolizumab in the Presence of Increased Radiographic Tumor Size

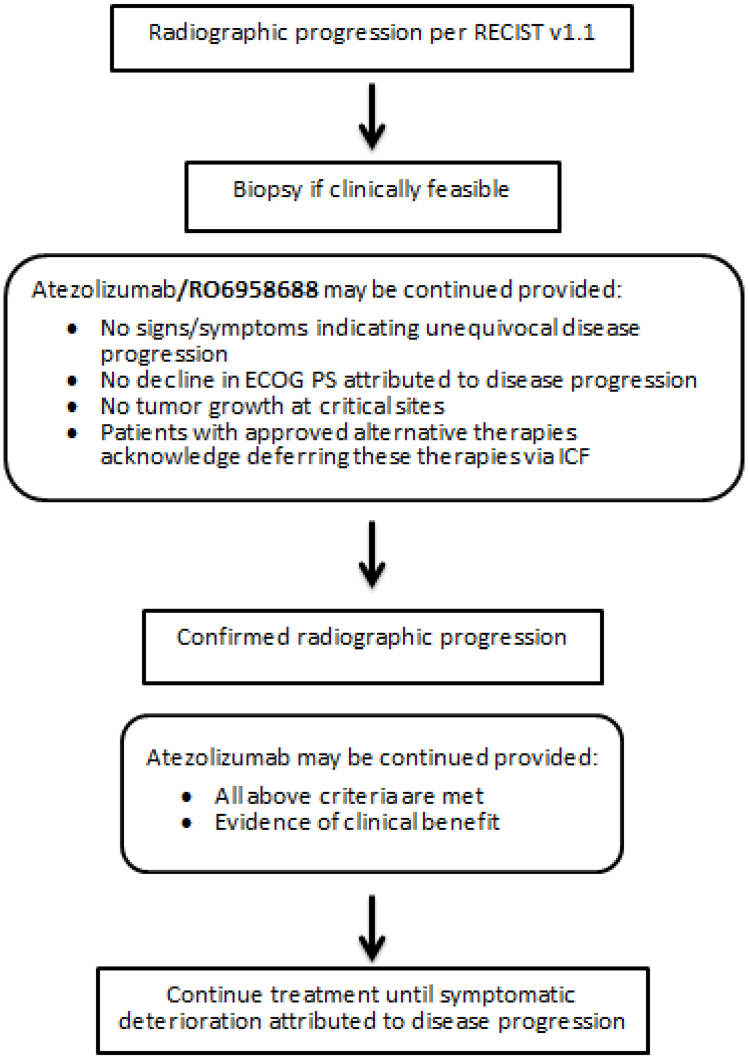

ECOG=Eastern Cooperative Oncology Group; ICF=Informed Consent Form; PS=performance status; RECIST=Response Evaluation Criteria in Solid Tumors.

Patients who discontinue study drugs prematurely will be asked to return to the clinic for a study completion/early termination visit (see Section 4.5.2.3) and may undergo follow-up assessments (see Section 4.5.2.3). The primary reason for premature study drugs discontinuation should be documented on the appropriate eCRF.

“Use of this document is governed by the terms of use on the first page of this document.”

4.6.1.2 Withdrawal from Study

Every effort should be made to obtain information on patients who withdraw from the study. The primary reason for withdrawal from the study should be documented on the appropriate eCRF.

If a patient withdraws consent prior to the first treatment administration, the patient will be replaced. Patients will not be followed for any reason after consent has been withdrawn.

During the study, patients who withdraw before the end of the DLT period (i.e., within 21 days of the first dose) for reasons other than DLTs will be replaced to ensure that at least 3 patients have been assessed for a full DLT period prior to moving to the next dose level.

4.6.2 Study and Site Discontinuation

The Sponsor has the right to terminate this study at any time. Reasons for terminating the study may include, but are not limited to, the following:

- The incidence or severity of adverse events in this or other studies indicates a potential health hazard to patients
- Patient enrollment is unsatisfactory.

The Sponsor will notify the investigator and Health Authorities if the study is placed on hold, or if the Sponsor decides to discontinue the study or development program.

The Sponsor has the right to replace a site at any time. Reasons for replacing a site may include, but are not limited to, the following:

- Excessively slow recruitment
- Poor protocol adherence
- Inaccurate or incomplete data recording
- Non-compliance with the International Conference on Harmonisation (ICH) guideline for Good Clinical Practice.

5. ASSESSMENT OF SAFETY

5.1 SAFETY PARAMETERS AND DEFINITIONS

Safety assessments will consist of monitoring and recording adverse events, including serious adverse events and non-serious adverse events of special interest; measurement of protocol-specified safety laboratory assessments; measurement of protocol-specified vital signs, ECGs; and other protocol-specified tests that are deemed critical to the safety evaluation of the study.

Certain types of events require immediate reporting to the Sponsor, as outlined in Sections 5.1.3 and 5.4.

"Use of this document is governed by the terms of use on the first page of this document."

5.1.1 Adverse Events

According to the ICH guideline for Good Clinical Practice, an adverse event is any untoward medical occurrence in a clinical investigation subject administered a pharmaceutical product, regardless of causal attribution. An adverse event can therefore be any of the following:

- Any unfavorable and unintended sign (including an abnormal laboratory finding), symptom, or disease temporally associated with the use of a medicinal product, whether or not considered related to the medicinal product
- Any new disease or exacerbation of an existing disease (a worsening in the character, frequency, or severity of a known condition), except as described in Section 5.3.5.10
- Recurrence of an intermittent medical condition (e.g., headache) not present at baseline
- Any deterioration in a laboratory value or other clinical test (e.g., ECG) that is associated with symptoms or leads to a change in study treatment or concomitant treatment or discontinuation from study drugs
- Adverse events that are related to a protocol-mandated intervention, including those that occur prior to assignment of study treatment (e.g., screening invasive procedures such as biopsies).

5.1.2 Serious Adverse Events (Immediately Reportable to the Sponsor)

A serious adverse event is any adverse event that meets any of the following criteria:

- Fatal (i.e., the adverse event actually causes or leads to death)
- Life threatening (i.e., the adverse event, in the view of the investigator, places the patient at immediate risk of death)

This does not include any adverse event that had it occurred in a more severe form or was allowed to continue might have caused death.

- Requires or prolongs inpatient hospitalization (see Section 5.3.5.11)
- Results in persistent or significant disability/incapacity (i.e., the adverse event results in substantial disruption of the patient's ability to conduct normal life functions)
- Congenital anomaly/birth defect in a neonate/infant born to a mother exposed to study drugs
- Significant medical event in the investigator's judgment (e.g., may jeopardize the patient or may require medical/surgical intervention to prevent one of the outcomes listed above).

"Use of this document is governed by the terms of use on the first page of this document."

The terms “severe” and “serious” are not synonymous. Severity refers to the intensity of an adverse event (rated as mild, moderate, or severe, or according to a pre-defined grading criteria (e.g., NCI CTCAE criteria; see Section 5.3.3); the event itself may be of relatively minor medical significance (such as severe headache without any further findings).

Severity and seriousness need to be independently assessed for each adverse event recorded on the eCRF.

Serious adverse events are required to be reported by the investigator to the Sponsor immediately (i.e., no more than 24 hours after learning of the event; see Section 5.4.2 for reporting instructions).

5.1.3 Non-Serious Adverse Events of Special Interest (Immediately Reportable to the Sponsor)

Non-serious adverse events of special interest are required to be reported by the investigator to the Sponsor immediately (i.e., no more than 24 hours after learning of the event; see Section 5.4.2 for reporting instructions). Adverse events of special interest for this study include the following, depending on causality assessment of the adverse event, if:

1. **Related to RO6958688**

- Cases of an elevated ALT or AST in combination with either an elevated bilirubin or clinical jaundice, as defined in Section 5.3.5.7
- Grade ≥ 2 CRS and IRR
- Grade ≥ 2 hypersensitivity
- Suspected transmission of an infectious agent by the study drugs, as defined below:
    - Any organism, virus, or infectious particle (e.g., prion protein transmitting transmissible spongiform encephalopathy), pathogenic or non-pathogenic, is considered an infectious agent. A transmission of an infectious agent may be suspected from clinical symptoms or laboratory findings that indicate an infection in a patient exposed to a medicinal product. This term applies only when a contamination of the study drugs is suspected.

2. **Related to Atezolizumab**

- Cases of an elevated ALT or AST in combination with either an elevated bilirubin or clinical jaundice, as defined in Section 5.3.5.7

“Use of this document is governed by the terms of use on the first page of this document.”

- Suspected transmission of an infectious agent by the study drugs, as defined below:
    - Any organism, virus, or infectious particle (e.g., prion protein transmitting transmissible spongiform encephalopathy), pathogenic or non-pathogenic, is considered an infectious agent. A transmission of an infectious agent may be suspected from clinical symptoms or laboratory findings that indicate an infection in a patient exposed to a medicinal product. This term applies only when a contamination of the study drugs is suspected.
- Pneumonitis
- Colitis
- Endocrinopathies: diabetes mellitus, pancreatitis, adrenal insufficiency, or hyperthyroidism
- Hepatitis
- Transaminitis Grade $\geq$2 (AST or ALT >3 $\times$ ULN and bilirubin >2 $\times$ ULN) or AST/ALT >10 $\times$ ULN
- Systemic lupus erythematosus
- Neurological: Guillain-Barre syndrome, myasthenia gravis, meningoencephalitis
- Nephritis
- Events suggestive of hypersensitivity, cytokine release syndrome, influenza like illness, systemic inflammatory response syndrome (SIRS), *and* infusion reactions syndrome.

5.2 SAFETY PLAN

5.2.1 Dose Modifications and Delays

The RO6958688 dose should not be escalated beyond 150 mg in any cohorts of the study. Patients who are already at doses higher than 150 mg should not be escalated any further, but can stay at their current RO6958688 dose levels following discussion with the Medical Monitor if the therapy is adequately tolerated.

Should a patient experience a DLT and/or grade ≥ 2 related adverse events in the first or in subsequent cycles or experience the same toxicity of same or higher grade following re-exposure to atezolizumab and RO6958688 combination, the investigator, after discussion with the Sponsor, will have the option to reduce the dose of RO6958688 and/or to delay the RO6958688 dose up to 14 days in the QW part and up to 21 days in the Q3W part to prevent the recurrence of adverse events. This can be done to allow patients who could potentially benefit from the combination of atezolizumab and RO6958688 to remain on the study drugs.

“Use of this document is governed by the terms of use on the first page of this document.”

Prior to dosing (Day 1, Day 8 and Day 15 at each cycle), toxicities from previous administration should be resolved in individual patient:

- For treatment-related non-hematological blood parameters abnormalities the values of total albumin, creatinine, GGT, must be resolved to screening baseline values or as defined by NCI CTCAE Grade ≤ 1. Total bilirubin levels must be resolved to screening values or as defined by NCI-CTCAE Grade ≤ 1, AST and/or ALT must be resolved to $\leq 3 \times$ ULN.
- Regarding fever, patients can continue treatment with RO6958688 if fever is Grade ≤ 1.
- Other non-hematological toxicities should be resolved to baseline values or Grade ≤ 1 (or Grade ≤ 2 if considered non-clinically significant by the investigator).
- For hematological blood parameters the blood values of thrombocyte, erythrocyte, and combination of erythrocyte, lymphocyte and thrombocyte must be resolved to screening baseline values or as defined by NCI-CTCAE Grade ≤ 2.

Investigators should contact the Sponsor before the next administration in case a patient experienced a change in blood inflammatory/immune activation parameters like serum CRP, sCD25 and serum ferritin and/or seroconversion of at least one of the autoantibodies (anti-nuclear antibody, anti-double-stranded DNA, cytoplasmic anti-neutrophil cytoplasmic antibody, and perinuclear anti-neutrophil cytoplasmic antibody).

A delay of RO6958688 administration for up to 14 days in the QW part and up to 21 days in the Q3W part will be acceptable to allow for resolution of toxicity NCI CTCAE as described above (with the exception of toxicity considered as non-RO6958688 related). No other delays of RO6958688 administration are foreseen.

There will be no dose reduction for atezolizumab in this study. Patients may temporarily suspend study treatment for up to 105 days beyond the last dose if they experience adverse events that require a dose to be withheld. If atezolizumab is withheld because of adverse events for > 12 weeks beyond the last dose, then the patient will be discontinued from atezolizumab and will be followed up for safety and efficacy as specified in Section 4.6.1.

If, in the judgment of the investigator, the patient is likely to derive clinical benefit from atezolizumab after a hold of >12 weeks, study drug may be restarted with the approval of the Medical Monitor.

If a patient must be tapered off steroids used to treat adverse events, atezolizumab may be withheld for additional time beyond 12 weeks from the last dose until steroids are discontinued or reduced to prednisone dose (or dose equivalent) ≤ 10 mg/day. The acceptable length of interruption will depend on an agreement between the investigator and the Medical Monitor.

"Use of this document is governed by the terms of use on the first page of this document."

Dose interruptions for reason(s) other than toxicity, such as surgical procedures, may be allowed with Medical Monitor approval. The acceptable length of interruption will depend on agreement between the investigator and the Medical Monitor.

It should be noted that in case of a dose delay, the infusions/cycles are not considered as missed but as delayed (visits should be followed as per SoA). In case the delay is $\geq$2 weeks the investigator should contact the Medical Monitor.

Management of atezolizumab-specific adverse events is presented in Appendix 11.

For patients participating in the step up cohorts who experience Grade 3 related adverse events associated with tumor inflammation:

- For patients who are in the step-up phase of the QW – Q3W dosing scheme, patient management should follow the guidelines provided in Appendix 10; the event should be resolved to Grade 1 prior to resuming RO6958688 treatment. The next RO6958688 dose for such patients will be up to or at the same level of RO6958688 dose that caused the event in the dose escalation phase.

- For patients who have reached the flat dose phase (Q3W), the event should be resolved to Grade 1 prior to resuming RO6958688 treatment and the patient should continue with the per-protocol planned dose. However, the Investigator, after discussion with the Sponsor, will have the option to reduce the dose of RO6958688 to a lower dose level or to allow for a more convenient dose/schedule to prevent the recurrence of adverse events and limit toxicity.

For patients participating in the step up cohorts who experience Grade 2 IRR/CRS considered related to RO6958688:

- For patients who are in the step-up phase of the QW – Q3W dosing scheme, patient management should follow the guidelines provided in Appendix 10. The next RO6958688 dose for such patients will be up to or at the same level of the RO6958688 dose that caused the event in the dose escalation phase.

- For patients who have reached the flat dose phase (Q3W), patient management should follow the guidelines provided in Appendix 10. The Investigator, after discussion with the Sponsor, will have the option to reduce the dose of RO6958688 to a lower dose level or to allow for a more convenient dose/schedule to prevent the recurrence of adverse events and limit toxicity.

Further RO6958688 dose reductions may be implemented once safety and toxicity data from the dose escalation Part I have been evaluated.

“Use of this document is governed by the terms of use on the first page of this document.”

5.2.2 Risks Associated with Atezolizumab

Atezolizumab has been associated with risks such as the following: IRRs, *immune-mediated* hepatitis, pneumonitis, colitis, pancreatitis, diabetes mellitus, hypothyroidism, hyperthyroidism, adrenal insufficiency, hypophysitis, myocarditis, Guillain-Barré syndrome, myasthenic syndrome or myasthenia gravis, meningoencephalitis, myositis, and nephritis. *Immune-mediated reactions may involve any organ system and may lead to hemophagocytic lymphohistiocytosis and macrophage activation syndrome (considered to be potential risks for atezolizumab).*

Please refer to Appendix 11 of the current protocol and Section 6 of the atezolizumab Investigator's Brochure for a detailed description of anticipated safety risks for atezolizumab.

5.2.3 Risks Associated with RO6958688

The following adverse events are classified as identified risks associated with RO6958688: pyrexia, IRR, CRS, hypoxia, hypotension, diarrhea, colitis, tumor inflammation/tumor flare events at the tumor site (tumor pain, dyspnea, hypoxia, increased liver enzymes (AST/ALT), blood bilirubin increased, colitis, enteritis), chills, vomiting, rash, skin exfoliation, nausea, dysgeusia, fatigue, arthritis and arthralgia.

Serious adverse drug reactions which are considered expected and associated with RO6958688 are: pyrexia, IRR, CRS, tumor pain, diarrhea, colitis, dyspnea, hypoxia, hypotension, AST increased, ALT increased and blood bilirubin increased.

The following adverse events are classified as potential risks associated with RO6958688: decreased appetite, IgE-mediated hypersensitivity reactions including anaphylaxis, hematotoxicities, and CNS disorders.

An observation period of at least 8 hours is required following completion of the RO6958688 infusion in any treatment administration visit where a Grade 3 or higher adverse event (with the exception of Grade 3 IRR/CRS) was observed within 24 hours of the previous RO6958688 administration. In a situation where the patient experienced a Grade 3 IRR/CRS in the previous administration visit, a 24-hour hospitalization will be required following the completion of RO6958688 infusion at the next administration visit.

For detailed information regarding identified and potential risks associated with RO6958688, refer to Section 6 of the most recent version of the RO6958688 Investigator's Brochure.

For management of specific adverse events, refer to Section 5.2.6.2.

"Use of this document is governed by the terms of use on the first page of this document."

5.2.4 Risks Associated with Combination Use of RO6958688 and Atezolizumab

The following adverse events are classified as identified risks associated with use of RO6958688 in combination with atezolizumab: conjunctivitis, diarrhea and colitis.

The following adverse events are potential risks associated with combination use of atezolizumab and RO6958688, based on the evidence from Study WP29945: IRR, dysphonia, paraesthesia, musculoskeletal pain, pulmonary events, hepatic events, and GI events (e.g., vomiting, nausea, and gastrointestinal pain).

Based on the mode of action and the nonclinical and/or clinical studies with each molecule as a single agent, the evidence indicates a potential for overlapping toxicity in patients treated with a combination of atezolizumab and RO6958688. Heightened anti-tumor T-cell activity locally in the tumor microenvironment represents the primary single-agent adverse events common to both molecules (see Section 6 of the Atezolizumab and RO6958688 Investigator's Brochures, respectively).

No nonclinical toxicology studies have been conducted with the combination of atezolizumab with RO6958688.

5.2.5 Risks Associated with Tocilizumab

Refer to Section 6 of the Tocilizumab Investigator's Brochure for a detailed description of anticipated safety risks for tocilizumab. Because the use of tocilizumab remains experimental in this protocol, it should be noted that the risks contained in the Tocilizumab Investigator's Brochure have been identified in patients treated in alternate settings and for different indications.

5.2.6 Management of Specific Adverse Events

5.2.6.1 Management of Atezolizumab-Specific Adverse Events

Guidelines for the management of atezolizumab-specific adverse events are outlined in Appendix 11.

Toxicities associated or possibly associated with atezolizumab treatment should be managed according to standard medical practice. Additional tests, such as autoimmune serology or biopsies, should be used to determine a possible immunogenic etiology.

Although most immune-mediated adverse events observed with immunomodulatory agents have been mild and self-limiting, such events should be recognized early and treated promptly to avoid potential major complications. Discontinuation of atezolizumab may not have an immediate therapeutic effect. Refer to Appendix 11 for specific management guidelines.

The investigator should consider the benefit-risk balance a given patient may be experiencing prior to further administration of atezolizumab. Atezolizumab should be

"Use of this document is governed by the terms of use on the first page of this document."

permanently discontinued in patients with life-threatening immune- related adverse events.

5.2.6.1.1 Management of Infusion-Related Reactions

Administration of therapeutic antibodies such as atezolizumab may cause IRRs characterized by symptoms such as fever, chills, dizziness, hypertension, hypotension, dyspnea, restlessness, sweating, flushing, skin rash, tachycardia, tachypnoea, headache, tumor pain, nausea, and/or vomiting. Such reactions typically occur during or shortly after an infusion, or within 24 h after study drugs infusion predominantly at the first infusion. The incidence and severity typically decrease with subsequent infusions. Patients may also develop IgE-mediated hypersensitivity reactions to atezolizumab. IRRs may be indistinguishable from an anaphylactic reaction.

However, if a patient experiences an isolated episode of e.g **"**fever**"** within 24 h after study drugs infusion, not accompanied by other IRR-like symptoms as described above, then the AE reporting term will be fever and not IRR.

If an IRR is considered related to atezolizumab, the infusion of atezolizumab should be managed as per Appendix 11. The patient should be monitored until complete resolution of the symptoms and treated as clinically indicated. Treatment or concomitant medication may include acetaminophen/paracetamol, antihistamine, IV saline, oxygen, bronchodilators, corticosteroids, and vasopressors depending on the symptoms. The infusion rate can be re-escalated to initial rate if considered well tolerated after 1 hour of infusion.

IRR related symptoms management should be symptomatic treatment including corticosteroids when required, see Appendix 11 IRR management guidelines.

5.2.6.2 Management of Specific Adverse Events to RO6958688

Refer to the RO6958688 Investigator's Brochure for additional information on the clinical safety of RO6958688.

5.2.6.2.1 Pyrexia

Investigators should provide guidance to patients for the management of isolated (no other signs and symptoms) episode of fever, in particular patient should regularly check their body temperature during the days following each RO6958688 administration and take early intervention with standard anti-pyrexia treatments (e.g., paracetamol, NSAIDs) as current practice. Occasionally, fever is accompanied with symptoms like chills, hypotension, shortness of breath, skin rash, headache, nausea, and/or vomiting, in which case this is considered an IRR/CRS and should be managed with urgency (Appendix 10).

"Use of this document is governed by the terms of use on the first page of this document."

5.2.6.2.2 Adverse Events Associated with Tumor Inflammation/Tumor Flare

Clinical evidence suggests that certain types of adverse events related to RO6958688 may be mediated by its mechanism of action at the tumor site (i.e., intratrumor immune activation and consequent tumor inflammation and flare). These adverse events tend to have a short onset following RO6958688 administration and affect organ systems with tumor lesions.

Tumor inflammation and tumor flare events are either associated with pain at the tumor site (e.g., tumor, abdominal, and GI pain) or affect function of the organ system with the tumor lesion (e.g., dyspnea, hypoxia, colitis, enteritis, and increased levels of liver function enzymes and bilirubin). These adverse events are more common after the first or second dose of RO6958688.

Guidelines for management of patients who experience adverse events associated with tumor inflammation and tumor flare are provided in Appendix 10.

5.2.6.2.3 Management of Gastrointestinal Toxicities: Diarrhea and Colitis

Diarrhea has been commonly observed in patients receiving RO6958688. Diarrhea events are usually transient and reversible. Diarrhea can be one of the manifestations of CRS, and dehydration secondary to diarrhea can worsen the hypotension associated with CRS. Patients should be closely monitored (including monitoring of renal function) and should be hydrated if clinically indicated to prevent renal insufficiency due to fluid depletion. Diarrhea can also be a symptom of undiagnosed colitis. All events of diarrhea or colitis should be thoroughly evaluated for other etiologies.

Guidelines for management of patients who experience diarrhea and colitis are provided in Appendix 10. Even though CTCAE v5 criteria for CRS is driven by the presence of hypotension and hypoxia, please consider use of tocilizumab per CRS management guidelines (Appendix 10) if per investigator clinical judgement diarrhea is deemed secondary to CRS.

5.2.6.2.4 Management of IRR/CRS

Administration of RO6958688 may cause a spectrum of infusion-related adverse events involving IRRs and CRS. The incidence and severity typically decrease with subsequent infusions, although instances of CRS have been reported in later cycles, as described below.

Infusion-related reactions associated with RO6958688 are typically seen with the first two cycles and without symptoms of hypoxia and/or hypotension. In contrast, cytokine-release syndrome is more likely to be associated with symptoms of hypoxia and/or hypotension along with other constitutional symptoms and can occur at any cycle. Please refer to Section 5.3.5 for guidance on recording and Section 5.4.2 for guidance on reporting of infusion-related reaction and cytokine-release syndrome events.

“Use of this document is governed by the terms of use on the first page of this document.”

Given the overlap in signs and symptoms, IRRs may be indistinguishable from CRS, which is defined as a disorder characterized by fever, tachypnea, headache, tachycardia, hypotension, rash, and/or hypoxia caused by the release of cytokines (CTCAE v5). Severe CRS may be associated with other clinical sequelae such as disseminated intravascular coagulation and capillary leak syndrome.

In Study BP29541, as of 23 March 2018, there was one case of Grade 5 IRR that met the criteria of CRS based on symptomatology and cytokine levels, as described below:

A [redacted] year old [redacted] patient enrolled in study BP29541 was reported to have suffered a Grade 5 IRR following the fifth administration of RO6958688 at a dose of 355.99 mg (planned dose 600 mg). The patient is suspected to have had a fatal CRS event following multiple re-treatment attempts with RO6958688 based on a very high serum IL-6 level of 75198.41 pg/mL, elevated levels for IFN-γ (1694.96 pg/mL), IL-8 (4328.17 pg/mL) and TNF-α (767.06 pg/mL) after the fourth administration, with a very similar clinical course at the fifth administration of RO6958688 (Cycle 5 cytokine levels will not be available). This patient had a recurring symptom of Grade 3 hypotension related to RO6958688 infusion at both administrations.

In view of the above data, and given that IRRs may be indistinguishable from CRS based on symptomatology, single treatment guidelines are being recommended for management of IRRs and CRS, during or up to 24 hours after infusion of RO6958688 (Appendix 10, Table 2 and Section 1.5). The approach to have single treatment guidelines for IRR and CRS will continue to be reviewed as further safety data is being accumulated.

Table 3 Cytokine Release Syndrome Grading According to CTCAE v5

Grade 1	Grade 2	Grade 3	Grade 4	Grade 5
Fever with or without constitutional symptoms*	Hypotension responding to fluids; hypoxia responding to <40% O2	Hypotension managed with one pressor; hypoxia requiring ≥ 40% O2	Life-threatening consequences; urgent intervention indicated	Death
Definition: A disorder characterized by fever, tachypnea, headache, tachycardia, hypotension, rash, and/or hypoxia caused by the release of cytokines.				
Navigational Note: Also consider reporting other organ dysfunctions including neurological toxicities such as: Psychiatric disorders: Hallucinations or Confusion; Nervous system disorders: Seizure, Dysphasia, Tremor, or Headache				

*e.g., rigors, malaise, fatigue, anorexia, myalgia, arthralgia, nausea, vomiting and headache

"Use of this document is governed by the terms of use on the first page of this document."

The risk of IRRs/CRS is managed in the clinic with appropriate risk minimization measures. RO6958688 should only be administered under the close supervision of an experienced clinician trained to monitor medical situations and to respond to medical emergencies in a clinical environment with full resuscitation equipment available for immediate use. Patients should receive full supportive care to treat IRRs/CRS according to institutional practice. In clinical studies with RO6958688, patients are required to stay overnight in the hospital after the first dose for clinical monitoring. If infusion-associated signs/symptoms or CRS manifestations occur, patients should be monitored until complete resolution. If a IRRs/CRS develops during the administration of RO6958688, the infusion should be temporarily slowed down or interrupted. Treatment or concomitant medication may include acetaminophen/paracetamol, antihistamine, IV saline, oxygen, bronchodilators, corticosteroids, vasopressors and tocilizumab depending on the symptoms and the severity. Resuscitation equipment should be available for immediate use.

In addition, to reduce the risk of the development of IRRs/CRS in a patient receiving RO6958688, premedication will be administered as per Section 4.3.2.5.

In a situation where the patient experienced a Grade 3 IRR/CRS in the previous administration visit, a 24-hour hospitalization will be required at the completion of the subsequent treatment administration.

5.2.6.2.5 Management of IgE-mediated Hypersensitivity Reactions Including Anaphylaxis

Patients may also develop IgE-mediated hypersensitivity reactions to RO6958688. The signs and symptoms of IRRs/CRS may be indistinguishable from an anaphylactic reaction. Medications including epinephrine, corticosteroids, diphenhydramine hydrochloride for IV injection, and resuscitation equipment should be available for immediate use. Guidelines for management of IRRs/CRS related to RO6958688 are summarized in Appendix 10. Guidelines for management of anaphylaxis are given in Appendix 14.

Patients with IRRs/CRS of Grade 2 or higher, should undergo special laboratory assessments including IgE and tryptase analysis (Appendix 1, Table A3). If the results support an allergic origin (i.e., increased tryptase levels and/or increased IgE levels), the reaction should be reported as an allergic reaction or anaphylactic reaction, as appropriate, and patients should stop treatment with RO6958688.

5.2.6.2.6 Elevated Liver Enzymes and Hepatotoxicity

Transient AST and ALT elevations have been observed in patients receiving RO6958688 and could occur in the setting of tumor inflammation/tumor flare.

“Use of this document is governed by the terms of use on the first page of this document.”

These events tend to occur predominantly after the first two infusions of RO6958688 in patients with liver lesions. AST and ALT elevations that occur after the third infusion (or later) are potentially a consequence of cytokine-release syndrome.

Please use guidelines in Appendix 10, Table 1 to manage AST/ALT elevation secondary to tumor inflammation and Appendix 10, Table 2 to manage AST/ALT elevation occurring as a consequence of CRS.

5.2.6.3 Management of Adverse Events Specific to Atezolizumab and to RO6958688

The attribution and management of certain adverse events that have been associated with each agent separately (e.g., pulmonary events of dyspnea and hypoxia, hepatic events, GI events) may be ambiguous when the agents are administered together. It is theoretically possible that allergic or inflammatory adverse events associated with RO6958688 symptoms could be exacerbated by the immunostimulatory activity of atezolizumab.

Toxicities should initially be managed according to the recommendations in Appendix 10 with dose holds applied to the component of the study treatment judged to be the primary cause. If an individual component for causality for the toxicity cannot be adequately determined, then the most conservative management recommendation should be applied.

5.2.6.3.1 Gastrointestinal Toxicities: Diarrhea and Colitis

Diarrhea that occurs during treatment with RO6958688 in combination with atezolizumab could be secondary to RO6958688-associated IRR and CRS, tumor inflammation, or an atezolizumab immune-mediated toxicity.

Colitis that occurs during treatment with RO6958688 in combination with atezolizumab could be secondary to RO6958688-associated tumor inflammation, or an atezolizumab immune-mediated toxicity.

Enteritis can also be a consequence of RO6958688-associated tumor inflammation.

Guidelines for management of patients who experience diarrhea and colitis are provided in Appendix 10.

5.2.6.3.2 Hepatotoxicity

Immune-mediated hepatitis has been associated with the administration of atezolizumab. Hepatic toxicity can also occur with RO6958688 either secondary to tumor inflammation or as a part of CRS. RO6958688-associated hepatic toxicity is usually transient compared with immune-mediated hepatitis that is associated with atezolizumab (which has a median duration of 2.1 months).

“Use of this document is governed by the terms of use on the first page of this document.”

Guidelines for management of patients who experience hepatotoxicity are provided in Appendix 10.

5.2.6.3.3 Pulmonary Toxicity

Immune-mediated pneumonitis has been associated with the administration of atezolizumab. Pulmonary toxicity (dyspnea, hypoxia or respiratory failure) can also occur with RO6958688 secondary to tumor inflammation or as a part of CRS.

Guidelines for management of patients who experience pulmonary toxicities are provided in Appendix 10.

5.2.6.3.4 Conjunctivitis

Conjunctivitis is not considered to be the risk that affects the benefit–risk profile of the combination or that requires active management given that events have been of mild severity and are manageable with current clinical practice.

5.3 METHODS AND TIMING FOR CAPTURING AND ASSESSING SAFETY PARAMETERS

The investigator is responsible for ensuring that all adverse events (see Section 5.1 for definition) are recorded on the Adverse Event eCRF and reported to the Sponsor in accordance with instructions provided in this section and in Sections 5.4-5.6.

For each adverse event recorded on the Adverse Event eCRF, the investigator will make an assessment of seriousness (see Section 5.1.2 for seriousness criteria), severity (see Section 5.3.3), and causality (see Section 5.3.4).

5.3.1 Adverse Event Reporting Period

Investigators will seek information on adverse events at each patient contact. All adverse events, whether reported by the patient or noted by study personnel, will be recorded in the patient's medical record. Adverse events will then be reported on the Adverse Event eCRF as follows:

After informed consent has been obtained but prior to initiation of study drugs, only serious adverse events caused by a protocol-mandated intervention should be reported (e.g., serious adverse events related to invasive procedures such as biopsies).
Any other adverse event should not be reported.

After initiation of study drugs, all adverse events, regardless of relationship to study drugs, will be reported until 28 days (for RO6958688) or 120 days (for atezolizumab) after the last dose of study drugs or until initiation of new systemic anti-cancer therapy, whichever occurs first.

After a period of 28 days or 120 days from the last dose, investigators should report any deaths, serious adverse events, or other adverse events of concern that are believed to be related to prior treatment with study drugs (see Section 5.6).

"Use of this document is governed by the terms of use on the first page of this document."

5.3.2 Eliciting Adverse Event Information

A consistent methodology of non-directive questioning should be adopted for eliciting adverse event information at all patient evaluation time-points. Examples of non-directive questions include the following:

"How have you felt since your last clinic visit?"

"Have you had any new or changed health problems since you were last here?"

5.3.3 Assessment of Severity of Adverse Events

The adverse event severity grading scale for the NCI CTCAE (v4.03) will be used for assessing adverse event severity (NCI CTCAE [v5] will be used for CRS). Table 4 will be used for assessing severity for adverse events that are not specifically listed in the NCI CTCAE.

Table 4 Adverse Event Severity Grading Scale

Grade	Severity
1	Mild; asymptomatic or mild symptoms; clinical or diagnostic observations only; or intervention not indicated.
2	Moderate; minimal, local, or non-invasive intervention indicated; or limiting age-appropriate instrumental activities of daily living [a].
3	Severe or medically significant, but not immediately life-threatening; hospitalization or prolongation of hospitalization indicated; disabling; or limiting self-care activities of daily living [b,c].
4	Life-threatening consequences or urgent intervention indicated [d].
5	Death related to adverse event [d].

NCI CTCAE = National Cancer Institute Common Terminology Criteria for Adverse Events.

Note: Based on the NCI CTCAE (v4.03) (except for CRS, which will be based on NCI CTCAE v5), which can be found at: https://evs.nci.nih.gov/ftp1/CTCAE/CTCAE_4.03/CTCAE_4.03_2010-06-14_QuickReference_8.5x11.pdf or https://ctep.cancer.gov/protocolDevelopment/electronic_applications/docs/CTCAE_v5_Quick_Reference_5x7.pdf

[a] Instrumental activities of daily living refer to preparing meals, shopping for groceries or clothes, using the telephone, managing money, etc.

[b] Examples of self-care activities of daily living include bathing, dressing and undressing, feeding one's self, using the toilet, and taking medications, as performed by patients who are not bedridden.

[c] If an event is assessed as a "significant medical event," it must be reported as a serious adverse event (see Section 5.4.2 for reporting instructions), per the definition of serious adverse event in Section 5.1.2.

[d] Grade 4 and 5 events must be reported as serious adverse events (see Section 5.4.2 for reporting instructions), per the definition of serious adverse event in Section 5.1.2.

CRS severity will be graded according to the CRS NCI CTCAE v5 for events that are considered related to RO6958688 only.

"Use of this document is governed by the terms of use on the first page of this document."

5.3.4 Assessment of Causality of Adverse Events

Investigators should use their knowledge of the patient, the circumstances surrounding the event, and an evaluation of any potential alternative causes to determine whether or not an adverse event is considered to be related to the study drugs, indicating "yes" or "no" accordingly. The following guidance should be taken into consideration:

- Temporal relationship of event onset to the initiation of study drugs
- Course of the event, considering especially the effects of dose reduction, discontinuation of study drugs, or reintroduction of study drugs (where applicable)
- Known association of the event with the study drugs or with similar treatments
- Known association of the event with the disease under study
- Presence of risk factors in the patient or use of concomitant medications known to increase the occurrence of the event
- Presence of non-treatment-related factors that are known to be associated with the occurrence of the event.

For patient receiving combination therapy, causality will be assessed individually for each protocol-mandated therapy.

5.3.5 Procedures for Recording Adverse Events

Investigators should use correct medical terminology/concepts when recording adverse events on the Adverse Event eCRF. Avoid colloquialisms and abbreviations.

Only one adverse event term should be recorded in the event field on the Adverse Event eCRF.

5.3.5.1 Infusion-Related Reactions/Hypersensitivity Reactions and Cytokine Release Syndrome Attributed to RO6958688

As IRRs and CRS may be indistinguishable from one another, the following procedure should be followed to record IRR/CRS events considered related to RO6958688:

- All infusion-related events occurring within 24 hours of the first two infusions of RO6958688 should be recorded as IRR unless hypotension and/or hypoxia is one of the predominant symptoms
- All infusion-related events occurring within 24 hours of RO6958688 infusion with hypotension as the predominant symptom, and infusion related events associated with 3rd infusion of RO6958688 or later should be recorded as CRS. Adverse events of CRS considered related to RO695688 will be graded according to CTCAE v5.)
- The one exception to this reporting guidance is if a clinical presentation suggests an immediate, acute hypersensitivity (e.g., generalized hives, mucosal edema, with or without wheezing and hypotension). Elevated IgE and/or tryptase levels support an allergic origin. In this case a diagnosis of “allergic reaction” or “hypersensitivity reaction” or “anaphylaxis” should be used.

“Use of this document is governed by the terms of use on the first page of this document.”

For adverse events with a diagnosis of IRR/CRS associated signs, symptoms, and laboratory abnormalities should be recorded on the dedicated Infusion-Related Reaction/Cytokine Release Syndrome eCRF. Each IRR/CRS should be recorded separately on the Adverse Event eCRF, with signs, symptoms, and laboratory abnormalities also recorded separately on the dedicated Infusion-Related Reaction/Cytokine Release Syndrome eCRF. Ambiguous terms such as "systemic reaction" should be avoided. In addition to documentation in the Adverse Event eCRF, non-serious Grade ≥ 2 CRS and IRR events should be reported as a non-serious adverse event of special interest (see Section 5.1.3).

Adverse events of IRR considered related to another study drug, and not RO6958688, will be graded using CTCAE v4.03.

5.3.5.2 Other Adverse Events

A diagnosis (if known) should be recorded on the Adverse Event eCRF rather than individual signs and symptoms (e.g., record only liver failure or hepatitis rather than jaundice, asterixis, and elevated transaminases). However, if a constellation of signs and/or symptoms cannot be medically characterized as a single diagnosis or syndrome at the time of reporting, each individual event should be recorded on the Adverse Event eCRF. If a diagnosis is subsequently established, all previously reported adverse events based on signs and symptoms should be nullified and replaced by one adverse event report based on the single diagnosis, with a starting date that corresponds to the starting date of the first symptom of the eventual diagnosis.

5.3.5.3 Adverse Events Occurring Secondary to Other Events

In general, adverse events occurring secondary to other events (e.g., cascade events or clinical sequelae) should be identified by their primary cause, with the exception of severe or serious secondary events. However, medically significant adverse events occurring secondary to an initiating event that are separated in time should be recorded as independent events on the Adverse Event eCRF. For example:

- If vomiting results in mild dehydration with no additional treatment in a healthy adult, only vomiting should be reported on the eCRF.
- If vomiting results in severe dehydration, both events should be reported separately on the eCRF.
- If a severe gastrointestinal hemorrhage leads to renal failure, both events should be reported separately on the eCRF.
- If dizziness leads to a fall and subsequent fracture, all three events should be reported separately on the eCRF.

All adverse events should be recorded separately on the Adverse Event eCRF if it is unclear as to whether the events are associated.

“Use of this document is governed by the terms of use on the first page of this document.”

5.3.5.4 Persistent or Recurrent Adverse Events

A persistent adverse event is one that extends continuously, without resolution, between patient evaluation time-points. Such events should only be recorded once on the Adverse Event eCRF. The initial severity of the event should be recorded, and the severity should be updated to reflect the most extreme severity any time the event worsens. If the event becomes serious, the Adverse Event eCRF should be updated to reflect this.

A recurrent adverse event is one that resolves between patient evaluation time-points and subsequently recurs. Each recurrence of an adverse event should be recorded separately on the Adverse Event eCRF.

5.3.5.5 Abnormal Laboratory Values

Not every laboratory abnormality qualifies as an adverse event. A laboratory test result should be reported as an adverse event if it meets any of the following criteria:

- Accompanied by clinical symptoms
- Results in a change in study treatment (e.g., dosage modification, treatment interruption, or treatment discontinuation)
- Results in a medical intervention (e.g., potassium supplementation for hypokalemia) or a change in concomitant therapy
- Clinically significant in the investigator’s judgment.

It is the investigator’s responsibility to review all laboratory findings. Medical and scientific judgment should be exercised in deciding whether an isolated laboratory abnormality should be classified as an adverse event.

If a clinically significant laboratory abnormality is a sign of a disease or syndrome (e.g., alkaline phosphatase and bilirubin 5 times the upper limit of normal (ULN) associated with cholecystitis), only the diagnosis (i.e., cholecystitis) should be recorded on the Adverse Event eCRF.

If a clinically significant laboratory abnormality is not a sign of a disease or syndrome, the abnormality itself should be recorded on the Adverse Event eCRF, along with a descriptor indicating if the test result is above or below the normal range (e.g., "elevated potassium", as opposed to "abnormal potassium"). If the laboratory abnormality can be characterized by a precise clinical term per standard definitions, the clinical term should be recorded as the adverse event. For example, an elevated serum potassium level of 7.0 mEq/L should be recorded as “hyperkalemia”.

Observations of the same clinically significant laboratory abnormality from visit to visit should not be repeatedly recorded on the Adverse Event eCRF, unless the etiology changes. The initial severity of the event should be recorded, and the severity or seriousness should be updated any time the event worsens.

“Use of this document is governed by the terms of use on the first page of this document.”

5.3.5.6 Abnormal Vital Sign Values

Not every vital sign abnormality qualifies as an adverse event. A vital sign result should be reported as an adverse event if it meets any of the following criteria:

- Accompanied by clinical symptoms
- Results in a change in study treatment (e.g., dosage modification, treatment interruption, or treatment discontinuation)
- Results in a medical intervention or a change in concomitant therapy
- Clinically significant in the investigator's judgment.

It is the investigator's responsibility to review all vital sign findings. Medical and scientific judgment should be exercised in deciding whether an isolated vital sign abnormality should be classified as an adverse event.

If a clinically significant vital sign abnormality is a sign of a disease or syndrome (e.g., high blood pressure), only the diagnosis (i.e., hypertension) should be recorded on the Adverse Event eCRF.

Observations of the same clinically significant vital sign abnormality from visit to visit should not be repeatedly recorded on the Adverse Event eCRF, unless the etiology changes. The initial severity of the event should be recorded, and the severity or seriousness should be updated any time the event worsens.

5.3.5.7 Abnormal Liver Function Tests

The finding of an elevated ALT or AST ($>3\times$ ULN) in combination with either an elevated total bilirubin ($>2\times$ ULN) or clinical jaundice in the absence of cholestasis or other causes of hyperbilirubinemia is considered to be an indicator of severe liver injury. Therefore, investigators must report as an adverse event the occurrence of either of the following:

- Treatment-emergent ALT or AST $> 3 \times$ ULN in combination with total bilirubin $> 2 \times$ ULN
- Treatment-emergent ALT or AST $> 3 \times$ ULN in combination with clinical jaundice.

The most appropriate diagnosis or (if a diagnosis cannot be established) the abnormal laboratory values should be recorded on the Adverse Event eCRF (see Section 5.3.5.1) and reported to the Sponsor immediately (i.e., no more than 24 hours after learning of the event), either as a serious adverse event or a non-serious adverse event of special interest (see Section 5.4.2).

5.3.5.8 Deaths

For this protocol, mortality is an efficacy endpoint. Deaths that occur during the protocol-specified adverse event reporting period (see Section 5.3.1) that are attributed by the investigator solely to progression of cancer should be recorded on the Death Attributed to Progressive Disease eCRF. All other on-study deaths, regardless of

"Use of this document is governed by the terms of use on the first page of this document."

relationship to study drugs, must be recorded on the Adverse Event eCRF and immediately reported to the Sponsor (see Section 5.4).

Death should be considered an outcome and not a distinct event. The event or condition that caused or contributed to the fatal outcome should be recorded as the single medical concept on the Adverse Event eCRF. Generally, only one such event should be reported. The term “sudden death” should only be used for the occurrence of an abrupt and unexpected death due to presumed cardiac causes in a patient with or without preexisting heart disease, within 1 hour of the onset of acute symptoms or, in the case of an unwitnessed death, within 24 hours after the patient was last seen alive and stable. If the cause of death is unknown and cannot be ascertained at the time of reporting, “unexplained death” should be recorded on the Adverse Event eCRF. If the cause of death later becomes available (e.g., after autopsy), “unexplained death” should be replaced by the established cause of death.

During post-study survival follow-up, deaths attributed to progression of cancer should be recorded only on the Survival eCRF.

5.3.5.9 Preexisting Medical Conditions

A preexisting medical condition is one that is present at the screening visit for this study. Such conditions should be recorded on the General Medical History and Baseline Conditions eCRF.

A preexisting medical condition should be recorded as an adverse event only if the frequency, severity, or character of the condition worsens during the study. When recording such events on the Adverse Event eCRF, it is important to convey the concept that the preexisting condition has changed by including applicable descriptors (e.g., “more frequent headaches”).

5.3.5.10 Lack of Efficacy or Worsening of Cancer

Events that are clearly consistent with the expected pattern of progression of the underlying disease should not be recorded as adverse events. These data will be captured as efficacy assessment data only. In most cases, the expected pattern of progression will be based on RECIST v1.1 criteria and modified RECIST criteria. In rare cases, the determination of clinical progression will be based on symptomatic deterioration. However, every effort should be made to document progression using objective criteria. If there is any uncertainty as to whether an event is due to disease progression, it should be reported as an adverse event.

5.3.5.11 Hospitalization or Prolonged Hospitalization

Any adverse event that results in hospitalization or prolonged hospitalization should be documented and reported as a serious adverse event (per the definition of serious adverse event in Section 5.1.2), except as outlined below.

“Use of this document is governed by the terms of use on the first page of this document.”

The following hospitalization scenarios are <u>not</u> considered to be serious adverse events:

- Hospitalization for respite care
- Planned hospitalization required by the protocol (e.g., for study drugs administration or insertion of access device for study drugs administration)
- Hospitalization for a preexisting condition, provided that all of the following criteria are met:
    - The hospitalization was planned prior to the study or was scheduled during the study when elective surgery became necessary because of the expected normal progression of the disease
    - The patient has not suffered an adverse event.
- Hospitalization due solely to progression of the underlying cancer.

The following hospitalization scenarios are not considered to be serious adverse events, but should be reported as adverse events instead:

- Hospitalization for an adverse event that would ordinarily have been treated in an outpatient setting had an outpatient clinic been available

5.3.5.12 Cases of Accidental Overdose or Medication Error

Accidental overdose and medication error (hereafter collectively referred to as "special situations"), are defined as follows:

- Accidental overdose: accidental administration of a drug in a quantity that is higher than the assigned dose
- Medication error: accidental deviation in the administration of a drug

    In some cases, a medication error may be intercepted prior to administration of the drug.

Special situations are not in themselves adverse events, but may result in adverse events. Each adverse event associated with a special situation should be recorded separately on the Adverse Event eCRF. If the associated adverse event fulfills seriousness criteria, the event should be reported to the Sponsor immediately (i.e., no more than 24 hours after learning of the event; see Section 5.4.2). For atezolizumab, RO6958688, or tocilizumab, adverse events associated with special situations should be recorded as described below for each situation:

- Accidental overdose: Enter the drug name and "accidental overdose" as the event term. Check the "Accidental overdose" and "Medication error" boxes.
- Medication error that does not qualify as an overdose: Enter the adverse event term. Check the "Medication error" box.
- Medication error that qualifies as an overdose: Enter the adverse event term. Check the "Accidental overdose" and "Medication error" boxes.

“Use of this document is governed by the terms of use on the first page of this document.”

In addition, all special situations associated with atezolizumab, RO6958688, or tocilizumab, regardless of whether they result in an adverse event, should be recorded on the Adverse Event eCRF as described below:

- Accidental overdose: Enter the adverse event term. Check the "Accidental overdose" and "Medication error" boxes.
- Medication error that does not qualify as an overdose: Enter the name of the drug administered and a description of the error (e.g., wrong dose administered, wrong dosing schedule, incorrect route of administration, wrong drug, expired drug administered) as the event term. Check the "Medication error" box.
- Medication error that qualifies as an overdose: Enter the drug name and "accidental overdose" as the event term. Check the "Accidental overdose" and "Medication error" boxes. Enter a description of the error in the additional case details.
- Intercepted medication error: Enter the drug name and "intercepted medication error" as the event term. Check the "Medication error" box. Enter a description of the error in the additional case details.

As an example, an accidental overdose that resulted in a headache would require the completion of two Adverse Event eCRF pages, one to report the accidental overdose and one to report the headache. The "Accidental overdose" and "Medication error" boxes need to be checked for both entries.

5.4 IMMEDIATE REPORTING REQUIREMENTS FROM INVESTIGATOR TO SPONSOR

Certain events require immediate reporting to allow the Sponsor to take appropriate measures to address potential new risks in a clinical trial. The investigator must report such events to the Sponsor immediately; under no circumstances should reporting take place more than 24 hours after the investigator learns of the event. The following is a list of events that the investigator must report to the Sponsor within 24 hours after learning of the event, regardless of relationship to study drug:

- Serious adverse events (defined in Section 5.1.2; see Section 5.4.2 for details on reporting requirements)
- Adverse events of special interest (defined in Section 5.1.3; see Section 5.4.2 for details on reporting requirements)
- Pregnancies (see Section 5.4.3 for details on reporting requirements)

5.4.1 Emergency Medical Contacts

To ensure the safety of study patients, access to the Medical Monitor is available 24 hours a day 7 days a week. Medical Monitor contact details are listed in the “Protocol Administrative and Contact Information & List of Investigators”.

“Use of this document is governed by the terms of use on the first page of this document.”

5.4.2 Reporting Requirements for Serious Adverse Events and Non-Serious Adverse Events of Special Interest

For reports of serious adverse events and non-serious adverse events of special interest (see Sections 5.1.2 and 5.1.3), investigators should record all case details that can be gathered on the Serious Adverse Reporting Form and forward this form to the Serious Adverse Event Responsible within 24 hours.

5.4.3 Reporting Requirements for Pregnancies

5.4.3.1 Pregnancies in Female Patients

Female patients of childbearing potential will be instructed to immediately inform the investigator if they become pregnant during the study or within 5 months after the last dose of RO6958688 and atezolizumab or within 2 months after the last dose of tocilizumab A Clinical Trial Pregnancy Reporting Form should be completed by the investigator and submitted to the sponsor within 24 hours after learning of the pregnancy. Pregnancy should not be recorded on the Adverse Event eCRF. The investigator should discontinue study drugs and counsel the patient, discussing the risks of the pregnancy and the possible effects on the fetus. Monitoring of the patient should continue until conclusion of the pregnancy. Any serious adverse events associated with the pregnancy (e.g., an event in the fetus, an event in the mother during or after the pregnancy, or a congenital anomaly/birth defect in the child) should be reported on the Adverse Event eCRF.

5.4.3.2 Pregnancies in Female Partners of Male Patient

Male patients will be instructed through the Informed Consent Form to immediately inform the investigator if their partner becomes pregnant during the study or within 3 months after the last dose of RO6958688 if the patient received RO6958688 only and within 2 months after the last dose of tocilizumab (if applicable). Men should not donate sperm for 3 months after the last dose of RO6958688 and for 2 months after the last dose of tocilizumab (if applicable). A Clinical Trial Pregnancy Reporting Form should be completed by the investigator and submitted to the sponsor within 24 hours after learning of the pregnancy. Attempts should be made to collect and report details of the course and outcome of any pregnancy in the partner of a male patient exposed to study drugs. The pregnant partner will need to sign an Authorization for Use and Disclosure of Pregnancy Health Information to allow for follow-up on her pregnancy. Once the authorization has been signed, the investigator will update the Clinical Trial Pregnancy Reporting Form with additional information on the course and outcome of the pregnancy. An investigator who is contacted by the male patient or his pregnant partner may provide information on the risks of the pregnancy and the possible effects on the fetus, to support an informed decision in cooperation with the treating physician and/or obstetrician.

5.4.3.3 Abortions

A spontaneous abortion should be classified as a serious adverse event (as the Sponsor considers spontaneous abortions to be medically significant events), recorded on the

"Use of this document is governed by the terms of use on the first page of this document."

Adverse Event eCRF, and reported to the Sponsor immediately (i.e., no more than 24 hours after learning of the event; see Section 5.4.2).

If a therapeutic or elective abortion was performed because of an underlying maternal or embryofetal toxicity, the toxicity should be classified as a serious adverse event, recorded on the Adverse Event eCRF, and reported to the Sponsor immediately (i.e., no more than 24 hours after learning of the event; see Section 5.4.2). A therapeutic or elective abortion performed for reasons other than an underlying maternal or embryofetal toxicity is not considered an adverse event. All abortions should be reported as pregnancy outcomes on the paper Clinical Trial Pregnancy Reporting Form.

5.4.3.4 Congenital Anomalies/Birth Defects

Any congenital anomaly/birth defect in a child born to a female patient or female partner of a male patient exposed to study drugs should be classified as a serious adverse event, recorded on the Adverse Event eCRF, and reported to the Sponsor immediately (i.e., no more than 24 hours after learning of the event; see Section 5.4.2).

5.5 FOLLOW-UP OF PATIENTS AFTER ADVERSE EVENTS

5.5.1 Investigator Follow-Up

The investigator should follow each adverse event until the event has resolved to baseline grade or better, the event is assessed as stable by the investigator, the patient is lost to follow-up, or the patient withdraws consent. Every effort should be made to follow all serious adverse events considered to be related to study drugs or trial-related procedures until a final outcome can be reported.

During the study period, resolution of adverse events (with dates) should be documented on the Adverse Event eCRF and in the patient's medical record to facilitate source data verification. If, after follow-up, return to baseline status or stabilization cannot be established, an explanation should be recorded on the Adverse Event eCRF.

All pregnancies reported during the study should be followed until pregnancy outcome and reported according to the instructions provided in Section 5.4.3.

The investigator must report new significant follow-up information for these events to the Sponsor immediately (i.e., no more than 24 hours after becoming aware of the information). New significant information includes the following:

- New signs or symptoms or a change in the diagnosis
- Significant new diagnostic test results
- Change in causality based on new information
- Change in the event's outcome, including recovery
- Additional narrative information on the clinical course of the event

"Use of this document is governed by the terms of use on the first page of this document."

Investigators must also comply with local requirements for reporting serious adverse events to the local health authority and IRB/EC.

5.5.2 Sponsor Follow-Up

For serious adverse events, non-serious adverse events of special interest, and pregnancies, the Sponsor or a designee may follow up by telephone, fax, electronic mail, and/or a monitoring visit to obtain additional case details and outcome information (e.g., from hospital discharge summaries, consultant reports, autopsy reports) in order to perform an independent medical assessment of the reported case.

5.6 POST-STUDY ADVERSE EVENTS

At the 28-day safety and follow-up visit, the investigator should instruct each patient to report to the investigator any subsequent AEs that the patient's personal physician believes could be related to prior study drug treatments or study procedures.

The investigator is not required to actively monitor patients for adverse events after the end of the adverse event reporting period (defined as 28 days or 120 days after the last dose of study drugs as applicable). However, the Sponsor should be notified if the investigator becomes aware of any death or any other serious adverse event occurring after the end of the adverse event reporting period, if the event is believed to be related to prior study drug treatments either by faxing or by scanning and emailing the Serious Adverse Event Reporting Form using the fax number or email address provided to investigators. The Sponsor should also be notified if the investigator becomes aware of the development of cancer or a congenital anomaly/birth defect in a subsequently conceived offspring of a patient that participated in this study.

During post-study survival follow-up, deaths attributed to progression of cancer should be recorded only on the Survival eCRF.

5.7 EXPEDITED REPORTING TO HEALTH AUTHORITIES, INVESTIGATORS, INSTITUTIONAL REVIEW BOARDS, AND ETHICS COMMITTEES

The Sponsor will promptly evaluate all serious adverse events and non-serious adverse events of special interest against cumulative product experience to identify and expeditiously communicate possible new safety findings to investigators, IRBs, ECs, and applicable health authorities based on applicable legislation.

To determine reporting requirements for single adverse event cases, the Sponsor will assess the expectedness of these events using the following reference documents:

- RO6958688 and Atezolizumab Investigator's Brochures
- Tocilizumab Investigator's Brochure

"Use of this document is governed by the terms of use on the first page of this document."

The Sponsor will compare the severity of each event and the cumulative event frequency reported for the study with the severity and frequency reported in the applicable reference document.

Reporting requirements will also be based on the investigator's assessment of causality and seriousness, with allowance for upgrading by the Sponsor as needed.

6. STATISTICAL CONSIDERATIONS AND ANALYSIS PLAN

The data will be analyzed by the Sponsor and/or designated CRO. Any data analysis carried out independently by the investigator should be submitted to the Sponsor before publication or presentation. The data will be summarized with respect to demographic and baseline characteristics, efficacy observations and measurements, safety observations and measurements, PK and biomarker measurements. Patients will be followed up until disease progression (according to RECIST 1.1 or to modified RECIST criteria, whichever occurs later) or until withdrawal from the study. All data up until this point will be listed by patient and summarized by regimen and dose, if appropriate.

6.1 PRIMARY STUDY VARIABLES

The primary study variables are as follows:

- Administration of study drugs data, including start and end of infusions and dose
- Adverse events data, including dates of onset and resolution, grade according to the NCI CTCAE v4.03 and whether the adverse event qualifies as dose-limiting toxicity (DLT). An exception to this is made for CRS adverse events considered related to RO6958688 which will be evaluated using NCI CTCAE v5.

6.2 SECONDARY STUDY VARIABLES

The secondary study variables are as follows:

- The number and quality (e.g., proliferation and activation markers) of immune cells in the tumor and peripheral blood
- The pharmacokinetics (PK) data, i.e., plasma concentrations of RO6958688 and atezolizumab when administered in combination and ADA titers against RO6958688
- Anti-tumor activity data, including CT tumor assessment data and derived per-patient summaries best overall response according to RECIST v1.1 and modified RECIST criteria, by investigator assessment for the whole study and by central assessment.
- Progression-free survival (PFS) data, by investigator assessment for the whole study and by central assessment
- Preliminary overall survival (OS) data

“Use of this document is governed by the terms of use on the first page of this document.”

6.3 DETERMINATION OF SAMPLE SIZE

6.3.1 Dose-Escalation

Approximately 30 patients will be enrolled in the dose-escalation Part IA to define the MTD, based on Table 5, see column n (number of patients needed to complete dose-escalation). The exact number of patients will depend on the number of dose-levels required to determine the MTD and/or the recommended dose and on the adaptive nature of the design. The estimated chance to require more than 45 patients for the dose escalation patients in the scenarios is usually less than 10% (see Table 1 in Appendix 6 for the scenario settings). Table 5 provides the operating characteristics across different assumed toxicity scenarios. These operating characteristics have been calculated with a Roche-internal R-package, using R version 3.1.2. Table 5 shows the following metrics, with their frequency distribution under the assumed scenarios summarized by means and 10 and 90–percent quantiles from 400 simulation runs (see Appendix 6 for more details):

- Dose: This is the final dose (mg) determined as the MTD by the mCRM design.
- Tox. at dose: This is the resulting true probability (%) of a DLT at the determined MTD.
- Prop. of DLTs: This is the proportion of patients (%) with a DLT.
- n: This is the total number of patients in the dose escalation.
- $n_{overdose}$: This is the number of overdosed patients, who have been exposed to doses with DLT probability above the target of 30%.

The operating characteristics indicate that, across a wide range of scenarios, the design performs reasonably well, both with steep as well as gradually increasing dose-toxicity curves. In particular, the resulting MTD estimates are on average in the target toxicity range of 20–30%, even for the very high toxicity scenario 3. An exception is clearly the very low toxicity scenario 7, where even at the highest considered dose of 1000 mg the DLT probability is only 1%. The reasonable behavior of the dose escalation design is also reflected in the number of overdosed patients (i.e., those treated at doses above the target interval), which is on average not exceeding 9 patients, except for the very high toxicity scenario 3 where on average 18 patients are overdosed.

This design has previously been used successfully in the literature (Neuenschwander et al. 2008, Bailey et al. 2009) as well as in Roche, with only slight modifications from the current set-up.

“Use of this document is governed by the terms of use on the first page of this document.”

Table 5 Operating Characteristics of the mCRM with EWOC Design with Respect to the Chosen Scenarios

No.	Dose	Tox. at dose	Prop. of DLTs	n	$n_{overdose}$
1	230 (145, 340)	29 % (14 %, 47 %)	16 % (12 %, 19 %)	38 (32, 45)	8 (2, 15)
2	3.3 (0.4, 5)	24 % (17 %, 29 %)	25 % (19 %, 31 %)	28 (21, 37)	3 (0, 11)
3	0.1 (0, 0.2)	17 % (0 %, 42 %)	52 % (32 %, 80 %)	18 (5, 32)	18 (5, 32)
4	81 (45, 125)	27 % (14 %, 43 %)	18 % (15 %, 21 %)	34 (27, 42)	7 (1, 15)
5	66 (45, 86)	30 % (5 %, 56 %)	18 % (15 %, 21 %)	31 (26, 36)	8 (4, 12)
6	897 (640, 1000)	30 % (18 %, 35 %)	8 % (3 %, 13 %)	41 (36, 46)	6 (2, 12)
7	1000 (1000, 1000)	1 % (1 %, 1 %)	1 % (0 %, 3 %)	38 (36, 44)	0 (0, 0)
8	1 (0.6, 1.5)	25 % (13 %, 38 %)	24 % (20 %, 29 %)	29 (22, 36)	8 (1, 17)
9	465 (305, 671)	30 % (16 %, 48 %)	14 % (11 %, 17 %)	42 (36, 48)	9 (2, 14)

DLT = dose limiting toxicity; Prop. = proportion; Tox. = toxicity
note: Means (10% and 90% quantiles) from 400 simulations per scenario are shown.

6.3.2 Dose/Schedule-Finding

The sample size of 20 evaluable patients per dose scheme in the schedule comparison expansion (cohort A) in Part IB allows for a reasonably precise differentiation between the QW and Q3W dose schemes. Specifically, an observed difference of ca. 10% points in ORR would lead to approximately 80% posterior probability of a true response rate difference between two dose schemes. For example, if the number of objective responses is 4 in one and 2 in the other dose scheme out of 20 patients each, then the posterior probability of a positive response rate difference is 79.5%.

For cohort C, the randomization of approximately 40 patients to each of the arms C1, C2 and the possibly 40 patients enrolled to a later optional arm C3 allows for a reasonably precise differentiation between the arms. Specifically, if one of the arms has 20% true ORR, and the other two arms have only 10% true ORR, then n=40 patients per arm gives 83% power to decide for the correct arm as the best one. In another scenario, where the first arm has 10% true ORR, the second arm 20% true ORR, and the third arm 30% true ORR, then n=40 patients per arm gives 85% power to pick the correct third arm as the best one. Hence, the sample size of 40 patients per arm in cohort C is justified. Note: Cohort C1 enrolled 39 patients, and Cohort C2 enrolled 35 patients; no further patients will be enrolled into these cohorts. Optional cohort C3 has not and will not enroll any patients.

For cohort G1, the inclusion of 40 patients will allow a reasonably precise estimation of the incidence of ADA. Specifically, if none of the 40 patients develops ADA by week 8, then the 95% confidence interval for the ADA incidence will be 0% to 9%, i.e., exclude values of 10% or higher. In addition to the assessment of immunogenicity reduction by obinutuzumab, cohort G1, as well as optionally cohorts G2, G3, will allow for a descriptive assessment of the primary safety and efficacy endpoints in the MSS CRC and optionally in the mGC, Pancreatic indications, respectively. Specifically, observing

“Use of this document is governed by the terms of use on the first page of this document.”

an ORR of 20% in a cohort would result in a 95% confidence interval from 9% to 36%, i.e., exclude values of 9% or lower. Note: Cohorts G1, G2 and G3 have not and will not enroll any patients.

The sample size of approximately 10-20 patients in each of the cohorts B (MSS and MSI-H CRC separately) and the safety cohorts in other indications allows for an initial assessment of safety and tolerability. This is shown in detail with simulations of the intra-patient dose escalation design in cohort B in Appendix 7. Note: Cohort B1 enrolled 17 patients; no further patients will be enrolled. Cohort B2 has not and will not enroll any patients.

6.4 SUMMARIES OF CONDUCT OF STUDY

All protocol deviations will be listed. The study is open-label therefore no blinded treatment will be administered.

6.5 ANALYSIS POPULATIONS

6.5.1 Safety Analysis Population

All patients enrolled in the study who received at least one dose of RO6958688 or atezolizumab will be included in the safety population.

For the purpose of establishing the maximum-tolerated dose, the dose-determining population, which is used to recommend the next dose level based on the mCRM with EWOC design, consists of all patients in Part IA from the safety population who did not receive RO6958688 before Cycle 1 Day 1, received the assigned doses of RO6958688 (three doses) and atezolizumab (one dose) within the DLT period and have undergone the scheduled safety evaluations, or experienced a DLT after administration of a study drug within the DLT period.

Patients who have been treated before Cycle 1 Day 1 with RO6958688 will be analyzed separately.

6.5.2 Pharmacokinetic Analysis Population

All patients who received at least one dose of RO6958688 or atezolizumab will be included in the PK analysis population. Patients will be excluded from the PK analysis population if they significantly violate the inclusion or exclusion criteria, deviate significantly from the protocol, or if data are unavailable or incomplete, which may influence the PK analysis. Excluded cases will be documented together with the reason for exclusion. All decisions on exclusions from the analysis will be made prior to database closure.

6.5.3 Pharmacodynamic Analysis Population

The PD analysis population will be a subset of the safety population, based on the availability of evaluable blood or tumor samples.

"Use of this document is governed by the terms of use on the first page of this document."

6.5.4 Efficacy Analysis Population

Two efficacy analyses will be performed. The primary efficacy analysis population will consist of all patients who receive at least one dose of any study treatment. If different, an additional ITT population will consist of all patients who are not screen failures and were included in the trial (ICF signature), and will be used for sensitivity analyses.

Patients who have been treated before Cycle 1 Day 1 with RO6958688 will be analyzed separately.

6.6 SUMMARIES OF TREATMENT GROUP COMPARABILITY

Comparability across dose-levels will be evaluated descriptively via tabulation by demographic characteristics (including age, sex, patient disposition, and previous therapies). Treatment administration will be reported on the basis of number of cycles and dose intensity. Baseline status of key pharmacodynamics endpoints will also be tabulated to determine comparability. The analysis population will be the safety population.

6.7 SAFETY ANALYSES

Safety analyses will be performed for all the patients in the safety analysis population. All clinically abnormal safety parameters will be listed by patient within regimen and summarized in tables, as appropriate. Safety will be determined, but not limited to, by AEs, laboratory tests, vital signs, electrocardiogram, physical examinations, and performance status. Exposure to study medication will be summarized by total duration of study medication, number of cycles started, cumulative dose, and dose-intensity using descriptive statistics. Dose modifications, interruptions, and their reasons will be presented.

6.7.1 Dose-Escalation Approach

6.7.1.1 Modified Continual Reassessment Method with Overdose Control in Part IA

Overview

The dose escalation in Part IA will employ a modified-Continual Reassessment Method with Overdose Control (mCRM with EWOC) design, in order to estimate the MTD of RO6958688 given QW in combination with 1200 mg atezolizumab given Q3W. The design is based on the binary variable “occurrence of a DLT”. The MTD is defined as the dose that maximizes the probability of a DLT being in the targeted toxicity interval of 20–30%, subject to the probability of DLT being in the overdosing interval of 30–100% being less than 25%. Patients within a cohort will be enrolled in a sequential manner in cohorts of at least 3 patients each, which, if required, can be expanded with additional patients. Each patient will be observed for 21 days for DLT assessment. The first patient in each cohort will be observed for safety for one week (2 weeks in Spain) before enrolling additional patients in the cohort. After the last patient in each cohort has completed the 21 day DLT observation period, the Sponsor and investigators will evaluate the next dose recommended by the mCRM design and agree on the dose and

“Use of this document is governed by the terms of use on the first page of this document.”

schedule for the subsequent cohort. If a DLT is already reported in the first patient during the first two weeks of safety observation, the Sponsor will organize a teleconference with the investigators to discuss the safety and tolerability of RO6958688 and atezolizumab and to decide whether the subsequent patients in the same cohort will be enrolled at the same dose. At each dose-escalation step, the dose can be escalated, de-escalated, or an additional cohort at the same dose-level could be enrolled.

There will be no dose-escalation in the Q3W schedule, however, DLTs observed in the Q3W patients would still be considered in the EWOC model. DLT-free Q3W patients would not be considered in the EWOC model.

Next dose recommendation

The starting dose for RO6958688 will be 5 mg and the fixed dose for atezolizumab will be 1200 mg. The dose-escalation is for the dose of RO6958688. The maximum allowable increment for RO6958688 between dose-levels will be 100% from the current dose level throughout the study. The dose of RO6958688 will not be escalated above the monotherapy MTD if defined in the BP29541 study. The mCRM with EWOC design will identify the next recommended dose-level subject to the following criteria:

- Overdose threshold: The posterior probability of being within the overdosing interval (above 30% DLT probability) must be below 25%.
- MTD target: The posterior probability of being within the target toxicity interval (20–30% DLT probability) is maximized.

Clinical judgment can always override mCRM recommendations in the dose-selection process, but cannot select doses exceeding the 100% maximum increment or the above described overdose threshold (i.e., the probability of overdosing must always be below 25%). In addition to modeling the probability of toxicity with the mCRM model (see below for the details), models relating PK and PD data will be explored in parallel to the mCRM model and may inform the dose escalation as part of the available exploratory data (provided that appropriate PK and PD data for all patients enrolled up to that timepoint are available).

Stopping rules

The dose-escalation will stop under any one of the following circumstances:

- At least 50 patients are DLT-evaluable with the same schedule/sequence/timing of the two study drugs.
- At least 15 patients are DLT-evaluable with the same schedule/sequence/timing of the two study drugs, of which at least 6 patients have been accrued near the estimated MTD dose (where near means differing from the estimated MTD by at most 20%), and in addition the probability that the estimated MTD dose lies within the target toxicity interval is above 40%.

Note that further patients might still be added to cohorts in order to obtain more safety, efficacy or PD data. The tentative MTD estimate of Part I is then defined as the lastly

“Use of this document is governed by the terms of use on the first page of this document.”

estimated MTD (mCRM with EWOC next recommended dose level). The DLT occurrence data from the Part II of the study will be used to update the statistical model used in the dose-escalation phase. This will lead to a final MTD estimate.

Model specification

The model is a mixture of two logistic regression models: The probability of a DLT at dose d_j is

$$p(d_j) = \pi\, p_1(d_j) + (1 - \pi) p_2(d_j)$$

where π is the probability for the combination model $p(d_j)$ being the same as the RO6958688 monotherapy model $p_1(d_j)$, and $1 - \pi$ is the probability of a separate model $p_2(d_j)$, where for $k = 1{,}2$:

$$p_k(d_j) = \frac{\exp\left(\alpha_k + \beta_k\, log\left(\frac{d_j}{d^*}\right)\right)}{1 + \exp\left(\alpha_k + \beta_k\, log\left(\frac{d_j}{d^*}\right)\right)}$$

and $d^* = 500$ is the reference dose. The monotherapy component $p_1(d_j)$ will always be informed by the latest available DLT data from the BP29541 study, by the corresponding likelihood contribution to the parameters α_1 and β_1.

This mixture model allows for dynamic borrowing from the BP29541 study (see e.g., Schmidli et al., 2004). Starting from a prior probability of $\pi = 10\%$, π is updated throughout the trial by the combination DLT data from this study and the monotherapy DLT data from the BP29541 study, in order to obtain the posterior probability π. If the combination DLT data is consistent with the monotherapy DLT data, then the posterior probability will increase above 10%. On the other hand, if the combination DLT data is not consistent with the monotherapy DLT data (e.g., if there are more DLTs at lower doses), then the posterior probability will decrease appropriately.

Minimally informative prior distributions (see Neuenschwander et al., 2008) are used for the parameters α_k and β_k $(k = 1{,}2)$, such that the prior 95% quantiles of $p_k(0.05)$ and $p_k(1000)$ are (0.3%, 24.7%) and (18.4%, 98.4%), giving a quite low probability on high toxicity at 0.05 mg and on low toxicity at 1000 mg, respectively. Specifically, the prior distributions are bivariate normal distributions on α_k and $\log(\beta_k)$.

$$\begin{pmatrix} \alpha_k \\ \log(\beta_k) \end{pmatrix} \sim \mathcal{N}\left(\begin{pmatrix} 0.996 \\ -0.729 \end{pmatrix}, \begin{pmatrix} 2.023 & 0.068 \\ 0.068 & 0.003 \end{pmatrix}\right)$$

for $k = 1{,}2$.

The dose grid for the design is 0.052, 0.1, 0.15, 0.2, 0.3, 0.45, 0.6, 0.8, 1, 1.3, 1.5, 2, 2.5, 5, 10, …, 995, 1000 mg. This grid includes also the low doses of the Part I of the

“Use of this document is governed by the terms of use on the first page of this document.”

BP29541 study, and also ensures that all doses are within 100% of each other, allowing potential escalation from all doses.

Design characteristics

For all characterizations of the mCRM with EWOC design, including the trial simulations in Section 6.3, we use the available monotherapy DLT data from BP29541 as of 17th August 2015.

The recommended starting dose from the prior model would be 2 mg, while 5 mg is the dose maximizing the probability of reaching a dose with 20 to 30% DLT rate. These quite low starting recommendations from the mCRM show that a conservative prior model has been specified, ensuring the safety of the patients.

If the first patient at the starting dose 5 mg in the trial had a DLT, and this cohort was closed subsequently, then the next recommended dose would be 0.3 mg. The strong reduction of the dose in this situation is another sign of the conservative nature of the prior model.

More generally, Table 6 shows hypothetical recommendations of the mCRM with EWOC design, assuming 4 patients per cohort and no DLTs having been observed until the respective dose. For example, it is shown that the design would recommend 15 mg, if no DLTs had been observed in the 4 patients at 5 mg and one DLT had been observed in the subsequent 4 patients at 10 mg, corresponding to only 50% increment compared to the maximum allowed increment of 100%. The table shows a reasonable behavior of the design, with higher dose reductions in the beginning of the escalation when only few patient data is yet available. Note that the final next dose will always be subject to clinical judgment and is always allowed to be lower than the doses listed in this table (see subsection “Next dose recommendation” above).

“Use of this document is governed by the terms of use on the first page of this document.”

Table 6 Hypothetical Recommendations of the mCRM with EWOC Design

dose [mg]	DLTs	next dose [mg]	increment [%]
5	0	10.000	100
5	1	2.500	-50
5	2	0.800	-84
5	3	0.200	-96
5	4	0.052	-99
10	0	20	100
10	1	15	50
10	2	5	-50
10	3	2	-80
10	4	0.8	-92
20	0	40	100
20	1	40	100
20	2	15	-25
20	3	5	-75
20	4	2.5	-88
40	0	80	100
40	1	80	100
40	2	45	12
40	3	20	-50
40	4	10	-75
80	0	160	100
80	1	160	100
80	2	115	44
80	3	55	-31
80	4	30	-62
160	0	320	100
160	1	320	100
160	2	245	53
160	3	125	-22
160	4	70	-56
320	0	640	100
320	1	640	100
320	2	505	58
320	3	255	-20
320	4	150	-53
640	0	1000	56
640	1	1000	56
640	2	1000	56
640	3	540	-16
640	4	320	-50

6.7.1.2 Intra-Patient Dose Escalation Design

Cohort B1 in Part IB of the study will employ an intra-patient dose escalation design for the step up dosing regimen, in order to define the late cycle MTD of RO6958688 in combination with 1200 mg Q3W atezolizumab. In B1 the RO6958688 dose at C1D1 will

"Use of this document is governed by the terms of use on the first page of this document."

be 40 mg followed by 150 mg at C1D8, 300 mg at C1D15, 600 mg at C2D1, 900 mg at C2D8 and 1200 mg at C2D15 and C3D1. Once the 1200 mg RO6958688 dose level is reached, RO6958688 will be administered every 3 weeks (1200 mg Q3W).

The dose of RO6958688 will not be escalated above the monotherapy late cycle MTD of RO6958688 if defined in the BP29541 protocol.

The late cycle MTD will initially be estimated separately in the MSS and MSI-H patients, since potentially the safety profile could be different. However, the Sponsor may in the end define only one late cycle MTD if appropriate. A patient will be considered evaluable for the purpose of estimating the late cycle MTD, if the patient was dosed up to reaching a DLT or the maximum possible dose at the time of treatment (i.e., 1200 mg or according to the safety rules below).

The DLT window is defined as 1 week, to allow dose escalation after 1 week, and a patient is defined as DLT-evaluable if the patient received the full planned dose of RO6958688. The following rules apply for the intra-patient dose escalation, separately for MSS and MSI-H patients:

1. The time interval between dosing of the first and second as well as the second and third patient within each cohort is at least 1 week, to allow for sufficient observation time. The following patients may be dosed one week after the third patient has been dosed.

2. The dose is increased every week within each patient until any of the below applies:

    a. A DLT is observed in this patient. The dose is then reduced to the next lower, and previously tolerated dose level, afterwards.

    b. Less than 6 patients have been treated and ≥ 2 DLTs have been observed at the next higher dose level or below. The dose is then kept constant.

    c. At least 6 patients have been treated and >33% of patients have had DLT at the next higher dose level or below. The dose is then kept constant.

The late cycle MTD is defined as follows:

1. A minimum of 6 patients must have been treated at this dose level or above.

2. If 6 patients are evaluable for DLT, the late cycle MTD is defined as the highest dose with ≤ 1 DLT having been observed

3. If more than 6 patients are evaluable for DLT, the late cycle MTD is defined as the highest dose where < 33% of patients have had DLT.

"Use of this document is governed by the terms of use on the first page of this document."

The acceptable characteristics of this dose escalation design are illustrated with simulations in Appendix 7.

6.7.2 Adverse Events

The original terms recorded on the eCRF by the investigator for adverse events will be standardized by the sponsor.

Adverse event data will be reported in listings and presented in frequency tables by MedDRA terms. Adverse events will be summarized by mapped term and appropriate thesaurus level. The severity of adverse events will be graded according to the NCI CTCAE v4.03 (or for CRS, NCI CTCAE v5). Summaries of adverse event by grade, seriousness, and relationship to study treatment will be presented, as well as summaries of adverse events leading to death, and premature withdrawal from study treatment.

6.7.3 Clinical Laboratory Test Results

All clinical laboratory data will be stored on the database in the units in which they were reported. Patient listings and summary statistics at each assessment time will be presented using the International System of Units (SI units; Système International d'Unités) where required. Laboratory data not reported in SI units will be converted to SI units before processing.

Clinically abnormal laboratory test values will be presented by individual listings with flagging of values outside the normal ranges where required. Summary tables of change from baseline over time based on SI units will be displayed as needed. Shifts in NCI CTCAE v4.03 from baseline to the worst grade observed during treatment will be presented for selected laboratory parameters.

6.7.3.1 Standard Reference Ranges and Transformation of Data

Where appropriate, Roche standard reference ranges, rather than the reference ranges of the investigator, may be used. For most parameters, the measured laboratory test result can be assessed directly using the Roche standard reference range. Certain laboratory parameters need to be transformed to Roche's standard measurement units first.

A transformation may be performed on certain laboratory tests that lack sufficiently common procedures and have a wide range of investigator ranges, e.g., enzyme tests that include AST, ALT, and alkaline phosphatase and total bilirubin. If the standard reference ranges for parameters have a lower limit of zero, only the upper limits of the ranges need to be used in transforming the data.

6.7.3.2 Definition of Laboratory Abnormalities

Laboratory values falling outside the standard reference range will be labeled "H" for high or "L" for low in patient listings of laboratory data.

"Use of this document is governed by the terms of use on the first page of this document."

In addition to the standard reference range, a marked reference range has been predefined by Roche for some laboratory parameters. The marked reference range is broader than the standard reference range. Values falling outside the marked reference range that also represent a defined change from baseline are considered marked laboratory abnormalities (i.e., potentially clinically relevant). If a baseline value is not available for a patient, the midpoint of the standard reference range will be used as the patient's baseline value for the purposes of determining marked laboratory abnormalities. Marked laboratory abnormalities will be labeled in the patient listings as "HH" for very high or "LL" for very low.

6.7.4 Vital Signs

Clinically abnormal vital signs data may be presented by individual listings with flagging of values outside the normal ranges/marked abnormalities as appropriate. In addition, tabular summaries will be used, as appropriate.

6.7.5 ECG Data Analysis

Clinically abnormal ECG data may be presented by individual listings with flagging of values outside the normal ranges/marked abnormalities as appropriate. In addition, the 12-lead triplicates will be averaged out and tabulated by dose level/regimen.

6.7.6 Concomitant Medications

The original terms recorded on the Patients' eCRF by the investigator for concomitant medications will be standardized by the sponsor by assigning preferred terms.

Concomitant medications will be presented in summary tables and listings, as appropriate.

6.8 EFFICACY ANALYSES

Tumor response data will be reported using descriptive statistics. ORR, SDR, and DCR and best overall response will be summarized using relative frequencies and 95% confidence limits. Duration of response (DOR) and progression free survival (PFS) on treatment will be summarized using time to event analyses and Kaplan Meier curves. Duration of response will only be analyzed in those cohorts with a sufficient number of responders. Preliminary overall survival (OS) data may be tabulated and summarized using time to event analyses and Kaplan Meier curves. Summaries will be carried out by cohort, dose, and overall. Statistical models may be used to summarize the efficacy data obtained across different dose levels and schedules, in order to contribute to the final selection of a recommended dose and schedule. This will be carried out for all patients in the respective efficacy analysis populations, for both RECIST and modified RECIST criteria efficacy endpoints, for investigator assessed data for the whole study and for centrally assessed data.

"Use of this document is governed by the terms of use on the first page of this document."

6.8.1 Primary Efficacy Endpoint

The primary efficacy endpoints, for the evaluation of the preliminary anti-tumor activity of RO6958688 in combination with atezolizumab, will be ORR, SDR and DCR according to RECIST v1.1.

The analysis of tumor response is based on the best overall response (BOR). BOR is defined as the best response recorded from the start of treatment until disease progression/recurrence or death whichever occurs first. Since tumor assessments take place every 8 weeks, 60 days is chosen so as to cover these assessments sufficiently.

Confirmation of partial and complete responses will be done at the next scheduled visit after at least 28 days from the initial response. A patient is assigned a best overall response SD if they have a response assessment of SD, PR, or CR at one or more visits at least 42 days (6 weeks) after start of study treatment, but are not a confirmed CR or PR.

For the ORR analyses in the primary and ITT efficacy analysis populations, patients who withdraw study treatment because of any reason, die or clinically progress before the first tumor assessment on treatment will be assigned a best overall response of Non Evaluable. These patients will be included in the denominator of the corresponding ORR estimate.

6.8.2 Secondary Efficacy Endpoints

The time-to-event endpoints duration of response (DOR), progression-free survival (PFS) and preliminary overall survival (OS) are the secondary efficacy endpoints in this study.

PFS per RECIST v1.1 is defined as time between enrollment or randomization date (whichever is applicable) and date of first documented disease progression per RECIST v1.1 or death from any cause, whichever occurs first. Patients who neither progressed nor died in this interval, or who are lost to follow-up are censored at the date of last tumor assessment within this time window or last follow-up for progression of disease. Patients for whom no post-baseline tumor assessments are available are censored at first study treatment.

6.9 PHARMACODYNAMIC ANALYSES

PD parameters will be listed by patient and tabulated by dose-level/regimen and timepoint, as appropriate. Descriptive statistics will be used in summarizing peripheral blood, and tumor PD markers. Absolute and percentage change from baseline will be calculated for the PD markers. Graphical techniques may be employed to better understand the relationship of the PD markers with dose and time. Correlations between PD markers, PK markers, and clinical response may be assessed through data tabulations and graphical techniques. The potential prognostic value of the PD markers may also be investigated.

"Use of this document is governed by the terms of use on the first page of this document."

6.10 PHARMACOKINETIC ANALYSES

PK data from patients who will receive at least one dose of RO6958688 or one dose of atezolizumab will be included in the PK analysis of RO6958688 and atezolizumab, respectively. Patients will be excluded from the PK analysis if they significantly violate the inclusion or exclusion criteria, deviate significantly from the protocol, or if data are unavailable or incomplete, which may influence the PK analysis.

Extensive and sparse PK sampling will be done in that study to properly characterize the PK of RO6958688 and atezolizumab when given in combination.

When appropriate, PK parameters will be derived from the plasma concentrations of RO6958688 and atezolizumab using standard non-compartmental methods. Individual data will be listed and summarized using descriptive statistics including but not limited to mean, standard deviation, geometric mean, coefficient of variation, median, and range, as appropriate. The parameters will include, e.g., AUC, CL, Vss, accumulation ratio, and terminal elimination half-life. Mean concentration versus time may be plotted on either semi-logarithmic or normal scales.

In addition, non-linear mixed effect modeling will be used to analyze the sparse sampling dose-concentration-time data of RO6958688. Population PK parameters such as CL and V will be estimated and the influence of various covariates on these parameters will be investigated. Secondary parameters such as AUC and C_{max} will be derived from the individual post-hoc predictions. The linearity of PK (AUC, C_{max}) will also be investigated.

Actual PK sampling times are presented in the Schedule of Assessments and Hourly tables (see Appendix 1).

6.11 Immunogenicity Analyses

Immunogenicity will be assessed for atezolizumab and RO6958688. The immunogenicity analyses will include all patients with at least one ADA assessment. Patients will be grouped according to treatment received or, if no treatment is received prior to study discontinuation, according to treatment assigned.

For atezolizumab and RO6958688, the number and proportion of ADA-positive patients and ADA-negative patients at baseline (baseline prevalence) and after baseline (post-baseline incidence) will be summarized by treatment group. When determining the post-baseline incidence, patients are considered to be ADA positive if they are ADA negative or have missing data at baseline but develop an ADA response following study drug exposure (treatment-induced ADA response), or if they are ADA positive at baseline and the titer of one or more post baseline samples is at least 4-fold (e.g., ≥ 0.60-titer unit) greater than the titer of the baseline sample (treatment-enhanced ADA response) considering drug concentration in individual post-dose sample. Patients are considered to be ADA negative if they are ADA negative or have missing data at baseline and all post-baseline samples are negative, or if they are ADA positive at

"Use of this document is governed by the terms of use on the first page of this document."

baseline but do not have any post-baseline samples with a titer that is at least 4-fold (e.g., ≥ 0.60-titer unit considering drug concentration in individual post-dose sample) greater than the titer of the baseline sample (treatment unaffected).

The relationship between ADA status and safety, efficacy, PK, and biomarker endpoints may be analyzed and reported via descriptive statistics and exploratory PK and PK–PD analyses.

6.12 INTERIM ANALYSES

Throughout the whole study, there will be ongoing safety and efficacy assessments and PK data analyses, which do not qualify as formal interim analyses. In particular, dose escalations in Part I will be based on the ongoing safety review of the data and especially DLT data.

In addition, there will be an internal interim analysis after Part I for selecting the recommended dose and schedule for Part II. Note: Part II will not open.

7. DATA COLLECTION AND MANAGEMENT

7.1 DATA QUALITY ASSURANCE

The Sponsor will be responsible for data management of this study, including quality checking of the data. Sites will be responsible for data entry into the EDC system.

A comprehensive validation check program will verify the data. Discrepancies will be generated automatically in the system at the point of entry or added manually for resolution by the investigator.

The Sponsor will produce a Data Handling Manual that describes the quality checking to be performed on the data. Central laboratory data will be sent directly to the Sponsor, using the Sponsor’s standard procedures to handle and process the electronic transfer of these data.

System backups for data stored by the Sponsor and records retention for the study data will be consistent with the Sponsor’s standard procedures.

7.2 ELECTRONIC CASE REPORT FORMS

Data for this study will be captured via an on line Electronic Data Capture (EDC) system. The data collected in the source documents is entered onto the study eCRF. An audit trail will maintain a record of initial entries and changes made; reasons for change; time and date of entry; and user name of person authorizing entry or change. For each patient enrolled, an eCRF must be completed and electronically signed by the principal investigator or authorized delegate from the study staff. If a patient withdraws from the study, the reason must be noted on the eCRF. If a patient is withdrawn from the study

“Use of this document is governed by the terms of use on the first page of this document.”

because of a treatment‑limiting adverse event, thorough efforts should be made to clearly document the outcome.

The investigator should ensure the accuracy, completeness and timeliness of the data reported to the sponsor/CRO in the eCRFs and in all required reports.

eCRFs will be submitted electronically to the Sponsor/CRO and should be handled in accordance with instructions from the Sponsor/CRO.

At the end of the study, the investigator will receive patient data for his or her site in a readable format on a compact disc that must be kept with the study records. Acknowledgement of receipt of the compact disc is required.

7.3 SOURCE DATA DOCUMENTATION

Study monitors will perform ongoing source data verification to confirm that critical protocol data (i.e., source data) entered into the eCRFs by authorized site personnel are accurate, complete, and verifiable from source documents.

Source documents (paper or electronic) are those in which patient data are recorded and documented for the first time. They include, but are not limited to, hospital records, clinical and office charts, laboratory notes, memoranda, patient-reported outcomes, evaluation checklists, pharmacy dispensing records, recorded data from automated instruments, copies of transcriptions that are certified after verification as being accurate and complete, microfiche, photographic negatives, microfilm or magnetic media, X‑rays, patient files, and records kept at pharmacies, laboratories, and medico‑technical departments involved in a clinical trial.

Before study initiation, data to be entered directly into the eCRFs (i.e., no prior written or electronic record of the data) and considered source data must be defined in the Trial Monitoring Plan.

Source documents that are required to verify the validity and completeness of data entered into the eCRFs must not be obliterated or destroyed and must be retained per the policy for retention of records described in Section 7.5.

To facilitate source data verification, the investigators and institutions must provide the Sponsor direct access to applicable source documents and reports for trial‑related monitoring, Sponsor audits, and IRB/EC review. The investigational site must also allow inspection by applicable health authorities.

7.4 USE OF COMPUTERIZED SYSTEMS

When clinical observations are entered directly into an investigational site's computerized medical record system (i.e., in lieu of original hardcopy records), the electronic record can serve as the source document if the system has been validated in

"Use of this document is governed by the terms of use on the first page of this document."

accordance with health authority requirements pertaining to computerized systems used in clinical research. An acceptable computerized data collection system allows preservation of the original entry of data. If original data are modified, the system should maintain a viewable audit trail that shows the original data as well as the reason for the change, name of the person making the change, and date of the change.

7.5 RETENTION OF RECORDS

Records and documents pertaining to the conduct of this study and the distribution of IMP, including eCRFs, Informed Consent Forms, laboratory test results, and medication inventory records, must be retained by the Principal Investigator for 15 years after completion or discontinuation of the study, or for the length of time required by relevant national or local health authorities, whichever is longer. After that period of time, the documents may be destroyed, subject to local regulations. No records may be disposed of without the written approval of the Sponsor. Written notification should be provided to the Sponsor prior to transferring any records to another party or moving them to another location.

Roche will retain study data for 25 years after the final Clinical Study Report has been completed or for the length of time required by relevant national or local health authorities, whichever is longer.

8. ETHICAL CONSIDERATIONS

8.1 COMPLIANCE WITH LAWS AND REGULATIONS

This study will be conducted in full conformance with the ICH E6 guideline for Good Clinical Practice and the principles of the Declaration of Helsinki, or the applicable laws and regulations of the country in which the research is conducted, whichever affords the greater protection to the individual. The study will comply with the requirements of the ICH E2A guideline (Clinical Safety Data Management: Definitions and Standards for Expedited Reporting). Studies conducted in the United States or under a U.S. Investigational New Drug (IND) application will comply with U.S. FDA regulations and applicable local, state, and federal laws. Studies conducted in the EU/EEA will comply with the EU Clinical Trial Directive (2001/20/EC) and applicable local, regional, and national laws.

8.2 INFORMED CONSENT

The Sponsor’s sample Informed Consent Form (and ancillary sample Informed Consent Forms such as a Child’s Assent or Caregiver's Informed Consent Form, if applicable) will be provided to each site. If applicable, it will be provided in a certified translation of the local language. The Sponsor or its designee must review and approve any proposed deviations from the Sponsor's sample Informed Consent Forms or any alternate consent forms proposed by the site (collectively, the “Consent Forms”) before IRB/EC submission. The final IRB/EC-approved Consent Forms must be provided to the Sponsor for health authority submission purposes according to local requirements.

“Use of this document is governed by the terms of use on the first page of this document.”

The Consent Forms must be signed and dated by the patient or the patient's legally authorized representative before his or her participation in the study. The case history or clinical records for each patient shall document the informed consent process and that written informed consent was obtained prior to participation in the study.

The Consent Forms should be revised whenever there are changes to study procedures or when new information becomes available that may affect the willingness of the patient to participate. The final revised IRB/EC-approved Consent Forms must be provided to the Sponsor for health authority submission purposes.

Patients must be re-consented to the most current version of the Consent Forms (or to a significant new information/findings addendum in accordance with applicable laws and IRB/EC policy) during their participation in the study. For any updated or revised Consent Forms, the case history or clinical records for each patient shall document the informed consent process and that written informed consent was obtained using the updated/revised Consent Forms for continued participation in the study.

A copy of each signed Consent Form must be provided to the patient or the patient's legally authorized representative. All signed and dated Consent Forms must remain in each patient's study file or in the site file and must be available for verification by study monitors at any time.

For sites in the United States, each Consent Form may also include patient authorization to allow use and disclosure of personal health information in compliance with the U.S. Health Insurance Portability and Accountability Act of 1996 (HIPAA). If the site utilizes a separate Authorization Form for patient authorization for use and disclosure of personal health information under the HIPAA regulations, the review, approval, and other processes outlined above apply except that IRB review and approval may not be required per study site policies.

8.3 INSTITUTIONAL REVIEW BOARD OR ETHICS COMMITTEE

This protocol, the Informed Consent Forms, any information to be given to the patient, and relevant supporting information must be submitted to the IRB/EC by the Principal Investigator and reviewed and approved by the IRB/EC before the study is initiated. In addition, any patient recruitment materials must be approved by the IRB/EC.

The Principal Investigator is responsible for providing written summaries of the status of the study to the IRB/EC annually or more frequently in accordance with the requirements, policies, and procedures established by the IRB/EC. Investigators are also responsible for promptly informing the IRB/EC of any protocol amendments (see Section 9.5).

In addition to the requirements for reporting all adverse events to the Sponsor, investigators must comply with requirements for reporting serious adverse events to the local health authority and IRB/EC. Investigators may receive written IND safety reports

"Use of this document is governed by the terms of use on the first page of this document."

or other safety‑related communications from the Sponsor. Investigators are responsible for ensuring that such reports are reviewed and processed in accordance with health authority requirements and the policies and procedures established by their IRB/EC, and archived in the site's study file.

8.4 CONFIDENTIALITY

The Sponsor maintains confidentiality standards by coding each patient enrolled in the study through assignment of a unique patient identification number. This means that patient names are not included in data sets that are transmitted to any Sponsor location.

Patient medical information obtained by this study is confidential and may only be disclosed to third parties as permitted by the Informed Consent Form (or separate authorization for use and disclosure of personal health information) signed by the patient, unless permitted or required by law.

Medical information may be given to a patient's personal physician or other appropriate medical personnel responsible for the patient's welfare, for treatment purposes.

Data generated by this study must be available for inspection upon request by representatives of the U.S. FDA and other national and local health authorities, Sponsor monitors, representatives, and collaborators, and the IRB/EC for each study site, as appropriate.

8.5 FINANCIAL DISCLOSURE

Investigators will provide the Sponsor with sufficient, accurate financial information in accordance with local regulations to allow the Sponsor to submit complete and accurate financial certification or disclosure statements to the appropriate health authorities. Investigators are responsible for providing information on financial interests during the course of the study and for one year after completion of the study (i.e., LPLV).

9. STUDY DOCUMENTATION, MONITORING, AND ADMINISTRATION

9.1 STUDY DOCUMENTATION

The investigator must maintain adequate and accurate records to enable the conduct of the study to be fully documented, including but not limited to the protocol, protocol amendments, Informed Consent Forms, and documentation of IRB/EC and governmental approval. In addition, at the end of the study, the investigator will receive the patient data, which includes an audit trail containing a complete record of all changes to data.

Roche shall also submit an Annual Safety Report once a year to the IEC and CAs according to local regulatory requirements and timelines of each country participating in the study.

"Use of this document is governed by the terms of use on the first page of this document."

Sampling for the RCR is contingent on review and approval for the exploratory biomarker assessments and written informed consent by an appropriate regulatory body (depending on the country where the study is performed) and a site's Institutional Review Board (IRB) / Ethics Committee (EC). If a regulatory or site's IRB/ EC does not approve the sampling for the exploratory assessments the section on biomarker sampling will not be applicable.

It is the understanding of the sponsor that this protocol (and any modifications) as well as appropriate consent procedures and advertisements, will be reviewed and approved by an Institutional Review Board (IRB). This board must operate in accordance with the current Federal Regulations. The sponsor will be sent a letter or certificate of approval prior to initiation of the study, and also whenever subsequent amendments /modifications are made to the protocol. Roche shall also submit an IND Annual Report to FDA according to local regulatory requirements and timelines.

9.2 SITE INSPECTIONS

Site visits will be conducted by the Sponsor or an authorized representative for inspection of study data, patients' medical records, and eCRFs. The investigator will permit national and local health authorities, Sponsor monitors, representatives, and collaborators, and the IRBs/ECs to inspect facilities and records relevant to this study.

9.3 ADMINISTRATIVE STRUCTURE

The Sponsor of the trial is F. Hoffmann-La Roche Ltd. The Sponsor is responsible for the study management (monitoring), data management, statistical analysis, and medical writing for this clinical study report. A clinical study report will be written and distributed to Health Authorities as required by applicable regulatory requirements.

The protocol will be submitted to country/institutional ethics committees.

9.4 PUBLICATION OF DATA AND PROTECTION OF TRADE SECRETS

The results of this study may be published or presented at scientific meetings. If this is foreseen, the investigator agrees to submit all manuscripts or abstracts to the Sponsor prior to submission. This allows the Sponsor to protect proprietary information and to provide comments based on information from other studies that may not yet be available to the investigator.

The Sponsor will comply with the requirements for publication of study results. In accordance with standard editorial and ethical practice, the Sponsor will generally support publication of multicenter trials only in their entirety and not as individual center data. In this case, a coordinating investigator will be designated by mutual agreement.

"Use of this document is governed by the terms of use on the first page of this document."

Any formal publication of the study in which contribution of Sponsor personnel exceeded that of conventional monitoring will be considered as a joint publication by the investigator and the appropriate Sponsor personnel.

General Guidelines

- Authorship will follow the guidelines for the target journal. This especially considering the maximum number of authors permitted for the target journal.
- The maximum number of authors will usually be included.
- The investigator who recruits the highest number of evaluable patients may choose to be the first or the last author and will be presenter/corresponding author on the main publication.
- Roche authors should not outnumber non-Roche authors and usually should not exceed three.
- Roche authors should not be first author.

Publication

The following are based on general rules for Roche–sponsored oncology studies:

- Subsets per country can be published by the country's primary investigator, after the main publication and in accordance with the requirements of the study contract held with the site
- Acknowledgement of supporting bodies and other investigators must be made
- Any manuscript must be passed through the Sponsor for review prior to submission. The Sponsor shall review such manuscripts within 30 working days
- Authorship will be in line with International Committee of Medical Journal Editors authorship requirements.

Any inventions and resulting patents, improvements, and/or know-how originating from the use of data from this study will become and remain the exclusive and unburdened property of the Sponsor, except where agreed otherwise.

9.5 PROTOCOL AMENDMENTS

Any substantial protocol amendments will be prepared by the Sponsor. Substantial protocol amendments will be submitted to the IRB/EC and to regulatory authorities in accordance with local regulatory requirements.

Approval must be obtained from the IRB/EC and regulatory authorities (as locally required) before implementation of any changes, except for changes necessary to eliminate an immediate hazard to patients or any non-substantial changes, as defined by regulatory requirements.

"Use of this document is governed by the terms of use on the first page of this document."

10. REFERENCES

Affara NI, Ruffell B, Medler TR, et al. B cells regulate macrophage phenotype and response to chemotherapy in squamous carcinomas. Cancer Cell 2014; 25(6):809–21.

Alexandrov LB, Nik-Zainal S, Wedge DC, et al. Signatures of mutational processes in human cancer. Nature 2013;500:415-21.

Bai S, Jorga K, Xin Yet al. A guide to rational dosing of monoclonal antibodies. Clin Pharmacokinet 2012;51:119-35.

Bacac M, Fauti T, Colombetti S, et al. Abstract 1494: combination of CEA TCB, a novel T-cell bispecific antibody for the treatment of solid tumors, with PD-L1 checkpoint blockade [abstract]. Cancer Res 2016b;76(14 Suppl)1494.

Bailey S, Neuenschwander B, Laird G, Branson M. A Bayesian Case Study in Oncology: Phase I Combination Dose-Finding Using Logistic Regression with Covariates. J Biopharm Stat 2009;19:469–84.

Bellone S, Black J, English DP, et al. Solitomab, an EpCam/CD3 bispecific antibody construct (BiTE©), is highly active against primary uterine serous papillary carcinoma cell lines in virto. Am J of Obstetrics and Gynecology 2015.

Callahan MK, Horak CE, Curran MA, et al. Peripheral and tumor immune correlates in patients with advanced melanoma treated with combination nivolumab (anti-PD-1, BMS-936558, ONO-4538) and ipilimumab [abstract]. J Clin Oncol 2013;31:3003.

Carosella ED, Ploussarrd G, LeMaoult, et al. A Systematic Review of Immunotherapy in Urologic Cancer: Evolving Roles for Trageting of CTLA-4, PD-1/PD-L1, and HLA-G. European Urology 2015; 68:267-279.

Chen DS, Irving BA, Hodi FS. Molecular pathways: next-generation immunotherapy inhibiting programmed death ligand 1 and programmed death-1. Clin. Cancer Res 2012;18:6580-7.

Chen F, Teachey DT, Pequignot E, et al. Measuring IL-6 and sIL-6R in serum from patients treated with tocilizumab and/or siltuximab following CAR T cell therapy. J Immunol Methods 2016;434,1−8.

Conrad ML, Davis WC, Koop BF. TCR and CD3 antibody cross-reactivity in 44 species. J Immunol 1991;147:3047–52.

Curiel TJ, Wei S, Dong H, et al. Blockade of B7-H1 improves myeloid dendritic cell-mediated antitumor immunity. Nat Med. 2003 9, 562-567.

De Benedetti F, Brunner HI, Ruperto N, Kenwright A, Wright S, et al. Randomized trial of tocilizumab in systemic juvenile idiopathic arthritis. N Engl J Med. 2012;367(25):2385-95.

“Use of this document is governed by the terms of use on the first page of this document.”

de la Hera A, Mueller U, Olsson C, et al. Structure of the T cell Antigen Receptor (TCR): Two CD3e Subunits in a Functional TCR/CD3 Complex. J Exp Med 1991;173:7–17.

Deng R, Bumbaca D, Pastuskovas CV, et al. Preclinical pharmacokinetics, pharmacodynamics, tissue distribution, and tumor penetration of anti-PD-L1 monoclonal antibody, an immune checkpoint inhibitor. MAbs 2016;8:593 –603.

Di Giacomo AM, Biagioli M, Maio M. The emerging toxicity profiles of anti–CTLA-4 antibodies across clinical indications. Semin Oncol 2010;37:499–507.

Doessegger L, Banholzer ML. Clinical development methodology for infusion-related reactions with monoclonal antibodies. Clin Transl Immunology. 2015;4:e39.

European Medicines Agency (EMA). Summary of opinion (post authorisation): RoActemra (tocilizumab). Committee for Medicinal Products for Human Use (CHMP); EMA/CHMP/419360/2018 (28 June 2018).

Fehrenbacher L,Spira A, Ballinger M, et al. Atezolizumab versus docetaxel for patients with previously treated non-small-cell lung cancer (POPLAR): a multicentre, open-label, phase 2 randomised controlled trial. Lancet, 2016;387(10030):1837-46.

Gomez-Mantila JD, Troconiz IF Review on modeling anti-antibody responses to monoclonal antibodies. J Pharmacokinet Pharmacodyn 2014;41:523-536.

Gunderson AJ, Coussens LM. B cells and their mediators as targets for therapy in solid tumors. Exp Cell Res 2013; 319(11):1644-9.

Grupp SA, Kalos M, Barrett D, Aplenc R, Porter DL, et al. Chimeric antigen receptor-modified T cells for acute lymphoid leukemia. N Engl J Med. 2013; 368(16):1509–18.

Hejblum G. et al. A web-based delphi study for eliciting helpful criteria in the positive diagnosis of hemophagocytic syndrome in adult patients. PLoS One (Public Library of Science) April 2014; Volume 9, Issue 4.

Hodi FS, O'Day SJ, McDermott DF, et al. Improved survival with ipilimumab in patients with metastatic melanoma. N Engl J Med 2010;363:711-23.

Honeychurch J, Cheadle EJ, Dovedi SJ, et al. Immuno-regulatory antibodies for the treatment of cancer. Expert Opin. Biol. Ther. 2015, 15(6): 787-801.

Hoos A, Eggermont AM, Janetzki S, et al. Improved endpoints for cancer immunotherapy trials. J Natl Cancer Inst 2010:102:1388–97.

"In vivo anti-tumor activity of CEA TCB (RO6958688) in combination with anti-PD-L1 blocking antibody". Roche Report No. 1066291, August, 2015.

Kang SH, Ahn C. An investigation of the traditional algorithm-based designs for phase I cancer clinical trials. Drug Information J 2002;36:865–73.

“Use of this document is governed by the terms of use on the first page of this document.”

Kang SH, Ahn C. The expected toxicity rate at the maximum tolerated dose in the standard phase I cancer clinical trial design. Drug Information J 2001;35:1189–1200.

Kilinc MO, Gu T, Harden JL, et al. Central role of tumor-associated CD8+ T effector/memory cells in restoring systemic antitumor immunity. J Immunol 2009;182:4217-25.

Krupka C, Kufer P, Kischel R, et al. Blockade of the PD-1/PD-L1 axis augments lysis of AML cells by the CD33/CD3-BiTE® antibody construct AMG 330: reversing a T-cell induced immune escape mechanism. Leukemia 2015; accepted article preview online 4 August 2015; Published ahead of advanced online publication.

Lawrence MS, Stojanov P, Polak P, et al. Mutational heterogeneity in cancer and the search for new cancer-associated genes. Nature 2013;499:214-18.

Le RQ, Li L, Yuan W, et al. FDA Approval Summary: Tocilizumab for Treatment of Chimeric Antigen Receptor T Cell ‐ Induced Severe or Life ‐ Threatening Cytokine Release Syndrome. Oncologist 2018; 23:943–7.

Lee DW, Gardner R, Porter DL, et al. Current concepts in the diagnosis and management of cytokine release syndrome. Blood. 2014;124:188–95.

Le Tourneau C, Lee JJ, Siu LL. Dose escalation methods in phase I cancer clinical trials. J Natl Cancer Inst 2009;101:708–20.

Levey AS, Stevens LA, Schmid CH, et al. A new equation to estimate glomerular filtration rate. Ann Intern Med 2009; 150(9):604-12.

Mahoney KM, Freeman GJ, McDermott DF The Next Immune-Checkpoint Inhibitors:PD-1/PD-L1 Blockade in Melanoma Clinical Therapeutics 2015; 37(4):764-782.

Maude SL, Barrett D, Teachey DT, Grupp SA. Managing cytokine release syndrome associated with novel T cell-engaging therapies. Cancer J. 2014;20:119–22.

Mössner E, Brünker P, Moser S, et al. Increasing the efficacy of CD20 antibody therapy through the engineering of a new type II anti-CD20 antibody with enhanced direct and immune effector cell–mediated B-cell cytotoxicity. Blood. 2010;115:4393-4402.

Nagorsen D and Baeuerle PA. Immunomodulatory therapy of cancer with T cell engaging BiTE antibody blinatumomab. Exp Cell Res. 2011;317(9):1255-1260.

Nagorsen D, Kufer P, Patrick A, et al. Blinatumomab: A historical perspective. Pharmacology & Therapeutics 2012;136:334–342.

National Institutes of Health (NIH). Recombinant DNA Advisory Committee (RAC). Cytokine Release Syndrome after T Cell Immunotherapy. Bethesda, Maryland. NIH Videocast 9 June 2015. Available at: https://videocast.nih.gov/summary.asp?Live=16420&bhcp=1. Accessed 30 January 2017.

“Use of this document is governed by the terms of use on the first page of this document.”

Neuenschwander, B., Branson, M., & Gsponer, T. (2008). Critical aspects of the Bayesian approach to phase I cancer trials. Statistics in Medicine, 27(13), 2420–39. doi:10.1002/sim.3230.

Nishino M, Giobbie-Hurder A, Gargano M, et al. Developing a common language for tumor response to immunotherapy: immune-related response criteria using unidimensional measurements. Clin Cancer Res 2013;19:3936–4.

Nishino M, Gargano M, Suda M, et al. Optimizing immune-related tumor response assessment: does reducing the number of lesions impact response assessment in melanoma patients treated with ipilimumab? J Immunother Cancer 2014;2:17.

Oberst MD, Fuhrmann S, Mulgrew K, et al. CEA/CD3 bispecific antibody MEDI-565/AMG211 activation of T cells and subsequent killing of human tumors is independent of mutations commonly found in colorectal adenocarcinomas. mAbs 2014, 6:6, 1571-1584.

Pages F, Berger A, Camus M , et al. Effector memory T cells, early metastasis, and survival in colorectal cancer. N Engl J Me d 2005;353:2654-66.

Panelli MC, White R, Foster M, et al. Forecasting the cytokine storm following systemic interleukin (IL)-2 administration. J Transl Med. 2004;2:17.

Pessano S, Oettgen H, Bhan AK, et al. The T3/T cell receptor complex: antigenic distinction between the two 20-kd T3 (T3-delta and T3-epsilon) subunits. EMBO J. 1985;4:337–44.

Roche Report No.1072962, 2016. Quantitative in vivo imaging and biodistribution of CEA-targeted T-cell bispecific antibodies in tumor bearing CD34+ human hematopoetic stem cell engrafted NSG mice.

Rosenberg JE, Hoffman-Censits J, Powles T, et al. Atezolizumab in patients with locally advanced and metastatic urothelial carcinoma who have progressed following treatment with platinum-based chemotherapy: a single-arm, multicentre, phase 2 trial. Lancet, 2016 ;387(10031):1909-20.

Russell et al. Vasopressin versus norepinephrine infusion in patients with septic shock. N Engl J Med 2008;358:877–88.

Salmerón A, Sanchez-Madrid F, Ursa MA, et al. A conformational epitope expressed upon association of CD3e with either CD3d or CD3g is the main target for recognition by anti-CD3 monoclonal antibodies. J Immunol 1991;147:3047–52.

Sauerborn M, Beers van M.C.M.M, Jiskoot W,. et al. Antibody Response Against Betaferon® in Immune Tolerant Mice: Involvement of Marginal Zone B-cells and CD4+ T-cells and Apparent Lack of Immunological Memory J Clin Immunol 2013; 33:255–263.

Schmidli, H., Gsteiger, S., Roychoudhury, S., O'Hagan, A., Spiegelhalter, D., & Neuenschwander, B. (2014). Robust meta-analytic-predictive priors in clinical trials with historical control information. Biometrics, 4(2004).

"Use of this document is governed by the terms of use on the first page of this document."

Segal NH, Parsons DW, Peggs KS, et al. Epitope landscape in breast and colorectal cancer. Cancer Res 2008;68:889-92.

Singh JA, Beg S, Lopez-Olivo MA. Tocilizumab for rheumatoid arthritis: a Cochrane systematic review. J Rheumatol. 2011;38(1):10-20.

Steiglmaier J, Benjamin J, Nagorsen D. Utilizing the BiTE (bispecific T-cell engager) platform for immunotherapy of cancer. Expert Opin. Biol. Ther 2015 15(8), 1093–1099.

Swaika A, Hammond WA, Joseph RW. Current state of anti-PD-L1 and anti-PD-1 agents in cancer therapy, Volume 67, Issue 2, Part A, October 2015, Pages 4–17.

Tabernero et al. Clinical evidence of intra-tumoral immune activation and tumor targeting with RG7813, a CEA-targeted engineered IL-2 immunocytokine. ESMO 2015 oral presentation. https://www.ecco-org.eu/Vienna2015/Scientific-Programme/Searchable-Programme?trackid=00179#anchorScpr

Teachey DT, Rheingold SR, Maude SL, et al. Cytokine release syndrome after blinatumomab treatment related to abnormal macrophage activation and ameliorated with cytokine-directed therapy. Blood. 2013;121:5154–7.

Tocilizumab (Actemra®) United States Package Insert. Available at: https://www.gene.com/download/pdf/actemra_prescribing.pdf, accessed on 17 September 2017.

Topalian et al. Safety, Activity, and Immune Correlates of Anti–PD-1 Antibody in Cancer. N Engl J Med 2012;366(26):2443-2454.

van Rooij N, van Buuren MM, Philips D, et al. Tumor exome analysis reveals neoantigen-specific T-cell reactivity in an ipilimumab-responsive melanoma. Clin Oncol 2013;31:e439-42.

Weber WA. Assessing tumor response to therapy. J Nucl Med. 2009 May;50 Suppl 1:1S-10S.

Weiner GJ: Making a better antibody: all is not lost, Blood 2010, 115:5127-5128.

Wolchok JD, Hoos A, O'Day S, et al. Guidelines for the evaluation of immune therapy activity in solid tumors: immune-related response criteria. Clin. Cancer Res 2009;15:7412–20.

Woo P, Wilkinson N, Prieur AM, Southwood T, Leone V, Livermore P, Wythe H, Thomson D, Kishimoto T. Open label phase II trial of single, ascending doses of MRA in Caucasian children with severe systemic juvenile idiopathic arthritis: proof of principle of the efficacy of IL-6 receptor blockade in this type of arthritis and demonstration of prolonged clinical improvement. Arthritis Res Ther. 2005;7(6):R1281-8.

"Use of this document is governed by the terms of use on the first page of this document."

Yokota S, Miyamae T, Imagawa T, Iwata N, Katakura S, et al. Therapeutic efficacy of humanized recombinant anti-interleukin-6 receptor antibody in children with systemic-onset juvenile idiopathic arthritis. Arthritis Rheum. 2005 Mar;52(3):818–25.

Zhang Y,Huang S, Gong D, et al. Programmed death-1 upregulation is correlated with dysfunction of tumor-infiltrating CD8+ T lymphocytes in human non-small cell lung cancer. Cell.Mol.Immunol 2010, 7, 389-395.

"Use of this document is governed by the terms of use on the first page of this document."

Appendix 1
Schedule of Assessments

TABLE A1: SCHEDULE OF ASSESSMENTS: QW RO6958688 ADMINISTRATION SCHEDULE

Cycle	Screening	Cycle 1							Cycle 2				Cycle 3			Cycle 4			Cycle 5			Cycle 6			Cycle 7 onwards	End of treatment	28-Day Safety Follow Up[m]
Day	D-28 to D1	Day 1[o]	Day 2	Day 3	Day 8	Day 9	Day 15	Day 16	Day 1	Day 2	Day 8	Day 15	Day 1	Day 8	Day 15	Day 1	Day 8	Day 15	Day 1	Day 8	Day 15	Day 1	Day 8	Day 15	Day 1		
Assessments[a]																											
Informed Consent[b]	x																										
Eligibility	x																										
Demography	x																										
Medical History	x																										
Physical Examination[c]	x	x			x		x		x		x	x	x	x	x	x	x	X	x	x	x	x	x	x	x	x	x
Vital Signs	x	x	x	x	x	x	x	x	x	x	x	x	x	x	x	x	x	X	x	x	x	x	x	x	x[p]	x	x
Administration of atezolizumab Q3W[x]		x[z]							x				x			x			x			x			x		
Administration of RO6958688 QW[y]		x[z]			x		x		x		x	x	x	x	x	x	x	X	x	x	x	x	x	x	x[ae]		
ECG-12 lead[d]	x	x			x		x		x		x	x	x	x	x	x	x	X	x	x	x	x	x	x	x	x	x
ECOG Performance Status	x	x			x		x		x		x	x	x	x	x	x	x	X	x	x	x	x	x	x	x[q]	x	x
Pulmonary Function test[d]	x																										
Hematology[g]	x	x	x	x	x	x	x	x	x	x	x	x	x	x	x	x	x	X	x	x	x	x	x	x	x[r]	x	x
Blood Chemistry[g,aa]	x	x	x	x	x	x	x	x	x	x	x	x	x	x	x	x	x	X	x	x	x	x	x	x	x[r]	x	x
Serum Thyroid-Stimulating Hormone Free T3-T4[l]		x																	x							x	x
Coagulation[g,h]	x	x	x	x	x	x	x	x	x	x	x	x	x	x	x	x	x	X	x	x	x	x	x	x	x[r]	x	x
Urinalysis[g]	x	x	x	x	x	x	x	x	x	x	x	x	x	x	x	x	x	X	x	x	x	x	x	x	x[r]	x	x
HBV and HCV Serology	x																										
Auto-Antibody Panel[v]	x								x							x						x			x[w]		
Pregnancy Test[e]	X													X												x	x

“Use of this document is governed by the terms of use on the first page of this document.”

Appendix 1
Schedule of Assessments (cont.)

TABLE A1: SCHEDULE OF ASSESSMENTS: QW RO6958688 ADMINISTRATION SCHEDULE (CON'T)

Cycle	Screening	Cycle 1							Cycle 2				Cycle 3			Cycle 4			Cycle 5			Cycle 6			Cycle 7 onwards	End of treatment	28-Day Safety Follow Up[m]
Day	D-28 to D1	Day 1[o]	Day 2	Day 3	Day 8	Day 9	Day 15	Day 16	Day 1	Day 2	Day 8	Day 15	Day 1	Day 8	Day 15	Day 1	Day 8	Day 15	Day 1	Day 8	Day 15	Day 1	Day 8	Day 15	Day 1		
Atezolizumab PK Sampling[ab]		x			x		x		x		x	x	x			x						x			x[af]	x	x
RO6958688 PK Sampling[ab]		x	x	x	x	x	x	x	x	x	x	x	x	x	x	x	x	X	x	x	x	x	x	x	x[t]	x	x
Atezolizumab Anti-Drug Antibody (ADA)[ac]		x							x				x			x									x[af]	x	
RO6958688 Anti-Drug Antibody (ADA)[ac]		x			x		x		x		x	x	x	x	x	x	x	X	x	x	x	x	x	x	x[u]	x	x
PD Blood Flow Cytometry		x	x		x				x				x														
PD Blood Cytokines[f]		x	x	x	x	x	x		x	x	x	x	x														
Archival tumor Biopsy	X																										
Fresh Tumor Biopsy[j]	X								x				x														
Clinical Genotyping[ag]		x																									
RCR Sample (DNA and RNA)[ag]		x																									
CEA confirmation on Archival Tissue[k]	X																										
FDG-PET[n]	x[s]										x[s]												x[s]				
TCR Vβ[ah]		x											x														
Sol CEA[ai]		x							x				x														
Tumor Assessment[l]	x														x								X			x	
Tumor Growth Kinetic[ad]	x																										
Adverse Events	X																										
Previous and Concomitant Treatments	X																										

"Use of this document is governed by the terms of use on the first page of this document."

TABLE A1: SCHEDULE OF ASSESSMENTS: QW RO6958688 ADMINISTRATION SCHEDULE (CON'T)

ADA = anti-drug antibody; CEA = carcinoembryonic antigen; D = day; ECOG = Eastern Cooperative Oncology Group; PD = pharmacodynamics; PK = pharmacokinetic.

[a] All visit and their assigned safety assessments (hematology, blood chemistry, coagulation, urinalysis, physical examination), unless otherwise indicated should occur within a 3-day time window.

[b] Informed consent must be obtained before any study-specific procedures. All patients will be tested for HBV and HCV serology. Patients with HIV infection, or active hepatitis B (chronic or acute), or active hepatitis C infection are not eligible.

[c] Physical examinations (including weight) and ECOG Performance Status will be done at screening, at the time of treatment administration, at the EOT and at the 28-day safety follow-up visits. Results must be obtained prior to infusion.

[d] Triplicate 12-lead ECG at screening (within 7 days before first dose of RO6958688) pre- and end of infusion on Cycle 1 Day 1 and Cycle 1 Day 15 and at the 28-day safety follow-up visit. Pre-infusion at all other study drug administrations. Additional unscheduled ECG assessments should be performed in case of abnormalities and if clinical symptoms occur. Recording must be done prior to PK sampling.

[e] Serum pregnancy test at screening, within 7 days prior to first dose. From the date of first serum pregnancy test at screening, a urine pregnancy test must be performed every 4 weeks. Also to be performed at study termination and safety follow-up visit (28 days post last infusion).

[f] Serum or plasma samples for the assessment of cytokine (PD blood cytokines) release will be collected. At the time of an IRR, please see IRR SoA Table A3.

[g] Hematology, blood chemistry, coagulation, and urinalysis can be performed up to 24 hours (72h if during weekend) prior to scheduled dosing. Results must be obtained prior to infusion. Soluble CEA will be measured as part of the blood chemistry measurements on Cycle 1 Day 1 and every 6 weeks thereafter for patients who continue treatment and at the end of treatment visit. Soluble CD25 is to be obtained at screening and during the treatment period at Day 1 of every cycle (samples can be drawn 7 days prior to Day 1 of each cycle). Before IMP administration on Day 1 of each cycle, the investigator should confirm that the result of soluble CD25 measurement from the previous week or if available from the same day is not significantly high. If there is a significantly high level, the investigator should discuss with the Medical Monitor if the patient can or cannot receive further IMP administration. Ferritin should be obtained at screening and every week during the treatment period: Day 1, Day 8 and Day 15 of every cycle (-2 days).

[h] For coagulation (including PT/INR and PTT), an additional sample will be taken at the time of an IRR or hypersensitivity reaction, see IRR SoA Table A3. Additional coagulation parameters (i.e., first chromogenic antithrombin III then antigenic antithrombin III in case of chromogenic antithrombin III decrease, fibrinogen, prothrombin time, fibrin degradation products, D-dimer) could be assessed according to clinical judgment.

[i] Serum TSH, free T3 (or total T3 for sites where free T3 is not performed) and T4 levels will be assessed at baseline pre-dose at Cycle 1, on study (every 3 months) and at study completion/early termination visit and safety follow-up visit (28 days after last dose of study drug).

"Use of this document is governed by the terms of use on the first page of this document."

TABLE A1: SCHEDULE OF ASSESSMENTS: QW RO6958688 ADMINISTRATION SCHEDULE (CON'T)

[j] Mandatory tumor biopsy samples will be collected on two occasions (once at baseline and once during the study treatment period), except for NSCLC patients for whom there is no accessible lesion. On treatment biopsies will be collected as per Section 3.3.2.2. If preliminary data suggest that modification of the on-treatment tumor biopsy timepoint would be more appropriate, alternative on-treatment tumor biopsy timepoints could be considered in the future cohorts. Unscheduled tumor biopsies can be taken at any time point, if considered necessary for tumor assessment and patient management as decided by the investigator.

[k] CEA expression for eligibility will be confirmed centrally for U.S. and Canada patients on archival tumor material (refer to inclusion criterion #13), if available, or confirmed from a freshly obtained tumor biopsy. Results available prior to screening (outside the 28-day window) can be used.

[l] Tumor assessments will be performed at screening, then every 8 weeks after Cycle 1 Day 1 for the first year, and thereafter every 12 weeks for the second year until disease progression or treatment discontinuation. Optional latest pre-study CT scan should be provided for assessment of tumor growth kinetics within 6 weeks of patient entering the study. For the scheduled tumor assessments beyond Screening a (+/- 7-day window is permitted).

[m] Patients who complete the study or discontinue from the study early will be asked to return to the clinic 28 days after the last dose of study drugs for post-study follow-up visits.

[n] FDG-PET should be done at baseline (Day-14 to D-1) but before the baseline biopsy. The on-treatment FDG-PET scan must be done at week 4 (C2D8 +1 week) and at week 16 (C6D8 pre-dose and up to 72h before the visit) in case of dose delay the FDG-PET should be obtained at week 4 (+1week) and week 16. If preliminary data suggest that modification of the on-treatment FDG-PET timepoint would be more appropriate, alternative on-treatment FDG PET timepoints could be considered. Please refer to Appendix 9 for further information.

[o] At least 24 hours (overnight) hospital stay required following administration of study drug at Cycle 1 Day 1.

[p] Vital signs to be assessed at D1, D8 and D15 from cycle 7 onwards.

[q] ECOG to be assessed at D1, D8 and D15 from cycle 7 onwards.

[r] Hematology, blood chemistry, coagulation and urinalysis (to be drawn at D1, D8 and D15 from cycle 7 onwards) can be performed up to 24-h (72-h if during weekend) hours prior to scheduled dosing. Results must be obtained prior to atezolizumab and RO6958688 infusion. Before each IMP administration, the investigator should confirm that the result of soluble CD25 measurement from the previous week or if available from the same day is not significantly high. If there is a significantly high level, the investigator should discuss with the Medical Monitor if the patient can or cannot receive further IMP administration.

[s] For FDG PET assessment, body weight, last calorific meal information, glucose level are needed (see Appendix 9 of the protocol for more information).

[t] RO6958688 PK sampling to be drawn preinfusion and at end of infusion at D1, D8 and D15 of each cycle from Cycle 7 to Cycle 10. At Cycle 11 and every second cycle, RO6958688 PK samples to be drawn preinfusion and at end of infusion on D1.

[u] RO6958688 ADA sampling to be drawn preinfusion at D1, D8 and D15 of each cycle from Cycle 7 to Cycle 10. At Cycle 11 and every second cycle, RO6958688 ADA samples to be drawn preinfusion on Day 1 at the mentioned timepoints.

"Use of this document is governed by the terms of use on the first page of this document."

TABLE A1: SCHEDULE OF ASSESSMENTS: QW RO6958688 ADMINISTRATION SCHEDULE (CON'T)

[v] In patients who develop signs and/or symptoms suggestive of auto-immune disease while on treatment, the antibody panel should be repeated. The autoantibody panel will be assessed at screening, pre-dose every second cycle, and every 3 months after the third month on therapy (samples can be drawn up to 1 week before Day 1 visit).

[w] To be performed every 3 months after Cycle 6 (ie. Cycle 10, Cycle 14…).

[x] Atezolizumab will be delivered over 60 (+/-15) minutes. If the first infusion is tolerated without infusion associated adverse events, the second infusion may be delivered over 30 (+/-10) minutes. If the 30-minute infusion is well tolerated, all subsequent infusions may be delivered over 30 (+/-10) minutes.

[y] The start of the RO6958688 infusion on Day 1 of each cycle should be after the end of the atezolizumab infusion as per Section 4.3.2.3.

[z] Prophylactic corticosteroids to be administered at C1D1 as per Table 2. The use of analgesics and/or antihistamines is allowed to minimize expected flu like symptoms prior to the first RO6958688 administration.

[aa] Serum CEA will also be measured as a disease monitoring marker (as part of the serum chemistry panel).

[ab] Blood for PK should always be drawn at the indicated time point.

[ac] Additional samples will be drawn at the time of an IRR, see IRR SoA Table A3 (if occurring at Cycle 2 or subsequent cycles) or hypersensitivity reaction, treatment discontinuation *and* at the 28-day safety follow-up visit (RO6958688 only). A PK sample will be taken at the time of ADA sampling.

[ad] An exploratory assessment of tumor growth kinetics will be made by comparing post-treatment scans with at least 2 pre-treatment scans not older than 12 weeks prior to C1D1, if available. The two pre-treatment scans will consist of a pre-treatment scan (if available) and the study baseline scan, and will allow estimation of tumor growth rate before start of treatment.

[ae] Administration of RO6958688 to be done at D1, D8 and D15 from Cycle 7 onwards.

[af] Atezolizumab PK and ADA sampling to be drawn at Cycle 8 Day 1 then every 8 cycles from this timepoint onwards.

[ag] Clinical genotyping and RCR (DNA and RNA) samples to be drawn before any IMP administration.

[ah] For TCR Vβ, at each time point Cycle 1 Day 1 and Cycle 3 Day 1, 2 whole blood samples should be drawn. Samples should be taken prior to IMP administration.

[ai] For soluble CEA central assessment: samples will be collected pre-dose at: C1D1 (baseline), C2D1 and C3D1

[aj] Evaluate DLCO (corrected for both alveolar volume and hemoglobin) and FEV1/VC/TLC at screening for patients with bilateral lung metastases or patients with lobectomy or pneumonectomy with lung metastases in the remaining lung. Additional tests may be performed if clinically indicated.

"Use of this document is governed by the terms of use on the first page of this document."

Appendix 1
Schedule of Assessments (cont.)

TABLE A2: SCHEDULE OF HOURLY ASSESSMENTS: QW RO6958688 ADMINISTRATION SCHEDULE

Cycle	Day	Scheduled Time (h)	Vital Signs[b,n,o]	ECG-12 lead[c]	RO6958688 PK Sampling[d]	Atezolizumab PK Sampling[e]	PD Blood Flow Cytometry[m]	PD Blood Cytokines[f]	RO6958688 Anti-Drug Ant body (ADA)[g]	Atezolizumab Anti-Drug Ant body (ADA)[g]	Tumor Biopsy[h]	FDG PET[i]	TCR Vβ[l]
Screening	D-28 to D-1[a]		x	x							x	x	
Cycle 1	Day 1	Pre-infusion[j]	x	x	x	x	x	x	x	x			x
		0.5h (+/- 10 min) atezolizumab post EOI				x							
		End of RO6958688 infusion	x	x	x			x					
		2 hours post end of RO6958688 infusion			x			x					
		4 hours post end of RO6958688 infusion						x					
	Day 2	24 hours	x		x		x	x					
	Day 3	48 hours	x		x			x					
	Day 8	Pre-infusion	x	x	x	x	x	x	x				
		End of RO6958688 infusion	x		x			x					
		2 hours post end of RO6958688 infusion			x			x					

“Use of this document is governed by the terms of use on the first page of this document.”

Appendix 1
Schedule of Assessments (cont.)

TABLE A2: SCHEDULE OF HOURLY ASSESSMENTS: QW RO6958688 ADMINISTRATION SCHEDULE (CON'T)

Cycle	Day	Scheduled Time (h)	Vital Signs[b,n,o]	ECG-12 lead[c]	RO6958688 PK Sampling[d]	Atezolizumab PK Sampling[e]	PD Blood Flow Cytometry[m]	PD Blood Cytokines[f]	RO6958688 Anti-Drug Antibody (ADA)[g]	Atezolizumab Anti-Drug Antibody (ADA)[g]	Tumor Biopsy[h]	FDG PET[i]	TCR Vβ[l]
Cycle 1	Day 9	192 hours	x		x			x					
	Day 15	Pre-infusion	x	X	x	x		x	x				
		End of infusion	x	X	x			x					
		2 hours post end of infusion			x								
		4 hours post end of RO6958688 infusion						x					
	Day 16	360 hours	x		x								
Cycle 2	Day 1	Pre-infusion[j]	x	X	x	x	X	x	x	x	x		
		0.5h (+/- 10 min) atezolizumab post EOI				x							
		End of RO6958688 infusion	x		x			x					
		2 hours post end of RO6958688 infusion			x								
	Day 2	24 hours	x		x			x					
	Day 8	Pre-infusion	x	X	x	x		x	x			x	
		End of RO6958688 infusion			x			x					

"Use of this document is governed by the terms of use on the first page of this document."

TABLE A2: SCHEDULE OF HOURLY ASSESSMENTS: QW RO6958688 ADMINISTRATION SCHEDULE (CON'T)

Cycle	Day	Scheduled Time (h)	Vital Signs[b,n,o]	ECG-12 lead[c]	RO6958688 PK Sampling[d]	Atezolizumab PK Sampling[e]	PD Blood Flow Cytometry[m]	PD Blood Cytokines[f]	RO6958688 Anti-Drug Antibody (ADA)[g]	Atezolizumab Anti-Drug Antibody (ADA)[g]	Tumor Biopsy[h]	FDG PET[i]	TCR Vβ[l]
Cycle 2	Day 15	Pre-infusion	x	X	x	x		x	x				
		End of RO6958688 infusion			x			x					
Cycle 3	Day 1	Pre-infusion[j]	x	X	x	x	X	x	x	x	x		X
		End of RO6958688 infusion			x			x					
	Day 8	Pre-infusion	x	X	x				x				
		End of RO6958688 infusion			x								
	Day 15	Pre-infusion	x	X	x				x				
		End of RO6958688 infusion			x								
Cycle 4	Day 1	Pre-infusion[j]	x	X	x	x			x	x			
		End of RO6958688 infusion			x								

"Use of this document is governed by the terms of use on the first page of this document."

TABLE A2: SCHEDULE OF HOURLY ASSESSMENTS: QW RO6958688 ADMINISTRATION SCHEDULE (CON'T)

Cycle	Day	Scheduled Time (h)	Vital Signs[b,n,o]	ECG-12 lead[c]	RO6958688 PK Sampling[d]	Atezolizumab PK Sampling[e]	PD Blood Flow Cytometry[m]	PD Blood Cytokines[f]	RO6958688 Anti-Drug Antibody (ADA)[g]	Atezolizumab Anti-Drug Antibody (ADA)[g]	Tumor Biopsy[h]	FDG PET[i]	TCR Vβ[l]
Cycle 4	Day 8	Pre-infusion	x	X	x				x				
		End of RO6958688 infusion			x								
	Day 15	Pre-infusion	x	X	x				x				
		End of RO6958688 infusion			x								
Cycle 5	Day 1	Pre-infusion[j]	x	X	x				x				
		End of RO6958688 infusion			x								
	Day 8	Pre-infusion	x	X	x				x				
		End of RO6958688 infusion			x								
	Day 15	Pre-infusion	x	X	x				x				
		End of RO6958688 infusion			x								

"Use of this document is governed by the terms of use on the first page of this document."

Appendix 1
Schedule of Assessments (cont.)

TABLE A2: SCHEDULE OF HOURLY ASSESSMENTS: QW RO6958688 ADMINISTRATION SCHEDULE (CON'T)

Cycle	Day	Scheduled Time (h)	Vital Signs[b,n,o]	ECG-12 lead[c]	RO6958688 PK Sampling[d]	Atezolizumab PK Sampling[e]	PD Blood Flow Cytometry[m]	PD Blood Cytokines[f]	RO6958688 Anti-Drug Antibody (ADA)[g]	Atezolizumab Anti-Drug Antibody (ADA)[g]	Tumor Biopsy[h]	FDG PET[i]	TCR Vβ[l]
Cycle 6	Day 1	Pre-infusion[j]	x	X	x	x			x				
		End of RO6958688 infusion			x								
	Day 8	Pre-infusion	x	X	x				x			x	
		End of RO6958688 infusion			x								
	Day 15	Pre-infusion	x	X	x				x				
		End of RO6958688 infusion			x								
Cycle 7 to Cycle 10	Day 1	Pre-infusion[j]	x	X	x	x[k]			x	x[k]			
		End of RO6958688 infusion			x								
	Day 8	Pre-infusion	x	X	x				x				
		End of RO6958688 infusion			x								

"Use of this document is governed by the terms of use on the first page of this document."

TABLE A2: SCHEDULE OF HOURLY ASSESSMENTS: QW RO6958688 ADMINISTRATION SCHEDULE (CON'T)

Cycle	Day	Scheduled Time (h)	Vital Signs[b,n,o]	ECG-12 lead[c]	RO6958688 PK Sampling[d]	Atezolizumab PK Sampling[e]	PD Blood Flow Cytometry[m]	PD Blood Cytokines[f]	RO6958688 Anti-Drug Antibody (ADA)[g]	Atezolizumab Anti-Drug Antibody (ADA)[g]	Tumor Biopsy[h]	FDG PET[i]	TCR Vβ[l]
Cycle 7 to Cycle 10	Day 15	Pre-infusion	x	X	x				x				
		End of RO6958688 infusion			x								
Cycle 11 and every 2nd cycle	Day 1	Pre-infusion	x	x	x				x				
		End of RO6958688 infusion			x								
End of treatment	Anytime		x	X	x	x			x	x			
28-Day Safety Follow-up	Anytime		x	X	x	x			x				

"Use of this document is governed by the terms of use on the first page of this document."

TABLE A2: SCHEDULE OF HOURLY ASSESSMENTS: QW RO6958688 ADMINISTRATION SCHEDULE (CON'T)

ADA = anti-drug antibody; CEA = carcinoembryonic antigen; D = day; ECOG = Eastern Cooperative Oncology Group; PD = pharmacodynamics; PK = pharmacokinetic.

[a] Screening assessments should be performed between D-28 and D-1, unless specified.

[b] The patient's vital signs (heart rate, respiratory rate, blood pressure, oxygen saturation and temperature) should be determined up to 60 (± 10) minutes before each atezolizumab infusion. Vital signs should also be obtained during or after the atezolizumab infusion if clinically indicated. Vital signs (including supine blood pressure and heart rate) will be monitored on Day 1 RO6958688 pre-infusion, every 15 minutes until the end of RO6958688 infusion and, thereafter, every 30 minutes until the infusion line is removed. Starting Cycle 1 Day 15: RO6958688 pre-infusion, every 30 minutes during RO6958688 infusion and every 30 minutes after the end of RO6958688 infusion until infusion line is removed. From Cycle 3 onwards vital signs will only be obtained on Day 1 RO6958688 pre-infusion. For the purposes of the eCRF vital signs will only be captured pre-infusion and in case of abnormalities

[c] Triplicate 12-lead ECG at screening (within 7 days before first dose of RO6958688) pre- and end of infusion on Cycle 1 Day 1 and Cycle 1 Day 15 and at the 28-day safety follow-up visit. Pre-infusion at all other study drug administrations. Additional unscheduled ECG assessments should be performed in case of abnormalities and if clinical symptoms occur. Recording must be done prior to PK sampling.

[d] All RO6958688 PK sampling Timepoints are reference from the start of RO6958688 infusion. PK time windows: Pre-infusion: up to – 4 h - EOI: up to + 30 min - 2 h post EOI: +/- 30 min - 24 h: +/- 2 h - 48 h: +/- 4 h - Later: +/- 12 h.

[e] All atezolizumab PK sampling Timepoints are reference from the start of atezolizumab infusion.

[f] Serum or plasma samples for the assessment of cytokine (PD blood cytokines) release will be collected. At the time of an IRR please see IRR SoA Table A3.

[g] Additional samples will be drawn at the time of an IRR, see IRR SoA Table A3 or hypersensitivity reaction, treatment discontinuation, *and* at the 28-day safety follow-up visit (RO6958688 only). A PK sample will be taken at the time of ADA sampling.

[h] Mandatory tumor biopsy samples will be collected on two occasions (once at baseline and once during the study treatment period), except for NSCLC patients for whom there is no accessible lesion. If preliminary data suggest that modification of the on-treatment tumor biopsy timepoint would be more appropriate, alternative on-treatment tumor biopsy timepoints could be considered in the future cohorts.

[i] FDG-PET should be done at baseline (Day-14 to D-1) but before the baseline biopsy. The on-treatment FDG-PET scan must be done at week 4 (C2D8 +1 week) and at week 16 (C6D8 pre-dose and up to 72h before the visit) in case of dose delay the FDG-PET should be obtained at week 4 (+1week) and week 16. If preliminary data suggest that modification of the on-treatment FDG-PET timepoint would be more appropriate, alternative on-treatment FDG PET timepoints could be considered in the future cohorts.

[j] Pre-infusion timepoints relate to RO6958688 or atezolizumab. On Cycle 1, pre-infusion PK samples (for both RO6958688 and atezolizumab) can be taken prior to both infusions. On Cycle 2 and beyond, pre-dose PK samples can be taken up to 1-hour before infusion start for atezolizumab; after atezolizumab end of infusion and before the start of infusion RO6958688 for the RO6958688 pre-dose sample.

[k] Atezolizumab PK and ADA sampling to be drawn at Cycle 8 Day 1 then every 8 cycles from this timepoint onwards.

"Use of this document is governed by the terms of use on the first page of this document."

TABLE A2: SCHEDULE OF HOURLY ASSESSMENTS: QW RO6958688 ADMINISTRATION SCHEDULE (CON'T)

[l] For TCR Vβ, at each time point Cycle 1 Day 1 and Cycle 3 Day 1, two whole blood samples should be drawn. Samples should be taken prior to IMP administration.

[m] Blood Flow Cytometry sample to be drawn pre-dose at C1D1, C1D8, C2D1, and C3D1.

[n] If a patient has experienced a Grade 3 IRR/CRS event during the previous treatment administration visit, the patient should be hospitalized for at least 24 hours after the end of infusion at next treatment administration visit during which vital signs will be monitored as follows: every 30 (± 10) minutes for the first hour post infusion, every 60 (± 15) minutes during the following 3 hours, every 120 (± 20) minutes for the next 8 hours and every 240 (± 30) minutes for the remaining 12 hours.

[o] If a patient experiences a Grade 3 or higher treatment-related adverse event (with the exception of IRR/CRS) within the 24 hours period following the previous infusion, the patient should be observed for at least 8 hours after the end of RO6958688 infusion at the next administration visit during which vital signs will be monitored as follows: every 30 (± 10) minutes for the first hour post infusion, every 60 (± 15) minutes during the following 3 hours, every 120 (± 20) minutes for the next 4 hours.

"Use of this document is governed by the terms of use on the first page of this document."

TABLE A3: SCHEDULE OF ASSESSMENTS FOR INFUSION-RELATED REACTION

Cycle	Day	Scheduled Time (h)	PK Sample	PD Blood Cytokines[a]	IgE and Tryptase		Hematology	Blood Chemistry	Coagulation
IRR	Unscheduled	Anytime	x	x	x		x	x	x

ADA = anti-drug antibody; h = hour; IRR = infusion-related reaction; PD = pharmacodynamics; PK = pharmacokinetic.

[a] At the time of an IRR (including repetitive occurrence of IRR) or hypersensitivity reaction, unscheduled samples will be collected. Cytokine analysis will include but not be limited to TNF-a, IL-6, and IFNγ. For patients who experience an IRR $\geq$ Grade 2, tryptase and total IgE will be analyzed.

“Use of this document is governed by the terms of use on the first page of this document.”

Appendix 1
Schedule of Assessments (cont.)

TABLE A4: SCHEDULE OF ASSESSMENTS: Q3W RO6958688 ADMINISTRATION SCHEDULE

Cycle	Screening	Cycle 1					Cycle 2					Cycle 3			Cycle 4		Cycle 5		Cycle 6		Cycle 7 onwards	End of treatment	28-Day Safety Follow Up[m]
Day	D-28 to D1	Day 1	Day 2	Day 3	Day 8	Day 15	Day 1	Day 2	Day 3	Day 8	Day 15	Day 1	Day 8	Day 15	Day 1	Day 8	Day 1	Day 8	Day 1	Day 8	Day 1		
Assessments[a]																							
Informed Consent[b]	x																						
Eligibility	x																						
Demography	x																						
Medical History	x																						
Physical Examination[c]	x	x			x	x	X		x	x	x	x	x	x	x	x	x	x	X	x	x	x	x
Vital Signs	x	x	x	x	x	x	X	x	x	x	x	x	x	x	x	x	x	x	X	x	x[p]	x[p]	x
Administration of atezolizumab Q3W[x]		x[z]					X					x			x		x		X		x		
Administration of RO6958688 Q3W[y]		x[z]					X					x			x		x		X		x[ae]		
ECG-12 lead[d]	x	x			x	x	X			x	x	x	x	x	x	x	x	x	X	x	x	x	x
ECOG Performance Status	x	x			x	x	X		x	x	x	x	x	x	x	x	x	x	X	x	x[q]	x[q]	x
Pulmonary Function test[aj]	x																						
Hematology[g]	x	x	x	x	x	x	X	x	X	x	x	x	x	x	x	x	x	x	X	x	x[r]	x[r]	x
Blood Chemistry[g,aa]	x	x	x	x	x	x	X	x	X	x	x	x	x	x	x	x	x	x	X	x	x[r]	x[r]	x
Serum Thyroid-Stimulating Hormone freeT3-T4[l]		x															x					x	x
Coagulation[g,h]	x	x	x	x	x	x	X	x	X	x	x	x	x	x	x	x	x	x	X	x	x[r]	x[r]	x
Urinalysis[g]	x	x	x	x	x	x	X	x	X	x	x	x	x	x	x	x	x	x	X	x	x[r]	x[r]	x
HBV and HCV Serology	x																						
Auto-Antibody Panel[v]	x						X								x				X		x[w]		
Pregnancy Test[e]	x	X																				x	x

"Use of this document is governed by the terms of use on the first page of this document."

Appendix 1
Schedule of Assessments (cont.)

TABLE A4: SCHEDULE OF ASSESSMENTS Q3W RO6958688 ADMINISTRATION SCHEDULE (CONT.)

Cycle	Screening	Cycle 1					Cycle 2					Cycle 3			Cycle 4		Cycle 5		Cycle 6		Cycle 7 onwards	End of treatment	28-Day Safety Follow Up[m]
Day	D-28 to D1	Day 1	Day 2	Day 3	Day 8	Day 15	Day 1	Day 2	Day 3	Day 8	Day 15	Day 1	Day 8	Day 15	Day 1	Day 8	Day 1	Day 8	Day 1	Day 8	Day 1		
Atezolizumab PK Sampling[ab]		x			x	x	x			x	x	x			x				X		x[af]	x	x
RO6958688 PK Sampling[ab]		x	x	x	x	x	x	X	x	x	x	x	x	x	x	x	X	x	X	x	x[t]	x	x
Atezolizumab Anti-Drug Antibody (ADA)[ac]		x					x					x			x						x[af]	x	
RO6958688 Anti-Drug Antibody (ADA)[ac]		x			x	x	x			x	x	x	x	x	x		X		X		x[u]	x	x
PD Blood Flow Cytometry		x	x		x		x					x											
PD Blood Cytokines[f]		x	x	x	x		x	X	x	x		x	x										
Archival Tumor Biopsy	x																						
Fresh Tumor Biopsy[j]	x						x					x											
Clinical Genotyping[ag]		x																					
RCR Sample (DNA and RNA) [ag]		x																					
CEA confirmation on Archival Tissue[k]	x																						
FDG-PET[n]	x[s]									x[s]										x[s]			
TCR Vβ[ah]		x										x											
Sol CEA[ai]		x					x					x											

"Use of this document is governed by the terms of use on the first page of this document."

TABLE A4: SCHEDULE OF ASSESSMENTS Q3W RO6958688 ADMINISTRATION SCHEDULE (CONT.)

Cycle	Screening	Cycle 1					Cycle 2					Cycle 3			Cycle 4		Cycle 5		Cycle 6		Cycle 7 onwards	End of treatment	28-Day Safety Follow Up[m]
Tumor Assessment[l]	x													x						x		x	
Tumor Growth Kinetic[ad]	x																						
Adverse Events	X																						
Previous and Concomitant Treatments	X																						

"Use of this document is governed by the terms of use on the first page of this document."

TABLE A4: SCHEDULE OF ASSESSMENTS Q3W RO6958688 ADMINISTRATION SCHEDULE (CONT.)

ADA = anti-drug antibody; CEA = carcinoembryonic antigen; D = day; ECOG = Eastern Cooperative Oncology Group; PD = pharmacodynamics; PK = pharmacokinetic.

[a] All visit and their assigned safety assessments (hematology, blood chemistry, coagulation, urinalysis, physical examination), unless otherwise indicated should occur within a 3-day time window.

[b] Informed consent must be obtained before any study-specific procedures. All patients will be tested for HBV and HCV serology. Patients with HIV infection, or active hepatitis B (chronic or acute), or active hepatitis C infection are not eligible.

[c] Physical examinations (including weight) and ECOG Performance Status will be done at screening, at the time of treatment administration, at the EOT and at the 28-day safety follow-up visits. Results must be obtained prior to infusion.

[d] Triplicate 12-lead ECG at screening (within 7 days before first dose of RO6958688) pre- and end of infusion on Cycle 1 Day 1. Pre-infusion at all other study drug administrations and at EOT and the 28-day safety follow-up visit Additional unscheduled ECG assessments should be performed in case of abnormalities and if clinical symptoms occur. Recording must be done prior to PK sampling.

[e] Serum pregnancy test at screening, within 7 days prior to first dose. From the date of first serum pregnancy test at screening, a urine pregnancy test must be performed every 4 weeks. Also to be performed at study termination and safety follow-up visit (28 days post last infusion).

[f] Serum or plasma samples for the assessment of cytokine (PD blood cytokines) release will be collected. At the time of an IRR, please see IRR SoA Table A3.

[g] Hematology, blood chemistry, coagulation, and urinalysis can be performed up to 24-h (72-h if during weekend) hours prior to scheduled dosing. Results must be obtained prior to infusion. Soluble CEA will be measured by the site as part of the blood chemistry measurements on Cycle 1 Day 1 and every 6 weeks thereafter for patients who continue treatment and at the end of treatment visit. Soluble CD25 is to be obtained at screening and during the treatment period at Day 1 of every cycle (samples can be drawn 7 days prior to Day 1 of each cycle). Before IMP administration on Day 1 of each cycle, the investigator should confirm that the result of soluble CD25 measurement from the previous week or if available from the same day is not significantly high. If there is a significantly high level, the investigator should discuss with the Medical Monitor if the patient can or cannot receive further IMP administration. Ferritin should be obtained at screening and during the treatment period: Day 1 of each cycle (-2 days).

[h] For coagulation (including PT/INR and PTT), an additional sample will be taken at the time of an IRR or hypersensitivity reaction, see IRR SoA Table A3. Additional coagulation parameters (i.e., first chromogenic antithrombin III then antigenic antithrombin III in case of chromogenic antithrombin III decrease, fibrinogen, prothrombin time, fibrin degradation products, D-dimer) could be assessed according to clinical judgment.

[i] Serum TSH, free T3 (or total T3 for sites where free T3 is not performed) and T4 levels will be assessed at baseline pre-dose at Cycle 1, on study (every 3 months) and at study completion/early termination visit and safety follow-up visit (28 days after last dose of study drug).

“Use of this document is governed by the terms of use on the first page of this document.”

Appendix 1
Schedule of Assessments (cont.)

TABLE A4: SCHEDULE OF ASSESSMENTS Q3W RO6958688 ADMINISTRATION SCHEDULE (CONT.)

[j] Mandatory tumor biopsy samples will be collected on two occasions (once at baseline and once during the study treatment period), except for NSCLC patients for whom there is no accessible lesion. On treatment biopsies will be collected as per Section 3.3.2.2. If preliminary data suggest that modification of the on-treatment tumor biopsy timepoint would be more appropriate, alternative on-treatment tumor biopsy timepoints could be considered in the future cohorts. Unscheduled tumor biopsies can be taken at any time point, if considered necessary for tumor assessment and patient management as decided by the investigator.

[k] CEA expression for eligibility will be confirmed centrally for U.S. patients on archival tumor material (refer to inclusion criterion #13), if available, or confirmed from a freshly obtained tumor biopsy. Results available prior to screening (outside the 28-day window) can be used.

[l] Tumor assessments will be performed at screening, then every 8 weeks after Cycle 1 Day 1 for the first year, and thereafter every 12 weeks for the second year until disease progression or treatment discontinuation. Optional latest pre-study CT scan should be provided for assessment of tumor growth kinetics within 6 weeks of patient entering the study. For the scheduled tumor assessments beyond Screening a (+/- 7-day window is permitted).

[m] Patients who complete the study or discontinue from the study early will be asked to return to the clinic 28 days after the last dose of study drugs for post-study follow-up visits.

[n] FDG-PET should be done at baseline (Day-14 to D-1) but before the baseline biopsy. The on-treatment FDG-PET scan must be done at week 4 (C2D8 + 1 week) and at week 16 (C6D8 and up to 72h before the visit) in case of dose delay the FDG-PET should be obtained at week 4 (+1week) and week 16. If preliminary data suggest that modification of the on-treatment FDG-PET timepoint would be more appropriate, alternative on-treatment FDG-PET timepoints could be considered in the future cohorts. Please refer to Appendix 9 for further information.

[o] At least 24 hours (overnight) hospital stay required following administration of study drug at Cycle 1 Day 1.

[p] Vital signs to be assessed at D1 from cycle 7 onwards.

[q] ECOG to be assessed at D1 from cycle 7 onwards.

[r] Hematology, blood chemistry, coagulation and urinalysis (to be drawn at D1 from cycle 7 onwards) can be performed up to 24 hours (72h if during weekend) prior to scheduled dosing. Results must be obtained prior to atezolizumab and RO6958688 infusion. Before each IMP administration on Day 1 or each cycle, the investigator should confirm that the result of soluble CD25 measurement from the previous week or if available from the same day is not significantly high. If there is a significantly high level, the investigator should discuss with the Medical Monitor if the patient can or cannot receive further IMP administration.

[s] For FDG PET assessment, body weight, last calorific meal information, glucose level are needed (see Appendix 9 of the protocol for more information).

[t] RO6958688 PK sampling to be drawn preinfusion and at end of infusion at D1 of each cycle from Cycle 7 to Cycle 10. At Cycle 11 and every second cycle, RO6958688 PK samples to be drawn preinfusion and at end of infusion on D1.

[u] RO6958688 ADA sampling to be drawn preinfusion at D1 of each cycle from Cycle 7 to Cycle 10. At Cycle 11 and every second cycle, RO6958688 ADA samples to be drawn preinfusion on Day 1 at the mentioned timepoints.

"Use of this document is governed by the terms of use on the first page of this document."

Appendix 1
Schedule of Assessments (cont.)

TABLE A4: SCHEDULE OF ASSESSMENTS Q3W RO6958688 ADMINISTRATION SCHEDULE (CONT.)

[v] In patients who develop signs and/or symptoms suggestive of auto-immune disease while on treatment, the antibody panel should be repeated. The autoantibody panel will be assessed at screening, pre-dose every second cycle, and every 3 months after the third month on therapy (samples can be drawn up to 1 week before Day 1 visit).

[w] To be performed every 3 months after Cycle 6 (ie. Cycle 10, Cycle 14…).

[x] Atezolizumab will be delivered over 60 (+/-15) minutes. If the first infusion is tolerated without infusion associated adverse events, the second infusion may be delivered over 30 (+/-10) minutes. If the 30-minute infusion is well tolerated, all subsequent infusions may be delivered over 30 (+/-10) minutes.

[y] The start of the RO6958688 infusion on Day 1 of each cycle should be after the end of the atezolizumab infusion as per Section 4.3.2.3.

[z] Prophylactic corticosteroids to be administered at C1D1 as per Table 2. The use of analgesics and/or antihistamines is allowed to minimize expected flu like symptoms prior to the first RO6958688 administration.

[aa] Serum CEA will also be measured as a disease monitoring marker (as part of the serum chemistry panel).

[ab] Blood for PK should always be drawn at the indicated time point.

[ac] Additional samples will be drawn at the time of an IRR, see IRR SoA Table A3 or hypersensitivity reaction, treatment discontinuation, and at the 28-day safety follow-up visit (RO6958688 only). A PK sample will be taken at the time of ADA sampling.

[ad] An exploratory assessment of tumor growth kinetics will be made by comparing post-treatment scans with at least 2 pre-treatment scans not older than 12 weeks prior to C1D1, if available. The two pre-treatment scans will consist of a pre-treatment scan (if available) and the study baseline scan, and will allow estimation of tumor growth rate before start of treatment.

[ae] Administration of RO6958688 to be done at D1 from Cycle 7 onwards.

[af] Atezolizumab PK and ADA sampling to be drawn at Cycle 8 Day 1 then every 8 cycles from this timepoint onwards.

[ag] Clinical genotyping and RCR (DNA and RNA) samples to be drawn before any IMP administration.

[ah] For TCR Vβ, at each time point Cycle 1 Day 1 and Cycle 3 Day 1, 2 whole blood samples should be drawn. Samples should be taken prior to IMP administration

[ai] For soluble CEA central assessment: samples will be collected pre-dose at: C1D1 (baseline), C2D1 and C3D1.

[aj] Evaluate DLCO (corrected for both alveolar volume and hemoglobin) and FEV1/VC/TLC at screening for patients with bilateral lung metastases or patients with lobectomy or pneumonectomy with lung metastases in the remaining lung. Additional tests may be performed if clinically indicated.

"Use of this document is governed by the terms of use on the first page of this document."

Appendix 1
Schedule of Assessments (cont.)

TABLE A5: SCHEDULE OF HOURLY ASSESSMENTS Q3W RO6958688 ADMINISTRATION SCHEDULE (CONT.)

Cycle	Day	Scheduled Time (h)	Vital signs[b,n,o]	ECG-12 lead[c]	RO6958688 PK Sampling[d]	Atezolizumab PK Sampling[e]	RO6958688 Anti-Drug Antibody (ADA)[g]	Atezolizumab Anti-Drug Antibody (ADA)[g]	PD Blood Flow Cytometry[m]	PD Blood Cytokines[f]	Tumor Biopsy[h]	FDG-PET[i]	TCR Vβ[l]
Screening	D-28 to D1[a]	Anytime	x	x							x	x	
Cycle 1	Day 1	Pre-infusion[j]	x	x	x	x	x	x	x	x			x
		0.5h (+/- 10 min) post atezolizumab EOI				x							
		RO6958688 EOI	x	x	x					x			
		2 hours post RO6958688 EOI			x					x			
		4 hours post RO6958688 EOI								x			
	Day 2	24 hours	x		x				x	x			
	Day 3	48 hours	x		x					x			
	Day 8	168 hours	x	x	x	x	x		x	x			
	Day 15	336 hours	x	x	x	x	x						
Cycle 2	Day 1	Pre-infusion[j]	x	x	x	x	x	x	x	x	x		
		0.5h (+/- 10 min) post atezolizumab EOI				x							
		RO6958688 EOI	x		x					x			
		2 hours post RO6958688 EOI			x					x			
	Day 2	24 hours	x		x					x			
	Day 3	48 hours	x		x					x			
	Day 8	168 hours	x	x	x	x	x			x		x	
	Day 15	336 hours	x	x	x	x	x						

"Use of this document is governed by the terms of use on the first page of this document."

Appendix 1
Schedule of Assessments (cont.)

TABLE A5: SCHEDULE OF HOURLY ASSESSMENTS Q3W RO6958688 ADMINISTRATION SCHEDULE (CONT.)

Cycle	Day	Scheduled Time (h)	Vital signs[b,n,o]	ECG-12 lead[c]	RO6958688 PK Sampling[d]	Atezolizumab PK Sampling[e]	RO6958688 Anti-Drug Antibody (ADA)[g]	Atezolizumab Anti-Drug Antibody (ADA)[g]	PD Blood Flow Cytometry[m]	PD Blood Cytokines[f]	Tumor Biopsy[h]	FDG-PET[i]	TCR Vβ[l]
Cycle 3	Day 1	Pre-infusion[j]	x	x	x	x	X	x	X	x	x		x
		RO6958688 EOI[j]			x					x			
	Day 8	168 hours	x	x	x		X			x			
	Day 15	336 hours	x	x	x		X						
Cycle 4	Day 1	Pre-infusion[j]	x	x	x	x	X	x					
		RO6958688 EOI[j]			x								
	Day 8	168 hours	x	x	x								
Cycle 5	Day 1	Pre-infusion[j]	x	x	x		X						
		RO6958688 EOI[j]			x								
	Day 8	168 hours	x	x	x								
Cycle 6	Day 1	Pre-infusion[j]	x	x	x	x	X						
		RO6958688 EOI[j]			x								
	Day 8	168 hours	x	x	x							X	
Cycle 7 to Cycle 10	Day 1	Pre-infusion[j]	x	x	x	x[k]	X	x[k]					
		RO6958688 EOI[j]			x								
Cycle 11 and every 2nd cycle	Day 1	Pre-infusion[j]	x	x	x		x						
		RO6958688 EOI[j]			x								
End of treatment	Anytime		x	x	x	x	X	x					
28-day Safety Follow Up	Anytime		x	x	x	x	X						

"Use of this document is governed by the terms of use on the first page of this document."

TABLE A5: SCHEDULE OF HOURLY ASSESSMENTS Q3W RO6958688 ADMINISTRATION SCHEDULE (CONT.)

ADA = anti-drug antibody; CEA = carcinoembryonic antigen; D = day; ECOG = Eastern Cooperative Oncology Group; PD = pharmacodynamics; PK = pharmacokinetic.

[a] Screening assessments should be performed between D-28 and D-1, unless specified.

[b] The patient's vital signs (heart rate, respiratory rate, blood pressure, oxygen saturation and temperature) should be determined up to 60 (± 10) minutes before each atezolizumab infusion. Vital signs should also be obtained during or after the atezolizumab infusion if clinically indicated. Vital signs (including supine blood pressure and heart rate) will be monitored on Day 1 RO6958688 pre-infusion, every 15 minutes until the end of RO6958688 infusion and, thereafter, every 30 minutes until the infusion line is removed. Starting Cycle 2 Day 1: RO6958688 pre-infusion, every 30 minutes during RO6958688 infusion and every 30 minutes after the end of RO6958688 infusion until infusion line is removed. From Cycle 3 onwards vital signs will only be obtained on Day 1 RO6958688 pre-infusion. For the purposes of the eCRF vital signs will only be captured pre-infusion and in case of abnormalities

[c] Triplicate 12-lead ECG at screening (within 7 days before first dose of RO6958688) pre- and end of infusion on Cycle 1 Day 1. Pre-infusion at all other study drug administrations and at EOT and at 28-day safety follow up visit. Additional unscheduled ECG assessments should be performed in case of abnormalities and if clinical symptoms occur. Recording must be done prior to PK sampling.

[d] All RO6958688 PK sampling Timepoints are reference from the start of RO6958688 infusion. PK time windows: Pre-infusion: up to – 4 h - EOI: up to + 30 min - 2 h post EOI: +/- 30 min - 24 h: +/- 2 h - 48 h: +/- 4 h - Later: +/- 12 h.

[e] All atezolizumab PK sampling Timepoints are reference from the start of atezolizumab infusion.

[f] Serum or plasma samples for the assessment of cytokine (PD blood cytokines) release will be collected. At the time of an IRR please see IRR SoA Table A3.

[g] Additional samples will be drawn at the time of an IRR, see IRR SoA Table A3 or hypersensitivity reaction, treatment discontinuation, *and* at the 28-day safety follow-up (RO6958688 only). A PK sample will be taken at the time of ADA sampling.

[h] Mandatory tumor biopsy samples will be collected on two occasions (once at baseline and once during the study treatment period), except for NSCLC patients for whom there is no accessible lesion. If preliminary data suggest that modification of the on-treatment tumor biopsy timepoint would be more appropriate, alternative on-treatment tumor biopsy timepoints could be considered in the future cohorts.

[i] FDG-PET should be done at baseline (Day-14 to D-1) but before the baseline biopsy. The on-treatment FDG-PET scan must be done at week 4 (C2D81+ 1 week) and at week 16 (C6D8and up to 72h before the visit) in case of dose delay the FDG-PET should be obtained at week 4 (+1week) and week 16. If preliminary data suggest that modification of the on-treatment FDG-PET timepoint would be more appropriate, alternative on-treatment FDG-PET timepoints could be considered in the future cohorts.

[j] Pre-infusion timepoints relate to RO6958688 or atezolizumab. Pre-dose PK samples can be taken up to 1-hour before infusion start for atezolizumab; after atezolizumab end of infusion and/or before the start of RO6958688 infusion for the RO6958688 pre-dose sample.

[k] Atezolizumab PK and ADA sampling to be drawn at Cycle 8 Day 1 then every 8 cycles from this timepoint onwards.

[l] For TCR Vβ, at each time point Cycle 1 Day 1 and Cycle 3 Day 1, 2 whole blood samples should be drawn. Samples should be taken prior to IMP administration.

"Use of this document is governed by the terms of use on the first page of this document."

TABLE A5: SCHEDULE OF HOURLY ASSESSMENTS Q3W RO6958688 ADMINISTRATION SCHEDULE (CONT.)

[m] Blood Flow Cytometry sample to be drawn pre-dose at C1D1, C1D8, C2D1, and C3D1.

[n] If a patient has experienced a Grade 3 IRR/CRS event during the previous treatment administration visit, the patient should be hospitalized for at least 24 hours after the end of infusion at next treatment administration visit during which vital signs will be monitored as follows: every 30 (± 10) minutes for the first hour post infusion, every 60 (± 15) minutes during the following 3 hours, every 120 (± 20) minutes for the next 8 hours and every 240 (± 30) minutes for the remaining 12 hours.

[o] If a patient experiences a Grade 3 or higher treatment-related adverse event (with the exception of IRR/CRS) within the 24 hours period following the previous infusion, the patient should be observed for at least 8 hours after the end of RO6958688 infusion at the next administration visit during which vital signs will be monitored as follows: every 30 (± 10) minutes for the first hour post infusion, every 60 (± 15) minutes during the following 3 hours, every 120 (± 20) minutes for the next 4 hours.

“Use of this document is governed by the terms of use on the first page of this document.”

Appendix 1
Schedule of Assessments (cont.)

TABLE A6: SCHEDULE OF ASSESSMENTS: COHORTS C & SAFETY RO6958688 ADMINISTRATION SCHEDULE

Cycle	Screening	Cycle 1							Cycle 2			Cycle 3			Cycle 4		Cycle 5		Cycle 6		Cycle 7 onwards	End of treatment	28-Day Safety Follow Up[m]
Day	D-28 to D1	Day 1	Day 2	Day 3	Day 8	Day 9	Day 15	Day 16	Day 1	Day 2	Day 8	Day 1	Day 2	Day 15	Day 1	Day 2	Day 1	Day 2	Day 1	Day 8	Day 1		
Assessments[a]																							
Informed Consent[b]	x																						
Eligibility	x																						
Demography	x																						
Medical History	x																						
Physical Examination[c]	x	x			x		x		x		x	x		x	x		x		x	x	x	x	x
Vital Signs	x	x	x	x	x	x	x	x	x	x	x	x	x	x	x	x	x	x	x	x	x[p]	x[p]	x
Administration of atezolizumab Q3W[x]		x[z]							x			x			x		x		x		x		
Administration of RO6958688 QW - Q3W[y]		x[z]			x		x		x			x			x		x		x		x[ae]		
ECG-12 lead[d]	x	x			x		x		x			x		x	x		x		x	x	x	x	x
ECOG Performance Status	x	x			x		x		x		x	x		x	x		x		x	x	x[q]	x[q]	x
Pulmonary Function test[al]	x																						
Hematology[g]	x	x	x	x	x	x	x	x	x	x	x	x	x	x	x	x	x	x	x	x	x[r]	x[r]	x
Blood Chemistry[g,aa]	x	x	x	x	x	x	x	x	x	x	x	x	x	x	x	x	x	x	x	x	x[r]	x[r]	x
Serum Thyroid-Stimulating Hormone freeT3-T4[l]		x															x					x	x
Coagulation[g,h]	x	x	x	x	x	x	x	x	x	x	x	x	x	x	x	x	x	x	x	x	x[r]	x[r]	x
Urinalysis[g]	x	x	x	x	x	x	x	x	x	x	x	x	x	x	x	x	x	x	x	x	x[r]	x[r]	x

"Use of this document is governed by the terms of use on the first page of this document."

Appendix 1
Schedule of Assessments (cont.)

TABLE A6: SCHEDULE OF ASSESSMENTS COHORTS C & SAFETY RO6958688 ADMINISTRATION SCHEDULE (CONT.)

Cycle	Screening	Cycle 1							Cycle 2			Cycle 3			Cycle 4		Cycle 5		Cycle 6		Cycle 7 onwards	End of treatment	28-Day Safety Follow Up[m]
Day	D-28 to D1	Day 1	Day 2	Day 3	Day 8	Day 9	Day 15	Day 16	Day 1	Day 2	Day 8	Day 1	Day 2	Day 15	Day 1	Day 2	Day 1	Day 2	Day 1	Day 8	Day 1		
Serology	x																						
Auto-Antibody Panel[v]	x								x						x				x		x[w]		
Pregnancy Test[e]	x	X																				x	x
Atezolizumab PK Sampling[ab]		x			x	x	x	x	x			X			x				x		x[af]	x	x
RO6958688 PK Sampling[ab]		x	x	x	x	x	x	x	x	x	x	X	x	x	x	x	x	x	x	X	x[t]	x	x
Atezolizumab Anti-Drug Antibody (ADA)[ac]		x							x			X			x						x[af]	x	
RO6958688 Anti-Drug Antibody (ADA)[ac]		x			x		x		x		x	X	x	x	x		x		x		x[u]	x	x
PD Blood Flow Cytometry		x	x		x				x			X											
PD Blood Cytokines[f]		x	x	x	x	x	x	x	x	x		X	x										
Archival Tumor Biopsy	x																						
Fresh Tumor Biopsy[j]	x								x			X											
Clinical Genotyping[ag]		x																					
RCR Sample (DNA and DNA) [ag]		x																					
CEA confirmation on Archival Tissue[k]	x																						
FDG-PET[n]	x[s]										x[s]									x[s]			
TCR Vβ[ah]		x										X											
Sol CEA[ai]		x							x			X											

"Use of this document is governed by the terms of use on the first page of this document."

Appendix 1
Schedule of Assessments (cont.)

TABLE A6: SCHEDULE OF ASSESSMENTS COHORTS C & SAFETY RO6958688 ADMINISTRATION SCHEDULE (CONT.)

Cycle	Screening	Cycle 1							Cycle 2			Cycle 3			Cycle 4		Cycle 5		Cycle 6		Cycle 7 onwards	End of treatment	28-Day Safety Follow Up[m]
Day	D-28 to D1	Day 1	Day 2	Day 3	Day 8	Day 9	Day 15	Day 16	Day 1	Day 2	Day 8	Day 1	Day 2	Day 15	Day 1	Day 2	Day 1	Day 2	Day 1	Day 8	Day 1		
Tumor Assessment[l]	x													x						x		x	
Tumor Growth Kineticad	x																						
Adverse Events	X																						
Previous and Concomitant Treatments	X																						

"Use of this document is governed by the terms of use on the first page of this document."

TABLE A6: SCHEDULE OF ASSESSMENTS COHORTS C & SAFETY RO6958688 ADMINISTRATION SCHEDULE (CONT.)

ADA = anti-drug antibody; CEA = carcinoembryonic antigen; D = day; ECOG = Eastern Cooperative Oncology Group; PD = pharmacodynamics; PK = pharmacokinetic.

a All visit and their assigned safety assessments (hematology, blood chemistry, coagulation, urinalysis, physical examination), unless otherwise indicated should occur within a 3-day time window.

b Informed consent must be obtained before any study-specific procedures. All patients will be tested for HBV and HCV serology. Patients with HIV infection, or active hepatitis B (chronic or acute), or active hepatitis C infection are not eligible.

c Physical examinations (including weight) and ECOG Performance Status will be done at screening, at the time of treatment administration, at the EOT and at the 28-day safety follow-up visits. Results must be obtained prior to infusion.

d Triplicate 12-lead ECG at screening (within 7 days before first dose of RO6958688) pre- and end of infusion on Cycle 1 Day 1 and Cycle 1 Day 15 and at the 28-day safety follow-up visit. Pre-infusion at all other study drug administrations. Additional unscheduled ECG assessments should be performed in case of abnormalities and if clinical symptoms occur. Recording must be done prior to PK sampling.

e Serum pregnancy test at screening, within 7 days prior to first dose. From the date of first serum pregnancy test at screening, a urine pregnancy test must be performed every 4 weeks. Also to be performed at study termination and safety follow-up visit (28 days post last infusion).

f Serum or plasma samples for the assessment of cytokine (PD blood cytokines) release will be collected. At the time of an IRR, please see IRR SoA Table A3.

g Hematology, blood chemistry, coagulation, and urinalysis can be performed up to 24 hours (72h if during weekend) prior to scheduled dosing. Results must be obtained prior to infusion. Soluble CEA will be measured as part of the blood chemistry measurements on Cycle 1 Day 1 and every 6 weeks thereafter for patients who continue treatment and at the end of treatment visit. Soluble CD25 is to be obtained at screening and during the treatment period at Day 1 of every cycle (samples can be drawn 7 days prior to Day 1 of each cycle). Before IMP administration on Day 1 of each cycle, the investigator should confirm that the result of soluble CD25 measurement from the previous week or if available from the same day is not significantly high. If there is a significantly high level, the investigator should discuss with the Medical Monitor if the patient can or cannot receive further IMP administration. Ferritin should be obtained at screening and every week during the treatment period: Day 1, Day 8 and Day 15 of every cycle (-2 days) when applicable.

h For coagulation (including PT/INR and PTT), an additional sample will be taken at the time of an IRR or hypersensitivity reaction, see IRR SoA Table A3. Additional coagulation parameters (i.e., first chromogenic antithrombin III then antigenic antithrombin III in case of chromogenic antithrombin III decrease, f brinogen, prothrombin time, f brin degradation products, D-dimer) could be assessed according to clinical judgment.

i Serum TSH, free T3 (or total T3 for sites where free T3 is not performed) and T4 levels will be assessed at baseline pre-dose at Cycle 1, on study (every 3 months) and at study completion/early termination visit and safety follow-up visit (28 days after last dose of study drug).

j Mandatory tumor biopsy samples will be collected on two occasions (once at baseline and once during the study treatment period), except for NSCLC patients for whom there is no accessible lesion. On treatment biopsies will be collected as per Section 3.3.2.2. If preliminary data suggest that modification of the on-treatment tumor biopsy timepoint would be more appropriate, alternative on-treatment tumor biopsy timepoints could be considered in the future cohorts. Unscheduled tumor biopsies can be taken at any time point, if considered necessary for tumor assessment and patient management as decided by the investigator.

k CEA expression for eligibility will be confirmed centrally for U.S. patients on archival tumor material (refer to inclusion criterion #13), if available, or confirmed from a freshly obtained tumor biopsy. Results available prior to screening (outside the 28 day window) can be used.

l Tumor assessments will be performed at screening, then every 8 weeks after Cycle 1 Day 1 for the first year, and thereafter every 12 weeks for the second year until disease progression or treatment discontinuation. Optional latest pre-study CT scan should be provided for assessment of tumor growth kinetics within 6 weeks of patient entering the study. For the scheduled tumor assessments beyond Screening a (+/- 7-day window is permitted).

m Patients who complete the study or discontinue from the study early will be asked to return to the clinic 28 days after the last dose of study drugs for post-study follow-up visits.

n FDG-PET should be done at baseline (Day-14 to D-1) but before the baseline biopsy. The on-treatment FDG-PET scan must be done at week 4 (C2D8 + 1 week) and at week 16 (C6D8and up to 72h before the visit) in case of dose delay the FDG-PET should be obtained at week 4 (+1week) and week 16. If preliminary data suggest that modification of the on-treatment FDG-PET timepoint would be more appropriate, alternative on-treatment FDG PET timepoints could be considered. Please refer to Appendix 9 for further information.

“Use of this document is governed by the terms of use on the first page of this document.”

TABLE A6: SCHEDULE OF ASSESSMENTS COHORTS C & SAFETY RO6958688 ADMINISTRATION SCHEDULE (CONT.)

o At least 24 hours (overnight) hospital stay required following administration of study drug at Cycle 1 Day 1.

p Vital signs to be assessed at D1 from cycle 7 onwards.

q ECOG to be assessed at D1 from cycle 7 onwards.

r Hematology, blood chemistry, coagulation and urinalysis (to be drawn at D1 from cycle 7 onwards) can be performed up to 24 hours (72h if during weekend) prior to scheduled dosing. Results must be obtained prior to atezolizumab and RO6958688 infusion. Before each IMP administration on Day 1 or each cycle, the investigator should confirm that the result of soluble CD25 measurement from the previous week or if available from the same day is not significantly high. If there is a significantly high level, the investigator should discuss with the Medical Monitor if the patient can or cannot receive further IMP administration.

s For FDG PET assessment, body weight, last calorific meal information, glucose level are needed (see Appendix 9 of the protocol for more information).

t RO6958688 PK sampling to be drawn preinfusion and at end of infusion at D1 of each cycle from Cycle 7 to Cycle 10. At Cycle 11 and every second cycle, RO6958688 PK samples to be drawn preinfusion and at end of infusion on D1.

u RO6958688 ADA sampling to be drawn preinfusion at D1 of each cycle from Cycle 7 to Cycle 10. At Cycle 11 and every second cycle, RO6958688 ADA samples to be drawn preinfusion on Day 1 at the mentioned timepoints.

v In patients who develop signs and/or symptoms suggestive of auto-immune disease while on treatment, the antibody panel should be repeated. The autoantibody panel will be assessed at screening, pre-dose every second cycle, and every 3 months after the third month on therapy (samples can be drawn up to 1 week before Day 1 visit).

w To be performed every 3 months after Cycle 6 (ie. Cycle 10, Cycle 14…).

x Atezolizumab will be delivered over 60 (+/-15) minutes. If the first infusion is tolerated without infusion associated adverse events, the second infusion may be delivered over 30 (+/-10) minutes. If the 30 minute infusion is well tolerated, all subsequent infusions may be delivered over 30 (+/-10) minutes.

y The start of the RO6958688 infusion on Day 1 of each cycle should be after the end of the atezolizumab infusion as per Section 4.3.2.3.

z Prophylactic corticosteroids to be administered at C1D1 as per Table 2. The use of analgesics and/or antihistamines is allowed to minimize expected flu like symptoms prior to the first RO6958688 administration.

aa Serum CEA will also be measured as a disease monitoring marker (as part of the serum chemistry panel).

ab Blood for PK should always be drawn at the indicated time point.

ac Additional samples will be drawn at the time of an IRR, see IRR SoA Table A3 or hypersensitivity reaction, treatment discontinuation, *and* at the 28-day safety follow-up visit (RO6958688 only). A PK sample will be taken at the time of ADA sampling.

ad An exploratory assessment of tumor growth kinetics will be made by comparing post-treatment scans with at least 2 pre-treatment scans not older than 12 weeks prior to C1D1, if available. The two pre-treatment scans will consist of a pre-treatment scan (if available) and the study baseline scan, and will allow estimation of tumor growth rate before start of treatment.

ae Administration of RO6958688 to be done at D1 from Cycle 7 onwards.

af Atezolizumab PK and ADA sampling to be drawn at Cycle 8 Day 1 then every 8 cycles from this timepoint onwards.

ag Clinical genotyping and RCR (DNA and RNA) samples to be drawn before any IMP administration.

ah For TCR Vβ, at each time point Cycle 1 Day 1 and Cycle 3 Day 1, 2 whole blood samples should be drawn. Samples should be taken prior to IMP administration

ai For soluble CEA central assessment: samples will be collected pre-dose at: C1D1 (baseline), C2D1 and C3D1.

aj Evaluate DLCO (corrected for both alveolar volume and hemoglobin) and FEV1/VC/TLC at screening for patients with bilateral lung metastases or patients with lobectomy or pneumonectomy with lung metastases in the remaining lung. Additional tests may be performed if clinically indicated.

“Use of this document is governed by the terms of use on the first page of this document.”

Appendix 1
Schedule of Assessments (cont.)

TABLE A7: SCHEDULE OF HOURLY ASSESSMENTS COHORTS C & SAFETY RO6958688 ADMINISTRATION SCHEDULE

Cycle	Day	Scheduled Time (h)	Vital signs[b,n,o]	ECG-12 lead[c]	RO6958688 PK Sampling[d]	Atezolizumab PK Sampling[e]	RO6958688 Anti-Drug Antibody (ADA)[g]	Atezolizumab Anti-Drug Antibody (ADA)[g]	PD Blood Flow Cytometry	PD Blood Cytokines[f]	Tumor Biopsy[h]	FDG-PET[i]	TCR Vβ[l]
Screening	D-28 to D1[a]	Anytime	x	x					x[o]		x	x	
Cycle 1	Day 1	Pre-infusion[j]	x	x	x	x	x	X	x	x			x
		0.5h (+/- 10 min) post atezolizumab EOI				x							
		RO6958688 EOI	x	x	x					x			
		2 hours post RO6958688 EOI			x					x			
		4 hours post RO6958688 EOI								x			
	Day 2	24 hours	x		x				x	x			
	Day 3	48 hours	x		x					x			
	Day 8	Pre-infusion[j]	x	x	x	x	x		x	x			
		RO6958688 EOI			x								
		2 hours post RO6958688 EOI			x								
	Day 9	192 hours	x		x	x				x			
	Day 15	Pre-infusion[j]	x	x	x	x	x			x			
		RO6958688 EOI		x	x					x			
		2 hours post RO6958688 EOI			x					x			
	Day 16	360 hours	x		x	x				x			

"Use of this document is governed by the terms of use on the first page of this document."

Appendix 1
Schedule of Assessments (cont.)

TABLE A7: SCHEDULE OF HOURLY ASSESSMENTS COHORTS C & SAFETY RO6958688 ADMINISTRATION SCHEDULE (CONT.)

Cycle	Day	Scheduled Time (h)	Vital signs[b,n,o]	ECG-12 lead[c]	RO6958688 PK Sampling[d]	Atezolizumab PK Sampling[e]	RO6958688 Anti-Drug Ant body (ADA)[g]	Atezolizumab Anti-Drug Antibody (ADA)[g]	PD Blood Flow Cytometry	PD Blood Cytokines[f]	Tumor Biopsy[h]	FDG-PET[i]	TCR Vβ[l]
Cycle 2	Day 1	Pre-infusion[j]	x	x	x	x	x	X	x	X	x		
		0.5h (+/- 10 min) post atezolizumab EOI				x							
		RO6958688 EOI	x		x					X			
		2 hours post RO6958688 EOI			x					X			
	Day 2	24 hours	x		x					X			
	Day 8	168 hours	x		x		x					x	
Cycle 3	Day 1	Pre-infusion[j]	x	x	x	x	x	X	x	X	x		x
		RO6958688 EOI[j]			x					X			
	Day 2	24 hours	x		x		x			X			
	Day 15	336 hours	x	x	x		x						
Cycle 4	Day 1	Pre-infusion[j]	x	x	x	x	x	X					
		RO6958688 EOI[j]			x								
	Day 2	24 hours	x		x								

“Use of this document is governed by the terms of use on the first page of this document.”

Appendix 1
Schedule of Assessments (cont.)

TABLE A7: SCHEDULE OF HOURLY ASSESSMENTS COHORTS C & SAFETY RO6958688 ADMINISTRATION SCHEDULE (CONT.)

Cycle	Day	Scheduled Time (h)	Vital signs[b, n,o]	ECG-12 lead[c]	RO6958688 PK Sampling[d]	Atezolizumab PK Sampling[e]	RO6958688 Anti-Drug Antibody (ADA)[g]	Atezolizumab Anti-Drug Antibody (ADA)[g]	PD Blood Flow Cytometry[m]	PD Blood Cytokines[f]	Tumor Biopsy[h]	FDG-PET[i]	TCR Vβ[l]
Cycle 5	Day 1	Pre-infusion[j]	x	x	x		x						
		RO6958688 EOI[j]			x								
	Day 2	24 hours	x		x								
Cycle 6	Day 1	Pre-infusion[j]	x	x	x	x	x						
		RO6958688 EOI[j]			x								
	Day 8	168 hours	x	x	x							X	
Cycle 7 to Cycle 10	Day 1	Pre-infusion[j]	x	x	x	x[k]	x	x[k]					
		RO6958688 EOI[j]			x								
Cycle 11 and every 2nd cycle	Day 1	Pre-infusion[j]	x	x	x		x						
		RO6958688 EOI[j]			x								
End of treatment	Anytime		x	x	x	x	x	x					
28-day Safety Follow Up	Anytime		x	x	x	x	x						

"Use of this document is governed by the terms of use on the first page of this document."

TABLE A7: SCHEDULE OF HOURLY ASSESSMENTS COHORTS C & SAFETY RO6958688 ADMINISTRATION SCHEDULE (CONT.)

ADA = anti-drug antibody; CEA = carcinoembryonic antigen; D = day; ECOG = Eastern Cooperative Oncology Group; PD = pharmacodynamics; PK = pharmacokinetic.

a Screening assessments should be performed between D-28 and D-1, unless specified.

b The patient's vital signs (heart rate, respiratory rate, blood pressure, oxygen saturation and temperature) should be determined up to 60 (± 10) minutes before each atezolizumab infusion. Vital signs should also be obtained during or after the atezolizumab infusion if clinically indicated. Vital signs (including supine blood pressure and heart rate) will be monitored on Day 1 RO6958688 pre-infusion, every 15 minutes until the end of RO6958688 infusion and, thereafter, every 30 minutes until the infusion line is removed. Starting Cycle 2 Day 1: RO6958688 pre-infusion, every 30 minutes during RO6958688 infusion and every 30 minutes after the end of RO6958688 infusion until infusion line is removed. From Cycle 3 onwards vital signs will only be obtained on Day 1 RO6958688 pre-infusion. For the purposes of the eCRF vital signs will only be captured pre-infusion and in case of abnormalities.

c Triplicate 12-lead ECG at screening (within 7 days before first dose of RO6958688) pre- and end of infusion on Cycle 1 Day 1 and C1D15. Pre-infusion at all other study drug administrations and at EOT and at 28-day safety follow up visit. Additional unscheduled ECG assessments should be performed in case of abnormalities and if clinical symptoms occur. Recording must be done prior to PK sampling.

d All RO6958688 PK sampling Timepoints are reference from the start of RO6958688 infusion. PK time windows: Pre-infusion: up to – 4 h - EOI: up to + 30 min - 2 h post EOI: +/- 30 min - 24 h: +/- 2 h - 48 h: +/- 4 h - Later: +/- 12 h.

e All atezolizumab PK sampling Timepoints are reference from the start of atezolizumab infusion.

f Serum or plasma samples for the assessment of cytokine (PD blood cytokines) release will be collected. At the time of an IRR please see IRR SoA Table A3.

g Additional samples will be drawn at the time of an IRR, see IRR SoA Table A3 or hypersensitivity reaction, treatment discontinuation *and* at the 28-day safety follow-up visit (RO6958688 only). A PK sample will be taken at the time of ADA sampling.

h Mandatory tumor biopsy samples will be collected on two occasions (once at baseline and once during the study treatment period), except for NSCLC patients for whom there is no accessible lesion. If preliminary data suggest that modification of the on-treatment tumor biopsy timepoint would be more appropriate, alternative on-treatment tumor biopsy timepoints could be considered in the future cohorts.

i FDG-PET should be done at baseline (Day-14 to D-1) but before the baseline biopsy. The on-treatment FDG-PET scan must be done at week 4 (C2D8 + 1 week) and at week 16 (C6D8 pre-dose and up to 72h before the visit) in case of dose delay the FDG-PET should be obtained at week 4 (+1week) and week 16. If preliminary data suggest that modification of the on-treatment FDG-PET timepoint would be more appropriate, alternative on-treatment FDG-PET timepoints could be considered in the future cohorts.

j Pre-infusion timepoints relate to RO6958688 or atezolizumab. Pre-dose PK samples can be taken up to 1-hour before infusion start for atezolizumab; after atezolizumab end of infusion and/or before the start of RO6958688 infusion for the RO6958688 pre-dose sample.

k Atezolizumab PK and ADA sampling to be drawn at Cycle 8 Day 1 then every 8 cycles from this timepoint onwards.

l For TCR Vβ, at each time point Cycle 1 Day 1 and Cycle 3 Day 1, 2 whole blood samples should be drawn. Samples should be taken prior to IMP administration.

m Blood Flow Cytometry sample to be drawn pre-dose at C1D1, C1D8, C2D1, and C3D1.

n If a patient has experienced a Grade 3 IRR/CRS event during the previous treatment administration visit, the patient should be hospitalized for at least 24 hours after the end of infusion at next treatment administration visit during which vital signs will be monitored as follows: every 30 (± 10) minutes for the first hour post infusion, every 60 (± 15) minutes during the following 3 hours, every 120 (± 20) minutes for the next 8 hours and every 240 (± 30) minutes for the remaining 12 hours.

o If a patient experiences a Grade 3 or higher treatment-related adverse event (with the exception of IRR/CRS) within the 24 hours period following the previous infusion, the patient should be observed for at least 8 hours after the end of RO6958688 infusion at the next administration visit during which vital signs will be monitored as follows: every 30 (± 10) minutes for the first hour post infusion, every 60 (± 15) minutes during the following 3 hours, every 120 (± 20) minutes for the next 4 hours.

"Use of this document is governed by the terms of use on the first page of this document."

Appendix 1
Schedule of Assessments (cont.)

TABLE A8: SCHEDULE OF ASSESSMENTS: STEP UP 1200 MG (COHORT B1) RO6958688 ADMINISTRATION SCHEDULE

Cycle	Screening	Cycle 1							Cycle 2				Cycle 3			Cycle 4		Cycle 5		Cycle 6		Cycle 7 onwards	End of treatment	28-Day Safety Follow Up[m]
Day	D-28 to D-1	Day 1[o]	Day 2	Day 3	Day 8	Day 9	Day 15	Day 16	Day 1	Day 2	Day 8	Day 15	Day 1	Day 2	Day 15	Day 1	Day 2	Day 1	Day 2	Day 1	Day 8	Day 1		
Assessments[a]																								
Informed Consent[b]	x																							
Eligibility	x																							
Demography	x																							
Medical History	x																							
Physical Examination[c]	x	x			x		x		x		x	x	X		x	x		x		x	x	x	x	x
Vital Signs	x	x	x	X	x	x	x	x	x	x	x	x	X	X	x	x	x	x	x	x	x	x[p]	x	x
Administration of atezolizumab Q3W[x]		x[z]							x				X			x		x		x		x		
Administration of RO6958688 QW[y]		x[z]			x		x		x		x	x	X			x		x		x		x[ae]		
ECG-12 lead[d]	x	x			x		x		x		x	x	X		x	x		x		x	x	x	x	x
ECOG Performance Status	x	x			x		x		x		x	x	X		x	x		x		x	x	x[q]	x	x
Pulmonary Function test[aj]	x																							
Hematology[g]	x	x	x	X	x	x	x	x	x	x	x	x	X	X	x	x	x	x	x	x	x	x[r]	x	x
Blood Chemistry[g,aa]	x	x	x	X	x	x	x	x	x	x	x	x	X	X	x	x	x	x	x	x	x	x[r]	x	x
Serum Thyroid-Stimulating Hormone Free T3-T4[i]		x																x					x	x
Coagulation[g,h]	x	x	x	X	x	x	x	x	x	x	x	x	X	X	x	x	x	x	x	x	x	x[r]	x	x
Urinalysis[g]	x	x	x	X	x	x	x	x	x	x	x	x	X	X	x	x	x	x	x	x	x	x[r]	x	x
HBV and HCV Serology	x																							
Auto-Antibody Panel[v]	x								x							x				x		x[w]		
Pregnancy Test[e]	x	X																					x	x

"Use of this document is governed by the terms of use on the first page of this document."

Appendix 1
Schedule of Assessments (cont.)

TABLE A8: SCHEDULE OF ASSESSMENTS STEP UP 1200 MG (COHORT B1) RO6958688 ADMINISTRATION SCHEDULE (CONT.)

Cycle	Screening	Cycle 1							Cycle 2				Cycle 3			Cycle 4		Cycle 5		Cycle 6		Cycle 7 onwards	End of treatment	28-Day Safety Follow Up[m]
Day	D-28 to D-1	Day 1[o]	Day 2	Day 3	Day 8	Day 9	Day 15	Day 16	Day 1	Day 2	Day 8	Day 15	Day 1	Day 2	Day 15	Day 1	Day 2	Day 1	Day 2	Day 1	Day 8	Day 1		
Atezolizumab PK Sampling[ab]		x			x		x		x		x	x	X			X				x		x[af]	x	x
RO6958688 PK Sampling[ab]		x	x	X	x	x	x	x	x	x	x	x	X	x	x	X	x	X	x	x	x	x[t]	x	x
Atezolizumab Anti-Drug Antibody (ADA)[ac]		x							x				X			X						x[af]	x	
RO6958688 Anti-Drug Antibody (ADA)[ac]		x			x		x		x		x	x	X	x	x	X	x	X	x	x	x	x[u]	x	x
PD Blood Flow Cytometry		x	x		x				x				x											
PD Blood Cytokines[f]		x	x	X	x	x	x	x	x	x	x	x	X	x										
Archival tumor Biopsy	x																							
Fresh Tumor Biopsy[j]	x								x				X											
Clinical Genotyping[ag]		x																						
RCR Sample (DNA and RNA)[ag]		x																						
CEA confirmation on Archival Tissue[k]	x																							
FDG-PET[n]	x[s]										x[s]										x[s]			
TCR Vβ[ah]		x											x											
Sol CEA[ai]		x							x				x											
Tumor Assessment[l]	x														x						x		x	
Tumor Growth Kinetic[ad]	x																							
Adverse Events	X																							
Previous and Concomitant Treatments	X																							

“Use of this document is governed by the terms of use on the first page of this document.”

TABLE A8: SCHEDULE OF ASSESSMENTS STEP UP 1200 MG (COHORT B1) RO6958688 ADMINISTRATION SCHEDULE (CONT.)

Notes:

ADA = anti-drug antibody; CEA = carcinoembryonic antigen; D = day; ECOG = Eastern Cooperative Oncology Group; PD = pharmacodynamics; PK = pharmacokinetic.

a All visit and their assigned safety assessments (hematology, blood chemistry, coagulation, urinalysis, physical examination), unless otherwise indicated should occur within a 3-day time window.

b Informed consent must be obtained before any study-specific procedures. All patients will be tested for HBV and HCV serology. Patients with HIV infection, or active hepatitis B (chronic or acute), or active hepatitis C infection are not eligible.

c Physical examinations (including weight) and ECOG Performance Status will be done at screening, at the time of treatment administration, at the EOT and at the 28-day safety follow-up visits. Results must be obtained prior to infusion.

d Triplicate 12-lead ECG at screening (within 7 days before first dose of RO6958688) pre- and end of infusion on Cycle 1 Day 1 and Cycle 1 Day 15 and at the 28-day safety follow-up visit. Pre-infusion at all other study drug administrations. Additional unscheduled ECG assessments should be performed in case of abnormalities and if clinical symptoms occur. Recording must be done prior to PK sampling.

e Serum pregnancy test at screening, within 7 days prior to first dose. From the date of first serum pregnancy test at screening, a urine pregnancy test must be performed every 4 weeks. Also to be performed at study termination and safety follow-up visit (28 days post last infusion).

f Serum or plasma samples for the assessment of cytokine (PD blood cytokines) release will be collected. At the time of an IRR, please see IRR SoA Table A3.

g Hematology, blood chemistry, coagulation, and urinalysis can be performed up to 24 hours (72h if during weekend) prior to scheduled dosing. Results must be obtained prior to infusion. Soluble CEA will be measured as part of the blood chemistry measurements on Cycle 1 Day 1 and every 6 weeks thereafter for patients who continue treatment and at the end of treatment visit. Soluble CD25 is to be obtained at screening and during the treatment period at Day 1 of every cycle (samples can be drawn 7 days prior to Day 1 of each cycle). Before IMP administration on Day 1 of each cycle, the investigator should confirm that the result of soluble CD25 measurement from the previous week or if available from the same day is not significantly high. If there is a significantly high level, the investigator should discuss with the Medical Monitor if the patient can or cannot receive further IMP administration. Ferritin should be obtained at screening and every week during the treatment period: Day 1, Day 8 and Day 15 of every cycle (-2 days) when applicable.

h For coagulation (including PT/INR and PTT), an additional sample will be taken at the time of an IRR or hypersensitivity reaction, see IRR SoA Table A3. Additional coagulation parameters (i.e., first chromogenic antithrombin III then antigenic antithrombin III in case of chromogenic antithrombin III decrease, f brinogen, prothrombin time, fibrin degradation products, D-dimer) could be assessed according to clinical judgment.

i Serum TSH, free T3 (or total T3 for sites where free T3 is not performed) and T4 levels will be assessed at baseline pre-dose at Cycle 1, on study (every 3 months) and at study completion/early termination visit and safety follow-up visit (28 days after last dose of study drug).

j Mandatory tumor biopsy samples will be collected on two occasions (once at baseline and once during the study treatment period), except for NSCLC patients for whom there is no accessible lesion. On treatment biopsies will be collected as per Section 3.3.2.2. If preliminary data suggest that modification of the on-treatment tumor biopsy timepoint would be more appropriate, alternative on-treatment tumor biopsy timepoints could be considered in the future cohorts. Unscheduled tumor biopsies can be taken at any time point, if considered necessary for tumor assessment and patient management as decided by the investigator.

k CEA expression for elig bility will be confirmed centrally for U.S. patients on archival tumor material (refer to inclusion criterion #13), if available, or confirmed from a freshly obtained tumor biopsy. Results available prior to screening (outside the 28 day window) can be used.

l Tumor assessments will be performed at screening, then every 8 weeks after Cycle 1 Day 1 for the first year, and thereafter every 12 weeks for the second year until disease progression or treatment discontinuation. Optional latest pre-study CT scan should be provided for assessment of tumor growth kinetics within 6 weeks of patient entering the study. For the scheduled tumor assessments beyond Screening a (+/- 7-day window is permitted).

m Patients who complete the study or discontinue from the study early will be asked to return to the clinic 28 days after the last dose of study drugs for post-study follow-up visits.

n FDG-PET should be done at baseline (Day-14 to D-1) but before the baseline biopsy. The on-treatment FDG-PET scan must be done at week 4 (C2D8 + 1 week) and at week 16 (C6D8 and up to 72h before the visit) in case of dose delay the FDG-PET should be obtained at week 4 (+1week) and week 16. If preliminary data suggest that modification of the on-treatment FDG-PET timepoint would be more appropriate, alternative on-treatment FDG PET timepoints could be considered. Please refer to Appendix 9 for further information.

“Use of this document is governed by the terms of use on the first page of this document.”

Appendix 1
Schedule of Assessments (cont.)

TABLE A8: SCHEDULE OF ASSESSMENTS STEP UP 1200 MG (COHORT B1) RO6958688 ADMINISTRATION SCHEDULE (CONT.)

o At least 24 hours (overnight) hospital stay required following administration of study drug at Cycle 1 Day 1.

p Vital signs to be assessed at D1 from cycle 7 onwards.

q ECOG to be assessed at D1 from cycle 7 onwards.

r Hematology, blood chemistry, coagulation and urinalysis (to be drawn at D1 from cycle 7 onwards) can be performed up to 24 hours (72h if during weekend) prior to scheduled dosing. Results must be obtained prior to atezolizumab and RO6958688 infusion. Before each IMP administration on Day 1 or each cycle, the investigator should confirm that the result of soluble CD25 measurement from the previous week or if available from the same day is not significantly high. If there is a significantly high level, the investigator should discuss with the Medical Monitor if the patient can or cannot receive further IMP administration.

s For FDG PET assessment, body weight, last calorific meal information, glucose level are needed (see Appendix 9 of the protocol for more information).

t RO6958688 PK sampling to be drawn preinfusion and at end of infusion at D1 of each cycle from Cycle 7 to Cycle 10. At Cycle 11 and every second cycle, RO6958688 PK samples to be drawn preinfusion and at end of infusion on D1.

u RO6958688 ADA sampling to be drawn preinfusion at D1 of each cycle from Cycle 7 to Cycle 10. At cycle 11 and every second cycle, RO6958688 ADA samples to be drawn preinfusion on Day 1 at the mentioned timepoints.

v In patients who develop signs and/or symptoms suggestive of auto-immune disease while on treatment, the antibody panel should be repeated. The autoant body panel will be assessed at screening, pre-dose every second cycle, and every 3 months after the third month on therapy (samples can be drawn up to 1 week before Day 1 visit).

w To be performed every 3 months after Cycle 6 (ie. Cycle 10, Cycle 14…).

x Atezolizumab will be delivered over 60 (+/-15) minutes. If the first infusion is tolerated without infusion associated adverse events, the second infusion may be delivered over 30 (+/-10) minutes. If the 30 minute infusion is well tolerated, all subsequent infusions may be delivered over 30 (+/-10) minutes.

y The start of the RO6958688 infusion on Day 1 of each cycle should be after the end of the atezolizumab infusion as per Section 4.3.2.3.

z Prophylactic corticosteroids to be administered at C1D1 as per Table 2. The use of analgesics and/or antihistamines is allowed to minimize expected flu like symptoms prior to the first RO6958688 administration.

aa Serum CEA will also be measured as a disease monitoring marker (as part of the serum chemistry panel).

ab Blood for PK should always be drawn at the indicated time point.

ac Additional samples will be drawn at the time of an IRR, see IRR SoA Table A3 or hypersensitivity reaction, treatment discontinuation *and* at the 28-day safety follow-up visit (RO6958688 only). A PK sample will be taken at the time of ADA sampling.

ad An exploratory assessment of tumor growth kinetics will be made by comparing post-treatment scans with at least 2 pre-treatment scans not older than 12 weeks prior to C1D1, if available. The two pre-treatment scans will consist of a pre-treatment scan (if available) and the study baseline scan, and will allow estimation of tumor growth rate before start of treatment.

ae Administration of RO6958688 to be done at D1 from Cycle 7 onwards.

af Atezolizumab PK and ADA sampling to be drawn at Cycle 8 Day 1 then every 8 cycles from this timepoint onwards.

ag Clinical genotyping and RCR (DNA and RNA) samples to be drawn before any IMP administration.

ah For TCR Vβ, at each time point Cycle 1 Day 1 and Cycle 3 Day 1, 2 whole blood samples should be drawn. Samples should be taken prior to IMP administration

ai For soluble CEA central assessment: samples will be collected pre-dose at: C1D1 (baseline), C2D1 and C3D1.

aj Evaluate DLCO (corrected for both alveolar volume and hemoglobin) and FEV1/VC/TLC at screening for patients with bilateral lung metastases or patients with lobectomy or pneumonectomy with lung metastases in the remaining lung. Additional tests may be performed if clinically indicated.

"Use of this document is governed by the terms of use on the first page of this document."

Appendix 1
Schedule of Assessments (cont.)

TABLE A9: SCHEDULE OF HOURLY ASSESSMENTS STEP UP 1200 MG (COHORT B1) RO6958688 ADMINISTRATION SCHEDULE

Cycle	Day	Scheduled Time (h)	Vital Signs[b,n,o]	ECG-12 lead[c]	RO6958688 PK Sampling[d]	Atezolizumab PK Sampling[e]	PD Blood Flow Cytometry[m]	PD Blood Cytokines[f]	RO6958688 Anti-Drug Ant body (ADA)[g]	Atezolizumab Anti-Drug Antibody (ADA)[g]	Tumor Biopsy[h]	FDG PET[i]	TCR Vβ[l]
Screening	D-28 to D1[a]		x	x							x	x	
Cycle 1	Day 1	Pre-infusion[j]	x	x	x	x	X	X	x	x			x
		0.5h (+/- 10 min) atezolizumab post EOI				x							
		End of RO6958688 infusion	x	x	x			X					
		2 hours post end of RO6958688 infusion			x			X					
		4 hours post end of RO6958688 infusion					X	X					
	Day 2	24 hours	x		x		X	X					
	Day 3	48 hours	x		x			X					
	Day 8	Pre-infusion	x	x	x	x	X	X	x				
		End of RO6958688 infusion	x		x			X					
		2 hours post end of RO6958688 infusion			x			X					

“Use of this document is governed by the terms of use on the first page of this document.”

Appendix 1
Schedule of Assessments (cont.)

TABLE A9: SCHEDULE OF HOURLY ASSESSMENTS STEP UP 1200 MG (COHORT B1) RO6958688 ADMINISTRATION SCHEDULE (CONT.)

Cycle	Day	Scheduled Time (h)	Vital Signs[b,n,o]	ECG-12 lead[c]	RO6958688 PK Sampling[d]	Atezolizumab PK Sampling[e]	PD Blood Flow Cytometry[m]	PD Blood Cytokines[f]	RO6958688 Anti-Drug Ant body (ADA)[g]	Atezolizumab Anti-Drug Antibody (ADA)[g]	Tumor Biopsy[h]	FDG PET[i]	TCR Vβ[l]
Cycle 1	Day 9	192 hours	x		x			X					
	Day 15	Pre-infusion	x	x	x	x		X	x				
		End of infusion	x	x	x			X					
		2 hours post end of infusion			x								
		4 hours post end of RO6958688 infusion						X					
	Day 16	360 hours	x		x			X					
Cycle 2	Day 1	Pre-infusion[j]	x	x	x	x	X	X	x	x	x		
		0.5h (+/- 10 min) atezolizumab post EOI				x							
		End of RO6958688 infusion	x		x			X					
		2 hours post end of RO6958688 infusion			x								
	Day 2	24 hours	x		x			X					
	Day 8	Pre-infusion	x	x	x	x		X	x			x	
		End of RO6958688 infusion			x			X					

"Use of this document is governed by the terms of use on the first page of this document."

TABLE A9: SCHEDULE OF HOURLY ASSESSMENTS STEP UP 1200 MG (COHORT B1) RO6958688 ADMINISTRATION SCHEDULE (CONT.)

Cycle	Day	Scheduled Time (h)	Vital Signs[b,n,o]	ECG-12 lead[c]	RO6958688 PK Sampling[d]	Atezolizumab PK Sampling[e]	PD Blood Flow Cytometry[m]	PD Blood Cytokines[f]	RO6958688 Anti-Drug Antibody (ADA)[g]	Atezolizumab Anti-Drug Antibody (ADA)[g]	Tumor Biopsy[h]	FDG PET[i]	TCR Vβ[l]
Cycle 2	Day 15	Pre-infusion	x	x	x	x		X	x				
		End of RO6958688 infusion			x			X					
Cycle 3	Day 1	Pre-infusion[j]	x	x	x	x	x	X	x	x	x		x
		End of RO6958688 infusion			x			X					
	Day 2	24 hours	x		x			X	x				
	Day 15	336 hours	x	x	x				x				
Cycle 4	Day 1	Pre-infusion[j]	x	x	x	x			x	x			
		End of RO6958688 infusion			x								
	Day 2	24 hours	x		x				x				
Cycle 5	Day 1	Pre-infusion[j]	x	x	x				x				
		End of RO6958688 infusion			x								
	Day 2	24 hours	x		x				x				

"Use of this document is governed by the terms of use on the first page of this document."

Appendix 1
Schedule of Assessments (cont.)

TABLE A9: SCHEDULE OF HOURLY ASSESSMENTS STEP UP 1200 MG (COHORT B1) RO6958688 ADMINISTRATION SCHEDULE (CONT.)

Cycle	Day	Scheduled Time (h)	Vital Signs[b,n,o]	ECG-12 lead[c]	RO6958688 PK Sampling[d]	Atezolizumab PK Sampling[e]	PD Blood Flow Cytometry[m]	PD Blood Cytokines[f]	RO6958688 Anti-Drug Antibody (ADA)[g]	Atezolizumab Anti-Drug Ant body (ADA)[g]	Tumor Biopsy[h]	FDG PET[i]	TCR Vβ[l]
Cycle 6	Day 1	Pre-infusion[j]	x	x	x	x			x				
		End of RO6958688 infusion			x								
	Day 8	168 hours	x	x	x				x			x	
Cycle 7 to Cycle 10	Day 1	Pre-infusion[j]	x	x	x	x[k]			x	x[k]			
		End of RO6958688 infusion			x								
Cycle 11 and every 2nd cycle	Day 1	Pre-infusion[j]	x	x	x				x				
		RO6958688 EOI[j]			x								
End of treatment	Anytime		x	x	x	x			x	x			
28-Day Safety Follow-up	Anytime		x	x	x	x			x				

"Use of this document is governed by the terms of use on the first page of this document."

TABLE A9: SCHEDULE OF HOURLY ASSESSMENTS STEP UP 1200 MG (COHORT B1) RO6958688 ADMINISTRATION SCHEDULE (CONT.)

ADA = anti-drug antibody; CEA = carcinoembryonic antigen; D = day; ECOG = Eastern Cooperative Oncology Group; PD = pharmacodynamics; PK = pharmacokinetic.

a Screening assessments should be performed between D-28 and D-1, unless specified.

b The patient's vital signs (heart rate, respiratory rate, blood pressure, oxygen saturation and temperature) should be determined up to 60 (± 10) minutes before each atezolizumab infusion. Vital signs should also be obtained during or after the atezolizumab infusion if clinically indicated. Vital signs (including supine blood pressure and heart rate) will be monitored on Day 1 RO6958688 pre-infusion, every 15 minutes until the end of RO6958688 infusion and, thereafter, every 30 minutes until the infusion line is removed. Starting Cycle 2 Day 1: RO6958688 pre-infusion, every 30 minutes during RO6958688 infusion and every 30 minutes after the end of RO6958688 infusion until infusion line is removed. From Cycle 3 onwards vital signs will only be obtained on Day 1 RO6958688 pre-infusion. For the purposes of the eCRF vital signs will only be captured pre-infusion and in case of abnormalities

c Triplicate 12-lead ECG at screening (within 7 days before first dose of RO6958688) pre- and end of infusion on Cycle 1 Day 1 and C1D15. Pre-infusion at all other study drug administrations and at EOT and at 28-day safety follow up visit. Additional unscheduled ECG assessments should be performed in case of abnormalities and if clinical symptoms occur. Recording must be done prior to PK sampling.

d All RO6958688 PK sampling Timepoints are reference from the start of RO6958688 infusion. PK time windows: Pre-infusion: up to – 4 h - EOI: up to + 30 min - 2 h post EOI: +/- 30 min - 24 h: +/- 2 h - 48 h: +/- 4 h - Later: +/- 12 h.

e All atezolizumab PK sampling Timepoints are reference from the start of atezolizumab infusion.

f Serum or plasma samples for the assessment of cytokine (PD blood cytokines) release will be collected. At the time of an IRR please see IRR SoA Table A3.

g Additional samples will be drawn at the time of an IRR, see IRR SoA Table A3 or hypersensitivity reaction, treatment discontinuation, *and* at the 28-day safety follow-up visit (RO6958688 only). A PK sample will be taken at the time of ADA sampling.

h Mandatory tumor biopsy samples will be collected on two occasions (once at baseline and once during the study treatment period), except for NSCLC patients for whom there is no accessible lesion. If preliminary data suggest that modification of the on-treatment tumor biopsy timepoint would be more appropriate, alternative on-treatment tumor biopsy timepoints could be considered in the future cohorts.

i FDG-PET should be done at baseline (Day-14 to D-1) but before the baseline biopsy. The on-treatment FDG-PET scan must be done at week 4 (C2D8 + 1 week) and at week 16 (C6D8 pre-dose and up to 72h before the visit) in case of dose delay the FDG-PET should be obtained at week 4 (+1week) and week 16. If preliminary data suggest that modification of the on-treatment FDG-PET timepoint would be more appropriate, alternative on-treatment FDG-PET timepoints could be considered in the future cohorts.

j Pre-infusion timepoints relate to RO6958688 or atezolizumab. Pre-dose PK samples can be taken up to 1-hour before infusion start for atezolizumab; after atezolizumab end of infusion and/or before the start of RO6958688 infusion for the RO6958688 pre-dose sample.

k Atezolizumab PK and ADA sampling to be drawn at Cycle 8 Day 1 then every 8 cycles from this timepoint onwards.

l For TCR Vβ, at each time point Cycle 1 Day 1 and Cycle 3 Day 1, 2 whole blood samples should be drawn. Samples should be taken prior to IMP administration.

m Blood Flow Cytometry sample to be drawn pre-dose at C1D1, C1D8, C2D1, and C3D1.

n If a patient has experienced a Grade 3 IRR/CRS event during the previous treatment administration visit, the patient should be hospitalized for at least 24 hours after the end of infusion at next treatment administration visit during which vital signs will be monitored as follows: every 30 (± 10) minutes for the first hour post infusion, every 60 (± 15) minutes during the following 3 hours, every 120 (± 20) minutes for the next 8 hours and every 240 (± 30) minutes for the remaining 12 hours.

o If a patient experiences a Grade 3 or higher treatment-related adverse event (with the exception of IRR/CRS) within the 24 hours period following the previous infusion, the patient should be observed for at least 8 hours after the end of RO6958688 infusion at the next administration visit during which vital signs will be monitored as follows: every 30 (± 10) minutes for the first hour post infusion, every 60 (± 15) minutes during the following 3 hours, every 120 (± 20) minutes for the next 4 hours.

"Use of this document is governed by the terms of use on the first page of this document."

Appendix 2
CKD-EPI equation for Calculation of Glomerular Filtration Rate (GFR)

CKD EPI Equation for Estimating GFR Expressed for Specified Race, Sex and Serum Creatinine in mg/dL (From Levey et al. Ann Intern Med 2009;150:604-612).

Race	Sex	Serum Creatinine, S_{cr} (mg/dL)	Equation (age in years for ≥ 18)
Black	Female	$\leqslant$ 0.7	$GFR = 166 \times (S_{cr}/0.7)^{-0.329} \times (0.993)^{Age}$
Black	Female	> 0.7	$GFR = 166 \times (S_{cr}/0.7)^{-1.209} \times (0.993)^{Age}$
Black	Male	$\leqslant$ 0.9	$GFR = 163 \times (S_{cr}/0.9)^{-0.411} \times (0.993)^{Age}$
Black	Male	> 0.9	$GFR = 163 \times (S_{cr}/0.9)^{-1.209} \times (0.993)^{Age}$
White or other	Female	$\leqslant$ 0.7	$GFR = 144 \times (S_{cr}/0.7)^{-0.329} \times (0.993)^{Age}$
White or other	Female	> 0.7	$GFR = 144 \times (S_{cr}/0.7)^{-1.209} \times (0.993)^{Age}$
White or other	Male	$\leqslant$ 0.9	$GFR = 141 \times (S_{cr}/0.9)^{-0.411} \times (0.993)^{Age}$
White or other	Male	> 0.9	$GFR = 141 \times (S_{cr}/0.9)^{-1.209} \times (0.993)^{Age}$

Normal glomerular filtration rate by age:

Age	Average estimated GFR
20-29	116
30-39	107
40-49	99
50-59	93
60-69	85
70+	75

"Use of this document is governed by the terms of use on the first page of this document."

Appendix 3
Eastern Cooperative Oncology Group Performance Status

ECOG PERFORMANCE STATUS	
**Grade**	**ECOG**
0	Fully active, able to carry on all pre-disease performance without restriction
1	Restricted in physically strenuous activity but ambulatory and able to carry out work of a light or sedentary nature, e.g., light house work, office work
2	Ambulatory and capable of all selfcare but unable to carry out any work activities. Up and about more than 50% of waking hours
3	Capable of only limited selfcare, confined to bed or chair more than 50% of waking hours
4	Completely disabled. Cannot carry on any selfcare. Totally confined to bed or chair
5	Dead

"Use of this document is governed by the terms of use on the first page of this document."

Appendix 4
Response Evaluation Criteria in Solid Tumors Version 1.1 Criteria

The investigator will evaluate response to treatment using Response Evaluation Criteria in Solid Tumors (RECIST) Version 1.1 and modified RECIST criteria.

DEFINITIONS OF MEASURABLE/NON-MEASURABLE LESIONS

At baseline, tumor lesions/lymph nodes will be categorized measurable or non-measurable as follows:

Measurable Tumor Lesions

Tumor lesions must be accurately measured in at least one dimension (longest diameter in the plane of measurement is to be recorded) with a minimum size of 10 mm by computed tomography (CT) or magnetic resonance imaging (MRI) scan (CT/MRI scan slice thickness/interval no greater than 5 mm).

Malignant lymph nodes: To be considered pathologically enlarged and measurable, a lymph node must be $\geq$15 mm in short axis when assessed by CT scan. Only the short axis will be measured and followed.

Non-Measurable Tumor Lesions

Non-measurable tumor lesions include:

- Small lesions (longest diameter $<$10 mm)
- Pathological lymph nodes with $\geq$10 to $<$15 mm short axis
- Truly non-measurable lesions. (e.g., ascites, pleural or pericardial effusion, lymphangitic involvement of skin or lung, peritoneal spread, abdominal masses/abdominal organomegaly identified by physical exam that is not measurable by reproducible imaging techniques).

Special considerations regarding lesion measurability:

Bone lesions, cystic lesions, and lesions previously treated with local therapy require particular comment:

Bone lesions:

- Lytic bone lesions or mixed lytic-blastic lesions with identifiable soft tissue components that can be evaluated by cross–sectional imaging techniques such as CT or MRI can be considered as measurable lesions if the soft tissue component meets the definition of measurability described above.
- Blastic bone lesions are non-measurable.

“Use of this document is governed by the terms of use on the first page of this document.”

Lesions with prior local treatment:

- Tumor lesions situated in a previously irradiated area, or in an area subjected to other loco-regional therapy, are usually not considered measurable unless there has been demonstrated progression in the lesion. Study protocols should detail the conditions under which such lesions would be considered measurable.

TARGET LESIONS: SPECIFICATIONS BY METHODS OF MEASUREMENTS

The same method of assessment and the same technique should be used to characterize each identified and reported lesion at baseline and during study.

TUMOR RESPONSE EVALUATION

Baseline documentation of ‘target’ and ‘non-target’ lesions

- When more than one measurable lesion is present at baseline, all lesions up to a maximum of five lesions total (and a maximum of two lesions per organ) representative of all involved organs should be identified as target lesions and will be recorded and measured at baseline.
- Where patients have only one or two organ sites involved a maximum of two (one site) and four lesions (two sites), respectively, can be recorded. Other lesions (including measurable lesion) in that organ will be recorded as non-measurable lesions.
- Target lesions should be selected on the basis of their size (lesions with the longest diameter), be representative of all involved organs, but in addition should be reproducible in repeated measurements.
- Lymph nodes: target lesions must have a short axis of $\geq$15 mm by CT scan. Only the short axis of these nodes will contribute to the baseline sum. Pathological lymph nodes with a short axis $\geq$10 mm but $<$15 mm should be considered as non-target lesions. Nodes that have a short axis $<$10 mm are considered non-pathological and should not be recorded or followed.
- A sum of the longest diameters (except for nodal lesions) for all target lesions will be calculated and reported as the baseline sum diameters. If lymph nodes are to be included in the sum then only the short axis is added to the sum. The baseline sum diameters will be used as reference to further characterize any objective tumor regression in the measurable dimension of the disease.
- Measurements are not required for non-target lesions. These should be followed as ‘present’, ‘absent’, or in rare cases ‘unequivocal progression’.

RESPONSE CRITERIA

This section provides the definitions of the criteria used to determine objective tumor response for target lesions.

“Use of this document is governed by the terms of use on the first page of this document.”

Appendix 4
Response Evaluation Criteria in Solid Tumors Version 1.1 Criteria (cont.)

EVALUATION OF TARGET LESIONS

- Complete Response (CR): Disappearance of all target lesions. Any pathological lymph nodes (whether target or non-target) must have reduction in short axis to $<$10 mm.
- Partial Response (PR): At least a 30% decrease in the sum of diameters of target lesions, taking as reference the baseline sum diameters.
- Progressive Disease (PD): At least a 20% increase in the sum of diameters of target lesions, taking as reference the smallest sum on study including baseline (nadir). In addition to the relative increase of 20%, the sum must also demonstrate an absolute increase of at least 5 mm.
- Stable Disease (SD): Neither sufficient shrinkage to qualify for PR nor sufficient increase to qualify for PD taking as a reference the smallest sum diameters while on study.

EVALUATION OF NON-TARGET LESIONS

- Complete Response (CR): Disappearance of all non-target lesions (and, if applicable, normalization of tumor marker level). All lymph nodes must be non-pathological in size ($<$10 mm short axis).
- Non-CR/Non-PD: Persistence of one or more non-target lesion(s) and/or maintenance of tumor marker level above the normal limits.
- Progressive Disease (PD): Unequivocal progression of existing non-target lesions. The appearance of one or more new lesions is also considered progression.

NEW LESIONS

- The appearance of new malignant lesions denotes disease progression. The finding of a new lesion should be unequivocal: i.e., not attributable to differences in scanning technique, change in imaging modality or findings thought to represent something other than tumor.
- A lesion identified during the study in an anatomical location that was not scanned at baseline is considered a new lesion and will indicate disease progression.
- If a new lesion is equivocal, for example because of its small size, continued therapy and follow-up evaluation will clarify if it represents truly new disease. If repeat scans confirm there is definitely a new lesion, then progression should be declared using the date of the initial scan.

EVALUATION OF RESPONSE

TIMEPOINT RESPONSE (OVERALL RESPONSE)

The table below provides a summary of the overall response status calculation at each timepoint for patients who have measurable disease at baseline.

“Use of this document is governed by the terms of use on the first page of this document.”

Target lesions	Non-target lesions	New lesions	Overall response
CR	CR	No	CR
CR	Non-CR/non-PD	No	PR
CR	Not evaluated	No	PR
PR	Non-PD or not all evaluated	No	PR
SD	Non-PD or not all evaluated	No	SD
Not all evaluated	Non-PD	No	NE
PD	Any	Yes or No	PD
Any	PD	Yes or No	PD
Any	Any	Yes	PD

CR = complete response, PR = partial response, SD = stable disease, PD = progressive disease, and NE = inevaluable.

Missing assessments and not-evaluable designation

When no imaging/measurement is done at all at a particular timepoint, the subject is not evaluable at that timepoint. If only a subset of measurements are made at an assessment, subject is also considered not evaluable at that timepoint, unless the missing lesion(s) would not change the assigned timepoint response, e.g., in the case of PD. If one or more target lesions were not assessed the Response for Target Lesions should be "Unable to Assess" (except where there is clear progression).

Overall response First time point	Overall response Subsequent time point	BEST overall response
CR	CR	CR
CR	PR	SD, PD or PR[a]
CR	SD	SD provided minimum criteria for SD duration met, otherwise, PD
CR	PD	SD provided minimum criteria for SD duration met, otherwise, PD
CR	NE	SD provided minimum criteria for SD duration met, otherwise NE
PR	CR	PR
PR	PR	PR
PR	SD	SD
PR	PD	SD provided minimum criteria for SD duration met, otherwise, PD
PR	NE	SD provided minimum criteria for SD duration met, otherwise NE
NE	NE	NE

CR = complete response, PR = partial response, SD = stable disease, PD = progressive disease, and NE = inevaluable.

a If a CR is truly met at first time point, then any disease seen at a subsequent time point, even disease meeting PR criteria relative to baseline, makes the disease PD at that point (since disease must have reappeared after CR). Best response would depend on whether minimum duration for SD was met. However, sometimes 'CR' may be claimed when subsequent scans suggest small lesions were likely still present and in fact the patient had PR, not CR at the first time point. Under these circumstances, the original CR should be changed to PR and the best response is PR.

"Use of this document is governed by the terms of use on the first page of this document."

Special notes on response assessment

When nodal disease is included in the sum of target lesions and the nodes decrease to 'normal' size ($<$10 mm), they may still have a measurement reported on scans. This measurement should be recorded even though the nodes are normal in order not to overstate progression should it be based on increase in size of the nodes. As noted earlier, this means that patients with CR may not have a total sum of 'zero' on the eCRF. Patients with a global deterioration of health status requiring discontinuation of treatment without objective evidence of disease progression at that time should be reported as 'symptomatic deterioration'. Every effort should be made to document objective progression even after discontinuation of treatment. Symptomatic deterioration is not a descriptor of an objective response: it is a reason for stopping study therapy. The objective response status of such patients is to be determined by evaluation of target and non-target disease.

In patients with advanced disease and the primary disease is still present or partially present, the primary tumor should be also captured under target or non-target lesions.

REFERENCES

Bogaerts J, Ford R, Sargent D, et al. Individual patient data analysis to assess modifications to the RECIST criteria. Eur J Cancer 2009;45:248–60.

Eisenhauer EA, Therasse P, Bogaerts J, et al. New response evaluation criteria in solid tumors: Revised RECIST guideline (version 1.1). Eur J Cancer 2009;45:228–47.

"Use of this document is governed by the terms of use on the first page of this document."

Appendix 5
Modified Response Evaluation Criteria in Solid Tumors

Conventional response criteria may not be adequate to characterize the anti-tumor activity of immunotherapeutic agents like atezolizumab, which can produce delayed responses that may be preceded by initial apparent radiological progression, including the appearance of new lesions. Therefore, modified response criteria have been developed that account for the possible appearance of new lesions and allow radiological progression to be confirmed at a subsequent assessment.

Modified Response Evaluation Criteria in Solid Tumors (RECIST) is derived from RECIST, Version 1.1 (v1.1) conventions[1] and immune-related response criteria[2] (irRC). When not otherwise specified, RECIST v1.1 conventions will apply.

Modified RECIST and RECIST v1.1: Summary of Changes

	RECIST v1.1	Modified RECIST
New lesions after baseline	Define progression	New measurable lesions are added into the total tumor burden and followed.
Non-target lesions	May contribute to the designation of overall progression	Contribute only in the assessment of a complete response
Radiographic progression	First instance of ≥ 20% increase in the sum of diameters or unequivocal progression in non-target disease	Determined only on the basis of measurable disease

RECIST = Response Evaluation Criteria in Solid Tumors.

A. DEFINITIONS OF MEASURABLE/NON-MEASURABLE LESIONS

All measurable and non-measurable lesions should be assessed at Screening and at the protocol-specified tumor assessment timepoints. Additional assessments may be performed, as clinically indicated for suspicion of progression.

---

[1] Eisenhauer et al. Eur J Cancer 2009;45: 228–47; Topalian et al. N Engl J Med 2012;366:2443–54; and Wolchok et al., Clin Can Res 2009;15:7412–20.
[2] Wolchok et al. Clin Can Res 2009;15:7412–20; Nishino et al. J Immunother Can 2014;2:17; Nishino et al. Clin Can Res 2013;19:3936–43.

“Use of this document is governed by the terms of use on the first page of this document.”

A.1 MEASURABLE LESIONS

Tumor Lesions. Tumor lesions must be accurately measured in at least one dimension (longest diameter in the plane of measurement is to be recorded) with a minimum size as follows:

- 10 mm by computed tomography (CT) or magnetic resonance imaging (MRI) scan (CT/MRI scan slice thickness/interval no greater than 5 mm)
- 10-mm caliper measurement by clinical examination (lesions that cannot be accurately measured with calipers should be recorded as non-measurable)

Malignant Lymph Nodes. To be considered pathologically enlarged and measurable, a lymph node must be $\geq$15 mm in the short axis when assessed by CT scan (CT scan slice thickness recommended to be no greater than 5 mm). At baseline and follow-up, only the short axis will be measured and followed.

A.2 NON-MEASURABLE LESIONS

Non-measurable tumor lesions encompass small lesions (longest diameter $<$10 mm or pathological lymph nodes with short axis $\geq$10 but $<$15 mm), as well as truly non-measurable lesions. Lesions considered truly non-measurable include leptomeningeal disease, ascites, pleural or pericardial effusion, inflammatory breast disease, lymphangitic involvement of skin or lung, peritoneal spread, and abdominal mass/abdominal organomegaly identified by physical examination that is not measurable by reproducible imaging techniques.

A.3 SPECIAL CONSIDERATIONS REGARDING LESION MEASURABILITY

Bone lesions, cystic lesions, and lesions previously treated with local therapy require particular comment, as outlined below.

Bone Lesions

Bone scan, positron emission tomography (PET) scan, or plain films are not considered adequate imaging techniques for measuring bone lesions. However, these techniques can be used to confirm the presence or disappearance of bone lesions.

Lytic bone lesions or mixed lytic–blastic lesions, with identifiable soft tissue components, that can be evaluated by cross-sectional imaging techniques such as CT or MRI can be considered as measurable lesions if the soft tissue component meets the definition of measurability described above.

Blastic bone lesions are non-measurable.

“Use of this document is governed by the terms of use on the first page of this document.”

Cystic Lesions

Lesions that meet the criteria for radiographically defined simple cysts should not be considered as malignant lesions (neither measurable nor non-measurable) since they are, by definition, simple cysts.

Cystic lesions thought to represent cystic metastases can be considered as measurable lesions, if they meet the definition of measurability described above. However, if non-cystic lesions are present in the same patient, these are preferred for selection as target lesions.

Lesions with Prior Local Treatment

Tumor lesions situated in a previously irradiated area or in an area subjected to other loco-regional therapy are usually not considered measurable unless there has been demonstrated progression in the lesion. Study protocols should detail the conditions under which such lesions would be considered measurable.

B. TUMOR RESPONSE EVALUATION

B.1 DEFINITIONS OF TARGET/NON-TARGET LESIONS

Target Lesions

When more than one measurable lesion is present at baseline, all lesions up to a maximum of five lesions total (and a maximum of two lesions per organ) representative of all involved organs should be identified as target lesions and will be recorded and measured at baseline. This means that, for instances in which patients have only one or two organ sites involved, a maximum of two lesions (one site) and four lesions (two sites), respectively, will be recorded. Other lesions (albeit measurable) in those organs will be recorded as non-measurable lesions (even if the size is > 10 mm by CT scan).

Target lesions should be selected on the basis of their size (lesions with the longest diameter) and be representative of all involved organs, but in addition, should lend themselves to reproducible repeated measurements. It may be the case that, on occasion, the largest lesion does not lend itself to reproducible measurement, in which circumstance, the next largest lesion that can be measured reproducibly should be selected.

Lymph nodes merit special mention since they are normal anatomical structures that may be visible by imaging even if not involved by tumor. As noted above, pathological nodes that are defined as measurable and may be identified as target lesions must meet the criterion of a short axis of ≥ 15 mm by CT scan. Only the short axis of these nodes will contribute to the baseline sum. The short axis of the node is the

“Use of this document is governed by the terms of use on the first page of this document.”

diameter normally used by radiologists to judge if a node is involved by solid tumor. Nodal size is normally reported as two dimensions in the plane in which the image is obtained (for CT, this is almost always the axial plane; for MRI, the plane of acquisition may be axial, sagittal, or coronal). The smaller of these measures is the short axis. For example, an abdominal node that is reported as being 20 mm $\times$ 30 mm has a short axis of 20 mm and qualifies as a malignant, measurable node. In this example, 20 mm should be recorded as the node measurement. All other pathological nodes (those with short axis $\geq$ 10 mm but $<$ 15 mm) should be considered non-target lesions. Nodes that have a short axis of $<$ 10 mm are considered non-pathological and should not be recorded or followed.

Lesions irradiated within 3 weeks prior to Cycle 1, Day 1 may not be counted as target lesions.

Non-Target Lesions

All other lesions (or sites of disease), including pathological lymph nodes, should be identified as non-target lesions and should also be recorded at baseline. Measurements are not required.

It is possible to record multiple non-target lesions involving the same organ as a single item on the Case Report Form (CRF) (e.g., “multiple enlarged pelvic lymph nodes” or “multiple liver metastases”).

After baseline, changes in non-target lesions will contribute only in the assessment of complete response (i.e., a complete response is attained only with the complete disappearance of all tumor lesions, including non-target lesions) and will not be used to assess progressive disease.

New Lesions

During the study, all new lesions identified and recorded after baseline must be assessed at all tumor assessment timepoints. New lesions will also be evaluated for measurability with use of the same criteria applied to prospective target lesions at baseline per RECIST, (e.g., non–lymph node lesions must be $\geq$ 10mm; see note for new lymph node lesions below). Up to a maximum of five new lesions total (and a maximum of two lesions per organ), all with measurements at all timepoints, can be included in the tumor response evaluation. New lesion types that would not qualify as target lesions per RECIST cannot be included in the tumor response evaluation.

New lesions that are not measurable at first appearance but meet measurability criteria at a subsequent timepoint will be measured from that point on and contribute to the sum

“Use of this document is governed by the terms of use on the first page of this document.”

of longest diameters (SLD), if the maximum number of 5 measurable new lesions being followed has not been reached.

B.2 CALCULATION OF SUM OF THE DIAMETERS

A sum of the diameters (longest for non-nodal lesions, short axis for nodal lesions) for all target lesions will be calculated as a measure of tumor burden.

The sum of the diameters is calculated at baseline and at each tumor assessment for the purpose of classification of tumor responses.

Sum of the Diameters at Baseline: The sum of the diameters for all target lesions identified at baseline prior to treatment on Day 1.

Sum of the Diameters at Tumor Assessment: For every on-study tumor assessment collected per protocol or as clinically indicated the sum of the diameters at tumor assessment will be calculated using tumor imaging scans. All target lesions selected at baseline and up to five new measurable lesions (with a maximum of two new lesions per organ) that have emerged after baseline will contribute to the sum of the diameters at tumor assessment. Hence, each net percentage change in tumor burden per assessment with use of modified RECIST accounts for the size and growth kinetics of both old and new lesions as they appear.

Note: In the case of new lymph nodes, RECIST v1.1 criteria for measurability (equivalent to baseline target lesion selection) will be followed. That is, if at first appearance the short axis of a new lymph node lesion ≥ 15 mm, it will be considered a measureable new lesion and will be tracked and included in the SLD. Thereafter, the lymph node lesion will be measured at subsequent timepoints and measurements will be included in the SLD, even if the short axis diameter decreases to < 15 mm (or even < 10 mm). However, if it subsequently decreases to < 10 mm, and all other lesions are no longer detectable (or have also decreased to a short axis diameter of < 10 mm if lymph nodes), then a response assessment of CR may be assigned.

If at first appearance the short axis of a new lymph node is ≥ 10 mm and < 15 mm, the lymph node will not be considered measurable but will still be considered a new lesion. It will not be included in the SLD unless it subsequently becomes measurable (short axis diameter ≥ 15 mm).

The appearance of new lymph nodes with diameter < 10 mm should not be considered pathological and not considered a new lesion.

“Use of this document is governed by the terms of use on the first page of this document.”

B.3 RESPONSE CRITERIA

Timepoint Response

It is assumed that at each protocol-specified timepoint, a response assessment occurs. Table 1 provides a summary of the overall response status calculation at each timepoint for patients who have measurable disease at baseline.

Complete Response (CR): Disappearance of all target and non-target lesions. Lymph nodes that shrink to < 10 mm short axis are considered normal.

Partial Response (PR): At least a 30% decrease in the sum of the diameters of all target and all new measurable lesions, taking as reference the baseline sum of diameters, in the absence of CR.

Note: the appearance of new measurable lesions is factored into the overall tumor burden, but *does not automatically qualify as progressive disease* until the sum of the diameters increases by ≥ 20% when compared with the sum of the diameters at nadir.

Stable Disease (SD): Neither sufficient shrinkage to qualify for PR nor sufficient increase to qualify for PD, taking as reference the smallest sum of the diameters while in the study.

Progressive Disease (PD): At least a 20% increase in the sum of diameters of all target and selected new measurable lesions, taking as reference the smallest sum during the study (nadir SLD; this includes the baseline sum if that is the smallest during the study). In addition to the relative increase of 20%, the sum must also demonstrate an absolute increase of at least 5 mm.

Impact of New Lesions on Modified RECIST

New lesions alone do not qualify as progressive disease. However, their contribution to total tumor burden is included in the sum of the diameters, which is used to determine the overall modified RECIST tumor response.

Missing Assessments and Not Evaluable Designation

When no imaging/measurement is done at all at a particular timepoint, the patient is considered not evaluable (NE) at that timepoint. If only a subset of lesion measurements are made at an assessment, usually the case is also considered NE at that timepoint, unless a convincing argument can be made that the contribution of the individual missing lesion(s) would not change the assigned timepoint response. This would only happen in the case of PD. For example, if a patient had a baseline sum of 50 mm with three measured lesions and at follow-up only two lesions were assessed but

“Use of this document is governed by the terms of use on the first page of this document.”

those gave a sum of 80 mm, the patient will be assigned PD status, regardless of the contribution of the missing lesion.

Table 1 Modified RECIST Timepoint Response Definitions

% Change in Sum of the Diameters [a]	Non-Target Lesion Response Assessment	Overall Modified RECIST Timepoint Response
−100% from baseline [b]	CR	CR
−100% from baseline [b]	Non-CR or not all evaluated	PR
≤−30% from baseline	Any	PR
>−30% to <+20%	Any	SD
Not all evaluated	Any	NE
≥+20%from nadir SLD	Any	PD

CR = complete response; NE = not evaluable; PD = progressive disease; PR = partial response; RECIST = Response Evaluation Criteria in Solid Tumors; SD = stable disease; SLD = sum of the longest diameter.

[a] Percent change in sum of the diameters (including measurable new lesions when present).

[b] When lymph nodes are included as target lesions, the % change in the sum of the diameters may not be 100% even if complete response criteria are met, since a normal lymph node is defined as having a short axis of < 10 mm. Any pathological lymph nodes (whether target or non-target) must have reduction in short axis to < 10 mm in order to meet the definition of CR.

“Use of this document is governed by the terms of use on the first page of this document.”

Appendix 6
Statistical Design of Modified Continual Reassessment Method with Escalation with Overdose Control

Various dose-toxicity scenarios have been investigated in order to cover a wide range of dose-toxicity possibilities and to be able to quantify the risk and benefit, should these scenarios actually occur.

In Table 1 the scenario settings are described in terms of the assumed parameters α and β and the reference dose d^* for a standard logistic regression model (see Section 6.7.1 – note that for the scenario specifications no mixture model is used). The dose range [mg] that results from the requirement of target toxicity between 20% and 30% is given in the last two columns. Scenario 2 is closest to the prior model, and scenario 3 is an extreme case with very high toxicity already at very low doses.

Table 1 Scenario Settings

No.	α	β	d^*	Lower target [mg]	Upper target [mg]
1	-3.00	2.00	80	179	235
2	0.50	0.30	500	0.9	5.6
3	1.30	0.30	45	< 0.052	< 0.052
4	-2.00	1.50	40	60	86
5	-5.00	5.00	30	62	69
6	-2.00	2.00	500	680	890
7	-5.00	0.20	35	> 1000	> 1000
8	1.30	1.50	5	0.8	1.2
9	-2.50	2.00	200	349	457

"Use of this document is governed by the terms of use on the first page of this document."

Figure 1, the resulting dose-toxicity curves from the scenarios (different colors and line types as per legend) are compared with the prior mean curve (grey continuous line). Scenario 2 is closest to the prior, while scenarios 6, 7, 9 have lower toxicity with varying steepness of the dose-toxicity curve and scenarios 1, 3, 4, 5 and 8 have higher toxicity than the prior curve from varying dose ranges onwards.

Operating characteristics were tabulated for each of these scenarios, as per Table 5 in Section 6.3. The numbers were computed based on 400 simulations for each of the scenarios. Four patients were used for each cohort, reflecting the typically used cohort size. In line with the protocol, the first patient was simulated separately. If a dose-limiting toxicity (DLT) occurred in this first patient, the cohort was closed and the next dose was recommended by the modified continual reassessment method (mCRM) with escalation with overdose control (EWOC) design. Otherwise 3 additional patients were recruited into the cohort, and only then the next dose was recommended by the mCRM with EWOC design. If at one point during the trial, no dose was acceptable because of the overdosing rule (the risk of having more than 30% DLT probability must be $<$ 25%), then the trial is stopped and a dose 0 is returned as the dose recommendation. Furthermore, if the maximum dose in the dose grid (1000 mg) is reached, at least 3 patients are already enrolled at that dose and the probability of underdosing (< 20% DLT probability) is greater than 50%, then the trial is stopped and 1000 mg is returned as the final dose recommendation.

In Figures 2–10, additional insight into the operating characteristics can be gained by graphical summaries of the distributions of important read-outs and resulting model fits, separately for each scenario. The frequentist distributions of the sample size n (“number of patients in total”), the final maximum-tolerated dose (MTD) estimate, the proportion of DLTs (%) and the number of overdosed patients $n_{\text{overdose}}\mathrm{n}_{\text{overdose}}$ (“number of patients above target”) are shown as histograms. In addition, the bottom panel in each figure compares the true toxicity curve from the scenario with the average model fit from the 400 simulation runs. Overall it can be said that the estimated curves approximate the true curves reasonably well in all scenarios in the relevant range up to ca. 30% probability of DLT.

“Use of this document is governed by the terms of use on the first page of this document.”

Appendix 6
Statistical Design of modified Continual Reassessment Method with Escalation with Overdose Control (cont.)

Figure 1 Comparison of Scenario Dose-Toxicity Curves

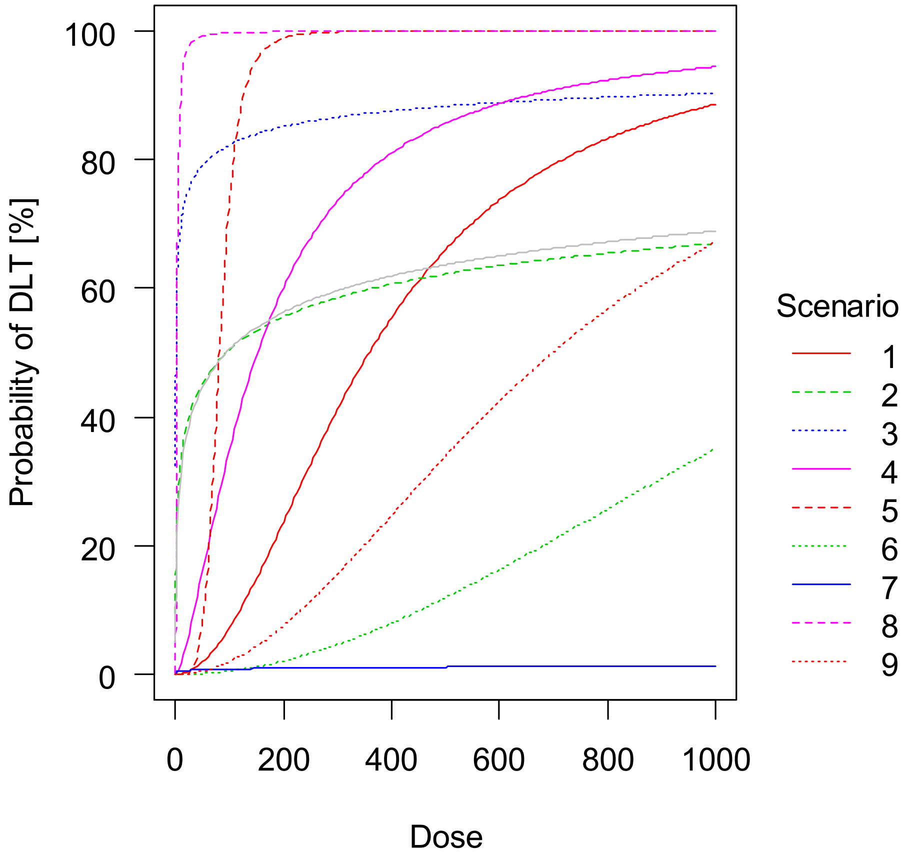

"Use of this document is governed by the terms of use on the first page of this document."

Appendix 6
Statistical Design of modified Continual Reassessment Method with Escalation with Overdose Control (cont.)

Figure 2 True versus Average Estimated Toxicity Curve in Scenario 1

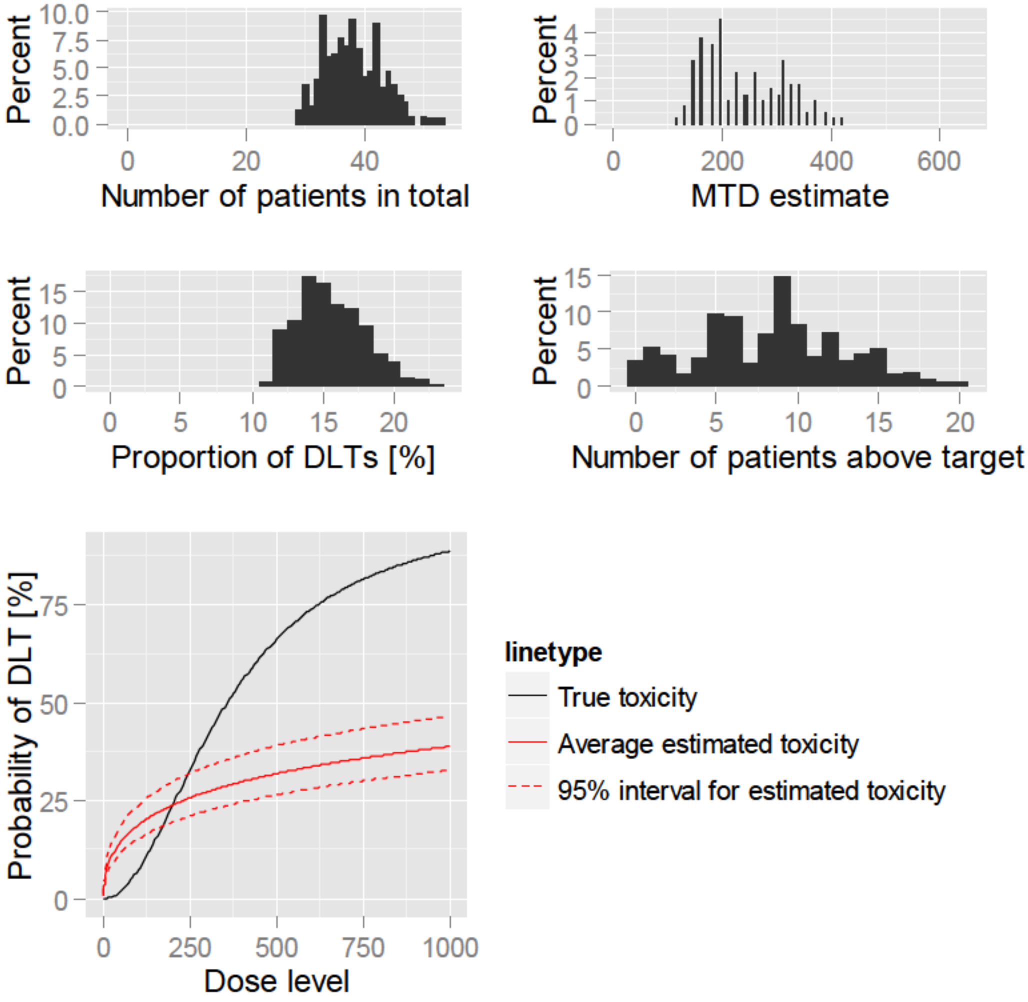

"Use of this document is governed by the terms of use on the first page of this document."

Figure 3 True versus Average Estimated Toxicity Curve in Scenario 2

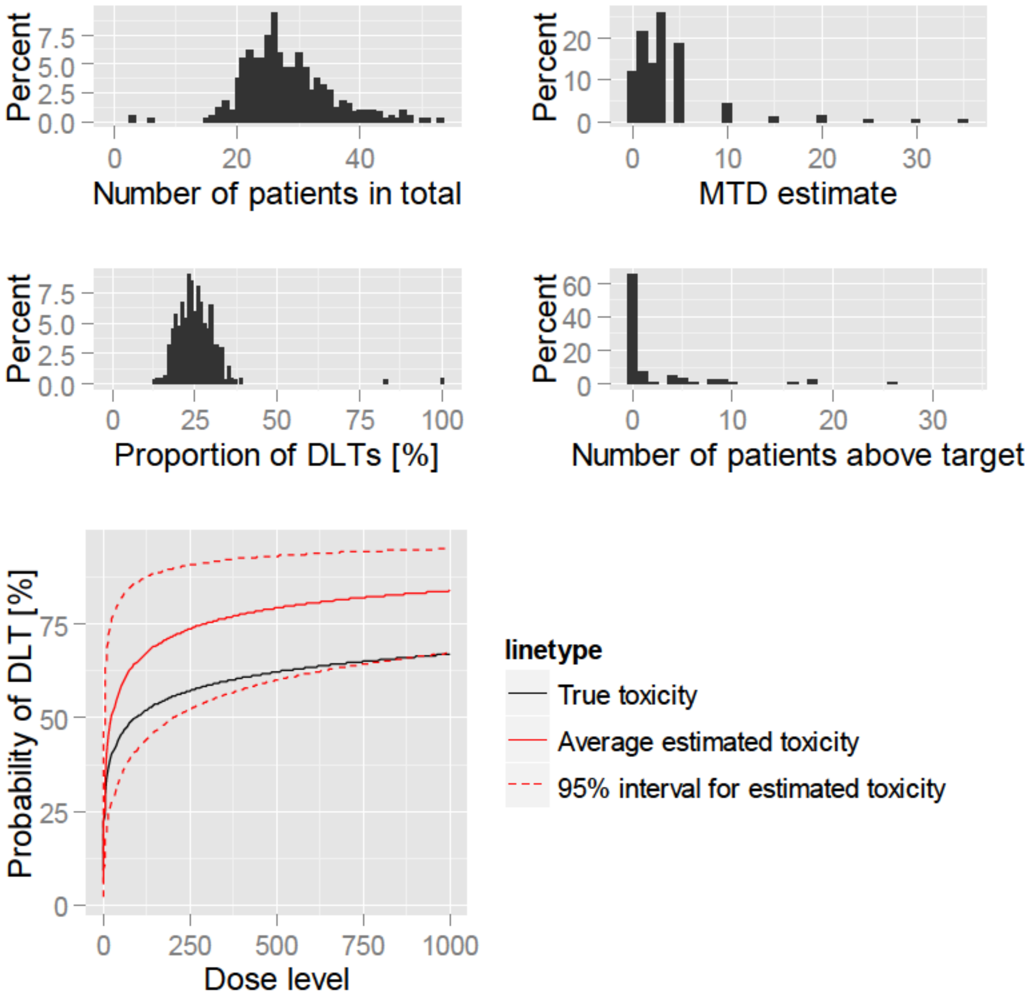

"Use of this document is governed by the terms of use on the first page of this document."

Figure 4 True versus Average Estimated Toxicity Curve in Scenario 3

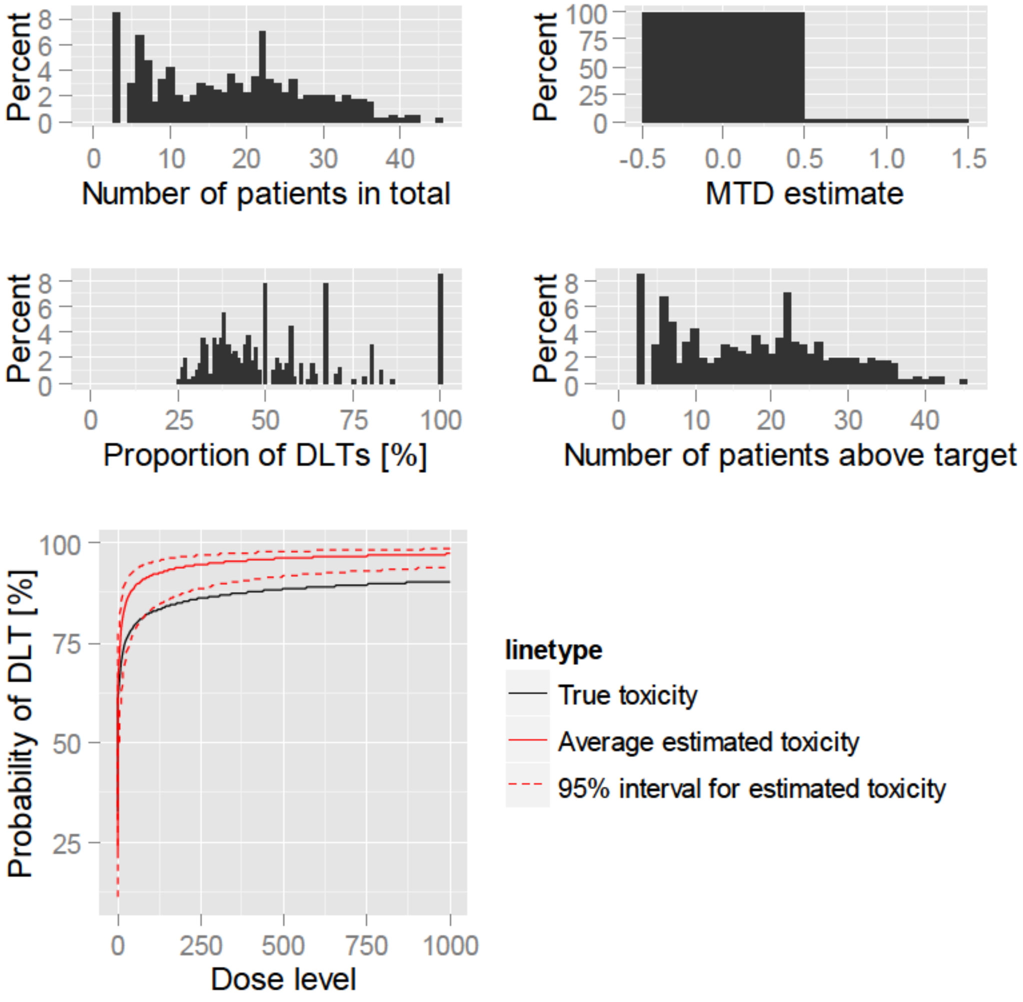

“Use of this document is governed by the terms of use on the first page of this document.”

Figure 5 True versus Average Estimated Toxicity Curve in Scenario 4

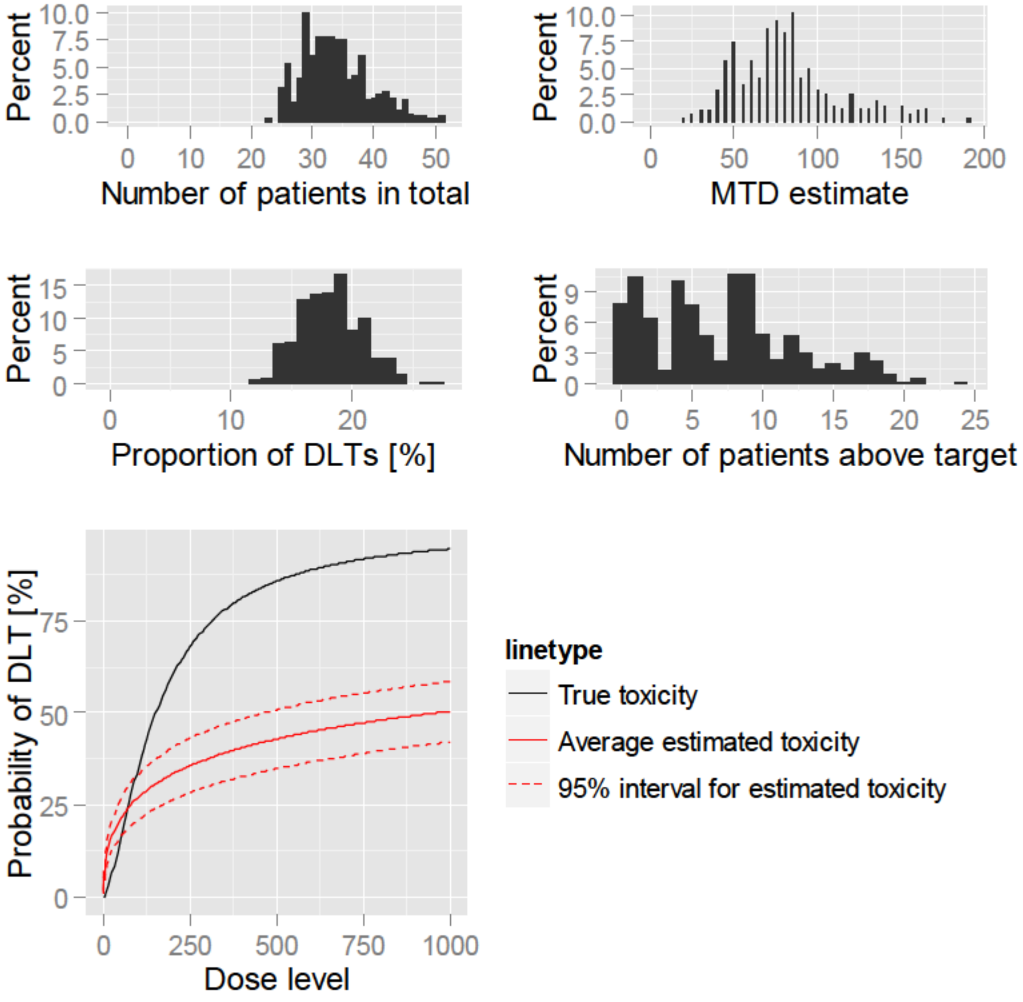

"Use of this document is governed by the terms of use on the first page of this document."

Appendix 6
Statistical Design of modified Continual Reassessment Method with Escalation with Overdose Control (cont.)

Figure 6 True versus Average Estimated Toxicity Curve in Scenario 5

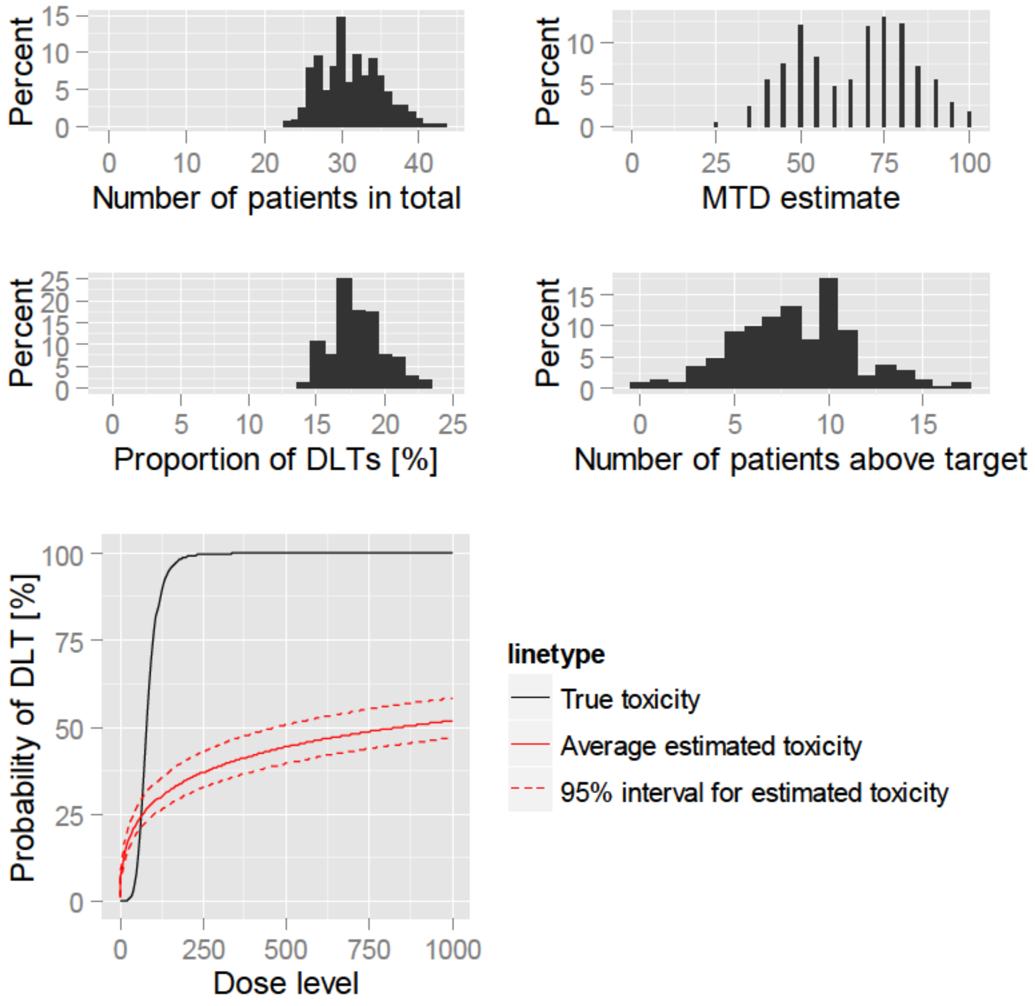

“Use of this document is governed by the terms of use on the first page of this document.”

Figure 7 True versus Average Estimated Toxicity Curve in Scenario 6

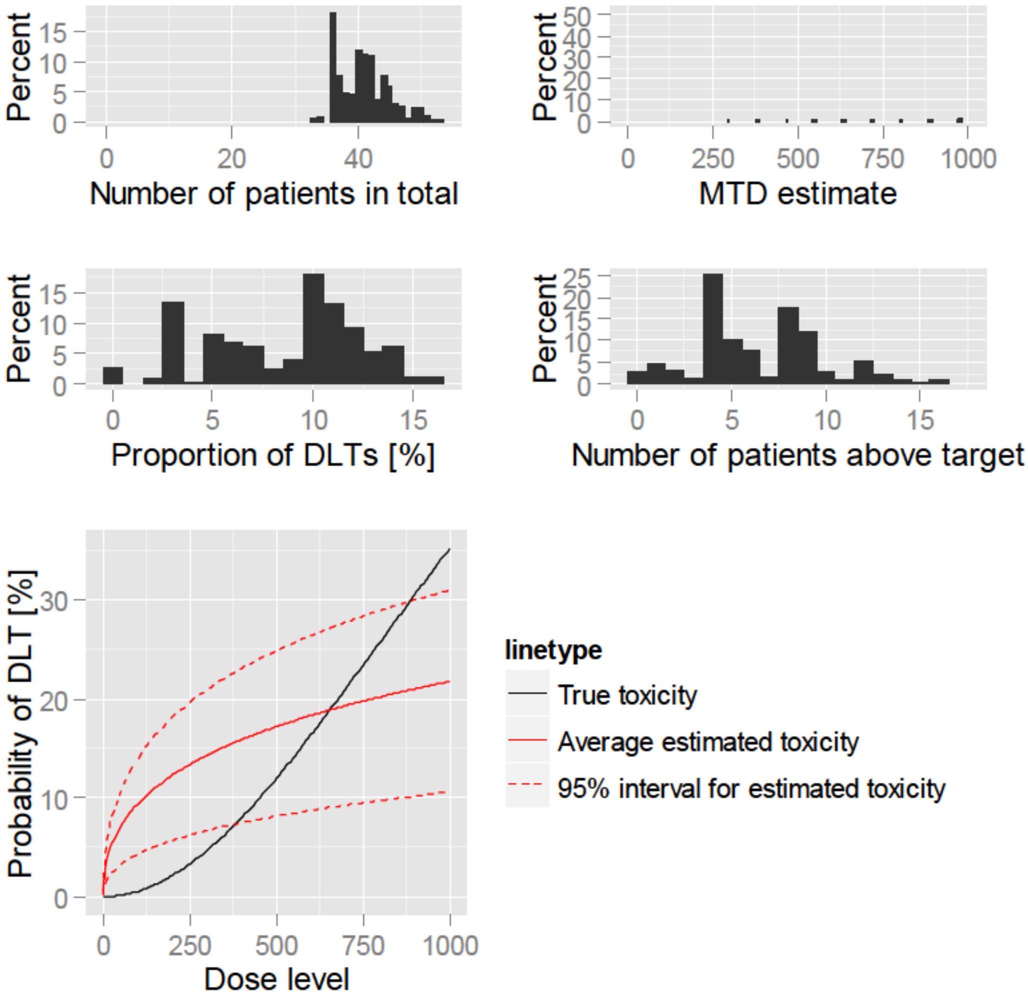

"Use of this document is governed by the terms of use on the first page of this document."

Figure 8 True versus Average Estimated Toxicity Curve in Scenario 7

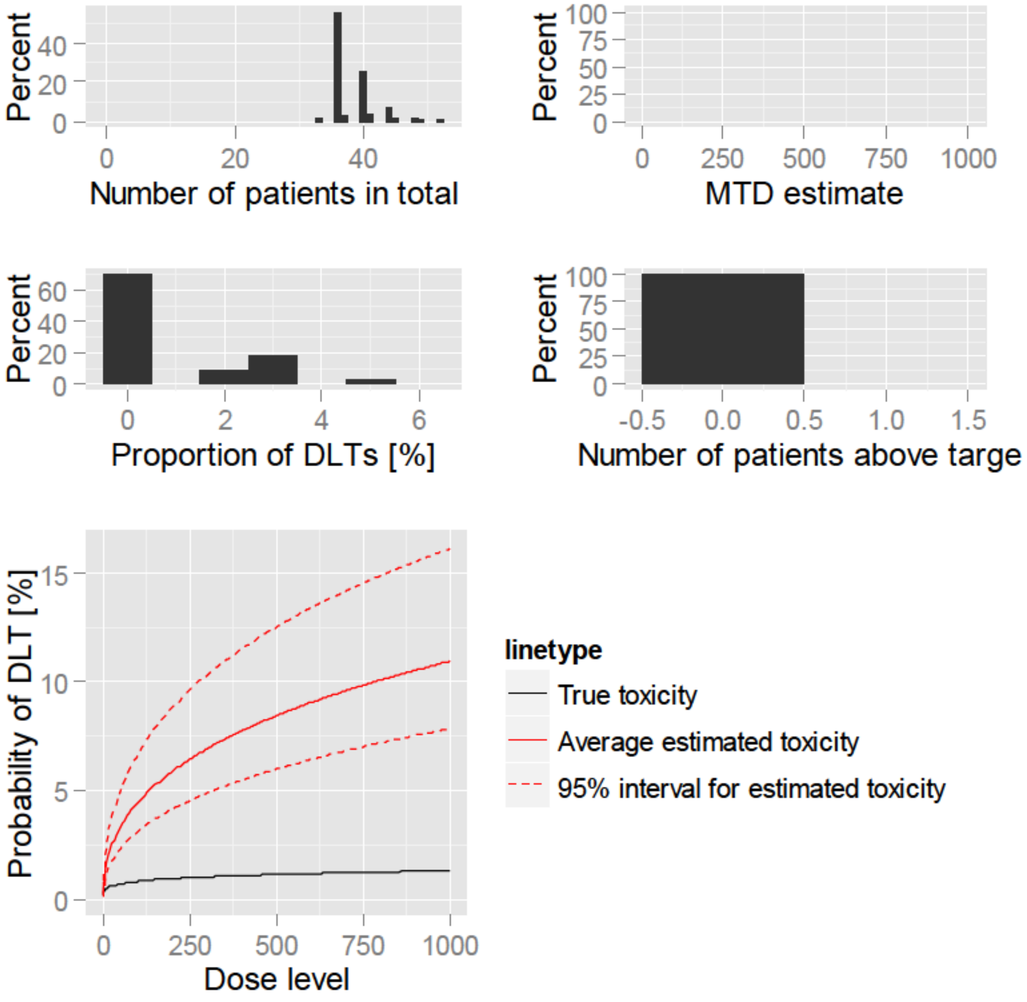

“Use of this document is governed by the terms of use on the first page of this document.”

Figure 9 True versus Average Estimated Toxicity Curve in Scenario 8

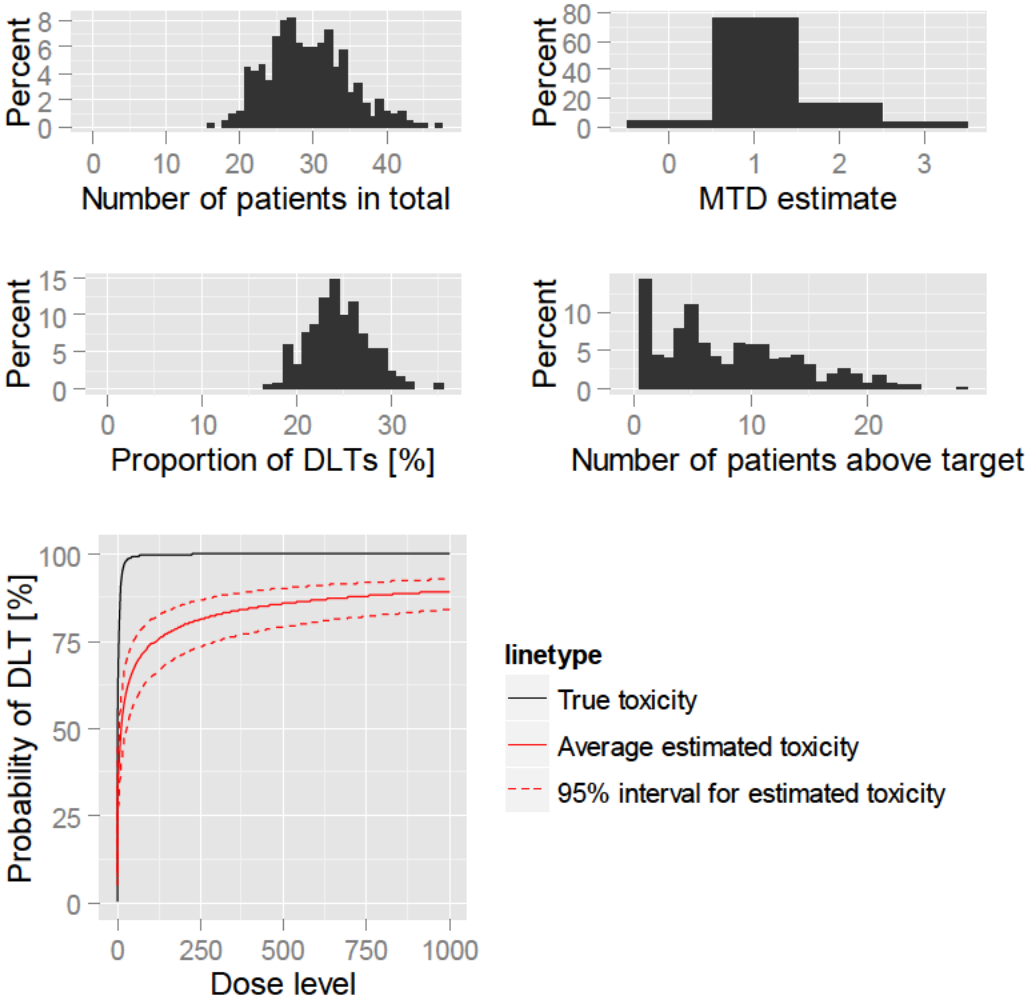

“Use of this document is governed by the terms of use on the first page of this document.”

Figure 10 True versus Average Estimated Toxicity Curve in Scenario 9

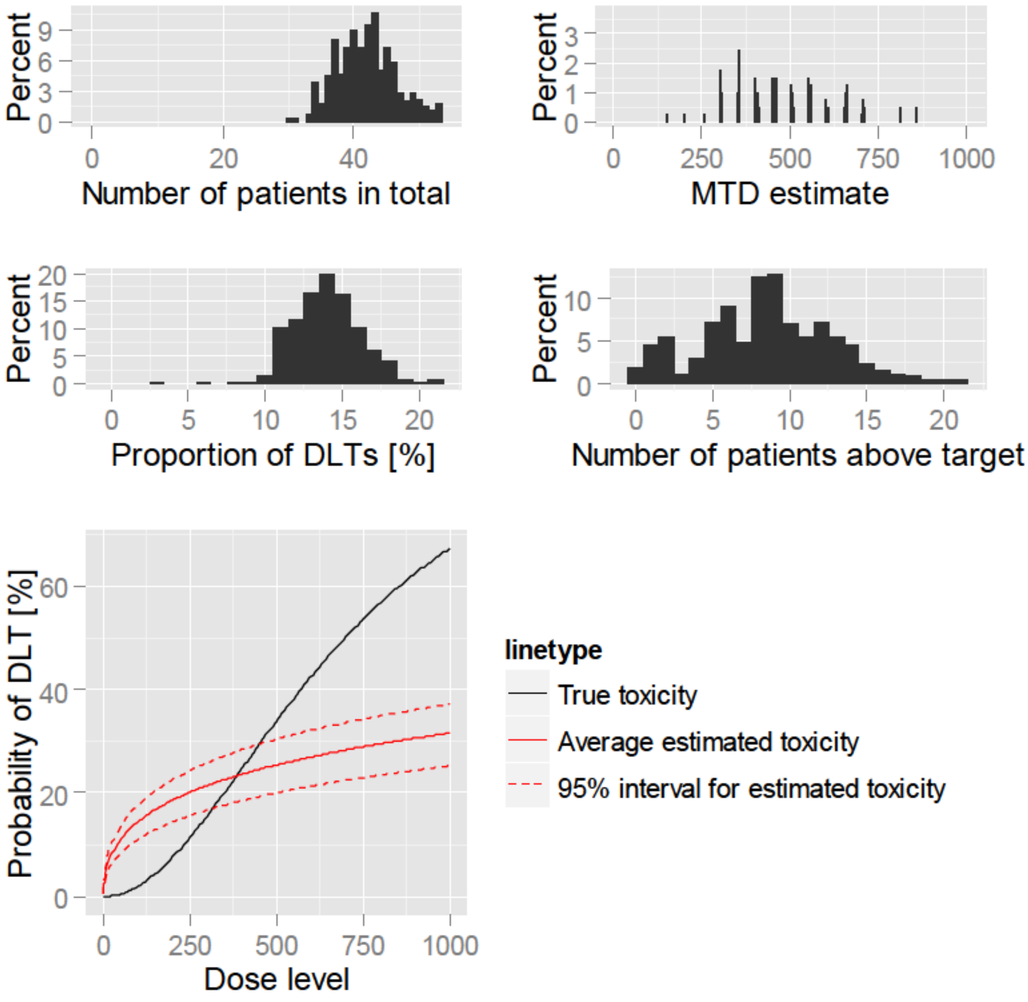

“Use of this document is governed by the terms of use on the first page of this document.”

Appendix 7
Intrapatient Dose Escalation Design Simulation Results

The intra-patient dose escalation design for cohort B**1** was tested in three scenarios, which featured low (150 mg), medium (600 mg) and high (1200 mg) late-cycle MTDs, respectively. The corresponding conditional probabilities of experiencing a DLT, given that no DLT appeared in lower dose levels, were defined as in Table 9. Please note that the mathematical definition of the late-cycle MTD is the highest dose level where the cumulative probability of experiencing a DLT at this or lower dose levels does not exceed 33%.

Table 9 Conditional probabilities of DLTs defining the scenarios

Dose:	40	150	300	600	900	1200
“Low”	0.1	0.2	0.3	0.4	0.5	0.5
“Medium”	0.01	0.02	0.04	0.15	0.3	0.4
“High”	0.01	0.01	0.01	0.01	0.04	0.05

The trials were executed as described in Section 6.7.1.2 with 6 patients in total (which is the minimum number of required evaluable patients), and 10,000 trials were simulated for each scenario. The performance of the intra-patient dose escalation design is summarized in the below figures. They comprise the scenario definition (cumulative DLT probabilities), the distribution of the final late-cycle MTD estimates, the number of weeks until it took to define the late cycle MTD, and the number of DLTs which occurred during the trial.

Overall, the design performs acceptably well. The true MTD is indeed the final MTD estimate in most of the simulations. The patients’ safety is protected, as even in the most severe toxicity scenario (low late cycle MTD), not more than 3 patients experience a DLT in more than 80% of all simulations.

“Use of this document is governed by the terms of use on the first page of this document.”

Appendix 7
Intrapatient dose escalation design simulation results (cont.)

Figure 1 Simulation results for the low late-cycle MTD scenario

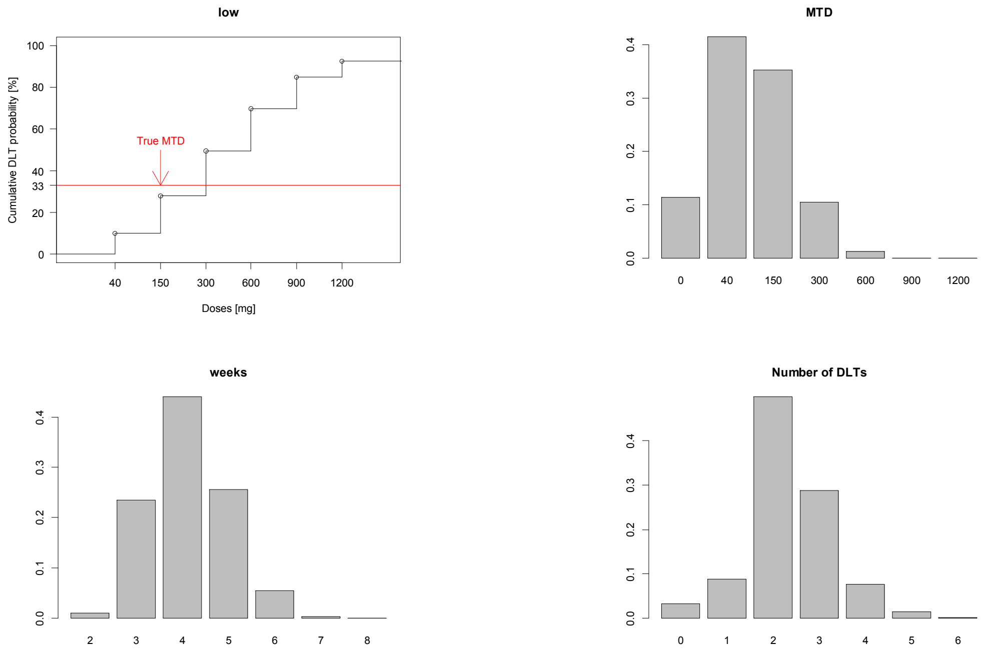

Figure 2 Simulation results for the medium late-cycle MTD scenario

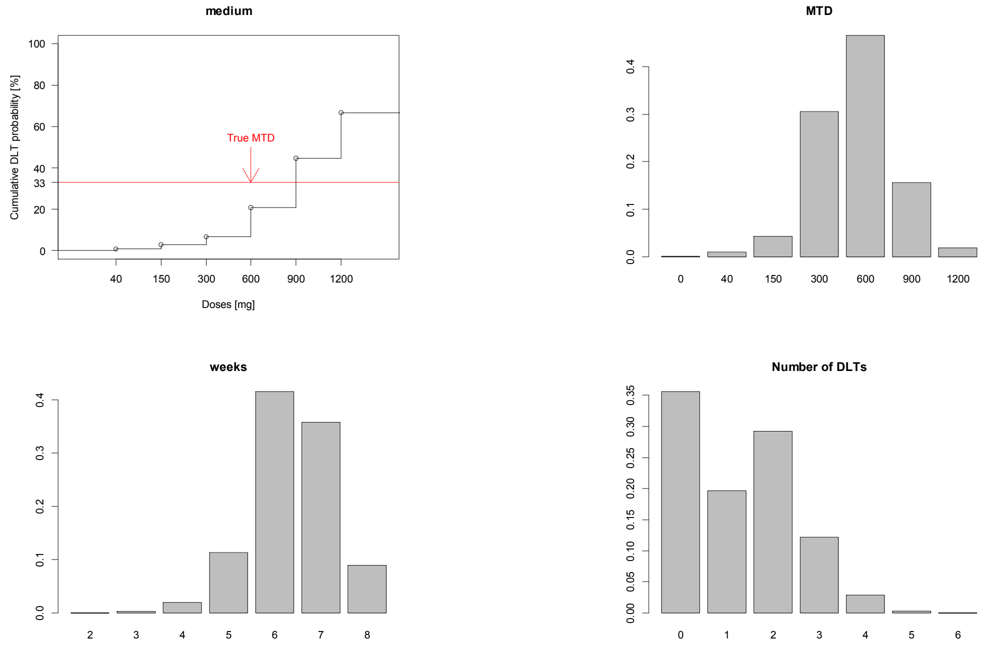

"Use of this document is governed by the terms of use on the first page of this document."

Appendix 7
Intrapatient dose escalation design simulation results (cont.)

Figure 3 Simulation results for the high late-cycle MTD scenario

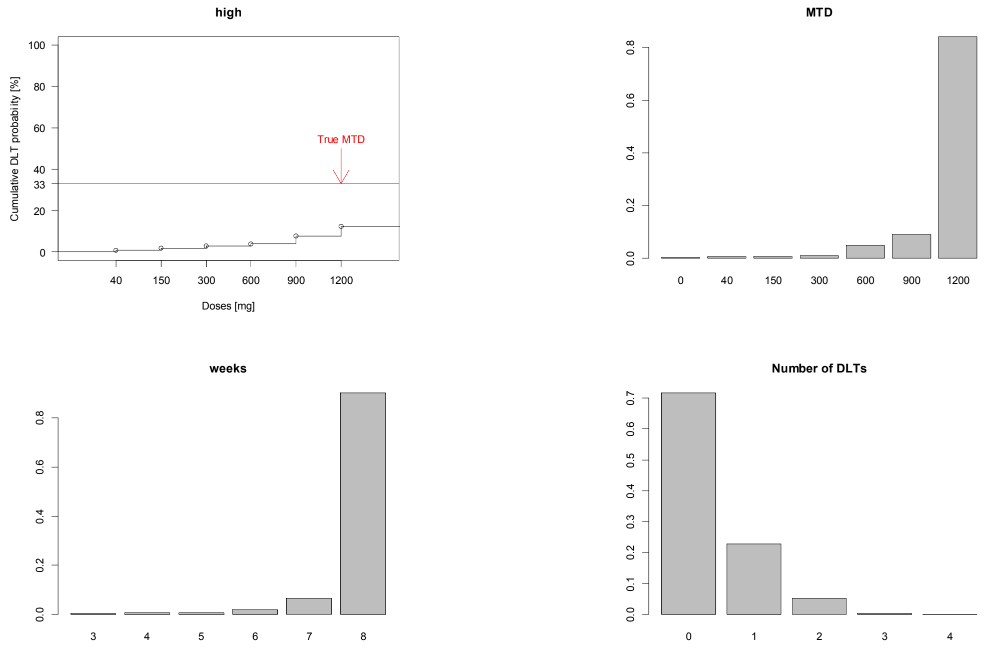

"Use of this document is governed by the terms of use on the first page of this document."

Appendix 8
Gilbert's Syndrome Definition

Patients with Gilbert's syndrome will be eligible for the study. The diagnosis of Gilbert's syndrome is suspected in people who have persistent, slightly elevated levels of unconjugated bilirubin without any other apparent cause. A diagnosis of Gilbert's syndrome will be based on the exclusion of other diseases based on the following criteria:

- Unconjugated hyperbilirubinemia noted on several occasions
- No evidence of hemolysis (normal hemoglobin, reticulocyte count and LDH)
- Normal liver function tests
- Absence of other diseases associated with unconjugated hyperbilirubinemia.

"Use of this document is governed by the terms of use on the first page of this document."

Appendix 9
[^{18}F]-FDG-PET

Introduction

FDG ([^{18}F]-Fluoro-Deoxy-Glucose) PET imaging will be performed in this study.

The patient needs to fast for typically 4 to 6 hours prior to the FDG-PET scan. The patient can drink water freely during the fast and should be encouraged to do so. He/she may also take regular medications as scheduled during the fast. Blood glucose level will be checked on the day of the FDG PET scan and results assessed prior to the administration of FDG. The patient should have a blood glucose level ≤ 180 mg/dL (≤ 10 mmol/L) in order to have the FDG-PET scan. If the level is higher, the scan should be rescheduled if possible.

Regular diet can be resumed after the scan.

The interval between FDG administration and scanning must be 60 (±10) minutes and it is particularly important that the time interval between injection and start of the scan is the same at follow-up compared to baseline.

Of note PET must be performed before biopsy to avoid potential false positive findings. During the screening period, baseline FDG-PET assessment should be performed at the closest date as possible from start of study treatment. All patients should be encouraged to increase fluid intake for a few hours after the scan to promote excretion of the FDG. A diuretic (furosemide, typically 20-40 mg IV) may be administered at the discretion of the investigator before or during the FDG-PET scan in order to accelerate elimination of the [^{18}F]-FDG from the renal collecting system.

Diazepam may be used to promote muscle relaxation and reduce muscular uptake if tumor deposits are in the neck or shoulder girdle area. Diazepam administration and diuretic administration must be recorded in the Concomitant Medications eCRF page.

Uniform image acquisition and analyses will be implemented across the study centers to minimize methodological variability. The scans will be analyzed by central reading and local assessment and parameters such as Standardized Uptake Value (SUV), Total Lesion Glycolysis (TLG) and Metabolic Tumor Volume (MTV) measured.

“Use of this document is governed by the terms of use on the first page of this document.”

Appendix 10
Overall Guidelines for Management of Patients Who Experience Adverse Events

MANAGEMENT GUIDELINES

Guidelines for management of patients who experience specific adverse events during treatment with RO6958688 and/or atezolizumab are provided in Table 1. These guidelines should be followed for both drugs for a given adverse event grade regardless of whether the adverse event is deemed related to atezolizumab, RO6958688, or both.

Guidelines for the management of patients who experience infusion-related reactions and cytokine-release syndrome events related to RO6958688 are provided in Table 2.

"Use of this document is governed by the terms of use on the first page of this document."

Appendix 10
Overall Guidelines for Management of Patients Who Experience Adverse Events (cont.)

Table 1 Guidelines for Management of Patients Who Experience Adverse Events Associated with RO6958688 and/or Atezolizumab

Event	Action to Be Taken
IRRs, CRS, and anaphylaxis	• Follow guidelines for atezolizumab in Appendix 11 for the management of IRRs related to atezolizumab.<br>• Follow guidelines for RO6958688 in Table 2 for the management of IRRs and CRS events related to RO6958688.<br>• For anaphylaxis precautions, see Appendix 14.
Grade 3 or higher treatment-related adverse events within 24 hours of previous infusion	• In the next treatment administration visit, after the end of RO6958688 infusion, patient will remain in the hospital for observation for 8 hours and vital signs will be checked as per Appendix 1.
Grade 3 IRR/CRS at previous infusion	• In the next treatment administration visit after the RO6958688 infusion, patient will remain in the hospital for observation for 24 hours and vital signs will be checked as per Appendix 1.

“Use of this document is governed by the terms of use on the first page of this document.”

Table 1 Guidelines for Management of Patients Who Experience Adverse Events Associated with RO6958688 and/or Atezolizumab (cont.)

Pulmonary events, excluding atezolizumab-related pneumonitis [a, b]	
Grade 1 or 2	• For Grade 1 events, continue atezolizumab and RO6958688.<br>• For Grade 2 events, withhold atezolizumab for up to 12 weeks after event onset and RO6958688 for up to 6 weeks after last administered dose.<br>• Monitor patient closely.<br>• If event resolves to Grade 1 or better, resume atezolizumab and RO6958688.<br>• For recurrent events, treat as a Grade 3 or 4 event.
Grade 3	• Permanently discontinue atezolizumab.<br>• Withhold RO6958688 for up to 6 weeks after last administered dose.<br>• Initiate treatment with corticosteroids equivalent to 2 mg/kg/day of IV or oral methylprednisone until resolution to Grade 1 or better. [c]<br>• If event does not improve within 48 hours after initiating corticosteroids, consider adding an immunosuppressive agent.<br>• If event resolves to Grade 1 or better, resume RO6958688.
Grade 4 [d]	• Permanently discontinue atezolizumab and RO6958688.<br>• Initiate treatment with corticosteroids equivalent to 2 mg/kg/day of IV or oral methylprednisone until resolution to Grade 1 or better. [c]<br>• If event does not improve within 48 hours after initiating corticosteroids, consider adding an immunosuppressive agent.

CRS = cytokine-release syndrome; IRR = infusion-related reaction.

[a] For hypoxia that occurs in the context of a RO6958688–treatment-related CRS event, please follow the IRR/CRS management guidelines in Table 2.

[b] Provide supportive care (e.g., oxygen support and intubation), as clinically indicated.

[c] If corticosteroids have been used for longer than 3 weeks, they must be tapered after event resolves to Grade 1 or better.

[d] Ensure patient has access to an intensive care unit.

“Use of this document is governed by the terms of use on the first page of this document.”

Table 1 Guidelines for Management of Patients Who Experience Adverse Events Associated with RO6958688 and/or Atezolizumab (cont.)

Event	Action to Be Taken
Atezolizumab-related pneumonitis	
Grade 1	• Follow guidelines for atezolizumab in Appendix 11.<br>• Continue RO6958688.
Grades ≥ 2	• Follow guidelines for atezolizumab in Appendix 11.<br>• Withhold RO6958688 for up to 6 weeks after last administered dose.<br>• If event resolves to Grade 1 or better, resume RO6958688.
Elevations in ALT and AST [a]	
AST/ALT > ULN to ≤ 3 × ULN	• Continue atezolizumab and RO6958688.<br>• Monitor LFTs weekly.
AST/ALT > 3 × ULN to < 5 × ULN	• Withhold atezolizumab for up to 12 weeks after event onset. [b]<br>• Withhold RO6958688 for up to 6 weeks after last administered dose.<br>• Monitor LFTs at least weekly until results return to normal or baseline.<br>• Consider patient referral to hepatologist.<br>• If event does not improve to AST/ALT ≤ 3 × ULN within 48 hours, initiate treatment with corticosteroids equivalent to 1 mg/kg/day of IV or oral methylprednisolone. [c]<br>• If event does not improve to AST/ALT ≤ 3 × ULN within 48 hours of treatment with corticosteroids equivalent to 1 mg/kg/day of corticosteroids, increase dose to 2 mg/kg/day IV or oral methylprednisolone (or equivalent). [c]<br>• If event resolves to < 3 × ULN, resume atezolizumab and RO6958688.

LFT = liver function test; ULN = upper limit of normal.

[a] For LFT elevations that occur in the context of a RO6958688–treatment-related CRS event, please follow the IRR/CRS management guidelines in Table 2.

[b] Atezolizumab may be withheld for a longer period of time (i.e., > 12 weeks after event onset) to allow for corticosteroids (if initiated) to be reduced to ≤ 10 mg/day oral prednisone or equivalent. The acceptable length of the extended period of time must be agreed upon by the investigator and the Medical Monitor.

[c] If corticosteroids have been used for longer than 3 weeks, they must be tapered before treatment can be resumed.

“Use of this document is governed by the terms of use on the first page of this document.”

Table 1 Guidelines for Management of Patients Who Experience Adverse Events Associated with RO6958688 and/or Atezolizumab (cont.)

Event	Action to Be Take
Elevations in ALT and AST (cont.) [a]	
AST/ALT $>5\times$ ULN to $<10\times$ ULN	• If event is considered related to atezolizumab, permanently discontinue atezolizumab.<br>• Withhold RO6958688 for up to 6 weeks after last administered dose.<br>• Monitor LFTs at least weekly until results return to normal or baseline.<br>• Consider patient referral to hepatologist.<br>• Initiate treatment with corticosteroids equivalent to 2 mg/kg/day of IV or oral methylprednisolone until resolution to AST/ALT $\leq 3\times$ ULN. [c]<br>• If event is considered related to atezolizumab and does not improve within 48 hours of corticosteroid treatment, consider adding an immunosuppressive agent.<br>• If event resolves to $<3\times$ ULN, resume RO6958688.
AST/ALT $\geq 10\times$ ULN	• Permanently discontinue atezolizumab and RO6958688.<br>• Monitor LFTs every other day until decreased to $<5\times$ ULN and then monitor at least weekly until results return to normal or baseline.<br>• Refer patient to hepatologist and consider liver biopsy to assess hepatic injury.<br>• Initiate treatment with corticosteroids equivalent to 2 mg/kg/day IV or oral methylprednisolone until improvement to AST/ALT $\leq 3\times$ ULN. [c]<br>• If event is considered related to atezolizumab and does not improve within 48 hours of corticosteroid treatment, consider adding an immunosuppressive agent.

LFT = liver function test; ULN = upper limit of normal.

[a] For LFT elevations that occur in the context of a RO6958688–treatment-related CRS event, please follow the IRR/CRS management guidelines.

[b] Atezolizumab may be withheld for a longer period of time (i.e., > 12 weeks after event onset) to allow for corticosteroids (if initiated) to be reduced to ≤ 10 mg/day oral prednisone or equivalent. The acceptable length of the extended period of time must be agreed upon by the investigator and the Medical Monitor.

[c] If corticosteroids have been used for longer than 3 weeks, they must be tapered before treatment can be resumed.

"Use of this document is governed by the terms of use on the first page of this document."

Table 1 Guidelines for Management of Patients Who Experience Adverse Events Associated with RO6958688 and/or Atezolizumab (cont.)

Event	Action to Be Take
Gastrointestinal events [a, b]	
Diarrhea, enteritis or colitis, Grade 1	• Continue atezolizumab and RO6958688.<br>• Initiate standard-of-care symptomatic treatment with anti-diarrheal agents (e.g., loperamide). [c]<br>• If diarrhea does not improve within 48 hours of anti-diarrheal agent treatment, initiate treatment with corticosteroids equivalent to 1 mg/kg/day IV or oral methylprednisolone. [e]<br>• If diarrhea does not improve within 48 hours of corticosteroid treatment, increase dose to 2 mg/kg/day IV or oral methylprednisolone (or equivalent). [e]<br>• Consider rectoscopy/colonoscopy with biopsy if symptoms of diarrhea persist for > 48 hours.

GI = gastrointestinal.

[a] For diarrhea that that occurs in the context of a RO6958688–treatment-related CRS event, please follow the IRR/CRS management guidelines in Table 2.

[b] Exclude infectious and other etiologies of diarrhea, enteritis, and colitis before following these management guidelines.

[c] Loperamide treatment should begin at an initial dose of 4 mg. After each loose stool, the dose may be increased by 4 mg to a maximum dose of 16 mg/day.

[d] Atezolizumab may be withheld for a longer period of time (i.e., > 12 weeks after event onset) to allow for corticosteroids (if initiated) to be reduced to ≤ 10 mg/day oral prednisone or equivalent. The acceptable length of the extended period of time must be agreed upon by the investigator and the Medical Monitor.

[e] If corticosteroids have been used for longer than 3 weeks, they must be tapered before treatment can be resumed.

[f] Resumption of atezolizumab may be considered in patients who are deriving clinical benefit and have fully recovered from the *immune-mediated* event. Patients can be re-challenged with atezolizumab only after approval has been documented by both the investigator (or an appropriate delegate) and the Medical Monitor.

"Use of this document is governed by the terms of use on the first page of this document."

Table 1 Guidelines for Management of Patients Who Experience Adverse Events Associated with RO6958688 and/or Atezolizumab (cont.)

Event	Action to Be Taken
Gastrointestinal events (cont.) [a, b]	
Diarrhea, enteritis or colitis, Grade 2	• Withhold atezolizumab for up to 12 weeks after event onset. [d]<br>• Withhold RO6958688 for up to 6 weeks after last administered dose.<br>• Initiate standard-of-care symptomatic treatment with anti-diarrheal agents (e.g., loperamide). [c]<br>• If diarrhea does not improve within 48 hours of anti-diarrheal agent treatment, initiate treatment with corticosteroids equivalent to 1 mg/kg/day IV or oral methylprednisolone. [e]<br>• If diarrhea does not improve within 48 hours of corticosteroid treatment, increase dose to 2 mg/kg/day IV or oral methylprednisolone (or equivalent). [e]<br>• If symptoms of diarrhea persist for > 48 hours, consider rectoscopy/colonoscopy with biopsy.<br>• Patient referral to GI specialist is recommended.<br>• If event resolves to Grade 1 or better, resume atezolizumab and RO6958688. [f]<br>• If event does not resolve to Grade 1 or better while withholding atezolizumab and RO6958688, permanently discontinue atezolizumab and RO6958688 and contact Medical Monitor. [f]

GI = gastrointestinal.

[a] For diarrhea that that occurs in the context of a RO6958688–treatment-related CRS event, please follow the IRR/CRS management guidelines.

[b] Exclude infectious and other etiologies of diarrhea, enteritis, and colitis before following these management guidelines.

[c] Loperamide treatment should begin at an initial dose of 4 mg. After each loose stool, the dose may be increased by 4 mg to a maximum dose of 16 mg/day.

[d] Atezolizumab may be withheld for a longer period of time (i.e., > 12 weeks after event onset) to allow for corticosteroids (if initiated) to be reduced to ≤ 10 mg/day oral prednisone or equivalent. The acceptable length of the extended period of time must be agreed upon by the investigator and the Medical Monitor.

[e] If corticosteroids have been initiated, they must be tapered before treatment can be resumed.

[f] Resumption of atezolizumab may be considered in patients who are deriving clinical benefit and have fully recovered from the *immune-mediated* event. Patients can be re-challenged with atezolizumab only after approval has been documented by both the investigator (or an appropriate delegate) and the Medical Monitor.

“Use of this document is governed by the terms of use on the first page of this document.”

Table 1 Guidelines for Management of Patients Who Experience Adverse Events Associated with RO6958688 and/or Atezolizumab (cont.)

Event	Action to Be Taken
Gastrointestinal events (cont.) [a, b]	
Diarrhea, enteritis or colitis, Grade 3	• Withhold atezolizumab for up to 12 weeks after event onset. [d]<br>• Withhold RO6958688 for up to 6 weeks after last administered dose.<br>• Refer patient to GI specialist for evaluation and confirmatory biopsy.<br>• Initiate treatment with corticosteroids equivalent to 2-mg/kg/day IV or oral methylprednisolone. [e]<br>• If event resolves to Grade 1 or better, resume atezolizumab and RO6958688. [f]<br>• If event does not resolve to Grade 1 or better within 24 hours, manage as a Grade 4 event.
Diarrhea, enteritis or colitis, Grade 4	• Permanently discontinue atezolizumab and RO6958688 and contact Medical Monitor.<br>• Refer patient to GI specialist for evaluation and confirmatory biopsy.<br>• Initiate treatment with corticosteroids equivalent to 2-mg/kg/day IV or oral methylprednisolone. [e]<br>• If event does not improve within 48 hours after initiating corticosteroids, consider adding an immunosuppressive agent (after rectoscopy/colonoscopy).

GI = gastrointestinal.

[a] For diarrhea that that occurs in the context of a RO6958688–treatment-related CRS event, please follow the IRR/CRS management guidelines.

[b] Exclude infectious and other etiologies of diarrhea, enteritis, and colitis before following these management guidelines.

[c] Loperamide treatment should begin at an initial dose of 4 mg. After each loose stool, the dose may be increased by 4 mg to a maximum dose of 16 mg/day.

[d] Atezolizumab may be withheld for a longer period of time (i.e., > 12 weeks after event onset) to allow for corticosteroids (if initiated) to be reduced to ≤ 10 mg/day oral prednisone or equivalent. The acceptable length of the extended period of time must be agreed upon by the investigator and the Medical Monitor.

[e] If corticosteroids have been used for longer than 3 weeks, they must be tapered before treatment can be resumed.

[f] Resumption of atezolizumab may be considered in patients who are deriving clinical benefit and have fully recovered from the *immune-mediated* event. Patients can be re-challenged with atezolizumab only after approval has been documented by both the investigator (or an appropriate delegate) and the Medical Monitor.

"Use of this document is governed by the terms of use on the first page of this document."

Table 1 Guidelines for Management of Patients Who Experience Adverse Events Associated with RO6958688 and/or Atezolizumab (cont.)

Event	Action to Be Taken
For Atezolizumab-related toxicity not described above	
Grade 1 or 2	• Follow guidelines for atezolizumab in Appendix 11.<br>• Continue RO6958688.
Grade 3 or 4	• Follow guidelines for atezolizumab in Appendix 11.<br>• Withhold RO6958688.<br>• If event resolves to Grade 2 or better within 6 weeks after last administered dose, resume RO6958688. If not, contact Medical Monitor to determine whether RO6958688 can be resumed.

"Use of this document is governed by the terms of use on the first page of this document."

GUIDELINES FOR MANAGEMENT OF INFUSION-RELATED REACTIONS AND CYTOKINE-RELEASE SYNDROME RELATED TO RO6958688

Guidelines for the management of patients who experience infusion-related reactions and cytokine-release syndrome events related to RO6958688 are provided below in Table 2.

Please note that infusion-related reactions (i.e., infusion-related events associated with the first and second RO6958688 infusion without hypotension or hypoxia as predominant symptoms) and cytokine-release syndrome events (i.e., infusion-related events occurring within 24 hours of the RO6958688 infusion with hypotension or hypoxia as the predominant symptom, and all infusion-related events associated with the third infusion of RO6958688 or later, irrespective of associated symptoms) should both be managed using the following guidance.

Infusion-related reactions (regardless of grade) without hypotension or hypoxia should be managed according to the Grade 1 cytokine-release syndrome guidelines below.

In patients with lung metastasis, hypoxia could also be secondary to tumor inflammation after the first and second dose of RO6958688. In such situations, please consider further evaluation with radiological imaging per clinical judgment, and if there is no associated hypotension, please follow guidelines in Table 2.

“Use of this document is governed by the terms of use on the first page of this document.”

Table 2 Guidelines for Management of Infusion-Related Reactions and Cytokine-Release Syndrome Related to RO6958688

Event	Action to Be Taken
CRS	
Grade 1<br>defined as fever, constitutional symptoms (first and second dose IRRs with no hypotension/hypoxia [see Section 5.2.6.2.4])	Immediate actions:<br>• If RO6958688 infusion is still ongoing, slow the infusion rate up to 50% or interrupt the infusion.<br>• Treat with antihistamines, antipyretics, and/or analgesics as clinically indicated.<br>• Monitor fluid balance and administer IV fluids as clinically indicated.<br>• If fluids are administered, manage as a Grade 2 event.<br>Restarting infusion:<br>• If RO6958688 infusion was interrupted, wait until 30 minutes after the event has resolved before restarting the infusion at 50% of the original infusion rate.<br>Next cycle:<br>• Pretreat with antihistamines, antipyretics/NSAIDS, and antiemetic medication. [a]

CRS = cytokine-release syndrome; CTCAE = Common Terminology Criteria for Adverse Events; IRR = infusion-related reaction; NCI = National Cancer Institute; NSAIDS = non-steroidal anti-inflammatory drugs.

Notes: Refer to NCI CTCAE v5 for a complete description of grading of symptoms.

[a] Guidelines for corticosteroid premedication refer only to the first cycle following the event. Premedication is not required for subsequent cycles if no event has occurred in the previous cycle. Please follow premedication guidance in Section 4.3.2.5 in addition to these guidelines.

“Use of this document is governed by the terms of use on the first page of this document.”

Table 2 Guidelines for Management of Infusion-Related Reactions and Cytokine-Release Syndrome Related to RO6958688 (cont.)

Event	Action to Be Taken
CRS (cont.)	
Grade 2<br>defined as hypotension (responding to fluids) OR hypoxia (requiring <40% FiO_2 to maintain adequate hemoglobin oxygen saturation) [a]	Immediate actions:<br>• If RO6958688 infusion is ongoing, stop the infusion immediately.<br>• Treat with antihistamines, antipyretics, and/or analgesics as clinically indicated.<br>• Consider treatment with IV corticosteroids (methylprednisolone 2 mg/kg/day or, if neurologic symptoms are present, dexamethasone 10 mg).<br>• Consider administering a single dose of tocilizumab IV 8 mg/kg (if administered, see Appendix 12).<br>• Monitor cardiac and other organ functions closely.<br>• Provide hemodynamic support as clinically indicated.<br>• Provide oxygen for hypoxia and fluids for hypotension.<br>• Collect all samples (including a sample for the cytokine panel) as described in Appendix 1, Table A3.<br>• Admit to ICU if clinically indicated and consider 24-hour hospitalization.<br>• If there is no improvement of hypotension or hypoxia within 24 hours, manage as a Grade 3 event.<br>• Notify the Medical Monitor.<br>Restarting infusion:<br>• Wait until 30 minutes after the event has resolved before restarting the infusion at up to 25% of the original infusion rate.<br>• If hypotension or hypoxia recurs, stop infusion immediately. RO6958688 should not be re-administered (re-started) again during this cycle.<br>• If hypotension or hypoxia recurs, manage as a Grade 3 event.

CRS = cytokine-release syndrome; CTCAE = Common Terminology Criteria for Adverse Events; FiO_2 = fraction of inspired oxygen; ICU = intensive care unit; IRR = infusion-related reaction; NCI = National Cancer Institute; NSAIDS = non-steroidal anti-inflammatory drugs.

Notes: Refer to NCI CTCAE v5 for a complete description of grading of symptoms.

[a] Guidelines for corticosteroid premedication refer only to the first cycle following the event. Premedication is not required for subsequent cycles if no event has occurred in the previous cycle. Please follow premedication guidance in Section 4.3.2.5 in addition to these guidelines.

“Use of this document is governed by the terms of use on the first page of this document.”

Table 2 Guidelines for Management of Infusion-Related Reactions and Cytokine-Release Syndrome Related to RO6958688 (cont.)

Event	Action to Be Taken
CRS (cont.)	
Grade 2 (cont.)<br>defined as hypotension (responding to fluids) OR hypoxia (requiring <40% FiO_2 to maintain adequate hemoglobin oxygen saturation) [a]	Next cycle:<br>• Patient may receive RO6958688 if symptoms resolve to Grade ≤ 1 and with approval of the Medical Monitor, as follows:<br>– Pretreat with antihistamines, antipyretics and/or analgesics as clinically indicated. [a]<br>– Pretreat with IV corticosteroids (methylprednisolone 80 mg or dexamethasone 16 mg) at least 60 minutes prior to the administration of RO6958688. [a]<br>– Administer RO6958688 at 50% of the initial infusion rate of the previous cycle.<br>Subsequent cycles:<br>• If there is an occurrence of IRR or CRS Grade ≥ 3 in any of the subsequent cycles, permanently discontinue RO6958688 regardless of recovery (see Grade 3 management guidelines).<br>• If there is an occurrence of a Grade ≤ 2 CRS in subsequent cycles, manage as indicated by severity (see Grade 1 or Grade 2 management guidelines).

CRS = cytokine-release syndrome; CTCAE = Common Terminology Criteria for Adverse Events; FiO_2 = fraction of inspired oxygen; ICU = intensive care unit; IRR = infusion-related reaction; NCI = National Cancer Institute; NSAIDS = non-steroidal anti-inflammatory drugs.

Notes: Refer to NCI CTCAE v5 for a complete description of grading of symptoms.

[a] Guidelines for corticosteroid premedication refer only to the first cycle following the event. Premedication is not required for subsequent cycles if no event has occurred in the previous cycle. Please follow premedication guidance in Section 4.3.2.5 in addition to these guidelines.

"Use of this document is governed by the terms of use on the first page of this document."

Table 2 Guidelines for Management of Infusion-Related Reactions and Cytokine-Release Syndrome Related to RO6958688 (cont.)

Event	Action to Be Taken
CRS (cont.)	
Grade 3<br>defined as hypotension (requiring one vasopressor [see Appendix 13 for definitions and calculations])<br>OR hypoxia (requiring ≥40% FiO_2 to maintain adequate hemoglobin oxygen saturation)	Immediate actions:<br>• If RO6958688 infusion is ongoing, stop the infusion immediately.<br>• Treat with antihistamines, antipyretics, and/or analgesics as clinically indicated.<br>• Treat with IV corticosteroids (methylprednisolone 2 mg/kg/day or, if neurologic symptoms are present, dexamethasone 10 mg).<br>• Administer tocilizumab IV 8 mg/kg (see Appendix 12).<br>If there is no improvement after 24 hours, repeat tocilizumab administration (see Appendix 12).<br>• Closely monitor and maintain fluid balance and administer IV fluids as clinically indicated.<br>• Provide oxygen for hypoxia.<br>• Provide vasopressor support for hypotension with high and repeated doses if required (see Appendix 13).<br>• Hospitalize patient for 24 hours.<br>• Perform a work up for organ functions (e.g., liver, cardiac) based on clinical assessment of the investigator.<br>• Cardiopulmonary and organ function monitoring in ICU is recommended.<br>• Collect all samples (including a sample for the cytokine panel) as described in Appendix 1, Table A3.<br>• Notify the Medical Monitor.<br>Restarting Infusion:<br>• RO6958688 should not be administered again during this cycle.

CRS = cytokine-release syndrome; CTCAE = Common Terminology Criteria for Adverse Events; FiO_2 = fraction of inspired oxygen; ICU = intensive care unit; IRR = infusion-related reaction; NCI = National Cancer Institute; NSAIDS = non-steroidal anti-inflammatory drugs.

Notes: Refer to NCI CTCAE v5 for a complete description of grading of symptoms.

[a] Guidelines for corticosteroid premedication refer only to the first cycle following the event. Premedication is not required for subsequent cycles if no event has occurred in the previous cycle. Please follow premedication guidance in Section 4.3.2.5 in addition to these guidelines.

"Use of this document is governed by the terms of use on the first page of this document."

Table 2 Guidelines for Management of Infusion-Related Reactions and Cytokine-Release Syndrome Related to RO6958688 (cont.)

Event	Action to Be Taken
CRS (cont.)	
Grade 3 (cont.)<br>defined as <u>hypotension</u> (managed with one vasopressor [see Appendix 13 for definitions and calculations])<br>OR <u>hypoxia</u> (requiring ≥40% FiO_2 to maintain adequate hemoglobin oxygen saturation)	Next Cycle/administration visit:<br>• If the patient had a Grade ≥2 IRR or CRS in any previous cycle, permanently discontinue RO6958688.<br>• If patient does not recover (is febrile or still on vasopressors) within 8 hours after corticosteroid and tocilizumab treatment, permanently discontinue RO6958688.<br>• If there is no improvement after 24 hours, repeat tocilizumab administration.<br>• If patient recovers (is afebrile and off vasopressors) within 8 hours following corticosteroid and tocilizumab treatment, RO6958688 can be administered in next cycle, as follows:<br>– Pretreat with antihistamines, antipyretics and/or analgesics as clinically indicated. [a]<br>– Pretreat with IV corticosteroids (methylprednisolone 80 mg or dexamethasone 16 mg) at least 60 minutes prior to the administration of RO6958688. [a]<br>– Hospitalize patient for 24 hours.<br>– Collect all samples (including a sample for the cytokine panel) as described in Appendix 1, Table A3.<br>• Administer RO6958688 at 50% of the initial infusion rate of the previous cycle.<br>• Hospitalize the patient for 24-hour observation<br>• Subsequent visit/cycles: If a Grade ≥3 CRS recurs, permanently discontinue RO6958688.<br>• If there is an occurrence of a Grade ≤2 CRS in subsequent cycles, manage as indicated by severity (i.e., Grade 1 or Grade 2 management guidelines).
Grade 4<br>defined as life threatening consequences; urgent intervention indicated (multiple vasopressors)	• Permanently discontinue RO6958688.<br>• Manage as a Grade 3 event.

CRS = cytokine-release syndrome; CTCAE = Common Terminology Criteria for Adverse Events; FiO_2 = fraction of inspired oxygen; ICU = intensive care unit; IRR = infusion-related reaction; NCI = National Cancer Institute; NSAIDS = non-steroidal anti-inflammatory drugs.

Notes: Refer to NCI CTCAE v5 for a complete description of grading of symptoms.

[a] Guidelines for corticosteroid premedication refer only to the first cycle following the event. Premedication is not required for subsequent cycles if no event has occurred in the previous cycle. Please follow premedication guidance in Section 4.3.2.5 in addition to these guidelines.

"Use of this document is governed by the terms of use on the first page of this document."

Appendix 11
Risks Associated with Atezolizumab and Guidelines for Management of Adverse Events Associated with Atezolizumab

Toxicities associated or possibly associated with atezolizumab treatment should be managed according to standard medical practice. Additional tests, such as autoimmune serology or biopsies, should be used to evaluate for a possible immunogenic etiology.

Although most *immune-mediated* adverse events observed with immunomodulatory agents have been mild and self-limiting, such events should be recognized early and treated promptly to avoid potential major complications. Discontinuation of atezolizumab may not have an immediate therapeutic effect, and in severe cases, *immune-mediated* toxicities may require acute management with topical corticosteroids, systemic corticosteroids, or other immunosuppressive agents.

The investigator should consider the benefit–risk balance a given patient may be experiencing prior to further administration of atezolizumab. In patients who have met the criteria for permanent discontinuation, resumption of atezolizumab may be considered if the patient is deriving benefit and has fully recovered from the *immune-mediated* event. Patients can be re-challenged with atezolizumab only after approval has been documented by both the investigator (or an appropriate delegate) and the Medical Monitor.

PULMONARY EVENTS

Dyspnea, cough, fatigue, hypoxia, pneumonitis, and pulmonary infiltrates have been associated with the administration of atezolizumab. Patients will be assessed for pulmonary signs and symptoms throughout the study and will also have computed tomography (CT) scans of the chest performed at every tumor assessment.

All pulmonary events should be thoroughly evaluated for other commonly reported etiologies such as pneumonia or other infection, lymphangitic carcinomatosis, pulmonary embolism, heart failure, chronic obstructive pulmonary disease, or pulmonary hypertension. Management guidelines for pulmonary events are provided in Table 1.

“Use of this document is governed by the terms of use on the first page of this document.”

Table 1 Management Guidelines for Pulmonary Events, Including Pneumonitis

Event	Management
Pulmonary event, Grade 1	• Continue atezolizumab and monitor closely.<br>• Re-evaluate on serial imaging.<br>• Consider patient referral to pulmonary specialist.
Pulmonary event, Grade 2	• Withhold atezolizumab for up to 12 weeks after event onset. [a]<br>• Refer patient to pulmonary and infectious disease specialists and consider bronchoscopy or BAL.<br>• Initiate treatment with 1–2 mg/kg/day oral prednisone or equivalent.<br>• If event resolves to Grade 1 or better, resume atezolizumab. [b]<br>• If event does not resolve to Grade 1 or better while withholding atezolizumab, permanently discontinue atezolizumab and contact Medical Monitor. [c]<br>• For recurrent events, treat as a Grade 3 or 4 event.
Pulmonary event, Grade 3 or 4	• Permanently discontinue atezolizumab and contact Medical Monitor. [c]<br>• Bronchoscopy or BAL is recommended.<br>• Initiate treatment with 1–2 mg/kg/day oral prednisone or equivalent.<br>• If event does not improve within 48 hours after initiating corticosteroids, consider adding an immunosuppressive agent.<br>• If event resolves to Grade 1 or better, taper corticosteroids over ≥ 1 month.

BAL = bronchoscopic alveolar lavage.

[a] Atezolizumab may be withheld for a longer period of time (i.e., > 12 weeks after event onset) to allow for corticosteroids (if initiated) to be reduced to ≤ 10 mg/day oral prednisone or equivalent. The acceptable length of the extended period of time must be agreed upon by the investigator and the Medical Monitor.

[b] If corticosteroids have been initiated, they must be tapered over ≥ 1 month to ≤ 10 mg/day oral prednisone or equivalent before atezolizumab can be resumed.

[c] Resumption of atezolizumab may be considered in patients who are deriving benefit and have fully recovered from the *immune-mediated* event. Patients can be re-challenged with atezolizumab only after approval has been documented by both the investigator (or an appropriate delegate) and the Medical Monitor.

"Use of this document is governed by the terms of use on the first page of this document."

HEPATIC EVENTS

Immune-mediated hepatitis has been associated with the administration of atezolizumab. Eligible patients must have adequate liver function, as manifested by measurements of total bilirubin and hepatic transaminases, and liver function will be monitored throughout study treatment. Management guidelines for hepatic events are provided in Table 2.

Patients with right upper-quadrant abdominal pain and/or unexplained nausea or vomiting should have liver function tests (LFTs) performed immediately and reviewed before administration of the next dose of study drug.

For patients with elevated LFTs, concurrent medication, viral hepatitis, and toxic or neoplastic etiologies should be considered and addressed, as appropriate.

Table 2 Management Guidelines for Hepatic Events

Event	Management
Hepatic event, Grade 1	• Continue atezolizumab.<br>• Monitor LFTs until values resolve to within normal limits.
Hepatic event, Grade 2	**All events:**<br>• Monitor LFTs more frequently until return to baseline values.<br>**Events of > 5 days' duration:**<br>• Withhold atezolizumab for up to 12 weeks after event onset. [a]<br>• Initiate treatment with 1–2 mg/kg/day oral prednisone or equivalent.<br>• If event resolves to Grade 1 or better, resume atezolizumab. [b]<br>• If event does not resolve to Grade 1 or better while withholding atezolizumab, permanently discontinue atezolizumab and contact Medical Monitor. [c]

LFT = liver function test.

[a] Atezolizumab may be withheld for a longer period of time (i.e., > 12 weeks after event onset) to allow for corticosteroids (if initiated) to be reduced to ≤ 10 mg/day oral prednisone or equivalent. The acceptable length of the extended period of time must be agreed upon by the investigator and the Medical Monitor.

[b] If corticosteroids have been initiated, they must be tapered over ≥ 1 month to ≤ 10 mg/day oral prednisone or equivalent before atezolizumab can be resumed.

[c] Resumption of atezolizumab may be considered in patients who are deriving benefit and have fully recovered from the *immune-mediated* event. Patients can be re-challenged with atezolizumab only after approval has been documented by both the investigator (or an appropriate delegate) and the Medical Monitor.

"Use of this document is governed by the terms of use on the first page of this document."

Table 2 Management Guidelines for Hepatic Events (cont.)

Event	Management
Hepatic event, Grade 3 or 4	• Permanently discontinue atezolizumab and contact Medical Monitor. [c]<br>• Consider patient referral to gastrointestinal specialist for evaluation and liver biopsy to establish etiology of hepatic injury.<br>• Initiate treatment with 1–2 mg/kg/day oral prednisone or equivalent.<br>• If event does not improve within 48 hours after initiating corticosteroids, consider adding an immunosuppressive agent.<br>• If event resolves to Grade 1 or better, taper corticosteroids over ≥1 month.

LFT = liver function test.

[a] Atezolizumab may be withheld for a longer period of time (i.e., > 12 weeks after event onset) to allow for corticosteroids (if initiated) to be reduced to ≤10 mg/day oral prednisone or equivalent. The acceptable length of the extended period of time must be agreed upon by the investigator and the Medical Monitor.

[b] If corticosteroids have been initiated, they must be tapered over ≥1 month to ≤10 mg/day oral prednisone or equivalent before atezolizumab can be resumed.

[c] Resumption of atezolizumab may be considered in patients who are deriving benefit and have fully recovered from the *immune-mediated* event. Patients can be re-challenged with atezolizumab only after approval has been documented by both the investigator (or an appropriate delegate) and the Medical Monitor.

GASTROINTESTINAL EVENTS

Immune-mediated colitis has been associated with the administration of atezolizumab. Management guidelines for diarrhea or colitis are provided in Table 3.

All events of diarrhea or colitis should be thoroughly evaluated for other more common etiologies. For events of significant duration or magnitude or associated with signs of systemic inflammation or acute-phase reactants (e.g., increased C-reactive protein, platelet count, or bandemia): Perform sigmoidoscopy (or colonoscopy, if appropriate) with colonic biopsy, with three to five specimens for standard paraffin block to check for inflammation and lymphocytic infiltrates to confirm colitis diagnosis.

“Use of this document is governed by the terms of use on the first page of this document.”

Table 3 Management Guidelines for Gastrointestinal Events (Diarrhea or Colitis)

Event	Management
Diarrhea or colitis, Grade 1	• Continue atezolizumab.<br>• Initiate symptomatic treatment.<br>• Endoscopy is recommended if symptoms persist for > 7 days.<br>• Monitor closely.
Diarrhea or colitis, Grade 2	• Withhold atezolizumab for up to 12 weeks after event onset. [a]<br>• Initiate symptomatic treatment.<br>• Patient referral to GI specialist is recommended.<br>• For recurrent events or events that persist > 5 days, initiate treatment with 1–2 mg/kg/day oral prednisone or equivalent.<br>• If event resolves to Grade 1 or better, resume atezolizumab. [b]<br>• If event does not resolve to Grade 1 or better while withholding atezolizumab, permanently discontinue atezolizumab and contact Medical Monitor. [c]
Diarrhea or colitis, Grade 3	• Withhold atezolizumab for up to 12 weeks after event onset. [a]<br>• Refer patient to GI specialist for evaluation and confirmatory biopsy.<br>• Initiate treatment with 1–2 mg/kg/day IV methylprednisolone or equivalent and convert to 1–2 mg/kg/day oral prednisone or equivalent upon improvement.<br>• If event resolves to Grade 1 or better, resume atezolizumab. [b]<br>• If event does not resolve to Grade 1 or better while withholding atezolizumab, permanently discontinue atezolizumab and contact Medical Monitor. [c]

GI = gastrointestinal.

[a] Atezolizumab may be withheld for a longer period of time (i.e., > 12 weeks after event onset) to allow for corticosteroids (if initiated) to be reduced to ≤ 10 mg/day oral prednisone or equivalent. The acceptable length of the extended period of time must be agreed upon by the investigator and the Medical Monitor.

[b] If corticosteroids have been initiated, they must be tapered over ≥ 1 month to ≤ 10 mg/day oral prednisone or equivalent before atezolizumab can be resumed.

[c] Resumption of atezolizumab may be considered in patients who are deriving benefit and have fully recovered from the *immune-mediated* event. Patients can be re-challenged with atezolizumab only after approval has been documented by both the investigator (or an appropriate delegate) and the Medical Monitor.

“Use of this document is governed by the terms of use on the first page of this document.”

Table 3 Management Guidelines for Gastrointestinal Events (Diarrhea or Colitis) (cont.)

Event	Management
Diarrhea or colitis, Grade 4	• Permanently discontinue atezolizumab and contact Medical Monitor. [c]<br>• Refer patient to GI specialist for evaluation and confirmation biopsy.<br>• Initiate treatment with 1–2 mg/kg/day IV methylprednisolone or equivalent and convert to 1–2 mg/kg/day oral prednisone or equivalent upon improvement.<br>• If event does not improve within 48 hours after initiating corticosteroids, consider adding an immunosuppressive agent.<br>• If event resolves to Grade 1 or better, taper corticosteroids over ≥1 month.

GI = gastrointestinal.

[a] Atezolizumab may be withheld for a longer period of time (i.e., > 12 weeks after event onset) to allow for corticosteroids (if initiated) to be reduced to ≤ 10 mg/day oral prednisone or equivalent. The acceptable length of the extended period of time must be agreed upon by the investigator and the Medical Monitor.

[b] If corticosteroids have been initiated, they must be tapered over ≥ 1 month to ≤ 10 mg/day oral prednisone or equivalent before atezolizumab can be resumed.

[c] Resumption of atezolizumab may be considered in patients who are deriving benefit and have fully recovered from the *immune-mediated* event. Patients can be re-challenged with atezolizumab only after approval has been documented by both the investigator (or an appropriate delegate) and the Medical Monitor.

ENDOCRINE EVENTS

Thyroid disorders, adrenal insufficiency, diabetes mellitus, and pituitary disorders have been associated with the administration of atezolizumab. Management guidelines for endocrine events are provided in Table 4.

Patients with unexplained symptoms such as headache, fatigue, myalgias, impotence, constipation, or mental status changes should be investigated for the presence of thyroid, pituitary, or adrenal endocrinopathies. The patient should be referred to an endocrinologist if an endocrinopathy is suspected. Thyroid-stimulating hormone (TSH) and free triiodothyronine and thyroxine levels should be measured to determine whether thyroid abnormalities are present. Pituitary hormone levels and function tests (e.g., TSH, growth hormone, luteinizing hormone, follicle-stimulating hormone, testosterone, prolactin, adrenocorticotropic hormone [ACTH] levels, and ACTH stimulation test) and magnetic resonance imaging (MRI) of the brain (with detailed pituitary sections) may help to differentiate primary pituitary insufficiency from primary adrenal insufficiency.

"Use of this document is governed by the terms of use on the first page of this document."

Table 4 Management Guidelines for Endocrine Events

Event	Management
Asymptomatic hypothyroidism	• Continue atezolizumab.<br>• Initiate treatment with thyroid replacement hormone.<br>• Monitor TSH weekly.
Symptomatic hypothyroidism	• Withhold atezolizumab.<br>• Initiate treatment with thyroid replacement hormone.<br>• Monitor TSH weekly.<br>• Consider patient referral to endocrinologist.<br>• Resume atezolizumab when symptoms are controlled and thyroid function is improving.
Asymptomatic hyperthyroidism	**TSH ≥ 0.1 mU/L and < 0.5 mU/L:**<br>• Continue atezolizumab.<br>• Monitor TSH every 4 weeks.<br>**TSH < 0.1 mU/L:**<br>• Follow guidelines for symptomatic hyperthyroidism.
Symptomatic hyperthyroidism	• Withhold atezolizumab.<br>• Initiate treatment with anti-thyroid drug such as methimazole or carbimazole as needed.<br>• Consider patient referral to endocrinologist.<br>• Resume atezolizumab when symptoms are controlled and thyroid function is improving.<br>• Permanently discontinue atezolizumab and contact Medical Monitor for life-threatening *immune-mediated* hyperthyroidism. [c]

MRI = magnetic resonance imaging; TSH = thyroid-stimulating hormone.

[a] Atezolizumab may be withheld for a longer period of time (i.e., > 12 weeks after event onset) to allow for corticosteroids (if initiated) to be reduced to ≤ 10 mg/day oral prednisone or equivalent. The acceptable length of the extended period of time must be agreed upon by the investigator and the Medical Monitor.

[b] If corticosteroids have been initiated, they must be tapered over ≥ 1 month to ≤ 10 mg/day oral prednisone or equivalent before atezolizumab can be resumed.

[c] Resumption of atezolizumab may be considered in patients who are deriving benefit and have fully recovered from the *immune-mediated* event. Patients can be re-challenged with atezolizumab only after approval has been documented by both the investigator (or an appropriate delegate) and the Medical Monitor.

“Use of this document is governed by the terms of use on the first page of this document.”

Table 4 Management Guidelines for Endocrine Events (cont.)

Event	Management
Symptomatic adrenal insufficiency, Grade 2–4	• Withhold atezolizumab for up to 12 weeks after event onset. [a]<br>• Refer patient to endocrinologist.<br>• Perform appropriate imaging.<br>• Initiate treatment with 1–2 mg/kg/day IV methylprednisolone or equivalent and convert to 1–2 mg/kg/day oral prednisone or equivalent upon improvement.<br>• If event resolves to Grade 1 or better and patient is stable on replacement therapy, resume atezolizumab. [b]<br>• If event does not resolve to Grade 1 or better or patient is not stable on replacement therapy while withholding atezolizumab, permanently discontinue atezolizumab and contact Medical Monitor. [c]
Hyperglycemia, Grade 1 or 2	• Continue atezolizumab.<br>• Initiate treatment with insulin if needed.<br>• Monitor for glucose control.
Hyperglycemia, Grade 3 or 4	• Withhold atezolizumab.<br>• Initiate treatment with insulin.<br>• Monitor for glucose control.<br>• Resume atezolizumab when symptoms resolve and glucose levels are stable.

MRI = magnetic resonance imaging; TSH = thyroid-stimulating hormone.

[a] Atezolizumab may be withheld for a longer period of time (i.e., > 12 weeks after event onset) to allow for corticosteroids (if initiated) to be reduced to ≤ 10 mg/day oral prednisone or equivalent. The acceptable length of the extended period of time must be agreed upon by the investigator and the Medical Monitor.

[b] If corticosteroids have been initiated, they must be tapered over ≥ 1 month to ≤ 10 mg/day oral prednisone or equivalent before atezolizumab can be resumed.

[c] Resumption of atezolizumab may be considered in patients who are deriving benefit and have fully recovered from the *immune-mediated* event. Patients can be re-challenged with atezolizumab only after approval has been documented by both the investigator (or an appropriate delegate) and the Medical Monitor.

"Use of this document is governed by the terms of use on the first page of this document."

Table 4 Management Guidelines for Endocrine Events (cont.)

Event	Management
Hypophysitis (pan-hypopituitarism), Grade 2 or 3	• Withhold atezolizumab for up to 12 weeks after event onset. [a]<br>• Refer patient to endocrinologist.<br>• Perform brain MRI (pituitary protocol).<br>• Initiate treatment with 1–2 mg/kg/day IV methylprednisolone or equivalent and convert to 1–2 mg/kg/day oral prednisone or equivalent upon improvement.<br>• Initiate hormone replacement if clinically indicated.<br>• If event resolves to Grade 1 or better, resume atezolizumab. [b]<br>• If event does not resolve to Grade 1 or better while withholding atezolizumab, permanently discontinue atezolizumab and contact Medical Monitor. [c]<br>• For recurrent hypophysitis, treat as a Grade 4 event.
Hypophysitis (pan-hypopituitarism), Grade 4	• Permanently discontinue atezolizumab and contact Medical Monitor. [c]<br>• Refer patient to endocrinologist.<br>• Perform brain MRI (pituitary protocol).<br>• Initiate treatment with 1–2 mg/kg/day IV methylprednisolone or equivalent and convert to 1–2 mg/kg/day oral prednisone or equivalent upon improvement.<br>• Initiate hormone replacement if clinically indicated.

MRI = magnetic resonance imaging; TSH = thyroid-stimulating hormone.

[a] Atezolizumab may be withheld for a longer period of time (i.e., > 12 weeks after event onset) to allow for corticosteroids (if initiated) to be reduced to ≤ 10 mg/day oral prednisone or equivalent. The acceptable length of the extended period of time must be agreed upon by the investigator and the Medical Monitor.

[b] If corticosteroids have been initiated, they must be tapered over ≥ 1 month to ≤ 10 mg/day oral prednisone or equivalent before atezolizumab can be resumed.

[c] Resumption of atezolizumab may be considered in patients who are deriving benefit and have fully recovered from the *immune-mediated* event. Patients can be re-challenged with atezolizumab only after approval has been documented by both the investigator (or an appropriate delegate) and the Medical Monitor.

"Use of this document is governed by the terms of use on the first page of this document."

Appendix 11
Risks Associated with Atezolizumab and Guidelines for Management of Adverse Events Associated with Atezolizumab (cont.)

OCULAR EVENTS

An ophthalmologist should evaluate visual complaints (e.g., uveitis, retinal events). Management guidelines for ocular events are provided in Table 5.

Table 5 Management Guidelines for Ocular Events

Event	Management
Ocular event, Grade 1	• Continue atezolizumab.<br>• Patient referral to ophthalmologist is strongly recommended.<br>• Initiate treatment with topical corticosteroid eye drops and topical immunosuppressive therapy.<br>• If symptoms persist, treat as a Grade 2 event.
Ocular event, Grade 2	• Withhold atezolizumab for up to 12 weeks after event onset. [a]<br>• Patient referral to ophthalmologist is strongly recommended.<br>• Initiate treatment with topical corticosteroid eye drops and topical immunosuppressive therapy.<br>• If event resolves to Grade 1 or better, resume atezolizumab. [b]<br>• If event does not resolve to Grade 1 or better while withholding atezolizumab, permanently discontinue atezolizumab and contact Medical Monitor. [c]
Ocular event, Grade 3 or 4	• Permanently discontinue atezolizumab and contact Medical Monitor. [c]<br>• Refer patient to ophthalmologist.<br>• Initiate treatment with 1–2 mg/kg/day oral prednisone or equivalent.<br>• If event resolves to Grade 1 or better, taper corticosteroids over ≥ 1 month.

[a] Atezolizumab may be withheld for a longer period of time (i.e., > 12 weeks after event onset) to allow for corticosteroids (if initiated) to be reduced to ≤ 10 mg/day oral prednisone or equivalent. The acceptable length of the extended period of time must be agreed upon by the investigator and the Medical Monitor.

[b] If corticosteroids have been initiated, they must be tapered over ≥ 1 month to ≤ 10 mg/day oral prednisone or equivalent before atezolizumab can be resumed.

[c] Resumption of atezolizumab may be considered in patients who are deriving benefit and have fully recovered from the *immune-mediated* event. Patients can be re-challenged with atezolizumab only after approval has been documented by both the investigator (or an appropriate delegate) and the Medical Monitor.

“Use of this document is governed by the terms of use on the first page of this document.”

IMMUNE-MEDIATED MYOCARDITIS

Immune-mediated myocarditis has been associated with the administration of atezolizumab. *Immune-mediated* myocarditis should be suspected in any patient presenting with signs or symptoms suggestive of myocarditis, including, but not limited to, *laboratory (e.g., B-type natriuretic peptide) or cardiac imaging abnormalities,* dyspnea, chest pain, palpitations, fatigue, decreased exercise tolerance, or syncope. *Immune-mediated* myocarditis needs to be distinguished from myocarditis resulting from infection (commonly viral, e.g., in a patient who reports a recent history of gastrointestinal illness), ischemic events, underlying arrhythmias, exacerbation of preexisting cardiac conditions, or progression of malignancy.

All patients with possible myocarditis should be urgently evaluated by performing cardiac enzyme assessment, an ECG, a chest X-ray, an echocardiogram, and a cardiac MRI as appropriate per institutional guidelines. A cardiologist should be consulted. An endomyocardial biopsy may be considered to enable a definitive diagnosis and appropriate treatment, if clinically indicated.

Patients with signs and symptoms of myocarditis, in the absence of an identified alternate etiology, should be treated according to the guidelines in Table 6.

"Use of this document is governed by the terms of use on the first page of this document."

Table 6 Management Guidelines for *Immune-Mediated* Myocarditis

Event	Management
Immune-mediated myocarditis, Grade 1	• Refer patient to cardiologist.<br>• Initiate treatment as per institutional guidelines.
Immune-mediated myocarditis, Grade 2	• Withhold atezolizumab for up to 12 weeks after event onset [a] and contact Medical Monitor.<br>• Refer patient to cardiologist.<br>• Initiate treatment as per institutional guidelines and consider antiarrhythmic drugs, temporary pacemaker, ECMO, or VAD as appropriate.<br>• Consider treatment with 1–2 mg/kg/day IV methylprednisolone or equivalent and convert to 1–2 mg/kg/day oral prednisone or equivalent upon improvement.[a]<br>• If event resolves to Grade 1 or better, resume atezolizumab. [b]<br>• If event does not resolve to Grade 1 or better while withholding atezolizumab, permanently discontinue atezolizumab and contact Medical Monitor. [c]
Immune-mediated myocarditis, Grade 3-4	• Permanently discontinue atezolizumab and contact Medical Monitor. [c]<br>• Refer patient to cardiologist.<br>• Initiate treatment as per institutional guidelines and consider antiarrhythmic drugs, temporary pacemaker, ECMO, or VAD as appropriate.<br>• Initiate treatment with 1–2 mg/kg/day IV methylprednisolone or equivalent and convert to 1–2 mg/kg/day oral prednisone or equivalent upon improvement. [a,b]<br>• If event does not improve within 48 hours after initiating corticosteroids, consider adding an immunosuppressive agent.<br>• If event resolves to Grade 1 or better, taper corticosteroids over ≥ 1 month.

ECMO = extracorporeal membrane oxygenation; VAD = ventricular assist device.

[a] Atezolizumab may be withheld for a longer period of time (i.e., > 12 weeks after event onset) to allow for corticosteroids (if initiated) to be reduced to ≤ 10 mg/day oral prednisone or equivalent. The acceptable length of the extended period of time must be agreed upon by the investigator and the Medical Monitor.

[b] If corticosteroids have been initiated, they must be tapered over ≥ 1 month to ≤ 10 mg/day oral prednisone or equivalent before atezolizumab can be resumed.

[c] Resumption of atezolizumab may be considered in patients who are deriving benefit and have fully recovered from the *immune-mediated* event. Patients can be re-challenged with atezolizumab only after approval has been documented by both the investigator (or an appropriate delegate) and the Medical Monitor.

“Use of this document is governed by the terms of use on the first page of this document.”

INFUSION-RELATED REACTIONS

No premedication is indicated for the administration of Cycle 1 of atezolizumab. However, patients who experience an infusion-related reaction (IRR) with Cycle 1 of atezolizumab may receive premedication with antihistamines or antipyretics/analgesics (e.g., acetaminophen) for subsequent infusions. Metamizole (dipyrone) is prohibited in treating atezolizumab-associated IRRs because of its potential for causing agranulocytosis.

Guidelines for medical management of IRRs during Cycle 1 are provided in Table 7. For subsequent cycles, IRRs should be managed according to institutional guidelines.

Table 7 Management Guidelines for Infusion-Related Reactions

Event	Management
IRR, Grade 1	• Reduce infusion rate to half the rate being given at the time of event onset.<br>• After the event has resolved, the investigator should wait for 30 minutes while delivering the infusion at the reduced rate.<br>• If the infusion is tolerated at the reduced rate for 30 minutes after symptoms have resolved, the infusion rate may be increased to the original rate.
IRR, Grade 2	• Interrupt atezolizumab infusion.<br>• Administer aggressive symptomatic treatment (e.g., oral or IV antihistamine, anti-pyretic medication, glucocorticoids, epinephrine, bronchodilators, oxygen, IV fluids).<br>• After symptoms have resolved to baseline, resume infusion at half the rate being given at the time of event onset.<br>• For subsequent infusions, consider administration of oral premedication with antihistamines, anti-pyretics, and/or analgesics and monitor closely for IRRs.
IRR, Grade 3 or 4	• Stop infusion.<br>• Administer aggressive symptomatic treatment (e.g., oral or IV antihistamine, anti-pyretic, glucocorticoids, epinephrine, bronchodilators, oxygen, IV fluids).<br>• Permanently discontinue atezolizumab and contact Medical Monitor. [a]

IRR = infusion-related reaction.

[a] Resumption of atezolizumab may be considered in patients who are deriving benefit and have fully recovered from the event. Patients can be re-challenged with atezolizumab only after approval has been documented by both the investigator (or an appropriate delegate) and the Medical Monitor.

"Use of this document is governed by the terms of use on the first page of this document."

PANCREATIC EVENTS

Symptoms of abdominal pain associated with elevations of amylase and lipase, suggestive of pancreatitis, have been associated with the administration of atezolizumab. The differential diagnosis of acute abdominal pain should include pancreatitis. Appropriate work-up should include an evaluation for ductal obstruction, as well as serum amylase and lipase tests. Management guidelines for pancreatic events, including pancreatitis, are provided in Table 8.

Table 8 Management Guidelines for Pancreatic Events, Including Pancreatitis

Event	Management
Amylase and/or lipase elevation, Grade 2	**Amylase and/or lipase > 1.5–2.0 × ULN:**<br>• Continue atezolizumab.<br>• Monitor amylase and lipase weekly.<br>• For prolonged elevation (e.g., > 3 weeks), consider treatment with 10 mg/day oral prednisone or equivalent.<br>**Asymptomatic with amylase and/or lipase > 2.0–5.0 × ULN:**<br>• Treat as a Grade 3 event.
Amylase and/or lipase elevation, Grade 3 or 4	• Withhold atezolizumab for up to 12 weeks after event onset. [a]<br>• Refer patient to GI specialist.<br>• Monitor amylase and lipase every other day.<br>• If no improvement, consider treatment with 1–2 mg/kg/day oral prednisone or equivalent.<br>• If event resolves to Grade 1 or better, resume atezolizumab. [b]<br>• If event does not resolve to Grade 1 or better while withholding atezolizumab, permanently discontinue atezolizumab and contact Medical Monitor. [c]<br>• For recurrent events, permanently discontinue atezolizumab and contact Medical Monitor. [c]

GI = gastrointestinal.

[a] Atezolizumab may be withheld for a longer period of time (i.e., > 12 weeks after event onset) to allow for corticosteroids (if initiated) to be reduced to ≤ 10 mg/day oral prednisone or equivalent. The acceptable length of the extended period of time must be agreed upon by the investigator and the Medical Monitor.

[b] If corticosteroids have been initiated, they must be tapered over ≥ 1 month to ≤ 10 mg/day oral prednisone or equivalent before atezolizumab can be resumed.

[c] Resumption of atezolizumab may be considered in patients who are deriving benefit and have fully recovered from the *immune-mediated* event. Patients can be re-challenged with atezolizumab only after approval has been documented by both the investigator (or an appropriate delegate) and the Medical Monitor.

“Use of this document is governed by the terms of use on the first page of this document.”

Appendix 11
Risks Associated with Atezolizumab and Guidelines for Management of Adverse Events Associated with Atezolizumab (cont.)

Table 8 Management Guidelines for Pancreatic Events, Including Pancreatitis (cont.)

Event	Management
Immune-mediated pancreatitis, Grade 2 or 3	• Withhold atezolizumab for up to 12 weeks after event onset. [a]<br>• Refer patient to GI specialist.<br>• Initiate treatment with 1–2 mg/kg/day IV methylprednisolone or equivalent and convert to 1–2 mg/kg/day oral prednisone or equivalent upon improvement.<br>• If event resolves to Grade 1 or better, resume atezolizumab. [b]<br>• If event does not resolve to Grade 1 or better while withholding atezolizumab, permanently discontinue atezolizumab and contact Medical Monitor. [c]<br>• For recurrent events, permanently discontinue atezolizumab and contact Medical Monitor. [c]
Immune-mediated pancreatitis, Grade 4	• Permanently discontinue atezolizumab and contact Medical Monitor. [c]<br>• Refer patient to GI specialist.<br>• Initiate treatment with 1–2 mg/kg/day IV methylprednisolone or equivalent and convert to 1–2 mg/kg/day oral prednisone or equivalent upon improvement.<br>• If event does not improve within 48 hours after initiating corticosteroids, consider adding an immunosuppressive agent.<br>• If event resolves to Grade 1 or better, taper corticosteroids over ≥ 1 month.

GI = gastrointestinal.

[a] Atezolizumab may be withheld for a longer period of time (i.e., > 12 weeks after event onset) to allow for corticosteroids (if initiated) to be reduced to ≤ 10 mg/day oral prednisone or equivalent. The acceptable length of the extended period of time must be agreed upon by the investigator and the Medical Monitor.

[b] If corticosteroids have been initiated, they must be tapered over ≥ 1 month to ≤ 10 mg/day oral prednisone or equivalent before atezolizumab can be resumed.

[c] Resumption of atezolizumab may be considered in patients who are deriving benefit and have fully recovered from the *immune-mediated* event. Patients can be re-challenged with atezolizumab only after approval has been documented by both the investigator (or an appropriate delegate) and the Medical Monitor.

“Use of this document is governed by the terms of use on the first page of this document.”

DERMATOLOGIC EVENTS

Treatment-emergent rash has been associated with atezolizumab. The majority of cases of rash were mild in severity and self limited, with or without pruritus. A dermatologist should evaluate persistent and/or severe rash or pruritus. A biopsy should be considered unless contraindicated. Management guidelines for dermatologic events are provided in Table 9.

Table 9 Management Guidelines for Dermatologic Events

Event	Management
Dermatologic event, Grade 1	• Continue atezolizumab.<br>• Consider treatment with topical corticosteroids and/or other symptomatic therapy (e.g., antihistamines).
Dermatologic event, Grade 2	• Continue atezolizumab.<br>• Consider patient referral to dermatologist.<br>• Initiate treatment with topical corticosteroids.<br>• Consider treatment with higher-potency topical corticosteroids if event does not improve.
Dermatologic event, Grade 3	• Withhold atezolizumab for up to 12 weeks after event onset. [a]<br>• Refer patient to dermatologist.<br>• Initiate treatment with 10 mg/day oral prednisone or equivalent, increasing dose to 1–2 mg/kg/day if event does not improve within 48–72 hours.<br>• If event resolves to Grade 1 or better, resume atezolizumab. [b]<br>• If event does not resolve to Grade 1 or better while withholding atezolizumab, permanently discontinue atezolizumab and contact Medical Monitor. [c]
Dermatologic event, Grade 4	• Permanently discontinue atezolizumab and contact Medical Monitor. [c]

[a] Atezolizumab may be withheld for a longer period of time (i.e., $>$ 12 weeks after event onset) to allow for corticosteroids (if initiated) to be reduced to $\leq$ 10 mg/day oral prednisone or equivalent. The acceptable length of the extended period of time must be agreed upon by the investigator and the Medical Monitor.

[b] If corticosteroids have been initiated, they must be tapered over $\geq$ 1 month to $\leq$ 10 mg/day oral prednisone or equivalent before atezolizumab can be resumed.

[c] Resumption of atezolizumab may be considered in patients who are deriving benefit and have fully recovered from the *immune-mediated* event. Patients can be re-challenged with atezolizumab only after approval has been documented by both the investigator (or an appropriate delegate) and the Medical Monitor.

"Use of this document is governed by the terms of use on the first page of this document."

NEUROLOGIC DISORDERS

Myasthenia gravis and Guillain-Barré syndrome have been observed with single-agent atezolizumab. Patients may present with signs and symptoms of sensory and/or motor neuropathy. Diagnostic work-up is essential for an accurate characterization to differentiate between alternative etiologies. Management guidelines for neurologic disorders are provided in Table 10.

Table 10 Management Guidelines for Neurologic Disorders

Event	Management
Immune-mediated neuropathy, Grade 1	• Continue atezolizumab.<br>• Investigate etiology.
Immune-mediated neuropathy, Grade 2	• Withhold atezolizumab for up to 12 weeks after event onset. [a]<br>• Investigate etiology.<br>• Initiate treatment as per institutional guidelines.<br>• If event resolves to Grade 1 or better, resume atezolizumab. [b]<br>• If event does not resolve to Grade 1 or better while withholding atezolizumab, permanently discontinue atezolizumab and contact Medical Monitor. [c]
Immune-mediated neuropathy, Grade 3 or 4	• Permanently discontinue atezolizumab and contact Medical Monitor. [c]<br>• Initiate treatment as per institutional guidelines.
Myasthenia gravis and Guillain-Barré syndrome (any grade)	• Permanently discontinue atezolizumab and contact Medical Monitor. [c]<br>• Refer patient to neurologist.<br>• Initiate treatment as per institutional guidelines.<br>• Consider initiation of 1–2 mg/kg/day oral or IV prednisone or equivalent.

[a] Atezolizumab may be withheld for a longer period of time (i.e., > 12 weeks after event onset) to allow for corticosteroids (if initiated) to be reduced to ≤ 10 mg/day oral prednisone or equivalent. The acceptable length of the extended period of time must be agreed upon by the investigator and the Medical Monitor.

[b] If corticosteroids have been initiated, they must be tapered over ≥ 1 month to ≤ 10 mg/day oral prednisone or equivalent before atezolizumab can be resumed.

[c] Resumption of atezolizumab may be considered in patients who are deriving benefit and have fully recovered from the *immune-mediated* event. Patients can be re-challenged with atezolizumab only after approval has been documented by both the investigator (or an appropriate delegate) and the Medical Monitor.

“Use of this document is governed by the terms of use on the first page of this document.”

IMMUNE-MEDIATED MENINGOENCEPHALITIS

Immune-mediated meningoencephalitis is an identified risk associated with the administration of atezolizumab. *Immune-mediated* meningoencephalitis should be suspected in any patient presenting with signs or symptoms suggestive of meningitis or encephalitis, including, but not limited to, headache, neck pain, confusion, seizure, motor or sensory dysfunction, and altered or depressed level of consciousness. Encephalopathy from metabolic or electrolyte imbalances needs to be distinguished from potential meningoencephalitis resulting from infection (bacterial, viral, or fungal) or progression of malignancy, or secondary to a paraneoplastic process.

All patients being considered for meningoencephalitis should be urgently evaluated with a CT scan and/or MRI scan of the brain to evaluate for metastasis, inflammation, or edema. If deemed safe by the treating physician, a lumbar puncture should be performed and a neurologist should be consulted.

Patients with signs and symptoms of meningoencephalitis, in the absence of an identified alternate etiology, should be treated according to the guidelines in Table 11.

Table 11 Management Guidelines for *Immune-Mediated* Meningoencephalitis

Event	Management
Immune-mediated meningoencephalitis, all grades	• Permanently discontinue atezolizumab and contact Medical Monitor. [a]<br>• Refer patient to neurologist.<br>• Initiate treatment with 1–2 mg/kg/day IV methylprednisolone or equivalent and convert to 1–2 mg/kg/day oral prednisone or equivalent upon improvement.<br>• If event does not improve within 48 hours after initiating corticosteroids, consider adding an immunosuppressive agent.<br>• If event resolves to Grade 1 or better, taper corticosteroids over ≥ 1 month.

[a] Resumption of atezolizumab may be considered in patients who are deriving benefit and have fully recovered from the *immune-mediated* event. Patients can be re-challenged with atezolizumab only after approval has been documented by both the investigator (or an appropriate delegate) and the Medical Monitor.

"Use of this document is governed by the terms of use on the first page of this document."

IMMUNE-MEDIATED NEPHRITIS

Immune-mediated nephritis has been associated with the administration of atezolizumab. Eligible patients must have adequate renal function. *Renal* function, including serum creatinine, should be monitored throughout study treatment. Patients with abnormal renal function should be evaluated and treated for other more common etiologies (including prerenal and postrenal causes, and concomitant medications such as non-steroidal anti-inflammatory drugs). Refer the patient to a renal specialist if clinically indicated. A renal biopsy may be required to enable a definitive diagnosis and appropriate treatment. If no alternative cause of acute kidney injury is identified, patients with signs and symptoms of acute kidney injury, in the absence of an identified alternate etiology, should be treated according to the management guidelines for *immune-mediated* renal events in Table 12.

“Use of this document is governed by the terms of use on the first page of this document.”

Table 12 Management Guidelines for Renal Events

Event	Management
Renal event, Grade 1	• Continue atezolizumab.<br>• Monitor kidney function, including creatinine, closely until values resolve to within normal limits or to baseline values.
Renal event, Grade 2	• Withhold atezolizumab for up to 12 weeks after event onset. [a]<br>• Refer patient to renal specialist.<br>• Initiate treatment with corticosteroids equivalent to 1–2 mg/kg/day oral prednisone.<br>• If event resolves to Grade 1 or better, resume atezolizumab. [b]<br>• If event does not resolve to Grade 1 or better while withholding atezolizumab, permanently discontinue atezolizumab and contact Medical Monitor. [c]
Renal event, Grade 3 or 4	• Permanently discontinue atezolizumab and contact Medical Monitor.<br>• Refer patient to renal specialist and consider renal biopsy.<br>• Initiate treatment with corticosteroids equivalent to 1–2 mg/kg/day oral prednisone.<br>• If event does not improve within 48 hours after initiating corticosteroids, consider adding an immunosuppressive agent.<br>• If event resolves to Grade 1 or better, taper corticosteroids over ≥ 1 month.

Note: Management guidelines are presented by adverse event severity based on NCI CTCAE and are applicable to both CTCAE Version 4.0 and CTCAE Version 5.0.

[a] Atezolizumab may be withheld for a longer period of time (i.e., > 12 weeks after event onset) to allow for corticosteroids (if initiated) to be reduced to the equivalent of ≤ 10 mg/day oral prednisone. The acceptable length of the extended period of time must be agreed upon by the investigator and the Medical Monitor.

[b] If corticosteroids have been initiated, they must be tapered over ≥ 1 month to the equivalent of ≤ 10 mg/day oral prednisone before atezolizumab can be resumed.

[c] Resumption of atezolizumab may be considered in patients who are deriving benefit and have fully recovered from the *immune-mediated* event. Patients can be re-challenged with atezolizumab only after approval has been documented by both the investigator (or an appropriate delegate) and the Medical Monitor.

“Use of this document is governed by the terms of use on the first page of this document.”

IMMUNE-MEDIATED MYOSITIS

Immune-mediated myositis has been associated with the administration of atezolizumab. Myositis or inflammatory myopathies are a group of disorders sharing the common feature of inflammatory muscle injury; dermatomyositis and polymyositis are among the most common disorders. Initial diagnosis is based on clinical (muscle weakness, muscle pain, skin rash in dermatomyositis), biochemical (serum creatine kinase increase), and imaging (electromyography/MRI) features, and is confirmed with a muscle biopsy.

Patients with signs and symptoms of myositis, in the absence of an identified alternate etiology, should be treated according to the guidelines in Table 13.

“Use of this document is governed by the terms of use on the first page of this document.”

Table 13 Management Guidelines for *Immune-Mediated* Myositis

Event	Management
Immune-mediated myositis, Grade 1	• Continue atezolizumab.<br>• Refer patient to rheumatologist or neurologist.<br>• Initiate treatment as per institutional guidelines.
Immune-mediated myositis, Grade 2	• Withhold atezolizumab for up to 12 weeks after event onset [a] and contact Medical Monitor.<br>• Refer patient to rheumatologist or neurologist.<br>• Initiate treatment as per institutional guidelines.<br>• Consider treatment with corticosteroids equivalent to 1–2 mg/kg/day IV methylprednisolone and convert to 1–2 mg/kg/day oral prednisone or equivalent upon improvement.<br>• If corticosteroids are initiated and event does not improve within 48 hours after initiating corticosteroids, consider adding an immunosuppressive agent.<br>• If event resolves to Grade 1 or better, resume atezolizumab. [b]<br>• If event does not resolve to Grade 1 or better while withholding atezolizumab, permanently discontinue atezolizumab and contact Medical Monitor. [c]

[a] Atezolizumab may be withheld for a longer period of time (i.e., > 12 weeks after event onset) to allow for corticosteroids (if initiated) to be reduced to the equivalent of ≤ 10 mg/day oral prednisone. The acceptable length of the extended period of time must be agreed upon by the investigator and the Medical Monitor.

[b] If corticosteroids have been initiated, they must be tapered over ≥ 1 month to the equivalent of ≤ 10 mg/day oral prednisone before atezolizumab can be resumed.

[c] Resumption of atezolizumab may be considered in patients who are deriving benefit and have fully recovered from the *immune-mediated* event. Patients can be re-challenged with atezolizumab only after approval has been documented by both the investigator (or an appropriate delegate) and the Medical Monitor.

“Use of this document is governed by the terms of use on the first page of this document.”

Table 13 Management Guidelines for *Immune-Mediated* Myositis (cont.)

Immune-mediated myositis, Grade 3	• Withhold atezolizumab for up to 12 weeks after event onset [a] and contact Medical Monitor.<br>• Refer patient to rheumatologist or neurologist.<br>• Initiate treatment as per institutional guidelines.<br>• Respiratory support may be required in more severe cases.<br>• Initiate treatment with corticosteroids equivalent to 1–2 mg/kg/day IV methylprednisolone, or higher-dose bolus if patient is severely compromised (e.g., cardiac or respiratory symptoms, dysphagia, or weakness that severely limits mobility); convert to 1–2 mg/kg/day oral prednisone or equivalent upon improvement.<br>• If event does not improve within 48 hours after initiating corticosteroids, consider adding an immunosuppressive agent.<br>• If event resolves to Grade 1 or better, resume atezolizumab. [b]<br>• If event does not resolve to Grade 1 or better while withholding atezolizumab, permanently discontinue atezolizumab and contact Medical Monitor. [c]<br>• For recurrent events, treat as a Grade 4 event.
Immune-mediated myositis, Grade 4	• Permanently discontinue atezolizumab and contact Medical Monitor. [c]<br>• Refer patient to rheumatologist or neurologist.<br>• Initiate treatment as per institutional guidelines.<br>• Respiratory support may be required in more severe cases.<br>• Initiate treatment with corticosteroids equivalent to 1–2 mg/kg/day IV methylprednisolone, or higher-dose bolus if patient is severely compromised (e.g., cardiac or respiratory symptoms, dysphagia, or weakness that severely limits mobility); convert to 1–2 mg/kg/day oral prednisone or equivalent upon improvement.<br>• If event does not improve within 48 hours after initiating corticosteroids, consider adding an immunosuppressive agent.<br>• If event resolves to Grade 1 or better, taper corticosteroids over ≥ 1 month.

[a] Atezolizumab may be withheld for a longer period of time (i.e., > 12 weeks after event onset) to allow for corticosteroids (if initiated) to be reduced to the equivalent of ≤ 10 mg/day oral prednisone. The acceptable length of the extended period of time must be agreed upon by the investigator and the Medical Monitor.

[b] If corticosteroids have been initiated, they must be tapered over ≥ 1 month to the equivalent of ≤ 10 mg/day oral prednisone before atezolizumab can be resumed.

[c] Resumption of atezolizumab may be considered in patients who are deriving benefit and have fully recovered from the *immune-mediated* event. Patients can be re-challenged with atezolizumab only after approval has been documented by both the investigator (or an appropriate delegate) and the Medical Monitor.

“Use of this document is governed by the terms of use on the first page of this document.”

HEMOPHAGOCYTIC LYMPHOHISTIOCYTOSIS AND MACROPHAGE ACTIVATION SYNDROME

Immune-mediated reactions may involve any organ system and may lead to hemophagocytic lymphohistiocytosis (HLH) and macrophage activation syndrome (MAS).

Patients with suspected HLH should be diagnosed according to published criteria by McClain and Eckstein (2014). A patient should be classified as having HLH if five of the following eight criteria are met:

- *Fever* $\geq$*38.5°C*
- *Splenomegaly*
- *Peripheral blood cytopenia consisting of at least two of the following:*
  - *Hemoglobin <90 g/L (9 g/dL) (<100 g/L [10 g/dL] for infants <4 weeks old)*
  - *Platelet count* $<100 \times 10^9$*/L (100,000/μL)*
  - *ANC* $<1.0 \times 10^9$*/L (1000/μL)*
- *Fasting triglycerides >2.992 mmol/L (265 mg/dL) and/or fibrinogen <1.5 g/L (150 mg/dL)*
- *Hemophagocytosis in bone marrow, spleen, lymph node, or liver*
- *Low or absent natural killer cell activity*
- *Ferritin >500 mg/L (500 ng/mL)*
- *Soluble interleukin 2 (IL-2) receptor (soluble CD25) elevated* $\geq$*2 standard deviations above age-adjusted laboratory-specific norms*

Patients with suspected MAS should be diagnosed according to published criteria for systemic juvenile idiopathic arthritis by Ravelli et al. (2016). A febrile patient should be classified as having MAS if the following criteria are met:

- *Ferritin >684 mg/L (684 ng/mL)*
- *At least two of the following:*
  - *Platelet count* $\leq 181 \times 10^9$*/L (181,000/μL)*
  - *AST* $\geq$*48 U/L*
  - *Triglycerides >1.761 mmol/L (156 mg/dL)*
  - *Fibrinogen* $\leq$*3.6 g/L (360 mg/dL)*

"Use of this document is governed by the terms of use on the first page of this document."

Patients with suspected HLH or MAS should be treated according to the guidelines in Table 14.

Table 14 Management Guidelines for Suspected Hemophagocytic Lymphohistiocytosis or Macrophage Activation Syndrome

Event	*Management*
Suspected HLH or MAS	• *Permanently discontinue atezolizumab and contact Medical Monitor.*<br>• *Consider patient referral to hematologist.*<br>• *Initiate supportive care, including intensive care monitoring if indicated per institutional guidelines.*<br>• *Consider initiation of IV corticosteroids and/or an immunosuppressive agent.*<br>• *If event does not improve within 48 hours after initiating corticosteroids, consider adding an immunosuppressive agent.*<br>• *If event resolves to Grade 1 or better, taper corticosteroids over ≥1 month.*

HLH =hemophagocytic lymphohistiocytosis; MAS =macrophage activation syndrome.

REFERENCES

McClain KL, Eckstein O. Clinical features and diagnosis of hemophagocytic lymphohistiocytosis. Up to Date [resource on the Internet]. 2014 [updated 29 October 2018; cited: 17 May 2019]. Available from: https://www.uptodate.com/contents/clinical-features-and-diagnosis-of-hemophagocytic-lymphohistiocytosis.

Ravelli A, Minoia F, Davi S, et al. 2016 classification criteria for macrophage activation syndrome complicating systemic juvenile idiopathic arthritis: a European League Against Rheumatism/American College of Rheumatology/Paediatric Rheumatology International Trials Organisation Collaborative Initiative. Ann Rheum Dis 2016;75:481–9.

"Use of this document is governed by the terms of use on the first page of this document."

Appendix 12 Schedule of Assessments for Tocilizumab Treatment of Severe or Life-Threatening Cytokine Release Syndrome

Assessment/Procedure	Pre-TCZ Treatment (within 24 hours)	TCZ Administration	Post-TCZ Treatment [c]		
			End of infusion	2 hours	24 hours
TCZ Administration (8 mg/kg)		x			
Vital signs [a]	x [g]		Measure at least every 6 hours until resolution to baseline [g]		
Pressor documentation [b]	x [g]		Record at least every 6 hours until pressors are discontinued [g]		
FiO_2	x [g]		Record at least every 6 hours until patient on room air [g]		
Pulse oximetry, resting	x [g]		Measure at least every 6 hours until resolution to baseline [g]		
Local Laboratory Assessments					
Hematology	x		x	x	x
Liver function tests (AST, ALT, total bilirubin)	x		x	x	x
Serum chemistry and creatinine [d]	x		x	x	x
CRP, LDH, and serum ferritin	x		x	x	x
Coagulation (aPTT, PT/INR, fibrinogen)	x		x	x	x
Infection workup [e]	x				
Central Laboratory Assessments					
Plasma cytokines	x		x	x	x
Plasma IL-6 pharmacodynamic markers [f]	x	x	x	x	x

"Use of this document is governed by the terms of use on the first page of this document."

Appendix 12
Schedule of Assessments for Tocilizumab Treatment of Severe or Life-Threatening Cytokine Release Syndrome (cont.)

aPTT = activated partial thromboplastin time; CRP = C-reactive protein; CRS = cytokine release syndrome; eCRF = electronic Case Report Form; INR = international normalized ratio; IL-6 = interleukin 6; LDH = lactate dehydrogenase; PT = prothrombin time; TCZ = tocilizumab.

Record abnormalities or worsened clinically significant abnormalities on the Adverse Event eCRF.

[a] Includes respiratory rate, heart rate, and systolic and diastolic blood pressure while the patient is in a seated or supine position, and temperature.

[b] Document vasopressor type and dose in the concomitant medication eCRF.

[c] If TCZ dose is repeated, follow Schedule of Assessments following the second TCZ dose.

[d] Includes sodium, potassium, chloride, bicarbonate, glucose and blood urea nitrogen

[e] Includes assessment for bacterial, fungal, and viral infections.

[f] Includes IL-6, and other cytokines such as soluble IL-6R, sgp130, IL-8 etc.

[g] The maximum and minimum values for any 24-hour period should be recorded in the clinical database.

"Use of this document is governed by the terms of use on the first page of this document."

Appendix 13
Vasopressor Use for CRS

High-Dose Vasopressor [a] (duration ≥3 hours)	
**Pressor**	**Dose**
Norepinephrine monotherapy	≥ 20 mcg/min
Dopamine monotherapy	≥ 10 mcg/kg/min
Phenylephrine monotherapy	≥ 200 mcg/min
Epinephrine monotherapy	≥ 10 mcg/min
If on vasopressin	Vasopressin + norepinephrine equivalent of ≥ 10 mcg/min [b]
If on combination vasopressors (not vasopressin)	Norepinephrine equivalent of ≥ 20 mcg/min [b]

mcg = microgram; min = minute; VASST = Vasopressin and Septic Shock Trial.

Source: Russell et al. N Engl J Med 2008;358:877–88.

[a] High-Dose Vasopressor: as defined in table above (low-dose vasopressor defined as single vasopressor at doses below those shown in table above).

[b] VASST vasopressor equivalent equation: norepinephrine equivalent dose = [norepinephrine (mcg/min)] + [dopamine (mcg/kg/min)] + [phenylephrine (mcg/min) ÷ 10].

“Use of this document is governed by the terms of use on the first page of this document.”

Appendix 14
Anaphylaxis Precautions

EQUIPMENT NEEDED

- Oxygen
- Epinephrine for subcutaneous, intravenous, and/or endotracheal use in accordance with standard practice
- Antihistamines
- Corticosteroids
- Intravenous infusion solutions, tubing, catheters, and tape

PROCEDURES

In the event of a suspected anaphylactic reaction during study treatment infusion, the following procedures should be performed:

1. Stop the study treatment infusion.
2. Maintain an adequate airway.
3. Administer antihistamines, epinephrine, or other medications as required by patient status and directed by the physician in charge.
4. Continue to observe the patient and document observations.

"Use of this document is governed by the terms of use on the first page of this document."
