# Supplementary material for: CEA-CD3 bispecific antibody cibisatamab with or without atezolizumab in patients with CEA-positive solid tumours: results of two multi-institutional Phase 1 trials
Source: Nat Commun. 2024 May 15;15:4091. doi: 10.1038/s41467-024-48479-8 (PMC11096172; doi:10.1038/s41467-024-48479-8)
Supplement: Supplementary file 3 — Reporting Summary [file 41467_2024_48479_MOESM3_ESM.pdf]

nature portfolio

Corresponding author(s): Neil H. Segal

Last updated by author(s): Apr 29, 2024

Reporting Summary

Nature Portfolio wishes to improve the reproducibility of the work that we publish. This form provides structure for consistency and transparency in reporting. For further information on Nature Portfolio policies, see our Editorial Policies and the Editorial Policy Checklist.

Statistics

For all statistical analyses, confirm that the following items are present in the figure legend, table legend, main text, or Methods section.

n/a	Confirmed	
☐	☒	The exact sample size (*n*) for each experimental group/condition, given as a discrete number and unit of measurement
☐	☒	A statement on whether measurements were taken from distinct samples or whether the same sample was measured repeatedly
☐	☒	The statistical test(s) used AND whether they are one- or two-sided<br>*Only common tests should be described solely by name; describe more complex techniques in the Methods section.*
☒	☐	A description of all covariates tested
☒	☐	A description of any assumptions or corrections, such as tests of normality and adjustment for multiple comparisons
☐	☒	A full description of the statistical parameters including central tendency (e.g. means) or other basic estimates (e.g. regression coefficient) AND variation (e.g. standard deviation) or associated estimates of uncertainty (e.g. confidence intervals)
☒	☐	For null hypothesis testing, the test statistic (e.g. *F*, *t*, *r*) with confidence intervals, effect sizes, degrees of freedom and *P* value noted<br>*Give P values as exact values whenever suitable.*
☒	☐	For Bayesian analysis, information on the choice of priors and Markov chain Monte Carlo settings
☒	☐	For hierarchical and complex designs, identification of the appropriate level for tests and full reporting of outcomes
☒	☐	Estimates of effect sizes (e.g. Cohen's *d*, Pearson's *r*), indicating how they were calculated

Our web collection on statistics for biologists contains articles on many of the points above.

Software and code

Policy information about availability of computer code

Data collection	Rave EDC (Medidata, New York, NY, USA)
Data analysis	SAS version 9.4 (SAS Institute Inc., Cary, NC, USA), NONMEM version 7.4.3 (Icon Development Solutions, Elliot City, Maryland, USA)

For manuscripts utilizing custom algorithms or software that are central to the research but not yet described in published literature, software must be made available to editors and reviewers. We strongly encourage code deposition in a community repository (e.g. GitHub). See the Nature Portfolio guidelines for submitting code & software for further information.

Data

Policy information about availability of data

All manuscripts must include a data availability statement. This statement should provide the following information, where applicable:

- Accession codes, unique identifiers, or web links for publicly available datasets
- A description of any restrictions on data availability
- For clinical datasets or third party data, please ensure that the statement adheres to our policy

Qualified researchers may request access to individual patient level clinical data through a data request platform. At the time of writing this request platform is Vivli: https://vivli.org/ourmember/roche/. This request process is necessary to comply with informed consent and General Data Protection Regulation requirements. For up to date details on Roche's Global Policy on the Sharing of Clinical Information and how to request access to related clinical study documents, see here: https://go.roche.com/data_sharing. Anonymised records for individual patients across more than one data source external to Roche can not, and should not, be linked due to a potential increase in risk of patient re-identification.

Research involving human participants, their data, or biological material

Policy information about studies with human participants or human data. See also policy information about sex, gender (identity/presentation), and sexual orientation and race, ethnicity and racism.

Reporting on sex and gender	Summarised data on participants' sex is provided in Tables S1 and S8. Table S8 provides the key result disaggregated by sex. Both male and female participants were recruited into the studies, and designation of sex was based on entry into the case report forms at each study site.
Reporting on race, ethnicity, or other socially relevant groupings	Constructs of race and/or ethnicity were not applicable in this manuscript and were not used as proxies for other variables.
Population characteristics	S2 included 192 patients with CRC (84.2%), 187 with confirmed MSS (82%) disease, 5 with confirmed MSI-H (2.2%) disease and remaining patients unknown. All patients had metastatic disease at study entry, most with metastases to the liver (n=56 [24.6%]), lung (n=33 [14.5%]) or both (n=127 [55.7%]). At study entry, patients' mean age was 57 years (range: 24-81 years), 96 (42.1%) were female. ECOG PS at baseline was 0 (n=132 [57.9%]) or 1 (n=96 [42.1%]). 227 patients (99.6%) had received at least one prior line of therapy for metastatic disease, 91 (39.9%) received prior adjuvant treatment and 140 (61.4%) received 3 or more prior lines of therapy. Detailed baseline characteristics for S1 and S2 are presented in Supplementary Table 1.
Recruitment	Investigators from each participating institution were responsible for identification of potential trial participants, their screening, and their inclusion into the study if eligible. If applicable, CEA expression as an eligibility criterion was assessed locally in Europe and centrally in North America as described in the manuscript. Full eligibility criteria are described in the study protocols, included as part of the Supplementary Information.
Ethics oversight	Both studies were designed and conducted in compliance with the principles of the Declaration of Helsinki and the Good Clinical Practice guidelines of the International Council for Harmonisation, and the study design and conduct complied with all relevant regulations regarding the use of human study participants. Both study protocols were approved by the institutional review boards or independent ethics committee at each study site. For study S1, the initial protocol was first approved by clinical research ethics committee at Vall d'Hebron Hospital (Barcelona, Spain) on December 12, 2014, for study conduct at Vall d'Hebron University Hospital (Barcelona, Spain) and the University of Navarra Hospital (Navarra, Spain). For study S2, the clinical research ethics committee of the Government of Navarra Department of Health (Navarra, Spain) first approved the initial protocol on December 15, 2015, for study conduct at the same sites as for S1. S1 and S2 were first authorised by The Spanish Agency for Medicine and Health Products on December 18, 2014, and December 28, 2015, respectively. Written informed consent was obtained from each patient before the initiation of study procedures.

Note that full information on the approval of the study protocol must also be provided in the manuscript.

Field-specific reporting

Please select the one below that is the best fit for your research. If you are not sure, read the appropriate sections before making your selection.

[x] Life sciences [ ] Behavioural & social sciences [ ] Ecological, evolutionary & environmental sciences

For a reference copy of the document with all sections, see nature.com/documents/nr-reporting-summary-flat.pdf

Life sciences study design

All studies must disclose on these points even when the disclosure is negative.

Sample size	Appropriate sample sizes were calculated for each part of studies S1 and S2. CONSORT-style diagrams for both studies are available in the Supplementary Materials (Supplementary Figures 1 and 2, respectively), which may be helpful in understanding the structure of both studies. For S1 and S2 a standard SAD+MAD dose escalation using a mCRM with EWOC design was employed. For S1, this approach was repeated for dose+schedule finding following obi pre-treatment followed by an expansion in the 60-mg cohort (aggregated in this manuscript for simplicity). The step-up expansion cohorts were planned to recruit up to 30 patients per indication to provide an initial ORR estimate, but stopped recruitment prior to that due to the complex safety profile observed. For S1, 40 patients were randomised at 100 mg at QW vs Q3W to detect a difference of 10% ORR vs 20% ORR to support schedule finding. A 40 patient expansion at 160 mg QW was conducted to provide an initial ORR estimate. Additional dose and schedule finding was performed with up to 40 patients per cohort to understand the complex ADA and safety profile and to provide initial ORR estimates with this approach. 149 and 228 patients were enrolled and treated in S1 and S2, respectively. All patients were included in the S1 and S2 safety- and efficacy-evaluable populations, which were defined, respectively, as all patients who received at least one dose of cibisatamab or obinutuzumab and all patients who received at least one dose of cibisatamab or atezolizumab.<br>Rationale. For S1, the sample size for the dose escalation part is based on the operating characteristics across different assumed toxicity scenarios, as detailed in the body of the protocol (available in the Supplementary Materials). This design has previously been used successfully in Bailey et al. (2009) with only slight modifications. The maximum sample size of approximately 30 patients in cohorts C, D, E, F and G each would allow for a sufficiently precise estimation of ORR per cancer type under the step-up QWx3/Q3W schedule. However, Cohorts D, E, F, and G did not enroll any patients. The sample size of approximately 15 patients in the Q3W cohort with obinutuzumab pretreatment would allow an initial exploration, while the sample size of approximately 40 patients in Cohort H would allow for a sufficiently precise estimation of ORR in MSS CRC under the step-up QWx3/Q3W schedule. Cohort H, however, did not enroll any patients.<br>For S2, sample size estimation for the dose escalation in Part IA was based on study simulations of the mCRM with EWOC design. The sample

size of 20 patients per dose scheme in the schedule comparison expansion (cohort A) in Part IB would allow for a reasonably precise differentiation between the QW and Q3W dose schemes. Specifically, an observed difference of ca. 10% points in ORR would lead to approximately 80% posterior probability of a true response rate difference between two dose schemes. For example, if the number of objective responses is 4 in one and 2 in the other dose ORR in scheme out of 20 patients each, then the posterior probability of a positive response rate difference is 79.5%. For cohort C, the randomization of approximately 40 patients to each of the arms C1, C2 and possibly 40 patients enrolled in a later optional arm C3 would allow for a reasonably precise differentiation between the arms. Specifically, if one of the arms has 20% true ORR, and the other two arms have only 10% true ORR, then n=40 patients per arm gives 83% power to decide for the correct arm as the best one. In another scenario, where the first arm has 10% true ORR, the second arm 20% true ORR, and the third arm 30% true ORR, then n=40 patients per arm gives 85% power to pick the correct third arm as the best one. Hence, the sample size of 40 patients per arm in cohort C is justified. Note: Cohort C1 enrolled 39 patients, and Cohort C2 enrolled 35 patients; no further patients will be enrolled into these cohorts. Cohort C3 did not enroll any patients. For Cohort G1, the inclusion of 40 patients would allow a reasonably precise estimation of the incidence of ADA. Specifically, if none of the 40 patients were to develop ADA by week 8, then the 95% confidence interval for the ADA incidence will be 0% to 9%, i.e., exclude values of 10% or higher. In addition to the assessment of immunogenicity reduction by obinutuzumab, Cohort G1, as well as optionally Cohorts G2 and G3 would allow for a descriptive assessment of the primary safety and efficacy endpoints in the MSS CRC and optionally in the gastric and pancreatic indications, respectively. Specifically, observing an ORR of 20% in a cohort would result in a 95% confidence interval from 9% to 36%, i.e., exclude values of 9% or lower. Cohorts G1, G2, and G3, however, did not enroll any patients. The sample size of approximately 10-20 patients in each of the cohorts B1 (MSS and MSI-H CRC separately), B2 and the safety cohorts in other indications would allow for an initial assessment of safety and tolerability. This is shown in detail with simulations of the intra-patient dose escalation design in cohort B1 in the protocol Appendix 7. Cohort B2 did not enroll any patients, and the other cohorts stopped enrollment.

Data exclusions

No data was excluded from the analyses as defined in the study protocol/manuscript.

Replication

Both studies were only conducted once.

Randomization

The schedule comparison extension in S2 randomised 40 patients to QW or Q3W with no stratification. Otherwise, in line with typical dose escalation and expansion approaches, no randomisation took place. CONSORT-style diagrams for both studies are available in the Supplementary Materials.

Blinding

None, open-label

Reporting for specific materials, systems and methods

We require information from authors about some types of materials, experimental systems and methods used in many studies. Here, indicate whether each material, system or method listed is relevant to your study. If you are not sure if a list item applies to your research, read the appropriate section before selecting a response.

Materials & experimental systems

n/a	Involved in the study	
☐	☒	Antibodies
☒	☐	Eukaryotic cell lines
☒	☐	Palaeontology and archaeology
☒	☐	Animals and other organisms
☐	☒	Clinical data
☒	☐	Dual use research of concern
☒	☐	Plants

Methods

n/a	Involved in the study	
☒	☐	ChIP-seq
☐	☒	Flow cytometry
☒	☐	MRI-based neuroimaging

Antibodies

Antibodies used

PanCK-CD8 assay, Clone SP239 and Clone AE1/AE3/PCK26, at HistoGeneX
PD-Ll Clone SP263 at Ventana
Cell Marque CEA31 monoclonal antibody, Catalog No. 236M-96, at Ventana

Validation

Anti-CDS alpha antibody [SP239]
Description: Rabbit monoclonal [SP239] to CD8 alpha
Host species: Rabbit
Tested applications Suitable for: IHC-P
Species reactivity Reacts with: Human
Immunogen Synthetic peptide. This information is proprietary to Abeam and/or its suppliers.
Positive control lHC-P: Human tonsil tissue.
General notes: This product is a recombinant monoclonal antibody, which offers several advantages including:
- High batch-to-batch consistency and reproducibility
- Improved sensitivity and specificity
- Long-term security of supply
- Animal-free production
Our RabMAb technology is a patented hybridoma-based technology for making rabbit monoclonal antibodies. For details on our patents, please refer to RabMAb patents.

Anti-Pan Keratin (AE1/AE3/PCK26) antibody
Anti-Pan Keratin (AE1/AE3/PCK26) antibody contains a cocktail of mouse monoclonal antibodies raised against human epidermal keratins. 1 This antibody cocktail reacts with the 56.SkD, 50kD, 48kD, and 40kD cytokeratins of the acidic subfamily and 65-67kD, 64kD, 59kD, 58kD, 56kD, and 52kD cytokeratins of the basic subfamily.2,3,4 Anti-Pan Keratin (AE1/AE3/PCK26) antibody binds to keratins in FFPE tissue and displays a cytoplasmic staining pattern.
1. Woodcock-Mitchell J, Eichner R, Nelson WG, et al. lmmunolocalization of keratin polypeptides in human epidermis using monoclonal antibodies. J Cell Biol. 1982;95(2):580-588.
2. Chu PG, Weiss LM. Keratin expression in human tissues and neoplasms. Histopathology. 2002;40(5):403-39.
3. Moll R, Diva M, Langbein L. The Human Keratins: Biology and Pathology. Histochem Cell Biol. 2008;129(6):705-733.
4. Bahrami A, Truong LD, Ro JV. Undifferentiated Tumor: True Identity by lmmunohistochemistry. Arch Pathol Lab Med. 2008;132(3):326-348.
VENTANA PD-L1 (SP263)
VENTANA PD-L1 (SP263) Rabbit Monoclonal Primary Antibody is a rabbit monoclonal primary antibody produced against programmed death-ligand 1 (PD-L1) also known as B7 homolog 1 (B7-H1) or CD274. It recognizes a transmembrane bound glycoprotein that has a molecular mass of 45-55 kDa. This antibody produces membranous and/or cytoplasmic staining.
Intended Use
VENTANA PD-L1 (SP263) Rabbit Monoclonal Primary Antibody is intended for laboratory use in the detection of the PD-Ll protein in formalin-fixed, paraffin-embedded tissue. It is intended to be stained with BenchMark IHC/ISH instruments. It is indicated as an aid in the assessment of PD-L1 expression in human tissues.
References:
1. Keir ME, Butte MJ, Freeman GJ, et al. PD-1 and its ligands in tolerance and immunity. Annu Rev lmmunol. 2008;26:677-704.
2. Blank C, Mackensen A. Contribution of the PD-Ll/PD-1 pathway to T-cell exhaustion: an update on implications for chronic infections and tumor evasion. Cancer lmmunol lmmunother. 2007;56(5) :739-745.
3. Butte MJ, Keir ME, Phamduy TB, et al. Programmed death-1 ligand 1 interacts specifically with the B7-1 costimulatory molecule to inhibit T-cell responses. Immunity. 2007;27(1):111-122.
4. Dong H, Zhu G, Tamada K, Chen L. B7-Hl, a third member of the B7 family, co-stimulates T-cell proliferation and interleukin-10 secretion. Nat Med. 1999;5(12) :1365-1369.
5. Massard C, Gordon MS, Sharma S, et al. Safety and efficacy of durvalumab (MEDl4736), an anti-programmed cell death ligand-1 immune checkpoint.
inhibitor, in patients with advanced urothelial bladder cancer. J Clin Oncol. 2016;34(26) :3119-3125.
6. Patel SP, Kurzrock R. PD-Ll Expression as a Predictive Biomarker in Cancer lmmunotherapy. Mal Cancer Ther. 2015;14(4):847-856.
Cell Marque CEA31
Anti-CEA is an antibody against carcinoembryonic antigen (CEA), a protein thought to promote tumor development through its role as a cell adhesion molecule. Anti-CEA positivity is seen in adenocarcinomas of many origins, especially colon and lung adenocarcinomas, but rarely seen in mesothelial cells and mesotheliomas.
References:
1. Tron, Vet al. Carcinoembryonic antigen and milk-fat globule protein staining of malignant mesothelioma and adenocarcinoma of the lung. Archives of pathology & laboratory medicine vol. 111,3 (1987):291-3.
2. Abutaily, AS et al. lmmunohistochemistry in the distinction between malignant mesothelioma and pulmonary adenocarcinoma: a critical evaluation of new antibodies. J Clin Pathol. 2002;55(9):662-8.
3.Carella, R et al. lmmunohistochemical panels for differentiating epithelial malignant mesothelioma from lung adenocarcinoma: a study with logistic regression analysis. Am J Surg Pathol. 2001;25(1):43-50.
4.Bhatnagar, Julu et al. lmmunohistochemical detection of carcinoembryonic antigen in esophageal carcinomas: a comparison with other gastrointestinal neoplasms. Anticancer Res. 2002; 22(3):1849-57.
5.Lagendijk, J H et al. lmmunohistochemical differentiation between primary adenocarcinomas of the ovary and ovarian metastases of colonic and breast origin. Comparison between a statistical and an intuitive approach. J Clin Pathol. 1999;52(4):283-90.

Clinical data

Policy information about clinical studies

All manuscripts should comply with the ICMJE guidelines for publication of clinical research and a completed CONSORT checklist must be included with all submissions.

Clinical trial registration	NCT02324257, NCT02650713
Study protocol	Study protocols are available as part of the Supplementary Materials
Data collection	Study data including data for evaluation of the study endpoints was collected via electronic case report forms populated by participating trial sites. Timelines for the data collection are as follows:<br>S1<br>First Patient Enrolled: 29-Dec-2014<br>Last patient last visit: 03-Sept-2019<br>Sites:<br>Cedars Sinai Medical Center; Samuel-Oschin Comprehensive Cancer Institute (Los Angeles, California, United States, 90048)<br>Stanford University (Palo Alto, California, United States, 94305)<br>UCLA Cancer Center (Santa Monica, California, United States, 90404)<br>University Of Colorado (Aurora, Colorado, United States, 80045)<br>Yale Cancer Center; Medical Oncology (New Haven, Connecticut, United States, 06520)<br>Dana Farber - Harvard (Boston, Massachusetts, United States)<br>Columbia University Medical Center (New York, New York, United States, 10032)<br>Medical University of South Carolina (Charleston, South Carolina, United States, 29425)<br>Sarah Cannon Cancer Center (Germantown, Tennessee, United States, 38138)<br>Princess Margaret Cancer Center (Toronto, Ontario, Canada, M5G 1Z5)<br>Rigshospitalet; Onkologisk Klinik (København Ø, Denmark, 2100)<br>Centre Leon Berard; Departement Oncologie Medicale (Lyon, France, 69373)<br>IRCCS IST. Tumori Fondaz. Pascale; S.C. Oncologia Medica,Melanoma,Immunoterapia E Terapie Innovative (Napoli, Campania, Italy, 80131)<br>Azienda Ospedaliera Universitaria Senese, U.O.C. Immunoterapia Oncologica (Siena, Toscana, Italy, 53100)<br>Antoni van Leeuwenhoek Ziekenhuis (Amsterdam, Netherlands, 1066 CX)<br>Hospital del Mar; Servicio de Oncologia (Barcelona, Spain, 08003)<br>Hospital Univ Vall d'Hebron; Servicio de Oncologia (Barcelona, Spain, 08035)<br>START Madrid-FJD, Hospital Fundacion Jimenez Diaz (Madrid, Spain, 28040)<br>Hospital Universitario 12 de Octubre; Servicio de Oncologia (Madrid, Spain, 28041)<br>START Madrid. Centro Integral Oncologico Clara Campal; CIOCC (Madrid, Spain, 28050)<br>Clinica Universitaria de Navarra; Servicio de Oncologia (Pamplona, Navarra, Spain, 31008)<br><br>S2<br>First Patient Enrolled: 07-Jan-2016<br>Last patient last visit: 13-Jan-2020<br>Sites:<br>Cedars Sinai Medical Center; Samuel-Oschin Comprehensive Cancer Institute (Los Angeles, California, United States, 90048)<br>Stanford University (Palo Alto, California, United States, 94305)<br>UCLA Cancer Center (Santa Monica, California, United States, 90404)<br>University Of Colorado (Aurora, Colorado, United States, 80045)<br>Yale Cancer Center; Medical Oncology (New Haven, Connecticut, United States, 06520)<br>Dana Farber - Harvard (Boston, Massachusetts, United States)<br>Columbia University Medical Center (New York, New York, United States, 10032)<br>Medical University of South Carolina (Charleston, South Carolina, United States, 29425)<br>Sarah Cannon Cancer Center (Germantown, Tennessee, United States, 38138)<br>Princess Margaret Cancer Center (Toronto, Ontario, Canada, M5G 1Z5)<br>Rigshospitalet; Onkologisk Klinik (København Ø, Denmark, 2100)<br>Centre Leon Berard; Departement Oncologie Medicale (Lyon, France, 69373)<br>IRCCS IST. Tumori Fondaz. Pascale; S.C. Oncologia Medica,Melanoma,Immunoterapia E Terapie Innovative (Napoli, Campania, Italy, 80131)<br>Azienda Ospedaliera Universitaria Senese, U.O.C. Immunoterapia Oncologica (Siena, Toscana, Italy, 53100)<br>Antoni van Leeuwenhoek Ziekenhuis (Amsterdam, Netherlands, 1066 CX)<br>Hospital del Mar; Servicio de Oncologia (Barcelona, Spain, 08003)<br>Hospital Univ Vall d'Hebron; Servicio de Oncologia (Barcelona, Spain, 08035)<br>START Madrid-FJD, Hospital Fundacion Jimenez Diaz (Madrid, Spain, 28040)<br>Hospital Universitario 12 de Octubre; Servicio de Oncologia (Madrid, Spain, 28041)<br>START Madrid. Centro Integral Oncologico Clara Campal; CIOCC (Madrid, Spain, 28050)<br>Clinica Universitaria de Navarra; Servicio de Oncologia (Pamplona, Navarra, Spain, 31008)

Outcomes	Study endpoints and outcome measures were defined in the study protocols by the trial sponsor team prior to the recruitment of any patients. Study outcomes were generally assessed by the investigator with the exception of tumour response according to RECIST 1.1 in S2, which was assessed by independent review.

Plants

Seed stocks	N/A
Novel plant genotypes	N/A
Authentication	N/A

Flow Cytometry

Plots

Confirm that:

- [ ] The axis labels state the marker and fluorochrome used (e.g. CD4-FITC).
- [ ] The axis scales are clearly visible. Include numbers along axes only for bottom left plot of group (a 'group' is an analysis of identical markers).
- [ ] All plots are contour plots with outliers or pseudocolor plots.
- [ ] A numerical value for number of cells or percentage (with statistics) is provided.

Methodology

Sample preparation	N/A
Instrument	N/A
Software	N/A
Cell population abundance	N/A
Gating strategy	N/A

- [ ] Tick this box to confirm that a figure exemplifying the gating strategy is provided in the Supplementary Information.
